# LINGO1-targeted antibody-drug conjugates improve efficacy and tolerability of antineoplastic therapies in Ewing sarcoma models

**DOI:** 10.1172/JCI204641

**Published:** 2026-08-03

**Authors:** Zhichuan Zhu, Yusha Liu, Yu Deng, Zhijun Li, Albert S. Baldwin, Pengda Liu

**Affiliations:** 1 Lineberger Comprehensive Cancer Center,; 2 Department of Biochemistry and Biophysics,; 3 Department of Biostatistics, and; 4 Department of Cell Biology and Physiology, The University of North Carolina at Chapel Hill, Chapel Hill, North Carolina, USA.

**Keywords:** Cell biology, Oncology, Bone disease, Cancer, Therapeutics

## Abstract

We reported LINGO1 as a potential marker on Ewing sarcoma cells that could enable targeted drug delivery, improving treatment effectiveness while reducing side effects.

**To the Editor:** Ewing sarcoma is an aggressive and rare pediatric cancer that develops in bones and soft tissues. Chemotherapy, radiotherapy, and surgery remain the first-line therapy for patients (1). Although effective in approximately 70% of patients, intensive multicycle chemotherapy, often requiring repetitive high doses, substantially compromises quality of life and increases the risk of treatment-related secondary morbidities. In the absence of approved targeted therapies, there remains a critical unmet medical need to develop additional therapeutic strategies that enhance chemotherapy efficacy while mitigating adverse effects associated with current treatment regimens.

A major limitation of conventional chemotherapy is its systemic administration, which dilutes the effective drug concentration within tumors and contributes to off-target toxicity. To address this challenge, we sought to enhance the selective delivery of therapeutics to Ewing sarcoma tumors while sparing healthy tissues. We therefore focused on the development of antibody-drug conjugates (ADCs), an approach that critically depends on the identification of tumor-specific cell-surface targets (2). To identify cell-surface targets suitable for ADC development, we applied a stepwise prioritization strategy. Among 883 plasma membrane proteins annotated in the Human Protein Atlas (Supplemental Table 1; supplemental material available online with this article; https://doi.org/10.1172/JCI204641DS1) and 39 ubiquitously expressed Ewing sarcoma surface proteins (3), we identified 8 overlapping candidates (Figure 1A). Four are known EWS:FLI1 transcriptional targets, supporting their relevance as ADC targets. To minimize off-tumor toxicity, we further assessed tumor specificity. Only LINGO1 (leucine-rich repeat and Ig domain–containing 1) and SLCO5A1 (Solute Carrier Organic Anion Transporter Family Member 5A1) exhibited peak expression in bone cancer cell lines (Supplemental Figure 1A). LINGO1 expression outside Ewing sarcoma was largely confined to brain tissue, where blood-brain barrier–restricted antibody access may limit toxicity, whereas SLCO5A1 was broadly expressed among tissues (Supplemental Figure 1B). We prioritized LINGO1, consistent with a previous report (4), for further study.

LINGO1 is a CNS-enriched, postnatally expressed transmembrane protein functioning within the Nogo receptor complex to regulate myelination and neuronal survival, minimizing concerns about developmental toxicity when therapeutically targeted. DepMap analysis and genetic depletion studies showed LINGO1 modestly supports Ewing sarcoma cell growth in vitro (Figure 1B and Supplemental Figure 1, C–E). LINGO1 expression was consistently detected in primary and relapsed Ewing sarcoma patient-derived xenografts (5) with cell-surface expression confirmed by FACS (Figure 1C and Supplemental Figure 2, A and B). The LINGO1 extracellular domain is recognized by Li81 antibody (opicinumab/BIIB033, currently in clinical trials for multiple sclerosis), which undergoes lysosomal trafficking following endocytosis (4), enabling payload release. Accordingly, opicinumab-SN38 ADCs (drug-to-antibody ratio [DAR] = 5.2) induced LINGO1-dependent apoptosis in Ewing sarcoma cells, with reduced killing upon LINGO1 depletion (Figure 1D and Supplemental Figure 2, C–F). A single opicinumab-SN38 injection modestly delayed tumor growth in MHH-ES-1 xenografts, though without statistical significance, likely reflecting limited payload exposure (Supplemental Figure 3, A–C).

To overcome limited payload efficacy, we replaced SN-38 with the more potent microtubule-disrupting agent monomethyl auristatin E (MMAE) (6). Five MMAE-based ADCs have been FDA approved, including Adcetris, Polivy, Padcev, Tivdak, and Aidixi (RC48), with additional agents (ABBV-399 and zilovertamab vedotin) in phase III trials. We synthesized opicinumab-MMAE ADCs (DAR = 4.6) and treated MHH-ES-1 xenograft-bearing mice with the same molecular mass of free MMAE or opicinumab-MMAE ADCs. Consistent with its known toxicity, free MMAE caused rapid body weight loss, necessitating early termination of the study (Figure 1G). In contrast, opicinumab-MMAE ADCs at comparable payload doses showed no overt toxicity and suppressed Ewing sarcoma tumor growth (Figure 1, E–G, and Supplemental Figure 3, D–G). Half-dose opicinumab-MMAE efficacy was validated in a TC-32 xenograft model (Supplemental Figure 3, H–J). Biodistribution revealed marked enrichment of free MMAE in tumors versus liver, brain, and serum (Figure 1H). Compared with irinotecan, a single dose of opicinumab-MMAE achieved more sustained tumor control (Figure 1, I and J, and Supplemental Figure 3K) without detectable liver or brain toxicity (Figure 1M and Supplemental Figure 3, L and M) or body weight loss (Figure 1N). Pharmacokinetic analysis showed similar profiles for opicinumab and opicinumab-MMAE (*t*_1/2β_ ~2 days), with released MMAE levels approximately 4 orders of magnitude lower (Figure 1, H, K, and L). Together, these findings support the therapeutic potential of opicinumab-MMAE ADCs in preclinical Ewing sarcoma models.

In summary, our study identifies LINGO1 as a selective and therapeutically actionable cell-surface target in Ewing sarcoma and demonstrates the preclinical efficacy and tolerability of LINGO1-targeted ADCs. Further studies are warranted to evaluate their clinical potential in immunocompetent models and patient cohorts and to explore LINGO1-directed cellular therapies such as CAR T cells.

For detailed methods, information regarding sex as a biological variable, statistics, study approval, and author contributions, see the supplemental materials.

## Conflict of interest

The authors have declared that no conflict of interest exists.

## Funding support

- North Carolina Biotechnology Center Flash Grant (to PL).
- Department of Defense, Congressionally Directed Medical Research Programs, Kidney Cancer Research Program, Idea Development Award HT9425-24-1-0644 (to PL).
- The University of North Carolina at Chapel Hill, University Cancer Research Fund (to PL).

## Supplementary Material

Supplemental data

Unedited blot and gel images

Supporting data values

## Figures and Tables

**Figure 1:**
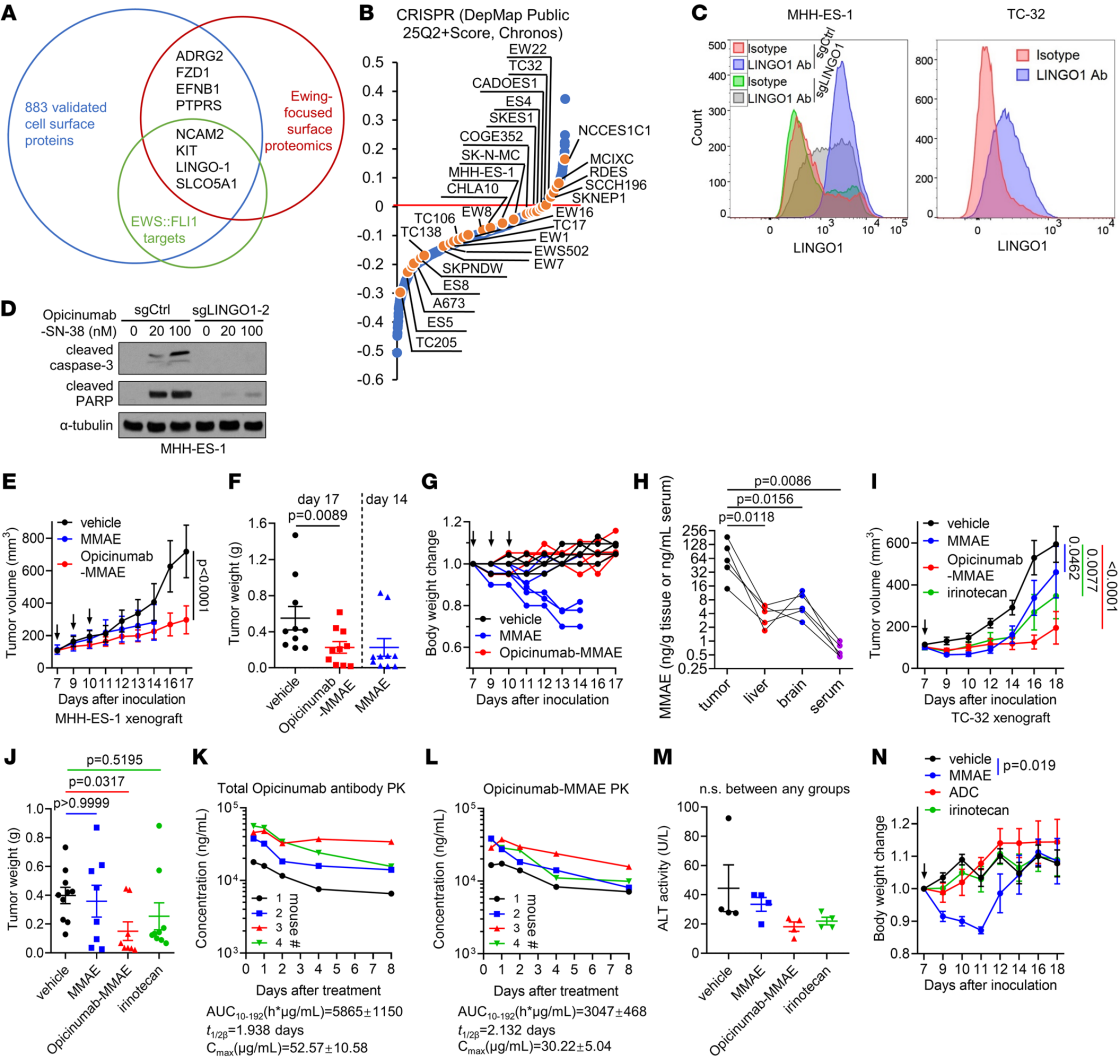
LINGO1-targeted opicinumab-MMAE ADCs show anti–Ewing sarcoma efficacy with reduced toxicity in vivo. (**A**) Venn diagram showing overlapping Ewing sarcoma cell-surface markers identified from 3 independent datasets: the Human Protein Atlas (blue), Ewing sarcoma–focused surface proteomics (red), and curated EWS:FLI1 target genes (green). (**B**) DepMap waterfall plot demonstrating cell viability changes following CRISPR-mediated LINGO1 depletion. (**C**) FACS analysis of cell-surface LINGO1 expression in indicated Ewing sarcoma cells. (**D**) Immunoblot for apoptosis markers in MHH-ES-1 cells 3 days after opicinumab-SN38 treatment. (**E** and **F**) Tumor volumes and weights of MHH-ES-1 xenografts (*n* = 10) treated with indicated agents. Arrows indicate treatments. (**G**) Body weights of mice from **E** and **F**. *n* = 5. (**H**) MMAE biodistribution 7 days after opicinumab-MMAE treatment. *n* = 5. (**I** and **J**) Tumor volumes and weights of TC-32 xenografts treated with indicated agents. *n* = 10 (vehicle), 8 (MMAE and opicinumab-MMAE), and 9 (irinotecan). (**K** and **L**) Serum concentrations of opicinumab and opicinumab-MMAE in opicinumab-MMAE–treated mice. *n* = 4. (**M**) Alanine aminotransferase (ALT) activity on day 17 in mice from **I** and **J**. *n* = 4. (**N**) Mouse body weights from **I** and **J**. *n* = 5 (vehicle/irinotecan) and 4 (MMAE/opicinumab-MMAE). Data represent mean ± SEM. *P* values were calculated using 2-way ANOVA with Bonferroni’s test (**E**) or Tukey’s test (**I** and **N**), unpaired 2-tailed Mann-Whitney test (**F**), 1-way ANOVA with Dunnett’s test (**H**), and Kruskal-Wallis with Dunn’s test (**J** and **M**).

## Version 1 08/03/2026

Electronic publication
