# Supplementary material for: LINGO1-targeted antibody-drug conjugates improve efficacy and tolerability of antineoplastic therapies in Ewing sarcoma models
Source: J Clin Invest. 2026 Aug 3;136(15):e204641. doi: 10.1172/JCI204641 (PMC13430015; doi:10.1172/JCI204641)
Supplement: Supplemental data [file jci-136-204641-s187.pdf]

Supplemental information

LINGO1-targeted antibody-drug conjugates improve efficacy and tolerability of antineoplastic therapies in Ewing sarcoma models

Zhichuan Zhu, Yusha Liu, Yu Deng, Zhijun Li, Albert S. Baldwin, and Pengda Liu

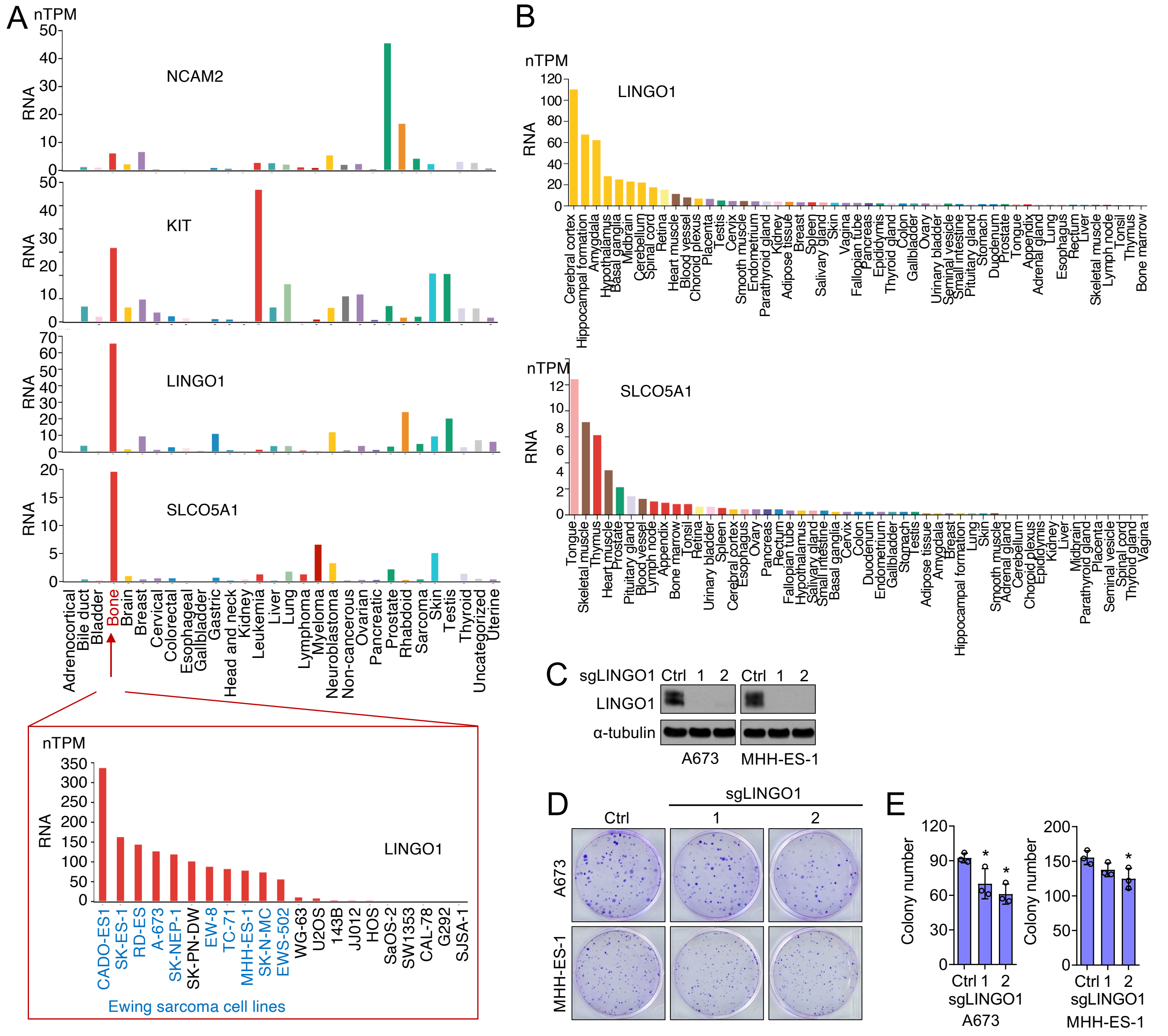

Figure S1. LINGO1 RNA expression is enriched in bone cancer cells and brain tissues.
(**A**) RNA expression levels of NCAM2, KIT, LINGO1 and SLCO5A1 in indicated cancer cell line datasets from Human Protein Atlas. (**B**) RNA expression of LINGO1 and SLCO5A1 in indicated human tissues (data obtained from Human Protein Atlas). (**C**) IB analysis for LINGO1 expression in indicated Ewing sarcoma cell lines. (**D**, **E**) Representative colony formation images (D) and corresponding quantification (E) of cells shown in (C). Data represent mean ± SD (n = 3). *P<0.05. One-way ANOVA with Dunnett's test.

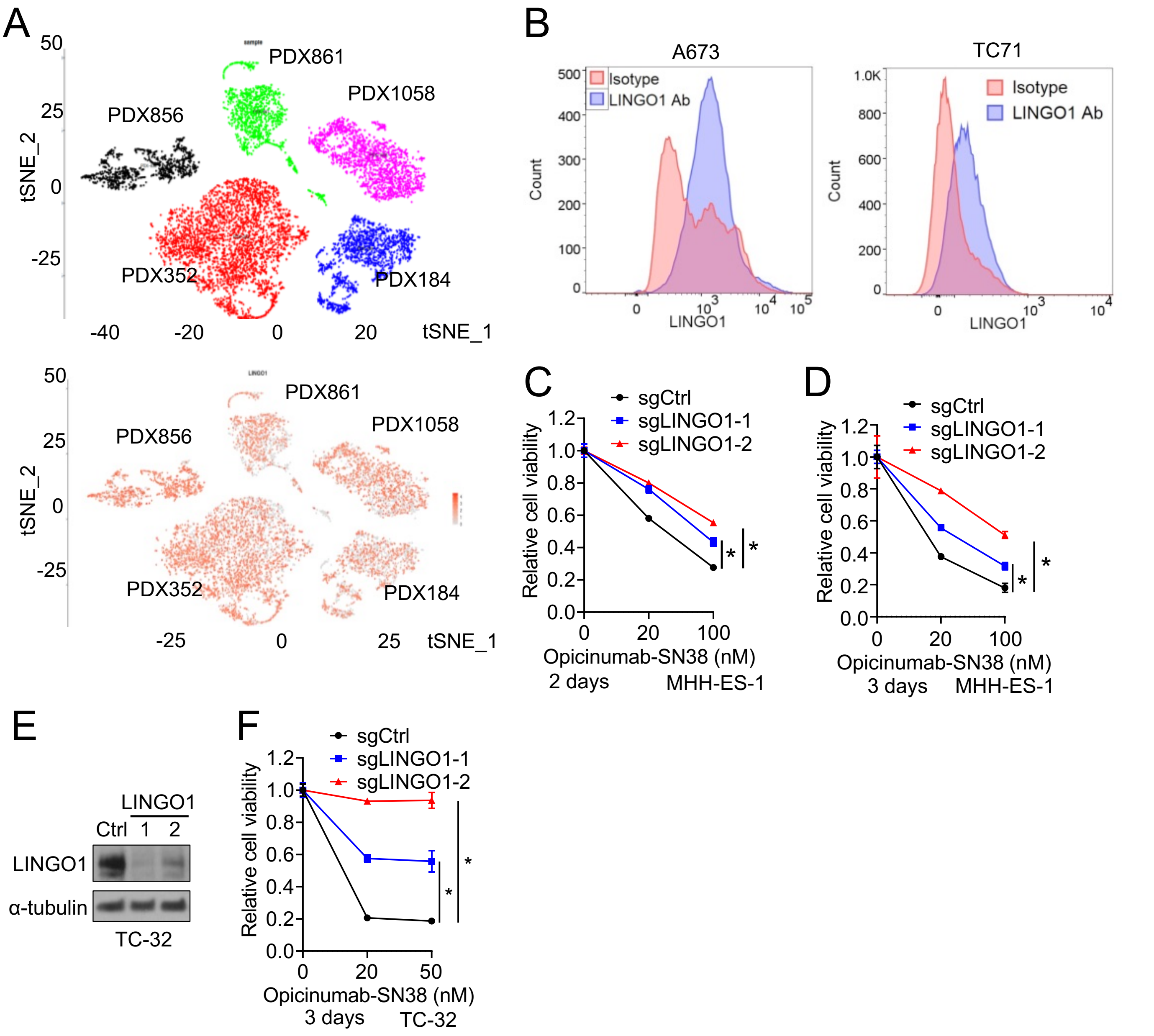

Figure S2. LINGO1 is an Ewing sarcoma surface marker, and LINGO1-targeting ADCs kill Ewing sarcoma cells depend on LINGO1.

(**A**) *t*-SNE plot of 9,654 cells from five PDXs from *EWS::FL1*-positive EwS (1), with cells colored by PDX tumor of origin (top) and expression level of LINGO-1 (bottom). The top panel shows that cell transcriptional profiles strongly cluster by PDX model, and the bottom panel confirms LINGO-1 expression across all PDX models. (**B**) FACS validation of surface LINGO-1 expression in A673 and TC71 Ewing sarcoma cells. (**C**-**F**) Relative viability of MHH-ES-1 cells (C, D) or TC-32 cells (F) treated with the indicated doses of opicinumab-SN-38 ADCs for indicated days. LINGO1 depletion in TC-32 cells was confirmed by IB (E). Data represent mean ± SD (n = 3). *P<0.05. Two-way ANOVA with Tukey's test.

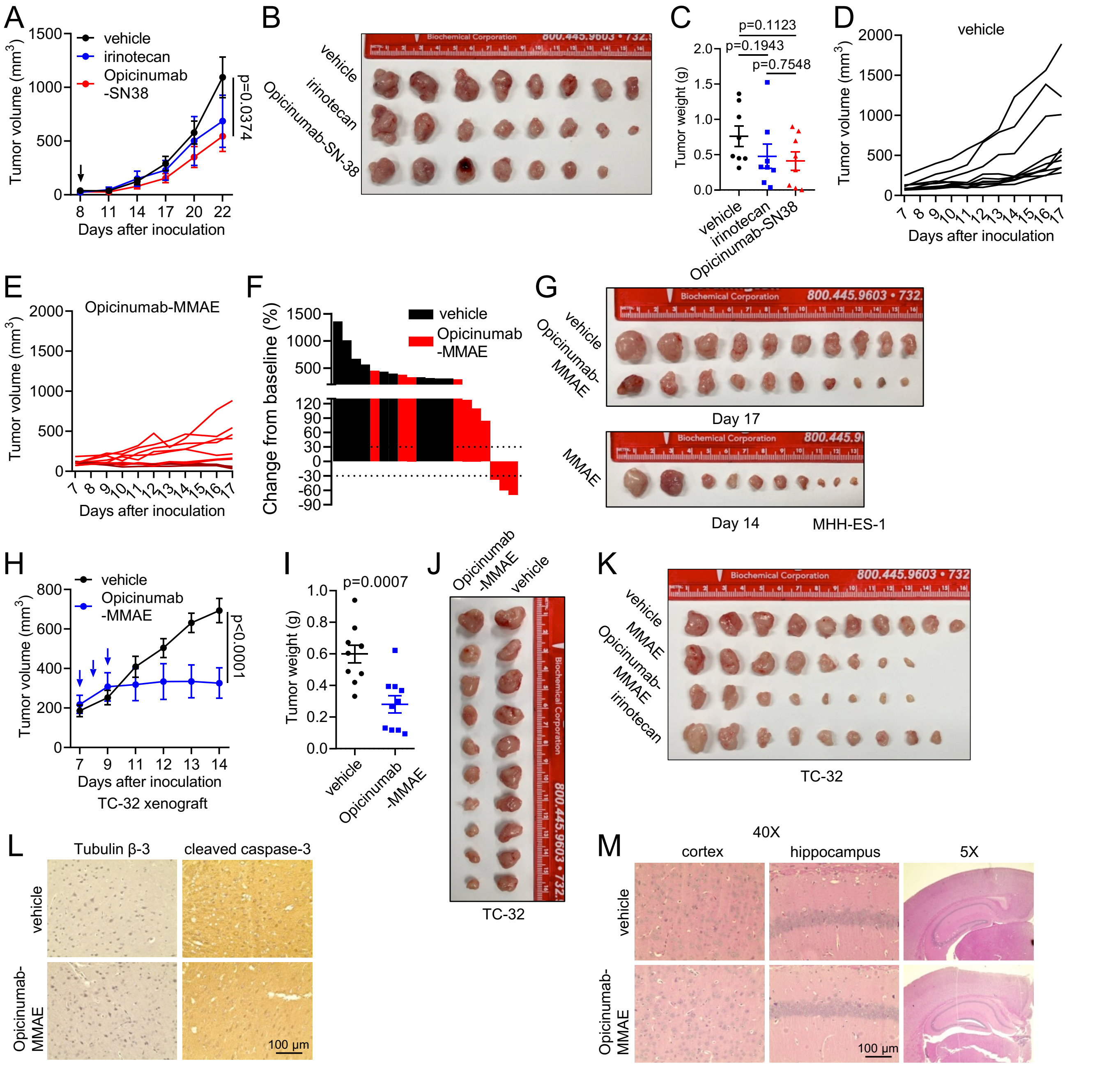

Figure S3. Opicinumab-MMAE ADCs demonstrate improved safety and efficacy in reducing xenografted Ewing sarcoma tumor growth *in vivo* with reduced toxicity.

(**A**) Volume of MHH-ES-1 xenografts in mice treated with the indicated agents. Arrow denotes the administration of indicated treatments. Data represent mean ± SEM (n = 8). (**B**, **C**) Representative tumors (B) isolated from (A) and weighted in (C). Data represent mean ± SEM (n = 8). (**D**, **E**) Volume of individual MHH-ES-1 xenografts in mice after indicated agent treatment. (**F**) Tumor volume changes from baseline (%) obtained from (D, E). (**G**) Tumors isolated from (D-F). (**H-J**) Volume of TC-32 xenografts in mice treated with the indicated agents (H). Tumors (J) isolated from (H) and weighted in (I). Data represent mean ± SEM (n = 10). (**K**) Xenografted TC-32 tumors treated with indicated agents. (**L**, **M**) Representative IHC images (40X) of neuron marker tubulin β-3 and apoptosis marker cleaved caspase-3, and HE staining images (40X and 5X) of brains from TC-32 tumor bearing mice in (K). P values were calculated using two-way ANOVA with Bonferroni test (A), one-way ANOVA with Fisher's LSD test (C), two-way ANOVA (H) and unpaired two-tailed Mann-Whitney test (I).

MATERIALS AND METHODS

Sex as a biological variable

Only female nude mice were used in human Ewing sarcoma models to ensure data reproducibility. Sex was not considered as a biological variable, as Ewing sarcoma occurs in both sexes in humans.

Cell culture and transfection

Human Ewing sarcoma cell lines A673, MHH-ES-1 (from Dr. Ian Davis, UNC-Chapel Hill), TC-32 and TC-71 from Children's Oncology Group (2-5); human kidney cell lines HEK293T (ATCC) were cultured in DMEM with 10% FBS, 100 U/mL penicillin, and 100 μg/mL streptomycin.

To package lentiviral sgRNA plasmids, lentiviral plasmid, psPAX2 and pMD2.G vectors were transfected into 293T cells (40% confluence) in a ratio of 1:0.65:0.35 using PEI (23966, Polysciences). After overnight maintenance, medium was refreshed followed by virus supernatant collection after 24 and 48 h. Combined supernatant was used for cell infection after removing cell debris by 0.45 μm filter. Following viral infection, cells were maintained in the presence of 2 μg/mL of puromycin (BP2956100, Fisher BioReagents). Knockout efficiency of sgRNAs in cells was determined by WB.

Reagents

Irinotecan hydrochloride trihydrate (I0714) was purchased from TCI. Opicinumab (HY-P99780) and monomethyl auristatin E (MMAE, HY-15162) were purchased from MedChemExpress. Antibody-drug conjugates opicinumab-VC-PAB-SN38 (DAR=5.2) and opicinumab-VC-PAB-MMAE (DAR=4.6 and 4.1) were synthesized by CellMosaic. Drug conjugation was >99% without free drug detected.

sgRNAs

sgRNAs were constructed by inserting synthesized oligos into lentiCRISPRv2-puro vector.
sgLINGO1-1: TCTCAGCCAAGAGCAATGGG
sgLINGO1-2: AGGCCAGTGAAGACGCCTAG

Immunoblots

Cells were lysed in RIPA buffer (50 mM Tris pH 7.5, 150 mM NaCl, 1% Triton X-100, 1% sodium deoxycholate, 0.1% SDS) supplemented with protease and phosphatase inhibitors (Apexbio, K1008, K1015). Protein concentrations were measured using the Bio-Rad protein assay reagent on a NanoDrop OneC. Equal amounts of lysates were resolved by SDS-PAGE and immunoblotted with indicated antibodies. Anti-LINGO1 antibody (80965), anti-cleaved caspase-3 antibody (9661) and anti-cleaved PARP antibody (5625) were purchased from Cell Signaling Technology. Anti-α-tubulin antibody (sc-32293) was purchased from Santa Cruz Biotechnology.

Colony formation assays

Cells were seeded into 6-well plates (500 cells/well) and cultured in 37°C incubator with 5% CO_2 for 10 days when colonies were visible. Colonies were washed with PBS, fixed with methanol for 30 min and stained with 0.5% crystal violet for 30 min. Colonies were then washed by distilled water and air-dried. Colony numbers were manually counted.

FACS

To evaluate cell surface LINGO1 protein level, cells were scraped off, resuspended in FACS buffer (1% FBS, 0.1% sodium azide in PBS) and incubated with 10 μg/mL of LINGO1 antibody opicinumab or human IgG isotype control (02-7102, Invitrogen) for 90 min at 4 °C. Cells were washed twice with ice-cold FACS buffer and incubated with goat anti-human IgG (H+L) FITC conjugated antibody (A18806, Invitrogen) for 30 min at 4°C. Cells were washed once with FACS buffer, resuspended in 1 mL FACS buffer and analyzed by BD Fortessa. LINGO1 fluorescence was analyzed using Flowjo 10.8.1.

MTT assays

5,000 cells were seeded in each well of 96-well plates and incubated with indicated dose of opicinumab-SN38 for 2 or 3 days. After 3 h incubation with 10 uL MTT (5 mg/mL), medium was removed, formazan crystals were resolved by 150 µL DMSO and optical density at 570 nm was measured by the microplate reader.

Treatment studies in MHH-ES-1 xenograft mouse models

All mouse works have been reviewed and approved by UNC Institutional Animal Care and Use Committee under IACUC#25-017. 0.5×10^6 MHH-ES-1 cells suspended in 50% serum-free medium and 50% Geltrex Flex LDEV-Free Reduced Growth Factor Basement Membrane Matrix (A4000046703, Gibco) were subcutaneously inoculated to both flanks of 4-week-old female nude mice (Jackson Laboratory; n = 5 per group, 10 injections total). Mice were randomized into three treatment groups when tumors became palpable on day 7 or 8. Opicinumab-SN38 (DAR=5.2), irinotecan and PBS were intraperitoneal (i.p.) injected on day 8 as indicated. Opicinumab-MMAE, MMAE and PBS were i.p. injected as indicated on day 7, 9 and 10. Tumor dimensions were measured using calipers, and volumes were calculated as $V = L \times W^2 \times 0.5$. Mice were euthanized when bearing tumor size reached 2,000 mm^3 or when they lost >20% body weight. Tumors were excised and weighed.

Biodistribution study in TC-32 xenograft mouse model

5×10^6 TC-32 cells in 50% serum-free medium and 50% Geltrex Matrix were subcutaneously inoculated to both flanks of 4-week-old female nude mice (Jackson Laboratory). When average tumor volume reached around 200 mm^3, Opicinumab-MMAE (DAR=4.1) and PBS were i.p. injected as indicated on day 7, 8 and 9. Tumor volumes were calculated as $V = L \times W^2 \times 0.5$. Mice were euthanized on day 14. Tumors were excised and weighed. Tail vein blood (before euthanasia), tumor, liver and brain from five Opicinumab-MMAE-treated mice were collected and MMAE content in each sample was quantified by Drumetix Laboratories. To prepare serum samples, 200 µL of chilled acetonitrile was added to precipitate protein after the addition of 20 µL of DMSO:acetonitrile 1:1 (v/v) and 20 µL of 50 ng/mL D8-MMAE in DMSO:acetonitrile 1:1 (v/v) as internal standard (IS solution) into 20 µL of serum sample. For calibration standard samples, 20 µL of standard working solution in DMSO:acetonitrile 1:1 (v/v) (0.1, 0.3, 1, 3, 10, 30, 100, and 300 ng/mL) and 20 µL of IS solution were spiked into 20 µL of blank plasma. Then 200 µL of chilled acetonitrile was added to precipitate protein. Samples were vortexed and centrifuged at 3500 rpm for 10 min. Supernatant was injected onto an AB Sciex Triple Quad 5500 LC-MS/MS system coupled with a Shimadzu Prominence HPLC for analysis.

Tissue samples were homogenized with a QIAGEN TissueLyser II Sample Disruption Preparation Bead Mill after the addition of 70 µL of homogenization solvent (200 mM HOAc 100 mM NH4OAc 50 µM EDTA in Acetonitrile:Water 68:32) per 10 mg of tissue and three 3.175-mm (1/8 in) stainless steel balls. 20 µL of tissue homogenate is pipetted out for analysis. Blank tissue homogenate is prepared from tissue samples from vehicle dosing or no dosing in the same way. After the addition of 20 µL of DMSO:acetonitrile 1:1 (v/v), 20 µL of IS solution, and 100 µL of 1000 mM HOAc 500 mM NH4OAc 50 µM EDTA in water into 20 µL of tissue homogenate sample, 150 µL of chilled acetonitrile is added to precipitate protein. For calibration standard samples, 20 µL of standard working solution in DMSO:acetonitrile 1:1 (v/v) (0.1, 0.3, 1, 3, 10, 30, 100, and 300 ng/mL), 20 µL of IS solution, and 100 µL of 1000 mM HOAc 500 mM NH4OAc 50 µM EDTA in water are spiked into 20 µL of blank tissue homogenate. Then 150 µL of chilled acetonitrile is added to precipitate protein. Samples are vortexed and centrifuged at 3500 rpm for 30 min. Supernatant is injected onto an AB Sciex Triple Quad 5500 LC-MS/MS system coupled with a Shimadzu Prominence HPLC for analysis.

LC separation is performed on a Zorbax Extended-C18 (5 µm, 2.1x50 mm) column with 0.1% acetic acid 1 mM ammonium acetate in acetonitrile:water 1:9 as mobile phase A and 50 mM acetic acid in acetonitrile as mobile phase B. A gradient elution at 0.75 mL/min started with 10% B. B component is increased linearly to 95% in 1 minutes. After holding at 95% B for 0. 5 minutes, the column is re-equilibrated with 0% B for 0.5 minutes. Mass spectrometric detection is performed with TurboSpray

ionization in positive ion mode as shown below.

HPLC Instrument:	Shimadzu LC-20AD Pumps and PE 200 Autosampler								
Column:	Zorbax Extended-C18, 5 µm, 2.1x50 mm				Flow Rate (mL/min)	Diverting to Waste (min)	SIL-20AC Cooler Temperature (°C)	Run Time (min)	
MPA:	0.1% Acetic Acid 1 mM Ammonium Acetate in Acetonitrile:Water 1:9							Last gradient time point shown below	
MPB:	50 mM Acetic Acid in Acetonitrile				0.75	0-0.5	NA		
Gradient for MMAE	Time (min)	0	1	1.5	1.51	2			
	% B	10	95	95	10	10			
AB Sciex Triple Quad 5500	Ion Source	Scan Type	Polarity	CUR	CAD	IS	TEM	GS1	GS2
	Turbo Spray	MRM	Positive	30.00	9.00	5000.00	500.00	50.00	50.00
		Q1	Q3	Time (msec)	DP	EP	CE	CXP	Injection Volume (µL)
MMAE		718.43	686.50	150.00	201.00	10.00	41.00	24.00	2.00
D8-MMAE (IS)		726.40	694.50	50.00	201.00	10.00	41.00	24.00	NA
Software: Analyst 1.7.2									

Pharmacokinetic study and toxicology assays in TC-32 xenograft mouse model

$2.5×10^6$ TC-32 cells in 50% serum-free medium and 50% Geltrex Matrix were subcutaneously inoculated to both flanks of 4-week-old female nude mice (Jackson Laboratory). Opicinumab-MMAE (DAR=4.1), MMAE, irinotecan and PBS were i.p. injected on day 7 as indicated when average tumor volume reached around 100 mm^3. Tail vein blood samples were collected from 4 Opicinumab-MMAE-treated mice at each time point (10 h, 1, 2, 4 and 8 days) and mixed with 10% 50 mM EDTA in PBS. After removing cells by centrifugation at 4000 × g at 4 °C for 15 min, plasma samples were stored at −80 °C until used for subsequent sandwich ELISA. Tumor volumes were calculated as $V = L × W^2 × 0.5$. Mice were euthanized on day 18 (11 days after injection). Toxicology-related analyses included alanine aminotransferase (ALT) blood test and histopathological evaluation of brain with high LINGO1 protein level. Blood from all groups was collected and serum after clotting at room temperature for 30 min and centrifugating at 2000 × g for 15 min were used for ALT test following the protocol of Alanine Transaminase Colorimetric Activity Assay Kit (700260, Cayman). Brain from vehicle and Opicinumab-MMAE groups was collected for HE staining and immunohistochemistry (IHC).

To determine the total antibody concentration (both conjugated Opicinumab-MMAE and unconjugated Opicinumab), a high-binding 96-well half-area microplate (Corning, CLS3690) was coated with goat anti-human IgG Fcγ antibody (250 ng/well) (Jackson ImmunoResearch, 109-005-098) diluted in PBS. After overnight incubation at 4 °C, the plate was washed by PBST (0.05% Tween 20 in PBS) three times and was blocked with 150 µL of 1% BSA in PBS at room temperature (RT) for 1 h. After washing the plate three times by PBST, each diluted plasma sample (1:20000) and standard Opicinumab samples (50 µL in 1% BSA in PBS) was added to each well and incubated at RT for 2 h. After washing the plate three times by PBST, 50 µL of goat anti-human IgG Fab-HRP conjugate (109-035-097) (1 : 5000, in 1% BSA in PBS) was added and incubated at RT for 1 h. After washing the plate three times by PBST, 50 µL of TMB ELISA substrate solution (ThermoFisher Scientific, 34021) was added and incubated at RT for 20 min. Reaction was stopped by adding 25 µL of 2N sulfuric acid (Fisher Chemical, A300-212). OD450 and OD570 was recorded immediately.

To determine conjugated Opicinumab-MMAE concentration, assays were performed in the same manner using goat anti-human IgG Fcγ antibody (250 ng/well) for coating, mouse anti-MMAE antibody (Genscript, A02224-40; 200 ng/ml) and goat anti-mouse IgG, Fcγ-HRP conjugate (Jackson ImmunoResearch, 115-035-071; 1:10000) as secondary and tertiary detection antibodies. All assays were performed in duplicate. Concentrations were calculated based on standard curves. DAR of

Opicinumab-MMAE was regarded as 4.1 (fully conjugated) or 0 (fully unconjugated). Conjugated Opicinumab-MMAE concentration was normalized by dividing the absolute readout by 4.1. Half-life of elimination phase ($t_{1/2\beta}$) and area under the curve ($AUC_{10\text{-}192}$, $h \times$ μg/mL) were estimated by Graphpad.

IHC

IHC was performed as previously described (6) using anti-cleaved caspase-3 (Cell Signaling, 9661) and anti-tubulin β-3 antibody (BioLegend, 802001).

H&E staining

Deparaffinize and rehydrate slides to distilled water. Stain in Hematoxylin++ (Fisher, 22-220-109) for 1 min. Wash with 4-5 changes of tap water. Blue nuclei in 1X PBS for 1 min. Wash with 3 changes of distilled water. Counterstain in Alcoholic-Eosin for 1 min. Dehydrate through 3 changes of 95% ethanol and 2 changes of 100% ethanol 1 min each. Clear in 3 changes of Xylene. Mount by Permount (Fisher, SP15-100) and coverslip.

PDX scRNA-Seq data analysis

We downloaded the scRNA-seq data of 5 PDXs from *EWS::FL1*-positive EwS that were generated using the 10x genomics sequencing platform by Aynaud et al. (1), which is available at Gene Expression Omnibus under accession number GSE130025. Following the quality control procedures in (1), we removed cells with too few (<5,000) or too many (> 40,000) total unique molecular identifiers (UMIs), and cells with high percentage of mitochondrial gene expression (>10%) from analysis, leaving 9,654 high-quality scRNA-seq profiles from 5 PDX samples. The standard Seurat pipeline (7) was used to analyze and visualize the filtered scRNA-seq data, including normalization of UMI counts for library size and log transformation, selection of the 10,000 most variable genes for scaling, linear dimensionality reduction using PCA on the scaled data, followed by *t*-SNE visualization using the top 50 principal components.

Statistical Analysis

Statistical analyses were performed using GraphPad Prism 8. Two-group nonparametric comparison used two-tailed Mann-Whitney tests. For three or more groups, parametric comparison used one- or two-way ANOVA with Dunnett's, Tukey's, Fisher's LSD or Bonferroni post hoc tests as appropriate; nonparametric comparison used Kruskal-Wallis with Dunn's test. P value less than 0.05 was considered significant. Results are shown as mean ± SD from representative experiments repeated three times, or as mean ± SEM from biological replicates.

Study approval

All mouse studies were reviewed and approved by the UNC Institutional Animal Care and Use Committee (IACUC #25-017.0). Experiments were conducted in the Genetic Medicine Animal Facility at UNC-Chapel Hill, an Office of Laboratory Animal Welfare–assured and AAALAC-accredited facility, following IACUC-approved protocols and in compliance with the Guide for the Care and Use of Laboratory Animals (National Research Council, 2011).

Data availability

All reported data values are available in the Supporting Data Values file. "Supporting data values" tables have been included in the supplement. All other data supporting the findings in this study are available from the corresponding authors upon reasonable request.

Acknowledgements

We sincerely thank Dr. Eva Anton Lab at UNC-Chapel Hill for sharing the anti-tubulin β-3 antibody.

References:

1. Aynaud MM, Mirabeau O, Gruel N, Grossetete S, Boeva V, Durand S, et al. Transcriptional Programs Define Intratumoral Heterogeneity of Ewing Sarcoma at Single-Cell Resolution. *Cell Rep.* 2020;30(6):1767-79 e6.
2. Batra S, Reynolds CP, and Maurer BJ. Fenretinide cytotoxicity for Ewing's sarcoma and primitive neuroectodermal tumor cell lines is decreased by hypoxia and synergistically enhanced by ceramide modulators. *Cancer Res.* 2004;64(15):5415-24.
3. Wang Y, Einhorn P, Triche TJ, Seeger RC, and Reynolds CP. Expression of protein gene product 9.5 and tyrosine hydroxylase in childhood small round cell tumors. *Clin Cancer Res.* 2000;6(2):551-8.
4. Thiele CJ, McKeon C, Triche TJ, Ross RA, Reynolds CP, and Israel MA. Differential protooncogene expression characterizes histopathologically indistinguishable tumors of the peripheral nervous system. *J Clin Invest.* 1987;80(3):804-11.
5. Whang-Peng J, Triche TJ, Knutsen T, Miser J, Kao-Shan S, Tsai S, et al. Cytogenetic characterization of selected small round cell tumors of childhood. *Cancer Genet Cytogenet.* 1986;21(3):185-208.
6. Zhu Z, Zhou X, Xu M, Chen J, Robertson KC, Atassi G, et al. Molecular glue degrader function of SPOP inhibitors enhances STING-dependent immunotherapy efficacy in melanoma models. *J Clin Invest.* 2025;135(24).
7. Stuart T, Butler A, Hoffman P, Hafemeister C, Papalexi E, Mauck WM, 3rd, et al. Comprehensive Integration of Single-Cell Data. *Cell.* 2019;177(7):1888-902 e21.

Author contributions:

Designing research studies (Z.Z. and P.L.), conducting experiments (Z.Z., Y.D., Z.L.), acquiring data (Z.Z., Y.D., Z.L.), analyzing data (Z.Z., Y.L.), providing reagents (A.S.B.), and writing the manuscript (Z.Z. and P.L.).

Table S1. Protein Atlas proteins with plasma membrane localization

Gene	Gene synonym	Ensembl	Gene description	Uniprot	Chromosome	Position
**AAK1**	DKFZp686K16132, KIAA1048	ENSG00000115977	AP2 associated kinase 1	Q2M2I8	2	69457997-69674349
**AAMP**		ENSG00000127837	Angio associated migratory cell protein	Q13685	2	218264129-218270178
**ABCA1**	ABC1, HDLDT1, TGD	ENSG00000165029	ATP binding cassette subfamily A member 1	O95477	9	104781006-104928155
**ABCA7**	ABCX	ENSG00000064687	ATP binding cassette subfamily A member 7	Q8IZY2	19	1039997-1065572
**ABCB1**	ABC20, CD243, CLCS, GP170, MDR1, p-170, P-gp, PGY1	ENSG00000085563	ATP binding cassette subfamily B member 1	P08183	7	87503017-87713323
**ABCB4**	GBD1, MDR2, MDR3, PFIC-3, PGY3	ENSG00000005471	ATP binding cassette subfamily B member 4	P21439	7	87401696-87480435
**ABCB6**	EST45597, MTABC3, umat	ENSG00000115657	ATP binding cassette subfamily B member 6 (Langereis blood group)	Q9NP58	2	219209772-219218994
**ABCC1**	GS-X, MRP, MRP1	ENSG00000103222	ATP binding cassette subfamily C member 1	P33527	16	15949138-16143257
**ABCC3**	cMOAT2, EST90757, MLP2, MOAT-D, MRP3	ENSG00000108846	ATP binding cassette subfamily C member 3	O15438	17	50634777-50692253
**ABCC4**	EST170205, MOAT-B, MOATB, MRP4	ENSG00000125257	ATP binding cassette subfamily C member 4	O15439	13	95019835-95301475
**ABCC6**	ARA, EST349056, MLP1, MRP6, PXE, URG7	ENSG00000091262	ATP binding cassette subfamily C member 6	O95255	16	16149565-16223522
**ABCG2**	ABCP, BCRP, CD338, EST157481, MXR	ENSG00000118777	ATP binding cassette subfamily G member 2 (Junior blood group)	Q9UNQ0	4	88090150-88231628
**ACKR3**	CMKOR1, CXCR7, GPR159, RDC1	ENSG00000144476	Atypical chemokine receptor 3	P25106	2	236567787-236582354
**ADA**	ADA1	ENSG00000196839	Adenosine deaminase	P00813	20	44584896-44652252
**ADAM10**	CD156C, HsT18717, kuz, MADM	ENSG00000137845	ADAM metallopeptidase domain 10	O14672	15	58588809-58749791
**ADAM12**	MCMPMltna, MLTN	ENSG00000148848	ADAM metallopeptidase domain 12	O43184	10	126012381-126388477
**ADAM28**	ADAM23, eMDCII, MDC-Lm, MDC-Ls	ENSG00000042980	ADAM metallopeptidase domain 28	Q9UKQ2	8	24294069-24359014
**ADCY3**	AC3	ENSG00000138031	Adenylate cyclase 3	O60266	2	24819169-24920237
**ADCY4**	AC4	ENSG00000129467	Adenylate cyclase 4	Q8NFM4	14	24318349-24335093
**ADCY9**	AC9	ENSG00000162104	Adenylate cyclase 9	O60503	16	3953387-4116442
**ADD1**		ENSG00000087274	Adducin 1	P35611	4	2843844-2930076
**ADD2**	ADDB	ENSG00000075340	Adducin 2	P35612	2	70607618-70768225
**ADD3**	ADDL	ENSG00000148700	Adducin 3	Q9UEY8	10	109996368-110135565
**ADGRA2**	DKFZp434C211, DKFZp434J0911, FLJ14390, GPR124, KIAA1531, TEM5	ENSG00000020181	Adhesion G protein-coupled receptor A2	Q96PE1	8	37784191-37844896
**ADGRB2**	BAI2	ENSG00000121753	Adhesion G protein-coupled receptor B2	O60241	1	31727117-31764893
**ADGRD1**	DKFZp434B1272, GPR133, PGR25	ENSG00000111452	Adhesion G protein-coupled receptor D1	Q6QNK2	12	130953907-131141469
**ADGRG2**	EDDM6, GPR64, HE6, TM7LN2	ENSG00000173698	Adhesion G protein-coupled receptor G2	Q8IZP9	X	18989307-19122637
**ADORA1**	RDC7	ENSG00000163485	Adenosine A1 receptor	P30542	1	203090654-203167405
**ADORA2B**		ENSG00000170425	Adenosine A2b receptor	P29275	17	15945130-15975746
**ADRA1B**		ENSG00000170214	Adrenoceptor alpha 1B	P35368	5	159865080-159973012
**ADRB2**	ADRB2R, ADRBR, B2AR, BAR	ENSG00000169252	Adrenoceptor beta 2	P07550	5	148826611-148828623
**AFDN**	AF-6, AF6, MLLT4	ENSG00000130396	Afadin, adherens junction formation factor	P55196	6	167826893-167972023
**AGER**	RAGE, SCARJ1, sRAGE	ENSG00000204305	Advanced glycosylation end-product specific receptor	Q15109	6	32180968-32184322
**AHNAK**	AHNAK1, MGC5395	ENSG00000124942	AHNAK nucleoprotein	Q09666	11	62433542-62556235
**AHNAK2**	C14orf78	ENSG00000185567	AHNAK nucleoprotein 2	Q8IVF2	14	104937244-104978374
**AKAP12**	AKAP250, SSeCKS	ENSG00000131016	A-kinase anchoring protein 12	Q02952	6	151239967-151358559
**AKR1B10**	AKR1B11, AKR1B12, ALDRLn, ARL-1, ARL1, HIS, HSI	ENSG00000198074	Aldo-keto reductase family 1 member B10	O60218	7	134527567-134541412
**AKR1B15**		ENSG00000227471	Aldo-keto reductase family 1 member B15	C9JRZ8	7	134549110-134579875
**AKTIP**	FLJ13258, FTS	ENSG00000166971	AKT interacting protein	Q9H8T0	16	53491040-53504411
**ALK**	ALK1, CD246	ENSG00000171094	ALK receptor tyrosine kinase	Q9UM73	2	29192774-29921586
**ALOX15B**	15-LOX-2	ENSG00000179593	Arachidonate 15-lipoxygenase type B	O15296	17	8039034-8049134
**AMER1**	FAM123B, FLJ39827, RP11-403E24.2, WTX	ENSG00000184675	APC membrane recruitment protein 1	Q5JTC6	X	64185117-64205708
**AMER2**	FAM123A, FLJ25477	ENSG00000165566	APC membrane recruitment protein 2	Q8N7J2	13	25161679-25172288
**AMOT**	KIAA1071	ENSG00000126016	Angiomotin	Q4VCS5	X	112774503-112840831
**ANK2**	CFAP87, FAP87, LQT4	ENSG00000145362	Ankyrin 2	Q01484	4	112818032-113384221
**ANK3**		ENSG00000151150	Ankyrin 3	Q12955	10	60026298-60733490
**ANKIB1**	DKFZP434A0225, KIAA1386	ENSG00000001629	Ankyrin repeat and IBR domain containing 1	Q9P2G1	7	92245974-92401383
**ANKRA2**		ENSG00000164331	Ankyrin repeat family A member 2	Q9H9E1	5	73552190-73565667
**ANKRD13A**	ANKRD13, NY-REN-25	ENSG00000076513	Ankyrin repeat domain 13A	Q8IZ07	12	109999186-110039763
**ANKRD13B**	FLJ25555	ENSG00000198720	Ankyrin repeat domain 13B	Q86YJ7	17	29589769-29614761
**ANO1**	DOG1, FLJ10261, ORAOV2, TAOS2, TMEM16A	ENSG00000131620	Anoctamin 1	Q5XXA6	11	69985907-70189530
**ANO10**	FLJ10375, MGC47890, SCAR10, TMEM16K	ENSG00000160746	Anoctamin 10	Q9NW15	3	43354859-43691594
**ANO2**	C12orf3, TMEM16B	ENSG00000047617	Anoctamin 2	Q9NQ90	12	5531869-5946232
**ANO6**	DKFZp313M0720, TMEM16F	ENSG00000177119	Anoctamin 6	Q4KMQ2	12	45215987-45482280
**ANPEP**	AP-N, CD13, gp150, hAPN, LAP1, p150, PEPN	ENSG00000166825	Alanyl aminopeptidase, membrane	P15144	15	89784895-89815401
**ANXA1**	ANX1, LPC1	ENSG00000135046	Annexin A1	P04083	9	73151865-73170393
**ANXA13**	ANX13	ENSG00000104537	Annexin A13	P27216	8	123680794-123737402
**ANXA3**	ANX3	ENSG00000138772	Annexin A3	P12429	4	78551747-78610451
**AP2M1**	AP50, CLAPM1, mu2	ENSG00000161203	Adaptor related protein complex 2 subunit mu 1	Q96CW1	3	184174689-184184214
**APBB1**	Fe65, RIR	ENSG00000166313	Amyloid beta precursor protein binding family B member 1	O00213	11	6395125-6419414
**APBB1IP**	INAG1, RIAM	ENSG00000077420	Amyloid beta precursor protein binding family B member 1 interacting protein	Q7Z5R6	10	26438341-26567803
**APC**	DP2, DP2.5, DP3, PPP1R46	ENSG00000134982	APC regulator of WNT signaling pathway	P25054	5	112707498-112846239
**APOBR**	APOB100R, APOB48R	ENSG00000184730	Apolipoprotein B receptor	Q0VD83	16	28494643-28498964
**APOLD1**	DKFZP434F0318, FLJ25138	ENSG00000178878	Apolipoprotein L domain containing 1	Q96LR9	12	12725917-12829975
**APPL1**	APPL	ENSG00000157500	Adaptor protein, phosphotyrosine interacting with PH domain and leucine zipp	Q9UKG1	3	57227726-57278105
**AQP1**	CHIP28, CO	ENSG00000240583	Aquaporin 1 (Colton blood group)	P29972	7	30911853-30925517
**AQP10**		ENSG00000143595	Aquaporin 10	Q96PS8	1	154321090-154325325
**AQP11**	AQPX1	ENSG00000178301	Aquaporin 11	Q8NBQ7	11	77589391-77610356
**AQP3**	GIL	ENSG00000165272	Aquaporin 3 (Gill blood group)	Q92482	9	33441156-33447596
**AQP4**	AQP-4, hAQP4, MIWC	ENSG00000171885	Aquaporin 4	P55087	18	26852043-26865771
**ARAF**	ARAF1	ENSG00000078061	A-Raf proto-oncogene, serine/threonine kinase	P10398	X	47561205-47571908
**ARHGAP15**	BM046	ENSG00000075884	Rho GTPase activating protein 15	Q53QZ3	2	143091362-143768352
**ARHGAP17**	FLJ10308, FLJ13219, NADRIN, RICH1, WBP15	ENSG00000140750	Rho GTPase activating protein 17	Q68EM7	16	24919389-25015666
**ARHGAP19**	FLJ00194, MGC14258	ENSG00000213390	Rho GTPase activating protein 19	Q14CB8	10	97222173-97292673
**ARHGAP21**	ARHGAP10, KIAA1424	ENSG00000107863	Rho GTPase activating protein 21	Q5T5U3	10	24583609-24723887
**ARHGAP22**	RhoGAP2	ENSG00000128805	Rho GTPase activating protein 22	Q7Z5H3	10	48446036-48656265
**ARHGAP45**	HA-1, HMHA1, KIAA0223	ENSG00000180448	Rho GTPase activating protein 45	Q92619	19	1065923-1086628
**ARHGEF1**	LBCL2, P115-RHOGEF, SUB1.5	ENSG00000076928	Rho guanine nucleotide exchange factor 1	Q92888	19	41883173-41930150
**ARHGEF11**	GTRAP48, KIAA0380, PDZ-RHOGEF	ENSG00000132694	Rho guanine nucleotide exchange factor 11	O15085	1	156934840-157045742
**ARHGEF15**	ARGEF15, FLJ13791, KIAA0915, MGC44868, Vsm-RhoGEF	ENSG00000198844	Rho guanine nucleotide exchange factor 15	O94989	17	8310241-8322514
**ARHGEF18**	KIAA0521, MGC15913, P114-RhoGEF, p114RhoGEF	ENSG00000104880	Rho/Rac guanine nucleotide exchange factor 18	Q6ZSZ5	19	7348937-7472485
**ARHGEF26**	DKFZP434D146, SGEF	ENSG00000114790	Rho guanine nucleotide exchange factor 26	Q96DR7	3	154121003-154257827
**ARHGEF5**	GEF5, P60, TIM, TIM1	ENSG00000050327	Rho guanine nucleotide exchange factor 5	Q12774	7	144355288-144380632
**ARL4C**	ARL7, LAK	ENSG00000188042	ADP ribosylation factor like GTPase 4C	P56559	2	234493041-234497081
**ARMH1**	C1orf228, MGC33556, NCRNA00082, p40	ENSG00000198520	Armadillo like helical domain containing 1	Q6PIY5	1	44674692-44725591
**ARRB2**	ARR2, BARR2, DKFZp686L0365	ENSG00000141480	Arrestin beta 2	P32121	17	4710596-4721499
**ARRDC4**	FLJ36045	ENSG00000140450	Arrestin domain containing 4	Q8NCT1	15	97960703-97973833
**ARVCF**		ENSG00000099889	ARVCF delta catenin family member	O00192	22	19969896-20016823
**ASAP2**	CENTB3, DDEF2, KIAA0400, PAP, SHAG1	ENSG00000151693	ArfGAP with SH3 domain, ankyrin repeat and PH domain 2	O43150	2	9206765-9405683
**ASGR2**	CLEC4H2	ENSG00000161944	Asialoglycoprotein receptor 2	P07307	17	7101322-7115700
**ASH2L**	ASH2, ASH2L1, ASH2L2, Bre2	ENSG00000129691	ASH2 like, histone lysine methyltransferase complex subunit	Q9UBL3	8	38105493-38144076
**ASIC1**	ACCN2, BNaC2, hBNaC2	ENSG00000110881	Acid sensing ion channel subunit 1	P78348	12	50057548-50083611
**ATG3**	APG3L, DKFZp564M1178, FLJ22125, MGC15201, PC3-96	ENSG00000144848	Autophagy related 3	Q9NT62	3	112532510-112562046
**ATP10D**	ATPVD, KIAA1487	ENSG00000145246	ATPase phospholipid transporting 10D (putative)	Q9P241	4	47485275-47593486
**ATP12A**	ATP1AL1, H-K-ATPase, HK	ENSG00000075673	ATPase H+/K+ transporting non-gastric alpha2 subunit	P54707	13	24680408-24712472
**ATP1B3**	CD298, FLJ29027	ENSG00000069849	ATPase Na+/K+ transporting subunit beta 3	P54709	3	141876124-141926549
**ATP23**	KUB3, XRCC6BP1	ENSG00000166896	ATP23 metallopeptidase and ATP synthase assembly factor homolog	Q9Y6H3	12	57906039-57959148
**ATP2B1**	PMCA1	ENSG00000070961	ATPase plasma membrane Ca2+ transporting 1	P20020	12	89588049-89709366
**ATP2B3**	CFAP39, CLA2, PMCA3, SCAX1	ENSG00000067842	ATPase plasma membrane Ca2+ transporting 3	Q16720	X	153517642-153582939
**ATP2B4**	ATP2B2, MXRA1, PMCA4	ENSG00000058668	ATPase plasma membrane Ca2+ transporting 4	P23634	1	203626832-203744081
**ATP6V0A2**	a2, a2V, ATP6a2, ATP6N1D, J6B7, RTF, Stv1, TJ6, TJ6M, TJ6s, Vph1	ENSG00000185344	ATPase H+ transporting V0 subunit a2	Q9Y487	12	123712353-123761755
**ATP6V1D**	ATP6M, VATD, VMA8	ENSG00000100554	ATPase H+ transporting V1 subunit D	Q9Y5K8	14	67294371-67360265
**ATP8A2**	ATPIB, ML-1	ENSG00000132932	ATPase phospholipid transporting 8A2	Q9NTI2	13	25371974-26025851
**ATP8B1**	ATPIC, BRIC, FIC1, PFIC, PFIC1	ENSG00000081923	ATPase phospholipid transporting 8B1	O43520	18	57646426-57803315
**ATP8B2**	ATPID, KIAA1137	ENSG00000143515	ATPase phospholipid transporting 8B2	P98198	1	154325525-154351304
**AXL**	ARK, JTK11, Tyro7, UFO	ENSG00000167601	AXL receptor tyrosine kinase	P30530	19	41219223-41261766
**B2M**		ENSG00000166710	Beta-2-microglobulin	P61769	15	44711358-44718851
**BACE1**	BACE	ENSG00000186318	Beta-secretase 1	P56817	11	117285232-117316259
**BAIAP2**	BAP2, IRSp53, WAML	ENSG00000175866	BAR/IMD domain containing adaptor protein 2	Q9UQB8	17	81035122-81117434
**BAIAP2L1**	IRTKS	ENSG00000006453	BAR/IMD domain containing adaptor protein 2 like 1	Q9UHR4	7	98291650-98401090
**BASP1**	CAP-23, CAP23, NAP-22, NAP22	ENSG00000176788	Brain abundant membrane attached signal protein 1	P80723	5	17065598-17276843
**BDKRB2**	BK-2	ENSG00000168398	Bradykinin receptor B2	P30411	14	96204679-96244166
**BFSP1**	CP115, CP94, filensin, LIFL-H	ENSG00000125864	Beaded filament structural protein 1	Q12934	20	17493905-17569220
**BIN2**	BRAP-1	ENSG00000110934	Bridging integrator 2	Q9UBW5	12	51281038-51324668
**BLNK**	BASH, bca, BLNK-s, Ly57, SLP-65, SLP65	ENSG00000095585	B cell linker	Q8WV28	10	96189171-96271587
**BMPR2**	BMPR-II, BMPR3, BRK-3, PPH1, T-ALK	ENSG00000204217	Bone morphogenetic protein receptor type 2	Q13873	2	202376327-202567751
**BMX**	ETK, PSCTK3	ENSG00000102010	BMX non-receptor tyrosine kinase	P51813	X	15464246-15556529
**BOC**	Boi, CDON2	ENSG00000144857	BOC cell adhesion associated, oncogene regulated	Q9BWV1	3	113211003-113287459
**BRAF**	BRAF-1, BRAF1	ENSG00000157764	B-Raf proto-oncogene, serine/threonine kinase	P15056	7	140719327-140924929
**BTK**	AGMX1, ATK, IMD1, PSCTK1, XLA	ENSG00000010671	Bruton tyrosine kinase	Q06187	X	101349338-101390796
**BVES**	HBVES, POP1, POPDC1	ENSG00000112276	Blood vessel epicardial substance	Q8NE79	6	105096822-105137157
**C11orf52**	FLJ25219, MGC14839	ENSG00000149300	Chromosome 11 open reading frame 52	Q96A22	11	111918032-111926871
**C1orf116**	FLJ36507, MGC2742, MGC4309, SARG	ENSG00000182795	Chromosome 1 open reading frame 116	Q9BW04	1	207018522-207032756
**C1QBP**	gC1Q-R, gC1qR, HABP1, p32, SF2p32	ENSG00000108561	Complement C1q binding protein	Q07021	17	5432777-5448830
**C2CD2L**	KIAA0285, TMEM24	ENSG00000172375	C2CD2 like	O14523	11	119102198-119118544
**C8A**		ENSG00000157131	Complement C8 alpha chain	P07357	1	56854768-56918223

**CA9**	CAIX, MN	ENSG00000107159	Carbonic anhydrase 9	Q16790		9	35673928-35681159
**CACNA1C**	CACH2, CACN2, CACNL1A1, Cav1.2, CCHL1A1, LQT8, TS	ENSG00000151067	Calcium voltage-gated channel subunit alpha1 C	Q13936		12	1970772-2697950
**CADM1**	BL2, IGSF4, IGSF4A, Necl-2, NECL2, RA175, ST17, SYNCAM, SYNCAM1,	ENSG00000182985	Cell adhesion molecule 1	Q9BY67		11	115169218-115504957
**CALCRL**	CGRPR, CRLR	ENSG00000064989	Calcitonin receptor like receptor	Q16602		2	187341964-187448460
**CALD1**	CDM, H-CAD, h-CD, L-CAD	ENSG00000122786	Caldesmon 1	Q05682		7	134744252-134970729
**CARMIL1**	CARMIL, dJ501N12.1, FLJ20048, LRRC16, LRRC16A	ENSG00000079691	Capping protein regulator and myosin 1 linker 1	Q5VZK9		6	25279078-25620530
**CASKIN2**	ANKS5B, FLJ21609, KIAA1139	ENSG00000177303	CASK interacting protein 2	Q8WXE0		17	75500261-75515537
**CAST**		ENSG00000153113	Calpastatin	P20810		5	96525267-96779595
**CAVIN1**	cavin-1, CGL4, PTRF	ENSG00000177469	Caveolae associated protein 1	Q6NZI2		17	42402449-42423256
**CAVIN2**	cavin-2, PS-p68, SDPR, SDR	ENSG00000168497	Caveolae associated protein 2	O95810		2	191834310-191847088
**CAVIN4**	cavin-4, MURC	ENSG00000170681	Caveolae associated protein 4	Q5BKX8		9	100578079-100588389
**CCDC115**	ccp1, FLJ30131, MGC12981	ENSG00000136710	Coiled-coil domain containing 115	Q96NT0		2	130337933-130342699
**CCDC124**	Lso2, oxs1	ENSG00000007080	Coiled-coil domain containing 124	Q96CT7		19	17933015-17943991
**CCDC85C**		ENSG00000205476	Coiled-coil domain containing 85C	A6NKD9		14	99500190-99604207
**CCDC88A**	APE, FLJ10392, GIV, GRDN, HkRP1, KIAA1212	ENSG00000115355	Coiled-coil domain containing 88A	Q3V6T2		2	55287842-55419895
**CCDC88C**	DAPLE, HkRP2, KIAA1509, SCA40	ENSG00000015133	Coiled-coil domain containing 88C	Q9P219		14	91271323-91417844
**CCDC93**	FLJ10996	ENSG00000125633	Coiled-coil domain containing 93	Q567U6		2	117915478-118014133
**CCKAR**		ENSG00000163394	Cholecystokinin A receptor	P32238		4	26481396-26490484
**CCKBR**		ENSG00000110148	Cholecystokinin B receptor	P32239		11	6259806-6272127
**CCNYL1**	FLJ40432	ENSG00000163249	Cyclin Y like 1	Q8N7R7		2	207711540-207761839
**CCR2**	CC-CKR-2, CD192, CKR2, CMKBR2, FLJ78302, MCP-1-R	ENSG00000121807	C-C motif chemokine receptor 2	P41597		3	46353864-46360940
**CCR4**	CC-CKR-4, CD194, ChemR13, CKR4, CMKBR4, k5-5	ENSG00000183813	C-C motif chemokine receptor 4	P51679		3	32951644-32956349
**CCRL2**	ACKR5, CKRX, CRAM-A, CRAM-B, HCR	ENSG00000121797	C-C motif chemokine receptor like 2	O00421		3	46407166-46412997
**CD109**	CPAMD7, DKFZp762L1111, FLJ38569	ENSG00000156535	CD109 molecule	Q6YHK3		6	73695785-73828316
**CD163**	M130, MM130, SCARI1	ENSG00000177575	CD163 molecule	Q86VB7		12	7470811-7503893
**CD180**	LY64, Ly78, RP105	ENSG00000134061	CD180 molecule	Q99467		5	67179613-67196799
**CD2**	SRBC	ENSG00000116824	CD2 molecule	P06729		1	116754430-116769229
**CD200R1**	CD200R, HCRTR2, MOX2R, OX2R	ENSG00000163606	CD200 receptor 1	Q8TD46		3	112921205-112975103
**CD226**	DNAM-1, DNAM1, PTA1, TLiSA1	ENSG00000150637	CD226 molecule	Q15762		18	69831158-69961803
**CD274**	B7-H, B7-H1, B7H1, PD-L1, PDCD1LG1, PDL1	ENSG00000120217	CD274 molecule	Q9NZQ7		9	5450503-5470566
**CD28**		ENSG00000178562	CD28 molecule	P10747		2	203706475-203738912
**CD2AP**	CMS	ENSG00000198087	CD2 associated protein	Q9Y5K6		6	47477789-47627263
**CD33**	FLJ00391, p67, SIGLEC-3, SIGLEC3	ENSG00000105383	CD33 molecule	P20138		19	51225064-51243860
**CD38**	cADPR1	ENSG00000004468	CD38 molecule	P28907		4	15778275-15853232
**CD3E**	CD3-epsilon, CD3epsilon	ENSG00000198851	CD3 epsilon subunit of T-cell receptor complex	P07766		11	118304730-118316175
**CD3G**	CD3-GAMMA, CD3GAMMA	ENSG00000160654	CD3 gamma subunit of T-cell receptor complex	P09693		11	118344344-118355161
**CD4**		ENSG00000010610	CD4 molecule	P01730		12	6786858-6820799
**CD40**	Bp50, p50, TNFRSF5	ENSG00000101017	CD40 molecule	P25942		20	46118271-46129863
**CD40LG**	CD154, CD40-L, CD40L, gp39, hCD40L, HIGM1, IMD3, T-BAM, TNFSF5, T	ENSG00000102245	CD40 ligand	P29965	X		136648158-136660390
**CD44**	CD44R, CSPG8, HCELL, IN, MC56, MDU2, MDU3, MIC4, Pgp1	ENSG00000026508	CD44 molecule (Indian blood group)	P16070		11	35138882-35232402
**CD47**	IAP, MER6, OA3	ENSG00000196776	CD47 molecule	Q08722		3	108043091-108091862
**CD79B**	B29, Ig-beta, IGB, Igbeta	ENSG00000007312	CD79b molecule	P40259		17	63928738-63932336
**CD84**	hCD84, mCD84, SLAMF5	ENSG00000066294	CD84 molecule	Q9UIB8		1	160541095-160579516
**CD86**	B7-2, B7.2, CD28LG2	ENSG00000114013	CD86 molecule	P42081		3	122055362-122121139
**CD8A**	CD8	ENSG00000153563	CD8a molecule	P01732		2	86784610-86808396
**CD8B**	CD8B1	ENSG00000172116	CD8b molecule	P10966		2	86815339-86861924
**CD9**	BA2, MIC3, MRP-1, P24, TSPAN29	ENSG00000010278	CD9 molecule	P21926		12	6199715-6238271
**CDAN1**	CDA-I, CDAI	ENSG00000140326	Codanin 1	Q8IWY9		15	42723544-42737128
**CDC42EP1**	Borg5, CEP1, MSE55	ENSG00000128283	CDC42 effector protein 1	Q00587		22	37560480-37569405
**CDC42EP3**	BORG2, CEP3, UB1	ENSG00000163171	CDC42 effector protein 3	Q9UKI2		2	37641882-37738468
**CDC42SE1**	SCIP1, SPEC1	ENSG00000197622	CDC42 small effector 1	Q9NRR8		1	151050971-151070325
**CDH1**	CD324, UVO, uvomorulin	ENSG00000039068	Cadherin 1	P12830		16	68737292-68835537
**CDH13**	CDHH	ENSG00000140945	Cadherin 13	P55290		16	82626965-83800640
**CDH15**	CDH14, CDH3	ENSG00000129910	Cadherin 15	P55291		16	89171748-89195492
**CDH17**	cadherin, HPT-1	ENSG00000079112	Cadherin 17	Q12864		8	94127162-94217303
**CDH2**	CD325, CDHN, NCAD	ENSG00000170558	Cadherin 2	P19022		18	27932879-28177946
**CDH26**	VR20	ENSG00000124215	Cadherin 26	Q8IXH8		20	59958423-60034011
**CDH3**	CDHP, PCAD	ENSG00000062038	Cadherin 3	P22223		16	68636189-68727468
**CDH4**	R-CAD	ENSG00000179242	Cadherin 4	P55283		20	61252261-61940617
**CDH5**	7B4, CD144	ENSG00000179776	Cadherin 5	P33151		16	66366622-66404784
**CDH6**		ENSG00000113361	Cadherin 6	P55285		5	31193686-31329146
**CDK16**	FLJ16665, PCTAIRE, PCTAIRE1, PCTGAIRE, PCTK1	ENSG00000102225	Cyclin dependent kinase 16	Q00536	X		47217860-47229997
**CDK5**	PSSALRE	ENSG00000164885	Cyclin dependent kinase 5	Q00535		7	151053815-151057897
**CEACAM5**	CD66e, CEA	ENSG00000105388	CEA cell adhesion molecule 5	P06731		19	41708585-41730433
**CEACAM7**	CEA, CGM2	ENSG00000007306	CEA cell adhesion molecule 7	Q14002		19	41673303-41706976
**CELSR1**	ADGRC1, CDHF9, FMI2, HFMI2, ME2	ENSG00000075275	Cadherin EGF LAG seven-pass G-type receptor 1	Q9NYQ6		22	46360834-46537620
**CEMIP2**	TMEM2	ENSG00000135048	Cell migration inducing hyaluronidase 2	Q9UHN6		9	71683366-71816690
**CFLAR**	c-FLIP, CASH, CASP8AP1, Casper, cFLIP, CLARP, FLAME, FLIP, I-FLICE, M	ENSG00000003402	CASP8 and FADD like apoptosis regulator	O15519		2	201116154-201176687
**CGN**	KIAA1319	ENSG00000143375	Cingulin	Q9P2M7		1	151510510-151538692
**CGNL1**	FLJ14957, JACOP, KIAA1749, PCING	ENSG00000128849	Cingulin like 1	Q0VF96		15	57375967-57550717
**CHIC2**	BTL	ENSG00000109220	Cysteine rich hydrophobic domain 2	Q9UKJ5		4	54009789-54064605
**CHRM3**		ENSG00000133019	Cholinergic receptor muscarinic 3	P20309		1	239386565-239915452
**CHRM4**		ENSG00000180720	Cholinergic receptor muscarinic 4	P08173		11	46383789-46391776
**CHRNA1**	CHRNA	ENSG00000138435	Cholinergic receptor nicotinic alpha 1 subunit	P02708		2	174747592-174787935
**CHRNA2**		ENSG00000120903	Cholinergic receptor nicotinic alpha 2 subunit	Q15822		8	27459756-27479883
**CHRNA3**		ENSG00000080644	Cholinergic receptor nicotinic alpha 3 subunit	P32297		15	78593052-78621295
**CHRNA5**		ENSG00000169684	Cholinergic receptor nicotinic alpha 5 subunit	P30532		15	78565520-78595269
**CHRNB4**		ENSG00000117971	Cholinergic receptor nicotinic beta 4 subunit	P30926		15	78624111-78727754
**CHRND**	ACHRD	ENSG00000135902	Cholinergic receptor nicotinic delta subunit	Q07001		2	232525993-232536667
**CIB1**	CIB, KIP, SIP2-28	ENSG00000185043	Calcium and integrin binding 1	Q99828		15	90229975-90234047
**CLCA2**	CLCRG2	ENSG00000137975	Chloride channel accessory 2	Q9UQC9		1	86424171-86456553
**CLCN5**	ClC-5, CLC5, DENTS, hClC-K2, hClC-K2, NPHL1, NPHL2, XLRH, XRN	ENSG00000171365	Chloride voltage-gated channel 5	P51795	X		49922596-50099235
**CLDN11**	OSP, OTM	ENSG00000013297	Claudin 11	O75508		3	170418868-170454733
**CLDN19**		ENSG00000164007	Claudin 19	Q8N6F1		1	42733093-42740254
**CLDN2**		ENSG00000165376	Claudin 2	P57739	X		106900164-106930861
**CLDN20**		ENSG00000171217	Claudin 20	P56880		6	155264013-155276548
**CLDN3**	C7orf1, CPE-R2, CPETR2, HRVP1, RVP1	ENSG00000165215	Claudin 3	O15551		7	73768997-73770270
**CLDN4**	CPE-R, CPETR, CPETR1, hCPE-R, WBSCR8	ENSG00000189143	Claudin 4	O14493		7	73799542-73832690
**CLDN7**	CEPTRL2, CPETRL2, Hs.84359	ENSG00000181885	Claudin 7	O95471		17	7259903-7263983
**CLDN9**		ENSG00000213937	Claudin 9	O95484		16	3012923-3014505
**CLEC5A**	CLECSF5, MDL-1	ENSG00000258227	C-type lectin domain containing 5A	Q9NY25		7	141927357-141947007
**CLIC2**	CLCNL2, XAP121	ENSG00000155962	Chloride intracellular channel 2	O15247	X		155276211-155334657
**CLIC4**	CLIC4L, DKFZP566G223, H1, huH1, P64H1	ENSG00000169504	Chloride intracellular channel 4	Q9Y696		1	24745382-24844321
**CLIC5**	DFNB102	ENSG00000112782	Chloride intracellular channel 5	Q9NZA1		6	45880827-46080348
**CLTA**	Lca	ENSG00000122705	Clathrin light chain A	P09496		9	36190856-36304781
**CLTB**	Lcb	ENSG00000175416	Clathrin light chain B	P09497		5	176392501-176416539
**CMKLR2**	GPR1	ENSG00000183671	Chemerin chemokine-like receptor 2	P46091		2	206175316-206218047
**CNGA1**	CNCG, CNCG1, CNG1, RCNC1, RCNCa, RP49	ENSG00000198515	Cyclic nucleotide gated channel subunit alpha 1	P29973		4	47935977-48016681
**CNNM4**	ACDP4, KIAA1592	ENSG00000158158	Cyclin and CBS domain divalent metal cation transport mediator 4	Q6P4Q7		2	96760902-96811874
**CNR1**	CANN6, CB-R, CB1, CB1A, CB1K5, CNR	ENSG00000118432	Cannabinoid receptor 1	P21554		6	88139864-88166347
**COG3**	SEC34	ENSG00000136152	Component of oligomeric golgi complex 3	Q96JB2		13	45464898-45536701
**COL17A1**	BP180, BPAG2	ENSG00000065618	Collagen type XVII alpha 1 chain	Q9UMD9		10	104031286-104085880
**COL6A2**		ENSG00000142173	Collagen type VI alpha 2 chain	P12110		21	46098112-46132848
**CORO1B**	coronin-2	ENSG00000172725	Coronin 1B	Q9BR76		11	67435510-67443821
**CPPED1**	CSTP1, FLJ11151	ENSG00000103381	Calcineurin like phosphoesterase domain containing 1	Q9BRF8		16	12659799-12803887
**CR1**	CD35, KN	ENSG00000203710	Complement C3b/C4b receptor 1 (Knops blood group)	P17927		1	207496147-207641765
**CRB3**	MGC17303	ENSG00000130545	Crumbs cell polarity complex component 3	Q9BUF7		19	6463777-6467221
**CRHR2**	CRF-RB, CRF2, HM-CRF	ENSG00000106113	Corticotropin releasing hormone receptor 2	Q13324		7	30651942-30700129
**CRYAB**	CRYA2, HSPB5	ENSG00000109846	Crystallin alpha B	P02511		11	111908564-111923722
**CSF1**	M-CSF, MCSF, MGC31930	ENSG00000184371	Colony stimulating factor 1	P09603		1	109910242-109930992
**CSF1R**	C-FMS, CD115, CSFR, FMS	ENSG00000182578	Colony stimulating factor 1 receptor	P07333		5	150053291-150113372
**CSNK1D**	CKID, CKIdelta, HCKID	ENSG00000141551	Casein kinase 1 delta	P48730		17	82239023-82273700
**CTNNA1**	CAP102	ENSG00000044115	Catenin alpha 1	P35221		5	138610967-138935034
**CTNNB1**	armadillo, beta-catenin, CTNNB	ENSG00000168036	Catenin beta 1	P35222		3	41194741-41260096
**CTNND1**	CTNND, KIAA0384, p120, p120cas, p120ctn	ENSG00000198561	Catenin delta 1	O60716		11	57753243-57819546
**CTTN**	EMS1	ENSG00000085733	Cortactin	Q14247		11	70398404-70436584
**CX3CL1**	ABCD-3, C3Xkine, CXC3, CXC3C, NTN, SCYD1	ENSG00000006210	C-X3-C motif chemokine ligand 1	P78423		16	57372477-57385044
**CX3CR1**	CCRL1, CMKBRL1, CMKDR1, GPR13, V28	ENSG00000168329	C-X3-C motif chemokine receptor 1	P49238		3	39263495-39281735
**CXADR**	CAR	ENSG00000154639	CXADR Ig-like cell adhesion molecule	P78310		21	17513043-17593579
**CXCR2**	CD182, CMKAR2, IL8RB	ENSG00000180871	C-X-C motif chemokine receptor 2	P25025		2	218125289-218137251
**CYBRD1**	CYB561A2, DCYTB, FLJ23462, FRRS3	ENSG00000071967	Cytochrome b reductase 1	Q53TN4		2	171522247-171558129
**CYTH2**	ARNO, CTS18.1, cytohesin-2, PSCD2, PSCD2L, Sec7p-L, Sec7p-like	ENSG00000105443	Cytohesin 2	Q99418		19	48469208-48482314
**CYTH4**	CYT4, cytohesin-4, PSCD4	ENSG00000100055	Cytohesin 4	Q9UIA0		22	37282027-37315341
**DAB2**	DOC-2	ENSG00000153071	DAB adaptor protein 2	P98082		5	39371675-39462300
**DAG1**	156DAG, A3a, AGRNR, DAG	ENSG00000173402	Dystroglycan 1	Q14118		3	49468713-49535618
**DAGLB**	DAGLBETA, KCCR13L	ENSG00000164535	Diacylglycerol lipase beta	Q8NCG7		7	6409126-6484190
**DBNL**	ABP1, HIP-55, SH3P7	ENSG00000136279	Drebrin like	Q9UJU6		7	44044640-44069456
**DCT**	TRP-2, TRP2, TYRP2	ENSG00000080166	Dopachrome tautomerase	P40126		13	94436811-94479682
**DDR1**	CAK, CD167, EDDR1, NEP, NTRK4, PTK3A, RTK6	ENSG00000204580	Discoidin domain receptor tyrosine kinase 1	Q08345		6	30876421-30900156

**DDR2**	NTRKR3, TKT, TYRO10	ENSG00000162733	Discoidin domain receptor tyrosine kinase 2	Q16832		1	162631373-162787405
**DGKH**	DGKeta	ENSG00000102780	Diacylglycerol kinase eta	Q86XP1		13	42040036-42256578
**DIAPH3**	AN, AUNA1, DRF3, FLJ34705, NSDAN	ENSG00000139734	Diaphanous related formin 3	Q9NSV4		13	59665583-60163928
**DIP2A**	C21orf106, Dip2, KIAA0184	ENSG00000160305	Disco interacting protein 2 homolog A	Q14689		21	46458891-46570015
**DLG1**	dJ1061C18.1.1, DLGH1, hdlg, SAP-97, SAP97	ENSG00000075711	Discs large MAGUK scaffold protein 1	Q12959		3	197042560-197299330
**DLG4**	PSD-95, PSD95, SAP-90, SAP90	ENSG00000132535	Discs large MAGUK scaffold protein 4	P78352		17	7187187-7219841
**DLG5**	KIAA0583, P-dlg	ENSG00000151208	Discs large MAGUK scaffold protein 5	Q8TDM6		10	77790791-77926755
**DNAJB4**	HLJ1	ENSG00000162616	DnaJ heat shock protein family (Hsp40) member B4	Q9UDY4		1	77979175-78017964
**DNAJC9**	JDD1, SB73	ENSG00000213551	DnaJ heat shock protein family (Hsp40) member C9	Q8WXX5		10	73183362-73247255
**DOCK4**	FLJ34238, KIAA0716	ENSG00000128512	Dedicator of cytokinesis 4	Q8N1I0		7	111726110-112206407
**DOCK8**	FLJ00026, FLJ00152, FLJ00346, ZIR8	ENSG00000107099	Dedicator of cytokinesis 8	Q8NF50		9	214854-465259
**DOK2**	Dok-2, p56dok-2	ENSG00000147443	Docking protein 2	O60496		8	21908873-21913690
**DOK3**	FLJ22570	ENSG00000146094	Docking protein 3	Q7L591		5	177501904-177511274
**DPEP1**		ENSG00000015413	Dipeptidase 1	P16444		16	89613308-89638456
**DRD4**		ENSG00000069696	Dopamine receptor D4	P21917		11	637269-640706
**DSC2**	CDHF2, DSC3	ENSG00000134755	Desmocollin 2	Q02487		18	31058840-31102522
**DSC3**	CDHF3, DSC, DSC1, DSC2, DSC4	ENSG00000134762	Desmocollin 3	Q14574		18	30989365-31042815
**DSG2**	CDHF5	ENSG00000046604	Desmoglein 2	Q14126		18	31498177-31549008
**DSP**	DPI, DPII, KPPS2, PPKS2	ENSG00000096696	Desmoplakin	P15924		6	7541617-7586714
**DTNA**	D18S892E, DRP3, DTN, DTN-1, DTN-2, DTN-3	ENSG00000134769	Dystrobrevin alpha	Q9Y4J8		18	34493291-34891844
**DYSF**	FER1L1, LGMD2B	ENSG00000135636	Dysferlin	O75923		2	71453561-71686763
**ECSCR**	ARIA, ECSM2	ENSG00000249751	Endothelial cell surface expressed chemotaxis and apoptosis regulator	Q19T08		5	139448560-139462743
**EDNRA**	ET-A, ETA-R, hET-AR	ENSG00000151617	Endothelin receptor type A	P25101		4	147480917-147544954
**EDNRB**	ETB, HSCR, HSCR2	ENSG00000136160	Endothelin receptor type B	P24530		13	77895481-77975529
**EFNB1**	CFNS, Elk-L, EPLG2, LERK2	ENSG00000090776	Ephrin B1	P98172	X		68829021-68842160
**EFR3A**	KIAA0143	ENSG00000132294	EFR3 homolog A	Q14156		8	131904093-132013642
**EFR3B**	FLJ37871, KIAA0953	ENSG00000084710	EFR3 homolog B	Q9Y2G0		2	25042076-25159135
**EGFR**	ERBB, ERBB1, ERRP	ENSG00000146648	Epidermal growth factor receptor	P00533		7	55019017-55211628
**EHBP1**	KIAA0903, NACSIN	ENSG00000115504	EH domain binding protein 1	Q8NDI1		2	62673851-63046487
**EHD1**	FLJ42622, FLJ44618, H-PAST, HPAST1, PAST1	ENSG00000110047	EH domain containing 1	Q9H4M9		11	64851642-64888296
**EHD2**	PAST2	ENSG00000024422	EH domain containing 2	Q9NZN4		19	47713422-47743134
**EHD3**	PAST3	ENSG00000013016	EH domain containing 3	Q9NZN3		2	31234152-31269451
**ELAPOR1**	EIG121, KIAA1324, maba1	ENSG00000116299	Endosome-lysosome associated apoptosis and autophagy regulator 1	Q6UXG2		1	109113679-109206781
**ENAH**	FLJ10773, MENA, NDPP1	ENSG00000154380	ENAH actin regulator	Q8N8S7		1	225486765-225653142
**ENG**	CD105, END, HHT1, ORW, ORW1	ENSG00000106991	Endoglin	P17813		9	127815013-127854658
**ENO1**	ENO1L1, MBP-1, MPB1, PPH	ENSG00000074800	Enolase 1	P06733		1	8861000-8879190
**ENO2**	NSE	ENSG00000111674	Enolase 2	P09104		12	6913745-6923698
**ENO3**		ENSG00000108515	Enolase 3	P13929		17	4948092-4957131
**ENOX1**	cCNOX, CNOX, FLJ10094, PIG38	ENSG00000120658	Ecto-NOX disulfide-thiol exchanger 1	Q8TC92		13	43213130-43786972
**ENOX2**	APK1, COVA1, tNOX	ENSG00000165675	Ecto-NOX disulfide-thiol exchanger 2	Q16206	X		130622325-130903234
**EPB41L1**	4.1N, KIAA0338	ENSG00000088367	Erythrocyte membrane protein band 4.1 like 1	Q9H4G0		20	36091504-36232799
**EPB41L2**	4.1-G, 4.1G	ENSG00000079819	Erythrocyte membrane protein band 4.1 like 2	O43491		6	130839347-131063322
**EPB41L3**	4.1B, DAL1, KIAA0987	ENSG00000082397	Erythrocyte membrane protein band 4.1 like 3	Q9Y2J2		18	5392381-5630700
**EPB41L5**	BE37, FLJ12957, KIAA1548, LULU, LULU1, YMO1, YRT	ENSG00000115109	Erythrocyte membrane protein band 4.1 like 5	Q9HCM4		2	120013077-120179119
**EPCAM**	17-1A, 323/A3, Ber-Ep4, BerEp4, CD326, CO-17A, EGP-2, EGP34, EGP40	ENSG00000119888	Epithelial cell adhesion molecule	P16422		2	47345158-47387601
**EPHA2**	ECK	ENSG00000142627	EPH receptor A2	P29317		1	16124337-16156069
**EPHA3**	ETK, ETK1, HEK, HEK4, TYRO4	ENSG00000044524	EPH receptor A3	P29320		3	89107621-89482134
**EPHA5**	CEK7, EHK1, Hek7, TYRO4	ENSG00000145242	EPH receptor A5	P54756		4	65319563-65670495
**EPHB1**	EPHT2, Hek6	ENSG00000154928	EPH receptor B1	P54762		3	134795260-135260467
**EPHB2**	DRT, EPHT3, ERK, Hek5, Tyro5	ENSG00000133216	EPH receptor B2	P29323		1	22710839-22921500
**EPOR**		ENSG00000187266	Erythropoietin receptor	P19235		19	11377207-11384342
**ERBB2**	c-ERB-2, c-ERB2, CD340, HER-2, HER2, MLN-19, NEU, NGL, p185(erbB2	ENSG00000141736	Erb-b2 receptor tyrosine kinase 2	P04626		17	39687914-39730426
**ERBB3**	HER3, LCCS2	ENSG00000065361	Erb-b2 receptor tyrosine kinase 3	P21860		12	56076799-56103505
**ERBIN**	ERBB2IP, LAP2	ENSG00000112851	Erbb2 interacting protein	Q96RT1		5	65883128-66082546
**ESYT2**	CHR2SYT, FAM62B, KIAA1228	ENSG00000117868	Extended synaptotagmin 2	A0FGR8		7	158730995-158830253
**EVI2A**	EVDA, EVI2	ENSG00000126860	Ecotropic viral integration site 2A	P22794		17	31316410-31321749
**EZR**	VIL2	ENSG00000092820	Ezrin	P15311		6	158765741-158819368
**F11R**	CD321, JAM-1, JAM-A, JAM1, JAMA, JCAM, PAM-1	ENSG00000158769	F11 receptor	Q9Y624		1	160995211-161021343
**F2RL3**	PAR4	ENSG00000127533	F2R like thrombin or trypsin receptor 3	Q96RI0		19	16888999-16892606
**FAS**	APO-1, APT1, CD95, FAS1, TNFRSF6	ENSG00000026103	Fas cell surface death receptor	P25445		10	88953813-89029605
**FASN**	FAS, SDR27X1	ENSG00000169710	Fatty acid synthase	P49327		17	82078338-82098294
**FBLIM1**	CAL, FBLP-1, migfilin	ENSG00000162458	Filamin binding LIM protein 1	Q8WUP2		1	15756607-15786594
**FBXL2**	FBL2, FBL3	ENSG00000153558	F-box and leucine rich repeat protein 2	Q9UKC9		3	33277025-33403662
**FCAR**	CD89, FcalphaR, FcalphaRI	ENSG00000186431	Fc alpha receptor	P24071		19	54874235-54891420
**FCGR1A**	CD64, CD64A, FCG1, FcgammaRI, FcgammaRIa, FCGR1	ENSG00000150337	Fc gamma receptor Ia	P12314		1	149782671-149791675
**FCGR2A**	CD32, CD32A, CDw32, Fc-gamma-RIIa, FCG2, FcgammaRIIa, FCGR2, FC	ENSG00000143226	Fc gamma receptor IIa	P12318		1	161505430-161524013
**FCHSD2**	KIAA0769, SH3MD3	ENSG00000137478	FCH and double SH3 domains 2	O94868		11	72836745-73142318
**FCMR**	FAIM3, FcmuR, TOSO	ENSG00000162894	Fc mu receptor	O60667		1	206903317-206923247
**FFAR1**	FFA1R, GPR40	ENSG00000126266	Free fatty acid receptor 1	O14842		19	35347902-35353864
**FFAR4**	GPR120, GPR129, O3FAR1, PGR4	ENSG00000186188	Free fatty acid receptor 4	Q5NUL3		10	93566665-93604480
**FGD1**	FGDY, ZFYVE3	ENSG00000102302	FYVE, RhoGEF and PH domain containing 1	P98174	X		54445454-54496234
**FGD5**	FLJ00274, FLJ39957, ZFYVE23	ENSG00000154783	FYVE, RhoGEF and PH domain containing 5	Q6ZNL6		3	14810853-14934571
**FHL1**	bA535K18.1, FHL1B, FLH1A, KYO-T, MGC111107, SLIM1, XMPMA	ENSG00000022267	Four and a half LIM domains 1	Q13642	X		136146702-136211359
**FLNB**	ABP-278, FH1, FLN1L, LRS1, TABP, TAP	ENSG00000136068	Filamin B	O75369		3	58008398-58172251
**FLOT1**		ENSG00000137312	Flotillin 1	O75955		6	30727709-30742732
**FLOT2**	ECS-1, ECS1, ESA, ESA1, M17S1	ENSG00000132589	Flotillin 2	Q14254		17	28879335-28897733
**FLRT3**		ENSG00000125848	Fibronectin leucine rich transmembrane protein 3	Q9NZU0		20	14322985-14337614
**FLT1**	FLT, VEGFR1	ENSG00000102755	Fms related receptor tyrosine kinase 1	P17948		13	28300346-28495145
**FLT4**	PCL, VEGFR-3, VEGFR3	ENSG00000037280	Fms related receptor tyrosine kinase 4	P35916		5	180601506-180649624
**FMNL3**	DKFZp762B245, MGC45819, WBP3	ENSG00000161791	Formin like 3	Q8IVF7		12	49636499-49708165
**FNBP1**	FBP17, KIAA0554	ENSG00000187239	Formin binding protein 1	Q96RU3		9	129887187-130043189
**FPR3**	FMLPY, FPRH1, FPRL2, RMLP-R-I	ENSG00000187474	Formyl peptide receptor 3	P25089		19	51795157-51826190
**FRMD8**	FKSG44, FLJ90369, iTAP	ENSG00000126391	FERM domain containing 8	Q9BZ67		11	65386621-65413525
**FYN**	MGC45350, SLK, SYN	ENSG00000010810	FYN proto-oncogene, Src family tyrosine kinase	P06241		6	111660332-111873452
**FZD1**	DKFZp564G072	ENSG00000157240	Frizzled class receptor 1	Q9UP38		7	91264433-91271326
**FZD4**	CD344, EVR1	ENSG00000174804	Frizzled class receptor 4	Q9ULV1		11	86945679-86955395
**FZD6**	Hfz6	ENSG00000164930	Frizzled class receptor 6	O60353		8	103298433-103332866
**GAB2**	KIAA0571	ENSG00000033327	GRB2 associated binding protein 2	Q9UQC2		11	78215293-78418348
**GABBR1**	GPRC3A, hGB1a	ENSG00000204681	Gamma-aminobutyric acid type B receptor subunit 1	Q9UBS5		6	29555629-29633976
**GABRA1**	EJM5	ENSG00000022355	Gamma-aminobutyric acid type A receptor subunit alpha1	P14867		5	161847063-161899981
**GABRA5**		ENSG00000186297	Gamma-aminobutyric acid type A receptor subunit alpha5	P31644		15	26866911-26949208
**GABRG1**		ENSG00000163285	Gamma-aminobutyric acid type A receptor subunit gamma1	Q8N1C3		4	46035769-46124054
**GABRG3**		ENSG00000182256	Gamma-aminobutyric acid type A receptor subunit gamma3	Q99928		15	26971181-27541984
**GALR1**	GALNR, GALNR1	ENSG00000166573	Galanin receptor 1	P47211		18	77249848-77277900
**GAP43**	B-50, GAP-43, PP46	ENSG00000172020	Growth associated protein 43	P17677		3	115623510-115721490
**GAPDH**	GAPD	ENSG00000111640	Glyceraldehyde-3-phosphate dehydrogenase	P04406		12	6534512-6538374
**GAPT**	C5orf29, FLJ33641	ENSG00000175857	GRB2 binding adaptor protein, transmembrane	Q8N292		5	58491435-58497090
**GAPVD1**	DKFZP434C212, KIAA1521	ENSG00000165219	GTPase activating protein and VPS9 domains 1	Q14C86		9	125261794-125367207
**GAS8**	DRC4, GAS11	ENSG00000141013	Growth arrest specific 8	O95995		16	90019629-90044975
**GFRA3**	GFRa-3	ENSG00000146013	GDNF family receptor alpha 3	O60609		5	138252380-138274621
**GFRAL**	bA360D14.1, C6orf144, GRAL, UNQ9356	ENSG00000187871	GDNF family receptor alpha like	Q6UXV0		6	55327469-55402493
**GHR**	GHBP	ENSG00000112964	Growth hormone receptor	P10912		5	42423439-42721878
**GID8**	bA305P22.1, C20orf11, FLJ20602, TWA1	ENSG00000101193	GID complex subunit 8 homolog	Q9NWU2		20	62938147-62948475
**GJA1**	CX43, GJAL, ODD, ODDD, ODOD, SDTY3	ENSG00000152661	Gap junction protein alpha 1	P17302		6	121435595-121449727
**GJB3**	CX31, DFNA2, EKV	ENSG00000188910	Gap junction protein beta 3	O75712		1	34781214-34786369
**GJB4**	CX30.3	ENSG00000189433	Gap junction protein beta 4	Q9NTQ9		1	34759740-34762327
**GJB6**	CX30, DFNA3, ED2, EDH, HED	ENSG00000121742	Gap junction protein beta 6	O95452		13	20221962-20232365
**GJC1**	CX45, GJA7	ENSG00000182963	Gap junction protein gamma 1	P36383		17	44798448-44830816
**GJC2**	CX46.6, CX47, GJA12, SPG44	ENSG00000198835	Gap junction protein gamma 2	Q5T442		1	228149930-228159826
**GLDN**	CLOM, COLM, colmedin, CRG-L2, UNC-122	ENSG00000186417	Gliomedin	Q6ZMI3		15	51341655-51408005
**GLO1**	GLOD1	ENSG00000124767	Glyoxalase I	Q04760		6	38675925-38703145
**GLP1R**	GLP-1R	ENSG00000112164	Glucagon like peptide 1 receptor	P43220		6	39048781-39091303
**GLP2R**		ENSG00000065325	Glucagon like peptide 2 receptor	O95838		17	9822206-9892099
**GLRA1**	STHE	ENSG00000145888	Glycine receptor alpha 1	P23415		5	151822513-151924851
**GLRA2**	GLR	ENSG00000101958	Glycine receptor alpha 2	P23416	X		14529298-14731812
**GLRA3**		ENSG00000145451	Glycine receptor alpha 3	O75311		4	174636920-174829247
**GNAI2**	GIP, GNAI2B	ENSG00000114353	G protein subunit alpha i2	P04899		3	50226292-50259362
**GNAQ**	G-ALPHA-q, GAQ	ENSG00000156052	G protein subunit alpha q	P50148		9	77716097-78031811
**GNAS**	GNAS1, GNASXL, GPSA, NESP, NESP55, SCG6, SgVI	ENSG00000087460	GNAS complex locus	O95467, P		20	58839718-58911192
**GNB2**		ENSG00000172354	G protein subunit beta 2	P62879		7	100673567-100679174
**GP6**	GPVI	ENSG00000088053	Glycoprotein VI platelet	Q9HCN6		19	55013705-55038264
**GPC1**	glypican	ENSG00000063660	Glypican 1	P35052		2	240435663-240468076
**GPC3**	DGSX, OCI-5, SDYS, SGB, SGBS, SGBS1	ENSG00000147257	Glypican 3	P51654	X		133535745-133987100
**GPR143**	OA1	ENSG00000101850	G protein-coupled receptor 143	P51810	X		9725346-9786297
**GPR148**	PGR6	ENSG00000173302	G protein-coupled receptor 148	Q8TDV2		2	130729070-130730336
**GPR156**	GABABL, PGR28	ENSG00000175697	G protein-coupled receptor 156	Q8NFN8		3	120164645-120285222
**GPR160**	GPCR1, GPCR150	ENSG00000173890	G protein-coupled receptor 160	Q9UJ42		3	170037995-170085392
**GPR174**	FKSG79	ENSG00000147138	G protein-coupled receptor 174	Q9BXC1	X		79144688-79175318
**GPR183**	EBI2	ENSG00000169508	G protein-coupled receptor 183	P32249		13	99294539-99307399

GPR20		ENSG00000204882	G protein-coupled receptor 20	Q99678	8	141356470-141367286
GPR37L1	ETBR-LP-2	ENSG00000170075	G protein-coupled receptor 37 like 1	O60883	1	202122886-202133592
GPR50	H9, Mel1c	ENSG00000102195	G protein-coupled receptor 50	Q13585	X	151176584-151181465
GPR75	WI-31133	ENSG00000119737	G protein-coupled receptor 75	O95800	2	53852912-53859967
GPRC5A	PEIG-1, RAI3, RAIG1, TIG1	ENSG00000013588	G protein-coupled receptor class C group 5 member A	Q8NFJ5	12	12891559-12917937
GPRC5C	RAIG-3	ENSG00000170412	G protein-coupled receptor class C group 5 member C	Q9NQ84	17	74424851-74451653
GPRIN1	GRIN1, KIAA1893	ENSG00000169258	G protein regulated inducer of neurite outgrowth 1	Q7Z2K8	5	176595802-176610156
GRAMD1A	FLJ90346, KIAA1533	ENSG00000089351	GRAM domain containing 1A	Q96CP6	19	34994784-35026471
GRAMD1B	KIAA1201, LINC01059	ENSG00000023171	GRAM domain containing 1B	Q3KR37	11	123358428-123627774
GRB7		ENSG00000141738	Growth factor receptor bound protein 7	Q14451	17	39737927-39747291
GRIK1	GluK1, GLUR5	ENSG00000171189	Glutamate ionotropic receptor kainate type subunit 1	P39086	21	29536933-29940033
GRIK5	GluK5, GRIK2, KA2	ENSG00000105737	Glutamate ionotropic receptor kainate type subunit 5	Q16478	19	41998321-42070206
GRIN2B	GluN2B, NMDAR2B	ENSG00000273079	Glutamate ionotropic receptor NMDA type subunit 2B	Q13224	12	13437942-13982002
GRK5	GPRK5	ENSG00000198873	G protein-coupled receptor kinase 5	P34947	10	119207571-119459745
GRPR	BB2, BB2R, BRS2	ENSG00000126010	Gastrin releasing peptide receptor	P30550	X	16123565-16153518
GSDMA	FLJ39120, GSDM, GSDM1	ENSG00000167914	Gasdermin A	Q96QA5	17	39953263-39977768
GTSE1	B99, GTSE-1, Lnc_bc060912	ENSG00000075218	G2 and S-phase expressed 1	Q9NYZ3	22	46296870-46330810
GYPA	CD235a, GPA, MN, MNS	ENSG00000170180	Glycophorin A (MNS blood group)	P02724	4	144109303-144140751
GYPC	CD236, CD236R, Ge, GPC, GYPD	ENSG00000136732	Glycophorin C (Gerbich blood group)	P04921	2	126656133-126696667
HARBI1	C11orf77, FLJ32675	ENSG00000180423	Harbinger transposase derived 1	Q96MB7	11	46602861-46617909
HAS1	HAS	ENSG00000105509	Hyaluronan synthase 1	Q92839	19	51713112-51723991
HCAR2	GPR109A, HCA2, HM74A, NIACR1, Puma-g, PUMAG	ENSG00000182782	Hydroxycarboxylic acid receptor 2	Q8TDS4	12	122701293-122703357
HCK	JTK9	ENSG00000101336	HCK proto-oncogene, Src family tyrosine kinase	P08631	20	32052197-32101856
HCLS1	CTTNL, HS1	ENSG00000180353	Hematopoietic cell-specific Lyn substrate 1	P14317	3	121631399-121660927
HCRTR2	ORXR2, OX2R, OXR2	ENSG00000137252	Hypocretin receptor 2	O43614	6	55106460-55282617
HFE	HFE1, HLA-H	ENSG00000010704	Homeostatic iron regulator	Q30201	6	26087281-26098343
HLA-A		ENSG00000206503	Major histocompatibility complex, class I, A	P04439	6	29941260-29949572
HLA-DPB1	HLA-DP1B	ENSG00000223865	Major histocompatibility complex, class II, DP beta 1	P04440	6	33075990-33089696
HLA-E		ENSG00000204592	Major histocompatibility complex, class I, E	P13747	6	30489509-30494194
HMGCS1	HMGCS	ENSG00000112972	3-hydroxy-3-methylglutaryl-CoA synthase 1	Q01581	5	43287470-43313512
HOMER3	HOMER-3	ENSG00000051128	Homer scaffold protein 3	Q9NSC5	19	18929201-18941261
HRH1		ENSG00000196639	Histamine receptor H1	P35367	3	11137093-11263557
HSP90AB1	HSPC2, HSPCB	ENSG00000096384	Heat shock protein 90 alpha family class B member 1	P08238	6	44246166-44253888
HSPB1	CMT2F, Hs.76067, Hsp25, HSP27, HSP28	ENSG00000106211	Heat shock protein family B (small) member 1	P04792	7	76302673-76304295
HTR7	5-HT7	ENSG00000148680	5-hydroxytryptamine receptor 7	P34969	10	90740823-90858039
HTRA1	ARMD7, HtrA, IGFBP5-protease, PRSS11	ENSG00000166033	HtrA serine peptidase 1	Q92743	10	122458551-122514907
HYCC1	DRCTNNB1A, FAM126A, HCC, hyccin	ENSG00000122591	Hyccin PI4KA lipid kinase complex subunit 1	Q9BYI3	7	22889371-23014130
HYCC2	FAM126B, MGC39518	ENSG00000155744	Hyccin PI4KA lipid kinase complex subunit 2	Q8IXS8	2	200973718-201071671
ICOSLG	B7-H2, B7h, B7H2, B7RP-1, B7RP1, CD275, GL50, ICOS-L, ICOSL, KIAA(	ENSG00000160223	Inducible T cell costimulator ligand	O75144	21	44215382-44241446
IFIT5	RI58	ENSG00000152778	Interferon induced protein with tetratricopeptide repeats 5	Q13325	10	89414568-89421605
IFITM1	9-27, CD225, DSPA2a, IFI17	ENSG00000185885	Interferon induced transmembrane protein 1	P13164	11	310041-315272
IFITM5	BRIL, DSPA1, fragilis4, Hrmp1	ENSG00000206013	Interferon induced transmembrane protein 5	A6NNB3	11	298200-299526
IFNGR1	CD119, IFNGR	ENSG00000027697	Interferon gamma receptor 1	P15260	6	137197483-137219449
IGF1R	CD221, IGFIR, IGFR, JTK13, MGC18216	ENSG00000140443	Insulin like growth factor 1 receptor	P08069	15	98648539-98964530
IGFLR1	FLJ22573, TMEM149, U2AF1L4	ENSG00000126246	IGF like family receptor 1	Q9H665	19	35738801-35742453
IL10RA	CD210, CD210a, CDW210A, HIL-10R, IL10R	ENSG00000110324	Interleukin 10 receptor subunit alpha	Q13651	11	117986370-118003037
IL15RA	CD215, IL-15RA	ENSG00000134470	Interleukin 15 receptor subunit alpha	Q13261	10	5943639-5978187
IL17RB	CRL4, EVI27, IL17BR, IL17RH1	ENSG00000056736	Interleukin 17 receptor B	Q9NRM6	3	53846568-53865794
IL1RL1	DER4, FIT-1, IL33R, ST2, ST2L, ST2V, T1	ENSG00000115602	Interleukin 1 receptor like 1	Q01638	2	102311502-102352037
IL2RG	CD132, CIDX, IMD4, SCIDX1	ENSG00000147168	Interleukin 2 receptor subunit gamma	P31785	X	71107404-71112108
IL4R	CD124	ENSG00000077238	Interleukin 4 receptor	P24394	16	27313668-27364778
IL6ST	CD130, GP130, IL-6RB, sGP130	ENSG00000134352	Interleukin 6 cytokine family signal transducer	P40189	5	55935095-55995022
IL7R	CD127, IL7RA, lnc-IL7R	ENSG00000168685	Interleukin 7 receptor	P16871	5	35852695-35879603
IQGAP1	HUMORFA01, KIAA0051, p195, SAR1	ENSG00000140575	IQ motif containing GTPase activating protein 1	P46940	15	90388242-90502239
IRF2		ENSG00000168310	Interferon regulatory factor 2	P14316	4	184385702-184474558
ITGA3	CD49c, GAP-B3, MSK18, VCA-2, VLA3a	ENSG00000005884	Integrin subunit alpha 3	P26006	17	50055968-50090481
ITGAX	CD11C	ENSG00000140678	Integrin subunit alpha X	P20702	16	31355134-31382999
ITGB1	CD29, FNRB, GPIIA, MDF2, MSK12	ENSG00000150093	Integrin subunit beta 1	P05556	10	32887273-33005792
ITGB2	CD18, LFA-1, MAC-1, MFI7	ENSG00000160255	Integrin subunit beta 2	P05107	21	44885953-44931989
ITGB3	CD61, GP3A, GPIIIa	ENSG00000259207	Integrin subunit beta 3	P05106	17	47253827-47313743
ITGB4	CD104	ENSG00000132470	Integrin subunit beta 4	P16144	17	75721328-75757818
ITSN1	ITSN, MGC134948, MGC134949, SH3D1A, SH3P17	ENSG00000205726	Intersectin 1	Q15811	21	33642400-33899861
IYD	C6orf71, DEHAL1, dJ422F24.1	ENSG00000009765	Iodotyrosine deiodinase	Q6PHW0	6	150368892-150405969
JAM2	C21orf43, CD322, JAM-2, JAM-B, JAMB, VE-JAM	ENSG00000154721	Junctional adhesion molecule 2	P57087	21	25639258-25717562
JAML	AMICA, AMICA1, Gm638	ENSG00000160593	Junction adhesion molecule like	Q86YT9	11	118193725-118225094
JUP	CTNNG, DP3, DPIII, PDGB, PG, PKGB	ENSG00000173801	Junction plakoglobin	P14923	17	41754604-41786931
KANK1	ANKRD15, KANK, KIAA0172	ENSG00000107104	KN motif and ankyrin repeat domains 1	Q14678	9	470291-746105
KANSL2	C12orf41, FLJ20436, NSL2	ENSG00000139620	KAT8 regulatory NSL complex subunit 2	Q9H9L4	12	48653211-48682238
KARS1	DFNB89, KARS, KARS2	ENSG00000065427	Lysyl-tRNA synthetase 1	Q15046	16	75627474-75648643
KCNA6	HBK2, Kv1.6, PPP1R96	ENSG00000151079	Potassium voltage-gated channel subfamily A member 6	P17658	12	4809334-4813318
KCNAB1	AKR6A3, hKvb3, hKvBeta3, KCNA1B, Kvb1.3	ENSG00000169282	Potassium voltage-gated channel subfamily A regulatory beta subunit 1	Q14722	3	156037701-156539138
KCNE3	HOKPP, MiRP2	ENSG00000175538	Potassium voltage-gated channel subfamily E regulatory subunit 3	Q9Y6H6	11	74454841-74467729
KCNG4	Kv6.4	ENSG00000168418	Potassium voltage-gated channel modifier subfamily G member 4	Q8TDN1	16	84218657-84240012
KCNH8	elk3, Kv12.1	ENSG00000183960	Potassium voltage-gated channel subfamily H member 8	Q96L42	3	19148510-19535642
KCNK17	K2p17.1, TALK-2, TALK2, TASK-4, TASK4	ENSG00000124780	Potassium two pore domain channel subfamily K member 17	Q96T54	6	39299001-39314461
KCNN4	hIKCa1, hKCa4, hSK4, IK, KCa3.1	ENSG00000104783	Potassium calcium-activated channel subfamily N member 4	O15554	19	43766533-43780976
KCNQ1	JLNS1, KCNA8, KCNA9, Kv7.1, KVLQT1, LQT, LQT1	ENSG00000053918	Potassium voltage-gated channel subfamily Q member 1	P51787	11	2444684-2849105
KCNS1	Kv9.1	ENSG00000124134	Potassium voltage-gated channel modifier subfamily S member 1	Q96KK3	20	45091214-45101127
KCNS3	Kv9.3	ENSG00000170745	Potassium voltage-gated channel modifier subfamily S member 3	Q9BQ31	2	17877847-18361616
KCNT1	KCa4.1, KIAA1422, SLACK, Slo2.2	ENSG00000107147	Potassium sodium-activated channel subfamily T member 1	Q5JUK3	9	135702185-135795508
KCNV1	Kv8.1	ENSG00000164794	Potassium voltage-gated channel modifier subfamily V member 1	Q6PIU1	8	109963636-109975771
KEL	CD238, ECE3	ENSG00000197993	Kell metallo-endopeptidase (Kell blood group)	P23276	7	142941114-142962363
KIAA1522		ENSG00000162522	KIAA1522	Q9P206	1	32741830-32774970
KISS1R	AXOR12, GPR54, HOT7T175	ENSG00000116014	KISS1 receptor	Q969F8	19	917287-921005
KIT	C-Kit, CD117, PBT, SCFR	ENSG00000157404	KIT proto-oncogene, receptor tyrosine kinase	P10721	4	54657267-54740783
KLC2	FLJ12387	ENSG00000174996	Kinesin light chain 2	Q9H0B6	11	66257294-66267860
KLK7	PRSS6, SCCE	ENSG00000169035	Kallikrein related peptidase 7	P49862	19	50976468-50984099
KLRK1	CD314, D12S2489E, KLR, NKG2-D, NKG2D	ENSG00000213809	Killer cell lectin like receptor K1	P26718	12	10372353-10391874
L1CAM	CAML1, CD171, HSAS, HSAS1, MASA, MIC5, NCAM-L1, S10, SPG1	ENSG00000198910	L1 cell adhesion molecule	P32004	X	153861514-153886173
LAIR1	CD305, LAIR-1	ENSG00000167613	Leukocyte associated immunoglobulin like receptor 1	Q6GTX8	19	54351384-54370558
LAMP2	CD107b	ENSG00000005893	Lysosomal associated membrane protein 2	P13473	X	120426148-120469365
LAMTOR1	C11orf59, FLJ20625, p18, p27RF-Rho, Pdro, Ragulator1	ENSG00000149357	Late endosomal/lysosomal adaptor, MAPK and MTOR activator 1	Q6IAA8	11	72085895-72103297
LAT	LAT1	ENSG00000213658	Linker for activation of T cells	O43561	16	28984826-28990784
LAT2	HSPC046, LAB, NTAL, WBSCR15, WBSCR5, WSCR5	ENSG00000086730	Linker for activation of T cells family member 2	Q9GZY6	7	74199652-74229834
LAX1	FLJ20340, LAX	ENSG00000122188	Lymphocyte transmembrane adaptor 1	Q8IWV1	1	203765177-203776372
LCP1	CP64, L-PLASTIN, LC64P, PLS2	ENSG00000136167	Lymphocyte cytosolic protein 1	P13796	13	46125920-46211871
LEPR	CD295, OBR	ENSG00000116678	Leptin receptor	P48357	1	65420652-65641559
LHCGR	HHG, LCGR, LGR2, LHR, ULG5	ENSG00000138039	Luteinizing hormone/choriogonadotropin receptor	P22888	2	48686774-48755730
LINGO1	FLJ14594, LERN1, LRRN6A	ENSG00000169783	Leucine rich repeat and Ig domain containing 1	Q96FE5	15	77613027-77820900
LIPI	CT17, LPDL, mPA-PLA1beta, PLA1C, PRED5	ENSG00000188992	Lipase I	Q6XZB0	21	14108813-14210955
LITAF	FLJ38636, PIG7, SIMPLE, TP53I7	ENSG00000189067	Lipopolysaccharide induced TNF factor	Q99732	16	11547722-11636381
LNX1	LNX, MPDZ, PDZRN2	ENSG00000072201	Ligand of numb-protein X 1	Q8TBB1	4	53459301-53701405
LPAR3	Edg-7, EDG7, HOFNH30, LP-A3, LPA3, RP4-678I3	ENSG00000171517	Lysophosphatidic acid receptor 3	Q9UBY5	1	84811602-84893206
LPAR4	GPR23, LPA4, P2RY9, P2Y5-LIKE, P2Y9	ENSG00000147145	Lysophosphatidic acid receptor 4	Q99677	X	78747709-78758714
LPAR6	P2RY5, P2Y5	ENSG00000139679	Lysophosphatidic acid receptor 6	P43657	13	48389567-48444704
LPP		ENSG00000145012	LIM domain containing preferred translocation partner in lipoma	Q93052	3	188153284-188890671
LRFN2	FIGLER2, KIAA1246, SALM1	ENSG00000156564	Leucine rich repeat and fibronectin type III domain containing 2	Q9ULH4	6	40391591-40587364
LRP1	A2MR, APOER, APR, CD91, IGFBP-3R, IGFBP3R1, LRP, LRP1A	ENSG00000123384	LDL receptor related protein 1	Q07954	12	57128483-57213361
LRRC26	bA350O14.10	ENSG00000184709	Leucine rich repeat containing 26	Q2I0M4	9	137168758-137170051
LSR	ILDR3, LISCH7	ENSG00000105699	Lipolysis stimulated lipoprotein receptor	Q86X29	19	35248330-35267964
LST1	B144, D6S49E, LST-1	ENSG00000204482	Leukocyte specific transcript 1	O00453	6	31586124-31588909
LTB4R2	BLT2, BLTR2, JULF2, NOP9	ENSG00000213906	Leukotriene B4 receptor 2	Q9NPC1	14	24305734-24312053
LYN	JTK8	ENSG00000254087	LYN proto-oncogene, Src family tyrosine kinase	P07948	8	55879835-56014169
MACF1	ABP620, ACF7, FLJ45612, FLJ46776, KIAA0465, KIAA0754, KIAA1251, Ln	ENSG00000127603	Microtubule actin crosslinking factor 1	Q9UPN3	1	39081316-39487177
MADD	DENN, KIAA0358, RAB3GEP	ENSG00000110514	MAP kinase activating death domain	Q8WXG6	11	47269161-47330031
MAGI1	AIP3, BAIAP1, BAP1, MAGI-1, TNRC19, WWP3	ENSG00000151276	Membrane associated guanylate kinase, WW and PDZ domain containing 1	Q96QZ7	3	65353525-66038918
MAGI3	MAGI-3	ENSG00000081026	Membrane associated guanylate kinase, WW and PDZ domain containing 3	Q5TCQ9	1	113390515-113685923
MAP2K1	MAPKK1, MEK1, PRKMK1	ENSG00000169032	Mitogen-activated protein kinase kinase 1	Q02750	15	66386837-66491656
MAP3K7	MEKK7, TAK1	ENSG00000135341	Mitogen-activated protein kinase kinase kinase 7	O43318	6	90513573-90587086
MAPK8IP1	IB1, JIP-1, JIP1, PRKM8IP	ENSG00000121653	Mitogen-activated protein kinase 8 interacting protein 1	Q9UQF2	11	45885651-45906465
MAPT	DDPAC, FLJ31424, FTDP-17, MAPTL, MGC138549, MSTD, MTBT1, MTBT	ENSG00000186868	Microtubule associated protein tau	P10636	17	45894527-46028334
MARCKS	80K-L, MACS, PKCSL	ENSG00000277443	Myristoylated alanine rich protein kinase C substrate	P29966	6	113857345-113863475
MARK2	EMK1, PAR-1, PAR-1B, Par1b	ENSG00000072518	Microtubule affinity regulating kinase 2	Q7KZI7	11	63838928-63911020
MARVELD2	DFNB49, FLJ30532, MRVLDC2, TRIC	ENSG00000152939	MARVEL domain containing 2	Q8N4S9	5	69415065-69444330
MAS1L	dJ994E9.2, MAS-L, MRG	ENSG00000204687	MAS1 proto-oncogene like, G protein-coupled receptor	P35410	6	29486697-29487956
MC5R		ENSG00000176136	Melanocortin 5 receptor	P33032	18	13824149-13827323
MCAM	CD146, HEMCAM, MelCAM, METCAM, MUC18	ENSG00000076706	Melanoma cell adhesion molecule	P43121	11	119308529-119321521
MCEMP1	C19orf59, MGC132456	ENSG00000183019	Mast cell expressed membrane protein 1	Q8IX19	19	7677088-7679829
MCF 2.00	ARHGEF21, DBL	ENSG00000101977	MCF.2 cell line derived transforming sequence	P10911	X	139581770-139708216

**MCHR2**	GPR145, MCH2, MCH2R, SLT	ENSG00000152034	Melanin concentrating hormone receptor 2	Q969V1		6	99918519-99994247
**MCOLN1**	ML4, MLIV, MST080, MSTP080, TRPM-L1, TRPML1	ENSG00000090674	Mucolipin TRP cation channel 1	Q9GZU1		19	7522624-7534009
**MDGA1**	GPIM, MAMDC3	ENSG00000112139	MAM domain containing glycosylphosphatidylinositol anchor 1	Q8NFP4		6	37630679-37699306
**MEFV**	FMF, MEF, TRIM20	ENSG00000103313	MEFV innate immuity regulator, pyrin	O15553		16	3242027-3256633
**MEGF10**	KIAA1780, SR-F3	ENSG00000145794	Multiple EGF like domains 10	Q96KG7		5	127290796-127465737
**MERTK**	c-Eyk, mer, RP38, Tyro12	ENSG00000153208	MER proto-oncogene, tyrosine kinase	Q12866		2	111898607-112029561
**MFAP3L**	KIAA0626, NYD-sp9	ENSG00000198948	Microfibril associated protein 3 like	O75121		4	169986597-170033031
**MFSD2A**	FLJ14490, MFSD2, SLC59A1	ENSG00000168389	Major facilitator superfamily domain containing 2A	Q8NA29		1	39955112-39969968
**MICALL1**	KIAA1668, MICAL-L1, MIRAB13	ENSG00000100139	MICAL like 1	Q8N3F8		22	37905657-37942822
**MIEN1**	C17orf37, C35, MGC14832, ORB3, Rdx12, XTP4	ENSG00000141741	Migration and invasion enhancer 1	Q9BRT3		17	39728496-39730532
**MISP**	C19orf21, Caprice, DKFZp686H18209, MISP1	ENSG00000099812	Mitotic spindle positioning	Q8IVT2		19	751112-764318
**MLKL**	FLJ34389	ENSG00000168404	Mixed lineage kinase domain like pseudokinase	Q8NB16		16	74671855-74700960
**MLNR**	GPR38	ENSG00000102539	Motilin receptor	O43193		13	49220338-49222377
**MME**	CALLA, CD10, NEP	ENSG00000196549	Membrane metalloendopeptidase	P08473		3	155024124-155183704
**MMP25**	MMP20, MMPL1, MT6-MMP	ENSG00000008516	Matrix metallopeptidase 25	Q9NPA2		16	3046062-3060726
**MPL**	CD110, THPOR, TPOR	ENSG00000117400	MPL proto-oncogene, thrombopoietin receptor	P40238		1	43337818-43354466
**MPP1**	DXS552E, PEMP	ENSG00000130830	MAGUK p55 scaffold protein 1	Q00013	X		154778684-154821007
**MPP7**	FLJ32798	ENSG00000150054	MAGUK p55 scaffold protein 7	Q5T2T1		10	28050993-28334486
**MPZL1**	FLJ21047, PZR	ENSG00000197965	Myelin protein zero like 1	O95297		1	167721192-167791919
**MR1**	HLALS	ENSG00000153029	Major histocompatibility complex, class I-related	Q95460		1	181033374-181061938
**MRGPRF**	GPR140, GPR168, MGC21621, mrgF	ENSG00000172935	MAS related GPR family member F	Q96AM1		11	69004395-69013382
**MS4A1**	B1, Bp35, CD20, FMC7	ENSG00000156738	Membrane spanning 4-domains A1	P11836		11	60455846-60470752
**MSLN**	CAK1, MPF	ENSG00000102854	Mesothelin	Q13421		16	760734-768865
**MSN**		ENSG00000147065	Moesin	P26038	X		65588377-65741931
**MTM1**		ENSG00000171100	Myotubularin 1	Q13496	X		150568417-150673143
**MUC1**	ADMCKD, ADMCKD1, Ca15-3, CD227, EMA, KL-6, MCD, MCKD, MCKD1,	ENSG00000185499	Mucin 1, cell surface associated	P15941		1	155185824-155192916
**MYH11**	SMHC, SMMHC, SMMS-1	ENSG00000133392	Myosin heavy chain 11	P35749		16	15703135-15857028
**MYLK**	KRP, MLCK, MLCK1, MLCK108, MLCK210, MYLK-L, MYLK1, smMLCK, T	ENSG00000065534	Myosin light chain kinase	Q15746		3	123610049-123884332
**MYO10**	KIAA0799, MyoX	ENSG00000145555	Myosin X	Q9HD67		5	16661907-16936288
**MYO1B**	myr1	ENSG00000128641	Myosin IB	O43795		2	191245185-191425389
**MYO1C**	MyoIC, myr2, NMI	ENSG00000197879	Myosin IC	O00159		17	1464186-1492686
**MYO1G**	HA-2	ENSG00000136286	Myosin IG	B0I1T2		7	44962662-44979088
**MYOF**	FER1L3, KIAA1207	ENSG00000138119	Myoferlin	Q9NZM1		10	93306429-93482334
**NALCN**	bA430M15.1, CanIon, VGCNL1	ENSG00000102452	Sodium leak channel, non-selective	Q8IZF0		13	101053776-101416508
**NAP1L2**	BPX, MGC26243	ENSG00000186462	Nucleosome assembly protein 1 like 2	Q9ULW6	X		73212299-73214851
**NAPG**		ENSG00000134265	NSF attachment protein gamma	Q99747		18	10525905-10552764
**NCAM1**	CD56, NCAM	ENSG00000149294	Neural cell adhesion molecule 1	P13591		11	112961247-113278436
**NCAM2**	MGC51008, NCAM21	ENSG00000154654	Neural cell adhesion molecule 2	O15394		21	20998409-21543329
**NCKAP1L**	HEM1	ENSG00000123338	NCK associated protein 1 like	P55160		12	54497752-54548243
**NCS1**	FREQ, NCS-1	ENSG00000107130	Neuronal calcium sensor 1	P62166		9	130172404-130237303
**NECTIN4**	LNIR, nectin-4, PRR4, PVRL4	ENSG00000143217	Nectin cell adhesion molecule 4	Q96NY8		1	161070998-161089558
**NEO1**	HsT17534, IGDCC2, NGN, NTN1R2	ENSG00000067141	Neogenin 1	Q92859		15	73051710-73305205
**NEU1**	NEU	ENSG00000204386	Neuraminidase 1	Q99519		6	31857659-31862905
**NF1**		ENSG00000196712	Neurofibromin 1	P21359		17	31094927-31382116
**NF2**	ACN, BANF, merlin, merlin-1, SCH	ENSG00000186575	NF2, moesin-ezrin-radixin like (MERLIN) tumor suppressor	P35240		22	29603556-29698598
**NGFR**	CD271, p75NTR, TNFRSF16	ENSG00000064300	Nerve growth factor receptor	P08138		17	49495293-49515008
**NHS**		ENSG00000188158	NHS actin remodeling regulator	Q6T4R5	X		17375200-17735994
**NIBAN2**	bA356B19.6, C9orf88, DKFZP434H0820, FAM129B, FLJ13518, FLJ22151,	ENSG00000136830	Niban apoptosis regulator 2	Q96TA1		9	127505339-127578989
**NID2**		ENSG00000087303	Nidogen 2	Q14112		14	52004809-52069059
**NKTR**	p104	ENSG00000114857	Natural killer cell triggering receptor	P30414		3	42600655-42648735
**NLGN3**	ASPGX1, AUTSX1, HNL3, KIAA1480	ENSG00000196338	Neuroligin 3	Q9NZ94	X		71144821-71175255
**NMBR**	BB1, BB1R, BRS1	ENSG00000135577	Neuromedin B receptor	P28336		6	142058330-142147122
**NMT2**		ENSG00000152465	N-myristoyltransferase 2	O60551		10	15102584-15168693
**NMUR2**	NMU2R	ENSG00000132911	Neuromedin U receptor 2	Q9GZQ4		5	152391546-152433368
**NOS1**	nNOS, NOS	ENSG00000089250	Nitric oxide synthase 1	P29475		12	117208142-117452170
**NOTCH2**		ENSG00000134250	Notch receptor 2	Q04721		1	119911553-120100779
**NOTCH3**	CADASIL, CASIL	ENSG00000074181	Notch receptor 3	Q9UM47		19	15159038-15200995
**NPFFR2**	GPR74, NPFF2, NPGPR	ENSG00000056291	Neuropeptide FF receptor 2	Q9Y5X5		4	72031902-72148305
**NRAS**	N-ras	ENSG00000213281	NRAS proto-oncogene, GTPase	P01111		1	114704469-114716771
**NT5E**	CALJA, CD73, eN, eNT, NT5	ENSG00000135318	5'-nucleotidase ecto	P21589		6	85449584-85495791
**NTNG1**	KIAA0976, Lmnt1	ENSG00000162631	Netrin G1	Q9Y2I2		1	107140007-107484923
**NTNG2**	KIAA1857, Lmnt2, NTNG1	ENSG00000196358	Netrin G2	Q96CW9		9	132162058-132244526
**NTRK2**	TRKB	ENSG00000148053	Neurotrophic receptor tyrosine kinase 2	Q16620		9	84668375-85095751
**NTSR1**	NTR	ENSG00000101188	Neurotensin receptor 1	P30989		20	62708836-62762771
**NUMB**	C14orf41	ENSG00000133961	NUMB endocytic adaptor protein	P49757		14	73275107-73458617
**OCLN**	PPP1R115	ENSG00000197822	Occludin	Q16625		5	69492292-69558104
**OGT**	FLJ23071, HRNT1, MGC22921, O-GLCNAC, OGT1	ENSG00000147162	O-linked N-acetylglucosamine (GlcNAc) transferase	O15294	X		71533087-71575892
**OLFM4**	GC1, GW112, OlfD	ENSG00000102837	Olfactomedin 4	Q6UX06		13	53028813-53052057
**OLR1**	CLEC8A, LOX-1, SCARE1	ENSG00000173391	Oxidized low density lipoprotein receptor 1	P78380		12	10158301-10172138
**OPN3**	ECPN, encephalopsin, ERO, NMO-1, PPP1R116	ENSG00000054277	Opsin 3	Q9H1Y3		1	241590102-241677376
**OPRK1**	KOR, OPRK	ENSG00000082556	Opioid receptor kappa 1	P41145		8	53225724-53251637
**OR10P1**	OR10P1P, OR10P2P, OR10P3P, OST701	ENSG00000175398	Olfactory receptor family 10 subfamily P member 1	Q8NGE3		12	55636892-55637833
**OR13C2**		ENSG00000276119	Olfactory receptor family 13 subfamily C member 2	Q8NGS9		9	104604671-104605627
**OR13C5**		ENSG00000277556	Olfactory receptor family 13 subfamily C member 5	Q8NGS8		9	104598457-104599413
**OR2C3**	OR2C4, OR2C5P, OST742	ENSG00000196242	Olfactory receptor family 2 subfamily C member 3	Q8N628		1	247524677-247536440
**OR6C6**		ENSG00000188324	Olfactory receptor family 6 subfamily C member 6	A6NF89		12	55293988-55296569
**OSBPL6**	ORP6	ENSG00000079156	Oxysterol binding protein like 6	Q9BZF3		2	178194481-178402893
**OSBPL7**	MGC71150, ORP7	ENSG00000006025	Oxysterol binding protein like 7	Q9BZF2		17	47807372-47821834
**OXGR1**	aKGR, GPR80, GPR99, P2RY15, P2Y15	ENSG00000165621	Oxoglutarate receptor 1	Q96P68		13	96985713-96994730
**PACSIN2**	SDPII	ENSG00000100266	Protein kinase C and casein kinase substrate in neurons 2	Q9UNF0		22	42835412-43015149
**PAG1**	CBP, PAG	ENSG00000076641	Phosphoprotein membrane anchor with glycosphingolipid microdomains 1	Q9NWQ8		8	80967810-81112068
**PAIP1**		ENSG00000172239	Poly(A) binding protein interacting protein 1	Q9H074		5	43526267-43557758
**PAK1**		ENSG00000149269	P21 (RAC1) activated kinase 1	Q13153		11	77322017-77474635
**PAK4**		ENSG00000130669	P21 (RAC1) activated kinase 4	O96013		19	39125770-39182816
**PALM**	KIAA0270, PALM1	ENSG00000099864	Paralemmin	O75781		19	708935-748329
**PALM2AKAF**	PALM2-AKAP2	ENSG00000157654	PALM2 and AKAP2 fusion	Q8IXS6, Q		9	109498325-110172512
**PALS1**	FLJ12615, MPP5	ENSG00000072415	Protein associated with LIN7 1, MAGUK p55 family member	Q8N3R9		14	67240713-67336061
**PANX1**	MRS1, PX1, UNQ2529	ENSG00000110218	Pannexin 1	Q96RD7		11	94128841-94181968
**PANX2**	hPANX2, PX2	ENSG00000073150	Pannexin 2	Q96RD6		22	50170731-50180295
**PAQR8**	C6orf33, LMPB1, MPRB	ENSG00000170915	Progestin and adipoQ receptor family member 8	Q8TEZ7		6	52361421-52407777
**PARD3**	ASIP, Baz, Bazooka, PAR3, PARD3A, PPP1R118	ENSG00000148498	Par-3 family cell polarity regulator	Q8TEW0		10	34109560-34815325
**PARD3B**	ALS2CR19, PAR3beta, Par3L	ENSG00000116117	Par-3 family cell polarity regulator beta	Q8TEW8		2	204545475-205620162
**PARD6A**	PAR-6, PAR-6A, PAR6alpha, TAX40, TIP-40	ENSG00000102981	Par-6 family cell polarity regulator alpha	Q9NPB6		16	67660946-67662774
**PARVB**	CGI-56	ENSG00000188677	Parvin beta	Q9HBI1		22	43999211-44172939
**PATJ**	Cipp, INADL	ENSG00000132849	PATJ crumbs cell polarity complex component	Q8NI35		1	61742477-62178675
**PCDH1**	pc42	ENSG00000156453	Protocadherin 1	Q08174		5	141853090-141879246
**PCDH7**	BH-Pcdh, PPP1R120	ENSG00000169851	Protocadherin 7	O60245		4	30720369-31146805
**PCDHA4**	CNR1, CNRN1, CRNR1, PCDH-ALPHA4	ENSG00000204967	Protocadherin alpha 4	Q9UN74		5	140806929-141012347
**PCDHGB2**	PCDH-GAMMA-B2	ENSG00000253910	Protocadherin gamma subfamily B, 2	Q9Y5G2		5	141359994-141512975
**PDAP1**	HASPP28, PAP, PAP1	ENSG00000106244	PDGFA associated protein 1	Q13442		7	99392048-99408597
**PDE4A**	DPDE2	ENSG00000065989	Phosphodiesterase 4A	P27815		19	10416773-10469630
**PDE4D**	DPDE3	ENSG00000113448	Phosphodiesterase 4D	Q08499		5	58969038-60522120
**PDE9A**		ENSG00000160191	Phosphodiesterase 9A	O76083		21	42653621-42775509
**PDGFC**	fallotein, SCDGF	ENSG00000145431	Platelet derived growth factor C	Q9NRA1		4	156760454-156971799
**PDGFRA**	CD140a, GAS9, PDGFR2	ENSG00000134853	Platelet derived growth factor receptor alpha	P16234		4	54229280-54298245
**PDPN**	aggrus, D2-40, Gp38, GP40, PA2.26, T1A-2	ENSG00000162493	Podoplanin	Q86YL7		1	13583465-13617957
**PDZD2**	KIAA0300, PDZK3	ENSG00000133401	PDZ domain containing 2	O15018		5	31639131-32110932
**PECAM1**	CD31	ENSG00000261371	Platelet and endothelial cell adhesion molecule 1	P16284		17	64319415-64413776
**PHEX**	HPDR, HPDR1, HYP, HYP1, PEX, XLH	ENSG00000102174	Phosphate regulating endopeptidase X-linked	P78562	X		22032325-22494713
**PHLDB2**	FLJ21791, LL5b, LL5beta	ENSG00000144824	Pleckstrin homology like domain family B member 2	Q86SQ0		3	111732497-111976517
**PIGN**	MCD4, PIG-N	ENSG00000197563	Phosphatidylinositol glycan anchor biosynthesis class N	O95427		18	61905255-62187118
**PIK3CG**		ENSG00000105851	Phosphatidylinositol-4,5-bisphosphate 3-kinase catalytic subunit gamma	P48736		7	106865278-106908980
**PIP5K1A**		ENSG00000143398	Phosphatidylinositol-4-phosphate 5-kinase type 1 alpha	Q99755		1	151197949-151249536
**PKD2**	Pc-2, PC2, PKD4, TRPP2	ENSG00000118762	Polycystin 2, transient receptor potential cation channel	Q13563		4	88007635-88077777
**PKP2**		ENSG00000057294	Plakophilin 2	Q99959		12	32790745-32896777
**PKP3**		ENSG00000184363	Plakophilin 3	Q9Y446		11	392614-404908
**PKP4**	p0071	ENSG00000144283	Plakophilin 4	Q99569		2	158456952-158682879
**PLA2G6**	iPLA2, iPLA2beta, NBIA2, PARK14, PNPLA9	ENSG00000184381	Phospholipase A2 group VI	O60733		22	38111495-38214778
**PLAA**	DOA1, FLJ11281, FLJ12699, PLA2P, PLAP	ENSG00000137055	Phospholipase A2 activating protein	Q9Y263		9	26903372-26947242
**PLAUR**	CD87, UPAR, URKR	ENSG00000011422	Plasminogen activator, urokinase receptor	Q03405		19	43646095-43670547
**PLCB4**		ENSG00000101333	Phospholipase C beta 4	Q15147		20	9067825-9504593
**PLCG1**	NCKAP3, PLC-II, PLC1, PLC148, PLCgamma1	ENSG00000124181	Phospholipase C gamma 1	P19174		20	41136960-41196801
**PLEKHA7**	DKFZp686M22243	ENSG00000166689	Pleckstrin homology domain containing A7	Q6IQ23		11	16777297-17014415
**PLEKHG6**	FLJ10665, MYOGEF	ENSG00000008323	Pleckstrin homology and RhoGEF domain containing G6	Q3KR16		12	6310436-6328506
**PLEKHN1**	DKFZP434H2010	ENSG00000187583	Pleckstrin homology domain containing N1	Q494U1		1	966482-975865
**PLIN4**	KIAA1881, S3-12	ENSG00000167676	Perilipin 4	Q96Q06		19	4502192-4518486
**PLPP1**	LPP1, PAP-2a, PPAP2A	ENSG00000067113	Phospholipid phosphatase 1	O14494		5	55424854-55534969
**PLPP2**	LPP2, PAP-2c, PPAP2C	ENSG00000141934	Phospholipid phosphatase 2	O43688		19	281040-291403
**PLSCR1**	MMTRA1B	ENSG00000188313	Phospholipid scramblase 1	O15162		3	146515180-146544856

**PODXL**	gp135, Gp200, PC, PCLP, PDX, PODXL1	ENSG00000128567	Podocalyxin like	O00592	7	131500262-131558217
**POLE**	POLE1	ENSG00000177084	DNA polymerase epsilon, catalytic subunit	Q07864	12	132623753-132687376
**PPFIBP1**	hSGT2, hSgt2p, L2, SGT2	ENSG00000110841	PPFIA binding protein 1	Q86W92	12	27523431-27695564
**PPIP5K1**	HISPPD2A, IPS1, KIAA0377, VIP1	ENSG00000168781	Diphosphoinositol pentakisphosphate kinase 1	Q6PFW1	15	43533462-43590208
**PPP1R16B**	ANKRD4, KIAA0823, TIMAP	ENSG00000101445	Protein phosphatase 1 regulatory subunit 16B	Q96T49	20	38805697-38923024
**PPP3R1**	CALNB1, CNB, CNB1	ENSG00000221823	Protein phosphatase 3 regulatory subunit B, alpha	P63098	2	68178857-68256237
**PPP6R3**	C11orf23, DKFZp781E17107, DKFZp781E2374, DKFZp781O2362, FLJ110	ENSG00000110075	Protein phosphatase 6 regulatory subunit 3	Q5H9R7	11	68460731-68615334
**PRAME**	CT130, MAPE	ENSG00000185686	PRAME nuclear receptor transcriptional regulator	P78395	22	22547701-22559361
**PRKCA**	PKCA, PKCalpha	ENSG00000154229	Protein kinase C alpha	P17252	17	66302613-66810743
**PRKCE**		ENSG00000171132	Protein kinase C epsilon	Q02156	2	45651345-46187990
**PRKD1**	PKC-mu, PKCM, PKD, PRKCM	ENSG00000184304	Protein kinase D1	Q15139	14	29576479-30191898
**PRKD3**	EPK2, PRKCN	ENSG00000115825	Protein kinase D3	O94806	2	37250502-37324833
**PRR7**	MGC10772	ENSG00000131188	Proline rich 7, synaptic	Q8TB68	5	177446445-177456286
**PRRT2**	DKFZp547J199, DSPB3, DYT10, EKD1, FICCA, FLJ25513, ICCA, IFITMD1	ENSG00000167371	Proline rich transmembrane protein 2	Q7Z6L0	16	29811382-29815892
**PSCA**	lncPSCA	ENSG00000167653	Prostate stem cell antigen	O43653	8	142670308-142682725
**PSKH1**		ENSG00000159792	Protein serine kinase H1	P11801	16	67893254-67929676
**PSMC5**	p45, p45/SUG, RPT6, S8, SUG-1, SUG1, TBP10, TRIP1	ENSG00000087191	Proteasome 26S subunit, ATPase 5	P62195	17	63827152-63832026
**PSTPIP1**	CD2BP1, CD2BP1L, CD2BP1S, H-PIP, PAPAS, PSTPIP	ENSG00000140368	Proline-serine-threonine phosphatase interacting protein 1	O43586	15	76993359-77037475
**PTAFR**		ENSG00000169403	Platelet activating factor receptor	P25105	1	28147166-28193936
**PTCH2**		ENSG00000117425	Patched 2	Q9Y6C5	1	44819844-44843253
**PTH1R**	PTHR, PTHR1	ENSG00000160801	Parathyroid hormone 1 receptor	Q03431	3	46877721-46903799
**PTK2B**	CADTK, CAKB, FAK2, PTK, PYK2, RAFTK	ENSG00000120899	Protein tyrosine kinase 2 beta	Q14289	8	27311482-27459391
**PTK7**	CCK4	ENSG00000112655	Protein tyrosine kinase 7 (inactive)	Q13308	6	43076307-43161719
**PTPRH**	SAP-1	ENSG00000080031	Protein tyrosine phosphatase receptor type H	Q9HD43	19	55181247-55209506
**PTPRJ**	CD148, DEP1, HPTPeta	ENSG00000149177	Protein tyrosine phosphatase receptor type J	Q12913	11	47980425-48170839
**PTPRK**	R-PTP-kappa	ENSG00000152894	Protein tyrosine phosphatase receptor type K	Q15262	6	127968785-128520616
**PTPRM**	hR-PTPu, PTPRL1, RPTPU	ENSG00000173482	Protein tyrosine phosphatase receptor type M	P28827	18	7566782-8406861
**PTPRR**	EC-PTP, PCPTP1, PTP-SL, PTPBR7, PTPRQ	ENSG00000153233	Protein tyrosine phosphatase receptor type R	Q15256	12	70638073-70920738
**PTPRS**		ENSG00000105426	Protein tyrosine phosphatase receptor type S	Q13332	19	5158495-5340812
**PVR**	CD155, HVED, Necl-5, NECL5, PVS, Tage4	ENSG00000073008	PVR cell adhesion molecule	P15151	19	44643798-44666162
**PXK**	FLJ20335, Slob	ENSG00000168297	PX domain containing serine/threonine kinase like	Q7Z7A4	3	58332880-58426127
**RAB13**		ENSG00000143545	RAB13, member RAS oncogene family	P51153	1	153981617-153986358
**RAB23**		ENSG00000112210	RAB23, member RAS oncogene family	Q9ULC3	6	57186992-57222307
**RAB25**	CATX-8	ENSG00000132698	RAB25, member RAS oncogene family	P57735	1	156061160-156070504
**RAB8A**	MEL, RAB8	ENSG00000167461	RAB8A, member RAS oncogene family	P61006	19	16111889-16134234
**RAET1G**	ULBP5	ENSG00000203722	Retinoic acid early transcript 1G	Q6H3X3	6	149916878-149923121
**RAET1L**		ENSG00000155918	Retinoic acid early transcript 1L	Q5VY80	6	150018334-150025532
**RAF1**	c-Raf, CRAF, Raf-1	ENSG00000132155	Raf-1 proto-oncogene, serine/threonine kinase	P04049	3	12582101-12664201
**RALA**	RAL	ENSG00000006451	RAS like proto-oncogene A	P11233	7	39623565-39708120
**RALBP1**	RIP, RIP1, RLIP76	ENSG00000017797	RalA binding protein 1	Q15311	18	9475009-9538114
**RAMP3**		ENSG00000122679	Receptor activity modifying protein 3	O60896	7	45157791-45186302
**RAPGEF2**	DKFZP586O1422, KIAA0313, PDZ-GEF1, PDZGEF1, RA-GEF	ENSG00000109756	Rap guanine nucleotide exchange factor 2	Q9Y4G8	4	159103013-159360174
**RAPH1**	ALS2CR18, ALS2CR9, KIAA1681	ENSG00000173166	Ras association (RalGDS/AF-6) and pleckstrin homology domains 1	Q70E73	2	203394345-203535335
**RASAL3**	FLJ21438	ENSG00000105122	RAS protein activator like 3	Q86YV0	19	15451624-15464544
**RASGRP1**	CalDAG-GEFII, RASGRP	ENSG00000172575	RAS guanyl releasing protein 1	O95267	15	38488103-38565575
**RASGRP2**	CALDAG-GEFI	ENSG00000068831	RAS guanyl releasing protein 2	Q7LDG7	11	64726911-64745456
**RASGRP4**		ENSG00000171777	RAS guanyl releasing protein 4	Q8TDF6	19	38409051-38426305
**RC3H1**	KIAA2025, RNF198, roquin, RP5-1198E17.5	ENSG00000135870	Ring finger and CCCH-type domains 1	Q5TC82	1	173931084-174022357
**RCCD1**	MGC14386	ENSG00000166965	RCC1 domain containing 1	A6NED2	15	90954881-90963125
**RDX**	DFNB24	ENSG00000137710	Radixin	P35241	11	109864295-110296712
**RECK**	hRECK, ST15	ENSG00000122707	Reversion inducing cysteine rich protein with kazal motifs	O95980	9	36036913-36124455
**RELL1**		ENSG00000181826	RELT like 1	Q8IUW5	4	37590800-37686376
**REPS1**		ENSG00000135597	RALBP1 associated Eps domain containing 1	Q96D71	6	138903493-138988261
**RET**	CDHF12, CDHR16, HSCR1, MEN2A, MEN2B, MTC1, PTC, RET51	ENSG00000165731	Ret proto-oncogene	P07949	10	43077064-43130351
**RGS19**	GAIP, RGSGAIP	ENSG00000171700	Regulator of G protein signaling 19	P49795	20	64073181-64079988
**RGS3**	C2PA, FLJ20370, PDZ-RGS3	ENSG00000138835	Regulator of G protein signaling 3	P49796	9	113444731-113597743
**RHEX**	C1orf186, FLJ16052	ENSG00000263961	Regulator of hemoglobinization and erythroid cell expansion	Q6ZWK4	1	206053173-206102449
**RHOA**	ARH12, ARHA, Rho12, RHOH12	ENSG00000067560	Ras homolog family member A	P61586	3	49359139-49412998
**RIGI**	DDX58, DKFZp434J1111, FLJ13599, RIG-1, RIG-I, RIG1	ENSG00000107201	RNA sensor RIG-I	O95786	9	32455302-32526208
**RND1**	ARHS, Rho6, RHOS	ENSG00000172602	Rho family GTPase 1	Q92730	12	48857145-48865870
**RNF146**	dactylidin, dJ351K20.1, DKFZp434O1427	ENSG00000118518	Ring finger protein 146	Q9NTX7	6	127266726-127288567
**ROBO1**	DUTT1, FLJ21882, SAX3	ENSG00000169855	Roundabout guidance receptor 1	Q9Y6N7	3	78597239-79767998
**RP2**	NM23-H10, NME10, TBCCD2	ENSG00000102218	RP2 activator of ARL3 GTPase	O75695	X	46837043-46882358
**RPSA**	37LRP, LAMR1, LRP, p40, SA	ENSG00000168028	Ribosomal protein SA	P08865	3	39406716-39426255
**RRP12**	KIAA0690	ENSG00000052749	Ribosomal RNA processing 12 homolog	Q5JTH9	10	97356358-97426076
**RSC1A1**	RS1	ENSG00000215695	Regulator of solute carriers 1	Q92681	1	15659713-15662033
**RTN4R**	NOGOR	ENSG00000040608	Reticulon 4 receptor	Q9BZR6	22	20241415-20283246
**RTN4RL1**	DKFZp547J144, NgR3, NGRH2	ENSG00000185924	Reticulon 4 receptor like 1	Q86UN2	17	1934677-2025334
**S100A3**	S100E	ENSG00000188015	S100 calcium binding protein A3	P33764	1	153547329-153549258
**S100A6**	2A9, CABP, CACY, PRA	ENSG00000197956	S100 calcium binding protein A6	P06703	1	153534599-153536244
**S100A9**	60B8AG, CAGB, CFAG, CGLB, LIAG, MAC387, MIF, MRP-14, MRP14, NIF,	ENSG00000163220	S100 calcium binding protein A9	P06702	1	153357854-153361023
**SCEL**	FLJ21667, MGC22531	ENSG00000136155	Sciellin	O95171	13	77535674-77645263
**SCN1A**	FEB3, GEFSP2, HBSCI, NAC1, Nav1.1, SCN1, SMEI	ENSG00000144285	Sodium voltage-gated channel alpha subunit 1	P35498	2	165984641-166149214
**SCN3B**	HSA243396, SCNB3	ENSG00000166257	Sodium voltage-gated channel beta subunit 3	Q9NY72	11	123629187-123655244
**SCN5A**	CDCD2, CMD1E, CMPD2, HB1, HB2, HBBD, HH1, ICCD, IVF, LQT3, Nav1.	ENSG00000183873	Sodium voltage-gated channel alpha subunit 5	Q14524	3	38548057-38649687
**SCN8A**	CerIII, CIAT, MED, NaCh6, Nav1.6, PN4	ENSG00000196876	Sodium voltage-gated channel alpha subunit 8	Q9UQD0	12	51590266-51812864
**SCNN1D**	dNaCh, ENaCdelta	ENSG00000162572	Sodium channel epithelial 1 subunit delta	P51172	1	1280436-1292029
**SCNN1G**	ENaCgamma, SCNEG	ENSG00000166828	Sodium channel epithelial 1 subunit gamma	P51170	16	23182745-23216883
**SCRIB**	KIAA0147, SCRB1, Vartul	ENSG00000180900	Scribble planar cell polarity protein	Q14160	8	143790920-143815773
**SCUBE3**	CEGF3, FLJ34743	ENSG00000146197	Signal peptide, CUB domain and EGF like domain containing 3	Q8IX30	6	35213956-35253079
**SDC2**	CD362, fibroglycan, HSPG, HSPG1, SYND2	ENSG00000169439	Syndecan 2	P34741	8	96493813-96611790
**SDE2**	C1orf55, FLJ35382	ENSG00000143751	SDE2 telomere maintenance homolog	Q6IQ49	1	225982702-225999343
**SEMA4D**	C9orf164, CD100, coll-4, FLJ39737, SEMAJ	ENSG00000187764	Semaphorin 4D	Q92854	9	89360787-89498130
**SERINC5**	C5orf12, TPO1	ENSG00000164300	Serine incorporator 5	Q86VE9	5	80111651-80256048
**SHANK1**	SPANK-1, SSTRIP, synamon	ENSG00000161681	SH3 and multiple ankyrin repeat domains 1	Q9Y566	19	50659255-50719802
**SIGLEC10**	MGC126774, PRO940, SIGLEC-10, SLG2	ENSG00000142512	Sialic acid binding Ig like lectin 10	Q96LC7	19	51410020-51417803
**SIGLEC14**		ENSG00000254415	Sialic acid binding Ig like lectin 14	Q08ET2	19	51639478-51646825
**SIPA1L1**	E6TP1, KIAA0440, SPAR1	ENSG00000197555	Signal induced proliferation associated 1 like 1	O43166	14	71320449-71741229
**SIPA1L3**	KIAA0545, SPAR3	ENSG00000105738	Signal induced proliferation associated 1 like 3	O60292	19	37907208-38208369
**SLC13A3**	NADC3, SDCT2	ENSG00000158296	Solute carrier family 13 member 3	Q8WWT9	20	46557823-46684467
**SLC13A5**	INDY, NACT	ENSG00000141485	Solute carrier family 13 member 5	Q86YT5	17	6684719-6713377
**SLC14A1**	HsT1341, JK, RACH1, RACH2	ENSG00000141469	Solute carrier family 14 member 1 (Kidd blood group)	Q13336	18	45687025-45752520
**SLC16A1**	MCT, MCT1	ENSG00000155380	Solute carrier family 16 member 1	P53985	1	112911847-112957593
**SLC16A10**	MCT10, TAT1	ENSG00000112394	Solute carrier family 16 member 10	Q8TF71	6	111087503-111231194
**SLC16A3**	MCT3, MCT4	ENSG00000141526	Solute carrier family 16 member 3	O15427	17	82217934-82261129
**SLC16A7**	MCT2	ENSG00000118596	Solute carrier family 16 member 7	O60669	12	59596029-59789855
**SLC17A5**	AST, ISSD, NSD, SD, SIALIN, SIASD, SLD	ENSG00000119899	Solute carrier family 17 member 5	Q9NRA2	6	73593379-73653992
**SLC1A5**	AAAT, ASCT2, M7V1, RDRC	ENSG00000105281	Solute carrier family 1 member 5	Q15758	19	46774883-46788594
**SLC1A6**	EAAT4	ENSG00000105143	Solute carrier family 1 member 6	P48664	19	14950033-15022990
**SLC20A1**	Glvr-1, GLVR1, PiT-1, PiT1	ENSG00000144136	Solute carrier family 20 member 1	Q8WUM9	2	112645939-112663825
**SLC24A2**	NCKX2	ENSG00000155886	Solute carrier family 24 member 2	Q9UI40	9	19507452-19789037
**SLC26A6**	DKFZp586E1422	ENSG00000225697	Solute carrier family 26 member 6	Q9BXS9	3	48625723-48635493
**SLC28A1**	CNT1	ENSG00000156222	Solute carrier family 28 member 1	O00337	15	84884654-84945798
**SLC2A1**	CSE, DYT18, DYT9, GLUT, GLUT-1, GLUT1, HTLVR	ENSG00000117394	Solute carrier family 2 member 1	P11166	1	42925353-42958893
**SLC2A12**	GLUT12, GLUT8	ENSG00000146411	Solute carrier family 2 member 12	Q8TD20	6	133987581-134052624
**SLC2A14**	GLUT14, SLC2A3P3	ENSG00000173262	Solute carrier family 2 member 14	Q8TDB8	12	7812512-7891148
**SLC2A2**	GLUT-2, GLUT2	ENSG00000163581	Solute carrier family 2 member 2	P11168	3	170996347-171026743
**SLC2A3**	GLUT3	ENSG00000059804	Solute carrier family 2 member 3	P11169	12	7919230-7936187
**SLC2A5**	GLUT5	ENSG00000142583	Solute carrier family 2 member 5	P22732	1	9035106-9088478
**SLC30A1**	ZNT1, ZRC1	ENSG00000170385	Solute carrier family 30 member 1	Q9Y6M5	1	211571568-211579161
**SLC30A8**	ZnT-8, ZNT8	ENSG00000164756	Solute carrier family 30 member 8	Q8IWU4	8	116950273-117176714
**SLC34A1**	NAPI-3, NPT2, NPTIIa, SLC11, SLC17A2	ENSG00000131183	Solute carrier family 34 member 1	Q06495	5	177379235-177398848
**SLC34A2**	NAPI-3B	ENSG00000157765	Solute carrier family 34 member 2	O95436	4	25648011-25678748
**SLC35G2**	DKFZp564K2464, MGC3295, TMEM22	ENSG00000168917	Solute carrier family 35 member G2	Q8TBE7	3	136818647-136855888
**SLC38A2**	ATA2, KIAA1382, SAT2, SNAT2	ENSG00000134294	Solute carrier family 38 member 2	Q96QD8	12	46358188-46372773
**SLC39A6**	LIV-1	ENSG00000141424	Solute carrier family 39 member 6	Q13433	18	36108531-36129385
**SLC3A2**	4F2, 4F2HC, 4T2HC, CD98, CD98HC, MDU1, NACAE	ENSG00000168003	Solute carrier family 3 member 2	P08195	11	62856004-62888880
**SLC40A1**	FPN1, HFE4, IREG1, MTP1, SLC11A3	ENSG00000138449	Solute carrier family 40 member 1	Q9NP59	2	189560590-189583758
**SLC41A3**	FLJ20473	ENSG00000114544	Solute carrier family 41 member 3	Q96GZ6	3	126006357-126101561
**SLC46A1**	HCP1, hPCFT, HsPCFT, MGC9564, PCFT	ENSG00000076351	Solute carrier family 46 member 1	Q96NT5	17	28394642-28407197
**SLC4A2**	AE2, BND3L, EPB3L1, HKB3, NBND3	ENSG00000164889	Solute carrier family 4 member 2	P04920	7	151057210-151076526
**SLC4A4**	hhNMC, HNBC1, NBC1, NBC2, pNBC, SLC4A5	ENSG00000080493	Solute carrier family 4 member 4	Q9Y6R1	4	71062667-71572087
**SLC4A7**	NBC3, SBC2, SLC4A6	ENSG00000033867	Solute carrier family 4 member 7	Q9Y6M7	3	27372721-27484420
**SLC5A1**	D22S675, NAGT, SGLT1	ENSG00000100170	Solute carrier family 5 member 1	P13866	22	32043261-32113029
**SLC5A12**	MGC52019, SMCT2	ENSG00000148942	Solute carrier family 5 member 12	Q1EHB4	11	26667020-26723427
**SLC5A3**	SMIT, SMIT1	ENSG00000198743	Solute carrier family 5 member 3	P53794	21	34073578-34106260
**SLC7A2**	ATRC2, CAT-2, HCAT2	ENSG00000003989	Solute carrier family 7 member 2	P52569	8	17497088-17570573
**SLC7A5**	CD98, D16S469E, E16, LAT1, MPE16	ENSG00000103257	Solute carrier family 7 member 5	Q01650	16	87830023-87869507
**SLC7A6**	KIAA0245, LAT-2, LAT3, y+LAT-2	ENSG00000103064	Solute carrier family 7 member 6	Q92536	16	68264516-68301823

**SLC8A1**	NCX1	ENSG00000183023	Solute carrier family 8 member A1	P32418		2	40097270-40611053
**SLC9A1**	APNH, NHE-1, NHE1, PPP1R143	ENSG00000090020	Solute carrier family 9 member A1	P19634		1	27098809-27166981
**SLC9A2**	NHE2	ENSG00000115616	Solute carrier family 9 member A2	Q9UBY0		2	102619553-102711355
**SLC9A3**	NHE-3, NHE3	ENSG00000066230	Solute carrier family 9 member A3	P48764		5	470456-524449
**SLC9A3R2**	E3KARP, NHERF-2, SIP-1, TKA-1	ENSG00000065054	SLC9A3 regulator 2	Q15599		16	2025356-2039026
**SLC9A6**	KIAA0267, NHE-6, NHE6	ENSG00000198689	Solute carrier family 9 member A6	Q92581	X		135973841-136047269
**SLC9C2**	MGC43026, SLC9A11	ENSG00000162753	Solute carrier family 9 member C2 (putative)	Q5TAH2		1	173500460-173603072
**SLCO1A2**	OATP, OATP-A, OATP1A2, SLC21A3	ENSG00000084453	Solute carrier organic anion transporter family member 1A2	P46721		12	21264600-21419594
**SLCO1B1**	LST-1, OATP-C, OATP1B1, SLC21A6	ENSG00000134538	Solute carrier organic anion transporter family member 1B1	Q9Y6L6		12	21131194-21239796
**SLCO1B3**	OATP1B3, OATP8, SLC21A8	ENSG00000111700	Solute carrier organic anion transporter family member 1B3	Q9NPD5		12	20810702-20916911
**SLCO4A1**	OATP-E, OATP4A1, SLC21A12	ENSG00000101187	Solute carrier organic anion transporter family member 4A1	Q96BD0		20	62642503-62685785
**SLCO5A1**	OATP-J, OATP5A1, OATPRP4, SLC21A15	ENSG00000137571	Solute carrier organic anion transporter family member 5A1	Q9H2Y9		8	69667046-69834978
**SLIT3**	MEGF5, SLIL2, Slit-3, SLIT1, slit2	ENSG00000184347	Slit guidance ligand 3	O75094		5	168661733-169301139
**SLITRK6**	FLJ22774	ENSG00000184564	SLIT and NTRK like family member 6	Q9H5Y7		13	85792790-85806683
**SMAGP**	hSMAGP, MGC149453, MGC149454	ENSG00000170545	Small cell adhesion glycoprotein	Q0VAQ4		12	51244558-51270415
**SNAP23**	HsT17016, SNAP23A, SNAP23B	ENSG00000092531	Synaptosome associated protein 23	O00161		15	42491233-42545356
**SNTB2**	D16S2531E, EST25263, SNT2B2, SNT3, SNTL	ENSG00000168807	Syntrophin beta 2	Q13425		16	69187147-69309052
**SNTG2**	G2SYN, SYN5	ENSG00000172554	Syntrophin gamma 2	Q9NY99		2	950849-1367613
**SNX9**	SDP1, SH3PX1, SH3PXD3A	ENSG00000130340	Sorting nexin 9	Q9Y5X1		6	157700387-157945077
**SORBS1**	CAP, FLJ12406, KIAA1296, ponsin, SH3D5, sh3p12	ENSG00000095637	Sorbin and SH3 domain containing 1	Q9BX66		10	95311771-95561414
**SORT1**	Gp95, NT3	ENSG00000134243	Sortilin 1	Q99523		1	109309568-109397918
**SPG11**	ALS5, FLJ21439, KIAA1840	ENSG00000104133	SPG11 vesicle trafficking associated, spatacsin	Q96JI7		15	44554818-44663688
**SPHK1**	SPHK	ENSG00000176170	Sphingosine kinase 1	Q9NYA1		17	76376584-76387860
**SPINT1**	HAI, HAI-1, MANSC2	ENSG00000166145	Serine peptidase inhibitor, Kunitz type 1	O43278		15	40844018-40858207
**SPIRE1**	KIAA1135, spir-1	ENSG00000134278	Spire type actin nucleation factor 1	Q08AE8		18	12446512-12658107
**SPPL2B**	IMP4, KIAA1532, PSL1	ENSG00000005206	Signal peptide peptidase like 2B	Q8TCT7		19	2328615-2355095
**SPPL3**	DKFZP586C1324, IMP2, MGC126674, MGC126676, MGC90402, PSL4	ENSG00000157837	Signal peptide peptidase like 3	Q8TCT6		12	120762510-120904358
**SRC**	ASV, c-src, SRC1	ENSG00000197122	SRC proto-oncogene, non-receptor tyrosine kinase	P12931		20	37344685-37406050
**SSTR5**		ENSG00000162009	Somatostatin receptor 5	P35346		16	1072747-1081454
**STAMBP**	AMSH	ENSG00000124356	STAM binding protein	O95630		2	73828916-73873659
**STEAP4**	FLJ23153, SchLAH, STAMP2, TIARP, TNFAIP9	ENSG00000127954	STEAP4 metalloreductase	Q687X5		7	88270892-88306894
**STK10**	LOK, PRO2729	ENSG00000072786	Serine/threonine kinase 10	O94804		5	172042079-172188224
**STOM**	BND7, EPB7, EPB72	ENSG00000148175	Stomatin	P27105		9	121338987-121370304
**STOML2**	HSPC108, SLP-2	ENSG00000165283	Stomatin like 2	Q9UJZ1		9	35099776-35103195
**STOML3**	Epb7.2l, SRO	ENSG00000133115	Stomatin like 3	Q8TAV4		13	38965925-38990859
**STX18**	Ufe1	ENSG00000168818	Syntaxin 18	Q9P2W9		4	4415742-4542346
**SVIL**		ENSG00000197321	Supervillin	O95425		10	29457338-29736959
**SWAP70**	KIAA0640, SWAP-70	ENSG00000133789	Switching B cell complex subunit SWAP70	Q9UH65		11	9664077-9752993
**SYMPK**	Pta1, SPK, SYM	ENSG00000125755	Symplekin scaffold protein	Q92797		19	45815410-45863194
**SYTL2**	CHR11SYT, exophilin-4, FLJ20163, FLJ21219, KIAA1597, MGC102768, PI	ENSG00000137501	Synaptotagmin like 2	Q9HCH5		11	85694224-85811159
**SYVN1**	DER3, HRD1	ENSG00000162298	Synoviolin 1	Q86TM6		11	65121780-65134532
**TACR1**	NK1R, NKIR, SPR, TAC1R	ENSG00000115353	Tachykinin receptor 1	P25103		2	75046463-75199520
**TACR2**	NK2R, NKNAR, SKR, TAC2R	ENSG00000075073	Tachykinin receptor 2	P21452		10	69403903-69416918
**TACSTD2**	EGP-1, GA733-1, M1S1, TROP2	ENSG00000184292	Tumor associated calcium signal transducer 2	P09758		1	58575433-58577252
**TAS1R3**	T1R3	ENSG00000169962	Taste 1 receptor member 3	Q7RTX0		1	1331280-1335314
**TBC1D10A**	AC004997.C22.2, EPI64, TBC1D10	ENSG00000099992	TBC1 domain family member 10A	Q9BXI6		22	30291990-30326947
**TBC1D24**	DFNA65, DFNB86, KIAA1171, TLDC6	ENSG00000162065	TBC1 domain family member 24	Q9ULP9		16	2475051-2509560
**TBC1D30**	KIAA0984	ENSG00000111490	TBC1 domain family member 30	Q9Y2I9		12	64759484-64881033
**TBXA2R**		ENSG00000006638	Thromboxane A2 receptor	P21731		19	3594507-3606875
**TCAF2**	FAM115C, FAM139A, FLJ40722, GATD9B	ENSG00000170379	TRPM8 channel associated factor 2	A6NFQ2		7	143620952-143730410
**TCAF2C**		ENSG00000283528	TRPM8 channel associated factor 2C			7	143639230-143647646
**TEC**	PSCTK4	ENSG00000135605	Tec protein tyrosine kinase	P42680		4	48135783-48269838
**TEK**	CD202b, TIE-2, TIE2, VMCM, VMCM1	ENSG00000120156	TEK receptor tyrosine kinase	Q02763		9	27109141-27230174
**TGFBR1**	ACVRLK4, ALK-5, ALK5, ESS1, MSSE, TBR-i, TBRI	ENSG00000106799	Transforming growth factor beta receptor 1	P36897		9	99104038-99154192
**TGFBR2**	MFS2, TBR-ii, TBRII	ENSG00000163513	Transforming growth factor beta receptor 2	P37173		3	30606601-30694142
**TIAM1**		ENSG00000156299	TIAM Rac1 associated GEF 1	Q13009		21	31118416-31559977
**TIGD7**	Sancho	ENSG00000140993	Tigger transposable element derived 7	Q6NT04		16	3298808-3305729
**TJP1**	DKFZp686M05161, MGC133289, ZO-1	ENSG00000104067	Tight junction protein 1	Q07157		15	29699367-29968915
**TJP2**	DFNA51, X104, ZO-2, ZO2	ENSG00000119139	Tight junction protein 2	Q9UDY2		9	69121264-69274615
**TJP3**	ZO-3	ENSG00000105289	Tight junction protein 3	O95049		19	3708362-3750813
**TLN1**	ILWEQ, TLN	ENSG00000137076	Talin 1	Q9Y490		9	35696948-35732195
**TLR2**	CD282, TIL4	ENSG00000137462	Toll like receptor 2	O60603		4	153684070-153706260
**TLR4**	ARMD10, CD284, hToll, TLR-4	ENSG00000136869	Toll like receptor 4	O00206		9	117704175-117724735
**TM4SF20**	FLJ22800, TCCE518	ENSG00000168955	Transmembrane 4 L six family member 20	Q53R12		2	227362038-227381995
**TMCO5A**	MGC35118, TMCO5	ENSG00000166069	Transmembrane and coiled-coil domains 5A	Q8N6Q1		15	37921939-37967724
**TMEM266**	C15orf27, FLJ38190	ENSG00000169758	Transmembrane protein 266	Q2M3C6		15	76059958-76229121
**TMEM63B**	C6orf110, dJ421H19.2, DKFZp434P0531	ENSG00000137216	Transmembrane protein 63B	Q5T3F8		6	44126914-44155519
**TMEM65**		ENSG00000164983	Transmembrane protein 65	Q6PI78		8	124306189-124372701
**TMPRSS2**	PRSS10	ENSG00000184012	Transmembrane serine protease 2	O15393		21	41464300-41531116
**TNFRSF11A**	CD265, FEO, LOH18CR1, ODFR, PDB2, RANK, TRANCE-R	ENSG00000141655	TNF receptor superfamily member 11a	Q9Y6Q6		18	62325287-62391288
**TNFRSF14**	ATAR, CD270, HVEA, HVEM, LIGHTR, TR2	ENSG00000157873	TNF receptor superfamily member 14	Q92956		1	2555639-2565382
**TNFRSF21**	CD358, DR6	ENSG00000146072	TNF receptor superfamily member 21	O75509		6	47231532-47309905
**TNFRSF8**	CD30, D1S166E, KI-1	ENSG00000120949	TNF receptor superfamily member 8	P28908		1	12063303-12144207
**TNFSF13B**	BAFF, BLYS, CD257, TALL-1, TALL1, THANK, TNFSF20	ENSG00000102524	TNF superfamily member 13b	Q9Y275		13	108251240-108308484
**TNK2**	ACK, ACK1, p21cdc42Hs	ENSG00000061938	Tyrosine kinase non receptor 2	Q07912		3	195863364-195911945
**TP53BP2**	53BP2, ASPP2, PPP1R13A	ENSG00000143514	Tumor protein p53 binding protein 2	Q13625		1	223779893-223845954
**TRABD2B**	TIKI2	ENSG00000269113	TraB domain containing 2B	A6NFA1		1	47760528-47997385
**TRAIP**	RNF206, TRIP	ENSG00000183763	TRAF interacting protein	Q9BWF2		3	49828601-49856574
**TRAT1**	HSPC062, TCRIM, TRIM	ENSG00000163519	T cell receptor associated transmembrane adaptor 1	Q6PIZ9		3	108822770-108855005
**TRDN**	TRISK	ENSG00000186439	Triadin	Q13061		6	123216339-123637093
**TREML1**	dJ238O23.3, TLT1	ENSG00000161911	Triggering receptor expressed on myeloid cells like 1	Q86YW5		6	41149337-41154347
**TRPM4**	FLJ20041	ENSG00000130529	Transient receptor potential cation channel subfamily M member 4	Q8TD43		19	49157741-49211836
**TRPV2**	VRL, VRL-1, VRL1	ENSG00000187688	Transient receptor potential cation channel subfamily V member 2	Q9Y5S1		17	16415571-16437003
**TSPAN15**	NET-7, TM4SF15	ENSG00000099282	Tetraspanin 15	O95858		10	69451465-69507666
**TSPO2**	BZRPL1, dJ34B21.2	ENSG00000112212	Translocator protein 2	Q5TGU0		6	41042467-41044337
**TTC17**	FLJ10890	ENSG00000052841	Tetratricopeptide repeat domain 17	Q96AE7		11	43358920-43494933
**TULP3**	TUBL3	ENSG00000078246	TUB like protein 3	O75386		12	2877223-2941138
**TXK**	BTKL, PSCTK5, PTK4, RLK, TKL	ENSG00000074966	TXK tyrosine kinase	P42681		4	48066393-48134250
**TXNDC11**	EFP1	ENSG00000153066	Thioredoxin domain containing 11	Q6PKC3		16	11679083-11742857
**TYROBP**	DAP12, KARAP, PLO-SL, PLOSL	ENSG00000011600	Transmembrane immune signaling adaptor TYROBP	O43914		19	35904401-35908295
**UBE2C**	UBCH10	ENSG00000175063	Ubiquitin conjugating enzyme E2 C	O00762		20	45812576-45816957
**UBE2D3**	UbcH5C	ENSG00000109332	Ubiquitin conjugating enzyme E2 D3	P61077		4	102794383-102868896
**ULBP1**	RAET1I	ENSG00000111981	UL16 binding protein 1	Q9BZM6		6	149963943-149973715
**ULBP2**	RAET1H	ENSG00000131015	UL16 binding protein 2	Q9BZM5		6	149942014-149949235
**USP14**	TGT, Ubp6	ENSG00000101557	Ubiquitin specific peptidase 14	P54578		18	158383-214629
**USP29**		ENSG00000131864	Ubiquitin specific peptidase 29	Q9HBJ7		19	57119138-57131926
**UTRN**	DMDL, DRP, DRP1	ENSG00000152818	Utrophin	P46939		6	144285335-144853034
**VASP**		ENSG00000125753	Vasodilator stimulated phosphoprotein	P50552		19	45506579-45526989
**VIL1**	D2S1471, VIL	ENSG00000127831	Villin 1	P09327		2	218419121-218453295
**VPS36**	C13orf9, CGI-145, Eap45	ENSG00000136100	Vacuolar protein sorting 36 homolog	Q86VN1		13	52412602-52450634
**WDR1**	AIP1	ENSG00000071127	WD repeat domain 1	O75083		4	10068089-10116972
**XPC**	RAD4, XPCC	ENSG00000154767	XPC complex subunit, DNA damage recognition and repair factor	Q01831		3	14145147-14178621
**XRN1**	SEP1	ENSG00000114127	5'-3' exoribonuclease 1	Q8IZH2		3	142306607-142448062
**YES1**	c-yes, HsT441, Yes	ENSG00000176105	YES proto-oncogene 1, Src family tyrosine kinase	P07947		18	721588-812546
**YOD1**	DKFZp451J1719, DUBA8, OTUD2	ENSG00000180667	YOD1 deubiquitinase	Q5VVQ6		1	207043849-207052980
**ZBTB33**	kaiso, WUGSC:H_DJ525N14.1, ZNF-kaiso, ZNF348	ENSG00000177485	Zinc finger and BTB domain containing 33	Q86T24	X		120250752-120258398
**ZDHHC2**	DHHC2, ZNF372	ENSG00000104219	Zinc finger DHHC-type palmitoyltransferase 2	Q9UIJ5		8	17156482-17224799
**ZDHHC20**	DHHC20, FLJ25952	ENSG00000180776	Zinc finger DHHC-type palmitoyltransferase 20	Q5W0Z9		13	21348784-21459370
**ZDHHC22**	C14orf59	ENSG00000177108	Zinc finger DHHC-type palmitoyltransferase 22	Q8N966		14	77131270-77142734
**ZDHHC5**	DHHC5, KIAA1748, ZNF375	ENSG00000156599	Zinc finger DHHC-type palmitoyltransferase 5	Q9C0B5		11	57667747-57701182
**ZNF185**	SCELL	ENSG00000147394	Zinc finger protein 185 with LIM domain	O15231	X		152898067-152973481
**ZNF655**	VIK, VIK-1	ENSG00000197343	Zinc finger protein 655	Q8N720		7	99558406-99576453

Protein class	Biological process
Enzymes, Predicted intracellular proteins	Endocytosis
Essential proteins, Plasma proteins, Predicted intracellular proteins	Angiogenesis, Differentiation
Disease related genes, Enzymes, FDA approved drug targets, Human disease related genes, Metabolic proteins, Plasma proteins, Predicted intracellular proteins, Predicted membrane proteins, Transporters	Cholesterol metabolism, Lipid metabolism, Steroid metabolism, Sterol metabolism, Transport
Disease related genes, Enzymes, Human disease related genes, Potential drug targets, Predicted intracellular proteins, Predicted membrane proteins, Transporters	Lipid transport, Phagocytosis, Transport
Cancer-related genes, CD markers, Disease related genes, Enzymes, Human disease related genes, Metabolic proteins, Plasma proteins, Predicted intracellular proteins, Predicted membrane proteins, Transporters	Lipid transport, Transport
Disease related genes, Enzymes, Human disease related genes, Metabolic proteins, Plasma proteins, Potential drug targets, Predicted intracellular proteins, Predicted membrane proteins, Transporters	Lipid transport, Transport
Disease related genes, Enzymes, Human disease related genes, Metabolic proteins, Potential drug targets, Predicted membrane proteins, Transporters	Transport
Cancer-related genes, Disease related genes, Enzymes, FDA approved drug targets, Human disease related genes, Metabolic proteins, Plasma proteins, Predicted membrane proteins, Transporters	Lipid transport, Transport
Cancer-related genes, Enzymes, Metabolic proteins, Predicted intracellular proteins, Predicted membrane proteins, Transporters	Lipid transport, Transport
Enzymes, Metabolic proteins, Plasma proteins, Predicted membrane proteins, Transporters	Lipid transport, Transport
Cancer-related genes, Disease related genes, Enzymes, Human disease related genes, Plasma proteins, Potential drug targets, Predicted membrane proteins, Transporters	Sensory transduction, Transport, Vision
Cancer-related genes, CD markers, Enzymes, Human disease related genes, Metabolic proteins, Predicted intracellular proteins, Predicted membrane proteins, Transporters	Lipid transport, Transport
Cancer-related genes, Disease related genes, G-protein coupled receptors, Potential drug targets, Predicted membrane proteins	Cell adhesion, Host-virus interaction
Disease related genes, Enzymes, FDA approved drug targets, Human disease related genes, Metabolic proteins, Plasma proteins, Predicted intracellular proteins, Predicted membrane proteins	Cell adhesion, Nucleotide metabolism
Cancer-related genes, CD markers, Disease related genes, Enzymes, Human disease related genes, Plasma proteins, Potential drug targets, Predicted intracellular proteins, Predicted membrane proteins, Transporters	Notch signaling pathway
Predicted membrane proteins, Predicted secreted proteins, Transporters	Cell adhesion
Plasma proteins, Predicted membrane proteins, Predicted secreted proteins, Transporters	
Disease related genes, Enzymes, Human disease related genes, Metabolic proteins, Plasma proteins, Predicted intracellular proteins, Predicted membrane proteins	cAMP biosynthesis, Olfaction, Sensory transduction
Enzymes, Metabolic proteins, Plasma proteins, Predicted intracellular proteins, Predicted membrane proteins	cAMP biosynthesis
Enzymes, Metabolic proteins, Predicted intracellular proteins, Predicted membrane proteins	cAMP biosynthesis
Plasma proteins, Predicted intracellular proteins	
Predicted intracellular proteins	
Disease related genes, Human disease related genes, Predicted intracellular proteins	
G-protein coupled receptors, Predicted intracellular proteins, Predicted membrane proteins	Angiogenesis, Wnt signaling pathway
G-protein coupled receptors, Predicted intracellular proteins, Predicted membrane proteins, Transporters	
G-protein coupled receptors, Predicted intracellular proteins, Predicted membrane proteins	
Disease related genes, G-protein coupled receptors, Human disease related genes, Plasma proteins, Potential drug targets, Predicted membrane proteins, Transporters	
FDA approved drug targets, G-protein coupled receptors, Predicted membrane proteins, Transporters	
FDA approved drug targets, G-protein coupled receptors, Predicted membrane proteins	
Cancer-related genes, FDA approved drug targets, G-protein coupled receptors, Plasma proteins, Predicted intracellular proteins, Predicted membrane proteins	
FDA approved drug targets, G-protein coupled receptors, Human disease related genes, Predicted membrane proteins, Transporters	
Cancer-related genes, Disease related genes, Plasma proteins, Predicted intracellular proteins, RAS pathway related proteins	Cell adhesion
Cancer-related genes, Plasma proteins, Predicted membrane proteins, Predicted secreted proteins, Transporters	DNA damage, DNA repair, DNA replication, Inflammatory response, Phagocytosis
Plasma proteins, Predicted intracellular proteins	
Cancer-related genes, Predicted intracellular proteins	
Cancer-related genes, Plasma proteins, Predicted intracellular proteins	
Enzymes, Metabolic proteins, Predicted intracellular proteins, Predicted secreted proteins	Lipid metabolism
Enzymes, Metabolic proteins, Predicted intracellular proteins	Lipid metabolism
Plasma proteins, Predicted intracellular proteins	Apoptosis, Protein transport, Transport
Cancer-related genes, CD markers, Disease related genes, Enzymes, FDA approved drug targets, Human disease related genes, Plasma proteins, Predicted intracellular proteins, Predicted membrane proteins	
Enzymes, Metabolic proteins, Predicted intracellular proteins	Lipid metabolism
Cancer-related genes, Disease related genes, Human disease related genes, Predicted intracellular proteins	Wnt signaling pathway
Predicted intracellular proteins	Wnt signaling pathway
Predicted intracellular proteins	
Disease related genes, Human disease related genes, Plasma proteins, Potential drug targets, Predicted intracellular proteins, Transporters	Endocytosis, Protein transport, Transport
Cancer-related genes, Disease related genes, Human disease related genes, Plasma proteins, Potential drug targets, Predicted intracellular proteins, Transporters	
Enzymes, Predicted intracellular proteins	Ubl conjugation pathway
Predicted intracellular proteins	
Predicted intracellular proteins	
Predicted intracellular proteins	
Disease related genes, FDA approved drug targets, Plasma proteins, Predicted intracellular proteins, Predicted membrane proteins, Transporters	Ion transport, Transport
Disease related genes, Human disease related genes, Potential drug targets, Predicted intracellular proteins, Predicted membrane proteins, Transporters	
Predicted membrane proteins, Transporters	Ion transport, Transport
Disease related genes, Human disease related genes, Potential drug targets, Predicted membrane proteins, Transporters	Host-virus interaction, Ion transport, Lipid transport, Transport
CD markers, Enzymes, Metabolic proteins, Plasma proteins, Predicted membrane proteins	Angiogenesis, Differentiation, Host-virus interaction
Cancer-related genes, FDA approved drug targets, Plasma proteins, Predicted intracellular proteins, Predicted secreted proteins, Transporters	Adaptive immunity, Immunity, Inflammatory response, Innate immunity
Plasma proteins, Predicted intracellular proteins	
FDA approved drug targets, Metabolic proteins, Plasma proteins, Predicted intracellular proteins	
Disease related genes, Essential proteins, Human disease related genes, Plasma proteins, Potential drug targets, Predicted intracellular proteins, Transporters	Endocytosis, Protein transport, Transport
Plasma proteins, Predicted intracellular proteins	Apoptosis, DNA damage, Transcription, Transcription regulation
Predicted intracellular proteins	
Cancer-related genes, Disease related genes, Human disease related genes, Plasma proteins, Predicted intracellular proteins	Wnt signaling pathway
Predicted intracellular proteins	Cholesterol metabolism, Lipid metabolism, Lipid transport, Steroid metabolism, Sterol metabolism, Transport
Predicted membrane proteins	
Disease related genes, Human disease related genes, Predicted intracellular proteins	Cell cycle
Blood group antigen proteins, Metabolic proteins, Predicted membrane proteins, Transporters	Transport
Metabolic proteins, Predicted membrane proteins, Transporters	Transport
Predicted membrane proteins, Transporters	Transport
Blood group antigen proteins, Metabolic proteins, Plasma proteins, Predicted membrane proteins, Transporters	Transport
Human disease related genes, Metabolic proteins, Predicted membrane proteins, Transporters	Transport
Enzymes, Human disease related genes, Plasma proteins, Predicted intracellular proteins	
Predicted intracellular proteins	
Predicted intracellular proteins	
Predicted intracellular proteins	
Predicted intracellular proteins	
Predicted intracellular proteins	Angiogenesis, Differentiation, Transcription, Transcription regulation
Plasma proteins, Predicted intracellular proteins	
Disease related genes, Human disease related genes, Plasma proteins, Predicted intracellular proteins	
Predicted intracellular proteins	
Predicted intracellular proteins	
Disease related genes, Human disease related genes, Predicted intracellular proteins	
Predicted intracellular proteins	
Essential proteins, Predicted intracellular proteins	
Predicted intracellular proteins	Transport
Predicted intracellular proteins	
Predicted intracellular proteins, Transporters	Protein transport, Transport
Predicted intracellular proteins	
Predicted intracellular proteins	Cell adhesion, mRNA processing, mRNA splicing
Predicted intracellular proteins	
Plasma proteins, Predicted intracellular proteins, Predicted membrane proteins	Endocytosis, Host-virus interaction
Predicted intracellular proteins	Transcription, Transcription regulation
Predicted membrane proteins	Calcium transport, Ion transport, Sodium transport, Transport
Predicted intracellular proteins, Transporters	Autophagy, Protein transport, Transport, Ubl conjugation pathway
Enzymes, Predicted membrane proteins, Transporters	Lipid transport, Transport
Enzymes, Metabolic proteins, Plasma proteins, Predicted membrane proteins, Transporters	Hydrogen ion transport, Ion transport, Potassium transport, Sodium transport, Transport
CD markers, Metabolic proteins, Predicted intracellular proteins, Predicted membrane proteins, Transporters	Ion transport, Potassium transport, Sodium transport, Sodium/potassium transport, Transport
Enzymes, Essential proteins, Predicted intracellular proteins	
Disease related genes, Enzymes, FDA approved drug targets, Metabolic proteins, Plasma proteins, Predicted membrane proteins, Transporters	Calcium transport, Ion transport, Transport
Cancer-related genes, Disease related genes, Enzymes, FDA approved drug targets, Human disease related genes, Metabolic proteins, Plasma proteins, Predicted intracellular proteins, Predicted membrane proteins, T	Calcium transport, Ion transport, Transport
Enzymes, FDA approved drug targets, Metabolic proteins, Predicted intracellular proteins, Predicted membrane proteins, Transporters	Calcium transport, Ion transport, Transport
Disease related genes, Human disease related genes, Metabolic proteins, Plasma proteins, Predicted intracellular proteins, Predicted membrane proteins	Hydrogen ion transport, Ion transport, Transport
Essential proteins, Metabolic proteins, Predicted intracellular proteins	Cilium biogenesis/degradation, Hydrogen ion transport, Ion transport, Transport
Disease related genes, Enzymes, Human disease related genes, Potential drug targets, Predicted membrane proteins	Lipid transport, Transport
Disease related genes, Enzymes, Human disease related genes, Metabolic proteins, Potential drug targets, Predicted intracellular proteins, Predicted membrane proteins, Transporters	Hearing, Lipid transport, Transport
Enzymes, Metabolic proteins, Predicted membrane proteins	Lipid transport, Transport
Disease related genes, Enzymes, FDA approved drug targets, Plasma proteins, Predicted membrane proteins	Differentiation, Host-virus interaction, Immunity, Innate immunity
Cancer-related genes, Disease related genes, Human disease related genes, Plasma proteins, Potential drug targets, Predicted secreted proteins, Transporters	Immunity
Enzymes, Predicted intracellular proteins, Predicted membrane proteins, Transporters	
Plasma proteins, Predicted intracellular proteins	
Predicted intracellular proteins	
Plasma proteins, Predicted intracellular proteins, Transporters	
FDA approved drug targets, G-protein coupled receptors, Predicted membrane proteins, Transporters	
Disease related genes, Human disease related genes, Predicted intracellular proteins	
Plasma proteins, Predicted intracellular proteins	
Disease related genes, Human disease related genes, Predicted intracellular proteins	B-cell activation
Disease related genes, Enzymes, FDA approved drug targets, Human disease related genes, Predicted membrane proteins	
Enzymes, Predicted intracellular proteins	Apoptosis, Cell adhesion, Stress response
Predicted intracellular proteins, Predicted membrane proteins	Cell adhesion
Cancer-related genes, Disease related genes, Enzymes, FDA approved drug targets, Human disease related genes, Predicted intracellular proteins, Transporters	
Cancer-related genes, Disease related genes, Enzymes, FDA approved drug targets, Human disease related genes, Plasma proteins, Predicted intracellular proteins	Adaptive immunity, Apoptosis, Immunity, Innate immunity, Transcription, Transcription regulation
Disease related genes, Human disease related genes, Potential drug targets, Predicted membrane proteins, Transporters	Cell adhesion
Predicted intracellular proteins	
Predicted intracellular proteins	
Cancer-related genes, Disease related genes, Human disease related genes, Plasma proteins, Potential drug targets, Predicted intracellular proteins, Predicted secreted proteins, Transporters	Adaptive immunity, Apoptosis, Complement pathway, DNA damage, Host-virus interaction, Immunity, Innate i
Predicted intracellular proteins, Predicted membrane proteins, Transporters	Lipid transport, Transport
Disease related genes, Human disease related genes, Plasma proteins, Potential drug targets, Predicted secreted proteins, Transporters	Complement alternate pathway, Complement pathway, Cytolysis, Immunity, Innate immunity

Enzymes, FDA approved drug targets, Metabolic proteins, Predicted membrane proteins, Transporters	
Disease related genes, FDA approved drug targets, Human disease related genes, Predicted membrane proteins, Transporters, Voltage-gated ion channels	Calcium transport, Host-virus interaction, Ion transport, Transport
Human disease related genes, Plasma proteins, Predicted intracellular proteins, Predicted membrane proteins	Apoptosis, Cell adhesion, Differentiation, Host-virus interaction, Immunity, Spermatogenesis
Disease related genes, FDA approved drug targets, G-protein coupled receptors, Human disease related genes, Plasma proteins, Predicted intracellular proteins, Predicted membrane proteins, Transporters	
Plasma proteins, Predicted intracellular proteins	
Predicted intracellular proteins	
Predicted intracellular proteins	
Cancer-related genes, Disease related genes, Human disease related genes, Plasma proteins, Predicted intracellular proteins	
Disease related genes, Human disease related genes, Plasma proteins, Predicted intracellular proteins	Transcription, Transcription regulation, Transcription termination
Plasma proteins, Predicted intracellular proteins	
Predicted intracellular proteins	Differentiation, Myogenesis, Transcription, Transcription regulation
Disease related genes, Essential proteins, Human disease related genes, Predicted intracellular proteins	
Predicted intracellular proteins	Cell cycle, Cell division
Predicted intracellular proteins	
Disease related genes, Human disease related genes, Predicted intracellular proteins	Cilium biogenesis/degradation, Neurogenesis
Disease related genes, Human disease related genes, Plasma proteins, Predicted intracellular proteins	Wnt signaling pathway
Predicted intracellular proteins, Transporters	Protein transport, Transport
FDA approved drug targets, G-protein coupled receptors, Predicted membrane proteins	
Cancer-related genes, FDA approved drug targets, G-protein coupled receptors, Predicted intracellular proteins, Predicted membrane proteins	
Predicted intracellular proteins	Differentiation, Neurogenesis, Spermatogenesis
CD markers, G-protein coupled receptors, Human disease related genes, Predicted membrane proteins	Host-virus interaction, Inflammatory response
CD markers, FDA approved drug targets, G-protein coupled receptors, Predicted membrane proteins	
G-protein coupled receptors, Predicted membrane proteins	
CD markers, Plasma proteins, Predicted membrane proteins	
CD markers, Plasma proteins, Predicted intracellular proteins, Predicted membrane proteins, Predicted secreted proteins	Acute phase, Inflammatory response
CD markers, Predicted membrane proteins	Immunity, Inflammatory response, Innate immunity
CD markers, FDA approved drug targets, Predicted intracellular proteins, Predicted membrane proteins	Cell adhesion
Predicted intracellular proteins, Predicted membrane proteins	Host-virus interaction
CD markers, Human disease related genes, Predicted intracellular proteins, Predicted membrane proteins	Cell adhesion
Cancer-related genes, CD markers, Disease related genes, FDA approved drug targets, Predicted membrane proteins, Predicted secreted proteins, Transporters	Adaptive immunity, Immunity
CD markers, Predicted membrane proteins	
Disease related genes, Human disease related genes, Potential drug targets, Predicted intracellular proteins, Transporters	Cell cycle, Cell division, Host-virus interaction, Mitosis
CD markers, FDA approved drug targets, Plasma proteins, Predicted membrane proteins	Cell adhesion
Cancer-related genes, CD markers, Enzymes, Metabolic proteins, Predicted intracellular proteins	
CD markers, Disease related genes, FDA approved drug targets, Human disease related genes, Predicted membrane proteins, Transporters	Adaptive immunity, Immunity
CD markers, Disease related genes, FDA approved drug targets, Human disease related genes, Plasma proteins, Predicted intracellular proteins, Predicted membrane proteins	Adaptive immunity, Immunity
CD markers, Disease related genes, FDA approved drug targets, Human disease related genes, Predicted intracellular proteins, Predicted membrane proteins, Transporters	Adaptive immunity, Host-virus interaction, Immunity
Cancer-related genes, CD markers, Disease related genes, Human disease related genes, Predicted membrane proteins, Predicted secreted proteins	Immunity
Cancer-related genes, Candidate cardiovascular disease genes, CD markers, Disease related genes, Human disease related genes, Predicted intracellular proteins, Predicted membrane proteins, Predicted secreted proteins	
Blood group antigen proteins, Cancer-related genes, CD markers, FDA approved drug targets, Plasma proteins, Predicted intracellular proteins, Predicted membrane proteins, Predicted secreted proteins, Transporters	Cell adhesion
CD markers, Predicted intracellular proteins, Predicted membrane proteins, Transporters	Angiogenesis, Apoptosis, Cell adhesion, Inflammatory response
Cancer-related genes, CD markers, Disease related genes, FDA approved drug targets, Human disease related genes, Predicted membrane proteins	Adaptive immunity, Immunity
CD markers, Predicted membrane proteins	Adaptive immunity, Autophagy, Cell adhesion, Immunity, Innate immunity
CD markers, FDA approved drug targets, Predicted intracellular proteins, Predicted membrane proteins	Adaptive immunity, Host-virus interaction, Immunity
CD markers, Disease related genes, Human disease related genes, Predicted intracellular proteins, Predicted membrane proteins	Adaptive immunity, Immunity
CD markers, Predicted intracellular proteins, Predicted membrane proteins	Adaptive immunity, Immunity
Cancer-related genes, CD markers, Plasma proteins, Predicted membrane proteins, Transporters	Cell adhesion, Fertilization
Disease related genes, Essential proteins, Human disease related genes, Plasma proteins, Predicted intracellular proteins, Predicted membrane proteins	
Plasma proteins, Predicted intracellular proteins	
Predicted intracellular proteins	Cell shape
Predicted intracellular proteins	Cell shape, Phagocytosis
Cancer-related genes, CD markers, Disease related genes, Human disease related genes, Plasma proteins, Potential drug targets, Predicted membrane proteins, Transporters	Cell adhesion
Plasma proteins, Predicted intracellular proteins	Cell adhesion
Disease related genes, Human disease related genes, Predicted membrane proteins	Cell adhesion
Cancer-related genes, Plasma proteins, Predicted intracellular proteins, Predicted membrane proteins	Cell adhesion, Transport
CD markers, Disease related genes, Human disease related genes, Plasma proteins, Predicted intracellular proteins, Predicted membrane proteins	Cell adhesion
Plasma proteins, Predicted intracellular proteins, Predicted membrane proteins	Cell adhesion
Disease related genes, Human disease related genes, Predicted intracellular proteins, Predicted membrane proteins	Cell adhesion, Sensory transduction, Vision
Predicted membrane proteins	Cell adhesion
Cancer-related genes, CD markers, Plasma proteins, Predicted intracellular proteins, Predicted membrane proteins	Cell adhesion
Plasma proteins, Predicted membrane proteins	Cell adhesion
Enzymes, Predicted intracellular proteins	Differentiation, Spermatogenesis
Disease related genes, Enzymes, Human disease related genes, Plasma proteins, Potential drug targets, Predicted intracellular proteins	Apoptosis, Biological rhythms, Cell cycle, Cell division, Neurogenesis
Cancer-related genes, CD markers, Plasma proteins, Predicted intracellular proteins, Predicted membrane proteins	Apoptosis, Cell adhesion
Predicted membrane proteins	
Disease related genes, G-protein coupled receptors, Human disease related genes, Potential drug targets, Predicted intracellular proteins, Predicted membrane proteins, Transporters	
Enzymes, Predicted intracellular proteins, Predicted membrane proteins	Angiogenesis
Cancer-related genes, Enzymes, Predicted intracellular proteins	Apoptosis, Host-virus interaction
Plasma proteins, Predicted intracellular proteins	
Disease related genes, Predicted intracellular proteins	
Disease related genes, Human disease related genes, Predicted intracellular proteins, Predicted membrane proteins	
Disease related genes, FDA approved drug targets, G-protein coupled receptors, Human disease related genes, Predicted membrane proteins	
FDA approved drug targets, G-protein coupled receptors, Predicted membrane proteins	
Disease related genes, Human disease related genes, Predicted membrane proteins, Transporters	Ion transport, Transport
Disease related genes, FDA approved drug targets, Human disease related genes, Predicted intracellular proteins, Predicted membrane proteins	Ion transport, Transport
Disease related genes, FDA approved drug targets, Predicted intracellular proteins, Predicted membrane proteins	Ion transport, Transport
FDA approved drug targets, Predicted intracellular proteins, Predicted membrane proteins	Ion transport, Transport
FDA approved drug targets, Predicted membrane proteins	Ion transport, Transport
Disease related genes, Human disease related genes, Potential drug targets, Predicted intracellular proteins, Predicted membrane proteins, Transporters	Ion transport, Transport
Disease related genes, Human disease related genes, Potential drug targets, Predicted intracellular proteins, Transporters	Angiogenesis, Apoptosis, Cell adhesion, Cell cycle, Cell division, Differentiation, Spermatogenesis
Predicted membrane proteins, Transporters	Cell adhesion, Ion transport, Transport
Disease related genes, Human disease related genes, Plasma proteins, Potential drug targets, Predicted intracellular proteins, Predicted membrane proteins, Transporters	Antiport, Ion transport, Transport
Disease related genes, Human disease related genes, Predicted membrane proteins	
Disease related genes, Human disease related genes, Potential drug targets, Predicted membrane proteins, Transporters	Sensory transduction, Vision
Disease related genes, Predicted membrane proteins	
Predicted membrane proteins	
Cancer-related genes, Disease related genes, Human disease related genes, Potential drug targets, Predicted membrane proteins, Transporters	
Cancer-related genes, Disease related genes, Human disease related genes, Potential drug targets, Predicted membrane proteins, Transporters	Ion transport, Transport
Cancer-related genes, Predicted intracellular proteins, Predicted membrane proteins, Transporters	
Disease related genes, Human disease related genes, Predicted membrane proteins	Host-virus interaction
Predicted intracellular proteins, Predicted membrane proteins	Host-virus interaction, Immunity, Innate immunity
Disease related genes, Human disease related genes, Potential drug targets, Predicted intracellular proteins, Transporters	Ion transport, Transport
Predicted intracellular proteins, Transporters	Ion transport, Transport
Disease related genes, Human disease related genes, Potential drug targets, Predicted intracellular proteins, Transporters	Hearing, Ion transport, Sensory transduction, Transport, Vision
Plasma proteins, Predicted intracellular proteins, Transporters	Cell cycle, Cell division, Mitosis
Plasma proteins, Predicted intracellular proteins, Transporters	
G-protein coupled receptors, Predicted intracellular proteins, Predicted membrane proteins	
Disease related genes, Human disease related genes, Potential drug targets, Predicted intracellular proteins, Predicted membrane proteins, Transporters, Voltage-gated ion channels	Calcium transport, Ion transport, Sensory transduction, Sodium transport, Transport, Vision
Disease related genes, Human disease related genes, Potential drug targets, Predicted membrane proteins, Transporters	Biomineralization, Ion transport, Sensory transduction, Transport, Vision
Disease related genes, FDA approved drug targets, G-protein coupled receptors, Predicted intracellular proteins, Predicted membrane proteins, Transporters	
Essential proteins, Predicted intracellular proteins	Protein transport, Transport
Cancer-related genes, Disease related genes, Human disease related genes, Plasma proteins, Predicted intracellular proteins, Predicted membrane proteins, Predicted secreted proteins	
Disease related genes, Human disease related genes, Plasma proteins, Predicted intracellular proteins, Predicted secreted proteins	Cell adhesion
Plasma proteins, Predicted intracellular proteins	
Enzymes, Predicted intracellular proteins	
Blood group antigen proteins, CD markers, Plasma proteins, Predicted membrane proteins	Complement pathway, Host-virus interaction, Immunity, Innate immunity
Predicted membrane proteins	
G-protein coupled receptors, Predicted membrane proteins, Predicted secreted proteins	
Cancer-related genes, Disease related genes, Human disease related genes, Potential drug targets, Predicted intracellular proteins, Predicted membrane proteins, Transporters	
Cancer-related genes, Candidate cardiovascular disease genes, Disease related genes, Plasma proteins, Predicted intracellular proteins, Predicted membrane proteins, Predicted secreted proteins, RAS pathway relatec	Immunity, Inflammatory response, Innate immunity
Cancer-related genes, CD markers, Disease related genes, Enzymes, FDA approved drug targets, Human disease related genes, Plasma proteins, Predicted intracellular proteins, Predicted membrane proteins, RAS pat	Immunity, Inflammatory response, Innate immunity
Disease related genes, Enzymes, Human disease related genes, Plasma proteins, Potential drug targets, Predicted intracellular proteins	Biological rhythms, Wnt signaling pathway
Disease related genes, Human disease related genes, Plasma proteins, Predicted intracellular proteins	Cell adhesion
Cancer-related genes, Disease related genes, Human disease related genes, Plasma proteins, Predicted intracellular proteins	Cell adhesion, Host-virus interaction, Neurogenesis, Transcription, Transcription regulation, Wnt signaling path
Cancer-related genes, Disease related genes, Human disease related genes, Plasma proteins, Potential drug targets, Predicted intracellular proteins, Transporters	Cell adhesion, Transcription, Transcription regulation, Wnt signaling pathway
Cancer-related genes, Plasma proteins, Predicted intracellular proteins	Endocytosis
Plasma proteins, Predicted membrane proteins, Predicted secreted proteins	Cell adhesion, Chemotaxis, Host-virus interaction, Inflammatory response
Disease related genes, G-protein coupled receptors, Human disease related genes, Plasma proteins, Potential drug targets, Predicted membrane proteins	Adaptive immunity, Host-virus interaction, Immunity, Inflammatory response, Innate immunity
Predicted membrane proteins, Predicted secreted proteins, Transporters	Cell adhesion, Host-virus interaction
Cancer-related genes, CD markers, Disease related genes, G-protein coupled receptors, Human disease related genes, Potential drug targets, Predicted intracellular proteins, Predicted membrane proteins	Chemotaxis
Enzymes, Metabolic proteins, Predicted membrane proteins, Transporters	Electron transport, Transport
Plasma proteins, Predicted intracellular proteins	
Predicted intracellular proteins	
Plasma proteins, Predicted intracellular proteins	Apoptosis, Differentiation, Endocytosis, Protein transport, Transport, Wnt signaling pathway
Disease related genes, Human disease related genes, Plasma proteins, Potential drug targets, Predicted membrane proteins, Transporters	Host-virus interaction
Enzymes, Metabolic proteins, Predicted membrane proteins	Lipid degradation, Lipid metabolism
Plasma proteins, Predicted intracellular proteins	Adaptive immunity, Endocytosis, Immunity, Transport
Disease related genes, Enzymes, Human disease related genes, Metabolic proteins, Potential drug targets, Predicted membrane proteins, Transporters	Melanin biosynthesis
CD markers, Enzymes, Predicted intracellular proteins, Predicted membrane proteins	Lactation, Pregnancy

Cancer-related genes, CD markers, Disease related genes, Enzymes, FDA approved drug targets, Human disease related genes, Predicted intracellular proteins, Predicted membrane proteins	Osteogenesis
Enzymes, Metabolic proteins, Predicted intracellular proteins	Lipid metabolism
Cancer-related genes, Disease related genes, Human disease related genes, Plasma proteins, Predicted intracellular proteins	
Enzymes, Predicted intracellular proteins, Predicted membrane proteins	Neurogenesis
Predicted intracellular proteins, Transporters	Host-virus interaction
Disease related genes, Human disease related genes, Potential drug targets, Predicted intracellular proteins, Transporters	
Predicted intracellular proteins	
Plasma proteins, Predicted intracellular proteins	Stress response
Essential proteins, Predicted intracellular proteins	
Plasma proteins, Predicted intracellular proteins	
Disease related genes, Human disease related genes, Predicted intracellular proteins	
Predicted intracellular proteins	Host-virus interaction
Predicted intracellular proteins	
Enzymes, FDA approved drug targets, Metabolic proteins, Predicted intracellular proteins	Lipid metabolism
FDA approved drug targets, G-protein coupled receptors, Human disease related genes, Predicted membrane proteins	Biological rhythms
Disease related genes, Human disease related genes, Plasma proteins, Predicted membrane proteins	Cell adhesion
Disease related genes, Human disease related genes, Plasma proteins, Predicted membrane proteins	Cell adhesion
Disease related genes, Human disease related genes, Plasma proteins, Predicted intracellular proteins, Predicted membrane proteins	Cell adhesion
Disease related genes, Human disease related genes, Plasma proteins, Potential drug targets, Predicted intracellular proteins, Transporters	
Disease related genes, Human disease related genes, Plasma proteins, Potential drug targets, Predicted intracellular proteins, Predicted membrane proteins, Transporters	
Disease related genes, Human disease related genes, Potential drug targets, Predicted intracellular proteins, Predicted membrane proteins, Transporters	
Predicted membrane proteins	Angiogenesis, Apoptosis, Chemotaxis, Differentiation
Disease related genes, FDA approved drug targets, G-protein coupled receptors, Human disease related genes, Predicted membrane proteins	
Disease related genes, FDA approved drug targets, G-protein coupled receptors, Human disease related genes, Predicted membrane proteins	
Cancer-related genes, Disease related genes, Human disease related genes, Plasma proteins, Predicted membrane proteins	Differentiation, Neurogenesis
Disease related genes, Predicted intracellular proteins	
Predicted intracellular proteins	
Cancer-related genes, Disease related genes, Enzymes, FDA approved drug targets, Human disease related genes, Metabolic proteins, Plasma proteins, Predicted membrane proteins, Predicted secreted proteins, RAS	Host-virus interaction
Disease related genes, Human disease related genes, Plasma proteins, Predicted intracellular proteins	Endocytosis, Protein transport, Transport
Plasma proteins, Predicted intracellular proteins, Transporters	Cilium biogenesis/degradation, Protein transport, Transport
Predicted intracellular proteins	
Plasma proteins, Predicted intracellular proteins	Cilium biogenesis/degradation, Protein transport, Transport
Predicted intracellular proteins, Predicted membrane proteins	Autophagy
Predicted intracellular proteins	
Cancer-related genes, Candidate cardiovascular disease genes, CD markers, Disease related genes, Human disease related genes, Plasma proteins, Predicted membrane proteins	Angiogenesis, Cell adhesion
Cancer-related genes, Enzymes, Metabolic proteins, Plasma proteins, Predicted intracellular proteins	Glycolysis, Plasminogen activation, Transcription, Transcription regulation
Cancer-related genes, Candidate cardiovascular disease genes, Enzymes, Metabolic proteins, Plasma proteins, Predicted intracellular proteins	Glycolysis
Candidate cardiovascular disease genes, Disease related genes, Enzymes, Human disease related genes, Metabolic proteins, Plasma proteins, Potential drug targets, Predicted intracellular proteins	Glycolysis
Predicted intracellular proteins	Biological rhythms, Electron transport, Transport
Predicted intracellular proteins	Biological rhythms, Electron transport, Growth regulation, Transport
Disease related genes, Human disease related genes, Predicted intracellular proteins	
Plasma proteins, Predicted intracellular proteins, Predicted membrane proteins	Cell cycle, Cell division, Mitosis, Transport
Plasma proteins, Predicted intracellular proteins, Predicted membrane proteins	Apoptosis
Predicted intracellular proteins	
Cancer-related genes, CD markers, Disease related genes, FDA approved drug targets, Human disease related genes, Plasma proteins, Predicted intracellular proteins, Predicted membrane proteins	
Cancer-related genes, Disease related genes, Enzymes, FDA approved drug targets, Human disease related genes, Predicted membrane proteins, RAS pathway related proteins	Angiogenesis, Apoptosis, Cell adhesion, Differentiation, Host-virus interaction
Cancer-related genes, Disease related genes, Enzymes, Potential drug targets, Predicted intracellular proteins, Predicted membrane proteins	Cell adhesion
Enzymes, Predicted membrane proteins	Neurogenesis
Enzymes, Predicted intracellular proteins, Predicted membrane proteins	Cell adhesion, Neurogenesis
Cancer-related genes, Disease related genes, Enzymes, Human disease related genes, Plasma proteins, Potential drug targets, Predicted intracellular proteins, Predicted membrane proteins	Neurogenesis
Cancer-related genes, Disease related genes, FDA approved drug targets, Human disease related genes, Predicted membrane proteins	
Cancer-related genes, CD markers, Disease related genes, Enzymes, FDA approved drug targets, Human disease related genes, Plasma proteins, Predicted intracellular proteins, Predicted membrane proteins, Transpo	Transcription, Transcription regulation
Cancer-related genes, Disease related genes, Enzymes, Human disease related genes, Plasma proteins, Potential drug targets, Predicted intracellular proteins, Predicted membrane proteins, Predicted secreted proteins, Transporters	
Predicted intracellular proteins	
Plasma proteins, Predicted membrane proteins, Transporters	Endocytosis, Lipid transport, Transport
Predicted membrane proteins	
Cancer-related genes, Plasma proteins, Predicted intracellular proteins	Cell shape
CD markers, Plasma proteins, Predicted membrane proteins	Host-virus interaction
G-protein coupled receptors, Plasma proteins, Predicted intracellular proteins, Predicted membrane proteins	Blood coagulation, Hemostasis
Cancer-related genes, Candidate cardiovascular disease genes, CD markers, Disease related genes, Human disease related genes, Predicted intracellular proteins, Predicted membrane proteins, Predicted secreted pro	Apoptosis
Cancer-related genes, Enzymes, Metabolic proteins, Plasma proteins, Predicted intracellular proteins	Fatty acid biosynthesis, Fatty acid metabolism, Lipid biosynthesis, Lipid metabolism
Essential proteins, Predicted intracellular proteins	Cell adhesion, Cell shape
Predicted intracellular proteins	Host-virus interaction, Ubl conjugation pathway
CD markers, Predicted intracellular proteins, Predicted membrane proteins	
CD markers, Essential proteins, FDA approved drug targets, Predicted membrane proteins	Immunity, Innate immunity
CD markers, FDA approved drug targets, Human disease related genes, Plasma proteins, Predicted intracellular proteins, Predicted membrane proteins	Immunity
Predicted intracellular proteins	Endocytosis, Protein transport, Transport
Predicted intracellular proteins, Predicted membrane proteins, Predicted secreted proteins	Immunity
FDA approved drug targets, G-protein coupled receptors, Predicted membrane proteins, Transporters	
FDA approved drug targets, G-protein coupled receptors, Human disease related genes, Predicted membrane proteins, Transporters	Differentiation, Inflammatory response
Disease related genes, Human disease related genes, Plasma proteins, Predicted intracellular proteins	
Predicted intracellular proteins	
Disease related genes, Human disease related genes, Plasma proteins, Predicted intracellular proteins	Differentiation
Disease related genes, Human disease related genes, Plasma proteins, Potential drug targets, Predicted intracellular proteins, Transporters	Differentiation, Myogenesis
Plasma proteins, Predicted intracellular proteins, Transporters	
Plasma proteins, Predicted intracellular proteins	Cell adhesion
Disease related genes, Human disease related genes, Potential drug targets, Predicted membrane proteins, Transporters	Cell adhesion
Cancer-related genes, Disease related genes, Enzymes, FDA approved drug targets, Plasma proteins, Predicted membrane proteins, Predicted secreted proteins, RAS pathway related proteins	Angiogenesis, Chemotaxis, Differentiation
Cancer-related genes, Disease related genes, Enzymes, FDA approved drug targets, Human disease related genes, Plasma proteins, Predicted intracellular proteins, Predicted membrane proteins, Predicted secreted p	Angiogenesis
Predicted intracellular proteins	Angiogenesis
Disease related genes, Plasma proteins, Predicted intracellular proteins	Endocytosis
G-protein coupled receptors, Predicted membrane proteins	Chemotaxis
Predicted intracellular proteins	
Enzymes, Plasma proteins, Predicted intracellular proteins	Adaptive immunity, Host-virus interaction, Immunity
Cancer-related genes, G-protein coupled receptors, Plasma proteins, Predicted membrane proteins, Transporters	Wnt signaling pathway
CD markers, Disease related genes, G-protein coupled receptors, Human disease related genes, Potential drug targets, Predicted membrane proteins	Wnt signaling pathway
Disease related genes, G-protein coupled receptors, Human disease related genes, Potential drug targets, Predicted membrane proteins	Wnt signaling pathway
Predicted intracellular proteins, RAS pathway related proteins	
FDA approved drug targets, G-protein coupled receptors, Plasma proteins, Predicted membrane proteins	
Disease related genes, FDA approved drug targets, Human disease related genes, Predicted intracellular proteins, Predicted membrane proteins, Transporters	Ion transport, Transport
Disease related genes, FDA approved drug targets, Human disease related genes, Predicted intracellular proteins, Predicted membrane proteins, Transporters	Ion transport, Transport
FDA approved drug targets, Predicted membrane proteins	Ion transport, Transport
FDA approved drug targets, Predicted intracellular proteins, Predicted membrane proteins	Ion transport, Transport
G-protein coupled receptors, Predicted membrane proteins	
Plasma proteins, Predicted intracellular proteins, Transporters	Differentiation, Growth regulation, Neurogenesis
Enzymes, Essential proteins, FDA approved drug targets, Metabolic proteins, Plasma proteins, Predicted intracellular proteins	Apoptosis, Glycolysis, Immunity, Innate immunity, Translation regulation
Predicted intracellular proteins	B-cell activation
Predicted intracellular proteins	Endocytosis
Disease related genes, Human disease related genes, Plasma proteins, Predicted intracellular proteins	
Predicted membrane proteins	
Predicted membrane proteins	
Disease related genes, FDA approved drug targets, Human disease related genes, Predicted membrane proteins, Predicted secreted proteins	Endocytosis
Predicted intracellular proteins	Wnt signaling pathway
Cancer-related genes, Disease related genes, Human disease related genes, Potential drug targets, Predicted membrane proteins, Transporters	
Disease related genes, Human disease related genes, Potential drug targets, Predicted membrane proteins, Transporters	
Disease related genes, Human disease related genes, Potential drug targets, Predicted membrane proteins, Transporters	
Disease related genes, Human disease related genes, Predicted membrane proteins	Hearing
Predicted intracellular proteins, Predicted membrane proteins, Transporters	
Disease related genes, Human disease related genes, Predicted membrane proteins	
Disease related genes, Human disease related genes, Predicted intracellular proteins, Predicted secreted proteins	Differentiation, Neurogenesis
Cancer-related genes, Enzymes, Metabolic proteins, Plasma proteins, Predicted intracellular proteins	
FDA approved drug targets, G-protein coupled receptors, Predicted membrane proteins, Transporters	
FDA approved drug targets, G-protein coupled receptors, Predicted membrane proteins	
Disease related genes, FDA approved drug targets, Human disease related genes, Predicted membrane proteins, Transporters	Ion transport, Transport
Disease related genes, FDA approved drug targets, Predicted membrane proteins, Transporters	Ion transport, Transport
FDA approved drug targets, Predicted membrane proteins, Transporters	Ion transport, Transport
Human disease related genes, Predicted intracellular proteins	Cell cycle, Cell division
Cancer-related genes, Disease related genes, Human disease related genes, Predicted intracellular proteins	
Cancer-related genes, Disease related genes, Human disease related genes, Potential drug targets, Predicted intracellular proteins, Predicted membrane proteins, Transporters	
Disease related genes, Human disease related genes, Potential drug targets, Predicted intracellular proteins, RAS pathway related proteins, Transporters	
Disease related genes, Human disease related genes, Plasma proteins, Predicted intracellular proteins, Predicted membrane proteins	Blood coagulation, Hemostasis
Disease related genes, Predicted intracellular proteins, Predicted membrane proteins	
Cancer-related genes, Disease related genes, Human disease related genes, Predicted membrane proteins	
Disease related genes, G-protein coupled receptors, Human disease related genes, Potential drug targets, Predicted membrane proteins, Transporters	
G-protein coupled receptors, Predicted membrane proteins	
G-protein coupled receptors, Predicted membrane proteins, Transporters	
G-protein coupled receptors, Predicted intracellular proteins, Predicted membrane proteins, Transporters	
G-protein coupled receptors, Predicted membrane proteins	
G-protein coupled receptors, Predicted membrane proteins, Transporters	Adaptive immunity, Immunity

G-protein coupled receptors, Predicted membrane proteins	
G-protein coupled receptors, Predicted membrane proteins	
G-protein coupled receptors, Plasma proteins, Predicted membrane proteins	
G-protein coupled receptors, Predicted membrane proteins	
G-protein coupled receptors, Predicted intracellular proteins, Predicted membrane proteins	
G-protein coupled receptors, Predicted intracellular proteins, Predicted membrane proteins, Transporters	
Predicted intracellular proteins	
Plasma proteins, Predicted intracellular proteins, Predicted membrane proteins, Transporters	Autophagy, Lipid transport, Transport
Predicted intracellular proteins, Predicted membrane proteins, Transporters	Lipid transport, Transport
Cancer-related genes, Predicted intracellular proteins	
FDA approved drug targets, Predicted membrane proteins	Ion transport, Transport
FDA approved drug targets, Predicted membrane proteins, Transporters	Ion transport, Transport
Disease related genes, FDA approved drug targets, Human disease related genes, Predicted intracellular proteins, Predicted membrane proteins, RAS pathway related proteins	Ion transport, Transport
Enzymes, Predicted intracellular proteins	Apoptosis, Wnt signaling pathway
G-protein coupled receptors, Predicted membrane proteins	
Essential proteins, Predicted intracellular proteins, Transporters	Necrosis
Plasma proteins, Predicted intracellular proteins	
Blood group antigen proteins, CD markers, Predicted membrane proteins, Transporters	Host-virus interaction
Blood group antigen proteins, CD markers, Predicted membrane proteins	
Predicted intracellular proteins	
Enzymes, Metabolic proteins, Predicted intracellular proteins, Predicted membrane proteins, Transporters	
FDA approved drug targets, G-protein coupled receptors, Predicted membrane proteins	Apoptosis
Disease related genes, Enzymes, Potential drug targets, Predicted intracellular proteins	Exocytosis, Host-virus interaction, Immunity, Inflammatory response, Innate immunity, Phagocytosis
Plasma proteins, Predicted intracellular proteins	
FDA approved drug targets, G-protein coupled receptors, Predicted membrane proteins	
Disease related genes, Human disease related genes, Predicted membrane proteins	Ion transport, Iron transport, Transport
Cancer-related genes, Disease related genes, Human disease related genes, Plasma proteins, Potential drug targets, Predicted intracellular proteins, Predicted membrane proteins, Transporters	Adaptive immunity, Host-virus interaction, Immunity, Innate immunity
Human disease related genes, Predicted membrane proteins	Adaptive immunity, Immunity
Plasma proteins, Predicted membrane proteins, Predicted secreted proteins	Adaptive immunity, Host-virus interaction, Immunity, Innate immunity
Enzymes, Essential proteins, Metabolic proteins, Plasma proteins, Predicted intracellular proteins	Cholesterol biosynthesis, Cholesterol metabolism, Lipid biosynthesis, Lipid metabolism, Steroid biosynthesis,
Predicted intracellular proteins	
FDA approved drug targets, G-protein coupled receptors, Predicted intracellular proteins, Predicted membrane proteins	
Cancer-related genes, Plasma proteins, Predicted intracellular proteins, Transporters	Stress response
Cancer-related genes, Disease related genes, Human disease related genes, Plasma proteins, Predicted intracellular proteins	Host-virus interaction, Stress response
FDA approved drug targets, G-protein coupled receptors, Predicted membrane proteins, RAS pathway related proteins	
Disease related genes, Enzymes, Human disease related genes, Plasma proteins, Potential drug targets, Predicted intracellular proteins, Predicted secreted proteins	
Disease related genes, Human disease related genes, Predicted intracellular proteins	
Predicted intracellular proteins	
CD markers, Plasma proteins, Predicted intracellular proteins, Predicted membrane proteins	Adaptive immunity, B-cell activation, Immunity
Predicted intracellular proteins	Antiviral defense, Immunity, Innate immunity
CD markers, Plasma proteins, Predicted membrane proteins, Transporters	Antiviral defense, Immunity, Innate immunity, Osteogenesis
Disease related genes, Human disease related genes, Predicted membrane proteins	Mineral balance
CD markers, Disease related genes, FDA approved drug targets, Human disease related genes, Predicted intracellular proteins, Predicted membrane proteins	
Cancer-related genes, CD markers, Disease related genes, Enzymes, FDA approved drug targets, Human disease related genes, Plasma proteins, Predicted intracellular proteins, Predicted membrane proteins, RAS pat	Host-virus interaction
Predicted intracellular proteins, Predicted membrane proteins	
CD markers, Disease related genes, Human disease related genes, Potential drug targets, Predicted membrane proteins, Transporters	
CD markers, Predicted intracellular proteins, Predicted membrane proteins, Predicted secreted proteins	
Predicted membrane proteins	
Candidate cardiovascular disease genes, Enzymes, Plasma proteins, Predicted intracellular proteins, Predicted membrane proteins, Predicted secreted proteins	
Cancer-related genes, CD markers, Disease related genes, FDA approved drug targets, Human disease related genes, Predicted intracellular proteins, Predicted membrane proteins	Host-virus interaction
Cancer-related genes, CD markers, FDA approved drug targets, Human disease related genes, Plasma proteins, Predicted intracellular proteins, Predicted membrane proteins, Predicted secreted proteins, Transporters	Immunity
Cancer-related genes, Candidate cardiovascular disease genes, CD markers, Disease related genes, Human disease related genes, Plasma proteins, Predicted membrane proteins, Predicted secreted proteins	Host-virus interaction
Cancer-related genes, CD markers, Disease related genes, Human disease related genes, Predicted intracellular proteins, Predicted membrane proteins	
Plasma proteins, Predicted intracellular proteins	Host-virus interaction
Predicted intracellular proteins, Transcription factors	Transcription, Transcription regulation
Cancer-related genes, CD markers, Disease related genes, Human disease related genes, Predicted intracellular proteins, Predicted membrane proteins	Cell adhesion
CD markers, FDA approved drug targets, Predicted intracellular proteins, Predicted membrane proteins	Cell adhesion
Cancer-related genes, CD markers, FDA approved drug targets, Plasma proteins, Predicted intracellular proteins, Predicted membrane proteins, Transporters	Cell adhesion, Host-virus interaction, Myogenesis
CD markers, Disease related genes, Human disease related genes, Plasma proteins, Predicted intracellular proteins, Predicted membrane proteins	Cell adhesion, Phagocytosis
Cancer-related genes, CD markers, Disease related genes, FDA approved drug targets, Human disease related genes, Plasma proteins, Predicted intracellular proteins, Predicted membrane proteins	Cell adhesion, Host-virus interaction
Cancer-related genes, CD markers, Disease related genes, Human disease related genes, Plasma proteins, Predicted intracellular proteins, Predicted membrane proteins	Cell adhesion
Plasma proteins, Predicted intracellular proteins, Predicted membrane proteins	Endocytosis, Exocytosis, Host-virus interaction, Protein transport, Transport
Disease related genes, Enzymes, Human disease related genes, Potential drug targets, Predicted membrane proteins	
CD markers, Disease related genes, Human disease related genes, Predicted membrane proteins	
Predicted intracellular proteins, Predicted membrane proteins	Cell adhesion, Immunity
Cancer-related genes, Disease related genes, Human disease related genes, Plasma proteins, Predicted intracellular proteins	Cell adhesion
Disease related genes, Human disease related genes, Plasma proteins, Predicted intracellular proteins	Transcription, Transcription regulation
Essential proteins, Predicted intracellular proteins	
Disease related genes, Enzymes, Essential proteins, Human disease related genes, Metabolic proteins, Potential drug targets, Predicted intracellular proteins	Host-virus interaction, Protein biosynthesis
FDA approved drug targets, Predicted membrane proteins, Transporters, Voltage-gated ion channels	Ion transport, Potassium transport, Transport
Predicted intracellular proteins, Transporters	Ion transport, Potassium transport, Transport
Disease related genes, Human disease related genes, Potential drug targets, Predicted intracellular proteins, Predicted membrane proteins, Transporters	Ion transport, Potassium transport, Transport
Predicted membrane proteins, Transporters, Voltage-gated ion channels	Ion transport, Potassium transport, Transport
Predicted membrane proteins, Voltage-gated ion channels	Ion transport, Potassium transport, Transport
Predicted membrane proteins, Transporters, Voltage-gated ion channels	Ion transport, Potassium transport, Transport
Disease related genes, FDA approved drug targets, Human disease related genes, Predicted intracellular proteins, Predicted membrane proteins, Transporters, Voltage-gated ion channels	Immunity, Ion transport, Transport
Disease related genes, FDA approved drug targets, Human disease related genes, Plasma proteins, Predicted intracellular proteins, Predicted membrane proteins, Transporters, Voltage-gated ion channels	Ion transport, Potassium transport, Transport
Predicted membrane proteins, Transporters, Voltage-gated ion channels	Ion transport, Potassium transport, Transport
Predicted intracellular proteins, Predicted membrane proteins, Transporters, Voltage-gated ion channels	Ion transport, Potassium transport, Transport
Disease related genes, Human disease related genes, Potential drug targets, Predicted membrane proteins, Transporters, Voltage-gated ion channels	Ion transport, Potassium transport, Transport
Predicted membrane proteins, Voltage-gated ion channels	Ion transport, Potassium transport, Transport
Blood group antigen proteins, Cancer-related genes, CD markers, Enzymes, Predicted intracellular proteins, Predicted membrane proteins	
Predicted intracellular proteins	
Disease related genes, G-protein coupled receptors, Human disease related genes, Potential drug targets, Predicted membrane proteins, Transporters	
Cancer-related genes, CD markers, Disease related genes, Enzymes, FDA approved drug targets, Human disease related genes, Plasma proteins, Predicted intracellular proteins, Predicted membrane proteins, RAS pathway related proteins	
Disease related genes, Essential proteins, Human disease related genes, Predicted intracellular proteins	
Cancer-related genes, Enzymes, Predicted intracellular proteins, Predicted secreted proteins	
Cancer-related genes, CD markers, Predicted membrane proteins, Transporters	Adaptive immunity, Differentiation, Immunity, Innate immunity
CD markers, Disease related genes, Human disease related genes, Plasma proteins, Predicted intracellular proteins, Predicted membrane proteins	Cell adhesion, Differentiation, Neurogenesis
CD markers, Predicted intracellular proteins, Predicted membrane proteins	Adaptive immunity, Immunity
CD markers, Disease related genes, Human disease related genes, Plasma proteins, Potential drug targets, Predicted membrane proteins, Transporters	Autophagy
Predicted intracellular proteins	
Disease related genes, Human disease related genes, Plasma proteins, Predicted intracellular proteins, Predicted membrane proteins, RAS pathway related proteins	Adaptive immunity, Immunity, Mast cell degranulation
Disease related genes, Human disease related genes, Predicted intracellular proteins	Adaptive immunity, Immunity, Mast cell degranulation
Predicted membrane proteins	Adaptive immunity, Immunity
Cancer-related genes, Disease related genes, Plasma proteins, Predicted intracellular proteins	
Cancer-related genes, Candidate cardiovascular disease genes, CD markers, Disease related genes, FDA approved drug targets, Human disease related genes, Plasma proteins, Predicted membrane proteins	
Disease related genes, FDA approved drug targets, G-protein coupled receptors, Human disease related genes, Predicted intracellular proteins, Predicted membrane proteins	
Disease related genes, Human disease related genes, Potential drug targets, Predicted intracellular proteins, Predicted membrane proteins, Transporters	
Predicted secreted proteins	Lipid degradation, Lipid metabolism
Disease related genes, Human disease related genes, Predicted intracellular proteins, Predicted membrane proteins	Transcription, Transcription regulation
Enzymes, Plasma proteins, Predicted intracellular proteins	Ubl conjugation pathway
G-protein coupled receptors, Predicted membrane proteins	
G-protein coupled receptors, Predicted intracellular proteins, Predicted membrane proteins, Transporters	
Disease related genes, G-protein coupled receptors, Human disease related genes, Potential drug targets, Predicted membrane proteins	
Cancer-related genes, Disease related genes, Human disease related genes, Plasma proteins, Predicted intracellular proteins	Cell adhesion
Predicted membrane proteins	
Candidate cardiovascular disease genes, CD markers, Disease related genes, Human disease related genes, Plasma proteins, Potential drug targets, Predicted intracellular proteins, Predicted membrane proteins, Trans	Endocytosis, Host-virus interaction
Predicted membrane proteins, Transporters	Ion transport, Transport
Disease related genes, Predicted intracellular proteins, Predicted membrane proteins	
Essential proteins, Predicted intracellular proteins, Predicted membrane proteins	Cell shape, Immunity
G-protein coupled receptors, Predicted membrane proteins	
Cancer-related genes, Disease related genes, Enzymes, Potential drug targets, Predicted intracellular proteins	Adaptive immunity, Host-virus interaction, Immunity, Inflammatory response, Innate immunity
Disease related genes, Human disease related genes, Potential drug targets, Predicted intracellular proteins, Predicted membrane proteins, Transporters	Wnt signaling pathway
Disease related genes, Human disease related genes, Predicted intracellular proteins, Predicted membrane proteins	Apoptosis
Cancer-related genes, Plasma proteins, Predicted intracellular proteins	
Predicted intracellular proteins	Host-virus interaction
Cancer-related genes, Disease related genes, Enzymes, FDA approved drug targets, Human disease related genes, Predicted intracellular proteins, RAS pathway related proteins	
Disease related genes, Enzymes, Human disease related genes, Potential drug targets, Predicted intracellular proteins	Apoptosis, Host-virus interaction, Stress response, Transcription, Transcription regulation
Cancer-related genes, Disease related genes, Plasma proteins, Predicted intracellular proteins	
Candidate cardiovascular disease genes, Disease related genes, FDA approved drug targets, Human disease related genes, Plasma proteins, Predicted intracellular proteins, Predicted secreted proteins	
Plasma proteins, Predicted intracellular proteins	
Enzymes, Predicted intracellular proteins	Differentiation, Wnt signaling pathway
Disease related genes, Human disease related genes, Potential drug targets, Predicted intracellular proteins, Predicted membrane proteins, Transporters	Hearing
G-protein coupled receptors, Predicted membrane proteins	
G-protein coupled receptors, Predicted membrane proteins	
CD markers, Plasma proteins, Predicted membrane proteins	Cell adhesion
Predicted membrane proteins	
Disease related genes, Plasma proteins, Predicted intracellular proteins	

G-protein coupled receptors, Predicted membrane proteins	
Disease related genes, Human disease related genes, Potential drug targets, Predicted membrane proteins, Transporters, Voltage-gated ion channels	Adaptive immunity, Calcium transport, Immunity, Ion transport, Transport
Predicted intracellular proteins	Differentiation, Neurogenesis
Disease related genes, Human disease related genes, Predicted intracellular proteins	Immunity, Inflammatory response, Innate immunity
Disease related genes, Human disease related genes, Predicted intracellular proteins, Predicted membrane proteins	Cell adhesion, Myogenesis, Phagocytosis
Disease related genes, Enzymes, Human disease related genes, Plasma proteins, Potential drug targets, Predicted intracellular proteins, Predicted membrane proteins	
Predicted intracellular proteins, Predicted membrane proteins	
Disease related genes, Human disease related genes, Potential drug targets, Predicted membrane proteins, Transporters	Lipid transport, Symport, Transport
Plasma proteins, Predicted intracellular proteins	Endocytosis, Protein transport, Transport
Predicted intracellular proteins	Apoptosis
Predicted intracellular proteins	Cell cycle, Cell division, Mitosis
Enzymes, Predicted intracellular proteins, Transporters	Necrosis
G-protein coupled receptors, Predicted membrane proteins	
Cancer-related genes, Candidate cardiovascular disease genes, CD markers, Disease related genes, Enzymes, FDA approved drug targets, Human disease related genes, Plasma proteins, Predicted intracellular proteins, Predicted membrane proteins	
Enzymes, Predicted secreted proteins	
Cancer-related genes, CD markers, Disease related genes, FDA approved drug targets, Human disease related genes, Predicted intracellular proteins, Predicted membrane proteins	
Predicted intracellular proteins	
Predicted intracellular proteins	
Predicted intracellular proteins, Predicted membrane proteins	
FDA approved drug targets, Predicted intracellular proteins, Predicted membrane proteins	Immunity, Innate immunity
G-protein coupled receptors, Predicted intracellular proteins, Predicted membrane proteins, Transporters	
CD markers, Disease related genes, FDA approved drug targets, Human disease related genes, Predicted intracellular proteins, Predicted membrane proteins, Transporters	B-cell activation
Cancer-related genes, Disease related genes, Plasma proteins, Predicted intracellular proteins, Predicted membrane proteins	Cell adhesion
Cancer-related genes, Disease related genes, Human disease related genes, Plasma proteins, Predicted intracellular proteins	Host-virus interaction
Disease related genes, Enzymes, Human disease related genes, Metabolic proteins, Plasma proteins, Potential drug targets, Predicted intracellular proteins	Lipid metabolism, Protein transport, Transport
Cancer-related genes, CD markers, Disease related genes, Human disease related genes, Predicted intracellular proteins, Predicted membrane proteins, Predicted secreted proteins	
Cancer-related genes, Candidate cardiovascular disease genes, Disease related genes, Human disease related genes, Plasma proteins, Predicted intracellular proteins	
Disease related genes, Enzymes, Human disease related genes, Metabolic proteins, Plasma proteins, Potential drug targets, Predicted intracellular proteins, Predicted membrane proteins	
Plasma proteins, Predicted intracellular proteins	Transport
Predicted intracellular proteins	
Predicted intracellular proteins, Transporters	
Predicted intracellular proteins	Adaptive immunity, Immunity
Disease related genes, Human disease related genes, Plasma proteins, Potential drug targets, Predicted intracellular proteins, Predicted membrane proteins, Transporters	
Disease related genes, Human disease related genes, Metabolic proteins, Potential drug targets, Predicted intracellular proteins, Predicted membrane proteins, Transporters	Ion transport, Sodium transport, Transport
Predicted intracellular proteins	
Essential proteins, Predicted intracellular proteins	ER-Golgi transport, Protein transport, Transport
Cancer-related genes, CD markers, Plasma proteins, Predicted intracellular proteins, Predicted membrane proteins, Predicted secreted proteins, Transporters	Cell adhesion, Host-virus interaction
Plasma proteins, Predicted membrane proteins	Cell adhesion
Disease related genes, Human disease related genes, Plasma proteins, Predicted membrane proteins	
Predicted intracellular proteins	
Disease related genes, Human disease related genes, Predicted membrane proteins, Predicted secreted proteins	Cell adhesion, Host-virus interaction
Cancer-related genes, Plasma proteins, Predicted membrane proteins	Cell adhesion
Disease related genes, Enzymes, FDA approved drug targets, Human disease related genes, Metabolic proteins, Predicted intracellular proteins	Carbohydrate metabolism, Lipid degradation, Lipid metabolism
Cancer-related genes, Disease related genes, Human disease related genes, Plasma proteins, Predicted intracellular proteins, Predicted membrane proteins, RAS pathway related proteins	
Cancer-related genes, Disease related genes, Human disease related genes, Plasma proteins, Predicted intracellular proteins	
Cancer-related genes, CD markers, Predicted intracellular proteins, Predicted membrane proteins, RAS pathway related proteins, Transporters	Apoptosis, Biological rhythms, Differentiation, Neurogenesis
Disease related genes, Human disease related genes, Plasma proteins, Predicted intracellular proteins	
Plasma proteins, Predicted intracellular proteins	
Plasma proteins, Predicted intracellular proteins, Predicted secreted proteins	Cell adhesion
Enzymes, Metabolic proteins, Predicted intracellular proteins	
Disease related genes, Human disease related genes, Potential drug targets, Predicted intracellular proteins, Predicted membrane proteins, Transporters	Cell adhesion
G-protein coupled receptors, Plasma proteins, Predicted membrane proteins	
Enzymes, Metabolic proteins, Predicted intracellular proteins	
G-protein coupled receptors, Predicted membrane proteins	
Cancer-related genes, Enzymes, FDA approved drug targets, Metabolic proteins, Plasma proteins, Predicted intracellular proteins, Transporters	
Cancer-related genes, Disease related genes, Human disease related genes, Plasma proteins, Predicted intracellular proteins, Predicted membrane proteins	Differentiation, Notch signaling pathway, Transcription, Transcription regulation
Cancer-related genes, Disease related genes, Human disease related genes, Plasma proteins, Predicted intracellular proteins, Predicted membrane proteins	Differentiation, Notch signaling pathway, Transcription, Transcription regulation
G-protein coupled receptors, Predicted membrane proteins	
Cancer-related genes, Disease related genes, Enzymes, Human disease related genes, Potential drug targets, Predicted intracellular proteins, RAS pathway related proteins	
CD markers, Disease related genes, Enzymes, Human disease related genes, Metabolic proteins, Potential drug targets, Predicted intracellular proteins, Predicted membrane proteins	
Predicted intracellular proteins	Differentiation, Neurogenesis
Disease related genes, Human disease related genes, Predicted intracellular proteins	Differentiation, Neurogenesis
Cancer-related genes, Disease related genes, Enzymes, FDA approved drug targets, Human disease related genes, Plasma proteins, Predicted intracellular proteins, Predicted membrane proteins, RAS pathway related	Differentiation, Neurogenesis
G-protein coupled receptors, Predicted membrane proteins, Transporters	
Cancer-related genes, Predicted intracellular proteins	Neurogenesis
Disease related genes, Human disease related genes, Potential drug targets, Predicted intracellular proteins, Predicted membrane proteins, Transporters	
Disease related genes, Enzymes, Essential proteins, Human disease related genes, Metabolic proteins, Potential drug targets, Predicted intracellular proteins	Apoptosis, Biological rhythms, Host-virus interaction, Ubl conjugation pathway
Predicted secreted proteins	Cell adhesion
Disease related genes, Human disease related genes, Predicted intracellular proteins, Predicted membrane proteins	Cell adhesion, Immunity, Inflammatory response
G-protein coupled receptors, Predicted membrane proteins	Sensory transduction
FDA approved drug targets, G-protein coupled receptors, Predicted membrane proteins, Transporters	Behavior
G-protein coupled receptors, Predicted membrane proteins	Olfaction, Sensory transduction
G-protein coupled receptors, Predicted membrane proteins	Olfaction, Sensory transduction
G-protein coupled receptors, Predicted membrane proteins	Olfaction, Sensory transduction
G-protein coupled receptors, Predicted membrane proteins	Olfaction, Sensory transduction
Essential proteins, G-protein coupled receptors, Predicted membrane proteins	Olfaction, Sensory transduction
Plasma proteins, Predicted intracellular proteins	Lipid transport, Transport
Predicted intracellular proteins	Lipid transport, Transport
G-protein coupled receptors, Predicted membrane proteins	Immunity, Innate immunity
Predicted intracellular proteins	Endocytosis
Predicted intracellular proteins	Adaptive immunity, Immunity
Predicted intracellular proteins	Host-virus interaction, Translation regulation
Disease related genes, Enzymes, Human disease related genes, Plasma proteins, Potential drug targets, Predicted intracellular proteins, RAS pathway related proteins	Apoptosis, Exocytosis
Enzymes, Predicted intracellular proteins, RAS pathway related proteins	Apoptosis, Cell cycle
Predicted intracellular proteins	Cell shape
Predicted intracellular proteins	
Plasma proteins, Predicted intracellular proteins	Host-virus interaction
Disease related genes, Human disease related genes, Potential drug targets, Predicted membrane proteins, Transporters	Calcium transport, Differentiation, Ion transport, Oogenesis, Transport
Predicted membrane proteins, Transporters	Ion transport, Transport
Predicted membrane proteins, Transporters	Differentiation, Oogenesis
Disease related genes, Plasma proteins, Predicted intracellular proteins	Cell cycle, Cell division, Differentiation
Plasma proteins, Predicted intracellular proteins	Cell cycle, Cell division
Plasma proteins, Predicted intracellular proteins	Cell cycle, Cell division
Cancer-related genes, Plasma proteins, Predicted intracellular proteins, Predicted membrane proteins	Cell adhesion
Predicted intracellular proteins	
Plasma proteins, Predicted intracellular proteins, Predicted membrane proteins	Cell adhesion
Plasma proteins, Predicted intracellular proteins, Predicted membrane proteins	Cell adhesion
Predicted membrane proteins	Cell adhesion
Predicted membrane proteins	Cell adhesion
Cancer-related genes, Essential proteins, Plasma proteins, Predicted intracellular proteins	
Enzymes, FDA approved drug targets, Metabolic proteins, Predicted intracellular proteins	
Disease related genes, Enzymes, FDA approved drug targets, Human disease related genes, Metabolic proteins, Predicted intracellular proteins	
Enzymes, Metabolic proteins, Predicted intracellular proteins	
Predicted secreted proteins, RAS pathway related proteins	
Cancer-related genes, CD markers, Disease related genes, Enzymes, FDA approved drug targets, Human disease related genes, Plasma proteins, Predicted intracellular proteins, Predicted membrane proteins, RAS pat	Chemotaxis, Host-virus interaction
Predicted membrane proteins, Transporters	Cell shape
Predicted intracellular proteins	Cell adhesion
Cancer-related genes, Candidate cardiovascular disease genes, CD markers, Plasma proteins, Predicted intracellular proteins, Predicted membrane proteins	Cell adhesion, Phagocytosis
Disease related genes, Enzymes, Human disease related genes, Plasma proteins, Potential drug targets, Predicted intracellular proteins, Predicted membrane proteins	Biomineralization
Predicted intracellular proteins, Predicted membrane proteins	
Disease related genes, Human disease related genes, Metabolic proteins, Predicted intracellular proteins, Predicted membrane proteins	GPI-anchor biosynthesis
Cancer-related genes, Disease related genes, Enzymes, FDA approved drug targets, Metabolic proteins, Predicted intracellular proteins	Angiogenesis, Chemotaxis, Endocytosis, Immunity, Inflammatory response, Lipid metabolism
Cancer-related genes, Enzymes, Essential proteins, Metabolic proteins, Predicted intracellular proteins	Lipid metabolism
Disease related genes, Human disease related genes, Plasma proteins, Potential drug targets, Predicted membrane proteins, Predicted secreted proteins, Transporters, Voltage-gated ion channels	Calcium transport, Ion transport, Potassium transport, Transport
Disease related genes, Human disease related genes, Plasma proteins, Predicted intracellular proteins	Cell adhesion
Plasma proteins, Predicted intracellular proteins	Cell adhesion
Plasma proteins, Predicted intracellular proteins	Cell adhesion
Disease related genes, Enzymes, Human disease related genes, Metabolic proteins, Potential drug targets, Predicted intracellular proteins, Predicted membrane proteins, RAS pathway related proteins	Chemotaxis, Lipid metabolism, Phospholipid metabolism
Disease related genes, Human disease related genes, Plasma proteins, Predicted intracellular proteins	Neurogenesis
Cancer-related genes, CD markers, FDA approved drug targets, Predicted intracellular proteins, Predicted membrane proteins	
Disease related genes, Enzymes, Human disease related genes, Metabolic proteins, Potential drug targets, Predicted intracellular proteins	Lipid degradation, Lipid metabolism
Cancer-related genes, Enzymes, Metabolic proteins, Plasma proteins, Predicted intracellular proteins, RAS pathway related proteins	Host-virus interaction, Lipid degradation, Lipid metabolism
Predicted intracellular proteins	
Predicted intracellular proteins	
Predicted intracellular proteins	
Metabolic proteins, Plasma proteins, Predicted intracellular proteins	
Enzymes, Metabolic proteins, Predicted membrane proteins	Lipid metabolism
Enzymes, Metabolic proteins, Predicted membrane proteins	Lipid metabolism
Predicted intracellular proteins, Transporters	Antiviral defense, Host-virus interaction, Lipid transport, Transport

Plasma proteins, Predicted membrane proteins, Transporters	Cell adhesion
Cancer-related genes, Disease related genes, Enzymes, Essential proteins, FDA approved drug targets, Human disease related genes, Metabolic proteins, Plasma proteins, Predicted intracellular proteins	DNA damage, DNA repair, DNA replication
Cancer-related genes, Plasma proteins, Predicted intracellular proteins	
Enzymes, Metabolic proteins, Predicted intracellular proteins	
Predicted intracellular proteins	
FDA approved drug targets, Predicted intracellular proteins	
Predicted intracellular proteins	
Plasma proteins, Predicted intracellular proteins	Apoptosis, Differentiation, Growth regulation, Transcription, Transcription regulation, Ubl conjugation pathway
Cancer-related genes, Enzymes, FDA approved drug targets, Plasma proteins, Predicted intracellular proteins, RAS pathway related proteins	Angiogenesis, Apoptosis, Cell adhesion
Cancer-related genes, Enzymes, FDA approved drug targets, Predicted intracellular proteins	Cell adhesion, Cell cycle, Cell division, Immunity
Disease related genes, Enzymes, Human disease related genes, Metabolic proteins, Plasma proteins, Potential drug targets, Predicted intracellular proteins	Angiogenesis, Apoptosis, Differentiation, Immunity, Inflammatory response, Innate immunity, Neurogenesis
Enzymes, Predicted intracellular proteins	
Predicted intracellular proteins, Transporters	Adaptive immunity, Immunity
Disease related genes, Human disease related genes, Potential drug targets, Predicted intracellular proteins, Predicted membrane proteins, Transporters	
Cancer-related genes, Predicted intracellular proteins, Predicted membrane proteins	
Enzymes, Predicted membrane proteins	
Essential proteins, Plasma proteins, Predicted intracellular proteins	
Disease related genes, Human disease related genes, Predicted intracellular proteins	Cell adhesion, Endocytosis, Immunity, Inflammatory response, Innate immunity
FDA approved drug targets, G-protein coupled receptors, Predicted membrane proteins, Transporters	Chemotaxis
Cancer-related genes, Disease related genes, Human disease related genes, Potential drug targets, Predicted intracellular proteins, Predicted membrane proteins, Transporters	
Disease related genes, FDA approved drug targets, G-protein coupled receptors, Human disease related genes, Predicted intracellular proteins, Predicted membrane proteins, Transporters	
Disease related genes, Enzymes, Plasma proteins, Potential drug targets, Predicted intracellular proteins	Adaptive immunity, Angiogenesis, Immunity
Enzymes, Plasma proteins, Predicted intracellular proteins, Predicted membrane proteins, Predicted secreted proteins	Cell adhesion, Wnt signaling pathway
Enzymes, Predicted membrane proteins	Apoptosis
CD markers, Enzymes, Plasma proteins, Predicted membrane proteins, Predicted secreted proteins	Angiogenesis
Cancer-related genes, Enzymes, Plasma proteins, Predicted intracellular proteins, Predicted membrane proteins	
Enzymes, Plasma proteins, Predicted intracellular proteins, Predicted membrane proteins	Cell adhesion
Enzymes, Plasma proteins, Predicted intracellular proteins, Predicted membrane proteins, Predicted secreted proteins	
Enzymes, Plasma proteins, Predicted membrane proteins, Predicted secreted proteins	Cell adhesion
CD markers, Plasma proteins, Predicted intracellular proteins, Predicted membrane proteins, Predicted secreted proteins	Cell adhesion, Host-virus interaction
Enzymes, Predicted intracellular proteins	
Predicted intracellular proteins	Protein transport, Transport
Disease related genes, Human disease related genes, Predicted intracellular proteins	Protein transport, Transport
Cancer-related genes, Predicted intracellular proteins	Protein transport, Transport
Enzymes, Predicted intracellular proteins	Autophagy, Cilium biogenesis/degradation, Protein transport, Transport
Predicted membrane proteins, Predicted secreted proteins	Host-virus interaction, Immunity
Predicted membrane proteins	Host-virus interaction, Immunity
Cancer-related genes, Disease related genes, Enzymes, FDA approved drug targets, Human disease related genes, Predicted intracellular proteins, RAS pathway related proteins	
Disease related genes, Enzymes, Human disease related genes, Potential drug targets, Predicted intracellular proteins, RAS pathway related proteins	Cell cycle, Cell division, Exocytosis
Enzymes, Metabolic proteins, Predicted intracellular proteins, RAS pathway related proteins, Transporters	Transport
FDA approved drug targets, Predicted membrane proteins, Transporters	Transport
Disease related genes, Human disease related genes, Predicted intracellular proteins	Differentiation, Neurogenesis
Predicted intracellular proteins	
Predicted intracellular proteins, RAS pathway related proteins	
Disease related genes, Human disease related genes, Predicted intracellular proteins, RAS pathway related proteins	Differentiation
Disease related genes, Human disease related genes, Plasma proteins, Predicted intracellular proteins, RAS pathway related proteins	
Predicted intracellular proteins, RAS pathway related proteins	Differentiation
Disease related genes, Enzymes, Human disease related genes, Potential drug targets, Predicted intracellular proteins	
Predicted intracellular proteins	
Disease related genes, Human disease related genes, Predicted intracellular proteins	
Plasma proteins, Predicted membrane proteins	Wnt signaling pathway
Predicted membrane proteins	
Disease related genes, Human disease related genes, Predicted intracellular proteins	
Cancer-related genes, Disease related genes, Enzymes, FDA approved drug targets, Human disease related genes, Plasma proteins, Predicted intracellular proteins, Predicted membrane proteins, Transporters	Cell adhesion
Predicted intracellular proteins	Autophagy
Plasma proteins, Predicted intracellular proteins	
Predicted membrane proteins	Differentiation, Erythrocyte maturation
Cancer-related genes, Disease related genes, Enzymes, Human disease related genes, Potential drug targets, Predicted intracellular proteins, RAS pathway related proteins	Cell cycle, Cell division, Host-virus interaction
Disease related genes, Enzymes, Human disease related genes, Potential drug targets, Predicted intracellular proteins	Antiviral defense, Host-virus interaction, Immunity, Innate immunity
Predicted intracellular proteins	
Disease related genes, Enzymes, Metabolic proteins, Potential drug targets, Predicted intracellular proteins	Ubl conjugation pathway, Wnt signaling pathway
Predicted intracellular proteins, Predicted membrane proteins	Chemotaxis, Differentiation, Neurogenesis
Disease related genes, Human disease related genes, Predicted intracellular proteins	Protein transport, Transport
Disease related genes, Essential proteins, Human disease related genes, Plasma proteins, Predicted intracellular proteins, Ribosomal proteins	Host-virus interaction
Essential proteins, Predicted intracellular proteins, Predicted membrane proteins	
Predicted intracellular proteins	Transcription, Transcription regulation
Disease related genes, Human disease related genes, Predicted membrane proteins	
Predicted intracellular proteins	
Predicted intracellular proteins	
Cancer-related genes, Plasma proteins, Predicted intracellular proteins	
Cancer-related genes, Plasma proteins, Predicted intracellular proteins, Predicted secreted proteins	Apoptosis, Autophagy, Chemotaxis, Immunity, Inflammatory response, Innate immunity
Plasma proteins, Predicted intracellular proteins	
Disease related genes, FDA approved drug targets, Human disease related genes, Metabolic proteins, Predicted intracellular proteins, Predicted membrane proteins, Transporters, Voltage-gated ion channels	Ion transport, Sodium transport, Transport
Disease related genes, FDA approved drug targets, Human disease related genes, Predicted membrane proteins	Ion transport, Sodium transport, Transport
Disease related genes, FDA approved drug targets, Human disease related genes, Metabolic proteins, Plasma proteins, Predicted intracellular proteins, Predicted membrane proteins, Transporters, Voltage-gated ion ch	Ion transport, Sodium transport, Transport
Disease related genes, FDA approved drug targets, Human disease related genes, Metabolic proteins, Plasma proteins, Predicted intracellular proteins, Predicted membrane proteins, Transporters, Voltage-gated ion ch	Ion transport, Sodium transport, Transport
FDA approved drug targets, Metabolic proteins, Predicted intracellular proteins, Predicted membrane proteins, Transporters	Ion transport, Sodium transport, Transport
Disease related genes, FDA approved drug targets, Human disease related genes, Metabolic proteins, Predicted membrane proteins, Transporters	Ion transport, Sodium transport, Transport
Disease related genes, Predicted intracellular proteins	Differentiation
Disease related genes, Human disease related genes, Predicted secreted proteins	
CD markers, Predicted membrane proteins	Differentiation, Neurogenesis
Essential proteins, Predicted intracellular proteins	Cell cycle, Cell division, DNA replication, Mitosis, mRNA processing, mRNA splicing, Ribosome biogenesis
CD markers, Plasma proteins, Predicted intracellular proteins, Predicted membrane proteins	Differentiation, Neurogenesis
Predicted membrane proteins, Transporters	Antiviral defense, Host-virus interaction, Immunity, Innate immunity, Lipid biosynthesis, Lipid metabolism, Pho
Plasma proteins, Predicted intracellular proteins	Differentiation, Neurogenesis
Predicted intracellular proteins, Predicted membrane proteins	Adaptive immunity, Cell adhesion, Immunity, Innate immunity
Plasma proteins, Predicted membrane proteins	Cell adhesion
Predicted intracellular proteins	
Disease related genes, Human disease related genes, Predicted intracellular proteins	
Disease related genes, Metabolic proteins, Potential drug targets, Predicted membrane proteins, Transporters	Ion transport, Lipid transport, Sodium transport, Symport, Transport
Disease related genes, Human disease related genes, Metabolic proteins, Potential drug targets, Predicted membrane proteins, Transporters	Ion transport, Sodium transport, Symport, Transport
Blood group antigen proteins, Metabolic proteins, Predicted intracellular proteins, Predicted membrane proteins, Transporters	Transport
Disease related genes, Human disease related genes, Metabolic proteins, Potential drug targets, Predicted membrane proteins, Transporters	Symport, Transport
Metabolic proteins, Predicted membrane proteins, Transporters	Transport
Metabolic proteins, Predicted membrane proteins, Transporters	Symport, Transport
Metabolic proteins, Predicted membrane proteins, Transporters	Symport, Transport
Disease related genes, Human disease related genes, Metabolic proteins, Potential drug targets, Predicted membrane proteins, Transporters	Amino-acid transport, Symport, Transport
FDA approved drug targets, Metabolic proteins, Plasma proteins, Predicted intracellular proteins, Predicted membrane proteins, Transporters	Amino-acid transport, Antiport, Host-virus interaction, Symport, Transport
Metabolic proteins, Predicted intracellular proteins, Predicted membrane proteins, Transporters	Amino-acid transport, Symport, Transport
Metabolic proteins, Predicted membrane proteins, Transporters	Host-virus interaction, Phosphate transport, Symport, Transport
Metabolic proteins, Predicted membrane proteins, Transporters	Antiport, Calcium transport, Ion transport, Potassium transport, Sodium transport, Symport, Transport
Metabolic proteins, Plasma proteins, Predicted membrane proteins, Transporters	Anion exchange, Antiport, Ion transport, Transport
Disease related genes, Metabolic proteins, Potential drug targets, Predicted membrane proteins, Transporters	Symport, Transport
Cancer-related genes, Disease related genes, Human disease related genes, Metabolic proteins, Plasma proteins, Potential drug targets, Predicted intracellular proteins, Predicted membrane proteins, Transporters	Sugar transport, Transport
Metabolic proteins, Predicted membrane proteins, Transporters	Sugar transport, Transport
Metabolic proteins, Predicted intracellular proteins, Predicted membrane proteins, Transporters	Differentiation, Spermatogenesis, Sugar transport, Transport
Disease related genes, Human disease related genes, Metabolic proteins, Potential drug targets, Predicted membrane proteins, Transporters	Sugar transport, Transport
Metabolic proteins, Predicted membrane proteins, Transporters	Sugar transport, Transport
Metabolic proteins, Plasma proteins, Predicted intracellular proteins, Predicted membrane proteins, Transporters	Sugar transport, Transport
Metabolic proteins, Predicted membrane proteins, Transporters	Antiport, Ion transport, Transport, Zinc transport
Human disease related genes, Predicted intracellular proteins, Predicted membrane proteins, Transporters	Antiport, Ion transport, Transport, Zinc transport
Disease related genes, Human disease related genes, Metabolic proteins, Plasma proteins, Potential drug targets, Predicted intracellular proteins, Predicted membrane proteins, Transporters	Ion transport, Sodium transport, Symport, Transport
Cancer-related genes, Disease related genes, Human disease related genes, Metabolic proteins, Potential drug targets, Predicted membrane proteins, Transporters	Ion transport, Sodium transport, Symport, Transport
Predicted membrane proteins, Transporters	
Essential proteins, FDA approved drug targets, Metabolic proteins, Predicted membrane proteins, Transporters	Amino-acid transport, Ion transport, Sodium transport, Symport, Transport
Metabolic proteins, Predicted membrane proteins, Transporters	Ion transport, Transport, Zinc transport
Cancer-related genes, CD markers, Essential proteins, Metabolic proteins, Plasma proteins, Predicted intracellular proteins, Predicted membrane proteins, Transporters	Amino-acid transport, Transport
Disease related genes, Human disease related genes, Metabolic proteins, Potential drug targets, Predicted intracellular proteins, Predicted membrane proteins, Transporters	Ion transport, Iron transport, Transport
Predicted membrane proteins, Transporters	Ion transport, Transport
Disease related genes, Essential proteins, Human disease related genes, Metabolic proteins, Potential drug targets, Predicted intracellular proteins, Predicted membrane proteins, Transporters	Symport, Transport
Metabolic proteins, Predicted intracellular proteins, Predicted membrane proteins, Transporters	Anion exchange, Antiport, Ion transport, Transport
Disease related genes, Human disease related genes, Metabolic proteins, Potential drug targets, Predicted intracellular proteins, Predicted membrane proteins, Transporters	Ion transport, Sodium transport, Symport, Transport
Essential proteins, Metabolic proteins, Predicted membrane proteins, Transporters	Ion transport, Sodium transport, Symport, Transport
Disease related genes, FDA approved drug targets, Human disease related genes, Metabolic proteins, Plasma proteins, Predicted membrane proteins, Transporters	Ion transport, Sodium transport, Sugar transport, Symport, Transport
Metabolic proteins, Predicted membrane proteins, Transporters	Ion transport, Sodium transport, Symport, Transport
Metabolic proteins, Predicted membrane proteins, Transporters	Ion transport, Sodium transport, Symport, Transport
Metabolic proteins, Predicted membrane proteins, Transporters	Amino-acid transport, Transport
Metabolic proteins, Predicted membrane proteins, Transporters	Amino-acid transport, Transport
Metabolic proteins, Predicted intracellular proteins, Predicted membrane proteins, Transporters	Amino-acid transport, Antiport, Transport

FDA approved drug targets, Metabolic proteins, Predicted intracellular proteins, Predicted membrane proteins, Transporters	Antiport, Calcium transport, Ion transport, Sodium transport, Transport
Disease related genes, Human disease related genes, Metabolic proteins, Potential drug targets, Predicted membrane proteins, Transporters	Antiport, Ion transport, Sodium transport, Transport
Metabolic proteins, Plasma proteins, Predicted membrane proteins, Transporters	Antiport, Ion transport, Sodium transport, Transport
Disease related genes, FDA approved drug targets, Human disease related genes, Metabolic proteins, Predicted membrane proteins, Transporters	Antiport, Ion transport, Sodium transport, Transport
Predicted intracellular proteins, Transporters	
Disease related genes, Human disease related genes, Potential drug targets, Predicted intracellular proteins, Predicted membrane proteins, Transporters	Antiport, Ion transport, Sodium transport, Transport
Predicted membrane proteins, Transporters	Antiport, Ion transport, Sodium transport, Transport
Metabolic proteins, Plasma proteins, Predicted intracellular proteins, Predicted membrane proteins, Transporters	Ion transport, Lipid transport, Transport
Disease related genes, Human disease related genes, Metabolic proteins, Plasma proteins, Potential drug targets, Predicted membrane proteins, Transporters	Ion transport, Lipid transport, Transport
Disease related genes, Human disease related genes, Metabolic proteins, Potential drug targets, Predicted membrane proteins, Transporters	Ion transport, Lipid transport, Transport
Metabolic proteins, Predicted membrane proteins, Transporters	Ion transport, Transport
Predicted membrane proteins, Transporters	Transport
Predicted intracellular proteins, Predicted secreted proteins	Differentiation, Neurogenesis
Disease related genes, Human disease related genes, Predicted membrane proteins	Hearing, Sensory transduction, Vision
Predicted intracellular proteins, Predicted membrane proteins	
Predicted intracellular proteins, Predicted membrane proteins, Transporters	Protein transport, Transport
Plasma proteins, Predicted intracellular proteins	
Predicted intracellular proteins	
Plasma proteins, Predicted intracellular proteins	Cell cycle, Cell division, Endocytosis, Mitosis, Protein transport, Transport
Predicted intracellular proteins	Transport
Disease related genes, Potential drug targets, Predicted intracellular proteins, Predicted membrane proteins, Transporters	Differentiation, Endocytosis, Osteogenesis, Transport
Disease related genes, Human disease related genes, Predicted intracellular proteins, Predicted membrane proteins	
Enzymes, Metabolic proteins, Predicted intracellular proteins	Lipid metabolism
Cancer-related genes, Essential proteins, Plasma proteins, Predicted intracellular proteins, Predicted membrane proteins, Predicted secreted proteins, Transporters	
Predicted intracellular proteins	Host-virus interaction, Immunity, Innate immunity, Protein transport, Transport
Enzymes, Predicted membrane proteins	
Enzymes, Predicted membrane proteins	
Cancer-related genes, Disease related genes, Enzymes, FDA approved drug targets, Human disease related genes, Plasma proteins, Predicted intracellular proteins, Transporters	Cell adhesion, Cell cycle, Host-virus interaction, Immunity
FDA approved drug targets, G-protein coupled receptors, Plasma proteins, Predicted membrane proteins	
Disease related genes, Enzymes, Human disease related genes, Metabolic proteins, Potential drug targets, Predicted intracellular proteins	Ubl conjugation pathway
Enzymes, Predicted membrane proteins, Transporters	Electron transport, Ion transport, Iron transport, Transport
Disease related genes, Enzymes, Plasma proteins, Potential drug targets, Predicted intracellular proteins	Cell cycle
Plasma proteins, Predicted membrane proteins, Transporters	
Predicted intracellular proteins	
Predicted membrane proteins, Transporters	
Essential proteins, Predicted intracellular proteins, Predicted membrane proteins	ER-Golgi transport, Protein transport, Transport
Disease related genes, Human disease related genes, Plasma proteins, Predicted intracellular proteins	
Predicted intracellular proteins	
Essential proteins, Plasma proteins, Predicted intracellular proteins	Cell adhesion, mRNA processing
Plasma proteins, Predicted intracellular proteins, Transporters	Exocytosis
Disease related genes, Enzymes, Essential proteins, Metabolic proteins, Potential drug targets, Predicted membrane proteins, Transporters	Stress response, Ubl conjugation pathway
FDA approved drug targets, G-protein coupled receptors, Predicted membrane proteins	
G-protein coupled receptors, Predicted membrane proteins	
Disease related genes, FDA approved drug targets, Human disease related genes, Predicted membrane proteins	Sensory transduction, Vision
G-protein coupled receptors, Predicted membrane proteins, Transporters	Sensory transduction, Taste
Predicted intracellular proteins	
Disease related genes, Human disease related genes, Predicted intracellular proteins, Predicted membrane proteins	
Predicted intracellular proteins, Predicted membrane proteins	
Disease related genes, G-protein coupled receptors, Human disease related genes, Predicted membrane proteins	
Predicted intracellular proteins	Transport
Predicted intracellular proteins	
Enzymes, Plasma proteins, Predicted intracellular proteins	Adaptive immunity, Immunity
Cancer-related genes, CD markers, Disease related genes, Enzymes, FDA approved drug targets, Human disease related genes, Plasma proteins, Predicted membrane proteins, Predicted secreted proteins, RAS pathw	Angiogenesis
Disease related genes, Enzymes, Human disease related genes, Potential drug targets, Predicted intracellular proteins, Predicted membrane proteins	Apoptosis, Differentiation, Growth regulation
Cancer-related genes, Disease related genes, Enzymes, FDA approved drug targets, Human disease related genes, Plasma proteins, Predicted membrane proteins, Predicted secreted proteins	Apoptosis, Differentiation, Growth regulation
Disease related genes, Plasma proteins, Predicted intracellular proteins, RAS pathway related proteins	
Predicted intracellular proteins	
Predicted intracellular proteins, Transporters	
Cancer-related genes, Disease related genes, Human disease related genes, Plasma proteins, Predicted intracellular proteins	
Predicted intracellular proteins, Transporters	
Plasma proteins, Predicted intracellular proteins	Host-virus interaction
CD markers, FDA approved drug targets, Human disease related genes, Predicted intracellular proteins, Predicted membrane proteins, Transporters	Immunity, Inflammatory response, Innate immunity
CD markers, FDA approved drug targets, Human disease related genes, Predicted intracellular proteins, Predicted membrane proteins, Transporters	Immunity, Inflammatory response, Innate immunity
Disease related genes, Potential drug targets, Predicted intracellular proteins, Predicted membrane proteins, Transporters	
Predicted intracellular proteins, Predicted membrane proteins	
Predicted membrane proteins	
Predicted membrane proteins, Transporters	Exocytosis, Hearing, Ion transport, Transport
Disease related genes, Potential drug targets, Predicted membrane proteins, Transporters	
Cancer-related genes, Enzymes, Predicted intracellular proteins, Predicted membrane proteins, Predicted secreted proteins, Transporters	Host-virus interaction
CD markers, Disease related genes, Human disease related genes, Predicted intracellular proteins, Predicted membrane proteins	
Cancer-related genes, CD markers, Human disease related genes, Predicted intracellular proteins, Predicted membrane proteins	Adaptive immunity, Host-virus interaction, Immunity, Innate immunity
CD markers, Plasma proteins, Predicted membrane proteins	Adaptive immunity, Apoptosis, Host-virus interaction, Immunity
Cancer-related genes, CD markers, FDA approved drug targets, Plasma proteins, Predicted intracellular proteins, Predicted membrane proteins	
Cancer-related genes, CD markers, FDA approved drug targets, Plasma proteins, Predicted intracellular proteins, Predicted membrane proteins, Predicted secreted proteins	Immunity
Cancer-related genes, Enzymes, Plasma proteins, Predicted intracellular proteins	Endocytosis
Cancer-related genes, Predicted intracellular proteins	Apoptosis, Cell cycle
Predicted intracellular proteins	Wnt signaling pathway
Disease related genes, Enzymes, Essential proteins, Human disease related genes, Potential drug targets, Predicted intracellular proteins	DNA damage, DNA repair, Ubl conjugation pathway
Predicted intracellular proteins, Predicted membrane proteins	Adaptive immunity, Immunity
Disease related genes, Human disease related genes, Plasma proteins, Potential drug targets, Predicted intracellular proteins, Predicted membrane proteins, Transporters	
Plasma proteins, Predicted membrane proteins	
Disease related genes, Human disease related genes, Potential drug targets, Predicted intracellular proteins, Predicted membrane proteins, Transporters, Voltage-gated ion channels	Adaptive immunity, Immunity, Ion transport, Transport
Predicted intracellular proteins, Predicted membrane proteins, Transporters	Calcium transport, Ion transport, Transport
Predicted membrane proteins, Transporters	
Predicted intracellular proteins, Predicted membrane proteins, Transporters	Transport
Predicted intracellular proteins	Cilium biogenesis/degradation
Disease related genes, Predicted intracellular proteins	
Enzymes, Predicted intracellular proteins	Adaptive immunity, Immunity, Transcription, Transcription regulation
Predicted intracellular proteins, Predicted membrane proteins	
Disease related genes, Human disease related genes, Potential drug targets, Predicted membrane proteins, Transporters	Immunity
Cancer-related genes, Enzymes, Essential proteins, Metabolic proteins, Predicted intracellular proteins	Cell cycle, Cell division, Mitosis, Ubl conjugation pathway
Enzymes, Essential proteins, Metabolic proteins, Predicted intracellular proteins	Apoptosis, DNA damage, DNA repair, Ubl conjugation pathway
Predicted membrane proteins	Host-virus interaction, Immunity
Predicted membrane proteins, Predicted secreted proteins	Host-virus interaction, Immunity
Enzymes, Metabolic proteins, Plasma proteins, Predicted intracellular proteins	Immunity, Innate immunity, Ubl conjugation pathway
Enzymes, Metabolic proteins, Plasma proteins, Predicted intracellular proteins	Immunity, Innate immunity, Ubl conjugation pathway
Plasma proteins, Predicted intracellular proteins	
Plasma proteins, Predicted intracellular proteins	
Cancer-related genes, Disease related genes, Plasma proteins, Predicted intracellular proteins	Apoptosis
Essential proteins, Predicted intracellular proteins	Protein transport, Transcription, Transcription regulation, Transport
Disease related genes, Essential proteins, Human disease related genes, Plasma proteins, Predicted intracellular proteins	
Cancer-related genes, Disease related genes, Human disease related genes, Plasma proteins, Predicted intracellular proteins	DNA damage, DNA repair, Transcription, Transcription regulation
Essential proteins, Predicted intracellular proteins	
Enzymes, FDA approved drug targets, Plasma proteins, Predicted intracellular proteins	
Enzymes, Predicted intracellular proteins	Ubl conjugation pathway, Unfolded protein response
Predicted intracellular proteins, Transcription factors	Transcription, Transcription regulation, Wnt signaling pathway
Enzymes, Metabolic proteins, Predicted intracellular proteins, Predicted membrane proteins	
Enzymes, Metabolic proteins, Predicted membrane proteins, Transporters	Host-virus interaction
Enzymes, Metabolic proteins, Predicted intracellular proteins, Predicted membrane proteins	
Enzymes, Metabolic proteins, Plasma proteins, Predicted intracellular proteins, Predicted membrane proteins	Immunity, Innate immunity, Lipid transport, Transport
Plasma proteins, Predicted intracellular proteins	
Predicted intracellular proteins, Transcription factors	Transcription, Transcription regulation

Molecular function	Disease involvement	Evidence	HPA evidence	UniProt evidence	NeXtProt evidence	RNA tissue specificity	RNA tissue distribution	RNA tissue specificity score
Kinase, Serine/threonine-protein kinase, Transferase		Evidence at protein level	Evidence at protein level	Evidence at protein level	Evidence at protein level	Tissue enhanced	Detected in all	
Developmental protein		Evidence at protein level	Evidence at protein level	Evidence at protein level	Evidence at protein level	Low tissue specificity	Detected in all	
Translocase	Atherosclerosis, Disease variant, FDA approved drug targets	Evidence at protein level	Evidence at protein level	Evidence at protein level	Evidence at protein level	Low tissue specificity	Detected in all	
Translocase	Alzheimer disease, Amyloidosis, Neurodegeneration	Evidence at protein level	Evidence at protein level	Evidence at protein level	Evidence at protein level	Tissue enhanced	Detected in many	
Translocase	Cancer-related genes, Disease variant	Evidence at protein level	Evidence at protein level	Evidence at protein level	Evidence at protein level	Tissue enhanced	Detected in many	
Translocase	Disease variant, Intrahepatic cholestasis	Evidence at protein level	Evidence at protein level	Evidence at protein level	Evidence at protein level	Tissue enriched	Detected in some	16
Translocase	Disease variant, Dyskeratosis congenita, Microphthalmia	Evidence at protein level	Evidence at protein level	Evidence at protein level	Evidence at protein level	Low tissue specificity	Detected in all	
Hydrolase, Translocase	Cancer-related genes, Deafness, FDA approved drug targets, Non-syndromic deafness	Evidence at protein level	Evidence at protein level	Evidence at protein level	Evidence at protein level	Low tissue specificity	Detected in many	
Translocase	Cancer-related genes	Evidence at protein level	Evidence at protein level	Evidence at protein level	Evidence at protein level	Tissue enhanced	Detected in many	
Translocase		Evidence at protein level	Evidence at protein level	Evidence at protein level	Evidence at protein level	Tissue enhanced	Detected in many	
Translocase	Cancer-related genes, Disease variant	Evidence at protein level	Evidence at protein level	Evidence at protein level	Evidence at protein level	Tissue enhanced	Detected in many	
Translocase	Cancer-related genes	Evidence at protein level	Evidence at protein level	Evidence at protein level	Evidence at protein level	Tissue enhanced	Detected in many	
Developmental protein, G-protein coupled receptor, Receptor, Transducer	Cancer-related genes, Disease variant	Evidence at protein level	Evidence at protein level	Evidence at protein level	Evidence at protein level	Low tissue specificity	Detected in all	
Hydrolase	Disease variant, FDA approved drug targets, Hereditary hemolytic anemia, SCID	Evidence at protein level	Evidence at protein level	Evidence at protein level	Evidence at protein level	Group enriched	Detected in many	8
Hydrolase, Metalloprotease, Protease	Alzheimer disease, Amyloidosis, Cancer-related genes, Disease variant, Neurodegeneration	Evidence at protein level	Evidence at protein level	Evidence at protein level	Evidence at protein level	Low tissue specificity	Detected in all	
Hydrolase, Metalloprotease, Protease		Evidence at protein level	Evidence at protein level	Evidence at protein level	Evidence at protein level	Tissue enriched	Detected in some	10
Hydrolase, Metalloprotease, Protease		Evidence at protein level	Evidence at protein level	Evidence at protein level	Evidence at protein level	Group enriched	Detected in many	6
Calmodulin-binding, Lyase	Obesity	Evidence at protein level	Evidence at protein level	Evidence at protein level	Evidence at protein level	Low tissue specificity	Detected in all	
Lyase		Evidence at protein level	Evidence at transcript level	Evidence at protein level	Evidence at protein level	Low tissue specificity	Detected in many	
Lyase		Evidence at protein level	Evidence at transcript level	Evidence at protein level	Evidence at protein level	Tissue enhanced	Detected in all	
Actin-binding, Calmodulin-binding		Evidence at protein level	Evidence at protein level	Evidence at protein level	Evidence at protein level	Low tissue specificity	Detected in all	
Actin-binding, Calmodulin-binding		Evidence at protein level	Evidence at protein level	Evidence at protein level	Evidence at protein level	Tissue enhanced	Detected in some	
Actin-binding, Calmodulin-binding	Disease variant	Evidence at protein level	Evidence at protein level	Evidence at protein level	Evidence at protein level	Low tissue specificity	Detected in all	
G-protein coupled receptor, Receptor, Transducer		Evidence at protein level	Evidence at protein level	Evidence at protein level	Evidence at protein level	Low tissue specificity	Detected in all	
G-protein coupled receptor, Receptor, Transducer		Evidence at protein level	Evidence at protein level	Evidence at protein level	Evidence at protein level	Tissue enriched	Detected in many	9
G-protein coupled receptor, Receptor, Transducer		Evidence at protein level	Evidence at transcript level	Evidence at protein level	Evidence at protein level	Tissue enhanced	Detected in many	
G-protein coupled receptor, Receptor, Transducer	Disease variant	Evidence at protein level	Evidence at protein level	Evidence at protein level	Evidence at protein level	Tissue enriched	Detected in many	8
G-protein coupled receptor, Receptor, Transducer	FDA approved drug targets	Evidence at protein level	Evidence at protein level	Evidence at protein level	Evidence at protein level	Tissue enhanced	Detected in many	
G-protein coupled receptor, Receptor, Transducer	FDA approved drug targets	Evidence at protein level	Evidence at protein level	Evidence at protein level	Evidence at protein level	Tissue enhanced	Detected in many	
G-protein coupled receptor, Receptor, Transducer	Cancer-related genes, FDA approved drug targets	Evidence at protein level	Evidence at protein level	Evidence at protein level	Evidence at protein level	Tissue enhanced	Detected in many	
G-protein coupled receptor, Receptor, Transducer	FDA approved drug targets	Evidence at protein level	Evidence at protein level	Evidence at protein level	Evidence at protein level	Tissue enhanced	Detected in many	
	Cancer-related genes, Proto-oncogene	Evidence at protein level	Evidence at protein level	Evidence at protein level	Evidence at protein level	Low tissue specificity	Detected in all	
DNA-binding, Receptor, RNA-binding	Cancer-related genes	Evidence at protein level	Evidence at protein level	Evidence at protein level	Evidence at protein level	Tissue enriched	Detected in many	17
		Evidence at protein level	Evidence at protein level	Evidence at protein level	Evidence at protein level	Low tissue specificity	Detected in all	
	Cancer-related genes	Evidence at protein level	Evidence at protein level	Evidence at protein level	Evidence at protein level	Tissue enhanced	Detected in many	
Calmodulin-binding	Cancer-related genes	Evidence at protein level	Evidence at protein level	Evidence at protein level	Evidence at protein level	Low tissue specificity	Detected in all	
Oxidoreductase		Evidence at protein level	Evidence at protein level	Evidence at protein level	Evidence at protein level	Tissue enhanced	Detected in many	
Oxidoreductase		Evidence at protein level	Evidence at protein level	Evidence at protein level	Evidence at protein level	Tissue enriched	Detected in many	9
		Evidence at protein level	Evidence at transcript level	Evidence at protein level	Evidence at protein level	Low tissue specificity	Detected in all	
Kinase, Receptor, Transferase, Tyrosine-protein kinase	Cancer-related genes, Disease variant, FDA approved drug targets, Proto-oncogene	Evidence at protein level	Evidence at protein level	Evidence at protein level	Evidence at protein level	Tissue enhanced	Detected in some	
Dioxygenase, Oxidoreductase		Evidence at protein level	Evidence at protein level	Evidence at protein level	Evidence at protein level	Tissue enhanced	Detected in many	
	Cancer-related genes	Evidence at protein level	Evidence at protein level	Evidence at protein level	Evidence at protein level	Tissue enhanced	Detected in many	
		Evidence at protein level	Evidence at protein level	Evidence at protein level	Evidence at protein level	Group enriched	Detected in some	9
		Evidence at protein level	Evidence at protein level	Evidence at protein level	Evidence at protein level	Tissue enhanced	Detected in many	
	Disease variant, Long QT syndrome	Evidence at protein level	Evidence at protein level	Evidence at protein level	Evidence at protein level	Tissue enhanced	Detected in many	
	Autism spectrum disorder, Cancer-related genes, Intellectual disability	Evidence at protein level	Evidence at protein level	Evidence at protein level	Evidence at protein level	Low tissue specificity	Detected in all	
Transferase		Evidence at protein level	Evidence at protein level	Evidence at protein level	Evidence at protein level	Low tissue specificity	Detected in all	
		Evidence at protein level	Evidence at protein level	Evidence at protein level	Evidence at protein level	Low tissue specificity	Detected in all	
		Evidence at protein level	Evidence at protein level	Evidence at protein level	Evidence at protein level	Low tissue specificity	Detected in all	
		Evidence at protein level	Evidence at protein level	Evidence at protein level	Evidence at protein level	Tissue enriched	Detected in many	5
Chloride channel, Developmental protein, Ion channel	Disease variant, FDA approved drug targets	Evidence at protein level	Evidence at protein level	Evidence at protein level	Evidence at protein level	Tissue enhanced	Detected in many	
	Disease variant, Neurodegeneration	Evidence at protein level	Evidence at protein level	Evidence at protein level	Evidence at protein level	Low tissue specificity	Detected in all	
Chloride channel, Ion channel		Evidence at protein level	Evidence at protein level	Evidence at protein level	Evidence at protein level	Tissue enriched	Detected in many	22
Chloride channel, Ion channel, Voltage-gated channel		Evidence at protein level	Evidence at transcript level	Evidence at protein level	Evidence at protein level	Low tissue specificity	Detected in all	
Aminopeptidase, Developmental protein, Host cell receptor for virus entry, Hydrolase, Metalloprotease, Protease, Receptor		Evidence at protein level	Evidence at protein level	Evidence at protein level	Evidence at protein level	Group enriched	Detected in many	6
Phospholipase A2 inhibitor	Cancer-related genes, FDA approved drug targets	Evidence at protein level	Evidence at protein level	Evidence at protein level	Evidence at protein level	Tissue enhanced	Detected in all	
		Evidence at protein level	Evidence at protein level	Evidence at protein level	Evidence at protein level	Tissue enhanced	Detected in many	
Phospholipase A2 inhibitor	FDA approved drug targets	Evidence at protein level	Evidence at protein level	Evidence at protein level	Evidence at protein level	Tissue enhanced	Detected in all	
	Disease variant, Epilepsy, Intellectual disability	Evidence at protein level	Evidence at protein level	Evidence at protein level	Evidence at protein level	Low tissue specificity	Detected in all	
Activator, Chromatin regulator, Repressor		Evidence at protein level	Evidence at protein level	Evidence at protein level	Evidence at protein level	Tissue enhanced	Detected in all	
		Evidence at protein level	Evidence at protein level	Evidence at protein level	Evidence at protein level	Tissue enhanced	Detected in many	
	Cancer-related genes, Disease variant, Tumor suppressor	Evidence at protein level	Evidence at protein level	Evidence at protein level	Evidence at protein level	Tissue enhanced	Detected in all	
Receptor	Atherosclerosis	Evidence at protein level	Evidence at protein level	Evidence at protein level	Evidence at protein level	Group enriched	Detected in many	6
	Disease variant	Evidence at protein level	Evidence at protein level	Evidence at transcript lev	Evidence at protein level	Tissue enhanced	Detected in many	
	Diabetes mellitus, Disease variant	Evidence at protein level	Evidence at protein level	Evidence at protein level	Evidence at protein level	Low tissue specificity	Detected in all	
Blood group antigen		Evidence at protein level	Evidence at protein level	Evidence at protein level	Evidence at protein level	Tissue enhanced	Detected in many	
		Evidence at protein level	Evidence at protein level	Evidence at protein level	Evidence at protein level	Tissue enriched	Detected in some	15
		Evidence at protein level	Evidence at protein level	Evidence at protein level	Evidence at protein level	Tissue enhanced	Detected in many	
Blood group antigen		Evidence at protein level	Evidence at protein level	Evidence at protein level	Evidence at protein level	Tissue enhanced	Detected in all	
	Disease variant	Evidence at protein level	Evidence at protein level	Evidence at protein level	Evidence at protein level	Group enriched	Detected in many	4
Kinase, Serine/threonine-protein kinase, Transferase	Proto-oncogene	Evidence at protein level	Evidence at protein level	Evidence at protein level	Evidence at protein level	Low tissue specificity	Detected in all	
GTPase activation		Evidence at protein level	Evidence at protein level	Evidence at protein level	Evidence at protein level	Group enriched	Detected in all	5
GTPase activation		Evidence at protein level	Evidence at protein level	Evidence at protein level	Evidence at protein level	Low tissue specificity	Detected in all	
GTPase activation		Evidence at protein level	Evidence at protein level	Evidence at protein level	Evidence at protein level	Tissue enhanced	Detected in many	
GTPase activation		Evidence at protein level	Evidence at transcript level	Evidence at protein level	Evidence at protein level	Tissue enhanced	Detected in all	
Developmental protein, GTPase activation		Evidence at protein level	Evidence at protein level	Evidence at protein level	Evidence at protein level	Tissue enriched	Detected in many	5
GTPase activation		Evidence at protein level	Evidence at protein level	Evidence at protein level	Evidence at protein level	Group enriched	Detected in many	6
GTPase activation, Guanine-nucleotide releasing factor		Evidence at protein level	Evidence at protein level	Evidence at protein level	Evidence at protein level	Tissue enhanced	Detected in all	
GTPase activation, Guanine-nucleotide releasing factor		Evidence at protein level	Evidence at protein level	Evidence at protein level	Evidence at protein level	Low tissue specificity	Detected in all	
GTPase activation, Guanine-nucleotide releasing factor		Evidence at protein level	Evidence at protein level	Evidence at protein level	Evidence at protein level	Tissue enhanced	Detected in many	
Guanine-nucleotide releasing factor	Disease variant, Retinitis pigmentosa	Evidence at protein level	Evidence at protein level	Evidence at protein level	Evidence at protein level	Low tissue specificity	Detected in all	
Guanine-nucleotide releasing factor		Evidence at protein level	Evidence at protein level	Evidence at protein level	Evidence at protein level	Low tissue specificity	Detected in many	
Guanine-nucleotide releasing factor	Proto-oncogene	Evidence at protein level	Evidence at protein level	Evidence at protein level	Evidence at protein level	Tissue enhanced	Detected in many	
		Evidence at protein level	Evidence at protein level	Evidence at protein level	Evidence at protein level	Tissue enhanced	Detected in all	
		Evidence at protein level	Evidence at protein level	Evidence at transcript lev	Evidence at protein level	Tissue enhanced	Detected in many	
Signal transduction inhibitor		Evidence at protein level	Evidence at protein level	Evidence at protein level	Evidence at protein level	Tissue enhanced	Detected in all	
		Evidence at protein level	Evidence at protein level	Evidence at protein level	Evidence at protein level	Tissue enhanced	Detected in all	
		Evidence at protein level	Evidence at protein level	Evidence at protein level	Evidence at protein level	Tissue enhanced	Detected in many	
GTPase activation		Evidence at protein level	Evidence at protein level	Evidence at protein level	Evidence at protein level	Tissue enhanced	Detected in many	
Receptor		Evidence at protein level	Evidence at protein level	Evidence at protein level	Evidence at protein level	Tissue enriched	Detected in some	34
Chromatin regulator, DNA-binding		Evidence at protein level	Evidence at protein level	Evidence at protein level	Evidence at protein level	Low tissue specificity	Detected in all	
Ion channel, Sodium channel		Evidence at protein level	Evidence at protein level	Evidence at protein level	Evidence at protein level	Tissue enriched	Detected in many	6
Transferase		Evidence at protein level	Evidence at protein level	Evidence at protein level	Evidence at protein level	Low tissue specificity	Detected in all	
Translocase		Evidence at protein level	Evidence at protein level	Evidence at protein level	Evidence at protein level	Low tissue specificity	Detected in all	
Translocase		Evidence at protein level	Evidence at transcript level	Evidence at protein level	Evidence at protein level	Group enriched	Detected in some	4
		Evidence at protein level	Evidence at protein level	Evidence at protein level	Evidence at protein level	Low tissue specificity	Detected in all	
Hydrolase, Metalloprotease, Protease		Evidence at protein level	Evidence at protein level	Evidence at protein level	Evidence at protein level	Tissue enhanced	Detected in many	
Calmodulin-binding, Translocase	Disease variant, FDA approved drug targets, Intellectual disability	Evidence at protein level	Evidence at protein level	Evidence at protein level	Evidence at protein level	Tissue enhanced	Detected in all	
Calmodulin-binding, Translocase	Cancer-related genes, Disease variant, FDA approved drug targets, Neurodegeneration	Evidence at protein level	Evidence at protein level	Evidence at protein level	Evidence at protein level	Tissue enriched	Detected in some	7
Calmodulin-binding, Translocase	FDA approved drug targets	Evidence at protein level	Evidence at protein level	Evidence at protein level	Evidence at protein level	Tissue enhanced	Detected in all	
		Evidence at protein level	Evidence at protein level	Evidence at protein level	Evidence at protein level	Low tissue specificity	Detected in all	
		Evidence at protein level	Evidence at protein level	Evidence at protein level	Evidence at protein level	Low tissue specificity	Detected in all	
Translocase	Disease variant, Intellectual disability	Evidence at protein level	Evidence at protein level	Evidence at protein level	Evidence at protein level	Group enriched	Detected in some	6
Translocase	Disease variant, Intrahepatic cholestasis	Evidence at protein level	Evidence at protein level	Evidence at protein level	Evidence at protein level	Tissue enhanced	Detected in many	
Translocase		Evidence at protein level	Evidence at protein level	Evidence at protein level	Evidence at protein level	Low tissue specificity	Detected in all	
Host cell receptor for virus entry, Kinase, Receptor, Transferase, Tyrosine-protein kinase	FDA approved drug targets, Oncogene, Proto-oncogene	Evidence at protein level	Evidence at protein level	Evidence at protein level	Evidence at protein level	Low tissue specificity	Detected in all	
	Amyloidosis, Cancer-related genes, Disease variant	Evidence at protein level	Evidence at protein level	Evidence at protein level	Evidence at protein level	Low tissue specificity	Detected in all	
Aspartyl protease, Hydrolase, Protease		Evidence at protein level	Evidence at protein level	Evidence at protein level	Evidence at protein level	Group enriched	Detected in all	4
		Evidence at protein level	Evidence at protein level	Evidence at protein level	Evidence at protein level	Low tissue specificity	Detected in all	
Actin-binding		Evidence at protein level	Evidence at protein level	Evidence at protein level	Evidence at protein level	Tissue enhanced	Detected in many	
		Evidence at protein level	Evidence at protein level	Evidence at protein level	Evidence at protein level	Tissue enhanced	Detected in all	
G-protein coupled receptor, Receptor, Transducer	FDA approved drug targets	Evidence at protein level	Evidence at protein level	Evidence at protein level	Evidence at protein level	Tissue enhanced	Detected in many	
Eye lens protein	Cataract	Evidence at protein level	Evidence at protein level	Evidence at protein level	Evidence at protein level	Low tissue specificity	Detected in many	
		Evidence at protein level	Evidence at protein level	Evidence at protein level	Evidence at protein level	Tissue enhanced	Detected in many	
		Evidence at protein level	Evidence at protein level	Evidence at protein level	Evidence at protein level	Tissue enhanced	Detected in many	
Kinase, Receptor, Serine/threonine-protein kinase, Transferase	Disease variant, FDA approved drug targets	Evidence at protein level	Evidence at protein level	Evidence at protein level	Evidence at protein level	Low tissue specificity	Detected in all	
Kinase, Transferase, Tyrosine-protein kinase		Evidence at protein level	Evidence at protein level	Evidence at protein level	Evidence at protein level	Tissue enriched	Detected in many	11
		Evidence at protein level	Evidence at protein level	Evidence at protein level	Evidence at protein level	Low tissue specificity	Detected in many	
Allosteric enzyme, Kinase, Serine/threonine-protein kinase, Transferase	Cancer-related genes, Cardiomyopathy, Deafness, Disease variant, Ectodermal dysplasia, F	Evidence at protein level	Evidence at protein level	Evidence at protein level	Evidence at protein level	Low tissue specificity	Detected in all	
Kinase, Transferase, Tyrosine-protein kinase	Cancer-related genes, Disease variant, Dwarfism, FDA approved drug targets	Evidence at protein level	Evidence at protein level	Evidence at protein level	Evidence at protein level	Tissue enhanced	Detected in many	
Developmental protein	Limb-girdle muscular dystrophy	Evidence at protein level	Evidence at protein level	Evidence at protein level	Evidence at protein level	Tissue enhanced	Detected in many	
		Evidence at protein level	Evidence at protein level	Evidence at protein level	Evidence at protein level	Low tissue specificity	Detected in many	
Receptor		Evidence at protein level	Evidence at protein level	Evidence at protein level	Evidence at protein level	Tissue enhanced	Detected in many	
	Cancer-related genes, Disease variant, Primary mitochondrial disease	Evidence at protein level	Evidence at protein level	Evidence at protein level	Evidence at protein level	Low tissue specificity	Detected in all	
		Evidence at protein level	Evidence at protein level	Evidence at protein level	Evidence at protein level	Low tissue specificity	Detected in all	
	Disease variant	Evidence at protein level	Evidence at protein level	Evidence at protein level	Evidence at protein level	Tissue enriched	Detected in some	277

Lyase	FDA approved drug targets	Evidence at protein level	Evidence at protein level	Evidence at protein level	Evidence at protein level	Tissue enriched	Detected in some	13
Calcium channel, Calmodulin-binding, Ion channel, Voltage-gated channel	Autism, Autism spectrum disorder, Brugada syndrome, Disease variant, Epilepsy, FDA appro	Evidence at protein level	Evidence at protein level	Evidence at protein level	Evidence at protein level	Tissue enhanced	Detected in many	
Developmental protein	Tumor suppressor	Evidence at protein level	Evidence at protein level	Evidence at protein level	Evidence at protein level	Tissue enhanced	Detected in many	
G-protein coupled receptor, Receptor, Transducer	Disease variant, FDA approved drug targets	Evidence at protein level	Evidence at protein level	Evidence at protein level	Evidence at protein level	Tissue enhanced	Detected in many	
Actin-binding, Calmodulin-binding, Muscle protein		Evidence at protein level	Evidence at protein level	Evidence at protein level	Evidence at protein level	Low tissue specificity	Detected in all	
		Evidence at protein level	Evidence at protein level	Evidence at protein level	Evidence at protein level	Low tissue specificity	Detected in many	
		Evidence at protein level	Evidence at protein level	Evidence at protein level	Evidence at protein level	Low tissue specificity	Detected in many	
Protease inhibitor, Thiol protease inhibitor	Cancer-related genes, Palmoplantar keratoderma	Evidence at protein level	Evidence at protein level	Evidence at protein level	Evidence at protein level	Low tissue specificity	Detected in all	
RNA-binding, rRNA-binding	Congenital generalized lipodystrophy, Diabetes mellitus	Evidence at protein level	Evidence at protein level	Evidence at protein level	Evidence at protein level	Low tissue specificity	Detected in all	
		Evidence at protein level	Evidence at protein level	Evidence at protein level	Evidence at protein level	Tissue enhanced	Detected in all	
Activator, Developmental protein		Evidence at protein level	Evidence at protein level	Evidence at protein level	Evidence at protein level	Group enriched	Detected in some	4
	Congenital disorder of glycosylation, Disease variant	Evidence at protein level	Evidence at protein level	Evidence at protein level	Evidence at protein level	Low tissue specificity	Detected in all	
		Evidence at protein level	Evidence at protein level	Evidence at protein level	Evidence at protein level	Low tissue specificity	Detected in all	
Developmental protein		Evidence at protein level	Evidence at transcript level	Evidence at protein level	Evidence at protein level	Tissue enhanced	Detected in all	
Guanine-nucleotide releasing factor	Epilepsy, Intellectual disability, Neurodegeneration	Evidence at protein level	Evidence at protein level	Evidence at protein level	Evidence at protein level	Tissue enhanced	Detected in all	
Guanine-nucleotide releasing factor	Disease variant, Neurodegeneration, Spinocerebellar ataxia	Evidence at protein level	Evidence at protein level	Evidence at protein level	Evidence at protein level	Tissue enhanced	Detected in many	
		Evidence at protein level	Evidence at protein level	Evidence at protein level	Evidence at protein level	Low tissue specificity	Detected in all	
G-protein coupled receptor, Receptor, Transducer	FDA approved drug targets	Evidence at protein level	Evidence at transcript level	Evidence at protein level	Evidence at protein level	Group enriched	Detected in some	7
G-protein coupled receptor, Receptor, Transducer	Cancer-related genes, FDA approved drug targets	Evidence at protein level	Evidence at transcript level	Evidence at protein level	Evidence at protein level	Group enriched	Detected in some	33
Cyclin		Evidence at protein level	Evidence at protein level	Evidence at protein level	Evidence at protein level	Low tissue specificity	Detected in all	
G-protein coupled receptor, Receptor, Transducer	Disease variant	Evidence at protein level	Evidence at protein level	Evidence at protein level	Evidence at protein level	Tissue enhanced	Detected in many	
G-protein coupled receptor, Receptor, Transducer	FDA approved drug targets	Evidence at protein level	Evidence at protein level	Evidence at protein level	Evidence at protein level	Tissue enhanced	Detected in some	
G-protein coupled receptor, Receptor, Transducer		Evidence at protein level	Evidence at protein level	Evidence at protein level	Evidence at protein level	Low tissue specificity	Detected in many	
Protease inhibitor, Serine protease inhibitor		Evidence at protein level	Evidence at protein level	Evidence at protein level	Evidence at protein level	Tissue enriched	Detected in many	17
		Evidence at protein level	Evidence at protein level	Evidence at protein level	Evidence at protein level	Tissue enhanced	Detected in all	
Receptor		Evidence at protein level	Evidence at protein level	Evidence at protein level	Evidence at protein level	Tissue enhanced	Detected in many	
	FDA approved drug targets	Evidence at protein level	Evidence at protein level	Evidence at protein level	Evidence at protein level	Tissue enriched	Detected in many	9
Receptor		Evidence at protein level	Evidence at protein level	Evidence at protein level	Evidence at protein level	Tissue enhanced	Detected in many	
Receptor		Evidence at protein level	Evidence at protein level	Evidence at protein level	Evidence at protein level	Group enriched	Detected in many	9
Receptor	Cancer-related genes, FDA approved drug targets	Evidence at protein level	Evidence at protein level	Evidence at protein level	Evidence at protein level	Tissue enhanced	Detected in many	
Receptor	Disease variant	Evidence at protein level	Evidence at protein level	Evidence at protein level	Evidence at protein level	Group enriched	Detected in many	5
		Evidence at protein level	Evidence at protein level	Evidence at protein level	Evidence at protein level	Low tissue specificity	Detected in all	
	FDA approved drug targets	Evidence at protein level	Evidence at protein level	Evidence at protein level	Evidence at protein level	Tissue enhanced	Detected in many	
Hydrolase, Receptor, Transferase	Cancer-related genes, Diabetes mellitus	Evidence at protein level	Evidence at protein level	Evidence at protein level	Evidence at protein level	Tissue enhanced	Detected in many	
Receptor	FDA approved drug targets	Evidence at protein level	Evidence at protein level	Evidence at protein level	Evidence at protein level	Tissue enriched	Detected in many	16
Receptor	FDA approved drug targets	Evidence at protein level	Evidence at protein level	Evidence at protein level	Evidence at protein level	Tissue enriched	Detected in some	9
Host cell receptor for virus entry, Receptor	FDA approved drug targets	Evidence at protein level	Evidence at protein level	Evidence at protein level	Evidence at protein level	Tissue enhanced	Detected in all	
Receptor	Cancer-related genes, Disease variant	Evidence at protein level	Evidence at protein level	Evidence at protein level	Evidence at protein level	Tissue enhanced	Detected in many	
Cytokine	Cancer-related genes, Disease variant	Evidence at protein level	Evidence at protein level	Evidence at protein level	Evidence at protein level	Tissue enhanced	Detected in some	
Blood group antigen, Receptor	Cancer-related genes, FDA approved drug targets	Evidence at protein level	Evidence at protein level	Evidence at protein level	Evidence at protein level	Tissue enhanced	Detected in all	
		Evidence at protein level	Evidence at protein level	Evidence at protein level	Evidence at protein level	Low tissue specificity	Detected in all	
Receptor	Cancer-related genes, Disease variant, FDA approved drug targets	Evidence at protein level	Evidence at protein level	Evidence at protein level	Evidence at protein level	Tissue enriched	Detected in many	4
Receptor		Evidence at protein level	Evidence at protein level	Evidence at protein level	Evidence at protein level	Tissue enhanced	Detected in many	
Host cell receptor for virus entry, Receptor	FDA approved drug targets	Evidence at protein level	Evidence at protein level	Evidence at protein level	Evidence at protein level	Tissue enhanced	Detected in many	
	Disease variant	Evidence at protein level	Evidence at protein level	Evidence at protein level	Evidence at protein level	Tissue enriched	Detected in many	13
		Evidence at protein level	Evidence at protein level	Evidence at protein level	Evidence at protein level	Tissue enriched	Detected in many	14
	Cancer-related genes	Evidence at protein level	Evidence at protein level	Evidence at protein level	Evidence at protein level	Low tissue specificity	Detected in all	
	Congenital dyserythropoietic anemia, Disease variant, Hereditary hemolytic anemia	Evidence at protein level	Evidence at protein level	Evidence at protein level	Evidence at protein level	Low tissue specificity	Detected in all	
		Evidence at protein level	Evidence at protein level	Evidence at protein level	Evidence at protein level	Low tissue specificity	Detected in all	
		Evidence at protein level	Evidence at protein level	Evidence at protein level	Evidence at protein level	Low tissue specificity	Detected in all	
		Evidence at protein level	Evidence at protein level	Evidence at protein level	Evidence at protein level	Low tissue specificity	Detected in all	
	Cancer-related genes, Disease variant, Ectodermal dysplasia	Evidence at protein level	Evidence at protein level	Evidence at protein level	Evidence at protein level	Tissue enhanced	Detected in many	
		Evidence at protein level	Evidence at protein level	Evidence at protein level	Evidence at protein level	Tissue enhanced	Detected in many	
	Disease variant, Intellectual disability	Evidence at protein level	Evidence at protein level	Evidence at protein level	Evidence at protein level	Group enriched	Detected in some	13
	Cancer-related genes	Evidence at protein level	Evidence at protein level	Evidence at protein level	Evidence at protein level	Tissue enriched	Detected in some	11
	Cardiomyopathy, Disease variant, Intellectual disability	Evidence at protein level	Evidence at protein level	Evidence at protein level	Evidence at protein level	Tissue enhanced	Detected in many	
		Evidence at protein level	Evidence at protein level	Evidence at protein level	Evidence at protein level	Tissue enhanced	Detected in some	
	Disease variant, Ectodermal dysplasia, Hypotrichosis	Evidence at protein level	Evidence at protein level	Evidence at protein level	Evidence at protein level	Tissue enhanced	Detected in many	
		Evidence at protein level	Evidence at protein level	Evidence at transcript lev	Evidence at protein level	Group enriched	Detected in some	5
	Cancer-related genes	Evidence at protein level	Evidence at protein level	Evidence at protein level	Evidence at protein level	Tissue enhanced	Detected in many	
		Evidence at protein level	Evidence at protein level	Evidence at protein level	Evidence at protein level	Tissue enhanced	Detected in many	
Kinase, Serine/threonine-protein kinase, Transferase		Evidence at protein level	Evidence at protein level	Evidence at protein level	Evidence at protein level	Low tissue specificity	Detected in all	
Kinase, Serine/threonine-protein kinase, Transferase	Lissencephaly, Neurodegeneration	Evidence at protein level	Evidence at protein level	Evidence at protein level	Evidence at protein level	Tissue enhanced	Detected in all	
	Cancer-related genes, Oncogene	Evidence at protein level	Evidence at protein level	Evidence at protein level	Evidence at protein level	Tissue enriched	Detected in many	5
		Evidence at protein level	Evidence at protein level	Evidence at protein level	Evidence at protein level	Tissue enriched	Detected in some	9
Developmental protein, G-protein coupled receptor, Receptor, Transducer	Disease variant	Evidence at protein level	Evidence at protein level	Evidence at protein level	Evidence at protein level	Tissue enhanced	Detected in many	
Developmental protein, Glycosidase, Hydrolase		Evidence at protein level	Evidence at protein level	Evidence at protein level	Evidence at protein level	Tissue enhanced	Detected in all	
	Cancer-related genes	Evidence at protein level	Evidence at protein level	Evidence at protein level	Evidence at protein level	Low tissue specificity	Detected in all	
		Evidence at protein level	Evidence at protein level	Evidence at protein level	Evidence at protein level	Tissue enhanced	Detected in many	
		Evidence at protein level	Evidence at protein level	Evidence at protein level	Evidence at protein level	Tissue enhanced	Detected in many	
		Evidence at protein level	Evidence at protein level	Evidence at protein level	Evidence at protein level	Low tissue specificity	Detected in all	
G-protein coupled receptor, Receptor, Transducer	FDA approved drug targets	Evidence at protein level	Evidence at protein level	Evidence at protein level	Evidence at protein level	Tissue enhanced	Detected in many	
G-protein coupled receptor, Receptor, Transducer	FDA approved drug targets	Evidence at protein level	Evidence at protein level	Evidence at protein level	Evidence at protein level	Tissue enhanced	Detected in some	
Ion channel, Ligand-gated ion channel, Receptor	Congenital myasthenic syndrome, Disease variant	Evidence at protein level	Evidence at protein level	Evidence at protein level	Evidence at protein level	Tissue enriched	Detected in some	8
Ion channel, Ligand-gated ion channel, Receptor	Disease variant, Epilepsy, FDA approved drug targets	Evidence at protein level	Evidence at protein level	Evidence at protein level	Evidence at protein level	Tissue enriched	Detected in some	8
Ion channel, Ligand-gated ion channel, Receptor	Disease variant, FDA approved drug targets	Evidence at protein level	Evidence at protein level	Evidence at protein level	Evidence at protein level	Group enriched	Detected in some	6
Ion channel, Ligand-gated ion channel, Receptor	FDA approved drug targets	Evidence at protein level	Evidence at transcript level	Evidence at protein level	Evidence at protein level	Low tissue specificity	Detected in many	
Ion channel, Ligand-gated ion channel, Receptor	FDA approved drug targets	Evidence at protein level	Evidence at transcript level	Evidence at protein level	Evidence at protein level	Tissue enhanced	Detected in some	
Ion channel, Ligand-gated ion channel, Receptor	Congenital myasthenic syndrome, Disease variant	Evidence at protein level	Evidence at protein level	Evidence at protein level	Evidence at protein level	Tissue enriched	Detected in some	13
		Evidence at protein level	Evidence at protein level	Evidence at protein level	Evidence at protein level	Low tissue specificity	Detected in all	
Hydrolase, Metalloprotease, Protease		Evidence at protein level	Evidence at protein level	Evidence at protein level	Evidence at protein level	Tissue enhanced	Detected in some	
	Disease variant	Evidence at protein level	Evidence at protein level	Evidence at protein level	Evidence at protein level	Group enriched	Detected in many	4
	Leukodystrophy	Evidence at protein level	Evidence at protein level	Evidence at protein level	Evidence at protein level	Group enriched	Detected in many	5
	Disease variant, Primary hypomagnesemia	Evidence at protein level	Evidence at protein level	Evidence at protein level	Evidence at protein level	Tissue enhanced	Detected in some	
	Disease variant	Evidence at protein level	Evidence at protein level	Evidence at protein level	Evidence at protein level	Tissue enhanced	Detected in many	
		Evidence at protein level	Evidence at transcript level	Evidence at protein level	Evidence at protein level	Low tissue specificity	Detected in single	
	Cancer-related genes, Williams-Beuren syndrome	Evidence at protein level	Evidence at protein level	Evidence at protein level	Evidence at protein level	Tissue enhanced	Detected in many	
Chloride channel, Ion channel	Cancer-related genes, Williams-Beuren syndrome	Evidence at protein level	Evidence at protein level	Evidence at protein level	Evidence at protein level	Tissue enhanced	Detected in many	
	Cancer-related genes	Evidence at protein level	Evidence at protein level	Evidence at protein level	Evidence at protein level	Tissue enhanced	Detected in many	
Host cell receptor for virus entry, Receptor	Deafness, Non-syndromic deafness	Evidence at protein level	Evidence at protein level	Evidence at protein level	Evidence at protein level	Tissue enhanced	Detected in some	
Host cell receptor for virus entry, Receptor		Evidence at protein level	Evidence at protein level	Evidence at protein level	Evidence at protein level	Tissue enriched	Detected in many	6
Chloride channel, Ion channel, Oxidoreductase, Voltage-gated channel		Evidence at protein level	Evidence at protein level	Evidence at protein level	Evidence at protein level	Low tissue specificity	Detected in all	
Chloride channel, Ion channel, Oxidoreductase, Voltage-gated channel		Evidence at protein level	Evidence at protein level	Evidence at protein level	Evidence at protein level	Tissue enhanced	Detected in all	
Chloride channel, Ion channel, Oxidoreductase, Voltage-gated channel	Deafness, Non-syndromic deafness	Evidence at protein level	Evidence at protein level	Evidence at protein level	Evidence at protein level	Tissue enhanced	Detected in many	
		Evidence at protein level	Evidence at protein level	Evidence at protein level	Evidence at protein level	Low tissue specificity	Detected in all	
		Evidence at protein level	Evidence at protein level	Evidence at protein level	Evidence at protein level	Low tissue specificity	Detected in all	
G-protein coupled receptor, Receptor, Transducer		Evidence at protein level	Evidence at protein level	Evidence at protein level	Evidence at protein level	Tissue enhanced	Detected in many	
Calcium channel, Ion channel, Ligand-gated ion channel, Sodium channel	Disease variant, Retinitis pigmentosa	Evidence at protein level	Evidence at protein level	Evidence at protein level	Evidence at protein level	Tissue enriched	Detected in many	11
	Amelogenesis imperfecta, Cone-rod dystrophy, Disease variant	Evidence at protein level	Evidence at protein level	Evidence at protein level	Evidence at protein level	Tissue enhanced	Detected in all	
G-protein coupled receptor, Receptor, Transducer	FDA approved drug targets, Neurodegeneration, Obesity	Evidence at protein level	Evidence at protein level	Evidence at protein level	Evidence at protein level	Tissue enhanced	Detected in many	
	Congenital disorder of glycosylation	Evidence at protein level	Evidence at protein level	Evidence at protein level	Evidence at protein level	Low tissue specificity	Detected in all	
	Cancer-related genes, Disease variant, Epidermolysis bullosa	Evidence at protein level	Evidence at protein level	Evidence at protein level	Evidence at protein level	Tissue enriched	Detected in many	7
	Congenital muscular dystrophy, Disease variant	Evidence at protein level	Evidence at protein level	Evidence at protein level	Evidence at protein level	Low tissue specificity	Detected in many	
Actin-binding		Evidence at protein level	Evidence at protein level	Evidence at protein level	Evidence at protein level	Low tissue specificity	Detected in all	
Hydrolase		Evidence at protein level	Evidence at protein level	Evidence at protein level	Evidence at protein level	Low tissue specificity	Detected in all	
Blood group antigen, Host cell receptor for virus entry, Receptor		Evidence at protein level	Evidence at protein level	Evidence at protein level	Evidence at protein level	Tissue enhanced	Detected in many	
		Evidence at protein level	Evidence at protein level	Evidence at protein level	Evidence at protein level	Tissue enhanced	Detected in many	
G-protein coupled receptor, Receptor, Transducer		Evidence at protein level	Evidence at protein level	Evidence at protein level	Evidence at protein level	Tissue enriched	Detected in some	5
Chaperone, Eye lens protein	Cancer-related genes, Cardiomyopathy, Cataract, Disease variant, Myofibrillar myopathy	Evidence at protein level	Evidence at protein level	Evidence at protein level	Evidence at protein level	Tissue enhanced	Detected in many	
Cytokine, Growth factor	Cancer-related genes	Evidence at protein level	Evidence at protein level	Evidence at protein level	Evidence at protein level	Low tissue specificity	Detected in all	
Kinase, Receptor, Transferase, Tyrosine-protein kinase	Cancer-related genes, Disease variant, FDA approved drug targets, Neurodegeneration, Prot	Evidence at protein level	Evidence at protein level	Evidence at protein level	Evidence at protein level	Tissue enhanced	Detected in all	
Kinase, Serine/threonine-protein kinase, Transferase	Disease variant	Evidence at protein level	Evidence at protein level	Evidence at protein level	Evidence at protein level	Low tissue specificity	Detected in all	
		Evidence at protein level	Evidence at protein level	Evidence at protein level	Evidence at protein level	Low tissue specificity	Detected in all	
Activator	Cancer-related genes, Disease variant, Intellectual disability	Evidence at protein level	Evidence at protein level	Evidence at protein level	Evidence at protein level	Low tissue specificity	Detected in all	
	Cancer-related genes, Disease variant, Ectodermal dysplasia	Evidence at protein level	Evidence at protein level	Evidence at protein level	Evidence at protein level	Low tissue specificity	Detected in all	
	Cancer-related genes	Evidence at protein level	Evidence at protein level	Evidence at protein level	Evidence at protein level	Low tissue specificity	Detected in all	
Cytokine		Evidence at protein level	Evidence at protein level	Evidence at protein level	Evidence at protein level	Low tissue specificity	Detected in many	
G-protein coupled receptor, Receptor, Transducer	Age-related macular degeneration	Evidence at protein level	Evidence at protein level	Evidence at protein level	Evidence at protein level	Tissue enhanced	Detected in many	
Host cell receptor for virus entry, Receptor		Evidence at protein level	Evidence at protein level	Evidence at protein level	Evidence at protein level	Tissue enhanced	Detected in many	
G-protein coupled receptor, Receptor, Transducer	Cancer-related genes	Evidence at protein level	Evidence at protein level	Evidence at protein level	Evidence at protein level	Tissue enhanced	Detected in many	
Oxidoreductase, Translocase		Evidence at protein level	Evidence at protein level	Evidence at protein level	Evidence at protein level	Low tissue specificity	Detected in all	
Guanine-nucleotide releasing factor		Evidence at protein level	Evidence at protein level	Evidence at protein level	Evidence at protein level	Low tissue specificity	Detected in all	
Guanine-nucleotide releasing factor		Evidence at protein level	Evidence at protein level	Evidence at protein level	Evidence at protein level	Tissue enhanced	Detected in many	
Developmental protein	Tumor suppressor	Evidence at protein level	Evidence at protein level	Evidence at protein level	Evidence at protein level	Tissue enhanced	Detected in all	
Host cell receptor for virus entry, Receptor	Congenital muscular dystrophy, Disease variant, Dystroglycanopathy, Limb-girdle muscular c	Evidence at protein level	Evidence at protein level	Evidence at protein level	Evidence at protein level	Low tissue specificity	Detected in all	
Hydrolase		Evidence at protein level	Evidence at protein level	Evidence at protein level	Evidence at protein level	Low tissue specificity	Detected in all	
Actin-binding		Evidence at protein level	Evidence at protein level	Evidence at protein level	Evidence at protein level	Low tissue specificity	Detected in all	
Isomerase	Albinism, Disease variant	Evidence at protein level	Evidence at protein level	Evidence at protein level	Evidence at protein level	Tissue enriched	Detected in some	35
Kinase, Receptor, Transferase, Tyrosine-protein kinase		Evidence at protein level	Evidence at protein level	Evidence at protein level	Evidence at protein level	Low tissue specificity	Detected in many	

Kinase, Receptor, Transferase, Tyrosine-protein kinase	Cancer-related genes, Disease variant, Dwarfism, FDA approved drug targets	Evidence at protein level	Evidence at transcript level	Evidence at protein level	Evidence at protein level	Low tissue specificity	Detected in many	
Kinase, Transferase		Evidence at protein level	Evidence at protein level	Evidence at protein level	Evidence at protein level	Low tissue specificity	Detected in many	
	Cancer-related genes, Deafness, Neuropathy, Non-syndromic deafness	Evidence at protein level	Evidence at protein level	Evidence at protein level	Evidence at protein level	Tissue enhanced	Detected in some	
Developmental protein, Ligase		Evidence at protein level	Evidence at protein level	Evidence at protein level	Evidence at protein level	Low tissue specificity	Detected in all	
		Evidence at protein level	Evidence at protein level	Evidence at protein level	Evidence at protein level	Low tissue specificity	Detected in all	
	Disease variant, Intellectual disability	Evidence at protein level	Evidence at protein level	Evidence at protein level	Evidence at protein level	Group enriched	Detected in all	4
	Disease variant	Evidence at protein level	Evidence at protein level	Evidence at protein level	Evidence at protein level	Low tissue specificity	Detected in many	
Chaperone	Disease variant	Evidence at protein level	Evidence at protein level	Evidence at protein level	Evidence at protein level	Low tissue specificity	Detected in all	
Chaperone		Evidence at protein level	Evidence at protein level	Evidence at protein level	Evidence at protein level	Tissue enhanced	Detected in all	
Guanine-nucleotide releasing factor	Proto-oncogene	Evidence at protein level	Evidence at transcript level	Evidence at protein level	Evidence at protein level	Tissue enhanced	Detected in many	
Guanine-nucleotide releasing factor	Disease variant, Intellectual disability	Evidence at protein level	Evidence at protein level	Evidence at protein level	Evidence at protein level	Tissue enhanced	Detected in many	
		Evidence at protein level	Evidence at protein level	Evidence at protein level	Evidence at protein level	Tissue enhanced	Detected in many	
		Evidence at protein level	Evidence at protein level	Evidence at protein level	Evidence at protein level	Group enriched	Detected in many	4
Dipeptidase, Hydrolase, Metalloprotease, Protease	FDA approved drug targets	Evidence at protein level	Evidence at protein level	Evidence at protein level	Evidence at protein level	Group enriched	Detected in many	8
G-protein coupled receptor, Receptor, Transducer	FDA approved drug targets	Evidence at protein level	Evidence at transcript level	Evidence at protein level	Evidence at protein level	Low tissue specificity	Detected in many	
	Cardiomyopathy, Disease variant	Evidence at protein level	Evidence at protein level	Evidence at protein level	Evidence at protein level	Tissue enhanced	Detected in many	
	Hypotrichosis	Evidence at protein level	Evidence at protein level	Evidence at protein level	Evidence at protein level	Group enriched	Detected in many	5
	Cardiomyopathy, Disease variant	Evidence at protein level	Evidence at protein level	Evidence at protein level	Evidence at protein level	Tissue enhanced	Detected in many	
	Cardiomyopathy, Disease variant, Epidermolysis bullosa, Palmoplantar keratoderma	Evidence at protein level	Evidence at protein level	Evidence at protein level	Evidence at protein level	Tissue enhanced	Detected in many	
	Cardiomyopathy	Evidence at protein level	Evidence at protein level	Evidence at protein level	Evidence at protein level	Group enriched	Detected in many	4
	Disease variant, Limb-girdle muscular dystrophy	Evidence at protein level	Evidence at protein level	Evidence at protein level	Evidence at protein level	Tissue enhanced	Detected in all	
Developmental protein		Evidence at protein level	Evidence at protein level	Evidence at protein level	Evidence at protein level	Low tissue specificity	Detected in many	
G-protein coupled receptor, Receptor, Transducer	Disease variant, FDA approved drug targets, Hypotrichosis	Evidence at protein level	Evidence at protein level	Evidence at protein level	Evidence at protein level	Tissue enhanced	Detected in many	
G-protein coupled receptor, Receptor, Transducer	Albinism, Deafness, Disease variant, FDA approved drug targets, Hirschsprung disease, Wa	Evidence at protein level	Evidence at protein level	Evidence at protein level	Evidence at protein level	Tissue enhanced	Detected in many	
Developmental protein	Cancer-related genes, Craniosynostosis, Disease variant	Evidence at protein level	Evidence at protein level	Evidence at protein level	Evidence at protein level	Low tissue specificity	Detected in all	
	Autism, Autism spectrum disorder	Evidence at protein level	Evidence at protein level	Evidence at protein level	Evidence at protein level	Tissue enhanced	Detected in all	
		Evidence at protein level	Evidence at protein level	Evidence at protein level	Evidence at protein level	Tissue enhanced	Detected in many	
Developmental protein, Host cell receptor for virus entry, Kinase, Receptor, Transferase, Tyrosine-protein kinase	Cancer-related genes, Disease variant, FDA approved drug targets, Proto-oncogene	Evidence at protein level	Evidence at protein level	Evidence at protein level	Evidence at protein level	Tissue enhanced	Detected in many	
		Evidence at protein level	Evidence at protein level	Evidence at protein level	Evidence at protein level	Low tissue specificity	Detected in all	
		Evidence at protein level	Evidence at protein level	Evidence at protein level	Evidence at protein level	Tissue enhanced	Detected in all	
Hydrolase		Evidence at protein level	Evidence at protein level	Evidence at protein level	Evidence at protein level	Low tissue specificity	Detected in many	
		Evidence at protein level	Evidence at protein level	Evidence at protein level	Evidence at protein level	Tissue enhanced	Detected in all	
		Evidence at protein level	Evidence at protein level	Evidence at protein level	Evidence at protein level	Tissue enhanced	Detected in many	
Actin-binding		Evidence at protein level	Evidence at protein level	Evidence at protein level	Evidence at protein level	Low tissue specificity	Detected in many	
	Cancer-related genes, Disease variant	Evidence at protein level	Evidence at protein level	Evidence at protein level	Evidence at protein level	Tissue enhanced	Detected in all	
DNA-binding, Lyase, Repressor	Cancer-related genes	Evidence at protein level	Evidence at protein level	Evidence at protein level	Evidence at protein level	Low tissue specificity	Detected in all	
Lyase	Cancer-related genes	Evidence at protein level	Evidence at protein level	Evidence at protein level	Evidence at protein level	Group enriched	Detected in all	7
Lyase	Disease variant, Glycogen storage disease	Evidence at protein level	Evidence at protein level	Evidence at protein level	Evidence at protein level	Group enriched	Detected in all	27
Oxidoreductase		Evidence at protein level	Evidence at protein level	Evidence at protein level	Evidence at protein level	Low tissue specificity	Detected in many	
Oxidoreductase		Evidence at protein level	Evidence at transcript level	Evidence at protein level	Evidence at protein level	Low tissue specificity	Detected in all	
Actin-binding	Disease variant, Intellectual disability	Evidence at protein level	Evidence at protein level	Evidence at protein level	Evidence at protein level	Low tissue specificity	Detected in many	
Actin-binding		Evidence at protein level	Evidence at protein level	Evidence at protein level	Evidence at protein level	Tissue enhanced	Detected in all	
Actin-binding	Tumor suppressor	Evidence at protein level	Evidence at protein level	Evidence at protein level	Evidence at protein level	Low tissue specificity	Detected in all	
		Evidence at protein level	Evidence at protein level	Evidence at protein level	Evidence at protein level	Low tissue specificity	Detected in all	
Tumor antigen	Cancer-related genes, Disease variant, FDA approved drug targets, Hereditary nonpolyposis	Evidence at protein level	Evidence at protein level	Evidence at protein level	Evidence at protein level	Tissue enhanced	Detected in many	
Host cell receptor for virus entry, Kinase, Receptor, Transferase, Tyrosine-protein kinase	Cancer-related genes, Cataract, Disease variant, FDA approved drug targets	Evidence at protein level	Evidence at protein level	Evidence at protein level	Evidence at protein level	Tissue enhanced	Detected in many	
Kinase, Receptor, Transferase, Tyrosine-protein kinase	Cancer-related genes	Evidence at protein level	Evidence at protein level	Evidence at protein level	Evidence at protein level	Tissue enhanced	Detected in many	
Kinase, Receptor, Transferase, Tyrosine-protein kinase		Evidence at protein level	Evidence at transcript level	Evidence at protein level	Evidence at protein level	Tissue enriched	Detected in some	6
Kinase, Receptor, Transferase, Tyrosine-protein kinase		Evidence at protein level	Evidence at protein level	Evidence at protein level	Evidence at protein level	Tissue enhanced	Detected in many	
Developmental protein, Kinase, Receptor, Transferase, Tyrosine-protein kinase	Cancer-related genes, Disease variant, Tumor suppressor	Evidence at protein level	Evidence at protein level	Evidence at protein level	Evidence at protein level	Tissue enhanced	Detected in many	
Receptor	Cancer-related genes, Congenital erythrocytosis, Disease variant, FDA approved drug target	Evidence at protein level	Evidence at protein level	Evidence at protein level	Evidence at protein level	Low tissue specificity	Detected in all	
Activator, Kinase, Receptor, Transferase, Tyrosine-protein kinase	Cancer-related genes, Disease variant, FDA approved drug targets	Evidence at protein level	Evidence at protein level	Evidence at protein level	Evidence at protein level	Low tissue specificity	Detected in all	
Kinase, Receptor, Transferase, Tyrosine-protein kinase	Cancer-related genes, Disease variant	Evidence at protein level	Evidence at protein level	Evidence at protein level	Evidence at protein level	Low tissue specificity	Detected in many	
		Evidence at protein level	Evidence at protein level	Evidence at protein level	Evidence at protein level	Low tissue specificity	Detected in all	
		Evidence at protein level	Evidence at transcript level	Evidence at protein level	Evidence at protein level	Low tissue specificity	Detected in all	
	Proto-oncogene	Evidence at protein level	Evidence at protein level	Evidence at protein level	Evidence at protein level	Group enriched	Detected in many	4
	Cancer-related genes	Evidence at protein level	Evidence at protein level	Evidence at protein level	Evidence at protein level	Low tissue specificity	Detected in all	
Host cell receptor for virus entry, Receptor		Evidence at protein level	Evidence at protein level	Evidence at protein level	Evidence at protein level	Low tissue specificity	Detected in all	
G-protein coupled receptor, Receptor, Transducer		Evidence at protein level	Evidence at protein level	Evidence at protein level	Evidence at protein level	Tissue enhanced	Detected in many	
Calmodulin-binding, Receptor	Cancer-related genes, Disease variant	Evidence at protein level	Evidence at protein level	Evidence at protein level	Evidence at protein level	Low tissue specificity	Detected in many	
Hydrolase, Lyase, Multifunctional enzyme, Oxidoreductase, Transferase	Cancer-related genes	Evidence at protein level	Evidence at protein level	Evidence at protein level	Evidence at protein level	Tissue enhanced	Detected in all	
		Evidence at protein level	Evidence at protein level	Evidence at protein level	Evidence at protein level	Tissue enriched	Detected in many	5
Calmodulin-binding		Evidence at protein level	Evidence at transcript level	Evidence at protein level	Evidence at protein level	Low tissue specificity	Detected in many	
IgA-binding protein, Receptor		Evidence at protein level	Evidence at protein level	Evidence at protein level	Evidence at protein level	Tissue enhanced	Detected in some	
IgG-binding protein, Receptor	FDA approved drug targets	Evidence at protein level	Evidence at transcript level	Evidence at protein level	Evidence at protein level	Tissue enhanced	Detected in many	
IgG-binding protein, Receptor	FDA approved drug targets	Evidence at protein level	Evidence at protein level	Evidence at protein level	Evidence at protein level	Tissue enhanced	Detected in all	
		Evidence at protein level	Evidence at protein level	Evidence at protein level	Evidence at protein level	Low tissue specificity	Detected in all	
Receptor		Evidence at protein level	Evidence at transcript level	Evidence at protein level	Evidence at protein level	Group enriched	Detected in many	6
G-protein coupled receptor, Receptor, Transducer	FDA approved drug targets	Evidence at protein level	Evidence at protein level	Evidence at protein level	Evidence at protein level	Group enriched	Detected in some	6
G-protein coupled receptor, Receptor, Transducer	FDA approved drug targets	Evidence at protein level	Evidence at protein level	Evidence at protein level	Evidence at protein level	Group enriched	Detected in some	5
Guanine-nucleotide releasing factor	Disease variant	Evidence at protein level	Evidence at protein level	Evidence at protein level	Evidence at protein level	Low tissue specificity	Detected in many	
Guanine-nucleotide releasing factor		Evidence at protein level	Evidence at protein level	Evidence at protein level	Evidence at protein level	Low tissue specificity	Detected in many	
Developmental protein	Disease variant, Emery-Dreifuss muscular dystrophy	Evidence at protein level	Evidence at protein level	Evidence at protein level	Evidence at protein level	Group enriched	Detected in all	7
Actin-binding, Developmental protein	Disease variant, Dwarfism	Evidence at protein level	Evidence at protein level	Evidence at protein level	Evidence at protein level	Low tissue specificity	Detected in all	
		Evidence at protein level	Evidence at protein level	Evidence at protein level	Evidence at protein level	Low tissue specificity	Detected in all	
		Evidence at protein level	Evidence at protein level	Evidence at protein level	Evidence at protein level	Low tissue specificity	Detected in all	
Developmental protein	Disease variant, Hypogonadotropic hypogonadism, Kallmann syndrome	Evidence at protein level	Evidence at protein level	Evidence at protein level	Evidence at protein level	Tissue enhanced	Detected in many	
Developmental protein, Kinase, Receptor, Transferase, Tyrosine-protein kinase	Cancer-related genes, FDA approved drug targets	Evidence at protein level	Evidence at protein level	Evidence at protein level	Evidence at protein level	Tissue enriched	Detected in many	9
Kinase, Receptor, Transferase, Tyrosine-protein kinase	Cancer-related genes, Disease variant, FDA approved drug targets	Evidence at protein level	Evidence at protein level	Evidence at protein level	Evidence at protein level	Low tissue specificity	Detected in many	
Developmental protein		Evidence at protein level	Evidence at protein level	Evidence at protein level	Evidence at protein level	Low tissue specificity	Detected in all	
		Evidence at protein level	Evidence at protein level	Evidence at protein level	Evidence at protein level	Tissue enhanced	Detected in all	
G-protein coupled receptor, Receptor, Transducer		Evidence at protein level	Evidence at transcript level	Evidence at transcript lev	Evidence at protein level	Tissue enhanced	Detected in many	
		Evidence at protein level	Evidence at transcript level	Evidence at protein level	Evidence at protein level	Low tissue specificity	Detected in all	
Developmental protein, Kinase, Transferase, Tyrosine-protein kinase	Proto-oncogene	Evidence at protein level	Evidence at protein level	Evidence at protein level	Evidence at protein level	Low tissue specificity	Detected in all	
Developmental protein, G-protein coupled receptor, Receptor, Transducer	Cancer-related genes	Evidence at protein level	Evidence at protein level	Evidence at protein level	Evidence at protein level	Low tissue specificity	Detected in all	
Developmental protein, G-protein coupled receptor, Receptor, Transducer	Disease variant	Evidence at protein level	Evidence at protein level	Evidence at protein level	Evidence at protein level	Tissue enhanced	Detected in many	
Developmental protein, G-protein coupled receptor, Receptor, Transducer	Disease variant	Evidence at protein level	Evidence at protein level	Evidence at protein level	Evidence at protein level	Low tissue specificity	Detected in many	
		Evidence at protein level	Evidence at protein level	Evidence at protein level	Evidence at protein level	Tissue enhanced	Detected in all	
G-protein coupled receptor, Receptor, Transducer	Disease variant, FDA approved drug targets, Intellectual disability	Evidence at protein level	Evidence at transcript level	Evidence at protein level	Evidence at protein level	Tissue enriched	Detected in many	5
Chloride channel, Ion channel, Ligand-gated ion channel, Receptor	Disease variant, Epilepsy, FDA approved drug targets	Evidence at protein level	Evidence at protein level	Evidence at protein level	Evidence at protein level	Group enriched	Detected in some	30
Chloride channel, Ion channel, Ligand-gated ion channel, Receptor	Disease variant, Epilepsy, FDA approved drug targets	Evidence at protein level	Evidence at protein level	Evidence at protein level	Evidence at protein level	Tissue enriched	Detected in some	14
Chloride channel, Ion channel	FDA approved drug targets	Evidence at protein level	Evidence at protein level	Evidence at protein level	Evidence at protein level	Tissue enriched	Detected in some	7
Chloride channel, Ion channel	FDA approved drug targets	Evidence at protein level	Evidence at transcript level	Evidence at transcript lev	Evidence at protein level	Tissue enhanced	Detected in some	
G-protein coupled receptor, Receptor, Transducer		Evidence at protein level	Evidence at protein level	Evidence at protein level	Evidence at protein level	Tissue enhanced	Detected in some	
Calmodulin-binding, Developmental protein		Evidence at protein level	Evidence at protein level	Evidence at protein level	Evidence at protein level	Tissue enriched	Detected in many	36
Oxidoreductase, Transferase	FDA approved drug targets	Evidence at protein level	Evidence at protein level	Evidence at protein level	Evidence at protein level	Group enriched	Detected in all	4
		Evidence at protein level	Evidence at protein level	Evidence at protein level	Evidence at protein level	Group enriched	Detected in many	4
GTPase activation, Guanine-nucleotide releasing factor		Evidence at protein level	Evidence at protein level	Evidence at protein level	Evidence at protein level	Low tissue specificity	Detected in all	
	Ciliopathy, Primary ciliary dyskinesia	Evidence at protein level	Evidence at protein level	Evidence at protein level	Evidence at protein level	Low tissue specificity	Detected in all	
Receptor		Evidence at protein level	Evidence at protein level	Evidence at protein level	Evidence at protein level	Tissue enhanced	Detected in many	
Hormone, Receptor		Evidence at protein level	Evidence at transcript level	Evidence at protein level	Evidence at protein level	Not detected	Not detected	
Receptor	Disease variant, Dwarfism, FDA approved drug targets	Evidence at protein level	Evidence at protein level	Evidence at protein level	Evidence at protein level	Tissue enhanced	Detected in many	
		Evidence at protein level	Evidence at protein level	Evidence at protein level	Evidence at protein level	Low tissue specificity	Detected in all	
	Cancer-related genes, Cataract, Disease variant, Hypotrichosis, Palmoplantar keratoderma	Evidence at protein level	Evidence at protein level	Evidence at protein level	Evidence at protein level	Low tissue specificity	Detected in many	
	Deafness, Disease variant, Non-syndromic deafness, Palmoplantar keratoderma	Evidence at protein level	Evidence at protein level	Evidence at protein level	Evidence at protein level	Group enriched	Detected in many	4
	Disease variant, Palmoplantar keratoderma	Evidence at protein level	Evidence at protein level	Evidence at protein level	Evidence at protein level	Tissue enriched	Detected in some	8
	Deafness, Disease variant, Ectodermal dysplasia, Non-syndromic deafness, Palmoplantar ke	Evidence at protein level	Evidence at protein level	Evidence at protein level	Evidence at protein level	Tissue enhanced	Detected in many	
		Evidence at protein level	Evidence at protein level	Evidence at protein level	Evidence at protein level	Tissue enhanced	Detected in many	
	Disease variant, Hereditary spastic paraplegia, Leukodystrophy, Neurodegeneration	Evidence at protein level	Evidence at protein level	Evidence at protein level	Evidence at protein level	Tissue enriched	Detected in many	11
Developmental protein	Disease variant	Evidence at protein level	Evidence at protein level	Evidence at protein level	Evidence at protein level	Tissue enhanced	Detected in many	
Lyase	Cancer-related genes	Evidence at protein level	Evidence at protein level	Evidence at protein level	Evidence at protein level	Low tissue specificity	Detected in all	
G-protein coupled receptor, Receptor, Transducer	FDA approved drug targets	Evidence at protein level	Evidence at transcript level	Evidence at protein level	Evidence at protein level	Tissue enhanced	Detected in some	
G-protein coupled receptor, Receptor, Transducer	FDA approved drug targets	Evidence at protein level	Evidence at protein level	Evidence at protein level	Evidence at protein level	Tissue enhanced	Detected in many	
Chloride channel, Ion channel, Ligand-gated ion channel, Receptor	Disease variant, FDA approved drug targets	Evidence at protein level	Evidence at transcript level	Evidence at protein level	Evidence at protein level	Tissue enriched	Detected in single	7
Chloride channel, Ion channel, Ligand-gated ion channel, Receptor	Disease variant, FDA approved drug targets, Intellectual disability	Evidence at protein level	Evidence at transcript level	Evidence at protein level	Evidence at protein level	Tissue enhanced	Detected in some	
Chloride channel, Ion channel, Ligand-gated ion channel, Receptor	FDA approved drug targets	Evidence at protein level	Evidence at transcript level	Evidence at protein level	Evidence at protein level	Group enriched	Detected in some	5
Transducer		Evidence at protein level	Evidence at protein level	Evidence at protein level	Evidence at protein level	Low tissue specificity	Detected in all	
Hydrolase, Transducer	Cancer-related genes, Disease variant	Evidence at protein level	Evidence at protein level	Evidence at protein level	Evidence at protein level	Low tissue specificity	Detected in all	
Hydrolase, Transducer	Cancer-related genes, Cushing syndrome, Disease variant, Dwarfism, Obesity, Proto-oncoge	Evidence at protein level	Evidence at transcript level	Evidence at protein level	Evidence at protein level	Low tissue specificity	Detected in all	
Transducer	Disease variant, Intellectual disability	Evidence at protein level	Evidence at protein level	Evidence at protein level	Evidence at protein level	Low tissue specificity	Detected in all	
Receptor	Disease variant	Evidence at protein level	Evidence at protein level	Evidence at protein level	Evidence at protein level	Tissue enhanced	Detected in some	
		Evidence at protein level	Evidence at protein level	Evidence at protein level	Evidence at protein level	Tissue enhanced	Detected in many	
Protease inhibitor	Cancer-related genes, Disease variant	Evidence at protein level	Evidence at protein level	Evidence at protein level	Evidence at protein level	Tissue enriched	Detected in many	5
G-protein coupled receptor, Receptor, Transducer	Albinism, Disease variant	Evidence at protein level	Evidence at protein level	Evidence at protein level	Evidence at protein level	Tissue enhanced	Detected in many	
G-protein coupled receptor, Receptor, Transducer		Evidence at protein level	Evidence at protein level	Evidence at protein level	Evidence at protein level	Not detected	Not detected	
G-protein coupled receptor, Receptor, Transducer	Deafness, Non-syndromic deafness	Evidence at protein level	Evidence at protein level	Evidence at transcript lev	Evidence at protein level	Tissue enhanced	Detected in single	
G-protein coupled receptor, Receptor, Transducer		Evidence at protein level	Evidence at protein level	Evidence at protein level	Evidence at protein level	Tissue enhanced	Detected in many	
G-protein coupled receptor, Receptor, Transducer		Evidence at protein level	Evidence at protein level	Evidence at transcript lev	Evidence at protein level	Tissue enriched	Detected in some	5
G-protein coupled receptor, Receptor, Transducer		Evidence at protein level	Evidence at protein level	Evidence at protein level	Evidence at protein level	Tissue enhanced	Detected in all	

G-protein coupled receptor, Receptor, Transducer		Evidence at protein level	Evidence at protein level	Evidence at protein level	Evidence at protein level	Tissue enhanced	Detected in some	
G-protein coupled receptor, Receptor, Transducer		Evidence at protein level	Evidence at protein level	Evidence at protein level	Evidence at protein level	Tissue enriched	Detected in some	9
G-protein coupled receptor, Receptor, Transducer		Evidence at protein level	Evidence at protein level	Evidence at protein level	Evidence at protein level	Tissue enriched	Detected in single	16
G-protein coupled receptor, Receptor, Transducer		Evidence at protein level	Evidence at protein level	Evidence at protein level	Evidence at protein level	Tissue enhanced	Detected in many	
G-protein coupled receptor, Receptor, Transducer	Tumor suppressor	Evidence at protein level	Evidence at protein level	Evidence at protein level	Evidence at protein level	Tissue enhanced	Detected in many	
G-protein coupled receptor, Receptor, Transducer		Evidence at protein level	Evidence at protein level	Evidence at protein level	Evidence at protein level	Tissue enhanced	Detected in many	
		Evidence at protein level	Evidence at protein level	Evidence at protein level	Evidence at protein level	Tissue enhanced	Detected in some	
		Evidence at protein level	Evidence at protein level	Evidence at protein level	Evidence at protein level	Low tissue specificity	Detected in all	
		Evidence at protein level	Evidence at protein level	Evidence at protein level	Evidence at protein level	Tissue enhanced	Detected in many	
Repressor, RNA-binding	Cancer-related genes	Evidence at protein level	Evidence at protein level	Evidence at protein level	Evidence at protein level	Tissue enhanced	Detected in many	
Ion channel, Ligand-gated ion channel, Receptor	FDA approved drug targets	Evidence at protein level	Evidence at protein level	Evidence at protein level	Evidence at protein level	Group enriched	Detected in some	8
Ion channel, Ligand-gated ion channel, Receptor	FDA approved drug targets	Evidence at protein level	Evidence at protein level	Evidence at transcript lev	Evidence at protein level	Tissue enhanced	Detected in many	
Ion channel, Ligand-gated ion channel, Receptor	Disease variant, Epilepsy, FDA approved drug targets, Intellectual disability	Evidence at protein level	Evidence at protein level	Evidence at protein level	Evidence at protein level	Tissue enriched	Detected in single	17
Kinase, Serine/threonine-protein kinase, Transferase		Evidence at protein level	Evidence at protein level	Evidence at protein level	Evidence at protein level	Low tissue specificity	Detected in many	
G-protein coupled receptor, Receptor, Transducer		Evidence at protein level	Evidence at protein level	Evidence at transcript lev	Evidence at protein level	Tissue enriched	Detected in some	10
		Evidence at protein level	Evidence at protein level	Evidence at protein level	Evidence at protein level	Tissue enriched	Detected in some	9
		Evidence at protein level	Evidence at protein level	Evidence at protein level	Evidence at protein level	Tissue enhanced	Detected in some	
Blood group antigen, Host cell receptor for virus entry, Receptor		Evidence at protein level	Evidence at protein level	Evidence at protein level	Evidence at protein level	Tissue enriched	Detected in some	38
Blood group antigen		Evidence at protein level	Evidence at protein level	Evidence at protein level	Evidence at protein level	Tissue enhanced	Detected in all	
Hydrolase, Nuclease		Evidence at protein level	Evidence at protein level	Evidence at protein level	Evidence at protein level	Tissue enhanced	Detected in all	
Glycosyltransferase, Transferase		Evidence at protein level	Evidence at protein level	Evidence at protein level	Evidence at protein level	Tissue enhanced	Detected in many	
G-protein coupled receptor, Receptor, Transducer	FDA approved drug targets	Evidence at protein level	Evidence at protein level	Evidence at protein level	Evidence at protein level	Tissue enhanced	Detected in many	
Kinase, Transferase, Tyrosine-protein kinase	Proto-oncogene	Evidence at protein level	Evidence at protein level	Evidence at protein level	Evidence at protein level	Tissue enhanced	Detected in many	
		Evidence at protein level	Evidence at protein level	Evidence at protein level	Evidence at protein level	Tissue enhanced	Detected in all	
G-protein coupled receptor, Receptor, Transducer	FDA approved drug targets	Evidence at protein level	Evidence at protein level	Evidence at protein level	Evidence at protein level	Not detected	Not detected	
	Disease variant	Evidence at protein level	Evidence at protein level	Evidence at protein level	Evidence at protein level	Low tissue specificity	Detected in many	
	Cancer-related genes	Evidence at protein level	Evidence at protein level	Evidence at protein level	Evidence at protein level	Low tissue specificity	Detected in many	
		Evidence at protein level	Evidence at protein level	Evidence at protein level	Evidence at protein level	Tissue enhanced	Detected in many	
		Evidence at protein level	Evidence at protein level	Evidence at protein level	Evidence at protein level	Low tissue specificity	Detected in many	
Transferase		Evidence at protein level	Evidence at protein level	Evidence at protein level	Evidence at protein level	Tissue enhanced	Detected in all	
		Evidence at protein level	Evidence at protein level	Evidence at protein level	Evidence at protein level	Low tissue specificity	Detected in all	
G-protein coupled receptor, Receptor, Transducer	FDA approved drug targets	Evidence at protein level	Evidence at protein level	Evidence at protein level	Evidence at protein level	Low tissue specificity	Detected in many	
Chaperone	Cancer-related genes	Evidence at protein level	Evidence at protein level	Evidence at protein level	Evidence at protein level	Low tissue specificity	Detected in all	
Chaperone	Cancer-related genes, Charcot-Marie-Tooth disease, Disease variant, Neurodegeneration, Ne	Evidence at protein level	Evidence at protein level	Evidence at protein level	Evidence at protein level	Tissue enhanced	Detected in all	
G-protein coupled receptor, Receptor, Transducer	FDA approved drug targets	Evidence at protein level	Evidence at protein level	Evidence at protein level	Evidence at protein level	Group enriched	Detected in some	5
Growth factor binding, Hydrolase, Protease, Serine protease	Age-related macular degeneration, Disease variant	Evidence at protein level	Evidence at protein level	Evidence at protein level	Evidence at protein level	Tissue enhanced	Detected in many	
	Cataract, Disease variant, Leukodystrophy	Evidence at protein level	Evidence at protein level	Evidence at protein level	Evidence at protein level	Low tissue specificity	Detected in all	
		Evidence at protein level	Evidence at protein level	Evidence at protein level	Evidence at protein level	Low tissue specificity	Detected in all	
		Evidence at protein level	Evidence at protein level	Evidence at protein level	Evidence at protein level	Low tissue specificity	Detected in many	
RNA-binding, tRNA-binding		Evidence at protein level	Evidence at protein level	Evidence at protein level	Evidence at protein level	Low tissue specificity	Detected in all	
		Evidence at protein level	Evidence at protein level	Evidence at protein level	Evidence at protein level	Low tissue specificity	Detected in all	
Developmental protein	Disease variant, Osteogenesis imperfecta	Evidence at protein level	Evidence at protein level	Evidence at protein level	Evidence at protein level	Tissue enriched	Detected in single	10
Receptor	Disease variant, FDA approved drug targets	Evidence at protein level	Evidence at protein level	Evidence at protein level	Evidence at protein level	Low tissue specificity	Detected in all	
Kinase, Receptor, Transferase, Tyrosine-protein kinase	Cancer-related genes, Disease variant, FDA approved drug targets	Evidence at protein level	Evidence at protein level	Evidence at protein level	Evidence at protein level	Low tissue specificity	Detected in many	
Receptor		Evidence at protein level	Evidence at transcript level	Evidence at protein level	Evidence at protein level	Tissue enhanced	Detected in all	
Receptor	Disease variant	Evidence at protein level	Evidence at protein level	Evidence at protein level	Evidence at protein level	Tissue enhanced	Detected in many	
Receptor		Evidence at protein level	Evidence at protein level	Evidence at protein level	Evidence at protein level	Low tissue specificity	Detected in many	
Receptor		Evidence at protein level	Evidence at protein level	Evidence at protein level	Evidence at protein level	Group enriched	Detected in many	4
Hydrolase, Receptor		Evidence at protein level	Evidence at protein level	Evidence at protein level	Evidence at protein level	Group enriched	Detected in many	4
Receptor	Cancer-related genes, Disease variant, FDA approved drug targets, SCID	Evidence at protein level	Evidence at protein level	Evidence at protein level	Evidence at protein level	Tissue enhanced	Detected in many	
Receptor	Cancer-related genes, FDA approved drug targets	Evidence at protein level	Evidence at protein level	Evidence at protein level	Evidence at protein level	Low tissue specificity	Detected in all	
Receptor	Cancer-related genes, Disease variant	Evidence at protein level	Evidence at protein level	Evidence at protein level	Evidence at protein level	Low tissue specificity	Detected in all	
Receptor	Cancer-related genes, Disease variant, SCID	Evidence at protein level	Evidence at protein level	Evidence at protein level	Evidence at protein level	Tissue enhanced	Detected in many	
Calmodulin-binding		Evidence at protein level	Evidence at protein level	Evidence at protein level	Evidence at protein level	Low tissue specificity	Detected in all	
Activator, DNA-binding, Repressor		Evidence at protein level	Evidence at protein level	Evidence at protein level	Evidence at protein level	Low tissue specificity	Detected in all	
Integrin, Receptor	Cancer-related genes, Disease variant, Epidermolysis bullosa	Evidence at protein level	Evidence at protein level	Evidence at protein level	Evidence at protein level	Low tissue specificity	Detected in many	
Integrin, Receptor	FDA approved drug targets	Evidence at protein level	Evidence at protein level	Evidence at protein level	Evidence at protein level	Tissue enhanced	Detected in many	
Host cell receptor for virus entry, Integrin, Receptor	Cancer-related genes, FDA approved drug targets	Evidence at protein level	Evidence at protein level	Evidence at protein level	Evidence at protein level	Low tissue specificity	Detected in all	
Integrin, Receptor	Disease variant	Evidence at protein level	Evidence at protein level	Evidence at protein level	Evidence at protein level	Tissue enhanced	Detected in all	
Host cell receptor for virus entry, Integrin, Receptor	Cancer-related genes, Disease variant, FDA approved drug targets	Evidence at protein level	Evidence at protein level	Evidence at protein level	Evidence at protein level	Tissue enhanced	Detected in many	
Integrin, Receptor	Cancer-related genes, Disease variant, Epidermolysis bullosa	Evidence at protein level	Evidence at protein level	Evidence at protein level	Evidence at protein level	Tissue enhanced	Detected in all	
		Evidence at protein level	Evidence at protein level	Evidence at protein level	Evidence at protein level	Low tissue specificity	Detected in all	
Oxidoreductase	Congenital hypothyroidism, Disease variant	Evidence at protein level	Evidence at protein level	Evidence at protein level	Evidence at protein level	Tissue enriched	Detected in some	14
	Disease variant	Evidence at protein level	Evidence at protein level	Evidence at protein level	Evidence at protein level	Tissue enhanced	Detected in many	
		Evidence at protein level	Evidence at protein level	Evidence at protein level	Evidence at protein level	Tissue enhanced	Detected in many	
	Cancer-related genes, Cardiomyopathy, Disease variant, Palmoplantar keratoderma	Evidence at protein level	Evidence at protein level	Evidence at protein level	Evidence at protein level	Tissue enhanced	Detected in all	
	Tumor suppressor	Evidence at protein level	Evidence at protein level	Evidence at protein level	Evidence at protein level	Low tissue specificity	Detected in all	
Chromatin regulator		Evidence at protein level	Evidence at protein level	Evidence at protein level	Evidence at protein level	Low tissue specificity	Detected in all	
Aminoacyl-tRNA synthetase, Ligase, Transferase	Charcot-Marie-Tooth disease, Deafness, Disease variant, Intellectual disability, Neurodegene	Evidence at protein level	Evidence at protein level	Evidence at protein level	Evidence at protein level	Low tissue specificity	Detected in all	
Ion channel, Potassium channel, Voltage-gated channel	FDA approved drug targets	Evidence at protein level	Evidence at transcript level	Evidence at protein level	Evidence at protein level	Tissue enriched	Detected in some	7
Oxidoreductase		Evidence at protein level	Evidence at protein level	Evidence at protein level	Evidence at protein level	Tissue enhanced	Detected in all	
	Brugada syndrome, Disease variant	Evidence at protein level	Evidence at protein level	Evidence at protein level	Evidence at protein level	Tissue enhanced	Detected in all	
Ion channel, Potassium channel, Voltage-gated channel		Evidence at protein level	Evidence at protein level	Evidence at protein level	Evidence at protein level	Tissue enriched	Detected in single	6
Ion channel, Potassium channel, Voltage-gated channel		Evidence at protein level	Evidence at protein level	Evidence at transcript lev	Evidence at protein level	Group enriched	Detected in some	5
Ion channel, Potassium channel, Voltage-gated channel		Evidence at protein level	Evidence at transcript level	Evidence at transcript lev	Evidence at protein level	Tissue enhanced	Detected in many	
Calmodulin-binding, Ion channel	Disease variant, FDA approved drug targets, Hereditary hemolytic anemia	Evidence at protein level	Evidence at transcript level	Evidence at protein level	Evidence at protein level	Tissue enhanced	Detected in many	
Calmodulin-binding, Ion channel, Potassium channel, Voltage-gated channel	Atrial fibrillation, Deafness, Diabetes mellitus, Disease variant, FDA approved drug targets, Lo	Evidence at protein level	Evidence at protein level	Evidence at protein level	Evidence at protein level	Tissue enhanced	Detected in many	
Ion channel, Potassium channel, Voltage-gated channel		Evidence at protein level	Evidence at transcript level	Evidence at protein level	Evidence at protein level	Tissue enriched	Detected in some	9
Ion channel, Potassium channel, Voltage-gated channel		Evidence at protein level	Evidence at protein level	Evidence at protein level	Evidence at protein level	Tissue enhanced	Detected in many	
Ion channel, Potassium channel	Disease variant, Epilepsy	Evidence at protein level	Evidence at protein level	Evidence at protein level	Evidence at protein level	Tissue enhanced	Detected in some	
Ion channel, Potassium channel, Voltage-gated channel		Evidence at protein level	Evidence at protein level	Evidence at protein level	Evidence at protein level	Tissue enriched	Detected in single	26
Blood group antigen, Hydrolase, Metalloprotease, Protease	Cancer-related genes	Evidence at protein level	Evidence at protein level	Evidence at protein level	Evidence at protein level	Group enriched	Detected in some	5
		Evidence at protein level	Evidence at protein level	Evidence at protein level	Evidence at protein level	Low tissue specificity	Detected in all	
G-protein coupled receptor, Receptor, Transducer	Disease variant, Hypogonadotropic hypogonadism	Evidence at protein level	Evidence at protein level	Evidence at protein level	Evidence at protein level	Group enriched	Detected in some	4
Kinase, Receptor, Transferase, Tyrosine-protein kinase	Cancer-related genes, Disease variant, FDA approved drug targets, Proto-oncogene	Evidence at protein level	Evidence at protein level	Evidence at protein level	Evidence at protein level	Tissue enhanced	Detected in many	
Motor protein	Hereditary spastic paraplegia, Neurodegeneration, Neuropathy	Evidence at protein level	Evidence at protein level	Evidence at protein level	Evidence at protein level	Tissue enhanced	Detected in all	
Hydrolase, Protease, Serine protease	Cancer-related genes	Evidence at protein level	Evidence at protein level	Evidence at protein level	Evidence at protein level	Tissue enhanced	Detected in many	
Receptor	Cancer-related genes	Evidence at protein level	Evidence at protein level	Evidence at protein level	Evidence at protein level	Tissue enhanced	Detected in many	
Developmental protein	Disease variant, Hereditary spastic paraplegia, Hirschsprung disease, Intellectual disability, N	Evidence at protein level	Evidence at protein level	Evidence at protein level	Evidence at protein level	Tissue enhanced	Detected in many	
Receptor		Evidence at protein level	Evidence at protein level	Evidence at protein level	Evidence at protein level	Tissue enhanced	Detected in many	
	Disease variant, Glycogen storage disease	Evidence at protein level	Evidence at protein level	Evidence at protein level	Evidence at protein level	Low tissue specificity	Detected in all	
		Evidence at protein level	Evidence at protein level	Evidence at protein level	Evidence at protein level	Low tissue specificity	Detected in all	
		Evidence at protein level	Evidence at protein level	Evidence at protein level	Evidence at protein level	Tissue enhanced	Detected in all	
	Williams-Beuren syndrome	Evidence at protein level	Evidence at protein level	Evidence at protein level	Evidence at protein level	Tissue enhanced	Detected in all	
		Evidence at protein level	Evidence at protein level	Evidence at protein level	Evidence at protein level	Tissue enriched	Detected in some	4
Actin-binding	Cancer-related genes, Disease variant	Evidence at protein level	Evidence at protein level	Evidence at protein level	Evidence at protein level	Tissue enhanced	Detected in all	
Receptor	Cancer-related genes, FDA approved drug targets, Obesity	Evidence at protein level	Evidence at transcript level	Evidence at protein level	Evidence at protein level	Tissue enriched	Detected in all	4
G-protein coupled receptor, Receptor, Transducer	Disease variant, FDA approved drug targets	Evidence at protein level	Evidence at protein level	Evidence at protein level	Evidence at protein level	Tissue enhanced	Detected in some	
	Disease variant, Intellectual disability	Evidence at protein level	Evidence at protein level	Evidence at protein level	Evidence at protein level	Tissue enriched	Detected in many	7
Heparin-binding, Hydrolase		Evidence at protein level	Evidence at protein level	Evidence at protein level	Evidence at protein level	Tissue enriched	Detected in some	169
DNA-binding	Charcot-Marie-Tooth disease, Disease variant, Neurodegeneration, Neuropathy	Evidence at protein level	Evidence at protein level	Evidence at protein level	Evidence at protein level	Low tissue specificity	Detected in all	
Transferase		Evidence at protein level	Evidence at protein level	Evidence at protein level	Evidence at protein level	Low tissue specificity	Detected in many	
G-protein coupled receptor, Receptor, Transducer		Evidence at protein level	Evidence at protein level	Evidence at protein level	Evidence at protein level	Tissue enhanced	Detected in many	
G-protein coupled receptor, Receptor, Transducer		Evidence at protein level	Evidence at protein level	Evidence at protein level	Evidence at protein level	Tissue enhanced	Detected in some	
G-protein coupled receptor, Receptor, Transducer	Disease variant, Hypotrichosis	Evidence at protein level	Evidence at protein level	Evidence at protein level	Evidence at protein level	Low tissue specificity	Detected in all	
Activator	Cancer-related genes	Evidence at protein level	Evidence at protein level	Evidence at protein level	Evidence at protein level	Tissue enhanced	Detected in all	
		Evidence at protein level	Evidence at protein level	Evidence at protein level	Evidence at protein level	Group enriched	Detected in some	5
Developmental protein, Receptor	Disease variant	Evidence at protein level	Evidence at protein level	Evidence at protein level	Evidence at protein level	Tissue enhanced	Detected in all	
Ion channel		Evidence at protein level	Evidence at protein level	Evidence at protein level	Evidence at protein level	Tissue enriched	Detected in some	6
Receptor		Evidence at protein level	Evidence at protein level	Evidence at protein level	Evidence at protein level	Tissue enhanced	Detected in all	
		Evidence at protein level	Evidence at protein level	Evidence at protein level	Evidence at protein level	Tissue enhanced	Detected in many	
G-protein coupled receptor, Receptor, Transducer		Evidence at protein level	Evidence at protein level	Evidence at transcript lev	Evidence at protein level	Tissue enhanced	Detected in many	
Kinase, Transferase, Tyrosine-protein kinase	Cancer-related genes, Disease variant, Proto-oncogene	Evidence at protein level	Evidence at protein level	Evidence at protein level	Evidence at protein level	Tissue enhanced	Detected in all	
Actin-binding	Disease variant, Lissencephaly	Evidence at protein level	Evidence at protein level	Evidence at protein level	Evidence at protein level	Low tissue specificity	Detected in all	
Guanine-nucleotide releasing factor	Intellectual disability	Evidence at protein level	Evidence at protein level	Evidence at protein level	Evidence at protein level	Tissue enhanced	Detected in all	
	Cancer-related genes	Evidence at protein level	Evidence at protein level	Evidence at protein level	Evidence at protein level	Low tissue specificity	Detected in many	
		Evidence at protein level	Evidence at protein level	Evidence at protein level	Evidence at protein level	Tissue enhanced	Detected in many	
Kinase, Serine/threonine-protein kinase, Transferase, Tyrosine-protein kinase	Cancer-related genes, Cardiomyopathy, Disease variant, Ectodermal dysplasia, FDA approve	Evidence at protein level	Evidence at protein level	Evidence at protein level	Evidence at protein level	Low tissue specificity	Detected in all	
Kinase, Serine/threonine-protein kinase, Transferase	Disease variant	Evidence at protein level	Evidence at protein level	Evidence at protein level	Evidence at protein level	Low tissue specificity	Detected in all	
	Cancer-related genes, Diabetes mellitus, Disease variant	Evidence at protein level	Evidence at protein level	Evidence at protein level	Evidence at protein level	Group enriched	Detected in all	5
	Alzheimer disease, Disease variant, FDA approved drug targets, Neurodegeneration, Parkins	Evidence at protein level	Evidence at protein level	Evidence at protein level	Evidence at protein level	Tissue enhanced	Detected in many	
Actin-binding, Calmodulin-binding		Evidence at protein level	Evidence at protein level	Evidence at protein level	Evidence at protein level	Low tissue specificity	Detected in all	
Developmental protein, Kinase, Serine/threonine-protein kinase, Transferase		Evidence at protein level	Evidence at protein level	Evidence at protein level	Evidence at protein level	Low tissue specificity	Detected in all	
	Deafness, Non-syndromic deafness	Evidence at protein level	Evidence at protein level	Evidence at protein level	Evidence at protein level	Tissue enhanced	Detected in many	
G-protein coupled receptor, Receptor, Transducer		Evidence at protein level	Evidence at protein level	Evidence at transcript lev	Evidence at transcript lev	Tissue enhanced	Detected in some	
G-protein coupled receptor, Receptor, Transducer		Evidence at protein level	Evidence at protein level	Evidence at protein level	Evidence at protein level	Tissue enhanced	Detected in some	
		Evidence at protein level	Evidence at protein level	Evidence at protein level	Evidence at protein level	Group enriched	Detected in all	4
		Evidence at protein level	Evidence at protein level	Evidence at protein level	Evidence at protein level	Group enriched	Detected in many	11
Guanine-nucleotide releasing factor	Proto-oncogene	Evidence at protein level	Evidence at protein level	Evidence at protein level	Evidence at protein level	Tissue enhanced	Detected in many	

G-protein coupled receptor, Receptor, Transducer		Evidence at protein level	Evidence at protein level	Evidence at protein level	Evidence at protein level	Group enriched	Detected in some	18
Ion channel	Corneal dystrophy, Disease variant, Mucolipidosis	Evidence at protein level	Evidence at protein level	Evidence at protein level	Evidence at protein level	Low tissue specificity	Detected in all	
Developmental protein		Evidence at protein level	Evidence at protein level	Evidence at protein level	Evidence at protein level	Tissue enriched	Detected in many	17
Actin-binding	Amyloidosis, Disease variant	Evidence at protein level	Evidence at protein level	Evidence at protein level	Evidence at protein level	Tissue enhanced	Detected in some	
	Disease variant	Evidence at protein level	Evidence at protein level	Evidence at protein level	Evidence at protein level	Tissue enhanced	Detected in some	
Kinase, Receptor, Transferase, Tyrosine-protein kinase	Disease variant, Proto-oncogene, Retinitis pigmentosa	Evidence at protein level	Evidence at protein level	Evidence at protein level	Evidence at protein level	Tissue enhanced	Detected in all	
		Evidence at protein level	Evidence at protein level	Evidence at protein level	Evidence at protein level	Tissue enhanced	Detected in many	
	Disease variant, Primary microcephaly	Evidence at protein level	Evidence at protein level	Evidence at protein level	Evidence at protein level	Tissue enhanced	Detected in many	
		Evidence at protein level	Evidence at protein level	Evidence at protein level	Evidence at protein level	Low tissue specificity	Detected in many	
		Evidence at protein level	Evidence at protein level	Evidence at protein level	Evidence at protein level	Low tissue specificity	Detected in all	
Actin-binding		Evidence at protein level	Evidence at protein level	Evidence at protein level	Evidence at protein level	Tissue enriched	Detected in many	9
		Evidence at protein level	Evidence at protein level	Evidence at protein level	Evidence at protein level	Low tissue specificity	Detected in many	
G-protein coupled receptor, Receptor, Transducer		Evidence at protein level	Evidence at transcript level	Evidence at transcript lev	Evidence at protein level	Tissue enhanced	Detected in some	
Hydrolase, Metalloprotease, Protease	Cancer-related genes, Charcot-Marie-Tooth disease, Disease variant, FDA approved drug tar	Evidence at protein level	Evidence at protein level	Evidence at protein level	Evidence at protein level	Tissue enhanced	Detected in many	
Hydrolase, Metalloprotease, Protease		Evidence at protein level	Evidence at protein level	Evidence at protein level	Evidence at protein level	Group enriched	Detected in many	4
Receptor	Cancer-related genes, Disease variant, FDA approved drug targets	Evidence at protein level	Evidence at protein level	Evidence at protein level	Evidence at protein level	Low tissue specificity	Detected in some	
		Evidence at protein level	Evidence at protein level	Evidence at protein level	Evidence at protein level	Tissue enhanced	Detected in all	
		Evidence at protein level	Evidence at protein level	Evidence at protein level	Evidence at protein level	Tissue enhanced	Detected in many	
		Evidence at protein level	Evidence at protein level	Evidence at protein level	Evidence at protein level	Low tissue specificity	Detected in all	
	FDA approved drug targets	Evidence at protein level	Evidence at protein level	Evidence at protein level	Evidence at protein level	Low tissue specificity	Detected in all	
G-protein coupled receptor, Receptor, Transducer		Evidence at protein level	Evidence at transcript level	Evidence at transcript lev	Evidence at protein level	Tissue enhanced	Detected in many	
	FDA approved drug targets	Evidence at protein level	Evidence at protein level	Evidence at protein level	Evidence at protein level	Tissue enriched	Detected in many	5
	Cancer-related genes	Evidence at protein level	Evidence at protein level	Evidence at protein level	Evidence at protein level	Tissue enhanced	Detected in many	
	Cancer-related genes, Disease variant	Evidence at protein level	Evidence at protein level	Evidence at protein level	Evidence at protein level	Low tissue specificity	Detected in all	
Hydrolase, Protein phosphatase	Disease variant	Evidence at protein level	Evidence at protein level	Evidence at protein level	Evidence at protein level	Low tissue specificity	Detected in all	
	Cancer-related genes, Tumor suppressor	Evidence at protein level	Evidence at protein level	Evidence at protein level	Evidence at protein level	Tissue enhanced	Detected in many	
Actin-binding, Calmodulin-binding, Motor protein, Muscle protein, Myosin	Aortic aneurysm, Cancer-related genes, Disease variant, Proto-oncogene	Evidence at protein level	Evidence at protein level	Evidence at protein level	Evidence at protein level	Tissue enhanced	Detected in many	
Actin-binding, Calmodulin-binding, Kinase, Serine/threonine-protein kinase, Transferase	Aortic aneurysm, Disease variant	Evidence at protein level	Evidence at protein level	Evidence at protein level	Evidence at protein level	Tissue enhanced	Detected in many	
Actin-binding, Calmodulin-binding, Motor protein, Myosin		Evidence at protein level	Evidence at protein level	Evidence at protein level	Evidence at protein level	Low tissue specificity	Detected in all	
Actin-binding, Calmodulin-binding, Motor protein, Myosin		Evidence at protein level	Evidence at protein level	Evidence at protein level	Evidence at protein level	Tissue enhanced	Detected in many	
Actin-binding, Calmodulin-binding, Motor protein, Myosin		Evidence at protein level	Evidence at protein level	Evidence at protein level	Evidence at protein level	Low tissue specificity	Detected in all	
Actin-binding, Calmodulin-binding, Motor protein, Myosin		Evidence at protein level	Evidence at protein level	Evidence at protein level	Evidence at protein level	Tissue enhanced	Detected in many	
		Evidence at protein level	Evidence at protein level	Evidence at protein level	Evidence at protein level	Low tissue specificity	Detected in many	
Ion channel, Sodium channel, Voltage-gated channel	Disease variant, Neurodegeneration	Evidence at protein level	Evidence at transcript level	Evidence at protein level	Evidence at protein level	Tissue enhanced	Detected in many	
		Evidence at protein level	Evidence at protein level	Evidence at protein level	Evidence at protein level	Tissue enriched	Detected in many	4
		Evidence at protein level	Evidence at protein level	Evidence at protein level	Evidence at protein level	Low tissue specificity	Detected in all	
Host cell receptor for virus entry, Receptor	Cancer-related genes	Evidence at protein level	Evidence at protein level	Evidence at protein level	Evidence at protein level	Tissue enhanced	Detected in many	
		Evidence at protein level	Evidence at protein level	Evidence at protein level	Evidence at protein level	Tissue enhanced	Detected in many	
	Disease variant	Evidence at protein level	Evidence at protein level	Evidence at protein level	Evidence at protein level	Tissue enhanced	Detected in many	
		Evidence at protein level	Evidence at protein level	Evidence at protein level	Evidence at protein level	Tissue enhanced	Detected in all	
Host cell receptor for virus entry, Receptor	Disease variant, Ectodermal dysplasia	Evidence at protein level	Evidence at protein level	Evidence at protein level	Evidence at protein level	Group enriched	Detected in many	4
	Cancer-related genes	Evidence at protein level	Evidence at protein level	Evidence at protein level	Evidence at protein level	Low tissue specificity	Detected in all	
Glycosidase, Hydrolase	Disease variant, FDA approved drug targets	Evidence at protein level	Evidence at protein level	Evidence at protein level	Evidence at protein level	Low tissue specificity	Detected in many	
GTPase activation	Cancer-related genes, Disease variant, Tumor suppressor	Evidence at protein level	Evidence at protein level	Evidence at protein level	Evidence at protein level	Low tissue specificity	Detected in all	
	Cancer-related genes, Deafness, Disease variant, Tumor suppressor	Evidence at protein level	Evidence at protein level	Evidence at protein level	Evidence at protein level	Low tissue specificity	Detected in all	
Developmental protein, Receptor	Cancer-related genes	Evidence at protein level	Evidence at protein level	Evidence at protein level	Evidence at protein level	Low tissue specificity	Detected in many	
	Cataract	Evidence at protein level	Evidence at protein level	Evidence at protein level	Evidence at protein level	Low tissue specificity	Detected in many	
		Evidence at protein level	Evidence at protein level	Evidence at protein level	Evidence at protein level	Tissue enhanced	Detected in all	
		Evidence at protein level	Evidence at protein level	Evidence at protein level	Evidence at protein level	Tissue enhanced	Detected in many	
Isomerase, Rotamase		Evidence at protein level	Evidence at protein level	Evidence at protein level	Evidence at protein level	Low tissue specificity	Detected in all	
	Asperger syndrome, Autism, Autism spectrum disorder, Disease variant	Evidence at protein level	Evidence at protein level	Evidence at protein level	Evidence at protein level	Tissue enhanced	Detected in many	
G-protein coupled receptor, Receptor, Transducer		Evidence at protein level	Evidence at protein level	Evidence at protein level	Evidence at protein level	Not detected	Not detected	
Acyltransferase, Transferase		Evidence at protein level	Evidence at protein level	Evidence at protein level	Evidence at protein level	Low tissue specificity	Detected in all	
G-protein coupled receptor, Receptor, Transducer		Evidence at protein level	Evidence at protein level	Evidence at protein level	Evidence at protein level	Tissue enhanced	Detected in some	
Calmodulin-binding, Oxidoreductase	Cancer-related genes, FDA approved drug targets	Evidence at protein level	Evidence at protein level	Evidence at protein level	Evidence at protein level	Group enriched	Detected in some	7
Activator, Developmental protein, Receptor	Cancer-related genes, Disease variant, Osteoporosis	Evidence at protein level	Evidence at protein level	Evidence at protein level	Evidence at protein level	Low tissue specificity	Detected in all	
Activator, Developmental protein, Receptor	Cancer-related genes, Disease variant	Evidence at protein level	Evidence at protein level	Evidence at protein level	Evidence at protein level	Tissue enhanced	Detected in many	
G-protein coupled receptor, Receptor, Transducer		Evidence at protein level	Evidence at transcript level	Evidence at protein level	Evidence at protein level	Tissue enhanced	Detected in some	
Hydrolase	Cancer-related genes, Disease variant, Proto-oncogene	Evidence at protein level	Evidence at protein level	Evidence at protein level	Evidence at protein level	Low tissue specificity	Detected in all	
Hydrolase	Disease variant	Evidence at protein level	Evidence at protein level	Evidence at protein level	Evidence at protein level	Tissue enhanced	Detected in many	
Developmental protein		Evidence at protein level	Evidence at protein level	Evidence at protein level	Evidence at protein level	Tissue enhanced	Detected in many	
Developmental protein	Autism spectrum disorder, Disease variant, Intellectual disability	Evidence at protein level	Evidence at transcript level	Evidence at protein level	Evidence at protein level	Tissue enhanced	Detected in some	
Developmental protein, Kinase, Receptor, Transferase, Tyrosine-protein kinase	Cancer-related genes, Disease variant, Epilepsy, FDA approved drug targets, Obesity	Evidence at protein level	Evidence at protein level	Evidence at protein level	Evidence at protein level	Tissue enhanced	Detected in many	
G-protein coupled receptor, Receptor, Transducer		Evidence at protein level	Evidence at protein level	Evidence at protein level	Evidence at protein level	Tissue enriched	Detected in some	7
Developmental protein	Cancer-related genes	Evidence at protein level	Evidence at protein level	Evidence at protein level	Evidence at protein level	Low tissue specificity	Detected in all	
	Disease variant	Evidence at protein level	Evidence at protein level	Evidence at protein level	Evidence at protein level	Tissue enhanced	Detected in many	
Chromatin regulator, Glycosyltransferase, Transferase	Disease variant, Intellectual disability	Evidence at protein level	Evidence at protein level	Evidence at protein level	Evidence at protein level	Low tissue specificity	Detected in all	
		Evidence at protein level	Evidence at protein level	Evidence at protein level	Evidence at protein level	Tissue enhanced	Detected in many	
Receptor		Evidence at protein level	Evidence at transcript level	Evidence at protein level	Evidence at protein level	Tissue enhanced	Detected in many	
G-protein coupled receptor, Photoreceptor protein, Receptor, Retinal protein, Transducer		Evidence at protein level	Evidence at protein level	Evidence at protein level	Evidence at protein level	Tissue enhanced	Detected in many	
G-protein coupled receptor, Receptor, Transducer	FDA approved drug targets	Evidence at protein level	Evidence at protein level	Evidence at protein level	Evidence at protein level	Tissue enhanced	Detected in some	
G-protein coupled receptor, Receptor, Transducer		Evidence at protein level	Evidence at protein level	Evidence at transcript lev	Evidence at transcript lev	Not detected	Not detected	
G-protein coupled receptor, Receptor, Transducer		Evidence at protein level	Evidence at protein level	Evidence at transcript lev	Evidence at transcript lev	Not detected	Not detected	
G-protein coupled receptor, Receptor, Transducer		Evidence at protein level	Evidence at protein level	Evidence at transcript lev	Evidence at transcript lev	Not detected	Not detected	
G-protein coupled receptor, Receptor, Transducer		Evidence at protein level	Evidence at protein level	Evidence at transcript lev	Evidence at transcript lev	Not detected	Not detected	
G-protein coupled receptor, Receptor, Transducer		Evidence at protein level	Evidence at transcript level	No human protein/transcr	No human protein/transcr	Not detected	Not detected	
		Evidence at protein level	Evidence at protein level	Evidence at protein level	Evidence at protein level	Tissue enhanced	Detected in many	
		Evidence at protein level	Evidence at protein level	Evidence at protein level	Evidence at protein level	Low tissue specificity	Detected in all	
G-protein coupled receptor, Receptor, Transducer	Disease variant	Evidence at protein level	Evidence at protein level	Evidence at transcript lev	Evidence at transcript lev	Tissue enhanced	Detected in some	
		Evidence at protein level	Evidence at protein level	Evidence at protein level	Evidence at protein level	Low tissue specificity	Detected in all	
		Evidence at protein level	Evidence at protein level	Evidence at protein level	Evidence at protein level	Low tissue specificity	Detected in many	
		Evidence at protein level	Evidence at protein level	Evidence at protein level	Evidence at protein level	Low tissue specificity	Detected in all	
Allosteric enzyme, Kinase, Serine/threonine-protein kinase, Transferase	Disease variant, Epilepsy, Intellectual disability	Evidence at protein level	Evidence at protein level	Evidence at protein level	Evidence at protein level	Tissue enhanced	Detected in all	
Kinase, Serine/threonine-protein kinase, Transferase		Evidence at protein level	Evidence at protein level	Evidence at protein level	Evidence at protein level	Low tissue specificity	Detected in all	
		Evidence at protein level	Evidence at protein level	Evidence at protein level	Evidence at protein level	Tissue enhanced	Detected in many	
		Evidence at protein level	Evidence at protein level	Evidence at protein level	Evidence at protein level	Low tissue specificity	Detected in all	
		Evidence at protein level	Evidence at protein level	Evidence at protein level	Evidence at protein level	Low tissue specificity	Detected in all	
Calcium channel, Ion channel	Disease variant	Evidence at protein level	Evidence at protein level	Evidence at protein level	Evidence at protein level	Low tissue specificity	Detected in all	
Ion channel		Evidence at protein level	Evidence at protein level	Evidence at transcript lev	Evidence at protein level	Tissue enriched	Detected in many	4
Developmental protein, Receptor		Evidence at protein level	Evidence at protein level	Evidence at protein level	Evidence at protein level	Tissue enhanced	Detected in many	
	Disease variant	Evidence at protein level	Evidence at protein level	Evidence at protein level	Evidence at protein level	Low tissue specificity	Detected in many	
		Evidence at protein level	Evidence at protein level	Evidence at protein level	Evidence at protein level	Low tissue specificity	Detected in many	
		Evidence at protein level	Evidence at protein level	Evidence at protein level	Evidence at protein level	Tissue enhanced	Detected in many	
Actin-binding	Cancer-related genes	Evidence at protein level	Evidence at protein level	Evidence at protein level	Evidence at protein level	Tissue enhanced	Detected in all	
		Evidence at protein level	Evidence at protein level	Evidence at protein level	Evidence at protein level	Low tissue specificity	Detected in all	
		Evidence at protein level	Evidence at protein level	Evidence at protein level	Evidence at protein level	Low tissue specificity	Detected in all	
		Evidence at protein level	Evidence at protein level	Evidence at protein level	Evidence at protein level	Tissue enhanced	Detected in many	
		Evidence at protein level	Evidence at protein level	Evidence at protein level	Evidence at protein level	Tissue enhanced	Detected in some	
		Evidence at protein level	Evidence at protein level	Evidence at transcript lev	Evidence at protein level	Low tissue specificity	Detected in many	
	Cancer-related genes	Evidence at protein level	Evidence at protein level	Evidence at protein level	Evidence at protein level	Low tissue specificity	Detected in all	
Hydrolase	FDA approved drug targets	Evidence at protein level	Evidence at protein level	Evidence at protein level	Evidence at protein level	Low tissue specificity	Detected in all	
Hydrolase	Disease variant, FDA approved drug targets	Evidence at protein level	Evidence at transcript level	Evidence at protein level	Evidence at protein level	Tissue enhanced	Detected in all	
Hydrolase		Evidence at protein level	Evidence at protein level	Evidence at protein level	Evidence at protein level	Tissue enhanced	Detected in many	
Developmental protein, Growth factor, Mitogen		Evidence at protein level	Evidence at protein level	Evidence at protein level	Evidence at protein level	Tissue enhanced	Detected in many	
Developmental protein, Kinase, Receptor, Transferase, Tyrosine-protein kinase	Cancer-related genes, Disease variant, FDA approved drug targets, Proto-oncogene	Evidence at protein level	Evidence at protein level	Evidence at protein level	Evidence at protein level	Tissue enhanced	Detected in all	
Developmental protein		Evidence at protein level	Evidence at protein level	Evidence at protein level	Evidence at protein level	Tissue enhanced	Detected in many	
		Evidence at protein level	Evidence at protein level	Evidence at protein level	Evidence at protein level	Tissue enhanced	Detected in many	
	Cancer-related genes	Evidence at protein level	Evidence at protein level	Evidence at protein level	Evidence at protein level	Tissue enhanced	Detected in all	
Hydrolase, Metalloprotease, Protease	Disease variant	Evidence at protein level	Evidence at transcript level	Evidence at protein level	Evidence at protein level	Tissue enhanced	Detected in some	
		Evidence at protein level	Evidence at protein level	Evidence at protein level	Evidence at protein level	Low tissue specificity	Detected in many	
Transferase	Disease variant, Epilepsy	Evidence at protein level	Evidence at protein level	Evidence at protein level	Evidence at protein level	Low tissue specificity	Detected in all	
Kinase, Serine/threonine-protein kinase, Transferase	Cancer-related genes, Disease variant, FDA approved drug targets	Evidence at protein level	Evidence at protein level	Evidence at protein level	Evidence at protein level	Group enriched	Detected in many	6
Kinase, Transferase	Cancer-related genes	Evidence at protein level	Evidence at protein level	Evidence at protein level	Evidence at protein level	Low tissue specificity	Detected in all	
Calcium channel, Ion channel, Potassium channel, Voltage-gated channel	Ciliopathy, Disease variant	Evidence at protein level	Evidence at protein level	Evidence at protein level	Evidence at protein level	Tissue enhanced	Detected in all	
	Cardiomyopathy, Disease variant	Evidence at protein level	Evidence at protein level	Evidence at protein level	Evidence at protein level	Tissue enhanced	Detected in many	
RNA-binding		Evidence at protein level	Evidence at protein level	Evidence at protein level	Evidence at protein level	Tissue enhanced	Detected in many	
		Evidence at protein level	Evidence at protein level	Evidence at protein level	Evidence at protein level	Tissue enriched	Detected in all	6
Calmodulin-binding, Hydrolase	Disease variant, Dystonia, Neurodegeneration, Parkinson disease, Parkinsonism	Evidence at protein level	Evidence at protein level	Evidence at protein level	Evidence at protein level	Low tissue specificity	Detected in all	
Developmental protein	Disease variant, Intellectual disability	Evidence at protein level	Evidence at protein level	Evidence at protein level	Evidence at protein level	Low tissue specificity	Detected in all	
Receptor	Cancer-related genes, FDA approved drug targets	Evidence at protein level	Evidence at protein level	Evidence at protein level	Evidence at protein level	Tissue enriched	Detected in all	6
Hydrolase, Transducer	Disease variant	Evidence at protein level	Evidence at transcript level	Evidence at protein level	Evidence at protein level	Tissue enhanced	Detected in many	
Hydrolase, Transducer	Cancer-related genes, Disease variant	Evidence at protein level	Evidence at protein level	Evidence at protein level	Evidence at protein level	Low tissue specificity	Detected in all	
		Evidence at protein level	Evidence at protein level	Evidence at protein level	Evidence at protein level	Low tissue specificity	Detected in many	
GTPase activation		Evidence at protein level	Evidence at protein level	Evidence at protein level	Evidence at protein level	Tissue enhanced	Detected in many	
		Evidence at protein level	Evidence at protein level	Evidence at protein level	Evidence at protein level	Tissue enhanced	Detected in many	
		Evidence at protein level	Evidence at protein level	Evidence at protein level	Evidence at protein level	Tissue enhanced	Detected in many	
Hydrolase		Evidence at protein level	Evidence at protein level	Evidence at protein level	Evidence at protein level	Low tissue specificity	Detected in many	
Hydrolase		Evidence at protein level	Evidence at protein level	Evidence at protein level	Evidence at protein level	Tissue enhanced	Detected in many	
DNA-binding, Host cell receptor for virus entry, Hydrolase, Nuclease, Receptor		Evidence at protein level	Evidence at protein level	Evidence at protein level	Evidence at protein level	Low tissue specificity	Detected in all	

		Evidence at protein level	Evidence at protein level	Evidence at protein level	Evidence at protein level	Tissue enhanced	Detected in many	
DNA-binding, DNA-directed DNA polymerase, Hydrolase, Nucleotidyltransferase, Transferase	Cancer-related genes, Disease variant, FDA approved drug targets	Evidence at protein level	Evidence at protein level	Evidence at protein level	Evidence at protein level	Tissue enhanced	Detected in many	
	Cancer-related genes, Disease variant, Epilepsy, Intellectual disability	Evidence at protein level	Evidence at protein level	Evidence at protein level	Evidence at protein level	Low tissue specificity	Detected in all	
Kinase, Transferase		Evidence at protein level	Evidence at protein level	Evidence at protein level	Evidence at protein level	Tissue enhanced	Detected in all	
		Evidence at protein level	Evidence at protein level	Evidence at protein level	Evidence at protein level	Tissue enhanced	Detected in many	
	FDA approved drug targets	Evidence at protein level	Evidence at protein level	Evidence at protein level	Evidence at protein level	Tissue enhanced	Detected in all	
		Evidence at protein level	Evidence at protein level	Evidence at protein level	Evidence at protein level	Low tissue specificity	Detected in all	
Repressor		Evidence at protein level	Evidence at protein level	Evidence at protein level	Evidence at protein level	Tissue enriched	Detected in some	11
Kinase, Serine/threonine-protein kinase, Transferase	Cancer-related genes, FDA approved drug targets, Proto-oncogene	Evidence at protein level	Evidence at protein level	Evidence at protein level	Evidence at protein level	Tissue enhanced	Detected in many	
Kinase, Serine/threonine-protein kinase, Transferase	Cancer-related genes, FDA approved drug targets	Evidence at protein level	Evidence at protein level	Evidence at protein level	Evidence at protein level	Tissue enhanced	Detected in all	
Kinase, Serine/threonine-protein kinase, Transferase	Disease variant, Ectodermal dysplasia	Evidence at protein level	Evidence at protein level	Evidence at protein level	Evidence at protein level	Low tissue specificity	Detected in many	
Kinase, Serine/threonine-protein kinase, Transferase		Evidence at protein level	Evidence at protein level	Evidence at protein level	Evidence at protein level	Low tissue specificity	Detected in all	
		Evidence at protein level	Evidence at protein level	Evidence at protein level	Evidence at protein level	Tissue enhanced	Detected in many	
	Disease variant, Dystonia, Epilepsy	Evidence at protein level	Evidence at protein level	Evidence at protein level	Evidence at protein level	Tissue enhanced	Detected in many	
	Cancer-related genes	Evidence at protein level	Evidence at protein level	Evidence at protein level	Evidence at protein level	Tissue enriched	Detected in many	5
Kinase, Serine/threonine-protein kinase, Transferase	Disease variant, Intrahepatic cholestasis	Evidence at protein level	Evidence at protein level	Evidence at protein level	Evidence at protein level	Low tissue specificity	Detected in all	
		Evidence at protein level	Evidence at protein level	Evidence at protein level	Evidence at protein level	Low tissue specificity	Detected in all	
	Disease variant	Evidence at protein level	Evidence at protein level	Evidence at protein level	Evidence at protein level	Group enriched	Detected in many	4
G-protein coupled receptor, Receptor, Transducer	FDA approved drug targets	Evidence at protein level	Evidence at protein level	Evidence at protein level	Evidence at protein level	Tissue enhanced	Detected in many	
Receptor	Cancer-related genes, Disease variant	Evidence at protein level	Evidence at protein level	Evidence at protein level	Evidence at protein level	Tissue enhanced	Detected in many	
G-protein coupled receptor, Receptor, Transducer	Disease variant, Dwarfism, FDA approved drug targets	Evidence at protein level	Evidence at protein level	Evidence at protein level	Evidence at protein level	Tissue enriched	Detected in many	7
Kinase, Transferase, Tyrosine-protein kinase		Evidence at protein level	Evidence at protein level	Evidence at protein level	Evidence at protein level	Tissue enhanced	Detected in all	
Receptor		Evidence at protein level	Evidence at transcript level	Evidence at protein level	Evidence at protein level	Low tissue specificity	Detected in many	
Hydrolase, Protein phosphatase		Evidence at protein level	Evidence at transcript level	Evidence at protein level	Evidence at protein level	Tissue enhanced	Detected in many	
Hydrolase, Protein phosphatase		Evidence at protein level	Evidence at transcript level	Evidence at protein level	Evidence at protein level	Low tissue specificity	Detected in all	
Hydrolase, Protein phosphatase, Receptor	Cancer-related genes	Evidence at protein level	Evidence at protein level	Evidence at protein level	Evidence at protein level	Low tissue specificity	Detected in many	
Hydrolase, Protein phosphatase, Receptor		Evidence at protein level	Evidence at protein level	Evidence at protein level	Evidence at protein level	Low tissue specificity	Detected in all	
Hydrolase, Protein phosphatase, Receptor		Evidence at protein level	Evidence at protein level	Evidence at protein level	Evidence at protein level	Tissue enhanced	Detected in some	
Heparin-binding, Hydrolase, Protein phosphatase, Receptor		Evidence at protein level	Evidence at transcript level	Evidence at protein level	Evidence at protein level	Low tissue specificity	Detected in all	
Host cell receptor for virus entry, Receptor		Evidence at protein level	Evidence at protein level	Evidence at protein level	Evidence at protein level	Low tissue specificity	Detected in all	
Actin-binding		Evidence at protein level	Evidence at protein level	Evidence at protein level	Evidence at protein level	Tissue enhanced	Detected in all	
Hydrolase		Evidence at protein level	Evidence at protein level	Evidence at protein level	Evidence at protein level	Low tissue specificity	Detected in all	
Developmental protein, Hydrolase	Craniosynostosis, Disease variant	Evidence at protein level	Evidence at protein level	Evidence at protein level	Evidence at protein level	Low tissue specificity	Detected in many	
Hydrolase	Cancer-related genes	Evidence at protein level	Evidence at protein level	Evidence at protein level	Evidence at protein level	Tissue enhanced	Detected in many	
Hydrolase	Proto-oncogene	Evidence at protein level	Evidence at protein level	Evidence at protein level	Evidence at protein level	Low tissue specificity	Detected in all	
		Evidence at protein level	Evidence at protein level	Evidence at protein level	Evidence at protein level	Tissue enhanced	Detected in some	
		Evidence at protein level	Evidence at protein level	Evidence at protein level	Evidence at protein level	Group enriched	Detected in some	4
Kinase, Serine/threonine-protein kinase, Transferase	Cancer-related genes, Cardiomyopathy, Deafness, Disease variant, FDA approved drug targe	Evidence at protein level	Evidence at protein level	Evidence at protein level	Evidence at protein level	Tissue enhanced	Detected in all	
Hydrolase	Disease variant, Intellectual disability	Evidence at protein level	Evidence at protein level	Evidence at protein level	Evidence at protein level	Low tissue specificity	Detected in all	
GTPase activation, Translocase		Evidence at protein level	Evidence at protein level	Evidence at protein level	Evidence at protein level	Low tissue specificity	Detected in all	
Receptor	FDA approved drug targets	Evidence at protein level	Evidence at protein level	Evidence at protein level	Evidence at protein level	Low tissue specificity	Detected in many	
Developmental protein, GTPase activation, Guanine-nucleotide releasing factor	Epilepsy	Evidence at protein level	Evidence at protein level	Evidence at protein level	Evidence at protein level	Low tissue specificity	Detected in all	
		Evidence at protein level	Evidence at protein level	Evidence at protein level	Evidence at protein level	Tissue enhanced	Detected in all	
GTPase activation		Evidence at protein level	Evidence at protein level	Evidence at protein level	Evidence at protein level	Group enriched	Detected in many	4
Guanine-nucleotide releasing factor	Disease variant, Systemic lupus erythematosus	Evidence at protein level	Evidence at protein level	Evidence at protein level	Evidence at protein level	Tissue enhanced	Detected in many	
Guanine-nucleotide releasing factor	Disease variant	Evidence at protein level	Evidence at protein level	Evidence at protein level	Evidence at protein level	Tissue enhanced	Detected in all	
Guanine-nucleotide releasing factor		Evidence at protein level	Evidence at protein level	Evidence at protein level	Evidence at protein level	Group enriched	Detected in many	5
RNA-binding, Transferase	Disease variant	Evidence at protein level	Evidence at protein level	Evidence at protein level	Evidence at protein level	Low tissue specificity	Detected in all	
Chromatin regulator		Evidence at protein level	Evidence at protein level	Evidence at protein level	Evidence at protein level	Low tissue specificity	Detected in many	
Actin capping, Actin-binding	Deafness, Disease variant, Non-syndromic deafness	Evidence at protein level	Evidence at protein level	Evidence at protein level	Evidence at protein level	Tissue enhanced	Detected in all	
Protease inhibitor, Serine protease inhibitor	Tumor suppressor	Evidence at protein level	Evidence at protein level	Evidence at protein level	Evidence at protein level	Low tissue specificity	Detected in all	
		Evidence at protein level	Evidence at protein level	Evidence at protein level	Evidence at protein level	Tissue enhanced	Detected in all	
	Disease variant, Neurodegeneration	Evidence at protein level	Evidence at protein level	Evidence at protein level	Evidence at protein level	Low tissue specificity	Detected in all	
Kinase, Receptor, Transferase, Tyrosine-protein kinase	Cancer-related genes, Disease variant, FDA approved drug targets, Hirschsprung disease, P	Evidence at protein level	Evidence at transcript level	Evidence at protein level	Evidence at protein level	Tissue enhanced	Detected in many	
Signal transduction inhibitor		Evidence at protein level	Evidence at protein level	Evidence at protein level	Evidence at protein level	Tissue enhanced	Detected in many	
Signal transduction inhibitor		Evidence at protein level	Evidence at protein level	Evidence at protein level	Evidence at protein level	Tissue enhanced	Detected in all	
		Evidence at protein level	Evidence at protein level	Evidence at protein level	Evidence at protein level	Tissue enhanced	Detected in many	
Hydrolase	Cancer-related genes, Disease variant, Ectodermal dysplasia, Proto-oncogene	Evidence at protein level	Evidence at protein level	Evidence at protein level	Evidence at protein level	Low tissue specificity	Detected in all	
Helicase, Hydrolase, RNA-binding	Disease variant	Evidence at protein level	Evidence at protein level	Evidence at protein level	Evidence at protein level	Low tissue specificity	Detected in all	
		Evidence at protein level	Evidence at protein level	Evidence at protein level	Evidence at protein level	Tissue enhanced	Detected in many	
Transferase		Evidence at protein level	Evidence at protein level	Evidence at protein level	Evidence at protein level	Low tissue specificity	Detected in all	
Developmental protein, Receptor	Disease variant, Dwarfism, Intellectual disability	Evidence at protein level	Evidence at protein level	Evidence at protein level	Evidence at protein level	Low tissue specificity	Detected in many	
GTPase activation	Disease variant, Retinitis pigmentosa	Evidence at protein level	Evidence at protein level	Evidence at protein level	Evidence at protein level	Tissue enhanced	Detected in all	
Host cell receptor for virus entry, Receptor, Ribonucleoprotein, Ribosomal protein	Disease variant	Evidence at protein level	Evidence at protein level	Evidence at protein level	Evidence at protein level	Low tissue specificity	Detected in all	
		Evidence at protein level	Evidence at protein level	Evidence at protein level	Evidence at protein level	Group enriched	Detected in all	4
		Evidence at protein level	Evidence at protein level	Evidence at protein level	Evidence at protein level	Low tissue specificity	Detected in many	
Receptor	Disease variant, Schizophrenia	Evidence at protein level	Evidence at transcript level	Evidence at protein level	Evidence at protein level	Tissue enriched	Detected in many	7
Receptor		Evidence at protein level	Evidence at transcript level	Evidence at protein level	Evidence at protein level	Tissue enhanced	Detected in many	
		Evidence at protein level	Evidence at protein level	Evidence at protein level	Evidence at protein level	Tissue enriched	Detected in many	5
	Cancer-related genes	Evidence at protein level	Evidence at protein level	Evidence at protein level	Evidence at protein level	Low tissue specificity	Detected in all	
Antimicrobial, Antioxidant	Cancer-related genes	Evidence at protein level	Evidence at protein level	Evidence at protein level	Evidence at protein level	Group enriched	Detected in all	4
		Evidence at protein level	Evidence at protein level	Evidence at protein level	Evidence at protein level	Tissue enhanced	Detected in many	
Ion channel, Sodium channel, Voltage-gated channel	Autism, Autism spectrum disorder, Disease variant, Epilepsy, FDA approved drug targets	Evidence at protein level	Evidence at protein level	Evidence at protein level	Evidence at protein level	Tissue enhanced	Detected in some	
Ion channel, Sodium channel, Voltage-gated channel	Atrial fibrillation, Brugada syndrome, Disease variant, FDA approved drug targets	Evidence at protein level	Evidence at transcript level	Evidence at protein level	Evidence at protein level	Tissue enhanced	Detected in many	
Calmodulin-binding, Ion channel, Sodium channel, Voltage-gated channel	Atrial fibrillation, Brugada syndrome, Cardiomyopathy, Disease variant, FDA approved drug t	Evidence at protein level	Evidence at protein level	Evidence at protein level	Evidence at protein level	Tissue enriched	Detected in some	26
Ion channel, Sodium channel, Voltage-gated channel	Disease variant, Epilepsy, FDA approved drug targets, Intellectual disability	Evidence at protein level	Evidence at transcript level	Evidence at protein level	Evidence at protein level	Group enriched	Detected in some	4
Ion channel, Sodium channel	FDA approved drug targets	Evidence at protein level	Evidence at protein level	Evidence at protein level	Evidence at protein level	Tissue enhanced	Detected in many	
Ion channel, Sodium channel	Disease variant, FDA approved drug targets	Evidence at protein level	Evidence at protein level	Evidence at protein level	Evidence at protein level	Tissue enhanced	Detected in many	
Developmental protein	Disease variant	Evidence at protein level	Evidence at protein level	Evidence at protein level	Evidence at protein level	Low tissue specificity	Detected in many	
	Disease variant, Dwarfism	Evidence at protein level	Evidence at protein level	Evidence at protein level	Evidence at protein level	Group enriched	Detected in many	7
		Evidence at protein level	Evidence at protein level	Evidence at protein level	Evidence at protein level	Tissue enhanced	Detected in all	
DNA-binding, RNA-binding		Evidence at protein level	Evidence at protein level	Evidence at protein level	Evidence at protein level	Low tissue specificity	Detected in all	
Developmental protein, Receptor		Evidence at protein level	Evidence at protein level	Evidence at protein level	Evidence at protein level	Tissue enhanced	Detected in all	
		Evidence at protein level	Evidence at protein level	Evidence at transcript lev	Evidence at protein level	Low tissue specificity	Detected in all	
		Evidence at protein level	Evidence at protein level	Evidence at protein level	Evidence at protein level	Tissue enriched	Detected in some	11
		Evidence at protein level	Evidence at protein level	Evidence at protein level	Evidence at protein level	Tissue enhanced	Detected in all	
		Evidence at protein level	Evidence at protein level	Evidence at protein level	Evidence at protein level	Group enriched	Detected in many	5
GTPase activation		Evidence at protein level	Evidence at protein level	Evidence at protein level	Evidence at protein level	Low tissue specificity	Detected in all	
GTPase activation	Cataract	Evidence at protein level	Evidence at protein level	Evidence at protein level	Evidence at protein level	Low tissue specificity	Detected in all	
	Disease variant	Evidence at protein level	Evidence at protein level	Evidence at protein level	Evidence at protein level	Tissue enriched	Detected in many	9
	Amelogenesis imperfecta, Disease variant, Epilepsy	Evidence at protein level	Evidence at transcript level	Evidence at protein level	Evidence at protein level	Tissue enriched	Detected in some	11
Blood group antigen		Evidence at protein level	Evidence at protein level	Evidence at protein level	Evidence at protein level	Tissue enhanced	Detected in many	
	Disease variant	Evidence at protein level	Evidence at protein level	Evidence at protein level	Evidence at protein level	Low tissue specificity	Detected in all	
		Evidence at protein level	Evidence at protein level	Evidence at protein level	Evidence at protein level	Tissue enhanced	Detected in many	
		Evidence at protein level	Evidence at protein level	Evidence at protein level	Evidence at protein level	Tissue enhanced	Detected in all	
		Evidence at protein level	Evidence at protein level	Evidence at protein level	Evidence at protein level	Tissue enhanced	Detected in many	
	Disease variant	Evidence at protein level	Evidence at protein level	Evidence at protein level	Evidence at protein level	Tissue enriched	Detected in all	7
Host cell receptor for virus entry, Receptor	FDA approved drug targets	Evidence at protein level	Evidence at protein level	Evidence at protein level	Evidence at protein level	Low tissue specificity	Detected in all	
		Evidence at protein level	Evidence at protein level	Evidence at protein level	Evidence at protein level	Tissue enriched	Detected in some	4
Receptor		Evidence at protein level	Evidence at protein level	Evidence at protein level	Evidence at protein level	Low tissue specificity	Detected in all	
		Evidence at protein level	Evidence at protein level	Evidence at protein level	Evidence at protein level	Tissue enhanced	Detected in some	
Chloride channel, Ion channel, Transferase		Evidence at protein level	Evidence at protein level	Evidence at protein level	Evidence at protein level	Low tissue specificity	Detected in all	
	Disease variant	Evidence at protein level	Evidence at protein level	Evidence at protein level	Evidence at protein level	Group enriched	Detected in some	21
	Cancer-related genes, Cataract, Disease variant, Dystonia, Epilepsy, Hereditary hemolytic an	Evidence at protein level	Evidence at protein level	Evidence at protein level	Evidence at protein level	Tissue enhanced	Detected in many	
		Evidence at protein level	Evidence at protein level	Evidence at transcript lev	Evidence at protein level	Tissue enriched	Detected in many	5
Developmental protein		Evidence at protein level	Evidence at protein level	Evidence at transcript lev	Evidence at protein level	Tissue enriched	Detected in some	12
	Disease variant	Evidence at protein level	Evidence at protein level	Evidence at protein level	Evidence at protein level	Tissue enriched	Detected in some	13
		Evidence at protein level	Evidence at protein level	Evidence at protein level	Evidence at protein level	Tissue enhanced	Detected in all	
		Evidence at protein level	Evidence at protein level	Evidence at protein level	Evidence at protein level	Tissue enhanced	Detected in many	
		Evidence at protein level	Evidence at protein level	Evidence at protein level	Evidence at protein level	Tissue enhanced	Detected in all	
	Diabetes mellitus	Evidence at protein level	Evidence at protein level	Evidence at protein level	Evidence at protein level	Tissue enriched	Detected in some	4
	Disease variant, Osteoporosis	Evidence at protein level	Evidence at transcript level	Evidence at protein level	Evidence at protein level	Tissue enriched	Detected in some	55
	Cancer-related genes, Disease variant	Evidence at protein level	Evidence at protein level	Evidence at protein level	Evidence at protein level	Tissue enriched	Detected in many	8
		Evidence at protein level	Evidence at protein level	Evidence at protein level	Evidence at protein level	Low tissue specificity	Detected in many	
	FDA approved drug targets	Evidence at protein level	Evidence at protein level	Evidence at protein level	Evidence at protein level	Low tissue specificity	Detected in all	
		Evidence at protein level	Evidence at protein level	Evidence at protein level	Evidence at protein level	Low tissue specificity	Detected in all	
Host cell receptor for virus entry, Receptor	Cancer-related genes	Evidence at protein level	Evidence at protein level	Evidence at protein level	Evidence at protein level	Low tissue specificity	Detected in all	
	Disease variant	Evidence at protein level	Evidence at protein level	Evidence at protein level	Evidence at protein level	Low tissue specificity	Detected in all	
		Evidence at protein level	Evidence at protein level	Evidence at protein level	Evidence at protein level	Low tissue specificity	Detected in all	
	Disease variant	Evidence at protein level	Evidence at protein level	Evidence at protein level	Evidence at protein level	Tissue enhanced	Detected in all	
	Osteopetrosis	Evidence at protein level	Evidence at protein level	Evidence at protein level	Evidence at protein level	Tissue enhanced	Detected in all	
	Disease variant, Retinitis pigmentosa	Evidence at protein level	Evidence at protein level	Evidence at protein level	Evidence at protein level	Tissue enhanced	Detected in many	
		Evidence at protein level	Evidence at protein level	Evidence at protein level	Evidence at protein level	Tissue enhanced	Detected in all	
	Disease variant, FDA approved drug targets	Evidence at protein level	Evidence at protein level	Evidence at protein level	Evidence at protein level	Tissue enriched	Detected in many	7
		Evidence at protein level	Evidence at protein level	Evidence at transcript lev	Evidence at protein level	Tissue enhanced	Detected in some	
		Evidence at protein level	Evidence at protein level	Evidence at protein level	Evidence at protein level	Tissue enhanced	Detected in all	
		Evidence at protein level	Evidence at protein level	Evidence at protein level	Evidence at protein level	Tissue enhanced	Detected in many	
		Evidence at protein level	Evidence at protein level	Evidence at protein level	Evidence at protein level	Tissue enhanced	Detected in all	
		Evidence at protein level	Evidence at transcript level	Evidence at protein level	Evidence at protein level	Tissue enhanced	Detected in all	

Calmodulin-binding	FDA approved drug targets	Evidence at protein level	Evidence at protein level	Evidence at protein level	Evidence at protein level	Tissue enriched	Detected in many	4
Calmodulin-binding	Deafness, Disease variant, Neurodegeneration	Evidence at protein level	Evidence at protein level	Evidence at protein level	Evidence at protein level	Tissue enhanced	Detected in all	
		Evidence at protein level	Evidence at protein level	Evidence at transcript lev	Evidence at protein level	Tissue enhanced	Detected in many	
	Disease variant, FDA approved drug targets	Evidence at protein level	Evidence at protein level	Evidence at protein level	Evidence at protein level	Tissue enhanced	Detected in all	
		Evidence at protein level	Evidence at protein level	Evidence at protein level	Evidence at protein level	Tissue enhanced	Detected in many	
	Disease variant, Epilepsy, Intellectual disability	Evidence at protein level	Evidence at protein level	Evidence at protein level	Evidence at protein level	Tissue enhanced	Detected in all	
		Evidence at protein level	Evidence at protein level	Evidence at transcript lev	Evidence at protein level	Tissue enriched	Detected in single	7
		Evidence at protein level	Evidence at protein level	Evidence at protein level	Evidence at protein level	Tissue enhanced	Detected in some	
		Evidence at protein level	Evidence at protein level	Evidence at protein level	Evidence at protein level	Tissue enriched	Detected in single	610
Protease inhibitor, Serine protease inhibitor		Evidence at protein level	Evidence at protein level	Evidence at protein level	Evidence at protein level	Tissue enriched	Detected in some	19
		Evidence at protein level	Evidence at protein level	Evidence at protein level	Evidence at protein level	Tissue enhanced	Detected in many	
		Evidence at protein level	Evidence at protein level	Evidence at transcript lev	Evidence at protein level	Tissue enhanced	Detected in some	
Developmental protein		Evidence at protein level	Evidence at protein level	Evidence at transcript lev	Evidence at protein level	Low tissue specificity	Detected in many	
	Deafness	Evidence at protein level	Evidence at protein level	Evidence at transcript lev	Evidence at protein level	Tissue enhanced	Detected in many	
		Evidence at protein level	Evidence at protein level	Evidence at protein level	Evidence at protein level	Tissue enhanced	Detected in many	
		Evidence at protein level	Evidence at protein level	Evidence at protein level	Evidence at protein level	Low tissue specificity	Detected in all	
Actin-binding, Calmodulin-binding		Evidence at protein level	Evidence at protein level	Evidence at protein level	Evidence at protein level	Low tissue specificity	Detected in all	
Actin-binding		Evidence at protein level	Evidence at protein level	Evidence at protein level	Evidence at protein level	Low tissue specificity	Detected in some	
		Evidence at protein level	Evidence at protein level	Evidence at protein level	Evidence at protein level	Low tissue specificity	Detected in all	
		Evidence at protein level	Evidence at protein level	Evidence at protein level	Evidence at protein level	Tissue enhanced	Detected in all	
Developmental protein, Receptor		Evidence at protein level	Evidence at protein level	Evidence at protein level	Evidence at protein level	Low tissue specificity	Detected in all	
	Amyotrophic lateral sclerosis, Charcot-Marie-Tooth disease, Hereditary spastic paraplegia, N	Evidence at protein level	Evidence at protein level	Evidence at protein level	Evidence at protein level	Low tissue specificity	Detected in all	
Calmodulin-binding, Kinase, Transferase		Evidence at protein level	Evidence at protein level	Evidence at protein level	Evidence at protein level	Low tissue specificity	Detected in many	
Protease inhibitor, Serine protease inhibitor	Cancer-related genes	Evidence at protein level	Evidence at protein level	Evidence at protein level	Evidence at protein level	Tissue enhanced	Detected in many	
Actin-binding		Evidence at protein level	Evidence at protein level	Evidence at protein level	Evidence at protein level	Low tissue specificity	Detected in all	
Hydrolase, Protease		Evidence at protein level	Evidence at protein level	Evidence at protein level	Evidence at protein level	Low tissue specificity	Detected in all	
Hydrolase, Protease		Evidence at protein level	Evidence at protein level	Evidence at protein level	Evidence at protein level	Low tissue specificity	Detected in all	
Kinase, Transferase, Tyrosine-protein kinase	Cancer-related genes, Disease variant, FDA approved drug targets, Proto-oncogene	Evidence at protein level	Evidence at protein level	Evidence at protein level	Evidence at protein level	Low tissue specificity	Detected in many	
G-protein coupled receptor, Receptor, Transducer	FDA approved drug targets	Evidence at protein level	Evidence at protein level	Evidence at protein level	Evidence at protein level	Tissue enhanced	Detected in some	
Hydrolase, Metalloprotease, Protease	Disease variant	Evidence at protein level	Evidence at protein level	Evidence at protein level	Evidence at protein level	Low tissue specificity	Detected in all	
Oxidoreductase		Evidence at protein level	Evidence at transcript level	Evidence at protein level	Evidence at protein level	Tissue enhanced	Detected in many	
Kinase, Serine/threonine-protein kinase, Transferase		Evidence at protein level	Evidence at protein level	Evidence at protein level	Evidence at protein level	Tissue enhanced	Detected in all	
		Evidence at protein level	Evidence at protein level	Evidence at protein level	Evidence at protein level	Low tissue specificity	Detected in all	
		Evidence at protein level	Evidence at protein level	Evidence at protein level	Evidence at protein level	Low tissue specificity	Detected in all	
		Evidence at protein level	Evidence at protein level	Evidence at protein level	Evidence at protein level	Tissue enriched	Detected in some	11
		Evidence at protein level	Evidence at protein level	Evidence at protein level	Evidence at protein level	Tissue enhanced	Detected in all	
Actin-binding	Desmin-related myopathy, Disease variant, Myofibrillar myopathy	Evidence at protein level	Evidence at protein level	Evidence at protein level	Evidence at protein level	Tissue enhanced	Detected in all	
DNA-binding		Evidence at protein level	Evidence at protein level	Evidence at protein level	Evidence at protein level	Low tissue specificity	Detected in all	
		Evidence at protein level	Evidence at protein level	Evidence at protein level	Evidence at protein level	Low tissue specificity	Detected in all	
		Evidence at protein level	Evidence at transcript level	Evidence at protein level	Evidence at protein level	Tissue enhanced	Detected in all	
Transferase		Evidence at protein level	Evidence at protein level	Evidence at protein level	Evidence at protein level	Low tissue specificity	Detected in all	
G-protein coupled receptor, Receptor, Transducer	FDA approved drug targets	Evidence at protein level	Evidence at protein level	Evidence at protein level	Evidence at protein level	Tissue enhanced	Detected in many	
G-protein coupled receptor, Receptor, Transducer		Evidence at protein level	Evidence at protein level	Evidence at protein level	Evidence at protein level	Tissue enhanced	Detected in many	
Tumor antigen	Amyloidosis, Corneal dystrophy, FDA approved drug targets	Evidence at protein level	Evidence at protein level	Evidence at protein level	Evidence at protein level	Tissue enhanced	Detected in many	
G-protein coupled receptor, Receptor, Transducer		Evidence at protein level	Evidence at protein level	Evidence at protein level	Evidence at protein level	Group enriched	Detected in some	4
GTPase activation, Guanine-nucleotide releasing factor		Evidence at protein level	Evidence at protein level	Evidence at protein level	Evidence at protein level	Low tissue specificity	Detected in all	
GTPase activation	Deafness, Disease variant, Epilepsy, Intellectual disability, Non-syndromic deafness	Evidence at protein level	Evidence at protein level	Evidence at protein level	Evidence at protein level	Tissue enhanced	Detected in many	
		Evidence at protein level	Evidence at protein level	Evidence at protein level	Evidence at protein level	Tissue enhanced	Detected in many	
G-protein coupled receptor, Receptor, Transducer	Disease variant	Evidence at protein level	Evidence at protein level	Evidence at protein level	Evidence at protein level	Tissue enriched	Detected in many	4
		Evidence at protein level	Evidence at protein level	Evidence at protein level	Evidence at protein level	Low tissue specificity	Detected in many	
		Evidence at protein level	Evidence at protein level			Not detected	Not detected	
Kinase, Transferase, Tyrosine-protein kinase		Evidence at protein level	Evidence at protein level	Evidence at protein level	Evidence at protein level	Low tissue specificity	Detected in many	
Kinase, Receptor, Transferase, Tyrosine-protein kinase	Cancer-related genes, Disease variant, FDA approved drug targets, Glaucoma	Evidence at protein level	Evidence at protein level	Evidence at protein level	Evidence at protein level	Tissue enhanced	Detected in many	
Kinase, Receptor, Serine/threonine-protein kinase, Transferase	Aortic aneurysm, Craniosynostosis, Disease variant	Evidence at protein level	Evidence at protein level	Evidence at protein level	Evidence at protein level	Low tissue specificity	Detected in all	
Kinase, Receptor, Serine/threonine-protein kinase, Transferase	Aortic aneurysm, Cancer-related genes, Craniosynostosis, Disease variant, FDA approved dr	Evidence at protein level	Evidence at protein level	Evidence at protein level	Evidence at protein level	Low tissue specificity	Detected in all	
Guanine-nucleotide releasing factor	Epilepsy, Intellectual disability	Evidence at protein level	Evidence at protein level	Evidence at protein level	Evidence at protein level	Tissue enriched	Detected in many	7
DNA-binding		Evidence at protein level	Evidence at protein level	Evidence at protein level	Evidence at protein level	Low tissue specificity	Detected in many	
Calmodulin-binding		Evidence at protein level	Evidence at protein level	Evidence at protein level	Evidence at protein level	Low tissue specificity	Detected in all	
	Cancer-related genes, Disease variant, Intrahepatic cholestasis	Evidence at protein level	Evidence at protein level	Evidence at protein level	Evidence at protein level	Low tissue specificity	Detected in all	
		Evidence at protein level	Evidence at protein level	Evidence at protein level	Evidence at protein level	Tissue enhanced	Detected in many	
		Evidence at protein level	Evidence at protein level	Evidence at protein level	Evidence at protein level	Tissue enhanced	Detected in all	
Receptor	FDA approved drug targets	Evidence at protein level	Evidence at protein level	Evidence at protein level	Evidence at protein level	Tissue enhanced	Detected in all	
Receptor	Age-related macular degeneration, FDA approved drug targets	Evidence at protein level	Evidence at protein level	Evidence at protein level	Evidence at protein level	Low tissue specificity	Detected in all	
	Autism spectrum disorder	Evidence at protein level	Evidence at protein level	Evidence at protein level	Evidence at protein level	Tissue enriched	Detected in some	76
		Evidence at protein level	Evidence at protein level	Evidence at protein level	Evidence at protein level	Tissue enriched	Detected in single	178
		Evidence at protein level	Evidence at protein level	Evidence at protein level	Evidence at protein level	Group enriched	Detected in some	5
Ion channel		Evidence at protein level	Evidence at protein level	Evidence at protein level	Evidence at protein level	Low tissue specificity	Detected in all	
		Evidence at protein level	Evidence at protein level	Evidence at protein level	Evidence at protein level	Low tissue specificity	Detected in all	
Hydrolase, Protease, Serine protease	Cancer-related genes	Evidence at protein level	Evidence at protein level	Evidence at protein level	Evidence at protein level	Tissue enhanced	Detected in many	
Receptor	Deafness, Disease variant, Osteopetrosis	Evidence at protein level	Evidence at protein level	Evidence at protein level	Evidence at protein level	Tissue enhanced	Detected in many	
Host cell receptor for virus entry, Receptor	Cancer-related genes	Evidence at protein level	Evidence at protein level	Evidence at protein level	Evidence at protein level	Low tissue specificity	Detected in many	
Receptor		Evidence at protein level	Evidence at protein level	Evidence at protein level	Evidence at protein level	Tissue enhanced	Detected in all	
Receptor	Cancer-related genes, FDA approved drug targets	Evidence at protein level	Evidence at protein level	Evidence at protein level	Evidence at protein level	Tissue enhanced	Detected in some	
Cytokine	Cancer-related genes, FDA approved drug targets	Evidence at protein level	Evidence at protein level	Evidence at protein level	Evidence at protein level	Tissue enhanced	Detected in many	
Kinase, Serine/threonine-protein kinase, Transferase, Tyrosine-protein kinase	Cancer-related genes	Evidence at protein level	Evidence at protein level	Evidence at protein level	Evidence at protein level	Low tissue specificity	Detected in all	
	Cancer-related genes	Evidence at protein level	Evidence at protein level	Evidence at protein level	Evidence at protein level	Low tissue specificity	Detected in all	
Hydrolase, Metalloprotease, Protease		Evidence at protein level	Evidence at protein level	Evidence at protein level	Evidence at protein level	Tissue enhanced	Detected in many	
Transferase	Disease variant, Dwarfism, Intellectual disability	Evidence at protein level	Evidence at protein level	Evidence at protein level	Evidence at protein level	Low tissue specificity	Detected in many	
		Evidence at protein level	Evidence at protein level	Evidence at protein level	Evidence at protein level	Tissue enriched	Detected in some	26
	Disease variant	Evidence at protein level	Evidence at protein level	Evidence at protein level	Evidence at protein level	Tissue enhanced	Detected in many	
Receptor		Evidence at protein level	Evidence at protein level	Evidence at protein level	Evidence at protein level	Group enriched	Detected in some	4
Calmodulin-binding, Ion channel	Disease variant, Palmoplantar keratoderma	Evidence at protein level	Evidence at protein level	Evidence at protein level	Evidence at protein level	Low tissue specificity	Detected in all	
Calcium channel, Ion channel		Evidence at protein level	Evidence at protein level	Evidence at protein level	Evidence at protein level	Low tissue specificity	Detected in many	
		Evidence at protein level	Evidence at protein level	Evidence at protein level	Evidence at protein level	Tissue enhanced	Detected in many	
Receptor		Evidence at protein level	Evidence at protein level	Evidence at protein level	Evidence at protein level	Tissue enriched	Detected in some	39
		Evidence at protein level	Evidence at protein level	Evidence at protein level	Evidence at protein level	Low tissue specificity	Detected in all	
Developmental protein	Ciliopathy, Disease variant	Evidence at protein level	Evidence at protein level	Evidence at protein level	Evidence at protein level	Low tissue specificity	Detected in all	
Kinase, Transferase, Tyrosine-protein kinase		Evidence at protein level	Evidence at protein level	Evidence at protein level	Evidence at protein level	Tissue enhanced	Detected in many	
		Evidence at protein level	Evidence at protein level	Evidence at protein level	Evidence at protein level	Low tissue specificity	Detected in all	
		Evidence at protein level	Evidence at protein level	Evidence at protein level	Evidence at protein level	Tissue enhanced	Detected in all	
Transferase	Cancer-related genes	Evidence at protein level	Evidence at protein level	Evidence at protein level	Evidence at protein level	Tissue enhanced	Detected in many	
Transferase		Evidence at protein level	Evidence at transcript level	Evidence at protein level	Evidence at protein level	Low tissue specificity	Detected in all	
		Evidence at protein level	Evidence at protein level	Evidence at protein level	Evidence at protein level	Group enriched	Detected in some	5
		Evidence at protein level	Evidence at protein level	Evidence at protein level	Evidence at protein level	Tissue enhanced	Detected in many	
Hydrolase, Protease, Thiol protease		Evidence at protein level	Evidence at protein level	Evidence at protein level	Evidence at protein level	Low tissue specificity	Detected in all	
Hydrolase, Protease, Thiol protease		Evidence at protein level	Evidence at protein level	Evidence at transcript lev	Evidence at transcript lev	Tissue enhanced	Detected in some	
Actin-binding		Evidence at protein level	Evidence at protein level	Evidence at protein level	Evidence at protein level	Low tissue specificity	Detected in all	
Actin-binding		Evidence at protein level	Evidence at protein level	Evidence at protein level	Evidence at protein level	Low tissue specificity	Detected in all	
Actin capping, Actin-binding	Cancer-related genes	Evidence at protein level	Evidence at protein level	Evidence at protein level	Evidence at protein level	Tissue enriched	Detected in many	14
		Evidence at protein level	Evidence at protein level	Evidence at protein level	Evidence at protein level	Low tissue specificity	Detected in all	
Actin-binding	Disease variant	Evidence at protein level	Evidence at protein level	Evidence at protein level	Evidence at protein level	Low tissue specificity	Detected in all	
DNA-binding	Cancer-related genes, Disease variant, Xeroderma pigmentosum	Evidence at protein level	Evidence at protein level	Evidence at protein level	Evidence at protein level	Low tissue specificity	Detected in all	
DNA-binding, Exonuclease, Hydrolase, Nuclease, RNA-binding	Tumor suppressor	Evidence at protein level	Evidence at protein level	Evidence at protein level	Evidence at protein level	Low tissue specificity	Detected in all	
Kinase, Transferase, Tyrosine-protein kinase	FDA approved drug targets, Proto-oncogene	Evidence at protein level	Evidence at protein level	Evidence at protein level	Evidence at protein level	Low tissue specificity	Detected in all	
Hydrolase, Protease, Thiol protease		Evidence at protein level	Evidence at protein level	Evidence at protein level	Evidence at protein level	Tissue enhanced	Detected in all	
Activator, DNA-binding, Repressor		Evidence at protein level	Evidence at protein level	Evidence at protein level	Evidence at protein level	Low tissue specificity	Detected in all	
Acyltransferase, Transferase		Evidence at protein level	Evidence at protein level	Evidence at protein level	Evidence at protein level	Tissue enhanced	Detected in all	
Acyltransferase, Transferase		Evidence at protein level	Evidence at protein level	Evidence at protein level	Evidence at protein level	Low tissue specificity	Detected in all	
Acyltransferase, Transferase		Evidence at protein level	Evidence at protein level	Evidence at protein level	Evidence at protein level	Tissue enriched	Detected in some	5
Acyltransferase, Transferase		Evidence at protein level	Evidence at transcript level	Evidence at protein level	Evidence at protein level	Low tissue specificity	Detected in all	
		Evidence at protein level	Evidence at protein level	Evidence at protein level	Evidence at protein level	Tissue enhanced	Detected in all	
DNA-binding		Evidence at protein level	Evidence at protein level	Evidence at protein level	Evidence at protein level	Low tissue specificity	Detected in all	

RNA tissue specific nTPM	RNA single cell type specificity	RNA single cell type distribution	RNA single cell type specificity score	RNA single cell type specific nCPM
parathyroid gland: 83.6	Low cell type specificity	Detected in all		
	Cell type enhanced	Detected in all		Cytotrophoblasts: 190.5;Syncytiotrophoblasts: 240.4
	Cell type enhanced	Detected in many		Adipocytes: 432.4;Adrenal cortex cells: 862.8;Kupffer cells: 1038.2;Macrophages: 728.9;Mast cells: 577.9;monocytes: 680.4;Neutrophils: 1571.1;Smooth muscle cells: 486.4
lymphoid tissue: 41.5;pituitary gland: 48.5	Cell type enhanced	Detected in many		Cone photoreceptor cells: 289.8;Extravillous trophoblasts: 473.9;Retinal bipolar cells: 235.3;Rod photoreceptor cells: 380.3
adrenal gland: 96.1;intestine: 66.6	Cell type enhanced	Detected in many		Adrenal cortex cells: 747.9;Colonocytes: 256.0;Enterocytes: 630.7;Hematopoietic stem cells: 640.0;Innate lymphoid cells: 147.7;NK-cells: 216.6;Thymocytes: 162.6;Vascular endothelial cells: 507.4
liver: 115.1	Cell type enriched	Detected in many	5	Hepatocytes: 614.1
	Group enriched	Detected in some	7	Astrocytes: 16.8;Bergmann glia: 11.0;Brain excitatory neurons: 15.5;Brain inhibitory neurons: 18.9;Choroid plexus epithelial cells: 9.2;Ependymal cells: 12.7;Oligodendrocyte progenitor cells: 12.3;Oligodendrocytes: 8.7;Other
	Cell type enhanced	Detected in many		Choroid plexus epithelial cells: 183.8;Papillary tip epithelial cells: 73.9;Podocytes: 98.6;Renal collecting duct intercalated cells: 104.3;Renal collecting duct principal cells: 72.1;Renal connecting tubule cells: 67.0
adrenal gland: 58.1;liver: 49.3	Cell type enhanced	Detected in many		Adrenal cortex cells: 816.2;Cholangiocytes: 415.9;Colonocytes: 358.2;Epididymal basal cells: 280.8;Foveolar cells: 490.2;Hepatocytes: 419.5;Pancreatic duct cells: 319.1;Papillary tip epithelial cells: 262.4;Platelets: 720.9
prostate: 38.7	Cell type enhanced	Detected in many		Hematopoietic stem cells: 355.2;Mast cells: 410.3;Megakaryocyte progenitors: 381.3;Megakaryocyte-Erythroid progenitors: 529.2;Microglia: 943.8;Platelets: 443.6;Proximal tubule cells: 323.3;Renal collecting duct intercalate
kidney: 21.7;liver: 82.0	Cell type enriched	Detected in some	7	Proximal tubule cells: 143.0
intestine: 83.2	Group enriched	Detected in many	4	Breast lactating cells: 316.5;Colonocytes: 104.6;Enterocytes: 267.3;Extravillous trophoblasts: 116.4;Hofbauer cells: 298.9;Vascular endothelial cells: 142.0
	Cell type enhanced	Detected in many		Endometrial luminal cells: 59.0;Fibro-adipogenic progenitors: 150.0;Fibroblasts: 192.3;Lymphatic endothelial cells: 174.0;Respiratory ionocytes: 73.7;Schwann cells: 116.4;Vascular smooth muscle cells: 182.8
intestine: 454.0;lymphoid tissue: 228.3	Cell type enhanced	Detected in many		Cardiomyocytes: 439.1;Extravillous trophoblasts: 213.3;Fibro-adipogenic progenitors: 109.6;monocytes: 87.1;Oocytes: 110.5;Parietal cells: 212.1;pDCs: 88.2
	Cell type enhanced	Detected in all		Neutrophils: 589.0
placenta: 126.8	Group enriched	Detected in many	4	Extravillous trophoblasts: 1570.4;Mast cells: 479.7;Syncytiotrophoblasts: 617.3
epididymis: 336.5;stomach 1: 103.8	Cell type enhanced	Detected in many		B-cells: 767.0;cDC: 219.8;Conjunctival goblet cells: 336.9;Epididymal principal cells: 592.4;Foveolar cells: 358.7;Hematopoietic stem cells: 215.9;Macrophages: 243.7;Microglia: 1136.4;Mucous neck cells: 377.1;Pituitary sten
	Cell type enhanced	Detected in many		Breast lactating cells: 277.4;Decidual stromal cells: 122.6;Endometrial stromal cells: 107.0;Leydig cells: 110.9;Pericytes: 335.0;Retinal ganglion cells: 158.4;Somatotrophs: 144.7;Vascular smooth muscle cells: 248.2
	Cell type enhanced	Detected in some		Epicardial cells: 9.1;Foveolar cells: 5.3;Late spermatids: 8.0;Lymphatic endothelial cells: 26.7;Pericytes: 4.2;Vascular endothelial cells: 39.2
skeletal muscle: 48.5	Cell type enhanced	Detected in many		Alveolar cells type 1: 294.6;Choroid plexus epithelial cells: 791.3;Myonuclei: 588.6;Oocytes: 350.0
	Cell type enhanced	Detected in all		Cone photoreceptor cells: 498.9;Rod photoreceptor cells: 990.1
bone marrow: 62.7;brain: 36.1	Cell type enhanced	Detected in many		Brain excitatory neurons: 162.0;Brain inhibitory neurons: 124.1;Early spermatids: 79.9;Erythrocyte progenitors: 246.5;Lactotrophs: 77.6;Late spermatids: 91.3;Other brain neurons: 90.1;Retinal amacrine cells: 86.9;Retinal bipo
	Cell type enhanced	Detected in all		Neutrophil progenitors: 796.6;Neutrophils: 898.0
	Cell type enhanced	Detected in many		Decidual stromal cells: 36.5;Endometrial glandular cells: 35.5;Endometrial luminal cells: 83.1;Endometrial stromal cells: 63.5;Fibro-adipogenic progenitors: 44.2;Fibroblasts: 53.5;Hepatic stellate cells: 51.6;Leydig cells: 84.1;L
brain: 74.7	Cell type enhanced	Detected in many		Astrocytes: 22.1;Bergmann glia: 12.4;Brain excitatory neurons: 47.6;Brain inhibitory neurons: 40.1;Ependymal cells: 17.0;Gonadotrophs: 14.7;Late spermatids: 24.8;Oligodendrocyte progenitor cells: 34.6;Oligodendrocytes: 1
heart muscle: 54.6	Cell type enhanced	Detected in many		Alveolar cells type 2: 133.4;Cardiomyocytes: 196.1;Decidual stromal cells: 74.8;Endometrial stromal cells: 135.6;Epicardial cells: 393.4;Fibro-adipogenic progenitors: 142.3;Fibroblasts: 139.3;Late spermatids: 125.3;Mesotheli
epididymis: 1159.4	Cell type enhanced	Detected in many		Epididymal efferent duct absorptive cells: 887.7;Epididymal efferent duct ciliated cells: 400.4;Epididymal principal cells: 2215.1
brain: 42.6;testis: 20.2	Cell type enhanced	Detected in many		Adipocytes: 20.5;Astrocytes: 30.3;Brain excitatory neurons: 21.9;Breast secretory cells: 19.9;Müller glia: 60.9;Oligodendrocyte progenitor cells: 54.5;Oligodendrocytes: 55.1;Pancreatic duct cells: 30.8;Podocytes: 138.9;Retina
brain: 12.5;urinary bladder: 15.6	Cell type enhanced	Detected in many		Basal keratinocytes: 81.9;Bergmann glia: 41.6;Conjunctival goblet cells: 45.6;Esophageal basal cells: 47.4;Respiratory basal cells: 30.1;Respiratory secretory cells: 34.5;Retinal pigment epithelial cells: 59.7;Suprabasal keratin
blood vessel: 17.2;liver: 11.4;lymphoid tissue: 10.6	Cell type enhanced	Detected in many		Brain excitatory neurons: 25.0;Brain inhibitory neurons: 23.8;Epicardial cells: 27.3;Fibro-adipogenic progenitors: 51.2;Hepatic stellate cells: 116.2;Hepatocytes: 60.7;Mesothelial cells: 37.7;Other brain neurons: 30.5;Proximal t
breast: 32.4;skin 1: 36.9	Cell type enhanced	Detected in many		Alveolar cells type 1: 175.2;Basal keratinocytes: 326.3;Breast lactating cells: 230.8;Conjunctival goblet cells: 70.9;Mast cells: 138.8;Ocular epithelial cells: 152.7;Suprabasal keratinocytes: 184.6
	Cell type enhanced	Detected in many		Endometrial ciliated cells: 840.1;Endometrial glandular cells: 1110.5;Esophageal apical cells: 1778.4
lung: 687.2	Cell type enriched	Detected in many	19	Alveolar cells type 1: 1356.7
	Cell type enhanced	Detected in many		Esophageal apical cells: 2909.8;Esophageal suprabasal cells: 1288.7;Schwann cells: 1021.9;Suprabasal keratinocytes: 1000.4
skin 1: 294.4	Cell type enhanced	Detected in many		Basal keratinocytes: 146.7;Esophageal apical cells: 425.7;Esophageal basal cells: 108.3;Esophageal suprabasal cells: 298.7;Ocular epithelial cells: 114.6;Salivary duct cells: 122.6;Smooth muscle cells: 134.8;Suprabasal kera
	Cell type enhanced	Detected in many		Late primary spermatocytes: 750.0;Lymphatic endothelial cells: 1618.9;Melanocytes: 615.6;Müller glia: 1424.2;Ovarian stromal cells: 912.6;Pericytes: 619.1;Retinal ganglion cells: 571.7;Schwann cells: 958.6;Sertoli cells: 794.
esophagus: 211.0;intestine: 577.8;stomach 1: 408.0	Cell type enhanced	Detected in many		Basal keratinocytes: 226.2;Cholangiocytes: 145.7;Colonocytes: 167.6;Enterocytes: 304.0;Esophageal apical cells: 141.7;Esophageal basal cells: 569.2;Esophageal suprabasal cells: 880.0;Foveolar cells: 853.4;Suprabasal ker
breast: 156.9	Cell type enhanced	Detected in some		Adipocytes: 3.0;Early spermatids: 3.3;Esophageal apical cells: 4.8;Esophageal suprabasal cells: 3.8;Granulosa cells: 3.2;Late spermatids: 5.9;Myonuclei: 2.6;Myosatellite cells: 8.0;Parietal cells: 3.5;Suprabasal keratinocytes:
	Cell type enhanced	Detected in many		Early spermatids: 225.1;Esophageal apical cells: 129.8;Late spermatids: 494.6
brain: 1.9;pituitary gland: 2.4;testis: 1.5	Cell type enhanced	Detected in many		Adrenal medulla cells: 138.3;Brain inhibitory neurons: 202.5;Early spermatids: 228.9;Lactotrophs: 1017.9;Late spermatids: 260.6;Oligodendrocyte progenitor cells: 306.6;Retinal amacrine cells: 128.4;Salivary ionocytes: 417.3
breast: 105.2;prostate: 68.6	Cell type enhanced	Detected in some		Alveolar cells type 2: 250.9;Breast hormone-responsive cells: 50.9;Conjunctival goblet cells: 71.8;Esophageal apical cells: 70.6;Esophageal basal cells: 46.9;Esophageal suprabasal cells: 127.5;Kupffer cells: 30.7;Macrophage
tongue: 14.3	Cell type enhanced	Detected in many		Adrenal medulla cells: 10.6;Epididymal efferent duct absorptive cells: 8.5;Thymic myoid cells: 10.9
brain: 35.0;retina: 23.9	Group enriched	Detected in some	4	Astrocytes: 131.8;Bergmann glia: 344.0;Müller glia: 278.9;Oligodendrocytes: 358.9;Retinal horizontal cells: 106.2
epididymis: 137.7;tongue: 51.2	Cell type enhanced	Detected in many		Astrocytes: 93.6;Cytotrophoblasts: 83.2;Epididymal principal cells: 323.7;Myonuclei: 83.7;Pituitary stem cells: 403.0;Prostatic glandular cells: 67.1
retina: 118.1;skeletal muscle: 120.2	Cell type enhanced	Detected in many		Astrocytes: 1654.3;Bergmann glia: 6456.2;Brain excitatory neurons: 1399.6;Brain inhibitory neurons: 1228.1;Ependymal cells: 1554.5;Myonuclei: 1069.4;Oligodendrocytes: 1582.7;Other brain neurons: 1483.3;Retinal ganglion
	Cell type enhanced	Detected in many		Alveolar cells type 2: 1895.9;Oligodendrocytes: 2382.8;Renal collecting duct principal cells: 2941.0;Retinal bipolar cells: 1780.3
	Cell type enhanced	Detected in all		Oligodendrocytes: 684.0
	Cell type enhanced	Detected in many		Adrenal cortex cells: 126.6
	Cell type enhanced	Detected in many		Late spermatids: 258.2;Neutrophil progenitors: 229.4;Neutrophils: 571.7
brain: 44.3	Cell type enhanced	Detected in some		Endometrial stromal cells: 1.1;Late spermatids: 3.0
epididymis: 81.3;seminal vesicle: 83.9	Cell type enhanced	Detected in many		Breast myoepithelial cells: 243.7;Early spermatids: 195.2;Epididymal efferent duct absorptive cells: 362.8;Epididymal efferent duct ciliated cells: 101.6;Epididymal principal cells: 229.9;Foveolar cells: 164.3;Hepatocytes: 411.8
	Cell type enhanced	Detected in all		Neutrophil progenitors: 715.2;Neutrophils: 371.8
retina: 190.9	Cell type enhanced	Detected in many		Cone photoreceptor cells: 1062.0;Late spermatids: 547.2;Rod photoreceptor cells: 2368.4;Vascular endothelial cells: 819.0
	Cell type enhanced	Detected in many		Adipocytes: 912.9;Myonuclei: 612.6;Platelets: 1145.3
intestine: 2245.0;pancreas: 643.6	Cell type enriched	Detected in many	9	Enterocytes: 6494.6
esophagus: 7699.2	Cell type enriched	Detected in many	4	Esophageal apical cells: 89658.7
gallbladder: 135.6;intestine: 275.9	Cell type enhanced	Detected in many		Cholangiocytes: 160.6;Enteric transient amplifying cells: 66.9;Enterocytes: 128.6;Epididymal efferent duct ciliated cells: 164.5;Fallopian tube ciliated cells: 183.5;Gastric progenitor cells: 53.8;Goblet cells: 173.1;Paneth cells: 6
bone marrow: 319.2	Cell type enhanced	Detected in many		Alveolar cells type 1: 816.7;Breast myoepithelial cells: 341.9;Esophageal apical cells: 750.9;Megakaryocytes: 463.6;Migrating cytotrophoblasts: 329.8;Neutrophil progenitors: 1295.8;Neutrophils: 784.3;Pancreatic duct cells: 3
	Cell type enhanced	Detected in many		Decidual stromal cells: 497.5;Hofbauer cells: 948.1;Late spermatids: 539.6;Platelets: 520.9;Syncytiotrophoblasts: 600.4
brain: 192.4	Cell type enhanced	Detected in many		Brain excitatory neurons: 75.7;Oligodendrocyte progenitor cells: 73.7;Oligodendrocytes: 151.7
lymphoid tissue: 135.3	Cell type enhanced	Detected in many		Adipocytes: 600.2;Kupffer cells: 435.6;Macrophages: 333.6;Microglia: 2215.0;Monocyte progenitors: 346.9;monocytes: 439.8;Neutrophil progenitors: 864.7;Neutrophils: 2909.8;NK-cells: 353.3;pDCs: 368.0;T-cells: 420.4
brain: 38.7	Cell type enhanced	Detected in all		Astrocytes: 738.9;Bergmann glia: 719.6;Ependymal cells: 304.0;Neutrophils: 301.6;Retinal bipolar cells: 296.1
bone marrow: 32.7;intestine: 24.9;lung: 11.1;lymphoid tissue: 35.2	Cell type enhanced	Detected in some		cDC: 32.5;Colonocytes: 111.2;Goblet cells: 55.4;Hofbauer cells: 33.9;Macrophages: 29.7;Monocyte progenitors: 44.6;monocytes: 54.3;Neutrophil progenitors: 54.6;Neutrophils: 261.5;NK-cells: 32.3;Tuft cells: 46.1
adipose tissue: 116.6	Cell type enhanced	Detected in many		Bergmann glia: 117.8;Choroid plexus epithelial cells: 137.7;Lymphatic endothelial cells: 254.4;Monocyte progenitors: 171.7;Müller glia: 240.5;Pericytes: 300.8;Retinal pigment epithelial cells: 238.7;Vascular endothelial cells: 3
	Low cell type specificity	Detected in many		
blood vessel: 765.7;choroid plexus: 764.5	Cell type enhanced	Detected in many		Alveolar cells type 1: 466.6;Alveolar cells type 2: 430.8;Cholangiocytes: 372.7;Mesothelial cells: 247.4;Pancreatic duct cells: 346.2;Smooth muscle cells: 141.5;Transitional alveolar cells: 126.2;Vascular endothelial cells: 740.7
intestine: 64.5	Cell type enriched	Detected in some	30	Platelets: 565.8
adrenal gland: 14.1;intestine: 27.0;liver: 15.1	Cell type enhanced	Detected in many		Enterocytes: 61.7;Hepatocytes: 50.0;Late primary spermatocytes: 86.0;Late spermatids: 104.7;Müller glia: 48.6
esophagus: 1457.5;skin 1: 554.4;urinary bladder: 642.4	Cell type enhanced	Detected in many		Basal keratinocytes: 1612.8;Esophageal apical cells: 4827.1;Esophageal basal cells: 3864.2;Esophageal suprabasal cells: 7328.2;Ocular epithelial cells: 1729.1;Prostatic hillock cells: 1115.0;Respiratory basal cells: 1096.3;Re
brain: 354.4;lung: 108.3	Cell type enhanced	Detected in many		Alveolar cells type 1: 568.4;Alveolar cells type 2: 318.0;Astrocytes: 742.9;Bergmann glia: 125.9;Ependymal cells: 261.0;Müller glia: 226.5;Parietal cells: 123.8;Transitional alveolar cells: 392.4
	Low cell type specificity	Detected in many		
bone marrow: 75.1;lymphoid tissue: 70.0	Group enriched	Detected in many	4	Neutrophil progenitors: 5134.5;Neutrophils: 10528.3
	Cell type enhanced	Detected in many		Astrocytes: 47.8;Bergmann glia: 47.4;Brain excitatory neurons: 45.9;Choroid plexus epithelial cells: 81.7;Distal convoluted tubule cells: 62.1;Loop of henle epithelial cells: 49.6;Microglia: 214.0;Oligodendrocyte progenitor cells
lymphoid tissue: 19.9	Cell type enhanced	Detected in some		Differentiating spermatogonia: 1.4;Early primary spermatocytes: 2.1;Early spermatids: 3.0;Erythrocyte progenitors: 3.6;Erythrocytes: 3.0;Late primary spermatocytes: 1.3;Late spermatids: 2.8;Monocyte progenitors: 2.8;Neutro
brain: 74.7	Cell type enhanced	Detected in all		Bergmann glia: 573.7;Oligodendrocytes: 1074.9;Platelets: 596.7
brain: 36.7	Cell type enriched	Detected in many	4	Microglia: 1230.8
bone marrow: 64.4;intestine: 49.3;lung: 37.7;lymphoid tissue: 100.9	Cell type enhanced	Detected in many		B-cells: 85.9;Breast lactating cells: 83.3;Epicardial cells: 156.9;Extravillous trophoblasts: 81.0;Megakaryocytes: 105.1;Monocyte progenitors: 90.0;monocytes: 90.3;Neutrophils: 194.5;NK-cells: 93.1;Oocytes: 184.2;Platelets: 3
lymphoid tissue: 165.2	Cell type enhanced	Detected in many		B-cells: 100.9;Neutrophils: 145.1;NK-cells: 165.3;T-cells: 141.9
	Cell type enhanced	Detected in many		Cone photoreceptor cells: 486.5;Tuft cells: 299.7
adipose tissue: 44.7;breast: 40.8	Group enriched	Detected in some	4	Lymphatic endothelial cells: 23.2;Vascular endothelial cells: 74.5
	Cell type enhanced	Detected in many		B-cells: 600.2;Esophageal apical cells: 269.8;Monocyte progenitors: 492.4;Neutrophil progenitors: 402.1;Neutrophils: 336.1;Thymocytes: 626.7
	Group enriched	Detected in many	6	Astrocytes: 341.7;Bergmann glia: 389.7;Choroid plexus epithelial cells: 306.7;Ependymal cells: 260.7;Podocytes: 836.0
skin 1: 45.2	Cell type enhanced	Detected in many		Distal convoluted tubule cells: 8.9;Foveolar cells: 8.0;Loop of henle epithelial cells: 15.5;Ocular epithelial cells: 13.7;Papillary tip epithelial cells: 8.8;Proximal tubule cells: 12.8;Renal collecting duct intercalated cells: 12.7;Rena
bone marrow: 61.3;lymphoid tissue: 68.9	Cell type enhanced	Detected in many		cDC: 308.9;Endometrial luminal cells: 266.3;Innate lymphoid cells: 186.0;Kupffer cells: 423.3;Macrophages: 271.9;Mast cells: 220.3;NK-cells: 439.9;pDCs: 322.8;T-cells: 323.8
fallopian tube: 7.7;testis: 10.9	Cell type enhanced	Detected in many		Astrocytes: 121.3;Choroid plexus epithelial cells: 126.7;Ependymal cells: 225.8;Late spermatids: 90.6;Respiratory ciliated cells: 239.3;Thymocytes: 87.2
bone marrow: 249.0;lymphoid tissue: 157.8	Cell type enhanced	Detected in many		cDC: 191.8;Hofbauer cells: 396.4;Kupffer cells: 251.1;Macrophages: 199.1;Microglia: 149.9;Monocyte progenitors: 134.9;monocytes: 334.4;Neutrophil progenitors: 241.3;Neutrophils: 933.6
brain: 75.7	Cell type enhanced	Detected in many		Colonocytes: 84.6;Epididymal principal cells: 122.5;Parietal cells: 132.6;Podocytes: 243.4
brain: 52.5	Cell type enhanced	Detected in many		Astrocytes: 36.6;Epididymal principal cells: 126.6
testis: 43.7	Cell type enhanced	Detected in many		Distal convoluted tubule cells: 463.9;Lactotrophs: 534.7;Megakaryocytes: 352.4;Platelets: 355.5;Renal connecting tubule cells: 617.6;Somatotrophs: 526.7;Thyrotrophs: 358.0
liver: 559.0	Cell type enriched	Detected in some	6	Hepatocytes: 951.2
	Cell type enhanced	Detected in all		Sertoli cells: 143.4
brain: 30.0	Cell type enhanced	Detected in some		Astrocytes: 43.4;Brain excitatory neurons: 11.9;Brain inhibitory neurons: 32.6;Choroid plexus epithelial cells: 21.7;Oligodendrocyte progenitor cells: 106.3;Oligodendrocytes: 30.2;Other brain neurons: 19.2;Tuft cells: 11.5
	Cell type enhanced	Detected in many		Early spermatids: 767.9;Late primary spermatocytes: 649.2;Neutrophils: 385.0;Oocytes: 327.0
	Cell type enhanced	Detected in many		Choroid plexus epithelial cells: 211.4;Leydig cells: 223.3;Pituicytes/FSCs: 206.0;Podocytes: 247.8;Syncytiotrophoblasts: 614.2
esophagus: 10.5;kidney: 5.0;lymphoid tissue: 13.9;placenta: 5.2;ski	Group enriched	Detected in some	5	Respiratory deuterosomal cells: 145.1;Respiratory secretory cells: 184.7
	Cell type enhanced	Detected in all		Lactotrophs: 1312.9;Late spermatids: 1625.2;monocytes: 1867.4;Somatotrophs: 3948.2;Vascular smooth muscle cells: 1251.8
testis: 6.0	Group enriched	Detected in many	4	Early spermatids: 58.8;Late primary spermatocytes: 82.6
bone marrow: 89.1	Cell type enhanced	Detected in all		Brain inhibitory neurons: 761.9;cDC: 982.0;monocytes: 2191.5;Neutrophils: 2144.2
choroid plexus: 106.2	Cell type enriched	Detected in some	6	Choroid plexus epithelial cells: 197.4
smooth muscle: 164.7	Cell type enhanced	Detected in many		Alveolar cells type 1: 486.4;Basal keratinocytes: 496.5;Smooth muscle cells: 476.1;Suprabasal keratinocytes: 691.2
	Cell type enhanced	Detected in many		Hematopoietic stem cells: 126.2;Mast cells: 178.8;Megakaryocyte progenitors: 168.3;Megakaryocyte-Erythroid progenitors: 174.4
	Cell type enriched	Detected in many	4	Esophageal apical cells: 1788.1
brain: 17.8;pituitary gland: 13.6;retina: 13.7	Cell type enhanced	Detected in many		Brain excitatory neurons: 814.7;Brain inhibitory neurons: 644.7;Cone photoreceptor cells: 467.8;Corticotrophs: 656.6;Gonadotrophs: 1438.7;Lactotrophs: 936.6;Other brain neurons: 711.3;Retinal ganglion cells: 969.6;Rod ph
intestine: 71.4	Cell type enhanced	Detected in many		Foveolar cells: 566.4;Prostatic club cells: 660.3;Prostatic hillock cells: 589.2;Urothelial cells: 1235.8
	Cell type enhanced	Detected in many		Choroid plexus epithelial cells: 56.0;Plasma cells: 51.3;Sertoli cells: 113.2
	Cell type enhanced	Detected in many		Decidual stromal cells: 293.9;Epididymal basal cells: 195.9;Fibro-adipogenic progenitors: 206.2;Fibroblasts: 236.4;Granulosa cells: 223.8;Hepatic stellate cells: 245.3;Kupffer cells: 591.4;Macrophages: 201.4;Pericytes: 323.7;
	Cell type enhanced	Detected in many		cDC: 3645.2;Decidual stromal cells: 4907.0;Enterocytes: 5902.7;Kupffer cells: 5841.0;Plasma cells: 3861.8;Platelets: 5107.2;Suprabasal keratinocytes: 3628.6;T-cells: 3310.0
brain: 113.1;pancreas: 243.6	Cell type enhanced	Detected in many		Cone photoreceptor cells: 180.1;Early spermatids: 124.6;Late spermatids: 587.5;Oligodendrocytes: 148.0;Pancreatic acinar cells: 126.3
	Cell type enhanced	Detected in many		Alveolar cells type 2: 274.0;Esophageal apical cells: 653.9
stomach 1: 55.5	Cell type enhanced	Detected in many		Foveolar cells: 734.9;Oocytes: 584.8;Pancreatic acinar cells: 541.4;Papillary tip epithelial cells: 425.5;Urothelial cells: 786.5
brain: 379.7;epididymis: 286.2	Cell type enriched	Detected in many	4	Neutrophils: 6905.2
urinary bladder: 38.4	Cell type enhanced	Detected in some		Brain excitatory neurons: 4.4;Choroid plexus epithelial cells: 2.2;Enterocytes: 1.0;Ependymal cells: 5.5;Esophageal apical cells: 1.6;Oligodendrocytes: 2.8
	Cell type enhanced	Detected in many		Alveolar cells type 1: 21.1;Alveolar cells type 2: 16.8;Cardiomyocytes: 12.5;Transitional alveolar cells: 16.6;Undifferentiated spermatogonia: 29.2
bone marrow: 95.7;lymphoid tissue: 48.5	Cell type enhanced	Detected in many		Cytotrophoblasts: 153.0;Extravillous trophoblasts: 293.3;Megakaryocytes: 531.6;Microglia: 175.4;monocytes: 163.2;Neutrophil progenitors: 389.9;Neutrophils: 547.9;NK-cells: 339.3;Platelets: 1232.5;Syncytiotrophoblasts: 19
lymphoid tissue: 52.9	Cell type enhanced	Detected in many		B-cells: 211.3;Distal convoluted tubule cells: 301.5;Epididymal clear cells: 210.3;Esophageal apical cells: 670.8;Microglia: 703.1;Ocular epithelial cells: 249.0;pDCs: 330.4;Plasma cells: 208.7;Renal collecting duct intercalated
	Cell type enhanced	Detected in many		Vascular endothelial cells: 1029.0
epididymis: 96.9	Cell type enriched	Detected in many	4	Tuft cells: 1037.5
	Cell type enhanced	Detected in many		Bergmann glia: 146.4;Choroid plexus epithelial cells: 125.3;Ependymal cells: 186.0;Fibro-adipogenic progenitors: 394.2;Leydig cells: 186.7;Mesothelial cells: 140.9;Pituicytes/FSCs: 645.9;Pituitary stem cells: 435.7;Retinal pig
	Cell type enhanced	Detected in all		Neutrophils: 2034.3
bone marrow: 24.2;lymphoid tissue: 62.7	Cell type enhanced	Detected in many		B-cells: 81.0;cDC: 73.7;Hofbauer cells: 75.2;Kupffer cells: 64.6;Macrophages: 66.5;Mast cells: 158.0;Megakaryocyte progenitors: 89.9;Megakaryocyte-Erythroid progenitors: 61.8;Megakaryocytes: 309.1;Microglia: 60.1;mono
heart muscle: 26.0;skeletal muscle: 41.0;tongue: 27.3	Cell type enhanced	Detected in many		Cardiomyocytes: 46.0;Early spermatids: 30.7;Late primary spermatocytes: 57.0;Late spermatids: 93.9;Myonuclei: 119.5;Myosatellite cells: 22.1;Pituicytes/FSCs: 25.8;Smooth muscle cells: 23.9
	Cell type enhanced	Detected in some		Astrocytes: 1.5;Brain excitatory neurons: 1.8;Choroid plexus epithelial cells: 5.0;Ependymal cells: 1.7;Oligodendrocytes: 1.0
esophagus: 98.1;lung: 68.8;stomach 1: 55.4	Cell type enhanced	Detected in many		Alveolar cells type 1: 244.4;Esophageal apical cells: 748.6;Esophageal suprabasal cells: 127.9;Foveolar cells: 126.2;Ocular epithelial cells: 379.8;Transitional alveolar cells: 137.1;Urothelial cells: 220.3
	Cell type enhanced	Detected in many		Esophageal basal cells: 790.0;Esophageal suprabasal cells: 627.3;Extravillous trophoblasts: 453.8;Gastric progenitor cells: 552.4;Migrating cytotrophoblasts: 544.9
	Cell type enhanced	Detected in many		Enterocytes: 32.5
liver: 464.3	Cell type enriched	Detected in some	33	Hepatocytes: 385.0

stomach 1: 293.9	Cell type enhanced	Detected in some		Cholangiocytes: 54.5;Early spermatids: 334.7;Foveolar cells: 82.8;Gastric chief cells: 287.2;Late primary spermatocytes: 43.3;Mucous neck cells: 80.2;Pancreatic duct cells: 35.2;Parietal cells: 107.8
intestine: 25.0	Cell type enhanced	Detected in some		Astrocytes: 180.2;Bergmann glia: 163.4;Brain excitatory neurons: 661.1;Brain inhibitory neurons: 711.5;Choroid plexus epithelial cells: 147.0;Oligodendrocyte progenitor cells: 284.3;Other brain neurons: 504.7;Pericytes: 220.8
retina: 266.5	Cell type enhanced	Detected in many		Alveolar cells type 1: 1114.7;Astrocytes: 1468.7;Cone photoreceptor cells: 1409.7;Müller glia: 1212.3;Retinal bipolar cells: 2456.0;Rod photoreceptor cells: 4758.6;Sertoli cells: 2066.8
adipose tissue: 66.3;lung: 72.4	Cell type enhanced	Detected in many		Adipocytes: 379.9;Bergmann glia: 332.9;Endometrial stromal cells: 222.1;Fibro-adipogenic progenitors: 158.1;Lymphatic endothelial cells: 598.3;Oligodendrocyte progenitor cells: 378.2;Pituicytes/FSCs: 176.1;Vascular endoth
	Cell type enhanced	Detected in many		Breast myoepithelial cells: 2360.2;Decidual stromal cells: 2069.1;Extravillous trophoblasts: 1516.8;Hepatic stellate cells: 4298.9;Leydig cells: 2586.7;Ovarian stromal cells: 1689.0;Pericytes: 2893.8;Peritubular myoid cells: 246
	Cell type enhanced	Detected in many		Astrocytes: 948.4;Bergmann glia: 1307.7;Sertoli cells: 1755.7;Tuft cells: 645.6
	Cell type enhanced	Detected in many		Lymphatic endothelial cells: 43.3;Müller glia: 26.2;Oligodendrocyte progenitor cells: 29.7;Vascular endothelial cells: 75.1
	Cell type enhanced	Detected in all		Esophageal apical cells: 3885.9;Ocular epithelial cells: 2239.1
	Cell type enhanced	Detected in many		Alveolar cells type 1: 483.2;Breast myoepithelial cells: 378.9;Hepatic stellate cells: 568.0;Leydig cells: 266.7;Lymphatic endothelial cells: 341.5;Pericytes: 311.9;Peritubular myoid cells: 412.9;Smooth muscle cells: 433.8;Vascu
adipose tissue: 154.8	Group enriched	Detected in many	4	Megakaryocytes: 1873.0;Platelets: 5719.2
skeletal muscle: 258.4;tongue: 86.3	Group enriched	Detected in many	4	Myonuclei: 172.6;Thymic myoid cells: 488.1
	Cell type enhanced	Detected in many		Esophageal apical cells: 239.3
	Cell type enhanced	Detected in many		Esophageal apical cells: 298.9;Esophageal suprabasal cells: 181.9;Late spermatids: 181.5
brain: 73.0	Cell type enhanced	Detected in many		Epicardial cells: 163.7;Esophageal apical cells: 101.3;Podocytes: 127.0;Rod photoreceptor cells: 94.5
brain: 65.7	Cell type enhanced	Detected in many		Early primary spermatocytes: 518.3;Early spermatids: 952.9;Late primary spermatocytes: 485.1;Late spermatids: 1269.6;Monocyte progenitors: 528.8;Oligodendrocytes: 952.9
lymphoid tissue: 37.7	Cell type enhanced	Detected in many		B-cells: 176.3;Endometrial ciliated cells: 152.2;NK-cells: 249.0;Pituitary stem cells: 169.2;Plasma cells: 246.1;Respiratory ciliated cells: 246.3;Respiratory deuterosomal cells: 160.7;T-cells: 219.9
	Cell type enhanced	Detected in many		Adrenal cortex cells: 217.5;Neutrophils: 258.0
gallbladder: 27.3;stomach 1: 15.6	Group enriched	Detected in some	5	Gastric chief cells: 31.7;Mucous neck cells: 25.4
brain: 21.1;pancreas: 25.5;stomach 1: 41.3	Group enriched	Detected in some	4	Gastric chief cells: 53.5;Granulosa cells: 72.2;Mucous neck cells: 45.8;Neuroendocrine cells: 32.3;Parietal cells: 83.1
	Cell type enhanced	Detected in many		Colonocytes: 189.2;Epicardial cells: 200.1;Esophageal apical cells: 170.9;Mesothelial cells: 265.2;Ocular epithelial cells: 181.8
lymphoid tissue: 23.1	Group enriched	Detected in some	4	cDC: 22.8;Monocyte progenitors: 87.5;monocytes: 65.3;pDCs: 73.3;Plasma cells: 32.2
lymphoid tissue: 12.1;urinary bladder: 5.1	Group enriched	Detected in some	7	Innate lymphoid cells: 13.6;Platelets: 32.5;T-cells: 17.6
	Cell type enhanced	Detected in some		cDC: 22.4;Enterocytes: 16.0;Goblet cells: 19.8;Hofbauer cells: 38.6;Kupffer cells: 18.4;Macrophages: 36.3;monocytes: 116.2;Neutrophils: 65.8;Paneth cells: 15.5;Platelets: 19.8
parathyroid gland: 310.7	Cell type enhanced	Detected in many		Ependymal cells: 127.3;Hematopoietic stem cells: 391.1;Hofbauer cells: 172.0;Macrophages: 103.5;Megakaryocyte progenitors: 118.2;Megakaryocyte-Erythroid progenitors: 200.6;monocytes: 122.3;Ocular epithelial cells: 19
lymphoid tissue: 224.8	Cell type enhanced	Detected in many		Hofbauer cells: 359.6;Kupffer cells: 2041.6;Macrophages: 893.3;Monocyte progenitors: 188.9;monocytes: 738.4;Neutrophils: 539.2
lymphoid tissue: 21.8	Cell type enhanced	Detected in some		B-cells: 47.7;Breast lactating cells: 57.6;cDC: 17.7;Epicardial cells: 22.7;Hofbauer cells: 150.3;Kupffer cells: 34.8;Macrophages: 26.6;Monocyte progenitors: 33.2;monocytes: 19.0;pDCs: 28.9;Plasma cells: 50.2
lymphoid tissue: 232.5	Group enriched	Detected in some	6	Innate lymphoid cells: 131.5;NK-cells: 142.3;T-cells: 525.2
lymphoid tissue: 7.5	Cell type enhanced	Detected in many		cDC: 66.7;Conjunctival goblet cells: 19.4;Hofbauer cells: 83.7;Innate lymphoid cells: 30.5;Kupffer cells: 16.4;Macrophages: 34.1;Mast cells: 35.2;Neutrophil progenitors: 78.8;Platelets: 42.5;T-cells: 29.6
lymphoid tissue: 19.0;parathyroid gland: 58.7	Cell type enriched	Detected in many	6	Platelets: 1418.7
lung: 16.1	Cell type enhanced	Detected in many		cDC: 33.1;Epididymal basal cells: 27.1;Extravillous trophoblasts: 48.3;Mast cells: 61.3;Megakaryocyte progenitors: 55.8;Microglia: 30.2;Neutrophils: 34.0;Syncytiotrophoblasts: 72.9
lymphoid tissue: 28.7;placenta: 13.9	Group enriched	Detected in some	6	Hofbauer cells: 112.8;T-cells: 147.1;Thymocytes: 50.8
	Cell type enhanced	Detected in all		Enterocytes: 647.5;pDCs: 811.4
bone marrow: 30.6;lymphoid tissue: 22.6	Cell type enhanced	Detected in some		cDC: 68.5;Hofbauer cells: 120.5;Kupffer cells: 195.8;Macrophages: 53.1;Mast cells: 46.8;Microglia: 25.2;Monocyte progenitors: 54.4;monocytes: 78.6
lymphoid tissue: 58.9	Cell type enhanced	Detected in many		Alveolar cells type 2: 162.3;Astrocytes: 200.6;Hematopoietic stem cells: 196.5;Kupffer cells: 137.3;Megakaryocyte progenitors: 177.9;Megakaryocyte-Erythroid progenitors: 278.9;Monocyte progenitors: 135.3;Myonuclei: 93.6
lymphoid tissue: 596.2	Cell type enhanced	Detected in some		Innate lymphoid cells: 58.7;NK-cells: 137.0;T-cells: 424.7;Thymocytes: 83.8
lymphoid tissue: 88.1	Cell type enriched	Detected in some	8	T-cells: 299.5
liver: 143.1;lymphoid tissue: 204.2;parathyroid gland: 163.6	Cell type enhanced	Detected in many		cDC: 136.3;Hofbauer cells: 224.0;Kupffer cells: 558.6;Macrophages: 175.1;Microglia: 130.8;monocytes: 112.5;pDCs: 315.1
lymphoid tissue: 52.9	Cell type enhanced	Detected in many		B-cells: 100.5;cDC: 102.8;Epididymal basal cells: 152.2;Vascular endothelial cells: 61.7
lymphoid tissue: 14.1	Cell type enhanced	Detected in some		Innate lymphoid cells: 20.5;Late spermatids: 10.9;Megakaryocyte-Erythroid progenitors: 9.7;Platelets: 35.3;Respiratory ionocytes: 5.1;T-cells: 61.2
salivary gland: 469.7	Cell type enhanced	Detected in many		Basal keratinocytes: 1692.5;cDC: 1408.4;Mast cells: 1993.4;monocytes: 2581.0;Neutrophils: 1651.0;Ocular epithelial cells: 1190.5;Pancreatic acinar cells: 2586.0;Pancreatic duct cells: 1846.1;Suprabasal keratinocytes: 1644.
	Cell type enhanced	Detected in all		Alveolar cells type 1: 618.0
lymphoid tissue: 247.5	Cell type enriched	Detected in some	7	B-cells: 18.6
lymphoid tissue: 22.6	Cell type enhanced	Detected in some		cDC: 74.1;Hofbauer cells: 91.4;Kupffer cells: 361.8;Macrophages: 165.7;Mast cells: 209.6;Megakaryocyte progenitors: 135.3;Megakaryocyte-Erythroid progenitors: 95.8;Megakaryocytes: 55.6;Microglia: 180.1;Monocyte prog
lymphoid tissue: 32.5	Group enriched	Detected in some	4	cDC: 462.0;Hofbauer cells: 272.7;Kupffer cells: 260.3;Macrophages: 338.1;Microglia: 365.5;monocytes: 296.9
lymphoid tissue: 216.0	Cell type enriched	Detected in many	6	T-cells: 249.0
lymphoid tissue: 427.3	Group enriched	Detected in some	9	Endometrial secretory cells: 46.3;T-cells: 153.6
	Cell type enhanced	Detected in many		Esophageal apical cells: 6751.8;Esophageal basal cells: 1957.6;Esophageal suprabasal cells: 3292.3;Respiratory ionocytes: 2532.4;Respiratory secretory cells: 1893.0
	Low cell type specificity	Detected in many		
	Cell type enhanced	Detected in many		Breast secretory cells: 143.2;Cholangiocytes: 147.6;Esophageal apical cells: 379.4;Esophageal basal cells: 129.7;Esophageal suprabasal cells: 126.6;Extravillous trophoblasts: 157.1;Fallopian secretory cells: 173.8;Gastric ch
	Cell type enhanced	Detected in many		Mast cells: 314.0;Melanocytes: 563.7;Neutrophils: 1010.4;Podocytes: 296.3;Thymic myoid cells: 315.4
	Cell type enhanced	Detected in all		Esophageal apical cells: 585.5;Neutrophils: 679.9;NK-cells: 268.3
parathyroid gland: 186.0	Cell type enhanced	Detected in many		Endometrial glandular cells: 1175.0;Endometrial luminal cells: 763.8;Ocular epithelial cells: 1009.0;Pancreatic acinar cells: 821.7;Urothelial cells: 1358.8
blood vessel: 140.5	Cell type enhanced	Detected in many		Basal keratinocytes: 363.9;Brain inhibitory neurons: 343.2;Cardiomyocytes: 1834.5;Myonuclei: 2264.9;Myosatellite cells: 612.5;Oligodendrocyte progenitor cells: 596.9;Salivary myoepithelial cells: 366.3;Thymic myoid cells: 1
brain: 79.7;skeletal muscle: 54.1	Group enriched	Detected in some	7	Brain excitatory neurons: 23.2;Myonuclei: 90.1;Myosatellite cells: 82.8;Thymic myoid cells: 85.7
intestine: 295.7	Cell type enhanced	Detected in some		Colonocytes: 702.7;Enteric stem cells: 219.3;Enteric transient amplifying cells: 327.1;Enterocytes: 556.2;Gastric progenitor cells: 184.6;Goblet cells: 268.8;Neuroendocrine cells: 293.0;Paneth cells: 139.2;Tuft cells: 219.0
heart muscle: 183.5	Cell type enhanced	Detected in many		Adrenal cortex cells: 850.4;Astrocytes: 308.9;Brain excitatory neurons: 291.5;Cardiomyocytes: 1145.4;Cone photoreceptor cells: 714.2;Ependymal cells: 382.0;Müller glia: 385.2;Oligodendrocyte progenitor cells: 309.6;Pituicy
prostate: 7.5	Cell type enhanced	Detected in many		Esophageal apical cells: 59.0;Prostatic glandular cells: 72.3;Prostatic hillock cells: 72.7;Respiratory basal cells: 209.8;Respiratory ciliated cells: 75.3;Respiratory deuterosomal cells: 188.7;Respiratory secretory cells: 205.5
choroid plexus: 51.8;ovary: 32.3	Cell type enhanced	Detected in many		Basal keratinocytes: 297.9;Choroid plexus epithelial cells: 127.2;Esophageal basal cells: 202.2;Granulosa cells: 350.3;Melanocytes: 242.5;Ocular epithelial cells: 148.7;Respiratory basal cells: 361.2;Retinal pigment epithelial c
brain: 12.7;lymphoid tissue: 3.4;ovary: 5.9;skeletal muscle: 3.5	Cell type enhanced	Detected in many		Astrocytes: 217.7;Bergmann glia: 322.4;Brain excitatory neurons: 97.3;Brain inhibitory neurons: 166.8;Ependymal cells: 107.7;Müller glia: 180.8;Myosatellite cells: 569.7;Oligodendrocyte progenitor cells: 181.5;Other brain neu
placenta: 221.7	Cell type enhanced	Detected in some		Early spermatids: 49.3;Extravillous trophoblasts: 69.0;Kupffer cells: 101.5;Late spermatids: 143.2;Lymphatic endothelial cells: 168.1;Migrating cytotrophoblasts: 47.0;Syncytiotrophoblasts: 126.4;Vascular endothelial cells: 243
blood vessel: 12.2;kidney: 13.8	Cell type enhanced	Detected in many		Brain inhibitory neurons: 71.3;Cholangiocytes: 101.7;Epididymal efferent duct absorptive cells: 102.8;Erythrocyte progenitors: 159.5;Megakaryocyte progenitors: 112.3;Oligodendrocyte progenitor cells: 66.6;Pericytes: 213.6;F
	Cell type enhanced	Detected in many		Late spermatids: 157.1
brain: 75.3	Cell type enhanced	Detected in many		Cytotrophoblasts: 31.6;Decidual stromal cells: 44.1;Hofbauer cells: 45.8
intestine: 920.3	Group enriched	Detected in some	4	Colonocytes: 184.2;Esophageal apical cells: 143.5;Foveolar cells: 51.2;Goblet cells: 86.0
intestine: 612.7	Group enriched	Detected in some	10	Colonocytes: 169.1;Esophageal apical cells: 98.7
esophagus: 32.9	Cell type enhanced	Detected in many		Endometrial luminal cells: 130.5;Esophageal apical cells: 133.4;Ocular epithelial cells: 169.8;Proximal tubule cells: 159.4;Respiratory basal cells: 211.2;Respiratory ciliated cells: 255.3;Respiratory deuterosomal cells: 218.7;Re
parathyroid gland: 87.0;placenta: 74.8	Cell type enhanced	Detected in many		Endometrial glandular cells: 761.6;NK-cells: 1572.1;T-cells: 945.5
	Cell type enhanced	Detected in all		Alveolar cells type 1: 787.2;Myonuclei: 754.3;Neutrophil progenitors: 823.9;Neutrophils: 2588.8;Platelets: 789.2
intestine: 53.1	Cell type enhanced	Detected in many		Alveolar cells type 1: 272.4;Alveolar cells type 2: 85.1;Colonocytes: 143.1;Enterocytes: 167.0;Foveolar cells: 94.2;Mesothelial cells: 88.7;Respiratory ciliated cells: 98.5;Transitional alveolar cells: 108.4;Urothelial cells: 88.8
kidney: 63.8	Cell type enhanced	Detected in many		Distal convoluted tubule cells: 973.6;Esophageal apical cells: 381.8;Loop of henle epithelial cells: 963.8;Podocytes: 590.0;Prostatic glandular cells: 345.2;Proximal tubule cells: 512.0;Renal collecting duct intercalated cells: 65
	Cell type enhanced	Detected in all		Esophageal apical cells: 393.5;Neutrophils: 630.9
salivary gland: 16.5	Cell type enhanced	Detected in many		Brain excitatory neurons: 469.0;Brain inhibitory neurons: 475.5;Gastric chief cells: 458.7;Granulosa cells: 429.8;Hematopoietic stem cells: 698.7;Lacrimal acinar cells: 585.2;Mucous neck cells: 597.4;Pancreatic acinar cells: 9
brain: 4.6;intestine: 5.8;lymphoid tissue: 14.4	Cell type enhanced	Detected in some		Cone photoreceptor cells: 6.2;Early spermatids: 2.5;Goblet cells: 14.8;Late primary spermatocytes: 8.0;Late spermatids: 10.0;Neuroendocrine cells: 3.5;Pancreatic islet cells: 5.8
skeletal muscle: 160.2	Group enriched	Detected in some	6	Myonuclei: 62.1;Myosatellite cells: 166.5;Thymic myoid cells: 55.7
prostate: 27.3	Group enriched	Detected in some	6	Epicardial cells: 22.7;Epididymal efferent duct absorptive cells: 19.6;Platelets: 38.0;Prostatic glandular cells: 34.5;Retinal amacrine cells: 52.8
adrenal gland: 37.1;lymphoid tissue: 61.2;retina: 49.6	Cell type enhanced	Detected in some		Adrenal medulla cells: 117.0;Cone photoreceptor cells: 159.4;Oocytes: 32.2;Retinal pigment epithelial cells: 503.0
	Cell type enhanced	Detected in many		Cone photoreceptor cells: 60.0;Differentiating spermatogonia: 64.6;Early primary spermatocytes: 37.2;Oocytes: 175.0;Retinal horizontal cells: 52.1
adrenal gland: 2.3;lymphoid tissue: 2.7;retina: 2.6;testis: 5.6	Group enriched	Detected in some	4	Early spermatids: 177.4;Late spermatids: 135.3;Oocytes: 152.0
skeletal muscle: 75.2	Group enriched	Detected in some	6	Müller glia: 21.7;Myonuclei: 14.7;Myosatellite cells: 5.6;Thymic myoid cells: 21.8
	Cell type enhanced	Detected in many		Colonocytes: 615.3;Fallopian tube ciliated cells: 911.2;Late spermatids: 2494.8;Respiratory ciliated cells: 963.3;Salivary duct cells: 645.2
cervix: 43.0;esophagus: 110.4;skin 1: 83.8;vagina: 53.4	Cell type enhanced	Detected in some		Basal keratinocytes: 124.1;Esophageal apical cells: 411.2;Esophageal basal cells: 217.0;Esophageal suprabasal cells: 480.0;Ocular epithelial cells: 204.0;Prostatic hillock cells: 155.5;Respiratory basal cells: 100.8;Respiratory
epididymis: 22.3;kidney: 43.1;liver: 15.8	Cell type enhanced	Detected in many		Distal convoluted tubule cells: 453.3;Loop of henle epithelial cells: 447.2;Myosatellite cells: 714.4;Proximal tubule cells: 173.6;Renal connecting tubule cells: 218.0
brain: 119.5;ovary: 46.0;testis: 50.6	Cell type enhanced	Detected in some		Oligodendrocytes: 2.8;Ovarian stromal cells: 1.8;Sertoli cells: 1.4
choroid plexus: 12.2;kidney: 42.4;placenta: 9.2	Group enriched	Detected in some	4	Extravillous trophoblasts: 360.7;Retinal pigment epithelial cells: 98.0
choroid plexus: 189.2;gallbladder: 95.3;kidney: 154.2;seminal vesic	Cell type enriched	Detected in some	6	Epididymal efferent duct absorptive cells: 351.7
	Cell type enhanced	Detected in many		Adipocytes: 21.4;Cardiomyocytes: 87.3;Epicardial cells: 25.0;Mast cells: 13.8;Myonuclei: 24.6;Retinal horizontal cells: 14.0
intestine: 248.6;pancreas: 92.7;thyroid gland: 110.9	Cell type enhanced	Detected in many		Breast secretory cells: 680.6;Colonocytes: 2780.7;Endometrial ciliated cells: 975.5;Endometrial glandular cells: 1468.5;Endometrial luminal cells: 1459.2;Enteric stem cells: 1410.9;Enteric transient amplifying cells: 1531.7;Ente
esophagus: 317.9;intestine: 265.3;urinary bladder: 282.7	Cell type enhanced	Detected in many		Breast secretory cells: 2532.4;Colonocytes: 2493.7;Endometrial glandular cells: 2506.9;Endometrial luminal cells: 1852.0;Enterocytes: 1748.9;Epididymal basal cells: 4215.3;Esophageal apical cells: 5918.8;Esophageal suprab
esophagus: 324.6;intestine: 321.6	Cell type enhanced	Detected in many		Alveolar cells type 1: 72.5;Alveolar cells type 2: 36.1;Breast secretory cells: 37.4;Colonocytes: 34.1;Conjunctival goblet cells: 35.0;Endometrial secretory cells: 54.1;Enterocytes: 52.9;Esophageal apical cells: 61.9;Ocular epith
brain: 27.1;pituitary gland: 28.6	Cell type enhanced	Detected in some		Cholangiocytes: 17.7;Corticotrophs: 39.4;Endometrial ciliated cells: 10.4;Endometrial secretory cells: 7.8;Epididymal basal cells: 13.5;Epididymal clear cells: 8.2;Epididymal efferent duct ciliated cells: 15.5;Fallopian secretory
bone marrow: 39.0	Cell type enhanced	Detected in some		cDC: 35.3;Early spermatids: 9.4;Epicardial cells: 11.4;Macrophages: 42.4;Microglia: 8.4;monocytes: 24.1;Neutrophil progenitors: 66.8;Neutrophils: 23.4
	Cell type enhanced	Detected in many		Alveolar cells type 1: 113.6;Alveolar cells type 2: 143.2;cDC: 196.6;Hematopoietic stem cells: 80.1;Kupffer cells: 210.1;Lymphatic endothelial cells: 110.7;Macrophages: 108.2;Transitional alveolar cells: 67.0;Vascular endothel
blood vessel: 408.2	Cell type enhanced	Detected in many		Endometrial stromal cells: 590.8;Epicardial cells: 755.0;Extravillous trophoblasts: 629.5;Mesothelial cells: 588.3;Plasma cells: 613.3;Schwann cells: 1130.9;Smooth muscle cells: 835.4
heart muscle: 96.2;skeletal muscle: 271.2;tongue: 91.9	Group enriched	Detected in many	4	Alveolar cells type 1: 992.7;Podocytes: 3048.4
	Cell type enhanced	Detected in many		Cytotrophoblasts: 1160.4;Esophageal apical cells: 1200.9;Hofbauer cells: 1405.7;Syncytiotrophoblasts: 1536.1
	Cell type enhanced	Detected in many		Colonocytes: 688.4;Esophageal apical cells: 4480.3;Esophageal basal cells: 952.5;Esophageal suprabasal cells: 2587.4;Extravillous trophoblasts: 907.9;Suprabasal keratinocytes: 993.7
choroid plexus: 31.5	Cell type enriched	Detected in some	23	Choroid plexus epithelial cells: 236.6
retina: 244.8	Group enriched	Detected in many	4	Esophageal apical cells: 336.9;Rod photoreceptor cells: 1024.5
retina: 36.0	Cell type enhanced	Detected in many		Colonocytes: 143.2;Cone photoreceptor cells: 186.3;Rod photoreceptor cells: 129.6
adipose tissue: 18.3;brain: 18.6	Cell type enhanced	Detected in many		Alveolar cells type 2: 41.3;Brain excitatory neurons: 73.2;Brain inhibitory neurons: 246.3;Corticotrophs: 73.8;Gonadotrophs: 40.4;Other brain neurons: 62.4;Pericytes: 138.8;Somatotrophs: 128.9;Thyrotrophs: 57.4
	Low cell type specificity	Detected in many		
skin 1: 548.0	Cell type enhanced	Detected in many		Basal keratinocytes: 1195.6;Breast myoepithelial cells: 265.2;Colonocytes: 148.3;Enterocytes: 121.0;Epididymal basal cells: 122.2;Esophageal basal cells: 151.4;Medullary thymic epithelial cells: 199.2;Ocular epithelial cells: 7
	Group enriched	Detected in many	4	Decidual stromal cells: 1279.3;Endometrial stromal cells: 498.8;Fibro-adipogenic progenitors: 384.4;Fibroblasts: 1238.3;Hepatic stellate cells: 916.6;Leydig cells: 746.7;Ovarian stromal cells: 425.3;Pericytes: 723.9;Peritubular
	Cell type enhanced	Detected in many		Colonocytes: 245.3;Enterocytes: 239.0;Esophageal apical cells: 395.2;Extravillous trophoblasts: 342.8;Hofbauer cells: 331.1
	Cell type enriched	Detected in all	4	Neutrophils: 1636.4
adipose tissue: 12.4;lymphoid tissue: 25.7	Cell type enhanced	Detected in many		B-cells: 176.8;Kupffer cells: 411.0;Neutrophil progenitors: 136.8;Neutrophils: 1929.7;Podocytes: 706.7
esophagus: 52.7	Cell type enhanced	Detected in many		Colonocytes: 131.8;Epididymal efferent duct absorptive cells: 103.1;Esophageal apical cells: 692.1;Esophageal suprabasal cells: 173.2;Ocular epithelial cells: 103.9;Suprabasal keratinocytes: 108.5
choroid plexus: 20.9	Cell type enhanced	Detected in some		Choroid plexus epithelial cells: 71.8;Epicardial cells: 22.7;Pericytes: 12.7;Retinal amacrine cells: 35.9;Retinal horizontal cells: 26.0;Somatotrophs: 11.4;Thyrotrophs: 16.0
heart muscle: 10537.6;skeletal muscle: 7429.7;tongue: 8772.8	Cell type enhanced	Detected in many		Breast myoepithelial cells: 1202.9;Endometrial glandular cells: 1087.8;Epididymal basal cells: 1094.8;Epididymal clear cells: 1281.9;Epididymal efferent duct absorptive cells: 3658.9;Epididymal efferent duct ciliated cells: 218
	Cell type enhanced	Detected in many		Erythrocyte progenitors: 101.5;Fibroblasts: 73.2;Mast cells: 343.1;Megakaryocyte progenitors: 125.9;Megakaryocyte-Erythroid progenitors: 451.5;Pancreatic duct cells: 107.7;Schwann cells: 75.3
lymphoid tissue: 106.8;placenta: 97.1	Cell type enhanced	Detected in many		cDC: 124.0;Extravillous trophoblasts: 226.4;Hofbauer cells: 647.5;Kupffer cells: 438.7;Macrophages: 330.0;Microglia: 701.8;monocytes: 160.2;Retinal pigment epithelial cells: 102.3
	Cell type enhanced	Detected in all		Early spermatids: 371.3;Late spermatids: 1358.7;Neutrophils: 605.0
	Cell type enhanced	Detected in all		Esophageal apical cells: 1095.4
	Cell type enhanced	Detected in all		monocytes: 871.7
	Cell type enhanced	Detected in many		Distal convoluted tubule cells: 113.4;Esophageal apical cells: 103.4;Foveolar cells: 138.5;Microglia: 134.0;Papillary tip epithelial cells: 128.0;Podocytes: 142.5;Renal collecting duct intercalated cells: 120.6;Renal collecting du
	Cell type enhanced	Detected in many		Astrocytes: 99.9;Bergmann glia: 85.5;Brain excitatory neurons: 96.3;Oligodendrocytes: 157.7;Papillary tip epithelial cells: 126.6;Platelets: 345.7;Proximal tubule cells: 100.3;Renal collecting duct intercalated cells: 115.3;Renal
	Cell type enhanced	Detected in many		Breast secretory cells: 132.7;Epididymal clear cells: 69.4;Epididymal efferent duct absorptive cells: 62.6;Lacrimal acinar cells: 59.8;Pancreatic duct cells: 77.8;Salivary duct cells: 71.3;Salivary myoepithelial cells: 53.3;Submuc
brain: 33.1	Cell type enriched	Detected in some	8	Microglia: 936.8
skin 1: 50.3	Cell type enhanced	Detected in many		Endometrial luminal cells: 393.9;Enterocytes: 530.9;Urothelial cells: 715.9
esophagus: 25.7;lymphoid tissue: 40.8	Cell type enriched	Detected in some	13	Neutrophils: 1412.1
	Cell type enhanced	Detected in many		Enterocytes: 872.6;Fibroblasts: 336.6;Leydig cells: 252.2;Peritubular myoid cells: 264.1
	Cell type enhanced	Detected in many		Early spermatids: 363.6;Esophageal apical cells: 324.1;Late primary spermatocytes: 179.1;Late spermatids: 233.2;Syncytiotrophoblasts: 175.6
bone marrow: 55.0;lymphoid tissue: 63.5	Cell type enhanced	Detected in some		cDC: 94.7;Hofbauer cells: 144.4;Kupffer cells: 85.1;Macrophages: 102.3;Mast cells: 54.4;Microglia: 116.3;monocytes: 86.0;Neutrophils: 336.9;NK-cells: 50.7;pDCs: 165.9;Plasma cells: 42.7;T-cells: 44.2
kidney: 167.4;placenta: 213.0	Cell type enhanced	Detected in many		Breast lactating cells: 199.5;Epididymal efferent duct absorptive cells: 453.0;Epididymal principal cells: 305.0;Hofbauer cells: 1695.9;Platelets: 847.3;Syncytiotrophoblasts: 941.8
	Cell type enhanced	Detected in many		Myonuclei: 285.5;Myosatellite cells: 229.0;Schwann cells: 221.4;Thymic myoid cells: 186.3
	Cell type enhanced	Detected in many		Endometrial glandular cells: 71.3;Microglia: 97.6;Neutrophil progenitors: 78.3
	Cell type enhanced	Detected in many		Esophageal apical cells: 950.6;Esophageal suprabasal cells: 366.6;Hofbauer cells: 222.7;Megakaryocytes: 315.3;Platelets: 371.1
skin 1: 101.6	Cell type enriched	Detected in many	90	Melanocytes: 9101.9
	Cell type enhanced	Detected in many		Astrocytes: 91.8;Bergmann glia: 29.4;Distal convoluted tubule cells: 24.4;Ependymal cells: 101.0;Loop of henle epithelial cells: 36.4;Oligodendrocyte progenitor cells: 97.9;Oligodendrocytes: 78.5;Papillary tip epithelial cells: 5

	Cell type enhanced	Detected in many		Adipocytes: 304.7;Adrenal cortex cells: 741.1;Choroid plexus epithelial cells: 586.4;Endometrial stromal cells: 184.4;Fibro-adipogenic progenitors: 516.1;Fibroblasts: 366.1;Retinal pigment epithelial cells: 306.9
	Cell type enhanced	Detected in many		Respiratory basal cells: 584.7;Respiratory ciliated cells: 453.4;Urothelial cells: 1355.4
bone marrow: 6.7;testis: 17.7	Cell type enhanced	Detected in many		Early primary spermatocytes: 135.7;Early spermatids: 534.8;Erythrocyte progenitors: 291.6;Extravillous trophoblasts: 183.3;Late primary spermatocytes: 390.1;Megakaryocyte progenitors: 150.9;Mesothelial cells: 200.1;Migra
	Cell type enhanced	Detected in all		Microglia: 322.1;Neutrophil progenitors: 297.7;NK-cells: 279.2;Rod photoreceptor cells: 396.2
	Cell type enhanced	Detected in all		Oligodendrocytes: 2027.4
brain: 211.2;retina: 200.9	Cell type enhanced	Detected in many		Choroid plexus epithelial cells: 48.6;Cone photoreceptor cells: 209.1;Retinal bipolar cells: 64.8;Rod photoreceptor cells: 141.9
	Cell type enhanced	Detected in many		Astrocytes: 49.7;Bergmann glia: 63.1;Choroid plexus epithelial cells: 129.9;Distal convoluted tubule cells: 95.4;Ependymal cells: 59.1;Loop of henle epithelial cells: 59.3;Papillary tip epithelial cells: 110.0;Podocytes: 243.4;Pro
	Cell type enhanced	Detected in many		Cardiomyocytes: 21.9;Early spermatids: 113.6;Late primary spermatocytes: 32.7;Late spermatids: 58.5;Pancreatic duct cells: 14.0
bone marrow: 86.6	Cell type enhanced	Detected in many		Erythrocyte progenitors: 257.2
brain: 28.0	Cell type enhanced	Detected in many		Astrocytes: 1167.0;Macrophages: 1540.4;Microglia: 6744.0;monocytes: 1634.2;Neutrophils: 3894.0;Oligodendrocytes: 1144.3;Thyrotrophs: 1035.9
bone marrow: 35.7;lymphoid tissue: 57.8	Cell type enhanced	Detected in many		Choroid plexus epithelial cells: 1461.7;Microglia: 2809.4;Monocyte progenitors: 606.3;monocytes: 637.7;Neutrophil progenitors: 1121.6;Neutrophils: 2638.1;Thymocytes: 605.6
lung: 24.2;lymphoid tissue: 44.0	Cell type enhanced	Detected in some		cDC: 166.0;Hofbauer cells: 202.0;Kupffer cells: 186.5;Macrophages: 97.1;Megakaryocytes: 123.6;monocytes: 140.6;NK-cells: 146.1;Platelets: 559.2;T-cells: 96.2
bone marrow: 88.4;lymphoid tissue: 83.8	Group enriched	Detected in many	4	Neutrophil progenitors: 173.8;Neutrophils: 585.6
intestine: 364.7;kidney: 245.4;pancreas: 477.7	Group enriched	Detected in many	8	Enterocytes: 4466.3;Peritubular myoid cells: 1128.1
	Cell type enhanced	Detected in some		Choroid plexus epithelial cells: 9.8;Cone photoreceptor cells: 8.3;Ependymal cells: 15.1;pDCs: 16.9;Retinal bipolar cells: 12.1;Retinal pigment epithelial cells: 42.6;Rod photoreceptor cells: 49.7
esophagus: 160.9;lymphoid tissue: 60.5	Cell type enhanced	Detected in many		Basal keratinocytes: 339.0;Colonocytes: 404.7;Esophageal apical cells: 2014.7;Esophageal basal cells: 337.3;Esophageal suprabasal cells: 1659.0;Neutrophils: 437.8;Ocular epithelial cells: 1279.1;Suprabasal keratinocytes: 1
esophagus: 93.2;skin 1: 351.6	Cell type enhanced	Detected in many		Basal keratinocytes: 836.6;Esophageal apical cells: 241.7;Esophageal basal cells: 506.0;Esophageal suprabasal cells: 534.3;Ocular epithelial cells: 841.5;Prostatic hillock cells: 147.8;Suprabasal keratinocytes: 1019.9
intestine: 99.0;parathyroid gland: 148.5	Cell type enhanced	Detected in many		Colonocytes: 380.6;Foveolar cells: 389.2;Gastric progenitor cells: 241.0;Parietal cells: 235.1;Urothelial cells: 345.5
esophagus: 437.5;skin 1: 807.7	Cell type enhanced	Detected in many		Basal keratinocytes: 1933.0;Esophageal apical cells: 3342.1;Esophageal basal cells: 1428.6;Esophageal suprabasal cells: 3402.7;Ocular epithelial cells: 2856.0;Suprabasal keratinocytes: 4671.6
brain: 204.2;choroid plexus: 72.0;heart muscle: 140.1;skeletal musc	Cell type enhanced	Detected in many		Astrocytes: 3051.9;Bergmann glia: 5155.1;Cardiomyocytes: 993.4;Ependymal cells: 1605.0;Myonuclei: 1700.4;Pancreatic acinar cells: 1233.1;Pituicytes/FSCs: 963.4
bone marrow: 49.3;skeletal muscle: 119.0	Cell type enriched	Detected in many	4	Neutrophils: 2844.8
	Group enriched	Detected in some	6	Lymphatic endothelial cells: 4.8;Vascular endothelial cells: 9.4
seminal vesicle: 107.2	Cell type enhanced	Detected in many		Cardiomyocytes: 83.6;Decidual stromal cells: 133.8;Endometrial stromal cells: 191.7;Leydig cells: 127.0;Ovarian stromal cells: 112.2;Pericytes: 260.6;Peritubular myoid cells: 161.1;Respiratory ionocytes: 55.9;Smooth muscle
placenta: 120.1	Cell type enhanced	Detected in many		Astrocytes: 304.8;Bergmann glia: 185.5;Fibroblasts: 111.2;Hepatic stellate cells: 174.3;Lymphatic endothelial cells: 193.0;Melanocytes: 750.2;Müller glia: 136.6;Pericytes: 261.6;Pituicytes/FSCs: 91.5;Schwann cells: 104.1;Vas
	Cell type enhanced	Detected in many		Basal keratinocytes: 112.0;Colonocytes: 53.1;Esophageal basal cells: 74.7;Extravillous trophoblasts: 119.5;Foveolar cells: 55.4;Podocytes: 77.4;Suprabasal keratinocytes: 88.5;Urothelial cells: 67.1;Vascular endothelial cells:
retina: 112.6	Cell type enhanced	Detected in all		Retinal bipolar cells: 680.1
brain: 32.5;skeletal muscle: 18.6	Cell type enhanced	Detected in many		Astrocytes: 207.7;Bergmann glia: 92.7;Brain excitatory neurons: 135.0;Brain inhibitory neurons: 164.0;Corticotrophs: 100.3;Lactotrophs: 125.1;Retinal ganglion cells: 239.5;Retinal pigment epithelial cells: 537.1;Thyrotrophs: 1
placenta: 61.8	Cell type enhanced	Detected in many		Cytotrophoblasts: 1190.3;Fibro-adipogenic progenitors: 1097.7;Myosatellite cells: 789.2;Respiratory basal cells: 935.5;Syncytiotrophoblasts: 1481.8
	Cell type enhanced	Detected in all		Adipocytes: 1746.1;Schwann cells: 1537.9
bone marrow: 143.0;testis: 148.3	Group enriched	Detected in many	5	Early spermatids: 1211.5;Late spermatids: 3099.1;Neutrophils: 1042.5
	Cell type enhanced	Detected in many		Alveolar cells type 1: 246.8;Decidual stromal cells: 129.5;Fibroblasts: 70.6;Hepatic stellate cells: 135.6;Leydig cells: 94.2;Lymphatic endothelial cells: 99.3;Pericytes: 179.2;Peritubular myoid cells: 101.4;Vascular endothelial ce
brain: 41.0;esophagus: 40.9	Cell type enhanced	Detected in many		Esophageal apical cells: 331.5;Esophageal suprabasal cells: 44.7;Megakaryocytes: 105.1;Ocular epithelial cells: 67.8;Oligodendrocyte progenitor cells: 58.5;Platelets: 273.4;Syncytiotrophoblasts: 88.3
pancreas: 147.7;salivary gland: 156.2;stomach 1: 83.2	Cell type enhanced	Detected in many		Corticotrophs: 466.9;Goblet cells: 332.0;Gonadotrophs: 313.6;Lacrimal acinar cells: 485.5;Lactotrophs: 452.9;Mucous neck cells: 297.6;Pancreatic islet cells: 392.9;Paneth cells: 506.6;Prostatic glandular cells: 738.4;Salivary
	Cell type enhanced	Detected in many		Sertoli cells: 614.7;Smooth muscle cells: 693.4
heart muscle: 518.6	Cell type enhanced	Detected in many		Extravillous trophoblasts: 494.5;Hepatic stellate cells: 264.6;Leydig cells: 764.5;Peritubular myoid cells: 1903.9;Syncytiotrophoblasts: 673.5;Vascular endothelial cells: 642.6
	Cell type enhanced	Detected in many		Esophageal apical cells: 2123.3;Esophageal basal cells: 2585.6;Esophageal suprabasal cells: 2329.2;Extravillous trophoblasts: 2438.6;Migrating cytotrophoblasts: 2045.2;Müller glia: 2322.9;Ocular epithelial cells: 1811.1
brain: 857.9;retina: 1573.2	Cell type enhanced	Detected in many		Brain excitatory neurons: 165.6;Brain inhibitory neurons: 124.1;Cone photoreceptor cells: 360.2;Other brain neurons: 131.7;Retinal bipolar cells: 164.1;Retinal horizontal cells: 198.3;Rod photoreceptor cells: 468.7
skeletal muscle: 13830.4;tongue: 10054.3	Cell type enhanced	Detected in many		Adipocytes: 62.2;Cardiomyocytes: 212.4;Epicardial cells: 150.1;Mesothelial cells: 87.0;Myonuclei: 163.8;Plasma cells: 82.2;Thymic myoid cells: 444.6;Thymocytes: 65.2
	Cell type enhanced	Detected in many		Brain excitatory neurons: 470.5;Brain inhibitory neurons: 519.6;Cardiomyocytes: 333.9;Early spermatids: 357.7;Late spermatids: 418.0;Loop of henle epithelial cells: 338.1;Medullary thymic epithelial cells: 420.0;Oligodendroc
	Low cell type specificity	Detected in many		
	Cell type enhanced	Detected in many		Cone photoreceptor cells: 432.7;Distal convoluted tubule cells: 287.7;Loop of henle epithelial cells: 288.0;Renal connecting tubule cells: 373.6
retina: 327.6	Cell type enhanced	Detected in all		Cone photoreceptor cells: 726.6;Fibro-adipogenic progenitors: 693.7;Kupffer cells: 805.6;Microglia: 1812.6;Müller glia: 869.1;Oligodendrocytes: 594.1;Rod photoreceptor cells: 2298.8;Sertoli cells: 651.8
	Cell type enhanced	Detected in many		Cone photoreceptor cells: 461.6;Enterocytes: 471.9;Esophageal apical cells: 440.7;Kupffer cells: 452.0;Late spermatids: 459.7;Macrophages: 479.3;monocytes: 600.8;Proximal tubule cells: 557.9;Retinal amacrine cells: 527.1
	Cell type enhanced	Detected in many		Podocytes: 1344.4
intestine: 756.2	Cell type enhanced	Detected in many		Colonocytes: 2714.8;Enteric stem cells: 1605.1;Enteric transient amplifying cells: 1859.2;Enterocytes: 2694.4;Goblet cells: 1377.2;Oocytes: 819.7;Pancreatic duct cells: 803.5;Paneth cells: 1256.9
esophagus: 142.7	Cell type enhanced	Detected in many		Endometrial glandular cells: 197.8;Endometrial luminal cells: 221.0;Esophageal apical cells: 1038.5;Esophageal suprabasal cells: 325.0;Ocular epithelial cells: 384.2;Prostatic hillock cells: 217.5;Respiratory basal cells: 192.0;S
prostate: 29.5	Cell type enhanced	Detected in many		Adipocytes: 82.8;Brain inhibitory neurons: 112.0;Corticotrophs: 300.2;Fibro-adipogenic progenitors: 210.2;Fibroblasts: 90.1;Hepatic stellate cells: 77.5;Other brain neurons: 80.8;Pericytes: 165.9;Retinal bipolar cells: 132.1;Re
brain: 10.5	Cell type enhanced	Detected in many		Adrenal medulla cells: 106.4;Brain excitatory neurons: 296.3;Brain inhibitory neurons: 137.5;Lactotrophs: 275.0;Melanocytes: 120.9;Oligodendrocyte progenitor cells: 111.6;Other brain neurons: 205.9;Prostatic glandular cells
brain: 19.3	Cell type enhanced	Detected in many		Astrocytes: 293.8;Brain excitatory neurons: 238.6;Brain inhibitory neurons: 209.1;Lactotrophs: 92.2;Oligodendrocyte progenitor cells: 151.8;Oligodendrocytes: 222.0;Other brain neurons: 121.0;pDCs: 235.3;Retinal ganglion c
intestine: 21.9	Cell type enhanced	Detected in many		Enteric stem cells: 337.1;Enteric transient amplifying cells: 209.9;Late spermatids: 366.1;Neuroendocrine cells: 100.4;Paneth cells: 425.4;Pituicytes/FSCs: 100.3;Retinal amacrine cells: 117.1
	Cell type enhanced	Detected in many		Erythrocyte progenitors: 125.8;Hofbauer cells: 103.5;Platelets: 98.8;Renal collecting duct principal cells: 47.2
	Cell type enhanced	Detected in many		Esophageal apical cells: 241.3;Prostatic club cells: 178.9
	Cell type enhanced	Detected in many		Enterocytes: 111.6;Esophageal apical cells: 275.6;Oligodendrocytes: 307.1
	Cell type enhanced	Detected in all		Neutrophils: 1130.9;Oligodendrocytes: 1371.3
	Cell type enhanced	Detected in many		Somatotrophs: 922.7;Vascular smooth muscle cells: 875.1
brain: 164.5;lymphoid tissue: 97.8	Cell type enhanced	Detected in many		Cardiomyocytes: 45.3;cDC: 15.9;Epicardial cells: 22.7;Innate lymphoid cells: 18.8;Kupffer cells: 18.4;Macrophages: 18.0;Mast cells: 17.8;Microglia: 19.4;Monocyte progenitors: 17.0;monocytes: 24.6;Neutrophils: 68.7;NK-cells
	Cell type enhanced	Detected in many		Enterocytes: 2059.1;Esophageal apical cells: 2062.0;Ocular epithelial cells: 3071.0;Syncytiotrophoblasts: 2089.8;Urothelial cells: 2184.8
	Cell type enhanced	Detected in many		Alveolar cells type 1: 164.7;Esophageal apical cells: 314.2;Extravillous trophoblasts: 254.6;Ocular epithelial cells: 242.2;Pancreatic acinar cells: 173.6;Urothelial cells: 235.0
adipose tissue: 18.9;breast: 19.0;lung: 33.0;thyroid gland: 19.3	Group enriched	Detected in some	4	Lymphatic endothelial cells: 48.9;Megakaryocytes: 61.8;Platelets: 108.8;Vascular endothelial cells: 97.2
	Cell type enhanced	Detected in many		Adrenal cortex cells: 93.9;Alveolar cells type 1: 104.1;Epicardial cells: 95.5;Epididymal basal cells: 143.7;Fallopian secretory cells: 108.1;Mesothelial cells: 106.6;Neutrophils: 161.2
adipose tissue: 364.3;breast: 270.5;liver: 192.6	Cell type enriched	Detected in many	4	Breast lactating cells: 3382.5
blood vessel: 444.4	Cell type enhanced	Detected in many		Colonocytes: 241.1;Decidual stromal cells: 123.1;Enterocytes: 268.0;Extravillous trophoblasts: 196.9;Foveolar cells: 228.4;Ocular epithelial cells: 180.7;Pericytes: 116.9;Peritubular myoid cells: 235.4
	Cell type enhanced	Detected in many		Choroid plexus epithelial cells: 312.3;Endometrial ciliated cells: 347.1;Ependymal cells: 316.1;Epididymal efferent duct ciliated cells: 282.5;Respiratory ciliated cells: 331.4
bone marrow: 24.7;lung: 10.7;lymphoid tissue: 14.5	Cell type enhanced	Detected in some		Microglia: 3.1;monocytes: 6.7;Neutrophil progenitors: 7.3;Neutrophils: 20.0
epididymis: 95.0	Cell type enhanced	Detected in some		Early spermatids: 30.2;Epididymal principal cells: 55.3;Hofbauer cells: 350.0;Kupffer cells: 73.8;Late spermatids: 88.1;Macrophages: 50.2;monocytes: 61.3;Neutrophils: 80.6
lymphoid tissue: 113.4;placenta: 126.7	Cell type enriched	Detected in some	4	Neutrophils: 452.3
	Cell type enhanced	Detected in many		B-cells: 1232.7;Microglia: 1168.0;Neutrophils: 884.3;pDCs: 2334.7
intestine: 56.3;lymphoid tissue: 169.0	Cell type enhanced	Detected in many		B-cells: 296.5;Epicardial cells: 91.0;Innate lymphoid cells: 42.3;NK-cells: 108.3;T-cells: 99.7;Thymocytes: 61.8
bone marrow: 2.9;brain: 2.2;ovary: 2.5;pancreas: 2.4	Cell type enriched	Detected in some	4	Pancreatic islet cells: 34.2
adipose tissue: 3.8;intestine: 10.7;lung: 3.4;pituitary gland: 10.4	Cell type enhanced	Detected in some		Adipocytes: 167.0;Corticotrophs: 30.4;Early spermatids: 66.0;Epicardial cells: 357.0;Goblet cells: 126.3;Microglia: 30.0;Somatotrophs: 111.5
	Cell type enhanced	Detected in many		Retinal horizontal cells: 30.1
	Cell type enhanced	Detected in many		Corticotrophs: 54.7;Decidual stromal cells: 30.6;Fibro-adipogenic progenitors: 97.4;Fibroblasts: 34.3;Hematopoietic stem cells: 50.9;Hofbauer cells: 44.5;Leydig cells: 43.3;Lymphatic endothelial cells: 130.5;Proximal tubule c
skeletal muscle: 10741.5;tongue: 9124.0	Cell type enhanced	Detected in many		Adipocytes: 294.0;Early spermatids: 439.1;Hepatic stellate cells: 367.9;Late spermatids: 1746.7;Myonuclei: 1299.3;Smooth muscle cells: 799.0;Thymic myoid cells: 909.6;Vascular smooth muscle cells: 434.1
	Cell type enhanced	Detected in many		Extravillous trophoblasts: 779.3;Pancreatic acinar cells: 673.0;Prostatic glandular cells: 611.2;Urothelial cells: 613.5
	Cell type enhanced	Detected in many		Late spermatids: 140.1;Neutrophils: 132.3;Oocytes: 225.6
	Cell type enhanced	Detected in many		Late spermatids: 353.8;Neutrophil progenitors: 335.2;Neutrophils: 1028.1
kidney: 21.7	Cell type enhanced	Detected in many		Alveolar cells type 1: 359.6;Alveolar cells type 2: 214.0;Basal prostatic cells: 168.0;Epididymal basal cells: 85.4;Podocytes: 214.7;Transitional alveolar cells: 203.6
placenta: 394.3	Cell type enhanced	Detected in many		Cytotrophoblasts: 308.8;Extravillous trophoblasts: 1644.7;Late spermatids: 279.5;Migrating cytotrophoblasts: 449.7;Müller glia: 545.6;Neutrophils: 284.2;Syncytiotrophoblasts: 1703.8;Vascular endothelial cells: 1218.5
	Cell type enhanced	Detected in some		Extravillous trophoblasts: 104.1;Lymphatic endothelial cells: 197.0;Podocytes: 47.2;Undifferentiated spermatogonia: 14.2;Vascular endothelial cells: 32.4
	Cell type enriched	Detected in many	5	Microglia: 566.6
lymphoid tissue: 101.9	Cell type enhanced	Detected in many		B-cells: 794.3;cDC: 934.8;Innate lymphoid cells: 811.8;Kupffer cells: 804.6;monocytes: 674.1;Neutrophils: 910.3;Oligodendrocytes: 669.4;Plasma cells: 701.5;T-cells: 883.6;Thymocytes: 672.5
lung: 26.6	Cell type enhanced	Detected in some		cDC: 304.3;Kupffer cells: 196.8;Macrophages: 236.0;monocytes: 74.5
	Cell type enhanced	Detected in many		Esophageal apical cells: 151.6;Urothelial cells: 125.9
	Cell type enhanced	Detected in many		Astrocytes: 707.1;Bergmann glia: 770.9;Innate lymphoid cells: 1307.3;NK-cells: 1966.4;Oligodendrocyte progenitor cells: 609.8;Oligodendrocytes: 813.6;Podocytes: 1092.5;T-cells: 2000.5
	Cell type enhanced	Detected in many		Basal prostatic cells: 46.3;Breast secretory cells: 31.9;Cholangiocytes: 52.4;Peritubular myoid cells: 50.3;Pituicytes/FSCs: 36.4;Retinal horizontal cells: 96.2
adipose tissue: 112.7	Cell type enhanced	Detected in many		Adipocytes: 189.5;Distal convoluted tubule cells: 78.1;Fibro-adipogenic progenitors: 53.6;Lymphatic endothelial cells: 43.1;Pituitary stem cells: 58.0;Renal connecting tubule cells: 65.7;Thymic myoid cells: 58.5;Vascular endo
	Cell type enhanced	Detected in many		Alveolar cells type 1: 209.5;Endometrial luminal cells: 162.1
brain: 54.0	Cell type enhanced	Detected in many		Microglia: 774.7;monocytes: 1008.5;Neutrophils: 3649.1;Oligodendrocytes: 609.3;Thymocytes: 726.7
brain: 224.4	Cell type enhanced	Detected in some		Astrocytes: 293.4;Bergmann glia: 307.7;Brain excitatory neurons: 97.1;Brain inhibitory neurons: 122.5;Choroid plexus epithelial cells: 41.5;Ependymal cells: 72.9;Oligodendrocyte progenitor cells: 120.7;Other brain neurons: 9
brain: 52.4;retina: 16.5	Group enriched	Detected in some	7	Brain excitatory neurons: 184.1;Brain inhibitory neurons: 156.6;Other brain neurons: 71.8;Retinal bipolar cells: 224.8
brain: 121.7	Cell type enriched	Detected in some	6	Retinal bipolar cells: 381.5
brain: 20.9	Cell type enhanced	Detected in some		Astrocytes: 158.3;Bergmann glia: 275.5;Brain inhibitory neurons: 36.8;Ependymal cells: 383.4;Epididymal efferent duct ciliated cells: 37.8;Other brain neurons: 78.0;Retinal amacrine cells: 88.2
prostate: 6.2;retina: 25.1;testis: 8.6	Cell type enhanced	Detected in many		Bergmann glia: 908.0;Brain excitatory neurons: 294.6;Brain inhibitory neurons: 525.3;Ependymal cells: 296.5;Lactotrophs: 387.6;Late spermatids: 332.2;Müller glia: 1541.1;Other brain neurons: 683.3;Pituicytes/FSCs: 287.2;P
adrenal gland: 1.4;pituitary gland: 1.3	Cell type enriched	Detected in some	6	Pituitary stem cells: 577.6
brain: 473.7	Cell type enhanced	Detected in many		Brain excitatory neurons: 234.8;Brain inhibitory neurons: 148.6;Oligodendrocyte progenitor cells: 54.9;Other brain neurons: 248.7;Pituicytes/FSCs: 70.7;Retinal pigment epithelial cells: 81.0;Schwann cells: 33.2
skeletal muscle: 25843.3;tongue: 17635.1	Cell type enhanced	Detected in all		Esophageal apical cells: 15050.4;Esophageal basal cells: 8938.4;Esophageal suprabasal cells: 9709.9;Extravillous trophoblasts: 13577.7;Migrating cytotrophoblasts: 8315.6
bone marrow: 10.8;lymphoid tissue: 28.2	Cell type enhanced	Detected in some		B-cells: 65.6;cDC: 34.1;Hematopoietic stem cells: 53.4;Macrophages: 27.5;Mast cells: 67.2;Monocyte progenitors: 97.5;monocytes: 51.3;Neutrophil progenitors: 71.0;Neutrophils: 158.7;pDCs: 206.4;Thymocytes: 32.2
	Low cell type specificity	Detected in all		
	Cell type enhanced	Detected in many		Early spermatids: 220.7;Fallopian tube ciliated cells: 108.0;Late spermatids: 408.8;Respiratory ciliated cells: 124.9
intestine: 13.3	Group enriched	Detected in some	6	Late spermatids: 34.5;Neuroendocrine cells: 62.5;Oocytes: 50.7;Schwann cells: 74.9
	Cell type enhanced	Detected in some		Bergmann glia: 4.8;Epicardial cells: 4.5;Fibro-adipogenic progenitors: 9.9;Hepatic stellate cells: 6.5;Pericytes: 3.0;Pituitary stem cells: 4.2;Retinal amacrine cells: 2.6;Undifferentiated spermatogonia: 2.9
adipose tissue: 79.5;liver: 199.5	Group enriched	Detected in many	4	Adipocytes: 3141.2;Myonuclei: 1159.6
	Cell type enriched	Detected in many	4	Esophageal apical cells: 1003.3
	Cell type enhanced	Detected in many		Alveolar cells type 1: 446.7;Astrocytes: 470.8;Basal keratinocytes: 182.5;Basal prostatic cells: 192.1;Bergmann glia: 217.1;Decidual stromal cells: 148.2;Endometrial stromal cells: 167.7;Ependymal cells: 288.7;Esophageal su
esophagus: 75.8;skin 1: 139.2	Group enriched	Detected in many	5	Basal keratinocytes: 113.0;Esophageal apical cells: 246.4;Esophageal basal cells: 189.9;Esophageal suprabasal cells: 241.6;Ocular epithelial cells: 146.0;Suprabasal keratinocytes: 147.5
skin 1: 58.6	Group enriched	Detected in some	4	Esophageal apical cells: 31.6;Ocular epithelial cells: 105.0
cervix: 159.8;esophagus: 212.4;vagina: 163.9	Cell type enriched	Detected in some	4	Esophageal apical cells: 1619.9
placenta: 37.5	Cell type enhanced	Detected in many		Differentiating spermatogonia: 48.6;Early primary spermatocytes: 35.0;Endometrial stromal cells: 28.2;Pericytes: 192.9;Schwann cells: 40.9;Smooth muscle cells: 75.0;Vascular smooth muscle cells: 78.1
brain: 67.0	Group enriched	Detected in many	4	Lymphatic endothelial cells: 58.3;Oligodendrocytes: 41.1
adipose tissue: 17.3;blood vessel: 20.7;brain: 44.1	Group enriched	Detected in many	4	Microglia: 221.0;Oligodendrocytes: 606.7;Syncytiotrophoblasts: 334.1
	Cell type enhanced	Detected in many		Extravillous trophoblasts: 424.3
heart muscle: 2.6;pancreas: 8.6	Cell type enhanced	Detected in some		Lacrimal acinar cells: 19.0;Neuroendocrine cells: 8.0;Other brain neurons: 10.8;Pancreatic acinar cells: 15.5;Pancreatic islet cells: 23.3;Proximal tubule cells: 5.7;Salivary acinar cells: 4.8;Tuft cells: 46.1
gallbladder: 11.4;intestine: 13.4;urinary bladder: 12.1	Cell type enhanced	Detected in some		Cone photoreceptor cells: 20.7;Fibro-adipogenic progenitors: 13.1;Fibroblasts: 7.6;Hepatic stellate cells: 12.9;Leydig cells: 8.8;Rod photoreceptor cells: 59.0
retina: 2.3	Cell type enhanced	Detected in many		Adipocytes: 19.4;Adrenal medulla cells: 42.5;Cardiomyocytes: 39.9;Epicardial cells: 15.9;Hepatic stellate cells: 25.8;Myonuclei: 21.4;Other brain neurons: 18.5;Retinal amacrine cells: 30.5;Retinal bipolar cells: 81.9;Retinal gan
brain: 8.9;intestine: 1.5;retina: 3.0	Cell type enhanced	Detected in many		Brain excitatory neurons: 60.2;Brain inhibitory neurons: 58.2;Colonocytes: 42.6;Epicardial cells: 22.7;Gonadotrophs: 24.2;Oligodendrocytes: 21.1;Other brain neurons: 61.7;Retinal amacrine cells: 176.8;Retinal ganglion cells:
brain: 5.2;breast: 1.9	Cell type enhanced	Detected in some		Adrenal medulla cells: 31.9;Brain excitatory neurons: 59.5;Brain inhibitory neurons: 33.8;Breast hormone-responsive cells: 55.4;Early spermatids: 42.0;Gonadotrophs: 63.0;Late spermatids: 30.7;Other brain neurons: 72.9;Pan
	Cell type enhanced	Detected in all		Hofbauer cells: 484.1;Late spermatids: 487.7;Monocyte progenitors: 498.2;monocytes: 676.1;Neutrophil progenitors: 445.2;Neutrophils: 1060.3
	Cell type enhanced	Detected in all		Neutrophil progenitors: 2052.7;Neutrophils: 3046.1
	Cell type enhanced	Detected in all		Lactotrophs: 4354.4;Late spermatids: 2985.7;Pancreatic islet cells: 6202.3;Platelets: 3230.5;Somatotrophs: 4131.7;Thyrotrophs: 4260.2
	Cell type enhanced	Detected in all		Esophageal apical cells: 1493.9;Esophageal suprabasal cells: 574.4;Neutrophils: 574.2
brain: 2.3;testis: 2.5	Cell type enhanced	Detected in some		Brain excitatory neurons: 8.7;Brain inhibitory neurons: 8.6;Distal convoluted tubule cells: 6.7;Loop of henle epithelial cells: 5.8;Other brain neurons: 6.2;Papillary tip epithelial cells: 12.8;Platelets: 15.5;Renal collecting duct inte
skin 1: 213.4	Cell type enhanced	Detected in many		Esophageal apical cells: 107.1;Esophageal basal cells: 101.9;Extravillous trophoblasts: 285.2;Schwann cells: 104.4;Syncytiotrophoblasts: 115.4
placenta: 669.8	Cell type enhanced	Detected in many		Cytotrophoblasts: 103.0;Epididymal basal cells: 99.6;Extravillous trophoblasts: 130.2;Fibro-adipogenic progenitors: 162.9;Fibroblasts: 326.8;Müller glia: 124.5;Parietal cells: 134.4;Pericytes: 105.5;Pituicytes/FSCs: 224.9;Pituit
choroid plexus: 23.3	Cell type enhanced	Detected in many		Cytotrophoblasts: 56.9;Melanocytes: 321.7;Migrating cytotrophoblasts: 44.8;Retinal pigment epithelial cells: 123.6
	Not detected	Not detected		
testis: 2.5	Cell type enhanced	Detected in many		Early primary spermatocytes: 34.8;Early spermatids: 30.4;Ependymal cells: 32.4;Late primary spermatocytes: 21.5;Late spermatids: 38.1;Müller glia: 34.3;Oligodendrocyte progenitor cells: 20.6;Retinal ganglion cells: 30.9;Un
bone marrow: 102.0;parathyroid gland: 203.7;retina: 96.9	Cell type enhanced	Detected in many		Enteric stem cells: 293.5;Enteric transient amplifying cells: 238.9;Enterocytes: 406.4;Gastric chief cells: 338.5;Gastric progenitor cells: 230.7;Mucous neck cells: 210.2;Neuroendocrine cells: 277.7;Neutrophil progenitors: 252.
lymphoid tissue: 15.7	Cell type enhanced	Detected in some		B-cells: 34.2;Hematopoietic stem cells: 9.7;Innate lymphoid cells: 26.7;NK-cells: 71.8;pDCs: 17.9;Platelets: 9.8;T-cells: 105.1;Thymocytes: 39.0
bone marrow: 106.3;lymphoid tissue: 79.7	Cell type enhanced	Detected in many		B-cells: 302.1;cDC: 1208.2;Innate lymphoid cells: 192.6;Macrophages: 786.0;Microglia: 155.1;monocytes: 811.1;pDCs: 915.2;T-cells: 230.7

blood vessel: 4.1	Group enriched	Detected in some	5	monocytes: 2.6;Vascular smooth muscle cells: 7.4
brain: 44.8	Cell type enhanced	Detected in some		Astrocytes: 153.1;Bergmann glia: 209.8;Ependymal cells: 63.4;Epicardial cells: 25.0;Müller glia: 59.5;Oligodendrocyte progenitor cells: 186.5;Oligodendrocytes: 42.0;Schwann cells: 23.4
pituitary gland: 15.6	Cell type enriched	Detected in single	15	Lactotrophs: 4.3
brain: 9.8;retina: 7.1	Not detected	Not detected		
lung: 190.3;urinary bladder: 121.8	Cell type enhanced	Detected in many		Alveolar cells type 1: 3635.1;Alveolar cells type 2: 899.5;Colonocytes: 729.4;Conjunctival goblet cells: 732.0;Esophageal apical cells: 664.7;Ocular epithelial cells: 1154.7;Prostatic club cells: 847.4;Prostatic hillock cells: 802.3
liver: 162.9;stomach 1: 180.1	Cell type enhanced	Detected in many		Conjunctival goblet cells: 227.2;Endometrial secretory cells: 190.9;Fallopian secretory cells: 151.7;Gastric chief cells: 217.3;Hepatocytes: 206.5;Parietal cells: 222.8;Salivary duct cells: 157.4
brain: 21.2;pituitary gland: 7.6;retina: 6.4	Cell type enhanced	Detected in some		Brain excitatory neurons: 12.4;Brain inhibitory neurons: 9.5;Corticotrophs: 12.0;Lactotrophs: 9.0;Other brain neurons: 12.5;Pancreatic islet cells: 11.0;Retinal amacrine cells: 23.4;Retinal bipolar cells: 21.2;Retinal horizontal ce
	Cell type enriched	Detected in many	4	Neutrophils: 679.5
adrenal gland: 204.1;brain: 111.7;retina: 89.2	Cell type enhanced	Detected in many		Adrenal cortex cells: 1195.6;Cardiomyocytes: 381.5;Choroid plexus epithelial cells: 912.9;Cone photoreceptor cells: 1246.2;Cytotrophoblasts: 362.6;Distal convoluted tubule cells: 604.7;pDCs: 517.8;Proximal tubule cells: 97
esophagus: 58.0;kidney: 59.1	Cell type enhanced	Detected in many		Esophageal apical cells: 384.1;Esophageal basal cells: 142.9;Esophageal suprabasal cells: 174.9;Ocular epithelial cells: 121.6;Paneth cells: 143.1;Prostatic club cells: 121.3;Prostatic hillock cells: 132.1;Syncytiotrophoblasts:
adrenal gland: 11.8;brain: 10.5;retina: 36.4	Group enriched	Detected in many	8	Adrenal medulla cells: 1297.7;Brain inhibitory neurons: 1598.6;Oligodendrocyte progenitor cells: 1553.2;Retinal bipolar cells: 2905.6
brain: 30.5;testis: 31.4	Cell type enhanced	Detected in many		Adrenal medulla cells: 53.2;Brain excitatory neurons: 66.0;Brain inhibitory neurons: 59.0;Choroid plexus epithelial cells: 49.4;Distal convoluted tubule cells: 44.5;Early primary spermatocytes: 43.6;Gonadotrophs: 49.6;Oligode
brain: 6.8	Cell type enhanced	Detected in many		Brain excitatory neurons: 677.9;Brain inhibitory neurons: 895.2;Oligodendrocyte progenitor cells: 253.8;Other brain neurons: 372.0;Pituitary stem cells: 97.1;Retinal amacrine cells: 169.6;Retinal ganglion cells: 104.3;Tuft cells:
	Cell type enhanced	Detected in many		Alveolar cells type 1: 295.4;B-cells: 252.1;Fibro-adipogenic progenitors: 466.1;Fibroblasts: 242.9;Hepatic stellate cells: 322.7;Pericytes: 448.2;Podocytes: 420.4;Tuft cells: 726.3;Vascular endothelial cells: 272.8;Vascular smoo
pancreas: 19.1	Cell type enhanced	Detected in some		Brain inhibitory neurons: 3.1;Breast hormone-responsive cells: 3.0;Cone photoreceptor cells: 2.1;Neuroendocrine cells: 3.6;Oocytes: 13.8;Pancreatic acinar cells: 10.8;Retinal pigment epithelial cells: 4.3;Sertoli cells: 2.5;Thyro
skin 1: 58.0	Cell type enhanced	Detected in some		Colonocytes: 4.4;Epididymal principal cells: 8.6;Esophageal apical cells: 18.7;Kupffer cells: 17.4;Macrophages: 5.0;Paneth cells: 11.6;Retinal horizontal cells: 4.0;Retinal pigment epithelial cells: 8.5
bone marrow: 10.5;lymphoid tissue: 18.2;testis: 7.1	Cell type enhanced	Detected in many		Differentiating spermatogonia: 49.4;Early primary spermatocytes: 71.6;Enteric transient amplifying cells: 43.5;Erythrocyte progenitors: 65.6;Esophageal basal cells: 32.4;Extravillous trophoblasts: 40.4;Gastric progenitor cells:
bone marrow: 147.2	Group enriched	Detected in some	36	Erythrocyte progenitors: 235.2;Erythrocytes: 111.1
bone marrow: 415.6	Cell type enhanced	Detected in many		Decidual stromal cells: 281.4;Erythrocyte progenitors: 730.9;Erythrocytes: 882.7;Hofbauer cells: 948.9;Lymphatic endothelial cells: 384.7;Melanocytes: 397.6;Plasma cells: 234.4
testis: 11.3	Cell type enhanced	Detected in many		Cardiomyocytes: 92.7;Early spermatids: 92.2;Epicardial cells: 45.5;Late primary spermatocytes: 34.8;Late spermatids: 110.2
adipose tissue: 46.2;ovary: 19.3	Group enriched	Detected in some	5	Epicardial cells: 270.6;Mesothelial cells: 255.9
esophagus: 39.2;skin 1: 28.9	Group enriched	Detected in many	4	Epididymal basal cells: 444.6;Esophageal apical cells: 441.5
lymphoid tissue: 106.7	Cell type enhanced	Detected in many		cDC: 207.9;Hofbauer cells: 273.5;Kupffer cells: 311.6;Macrophages: 240.3;Microglia: 233.8;Monocyte progenitors: 186.6;monocytes: 572.5;Neutrophil progenitors: 433.6;Neutrophils: 2704.5;Tuft cells: 979.9
bone marrow: 145.0;lymphoid tissue: 290.6	Cell type enhanced	Detected in many		cDC: 293.7;Kupffer cells: 1301.6;Macrophages: 412.0;Monocyte progenitors: 208.8;monocytes: 470.3;Neutrophil progenitors: 443.0;Neutrophils: 1495.5;Syncytiotrophoblasts: 201.4
	Cell type enhanced	Detected in some		Brain excitatory neurons: 17.2;Brain inhibitory neurons: 24.1;Gonadotrophs: 8.5;Other brain neurons: 34.7;Retinal amacrine cells: 22.1
	Cell type enhanced	Detected in many		Hofbauer cells: 27.7;Kupffer cells: 74.8;Platelets: 22.6
	Cell type enhanced	Detected in many		Decidual stromal cells: 346.9;Enterocytes: 391.9;Epididymal efferent duct absorptive cells: 304.0;Neutrophils: 349.5;Platelets: 739.3
lung: 261.7;lymphoid tissue: 376.0	Cell type enhanced	Detected in many		B-cells: 630.3;cDC: 6181.4;Kupffer cells: 1637.8;Macrophages: 1467.9;monocytes: 551.9;pDCs: 486.5
	Cell type enhanced	Detected in many		Esophageal apical cells: 84.6;Kupffer cells: 90.2;Lymphatic endothelial cells: 85.2;Microglia: 148.7;Neutrophils: 206.6;NK-cells: 96.3;Plasma cells: 120.6;Platelets: 271.6;Vascular endothelial cells: 93.3
liver: 364.9	Cell type enhanced	Detected in many		Alveolar cells type 2: 590.3;Breast lactating cells: 390.4;Colonocytes: 539.3;Epididymal principal cells: 1140.0;Esophageal apical cells: 526.1;Esophageal suprabasal cells: 633.0;Retinal ganglion cells: 498.3;Suprabasal kerati
	Cell type enhanced	Detected in many		Endometrial secretory cells: 5.5;Lymphatic endothelial cells: 11.2;monocytes: 5.4;Ocular epithelial cells: 10.3;Retinal pigment epithelial cells: 8.5
	Cell type enhanced	Detected in many		Endometrial glandular cells: 244.0;Endometrial luminal cells: 139.3;Endometrial secretory cells: 179.9;Enterocytes: 141.1;Epididymal basal cells: 161.2;Epididymal efferent duct ciliated cells: 169.1;Fallopian secretory cells: 21
	Cell type enhanced	Detected in all		Pancreatic duct cells: 2924.7
esophagus: 6918.5	Cell type enhanced	Detected in many		Decidual stromal cells: 6655.6;Epididymal efferent duct absorptive cells: 4667.1;Esophageal apical cells: 36565.4;Esophageal basal cells: 10114.9;Esophageal suprabasal cells: 24428.9
parathyroid gland: 12.3;testis: 8.2	Cell type enhanced	Detected in some		Brain excitatory neurons: 23.4;Brain inhibitory neurons: 19.7;cDC: 42.1;Hepatic stellate cells: 45.2;Hofbauer cells: 15.0;Late primary spermatocytes: 21.6;Macrophages: 30.1;monocytes: 16.0;Other brain neurons: 25.0;Undiffe
ovary: 751.4	Cell type enhanced	Detected in many		Extravillous trophoblasts: 291.5;Microglia: 310.8;Müller glia: 431.4;Oligodendrocyte progenitor cells: 316.1;Podocytes: 616.6;Retinal horizontal cells: 530.9;Retinal pigment epithelial cells: 268.6;Schwann cells: 270.3
	Cell type enhanced	Detected in many		Choroid plexus epithelial cells: 252.8;Esophageal apical cells: 248.7;Melanocytes: 257.2;Pituicytes/FSCs: 570.7;Retinal pigment epithelial cells: 264.3;Schwann cells: 248.4
	Cell type enhanced	Detected in all		Cardiomyocytes: 494.9;Neutrophil progenitors: 295.5;Neutrophils: 1348.6
	Cell type enhanced	Detected in some		B-cells: 2.6;cDC: 2.1;Endometrial glandular cells: 1.8;Esophageal apical cells: 2.5;Foveolar cells: 2.7;pDCs: 3.2;Podocytes: 3.1;Renal connecting tubule cells: 2.1
	Cell type enhanced	Detected in many		Alveolar cells type 1: 39.5;Epididymal basal cells: 47.5
	Cell type enhanced	Detected in many		Decidual stromal cells: 121.3;Endometrial secretory cells: 263.5;Epididymal efferent duct absorptive cells: 358.1;Epididymal principal cells: 250.8;Fallopian secretory cells: 105.2;Leydig cells: 177.0;Neutrophils: 238.3;NK-cells
pancreas: 6.9	Not detected	Not detected		
	Cell type enhanced	Detected in many		cDC: 374.8;Kupffer cells: 520.7;Microglia: 673.8;monocytes: 360.1;Neutrophils: 1082.7
	Cell type enhanced	Detected in many		Neutrophils: 2488.0;Podocytes: 1295.1;Proximal tubule cells: 1339.2
liver: 47.1	Cell type enhanced	Detected in many		cDC: 17.1;Hepatocytes: 22.1;Hofbauer cells: 13.0;Macrophages: 8.8;Mast cells: 7.7;Microglia: 8.3;monocytes: 10.3;NK-cells: 7.8;pDCs: 23.0;Plasma cells: 9.9;Tuft cells: 11.5
bone marrow: 54.5;lymphoid tissue: 71.6	Cell type enhanced	Detected in many		cDC: 186.9;Extravillous trophoblasts: 162.3;Hofbauer cells: 83.9;Kupffer cells: 342.3;Macrophages: 166.4;Microglia: 94.5;monocytes: 232.6;Neutrophils: 206.8;NK-cells: 87.3;pDCs: 108.8;Plasma cells: 87.3;T-cells: 113.9
	Cell type enhanced	Detected in many		Alveolar cells type 1: 53.0;Cholangiocytes: 63.3;Decidual stromal cells: 91.7;Endometrial stromal cells: 53.2;Fallopian secretory cells: 50.1;Fibroblasts: 69.8;Pancreatic acinar cells: 68.3;Pancreatic duct cells: 47.7;Sertoli cells:
brain: 17.2;cervix: 16.9;kidney: 55.8;liver: 31.1	Cell type enriched	Detected in many	9	Tuft cells: 657.1
choroid plexus: 58.2;kidney: 116.5;lung: 158.6;placenta: 187.6	Group enriched	Detected in some	5	Epididymal basal cells: 119.9;Mast cells: 329.3
intestine: 109.3;lymphoid tissue: 317.8	Cell type enhanced	Detected in some		B-cells: 3.2;cDC: 2.3;Enterocytes: 2.0;Hematopoietic stem cells: 1.9;Innate lymphoid cells: 7.7;Megakaryocyte progenitors: 2.0;Neutrophils: 7.4;NK-cells: 11.3;pDCs: 3.2;Plasma cells: 9.3;T-cells: 6.8;Thymocytes: 2.5
	Cell type enhanced	Detected in many		Epicardial cells: 277.4;Mast cells: 319.7;Neutrophil progenitors: 208.4;Neutrophils: 757.8
	Cell type enhanced	Detected in all		Endometrial glandular cells: 1204.2;Microglia: 759.0;Platelets: 681.9
lymphoid tissue: 171.2	Group enriched	Detected in many	5	Innate lymphoid cells: 3240.4;T-cells: 1496.2
	Cell type enhanced	Detected in all		Neutrophils: 3203.3
	Cell type enhanced	Detected in many		Neutrophils: 377.5
	Cell type enhanced	Detected in many		Alveolar cells type 1: 500.5;Papillary tip epithelial cells: 210.3;Podocytes: 417.1;Transitional alveolar cells: 168.5;Urothelial cells: 243.2
bone marrow: 45.4;lung: 56.9;lymphoid tissue: 66.2	Cell type enhanced	Detected in some		cDC: 292.5;Macrophages: 204.2;Mast cells: 195.6;Microglia: 386.4;monocytes: 557.7;Neutrophils: 1628.0
	Cell type enhanced	Detected in all		Endometrial glandular cells: 770.3;Pancreatic duct cells: 750.3;Platelets: 1111.7
bone marrow: 343.1;lymphoid tissue: 162.7	Cell type enhanced	Detected in many		cDC: 399.5;Hofbauer cells: 522.8;Kupffer cells: 222.4;Macrophages: 344.6;Monocyte progenitors: 358.2;monocytes: 613.7;Neutrophil progenitors: 309.9;Neutrophils: 726.1;NK-cells: 269.4;T-cells: 148.4
blood vessel: 37.6;thyroid gland: 52.7	Cell type enriched	Detected in some	6	Platelets: 76.0
salivary gland: 198.1;skin 1: 198.6	Cell type enhanced	Detected in many		Foveolar cells: 218.4;Respiratory basal cells: 233.5;Salivary basal cells: 319.2;Salivary myoepithelial cells: 212.2;Schwann cells: 309.7
	Cell type enhanced	Detected in all		Adipocytes: 928.3;Macrophages: 452.1;Rod photoreceptor cells: 517.7
thyroid gland: 442.1	Cell type enhanced	Detected in some		Colonocytes: 26.1;Enteric transient amplifying cells: 12.8;Enterocytes: 102.1;Foveolar cells: 14.0;Gastric progenitor cells: 24.4;Goblet cells: 33.1;Hepatocytes: 35.7;Paneth cells: 19.3;Proximal tubule cells: 99.3
placenta: 59.6	Cell type enhanced	Detected in many		Adrenal cortex cells: 76.3;Astrocytes: 114.8;Bergmann glia: 150.5;Cardiomyocytes: 93.0;Cone photoreceptor cells: 64.2;Microglia: 101.3;Oligodendrocyte progenitor cells: 75.6;Pituicytes/FSCs: 107.5;Sertoli cells: 322.1;Vasc
lung: 40.7;lymphoid tissue: 64.8	Cell type enhanced	Detected in many		cDC: 419.8;Hematopoietic stem cells: 70.4;Innate lymphoid cells: 120.2;Macrophages: 136.8;monocytes: 203.1;Neutrophils: 649.0;pDCs: 284.8;T-cells: 160.5
esophagus: 914.3;skin 1: 886.7	Cell type enhanced	Detected in many		Esophageal apical cells: 2248.1;Esophageal basal cells: 688.2;Esophageal suprabasal cells: 1555.8;Ocular epithelial cells: 617.7;Suprabasal keratinocytes: 998.3;Urothelial cells: 525.0
	Cell type enhanced	Detected in many		Choroid plexus epithelial cells: 1717.9;Cytotrophoblasts: 1022.3;Distal convoluted tubule cells: 1880.8;Pituitary stem cells: 3107.4;Podocytes: 2505.6
	Low cell type specificity	Detected in all		
	Cell type enhanced	Detected in all		Cardiomyocytes: 269.0;Late primary spermatocytes: 281.3;Late spermatids: 394.5
brain: 9.1	Not detected	Not detected		
blood vessel: 91.5;brain: 61.2	Cell type enhanced	Detected in some		Early spermatids: 45.9;Gonadotrophs: 47.7;Medullary thymic epithelial cells: 28.8;Oligodendrocytes: 25.9;Pericytes: 36.6;Vascular smooth muscle cells: 167.5
intestine: 66.2;stomach 1: 53.0	Cell type enhanced	Detected in many		Conjunctival goblet cells: 45.6;Enteric stem cells: 45.2;Foveolar cells: 50.1;Gastric progenitor cells: 87.2;Hofbauer cells: 68.3;Kupffer cells: 133.2;Megakaryocytes: 98.9;Monocyte progenitors: 56.9;monocytes: 73.4;Mucous n
retina: 3.6	Cell type enriched	Detected in some	6	Retinal bipolar cells: 74.0
brain: 10.2;pituitary gland: 10.5;retina: 3.2	Cell type enhanced	Detected in many		Adrenal medulla cells: 510.6;Corticotrophs: 377.5;Lactotrophs: 885.9;Oligodendrocyte progenitor cells: 436.4;Oligodendrocytes: 1012.7;Sertoli cells: 233.7;Somatotrophs: 1041.5;Thyrotrophs: 745.9
blood vessel: 26.6	Cell type enhanced	Detected in some		Epididymal efferent duct absorptive cells: 10.3;Fibroblasts: 6.5;Hepatic stellate cells: 19.4;Innate lymphoid cells: 7.0;Pancreatic islet cells: 38.2;pDCs: 33.2;Pericytes: 19.6;Thymocytes: 46.6;Vascular smooth muscle cells: 6.7
salivary gland: 119.1	Cell type enhanced	Detected in many		Breast lactating cells: 306.6;Breast secretory cells: 106.7;Cytotrophoblasts: 282.4;Migrating cytotrophoblasts: 159.9;Salivary acinar cells: 90.8;Syncytiotrophoblasts: 994.7
adrenal gland: 196.1;stomach 1: 96.3	Group enriched	Detected in many	4	Adrenal cortex cells: 1118.5;Epicardial cells: 2417.4
brain: 24.0	Cell type enhanced	Detected in some		Alveolar cells type 2: 2.7;Brain excitatory neurons: 1.5;Breast secretory cells: 1.1;Cholangiocytes: 6.7;Differentiating spermatogonia: 1.4;Mesothelial cells: 1.0;Salivary acinar cells: 1.2;Salivary myoepithelial cells: 1.3
skeletal muscle: 80.6;tongue: 42.5	Cell type enhanced	Detected in many		Bergmann glia: 51.1;Breast lactating cells: 50.3;Fibro-adipogenic progenitors: 63.7;Myonuclei: 160.7;Myosatellite cells: 57.5;Oligodendrocyte progenitor cells: 52.4;Retinal pigment epithelial cells: 63.9
brain: 53.2;lymphoid tissue: 14.7;skeletal muscle: 12.6	Cell type enhanced	Detected in some		Brain excitatory neurons: 49.2;Brain inhibitory neurons: 66.8;Gonadotrophs: 25.6;Lactotrophs: 14.5;Myonuclei: 22.6;Oocytes: 18.4;Other brain neurons: 23.6;Retinal ganglion cells: 11.6
brain: 12.7	Cell type enhanced	Detected in some		Brain excitatory neurons: 12.9;Brain inhibitory neurons: 3.3;Enteric stem cells: 3.7;Enteric transient amplifying cells: 1.7;Goblet cells: 2.2;Lactotrophs: 2.1;Neuroendocrine cells: 5.6;Other brain neurons: 1.9;Paneth cells: 7.7
bone marrow: 53.1;testis: 36.6	Group enriched	Detected in some	4	Erythrocyte progenitors: 28.6;Oligodendrocytes: 32.9;Sertoli cells: 22.8
	Low cell type specificity	Detected in many		
brain: 3.5;pancreas: 1.3;pituitary gland: 1.0	Group enriched	Detected in some	10	Extravillous trophoblasts: 73.4;Pancreatic acinar cells: 105.6
breast: 79.2	Cell type enhanced	Detected in many		Breast secretory cells: 230.0;Innate lymphoid cells: 512.9;Mast cells: 1765.7;Melanocytes: 649.5;Renal collecting duct intercalated cells: 569.2;Respiratory ionocytes: 396.2;Salivary ionocytes: 263.8;Tuft cells: 253.6
brain: 147.1	Cell type enhanced	Detected in many		Brain excitatory neurons: 42.6
esophagus: 90.8;skin 1: 189.2	Group enriched	Detected in some	4	Esophageal apical cells: 1071.4;Salivary duct cells: 306.6
bone marrow: 20.8;lymphoid tissue: 39.4	Group enriched	Detected in some	12	NK-cells: 22.5;Oligodendrocyte progenitor cells: 25.1;Oligodendrocytes: 8.3;T-cells: 12.0
brain: 133.9;intestine: 47.5	Cell type enhanced	Detected in some		Adrenal medulla cells: 85.1;Brain excitatory neurons: 32.9;Brain inhibitory neurons: 25.3;Melanocytes: 47.9;Other brain neurons: 44.3;Renal collecting duct principal cells: 41.0;Retinal ganglion cells: 34.8;Retinal horizontal cel
lung: 27.1;lymphoid tissue: 44.5	Cell type enriched	Detected in some	5	Microglia: 84.6
	Cell type enhanced	Detected in all		Esophageal apical cells: 611.4;Neutrophils: 799.9;Oligodendrocytes: 577.1;Syncytiotrophoblasts: 572.9
	Cell type enhanced	Detected in all		Hofbauer cells: 630.3;Megakaryocytes: 1199.2;Platelets: 2059.1
lymphoid tissue: 105.7	Cell type enhanced	Detected in some		Astrocytes: 9.0;Bergmann glia: 5.0;Brain excitatory neurons: 5.8;Brain inhibitory neurons: 7.2;Choroid plexus epithelial cells: 4.7;Distal convoluted tubule cells: 6.8;Loop of henle epithelial cells: 7.2;Mast cells: 5.0;Megakaryoc
bone marrow: 57.8;lymphoid tissue: 53.0	Cell type enhanced	Detected in many		B-cells: 118.3;cDC: 132.4;Hofbauer cells: 106.7;Macrophages: 102.1;Mast cells: 199.4;Megakaryocyte progenitors: 76.9;Microglia: 142.1;Monocyte progenitors: 125.5;monocytes: 124.5;Neutrophil progenitors: 75.1;Neutroph
lymphoid tissue: 19.8	Cell type enhanced	Detected in many		B-cells: 21.4;Mast cells: 226.7;NK-cells: 51.2;Plasma cells: 103.0;T-cells: 60.7
bone marrow: 669.6;epididymis: 380.4;lymphoid tissue: 566.7	Cell type enhanced	Detected in many		cDC: 637.7;Epididymal principal cells: 754.0;Kupffer cells: 562.7;Late spermatids: 534.6;Monocyte progenitors: 1308.9;monocytes: 1174.8;Neutrophil progenitors: 1996.1;Neutrophils: 6753.3;NK-cells: 569.2
liver: 121.9	Cell type enhanced	Detected in many		Choroid plexus epithelial cells: 174.1;Fibro-adipogenic progenitors: 188.5;Fibroblasts: 237.9;Gastric chief cells: 252.3;Hepatocytes: 226.1;Parietal cells: 139.7;Podocytes: 204.7;Vascular endothelial cells: 189.8
ovary: 2.1;testis: 2.1	Cell type enriched	Detected in single	7	Adipocytes: 3.4
brain: 109.9	Cell type enhanced	Detected in many		Distal convoluted tubule cells: 244.0;Esophageal apical cells: 382.1;Foveolar cells: 346.1;Late spermatids: 288.7;Loop of henle epithelial cells: 270.9;Mucous neck cells: 269.6;Papillary tip epithelial cells: 302.6;Podocytes: 28
epididymis: 395.8	Cell type enriched	Detected in some	9	Epididymal principal cells: 497.2
	Cell type enriched	Detected in many	8	Neutrophils: 7072.3
	Cell type enhanced	Detected in many		Distal convoluted tubule cells: 252.4;Early spermatids: 246.9;Endometrial ciliated cells: 291.1;Endometrial glandular cells: 998.6;Endometrial luminal cells: 500.4;Esophageal apical cells: 652.3
fallopian tube: 34.2	Cell type enhanced	Detected in many		Basal prostatic cells: 49.6;Endometrial luminal cells: 124.8;Ependymal cells: 59.5;Fallopian secretory cells: 36.4;Fallopian tube ciliated cells: 106.4;Pancreatic acinar cells: 36.8;Prostatic glandular cells: 84.4;Prostatic hillock c
ovary: 3.1	Cell type enhanced	Detected in some		Astrocytes: 19.6;Bergmann glia: 19.3;Ependymal cells: 34.8;Fibro-adipogenic progenitors: 7.3;Leydig cells: 44.7;Megakaryocytes: 6.2;Oligodendrocyte progenitor cells: 9.9;Ovarian stromal cells: 6.2;Peritubular myoid cells: 6.
	Cell type enhanced	Detected in many		cDC: 313.3;Early spermatids: 442.5;Epicardial cells: 343.4;Esophageal apical cells: 319.1;Late spermatids: 412.9;Macrophages: 227.8;Microglia: 893.3
blood vessel: 128.4	Cell type enhanced	Detected in many		Late spermatids: 2886.1;Salivary myoepithelial cells: 2477.7;Smooth muscle cells: 3744.0;Vascular smooth muscle cells: 2741.1
brain: 6.3;retina: 2.9	Cell type enriched	Detected in many	8	Cone photoreceptor cells: 865.3
adipose tissue: 102.4	Cell type enhanced	Detected in many		Adipocytes: 513.4;Fibro-adipogenic progenitors: 289.7;Fibroblasts: 444.0;Hepatocytes: 236.8;Kupffer cells: 253.2;Leydig cells: 345.4;monocytes: 220.6;Oligodendrocyte progenitor cells: 334.9;Peritubular myoid cells: 279.1
salivary gland: 285.7	Cell type enhanced	Detected in many		Breast hormone-responsive cells: 74.9;Breast lactating cells: 112.7;Breast secretory cells: 116.5;Gastric chief cells: 131.0;Goblet cells: 582.6;Lacrimal acinar cells: 317.9;pDCs: 113.0;Prostatic glandular cells: 180.2;Salivary a
liver: 138.5	Cell type enhanced	Detected in many		Colonocytes: 294.5;Cytotrophoblasts: 246.0;Esophageal apical cells: 228.9;Migrating cytotrophoblasts: 222.8;Retinal pigment epithelial cells: 362.4;Syncytiotrophoblasts: 286.3;Urothelial cells: 228.4
bone marrow: 94.4;lymphoid tissue: 180.8	Cell type enhanced	Detected in some		cDC: 432.6;Hofbauer cells: 210.4;Innate lymphoid cells: 105.7;Kupffer cells: 966.5;Macrophages: 204.2;Megakaryocytes: 105.1;Microglia: 150.9;Monocyte progenitors: 134.4;monocytes: 476.4;Neutrophil progenitors: 120.2;N
esophagus: 36.1;skin 1: 44.9	Not detected	Not detected		
bone marrow: 69.6;lymphoid tissue: 80.3	Cell type enriched	Detected in many	4	Neutrophils: 6578.7
	Cell type enhanced	Detected in all		Ocular epithelial cells: 2163.3
brain: 101.0	Cell type enhanced	Detected in many		Cone photoreceptor cells: 180.1;Platelets: 218.8;Retinal horizontal cells: 150.3;Rod photoreceptor cells: 154.2
	Cell type enhanced	Detected in many		Choroid plexus epithelial cells: 2121.6;Renal collecting duct intercalated cells: 8162.6;Retinal horizontal cells: 2414.1
retina: 32.2	Cell type enhanced	Detected in many		Alveolar cells type 1: 1287.9;Renal collecting duct intercalated cells: 1059.8;Transitional alveolar cells: 1217.7
	Cell type enhanced	Detected in all		cDC: 568.3;Esophageal apical cells: 779.4;monocytes: 1194.8;Neutrophil progenitors: 560.0;Neutrophils: 799.4
	Low cell type specificity	Detected in many		
brain: 255.8;pituitary gland: 113.0	Cell type enhanced	Detected in many		Corticotrophs: 49.6;Gonadotrophs: 64.0;Late spermatids: 43.2;Oligodendrocytes: 76.1;Pancreatic islet cells: 38.5;Retinal horizontal cells: 52.1;Somatotrophs: 38.7
brain: 147.9;skeletal muscle: 83.6	Group enriched	Detected in many	4	Astrocytes: 295.6;Bergmann glia: 241.1;Brain excitatory neurons: 320.3;Brain inhibitory neurons: 251.0;Late spermatids: 124.4;Oligodendrocyte progenitor cells: 237.0;Oligodendrocytes: 379.1;Other brain neurons: 295.2;Po
	Cell type enhanced	Detected in many		Esophageal apical cells: 931.9;Neutrophils: 1059.3;Urothelial cells: 964.5
	Cell type enhanced	Detected in many		Neutrophils: 377.7
thyroid gland: 14.6	Cell type enhanced	Detected in many		Choroid plexus epithelial cells: 26.6;Distal convoluted tubule cells: 44.4;Loop of henle epithelial cells: 43.1;Papillary tip epithelial cells: 26.1;Renal collecting duct intercalated cells: 31.6;Renal collecting duct principal cells: 21.
cervix: 2.9;epididymis: 1.8	Not detected	Not detected		
epididymis: 2.5;lymphoid tissue: 1.0	Group enriched	Detected in some	4	Early spermatids: 7.0;Esophageal apical cells: 2.4;Late spermatids: 9.0;Medullary thymic epithelial cells: 4.8
adipose tissue: 312.2;blood vessel: 1211.4	Cell type enhanced	Detected in many		Adipocytes: 110.0;Breast myoepithelial cells: 86.1;Müller glia: 128.3;Pericytes: 272.2;Salivary myoepithelial cells: 82.1;Schwann cells: 79.7;Smooth muscle cells: 290.4;Vascular endothelial cells: 102.6;Vascular smooth muscl
bone marrow: 77.3;lung: 82.9;lymphoid tissue: 49.5	Cell type enriched	Detected in some	4	Neutrophils: 82.5
adrenal gland: 22.6;brain: 11.6;seminal vesicle: 14.3;testis: 14.0	Cell type enhanced	Detected in many		Adrenal cortex cells: 139.3;Brain inhibitory neurons: 42.4;Cone photoreceptor cells: 111.8;Gonadotrophs: 64.1;Retinal ganglion cells: 42.5;Sertoli cells: 378.7

brain: 5.1;testis: 1.4	Cell type enhanced	Detected in some		Brain excitatory neurons: 19.7;Brain inhibitory neurons: 7.9;Late primary spermatocytes: 3.4;Late spermatids: 4.4;Medullary thymic epithelial cells: 2.4;Retinal amacrine cells: 6.2;Retinal pigment epithelial cells: 4.3
	Cell type enhanced	Detected in many		Gonadotrophs: 84.9;Hofbauer cells: 111.6;Kupffer cells: 136.3;Macrophages: 67.5;Neutrophils: 74.0
brain: 80.5	Cell type enhanced	Detected in many		Adrenal medulla cells: 53.2;Brain excitatory neurons: 50.5;Cone photoreceptor cells: 60.0;Gonadotrophs: 45.7;Pituitary stem cells: 39.1;Retinal amacrine cells: 48.2;Retinal bipolar cells: 59.4;Retinal horizontal cells: 66.1
bone marrow: 5.1;lung: 3.9;lymphoid tissue: 8.5	Cell type enriched	Detected in some	4	Neutrophils: 286.6
brain: 12.2;retina: 7.4	Cell type enhanced	Detected in many		Astrocytes: 300.8;Ependymal cells: 70.4;Müller glia: 186.0;Myosatellite cells: 365.0;Oligodendrocyte progenitor cells: 108.9;Oligodendrocytes: 455.9;Other brain neurons: 109.2;Pituicytes/FSCs: 127.5;Pituitary stem cells: 80.
choroid plexus: 89.9	Cell type enhanced	Detected in many		Adrenal cortex cells: 246.3;Astrocytes: 223.7;Bergmann glia: 193.1;Choroid plexus epithelial cells: 188.0;Kupffer cells: 732.8;Macrophages: 427.7;Microglia: 378.9;monocytes: 167.3;Pituicytes/FSCs: 247.6
testis: 42.4	Cell type enriched	Detected in many	5	Late spermatids: 3094.5
epididymis: 92.1;liver: 153.9;seminal vesicle: 75.3;skin 1: 84.5	Cell type enhanced	Detected in many		Alveolar cells type 2: 353.3;Enterocytes: 345.7;Epididymal efferent duct absorptive cells: 262.4;Epididymal principal cells: 323.0;Hepatocytes: 354.4;Sertoli cells: 102.7
	Cell type enhanced	Detected in many		Cytotrophoblasts: 67.3;Esophageal apical cells: 84.4;Esophageal suprabasal cells: 80.4;Migrating cytotrophoblasts: 63.1;Parietal cells: 93.7;Syncytiotrophoblasts: 69.9
	Cell type enhanced	Detected in many		Esophageal apical cells: 443.5;Esophageal suprabasal cells: 269.6;Gastric progenitor cells: 464.0;Late primary spermatocytes: 271.9;Syncytiotrophoblasts: 341.9
intestine: 185.2	Cell type enhanced	Detected in many		Alveolar cells type 1: 116.6;Colonocytes: 703.5;Enteric stem cells: 135.8;Enteric transient amplifying cells: 190.7;Enterocytes: 1134.4;Fallopian tube ciliated cells: 126.0;Foveolar cells: 103.8;Goblet cells: 251.5;Paneth cells: 1
	Cell type enhanced	Detected in many		Hofbauer cells: 37.5;Macrophages: 46.9;Monocyte progenitors: 54.3;monocytes: 69.4;Neutrophil progenitors: 74.8;Neutrophils: 118.8;Plasma cells: 58.0;Vascular endothelial cells: 69.7
bone marrow: 1.2;thyroid gland: 1.6	Cell type enhanced	Detected in single		Neutrophil progenitors: 1.8
intestine: 314.1;kidney: 282.6	Cell type enhanced	Detected in many		Adipocytes: 329.8;Basal prostatic cells: 283.8;Breast myoepithelial cells: 274.7;Endometrial stromal cells: 456.0;Enterocytes: 898.7;Fibro-adipogenic progenitors: 274.6;Neutrophils: 1431.5;Podocytes: 2387.7;Prostatic glandu
bone marrow: 31.3;lymphoid tissue: 75.1	Cell type enriched	Detected in many	8	Neutrophils: 577.0
	Cell type enhanced	Detected in some		Cardiomyocytes: 14.8;Hematopoietic stem cells: 40.3;Megakaryocyte progenitors: 44.2;Megakaryocytes: 30.9;Platelets: 111.2;Thymocytes: 10.2
bone marrow: 110.2	Cell type enhanced	Detected in many		Cytotrophoblasts: 528.4;Erythrocyte progenitors: 184.6;Erythrocytes: 305.6;Hofbauer cells: 412.4;Megakaryocytes: 463.6;Migrating cytotrophoblasts: 211.1;Neutrophils: 297.6;Platelets: 1825.7;Syncytiotrophoblasts: 518.1
esophagus: 34.6	Cell type enhanced	Detected in many		Cytotrophoblasts: 381.2;Lymphatic endothelial cells: 424.1;Neutrophil progenitors: 655.5;Neutrophils: 609.7;Renal collecting duct intercalated cells: 408.4
	Low cell type specificity	Detected in many		
	Cell type enhanced	Detected in many		Neutrophils: 79.2
endometrium 1: 169.7;intestine: 162.2	Cell type enhanced	Detected in some		Decidual stromal cells: 136.3;Fibroblasts: 45.8;Hepatic stellate cells: 64.5;Leydig cells: 42.0;Ovarian stromal cells: 29.0;Pericytes: 81.4;Peritubular myoid cells: 101.6;Podocytes: 56.5;Smooth muscle cells: 157.5;Vascular smo
lymphoid tissue: 631.0	Cell type enriched	Detected in some	52	B-cells: 884.8
adipose tissue: 89.2;fallopian tube: 261.5	Cell type enhanced	Detected in many		Alveolar cells type 1: 375.8;Conjunctival goblet cells: 998.0;Endometrial luminal cells: 129.4;Endometrial secretory cells: 125.4;Fallopian secretory cells: 685.4;Mesothelial cells: 266.2;Respiratory deuterosomal cells: 215.6;Re
	Cell type enhanced	Detected in many		Alveolar cells type 1: 480.3;Megakaryocytes: 544.0;Monocyte progenitors: 438.5;monocytes: 512.9;Neutrophil progenitors: 510.8;Neutrophils: 900.6;Platelets: 881.1;Vascular endothelial cells: 465.1
	Cell type enhanced	Detected in all		Sertoli cells: 189.5
kidney: 416.9;stomach 1: 998.2	Cell type enhanced	Detected in many		Alveolar cells type 1: 192.9;Alveolar cells type 2: 236.0;Breast lactating cells: 217.7;Conjunctival goblet cells: 94.2;Endometrial luminal cells: 102.3;Endometrial secretory cells: 154.6;Foveolar cells: 271.9;Gastric chief cells: 1
blood vessel: 2385.4;intestine: 3136.0;urinary bladder: 2375.2	Group enriched	Detected in many	4	Peritubular myoid cells: 550.0;Smooth muscle cells: 1993.2;Vascular smooth muscle cells: 1312.5
seminal vesicle: 1572.0;smooth muscle: 1933.2	Cell type enhanced	Detected in many		Breast myoepithelial cells: 2281.9;Hepatic stellate cells: 645.5;Megakaryocytes: 537.8;Peritubular myoid cells: 652.1;Platelets: 660.1;Salivary myoepithelial cells: 1108.7;Smooth muscle cells: 5303.5;Vascular smooth muscle
	Cell type enhanced	Detected in many		Endometrial glandular cells: 638.1;Endometrial luminal cells: 973.4;Granulosa cells: 665.0;Melanocytes: 829.4;Renal collecting duct intercalated cells: 607.5;Sertoli cells: 605.9
liver: 238.9	Cell type enhanced	Detected in many		Alveolar cells type 1: 592.5;Alveolar cells type 2: 394.4;Hepatocytes: 807.1;Pancreatic duct cells: 354.7;Pericytes: 639.3;Podocytes: 772.0;Renal connecting tubule cells: 492.2;Tuft cells: 461.1
	Cell type enhanced	Detected in many		Adipocytes: 194.1;Alveolar cells type 1: 295.3
bone marrow: 41.1;lymphoid tissue: 25.3	Cell type enhanced	Detected in some		B-cells: 41.3;cDC: 44.2;Innate lymphoid cells: 26.7;Macrophages: 29.7;Megakaryocyte-Erythroid progenitors: 24.1;Monocyte progenitors: 98.6;monocytes: 154.3;Neutrophil progenitors: 47.6;Neutrophils: 30.6;NK-cells: 69.0;
	Cell type enhanced	Detected in many		Endometrial glandular cells: 1114.0;Endometrial secretory cells: 712.1;Prostatic hillock cells: 676.7;Urothelial cells: 1157.3
brain: 15.1	Cell type enhanced	Detected in many		Adrenal medulla cells: 255.3;Astrocytes: 233.4;Bergmann glia: 241.6;Brain excitatory neurons: 331.6;Brain inhibitory neurons: 382.8;Corticotrophs: 326.5;Gonadotrophs: 418.8;Lactotrophs: 453.9;Oligodendrocyte progenitor
brain: 76.3	Cell type enhanced	Detected in many		Brain excitatory neurons: 36.6;Brain inhibitory neurons: 33.5;Corticotrophs: 20.4;Lactotrophs: 17.4;Medullary thymic epithelial cells: 16.8;Other brain neurons: 35.5;Prostatic glandular cells: 29.8;Retinal amacrine cells: 27.3;S
	Low cell type specificity	Detected in all		
brain: 187.4;heart muscle: 136.4	Cell type enhanced	Detected in many		Adrenal medulla cells: 819.0;Astrocytes: 954.9;Bergmann glia: 1167.3;Brain excitatory neurons: 590.3;Brain inhibitory neurons: 699.3;Cardiomyocytes: 1069.7;Lactotrophs: 552.0;Müller glia: 780.8;Oligodendrocyte progenitor
adrenal gland: 11.3;brain: 38.9	Cell type enhanced	Detected in many		Adrenal cortex cells: 1097.0;Adrenal medulla cells: 1106.2;Astrocytes: 1271.1;Bergmann glia: 2749.5;Brain excitatory neurons: 1376.0;Brain inhibitory neurons: 926.8;Ependymal cells: 975.9;Oligodendrocyte progenitor cells:
lymphoid tissue: 43.0	Cell type enhanced	Detected in many		B-cells: 112.0;cDC: 143.8;Hematopoietic stem cells: 111.1;Hofbauer cells: 199.5;Kupffer cells: 260.3;Macrophages: 193.2;Microglia: 262.5;Monocyte progenitors: 162.5;monocytes: 227.4;Neutrophil progenitors: 263.0;Neutr
brain: 357.2	Cell type enhanced	Detected in many		Brain excitatory neurons: 63.4;Ependymal cells: 61.2;Proximal tubule cells: 64.5;Retinal amacrine cells: 69.4;Retinal horizontal cells: 70.1;Smooth muscle cells: 150.9;Vascular smooth muscle cells: 65.8
cervix: 40.7;esophagus: 96.0;salivary gland: 32.2;skin 1: 110.2;vagi	Cell type enhanced	Detected in many		Esophageal apical cells: 743.5;Esophageal suprabasal cells: 312.2;Extravillous trophoblasts: 115.1;Ocular epithelial cells: 247.1;Prostatic hillock cells: 174.2;Suprabasal keratinocytes: 233.0;Syncytiotrophoblasts: 571.5;Uroth
	Cell type enhanced	Detected in many		Astrocytes: 371.6;Bergmann glia: 368.6;Myonuclei: 631.1;Oligodendrocytes: 518.2
	Cell type enhanced	Detected in some		Astrocytes: 8.0;Brain excitatory neurons: 10.3;Brain inhibitory neurons: 9.1;Distal convoluted tubule cells: 10.7;Ependymal cells: 7.0;Loop of henle epithelial cells: 9.7;Microglia: 9.2;Oligodendrocyte progenitor cells: 8.5;Other
	Low cell type specificity	Detected in all		
	Cell type enhanced	Detected in all		Late spermatids: 176.3
	Cell type enhanced	Detected in many		Basal prostatic cells: 43.9;Breast myoepithelial cells: 54.2;Epididymal basal cells: 36.2;Hepatic stellate cells: 32.3;Leydig cells: 111.7;Müller glia: 157.1;Peritubular myoid cells: 30.4;Respiratory ionocytes: 76.2;Salivary ionocy
	Cell type enhanced	Detected in many		Distal convoluted tubule cells: 943.6;Endometrial glandular cells: 697.0;Loop of henle epithelial cells: 588.4;Pituicytes/FSCs: 936.1;Proximal tubule cells: 1595.4;Sertoli cells: 743.1
esophagus: 591.8	Cell type enriched	Detected in many	4	Esophageal apical cells: 2283.1
ovary: 57.9;placenta: 57.1	Cell type enhanced	Detected in many		Adipocytes: 41.5;Cardiomyocytes: 72.4;Choroid plexus epithelial cells: 97.5;Epicardial cells: 70.5;Fibro-adipogenic progenitors: 93.2;Fibroblasts: 53.8;Hepatic stellate cells: 58.1;Mesothelial cells: 37.7;Ovarian stromal cells: 3
	Low cell type specificity	Detected in all		
brain: 31.9;seminal vesicle: 11.4	Cell type enhanced	Detected in many		Adrenal cortex cells: 20.4;Astrocytes: 34.6;Bergmann glia: 57.5;Brain inhibitory neurons: 20.7;Neutrophils: 22.8;Oligodendrocyte progenitor cells: 144.7;Oligodendrocytes: 58.2;Peritubular myoid cells: 33.8
	Cell type enhanced	Detected in some		Adrenal medulla cells: 10.6;Early primary spermatocytes: 16.0;Ependymal cells: 10.9;Fallopian tube ciliated cells: 7.8;Late primary spermatocytes: 19.7;Other brain neurons: 9.7;Respiratory ciliated cells: 23.6
	Cell type enhanced	Detected in many		Hepatocytes: 193.6;Mast cells: 189.7;Sertoli cells: 205.2
stomach 1: 1.0;testis: 1.5	Cell type enhanced	Detected in some		Cardiomyocytes: 21.6;Foveolar cells: 20.9;Gastric progenitor cells: 10.3;Myonuclei: 9.8;Oligodendrocytes: 11.9;Retinal ganglion cells: 3.9;Sertoli cells: 17.4
skeletal muscle: 34.5;tongue: 53.5	Cell type enriched	Detected in many	4	Myonuclei: 889.9
	Cell type enhanced	Detected in many		monocytes: 320.6;Neutrophils: 726.4
blood vessel: 200.8	Cell type enhanced	Detected in many		Esophageal apical cells: 126.7;Esophageal basal cells: 100.5;Esophageal suprabasal cells: 164.7;Hepatic stellate cells: 96.8;Myosatellite cells: 145.8;Pericytes: 497.7;Suprabasal keratinocytes: 127.3;Vascular smooth muscle
lymphoid tissue: 6.7;seminal vesicle: 3.0	Cell type enriched	Detected in some	9	Breast lactating cells: 379.6
	Cell type enhanced	Detected in many		Esophageal apical cells: 86.6
cervix: 105.8	Cell type enhanced	Detected in many		Adrenal medulla cells: 74.5;Corticotrophs: 175.9;Endometrial glandular cells: 116.3;Lymphatic endothelial cells: 78.6;Oligodendrocyte progenitor cells: 89.3;Pituicytes/FSCs: 118.2;Podocytes: 70.5;Respiratory deuterosomal
brain: 9.2;kidney: 8.3;retina: 9.5	Cell type enriched	Detected in many	4	Podocytes: 3414.9
bone marrow: 23.6;brain: 19.6;retina: 19.3	Cell type enhanced	Detected in many		Astrocytes: 54.6;Bergmann glia: 113.2;Cone photoreceptor cells: 171.8;Neutrophil progenitors: 167.8;Neutrophils: 110.4;Retinal bipolar cells: 95.5;Retinal ganglion cells: 46.4;Rod photoreceptor cells: 128.8
brain: 233.3;thyroid gland: 118.0	Cell type enhanced	Detected in many		Astrocytes: 2373.3;Bergmann glia: 364.4;Brain excitatory neurons: 502.7;Brain inhibitory neurons: 585.1;Breast lactating cells: 558.5;Choroid plexus epithelial cells: 430.8;Ependymal cells: 1515.5;Oligodendrocyte progenitor
intestine: 9.8	Cell type enhanced	Detected in some		Epicardial cells: 6.8;Kupffer cells: 5.1;monocytes: 15.2;Müller glia: 8.6;Neutrophil progenitors: 5.0;Neutrophils: 33.0;Renal collecting duct principal cells: 5.3;Renal connecting tubule cells: 5.5;Retinal bipolar cells: 17.7;Rod ph
	Cell type enriched	Detected in all	4	Neutrophils: 2516.4
thyroid gland: 29.4	Cell type enhanced	Detected in many		Choroid plexus epithelial cells: 62.6;Distal convoluted tubule cells: 134.9;Loop of henle epithelial cells: 95.8;Papillary tip epithelial cells: 80.2;Proximal tubule cells: 45.1;Renal collecting duct intercalated cells: 111.2;Renal coll
	Low cell type specificity	Detected in many		
intestine: 1220.0;pancreas: 666.4	Cell type enhanced	Detected in many		Enteric stem cells: 3771.2;Enteric transient amplifying cells: 1899.8;Gastric progenitor cells: 1996.8;Neutrophil progenitors: 3094.3;Pancreatic acinar cells: 888.4;Paneth cells: 6605.5;Prostatic club cells: 5605.8;Prostatic hillo
lung: 94.3;placenta: 107.4	Group enriched	Detected in many	4	Cytotrophoblasts: 590.9;Macrophages: 318.3;Microglia: 441.5;Migrating cytotrophoblasts: 288.4;monocytes: 615.5;Neutrophil progenitors: 192.1;Neutrophils: 172.8;Syncytiotrophoblasts: 572.1
placenta: 17.6	Cell type enhanced	Detected in many		cDC: 115.7;Early spermatids: 197.8;Extravillous trophoblasts: 377.6;Late spermatids: 160.0;Migrating cytotrophoblasts: 142.3;pDCs: 172.3;Syncytiotrophoblasts: 187.2
brain: 3.8;prostate: 1.5	Cell type enhanced	Detected in some		Brain excitatory neurons: 5.3;Brain inhibitory neurons: 14.9;Endometrial glandular cells: 9.6;Endometrial luminal cells: 12.8;Extravillous trophoblasts: 13.5;Leydig cells: 5.5;Other brain neurons: 17.2;Pituicytes/FSCs: 5.1;Prosta
	Not detected	Not detected		
	Not detected	Not detected		
	Not detected	Not detected		
	Cell type enhanced	Detected in some		Adipocytes: 1.8;Early primary spermatocytes: 7.6;Early spermatids: 3.2;Fibro-adipogenic progenitors: 1.7;Kupffer cells: 2.0;Late primary spermatocytes: 8.5;Late spermatids: 6.5;Mast cells: 4.8;pDCs: 2.1;Prostatic glandular
	Not detected	Not detected		
skeletal muscle: 31.4;tongue: 19.0	Cell type enhanced	Detected in many		Astrocytes: 366.9;Brain excitatory neurons: 271.0;Brain inhibitory neurons: 348.5;Corticotrophs: 324.8;Ependymal cells: 338.1;Myonuclei: 664.7;Pituicytes/FSCs: 526.9;Respiratory ciliated cells: 382.4;Retinal ganglion cells: 3
	Cell type enhanced	Detected in many		Colonocytes: 42.6;Enterocytes: 39.3;Foveolar cells: 151.6;Goblet cells: 44.9;Mucous neck cells: 29.4
breast: 2.8	Cell type enhanced	Detected in some		Extravillous trophoblasts: 71.2;Lacrimal acinar cells: 7.2;Lymphatic endothelial cells: 6.7;Migrating cytotrophoblasts: 15.8;Paneth cells: 7.7;Renal collecting duct intercalated cells: 22.1
	Cell type enhanced	Detected in all		Alveolar cells type 2: 577.8;Neutrophils: 1361.0;Pancreatic acinar cells: 524.8
	Cell type enhanced	Detected in many		Mast cells: 612.7;Melanocytes: 842.8;Microglia: 548.9;monocytes: 466.6;Neutrophil progenitors: 1964.4;Neutrophils: 2606.1
	Cell type enhanced	Detected in all		Oocytes: 534.2
brain: 118.6	Cell type enhanced	Detected in all		Mast cells: 501.7;Neutrophils: 806.4
	Low cell type specificity	Detected in many		
brain: 292.8	Cell type enhanced	Detected in many		Astrocytes: 125.4;Bergmann glia: 64.4;Distal convoluted tubule cells: 65.0;Ependymal cells: 82.6;Fibro-adipogenic progenitors: 80.9;Gonadotrophs: 64.5;Leydig cells: 70.4;Lymphatic endothelial cells: 108.0;Müller glia: 97.6;
	Cell type enhanced	Detected in many		Adipocytes: 916.8;Alveolar cells type 1: 485.6;Ependymal cells: 455.4;Lymphatic endothelial cells: 404.0;Mast cells: 682.4;Oligodendrocytes: 987.2;Vascular endothelial cells: 648.1
	Cell type enhanced	Detected in all		Epicardial cells: 329.8;Podocytes: 637.0
	Cell type enhanced	Detected in many		Basal keratinocytes: 129.4;Platelets: 88.8;Suprabasal keratinocytes: 77.1
brain: 39.0	Cell type enhanced	Detected in many		Bergmann glia: 43.1;Brain excitatory neurons: 35.9;Brain inhibitory neurons: 27.2;Corticotrophs: 23.6;Esophageal apical cells: 23.6;Lactotrophs: 37.0;Neutrophils: 32.4;Other brain neurons: 37.0
brain: 138.7	Cell type enhanced	Detected in some		Enterocytes: 2.0;Foveolar cells: 1.6;Medullary thymic epithelial cells: 2.4;Oligodendrocytes: 1.1
	Cell type enhanced	Detected in all		Esophageal apical cells: 2031.3;Podocytes: 2848.1
	Cell type enhanced	Detected in many		Ependymal cells: 2108.7;Podocytes: 4928.4
brain: 45.1;testis: 41.3	Cell type enriched	Detected in many	9	Late spermatids: 487.2
skeletal muscle: 183.6;tongue: 159.4	Cell type enhanced	Detected in many		Erythrocyte progenitors: 307.3;Mast cells: 211.9;Megakaryocyte progenitors: 192.1;Megakaryocytes: 1193.1;Myonuclei: 227.5;pDCs: 213.6;Platelets: 751.8
	Cell type enhanced	Detected in many		Proximal tubule cells: 1347.0
	Cell type enhanced	Detected in many		Esophageal apical cells: 272.7;Foveolar cells: 65.5;Salivary duct cells: 80.5
blood vessel: 48.2	Cell type enhanced	Detected in many		Astrocytes: 554.1;Basal keratinocytes: 1177.1;Basal prostatic cells: 863.1;Brain excitatory neurons: 781.9;Brain inhibitory neurons: 732.5;Cardiomyocytes: 920.8;Choroid plexus epithelial cells: 777.1;Oligodendrocyte progeni
brain: 1.9	Cell type enhanced	Detected in some		Astrocytes: 1.3;Bergmann glia: 5.6;Brain excitatory neurons: 2.1;Brain inhibitory neurons: 2.3;Ependymal cells: 1.8;Oligodendrocyte progenitor cells: 7.4;Oligodendrocytes: 1.4;Other brain neurons: 1.1;Respiratory deuteroso
	Cell type enhanced	Detected in some		Bergmann glia: 1.1;Oligodendrocyte progenitor cells: 1.4
	Cell type enhanced	Detected in all		Esophageal basal cells: 307.3;Esophageal suprabasal cells: 310.1
	Cell type enhanced	Detected in many		cDC: 306.8;Innate lymphoid cells: 297.4;Late spermatids: 399.1;Macrophages: 130.5;Mast cells: 256.9;monocytes: 205.8;NK-cells: 237.4;pDCs: 153.3;T-cells: 187.5
bone marrow: 70.7;skeletal muscle: 63.5	Cell type enhanced	Detected in all		Myonuclei: 8670.8;Neutrophil progenitors: 5306.2;Prostatic glandular cells: 7016.6;Proximal tubule cells: 4114.6;Renal collecting duct intercalated cells: 4569.2;Renal collecting duct principal cells: 4333.3;Renal connecting t
intestine: 69.9	Cell type enhanced	Detected in many		Enterocytes: 513.9;Loop of henle epithelial cells: 197.6;Oocytes: 216.4;Prostatic glandular cells: 323.4;Renal collecting duct principal cells: 165.5;Renal connecting tubule cells: 184.7;Retinal horizontal cells: 202.3;Somatotro
parathyroid gland: 19.1	Cell type enhanced	Detected in many		Macrophages: 387.3;Medullary thymic epithelial cells: 364.8;Proximal tubule cells: 410.0;Salivary acinar cells: 480.4
ovary: 188.3	Group enriched	Detected in many	4	Decidual stromal cells: 203.7;Endometrial stromal cells: 261.4;Fibroblasts: 245.6;Hepatic stellate cells: 154.9;Leydig cells: 301.5;Oligodendrocyte progenitor cells: 296.2;Ovarian stromal cells: 108.6;Peritubular myoid cells: 3
placenta: 127.2	Cell type enhanced	Detected in many		Alveolar cells type 1: 108.9;Breast myoepithelial cells: 85.2;Decidual stromal cells: 204.4;Epicardial cells: 170.6;Epididymal basal cells: 98.5;Fibroblasts: 145.2;Lymphatic endothelial cells: 175.0;Mesothelial cells: 167.1;Müller
heart muscle: 17.6	Cell type enhanced	Detected in many		Adipocytes: 797.9;Cardiomyocytes: 875.8;Choroid plexus epithelial cells: 2846.4;Cone photoreceptor cells: 772.2;Oligodendrocyte progenitor cells: 1244.7;Proximal tubule cells: 1410.4;Rod photoreceptor cells: 745.2
placenta: 427.4	Cell type enhanced	Detected in many		Kupffer cells: 171.2;Lymphatic endothelial cells: 440.1;Macrophages: 152.3;Megakaryocyte progenitors: 170.0;Megakaryocytes: 160.7;Monocyte progenitors: 216.7;monocytes: 283.8;Neutrophils: 524.5;pDCs: 168.1;Plasma
ovary: 4.8	Cell type enhanced	Detected in many		Endometrial glandular cells: 54.5;Endometrial luminal cells: 47.0;Ependymal cells: 53.4;Granulosa cells: 43.0;Myonuclei: 69.1;Myosatellite cells: 95.6;pDCs: 334.8;Retinal horizontal cells: 72.1
	Cell type enhanced	Detected in many		Adipocytes: 463.8;Alveolar cells type 1: 397.3;Basal keratinocytes: 656.2;Distal convoluted tubule cells: 453.8;Epicardial cells: 1132.5;Ocular epithelial cells: 669.2;Salivary basal cells: 479.7;Salivary ionocytes: 693.4;Vascular
	Low cell type specificity	Detected in all		
bone marrow: 41.2;lymphoid tissue: 18.4	Cell type enhanced	Detected in some		B-cells: 66.2;Microglia: 72.4;Monocyte progenitors: 82.7;monocytes: 64.6;Neutrophil progenitors: 331.3;Neutrophils: 326.7;NK-cells: 66.8;pDCs: 112.3;Plasma cells: 146.5;Tuft cells: 564.9
	Low cell type specificity	Detected in many		
blood vessel: 106.7	Cell type enhanced	Detected in many		Mesothelial cells: 364.2;Renal collecting duct principal cells: 325.2
heart muscle: 185.4	Cell type enhanced	Detected in many		Cardiomyocytes: 661.1;Epididymal clear cells: 263.3;Extravillous trophoblasts: 408.4;Fallopian tube ciliated cells: 346.0;Lacrimal acinar cells: 489.1;Salivary acinar cells: 278.0;Salivary basal cells: 268.1;Salivary ionocytes: 6
esophagus: 283.5;skin 1: 212.8	Cell type enhanced	Detected in many		Colonocytes: 249.2;Enterocytes: 182.8;Esophageal apical cells: 1282.3;Esophageal basal cells: 314.4;Esophageal suprabasal cells: 736.9;Extravillous trophoblasts: 239.4;Migrating cytotrophoblasts: 167.2;Suprabasal keratin
brain: 336.1	Cell type enhanced	Detected in many		Distal convoluted tubule cells: 1480.6;Loop of henle epithelial cells: 1143.2;Oligodendrocytes: 1268.3
	Cell type enhanced	Detected in many		Endometrial glandular cells: 162.1;Enterocytes: 176.3;Epicardial cells: 627.7;Megakaryocyte-Erythroid progenitors: 250.9
	Low cell type specificity	Detected in many		
bone marrow: 650.6	Cell type enhanced	Detected in many		cDC: 1115.4;Endometrial glandular cells: 779.5;Endometrial secretory cells: 874.8;Epididymal basal cells: 3927.4;Macrophages: 787.2;monocytes: 2786.3;Neutrophils: 7137.8;Ocular epithelial cells: 2333.0;Urothelial cells: 77
salivary gland: 47.7	Cell type enhanced	Detected in many		Adrenal medulla cells: 659.5;Choroid plexus epithelial cells: 1249.2;Pituitary stem cells: 699.6;Retinal amacrine cells: 668.2;Retinal bipolar cells: 674.0;Retinal horizontal cells: 1170.0;Salivary acinar cells: 1050.7;Thyrotrophs:
	Cell type enhanced	Detected in many		Astrocytes: 94.6;Bergmann glia: 87.7;Cardiomyocytes: 117.3;Epicardial cells: 261.5
	Cell type enhanced	Detected in all		Choroid plexus epithelial cells: 665.4;Cone photoreceptor cells: 728.7;Ependymal cells: 619.0;Esophageal apical cells: 643.0;Proximal tubule cells: 879.2;Retinal pigment epithelial cells: 656.5
esophagus: 24.5;intestine: 37.7	Cell type enhanced	Detected in many		Colonocytes: 152.3;Enterocytes: 170.0;Epicardial cells: 129.6;Esophageal apical cells: 124.8;Extravillous trophoblasts: 122.4;Paneth cells: 69.6;Tuft cells: 92.2;Urothelial cells: 77.4
cervix: 23.9;esophagus: 58.4;skin 1: 48.4;vagina: 25.0	Cell type enhanced	Detected in many		Cardiomyocytes: 132.4;Epididymal basal cells: 49.8;Esophageal apical cells: 306.9;Esophageal basal cells: 42.0;Esophageal suprabasal cells: 144.5;Ocular epithelial cells: 46.0;Prostatic hillock cells: 46.2;Suprabasal keratino
adipose tissue: 715.6;breast: 303.4;skeletal muscle: 235.5	Cell type enriched	Detected in many	9	Adipocytes: 518.0
	Cell type enriched	Detected in many	4	Prostatic glandular cells: 7829.4
salivary gland: 103.7	Cell type enhanced	Detected in many		Breast lactating cells: 209.5;Breast secretory cells: 226.9;Cholangiocytes: 219.1;Enterocytes: 188.7;Fallopian tube ciliated cells: 299.2;Pancreatic duct cells: 187.3;Prostatic club cells: 175.2;Respiratory ciliated cells: 298.1;R
	Cell type enhanced	Detected in many		Esophageal apical cells: 626.4;monocytes: 403.3;Neutrophil progenitors: 470.1;Neutrophils: 830.3

kidney: 130.6	Cell type enriched	Detected in many	6	Podocytes: 2497.9
bone marrow: 20.8	Cell type enhanced	Detected in many		Early primary spermatocytes: 97.0;Erythrocyte progenitors: 74.0;Megakaryocyte progenitors: 60.6;Megakaryocyte-Erythroid progenitors: 57.5;Neutrophil progenitors: 125.6
	Cell type enhanced	Detected in many		Cardiomyocytes: 560.6;Lymphatic endothelial cells: 1720.5;Proximal tubule cells: 625.9
brain: 65.3	Cell type enhanced	Detected in many		Brain excitatory neurons: 68.8;Cone photoreceptor cells: 93.2;Rod photoreceptor cells: 99.8;Sertoli cells: 125.0
brain: 73.0;lymphoid tissue: 39.3	Cell type enhanced	Detected in many		B-cells: 365.8;Brain inhibitory neurons: 162.4;Innate lymphoid cells: 368.0;Lymphatic endothelial cells: 166.8;NK-cells: 516.2;Oligodendrocytes: 421.9;pDCs: 409.8;Proximal tubule cells: 305.6;Retinal horizontal cells: 296.5;T-
brain: 153.9;retina: 175.8	Cell type enhanced	Detected in many		Early spermatids: 12.0;Late primary spermatocytes: 7.2;Megakaryocyte progenitors: 8.7;Megakaryocytes: 24.7;Platelets: 18.2;Rod photoreceptor cells: 8.1
	Low cell type specificity	Detected in all		
testis: 102.1	Cell type enhanced	Detected in some		Differentiating spermatogonia: 41.1;Early primary spermatocytes: 13.8;Endometrial ciliated cells: 2.9;Late primary spermatocytes: 3.6;Late spermatids: 4.7;Oocytes: 9.2;Sertoli cells: 4.0;Undifferentiated spermatogonia: 12.3
brain: 32.9	Cell type enhanced	Detected in all		Adrenal cortex cells: 995.0;Astrocytes: 1157.1;Bergmann glia: 1773.9;Choroid plexus epithelial cells: 988.3;Oligodendrocyte progenitor cells: 1180.8;Renal connecting tubule cells: 967.4;Retinal bipolar cells: 1304.1
brain: 28.9	Cell type enhanced	Detected in many		Late spermatids: 932.2
	Cell type enhanced	Detected in many		Distal convoluted tubule cells: 710.1;Ependymal cells: 619.6;Loop of henle epithelial cells: 765.5;Podocytes: 722.7;Prostatic glandular cells: 1753.5;Sertoli cells: 1511.7;Somatotrophs: 796.9
	Cell type enhanced	Detected in all		Melanocytes: 342.2;Rod photoreceptor cells: 285.2;Somatotrophs: 250.3
brain: 31.9	Cell type enhanced	Detected in many		Endometrial ciliated cells: 45.3;Epididymal efferent duct ciliated cells: 45.7;Fallopian tube ciliated cells: 67.7;Megakaryocytes: 123.6;Platelets: 60.6;Respiratory ciliated cells: 63.9;Suprabasal keratinocytes: 46.0
brain: 305.2	Cell type enhanced	Detected in some		Astrocytes: 55.4;Bergmann glia: 63.6;Brain excitatory neurons: 57.6;Brain inhibitory neurons: 40.5;Ependymal cells: 72.2;Late spermatids: 11.9;Oligodendrocyte progenitor cells: 16.4;Other brain neurons: 28.7
stomach 1: 3010.1	Group enriched	Detected in many	5	Esophageal apical cells: 9592.0;Foveolar cells: 7558.7;Parietal cells: 10610.9;Prostatic club cells: 3521.6
	Cell type enhanced	Detected in many		Myonuclei: 50.7
	Cell type enhanced	Detected in all		Early spermatids: 578.0;Late primary spermatocytes: 496.8;Late spermatids: 2133.5;Oocytes: 455.9;Syncytiotrophoblasts: 459.9
bone marrow: 55.1;lymphoid tissue: 30.4	Cell type enhanced	Detected in many		cDC: 51.5;Hofbauer cells: 65.6;Monocyte progenitors: 70.1;monocytes: 77.1;Neutrophil progenitors: 128.8;Neutrophils: 130.1;NK-cells: 59.0;Oocytes: 520.4;T-cells: 62.2
bone marrow: 38.8;lymphoid tissue: 29.0	Cell type enhanced	Detected in many		cDC: 80.2;Hofbauer cells: 113.5;Kupffer cells: 146.6;Macrophages: 83.7;Mast cells: 71.9;Microglia: 114.8;Monocyte progenitors: 64.9;monocytes: 144.8;Neutrophil progenitors: 117.1;Neutrophils: 466.0;Platelets: 94.8
parathyroid gland: 55.6;salivary gland: 70.9	Group enriched	Detected in many	5	Leydig cells: 1885.2;Peritubular myoid cells: 756.4
kidney: 583.6	Cell type enhanced	Detected in many		Distal convoluted tubule cells: 339.1;Extravillous trophoblasts: 123.9;Hepatic stellate cells: 142.0;Loop of henle epithelial cells: 236.9;Pericytes: 138.9;Podocytes: 1120.4;Proximal tubule cells: 1001.2;Renal connecting tubule
bone marrow: 100.4;brain: 136.7	Cell type enhanced	Detected in many		Monocyte progenitors: 405.5;monocytes: 590.0;Neutrophil progenitors: 771.7;Neutrophils: 1387.8
	Cell type enhanced	Detected in many		Cone photoreceptor cells: 184.2;Early spermatids: 424.1;Endometrial stromal cells: 165.1;Retinal ganglion cells: 139.1
gallbladder: 22.8;intestine: 64.6;stomach 1: 22.3	Cell type enhanced	Detected in many		Adrenal cortex cells: 28.2;Cholangiocytes: 28.0;Colonocytes: 101.1;Enteric transient amplifying cells: 32.8;Enterocytes: 274.3;Foveolar cells: 78.5;Goblet cells: 38.1;Pancreatic acinar cells: 47.8;Pancreatic duct cells: 29.8;Par
	Cell type enhanced	Detected in many		Microglia: 975.6;Neutrophil progenitors: 1712.9;Neutrophils: 3323.0;Proximal tubule cells: 833.2;Renal collecting duct intercalated cells: 1339.7;Tuft cells: 1095.2
	Cell type enhanced	Detected in many		Astrocytes: 229.4;Brain excitatory neurons: 404.3;Brain inhibitory neurons: 254.2;Choroid plexus epithelial cells: 333.4;Distal convoluted tubule cells: 314.4;Ependymal cells: 554.5;Loop of henle epithelial cells: 371.3;Oligoder
	Cell type enhanced	Detected in many		Bergmann glia: 1139.2;Choroid plexus epithelial cells: 1448.8;Endometrial glandular cells: 1120.2;Endometrial luminal cells: 1179.2;Melanocytes: 1194.2;Urothelial cells: 1106.6;Vascular endothelial cells: 1535.2
brain: 12.1;endometrium 1: 5.2;intestine: 7.2	Cell type enhanced	Detected in many		Adrenal medulla cells: 159.6;Brain excitatory neurons: 377.5;Brain inhibitory neurons: 212.4;Colonocytes: 137.3;Enterocytes: 122.3;Foveolar cells: 163.5;Other brain neurons: 154.8;Retinal amacrine cells: 249.4;Retinal bipolar
	Cell type enhanced	Detected in many		Adipocytes: 476.7;Hepatic stellate cells: 296.9;pDCs: 589.9;Retinal amacrine cells: 356.9
	Cell type enhanced	Detected in many		Cardiomyocytes: 67.5;Epicardial cells: 29.6;Podocytes: 48.0;Salivary myoepithelial cells: 36.8;Tuft cells: 34.6
brain: 121.9	Cell type enhanced	Detected in many		Neutrophil progenitors: 256.0;Neutrophils: 568.8;Oligodendrocytes: 824.6;Renal collecting duct intercalated cells: 353.1
	Cell type enhanced	Detected in many		Epididymal principal cells: 10.1;Gastric chief cells: 10.9;Loop of henle epithelial cells: 16.5;Papillary tip epithelial cells: 25.7;Renal collecting duct intercalated cells: 10.2;Renal collecting duct principal cells: 24.5;Renal connect
	Cell type enhanced	Detected in many		Cardiomyocytes: 42.9;Retinal pigment epithelial cells: 42.6;Smooth muscle cells: 106.4
esophagus: 407.1;skin 1: 203.2	Group enriched	Detected in many	7	Esophageal apical cells: 3926.5;Esophageal suprabasal cells: 1503.5
	Cell type enhanced	Detected in many		Megakaryocyte progenitors: 189.7;Megakaryocytes: 364.7;Platelets: 660.9
adrenal gland: 7.5;esophagus: 13.8	Cell type enhanced	Detected in some		Esophageal apical cells: 74.4;Esophageal basal cells: 15.0;Esophageal suprabasal cells: 78.0;Mesothelial cells: 16.7;Ocular epithelial cells: 28.9;Suprabasal keratinocytes: 21.7
cervix: 11.9;esophagus: 45.6;vagina: 13.4	Cell type enriched	Detected in some	6	Esophageal apical cells: 594.2
skeletal muscle: 163.1	Cell type enhanced	Detected in all		Neutrophils: 859.1
	Cell type enhanced	Detected in all		Endometrial glandular cells: 461.5;Endometrial secretory cells: 445.0;Esophageal apical cells: 1857.8;Esophageal suprabasal cells: 532.7
	Cell type enhanced	Detected in all		Esophageal apical cells: 513.9;Esophageal suprabasal cells: 395.9;Extravillous trophoblasts: 783.6;Renal collecting duct principal cells: 465.3;Urothelial cells: 385.4
	Cell type enhanced	Detected in some		Decidual stromal cells: 29.9;Hepatic stellate cells: 51.6;Lymphatic endothelial cells: 184.9;Podocytes: 87.0;Vascular endothelial cells: 409.5
	Cell type enhanced	Detected in many		Breast lactating cells: 943.8;Neutrophils: 953.7
liver: 57.5	Cell type enhanced	Detected in many		Cardiomyocytes: 391.9;Epicardial cells: 384.3
bone marrow: 22.2;intestine: 19.0;lung: 10.6;lymphoid tissue: 39.2	Cell type enhanced	Detected in some		B-cells: 45.9;Hematopoietic stem cells: 25.2;Innate lymphoid cells: 54.8;Mast cells: 29.2;Microglia: 60.6;Neutrophil progenitors: 64.6;Neutrophils: 42.1;NK-cells: 98.1;Plasma cells: 23.9;T-cells: 92.5;Thymocytes: 33.9
brain: 23.3;lymphoid tissue: 35.6;retina: 16.6	Cell type enhanced	Detected in many		Brain excitatory neurons: 100.9;Esophageal apical cells: 92.9;Müller glia: 262.2;NK-cells: 153.8;Renal collecting duct principal cells: 112.0;Renal connecting tubule cells: 95.6;T-cells: 204.6
bone marrow: 139.0;lymphoid tissue: 137.9	Group enriched	Detected in many	6	Megakaryocytes: 636.7;Platelets: 1342.3
bone marrow: 19.6;lymphoid tissue: 9.4	Cell type enriched	Detected in some	4	Neutrophils: 218.8
	Cell type enhanced	Detected in all		Neutrophils: 827.8
	Cell type enhanced	Detected in many		Oocytes: 96.7;Retinal pigment epithelial cells: 42.6
adrenal gland: 146.4	Cell type enhanced	Detected in many		Adrenal cortex cells: 1292.9
	Cell type enhanced	Detected in many		Breast lactating cells: 123.0;Early primary spermatocytes: 137.5;Fibro-adipogenic progenitors: 155.4;Microglia: 153.0;Oligodendrocytes: 137.2
brain: 33.6	Cell type enhanced	Detected in all		Neutrophil progenitors: 477.4;Neutrophils: 525.7;Parietal cells: 449.0;pDCs: 218.1
	Cell type enhanced	Detected in all		Bergmann glia: 402.6;Lactotrophs: 451.6;Sertoli cells: 444.5
adrenal gland: 10.9;parathyroid gland: 18.7	Cell type enhanced	Detected in many		Adrenal medulla cells: 234.0;Breast lactating cells: 62.8;Corticotrophs: 36.2;Fibro-adipogenic progenitors: 52.0;Myosatellite cells: 24.1;Respiratory ionocytes: 86.4;Retinal horizontal cells: 86.1
bone marrow: 54.1;lymphoid tissue: 73.8	Cell type enhanced	Detected in many		B-cells: 83.6;cDC: 87.3;Hofbauer cells: 160.5;Kupffer cells: 123.0;Macrophages: 81.3;Megakaryocytes: 111.3;Microglia: 63.1;Monocyte progenitors: 80.8;monocytes: 110.1;Neutrophil progenitors: 128.7;Neutrophils: 273.7;pD
choroid plexus: 142.2	Cell type enhanced	Detected in many		Cholangiocytes: 101.8;Distal convoluted tubule cells: 116.4;Megakaryocytes: 98.9;Vascular endothelial cells: 107.2
cervix: 22.9	Cell type enhanced	Detected in many		Endometrial ciliated cells: 393.8;Endometrial glandular cells: 1237.2;Endometrial luminal cells: 1014.0;Endometrial secretory cells: 295.3;Loop of henle epithelial cells: 312.3;Mast cells: 699.4;Papillary tip epithelial cells: 637.1
	Cell type enhanced	Detected in many		Cardiomyocytes: 98.6;Choroid plexus epithelial cells: 53.5;Distal convoluted tubule cells: 66.0;Ependymal cells: 58.8;Loop of henle epithelial cells: 78.0;Microglia: 107.5;Oligodendrocytes: 77.0;Other brain neurons: 52.2;Papil
	Cell type enhanced	Detected in some		Astrocytes: 37.2;Bergmann glia: 34.6;Brain excitatory neurons: 21.1;Brain inhibitory neurons: 19.0;Choroid plexus epithelial cells: 35.6;Distal convoluted tubule cells: 24.7;Ependymal cells: 32.4;Loop of henle epithelial cells: 3
brain: 48.8;liver: 119.8	Cell type enhanced	Detected in many		Alveolar cells type 2: 72.2;Breast hormone-responsive cells: 74.8;Epididymal clear cells: 49.0;Epididymal efferent duct absorptive cells: 127.4;Pancreatic acinar cells: 45.3;Salivary acinar cells: 87.9;Transitional alveolar cells: 5
	Cell type enhanced	Detected in all		Myonuclei: 168.4;Neutrophils: 173.1
	Cell type enhanced	Detected in many		Adrenal medulla cells: 1648.7;Brain excitatory neurons: 1017.0;Brain inhibitory neurons: 2256.4;Lactotrophs: 5760.6;Other brain neurons: 1644.2;Prostatic club cells: 945.3;Thyrotrophs: 1197.6
bone marrow: 46.7	Cell type enhanced	Detected in all		Kupffer cells: 109.7;Monocyte progenitors: 164.7;monocytes: 175.1;Neutrophil progenitors: 261.9;Neutrophils: 524.8
	Cell type enhanced	Detected in all		Esophageal basal cells: 4756.0;Esophageal suprabasal cells: 4637.5;Extravillous trophoblasts: 4805.1;Migrating cytotrophoblasts: 4135.2
bone marrow: 85.9;skeletal muscle: 36.0	Cell type enhanced	Detected in many		Kupffer cells: 222.4;Monocyte progenitors: 319.4;monocytes: 176.4;Neutrophil progenitors: 288.4;Neutrophils: 284.8
	Cell type enhanced	Detected in some		Choroid plexus epithelial cells: 1.5;Microglia: 1.3
brain: 82.6	Cell type enhanced	Detected in many		Bergmann glia: 9.9;Brain excitatory neurons: 26.2;Extravillous trophoblasts: 13.0;Müller glia: 11.3;Respiratory deuterosomal cells: 11.4;Retinal bipolar cells: 10.4
brain: 12.8	Group enriched	Detected in many	5	Early spermatids: 432.8;Late spermatids: 1254.8
skin 1: 98.8	Not detected	Not detected		
	Cell type enhanced	Detected in many		Alveolar cells type 1: 9120.5;Colonocytes: 21095.2;Decidual stromal cells: 9939.1;Endometrial secretory cells: 9533.5;Esophageal apical cells: 13496.6;Gastric progenitor cells: 11251.8;Goblet cells: 11783.0;Ocular epithelial c
bone marrow: 30401.1;cervix: 13503.3;esophagus: 30106.4;vagina	Cell type enriched	Detected in many	4	Esophageal apical cells: 540718.9
esophagus: 350.3;skin 1: 285.8	Cell type enriched	Detected in many	8	Esophageal apical cells: 7219.7
brain: 7.9;retina: 3.1	Cell type enhanced	Detected in many		Alveolar cells type 2: 178.2;Brain excitatory neurons: 336.0;Brain inhibitory neurons: 430.0;Ependymal cells: 573.7;Fallopian tube ciliated cells: 190.3;Oligodendrocyte progenitor cells: 835.0;Other brain neurons: 230.2;Pituicy
brain: 73.3;pituitary gland: 26.6	Cell type enhanced	Detected in many		Adrenal medulla cells: 74.5;Brain excitatory neurons: 100.9;Brain inhibitory neurons: 80.6;Corticotrophs: 40.7;Lactotrophs: 178.4;Late spermatids: 50.9;Lymphatic endothelial cells: 175.2;Other brain neurons: 96.6;Retinal ama
heart muscle: 49.7	Cell type enhanced	Detected in many		Cardiomyocytes: 229.0;Cone photoreceptor cells: 51.8;Myosatellite cells: 20.0;Neutrophil progenitors: 68.2;Neutrophils: 41.9;Oocytes: 27.6;Pituicytes/FSCs: 21.7;Retinal amacrine cells: 19.9
brain: 16.9;pituitary gland: 6.3;retina: 7.3	Cell type enhanced	Detected in many		Bergmann glia: 152.4;Brain excitatory neurons: 367.3;Brain inhibitory neurons: 321.4;Corticotrophs: 194.7;Gonadotrophs: 255.9;Lactotrophs: 245.8;Oligodendrocyte progenitor cells: 137.2;Other brain neurons: 185.7;Retinal a
brain: 35.6;testis: 34.7	Cell type enhanced	Detected in many		Astrocytes: 13.1;Early spermatids: 16.9;Ependymal cells: 9.9;Esophageal apical cells: 13.0;Esophageal suprabasal cells: 12.6;Oligodendrocyte progenitor cells: 11.6;Proximal tubule cells: 13.8;Retinal ganglion cells: 11.6
kidney: 39.6	Cell type enhanced	Detected in many		Basal prostatic cells: 103.4;Conjunctival goblet cells: 78.6;Esophageal apical cells: 184.0;Renal collecting duct principal cells: 252.9;Renal connecting tubule cells: 305.3;Respiratory ionocytes: 142.2;Respiratory secretory cel
	Cell type enhanced	Detected in many		Astrocytes: 38.8;Bergmann glia: 16.6;Distal convoluted tubule cells: 16.5;Ependymal cells: 21.4;Loop of henle epithelial cells: 13.7;Oligodendrocyte progenitor cells: 16.9;Oligodendrocytes: 20.7;Papillary tip epithelial cells: 18
blood vessel: 42.6;thyroid gland: 25.9	Cell type enriched	Detected in many	6	Epicardial cells: 577.6
liver: 430.0;thyroid gland: 397.8	Cell type enhanced	Detected in many		Breast lactating cells: 359.1;Decidual stromal cells: 309.6;Fibro-adipogenic progenitors: 306.5;Gonadotrophs: 295.3;Hepatocytes: 327.5;Pituicytes/FSCs: 282.3;Pituitary stem cells: 272.7
	Cell type enhanced	Detected in many		Late primary spermatocytes: 82.9
brain: 325.7	Cell type enhanced	Detected in many		Esophageal apical cells: 269.9;Microglia: 235.2;Neutrophils: 756.5;Oligodendrocytes: 483.4;Platelets: 219.5;T-cells: 239.3
	Cell type enhanced	Detected in many		Endometrial glandular cells: 940.8;Prostatic glandular cells: 937.0;Sertoli cells: 709.6;Urothelial cells: 1070.6
brain: 60.6	Cell type enhanced	Detected in many		Adrenal medulla cells: 42.5;Brain excitatory neurons: 40.3;Brain inhibitory neurons: 32.8;Epididymal efferent duct absorptive cells: 53.4;Innate lymphoid cells: 34.1;Lactotrophs: 44.4;Other brain neurons: 31.7;Retinal amacrine
lymphoid tissue: 45.5	Cell type enhanced	Detected in some		B-cells: 21.8;cDC: 27.4;Kupffer cells: 139.4;Macrophages: 67.4;Mast cells: 15.7;Microglia: 54.9;Monocyte progenitors: 18.6;monocytes: 76.1;Neutrophils: 63.8
bone marrow: 22.0;lymphoid tissue: 21.0	Cell type enhanced	Detected in some		cDC: 1.1;Kupffer cells: 4.1;Macrophages: 1.2;Monocyte progenitors: 1.0;monocytes: 1.8;Neutrophil progenitors: 1.0;Neutrophils: 8.9
	Cell type enhanced	Detected in many		monocytes: 2647.2;Neutrophils: 3404.9;Pituicytes/FSCs: 1745.7
	Cell type enhanced	Detected in many		Retinal amacrine cells: 1038.7
kidney: 379.8	Cell type enriched	Detected in many	4	Proximal tubule cells: 1346.5
liver: 393.9	Cell type enhanced	Detected in some		Astrocytes: 42.5;Bergmann glia: 29.1;Hepatocytes: 440.0;Salivary acinar cells: 242.2;Salivary myoepithelial cells: 97.6
blood vessel: 46.0;brain: 51.7;kidney: 46.4;prostate: 73.4;urinary bl	Cell type enhanced	Detected in many		Astrocytes: 477.7;Basal prostatic cells: 150.3;Ependymal cells: 100.6;Lacrimal acinar cells: 106.0;Medullary thymic epithelial cells: 285.6;Megakaryocyte-Erythroid progenitors: 128.9;Papillary tip epithelial cells: 168.2;Prostati
	Cell type enhanced	Detected in many		Choroid plexus epithelial cells: 54.6;Early spermatids: 46.3;Ependymal cells: 50.0;Late spermatids: 57.6;Müller glia: 56.0;Oligodendrocytes: 38.1;Proximal tubule cells: 30.4;Retinal pigment epithelial cells: 38.4;Rod photorecep
choroid plexus: 24.8;pancreas: 28.0;skeletal muscle: 33.7	Cell type enhanced	Detected in many		Choroid plexus epithelial cells: 986.0;Macrophages: 529.7;Melanocytes: 400.0;monocytes: 548.4;Myonuclei: 1010.3;Pancreatic acinar cells: 217.6;Podocytes: 374.8
skeletal muscle: 134.4	Cell type enhanced	Detected in many		Epicardial cells: 379.8;Esophageal apical cells: 585.4;Extravillous trophoblasts: 2420.5;Migrating cytotrophoblasts: 355.8;Neutrophils: 849.6;Retinal pigment epithelial cells: 660.8;Syncytiotrophoblasts: 884.7
adipose tissue: 18.8;heart muscle: 32.2	Cell type enhanced	Detected in many		Adipocytes: 749.1;Cardiomyocytes: 506.0;Distal convoluted tubule cells: 302.0;Parietal cells: 401.3
parathyroid gland: 292.0	Cell type enhanced	Detected in many		Retinal ganglion cells: 181.6;Rod photoreceptor cells: 181.4
	Cell type enhanced	Detected in many		Basal prostatic cells: 157.5;Extravillous trophoblasts: 177.6;Prostatic hillock cells: 188.0
brain: 105.2	Cell type enhanced	Detected in some		Brain excitatory neurons: 18.2;Brain inhibitory neurons: 20.3;Early spermatids: 52.6;Extravillous trophoblasts: 17.4;Late primary spermatocytes: 48.4;Late spermatids: 115.6;Other brain neurons: 18.9;Retinal horizontal cells: 2
	Cell type enhanced	Detected in many		Colonocytes: 373.5;Endometrial glandular cells: 368.9;Epicardial cells: 434.4;Ocular epithelial cells: 948.8;Prostatic hillock cells: 417.1;Urothelial cells: 797.5
brain: 25.6;retina: 15.2	Cell type enhanced	Detected in many		Adrenal medulla cells: 797.8;Brain excitatory neurons: 343.7;Brain inhibitory neurons: 567.3;Cone photoreceptor cells: 2155.0;Gonadotrophs: 481.2;Oligodendrocytes: 3412.9;Retinal amacrine cells: 396.9;Retinal bipolar cells
	Cell type enhanced	Detected in many		Colonocytes: 59.1;Enterocytes: 41.5;Goblet cells: 41.4;Mucous neck cells: 34.3;Proximal tubule cells: 51.6
intestine: 46.2;kidney: 46.6;liver: 106.7	Group enriched	Detected in many	6	Enterocytes: 248.3;Hepatocytes: 149.8;Hofbauer cells: 123.5;Proximal tubule cells: 406.1
placenta: 92.7	Cell type enhanced	Detected in many		Cytotrophoblasts: 987.3;Esophageal apical cells: 672.8;Extravillous trophoblasts: 730.6;Migrating cytotrophoblasts: 755.2;Müller glia: 484.8;Ocular epithelial cells: 855.1;Prostatic hillock cells: 375.5;Retinal pigment epithelial
choroid plexus: 102.3	Group enriched	Detected in many	6	Choroid plexus epithelial cells: 992.6;Retinal pigment epithelial cells: 601.1
testis: 76.4	Cell type enhanced	Detected in many		Adrenal cortex cells: 89.3;Early primary spermatocytes: 64.0;Erythrocyte progenitors: 45.7;Granulosa cells: 61.6;Late primary spermatocytes: 103.0;Megakaryocyte-Erythroid progenitors: 23.4;Neutrophils: 38.6;Pancreatic aci
liver: 781.9	Cell type enhanced	Detected in some		Cholangiocytes: 141.1;Enterocytes: 226.3;Hepatocytes: 749.7;Proximal tubule cells: 56.5
bone marrow: 404.4	Cell type enhanced	Detected in many		cDC: 488.0;Decidual stromal cells: 608.8;Extravillous trophoblasts: 653.6;Mast cells: 611.2;Migrating cytotrophoblasts: 434.8;monocytes: 1083.6;Neutrophil progenitors: 528.2;Neutrophils: 2917.4;Pericytes: 419.9;Platelets: 6
bone marrow: 91.5;intestine: 102.6;skeletal muscle: 73.9;testis: 71.	Cell type enhanced	Detected in many		Early primary spermatocytes: 32.5;Early spermatids: 51.2;Enterocytes: 211.9;Hematopoietic stem cells: 48.5;Late primary spermatocytes: 158.2;Microglia: 179.4;Myonuclei: 35.3;Neutrophil progenitors: 80.2;Plasma cells: 38.
liver: 80.5	Cell type enhanced	Detected in many		Endometrial luminal cells: 77.1;Epididymal efferent duct absorptive cells: 76.9;Epididymal efferent duct ciliated cells: 98.2;Hepatocytes: 105.6
pancreas: 49.4	Cell type enriched	Detected in many	5	Pancreatic islet cells: 664.9
kidney: 145.2	Cell type enriched	Detected in some	25	Proximal tubule cells: 181.7
lung: 709.6	Cell type enhanced	Detected in many		Alveolar cells type 1: 867.2;Alveolar cells type 2: 1909.3;Breast lactating cells: 291.0;Endometrial glandular cells: 419.7;Endometrial secretory cells: 444.3;Respiratory ciliated cells: 218.0;Transitional alveolar cells: 1120.7
	Cell type enhanced	Detected in many		Adipocytes: 99.3;Astrocytes: 87.7;Early primary spermatocytes: 72.0;Gonadotrophs: 81.1;Lactotrophs: 98.1;Late primary spermatocytes: 89.5;Microglia: 70.6;Other brain neurons: 75.0;Pituicytes/FSCs: 71.5;Sertoli cells: 107.
	Cell type enhanced	Detected in all		Basal keratinocytes: 1107.2;Suprabasal keratinocytes: 912.3
	Cell type enhanced	Detected in many		Breast hormone-responsive cells: 533.6;Prostatic club cells: 181.0;Prostatic glandular cells: 585.7
	Cell type enhanced	Detected in many		Breast lactating cells: 400.9;Retinal pigment epithelial cells: 443.3;Syncytiotrophoblasts: 1124.0
	Group enriched	Detected in many	4	Kupffer cells: 3944.9;Platelets: 987.0;Syncytiotrophoblasts: 3638.1
	Low cell type specificity	Detected in many		
intestine: 88.2	Cell type enhanced	Detected in many		Epicardial cells: 81.9;Hepatocytes: 37.2;Hofbauer cells: 45.7;Kupffer cells: 60.5;Late spermatids: 35.4
choroid plexus: 264.8	Group enriched	Detected in many	4	Choroid plexus epithelial cells: 329.3;Parietal cells: 169.7;Retinal pigment epithelial cells: 332.5;Syncytiotrophoblasts: 127.0
kidney: 111.6;pancreas: 245.3	Cell type enhanced	Detected in many		Astrocytes: 1379.0;Bergmann glia: 4233.7;Colonocytes: 579.5;Pancreatic duct cells: 3794.9;Pituicytes/FSCs: 1072.2;Prostatic glandular cells: 666.1;Proximal tubule cells: 967.8;Retinal ganglion cells: 691.4
intestine: 53.6;retina: 67.5	Cell type enhanced	Detected in all		Cone photoreceptor cells: 892.2;Loop of henle epithelial cells: 613.9;Microglia: 618.3;Rod photoreceptor cells: 995.3
intestine: 497.5	Cell type enriched	Detected in many	8	Enterocytes: 1403.9
epididymis: 45.1;intestine: 28.2;kidney: 160.2	Cell type enhanced	Detected in some		Enterocytes: 377.7;Epididymal principal cells: 223.1;Proximal tubule cells: 1239.1
kidney: 27.1	Cell type enhanced	Detected in many		Endometrial stromal cells: 4.8;Epididymal efferent duct absorptive cells: 4.4;Pituicytes/FSCs: 5.2
liver: 165.3;parathyroid gland: 210.8	Cell type enhanced	Detected in many		Endometrial glandular cells: 574.1;Endometrial luminal cells: 257.0;Epicardial cells: 482.1;Fallopian secretory cells: 278.6;Fallopian tube ciliated cells: 329.1;Hepatocytes: 379.7;Myonuclei: 662.0;Pancreatic acinar cells: 609.1;
bone marrow: 104.4;esophagus: 96.2	Cell type enhanced	Detected in many		Basal keratinocytes: 201.4;monocytes: 174.9;Neutrophils: 266.6;pDCs: 415.3;Plasma cells: 292.2;Retinal pigment epithelial cells: 400.7;Syncytiotrophoblasts: 203.2
skeletal muscle: 67.0	Cell type enhanced	Detected in many		Adipocytes: 306.6;Choroid plexus epithelial cells: 224.3;Myonuclei: 318.2;Syncytiotrophoblasts: 186.6;Thymic myoid cells: 738.3

heart muscle: 144.0	Group enriched	Detected in many	4	Cardiomyocytes: 14581.0;Neutrophils: 8664.3;Renal connecting tubule cells: 12731.4
salivary gland: 72.1;stomach 1: 62.3	Cell type enhanced	Detected in many		Foveolar cells: 376.8;Neutrophils: 312.5
intestine: 19.4;stomach 1: 24.9	Cell type enhanced	Detected in many		Colonocytes: 152.7;Enteric stem cells: 77.6;Enteric transient amplifying cells: 84.3;Foveolar cells: 150.5;Gastric chief cells: 120.1;Goblet cells: 72.8;Mucous neck cells: 191.7;Papillary tip epithelial cells: 67.3;Parietal cells: 76.
gallbladder: 42.6;intestine: 88.4;kidney: 64.4;stomach 1: 47.2	Group enriched	Detected in some	4	Epicardial cells: 50.0;Loop of henle epithelial cells: 135.2;Proximal tubule cells: 67.4
heart muscle: 571.9	Cell type enhanced	Detected in many		Breast lactating cells: 199.5;Hepatocytes: 250.6;Lymphatic endothelial cells: 301.0;Respiratory secretory cells: 221.7;Vascular endothelial cells: 486.7
brain: 36.5	Cell type enriched	Detected in many	5	Pituicytes/FSCs: 306.8
testis: 5.6	Cell type enhanced	Detected in many		Choroid plexus epithelial cells: 77.9;Cone photoreceptor cells: 95.2;Endometrial ciliated cells: 35.2;Ependymal cells: 326.4;Fallopian tube ciliated cells: 44.9;Respiratory ciliated cells: 121.6;Sertoli cells: 121.1
brain: 15.0;choroid plexus: 7.0;liver: 7.5;salivary gland: 14.6	Group enriched	Detected in some	8	Oligodendrocytes: 530.1;Retinal pigment epithelial cells: 225.9
liver: 502.3	Cell type enriched	Detected in some	25	Hepatocytes: 247.1
liver: 247.7	Cell type enriched	Detected in some	6	Hepatocytes: 66.3
lung: 56.0	Cell type enhanced	Detected in many		Endometrial glandular cells: 87.3;Esophageal apical cells: 157.7;Extravillous trophoblasts: 362.9;Melanocytes: 102.7;Rod photoreceptor cells: 54.6;Vascular endothelial cells: 83.4
lymphoid tissue: 6.1;skeletal muscle: 7.1;tongue: 10.4	Cell type enhanced	Detected in many		Cardiomyocytes: 133.8;cDC: 78.6;Corticotrophs: 271.0;Gonadotrophs: 295.1;Myonuclei: 603.7;Prostatic glandular cells: 166.2;Retinal amacrine cells: 108.8;Thyrotrophs: 94.9
	Cell type enhanced	Detected in many		Adipocytes: 586.7;Adrenal medulla cells: 712.7;Brain excitatory neurons: 566.7;Endometrial stromal cells: 339.7;Epicardial cells: 620.8;Fibro-adipogenic progenitors: 701.5;Fibroblasts: 381.8;Medullary thymic epithelial cells: 3
salivary gland: 26.2;urinary bladder: 13.8	Cell type enhanced	Detected in some		Basal prostatic cells: 37.0;Conjunctival goblet cells: 62.1;Lacrimal acinar cells: 39.9;Medullary thymic epithelial cells: 28.8;Prostatic club cells: 38.5;Prostatic hillock cells: 82.9;Respiratory basal cells: 95.2;Respiratory ciliated c
esophagus: 136.4;placenta: 96.2	Cell type enhanced	Detected in many		Cytotrophoblasts: 1624.2;Esophageal apical cells: 1505.9;Esophageal suprabasal cells: 403.7;Migrating cytotrophoblasts: 561.4;Syncytiotrophoblasts: 901.2
	Cell type enhanced	Detected in all		Late spermatids: 400.6;Megakaryocytes: 500.7;Platelets: 1140.4
	Cell type enhanced	Detected in many		Choroid plexus epithelial cells: 208.0;Müller glia: 271.6;Rod photoreceptor cells: 177.2;Smooth muscle cells: 161.0
	Cell type enhanced	Detected in many		Adipocytes: 156.8;Choroid plexus epithelial cells: 323.0;Corticotrophs: 614.1;Gonadotrophs: 267.0;Lymphatic endothelial cells: 386.9;Other brain neurons: 180.0;Retinal ganglion cells: 204.7;Retinal horizontal cells: 1238.1;Sc
	Cell type enhanced	Detected in many		Endometrial glandular cells: 2921.6;Endometrial luminal cells: 2424.3;Esophageal apical cells: 1151.8
blood vessel: 482.7	Cell type enhanced	Detected in many		Adipocytes: 1968.4;Astrocytes: 1464.9;Bergmann glia: 1572.2;Cardiomyocytes: 2244.5;Ependymal cells: 1443.6;Hepatic stellate cells: 884.3;Myonuclei: 1611.1;Salivary myoepithelial cells: 1156.8;Smooth muscle cells: 2332.
	Cell type enhanced	Detected in many		Esophageal apical cells: 446.5;Neutrophil progenitors: 440.2;Neutrophils: 408.7;Oligodendrocytes: 612.1;Podocytes: 446.5;Thymic myoid cells: 407.9;Vascular smooth muscle cells: 375.1
	Cell type enhanced	Detected in all		Neutrophils: 516.9
	Cell type enhanced	Detected in many		Breast myoepithelial cells: 51.7;Breast secretory cells: 104.4;Cholangiocytes: 60.9;Decidual stromal cells: 86.4;Endometrial glandular cells: 77.3;Epididymal basal cells: 61.7;Esophageal apical cells: 194.5;Lymphatic endothel
esophagus: 223.2	Cell type enhanced	Detected in many		Cytotrophoblasts: 1470.5;Enterocytes: 488.4;Esophageal apical cells: 2822.0;Migrating cytotrophoblasts: 977.6;Syncytiotrophoblasts: 2062.9
	Cell type enhanced	Detected in many		Alveolar cells type 2: 395.1;Late spermatids: 491.0;Renal connecting tubule cells: 616.6
	Cell type enhanced	Detected in many		Late spermatids: 168.4
	Low cell type specificity	Detected in all		
	Cell type enhanced	Detected in many		Esophageal apical cells: 82.5;Foveolar cells: 96.8;Megakaryocytes: 173.1;Platelets: 119.8;Urothelial cells: 121.7
adrenal gland: 2.1;blood vessel: 2.6;heart muscle: 2.4;kidney: 3.9;p	Group enriched	Detected in some	4	Neuroendocrine cells: 15.8;Retinal horizontal cells: 48.1
	Cell type enhanced	Detected in all		Esophageal apical cells: 228.8;Late spermatids: 211.9
adipose tissue: 106.6	Cell type enhanced	Detected in many		Alveolar cells type 2: 288.2;Conjunctival goblet cells: 310.7;Neutrophils: 826.0;Pancreatic acinar cells: 270.3;Pericytes: 1115.7;Respiratory secretory cells: 272.4;Transitional alveolar cells: 282.5
bone marrow: 72.3	Cell type enhanced	Detected in many		cDC: 237.9;Kupffer cells: 494.0;Macrophages: 264.6;monocytes: 649.2;Neutrophil progenitors: 459.1;Neutrophils: 1534.1;NK-cells: 398.4;Sertoli cells: 269.6;T-cells: 277.0
	Cell type enhanced	Detected in many		Alveolar cells type 1: 458.5;Kupffer cells: 540.1;Neutrophil progenitors: 1252.5;Neutrophils: 456.6;Platelets: 1321.0;Smooth muscle cells: 481.7;Vascular endothelial cells: 572.5
	Cell type enhanced	Detected in many		Cytotrophoblasts: 276.0;Esophageal basal cells: 374.6;Esophageal suprabasal cells: 308.1;Extravillous trophoblasts: 276.6;Gastric progenitor cells: 297.3;Migrating cytotrophoblasts: 301.4;Oocytes: 331.6
fallopian tube: 32.8	Group enriched	Detected in some	7	Endometrial ciliated cells: 100.0;Ependymal cells: 79.8;Epididymal efferent duct ciliated cells: 110.0;Fallopian tube ciliated cells: 196.7;Respiratory ciliated cells: 153.8
cervix: 112.8	Cell type enhanced	Detected in all		Endometrial ciliated cells: 392.8;Endometrial glandular cells: 452.9;Endometrial luminal cells: 463.4
skeletal muscle: 750.5;tongue: 518.7	Cell type enhanced	Detected in many		Myonuclei: 4264.9;Neutrophil progenitors: 1240.2;Neutrophils: 2215.9;Salivary myoepithelial cells: 1298.6;Smooth muscle cells: 2168.5;Thymic myoid cells: 1389.5
	Cell type enhanced	Detected in many		B-cells: 423.7;Extravillous trophoblasts: 274.8;Vascular endothelial cells: 322.2
	Cell type enhanced	Detected in many		Late spermatids: 227.4
intestine: 68.5;stomach 1: 118.9	Cell type enhanced	Detected in many		Foveolar cells: 1989.1;Goblet cells: 942.2;Papillary tip epithelial cells: 427.9;Prostatic hillock cells: 353.6;Renal connecting tubule cells: 351.4;Salivary duct cells: 367.9;Urothelial cells: 556.9
	Cell type enhanced	Detected in many		Hepatocytes: 90.2;Plasma cells: 97.5
adipose tissue: 6.2	Cell type enhanced	Detected in many		Brain inhibitory neurons: 60.4;Cone photoreceptor cells: 161.5;Epicardial cells: 341.1;Other brain neurons: 176.7;Retinal amacrine cells: 96.6;Rod photoreceptor cells: 271.4;Vascular endothelial cells: 153.6
intestine: 134.1	Cell type enhanced	Detected in some		Cardiomyocytes: 9.7;Epicardial cells: 6.8;Hepatic stellate cells: 6.5;Retinal horizontal cells: 10.0;Smooth muscle cells: 43.6;Tuft cells: 11.5
esophagus: 1245.4;salivary gland: 585.3;skin 1: 554.9	Cell type enhanced	Detected in many		Breast secretory cells: 1810.4;Epididymal basal cells: 7482.4;Esophageal apical cells: 17505.8;Esophageal basal cells: 2063.6;Esophageal suprabasal cells: 5347.3;Ocular epithelial cells: 3288.3;Suprabasal keratinocytes: 216
epididymis: 11.5;lymphoid tissue: 3.4;salivary gland: 7.4	Group enriched	Detected in some	10	Epididymal principal cells: 81.4;Tuft cells: 149.9
	Cell type enhanced	Detected in many		Cone photoreceptor cells: 55.9;Pituicytes/FSCs: 82.9;Proximal tubule cells: 54.5;Rod photoreceptor cells: 82.1
salivary gland: 15.5	Cell type enhanced	Detected in many		Astrocytes: 17.2;Brain excitatory neurons: 38.6;Brain inhibitory neurons: 34.4;Distal convoluted tubule cells: 16.4;Oligodendrocyte progenitor cells: 32.6;Oligodendrocytes: 12.0;Other brain neurons: 34.3;Renal collecting duct
pancreas: 10.1	Cell type enhanced	Detected in some		Astrocytes: 20.3;Bergmann glia: 18.3;Brain excitatory neurons: 44.9;Brain inhibitory neurons: 50.6;Choroid plexus epithelial cells: 140.8;Distal convoluted tubule cells: 30.8;Ependymal cells: 18.8;Loop of henle epithelial cells:
blood vessel: 34.6	Cell type enhanced	Detected in some		Hepatic stellate cells: 64.5;Megakaryocytes: 92.7;Platelets: 361.1
	Cell type enhanced	Detected in some		Distal convoluted tubule cells: 5.7;Loop of henle epithelial cells: 6.4;Papillary tip epithelial cells: 25.2;Podocytes: 20.9;Proximal tubule cells: 7.6;Renal collecting duct intercalated cells: 16.2;Renal collecting duct principal cells
	Not detected	Not detected		
	Cell type enhanced	Detected in many		Endometrial glandular cells: 157.2;Esophageal apical cells: 172.5;Hematopoietic stem cells: 248.4;Innate lymphoid cells: 154.8;Mast cells: 164.4;Megakaryocyte progenitors: 353.8;Megakaryocyte-Erythroid progenitors: 163.5
placenta: 43.5	Group enriched	Detected in many	6	Lymphatic endothelial cells: 124.0;Oligodendrocyte progenitor cells: 141.9;Vascular endothelial cells: 321.2
	Cell type enhanced	Detected in all		Microglia: 420.0;Neutrophil progenitors: 220.0;Neutrophils: 230.5;Renal collecting duct principal cells: 288.6
	Cell type enhanced	Detected in many		Alveolar cells type 1: 446.4;Choroid plexus epithelial cells: 582.0;Lymphatic endothelial cells: 890.2;Microglia: 559.4;Neutrophils: 767.3;pDCs: 462.8;Vascular endothelial cells: 805.9
brain: 264.4	Cell type enhanced	Detected in many		Brain excitatory neurons: 848.5;Corticotrophs: 847.3;Epididymal basal cells: 501.8;Esophageal apical cells: 615.2;Esophageal suprabasal cells: 509.4;Microglia: 499.0;Ocular epithelial cells: 708.3;Retinal horizontal cells: 585.
	Cell type enhanced	Detected in many		Cardiomyocytes: 66.1;Choroid plexus epithelial cells: 46.2
	Cell type enhanced	Detected in many		Astrocytes: 502.5;Brain inhibitory neurons: 339.1;Oligodendrocyte progenitor cells: 519.1;Oligodendrocytes: 1082.2;Podocytes: 1678.9
	Cell type enhanced	Detected in many		Astrocytes: 113.3;Bergmann glia: 343.0;Foveolar cells: 39.9;Oligodendrocytes: 63.4;Tuft cells: 34.6
esophagus: 24.6;intestine: 40.5;stomach 1: 25.5	Cell type enhanced	Detected in many		Breast lactating cells: 146.5;Colonocytes: 257.4;Early spermatids: 404.4;Enterocytes: 197.9;Esophageal apical cells: 313.3;Foveolar cells: 173.4;Late primary spermatocytes: 321.5;Respiratory ciliated cells: 190.9
blood vessel: 345.2	Group enriched	Detected in many	6	Megakaryocytes: 2812.6;Platelets: 5578.5
bone marrow: 66.0	Cell type enriched	Detected in many	5	Neutrophils: 3164.5
	Cell type enhanced	Detected in some		Astrocytes: 1.0;Foveolar cells: 1.0;monocytes: 1.3;Neutrophils: 4.4
intestine: 251.2	Group enriched	Detected in some	4	Enterocytes: 796.4;Gastric progenitor cells: 271.7
testis: 113.4	Cell type enriched	Detected in some	4	Late spermatids: 6178.1
brain: 17.4;skeletal muscle: 5.5	Cell type enhanced	Detected in many		Adipocytes: 71.6;Brain excitatory neurons: 125.2;Cardiomyocytes: 147.4;Epicardial cells: 72.8;Gonadotrophs: 104.3;Lactotrophs: 67.3;Oocytes: 101.3;Podocytes: 326.2;Retinal amacrine cells: 73.2
	Cell type enhanced	Detected in all		Alveolar cells type 1: 153.5;Late spermatids: 141.7
	Cell type enhanced	Detected in many		Cardiomyocytes: 391.5;Myonuclei: 440.0;Neutrophils: 304.8;Pituitary stem cells: 239.6;Retinal horizontal cells: 342.6
prostate: 263.4;stomach 1: 138.0	Cell type enhanced	Detected in many		Alveolar cells type 2: 262.1;Colonocytes: 465.3;Distal convoluted tubule cells: 339.8;Enterocytes: 321.4;Esophageal apical cells: 423.6;Foveolar cells: 465.0;Mucous neck cells: 293.7;Parietal cells: 422.5;Prostatic club cells: 4
intestine: 28.5;salivary gland: 23.7	Cell type enhanced	Detected in many		Colonocytes: 60.9;Corticotrophs: 41.7;Enteric stem cells: 66.5;Enteric transient amplifying cells: 55.1;Innate lymphoid cells: 51.0;Kupffer cells: 43.0;Lacrimal acinar cells: 85.1;Lactotrophs: 64.2;Lymphatic endothelial cells: 63
	Cell type enhanced	Detected in many		Choroid plexus epithelial cells: 23.5;Distal convoluted tubule cells: 20.0;Loop of henle epithelial cells: 15.1;Papillary tip epithelial cells: 31.7;Podocytes: 28.1;Proximal tubule cells: 92.7;Renal collecting duct intercalated cells: 3
brain: 108.8;urinary bladder: 138.2	Cell type enhanced	Detected in many		Kupffer cells: 236.8;Ocular epithelial cells: 323.3;Papillary tip epithelial cells: 358.7;Prostatic club cells: 338.6;Syncytiotrophoblasts: 274.7;Urothelial cells: 982.2
adipose tissue: 5.1	Cell type enhanced	Detected in many		Epicardial cells: 100.1;Late spermatids: 13.4;Mast cells: 13.9;Mesothelial cells: 36.3;monocytes: 16.8;Neutrophils: 20.2;Podocytes: 12.9
lymphoid tissue: 24.4	Cell type enhanced	Detected in many		Adrenal cortex cells: 86.2;cDC: 218.9;Choroid plexus epithelial cells: 144.1;Kupffer cells: 188.6;Macrophages: 100.6;Monocyte progenitors: 201.8;monocytes: 257.5;Neutrophil progenitors: 133.3;Neutrophils: 419.5;pDCs: 10
	Cell type enhanced	Detected in many		Myonuclei: 115.0;Oligodendrocyte progenitor cells: 391.1
	Cell type enhanced	Detected in many		Endometrial glandular cells: 579.5;Endometrial luminal cells: 345.3;Ocular epithelial cells: 352.5
blood vessel: 20.3;kidney: 15.0	Cell type enhanced	Detected in many		Distal convoluted tubule cells: 686.9;Loop of henle epithelial cells: 227.9;Papillary tip epithelial cells: 132.0;Pituitary stem cells: 128.3;Proximal tubule cells: 1139.1;Renal collecting duct principal cells: 133.0;Renal connecting
	Cell type enhanced	Detected in many		Differentiating spermatogonia: 13.7;Early primary spermatocytes: 17.5;Erythrocyte progenitors: 11.0;Extravillous trophoblasts: 10.7;Late primary spermatocytes: 12.6;Megakaryocyte progenitors: 11.4;Monocyte progenitors: 1
lymphoid tissue: 74.3	Cell type enhanced	Detected in some		Cone photoreceptor cells: 18.6;Innate lymphoid cells: 24.1;NK-cells: 42.1;T-cells: 145.4;Thymocytes: 52.5
heart muscle: 394.7;skeletal muscle: 1819.8;tongue: 951.2	Group enriched	Detected in many	6	Cardiomyocytes: 11282.5;Myonuclei: 8756.3;Thymic myoid cells: 3855.9
bone marrow: 7.7;brain: 2.7;choroid plexus: 2.2;lung: 1.9;lymphoid	Cell type enriched	Detected in some	4	Platelets: 2534.0
	Cell type enhanced	Detected in many		Cardiomyocytes: 119.1;Colonocytes: 189.8;Enteric transient amplifying cells: 91.5;Goblet cells: 129.5;Prostatic glandular cells: 93.7;Salivary duct cells: 89.1
	Cell type enhanced	Detected in many		cDC: 30.7;Hematopoietic stem cells: 21.8;Hepatic stellate cells: 51.6;Hofbauer cells: 39.6;Macrophages: 34.8;Mast cells: 39.2;Melanocytes: 54.3;monocytes: 27.4;NK-cells: 37.9;pDCs: 34.1;Pericytes: 39.7;Syncytiotrophoblas
brain: 98.0	Cell type enhanced	Detected in many		Adipocytes: 123.4;Alveolar cells type 1: 145.7;Colonocytes: 127.4;Foveolar cells: 149.3;Oligodendrocytes: 134.7;Urothelial cells: 230.1
bone marrow: 47.3	Group enriched	Detected in some	23	Erythrocyte progenitors: 31.6;Erythrocytes: 61.5
	Low cell type specificity	Detected in many		
	Cell type enhanced	Detected in many		Retinal horizontal cells: 180.3
epididymis: 7.9;lymphoid tissue: 14.4	Cell type enhanced	Detected in many		Hematopoietic stem cells: 198.5;Innate lymphoid cells: 490.6;Megakaryocyte progenitors: 141.0;Megakaryocyte-Erythroid progenitors: 132.9;NK-cells: 792.4;Retinal ganglion cells: 154.5;T-cells: 215.8;Thymocytes: 468.4
	Cell type enhanced	Detected in all		Neutrophils: 308.7;Plasma cells: 588.1
bone marrow: 562.9;lymphoid tissue: 411.3	Cell type enhanced	Detected in many		cDC: 1347.0;Hofbauer cells: 3768.1;Kupffer cells: 3579.0;Macrophages: 1616.8;Mast cells: 614.0;monocytes: 2170.7;Neutrophils: 2394.5;NK-cells: 645.5;pDCs: 661.7
bone marrow: 74.0;lymphoid tissue: 105.6	Cell type enhanced	Detected in many		Cytotrophoblasts: 134.4;Enteric transient amplifying cells: 232.5;Esophageal basal cells: 179.4;Extravillous trophoblasts: 511.3;Gastric progenitor cells: 528.0;Hofbauer cells: 143.6;Megakaryocyte progenitors: 337.6;Megakar
	Cell type enhanced	Detected in all		Esophageal apical cells: 1697.9;Neutrophils: 1720.8
brain: 3.6;lung: 1.5;testis: 3.2	Cell type enhanced	Detected in some		Breast myoepithelial cells: 1.7;Breast secretory cells: 1.9;cDC: 3.3;Epididymal basal cells: 4.2;Esophageal apical cells: 2.9;Esophageal suprabasal cells: 2.6;Ocular epithelial cells: 15.7
esophagus: 5.9	Cell type enhanced	Detected in some		Early primary spermatocytes: 7.9;Early spermatids: 7.5;Epididymal basal cells: 10.2;Esophageal apical cells: 15.5;Esophageal suprabasal cells: 15.3;Late spermatids: 13.3;NK-cells: 6.5;Ocular epithelial cells: 44.2;Pancreatic a
	Low cell type specificity	Detected in all		
bone marrow: 1.5;testis: 4.6	Cell type enhanced	Detected in some		Differentiating spermatogonia: 3.1;Early primary spermatocytes: 6.9;Early spermatids: 2.8;Ependymal cells: 1.1;Foveolar cells: 1.2;Gastric chief cells: 1.1;Late primary spermatocytes: 6.2;Undifferentiated spermatogonia: 2.3
	Cell type enhanced	Detected in many		Alveolar cells type 1: 1287.3
	Cell type enhanced	Detected in all		Extravillous trophoblasts: 691.0;Megakaryocytes: 1112.7;Neutrophil progenitors: 496.9;Neutrophils: 1519.2;Platelets: 358.2
intestine: 410.7	Cell type enhanced	Detected in many		Colonocytes: 251.7;Enteric stem cells: 167.2;Enteric transient amplifying cells: 180.1;Enterocytes: 558.6;Epididymal efferent duct absorptive cells: 62.3;Gastric progenitor cells: 105.1;Goblet cells: 160.1;Neuroendocrine cells:
	Cell type enhanced	Detected in all		Rod photoreceptor cells: 253.6
	Cell type enhanced	Detected in many		Early spermatids: 562.5;Late spermatids: 1760.1;Megakaryocytes: 649.1;Platelets: 1221.3
	Low cell type specificity	Detected in many		
	Low cell type specificity	Detected in all		
	Cell type enhanced	Detected in many		Lymphatic endothelial cells: 307.0;NK-cells: 528.7
bone marrow: 55.3	Cell type enhanced	Detected in many		Endometrial glandular cells: 171.1;Endometrial luminal cells: 97.4;Erythrocytes: 165.3;Esophageal apical cells: 611.5;Late spermatids: 170.9;Platelets: 123.1
	Low cell type specificity	Detected in many		
retina: 72.0	Cell type enhanced	Detected in many		Cone photoreceptor cells: 236.0;Rod photoreceptor cells: 336.9
	Cell type enhanced	Detected in many		monocytes: 564.1;Neutrophils: 1413.4;Oligodendrocytes: 445.4
brain: 35.0	Cell type enhanced	Detected in some		Brain excitatory neurons: 13.2;Brain inhibitory neurons: 9.7;Late spermatids: 9.1;Oligodendrocyte progenitor cells: 41.7;Oligodendrocytes: 6.2;Other brain neurons: 18.1;Retinal amacrine cells: 11.0;Retinal bipolar cells: 14.9;F
	Cell type enhanced	Detected in many		Esophageal apical cells: 155.9
epididymis: 310.5;esophagus: 317.2	Cell type enhanced	Detected in many		Epididymal principal cells: 279.2;Esophageal apical cells: 1653.1;Esophageal suprabasal cells: 528.8;Platelets: 255.8;Somatotrophs: 158.7;Suprabasal keratinocytes: 175.8
	Cell type enhanced	Detected in many		Cardiomyocytes: 18.4;Epicardial cells: 9.1;Esophageal apical cells: 7.2

RNA single nuclei brain specificity	RNA single nuclei brain distribution	RNA single nuclei brain specificity score	RNA single nuclei brain specific nCPM	RNA cancer specificity	RNA cancer distribution
Low cell type specificity	Detected in all			Low cancer specificity	Detected in all
Low cell type specificity	Detected in all			Low cancer specificity	Detected in all
Cell type enhanced	Detected in all		astrocyte: 485.5;fibroblast: 338.6	Low cancer specificity	Detected in all
Low cell type specificity	Detected in all			Low cancer specificity	Detected in all
Cell type enriched	Detected in many	23	endothelial cell: 4738.8	Cancer enhanced	Detected in many
Group enriched	Detected in many	4	central nervous system macrophage: 26.6;endothelial cell: 25.7;lower rhombic lip: 12.0	Cancer enriched	Detected in some
Low cell type specificity	Detected in all			Low cancer specificity	Detected in all
Low cell type specificity	Detected in all			Low cancer specificity	Detected in many
Group enriched	Detected in many	4	astrocyte: 7.7;central nervous system macrophage: 15.2;deep-layer intratelencephalic: 12.3;ependymal cell: 11.5;leukocyte: 17.0;midbrain-derived inhibitory: 5.1;thalamic excitatory: 16.8;upper-layer intratelencephalic: 4.7	Low cancer specificity	Detected in many
Cell type enriched	Detected in all	7	central nervous system macrophage: 1176.0	Cancer enriched	Detected in all
Cell type enhanced	Detected in many		endothelial cell: 19.5;ependymal cell: 11.0	Cancer enhanced	Detected in many
Cell type enriched	Detected in many	17	endothelial cell: 1911.5	Cancer enhanced	Detected in many
Cell type enhanced	Detected in many		astrocyte: 66.2;vascular associated smooth muscle cell: 193.2	Low cancer specificity	Detected in all
Cell type enhanced	Detected in some		committed oligodendrocyte precursor: 9.8;leukocyte: 24.5;oligodendrocyte: 12.4;pericyte: 12.3	Low cancer specificity	Detected in all
Low cell type specificity	Detected in all			Low cancer specificity	Detected in all
Cell type enhanced	Detected in all		cerebellar inhibitory: 292.4	Cancer enhanced	Detected in many
Cell type enriched	Detected in many	40	central nervous system macrophage: 1395.1	Cancer enhanced	Detected in all
Low cell type specificity	Detected in all			Low cancer specificity	Detected in all
Cell type enriched	Detected in some	8	endothelial cell: 108.9	Low cancer specificity	Detected in many
Low cell type specificity	Detected in all			Low cancer specificity	Detected in all
Low cell type specificity	Detected in all			Low cancer specificity	Detected in all
Low cell type specificity	Detected in all			Group enriched	Detected in many
Low cell type specificity	Detected in all			Low cancer specificity	Detected in all
Cell type enhanced	Detected in many		fibroblast: 101.6;pericyte: 98.3;vascular associated smooth muscle cell: 139.6	Low cancer specificity	Detected in all
Low cell type specificity	Detected in all			Cancer enriched	Detected in many
Cell type enhanced	Detected in many		fibroblast: 194.7;pericyte: 161.1	Cancer enhanced	Detected in many
Low cell type specificity	Detected in all			Cancer enhanced	Detected in many
Low cell type specificity	Detected in many			Cancer enhanced	Detected in many
Cell type enhanced	Detected in many		Bergmann glia: 42.2;pericyte: 75.6	Low cancer specificity	Detected in many
Cell type enhanced	Detected in many		CGE interneuron: 144.7;hippocampal dentate gyrus: 115.4	Cancer enhanced	Detected in many
Cell type enhanced	Detected in some		central nervous system macrophage: 63.3;leukocyte: 27.5	Cancer enhanced	Detected in many
Low cell type specificity	Detected in all			Low cancer specificity	Detected in all
Cell type enhanced	Detected in many		central nervous system macrophage: 11.5	Group enriched	Detected in some
Cell type enhanced	Detected in many		endothelial cell: 159.9;fibroblast: 243.7;leukocyte: 254.5	Low cancer specificity	Detected in all
Low cell type specificity	Detected in many			Low cancer specificity	Detected in all
Low cell type specificity	Detected in all			Cancer enhanced	Detected in all
Not detected	Not detected			Cancer enhanced	Detected in many
Cell type enhanced	Detected in single		committed oligodendrocyte precursor: 1.5	Low cancer specificity	Detected in many
Low cell type specificity	Detected in all			Low cancer specificity	Detected in all
Cell type enhanced	Detected in all		LAMP5-LHX6 and Chandelier: 876.2	Group enriched	Detected in some
Cell type enhanced	Detected in single		central nervous system macrophage: 2.0	Cancer enriched	Detected in many
Low cell type specificity	Detected in many			Low cancer specificity	Detected in all
Group enriched	Detected in all	4	astrocyte: 131.4;Bergmann glia: 341.1;committed oligodendrocyte precursor: 119.8;oligodendrocyte: 405.3	Cancer enriched	Detected in single
Cell type enhanced	Detected in many		astrocyte: 101.7;central nervous system macrophage: 55.9	Low cancer specificity	Detected in all
Cell type enhanced	Detected in all		Bergmann glia: 6675.2	Cancer enhanced	Detected in many
Low cell type specificity	Detected in all			Low cancer specificity	Detected in all
Low cell type specificity	Detected in all			Low cancer specificity	Detected in all
Low cell type specificity	Detected in all			Low cancer specificity	Detected in all
Low cell type specificity	Detected in all			Low cancer specificity	Detected in all
Not detected	Not detected			Low cancer specificity	Detected in many
Cell type enhanced	Detected in some		LAMP5-LHX6 and Chandelier: 79.2;vascular associated smooth muscle cell: 45.9	Low cancer specificity	Detected in all
Low cell type specificity	Detected in all			Low cancer specificity	Detected in all
Cell type enriched	Detected in many	4	endothelial cell: 1260.0	Cancer enhanced	Detected in some
Low cell type specificity	Detected in all			Low cancer specificity	Detected in all
Cell type enriched	Detected in some	4	pericyte: 83.6	Cancer enhanced	Detected in all
Group enriched	Detected in many	13	endothelial cell: 140.6;ependymal cell: 259.6;fibroblast: 148.8;leukocyte: 226.5;vascular associated smooth muscle cell: 74.6	Low cancer specificity	Detected in all
Cell type enhanced	Detected in some		oligodendrocyte: 5.1	Low cancer specificity	Detected in many
Cell type enriched	Detected in many	5	endothelial cell: 341.4	Low cancer specificity	Detected in all
Low cell type specificity	Detected in all			Low cancer specificity	Detected in all
Low cell type specificity	Detected in all			Low cancer specificity	Detected in all
Cell type enriched	Detected in many	4	central nervous system macrophage: 2560.2	Cancer enhanced	Detected in all
Low cell type specificity	Detected in all			Low cancer specificity	Detected in all
Group enriched	Detected in some	24	central nervous system macrophage: 10.5;leukocyte: 26.8	Low cancer specificity	Detected in all
Cell type enhanced	Detected in all		choroid plexus epithelial cell: 166.9;endothelial cell: 270.2	Cancer enhanced	Detected in all
Low cell type specificity	Detected in all			Low cancer specificity	Detected in all
Group enriched	Detected in many	6	astrocyte: 220.1;choroid plexus epithelial cell: 82.5	Cancer enhanced	Detected in all
Not detected	Not detected			Not detected	Not detected
Low cell type specificity	Detected in all			Low cancer specificity	Detected in many
Group enriched	Detected in some	4	hippocampal dentate gyrus: 6.8;leukocyte: 21.1;lower rhombic lip: 16.7	Group enriched	Detected in all
Cell type enhanced	Detected in many		astrocyte: 951.4;Bergmann glia: 217.4;ependymal cell: 376.8	Cancer enriched	Detected in some
Low cell type specificity	Detected in all			Low cancer specificity	Detected in all
Group enriched	Detected in all	4	central nervous system macrophage: 1510.9;leukocyte: 2114.7	Low cancer specificity	Detected in all
Cell type enhanced	Detected in all		vascular associated smooth muscle cell: 355.4	Low cancer specificity	Detected in all
Cell type enhanced	Detected in single		upper rhombic lip: 1.0	Low cancer specificity	Detected in many
Cell type enhanced	Detected in all		oligodendrocyte: 1542.2	Low cancer specificity	Detected in all
Cell type enriched	Detected in all	4	central nervous system macrophage: 1542.0	Low cancer specificity	Detected in many
Group enriched	Detected in some	65	central nervous system macrophage: 70.6;leukocyte: 106.4	Low cancer specificity	Detected in all
Cell type enhanced	Detected in all		leukocyte: 166.3	Low cancer specificity	Detected in all
Low cell type specificity	Detected in all			Low cancer specificity	Detected in all
Cell type enriched	Detected in some	8	endothelial cell: 56.2	Low cancer specificity	Detected in all
Cell type enhanced	Detected in all		leukocyte: 135.0	Low cancer specificity	Detected in all
Group enriched	Detected in all	4	astrocyte: 375.8;Bergmann glia: 418.7;choroid plexus epithelial cell: 261.6;ependymal cell: 281.0	Cancer enhanced	Detected in many
Cell type enhanced	Detected in some		choroid plexus epithelial cell: 4.3;fibroblast: 2.6	Low cancer specificity	Detected in many
Cell type enhanced	Detected in many		CGE interneuron: 271.4;LAMP5-LHX6 and Chandelier: 266.9;leukocyte: 245.6	Low cancer specificity	Detected in all
Cell type enhanced	Detected in all		ependymal cell: 224.2	Low cancer specificity	Detected in some
Cell type enhanced	Detected in all		central nervous system macrophage: 188.9	Low cancer specificity	Detected in all
Low cell type specificity	Detected in all			Low cancer specificity	Detected in all
Low cell type specificity	Detected in all			Low cancer specificity	Detected in all
Low cell type specificity	Detected in all			Low cancer specificity	Detected in all
Cell type enriched	Detected in some	4	hippocampal CA1-3: 10.3	Cancer enriched	Detected in many
Low cell type specificity	Detected in all			Low cancer specificity	Detected in all
Cell type enhanced	Detected in all		oligodendrocyte precursor cell: 114.0	Cancer enriched	Detected in many
Low cell type specificity	Detected in all			Low cancer specificity	Detected in all
Low cell type specificity	Detected in all			Cancer enhanced	Detected in many
Cell type enhanced	Detected in some		ependymal cell: 1.8	Cancer enhanced	Detected in many
Low cell type specificity	Detected in all			Low cancer specificity	Detected in all
Low cell type specificity	Detected in all			Cancer enriched	Detected in single
Low cell type specificity	Detected in all			Low cancer specificity	Detected in all
Cell type enhanced	Detected in many		choroid plexus epithelial cell: 159.2	Not detected	Not detected
Low cell type specificity	Detected in all			Low cancer specificity	Detected in all
Low cell type specificity	Detected in all			Low cancer specificity	Detected in all
Low cell type specificity	Detected in all			Low cancer specificity	Detected in all
Low cell type specificity	Detected in all			Low cancer specificity	Detected in some
Low cell type specificity	Detected in all			Low cancer specificity	Detected in all
Low cell type specificity	Detected in all			Low cancer specificity	Detected in all
Cell type enhanced	Detected in many		central nervous system macrophage: 193.4;pericyte: 261.6;vascular associated smooth muscle cell: 197.3	Low cancer specificity	Detected in all
Cell type enhanced	Detected in all		endothelial cell: 2406.5;leukocyte: 2950.5	Low cancer specificity	Detected in all
Low cell type specificity	Detected in all			Low cancer specificity	Detected in all
Low cell type specificity	Detected in all			Low cancer specificity	Detected in all
Cell type enhanced	Detected in all		endothelial cell: 63.4;pericyte: 45.4	Low cancer specificity	Detected in many
Low cell type specificity	Detected in all			Low cancer specificity	Detected in all
Cell type enhanced	Detected in many		lower rhombic lip: 13.6;upper rhombic lip: 11.8	Low cancer specificity	Detected in all
Low cell type specificity	Detected in many			Low cancer specificity	Detected in many
Group enriched	Detected in some	98	central nervous system macrophage: 205.0;leukocyte: 201.4	Low cancer specificity	Detected in all
Cell type enriched	Detected in all	14	central nervous system macrophage: 868.9	Low cancer specificity	Detected in all
Low cell type specificity	Detected in all			Low cancer specificity	Detected in all
Cell type enriched	Detected in many	4	endothelial cell: 41.4	Cancer enhanced	Detected in many
Cell type enhanced	Detected in many		astrocyte: 186.4;Bergmann glia: 168.7;ependymal cell: 208.7	Low cancer specificity	Detected in many
Low cell type specificity	Detected in all			Low cancer specificity	Detected in all
Cell type enriched	Detected in some	4	central nervous system macrophage: 76.5	Low cancer specificity	Detected in all
Cell type enhanced	Detected in many		central nervous system macrophage: 25.0;oligodendrocyte: 23.3	Low cancer specificity	Detected in many
Cell type enhanced	Detected in some		choroid plexus epithelial cell: 9.1;upper rhombic lip: 5.7	Low cancer specificity	Detected in many
Not detected	Not detected			Cancer enhanced	Detected in many
Low cell type specificity	Detected in all			Low cancer specificity	Detected in all
Low cell type specificity	Detected in all			Low cancer specificity	Detected in all
Not detected	Not detected			Cancer enriched	Detected in single

Cell type enriched	Detected in some	11	upper rhombic lip: 12.8	Cancer enhanced	Detected in many
Low cell type specificity	Detected in all			Low cancer specificity	Detected in many
Low cell type specificity	Detected in all			Low cancer specificity	Detected in all
Cell type enhanced	Detected in many		Bergmann glia: 365.9;endothelial cell: 403.4;oligodendrocyte precursor cell: 468.1	Cancer enhanced	Detected in all
Group enriched	Detected in all	5	pericyte: 1943.5;vascular associated smooth muscle cell: 2377.0	Low cancer specificity	Detected in all
Cell type enhanced	Detected in all		astrocyte: 914.3;Bergmann glia: 1405.6	Low cancer specificity	Detected in all
Cell type enhanced	Detected in many		endothelial cell: 43.8;oligodendrocyte precursor cell: 28.1	Low cancer specificity	Detected in all
Low cell type specificity	Detected in all			Low cancer specificity	Detected in all
Group enriched	Detected in some	5	endothelial cell: 141.4;vascular associated smooth muscle cell: 266.3	Low cancer specificity	Detected in all
Group enriched	Detected in some	4	endothelial cell: 237.5;fibroblast: 64.5	Cancer enhanced	Detected in all
Cell type enhanced	Detected in many		oligodendrocyte: 45.7	Cancer enhanced	Detected in some
Low cell type specificity	Detected in all			Low cancer specificity	Detected in all
Low cell type specificity	Detected in all			Low cancer specificity	Detected in all
Low cell type specificity	Detected in all			Low cancer specificity	Detected in all
Low cell type specificity	Detected in all			Low cancer specificity	Detected in all
Cell type enhanced	Detected in many		ependymal cell: 138.9;leukocyte: 315.7;medium spiny neuron: 222.3	Low cancer specificity	Detected in all
Low cell type specificity	Detected in all			Low cancer specificity	Detected in all
Cell type enhanced	Detected in some		hippocampal CA1-3: 2.1;mammillary body: 3.8;oligodendrocyte precursor cell: 2.2	Cancer enriched	Detected in single
Low cell type specificity	Detected in many			Cancer enriched	Detected in some
Low cell type specificity	Detected in all			Low cancer specificity	Detected in all
Cell type enriched	Detected in single	60	leukocyte: 30.3	Low cancer specificity	Detected in many
Cell type enriched	Detected in single	5	leukocyte: 2.9	Low cancer specificity	Detected in many
Cell type enhanced	Detected in some		central nervous system macrophage: 2.4;endothelial cell: 7.7;vascular associated smooth muscle cell: 3.1	Cancer enhanced	Detected in all
Group enriched	Detected in many	6	astrocyte: 107.9;endothelial cell: 116.4;ependymal cell: 153.0	Low cancer specificity	Detected in all
Cell type enriched	Detected in some	6	central nervous system macrophage: 33.5	Cancer enhanced	Detected in all
Group enriched	Detected in some	5	central nervous system macrophage: 17.2;leukocyte: 5.4	Low cancer specificity	Detected in many
Cell type enriched	Detected in some	242	leukocyte: 345.7	Low cancer specificity	Detected in all
Cell type enhanced	Detected in many		leukocyte: 35.9	Low cancer specificity	Detected in some
Cell type enhanced	Detected in all		leukocyte: 317.1	Low cancer specificity	Detected in many
Cell type enriched	Detected in many	5	central nervous system macrophage: 36.7	Low cancer specificity	Detected in many
Cell type enriched	Detected in some	19	leukocyte: 55.4	Low cancer specificity	Detected in many
Cell type enhanced	Detected in all		endothelial cell: 336.5	Low cancer specificity	Detected in all
Cell type enriched	Detected in some	7	central nervous system macrophage: 37.3	Low cancer specificity	Detected in all
Group enriched	Detected in many	4	astrocyte: 240.4;leukocyte: 63.7;pericyte: 120.4	Low cancer specificity	Detected in many
Cell type enriched	Detected in some	179	leukocyte: 199.2	Low cancer specificity	Detected in all
Cell type enriched	Detected in single	470	leukocyte: 168.1	Low cancer specificity	Detected in many
Cell type enhanced	Detected in many		central nervous system macrophage: 162.0;fibroblast: 71.2;pericyte: 48.5	Low cancer specificity	Detected in all
Cell type enhanced	Detected in many		central nervous system macrophage: 29.2;pericyte: 32.3	Low cancer specificity	Detected in all
Cell type enriched	Detected in single	20	leukocyte: 19.1	Low cancer specificity	Detected in some
Cell type enhanced	Detected in many		astrocyte: 403.2;leukocyte: 474.2;vascular associated smooth muscle cell: 152.8	Low cancer specificity	Detected in all
Low cell type specificity	Detected in all			Low cancer specificity	Detected in all
Group enriched	Detected in some	5	fibroblast: 8.8;leukocyte: 18.7	Low cancer specificity	Detected in all
Group enriched	Detected in some	45	central nervous system macrophage: 226.4;leukocyte: 62.6	Low cancer specificity	Detected in many
Cell type enriched	Detected in some	8	central nervous system macrophage: 472.7	Low cancer specificity	Detected in all
Cell type enriched	Detected in many	9	leukocyte: 170.6	Low cancer specificity	Detected in all
Cell type enriched	Detected in some	14	leukocyte: 126.3	Low cancer specificity	Detected in many
Cell type enhanced	Detected in many		oligodendrocyte: 263.5;pericyte: 336.9;vascular associated smooth muscle cell: 199.9	Low cancer specificity	Detected in all
Low cell type specificity	Detected in all			Low cancer specificity	Detected in all
Cell type enhanced	Detected in many		oligodendrocyte: 72.2;pericyte: 38.6	Low cancer specificity	Detected in all
Cell type enhanced	Detected in many		choroid plexus epithelial cell: 100.7;leukocyte: 104.4	Low cancer specificity	Detected in all
Low cell type specificity	Detected in all			Low cancer specificity	Detected in all
Group enriched	Detected in many	8	choroid plexus epithelial cell: 52.1;committed oligodendrocyte precursor: 41.5;fibroblast: 17.4;oligodendrocyte: 53.1	Low cancer specificity	Detected in all
Cell type enhanced	Detected in all		midbrain-derived inhibitory: 1191.2	Cancer enhanced	Detected in all
Cell type enriched	Detected in some	34	upper rhombic lip: 63.9	Cancer enhanced	Detected in some
Low cell type specificity	Detected in some			Group enriched	Detected in many
Low cell type specificity	Detected in all			Cancer enhanced	Detected in many
Low cell type specificity	Detected in all			Low cancer specificity	Detected in some
Cell type enriched	Detected in many	4	choroid plexus epithelial cell: 123.0	Low cancer specificity	Detected in many
Low cell type specificity	Detected in all			Group enriched	Detected in some
Cell type enriched	Detected in some	4	endothelial cell: 186.4	Cancer enhanced	Detected in all
Cell type enhanced	Detected in many		pericyte: 1000.4;vascular associated smooth muscle cell: 631.5	Group enriched	Detected in many
Low cell type specificity	Detected in all			Low cancer specificity	Detected in all
Low cell type specificity	Detected in all			Low cancer specificity	Detected in all
Not detected	Not detected			Cancer enhanced	Detected in many
Not detected	Not detected			Cancer enhanced	Detected in many
Cell type enriched	Detected in many	14	ependymal cell: 110.8	Low cancer specificity	Detected in all
Cell type enhanced	Detected in all		endothelial cell: 778.3;leukocyte: 411.2	Low cancer specificity	Detected in all
Cell type enhanced	Detected in all		endothelial cell: 507.5	Low cancer specificity	Detected in all
Low cell type specificity	Detected in many			Low cancer specificity	Detected in all
Cell type enhanced	Detected in many		choroid plexus epithelial cell: 208.3;endothelial cell: 760.9	Cancer enhanced	Detected in all
Low cell type specificity	Detected in all			Low cancer specificity	Detected in all
Low cell type specificity	Detected in all			Low cancer specificity	Detected in many
Not detected	Not detected			Low cancer specificity	Detected in single
Cell type enriched	Detected in some	5	hippocampal dentate gyrus: 60.0	Cancer enhanced	Detected in many
Cell type enhanced	Detected in some		cerebellar inhibitory: 18.9;CGE interneuron: 27.8;thalamic excitatory: 26.3	Cancer enriched	Detected in single
Group enriched	Detected in some	8	cerebellar inhibitory: 55.5;midbrain-derived inhibitory: 49.0	Cancer enhanced	Detected in some
Low cell type specificity	Detected in many			Low cancer specificity	Detected in many
Low cell type specificity	Detected in some			Cancer enhanced	Detected in some
Cell type enhanced	Detected in single		lower rhombic lip: 1.9	Cancer enriched	Detected in single
Cell type enhanced	Detected in all		ependymal cell: 74.7	Low cancer specificity	Detected in all
Cell type enriched	Detected in single	4	oligodendrocyte: 2.8	Group enriched	Detected in many
Cell type enhanced	Detected in all		central nervous system macrophage: 169.2	Cancer enhanced	Detected in all
Cell type enhanced	Detected in some		fibroblast: 1.1;oligodendrocyte: 3.7	Cancer enriched	Detected in some
Cell type enriched	Detected in single	11	choroid plexus epithelial cell: 4.3	Cancer enhanced	Detected in many
Cell type enriched	Detected in some	19	choroid plexus epithelial cell: 70.9	Cancer enhanced	Detected in many
Low cell type specificity	Detected in all			Cancer enhanced	Detected in single
Group enriched	Detected in some	4	choroid plexus epithelial cell: 27.3;ependymal cell: 16.9	Cancer enhanced	Detected in many
Cell type enhanced	Detected in many		choroid plexus epithelial cell: 16.5;ependymal cell: 13.2	Low cancer specificity	Detected in many
Cell type enhanced	Detected in single		central nervous system macrophage: 1.7	Low cancer specificity	Detected in all
Cell type enhanced	Detected in some		upper rhombic lip: 4.8	Cancer enhanced	Detected in many
Cell type enriched	Detected in some	4	central nervous system macrophage: 11.6	Low cancer specificity	Detected in many
Cell type enhanced	Detected in all		endothelial cell: 63.4	Low cancer specificity	Detected in all
Cell type enhanced	Detected in all		endothelial cell: 719.9;oligodendrocyte: 290.2;vascular associated smooth muscle cell: 335.8	Low cancer specificity	Detected in all
Cell type enriched	Detected in many	5	endothelial cell: 204.7	Cancer enhanced	Detected in many
Low cell type specificity	Detected in all			Low cancer specificity	Detected in all
Low cell type specificity	Detected in all			Low cancer specificity	Detected in all
Cell type enriched	Detected in many	5	choroid plexus epithelial cell: 153.6	Cancer enhanced	Detected in some
Cell type enhanced	Detected in many		pericyte: 67.8;vascular associated smooth muscle cell: 38.8	Low cancer specificity	Detected in many
Low cell type specificity	Detected in all			Low cancer specificity	Detected in all
Cell type enhanced	Detected in many		cerebellar inhibitory: 394.9;CGE interneuron: 892.7	Cancer enriched	Detected in many
Low cell type specificity	Detected in all			Low cancer specificity	Detected in all
Cell type enhanced	Detected in some		ependymal cell: 1.7;medium spiny neuron: 1.6;midbrain-derived inhibitory: 1.4	Cancer enriched	Detected in many
Group enriched	Detected in many	9	ependymal cell: 95.5;fibroblast: 301.2;pericyte: 243.9;vascular associated smooth muscle cell: 256.5	Low cancer specificity	Detected in all
Cell type enhanced	Detected in many		endothelial cell: 35.6	Low cancer specificity	Detected in all
Cell type enhanced	Detected in all		pericyte: 169.1	Low cancer specificity	Detected in all
Cell type enhanced	Detected in some		fibroblast: 7.1;leukocyte: 12.1;oligodendrocyte: 8.2	Low cancer specificity	Detected in some
Cell type enhanced	Detected in single		choroid plexus epithelial cell: 1.1	Low cancer specificity	Detected in many
Cell type enriched	Detected in many	4	choroid plexus epithelial cell: 100.8	Not detected	Not detected
Cell type enhanced	Detected in all		astrocyte: 484.0;oligodendrocyte: 1086.7	Group enriched	Detected in all
Cell type enhanced	Detected in many		fibroblast: 159.8	Low cancer specificity	Detected in all
Cell type enriched	Detected in many	17	central nervous system macrophage: 840.3	Low cancer specificity	Detected in all
Low cell type specificity	Detected in all			Low cancer specificity	Detected in all
Low cell type specificity	Detected in all			Low cancer specificity	Detected in all
Cell type enhanced	Detected in all		endothelial cell: 823.7	Low cancer specificity	Detected in all
Low cell type specificity	Detected in all			Low cancer specificity	Detected in all
Low cell type specificity	Detected in all			Low cancer specificity	Detected in all
Cell type enhanced	Detected in all		vascular associated smooth muscle cell: 136.3	Low cancer specificity	Detected in all
Cell type enriched	Detected in many	12	central nervous system macrophage: 983.8	Group enriched	Detected in many
Low cell type specificity	Detected in all			Low cancer specificity	Detected in all
Group enriched	Detected in some	14	central nervous system macrophage: 1.3;leukocyte: 4.5	Low cancer specificity	Detected in many
Cell type enhanced	Detected in many		fibroblast: 169.3	Low cancer specificity	Detected in all
Low cell type specificity	Detected in all			Low cancer specificity	Detected in all
Group enriched	Detected in some	70	central nervous system macrophage: 144.5;leukocyte: 75.8	Low cancer specificity	Detected in all
Cell type enhanced	Detected in many		Bergmann glia: 32.9;endothelial cell: 19.4;fibroblast: 19.5	Cancer enhanced	Detected in all
Low cell type specificity	Detected in all			Low cancer specificity	Detected in all
Cell type enhanced	Detected in all		central nervous system macrophage: 118.5	Low cancer specificity	Detected in all
Low cell type specificity	Detected in all			Low cancer specificity	Detected in all
Cell type enriched	Detected in many	9	committed oligodendrocyte precursor: 152.3	Cancer enriched	Detected in some
Cell type enhanced	Detected in all		astrocyte: 116.1;ependymal cell: 130.3;oligodendrocyte: 97.1;oligodendrocyte precursor cell: 99.8	Low cancer specificity	Detected in all

Cell type enhanced	Detected in all		choroid plexus epithelial cell: 472.9;fibroblast: 253.8	Low cancer specificity	Detected in all
Cell type enhanced	Detected in all		medium spiny neuron: 849.1	Low cancer specificity	Detected in many
Cell type enhanced	Detected in many		ependymal cell: 105.8	Low cancer specificity	Detected in many
Low cell type specificity	Detected in all			Low cancer specificity	Detected in all
Cell type enhanced	Detected in all		committed oligodendrocyte precursor: 2251.0;oligodendrocyte: 2396.9	Low cancer specificity	Detected in all
Low cell type specificity	Detected in all			Cancer enhanced	Detected in all
Low cell type specificity	Detected in all			Low cancer specificity	Detected in all
Low cell type specificity	Detected in many			Low cancer specificity	Detected in all
Low cell type specificity	Detected in all			Low cancer specificity	Detected in all
Cell type enriched	Detected in all	4	central nervous system macrophage: 8867.4	Low cancer specificity	Detected in all
Group enriched	Detected in all	7	central nervous system macrophage: 3289.7;choroid plexus epithelial cell: 1065.3;leukocyte: 910.6	Low cancer specificity	Detected in all
Cell type enriched	Detected in single	75	leukocyte: 59.6	Low cancer specificity	Detected in all
Cell type enhanced	Detected in many		central nervous system macrophage: 37.8	Low cancer specificity	Detected in all
Cell type enhanced	Detected in some		ependymal cell: 3.9	Group enriched	Detected in many
Cell type enhanced	Detected in many		choroid plexus epithelial cell: 14.4	Cancer enhanced	Detected in single
Cell type enhanced	Detected in many		central nervous system macrophage: 26.9	Cancer enhanced	Detected in all
Cell type enhanced	Detected in some		amygdala excitatory: 1.9;fibroblast: 2.7;miscellaneous: 3.9	Group enriched	Detected in many
Cell type enriched	Detected in many	5	choroid plexus epithelial cell: 54.3	Low cancer specificity	Detected in all
Group enriched	Detected in many	5	choroid plexus epithelial cell: 20.6;endothelial cell: 40.8;fibroblast: 58.6;hippocampal dentate gyrus: 23.6	Cancer enhanced	Detected in many
Cell type enhanced	Detected in all		astrocyte: 3777.2;Bergmann glia: 5440.0	Cancer enriched	Detected in all
Cell type enhanced	Detected in all		mammillary body: 168.4	Cancer enhanced	Detected in many
Cell type enriched	Detected in some	4	endothelial cell: 9.7	Cancer enhanced	Detected in all
Group enriched	Detected in many	24	pericyte: 307.5;vascular associated smooth muscle cell: 339.4	Low cancer specificity	Detected in all
Group enriched	Detected in many	4	astrocyte: 449.1;Bergmann glia: 203.3	Cancer enhanced	Detected in all
Cell type enhanced	Detected in many		central nervous system macrophage: 12.5;fibroblast: 14.4	Low cancer specificity	Detected in all
Low cell type specificity	Detected in all			Low cancer specificity	Detected in all
Low cell type specificity	Detected in all			Cancer enhanced	Detected in many
Cell type enhanced	Detected in many		astrocyte: 363.8;Bergmann glia: 419.5	Cancer enriched	Detected in all
Low cell type specificity	Detected in all			Low cancer specificity	Detected in all
Cell type enhanced	Detected in all		leukocyte: 56.0	Low cancer specificity	Detected in all
Group enriched	Detected in many	5	pericyte: 142.0;vascular associated smooth muscle cell: 126.6	Low cancer specificity	Detected in all
Low cell type specificity	Detected in all			Low cancer specificity	Detected in all
Low cell type specificity	Detected in all			Cancer enhanced	Detected in many
Low cell type specificity	Detected in all			Low cancer specificity	Detected in all
Cell type enhanced	Detected in many		endothelial cell: 451.0;fibroblast: 178.6	Low cancer specificity	Detected in all
Low cell type specificity	Detected in all			Low cancer specificity	Detected in all
Low cell type specificity	Detected in all			Low cancer specificity	Detected in all
Cell type enhanced	Detected in many		upper rhombic lip: 12.2	Cancer enhanced	Detected in all
Low cell type specificity	Detected in all			Low cancer specificity	Detected in many
Low cell type specificity	Detected in all			Low cancer specificity	Detected in all
Low cell type specificity	Detected in all			Low cancer specificity	Detected in all
Cell type enhanced	Detected in all		central nervous system macrophage: 2248.2;committed oligodendrocyte precursor: 1661.0	Low cancer specificity	Detected in all
Low cell type specificity	Detected in all			Low cancer specificity	Detected in many
Low cell type specificity	Detected in all			Low cancer specificity	Detected in all
Low cell type specificity	Detected in many			Low cancer specificity	Detected in many
Group enriched	Detected in some	4	endothelial cell: 18.5;pericyte: 45.1;vascular associated smooth muscle cell: 30.3	Low cancer specificity	Detected in all
Low cell type specificity	Detected in all			Low cancer specificity	Detected in many
Cell type enhanced	Detected in all		hippocampal CA4: 930.6	Cancer enhanced	Detected in some
Cell type enhanced	Detected in all		committed oligodendrocyte precursor: 823.0	Cancer enriched	Detected in many
Low cell type specificity	Detected in many			Cancer enhanced	Detected in all
Cell type enhanced	Detected in all		astrocyte: 24.6	Low cancer specificity	Detected in all
Group enriched	Detected in many	4	astrocyte: 27.0;Bergmann glia: 16.9;choroid plexus epithelial cell: 15.4;ependymal cell: 55.2;fibroblast: 34.0;pericyte: 14.2;vascular associated smooth muscle cell: 20.0	Low cancer specificity	Detected in all
Group enriched	Detected in many	17	committed oligodendrocyte precursor: 315.9;oligodendrocyte: 330.4;oligodendrocyte precursor cell: 87.7	Low cancer specificity	Detected in all
Cell type enhanced	Detected in all		oligodendrocyte: 1720.4	Low cancer specificity	Detected in all
Cell type enhanced	Detected in all		endothelial cell: 1378.2;vascular associated smooth muscle cell: 1373.9	Low cancer specificity	Detected in all
Cell type enhanced	Detected in many		central nervous system macrophage: 24.2;oligodendrocyte: 95.4	Cancer enhanced	Detected in all
Group enriched	Detected in many	4	astrocyte: 190.9;choroid plexus epithelial cell: 490.9;ependymal cell: 300.3;leukocyte: 200.5	Low cancer specificity	Detected in all
Cell type enhanced	Detected in many		endothelial cell: 54.7;ependymal cell: 30.5	Low cancer specificity	Detected in all
Cell type enhanced	Detected in single		endothelial cell: 1.1	Cancer enhanced	Detected in many
Group enriched	Detected in many	4	astrocyte: 31.2;central nervous system macrophage: 20.9;endothelial cell: 20.1;ependymal cell: 26.1;fibroblast: 29.7;leukocyte: 33.9;pericyte: 33.9;vascular associated smooth muscle cell: 21.0	Low cancer specificity	Detected in all
Cell type enhanced	Detected in all		cerebellar inhibitory: 93.4	Low cancer specificity	Detected in all
Cell type enriched	Detected in many	4	vascular associated smooth muscle cell: 64.7	Low cancer specificity	Detected in all
Low cell type specificity	Detected in all			Low cancer specificity	Detected in many
Group enriched	Detected in some	4	central nervous system macrophage: 5.4;leukocyte: 6.8	Not detected	Not detected
Cell type enriched	Detected in single	27	central nervous system macrophage: 24.2	Cancer enhanced	Detected in all
Group enriched	Detected in some	31	central nervous system macrophage: 82.9;leukocyte: 22.1	Low cancer specificity	Detected in all
Cell type enhanced	Detected in all		central nervous system macrophage: 1570.0	Low cancer specificity	Detected in all
Cell type enriched	Detected in single	81	leukocyte: 71.3	Low cancer specificity	Detected in all
Group enriched	Detected in some	11	committed oligodendrocyte precursor: 2.8;oligodendrocyte: 6.0	Cancer enriched	Detected in single
Cell type enhanced	Detected in many		central nervous system macrophage: 36.7	Cancer enhanced	Detected in many
Low cell type specificity	Detected in all			Cancer enhanced	Detected in all
Cell type enriched	Detected in many	4	endothelial cell: 240.3	Low cancer specificity	Detected in all
Low cell type specificity	Detected in all			Cancer enhanced	Detected in all
Cell type enhanced	Detected in all		endothelial cell: 440.4	Low cancer specificity	Detected in all
Low cell type specificity	Detected in all			Low cancer specificity	Detected in all
Low cell type specificity	Detected in all			Low cancer specificity	Detected in all
Cell type enhanced	Detected in all		hippocampal dentate gyrus: 132.0	Cancer enhanced	Detected in many
Cell type enriched	Detected in many	12	endothelial cell: 3142.1	Cancer enhanced	Detected in all
Cell type enriched	Detected in some	8	endothelial cell: 32.6	Cancer enhanced	Detected in all
Group enriched	Detected in all	5	central nervous system macrophage: 695.8;endothelial cell: 316.3	Low cancer specificity	Detected in all
Low cell type specificity	Detected in all			Low cancer specificity	Detected in all
Cell type enriched	Detected in some	5	central nervous system macrophage: 17.3	Low cancer specificity	Detected in all
Low cell type specificity	Detected in all			Low cancer specificity	Detected in all
Cell type enhanced	Detected in all		committed oligodendrocyte precursor: 3021.6	Cancer enhanced	Detected in all
Cell type enhanced	Detected in many		midbrain-derived inhibitory: 27.6;pericyte: 27.8	Low cancer specificity	Detected in all
Cell type enriched	Detected in many	4	endothelial cell: 83.6	Cancer enhanced	Detected in all
Cell type enhanced	Detected in all		endothelial cell: 156.0	Low cancer specificity	Detected in all
Cell type enhanced	Detected in all		central nervous system macrophage: 1026.5;oligodendrocyte: 836.4	Cancer enhanced	Detected in all
Low cell type specificity	Detected in all			Low cancer specificity	Detected in many
Cell type enhanced	Detected in all		cerebellar inhibitory: 583.1	Cancer enhanced	Detected in single
Low cell type specificity	Detected in all			Group enriched	Detected in some
Cell type enhanced	Detected in many		Bergmann glia: 350.0;ependymal cell: 484.2	Not detected	Not detected
Cell type enhanced	Detected in all		eccentric medium spiny neuron: 1425.8	Cancer enhanced	Detected in some
Cell type enriched	Detected in many	6	oligodendrocyte precursor cell: 99.0	Group enriched	Detected in some
Low cell type specificity	Detected in all			Cancer enriched	Detected in many
Low cell type specificity	Detected in all			Low cancer specificity	Detected in all
Cell type enriched	Detected in some	4	central nervous system macrophage: 15.6	Low cancer specificity	Detected in many
Low cell type specificity	Detected in all			Low cancer specificity	Detected in all
Low cell type specificity	Detected in all			Low cancer specificity	Detected in all
Cell type enhanced	Detected in single		upper rhombic lip: 3.1	Cancer enhanced	Detected in many
Cell type enhanced	Detected in many		Bergmann glia: 4.3	Not detected	Not detected
Low cell type specificity	Detected in all			Cancer enhanced	Detected in many
Low cell type specificity	Detected in all			Low cancer specificity	Detected in all
Group enriched	Detected in many	7	astrocyte: 592.6;Bergmann glia: 287.4;ependymal cell: 388.5	Low cancer specificity	Detected in all
Cell type enriched	Detected in some	4	cerebellar inhibitory: 6.1	Cancer enhanced	Detected in many
Not detected	Not detected			Cancer enhanced	Detected in many
Group enriched	Detected in some	27	astrocyte: 82.3;fibroblast: 27.3	Cancer enhanced	Detected in many
Group enriched	Detected in many	17	pericyte: 520.7;vascular associated smooth muscle cell: 200.1	Cancer enhanced	Detected in all
Group enriched	Detected in many	14	committed oligodendrocyte precursor: 19.0;oligodendrocyte: 61.3	Low cancer specificity	Detected in many
Group enriched	Detected in many	7	central nervous system macrophage: 338.5;committed oligodendrocyte precursor: 230.5;oligodendrocyte: 847.0	Cancer enhanced	Detected in many
Low cell type specificity	Detected in all			Low cancer specificity	Detected in all
Cell type enhanced	Detected in some		splatter: 5.3;vascular associated smooth muscle cell: 3.0	Cancer enhanced	Detected in some
Cell type enhanced	Detected in some		amygdala excitatory: 12.2;upper-layer intratelencephalic: 33.8	Cancer enhanced	Detected in single
Cell type enhanced	Detected in many		lower rhombic lip: 33.0;splatter: 41.8	Not detected	Not detected
Low cell type specificity	Detected in many			Cancer enhanced	Detected in some
Cell type enhanced	Detected in many		amygdala excitatory: 161.0;upper-layer intratelencephalic: 145.1	Cancer enriched	Detected in single
Cell type enhanced	Detected in all		endothelial cell: 265.0	Low cancer specificity	Detected in all
Low cell type specificity	Detected in all			Low cancer specificity	Detected in all
Low cell type specificity	Detected in all			Low cancer specificity	Detected in all
Low cell type specificity	Detected in all			Low cancer specificity	Detected in all
Low cell type specificity	Detected in many			Not detected	Not detected
Low cell type specificity	Detected in all			Low cancer specificity	Detected in all
Cell type enriched	Detected in many	4	fibroblast: 244.5	Cancer enhanced	Detected in all
Cell type enhanced	Detected in many		astrocyte: 22.4;choroid plexus epithelial cell: 23.4	Cancer enriched	Detected in all
Not detected	Not detected			Not detected	Not detected
Cell type enhanced	Detected in many		ependymal cell: 31.0;oligodendrocyte precursor cell: 27.2	Not detected	Not detected
Cell type enriched	Detected in many	4	central nervous system macrophage: 41.5	Low cancer specificity	Detected in all
Cell type enriched	Detected in some	100	leukocyte: 204.7	Low cancer specificity	Detected in some
Group enriched	Detected in many	38	central nervous system macrophage: 216.7;leukocyte: 58.1	Low cancer specificity	Detected in all

Cell type enriched	Detected in some	10	vascular associated smooth muscle cell: 35.4	Low cancer specificity	Detected in some
Cell type enhanced	Detected in many		astrocyte: 161.2;Bergmann glia: 238.4;oligodendrocyte precursor cell: 211.6	Cancer enriched	Detected in some
Not detected	Not detected			Cancer enhanced	Detected in some
Not detected	Not detected			Cancer enriched	Detected in some
Cell type enhanced	Detected in some		endothelial cell: 18.0;fibroblast: 11.7;vascular associated smooth muscle cell: 53.4	Low cancer specificity	Detected in many
Group enriched	Detected in many	5	pericyte: 153.3;upper rhombic lip: 49.1;vascular associated smooth muscle cell: 160.1	Low cancer specificity	Detected in all
Low cell type specificity	Detected in many			Low cancer specificity	Detected in many
Low cell type specificity	Detected in all			Low cancer specificity	Detected in all
Cell type enhanced	Detected in all		choroid plexus epithelial cell: 771.7	Low cancer specificity	Detected in many
Group enriched	Detected in some	4	choroid plexus epithelial cell: 10.0;upper rhombic lip: 5.7	Low cancer specificity	Detected in many
Cell type enhanced	Detected in all		lower rhombic lip: 3228.1;MGE interneuron: 3708.2;midbrain-derived inhibitory: 8188.5	Cancer enriched	Detected in some
Low cell type specificity	Detected in all			Group enriched	Detected in many
Low cell type specificity	Detected in all			Cancer enhanced	Detected in some
Cell type enhanced	Detected in all		pericyte: 461.8;vascular associated smooth muscle cell: 461.0	Low cancer specificity	Detected in many
Group enriched	Detected in some	7	CGE interneuron: 19.2;LAMP5-LHX6 and Chandelier: 19.3;mammillary body: 7.0	Cancer enriched	Detected in some
Low cell type specificity	Detected in some			Cancer enhanced	Detected in many
Low cell type specificity	Detected in some			Low cancer specificity	Detected in many
Low cell type specificity	Detected in many			Not detected	Not detected
Cell type enhanced	Detected in many		central nervous system macrophage: 87.4;fibroblast: 71.2;leukocyte: 65.8;vascular associated smooth muscle cell: 43.7	Cancer enhanced	Detected in all
Low cell type specificity	Detected in many			Low cancer specificity	Detected in all
Low cell type specificity	Detected in many			Cancer enhanced	Detected in some
Not detected	Not detected			Cancer enhanced	Detected in many
Group enriched	Detected in many	6	central nervous system macrophage: 286.3;leukocyte: 89.3	Low cancer specificity	Detected in all
Group enriched	Detected in many	16	central nervous system macrophage: 224.3;leukocyte: 158.3	Low cancer specificity	Detected in all
Cell type enhanced	Detected in many		cerebellar inhibitory: 120.7;deep-layer corticothalamic and 6b: 132.2;LAMP5-LHX6 and Chandelier: 186.6;lower rhombic lip: 211.5	Not detected	Not detected
Cell type enhanced	Detected in many		fibroblast: 13.4	Low cancer specificity	Detected in all
Cell type enhanced	Detected in all		endothelial cell: 514.2;leukocyte: 779.7	Low cancer specificity	Detected in all
Group enriched	Detected in many	5	central nervous system macrophage: 263.9;leukocyte: 90.2	Low cancer specificity	Detected in all
Cell type enhanced	Detected in many		endothelial cell: 878.4;leukocyte: 507.1	Low cancer specificity	Detected in all
Low cell type specificity	Detected in all			Low cancer specificity	Detected in all
Cell type enhanced	Detected in some		Bergmann glia: 6.2;cerebellar inhibitory: 19.8	Low cancer specificity	Detected in all
Low cell type specificity	Detected in many			Low cancer specificity	Detected in many
Low cell type specificity	Detected in all			Low cancer specificity	Detected in all
Cell type enhanced	Detected in all		endothelial cell: 342.9;vascular associated smooth muscle cell: 632.2	Low cancer specificity	Detected in all
Cell type enhanced	Detected in many		LAMP5-LHX6 and Chandelier: 134.7;mammillary body: 335.5	Group enriched	Detected in some
Cell type enhanced	Detected in many		central nervous system macrophage: 348.6;oligodendrocyte: 220.5;oligodendrocyte precursor cell: 347.2	Low cancer specificity	Detected in all
Cell type enhanced	Detected in all		hippocampal CA4: 324.2	Low cancer specificity	Detected in all
Low cell type specificity	Detected in all			Low cancer specificity	Detected in all
Not detected	Not detected			Low cancer specificity	Detected in all
Low cell type specificity	Detected in all			Low cancer specificity	Detected in all
Not detected	Not detected			Low cancer specificity	Detected in all
Not detected	Not detected			Low cancer specificity	Detected in some
Cell type enhanced	Detected in all		central nervous system macrophage: 750.0;endothelial cell: 370.8	Low cancer specificity	Detected in all
Cell type enhanced	Detected in all		endothelial cell: 1591.5	Low cancer specificity	Detected in many
Group enriched	Detected in many	4	central nervous system macrophage: 12.1;leukocyte: 22.8	Low cancer specificity	Detected in all
Group enriched	Detected in many	32	central nervous system macrophage: 125.7;leukocyte: 186.8	Low cancer specificity	Detected in all
Group enriched	Detected in many	4	central nervous system macrophage: 24.9;endothelial cell: 37.7;fibroblast: 30.5;leukocyte: 26.0	Low cancer specificity	Detected in all
Group enriched	Detected in many	4	astrocyte: 53.1;Bergmann glia: 86.5;committed oligodendrocyte precursor: 79.2;oligodendrocyte precursor cell: 68.3	Low cancer specificity	Detected in all
Cell type enhanced	Detected in single		endothelial cell: 1.1	Cancer enhanced	Detected in many
Cell type enriched	Detected in single	116	leukocyte: 11.7	Low cancer specificity	Detected in all
Cell type enhanced	Detected in many		central nervous system macrophage: 90.5;endothelial cell: 197.2;vascular associated smooth muscle cell: 79.7	Low cancer specificity	Detected in all
Cell type enhanced	Detected in all		central nervous system macrophage: 924.1	Low cancer specificity	Detected in all
Cell type enriched	Detected in some	115	leukocyte: 454.7	Low cancer specificity	Detected in all
Cell type enhanced	Detected in all		oligodendrocyte: 555.0	Low cancer specificity	Detected in all
Low cell type specificity	Detected in all			Low cancer specificity	Detected in all
Cell type enhanced	Detected in many		vascular associated smooth muscle cell: 121.7	Low cancer specificity	Detected in all
Cell type enriched	Detected in some	5	central nervous system macrophage: 457.7	Low cancer specificity	Detected in all
Low cell type specificity	Detected in all			Low cancer specificity	Detected in all
Group enriched	Detected in some	15	central nervous system macrophage: 126.3;leukocyte: 200.1	Low cancer specificity	Detected in all
Not detected	Not detected			Cancer enhanced	Detected in all
Group enriched	Detected in many	5	astrocyte: 182.1;Bergmann glia: 78.1;ependymal cell: 126.6	Low cancer specificity	Detected in all
Low cell type specificity	Detected in all			Low cancer specificity	Detected in all
Group enriched	Detected in some	6	lower rhombic lip: 6.7;thalamic excitatory: 1.7	Cancer enriched	Detected in many
Cell type enhanced	Detected in all		endothelial cell: 440.2	Cancer enriched	Detected in all
Cell type enriched	Detected in some	7	leukocyte: 106.2	Low cancer specificity	Detected in all
Cell type enhanced	Detected in many		Bergmann glia: 36.7;fibroblast: 44.6;pericyte: 38.9	Low cancer specificity	Detected in all
Cell type enhanced	Detected in all		choroid plexus epithelial cell: 1361.2	Low cancer specificity	Detected in all
Low cell type specificity	Detected in all			Low cancer specificity	Detected in all
Low cell type specificity	Detected in all			Low cancer specificity	Detected in all
Not detected	Not detected			Group enriched	Detected in some
Cell type enhanced	Detected in many		oligodendrocyte: 22.9;vascular associated smooth muscle cell: 41.1	Cancer enhanced	Detected in many
Group enriched	Detected in some	4	central nervous system macrophage: 27.9;endothelial cell: 9.8;fibroblast: 18.0;leukocyte: 7.2;pericyte: 20.2;vascular associated smooth muscle cell: 8.2	Low cancer specificity	Detected in all
Cell type enriched	Detected in some	13	oligodendrocyte precursor cell: 14.6	Not detected	Not detected
Group enriched	Detected in many	9	committed oligodendrocyte precursor: 514.5;oligodendrocyte: 1153.5;oligodendrocyte precursor cell: 520.2	Low cancer specificity	Detected in some
Cell type enriched	Detected in some	21	fibroblast: 115.2	Cancer enhanced	Detected in many
Cell type enhanced	Detected in many		central nervous system macrophage: 11.0;leukocyte: 9.5	Low cancer specificity	Detected in all
Cell type enhanced	Detected in all		central nervous system macrophage: 392.3	Cancer enhanced	Detected in all
Cell type enhanced	Detected in some		upper-layer intratelencephalic: 6.1	Cancer enhanced	Detected in many
Cell type enhanced	Detected in many		committed oligodendrocyte precursor: 165.1;MGE interneuron: 103.0	Low cancer specificity	Detected in all
Low cell type specificity	Detected in many			Cancer enhanced	Detected in single
Cell type enhanced	Detected in many		deep-layer intratelencephalic: 42.7;upper-layer intratelencephalic: 35.0	Cancer enriched	Detected in single
Cell type enriched	Detected in many	5	oligodendrocyte: 54.7	Cancer enhanced	Detected in some
Cell type enhanced	Detected in many		choroid plexus epithelial cell: 78.0	Low cancer specificity	Detected in all
Cell type enhanced	Detected in many		deep-layer near-projecting: 11.1;splatter: 7.8	Cancer enriched	Detected in many
Cell type enhanced	Detected in many		cerebellar inhibitory: 699.5;CGE interneuron: 325.6;midbrain-derived inhibitory: 571.8	Cancer enhanced	Detected in many
Low cell type specificity	Detected in all			Low cancer specificity	Detected in all
Cell type enhanced	Detected in some		deep-layer intratelencephalic: 12.6;hippocampal CA4: 18.6;lower rhombic lip: 9.3	Cancer enhanced	Detected in many
Cell type enriched	Detected in some	11	leukocyte: 279.1	Low cancer specificity	Detected in all
Low cell type specificity	Detected in many			Cancer enhanced	Detected in many
Group enriched	Detected in some	33	central nervous system macrophage: 108.8;leukocyte: 39.5	Low cancer specificity	Detected in many
Cell type enhanced	Detected in all		oligodendrocyte: 659.3	Low cancer specificity	Detected in all
Low cell type specificity	Detected in all			Low cancer specificity	Detected in all
Cell type enriched	Detected in all	6	leukocyte: 75.1	Low cancer specificity	Detected in all
Cell type enriched	Detected in all	5	central nervous system macrophage: 173.5	Low cancer specificity	Detected in all
Cell type enriched	Detected in single	34	leukocyte: 25.8	Low cancer specificity	Detected in many
Cell type enriched	Detected in many	4	leukocyte: 549.0	Low cancer specificity	Detected in all
Group enriched	Detected in all	4	choroid plexus epithelial cell: 132.0;endothelial cell: 493.1;fibroblast: 492.0	Cancer enriched	Detected in many
Cell type enriched	Detected in single	19	fibroblast: 2.4	Not detected	Not detected
Low cell type specificity	Detected in all			Cancer enhanced	Detected in all
Low cell type specificity	Detected in many			Not detected	Not detected
Cell type enhanced	Detected in many		leukocyte: 212.6;oligodendrocyte: 117.8	Low cancer specificity	Detected in all
Low cell type specificity	Detected in all			Low cancer specificity	Detected in all
Cell type enriched	Detected in some	5	ependymal cell: 59.7	Cancer enhanced	Detected in many
Cell type enhanced	Detected in some		astrocyte: 22.6;Bergmann glia: 20.2;ependymal cell: 38.6	Cancer enhanced	Detected in some
Cell type enriched	Detected in all	5	central nervous system macrophage: 1088.8	Low cancer specificity	Detected in all
Cell type enhanced	Detected in all		vascular associated smooth muscle cell: 2556.0	Cancer enhanced	Detected in all
Low cell type specificity	Detected in many			Not detected	Not detected
Cell type enhanced	Detected in all		fibroblast: 578.6	Low cancer specificity	Detected in all
Cell type enriched	Detected in some	5	upper rhombic lip: 9.6	Cancer enriched	Detected in many
Cell type enriched	Detected in many	7	endothelial cell: 195.4	Low cancer specificity	Detected in all
Group enriched	Detected in some	70	central nervous system macrophage: 169.7;leukocyte: 72.3	Low cancer specificity	Detected in all
Not detected	Not detected			Cancer enhanced	Detected in many
Group enriched	Detected in all	6	central nervous system macrophage: 650.5;leukocyte: 398.7	Low cancer specificity	Detected in all
Low cell type specificity	Detected in all			Low cancer specificity	Detected in all
Low cell type specificity	Detected in all			Low cancer specificity	Detected in all
Low cell type specificity	Detected in all			Low cancer specificity	Detected in many
Low cell type specificity	Detected in all			Low cancer specificity	Detected in all
Low cell type specificity	Detected in all			Low cancer specificity	Detected in all
Low cell type specificity	Detected in all			Low cancer specificity	Detected in all
Cell type enhanced	Detected in all		oligodendrocyte: 102.4	Cancer enhanced	Detected in all
Low cell type specificity	Detected in all			Cancer enhanced	Detected in many
Cell type enhanced	Detected in all		oligodendrocyte precursor cell: 489.9	Low cancer specificity	Detected in all
Low cell type specificity	Detected in all			Low cancer specificity	Detected in all
Cell type enhanced	Detected in many		choroid plexus epithelial cell: 20.8;endothelial cell: 45.8;ependymal cell: 16.7	Low cancer specificity	Detected in many
Not detected	Not detected			Not detected	Not detected
Not detected	Not detected			Not detected	Not detected
Cell type enriched	Detected in many	5	vascular associated smooth muscle cell: 849.7	Cancer enhanced	Detected in all
Not detected	Not detected			Cancer enhanced	Detected in many
Cell type enhanced	Detected in many		thalamic excitatory: 131.9	Not detected	Not detected

Cell type enhanced	Detected in many		deep-layer intratelencephalic: 69.1;upper-layer intratelencephalic: 116.6	Not detected	Not detected
Low cell type specificity	Detected in all			Low cancer specificity	Detected in all
Cell type enhanced	Detected in all		upper rhombic lip: 125.2	Low cancer specificity	Detected in many
Cell type enriched	Detected in some	4	leukocyte: 8.9	Low cancer specificity	Detected in some
Cell type enhanced	Detected in all		astrocyte: 343.9;cerebellar inhibitory: 510.3;oligodendrocyte: 637.4	Cancer enhanced	Detected in some
Cell type enhanced	Detected in many		Bergmann glia: 214.6;central nervous system macrophage: 478.3	Low cancer specificity	Detected in all
Cell type enhanced	Detected in all		choroid plexus epithelial cell: 219.7	Low cancer specificity	Detected in all
Cell type enriched	Detected in many	34	endothelial cell: 392.2	Low cancer specificity	Detected in many
Low cell type specificity	Detected in many			Low cancer specificity	Detected in all
Low cell type specificity	Detected in all			Low cancer specificity	Detected in all
Not detected	Not detected			Cancer enhanced	Detected in many
Group enriched	Detected in some	4	central nervous system macrophage: 32.7;endothelial cell: 57.5;leukocyte: 28.3	Low cancer specificity	Detected in all
Not detected	Not detected			Cancer enriched	Detected in single
Group enriched	Detected in many	7	lower rhombic lip: 200.6;medium spiny neuron: 247.5	Low cancer specificity	Detected in many
Cell type enhanced	Detected in many		leukocyte: 15.3	Cancer enriched	Detected in many
Cell type enhanced	Detected in some		ependymal cell: 3.1;upper-layer intratelencephalic: 4.1	Low cancer specificity	Detected in some
Low cell type specificity	Detected in all			Low cancer specificity	Detected in all
Low cell type specificity	Detected in all			Low cancer specificity	Detected in many
Cell type enhanced	Detected in all		committed oligodendrocyte precursor: 337.9	Low cancer specificity	Detected in all
Cell type enhanced	Detected in many		central nervous system macrophage: 50.4;leukocyte: 48.9	Low cancer specificity	Detected in all
Group enriched	Detected in some	5	fibroblast: 26.4;vascular associated smooth muscle cell: 75.5	Low cancer specificity	Detected in all
Cell type enriched	Detected in single	99	leukocyte: 50.1	Low cancer specificity	Detected in many
Cell type enriched	Detected in single	16	central nervous system macrophage: 6.8	Cancer enhanced	Detected in many
Cell type enhanced	Detected in many		central nervous system macrophage: 212.6;endothelial cell: 342.0;leukocyte: 365.0	Low cancer specificity	Detected in all
Low cell type specificity	Detected in all			Low cancer specificity	Detected in all
Not detected	Not detected			Low cancer specificity	Detected in all
Cell type enriched	Detected in many	28	vascular associated smooth muscle cell: 1444.2	Group enriched	Detected in all
Cell type enhanced	Detected in all		astrocyte: 141.2;vascular associated smooth muscle cell: 308.4	Cancer enhanced	Detected in all
Cell type enhanced	Detected in all		choroid plexus epithelial cell: 379.0;endothelial cell: 689.7	Low cancer specificity	Detected in all
Group enriched	Detected in many	5	pericyte: 1572.8;vascular associated smooth muscle cell: 673.7	Low cancer specificity	Detected in all
Cell type enhanced	Detected in many		endothelial cell: 60.5;pericyte: 74.7;vascular associated smooth muscle cell: 86.6	Low cancer specificity	Detected in all
Cell type enriched	Detected in some	49	leukocyte: 72.2	Cancer enhanced	Detected in many
Cell type enhanced	Detected in many		central nervous system macrophage: 264.4;choroid plexus epithelial cell: 346.6;endothelial cell: 251.4;pericyte: 248.4;vascular associated smooth muscle cell: 224.5	Low cancer specificity	Detected in all
Low cell type specificity	Detected in all			Low cancer specificity	Detected in many
Low cell type specificity	Detected in all			Cancer enhanced	Detected in many
Low cell type specificity	Detected in all			Low cancer specificity	Detected in all
Low cell type specificity	Detected in all			Cancer enriched	Detected in all
Low cell type specificity	Detected in all			Group enriched	Detected in some
Group enriched	Detected in many	86	central nervous system macrophage: 334.1;leukocyte: 155.6	Low cancer specificity	Detected in all
Low cell type specificity	Detected in all			Low cancer specificity	Detected in all
Not detected	Not detected			Cancer enhanced	Detected in many
Low cell type specificity	Detected in all			Low cancer specificity	Detected in all
Low cell type specificity	Detected in all			Cancer enhanced	Detected in many
Low cell type specificity	Detected in all			Low cancer specificity	Detected in all
Low cell type specificity	Detected in all			Low cancer specificity	Detected in all
Group enriched	Detected in some	4	fibroblast: 9.4;oligodendrocyte: 11.4	Cancer enhanced	Detected in all
Low cell type specificity	Detected in all			Low cancer specificity	Detected in many
Cell type enhanced	Detected in many		endothelial cell: 56.2;pericyte: 55.5	Low cancer specificity	Detected in all
Cell type enhanced	Detected in many		choroid plexus epithelial cell: 86.6;fibroblast: 182.5	Cancer enhanced	Detected in all
Low cell type specificity	Detected in all			Low cancer specificity	Detected in all
Cell type enhanced	Detected in all		oligodendrocyte precursor cell: 148.7	Cancer enriched	Detected in some
Cell type enhanced	Detected in many		eccentric medium spiny neuron: 30.8	Not detected	Not detected
Low cell type specificity	Detected in all			Low cancer specificity	Detected in all
Cell type enhanced	Detected in some		endothelial cell: 10.0;oligodendrocyte: 28.6	Group enriched	Detected in some
Cell type enhanced	Detected in all		cerebellar inhibitory: 194.9;LAMP5-LHX6 and Chandelier: 205.1;splatter: 285.6	Low cancer specificity	Detected in some
Cell type enhanced	Detected in all		Bergmann glia: 211.1	Low cancer specificity	Detected in all
Group enriched	Detected in many	95	pericyte: 876.5;vascular associated smooth muscle cell: 608.3	Low cancer specificity	Detected in all
Cell type enriched	Detected in many	4	eccentric medium spiny neuron: 296.7	Low cancer specificity	Detected in some
Low cell type specificity	Detected in all			Low cancer specificity	Detected in all
Group enriched	Detected in many	4	astrocyte: 61.5;committed oligodendrocyte precursor: 49.2;fibroblast: 112.0;oligodendrocyte precursor cell: 93.7	Low cancer specificity	Detected in all
Cell type enhanced	Detected in all		thalamic excitatory: 2777.8	Cancer enhanced	Detected in some
Cell type enhanced	Detected in many		Bergmann glia: 126.1;deep-layer intratelencephalic: 87.4	Cancer enhanced	Detected in some
Cell type enhanced	Detected in all		astrocyte: 2595.8	Cancer enhanced	Detected in many
Cell type enhanced	Detected in many		midbrain-derived inhibitory: 15.5;miscellaneous: 19.2	Cancer enhanced	Detected in some
Low cell type specificity	Detected in all			Low cancer specificity	Detected in all
Group enriched	Detected in many	8	choroid plexus epithelial cell: 59.7;endothelial cell: 113.3	Low cancer specificity	Detected in all
Low cell type specificity	Detected in all			Low cancer specificity	Detected in all
Low cell type specificity	Detected in some			Group enriched	Detected in many
Cell type enriched	Detected in many	35	central nervous system macrophage: 466.8	Low cancer specificity	Detected in all
Low cell type specificity	Detected in all			Low cancer specificity	Detected in all
Low cell type specificity	Detected in many			Cancer enhanced	Detected in some
Not detected	Not detected			Not detected	Not detected
Not detected	Not detected			Not detected	Not detected
Not detected	Not detected			Not detected	Not detected
Not detected	Not detected			Not detected	Not detected
Not detected	Not detected			Not detected	Not detected
Low cell type specificity	Detected in all			Cancer enhanced	Detected in many
Low cell type specificity	Detected in many			Low cancer specificity	Detected in all
Cell type enhanced	Detected in some		mammillary body: 3.8	Cancer enriched	Detected in many
Low cell type specificity	Detected in all			Low cancer specificity	Detected in all
Cell type enhanced	Detected in all		central nervous system macrophage: 712.7;pericyte: 609.7	Low cancer specificity	Detected in all
Low cell type specificity	Detected in all			Low cancer specificity	Detected in all
Low cell type specificity	Detected in all			Low cancer specificity	Detected in all
Low cell type specificity	Detected in all			Low cancer specificity	Detected in all
Low cell type specificity	Detected in all			Low cancer specificity	Detected in all
Low cell type specificity	Detected in all			Low cancer specificity	Detected in all
Cell type enhanced	Detected in all		choroid plexus epithelial cell: 268.1	Low cancer specificity	Detected in all
Low cell type specificity	Detected in many			Low cancer specificity	Detected in all
Low cell type specificity	Detected in many			Low cancer specificity	Detected in many
Cell type enhanced	Detected in some		Bergmann glia: 1.5;oligodendrocyte: 1.7	Low cancer specificity	Detected in all
Cell type enhanced	Detected in all		pericyte: 2229.7	Low cancer specificity	Detected in all
Cell type enhanced	Detected in all		ependymal cell: 2249.2	Low cancer specificity	Detected in all
Low cell type specificity	Detected in all			Low cancer specificity	Detected in all
Cell type enhanced	Detected in all		endothelial cell: 188.8	Group enriched	Detected in all
Cell type enhanced	Detected in all		upper rhombic lip: 969.6	Low cancer specificity	Detected in all
Low cell type specificity	Detected in many			Low cancer specificity	Detected in all
Low cell type specificity	Detected in all			Low cancer specificity	Detected in many
Low cell type specificity	Detected in many			Low cancer specificity	Detected in many
Cell type enhanced	Detected in some		oligodendrocyte precursor cell: 2.1	Cancer enriched	Detected in many
Low cell type specificity	Detected in all			Low cancer specificity	Detected in all
Low cell type specificity	Detected in all			Low cancer specificity	Detected in all
Low cell type specificity	Detected in all			Low cancer specificity	Detected in all
Low cell type specificity	Detected in many			Cancer enriched	Detected in all
Low cell type specificity	Detected in all			Low cancer specificity	Detected in many
Group enriched	Detected in some	13	fibroblast: 133.8;oligodendrocyte precursor cell: 320.9	Cancer enhanced	Detected in all
Group enriched	Detected in many	6	astrocyte: 19.5;Bergmann glia: 26.1;central nervous system macrophage: 31.6;choroid plexus epithelial cell: 40.0;ependymal cell: 24.2;fibroblast: 21.6;oligodendrocyte precursor cell: 40.4	Cancer enhanced	Detected in all
Cell type enhanced	Detected in all		choroid plexus epithelial cell: 2518.2	Low cancer specificity	Detected in many
Cell type enhanced	Detected in many		central nervous system macrophage: 140.9;endothelial cell: 446.3;leukocyte: 107.9	Cancer enhanced	Detected in all
Cell type enhanced	Detected in many		astrocyte: 32.1;Bergmann glia: 32.5;ependymal cell: 67.3	Cancer enriched	Detected in some
Cell type enhanced	Detected in many		fibroblast: 328.1;pericyte: 431.3;vascular associated smooth muscle cell: 523.8	Low cancer specificity	Detected in all
Low cell type specificity	Detected in all			Low cancer specificity	Detected in all
Group enriched	Detected in some	20	central nervous system macrophage: 97.0;leukocyte: 90.7	Low cancer specificity	Detected in many
Low cell type specificity	Detected in all			Low cancer specificity	Detected in all
Low cell type specificity	Detected in all			Low cancer specificity	Detected in all
Group enriched	Detected in many	4	CGE interneuron: 136.5;fibroblast: 159.9;hippocampal CA1-3: 52.8;hippocampal CA4: 66.0;LAMP5-LHX6 and Chandelier: 179.7	Low cancer specificity	Detected in many
Not detected	Not detected			Low cancer specificity	Detected in many
Cell type enhanced	Detected in all		committed oligodendrocyte precursor: 2317.7	Low cancer specificity	Detected in all
Low cell type specificity	Detected in all			Low cancer specificity	Detected in all
Low cell type specificity	Detected in all			Low cancer specificity	Detected in all
Cell type enhanced	Detected in all		central nervous system macrophage: 39.5;leukocyte: 49.2	Low cancer specificity	Detected in all
Cell type enhanced	Detected in all		pericyte: 1570.4	Cancer enhanced	Detected in many
Low cell type specificity	Detected in all			Low cancer specificity	Detected in all
Group enriched	Detected in all	4	astrocyte: 194.4;Bergmann glia: 195.6;choroid plexus epithelial cell: 484.7;ependymal cell: 620.6	Low cancer specificity	Detected in all
Cell type enhanced	Detected in single		endothelial cell: 1.1	Low cancer specificity	Detected in many
Cell type enhanced	Detected in single		leukocyte: 1.8	Low cancer specificity	Detected in many
Group enriched	Detected in some	4	astrocyte: 21.7;Bergmann glia: 6.0;ependymal cell: 6.1	Cancer enhanced	Detected in many
Cell type enhanced	Detected in all		endothelial cell: 1275.2	Group enriched	Detected in all
Cell type enhanced	Detected in many		committed oligodendrocyte precursor: 30.3;endothelial cell: 24.2;oligodendrocyte: 97.4	Low cancer specificity	Detected in all
Cell type enhanced	Detected in many		central nervous system macrophage: 138.4;endothelial cell: 201.9;pericyte: 148.7	Low cancer specificity	Detected in all

Cell type enriched	Detected in all	8	endothelial cell: 580.7	Low cancer specificity	Detected in all
Low cell type specificity	Detected in all			Low cancer specificity	Detected in all
Cell type enhanced	Detected in all		endothelial cell: 1073.5;fibroblast: 894.3	Low cancer specificity	Detected in all
Low cell type specificity	Detected in all			Low cancer specificity	Detected in all
Cell type enhanced	Detected in all		committed oligodendrocyte precursor: 626.3	Low cancer specificity	Detected in all
Low cell type specificity	Detected in many			Low cancer specificity	Detected in all
Low cell type specificity	Detected in all			Low cancer specificity	Detected in all
Not detected	Not detected			Cancer enhanced	Detected in many
Low cell type specificity	Detected in all			Low cancer specificity	Detected in all
Low cell type specificity	Detected in all			Low cancer specificity	Detected in all
Low cell type specificity	Detected in all			Low cancer specificity	Detected in many
Cell type enhanced	Detected in all		endothelial cell: 225.7	Low cancer specificity	Detected in all
Low cell type specificity	Detected in all			Low cancer specificity	Detected in all
Low cell type specificity	Detected in all			Cancer enhanced	Detected in many
Low cell type specificity	Detected in some			Cancer enriched	Detected in many
Low cell type specificity	Detected in all			Low cancer specificity	Detected in all
Low cell type specificity	Detected in all			Low cancer specificity	Detected in all
Cell type enriched	Detected in many	9	leukocyte: 79.9	Low cancer specificity	Detected in many
Cell type enriched	Detected in many	10	central nervous system macrophage: 142.7	Low cancer specificity	Detected in all
Cell type enhanced	Detected in all		Bergmann glia: 220.2	Cancer enhanced	Detected in many
Cell type enriched	Detected in many	6	pericyte: 798.2	Cancer enriched	Detected in many
Low cell type specificity	Detected in all			Low cancer specificity	Detected in all
Cell type enhanced	Detected in all		choroid plexus epithelial cell: 104.3;fibroblast: 99.5	Low cancer specificity	Detected in all
Cell type enhanced	Detected in some		oligodendrocyte: 6.6	Cancer enhanced	Detected in many
Cell type enhanced	Detected in all		central nervous system macrophage: 1249.8	Low cancer specificity	Detected in all
Cell type enhanced	Detected in all		pericyte: 3521.5	Low cancer specificity	Detected in all
Cell type enhanced	Detected in all		endothelial cell: 1825.7	Low cancer specificity	Detected in all
Low cell type specificity	Detected in all			Low cancer specificity	Detected in many
Low cell type specificity	Detected in all			Low cancer specificity	Detected in all
Low cell type specificity	Detected in many			Low cancer specificity	Detected in all
Group enriched	Detected in all	4	committed oligodendrocyte precursor: 356.6;oligodendrocyte: 1093.6	Cancer enhanced	Detected in all
Not detected	Not detected			Low cancer specificity	Detected in all
Low cell type specificity	Detected in all			Low cancer specificity	Detected in all
Not detected	Not detected			Low cancer specificity	Detected in many
Low cell type specificity	Detected in all			Low cancer specificity	Detected in all
Cell type enhanced	Detected in single		endothelial cell: 1.4	Cancer enhanced	Detected in many
Not detected	Not detected			Group enriched	Detected in many
Low cell type specificity	Detected in all			Low cancer specificity	Detected in all
Low cell type specificity	Detected in all			Low cancer specificity	Detected in all
Low cell type specificity	Detected in all			Low cancer specificity	Detected in all
Cell type enriched	Detected in many	6	endothelial cell: 143.3	Low cancer specificity	Detected in all
Cell type enhanced	Detected in all		endothelial cell: 1728.4	Low cancer specificity	Detected in all
Low cell type specificity	Detected in all			Low cancer specificity	Detected in all
Group enriched	Detected in some	115	central nervous system macrophage: 70.8;leukocyte: 97.9	Low cancer specificity	Detected in all
Cell type enhanced	Detected in all		eccentric medium spiny neuron: 296.2;leukocyte: 303.0	Low cancer specificity	Detected in many
Cell type enhanced	Detected in all		pericyte: 98.7;vascular associated smooth muscle cell: 79.8	Cancer enhanced	Detected in all
Group enriched	Detected in some	10	central nervous system macrophage: 17.1;leukocyte: 5.3	Cancer enhanced	Detected in some
Low cell type specificity	Detected in all			Low cancer specificity	Detected in all
Low cell type specificity	Detected in all			Low cancer specificity	Detected in all
Low cell type specificity	Detected in all			Low cancer specificity	Detected in all
Low cell type specificity	Detected in all			Low cancer specificity	Detected in all
Cell type enhanced	Detected in many		endothelial cell: 159.3;pericyte: 180.9;vascular associated smooth muscle cell: 155.2	Low cancer specificity	Detected in all
Low cell type specificity	Detected in all			Low cancer specificity	Detected in all
Cell type enhanced	Detected in many		lower rhombic lip: 23.0	Cancer enhanced	Detected in many
Group enriched	Detected in many	6	central nervous system macrophage: 74.2;leukocyte: 46.4	Low cancer specificity	Detected in all
Cell type enhanced	Detected in all		endothelial cell: 136.1;ependymal cell: 88.5	Low cancer specificity	Detected in all
Cell type enhanced	Detected in single		leukocyte: 2.0	Cancer enhanced	Detected in many
Low cell type specificity	Detected in all			Low cancer specificity	Detected in all
Cell type enhanced	Detected in all		endothelial cell: 162.8	Low cancer specificity	Detected in all
Cell type enhanced	Detected in many		endothelial cell: 39.6	Cancer enhanced	Detected in many
Low cell type specificity	Detected in all			Low cancer specificity	Detected in all
Low cell type specificity	Detected in all			Low cancer specificity	Detected in all
Low cell type specificity	Detected in all			Low cancer specificity	Detected in all
Cell type enhanced	Detected in all		leukocyte: 437.1	Low cancer specificity	Detected in all
Low cell type specificity	Detected in all			Low cancer specificity	Detected in all
Low cell type specificity	Detected in some			Cancer enhanced	Detected in some
Low cell type specificity	Detected in many			Low cancer specificity	Detected in all
Low cell type specificity	Detected in many			Cancer enhanced	Detected in many
Not detected	Not detected			Group enriched	Detected in all
Cell type enhanced	Detected in all		leukocyte: 299.6;vascular associated smooth muscle cell: 612.4	Low cancer specificity	Detected in all
Cell type enriched	Detected in single	57	leukocyte: 20.6	Cancer enhanced	Detected in all
Cell type enhanced	Detected in some		Bergmann glia: 3.3;cerebellar inhibitory: 4.4;oligodendrocyte: 4.9	Cancer enhanced	Detected in many
Low cell type specificity	Detected in all			Cancer enriched	Detected in single
Low cell type specificity	Detected in many			Cancer enriched	Detected in some
Cell type enhanced	Detected in many		eccentric medium spiny neuron: 37.8;fibroblast: 22.6	Low cancer specificity	Detected in some
Low cell type specificity	Detected in all			Low cancer specificity	Detected in some
Low cell type specificity	Detected in all			Low cancer specificity	Detected in many
Cell type enhanced	Detected in some		hippocampal dentate gyrus: 9.7;upper rhombic lip: 12.1	Cancer enhanced	Detected in many
Cell type enhanced	Detected in all		astrocyte: 52.2	Low cancer specificity	Detected in all
Cell type enhanced	Detected in all		LAMP5-LHX6 and Chandelier: 30.8	Cancer enhanced	Detected in many
Cell type enhanced	Detected in all		Bergmann glia: 209.3	Low cancer specificity	Detected in all
Low cell type specificity	Detected in all			Low cancer specificity	Detected in all
Group enriched	Detected in all	4	central nervous system macrophage: 286.4;committed oligodendrocyte precursor: 512.8;leukocyte: 361.0;oligodendrocyte: 549.5	Low cancer specificity	Detected in all
Cell type enhanced	Detected in all		committed oligodendrocyte precursor: 623.8	Low cancer specificity	Detected in all
Low cell type specificity	Detected in many			Cancer enhanced	Detected in some
Cell type enriched	Detected in some	6	central nervous system macrophage: 69.5	Low cancer specificity	Detected in all
Not detected	Not detected			Low cancer specificity	Detected in many
Low cell type specificity	Detected in all			Low cancer specificity	Detected in all
Cell type enhanced	Detected in all		cerebellar inhibitory: 750.3	Low cancer specificity	Detected in all
Low cell type specificity	Detected in all			Low cancer specificity	Detected in all
Group enriched	Detected in some	4	astrocyte: 36.0;Bergmann glia: 28.8	Cancer enriched	Detected in many
Cell type enriched	Detected in many	9	astrocyte: 756.9	Cancer enhanced	Detected in many
Cell type enhanced	Detected in all		endothelial cell: 178.6	Low cancer specificity	Detected in all
Cell type enhanced	Detected in all		choroid plexus epithelial cell: 788.2	Group enriched	Detected in all
Cell type enhanced	Detected in all		central nervous system macrophage: 45.2;leukocyte: 34.6	Low cancer specificity	Detected in all
Low cell type specificity	Detected in all			Cancer enriched	Detected in many
Low cell type specificity	Detected in all			Cancer enhanced	Detected in all
Cell type enhanced	Detected in some		central nervous system macrophage: 30.1;leukocyte: 12.9	Low cancer specificity	Detected in all
Low cell type specificity	Detected in many			Cancer enriched	Detected in many
Low cell type specificity	Detected in all			Low cancer specificity	Detected in all
Cell type enhanced	Detected in all		oligodendrocyte: 4299.7	Group enriched	Detected in some
Low cell type specificity	Detected in all			Low cancer specificity	Detected in all
Cell type enhanced	Detected in some		astrocyte: 7.2;ependymal cell: 6.0	Cancer enhanced	Detected in some
Cell type enriched	Detected in all	8	endothelial cell: 678.3	Low cancer specificity	Detected in all
Cell type enriched	Detected in many	10	choroid plexus epithelial cell: 843.7	Cancer enriched	Detected in many
Cell type enhanced	Detected in many		hippocampal CA4: 19.3	Cancer enriched	Detected in many
Cell type enhanced	Detected in some		choroid plexus epithelial cell: 3.6;fibroblast: 4.6;oligodendrocyte: 6.1	Cancer enriched	Detected in some
Cell type enhanced	Detected in all		endothelial cell: 423.8;leukocyte: 381.3	Cancer enhanced	Detected in all
Cell type enriched	Detected in many	33	central nervous system macrophage: 213.3	Group enriched	Detected in many
Cell type enriched	Detected in all	6	endothelial cell: 224.4	Low cancer specificity	Detected in all
Cell type enhanced	Detected in many		lower rhombic lip: 28.0	Group enriched	Detected in some
Not detected	Not detected			Group enriched	Detected in some
Cell type enriched	Detected in single	19	choroid plexus epithelial cell: 8.7	Cancer enhanced	Detected in many
Low cell type specificity	Detected in all			Cancer enhanced	Detected in all
Low cell type specificity	Detected in all			Low cancer specificity	Detected in all
Low cell type specificity	Detected in all			Cancer enhanced	Detected in all
Cell type enhanced	Detected in all		endothelial cell: 215.8	Low cancer specificity	Detected in all
Cell type enhanced	Detected in many		central nervous system macrophage: 73.7;fibroblast: 99.8;pericyte: 129.1	Low cancer specificity	Detected in all
Low cell type specificity	Detected in all			Low cancer specificity	Detected in all
Low cell type specificity	Detected in all			Low cancer specificity	Detected in all
Cell type enriched	Detected in many	12	choroid plexus epithelial cell: 368.4	Low cancer specificity	Detected in all
Cell type enhanced	Detected in all		astrocyte: 1523.4;Bergmann glia: 4574.7	Group enriched	Detected in many
Cell type enhanced	Detected in all		central nervous system macrophage: 787.9	Low cancer specificity	Detected in all
Low cell type specificity	Detected in some			Cancer enhanced	Detected in many
Cell type enhanced	Detected in some		astrocyte: 6.4;committed oligodendrocyte precursor: 5.8	Cancer enriched	Detected in some
Cell type enhanced	Detected in many		choroid plexus epithelial cell: 8.0;endothelial cell: 8.8	Cancer enhanced	Detected in all
Cell type enhanced	Detected in many		astrocyte: 94.1;fibroblast: 129.3	Cancer enhanced	Detected in many
Cell type enriched	Detected in all	24	endothelial cell: 994.5	Cancer enhanced	Detected in all
Low cell type specificity	Detected in all			Low cancer specificity	Detected in all

Low cell type specificity	Detected in all			Low cancer specificity	Detected in many
Cell type enhanced	Detected in all		pericyte: 109.2	Low cancer specificity	Detected in all
Cell type enhanced	Detected in many		endothelial cell: 56.7;hippocampal CA4: 53.0	Cancer enhanced	Detected in many
Cell type enhanced	Detected in some		mammillary body: 4.7	Low cancer specificity	Detected in all
Cell type enriched	Detected in all	4	endothelial cell: 252.0	Low cancer specificity	Detected in all
Low cell type specificity	Detected in all			Low cancer specificity	Detected in all
Cell type enriched	Detected in many	4	ependymal cell: 336.6	Not detected	Not detected
Group enriched	Detected in many	4	endothelial cell: 830.6;oligodendrocyte: 712.7	Cancer enhanced	Detected in many
Group enriched	Detected in some	4	choroid plexus epithelial cell: 4.8;endothelial cell: 7.9;oligodendrocyte: 18.1	Cancer enriched	Detected in single
Cell type enriched	Detected in single	24	choroid plexus epithelial cell: 12.4	Cancer enhanced	Detected in many
Cell type enriched	Detected in many	6	endothelial cell: 70.6	Low cancer specificity	Detected in all
Cell type enhanced	Detected in many		LAMP5-LHX6 and Chandelier: 208.6;midbrain-derived inhibitory: 87.9	Cancer enhanced	Detected in some
Cell type enhanced	Detected in all		vascular associated smooth muscle cell: 1918.0	Cancer enhanced	Detected in all
Cell type enhanced	Detected in some		medium spiny neuron: 19.8;thalamic excitatory: 34.0	Cancer enhanced	Detected in many
Cell type enhanced	Detected in many		endothelial cell: 15.1;fibroblast: 11.5	Low cancer specificity	Detected in all
Low cell type specificity	Detected in all			Low cancer specificity	Detected in all
Low cell type specificity	Detected in all			Low cancer specificity	Detected in all
Cell type enhanced	Detected in all		hippocampal CA4: 303.9;lower rhombic lip: 283.5	Not detected	Not detected
Low cell type specificity	Detected in all			Low cancer specificity	Detected in all
Cell type enhanced	Detected in all		astrocyte: 2090.0	Low cancer specificity	Detected in all
Cell type enhanced	Detected in all		oligodendrocyte: 865.7	Low cancer specificity	Detected in all
Low cell type specificity	Detected in all			Low cancer specificity	Detected in all
Cell type enhanced	Detected in many		lower rhombic lip: 9.2;vascular associated smooth muscle cell: 8.2	Low cancer specificity	Detected in all
Cell type enhanced	Detected in some		central nervous system macrophage: 6.8;leukocyte: 5.3;upper rhombic lip: 6.0	Low cancer specificity	Detected in all
Low cell type specificity	Detected in all			Cancer enhanced	Detected in all
Low cell type specificity	Detected in all			Low cancer specificity	Detected in all
Low cell type specificity	Detected in all			Low cancer specificity	Detected in all
Low cell type specificity	Detected in many			Low cancer specificity	Detected in all
Not detected	Not detected			Low cancer specificity	Detected in many
Low cell type specificity	Detected in all			Low cancer specificity	Detected in all
Cell type enriched	Detected in many	4	fibroblast: 39.7	Cancer enhanced	Detected in many
Cell type enhanced	Detected in all		central nervous system macrophage: 173.1;leukocyte: 481.1	Low cancer specificity	Detected in all
Cell type enhanced	Detected in many		endothelial cell: 560.8;pericyte: 212.8;vascular associated smooth muscle cell: 269.3	Low cancer specificity	Detected in all
Low cell type specificity	Detected in all			Low cancer specificity	Detected in all
Cell type enriched	Detected in some	54	ependymal cell: 90.3	Cancer enhanced	Detected in some
Low cell type specificity	Detected in all			Low cancer specificity	Detected in all
Cell type enhanced	Detected in all		fibroblast: 948.5;vascular associated smooth muscle cell: 533.9	Low cancer specificity	Detected in all
Cell type enhanced	Detected in many		central nervous system macrophage: 299.4;endothelial cell: 253.8	Low cancer specificity	Detected in all
Low cell type specificity	Detected in all			Low cancer specificity	Detected in all
Cell type enhanced	Detected in all		vascular associated smooth muscle cell: 362.5	Low cancer specificity	Detected in all
Low cell type specificity	Detected in all			Low cancer specificity	Detected in all
Cell type enhanced	Detected in all		eccentric medium spiny neuron: 156.4;LAMP5-LHX6 and Chandelier: 305.0;splatter: 155.2	Low cancer specificity	Detected in some
Cell type enhanced	Detected in single		hippocampal CA4: 1.9	Cancer enhanced	Detected in many
Not detected	Not detected			Low cancer specificity	Detected in many
Not detected	Not detected			Low cancer specificity	Detected in many
Cell type enhanced	Detected in all		leukocyte: 42.8	Low cancer specificity	Detected in all
Low cell type specificity	Detected in all			Cancer enhanced	Detected in all
Low cell type specificity	Detected in all			Low cancer specificity	Detected in many
Cell type enriched	Detected in some	4	pericyte: 43.3	Low cancer specificity	Detected in all
Cell type enhanced	Detected in many		leukocyte: 6.7	Low cancer specificity	Detected in all
				Not detected	Not detected
Cell type enhanced	Detected in many		endothelial cell: 50.6	Low cancer specificity	Detected in many
Group enriched	Detected in all	8	endothelial cell: 417.7;oligodendrocyte precursor cell: 143.8	Cancer enhanced	Detected in all
Cell type enhanced	Detected in all		central nervous system macrophage: 557.5;leukocyte: 233.6	Low cancer specificity	Detected in all
Group enriched	Detected in many	9	central nervous system macrophage: 646.8;choroid plexus epithelial cell: 610.3;endothelial cell: 572.4;fibroblast: 371.1;leukocyte: 407.5;pericyte: 296.7;vascular associated smooth muscle cell: 163.8	Low cancer specificity	Detected in all
Cell type enhanced	Detected in all		upper rhombic lip: 2286.5	Cancer enhanced	Detected in all
Low cell type specificity	Detected in all			Low cancer specificity	Detected in many
Low cell type specificity	Detected in all			Low cancer specificity	Detected in all
Cell type enhanced	Detected in all		astrocyte: 211.9;Bergmann glia: 370.8	Low cancer specificity	Detected in all
Cell type enriched	Detected in many	4	choroid plexus epithelial cell: 24.5	Low cancer specificity	Detected in many
Cell type enhanced	Detected in all		vascular associated smooth muscle cell: 215.3	Low cancer specificity	Detected in all
Cell type enriched	Detected in some	5	central nervous system macrophage: 299.3	Low cancer specificity	Detected in all
Cell type enhanced	Detected in single		central nervous system macrophage: 1.0	Low cancer specificity	Detected in all
Low cell type specificity	Detected in some			Group enriched	Detected in many
Cell type enhanced	Detected in single		oligodendrocyte: 1.2	Not detected	Not detected
Cell type enhanced	Detected in all		upper rhombic lip: 291.2	Not detected	Not detected
Low cell type specificity	Detected in all			Low cancer specificity	Detected in all
Low cell type specificity	Detected in all			Low cancer specificity	Detected in all
Cell type enriched	Detected in single	9	choroid plexus epithelial cell: 6.5	Cancer enriched	Detected in many
Cell type enhanced	Detected in many		central nervous system macrophage: 61.8	Cancer enhanced	Detected in many
Cell type enhanced	Detected in many		endothelial cell: 54.1;leukocyte: 56.5	Low cancer specificity	Detected in all
Cell type enhanced	Detected in many		committed oligodendrocyte precursor: 280.3	Low cancer specificity	Detected in all
Cell type enhanced	Detected in some		leukocyte: 6.9;medium spiny neuron: 5.9	Cancer enriched	Detected in some
Low cell type specificity	Detected in all			Low cancer specificity	Detected in all
Cell type enhanced	Detected in all		committed oligodendrocyte precursor: 301.5;oligodendrocyte precursor cell: 409.0	Low cancer specificity	Detected in all
Cell type enhanced	Detected in all		astrocyte: 121.7	Low cancer specificity	Detected in all
Group enriched	Detected in some	6	fibroblast: 54.4;vascular associated smooth muscle cell: 173.3	Group enriched	Detected in many
Cell type enhanced	Detected in many		medium spiny neuron: 16.6	Low cancer specificity	Detected in all
Cell type enriched	Detected in single	67	leukocyte: 56.0	Low cancer specificity	Detected in many
Cell type enriched	Detected in many	8	ependymal cell: 549.5	Cancer enhanced	Detected in some
Cell type enriched	Detected in single	42	central nervous system macrophage: 32.8	Cancer enhanced	Detected in some
Low cell type specificity	Detected in many			Cancer enriched	Detected in all
Cell type enhanced	Detected in many		central nervous system macrophage: 20.1;leukocyte: 37.1	Cancer enriched	Detected in all
Cell type enhanced	Detected in many		committed oligodendrocyte precursor: 110.3;oligodendrocyte: 150.6;upper rhombic lip: 71.7	Low cancer specificity	Detected in all
Not detected	Not detected			Cancer enhanced	Detected in some
Low cell type specificity	Detected in all			Low cancer specificity	Detected in all
Low cell type specificity	Detected in all			Cancer enhanced	Detected in all
Cell type enhanced	Detected in many		leukocyte: 251.1;lower rhombic lip: 85.6	Low cancer specificity	Detected in some
Low cell type specificity	Detected in all			Low cancer specificity	Detected in all
Group enriched	Detected in some	41	central nervous system macrophage: 253.3;leukocyte: 106.7	Low cancer specificity	Detected in all
Not detected	Not detected			Low cancer specificity	Detected in all
Low cell type specificity	Detected in all			Low cancer specificity	Detected in all
Cell type enhanced	Detected in some		hippocampal CA4: 2.7;hippocampal dentate gyrus: 1.7;upper rhombic lip: 1.1	Low cancer specificity	Detected in some
Cell type enhanced	Detected in many		lower rhombic lip: 7.5	Cancer enhanced	Detected in many
Low cell type specificity	Detected in all			Low cancer specificity	Detected in all
Cell type enriched	Detected in single	4	ependymal cell: 1.5	Low cancer specificity	Detected in some
Cell type enhanced	Detected in all		endothelial cell: 1762.0;pericyte: 1749.0;vascular associated smooth muscle cell: 2274.1	Low cancer specificity	Detected in all
Cell type enhanced	Detected in all		leukocyte: 56.3;pericyte: 44.7;vascular associated smooth muscle cell: 51.0	Low cancer specificity	Detected in all
Low cell type specificity	Detected in many			Group enriched	Detected in many
Low cell type specificity	Detected in all			Low cancer specificity	Detected in all
Cell type enhanced	Detected in all		vascular associated smooth muscle cell: 128.8	Low cancer specificity	Detected in all
Low cell type specificity	Detected in all			Low cancer specificity	Detected in all
Low cell type specificity	Detected in all			Low cancer specificity	Detected in all
Group enriched	Detected in many	4	endothelial cell: 298.3;leukocyte: 168.4	Low cancer specificity	Detected in all
Cell type enhanced	Detected in all		astrocyte: 45.6	Low cancer specificity	Detected in all
Low cell type specificity	Detected in all			Low cancer specificity	Detected in all
Low cell type specificity	Detected in all			Low cancer specificity	Detected in all
Cell type enhanced	Detected in all		oligodendrocyte: 724.3	Low cancer specificity	Detected in all
Low cell type specificity	Detected in many			Cancer enriched	Detected in single
Low cell type specificity	Detected in all			Low cancer specificity	Detected in all
Cell type enhanced	Detected in many		choroid plexus epithelial cell: 16.3	Cancer enhanced	Detected in all
Low cell type specificity	Detected in some			Low cancer specificity	Detected in all

RNA cancer specificity score	RNA cancer specific pTPM	RNA brain regional specificity	RNA brain regional distribution	RNA brain regional specificity score	RNA brain regional specific nTPM	RNA blood cell specificity	RNA blood cell distribution
		Low region specificity	Detected in all			Low immune cell specificity	Detected in many
		Low region specificity	Detected in all			Low immune cell specificity	Detected in all
		Low region specificity	Detected in all			Group enriched	Detected in many
		Low region specificity	Detected in all			Low immune cell specificity	Detected in many
	Kidney Renal Papillary Cell Carcinoma (TCGA): 35.2	Low region specificity	Detected in all			Immune cell enhanced	Detected in many
10	Liver Hepatocellular Carcinoma (TCGA): 36.5	Low region specificity	Detected in many			Group enriched	Detected in some
		Low region specificity	Detected in all			Low immune cell specificity	Detected in some
		Low region specificity	Detected in all			Not detected in immune cells	Not detected
		Low region specificity	Detected in all			Immune cell enhanced	Detected in some
6	Prostate Adenocarcinoma (TCGA): 167.6	Low region specificity	Detected in all			Low immune cell specificity	Detected in many
	Liver Hepatocellular Carcinoma (TCGA): 49.1	Low region specificity	Detected in many			Immune cell enriched	Detected in single
	Glioblastoma Multiforme (TCGA): 17.5	Low region specificity	Detected in all			Immune cell enhanced	Detected in some
		Low region specificity	Detected in all			Low immune cell specificity	Detected in many
		Low region specificity	Detected in all			Group enriched	Detected in many
		Low region specificity	Detected in all			Low immune cell specificity	Detected in all
	Breast Invasive Carcinoma (TCGA): 24.4	Low region specificity	Detected in all			Immune cell enriched	Detected in single
	Pancreatic Adenocarcinoma (TCGA): 34.9	Low region specificity	Detected in all			Immune cell enhanced	Detected in many
		Low region specificity	Detected in all			Low immune cell specificity	Detected in many
		Low region specificity	Detected in all			Immune cell enriched	Detected in single
		Low region specificity	Detected in all			Not detected in immune cells	Not detected
		Low region specificity	Detected in all			Low immune cell specificity	Detected in all
5	Glioblastoma Multiforme (TCGA): 16.6;Testicular Germ Cell Tumor (TCGA): 40.2	Low region specificity	Detected in all			Immune cell enhanced	Detected in some
		Low region specificity	Detected in all			Low immune cell specificity	Detected in all
		Low region specificity	Detected in all			Not detected in immune cells	Not detected
4	Glioblastoma Multiforme (TCGA): 40.9	Low region specificity	Detected in all			Not detected in immune cells	Not detected
	Lung Adenocarcinoma (TCGA): 11.4	Low region specificity	Detected in all			Group enriched	Detected in some
	Ovary Serous Cystadenocarcinoma (TCGA): 14.8	Low region specificity	Detected in many			Not detected in immune cells	Not detected
	Glioblastoma Multiforme (TCGA): 66.0	Low region specificity	Detected in all			Not detected in immune cells	Not detected
		Low region specificity	Detected in all			Immune cell enhanced	Detected in many
	Kidney Renal Clear Cell Carcinoma (TCGA): 7.4	Low region specificity	Detected in all			Not detected in immune cells	Not detected
	Prostate Adenocarcinoma (TCGA): 22.3	Low region specificity	Detected in all			Low immune cell specificity	Detected in all
		Low region specificity	Detected in all			Low immune cell specificity	Detected in single
11	Lung Adenocarcinoma (TCGA): 20.0;Lung Squamous Cell Carcinoma (TCGA): 9.8;Thyroid Carcinoma (TCGA): 12.1	Not detected	Not detected			Not detected in immune cells	Not detected
		Low region specificity	Detected in all			Low immune cell specificity	Detected in many
		Low region specificity	Detected in all			Not detected in immune cells	Not detected
	Testicular Germ Cell Tumor (TCGA): 70.8	Low region specificity	Detected in all			Immune cell enriched	Detected in single
	Liver Hepatocellular Carcinoma (TCGA): 484.3;Lung Squamous Cell Carcinoma (TCGA): 382.5	Low region specificity	Detected in many			Not detected in immune cells	Not detected
		Low region specificity	Detected in all			Not detected in immune cells	Not detected
		Low region specificity	Detected in all			Low immune cell specificity	Detected in all
4	Glioblastoma Multiforme (TCGA): 2.1;Ovary Serous Cystadenocarcinoma (TCGA): 1.2;Skin Cutaneous Melanoma (TCGA): 2.9;Thyroid Carcinoma (TCGA): 1.8	Low region specificity	Detected in all			Not detected in immune cells	Not detected
4	Prostate Adenocarcinoma (TCGA): 140.1	Low region specificity	Detected in many			Group enriched	Detected in some
		Low region specificity	Detected in all			Not detected in immune cells	Not detected
20	Glioblastoma Multiforme (TCGA): 8.4	Low region specificity	Detected in all			Not detected in immune cells	Not detected
		Low region specificity	Detected in all			Not detected in immune cells	Not detected
	Glioblastoma Multiforme (TCGA): 51.3	Low region specificity	Detected in all			Not detected in immune cells	Not detected
		Low region specificity	Detected in all			Low immune cell specificity	Detected in many
		Low region specificity	Detected in all			Low immune cell specificity	Detected in many
		Low region specificity	Detected in all			Low immune cell specificity	Detected in all
		Low region specificity	Detected in all			Low immune cell specificity	Detected in many
		Low region specificity	Detected in all			Not detected in immune cells	Not detected
		Low region specificity	Detected in many			Not detected in immune cells	Not detected
		Low region specificity	Detected in all			Low immune cell specificity	Detected in all
	Testicular Germ Cell Tumor (TCGA): 8.7	Low region specificity	Detected in all			Immune cell enriched	Detected in single
		Low region specificity	Detected in all			Low immune cell specificity	Detected in many
	Kidney Renal Papillary Cell Carcinoma (TCGA): 311.1	Group enriched	Detected in many	5	cerebral cortex: 19.2;thalamus: 38.8	Immune cell enriched	Detected in many
		Low region specificity	Detected in all			Immune cell enhanced	Detected in all
		Low region specificity	Detected in many			Not detected in immune cells	Not detected
		Low region specificity	Detected in all			Immune cell enriched	Detected in single
		Low region specificity	Detected in all			Low immune cell specificity	Detected in all
		Low region specificity	Detected in all			Immune cell enhanced	Detected in many
	Kidney Renal Papillary Cell Carcinoma (TCGA): 42.2	Low region specificity	Detected in all			Immune cell enhanced	Detected in all
		Low region specificity	Detected in all			Low immune cell specificity	Detected in all
		Low region specificity	Detected in all			Low immune cell specificity	Detected in many
	Kidney Renal Clear Cell Carcinoma (TCGA): 88.6	Low region specificity	Detected in all			Low immune cell specificity	Detected in some
		Low region specificity	Detected in all			Immune cell enhanced	Detected in all
	Glioblastoma Multiforme (TCGA): 577.9;Kidney Renal Papillary Cell Carcinoma (TCGA): 736.6	Low region specificity	Detected in all			Not detected in immune cells	Not detected
		Not detected	Not detected			Not detected in immune cells	Not detected
		Low region specificity	Detected in all			Not detected in immune cells	Not detected
5	Bladder Urothelial Carcinoma (TCGA): 524.5;Cervical Squamous Cell Carcinoma and Endocervical Adenocarcinoma (TCGA): 859.9;Head and Neck Squamous	Region enhanced	Detected in all		pons: 47.2	Immune cell enhanced	Detected in many
16	Glioblastoma Multiforme (TCGA): 397.9	Low region specificity	Detected in all			Not detected in immune cells	Not detected
		Low region specificity	Detected in all			Low immune cell specificity	Detected in all
		Low region specificity	Detected in all			Low immune cell specificity	Detected in all
		Low region specificity	Detected in all			Low immune cell specificity	Detected in all
		Low region specificity	Detected in all			Low immune cell specificity	Detected in all
		Low region specificity	Detected in all			Not detected in immune cells	Not detected
		Low region specificity	Detected in all			Immune cell enhanced	Detected in single
		Low region specificity	Detected in all			Low immune cell specificity	Detected in all
		Low region specificity	Detected in all			Low immune cell specificity	Detected in all
		Low region specificity	Detected in all			Not detected in immune cells	Not detected
		Low region specificity	Detected in all			Not detected in immune cells	Not detected
		Low region specificity	Detected in all			Low immune cell specificity	Detected in many
	Glioblastoma Multiforme (TCGA): 12.7	Low region specificity	Detected in all			Not detected in immune cells	Not detected
		Low region specificity	Detected in all			Immune cell enhanced	Detected in single
		Low region specificity	Detected in all			Low immune cell specificity	Detected in many
		Low region specificity	Detected in all			Immune cell enhanced	Detected in many
		Low region specificity	Detected in all			Low immune cell specificity	Detected in all
		Low region specificity	Detected in all			Group enriched	Detected in many
		Low region specificity	Detected in all			Not detected in immune cells	Not detected
		Low region specificity	Detected in all			Immune cell enriched	Detected in single
37	Liver Hepatocellular Carcinoma (TCGA): 500.0	Low region specificity	Detected in many			Group enriched	Detected in many
		Low region specificity	Detected in all			Low immune cell specificity	Detected in all
5	Glioblastoma Multiforme (TCGA): 25.7	Low region specificity	Detected in all			Not detected in immune cells	Not detected
		Low region specificity	Detected in all			Low immune cell specificity	Detected in all
	Kidney Chromophobe (TCGA): 18.0	Low region specificity	Detected in all			Immune cell enriched	Detected in many
	Testicular Germ Cell Tumor (TCGA): 18.5	Not detected	Not detected			Not detected in immune cells	Not detected
		Low region specificity	Detected in all			Low immune cell specificity	Detected in all
4	Stomach Adenocarcinoma (TCGA): 1.4	Low region specificity	Detected in all			Immune cell enriched	Detected in single
		Low region specificity	Detected in all			Low immune cell specificity	Detected in all
		Region enhanced	Detected in all		choroid plexus: 503.4	Not detected in immune cells	Not detected
		Low region specificity	Detected in all			Low immune cell specificity	Detected in many
		Low region specificity	Detected in all			Immune cell enhanced	Detected in many
		Low region specificity	Detected in all			Low immune cell specificity	Detected in all
		Low region specificity	Detected in all			Not detected in immune cells	Not detected
		Low region specificity	Detected in all			Not detected in immune cells	Not detected
		Low region specificity	Detected in all			Immune cell enhanced	Detected in many
		Low region specificity	Detected in all			Immune cell enhanced	Detected in some
		Low region specificity	Detected in all			Low immune cell specificity	Detected in all
		Low region specificity	Detected in all			Not detected in immune cells	Not detected
		Low region specificity	Detected in all			Immune cell enhanced	Detected in many
		Low region specificity	Detected in all			Not detected in immune cells	Not detected
		Low region specificity	Detected in all			Immune cell enriched	Detected in many
		Low region specificity	Detected in many			Not detected in immune cells	Not detected
		Low region specificity	Detected in all			Immune cell enhanced	Detected in single
		Low region specificity	Detected in all			Low immune cell specificity	Detected in all
		Low region specificity	Detected in all			Group enriched	Detected in some
		Low region specificity	Detected in all			Not detected in immune cells	Not detected
	Skin Cutaneous Melanoma (TCGA): 5.2	Low region specificity	Detected in single			Immune cell enriched	Detected in single
		Low region specificity	Detected in all			Not detected in immune cells	Not detected
		Low region specificity	Detected in all			Low immune cell specificity	Detected in many
		Low region specificity	Detected in many			Immune cell enriched	Detected in many
		Low region specificity	Detected in all			Not detected in immune cells	Not detected
		Low region specificity	Detected in many			Not detected in immune cells	Not detected
	Prostate Adenocarcinoma (TCGA): 118.7	Not detected	Not detected			Not detected in immune cells	Not detected
		Low region specificity	Detected in all			Low immune cell specificity	Detected in all
		Low region specificity	Detected in all			Low immune cell specificity	Detected in some
123	Liver Hepatocellular Carcinoma (TCGA): 105.3	Not detected	Not detected			Not detected in immune cells	Not detected

	Kidney Renal Clear Cell Carcinoma (TCGA): 219.1	Region enriched	Detected in some	9	cerebellum: 11.9	Not detected in immune cells	Not detected
		Low region specificity	Detected in all			Not detected in immune cells	Not detected
		Low region specificity	Detected in all			Immune cell enhanced	Detected in many
	Kidney Renal Clear Cell Carcinoma (TCGA): 41.2	Low region specificity	Detected in all			Group enriched	Detected in some
		Low region specificity	Detected in all			Immune cell enriched	Detected in single
		Low region specificity	Detected in all			Low immune cell specificity	Detected in some
		Low region specificity	Detected in all			Not detected in immune cells	Not detected
		Low region specificity	Detected in all			Low immune cell specificity	Detected in all
		Low region specificity	Detected in all			Not detected in immune cells	Not detected
	Kidney Renal Clear Cell Carcinoma (TCGA): 48.3	Low region specificity	Detected in all			Group enriched	Detected in many
	Head and Neck Squamous Cell Carcinoma (TCGA): 3.4	Low region specificity	Detected in many			Not detected in immune cells	Not detected
		Low region specificity	Detected in all			Low immune cell specificity	Detected in all
		Low region specificity	Detected in all			Low immune cell specificity	Detected in all
		Low region specificity	Detected in all			Not detected in immune cells	Not detected
		Low region specificity	Detected in all			Group enriched	Detected in many
		Region enhanced	Detected in all		basal ganglia: 47.2	Low immune cell specificity	Detected in all
		Low region specificity	Detected in all			Low immune cell specificity	Detected in many
5	Kidney Chromophobe (TCGA): 4.0	Group enriched	Detected in many	5	hypothalamus: 11.0;midbrain: 14.6	Not detected in immune cells	Not detected
9	Testicular Germ Cell Tumor (TCGA): 26.7	Low region specificity	Detected in many			Not detected in immune cells	Not detected
		Low region specificity	Detected in all			Low immune cell specificity	Detected in some
		Not detected	Not detected			Immune cell enhanced	Detected in many
		Low region specificity	Detected in all			Immune cell enriched	Detected in some
	Kidney Chromophobe (TCGA): 25.1	Low region specificity	Detected in all			Low immune cell specificity	Detected in all
		Low region specificity	Detected in all			Not detected in immune cells	Not detected
	Glioblastoma Multiforme (TCGA): 100.8	Region enhanced	Detected in all		thalamus: 112.0	Group enriched	Detected in many
		Low region specificity	Detected in all			Immune cell enhanced	Detected in many
		Low region specificity	Detected in many			Group enriched	Detected in all
		Low region specificity	Detected in some			Immune cell enriched	Detected in many
		Low region specificity	Detected in all			Group enriched	Detected in many
		Low region specificity	Detected in all			Immune cell enhanced	Detected in some
		Low region specificity	Detected in single			Immune cell enhanced	Detected in many
		Low region specificity	Detected in all			Immune cell enhanced	Detected in many
		Low region specificity	Detected in all			Group enriched	Detected in many
		Low region specificity	Detected in all			Immune cell enhanced	Detected in many
		Low region specificity	Detected in many			Group enriched	Detected in all
		Low region specificity	Detected in all			Group enriched	Detected in many
		Low region specificity	Detected in all			Group enriched	Detected in many
		Low region specificity	Detected in all			Immune cell enhanced	Detected in many
		Low region specificity	Detected in single			Group enriched	Detected in many
		Low region specificity	Detected in all			Low immune cell specificity	Detected in all
		Low region specificity	Detected in all			Low immune cell specificity	Detected in all
		Not detected	Not detected			Group enriched	Detected in all
		Low region specificity	Detected in all			Low immune cell specificity	Detected in all
		Low region specificity	Detected in all			Group enriched	Detected in many
		Low region specificity	Detected in all			Group enriched	Detected in many
		Low region specificity	Detected in many			Group enriched	Detected in many
		Low region specificity	Detected in all			Group enriched	Detected in many
		Low region specificity	Detected in all			Immune cell enhanced	Detected in single
		Low region specificity	Detected in all			Group enriched	Detected in some
		Low region specificity	Detected in all			Low immune cell specificity	Detected in many
		Low region specificity	Detected in all			Low immune cell specificity	Detected in all
		Region enriched	Detected in all	7	choroid plexus: 43.1	Group enriched	Detected in some
	Kidney Renal Clear Cell Carcinoma (TCGA): 35.6	Low region specificity	Detected in all			Immune cell enhanced	Detected in some
	Lung Adenocarcinoma (TCGA): 2.4	Region enriched	Detected in many	14	cerebellum: 51.0	Not detected in immune cells	Not detected
7	Colon Adenocarcinoma (TCGA): 241.8;Rectum Adenocarcinoma (TCGA): 243.5;Stomach Adenocarcinoma (TCGA): 83.5	Low region specificity	Detected in all			Immune cell enriched	Detected in some
	Glioblastoma Multiforme (TCGA): 82.4	Low region specificity	Detected in all			Group enriched	Detected in some
		Low region specificity	Detected in all			Not detected in immune cells	Not detected
		Region enriched	Detected in all	12	choroid plexus: 100.6	Not detected in immune cells	Not detected
6	Glioblastoma Multiforme (TCGA): 6.1;Kidney Renal Clear Cell Carcinoma (TCGA): 3.8;Kidney Renal Papillary Cell Carcinoma (TCGA): 1.9;Thyroid Carcinoma (T	Low region specificity	Detected in all			Not detected in immune cells	Not detected
	Kidney Renal Clear Cell Carcinoma (TCGA): 64.9	Low region specificity	Detected in all			Not detected in immune cells	Not detected
7	Kidney Renal Clear Cell Carcinoma (TCGA): 63.9;Kidney Renal Papillary Cell Carcinoma (TCGA): 79.9;Ovary Serous Cystadenocarcinoma (TCGA): 33.8	Low region specificity	Detected in all			Not detected in immune cells	Not detected
		Low region specificity	Detected in all			Low immune cell specificity	Detected in all
		Low region specificity	Detected in all			Low immune cell specificity	Detected in all
	Colon Adenocarcinoma (TCGA): 1966.2;Rectum Adenocarcinoma (TCGA): 2245.2	Low region specificity	Detected in some			Not detected in immune cells	Not detected
	Colon Adenocarcinoma (TCGA): 77.2;Rectum Adenocarcinoma (TCGA): 105.7	Not detected	Not detected			Not detected in immune cells	Not detected
		Low region specificity	Detected in all			Group enriched	Detected in some
		Low region specificity	Detected in all			Low immune cell specificity	Detected in many
		Low region specificity	Detected in all			Immune cell enriched	Detected in all
		Low region specificity	Detected in all			Not detected in immune cells	Not detected
	Kidney Chromophobe (TCGA): 88.9	Low region specificity	Detected in all			Not detected in immune cells	Not detected
		Low region specificity	Detected in all			Low immune cell specificity	Detected in all
		Low region specificity	Detected in all			Not detected in immune cells	Not detected
		Region enhanced	Detected in many		basal ganglia: 44.2	Not detected in immune cells	Not detected
	Glioblastoma Multiforme (TCGA): 11.1	Group enriched	Detected in some	15	cerebral cortex: 27.1;hippocampal fo	Not detected in immune cells	Not detected
200	Prostate Adenocarcinoma (TCGA): 51.0	Region enhanced	Detected in all		thalamus: 157.4	Not detected in immune cells	Not detected
	Testicular Germ Cell Tumor (TCGA): 3.4	Low region specificity	Detected in all			Not detected in immune cells	Not detected
		Low region specificity	Detected in all			Not detected in immune cells	Not detected
	Testicular Germ Cell Tumor (TCGA): 6.7	Low region specificity	Detected in all			Not detected in immune cells	Not detected
5	Head and Neck Squamous Cell Carcinoma (TCGA): 2.2	Not detected	Not detected			Not detected in immune cells	Not detected
		Low region specificity	Detected in all			Low immune cell specificity	Detected in all
6	Bladder Urothelial Carcinoma (TCGA): 61.6;Cervical Squamous Cell Carcinoma and Endocervical Adenocarcinoma (TCGA): 121.1;Head and Neck Squamous (	Low region specificity	Detected in single			Not detected in immune cells	Not detected
	Kidney Renal Papillary Cell Carcinoma (TCGA): 18.7	Low region specificity	Detected in all			Immune cell enhanced	Detected in some
5	Glioblastoma Multiforme (TCGA): 10.8	Low region specificity	Detected in all			Not detected in immune cells	Not detected
	Testicular Germ Cell Tumor (TCGA): 7.2	Region enriched	Detected in many	14	choroid plexus: 43.0	Not detected in immune cells	Not detected
	Kidney Renal Papillary Cell Carcinoma (TCGA): 221.1	Region enriched	Detected in many	13	choroid plexus: 243.0	Not detected in immune cells	Not detected
	Lung Squamous Cell Carcinoma (TCGA): 1.6	Low region specificity	Detected in all			Not detected in immune cells	Not detected
	Ovary Serous Cystadenocarcinoma (TCGA): 710.4	Region enriched	Detected in all	8	choroid plexus: 81.3	Not detected in immune cells	Not detected
		Region enhanced	Detected in many		choroid plexus: 11.2	Not detected in immune cells	Not detected
		Low region specificity	Detected in many			Low immune cell specificity	Detected in some
	Ovary Serous Cystadenocarcinoma (TCGA): 18.0	Low region specificity	Detected in many			Not detected in immune cells	Not detected
		Low region specificity	Detected in all			Immune cell enriched	Detected in some
		Low region specificity	Detected in all			Group enriched	Detected in many
		Low region specificity	Detected in all			Immune cell enhanced	Detected in many
	Ovary Serous Cystadenocarcinoma (TCGA): 26.8	Low region specificity	Detected in all			Immune cell enhanced	Detected in some
		Low region specificity	Detected in all			Low immune cell specificity	Detected in all
		Low region specificity	Detected in all			Low immune cell specificity	Detected in all
	Head and Neck Squamous Cell Carcinoma (TCGA): 2.6	Region enriched	Detected in many	12	choroid plexus: 35.6	Not detected in immune cells	Not detected
		Low region specificity	Detected in all			Not detected in immune cells	Not detected
		Low region specificity	Detected in all			Low immune cell specificity	Detected in single
9	Glioblastoma Multiforme (TCGA): 26.4	Low region specificity	Detected in all			Not detected in immune cells	Not detected
		Low region specificity	Detected in all			Low immune cell specificity	Detected in all
4	Head and Neck Squamous Cell Carcinoma (TCGA): 509.1	Low region specificity	Detected in many			Not detected in immune cells	Not detected
		Low region specificity	Detected in all			Group enriched	Detected in some
		Low region specificity	Detected in all			Immune cell enhanced	Detected in all
		Low region specificity	Detected in all			Immune cell enhanced	Detected in all
		Low region specificity	Detected in all			Group enriched	Detected in many
		Region enhanced	Detected in all		choroid plexus: 14.1	Immune cell enhanced	Detected in many
		Region enhanced	Detected in all		choroid plexus: 48.3	Not detected in immune cells	Not detected
6	Glioblastoma Multiforme (TCGA): 3128.0;Kidney Renal Clear Cell Carcinoma (TCGA): 2118.3;Kidney Renal Papillary Cell Carcinoma (TCGA): 7571.6	Low region specificity	Detected in all			Not detected in immune cells	Not detected
		Low region specificity	Detected in all			Group enriched	Detected in some
		Low region specificity	Detected in all			Immune cell enhanced	Detected in many
		Low region specificity	Detected in all			Low immune cell specificity	Detected in all
		Low region specificity	Detected in all			Low immune cell specificity	Detected in many
		Low region specificity	Detected in all			Low immune cell specificity	Detected in all
		Low region specificity	Detected in all			Group enriched	Detected in some
		Low region specificity	Detected in all			Immune cell enhanced	Detected in some
		Low region specificity	Detected in all			Not detected in immune cells	Not detected
4	Glioblastoma Multiforme (TCGA): 39.8;Kidney Renal Clear Cell Carcinoma (TCGA): 11.6	Low region specificity	Detected in all			Immune cell enhanced	Detected in many
		Low region specificity	Detected in all			Not detected in immune cells	Not detected
		Low region specificity	Detected in all			Immune cell enriched	Detected in many
		Low region specificity	Detected in all			Group enriched	Detected in many
		Low region specificity	Detected in all			Low immune cell specificity	Detected in all
		Low region specificity	Detected in all			Low immune cell specificity	Detected in all
	Kidney Renal Clear Cell Carcinoma (TCGA): 177.1	Low region specificity	Detected in all			Immune cell enriched	Detected in many
		Low region specificity	Detected in all			Low immune cell specificity	Detected in some
		Low region specificity	Detected in all			Low immune cell specificity	Detected in all
		Low region specificity	Detected in all			Low immune cell specificity	Detected in all
203	Skin Cutaneous Melanoma (TCGA): 338.3	Low region specificity	Detected in many			Not detected in immune cells	Not detected
		Low region specificity	Detected in all			Not detected in immune cells	Not detected

		Low region specificity	Detected in all			Not detected in immune cells	Not detected
		Low region specificity	Detected in all			Not detected in immune cells	Not detected
		Not detected	Not detected			Not detected in immune cells	Not detected
		Low region specificity	Detected in all			Low immune cell specificity	Detected in all
		Low region specificity	Detected in all			Low immune cell specificity	Detected in many
	Glioblastoma Multiforme (TCGA): 28.2	Low region specificity	Detected in all			Not detected in immune cells	Not detected
		Low region specificity	Detected in all			Not detected in immune cells	Not detected
		Low region specificity	Detected in all			Low immune cell specificity	Detected in many
		Low region specificity	Detected in all			Low immune cell specificity	Detected in all
		Low region specificity	Detected in all			Not detected in immune cells	Not detected
		Region enhanced	Detected in all		choroid plexus: 64.7	Low immune cell specificity	Detected in all
		Low region specificity	Detected in many			Immune cell enhanced	Detected in all
		Low region specificity	Detected in all			Immune cell enhanced	Detected in many
5	Colon Adenocarcinoma (TCGA): 177.2;Rectum Adenocarcinoma (TCGA): 210.7	Low region specificity	Detected in some			Not detected in immune cells	Not detected
	Ovary Serous Cystadenocarcinoma (TCGA): 1.2	Low region specificity	Detected in all			Not detected in immune cells	Not detected
	Head and Neck Squamous Cell Carcinoma (TCGA): 142.2	Low region specificity	Detected in all			Group enriched	Detected in some
6	Bladder Urothelial Carcinoma (TCGA): 44.6;Cervical Squamous Cell Carcinoma and Endocervical Adenocarcinoma (TCGA): 130.8;Head and Neck Squamous	Low region specificity	Detected in single			Not detected in immune cells	Not detected
		Region enriched	Detected in all	10	choroid plexus: 63.2	Immune cell enriched	Detected in single
	Head and Neck Squamous Cell Carcinoma (TCGA): 478.3	Region enriched	Detected in all	4	choroid plexus: 42.3	Immune cell enriched	Detected in single
15	Glioblastoma Multiforme (TCGA): 197.2	Low region specificity	Detected in all			Low immune cell specificity	Detected in many
	Kidney Renal Clear Cell Carcinoma (TCGA): 22.5	Low region specificity	Detected in all			Immune cell enriched	Detected in some
	Kidney Renal Clear Cell Carcinoma (TCGA): 31.4	Low region specificity	Detected in all			Not detected in immune cells	Not detected
		Low region specificity	Detected in all			Not detected in immune cells	Not detected
	Skin Cutaneous Melanoma (TCGA): 284.6	Low region specificity	Detected in all			Not detected in immune cells	Not detected
		Low region specificity	Detected in all			Immune cell enhanced	Detected in some
		Low region specificity	Detected in all			Low immune cell specificity	Detected in all
	Skin Cutaneous Melanoma (TCGA): 10.9	Low region specificity	Detected in all			Immune cell enhanced	Detected in single
4	Glioblastoma Multiforme (TCGA): 324.2	Low region specificity	Detected in all			Not detected in immune cells	Not detected
		Low region specificity	Detected in all			Low immune cell specificity	Detected in many
		Low region specificity	Detected in all			Low immune cell specificity	Detected in all
		Low region specificity	Detected in all			Not detected in immune cells	Not detected
		Low region specificity	Detected in all			Group enriched	Detected in many
	Breast Invasive Carcinoma (TCGA): 79.6;Prostate Adenocarcinoma (TCGA): 78.9	Low region specificity	Detected in all			Immune cell enriched	Detected in many
		Low region specificity	Detected in all			Not detected in immune cells	Not detected
		Low region specificity	Detected in all			Group enriched	Detected in many
		Low region specificity	Detected in all			Low immune cell specificity	Detected in all
		Low region specificity	Detected in all			Group enriched	Detected in many
	Uterine Corpus Endometrial Carcinoma (TCGA): 64.6	Low region specificity	Detected in all			Low immune cell specificity	Detected in all
		Low region specificity	Detected in all			Not detected in immune cells	Not detected
		Low region specificity	Detected in all			Low immune cell specificity	Detected in all
		Low region specificity	Detected in all			Immune cell enriched	Detected in single
		Low region specificity	Detected in all			Low immune cell specificity	Detected in many
		Low region specificity	Detected in all			Immune cell enhanced	Detected in some
		Low region specificity	Detected in all			Low immune cell specificity	Detected in some
		Low region specificity	Detected in many			Not detected in immune cells	Not detected
		Low region specificity	Detected in all			Immune cell enriched	Detected in single
		Low region specificity	Detected in all			Not detected in immune cells	Not detected
	Glioblastoma Multiforme (TCGA): 1.7;Prostate Adenocarcinoma (TCGA): 1.2	Low region specificity	Detected in all			Not detected in immune cells	Not detected
6	Glioblastoma Multiforme (TCGA): 18.5	Low region specificity	Detected in all			Group enriched	Detected in some
	Colon Adenocarcinoma (TCGA): 74.6;Rectum Adenocarcinoma (TCGA): 72.5	Low region specificity	Detected in all			Not detected in immune cells	Not detected
		Low region specificity	Detected in all			Low immune cell specificity	Detected in many
		Low region specificity	Detected in all			Immune cell enhanced	Detected in some
		Low region specificity	Detected in all			Immune cell enhanced	Detected in single
		Low region specificity	Detected in all			Low immune cell specificity	Detected in all
		Low region specificity	Detected in all			Low immune cell specificity	Detected in many
	Glioblastoma Multiforme (TCGA): 35.9	Low region specificity	Detected in all			Low immune cell specificity	Detected in all
		Low region specificity	Detected in all			Low immune cell specificity	Detected in all
		Low region specificity	Detected in all			Group enriched	Detected in all
	Kidney Renal Clear Cell Carcinoma (TCGA): 15.7	Low region specificity	Detected in all			Low immune cell specificity	Detected in many
		Low region specificity	Detected in all			Immune cell enhanced	Detected in many
		Low region specificity	Detected in all			Not detected in immune cells	Not detected
		Low region specificity	Detected in all			Immune cell enhanced	Detected in single
		Low region specificity	Detected in all			Not detected in immune cells	Not detected
		Not detected	Not detected			Group enriched	Detected in some
	Glioblastoma Multiforme (TCGA): 65.4	Low region specificity	Detected in all			Group enriched	Detected in many
		Low region specificity	Detected in all			Immune cell enriched	Detected in many
		Low region specificity	Detected in all			Low immune cell specificity	Detected in all
		Low region specificity	Detected in all			Low immune cell specificity	Detected in all
9	Pancreatic Adenocarcinoma (TCGA): 3.1	Low region specificity	Detected in all			Immune cell enhanced	Detected in single
	Colon Adenocarcinoma (TCGA): 7.3;Rectum Adenocarcinoma (TCGA): 5.6	Low region specificity	Detected in all			Not detected in immune cells	Not detected
	Testicular Germ Cell Tumor (TCGA): 33.1	Low region specificity	Detected in all			Not detected in immune cells	Not detected
		Low region specificity	Detected in all			Not detected in immune cells	Not detected
	Kidney Renal Papillary Cell Carcinoma (TCGA): 624.9	Low region specificity	Detected in all			Immune cell enhanced	Detected in many
		Low region specificity	Detected in all			Low immune cell specificity	Detected in some
		Low region specificity	Detected in all			Immune cell enhanced	Detected in all
		Low region specificity	Detected in all			Low immune cell specificity	Detected in all
	Kidney Renal Papillary Cell Carcinoma (TCGA): 48.6	Low region specificity	Detected in all			Not detected in immune cells	Not detected
	Kidney Renal Clear Cell Carcinoma (TCGA): 123.0	Low region specificity	Detected in all			Not detected in immune cells	Not detected
	Kidney Renal Clear Cell Carcinoma (TCGA): 20.8	Low region specificity	Detected in all			Immune cell enriched	Detected in single
		Low region specificity	Detected in all			Immune cell enhanced	Detected in some
		Low region specificity	Detected in all			Low immune cell specificity	Detected in all
		Low region specificity	Detected in all			Group enriched	Detected in some
		Low region specificity	Detected in all			Low immune cell specificity	Detected in all
	Glioblastoma Multiforme (TCGA): 182.7	Low region specificity	Detected in all			Low immune cell specificity	Detected in many
		Low region specificity	Detected in all			Immune cell enhanced	Detected in some
	Kidney Renal Clear Cell Carcinoma (TCGA): 42.1	Low region specificity	Detected in all			Not detected in immune cells	Not detected
		Low region specificity	Detected in all			Immune cell enriched	Detected in single
	Skin Cutaneous Melanoma (TCGA): 63.0	Low region specificity	Detected in all			Immune cell enhanced	Detected in many
		Low region specificity	Detected in all			Not detected in immune cells	Not detected
	Glioblastoma Multiforme (TCGA): 1.5	Low region specificity	Detected in all			Not detected in immune cells	Not detected
6	Glioblastoma Multiforme (TCGA): 2.4;Lung Squamous Cell Carcinoma (TCGA): 1.4;Skin Cutaneous Melanoma (TCGA): 1.6;Testicular Germ Cell Tumor (TCGA):	Low region specificity	Detected in all			Not detected in immune cells	Not detected
		Low region specificity	Detected in all			Not detected in immune cells	Not detected
	Prostate Adenocarcinoma (TCGA): 9.5	Low region specificity	Detected in all			Not detected in immune cells	Not detected
8	Glioblastoma Multiforme (TCGA): 1.7;Kidney Chromophobe (TCGA): 2.5	Low region specificity	Detected in all			Not detected in immune cells	Not detected
45	Glioblastoma Multiforme (TCGA): 579.4	Low region specificity	Detected in all			Not detected in immune cells	Not detected
		Low region specificity	Detected in all			Low immune cell specificity	Detected in all
		Low region specificity	Detected in all			Immune cell enriched	Detected in many
		Low region specificity	Detected in all			Low immune cell specificity	Detected in all
		Low region specificity	Detected in all			Low immune cell specificity	Detected in many
	Lung Adenocarcinoma (TCGA): 18.0	Region enriched	Detected in single	9	cerebellum: 5.2	Not detected in immune cells	Not detected
		Low region specificity	Detected in some			Not detected in immune cells	Not detected
	Prostate Adenocarcinoma (TCGA): 17.1	Low region specificity	Detected in all			Not detected in immune cells	Not detected
		Low region specificity	Detected in all			Low immune cell specificity	Detected in all
		Low region specificity	Detected in all			Not detected in immune cells	Not detected
	Cervical Squamous Cell Carcinoma and Endocervical Adenocarcinoma (TCGA): 95.2;Head and Neck Squamous Cell Carcinoma (TCGA): 105.2	Low region specificity	Detected in many			Not detected in immune cells	Not detected
	Head and Neck Squamous Cell Carcinoma (TCGA): 18.5	Low region specificity	Detected in some			Not detected in immune cells	Not detected
	Head and Neck Squamous Cell Carcinoma (TCGA): 261.6	Low region specificity	Detected in all			Immune cell enhanced	Detected in some
	Testicular Germ Cell Tumor (TCGA): 27.9	Low region specificity	Detected in all			Immune cell enhanced	Detected in single
		Low region specificity	Detected in all			Not detected in immune cells	Not detected
	Kidney Chromophobe (TCGA): 24.6	Low region specificity	Detected in all			Not detected in immune cells	Not detected
		Low region specificity	Detected in all			Low immune cell specificity	Detected in all
	Pancreatic Adenocarcinoma (TCGA): 1.7	Low region specificity	Detected in many			Not detected in immune cells	Not detected
	Pancreatic Adenocarcinoma (TCGA): 2.4	Low region specificity	Detected in many			Not detected in immune cells	Not detected
		Low region specificity	Detected in all			Not detected in immune cells	Not detected
	Rectum Adenocarcinoma (TCGA): 1.7	Low region specificity	Detected in many			Not detected in immune cells	Not detected
4	Breast Invasive Carcinoma (TCGA): 2.7	Low region specificity	Detected in all			Not detected in immune cells	Not detected
		Low region specificity	Detected in all			Low immune cell specificity	Detected in all
		Low region specificity	Detected in all			Immune cell enhanced	Detected in many
		Low region specificity	Detected in all			Low immune cell specificity	Detected in all
		Low region specificity	Detected in all			Low immune cell specificity	Detected in all
		Low region specificity	Detected in many			Not detected in immune cells	Not detected
		Low region specificity	Detected in all			Not detected in immune cells	Not detected
	Liver Hepatocellular Carcinoma (TCGA): 291.2	Low region specificity	Detected in all			Immune cell enhanced	Detected in some
5	Skin Cutaneous Melanoma (TCGA): 198.9	Low region specificity	Detected in all			Immune cell enriched	Detected in single
		Low region specificity	Detected in many			Not detected in immune cells	Not detected
		Low region specificity	Detected in some			Not detected in immune cells	Not detected
		Low region specificity	Detected in all			Low immune cell specificity	Detected in many
		Low region specificity	Detected in all			Low immune cell specificity	Detected in many
		Low region specificity	Detected in all			Immune cell enhanced	Detected in all

		Low region specificity	Detected in single			Group enriched	Detected in some
45	Glioblastoma Multiforme (TCGA): 55.2	Low region specificity	Detected in all			Not detected in immune cells	Not detected
	Head and Neck Squamous Cell Carcinoma (TCGA): 2.2;Lung Squamous Cell Carcinoma (TCGA): 2.0;Testicular Germ Cell Tumor (TCGA): 1.8	Region enhanced	Detected in many		hypothalamus: 7.2	Not detected in immune cells	Not detected
5	Glioblastoma Multiforme (TCGA): 7.0	Low region specificity	Detected in all			Not detected in immune cells	Not detected
		Low region specificity	Detected in all			Immune cell enriched	Detected in single
		Low region specificity	Detected in all			Immune cell enhanced	Detected in some
		Low region specificity	Detected in all			Not detected in immune cells	Not detected
		Low region specificity	Detected in all			Low immune cell specificity	Detected in many
		Low region specificity	Detected in all			Immune cell enriched	Detected in many
		Group enriched	Detected in many	9	cerebellum: 11.6;choroid plexus: 9.6	Not detected in immune cells	Not detected
5	Glioblastoma Multiforme (TCGA): 9.1	Low region specificity	Detected in all			Not detected in immune cells	Not detected
6	Glioblastoma Multiforme (TCGA): 35.3;Kidney Chromophobe (TCGA): 10.6;Ovary Serous Cystadenocarcinoma (TCGA): 11.9;Thyroid Carcinoma (TCGA): 11.8	Low region specificity	Detected in all			Not detected in immune cells	Not detected
	Colon Adenocarcinoma (TCGA): 1.1;Rectum Adenocarcinoma (TCGA): 1.1	Low region specificity	Detected in all			Not detected in immune cells	Not detected
		Low region specificity	Detected in all			Low immune cell specificity	Detected in many
5	Breast Invasive Carcinoma (TCGA): 11.7	Low region specificity	Detected in many			Not detected in immune cells	Not detected
	Head and Neck Squamous Cell Carcinoma (TCGA): 11.6;Skin Cutaneous Melanoma (TCGA): 10.4	Low region specificity	Detected in all			Low immune cell specificity	Detected in some
		Low region specificity	Detected in all			Not detected in immune cells	Not detected
		Low region specificity	Detected in all			Not detected in immune cells	Not detected
	Skin Cutaneous Melanoma (TCGA): 387.0	Low region specificity	Detected in all			Low immune cell specificity	Detected in many
		Low region specificity	Detected in all			Low immune cell specificity	Detected in all
	Pancreatic Adenocarcinoma (TCGA): 4.1	Low region specificity	Detected in many			Immune cell enriched	Detected in single
	Head and Neck Squamous Cell Carcinoma (TCGA): 37.6	Region enriched	Detected in some	5	cerebral cortex: 12.1	Immune cell enriched	Detected in many
		Low region specificity	Detected in all			Group enriched	Detected in many
		Low region specificity	Detected in all			Low immune cell specificity	Detected in all
		Low region specificity	Detected in many			Not detected in immune cells	Not detected
		Low region specificity	Detected in all			Low immune cell specificity	Detected in many
		Low region specificity	Detected in all			Low immune cell specificity	Detected in all
		Low region specificity	Detected in all			Immune cell enhanced	Detected in many
		Low region specificity	Detected in all			Low immune cell specificity	Detected in all
		Low region specificity	Detected in all			Low immune cell specificity	Detected in all
		Low region specificity	Detected in all			Immune cell enhanced	Detected in many
		Low region specificity	Detected in many			Not detected in immune cells	Not detected
		Low region specificity	Detected in all			Low immune cell specificity	Detected in all
		Low region specificity	Detected in all			Low immune cell specificity	Detected in all
4	Bladder Urothelial Carcinoma (TCGA): 3.1;Cervical Squamous Cell Carcinoma and Endocervical Adenocarcinoma (TCGA): 2.5;Head and Neck Squamous Cell	Low region specificity	Detected in all			Immune cell enriched	Detected in single
		Low region specificity	Detected in all			Not detected in immune cells	Not detected
		Low region specificity	Detected in all			Low immune cell specificity	Detected in many
		Low region specificity	Detected in all			Immune cell enriched	Detected in all
		Low region specificity	Detected in all			Low immune cell specificity	Detected in many
		Low region specificity	Detected in all			Low immune cell specificity	Detected in all
		Low region specificity	Detected in all			Group enriched	Detected in all
		Region enriched	Detected in some	7	cerebral cortex: 10.9	Not detected in immune cells	Not detected
		Low region specificity	Detected in all			Low immune cell specificity	Detected in all
		Low region specificity	Detected in all			Immune cell enhanced	Detected in some
		Low region specificity	Detected in all			Immune cell enhanced	Detected in all
		Low region specificity	Detected in many			Low immune cell specificity	Detected in all
		Low region specificity	Detected in all			Low immune cell specificity	Detected in many
		Low region specificity	Detected in all			Immune cell enhanced	Detected in single
	Thyroid Carcinoma (TCGA): 17.6	Low region specificity	Detected in all			Group enriched	Detected in some
		Low region specificity	Detected in many			Low immune cell specificity	Detected in all
		Low region specificity	Detected in all			Immune cell enhanced	Detected in all
		Low region specificity	Detected in all			Immune cell enhanced	Detected in many
		Low region specificity	Detected in all			Group enriched	Detected in many
		Low region specificity	Detected in all			Low immune cell specificity	Detected in all
		Low region specificity	Detected in all			Low immune cell specificity	Detected in all
		Low region specificity	Detected in all			Not detected in immune cells	Not detected
		Low region specificity	Detected in all			Immune cell enhanced	Detected in many
		Low region specificity	Detected in all			Low immune cell specificity	Detected in all
		Low region specificity	Detected in all			Low immune cell specificity	Detected in all
	Skin Cutaneous Melanoma (TCGA): 42.7;Thyroid Carcinoma (TCGA): 54.7	Low region specificity	Detected in all			Group enriched	Detected in some
		Low region specificity	Detected in all			Immune cell enhanced	Detected in some
		Low region specificity	Detected in all			Immune cell enhanced	Detected in some
17	Thyroid Carcinoma (TCGA): 163.5	Low region specificity	Detected in all			Immune cell enhanced	Detected in some
8	Glioblastoma Multiforme (TCGA): 54.4	Low region specificity	Detected in all			Not detected in immune cells	Not detected
		Low region specificity	Detected in some			Immune cell enhanced	Detected in all
		Low region specificity	Detected in all			Group enriched	Detected in many
		Low region specificity	Detected in all			Immune cell enhanced	Detected in some
		Low region specificity	Detected in all			Low immune cell specificity	Detected in all
		Low region specificity	Detected in all			Low immune cell specificity	Detected in all
6	Glioblastoma Multiforme (TCGA): 4.6;Testicular Germ Cell Tumor (TCGA): 3.3	Low region specificity	Detected in all			Not detected in immune cells	Not detected
	Thyroid Carcinoma (TCGA): 23.3	Low region specificity	Detected in all			Low immune cell specificity	Detected in single
		Low region specificity	Detected in all			Group enriched	Detected in many
		Low region specificity	Detected in all			Not detected in immune cells	Not detected
		Low region specificity	Detected in many			Immune cell enhanced	Detected in single
	Thyroid Carcinoma (TCGA): 4.2	Low region specificity	Detected in many			Immune cell enriched	Detected in some
		Low region specificity	Detected in all			Low immune cell specificity	Detected in many
	Kidney Chromophobe (TCGA): 119.6	Low region specificity	Detected in all			Low immune cell specificity	Detected in many
	Cervical Squamous Cell Carcinoma and Endocervical Adenocarcinoma (TCGA): 9.5	Region enhanced	Detected in all		cerebral cortex: 58.0	Not detected in immune cells	Not detected
		Low region specificity	Detected in all			Not detected in immune cells	Not detected
	Ovary Serous Cystadenocarcinoma (TCGA): 1.9	Low region specificity	Detected in all			Not detected in immune cells	Not detected
4	Kidney Renal Papillary Cell Carcinoma (TCGA): 2.7	Low region specificity	Detected in all			Not detected in immune cells	Not detected
	Testicular Germ Cell Tumor (TCGA): 4.3	Low region specificity	Detected in many			Not detected in immune cells	Not detected
		Low region specificity	Detected in all			Not detected in immune cells	Not detected
5	Kidney Renal Clear Cell Carcinoma (TCGA): 9.9	Low region specificity	Detected in many			Not detected in immune cells	Not detected
	Kidney Chromophobe (TCGA): 143.8	Low region specificity	Detected in all			Group enriched	Detected in many
		Low region specificity	Detected in all			Low immune cell specificity	Detected in many
	Ovary Serous Cystadenocarcinoma (TCGA): 241.8	Low region specificity	Detected in all			Not detected in immune cells	Not detected
		Low region specificity	Detected in many			Group enriched	Detected in many
	Skin Cutaneous Melanoma (TCGA): 114.3	Low region specificity	Detected in all			Not detected in immune cells	Not detected
		Low region specificity	Detected in all			Low immune cell specificity	Detected in many
		Low region specificity	Detected in all			Immune cell enhanced	Detected in all
		Low region specificity	Detected in all			Low immune cell specificity	Detected in all
		Low region specificity	Detected in all			Group enriched	Detected in many
		Low region specificity	Detected in all			Immune cell enhanced	Detected in many
		Low region specificity	Detected in all			Low immune cell specificity	Detected in many
		Low region specificity	Detected in all			Low immune cell specificity	Detected in all
5	Liver Hepatocellular Carcinoma (TCGA): 39.8	Low region specificity	Detected in all			Immune cell enhanced	Detected in some
		Low region specificity	Detected in many			Not detected in immune cells	Not detected
	Glioblastoma Multiforme (TCGA): 45.0	Low region specificity	Detected in all			Not detected in immune cells	Not detected
		Not detected	Not detected			Not detected in immune cells	Not detected
		Low region specificity	Detected in all			Immune cell enriched	Detected in all
		Low region specificity	Detected in all			Not detected in immune cells	Not detected
	Ovary Serous Cystadenocarcinoma (TCGA): 30.5	Low region specificity	Detected in all			Not detected in immune cells	Not detected
	Glioblastoma Multiforme (TCGA): 2.1	Low region specificity	Detected in all			Immune cell enriched	Detected in some
		Low region specificity	Detected in all			Low immune cell specificity	Detected in many
	Stomach Adenocarcinoma (TCGA): 77.6	Low region specificity	Detected in all			Immune cell enhanced	Detected in many
		Low region specificity	Detected in all			Not detected in immune cells	Not detected
		Low region specificity	Detected in all			Group enriched	Detected in some
6	Prostate Adenocarcinoma (TCGA): 92.6	Region enhanced	Detected in many		cerebellum: 6.5	Immune cell enriched	Detected in single
		Low region specificity	Detected in all			Low immune cell specificity	Detected in many
		Low region specificity	Detected in all			Immune cell enhanced	Detected in all
	Head and Neck Squamous Cell Carcinoma (TCGA): 11.3	Low region specificity	Detected in all			Not detected in immune cells	Not detected
		Low region specificity	Detected in all			Immune cell enhanced	Detected in many
		Low region specificity	Detected in all			Low immune cell specificity	Detected in many
		Low region specificity	Detected in all			Immune cell enhanced	Detected in many
		Low region specificity	Detected in all			Not detected in immune cells	Not detected
		Low region specificity	Detected in all			Not detected in immune cells	Not detected
		Low region specificity	Detected in all			Low immune cell specificity	Detected in all
		Low region specificity	Detected in all			Low immune cell specificity	Detected in all
	Glioblastoma Multiforme (TCGA): 110.3	Low region specificity	Detected in all			Not detected in immune cells	Not detected
	Breast Invasive Carcinoma (TCGA): 30.6	Low region specificity	Detected in all			Immune cell enhanced	Detected in single
		Low region specificity	Detected in all			Immune cell enhanced	Detected in many
		Low region specificity	Detected in all			Low immune cell specificity	Detected in many
		Low region specificity	Detected in all			Not detected in immune cells	Not detected
		Not detected	Not detected			Not detected in immune cells	Not detected
		Low region specificity	Detected in all			Not detected in immune cells	Not detected
	Skin Cutaneous Melanoma (TCGA): 309.5	Low region specificity	Detected in all			Not detected in immune cells	Not detected
	Lung Adenocarcinoma (TCGA): 10.2	Group enriched	Detected in some	5	cerebral cortex: 6.2;pons: 2.8;thalam	Group enriched	Detected in many
		Low region specificity	Detected in all			Not detected in immune cells	Not detected

		Low region specificity	Detected in many			Not detected in immune cells	Not detected
		Low region specificity	Detected in all			Group enriched	Detected in all
		Region enriched	Detected in all	7	cerebellum: 175.0	Not detected in immune cells	Not detected
		Low region specificity	Detected in all			Immune cell enhanced	Detected in many
	Glioblastoma Multiforme (TCGA): 6.8	Low region specificity	Detected in all			Not detected in immune cells	Not detected
		Low region specificity	Detected in all			Group enriched	Detected in some
		Low region specificity	Detected in all			Group enriched	Detected in some
		Low region specificity	Detected in all			Group enriched	Detected in many
		Low region specificity	Detected in all			Not detected in immune cells	Not detected
		Low region specificity	Detected in all			Low immune cell specificity	Detected in all
	Colon Adenocarcinoma (TCGA): 185.9;Rectum Adenocarcinoma (TCGA): 174.3	Not detected	Not detected			Not detected in immune cells	Not detected
		Low region specificity	Detected in all			Low immune cell specificity	Detected in all
5	Testicular Germ Cell Tumor (TCGA): 2.7	Low region specificity	Detected in all			Not detected in immune cells	Not detected
		Region enhanced	Detected in all		basal ganglia: 37.9	Immune cell enriched	Detected in some
4	Testicular Germ Cell Tumor (TCGA): 11.9	Low region specificity	Detected in many			Group enriched	Detected in some
		Low region specificity	Detected in all			Immune cell enhanced	Detected in single
		Low region specificity	Detected in all			Immune cell enhanced	Detected in all
		Low region specificity	Detected in all			Low immune cell specificity	Detected in many
		Low region specificity	Detected in all			Immune cell enriched	Detected in many
		Low region specificity	Detected in all			Low immune cell specificity	Detected in all
		Region enhanced	Detected in all		cerebellum: 21.9	Not detected in immune cells	Not detected
		Not detected	Not detected			Group enriched	Detected in many
	Lung Adenocarcinoma (TCGA): 473.2;Ovary Serous Cystadenocarcinoma (TCGA): 670.7	Low region specificity	Detected in many			Immune cell enhanced	Detected in some
		Low region specificity	Detected in all			Low immune cell specificity	Detected in all
		Low region specificity	Detected in all			Low immune cell specificity	Detected in all
		Low region specificity	Detected in all			Immune cell enhanced	Detected in single
4	Prostate Adenocarcinoma (TCGA): 439.6;Stomach Adenocarcinoma (TCGA): 369.4	Low region specificity	Detected in all			Not detected in immune cells	Not detected
	Prostate Adenocarcinoma (TCGA): 232.3	Low region specificity	Detected in all			Immune cell enhanced	Detected in some
		Low region specificity	Detected in all			Immune cell enriched	Detected in single
		Low region specificity	Detected in all			Not detected in immune cells	Not detected
		Low region specificity	Detected in all			Low immune cell specificity	Detected in many
	Lung Adenocarcinoma (TCGA): 16.5	Low region specificity	Detected in many			Low immune cell specificity	Detected in all
		Region enhanced	Detected in all		choroid plexus: 78.3	Immune cell enhanced	Detected in some
		Low region specificity	Detected in all			Immune cell enriched	Detected in single
	Prostate Adenocarcinoma (TCGA): 11.1	Low region specificity	Detected in all			Low immune cell specificity	Detected in some
		Low region specificity	Detected in all			Low immune cell specificity	Detected in all
10	Glioblastoma Multiforme (TCGA): 129.5	Low region specificity	Detected in all			Immune cell enriched	Detected in some
4	Breast Invasive Carcinoma (TCGA): 6.5;Glioblastoma Multiforme (TCGA): 12.8	Low region specificity	Detected in all			Not detected in immune cells	Not detected
		Low region specificity	Detected in all			Low immune cell specificity	Detected in all
		Low region specificity	Detected in all			Not detected in immune cells	Not detected
	Bladder Urothelial Carcinoma (TCGA): 112.2	Low region specificity	Detected in many			Not detected in immune cells	Not detected
		Low region specificity	Detected in all			Not detected in immune cells	Not detected
	Kidney Chromophobe (TCGA): 5.3	Low region specificity	Detected in all			Immune cell enhanced	Detected in some
		Low region specificity	Detected in all			Low immune cell specificity	Detected in many
		Low region specificity	Detected in all			Low immune cell specificity	Detected in all
	Skin Cutaneous Melanoma (TCGA): 64.8	Region enhanced	Detected in all		cerebral cortex: 187.1	Immune cell enriched	Detected in single
		Low region specificity	Detected in all			Immune cell enhanced	Detected in single
		Low region specificity	Detected in all			Immune cell enhanced	Detected in single
	Thyroid Carcinoma (TCGA): 48.6	Region enriched	Detected in all	18	choroid plexus: 97.5	Not detected in immune cells	Not detected
		Low region specificity	Detected in all			Low immune cell specificity	Detected in all
17	Glioblastoma Multiforme (TCGA): 39.5	Low region specificity	Detected in all			Immune cell enhanced	Detected in single
		Low region specificity	Detected in many			Not detected in immune cells	Not detected
		Low region specificity	Detected in all			Group enriched	Detected in many
5	Pancreatic Adenocarcinoma (TCGA): 3.2;Stomach Adenocarcinoma (TCGA): 3.8	Low region specificity	Detected in many			Not detected in immune cells	Not detected
		Low region specificity	Detected in all			Not detected in immune cells	Not detected
		Low region specificity	Detected in all			Low immune cell specificity	Detected in many
		Low region specificity	Detected in all			Not detected in immune cells	Not detected
		Low region specificity	Detected in many			Not detected in immune cells	Not detected
		Low region specificity	Detected in all			Low immune cell specificity	Detected in all
		Low region specificity	Detected in all			Immune cell enhanced	Detected in many
	Glioblastoma Multiforme (TCGA): 5.0	Region enhanced	Detected in all		thalamus: 164.8	Immune cell enriched	Detected in single
	Glioblastoma Multiforme (TCGA): 4.2	Region enhanced	Detected in all		cerebral cortex: 149.3	Immune cell enriched	Detected in single
	Glioblastoma Multiforme (TCGA): 135.6;Thyroid Carcinoma (TCGA): 104.6	Low region specificity	Detected in all			Not detected in immune cells	Not detected
	Pancreatic Adenocarcinoma (TCGA): 1.7;Stomach Adenocarcinoma (TCGA): 2.4	Region enriched	Detected in many	6	midbrain: 25.2	Not detected in immune cells	Not detected
		Low region specificity	Detected in all			Immune cell enhanced	Detected in all
		Low region specificity	Detected in all			Immune cell enhanced	Detected in single
		Low region specificity	Detected in all			Low immune cell specificity	Detected in all
12	Colon Adenocarcinoma (TCGA): 1060.5;Pancreatic Adenocarcinoma (TCGA): 847.1;Rectum Adenocarcinoma (TCGA): 758.1;Stomach Adenocarcinoma (TCGA	Not detected	Not detected			Not detected in immune cells	Not detected
		Low region specificity	Detected in all			Immune cell enhanced	Detected in some
		Low region specificity	Detected in all			Immune cell enriched	Detected in many
	Prostate Adenocarcinoma (TCGA): 3.0	Low region specificity	Detected in many			Not detected in immune cells	Not detected
		Not detected	Not detected			Not detected in immune cells	Not detected
		Low region specificity	Detected in some			Not detected in immune cells	Not detected
		Low region specificity	Detected in many			Not detected in immune cells	Not detected
		Low region specificity	Detected in all			Immune cell enriched	Detected in single
		Low region specificity	Detected in many			Not detected in immune cells	Not detected
	Glioblastoma Multiforme (TCGA): 11.0	Low region specificity	Detected in all			Not detected in immune cells	Not detected
		Low region specificity	Detected in all			Low immune cell specificity	Detected in some
4	Kidney Chromophobe (TCGA): 21.3	Low region specificity	Detected in many			Immune cell enhanced	Detected in single
		Low region specificity	Detected in all			Immune cell enhanced	Detected in all
		Low region specificity	Detected in all			Low immune cell specificity	Detected in all
		Low region specificity	Detected in all			Low immune cell specificity	Detected in all
		Low region specificity	Detected in all			Group enriched	Detected in all
		Low region specificity	Detected in all			Low immune cell specificity	Detected in some
		Low region specificity	Detected in all			Immune cell enriched	Detected in single
		Low region specificity	Detected in all			Immune cell enhanced	Detected in many
		Low region specificity	Detected in all			Low immune cell specificity	Detected in some
		Low region specificity	Detected in all			Low immune cell specificity	Detected in many
		Low region specificity	Detected in all			Immune cell enhanced	Detected in single
		Low region specificity	Detected in all			Low immune cell specificity	Detected in many
		Low region specificity	Detected in all			Immune cell enriched	Detected in single
		Low region specificity	Detected in all			Not detected in immune cells	Not detected
		Low region specificity	Detected in all			Low immune cell specificity	Detected in many
4	Kidney Chromophobe (TCGA): 138.3;Skin Cutaneous Melanoma (TCGA): 127.6	Low region specificity	Detected in all			Low immune cell specificity	Detected in many
		Region enhanced	Detected in all		cerebellum: 161.2	Low immune cell specificity	Detected in some
		Low region specificity	Detected in all			Not detected in immune cells	Not detected
		Low region specificity	Detected in all			Not detected in immune cells	Not detected
		Low region specificity	Detected in all			Not detected in immune cells	Not detected
6	Glioblastoma Multiforme (TCGA): 22.5	Low region specificity	Detected in all			Not detected in immune cells	Not detected
		Low region specificity	Detected in all			Low immune cell specificity	Detected in all
		Low region specificity	Detected in all			Low immune cell specificity	Detected in many
		Low region specificity	Detected in all			Immune cell enriched	Detected in many
4	Prostate Adenocarcinoma (TCGA): 97.5	Low region specificity	Detected in all			Immune cell enhanced	Detected in some
		Low region specificity	Detected in all			Not detected in immune cells	Not detected
	Glioblastoma Multiforme (TCGA): 101.1	Low region specificity	Detected in all			Not detected in immune cells	Not detected
	Glioblastoma Multiforme (TCGA): 216.3;Testicular Germ Cell Tumor (TCGA): 288.4	Low region specificity	Detected in all			Not detected in immune cells	Not detected
		Low region specificity	Detected in all			Not detected in immune cells	Not detected
	Kidney Renal Clear Cell Carcinoma (TCGA): 166.1	Low region specificity	Detected in all			Immune cell enhanced	Detected in all
4	Thyroid Carcinoma (TCGA): 8.9	Low region specificity	Detected in many			Immune cell enriched	Detected in single
		Low region specificity	Detected in all			Low immune cell specificity	Detected in many
		Low region specificity	Detected in all			Low immune cell specificity	Detected in all
		Low region specificity	Detected in all			Low immune cell specificity	Detected in all
		Low region specificity	Detected in all			Low immune cell specificity	Detected in many
		Low region specificity	Detected in all			Immune cell enhanced	Detected in single
		Low region specificity	Detected in all			Group enriched	Detected in some
		Low region specificity	Detected in some			Not detected in immune cells	Not detected
		Low region specificity	Detected in all			Immune cell enhanced	Detected in single
		Low region specificity	Detected in all			Immune cell enhanced	Detected in many
		Low region specificity	Detected in all			Low immune cell specificity	Detected in all
		Low region specificity	Detected in all			Immune cell enhanced	Detected in all
	Colon Adenocarcinoma (TCGA): 38.5;Rectum Adenocarcinoma (TCGA): 46.7	Low region specificity	Detected in all			Group enriched	Detected in some
		Low region specificity	Detected in all			Group enriched	Detected in many
		Low region specificity	Detected in all			Group enriched	Detected in some
		Low region specificity	Detected in all			Immune cell enhanced	Detected in single
		Not detected	Not detected			Not detected in immune cells	Not detected
	Breast Invasive Carcinoma (TCGA): 15.3	Low region specificity	Detected in all			Not detected in immune cells	Not detected
4	Kidney Renal Clear Cell Carcinoma (TCGA): 226.7;Prostate Adenocarcinoma (TCGA): 555.0	Low region specificity	Detected in all			Low immune cell specificity	Detected in many
		Low region specificity	Detected in all			Immune cell enhanced	Detected in some
		Low region specificity	Detected in all			Low immune cell specificity	Detected in all

		Low region specificity	Detected in all			Not detected in immune cells	Not detected
		Low region specificity	Detected in all			Not detected in immune cells	Not detected
		Low region specificity	Detected in all			Immune cell enhanced	Detected in single
		Low region specificity	Detected in all			Low immune cell specificity	Detected in many
		Low region specificity	Detected in all			Low immune cell specificity	Detected in many
		Low region specificity	Detected in all			Immune cell enhanced	Detected in some
		Low region specificity	Detected in all			Low immune cell specificity	Detected in all
	Skin Cutaneous Melanoma (TCGA): 192.8	Not detected	Not detected			Not detected in immune cells	Not detected
		Low region specificity	Detected in all			Low immune cell specificity	Detected in some
		Low region specificity	Detected in all			Low immune cell specificity	Detected in some
		Low region specificity	Detected in all			Not detected in immune cells	Not detected
		Low region specificity	Detected in all			Low immune cell specificity	Detected in all
		Low region specificity	Detected in all			Low immune cell specificity	Detected in many
	Glioblastoma Multiforme (TCGA): 14.9	Low region specificity	Detected in all			Immune cell enriched	Detected in single
4	Bladder Urothelial Carcinoma (TCGA): 1552.3	Low region specificity	Detected in single			Not detected in immune cells	Not detected
		Low region specificity	Detected in all			Low immune cell specificity	Detected in many
		Low region specificity	Detected in all			Low immune cell specificity	Detected in all
		Low region specificity	Detected in all			Low immune cell specificity	Detected in all
		Low region specificity	Detected in all			Group enriched	Detected in many
	Testicular Germ Cell Tumor (TCGA): 16.0	Low region specificity	Detected in all			Low immune cell specificity	Detected in many
6	Kidney Renal Papillary Cell Carcinoma (TCGA): 141.0	Low region specificity	Detected in all			Not detected in immune cells	Not detected
		Low region specificity	Detected in all			Low immune cell specificity	Detected in all
		Region enriched	Detected in all	4	choroid plexus: 68.9	Immune cell enriched	Detected in some
	Pancreatic Adenocarcinoma (TCGA): 26.4	Low region specificity	Detected in many			Not detected in immune cells	Not detected
		Low region specificity	Detected in all			Immune cell enhanced	Detected in many
		Low region specificity	Detected in all			Group enriched	Detected in some
		Low region specificity	Detected in all			Immune cell enhanced	Detected in many
		Low region specificity	Detected in all			Not detected in immune cells	Not detected
		Low region specificity	Detected in all			Immune cell enriched	Detected in single
		Low region specificity	Detected in all			Group enriched	Detected in some
	Kidney Chromophobe (TCGA): 100.7	Low region specificity	Detected in all			Low immune cell specificity	Detected in all
		Low region specificity	Detected in all			Group enriched	Detected in some
		Low region specificity	Detected in all			Not detected in immune cells	Not detected
		Not detected	Not detected			Immune cell enhanced	Detected in some
		Low region specificity	Detected in all			Low immune cell specificity	Detected in all
	Head and Neck Squamous Cell Carcinoma (TCGA): 7.1	Low region specificity	Detected in all			Not detected in immune cells	Not detected
4	Cervical Squamous Cell Carcinoma and Endocervical Adenocarcinoma (TCGA): 8.1;Head and Neck Squamous Cell Carcinoma (TCGA): 19.4;Lung Squamous	Not detected	Not detected			Not detected in immune cells	Not detected
		Low region specificity	Detected in all			Immune cell enhanced	Detected in all
		Low region specificity	Detected in all			Low immune cell specificity	Detected in all
		Low region specificity	Detected in all			Immune cell enriched	Detected in all
		Low region specificity	Detected in all			Not detected in immune cells	Not detected
		Low region specificity	Detected in all			Immune cell enhanced	Detected in some
		Low region specificity	Detected in all			Not detected in immune cells	Not detected
		Low region specificity	Detected in all			Low immune cell specificity	Detected in all
		Low region specificity	Detected in all			Immune cell enhanced	Detected in many
	Testicular Germ Cell Tumor (TCGA): 25.3	Low region specificity	Detected in all			Low immune cell specificity	Detected in all
	Testicular Germ Cell Tumor (TCGA): 3.7	Low region specificity	Detected in all			Immune cell enhanced	Detected in many
		Low region specificity	Detected in all			Low immune cell specificity	Detected in many
		Low region specificity	Detected in all			Not detected in immune cells	Not detected
		Low region specificity	Detected in all			Low immune cell specificity	Detected in all
		Low region specificity	Detected in all			Low immune cell specificity	Detected in many
		Low region specificity	Detected in all			Immune cell enhanced	Detected in many
		Low region specificity	Detected in all			Low immune cell specificity	Detected in many
	Breast Invasive Carcinoma (TCGA): 20.0	Low region specificity	Detected in all			Not detected in immune cells	Not detected
		Low region specificity	Detected in all			Low immune cell specificity	Detected in all
		Low region specificity	Detected in all			Low immune cell specificity	Detected in many
	Kidney Renal Papillary Cell Carcinoma (TCGA): 130.9	Not detected	Not detected			Immune cell enriched	Detected in some
		Low region specificity	Detected in all			Low immune cell specificity	Detected in all
		Low region specificity	Detected in all			Immune cell enhanced	Detected in single
	Liver Hepatocellular Carcinoma (TCGA): 39.6	Low region specificity	Detected in all			Not detected in immune cells	Not detected
		Low region specificity	Detected in all			Low immune cell specificity	Detected in all
		Low region specificity	Detected in all			Not detected in immune cells	Not detected
		Low region specificity	Detected in all			Low immune cell specificity	Detected in all
		Low region specificity	Detected in all			Low immune cell specificity	Detected in all
		Low region specificity	Detected in all			Immune cell enhanced	Detected in many
	Stomach Adenocarcinoma (TCGA): 3.8	Low region specificity	Detected in all			Not detected in immune cells	Not detected
		Low region specificity	Detected in all			Immune cell enhanced	Detected in many
	Breast Invasive Carcinoma (TCGA): 12.3	Low region specificity	Detected in all			Not detected in immune cells	Not detected
4	Bladder Urothelial Carcinoma (TCGA): 107.5;Cervical Squamous Cell Carcinoma and Endocervical Adenocarcinoma (TCGA): 37.7;Uterine Corpus Endometrial	Low region specificity	Detected in many			Group enriched	Detected in some
		Low region specificity	Detected in all			Low immune cell specificity	Detected in all
	Cervical Squamous Cell Carcinoma and Endocervical Adenocarcinoma (TCGA): 9596.7;Head and Neck Squamous Cell Carcinoma (TCGA): 12365.7	Region enhanced	Detected in all		cerebral cortex: 97.1	Group enriched	Detected in all
	Thyroid Carcinoma (TCGA): 34.6	Not detected	Not detected			Not detected in immune cells	Not detected
7	Glioblastoma Multiforme (TCGA): 2.5	Low region specificity	Detected in all			Not detected in immune cells	Not detected
4	Glioblastoma Multiforme (TCGA): 6.0	Low region specificity	Detected in all			Not detected in immune cells	Not detected
		Low region specificity	Detected in many			Not detected in immune cells	Not detected
		Low region specificity	Detected in all			Not detected in immune cells	Not detected
		Low region specificity	Detected in all			Not detected in immune cells	Not detected
	Bladder Urothelial Carcinoma (TCGA): 15.0	Low region specificity	Detected in all			Not detected in immune cells	Not detected
		Low region specificity	Detected in all			Not detected in immune cells	Not detected
	Thyroid Carcinoma (TCGA): 12.7	Low region specificity	Detected in all			Not detected in immune cells	Not detected
		Low region specificity	Detected in all			Immune cell enriched	Detected in some
		Low region specificity	Detected in all			Low immune cell specificity	Detected in all
		Low region specificity	Detected in all			Low immune cell specificity	Detected in all
		Low region specificity	Detected in all			Low immune cell specificity	Detected in many
	Glioblastoma Multiforme (TCGA): 2.7;Ovary Serous Cystadenocarcinoma (TCGA): 3.4	Low region specificity	Detected in all			Not detected in immune cells	Not detected
		Low region specificity	Detected in all			Group enriched	Detected in many
		Low region specificity	Detected in all			Group enriched	Detected in many
		Low region specificity	Detected in all			Immune cell enhanced	Detected in many
		Low region specificity	Detected in all			Low immune cell specificity	Detected in some
		Low region specificity	Detected in all			Not detected in immune cells	Not detected
13	Liver Hepatocellular Carcinoma (TCGA): 91.3	Low region specificity	Detected in many			Not detected in immune cells	Not detected
	Bladder Urothelial Carcinoma (TCGA): 36.9	Low region specificity	Detected in all			Immune cell enhanced	Detected in some
		Low region specificity	Detected in all			Immune cell enhanced	Detected in many
4	Kidney Chromophobe (TCGA): 6.0;Testicular Germ Cell Tumor (TCGA): 24.0	Region enhanced	Detected in all		choroid plexus: 86.5	Immune cell enhanced	Detected in some
		Low region specificity	Detected in all			Immune cell enhanced	Detected in all
8	Kidney Chromophobe (TCGA): 37.2	Low region specificity	Detected in all			Low immune cell specificity	Detected in many
	Thyroid Carcinoma (TCGA): 73.6	Low region specificity	Detected in all			Low immune cell specificity	Detected in all
		Low region specificity	Detected in all			Immune cell enhanced	Detected in many
7	Bladder Urothelial Carcinoma (TCGA): 45.4	Region enriched	Detected in all	5	cerebellum: 159.2	Not detected in immune cells	Not detected
		Low region specificity	Detected in all			Low immune cell specificity	Detected in all
5	Breast Invasive Carcinoma (TCGA): 1.0;Glioblastoma Multiforme (TCGA): 2.6;Kidney Chromophobe (TCGA): 2.3	Low region specificity	Detected in all			Not detected in immune cells	Not detected
		Low region specificity	Detected in all			Low immune cell specificity	Detected in many
	Kidney Renal Clear Cell Carcinoma (TCGA): 101.5;Kidney Renal Papillary Cell Carcinoma (TCGA): 48.5	Low region specificity	Detected in some			Not detected in immune cells	Not detected
		Low region specificity	Detected in all			Low immune cell specificity	Detected in many
5	Prostate Adenocarcinoma (TCGA): 35.0	Region enriched	Detected in all	11	choroid plexus: 184.7	Not detected in immune cells	Not detected
11	Testicular Germ Cell Tumor (TCGA): 25.9	Low region specificity	Detected in all			Immune cell enhanced	Detected in some
9	Liver Hepatocellular Carcinoma (TCGA): 135.0	Low region specificity	Detected in many			Not detected in immune cells	Not detected
5	Testicular Germ Cell Tumor (TCGA): 269.8	Low region specificity	Detected in all			Group enriched	Detected in many
5	Glioblastoma Multiforme (TCGA): 33.2;Kidney Renal Clear Cell Carcinoma (TCGA): 55.3	Low region specificity	Detected in all			Immune cell enriched	Detected in some
		Low region specificity	Detected in all			Low immune cell specificity	Detected in some
28	Breast Invasive Carcinoma (TCGA): 24.5;Pancreatic Adenocarcinoma (TCGA): 39.5	Low region specificity	Detected in all			Not detected in immune cells	Not detected
10	Kidney Renal Clear Cell Carcinoma (TCGA): 3.9;Kidney Renal Papillary Cell Carcinoma (TCGA): 3.6	Not detected	Not detected			Not detected in immune cells	Not detected
	Lung Adenocarcinoma (TCGA): 764.9;Thyroid Carcinoma (TCGA): 986.8	Group enriched	Detected in many	6	cerebellum: 5.4;choroid plexus: 15.6	Not detected in immune cells	Not detected
	Glioblastoma Multiforme (TCGA): 24.7	Low region specificity	Detected in all			Immune cell enhanced	Detected in single
		Low region specificity	Detected in all			Low immune cell specificity	Detected in all
	Breast Invasive Carcinoma (TCGA): 645.3	Low region specificity	Detected in all			Low immune cell specificity	Detected in all
		Low region specificity	Detected in all			Low immune cell specificity	Detected in all
		Low region specificity	Detected in all			Immune cell enhanced	Detected in many
		Low region specificity	Detected in all			Low immune cell specificity	Detected in all
		Low region specificity	Detected in all			Low immune cell specificity	Detected in many
		Region enriched	Detected in all	13	choroid plexus: 1044.1	Low immune cell specificity	Detected in some
5	Glioblastoma Multiforme (TCGA): 40.3;Kidney Renal Clear Cell Carcinoma (TCGA): 36.9;Kidney Renal Papillary Cell Carcinoma (TCGA): 26.3;Pancreatic Adeno	Low region specificity	Detected in all			Not detected in immune cells	Not detected
		Low region specificity	Detected in all			Low immune cell specificity	Detected in some
	Rectum Adenocarcinoma (TCGA): 30.1	Low region specificity	Detected in many			Not detected in immune cells	Not detected
5	Kidney Renal Clear Cell Carcinoma (TCGA): 19.2	Low region specificity	Detected in all			Not detected in immune cells	Not detected
	Thyroid Carcinoma (TCGA): 32.2	Region enriched	Detected in all	4	choroid plexus: 208.2	Not detected in immune cells	Not detected
	Breast Invasive Carcinoma (TCGA): 55.8	Low region specificity	Detected in all			Not detected in immune cells	Not detected
	Skin Cutaneous Melanoma (TCGA): 327.6	Low region specificity	Detected in all			Immune cell enriched	Detected in some
		Low region specificity	Detected in all			Low immune cell specificity	Detected in all

		Low region specificity	Detected in all			Immune cell enhanced	Detected in some
		Low region specificity	Detected in all			Low immune cell specificity	Detected in some
	Kidney Chromophobe (TCGA): 12.0	Low region specificity	Detected in many			Not detected in immune cells	Not detected
		Low region specificity	Detected in all			Low immune cell specificity	Detected in many
		Low region specificity	Detected in all			Group enriched	Detected in some
		Low region specificity	Detected in all			Low immune cell specificity	Detected in all
		Low region specificity	Detected in many			Not detected in immune cells	Not detected
	Testicular Germ Cell Tumor (TCGA): 25.5	Low region specificity	Detected in all			Low immune cell specificity	Detected in single
84	Liver Hepatocellular Carcinoma (TCGA): 67.4	Low region specificity	Detected in some			Not detected in immune cells	Not detected
	Liver Hepatocellular Carcinoma (TCGA): 19.0	Region enriched	Detected in some	30	choroid plexus: 34.7	Not detected in immune cells	Not detected
		Low region specificity	Detected in all			Immune cell enhanced	Detected in single
	Prostate Adenocarcinoma (TCGA): 2.7	Low region specificity	Detected in many			Not detected in immune cells	Not detected
	Thyroid Carcinoma (TCGA): 36.8	Low region specificity	Detected in all			Immune cell enriched	Detected in single
	Bladder Urothelial Carcinoma (TCGA): 21.1;Breast Invasive Carcinoma (TCGA): 36.6	Low region specificity	Detected in many			Not detected in immune cells	Not detected
		Low region specificity	Detected in all			Low immune cell specificity	Detected in all
		Low region specificity	Detected in all			Immune cell enhanced	Detected in all
		Low region specificity	Detected in all			Low immune cell specificity	Detected in single
		Low region specificity	Detected in all			Not detected in immune cells	Not detected
		Low region specificity	Detected in all			Immune cell enhanced	Detected in many
		Low region specificity	Detected in all			Immune cell enriched	Detected in some
		Low region specificity	Detected in all			Immune cell enhanced	Detected in many
		Low region specificity	Detected in all			Low immune cell specificity	Detected in many
		Low region specificity	Detected in all			Immune cell enhanced	Detected in some
		Low region specificity	Detected in all			Low immune cell specificity	Detected in many
	Kidney Chromophobe (TCGA): 48.0	Low region specificity	Detected in all			Not detected in immune cells	Not detected
		Low region specificity	Detected in all			Low immune cell specificity	Detected in all
		Low region specificity	Detected in all			Low immune cell specificity	Detected in all
		Low region specificity	Detected in all			Immune cell enhanced	Detected in some
		Region enhanced	Detected in single		hypothalamus: 2.6	Not detected in immune cells	Not detected
		Low region specificity	Detected in all			Low immune cell specificity	Detected in all
	Prostate Adenocarcinoma (TCGA): 109.9	Region enriched	Detected in all	4	choroid plexus: 39.4	Immune cell enriched	Detected in some
		Low region specificity	Detected in all			Low immune cell specificity	Detected in all
		Low region specificity	Detected in all			Low immune cell specificity	Detected in all
		Low region specificity	Detected in all			Low immune cell specificity	Detected in all
	Uterine Corpus Endometrial Carcinoma (TCGA): 2.8	Low region specificity	Detected in many			Not detected in immune cells	Not detected
		Low region specificity	Detected in all			Low immune cell specificity	Detected in all
		Low region specificity	Detected in all			Immune cell enriched	Detected in many
		Low region specificity	Detected in all			Immune cell enhanced	Detected in many
		Low region specificity	Detected in all			Low immune cell specificity	Detected in all
		Low region specificity	Detected in all			Immune cell enhanced	Detected in many
		Low region specificity	Detected in all			Low immune cell specificity	Detected in all
		Low region specificity	Detected in all			Immune cell enriched	Detected in single
	Stomach Adenocarcinoma (TCGA): 11.3	Low region specificity	Detected in all			Immune cell enhanced	Detected in single
		Group enriched	Detected in some	4	choroid plexus: 3.9;white matter: 1.1	Immune cell enhanced	Detected in some
		Low region specificity	Detected in all			Immune cell enriched	Detected in single
		Low region specificity	Detected in all			Low immune cell specificity	Detected in all
	Kidney Chromophobe (TCGA): 17.4	Low region specificity	Detected in all			Not detected in immune cells	Not detected
		Low region specificity	Detected in all			Not detected in immune cells	Not detected
		Low region specificity	Detected in all			Immune cell enhanced	Detected in many
		Low region specificity	Detected in all			Low immune cell specificity	Detected in many
		Not detected	Not detected			Low immune cell specificity	Detected in some
		Low region specificity	Detected in many			Group enriched	Detected in some
	Kidney Renal Clear Cell Carcinoma (TCGA): 13.4	Low region specificity	Detected in all			Not detected in immune cells	Not detected
		Low region specificity	Detected in all			Low immune cell specificity	Detected in all
		Low region specificity	Detected in all			Low immune cell specificity	Detected in many
	Testicular Germ Cell Tumor (TCGA): 43.2	Region enriched	Detected in all	4	cerebellum: 330.3	Immune cell enhanced	Detected in many
		Low region specificity	Detected in all			Low immune cell specificity	Detected in many
		Low region specificity	Detected in all			Not detected in immune cells	Not detected
		Low region specificity	Detected in all			Immune cell enhanced	Detected in some
		Region enriched	Detected in many	9	choroid plexus: 16.2	Group enriched	Detected in many
		Low region specificity	Detected in all			Low immune cell specificity	Detected in all
		Low region specificity	Detected in all			Group enriched	Detected in many
		Low region specificity	Detected in all			Immune cell enhanced	Detected in many
5	Colon Adenocarcinoma (TCGA): 7.6;Liver Hepatocellular Carcinoma (TCGA): 7.3;Pancreatic Adenocarcinoma (TCGA): 9.1;Rectum Adenocarcinoma (TCGA): 8.	Low region specificity	Detected in all			Not detected in immune cells	Not detected
		Low region specificity	Detected in many			Not detected in immune cells	Not detected
		Region enriched	Detected in many	5	cerebellum: 18.2	Not detected in immune cells	Not detected
		Low region specificity	Detected in all			Not detected in immune cells	Not detected
		Low region specificity	Detected in all			Low immune cell specificity	Detected in single
5	Prostate Adenocarcinoma (TCGA): 644.6	Low region specificity	Detected in all			Not detected in immune cells	Not detected
	Stomach Adenocarcinoma (TCGA): 15.8	Low region specificity	Detected in all			Immune cell enriched	Detected in single
		Low region specificity	Detected in all			Low immune cell specificity	Detected in all
		Low region specificity	Detected in all			Immune cell enriched	Detected in some
10	Testicular Germ Cell Tumor (TCGA): 11.6	Group enriched	Detected in some	4	basal ganglia: 4.8;thalamus: 1.5	Group enriched	Detected in many
		Low region specificity	Detected in all			Immune cell enhanced	Detected in all
		Low region specificity	Detected in all			Low immune cell specificity	Detected in many
		Low region specificity	Detected in all			Low immune cell specificity	Detected in many
5	Kidney Renal Clear Cell Carcinoma (TCGA): 14.7;Kidney Renal Papillary Cell Carcinoma (TCGA): 25.9	Low region specificity	Detected in some			Not detected in immune cells	Not detected
		Low region specificity	Detected in many			Low immune cell specificity	Detected in many
		Not detected	Not detected			Immune cell enhanced	Detected in many
	Head and Neck Squamous Cell Carcinoma (TCGA): 7.0	Low region specificity	Detected in all			Not detected in immune cells	Not detected
	Glioblastoma Multiforme (TCGA): 3.0	Low region specificity	Detected in all			Immune cell enriched	Detected in some
5	Prostate Adenocarcinoma (TCGA): 154.3	Low region specificity	Detected in all			Low immune cell specificity	Detected in many
8	Skin Cutaneous Melanoma (TCGA): 162.4	Low region specificity	Detected in all			Low immune cell specificity	Detected in all
		Low region specificity	Detected in all			Immune cell enriched	Detected in many
	Liver Hepatocellular Carcinoma (TCGA): 1.4	Low region specificity	Detected in all			Not detected in immune cells	Not detected
		Low region specificity	Detected in all			Low immune cell specificity	Detected in all
	Testicular Germ Cell Tumor (TCGA): 76.6	Low region specificity	Detected in all			Low immune cell specificity	Detected in many
		Low region specificity	Detected in some			Immune cell enhanced	Detected in many
		Low region specificity	Detected in all			Low immune cell specificity	Detected in many
		Low region specificity	Detected in all			Immune cell enhanced	Detected in all
		Low region specificity	Detected in many			Immune cell enhanced	Detected in some
		Low region specificity	Detected in all			Low immune cell specificity	Detected in all
		Region enhanced	Detected in many		cerebellum: 10.3	Not detected in immune cells	Not detected
	Head and Neck Squamous Cell Carcinoma (TCGA): 22.6	Low region specificity	Detected in many			Immune cell enhanced	Detected in some
		Low region specificity	Detected in all			Low immune cell specificity	Detected in all
		Low region specificity	Detected in all			Immune cell enhanced	Detected in single
		Low region specificity	Detected in all			Immune cell enhanced	Detected in all
		Low region specificity	Detected in all			Low immune cell specificity	Detected in all
5	Colon Adenocarcinoma (TCGA): 245.1;Rectum Adenocarcinoma (TCGA): 254.5;Stomach Adenocarcinoma (TCGA): 96.1	Low region specificity	Detected in some			Immune cell enhanced	Detected in single
		Low region specificity	Detected in all			Low immune cell specificity	Detected in all
		Low region specificity	Detected in all			Low immune cell specificity	Detected in all
		Low region specificity	Detected in all			Low immune cell specificity	Detected in all
		Low region specificity	Detected in all			Low immune cell specificity	Detected in all
		Low region specificity	Detected in all			Immune cell enriched	Detected in many
		Low region specificity	Detected in all			Low immune cell specificity	Detected in single
		Low region specificity	Detected in all			Low immune cell specificity	Detected in many
		Low region specificity	Detected in all			Low immune cell specificity	Detected in many
		Low region specificity	Detected in all			Low immune cell specificity	Detected in all
37	Glioblastoma Multiforme (TCGA): 15.0	Low region specificity	Detected in all			Not detected in immune cells	Not detected
		Low region specificity	Detected in all			Low immune cell specificity	Detected in all
	Cervical Squamous Cell Carcinoma and Endocervical Adenocarcinoma (TCGA): 87.4	Low region specificity	Detected in all			Immune cell enhanced	Detected in many
		Low region specificity	Detected in all			Low immune cell specificity	Detected in all

RNA blood cell specificity score	RNA blood cell specific nTPM	RNA blood lineage specificity	RNA blood lineage distribution	RNA blood lineage specificity score	RNA blood lineage specific nTPM	RNA cell line specificity	RNA cell line distribution	RNA cell line specificity score
		Low lineage specificity	Detected in many			Low cancer specificity	Detected in all	
		Low lineage specificity	Detected in all			Low cancer specificity	Detected in all	
5	eosinophil: 13.1;neutrophil: 10.7	Lineage enriched	Detected in many	8	granulocytes: 13.1	Low cancer specificity	Detected in many	
		Low lineage specificity	Detected in many			Low cancer specificity	Detected in many	
	MAIT T-cell: 27.2;NK-cell: 17.9	Group enriched	Detected in many	9	NK-cells: 17.9;T-cells: 27.2	Cancer enhanced	Detected in many	
5	memory B-cell: 4.4;naive B-cell: 8.2	Lineage enriched	Detected in many	7	B-cells: 8.2	Cancer enhanced	Detected in some	
		Low lineage specificity	Detected in many			Low cancer specificity	Detected in all	
		Not detected	Not detected			Low cancer specificity	Detected in all	
	intermediate monocyte: 5.1;non-classical monocyte: 6.4	Lineage enriched	Detected in many	5	monocytes: 6.4	Low cancer specificity	Detected in many	
		Low lineage specificity	Detected in many			Cancer enhanced	Detected in all	
5	classical monocyte: 3.9	Lineage enriched	Detected in single	5	monocytes: 3.9	Cancer enhanced	Detected in many	
	MAIT T-cell: 2.2	Low lineage specificity	Detected in many			Low cancer specificity	Detected in many	
		Group enriched	Detected in many	7	NK-cells: 4.7;T-cells: 3.8	Cancer enhanced	Detected in many	
4	intermediate monocyte: 163.0;NK-cell: 90.4;non-classical monocyte: 351.2;plasmacytoid DC: 211.2	Group enriched	Detected in all	4	dendritic cells: 211.2;monocytes: 351.2;NK-cells: 90.4	Cancer enhanced	Detected in all	
		Low lineage specificity	Detected in all			Low cancer specificity	Detected in all	
5	MAIT T-cell: 4.5	Lineage enriched	Detected in single	45	T-cells: 4.5	Cancer enhanced	Detected in many	
	memory B-cell: 72.0;naive B-cell: 87.4	Low lineage specificity	Detected in many			Cancer enhanced	Detected in many	
		Low lineage specificity	Detected in all			Low cancer specificity	Detected in all	
9	basophil: 2.9	Lineage enriched	Detected in single	9	granulocytes: 2.9	Not detected	Not detected	
		Not detected	Not detected			Low cancer specificity	Detected in many	
		Low lineage specificity	Detected in all			Low cancer specificity	Detected in all	
	memory B-cell: 2.3;non-classical monocyte: 2.3	Low lineage specificity	Detected in many			Cancer enhanced	Detected in many	
		Low lineage specificity	Detected in all			Low cancer specificity	Detected in all	
		Not detected	Not detected			Cancer enhanced	Detected in many	
		Not detected	Not detected			Low cancer specificity	Detected in many	
6	classical monocyte: 2.0;intermediate monocyte: 2.3;myeloid DC: 1.1;non-classical monocyte: 1.9	Group enriched	Detected in many	6	dendritic cells: 1.1;monocytes: 2.3	Low cancer specificity	Detected in many	
		Not detected	Not detected			Cancer enhanced	Detected in many	
		Not detected	Not detected			Cancer enhanced	Detected in many	
	basophil: 17.3;myeloid DC: 10.5	Group enriched	Detected in many	113	dendritic cells: 10.5;granulocytes: 17.3;monocytes: 5.9	Low cancer specificity	Detected in many	
		Not detected	Not detected			Low cancer specificity	Detected in many	
		Low lineage specificity	Detected in all			Cancer enhanced	Detected in many	
		Low lineage specificity	Detected in single			Low cancer specificity	Detected in all	
		Not detected	Not detected			Cancer enhanced	Detected in some	
		Low lineage specificity	Detected in all			Low cancer specificity	Detected in all	
		Not detected	Not detected			Low cancer specificity	Detected in many	
236	basophil: 37.3	Lineage enriched	Detected in single	236	granulocytes: 37.3	Low cancer specificity	Detected in all	
		Not detected	Not detected			Cancer enriched	Detected in many	4
		Not detected	Not detected			Cancer enhanced	Detected in many	
		Low lineage specificity	Detected in all			Low cancer specificity	Detected in all	
		Not detected	Not detected			Cancer enriched	Detected in some	9
8	classical monocyte: 3.9;myeloid DC: 4.3	Group enriched	Detected in many	8	dendritic cells: 4.3;monocytes: 3.9	Cancer enriched	Detected in some	10
		Not detected	Not detected			Low cancer specificity	Detected in all	
		Not detected	Not detected			Cancer enriched	Detected in some	5
		Not detected	Not detected			Cancer enhanced	Detected in many	
		Not detected	Not detected			Cancer enhanced	Detected in many	
		Low lineage specificity	Detected in many			Low cancer specificity	Detected in many	
		Low lineage specificity	Detected in many			Low cancer specificity	Detected in all	
		Low lineage specificity	Detected in all			Low cancer specificity	Detected in all	
		Low lineage specificity	Detected in many			Low cancer specificity	Detected in all	
		Not detected	Not detected			Low cancer specificity	Detected in all	
		Not detected	Not detected			Cancer enhanced	Detected in many	
		Low lineage specificity	Detected in all			Low cancer specificity	Detected in all	
5	basophil: 1.3	Lineage enriched	Detected in single	13	granulocytes: 1.3	Cancer enhanced	Detected in many	
		Low lineage specificity	Detected in all			Low cancer specificity	Detected in many	
4	neutrophil: 227.8	Lineage enriched	Detected in many	4	granulocytes: 227.8	Cancer enhanced	Detected in many	
	basophil: 2824.3	Low lineage specificity	Detected in all			Low cancer specificity	Detected in all	
		Not detected	Not detected			Cancer enhanced	Detected in many	
328	neutrophil: 208.7	Lineage enriched	Detected in single	328	granulocytes: 208.7	Low cancer specificity	Detected in many	
		Low lineage specificity	Detected in all			Low cancer specificity	Detected in all	
	naive CD4 T-cell: 85.7	Lineage enriched	Detected in all	5	T-cells: 85.7	Low cancer specificity	Detected in all	
	neutrophil: 183.4	Low lineage specificity	Detected in all			Group enriched	Detected in many	5
		Low lineage specificity	Detected in all			Low cancer specificity	Detected in all	
		Low lineage specificity	Detected in many			Cancer enriched	Detected in some	7
		Low lineage specificity	Detected in many			Cancer enriched	Detected in many	9
	naive B-cell: 28.4	Low lineage specificity	Detected in all			Low cancer specificity	Detected in all	
		Not detected	Not detected			Cancer enhanced	Detected in many	
		Not detected	Not detected			Cancer enhanced	Detected in some	
		Not detected	Not detected			Low cancer specificity	Detected in all	
	MAIT T-cell: 182.0;T-reg: 205.9	Lineage enriched	Detected in many	18	T-cells: 205.9	Cancer enhanced	Detected in many	
		Not detected	Not detected			Group enriched	Detected in some	4
		Low lineage specificity	Detected in all			Low cancer specificity	Detected in all	
		Low lineage specificity	Detected in all			Group enriched	Detected in some	6
		Low lineage specificity	Detected in all			Low cancer specificity	Detected in all	
		Low lineage specificity	Detected in all			Low cancer specificity	Detected in all	
		Not detected	Not detected			Low cancer specificity	Detected in all	
	classical monocyte: 1.1	Low lineage specificity	Detected in single			Low cancer specificity	Detected in many	
		Low lineage specificity	Detected in all			Group enriched	Detected in many	4
		Low lineage specificity	Detected in all			Low cancer specificity	Detected in all	
		Not detected	Not detected			Low cancer specificity	Detected in all	
		Not detected	Not detected			Cancer enriched	Detected in single	7
		Low lineage specificity	Detected in many			Low cancer specificity	Detected in all	
		Not detected	Not detected			Low cancer specificity	Detected in many	
	T-reg: 1.8	Low lineage specificity	Detected in single			Low cancer specificity	Detected in many	
		Low lineage specificity	Detected in all			Cancer enhanced	Detected in all	
	basophil: 8.8	Low lineage specificity	Detected in many			Cancer enhanced	Detected in some	
		Low lineage specificity	Detected in all			Low cancer specificity	Detected in all	
5	basophil: 1.1;classical monocyte: 2.5;eosinophil: 1.5;intermediate monocyte: 1.7;myeloid DC: 1.1;neu	Group enriched	Detected in many	8	dendritic cells: 1.1;granulocytes: 1.5;monocytes: 2.5	Low cancer specificity	Detected in many	
		Not detected	Not detected			Cancer enhanced	Detected in many	
7	basophil: 2.7	Lineage enriched	Detected in single	7	granulocytes: 2.7	Low cancer specificity	Detected in all	
8	classical monocyte: 110.6;myeloid DC: 64.9	Group enriched	Detected in many	52	dendritic cells: 64.9;monocytes: 110.6	Cancer enriched	Detected in some	12
		Low lineage specificity	Detected in all			Low cancer specificity	Detected in all	
		Not detected	Not detected			Low cancer specificity	Detected in many	
		Low lineage specificity	Detected in all			Low cancer specificity	Detected in all	
12	basophil: 84.5	Lineage enriched	Detected in all	12	granulocytes: 84.5	Low cancer specificity	Detected in all	
		Not detected	Not detected			Cancer enriched	Detected in single	6
		Low lineage specificity	Detected in all			Low cancer specificity	Detected in all	
4	neutrophil: 1.6	Lineage enriched	Detected in single	4	granulocytes: 1.6	Low cancer specificity	Detected in some	
		Low lineage specificity	Detected in all			Low cancer specificity	Detected in all	
		Not detected	Not detected			Cancer enhanced	Detected in some	
		Low lineage specificity	Detected in many			Cancer enriched	Detected in all	4
	eosinophil: 13.5	Low lineage specificity	Detected in all			Low cancer specificity	Detected in all	
		Low lineage specificity	Detected in all			Low cancer specificity	Detected in all	
		Not detected	Not detected			Cancer enriched	Detected in some	4
		Not detected	Not detected			Low cancer specificity	Detected in many	
	T-reg: 4.9	Group enriched	Detected in many	6	granulocytes: 2.7;T-cells: 4.9	Cancer enhanced	Detected in all	
	NK-cell: 11.3;plasmacytoid DC: 25.8	Group enriched	Detected in many	12	dendritic cells: 25.8;NK-cells: 11.3	Cancer enhanced	Detected in many	
		Low lineage specificity	Detected in all			Low cancer specificity	Detected in all	
		Not detected	Not detected			Low cancer specificity	Detected in all	
	plasmacytoid DC: 21.3	Low lineage specificity	Detected in many			Low cancer specificity	Detected in all	
		Not detected	Not detected			Low cancer specificity	Detected in all	
5	neutrophil: 128.9	Lineage enriched	Detected in many	16	granulocytes: 128.9	Cancer enhanced	Detected in all	
		Not detected	Not detected			Cancer enhanced	Detected in many	
	gdT-cell: 2.5	Lineage enhanced	Detected in single		T-cells: 2.5	Low cancer specificity	Detected in many	
		Low lineage specificity	Detected in all			Group enriched	Detected in some	5
9	memory B-cell: 111.3;naive B-cell: 94.9;plasmacytoid DC: 159.3	Group enriched	Detected in many	83	B-cells: 111.3;dendritic cells: 159.3	Cancer enhanced	Detected in many	
		Not detected	Not detected			Low cancer specificity	Detected in all	
16	neutrophil: 7.6	Lineage enriched	Detected in single	77	granulocytes: 7.6	Group enriched	Detected in some	6
		Not detected	Not detected			Low cancer specificity	Detected in many	
		Low lineage specificity	Detected in all			Low cancer specificity	Detected in all	
4	basophil: 515.1	Lineage enriched	Detected in many	4	granulocytes: 515.1	Group enriched	Detected in some	93
		Not detected	Not detected			Cancer enhanced	Detected in many	
		Not detected	Not detected			Low cancer specificity	Detected in many	
		Not detected	Not detected			Cancer enhanced	Detected in many	
		Low lineage specificity	Detected in all			Low cancer specificity	Detected in all	
		Low lineage specificity	Detected in many			Low cancer specificity	Detected in all	
		Not detected	Not detected			Not detected	Not detected	

		Not detected	Not detected			Cancer enhanced	Detected in many	
		Not detected	Not detected			Cancer enhanced	Detected in some	
	gdT-cell: 8.0;myeloid DC: 8.1	Group enriched	Detected in many	5	dendritic cells: 8.1;T-cells: 8.0	Low cancer specificity	Detected in many	
4	classical monocyte: 2.6;myeloid DC: 9.2;plasmacytoid DC: 2.6	Group enriched	Detected in many	6	dendritic cells: 9.2;monocytes: 2.6	Cancer enhanced	Detected in many	
8	neutrophil: 1.9	Lineage enriched	Detected in single	8	granulocytes: 1.9	Low cancer specificity	Detected in all	
		Low lineage specificity	Detected in many			Low cancer specificity	Detected in all	
		Not detected	Not detected			Low cancer specificity	Detected in all	
		Low lineage specificity	Detected in all			Low cancer specificity	Detected in all	
		Not detected	Not detected			Low cancer specificity	Detected in many	
12	basophil: 48.8;neutrophil: 24.3	Lineage enriched	Detected in many	15	granulocytes: 48.8	Cancer enhanced	Detected in many	
		Not detected	Not detected			Cancer enhanced	Detected in many	
		Low lineage specificity	Detected in all			Low cancer specificity	Detected in all	
		Low lineage specificity	Detected in all			Low cancer specificity	Detected in all	
		Not detected	Not detected			Low cancer specificity	Detected in all	
5	basophil: 6.1;classical monocyte: 7.1;intermediate monocyte: 7.0;myeloid DC: 11.3;non-classical mo	Group enriched	Detected in all	6	dendritic cells: 23.7;granulocytes: 6.1;monocytes: 7.1	Low cancer specificity	Detected in all	
		Low lineage specificity	Detected in all			Cancer enhanced	Detected in all	
		Low lineage specificity	Detected in all			Low cancer specificity	Detected in all	
		Not detected	Not detected			Group enriched	Detected in some	5
		Not detected	Not detected			Group enriched	Detected in some	6
		Low lineage specificity	Detected in many			Low cancer specificity	Detected in all	
	basophil: 557.4	Low lineage specificity	Detected in all			Cancer enriched	Detected in some	4
5	T-reg: 60.8	Lineage enriched	Detected in many	41	T-cells: 60.8	Cancer enriched	Detected in some	4
		Low lineage specificity	Detected in all			Low cancer specificity	Detected in many	
		Not detected	Not detected			Low cancer specificity	Detected in all	
11	classical monocyte: 76.1;myeloid DC: 65.3	Group enriched	Detected in many	40	dendritic cells: 65.3;monocytes: 76.1	Cancer enriched	Detected in single	9
	memory B-cell: 77.2;naive B-cell: 79.2	Group enriched	Detected in many	25	B-cells: 79.2;dendritic cells: 23.5;monocytes: 34.7	Group enriched	Detected in some	4
6	gdT-cell: 422.7;MAIT T-cell: 403.9;memory CD4 T-cell: 611.9;memory CD8 T-cell: 713.9;naive CD4 T-c	Group enriched	Detected in all	10	NK-cells: 1110.8;T-cells: 713.9	Group enriched	Detected in some	66
4	basophil: 235.9	Lineage enriched	Detected in all	10	granulocytes: 235.9	Cancer enhanced	Detected in some	
7	gdT-cell: 36.7;MAIT T-cell: 13.4;memory CD4 T-cell: 28.9;memory CD8 T-cell: 22.5;naive CD4 T-cell: 1	Group enriched	Detected in many	11	NK-cells: 29.7;T-cells: 36.7	Group enriched	Detected in some	6
	basophil: 6.7	Lineage enriched	Detected in many	5	granulocytes: 6.7	Low cancer specificity	Detected in many	
	T-reg: 102.2	Lineage enriched	Detected in many	7	T-cells: 102.2	Cancer enriched	Detected in some	13
	plasmacytoid DC: 9.9	Low lineage specificity	Detected in many			Low cancer specificity	Detected in all	
7	classical monocyte: 166.7;intermediate monocyte: 122.1;myeloid DC: 213.7;non-classical monocyte:	Group enriched	Detected in many	9	dendritic cells: 213.7;monocytes: 166.7	Cancer enriched	Detected in many	8
	NK-cell: 18.3	Group enriched	Detected in all	5	B-cells: 9.9;dendritic cells: 5.8;NK-cells: 18.3	Group enriched	Detected in some	15
26	gdT-cell: 491.6;MAIT T-cell: 421.5;memory CD4 T-cell: 415.6;memory CD8 T-cell: 463.4;naive CD4 T-c	Group enriched	Detected in all	21	NK-cells: 203.3;T-cells: 502.8	Cancer enriched	Detected in some	8
21	gdT-cell: 228.7;MAIT T-cell: 135.3;memory CD4 T-cell: 187.4;memory CD8 T-cell: 196.4;naive CD4 T-c	Lineage enriched	Detected in many	26	T-cells: 241.8	Cancer enriched	Detected in some	6
18	classical monocyte: 247.4;intermediate monocyte: 241.4;memory CD4 T-cell: 153.8;myeloid DC: 166	Group enriched	Detected in many	20	dendritic cells: 264.3;monocytes: 247.4;T-cells: 154.9	Group enriched	Detected in some	35
	memory B-cell: 108.3;naive B-cell: 123.7	Group enriched	Detected in all	6	B-cells: 123.7;monocytes: 34.9	Cancer enhanced	Detected in many	
5	gdT-cell: 26.4;MAIT T-cell: 64.4;memory CD4 T-cell: 80.5;naive CD4 T-cell: 76.3	Lineage enriched	Detected in many	34	T-cells: 80.5	Cancer enriched	Detected in single	7
		Low lineage specificity	Detected in all			Cancer enhanced	Detected in all	
		Low lineage specificity	Detected in all			Low cancer specificity	Detected in all	
4	memory B-cell: 1119.4;naive B-cell: 1488.8	Lineage enriched	Detected in all	5	B-cells: 1488.8	Cancer enriched	Detected in some	6
		Low lineage specificity	Detected in all			Group enriched	Detected in some	61
8	classical monocyte: 80.1;intermediate monocyte: 79.2;memory B-cell: 41.1;myeloid DC: 79.0;naive B	Group enriched	Detected in many	18	B-cells: 41.1;dendritic cells: 79.0;monocytes: 85.6	Cancer enriched	Detected in some	6
14	MAIT T-cell: 227.9;memory CD8 T-cell: 509.4;naive CD8 T-cell: 461.5	Lineage enriched	Detected in many	19	T-cells: 509.4	Group enriched	Detected in some	9
10	memory CD8 T-cell: 602.5;naive CD8 T-cell: 1058.1	Lineage enriched	Detected in all	25	T-cells: 1058.1	Group enriched	Detected in some	9
26	basophil: 1961.8;eosinophil: 1159.2	Lineage enriched	Detected in all	32	granulocytes: 1961.8	Low cancer specificity	Detected in all	
	basophil: 1.0	Lineage enriched	Detected in single	5	granulocytes: 1.0	Low cancer specificity	Detected in all	
8	classical monocyte: 7.3;intermediate monocyte: 1.9;myeloid DC: 2.4	Group enriched	Detected in many	10	dendritic cells: 2.4;monocytes: 7.3	Low cancer specificity	Detected in all	
		Low lineage specificity	Detected in all			Low cancer specificity	Detected in all	
		Low lineage specificity	Detected in all			Low cancer specificity	Detected in all	
13	myeloid DC: 1.7;plasmacytoid DC: 4.7	Lineage enriched	Detected in single	19	dendritic cells: 4.7	Low cancer specificity	Detected in many	
	non-classical monocyte: 4.3	Group enriched	Detected in many	5	granulocytes: 1.4;monocytes: 4.3	Cancer enhanced	Detected in many	
		Not detected	Not detected			Cancer enriched	Detected in some	18
5	neutrophil: 14.8	Lineage enriched	Detected in single	18	granulocytes: 14.8	Group enriched	Detected in many	5
5	basophil: 1.7;neutrophil: 1.3	Lineage enriched	Detected in single	6	granulocytes: 1.7	Low cancer specificity	Detected in many	
		Not detected	Not detected			Cancer enhanced	Detected in some	
		Not detected	Not detected			Cancer enhanced	Detected in many	
		Not detected	Not detected			Cancer enhanced	Detected in many	
		Not detected	Not detected			Cancer enhanced	Detected in many	
		Not detected	Not detected			Cancer enhanced	Detected in many	
		Low lineage specificity	Detected in all			Low cancer specificity	Detected in all	
		Low lineage specificity	Detected in all			Low cancer specificity	Detected in all	
		Not detected	Not detected			Cancer enhanced	Detected in many	
		Not detected	Not detected			Cancer enhanced	Detected in some	
5	memory B-cell: 2.2;naive B-cell: 1.9	Lineage enriched	Detected in single	6	B-cells: 2.2	Low cancer specificity	Detected in all	
		Low lineage specificity	Detected in many			Low cancer specificity	Detected in all	
4	neutrophil: 394.0	Lineage enriched	Detected in all	6	granulocytes: 394.0	Low cancer specificity	Detected in all	
		Not detected	Not detected			Low cancer specificity	Detected in all	
		Not detected	Not detected			Cancer enhanced	Detected in many	
		Lineage enriched	Detected in all	4	granulocytes: 366.4	Low cancer specificity	Detected in all	
		Not detected	Not detected			Cancer enhanced	Detected in many	
		Not detected	Not detected			Low cancer specificity	Detected in many	
		Not detected	Not detected			Cancer enriched	Detected in some	16
		Not detected	Not detected			Cancer enriched	Detected in single	36
		Not detected	Not detected			Cancer enriched	Detected in some	20
		Not detected	Not detected			Low cancer specificity	Detected in all	
		Not detected	Not detected			Cancer enriched	Detected in single	35
		Not detected	Not detected			Cancer enriched	Detected in single	27
		Low lineage specificity	Detected in all			Low cancer specificity	Detected in all	
		Not detected	Not detected			Group enriched	Detected in some	4
	myeloid DC: 2.6;plasmacytoid DC: 2.8	Low lineage specificity	Detected in single			Low cancer specificity	Detected in all	
		Not detected	Not detected			Cancer enhanced	Detected in many	
		Not detected	Not detected			Cancer enhanced	Detected in some	
		Not detected	Not detected			Cancer enhanced	Detected in many	
		Not detected	Not detected			Low cancer specificity	Detected in some	
		Not detected	Not detected			Cancer enhanced	Detected in many	
		Not detected	Not detected			Cancer enhanced	Detected in many	
		Low lineage specificity	Detected in single			Low cancer specificity	Detected in many	
		Not detected	Not detected			Low cancer specificity	Detected in many	
8	classical monocyte: 9.1	Lineage enriched	Detected in many	8	monocytes: 9.1	Cancer enriched	Detected in single	128
5	intermediate monocyte: 18.6;myeloid DC: 48.0;non-classical monocyte: 14.4	Group enriched	Detected in many	16	dendritic cells: 48.0;monocytes: 18.6	Cancer enhanced	Detected in many	
	memory B-cell: 13.5;naive B-cell: 11.9	Low lineage specificity	Detected in many			Low cancer specificity	Detected in all	
	MAIT T-cell: 5.1	Lineage enriched	Detected in single	17	T-cells: 5.1	Cancer enhanced	Detected in many	
		Low lineage specificity	Detected in all			Low cancer specificity	Detected in all	
		Low lineage specificity	Detected in all			Low cancer specificity	Detected in all	
		Not detected	Not detected			Low cancer specificity	Detected in many	
		Not detected	Not detected			Low cancer specificity	Detected in many	
		Low lineage specificity	Detected in single			Low cancer specificity	Detected in all	
		Not detected	Not detected			Group enriched	Detected in some	4
		Low lineage specificity	Detected in all			Low cancer specificity	Detected in all	
		Not detected	Not detected			Cancer enhanced	Detected in many	
6	gdT-cell: 4.5;MAIT T-cell: 3.1;memory CD8 T-cell: 2.4;naive CD8 T-cell: 1.2	Lineage enriched	Detected in single	13	T-cells: 4.5	Cancer enhanced	Detected in many	
	non-classical monocyte: 222.9	Low lineage specificity	Detected in all			Low cancer specificity	Detected in all	
	neutrophil: 676.1	Low lineage specificity	Detected in all			Low cancer specificity	Detected in all	
6	basophil: 53.8;eosinophil: 17.2;neutrophil: 15.6	Lineage enriched	Detected in many	12	granulocytes: 53.8	Group enriched	Detected in some	9
	naive CD4 T-cell: 9.2	Group enriched	Detected in many	21	B-cells: 3.1;T-cells: 9.2	Low cancer specificity	Detected in many	
		Not detected	Not detected			Cancer enriched	Detected in single	6
		Not detected	Not detected			Cancer enhanced	Detected in many	
13	basophil: 13.2;eosinophil: 22.3	Lineage enriched	Detected in many	16	granulocytes: 22.3	Low cancer specificity	Detected in all	
	intermediate monocyte: 444.5;non-classical monocyte: 650.1	Lineage enriched	Detected in many	5	monocytes: 650.1	Cancer enhanced	Detected in many	
		Low lineage specificity	Detected in all			Low cancer specificity	Detected in all	
		Low lineage specificity	Detected in many			Low cancer specificity	Detected in all	
		Low lineage specificity	Detected in all			Low cancer specificity	Detected in all	
5	classical monocyte: 4.8;intermediate monocyte: 9.4;myeloid DC: 6.4;non-classical monocyte: 5.8	Group enriched	Detected in many	19	dendritic cells: 6.4;monocytes: 9.4	Low cancer specificity	Detected in all	
	basophil: 1.2;neutrophil: 2.1	Lineage enriched	Detected in single	5	granulocytes: 2.1	Low cancer specificity	Detected in all	
		Not detected	Not detected			Low cancer specificity	Detected in many	
	non-classical monocyte: 451.3	Low lineage specificity	Detected in many			Cancer enriched	Detected in some	6
		Not detected	Not detected			Low cancer specificity	Detected in all	
52	neutrophil: 3297.5	Lineage enriched	Detected in many	109	granulocytes: 3297.5	Cancer enriched	Detected in single	9
4	classical monocyte: 17.4;intermediate monocyte: 17.8;myeloid DC: 16.8;neutrophil: 9.8;non-classical	Group enriched	Detected in many	4	dendritic cells: 16.8;granulocytes: 9.8;monocytes: 17.8	Low cancer specificity	Detected in many	
		Low lineage specificity	Detected in all			Low cancer specificity	Detected in all	
		Low lineage specificity	Detected in all			Group enriched	Detected in some	13
8	plasmacytoid DC: 48.8	Lineage enriched	Detected in many	8	dendritic cells: 48.8	Low cancer specificity	Detected in many	
		Low lineage specificity	Detected in many			Low cancer specificity	Detected in all	
		Low lineage specificity	Detected in all			Cancer enhanced	Detected in all	
		Low lineage specificity	Detected in all			Low cancer specificity	Detected in all	
		Not detected	Not detected			Cancer enriched	Detected in some	26
		Not detected	Not detected			Low cancer specificity	Detected in all	

		Not detected	Not detected			Cancer enhanced	Detected in many	
		Not detected	Not detected			Low cancer specificity	Detected in all	
		Not detected	Not detected			Low cancer specificity	Detected in all	
		Low lineage specificity	Detected in all			Low cancer specificity	Detected in all	
		Low lineage specificity	Detected in many			Low cancer specificity	Detected in all	
		Not detected	Not detected			Low cancer specificity	Detected in all	
		Not detected	Not detected			Low cancer specificity	Detected in many	
		Low lineage specificity	Detected in many			Low cancer specificity	Detected in all	
		Low lineage specificity	Detected in all			Low cancer specificity	Detected in all	
		Not detected	Not detected			Low cancer specificity	Detected in many	
		Low lineage specificity	Detected in all			Group enriched	Detected in many	5
	eosinophil: 1568.8	Low lineage specificity	Detected in all			Group enriched	Detected in some	49
	neutrophil: 134.3	Group enriched	Detected in many	6	B-cells: 37.3;granulocytes: 134.3;monocytes: 78.1	Group enriched	Detected in all	6
		Not detected	Not detected			Cancer enhanced	Detected in many	5
		Not detected	Not detected			Not detected	Not detected	
9	eosinophil: 29.9;neutrophil: 8.4	Lineage enriched	Detected in many	14	granulocytes: 29.9	Low cancer specificity	Detected in many	
		Not detected	Not detected			Group enriched	Detected in many	5
12	basophil: 8.7	Lineage enriched	Detected in single	12	granulocytes: 8.7	Low cancer specificity	Detected in all	
16	naive B-cell: 2.3	Lineage enriched	Detected in single	24	B-cells: 2.3	Low cancer specificity	Detected in many	
		Group enriched	Detected in many	4	dendritic cells: 3.4;T-cells: 4.4	Low cancer specificity	Detected in many	
6	neutrophil: 51.7	Lineage enriched	Detected in many	6	granulocytes: 51.7	Low cancer specificity	Detected in many	
		Not detected	Not detected			Cancer enhanced	Detected in many	
		Not detected	Not detected			Low cancer specificity	Detected in many	
		Not detected	Not detected			Group enriched	Detected in many	12
	myeloid DC: 3.5	Low lineage specificity	Detected in many			Low cancer specificity	Detected in all	
		Low lineage specificity	Detected in all			Low cancer specificity	Detected in all	
	basophil: 1.3	Lineage enriched	Detected in single	5	granulocytes: 1.3	Low cancer specificity	Detected in many	
		Not detected	Not detected			Low cancer specificity	Detected in many	
		Low lineage specificity	Detected in all			Low cancer specificity	Detected in all	
		Low lineage specificity	Detected in all			Low cancer specificity	Detected in all	
		Not detected	Not detected			Low cancer specificity	Detected in many	
5	memory B-cell: 20.9;naive B-cell: 5.6	Lineage enriched	Detected in many	7	B-cells: 20.9	Low cancer specificity	Detected in many	
7	neutrophil: 65.7	Lineage enriched	Detected in many	7	granulocytes: 65.7	Cancer enhanced	Detected in many	
		Not detected	Not detected			Low cancer specificity	Detected in all	
5	classical monocyte: 19.9;intermediate monocyte: 28.2;myeloid DC: 10.3;NK-cell: 7.6;non-classical m	Group enriched	Detected in many	4	dendritic cells: 10.3;monocytes: 28.2;NK-cells: 7.6	Low cancer specificity	Detected in all	
		Low lineage specificity	Detected in all			Low cancer specificity	Detected in all	
4	gdT-cell: 9.9;MAIT T-cell: 11.4;memory B-cell: 6.8;memory CD4 T-cell: 13.1;memory CD8 T-cell: 11.4;	Group enriched	Detected in many	7	B-cells: 6.8;granulocytes: 7.0;T-cells: 13.1	Low cancer specificity	Detected in all	
		Low lineage specificity	Detected in all			Low cancer specificity	Detected in all	
		Not detected	Not detected			Cancer enhanced	Detected in many	
		Low lineage specificity	Detected in all			Low cancer specificity	Detected in all	
10	plasmacytoid DC: 1.0	Lineage enriched	Detected in single	10	dendritic cells: 1.0	Low cancer specificity	Detected in all	
		Low lineage specificity	Detected in many			Low cancer specificity	Detected in all	
	intermediate monocyte: 7.7;non-classical monocyte: 12.5	Lineage enriched	Detected in many	5	monocytes: 12.5	Low cancer specificity	Detected in many	
		Low lineage specificity	Detected in many			Low cancer specificity	Detected in all	
		Not detected	Not detected			Cancer enhanced	Detected in many	
94	plasmacytoid DC: 9.4	Lineage enriched	Detected in single	94	dendritic cells: 9.4	Low cancer specificity	Detected in many	
		Not detected	Not detected			Cancer enhanced	Detected in many	
		Not detected	Not detected			Cancer enhanced	Detected in many	
23	neutrophil: 10.1;plasmacytoid DC: 23.3	Group enriched	Detected in many	23	dendritic cells: 23.3;granulocytes: 10.1	Cancer enhanced	Detected in many	
		Not detected	Not detected			Low cancer specificity	Detected in all	
		Low lineage specificity	Detected in many			Low cancer specificity	Detected in all	
	gdT-cell: 5.0	Group enriched	Detected in many	5	granulocytes: 1.2;T-cells: 5.0	Group enriched	Detected in all	5
	basophil: 1.0	Lineage enriched	Detected in single	5	granulocytes: 1.0	Low cancer specificity	Detected in many	
		Low lineage specificity	Detected in all			Low cancer specificity	Detected in all	
		Low lineage specificity	Detected in all			Low cancer specificity	Detected in all	
		Low lineage specificity	Detected in all			Cancer enhanced	Detected in many	
		Low lineage specificity	Detected in all			Low cancer specificity	Detected in all	
4	eosinophil: 166.2;neutrophil: 114.1	Lineage enriched	Detected in all	5	granulocytes: 166.2	Cancer enhanced	Detected in all	
		Low lineage specificity	Detected in many			Cancer enhanced	Detected in some	
	neutrophil: 101.1	Group enriched	Detected in all	5	granulocytes: 101.1;T-cells: 90.6	Low cancer specificity	Detected in all	
		Not detected	Not detected			Low cancer specificity	Detected in all	
	basophil: 1.5	Low lineage specificity	Detected in single			Low cancer specificity	Detected in many	
		Not detected	Not detected			Low cancer specificity	Detected in many	
8	classical monocyte: 1.3;neutrophil: 4.1	Group enriched	Detected in many	27	granulocytes: 4.1;monocytes: 1.3	Not detected	Not detected	
6	classical monocyte: 137.1;intermediate monocyte: 57.3;myeloid DC: 37.3;neutrophil: 98.7	Group enriched	Detected in many	55	dendritic cells: 37.3;granulocytes: 98.7;monocytes: 137.	Cancer enriched	Detected in single	27
6	neutrophil: 2531.1	Lineage enriched	Detected in many	6	granulocytes: 2531.1	Cancer enhanced	Detected in some	5
		Low lineage specificity	Detected in all			Low cancer specificity	Detected in all	
		Low lineage specificity	Detected in all			Group enriched	Detected in some	10
	basophil: 1.0	Lineage enriched	Detected in single	6	granulocytes: 1.0	Cancer enhanced	Detected in single	
		Not detected	Not detected			Cancer enhanced	Detected in some	
		Not detected	Not detected			Low cancer specificity	Detected in many	
		Not detected	Not detected			Cancer enhanced	Detected in many	
	NK-cell: 36.3	Group enriched	Detected in many	4	dendritic cells: 19.5;NK-cells: 36.3;T-cells: 18.3	Low cancer specificity	Detected in all	
		Low lineage specificity	Detected in many			Low cancer specificity	Detected in all	
	neutrophil: 314.7	Low lineage specificity	Detected in all			Low cancer specificity	Detected in all	
		Low lineage specificity	Detected in all			Low cancer specificity	Detected in all	
		Not detected	Not detected			Low cancer specificity	Detected in many	
		Not detected	Not detected			Cancer enhanced	Detected in many	
4	MAIT T-cell: 3.8	Lineage enriched	Detected in single	14	T-cells: 3.8	Low cancer specificity	Detected in many	
	plasmacytoid DC: 5.1	Low lineage specificity	Detected in many			Low cancer specificity	Detected in all	
		Low lineage specificity	Detected in all			Low cancer specificity	Detected in all	
20	classical monocyte: 2.3;intermediate monocyte: 6.7;myeloid DC: 2.4	Group enriched	Detected in many	46	dendritic cells: 2.4;monocytes: 6.7	Cancer enriched	Detected in some	5
		Low lineage specificity	Detected in all			Low cancer specificity	Detected in all	
		Group enriched	Detected in all	17	NK-cells: 69.0;T-cells: 174.7	Low cancer specificity	Detected in all	
	intermediate monocyte: 2.0;non-classical monocyte: 2.8	Lineage enriched	Detected in single	5	monocytes: 2.8	Low cancer specificity	Detected in all	
		Not detected	Not detected			Low cancer specificity	Detected in many	
4	NK-cell: 4.0	Lineage enriched	Detected in single	4	NK-cells: 4.0	Low cancer specificity	Detected in all	
	basophil: 13.1;neutrophil: 20.2	Lineage enriched	Detected in many	4	granulocytes: 20.2	Low cancer specificity	Detected in all	
		Not detected	Not detected			Low cancer specificity	Detected in many	
		Not detected	Not detected			Cancer enriched	Detected in single	7
		Not detected	Not detected			Cancer enriched	Detected in some	6
		Not detected	Not detected			Group enriched	Detected in some	5
		Not detected	Not detected			Cancer enhanced	Detected in some	
		Not detected	Not detected			Cancer enriched	Detected in single	5
		Not detected	Not detected			Cancer enriched	Detected in many	8
		Low lineage specificity	Detected in all			Low cancer specificity	Detected in all	
5	eosinophil: 647.3	Lineage enriched	Detected in many	5	granulocytes: 647.3	Group enriched	Detected in some	27
		Low lineage specificity	Detected in all			Low cancer specificity	Detected in all	
		Low lineage specificity	Detected in many			Low cancer specificity	Detected in all	
		Not detected	Not detected			Cancer enriched	Detected in some	9
		Not detected	Not detected			Not detected	Not detected	
		Not detected	Not detected			Cancer enhanced	Detected in some	
		Low lineage specificity	Detected in all			Low cancer specificity	Detected in all	
		Not detected	Not detected			Cancer enhanced	Detected in many	
		Not detected	Not detected			Low cancer specificity	Detected in many	
		Not detected	Not detected			Cancer enhanced	Detected in many	
	T-reg: 2.6	Lineage enriched	Detected in single	5	T-cells: 2.6	Group enriched	Detected in many	5
	basophil: 1.9	Lineage enriched	Detected in single	4	granulocytes: 1.9	Low cancer specificity	Detected in all	
		Not detected	Not detected			Low cancer specificity	Detected in many	
		Not detected	Not detected			Low cancer specificity	Detected in some	
		Low lineage specificity	Detected in all			Low cancer specificity	Detected in all	
		Not detected	Not detected			Cancer enriched	Detected in single	5
		Not detected	Not detected			Cancer enhanced	Detected in many	
		Not detected	Not detected			Not detected	Not detected	
		Not detected	Not detected			Not detected	Not detected	
		Not detected	Not detected			Cancer enhanced	Detected in single	
		Low lineage specificity	Detected in all			Low cancer specificity	Detected in all	
	basophil: 26.9	Lineage enriched	Detected in many	6	granulocytes: 26.9	Low cancer specificity	Detected in all	
		Low lineage specificity	Detected in all			Low cancer specificity	Detected in all	
		Low lineage specificity	Detected in all			Low cancer specificity	Detected in all	
		Not detected	Not detected			Cancer enhanced	Detected in single	
		Not detected	Not detected			Cancer enhanced	Detected in all	
	naive CD4 T-cell: 1.9;naive CD8 T-cell: 1.2	Low lineage specificity	Detected in single			Group enriched	Detected in many	6
9	basophil: 3.1	Lineage enriched	Detected in single	19	granulocytes: 3.1	Cancer enhanced	Detected in many	5
		Not detected	Not detected			Not detected	Not detected	
		Not detected	Not detected			Low cancer specificity	Detected in single	
		Low lineage specificity	Detected in many			Low cancer specificity	Detected in many	
		Low lineage specificity	Detected in all			Group enriched	Detected in some	5
	basophil: 1054.3	Low lineage specificity	Detected in all			Cancer enhanced	Detected in some	

18	intermediate monocyte: 2.6;non-classical monocyte: 4.3	Lineage enriched	Detected in single	22	monocytes: 4.3	Low cancer specificity	Detected in single	
		Not detected	Not detected			Not detected	Not detected	
		Not detected	Not detected			Cancer enriched	Detected in some	6
		Not detected	Not detected			Low cancer specificity	Detected in all	
5	basophil: 3.6	Lineage enriched	Detected in single	13	granulocytes: 3.6	Low cancer specificity	Detected in many	
	plasmacytoid DC: 7.9	Group enriched	Detected in many	9	dendritic cells: 7.9;monocytes: 3.8	Low cancer specificity	Detected in all	
		Not detected	Not detected			Low cancer specificity	Detected in all	
		Low lineage specificity	Detected in all			Low cancer specificity	Detected in all	
4	plasmacytoid DC: 6.8	Lineage enriched	Detected in many	4	dendritic cells: 6.8	Cancer enhanced	Detected in many	
		Not detected	Not detected			Cancer enhanced	Detected in many	
		Not detected	Not detected			Not detected	Not detected	
		Not detected	Not detected			Cancer enhanced	Detected in many	
		Not detected	Not detected			Cancer enhanced	Detected in some	
		Low lineage specificity	Detected in many			Low cancer specificity	Detected in many	
		Not detected	Not detected			Cancer enhanced	Detected in many	
		Group enriched	Detected in many	7	granulocytes: 1.0;T-cells: 2.6	Cancer enhanced	Detected in some	
		Not detected	Not detected			Low cancer specificity	Detected in all	
		Not detected	Not detected			Cancer enriched	Detected in some	18
		Low lineage specificity	Detected in all			Cancer enhanced	Detected in many	
		Low lineage specificity	Detected in all			Low cancer specificity	Detected in all	
4	eosinophil: 2.5	Lineage enriched	Detected in single	18	granulocytes: 2.5	Cancer enhanced	Detected in some	
5	neutrophil: 177.3	Lineage enriched	Detected in many	7	granulocytes: 177.3	Cancer enhanced	Detected in some	
16	classical monocyte: 574.4;eosinophil: 708.2;intermediate monocyte: 1057.2;myeloid DC: 335.8;neutr	Group enriched	Detected in all	16	dendritic cells: 335.8;granulocytes: 803.1;monocytes: 1	Group enriched	Detected in some	7
		Low lineage specificity	Detected in all			Group enriched	Detected in many	11
		Not detected	Not detected			Cancer enhanced	Detected in single	
		Low lineage specificity	Detected in many			Low cancer specificity	Detected in many	
		Low lineage specificity	Detected in all			Low cancer specificity	Detected in all	
	myeloid DC: 286.9	Group enriched	Detected in all	10	B-cells: 229.7;dendritic cells: 286.9;monocytes: 138.3	Group enriched	Detected in some	6
		Low lineage specificity	Detected in all			Low cancer specificity	Detected in many	
		Low lineage specificity	Detected in all			Low cancer specificity	Detected in all	
	classical monocyte: 15.1	Low lineage specificity	Detected in many			Low cancer specificity	Detected in all	
		Not detected	Not detected			Low cancer specificity	Detected in many	
		Low lineage specificity	Detected in all			Low cancer specificity	Detected in all	
		Low lineage specificity	Detected in all			Low cancer specificity	Detected in all	
12	myeloid DC: 1.4	Lineage enriched	Detected in single	12	dendritic cells: 1.4	Low cancer specificity	Detected in many	
		Not detected	Not detected			Low cancer specificity	Detected in many	
		Low lineage specificity	Detected in all			Low cancer specificity	Detected in all	
4	neutrophil: 25.9	Lineage enriched	Detected in all	6	granulocytes: 25.9	Low cancer specificity	Detected in all	
		Low lineage specificity	Detected in many			Low cancer specificity	Detected in all	
		Low lineage specificity	Detected in all			Low cancer specificity	Detected in all	
5	eosinophil: 1500.1;gdT-cell: 2915.2;MAIT T-cell: 2008.6;memory CD4 T-cell: 3131.5;memory CD8 T-c	Group enriched	Detected in all	7	granulocytes: 2758.0;NK-cells: 4694.8;T-cells: 3131.5	Low cancer specificity	Detected in all	
		Not detected	Not detected			Not detected	Not detected	
		Low lineage specificity	Detected in all			Low cancer specificity	Detected in all	
	neutrophil: 4.3	Group enriched	Detected in many	6	granulocytes: 4.3;T-cells: 1.4	Low cancer specificity	Detected in all	
	plasmacytoid DC: 901.7	Lineage enriched	Detected in all	4	dendritic cells: 901.7	Cancer enhanced	Detected in all	
		Low lineage specificity	Detected in all			Cancer enhanced	Detected in some	
		Low lineage specificity	Detected in all			Low cancer specificity	Detected in many	
	basophil: 1.3	Low lineage specificity	Detected in single			Low cancer specificity	Detected in many	
64	basophil: 38.4;eosinophil: 29.4	Lineage enriched	Detected in single	73	granulocytes: 38.4	Cancer enhanced	Detected in many	
		Low lineage specificity	Detected in all			Group enriched	Detected in many	8
	naive B-cell: 77.1	Group enriched	Detected in all	5	B-cells: 77.1;granulocytes: 31.1	Cancer enhanced	Detected in all	
	naive CD4 T-cell: 22.8	Lineage enriched	Detected in many	5	T-cells: 22.8	Low cancer specificity	Detected in all	
4	gdT-cell: 102.3;MAIT T-cell: 370.2;memory CD4 T-cell: 214.4;memory CD8 T-cell: 151.1;naive CD4 T-c	Group enriched	Detected in all	34	NK-cells: 113.4;T-cells: 370.2	Low cancer specificity	Detected in many	
		Low lineage specificity	Detected in all			Low cancer specificity	Detected in all	
		Low lineage specificity	Detected in all			Low cancer specificity	Detected in all	
		Not detected	Not detected			Low cancer specificity	Detected in all	
	neutrophil: 86.3	Low lineage specificity	Detected in all			Cancer enhanced	Detected in some	
		Low lineage specificity	Detected in all			Low cancer specificity	Detected in all	
		Low lineage specificity	Detected in all			Group enriched	Detected in many	6
17	basophil: 5.6;neutrophil: 3.0	Lineage enriched	Detected in single	22	granulocytes: 5.6	Cancer enhanced	Detected in many	
	plasmacytoid DC: 4.6	Low lineage specificity	Detected in many			Low cancer specificity	Detected in all	
	eosinophil: 5.5;non-classical monocyte: 5.1	Low lineage specificity	Detected in many			Low cancer specificity	Detected in all	
	neutrophil: 2.0	Low lineage specificity	Detected in single			Cancer enhanced	Detected in some	
		Not detected	Not detected			Cancer enhanced	Detected in many	
	eosinophil: 3539.1	Group enriched	Detected in all	5	dendritic cells: 1018.9;granulocytes: 3539.1;monocytes:	Group enriched	Detected in some	11
5	classical monocyte: 13.2;eosinophil: 28.8;intermediate monocyte: 22.6;memory B-cell: 13.5;myeloid	Low lineage specificity	Detected in many			Low cancer specificity	Detected in all	
	plasmacytoid DC: 3.5	Low lineage specificity	Detected in many			Low cancer specificity	Detected in many	
		Low lineage specificity	Detected in all			Low cancer specificity	Detected in all	
		Low lineage specificity	Detected in all			Low cancer specificity	Detected in all	
		Not detected	Not detected			Group enriched	Detected in some	4
		Low lineage specificity	Detected in single			Cancer enhanced	Detected in some	
49	basophil: 51.7;classical monocyte: 38.6;eosinophil: 23.5;intermediate monocyte: 41.8;myeloid DC: 18	Group enriched	Detected in many	123	dendritic cells: 18.3;granulocytes: 53.4;monocytes: 41.8	Cancer enhanced	Detected in many	
		Not detected	Not detected			Not detected	Not detected	
	naive B-cell: 1.4	Lineage enriched	Detected in single	4	B-cells: 1.4	Cancer enhanced	Detected in some	
34	plasmacytoid DC: 63.9	Lineage enriched	Detected in many	34	dendritic cells: 63.9	Cancer enhanced	Detected in some	
		Low lineage specificity	Detected in many			Low cancer specificity	Detected in many	
		Low lineage specificity	Detected in many			Cancer enhanced	Detected in many	
		Not detected	Not detected			Group enriched	Detected in some	6
		Not detected	Not detected			Low cancer specificity	Detected in many	
		Not detected	Not detected			Cancer enriched	Detected in single	6
		Not detected	Not detected			Cancer enhanced	Detected in some	
		Not detected	Not detected			Cancer enriched	Detected in single	19
		Not detected	Not detected			Low cancer specificity	Detected in many	
		Not detected	Not detected			Cancer enhanced	Detected in many	
6	basophil: 16.1;eosinophil: 9.6;NK-cell: 11.1	Group enriched	Detected in many	7	granulocytes: 16.1;NK-cells: 11.1	Cancer enhanced	Detected in many	
		Low lineage specificity	Detected in many			Low cancer specificity	Detected in all	
		Not detected	Not detected			Group enriched	Detected in many	5
40	gdT-cell: 287.0;MAIT T-cell: 111.2;memory CD8 T-cell: 260.3;naive CD8 T-cell: 258.6;NK-cell: 335.6	Group enriched	Detected in all	49	NK-cells: 335.6;T-cells: 287.0	Group enriched	Detected in some	15
		Not detected	Not detected			Cancer enhanced	Detected in many	
		Low lineage specificity	Detected in all			Group enriched	Detected in some	5
	neutrophil: 564.0	Lineage enriched	Detected in all	5	granulocytes: 564.0	Low cancer specificity	Detected in all	
		Low lineage specificity	Detected in all			Low cancer specificity	Detected in all	
20	basophil: 308.6;gdT-cell: 303.5;MAIT T-cell: 212.3;memory CD4 T-cell: 295.3;memory CD8 T-cell: 306	Group enriched	Detected in all	172	granulocytes: 308.6;NK-cells: 114.7;T-cells: 411.7	Cancer enhanced	Detected in all	
	basophil: 873.1	Low lineage specificity	Detected in all			Group enriched	Detected in many	4
		Group enriched	Detected in many	14	B-cells: 7.5;NK-cells: 5.3;T-cells: 12.0	Cancer enriched	Detected in some	6
		Low lineage specificity	Detected in all			Cancer enhanced	Detected in many	
	basophil: 5.0	Lineage enriched	Detected in many	5	granulocytes: 5.0	Low cancer specificity	Detected in all	
		Not detected	Not detected			Not detected	Not detected	
		Not detected	Not detected			Cancer enhanced	Detected in many	
		Not detected	Not detected			Cancer enriched	Detected in single	54
5	neutrophil: 2587.2	Lineage enriched	Detected in all	6	granulocytes: 2587.2	Low cancer specificity	Detected in all	
		Not detected	Not detected			Low cancer specificity	Detected in many	
		Not detected	Not detected			Cancer enhanced	Detected in many	
7	basophil: 7.8	Lineage enriched	Detected in many	7	granulocytes: 7.8	Cancer enhanced	Detected in some	
		Low lineage specificity	Detected in all			Low cancer specificity	Detected in many	
	neutrophil: 18.8	Low lineage specificity	Detected in all			Low cancer specificity	Detected in all	
		Not detected	Not detected			Cancer enhanced	Detected in some	
18	classical monocyte: 4.3;intermediate monocyte: 2.2;myeloid DC: 1.6;non-classical monocyte: 2.5	Group enriched	Detected in many	21	dendritic cells: 1.6;monocytes: 4.3	Low cancer specificity	Detected in all	
1179	plasmacytoid DC: 117.8	Lineage enriched	Detected in single	1179	dendritic cells: 117.8	Group enriched	Detected in many	4
		Low lineage specificity	Detected in many			Low cancer specificity	Detected in all	
	non-classical monocyte: 1767.7	Low lineage specificity	Detected in all			Cancer enriched	Detected in some	17
		Not detected	Not detected			Low cancer specificity	Detected in all	
	neutrophil: 64.6	Low lineage specificity	Detected in all			Low cancer specificity	Detected in all	
		Low lineage specificity	Detected in all			Low cancer specificity	Detected in all	
	basophil: 22.6	Low lineage specificity	Detected in all			Low cancer specificity	Detected in all	
		Not detected	Not detected			Low cancer specificity	Detected in all	
		Not detected	Not detected			Low cancer specificity	Detected in all	
		Low lineage specificity	Detected in all			Low cancer specificity	Detected in all	
		Low lineage specificity	Detected in all			Low cancer specificity	Detected in all	
		Not detected	Not detected			Low cancer specificity	Detected in many	
	non-classical monocyte: 1.7	Low lineage specificity	Detected in single			Group enriched	Detected in many	4
	intermediate monocyte: 12.8;neutrophil: 18.8	Low lineage specificity	Detected in many			Low cancer specificity	Detected in all	
		Low lineage specificity	Detected in all			Low cancer specificity	Detected in all	
		Not detected	Not detected			Low cancer specificity	Detected in many	
		Not detected	Not detected			Not detected	Not detected	
		Not detected	Not detected			Not detected	Not detected	
		Not detected	Not detected			Cancer enhanced	Detected in all	
5	classical monocyte: 124.0;neutrophil: 57.5	Group enriched	Detected in many	5	granulocytes: 57.5;monocytes: 124.0	Cancer enriched	Detected in some	6
		Not detected	Not detected			Not detected	Not detected	

		Not detected	Not detected			Not detected	Not detected	
4	basophil: 118.2;classical monocyte: 45.3;eosinophil: 106.1;intermediate monocyte: 95.7;neutrophil: 4	Group enriched	Detected in all	5	granulocytes: 118.2;monocytes: 105.7	Low cancer specificity	Detected in all	
		Not detected	Not detected			Low cancer specificity	Detected in many	
	neutrophil: 7.6	Group enriched	Detected in many	4	granulocytes: 7.6;monocytes: 5.6	Cancer enhanced	Detected in single	
		Not detected	Not detected			Group enriched	Detected in some	4
11	intermediate monocyte: 8.7;non-classical monocyte: 5.5	Lineage enriched	Detected in single	50	monocytes: 8.7	Low cancer specificity	Detected in many	
5	basophil: 5.7;eosinophil: 1.8;neutrophil: 3.5	Lineage enriched	Detected in single	8	granulocytes: 5.7	Low cancer specificity	Detected in many	
10	classical monocyte: 13.2;intermediate monocyte: 6.6;myeloid DC: 14.7;plasmacytoid DC: 24.4	Group enriched	Detected in many	12	dendritic cells: 24.4;monocytes: 13.2	Low cancer specificity	Detected in all	
		Not detected	Not detected			Low cancer specificity	Detected in all	
		Low lineage specificity	Detected in all			Cancer enhanced	Detected in all	
		Not detected	Not detected			Cancer enhanced	Detected in many	
		Low lineage specificity	Detected in all			Low cancer specificity	Detected in many	
		Not detected	Not detected			Not detected	Not detected	
123	neutrophil: 139.9	Lineage enriched	Detected in many	123	granulocytes: 139.9	Low cancer specificity	Detected in many	
5	basophil: 18.3;neutrophil: 20.3	Lineage enriched	Detected in single	39	granulocytes: 20.3	Cancer enhanced	Detected in some	
	neutrophil: 1.4	Lineage enriched	Detected in single	4	granulocytes: 1.4	Cancer enriched	Detected in single	9
	eosinophil: 216.2;neutrophil: 203.0	Group enriched	Detected in all	8	dendritic cells: 57.8;granulocytes: 216.2;monocytes: 99.	Cancer enhanced	Detected in many	
		Low lineage specificity	Detected in all			Low cancer specificity	Detected in many	
4	neutrophil: 184.9	Lineage enriched	Detected in all	4	granulocytes: 184.9	Low cancer specificity	Detected in all	
		Low lineage specificity	Detected in all			Low cancer specificity	Detected in all	
		Not detected	Not detected			Cancer enhanced	Detected in many	
96	memory B-cell: 627.9;naive B-cell: 630.0	Lineage enriched	Detected in many	96	B-cells: 630.0	Cancer enriched	Detected in some	6
	myeloid DC: 5.9	Group enriched	Detected in many	39	dendritic cells: 5.9;monocytes: 1.8	Cancer enhanced	Detected in many	
		Low lineage specificity	Detected in all			Low cancer specificity	Detected in all	
		Low lineage specificity	Detected in all			Low cancer specificity	Detected in all	
	T-reg: 1.2	Lineage enhanced	Detected in single		T-cells: 1.2	Cancer enhanced	Detected in all	
		Not detected	Not detected			Cancer enhanced	Detected in some	
	basophil: 3.7;neutrophil: 3.5	Low lineage specificity	Detected in single			Cancer enhanced	Detected in all	
4	non-classical monocyte: 2.6	Lineage enriched	Detected in single	5	monocytes: 2.6	Low cancer specificity	Detected in all	
		Not detected	Not detected			Low cancer specificity	Detected in all	
		Low lineage specificity	Detected in many			Cancer enhanced	Detected in all	
		Low lineage specificity	Detected in all			Group enriched	Detected in some	15
	intermediate monocyte: 20.4;non-classical monocyte: 26.7	Group enriched	Detected in many	57	dendritic cells: 8.1;monocytes: 26.7	Low cancer specificity	Detected in many	
5	basophil: 2.5	Lineage enriched	Detected in single	7	granulocytes: 2.5	Low cancer specificity	Detected in many	
		Low lineage specificity	Detected in single			Cancer enhanced	Detected in many	
		Low lineage specificity	Detected in all			Low cancer specificity	Detected in all	
15	NK-cell: 53.9	Lineage enriched	Detected in many	15	NK-cells: 53.9	Cancer enhanced	Detected in many	
		Not detected	Not detected			Group enriched	Detected in many	5
		Low lineage specificity	Detected in all			Cancer enhanced	Detected in some	
		Not detected	Not detected			Low cancer specificity	Detected in all	
		Not detected	Not detected			Cancer enhanced	Detected in many	
		Not detected	Not detected			Low cancer specificity	Detected in all	
	neutrophil: 3.0	Lineage enriched	Detected in single	5	granulocytes: 3.0	Low cancer specificity	Detected in some	
		Low lineage specificity	Detected in many			Low cancer specificity	Detected in all	
		Low lineage specificity	Detected in all			Low cancer specificity	Detected in all	
8	plasmacytoid DC: 1.2	Lineage enriched	Detected in single	8	dendritic cells: 1.2	Cancer enhanced	Detected in many	
	neutrophil: 1.3	Low lineage specificity	Detected in single			Low cancer specificity	Detected in many	
	classical monocyte: 1.1	Low lineage specificity	Detected in single			Low cancer specificity	Detected in all	
		Not detected	Not detected			Cancer enhanced	Detected in many	
		Low lineage specificity	Detected in all			Low cancer specificity	Detected in all	
	neutrophil: 1.0	Low lineage specificity	Detected in single			Cancer enhanced	Detected in some	
		Not detected	Not detected			Not detected	Not detected	
7	basophil: 14.5;eosinophil: 13.6;gdT-cell: 11.0;MAIT T-cell: 19.8;memory CD4 T-cell: 17.5;memory CD8	Group enriched	Detected in many	9	granulocytes: 14.5;NK-cells: 20.9;T-cells: 29.5	Low cancer specificity	Detected in all	
		Not detected	Not detected			Cancer enhanced	Detected in some	
		Not detected	Not detected			Cancer enhanced	Detected in some	
		Low lineage specificity	Detected in all			Low cancer specificity	Detected in all	
		Not detected	Not detected			Cancer enhanced	Detected in many	
		Not detected	Not detected			Cancer enhanced	Detected in many	
		Low lineage specificity	Detected in all			Low cancer specificity	Detected in all	
	naive B-cell: 33.1;naive CD8 T-cell: 30.8	Group enriched	Detected in many	41	B-cells: 33.1;T-cells: 30.8	Cancer enhanced	Detected in all	
4	memory B-cell: 1.1	Lineage enriched	Detected in single	4	B-cells: 1.1	Cancer enhanced	Detected in many	
5	neutrophil: 1.1	Lineage enriched	Detected in single	11	granulocytes: 1.1	Cancer enhanced	Detected in some	
		Not detected	Not detected			Cancer enhanced	Detected in many	
		Not detected	Not detected			Cancer enhanced	Detected in many	
	neutrophil: 292.1	Low lineage specificity	Detected in all			Low cancer specificity	Detected in all	
	neutrophil: 2.6	Lineage enriched	Detected in single	6	granulocytes: 2.6	Low cancer specificity	Detected in many	
		Low lineage specificity	Detected in all			Low cancer specificity	Detected in all	
		Not detected	Not detected			Group enriched	Detected in some	17
	myeloid DC: 2.8	Low lineage specificity	Detected in many			Cancer enhanced	Detected in many	
4	plasmacytoid DC: 10.0	Lineage enriched	Detected in many	5	dendritic cells: 10.0	Low cancer specificity	Detected in all	
		Not detected	Not detected			Cancer enhanced	Detected in some	
		Not detected	Not detected			Not detected	Not detected	
		Not detected	Not detected			Not detected	Not detected	
		Not detected	Not detected			Not detected	Not detected	
5	basophil: 1.9	Lineage enriched	Detected in single	5	granulocytes: 1.9	Not detected	Not detected	
		Not detected	Not detected			Not detected	Not detected	
		Not detected	Not detected			Low cancer specificity	Detected in many	
		Low lineage specificity	Detected in many			Low cancer specificity	Detected in many	
	plasmacytoid DC: 1.3	Lineage enriched	Detected in single	14	dendritic cells: 1.3	Cancer enhanced	Detected in some	
	neutrophil: 96.2	Low lineage specificity	Detected in all			Low cancer specificity	Detected in all	
		Low lineage specificity	Detected in all			Cancer enhanced	Detected in many	
		Low lineage specificity	Detected in all			Low cancer specificity	Detected in all	
9	classical monocyte: 182.4;eosinophil: 293.3;intermediate monocyte: 177.8;myeloid DC: 264.5;neutro	Group enriched	Detected in all	11	dendritic cells: 264.5;granulocytes: 433.6;monocytes: 2(	Low cancer specificity	Detected in all	
		Low lineage specificity	Detected in many			Low cancer specificity	Detected in all	
5	non-classical monocyte: 1.9	Lineage enriched	Detected in single	19	monocytes: 1.9	Cancer enhanced	Detected in many	
	memory B-cell: 10.7;naive B-cell: 10.3	Low lineage specificity	Detected in many			Low cancer specificity	Detected in all	
		Low lineage specificity	Detected in many			Low cancer specificity	Detected in all	
		Low lineage specificity	Detected in all			Low cancer specificity	Detected in all	
	neutrophil: 2.0	Lineage enhanced	Detected in single		granulocytes: 2.0	Low cancer specificity	Detected in many	
		Low lineage specificity	Detected in all			Cancer enhanced	Detected in many	
50	basophil: 9.6	Lineage enriched	Detected in single	96	granulocytes: 9.6	Low cancer specificity	Detected in all	
		Not detected	Not detected			Low cancer specificity	Detected in many	
		Low lineage specificity	Detected in many			Low cancer specificity	Detected in many	
		Low lineage specificity	Detected in many			Low cancer specificity	Detected in all	
		Low lineage specificity	Detected in many			Low cancer specificity	Detected in many	
		Not detected	Not detected			Cancer enhanced	Detected in many	
		Not detected	Not detected			Low cancer specificity	Detected in many	
		Not detected	Not detected			Cancer enhanced	Detected in many	
		Not detected	Not detected			Low cancer specificity	Detected in many	
		Low lineage specificity	Detected in all			Low cancer specificity	Detected in all	
		Low lineage specificity	Detected in many			Low cancer specificity	Detected in many	
5	eosinophil: 58.2	Lineage enriched	Detected in many	5	granulocytes: 58.2	Low cancer specificity	Detected in all	
	naive CD4 T-cell: 3.1	Low lineage specificity	Detected in single			Cancer enhanced	Detected in many	
		Not detected	Not detected			Low cancer specificity	Detected in many	
		Not detected	Not detected			Cancer enhanced	Detected in many	
		Not detected	Not detected			Cancer enhanced	Detected in many	
		Not detected	Not detected			Low cancer specificity	Detected in many	
	non-classical monocyte: 706.3	Low lineage specificity	Detected in all			Cancer enriched	Detected in many	5
27	plasmacytoid DC: 5.8	Lineage enriched	Detected in single	27	dendritic cells: 5.8	Low cancer specificity	Detected in many	
		Low lineage specificity	Detected in many			Low cancer specificity	Detected in all	
		Low lineage specificity	Detected in all			Low cancer specificity	Detected in all	
		Low lineage specificity	Detected in all			Group enriched	Detected in some	14
		Low lineage specificity	Detected in all			Low cancer specificity	Detected in all	
	basophil: 1.4	Low lineage specificity	Detected in single			Low cancer specificity	Detected in all	
4	basophil: 5.6;eosinophil: 6.9;myeloid DC: 2.7	Low lineage specificity	Detected in many			Cancer enhanced	Detected in many	
		Not detected	Not detected			Low cancer specificity	Detected in many	
	non-classical monocyte: 2.0	Low lineage specificity	Detected in single			Low cancer specificity	Detected in all	
	NK-cell: 3.8	Low lineage specificity	Detected in many			Cancer enhanced	Detected in all	
		Low lineage specificity	Detected in all			Low cancer specificity	Detected in all	
	neutrophil: 1177.3	Group enriched	Detected in all	4	granulocytes: 1177.3;monocytes: 553.8	Low cancer specificity	Detected in all	
4	basophil: 1.8;plasmacytoid DC: 2.0	Group enriched	Detected in many	4	dendritic cells: 2.0;granulocytes: 1.8	Cancer enhanced	Detected in many	
8	gdT-cell: 4.7;MAIT T-cell: 6.4;memory CD4 T-cell: 4.1;memory CD8 T-cell: 3.9;naive CD4 T-cell: 4.3;na	Group enriched	Detected in many	9	NK-cells: 3.4;T-cells: 6.4	Low cancer specificity	Detected in all	
6	eosinophil: 3.7;MAIT T-cell: 1.9	Group enriched	Detected in many	6	granulocytes: 3.7;T-cells: 1.9	Low cancer specificity	Detected in all	
	plasmacytoid DC: 1.1	Low lineage specificity	Detected in single			Low cancer specificity	Detected in many	
		Not detected	Not detected			Cancer enhanced	Detected in many	
		Not detected	Not detected			Cancer enhanced	Detected in many	
		Low lineage specificity	Detected in many			Cancer enhanced	Detected in all	
	gdT-cell: 3.3;MAIT T-cell: 6.6	Lineage enriched	Detected in single	40	T-cells: 6.6	Low cancer specificity	Detected in many	
		Low lineage specificity	Detected in all			Low cancer specificity	Detected in all	

		Not detected	Not detected			Cancer enriched	Detected in all	5
		Not detected	Not detected			Low cancer specificity	Detected in all	
	naive B-cell: 2.7	Low lineage specificity	Detected in single			Low cancer specificity	Detected in all	
		Low lineage specificity	Detected in all			Low cancer specificity	Detected in all	
		Low lineage specificity	Detected in many			Group enriched	Detected in some	17
	neutrophil: 2.7	Low lineage specificity	Detected in single			Low cancer specificity	Detected in all	
		Low lineage specificity	Detected in all			Low cancer specificity	Detected in all	
		Not detected	Not detected			Cancer enhanced	Detected in many	
		Low lineage specificity	Detected in many			Low cancer specificity	Detected in all	
		Low lineage specificity	Detected in single			Low cancer specificity	Detected in all	
		Not detected	Not detected			Cancer enhanced	Detected in many	
		Low lineage specificity	Detected in all			Low cancer specificity	Detected in all	
		Low lineage specificity	Detected in many			Low cancer specificity	Detected in many	
8	NK-cell: 3.3	Lineage enriched	Detected in single	8	NK-cells: 3.3	Cancer enhanced	Detected in many	
		Not detected	Not detected			Cancer enhanced	Detected in many	
		Low lineage specificity	Detected in all			Low cancer specificity	Detected in all	
		Low lineage specificity	Detected in all			Low cancer specificity	Detected in all	
		Low lineage specificity	Detected in all			Group enriched	Detected in some	5
5	classical monocyte: 256.9;eosinophil: 908.6;neutrophil: 749.9	Group enriched	Detected in many	5	granulocytes: 908.6;monocytes: 256.9	Cancer enhanced	Detected in many	
		Low lineage specificity	Detected in many			Low cancer specificity	Detected in many	
		Not detected	Not detected			Cancer enhanced	Detected in many	
		Low lineage specificity	Detected in all			Group enriched	Detected in all	4
10	plasmacytoid DC: 13.5	Lineage enriched	Detected in many	10	dendritic cells: 13.5	Low cancer specificity	Detected in many	
		Not detected	Not detected			Low cancer specificity	Detected in many	
	neutrophil: 9.5	Group enriched	Detected in many	4	granulocytes: 9.5;monocytes: 4.8	Low cancer specificity	Detected in all	
6	memory B-cell: 1.6;naive B-cell: 2.9	Lineage enriched	Detected in single	7	B-cells: 2.9	Low cancer specificity	Detected in all	
	memory CD8 T-cell: 4.7	Group enriched	Detected in many	33	dendritic cells: 1.8;T-cells: 4.7	Low cancer specificity	Detected in all	
		Not detected	Not detected			Cancer enhanced	Detected in many	
197	plasmacytoid DC: 153.7	Lineage enriched	Detected in single	197	dendritic cells: 153.7	Low cancer specificity	Detected in all	
5	classical monocyte: 4.4;intermediate monocyte: 4.4;myeloid DC: 3.6;neutrophil: 1.4;non-classical mo	Group enriched	Detected in many	4	dendritic cells: 3.6;granulocytes: 1.4;monocytes: 5.1	Low cancer specificity	Detected in all	
		Low lineage specificity	Detected in all			Cancer enhanced	Detected in all	
10	classical monocyte: 7.0;myeloid DC: 10.5;plasmacytoid DC: 12.9	Group enriched	Detected in many	11	dendritic cells: 12.9;monocytes: 7.0	Low cancer specificity	Detected in all	
		Not detected	Not detected			Low cancer specificity	Detected in all	
	MAIT T-cell: 1.3;naive CD4 T-cell: 1.2	Lineage enriched	Detected in single	13	T-cells: 1.3	Low cancer specificity	Detected in many	
		Low lineage specificity	Detected in all			Low cancer specificity	Detected in all	
		Not detected	Not detected			Cancer enhanced	Detected in many	
		Not detected	Not detected			Cancer enhanced	Detected in many	
	neutrophil: 150.3	Lineage enriched	Detected in all	5	granulocytes: 150.3	Low cancer specificity	Detected in all	
		Low lineage specificity	Detected in all			Low cancer specificity	Detected in all	
10	neutrophil: 196.5	Lineage enriched	Detected in all	10	granulocytes: 196.5	Low cancer specificity	Detected in all	
		Not detected	Not detected			Group enriched	Detected in some	6
	neutrophil: 2.3	Low lineage specificity	Detected in many			Low cancer specificity	Detected in all	
		Not detected	Not detected			Low cancer specificity	Detected in many	
		Low lineage specificity	Detected in all			Group enriched	Detected in some	17
	T-reg: 4.8	Lineage enriched	Detected in single	8	T-cells: 4.8	Group enriched	Detected in many	4
		Low lineage specificity	Detected in all			Group enriched	Detected in many	9
	neutrophil: 39.5	Low lineage specificity	Detected in many			Cancer enriched	Detected in some	6
		Low lineage specificity	Detected in all			Low cancer specificity	Detected in all	
		Not detected	Not detected			Low cancer specificity	Detected in all	
		Low lineage specificity	Detected in all			Low cancer specificity	Detected in all	
		Low lineage specificity	Detected in all			Low cancer specificity	Detected in many	
	neutrophil: 58.9	Low lineage specificity	Detected in many			Low cancer specificity	Detected in all	
		Low lineage specificity	Detected in all			Low cancer specificity	Detected in all	
		Not detected	Not detected			Group enriched	Detected in some	6
		Low lineage specificity	Detected in all			Low cancer specificity	Detected in all	
		Group enriched	Detected in all	4	granulocytes: 19.0;NK-cells: 42.5;T-cells: 24.9	Low cancer specificity	Detected in all	
152	basophil: 1318.2	Lineage enriched	Detected in single	1550	granulocytes: 1318.2	Cancer enhanced	Detected in many	
		Low lineage specificity	Detected in all			Low cancer specificity	Detected in all	
	neutrophil: 1.3	Low lineage specificity	Detected in single			Low cancer specificity	Detected in many	
		Not detected	Not detected			Cancer enhanced	Detected in many	
		Low lineage specificity	Detected in all			Low cancer specificity	Detected in all	
		Not detected	Not detected			Low cancer specificity	Detected in all	
		Low lineage specificity	Detected in all			Low cancer specificity	Detected in all	
		Low lineage specificity	Detected in all			Low cancer specificity	Detected in all	
	neutrophil: 26.8	Low lineage specificity	Detected in all			Low cancer specificity	Detected in all	
		Not detected	Not detected			Low cancer specificity	Detected in all	
	MAIT T-cell: 10.8	Lineage enriched	Detected in many	9	T-cells: 10.8	Low cancer specificity	Detected in many	
		Not detected	Not detected			Cancer enhanced	Detected in many	
9	intermediate monocyte: 3.1;non-classical monocyte: 6.3;plasmacytoid DC: 7.5	Group enriched	Detected in many	11	dendritic cells: 7.5;monocytes: 6.3	Low cancer specificity	Detected in many	
		Low lineage specificity	Detected in all			Low cancer specificity	Detected in all	
6	classical monocyte: 24645.2;neutrophil: 33874.3	Group enriched	Detected in all	6	granulocytes: 33874.3;monocytes: 24645.2	Cancer enhanced	Detected in many	
		Not detected	Not detected			Cancer enhanced	Detected in many	
		Not detected	Not detected			Not detected	Not detected	
		Not detected	Not detected			Cancer enriched	Detected in some	12
		Not detected	Not detected			Cancer enhanced	Detected in some	
		Not detected	Not detected			Low cancer specificity	Detected in many	
		Not detected	Not detected			Low cancer specificity	Detected in many	
		Not detected	Not detected			Cancer enriched	Detected in many	5
		Not detected	Not detected			Low cancer specificity	Detected in all	
		Not detected	Not detected			Cancer enhanced	Detected in many	
7	neutrophil: 13.7	Lineage enriched	Detected in many	7	granulocytes: 13.7	Low cancer specificity	Detected in many	
		Low lineage specificity	Detected in all			Low cancer specificity	Detected in all	
		Low lineage specificity	Detected in all			Cancer enhanced	Detected in all	
		Low lineage specificity	Detected in many			Low cancer specificity	Detected in all	
		Not detected	Not detected			Cancer enhanced	Detected in some	
6	eosinophil: 767.4;intermediate monocyte: 252.2;non-classical monocyte: 542.7	Group enriched	Detected in many	16	granulocytes: 767.4;monocytes: 542.7	Group enriched	Detected in many	6
5	classical monocyte: 57.8;intermediate monocyte: 71.1;myeloid DC: 34.7;neutrophil: 80.0;non-classica	Group enriched	Detected in many	5	dendritic cells: 34.7;granulocytes: 80.0;monocytes: 71.1	Cancer enhanced	Detected in single	
	neutrophil: 21.6	Low lineage specificity	Detected in all			Low cancer specificity	Detected in all	
		Low lineage specificity	Detected in many			Low cancer specificity	Detected in all	
		Not detected	Not detected			Low cancer specificity	Detected in many	
		Not detected	Not detected			Group enriched	Detected in some	8
	T-reg: 4.1	Low lineage specificity	Detected in many			Cancer enhanced	Detected in many	
	T-reg: 6.2	Low lineage specificity	Detected in many			Low cancer specificity	Detected in all	
	naive CD4 T-cell: 1.7;T-reg: 1.6	Low lineage specificity	Detected in single			Low cancer specificity	Detected in many	
	neutrophil: 110.1	Low lineage specificity	Detected in all			Low cancer specificity	Detected in all	
		Low lineage specificity	Detected in many			Low cancer specificity	Detected in many	
		Low lineage specificity	Detected in all			Low cancer specificity	Detected in all	
	plasmacytoid DC: 96.0	Low lineage specificity	Detected in all			Low cancer specificity	Detected in all	
		Not detected	Not detected			Cancer enhanced	Detected in many	
		Low lineage specificity	Detected in all			Low cancer specificity	Detected in all	
		Not detected	Not detected			Cancer enhanced	Detected in single	
		Low lineage specificity	Detected in many			Low cancer specificity	Detected in all	
		Not detected	Not detected			Cancer enriched	Detected in single	6
		Low lineage specificity	Detected in many			Low cancer specificity	Detected in all	
		Not detected	Not detected			Cancer enhanced	Detected in many	
	basophil: 3.7;neutrophil: 3.3	Low lineage specificity	Detected in many			Cancer enriched	Detected in many	6
		Not detected	Not detected			Cancer enhanced	Detected in some	
7	basophil: 166.9;neutrophil: 225.3	Lineage enriched	Detected in all	9	granulocytes: 225.3	Cancer enriched	Detected in many	7
5	naive B-cell: 23.3	Lineage enriched	Detected in many	5	B-cells: 23.3	Group enriched	Detected in many	8
		Low lineage specificity	Detected in many			Cancer enhanced	Detected in all	
		Not detected	Not detected			Cancer enhanced	Detected in some	
		Not detected	Not detected			Not detected	Not detected	
		Not detected	Not detected			Cancer enhanced	Detected in many	
	memory CD8 T-cell: 1.0	Lineage enriched	Detected in single	10	T-cells: 1.0	Low cancer specificity	Detected in many	
		Low lineage specificity	Detected in all			Low cancer specificity	Detected in all	
		Low lineage specificity	Detected in all			Low cancer specificity	Detected in all	
		Low lineage specificity	Detected in all			Low cancer specificity	Detected in all	
	neutrophil: 327.7	Low lineage specificity	Detected in many			Cancer enhanced	Detected in many	
		Low lineage specificity	Detected in all			Low cancer specificity	Detected in all	
		Low lineage specificity	Detected in many			Low cancer specificity	Detected in all	
		Low lineage specificity	Detected in many			Low cancer specificity	Detected in all	
		Not detected	Not detected			Cancer enhanced	Detected in many	
		Low lineage specificity	Detected in many			Low cancer specificity	Detected in all	
		Not detected	Not detected			Group enriched	Detected in some	4
		Not detected	Not detected			Cancer enhanced	Detected in some	
		Not detected	Not detected			Low cancer specificity	Detected in all	
		Not detected	Not detected			Low cancer specificity	Detected in many	
10	plasmacytoid DC: 17.2	Lineage enriched	Detected in many	10	dendritic cells: 17.2	Cancer enhanced	Detected in all	
		Low lineage specificity	Detected in all			Low cancer specificity	Detected in all	

	neutrophil: 39.9	Group enriched	Detected in many	6	granulocytes: 39.9;monocytes: 12.3	Low cancer specificity	Detected in many	
		Low lineage specificity	Detected in many			Low cancer specificity	Detected in all	
		Not detected	Not detected			Low cancer specificity	Detected in many	
		Low lineage specificity	Detected in many			Low cancer specificity	Detected in all	
5	classical monocyte: 2.2;intermediate monocyte: 1.6;myeloid DC: 3.9;plasmacytoid DC: 2.9	Group enriched	Detected in many	5	dendritic cells: 3.9;monocytes: 2.2	Low cancer specificity	Detected in all	
		Low lineage specificity	Detected in all			Low cancer specificity	Detected in all	
		Not detected	Not detected			Not detected	Not detected	
		Low lineage specificity	Detected in single			Low cancer specificity	Detected in many	
		Not detected	Not detected			Cancer enhanced	Detected in some	
		Not detected	Not detected			Cancer enhanced	Detected in many	
	T-reg: 1.6	Low lineage specificity	Detected in single			Low cancer specificity	Detected in all	
		Not detected	Not detected			Cancer enhanced	Detected in some	
8	NK-cell: 1.2	Lineage enriched	Detected in single	8	NK-cells: 1.2	Cancer enhanced	Detected in many	
		Not detected	Not detected			Cancer enhanced	Detected in many	
		Low lineage specificity	Detected in all			Low cancer specificity	Detected in all	
	eosinophil: 681.5	Low lineage specificity	Detected in all			Low cancer specificity	Detected in all	
		Low lineage specificity	Detected in single			Low cancer specificity	Detected in all	
		Not detected	Not detected			Cancer enhanced	Detected in single	
	plasmacytoid DC: 10.2	Low lineage specificity	Detected in many			Low cancer specificity	Detected in all	
5	basophil: 6.1	Lineage enriched	Detected in single	10	granulocytes: 6.1	Low cancer specificity	Detected in many	
	intermediate monocyte: 9.2;non-classical monocyte: 10.9	Low lineage specificity	Detected in many			Low cancer specificity	Detected in all	
		Low lineage specificity	Detected in all			Low cancer specificity	Detected in all	
	plasmacytoid DC: 4.8	Low lineage specificity	Detected in many			Low cancer specificity	Detected in all	
		Low lineage specificity	Detected in all			Low cancer specificity	Detected in many	
		Not detected	Not detected			Cancer enhanced	Detected in all	
		Low lineage specificity	Detected in all			Low cancer specificity	Detected in all	
		Low lineage specificity	Detected in all			Low cancer specificity	Detected in all	
	intermediate monocyte: 1.9	Group enriched	Detected in many	4	dendritic cells: 1.0;monocytes: 1.9	Low cancer specificity	Detected in all	
		Not detected	Not detected			Cancer enhanced	Detected in many	
		Low lineage specificity	Detected in all			Low cancer specificity	Detected in all	
12	neutrophil: 161.9	Lineage enriched	Detected in many	12	granulocytes: 161.9	Cancer enriched	Detected in many	6
		Low lineage specificity	Detected in all			Low cancer specificity	Detected in all	
		Low lineage specificity	Detected in all			Low cancer specificity	Detected in all	
		Low lineage specificity	Detected in all			Low cancer specificity	Detected in all	
		Not detected	Not detected			Not detected	Not detected	
		Low lineage specificity	Detected in all			Low cancer specificity	Detected in all	
4	neutrophil: 260.9	Lineage enriched	Detected in all	9	granulocytes: 260.9	Low cancer specificity	Detected in all	
	memory B-cell: 80.5;naive B-cell: 84.7	Group enriched	Detected in all	7	B-cells: 84.7;granulocytes: 48.5;monocytes: 29.6	Cancer enhanced	Detected in all	
		Low lineage specificity	Detected in all			Low cancer specificity	Detected in all	
	gdT-cell: 13.9;MAIT T-cell: 23.8	Lineage enriched	Detected in many	6	T-cells: 23.8	Cancer enhanced	Detected in many	
		Low lineage specificity	Detected in all			Low cancer specificity	Detected in all	
23	basophil: 2.2	Lineage enriched	Detected in single	23	granulocytes: 2.2	Group enriched	Detected in some	4
	intermediate monocyte: 1.3	Low lineage specificity	Detected in single			Low cancer specificity	Detected in some	
	eosinophil: 15.1	Group enriched	Detected in many	79	dendritic cells: 4.3;granulocytes: 15.1;monocytes: 4.2	Cancer enhanced	Detected in many	
17	memory B-cell: 1.6	Lineage enriched	Detected in single	17	B-cells: 1.6	Cancer enhanced	Detected in some	
		Low lineage specificity	Detected in all			Low cancer specificity	Detected in all	
		Not detected	Not detected			Low cancer specificity	Detected in all	
		Not detected	Not detected			Low cancer specificity	Detected in many	
	naive B-cell: 11.1	Low lineage specificity	Detected in many			Low cancer specificity	Detected in many	
		Low lineage specificity	Detected in many			Low cancer specificity	Detected in many	
		Low lineage specificity	Detected in many			Not detected	Not detected	
5	basophil: 20.8;eosinophil: 13.4	Lineage enriched	Detected in many	6	granulocytes: 20.8	Cancer enhanced	Detected in many	
		Not detected	Not detected			Group enriched	Detected in some	6
		Low lineage specificity	Detected in all			Cancer enhanced	Detected in all	
		Low lineage specificity	Detected in all			Low cancer specificity	Detected in all	
	T-reg: 33.6	Group enriched	Detected in many	4	monocytes: 12.4;T-cells: 33.6	Low cancer specificity	Detected in all	
		Low lineage specificity	Detected in many			Low cancer specificity	Detected in many	
		Not detected	Not detected			Low cancer specificity	Detected in many	
	non-classical monocyte: 2.3	Low lineage specificity	Detected in single			Low cancer specificity	Detected in all	
5	gdT-cell: 1.2;MAIT T-cell: 3.9;memory CD4 T-cell: 3.1;memory CD8 T-cell: 2.4;naive CD4 T-cell: 3.3;na	Lineage enriched	Detected in single	7	T-cells: 3.9	Low cancer specificity	Detected in many	
		Low lineage specificity	Detected in all			Low cancer specificity	Detected in all	
4	classical monocyte: 200.3;intermediate monocyte: 179.4;myeloid DC: 121.5;neutrophil: 460.9;non-cl	Group enriched	Detected in all	48	dendritic cells: 121.5;granulocytes: 460.9;monocytes: 2	Cancer enhanced	Detected in many	
	neutrophil: 86.8	Group enriched	Detected in many	33	dendritic cells: 22.4;granulocytes: 86.8;monocytes: 65.9	Cancer enhanced	Detected in many	
		Not detected	Not detected			Cancer enhanced	Detected in some	
		Not detected	Not detected			Not detected	Not detected	
		Not detected	Not detected			Not detected	Not detected	
		Not detected	Not detected			Low cancer specificity	Detected in all	
		Low lineage specificity	Detected in single			Low cancer specificity	Detected in all	
		Not detected	Not detected			Cancer enriched	Detected in many	6
8	NK-cell: 2.3	Lineage enriched	Detected in single	8	NK-cells: 2.3	Cancer enhanced	Detected in many	
		Low lineage specificity	Detected in all			Cancer enhanced	Detected in many	
35	plasmacytoid DC: 69.3	Lineage enriched	Detected in single	302	dendritic cells: 69.3	Low cancer specificity	Detected in many	
4	intermediate monocyte: 59.4;non-classical monocyte: 97.0	Lineage enriched	Detected in many	5	monocytes: 97.0	Cancer enriched	Detected in some	7
	neutrophil: 509.3	Group enriched	Detected in all	11	dendritic cells: 207.0;granulocytes: 509.3;monocytes: 3	Cancer enriched	Detected in some	10
		Low lineage specificity	Detected in many			Low cancer specificity	Detected in all	
		Low lineage specificity	Detected in many			Low cancer specificity	Detected in all	
		Not detected	Not detected			Cancer enhanced	Detected in some	
		Low lineage specificity	Detected in many			Low cancer specificity	Detected in all	
	basophil: 396.7	Group enriched	Detected in many	155	granulocytes: 396.7;T-cells: 314.1	Cancer enhanced	Detected in some	
		Not detected	Not detected			Cancer enriched	Detected in single	22
9	basophil: 91.2	Lineage enriched	Detected in many	51	granulocytes: 91.2	Not detected	Not detected	
		Group enriched	Detected in many	8	dendritic cells: 3.3;granulocytes: 2.8;monocytes: 4.3	Low cancer specificity	Detected in many	
		Low lineage specificity	Detected in all			Cancer enhanced	Detected in many	
6	MAIT T-cell: 19.5	Lineage enriched	Detected in many	6	T-cells: 19.5	Low cancer specificity	Detected in all	
		Not detected	Not detected			Cancer enhanced	Detected in some	
		Low lineage specificity	Detected in all			Low cancer specificity	Detected in all	
		Low lineage specificity	Detected in many			Low cancer specificity	Detected in all	
	NK-cell: 118.7	Group enriched	Detected in many	18	NK-cells: 118.7;T-cells: 49.8	Low cancer specificity	Detected in some	
		Low lineage specificity	Detected in all			Cancer enhanced	Detected in all	
	neutrophil: 6461.2	Low lineage specificity	Detected in all			Cancer enriched	Detected in some	26
	T-reg: 8.2	Group enriched	Detected in many	5	granulocytes: 2.1;NK-cells: 2.1;T-cells: 8.2	Low cancer specificity	Detected in all	
		Low lineage specificity	Detected in all			Low cancer specificity	Detected in all	
		Not detected	Not detected			Low cancer specificity	Detected in many	
	gdT-cell: 2.7	Lineage enriched	Detected in single	6	T-cells: 2.7	Low cancer specificity	Detected in many	
		Low lineage specificity	Detected in all			Low cancer specificity	Detected in all	
	basophil: 1.2	Low lineage specificity	Detected in single			Not detected	Not detected	
	non-classical monocyte: 157.0	Lineage enriched	Detected in all	4	monocytes: 157.0	Low cancer specificity	Detected in all	
		Group enriched	Detected in all	5	granulocytes: 88.1;monocytes: 50.1	Low cancer specificity	Detected in all	
	neutrophil: 1.3	Lineage enriched	Detected in single	6	granulocytes: 1.3	Group enriched	Detected in many	5
		Low lineage specificity	Detected in all			Low cancer specificity	Detected in all	
		Low lineage specificity	Detected in all			Low cancer specificity	Detected in all	
		Low lineage specificity	Detected in all			Low cancer specificity	Detected in all	
		Low lineage specificity	Detected in all			Low cancer specificity	Detected in all	
9	basophil: 20.1	Lineage enriched	Detected in many	9	granulocytes: 20.1	Low cancer specificity	Detected in all	
		Low lineage specificity	Detected in single			Low cancer specificity	Detected in all	
		Low lineage specificity	Detected in all			Low cancer specificity	Detected in all	
		Low lineage specificity	Detected in all			Low cancer specificity	Detected in all	
		Low lineage specificity	Detected in all			Low cancer specificity	Detected in all	
		Not detected	Not detected			Cancer enhanced	Detected in some	
		Low lineage specificity	Detected in all			Low cancer specificity	Detected in all	
	basophil: 48.9	Group enriched	Detected in many	5	granulocytes: 48.9;monocytes: 24.0	Low cancer specificity	Detected in all	
		Low lineage specificity	Detected in all			Low cancer specificity	Detected in all	

RNA cell line specific nTPM	RNA tissue cell type enrichment	RNA mouse brain regional specificity	RNA mouse brain regional distribution
	Thyroid gland - Thyroid glandular cells	Low region specificity	Detected in all
		Low region specificity	Detected in all
	Adrenal gland - Adrenal cortex cells, Minor Salivary Gland - Macrophages	Low region specificity	Detected in all
		Low region specificity	Detected in all
colorectal cancer: 26.0;liver cancer: 17.4	Adrenal gland - Adrenal cortex cells, Prostate - Endothelial cells, Spleen - NK-cells (Spleen)	Low region specificity	Detected in all
skin cancer: 9.0	Colon - Enteric glia cells, Heart muscle - Cardiomyocytes, Minor Salivary Gland - Fibroblasts, Skeletal muscle - Skeletal myocytes, Spleen - B-cells (Spleen), Stomach - Fibroblasts	Low region specificity	Detected in many
	Stomach - Mitotic cells (Stomach), Testis - Early spermatids	Low region specificity	Detected in all
	Thyroid gland - Thyroid glandular cells	Low region specificity	Detected in all
	Minor Salivary Gland - Minor salivary glandular cells, Prostate - Prostate basal glandular cells, Stomach - Gastric mucous cells	Low region specificity	Detected in many
prostate cancer: 68.3	Lung - Alveolar cells type 2, Prostate - Prostate glandular cells, Testis - Spermatogonia	Low region specificity	Detected in all
liver cancer: 9.2	Adipose visceral - Adipocytes (Visceral), Kidney - Proximal tubular cells, Kidney - Proximal tubular cells, Liver - Hepatocytes, Lung - Alveolar cells type 2	Not detected	Not detected
	Liver - Hepatocytes	Not detected	Not detected
Kidney cancer: 106.8	Testis - Sertoli cells	Low region specificity	Detected in all
Leukemia: 168.9	Kidney - Endothelial cells, Minor Salivary Gland - Endothelial cells, Prostate - Fibroblasts, Stomach - Parietal cells	Low region specificity	Detected in many
	Thyroid gland - Thyroid glandular cells	Low region specificity	Detected in all
Brain cancer: 41.1;Kidney cancer: 31.8	Prostate - Fibroblasts, Skeletal muscle - Fibroblasts	Low region specificity	Detected in many
pancreatic cancer: 9.9	Adrenal gland - Macrophages, Spleen - B-cells (Spleen), Stomach - Gastric mucous cells, Thyroid gland - T-cells	Not detected	Not detected
	Adrenal gland - Fibroblasts, Skin - Macrophages, Spleen - Fibroblasts_2	Low region specificity	Detected in all
	Adipose subcutaneous - Endothelial cells, Adipose visceral - Endothelial cells, Adrenal gland - Endothelial cells, Breast - Endothelial cells, Colon - Endothelial cells, Kidney - Endothelial cells, Liver - Vascular endothelial cells,	Low region specificity	Detected in all
	Liver - Hepatocytes, Testis - Sertoli cells, Thyroid gland - Thyroid glandular cells	Low region specificity	Detected in all
	Breast - Fibroblasts, Kidney - Fibroblasts, Prostate - Smooth muscle cells	Low region specificity	Detected in all
Neuroblastoma: 43.0	Adrenal gland - Adrenal medulla cells, Breast - Endothelial cells, Minor Salivary Gland - Endothelial cells, Pituitary gland - Lactotropes, Testis - Late spermatids	Low region specificity	Detected in all
	Adrenal gland - Fibroblasts, Breast - Fibroblasts	Low region specificity	Detected in all
Bone cancer: 29.9	Adrenal gland - Fibroblasts, Breast - Fibroblasts, Kidney - Fibroblasts, Liver - Hepatic stellate cells, Minor Salivary Gland - Fibroblasts, Pituitary gland - Fibroblasts, Skeletal muscle - Fibroblasts, Spleen - Fibroblasts_1, Stoma	Low region specificity	Detected in all
	Adrenal gland - Adrenal medulla cells, Stomach - Fibroblasts	Low region specificity	Detected in all
	Adipose visceral - Mesothelial cells, Kidney - Fibroblasts, Pituitary gland - Fibroblasts, Spleen - Fibroblasts_1, Stomach - Parietal cells	Region enhanced	Detected in some
Bone cancer: 16.6	Adrenal gland - Fibroblasts, Breast - Adipocytes (Breast), Colon - Colon enteroendocrine cells, Prostate - Prostate glandular cells, Stomach - Gastric mucous cells	Low region specificity	Detected in many
Kidney cancer: 51.9	Adipose subcutaneous - Adipocytes (Subcutaneous), Heart muscle - Cardiomyocytes, Kidney - Podocytes, Prostate - Fibroblasts, Skin - Eccrine sweat gland cells, Testis - Sertoli cells	Low region specificity	Detected in all
	Breast - Breast myoepithelial cells, Prostate - Urothelial cells, Skin - Keratinocyte (other)	Low region specificity	Detected in all
	Adrenal gland - Fibroblasts, Breast - Adipocytes (Breast), Skin - Fibroblasts_2, Spleen - Fibroblasts_2	Low region specificity	Detected in all
head and neck cancer: 50.9	Colon - Endothelial cells	Low region specificity	Detected in all
	Adipose subcutaneous - Endothelial cells, Heart muscle - Endothelial cells, Lung - Alveolar cells type 1, Skeletal muscle - Endothelial cells, Spleen - Endothelial cells	Low region specificity	Detected in all
Kidney cancer: 2.2	Colon - Enteric glia cells, Lung - Alveolar cells type 1	Low region specificity	Detected in some
	Adrenal gland - Fibroblasts, Thyroid gland - Fibroblasts	Low region specificity	Detected in all
	Adipose subcutaneous - Adipose progenitor cells, Breast - Fibroblasts, Prostate - Smooth muscle cells, Skin - Keratinocyte (granular), Spleen - Smooth muscle cells	Low region specificity	Detected in single
	Breast - Endothelial cells, Testis - Early spermatids	Low region specificity	Detected in all
Gallbladder cancer: 2714.5	Colon - Colon enterocytes, Pituitary gland - Thyrotropes, Stomach - Gastric mucous cells	Low region specificity	Detected in all
liver cancer: 7.3	Stomach - Gastric mucous cells, Testis - Spermatogonia		
	Prostate - Smooth muscle cells, Skeletal muscle - Skeletal myocytes, Testis - Early spermatids, Thyroid gland - Thyroid glandular cells	Low region specificity	Detected in all
Neuroblastoma: 84.2	Adrenal gland - Adrenal medulla cells, Colon - Enteric glia cells, Pituitary gland - Lactotropes, Testis - Early spermatids, Testis - Late spermatids	Low region specificity	Detected in many
breast cancer: 26.1	Adrenal gland - Macrophages, Breast - Breast glandular cells, Kidney - Macrophages, Pancreas - Macrophages, Prostate - Prostate glandular cells, Skin - Sebaceous gland cells	Low region specificity	Detected in many
		Low region specificity	Detected in all
Bone cancer: 21.5	Adrenal gland - Adrenal medulla cells, Testis - Early spermatids, Testis - Late spermatids	Low region specificity	Detected in all
testis cancer: 33.5	Skeletal muscle - Skeletal myocytes, Testis - Spermatogonia	Low region specificity	Detected in all
Neuroblastoma: 43.8;Rhabdoid: 31.6	Adipose subcutaneous - Adipose progenitor cells, Colon - Enteric glia cells, Heart muscle - Cardiomyocytes, Liver - Hepatic stellate cells, Lung - Fibroblasts_2, Prostate - Fibroblasts, Spleen - Fibroblasts_1, Testis - Early spe	Low region specificity	Detected in all
	Colon - Enteric glia cells, Liver - Hepatic stellate cells, Lung - Alveolar cells type 2, Spleen - Fibroblasts_2, Thyroid gland - Thyroid glandular cells	Low region specificity	Detected in all
	Adrenal gland - Adrenal cortex cells, Testis - Spermatocytes	Low region specificity	Detected in all
	Adrenal gland - Adrenal cortex cells, Breast - Breast glandular cells	Low region specificity	Detected in all
	Skeletal muscle - Endothelial cells, Spleen - B-cells (Spleen), Testis - Early spermatids	Low region specificity	Detected in all
	Adipose subcutaneous - Adipose progenitor cells, Colon - Enteric glia cells, Liver - Vascular endothelial cells, Minor Salivary Gland - Endothelial cells, Testis - Late spermatids	Low region specificity	Detected in all
Esophageal cancer: 62.2;pancreatic cancer: 72.4	Breast - Breast myoepithelial cells, Kidney - Fibroblasts, Liver - Hepatocytes, Lung - Fibroblasts_1, Pituitary gland - Fibroblasts, Testis - Late spermatids	Low region specificity	Detected in many
	Spleen - Fibroblasts_1, Testis - Spermatogonia, Thyroid gland - Thyroid glandular cells	Low region specificity	Detected in all
cervical cancer: 7.6	Adipose visceral - Endothelial cells, Breast - Endothelial cells, Colon - Endothelial cells, Minor Salivary Gland - Endothelial cells, Prostate - Endothelial cells, Skin - Endothelial cells, Testis - Late spermatids	Region enriched	Detected in many
	Adrenal gland - Adrenal cortex cells, Liver - Hepatocytes	Low region specificity	Detected in all
Adrenocortical cancer: 455.0	Adipose visceral - Macrophages, Kidney - Proximal tubular cells, Liver - Hepatocytes, Prostate - Prostate glandular cells, Skeletal muscle - Macrophages, Spleen - Neutrophils_2, Stomach - Macrophages	Low region specificity	Detected in all
	Skeletal muscle - Fibroblasts	Low region specificity	Detected in all
colorectal cancer: 18.8;Gastric cancer: 33.6;Kidney cancer: 22.6	Lung - Respiratory ciliated cells, Pancreas - Ductal cells	Not detected	Not detected
	Lung - Alveolar cells type 1, Minor Salivary Gland - Minor salivary glandular cells, Pancreas - Ductal cells, Prostate - Prostate glandular cells, Spleen - Neutrophils_2	Low region specificity	Detected in all
	Adipose visceral - Adipose progenitor cells, Prostate - Smooth muscle cells	Low region specificity	Detected in all
	Adipose subcutaneous - Smooth muscle cells, Prostate - Smooth muscle cells, Testis - Spermatogonia	Low region specificity	Detected in all
Kidney cancer: 43.1;Leukemia: 69.3;lymphoma: 96.1;Myeloma: 25.7	Adrenal gland - Macrophages, Colon - Macrophages, Heart muscle - Macrophages, Minor Salivary Gland - Macrophages, Prostate - Macrophages, Skeletal muscle - Macrophages, Spleen - Neutrophils_2, Testis - Macrophag	Low region specificity	Detected in many
	Pituitary gland - Undifferentiated cells (Pituitary gland), Testis - Spermatocytes, Testis - Spermatogonia	Low region specificity	Detected in all
Leukemia: 27.3	Adipose subcutaneous - Macrophages, Adrenal gland - Macrophages, Skin - Langerhans cells, Spleen - Neutrophils_2	Not detected	Not detected
skin cancer: 43.6	Breast - Endothelial cells, Prostate - Endothelial cells	Group enriched	Detected in all
	Adrenal gland - Adrenal cortex cells	Low region specificity	Detected in all
Bile duct cancer: 50.3	Breast - Endothelial cells, Minor Salivary Gland - Endothelial cells, Prostate - Endothelial cells, Skeletal muscle - Endothelial cells, Testis - Endothelial cells	Region enriched	Detected in many
Leukemia: 1.5;Neuroblastoma: 1.1	Testis - Sertoli cells		
	Adipose visceral - Adipocytes (Visceral), Kidney - Proximal tubular cells, Liver - Hepatocytes, Prostate - Prostate glandular cells, Testis - Early spermatids	Low region specificity	Detected in all
Gallbladder cancer: 403.6	Lung - Alveolar cells type 2, Minor Salivary Gland - Minor salivary gland basal cells, Skin - Keratinocyte (other)	Not detected	Not detected
Brain cancer: 2.5;Rhabdoid: 1.7	Colon - Enteric glia cells, Heart muscle - Cardiomyocytes, Skeletal muscle - Skeletal myocytes	Low region specificity	Detected in all
	Liver - Hepatocytes, Minor Salivary Gland - Minor salivary glandular cells	Low region specificity	Detected in all
Leukemia: 69.1;lymphoma: 63.6	Adipose visceral - T-cells, Breast - T-cells, Colon - Enteric glia cells, Liver - Hepatic stellate cells, Skeletal muscle - Fibroblasts, Stomach - Fibroblasts, Testis - Early spermatids, Testis - Late spermatids	Low region specificity	Detected in all
	Breast - Endothelial cells, Colon - Endothelial cells, Minor Salivary Gland - Endothelial cells, Prostate - Endothelial cells, Testis - Sertoli cells	Low region specificity	Detected in all
	Adrenal gland - Fibroblasts, Colon - Enteric glia cells, Pancreas - Endothelial cells, Stomach - Mitotic cells (Stomach), Testis - Spermatocytes	Low region specificity	Detected in all
	Prostate - Fibroblasts, Spleen - Endothelial cells, Stomach - Gastric mucous cells, Thyroid gland - Fibroblasts	Low region specificity	Detected in all
	Adipose subcutaneous - Adipose progenitor cells, Heart muscle - Fibroblasts, Liver - Vascular endothelial cells, Skeletal muscle - Fibroblasts, Stomach - Fibroblasts, Testis - Late spermatids	Low region specificity	Detected in all
Leukemia: 93.4;lymphoma: 73.9;Myeloma: 97.1	Colon - T-cells, Heart muscle - Macrophages, Kidney - T-cells, Liver - T-cells, Prostate - T-cells, Skeletal muscle - Macrophages, Skin - T-cells, Thyroid gland - T-cells	Low region specificity	Detected in many
	Prostate - T-cells, Spleen - B-cells (Spleen), Testis - Endothelial cells, Thyroid gland - T-cells	Low region specificity	Detected in all
		Low region specificity	Detected in all
Sarcoma: 1.3	Adipose subcutaneous - Endothelial cells, Adipose visceral - Endothelial cells, Adrenal gland - Endothelial cells, Breast - Endothelial cells, Colon - Endothelial cells, Liver - Vascular endothelial cells, Minor Salivary Gland - End	Low region specificity	Detected in all
	Skin - Keratinocyte (granular), Spleen - B-cells (Spleen)	Low region specificity	Detected in all
	Kidney - Podocytes, Lung - Alveolar cells type 1, Testis - Spermatogonia	Low region specificity	Detected in all
	Adipose visceral - Mesothelial cells, Stomach - Gastric mucous cells, Thyroid gland - Thyroid glandular cells	Low region specificity	Detected in many
Kidney cancer: 169.9	Thyroid gland - T-cells	Low region specificity	Detected in all
Leukemia: 3.8	Lung - Respiratory ciliated cells, Testis - Early spermatids, Testis - Late spermatids, Thyroid gland - T-cells	Low region specificity	Detected in many
	Adipose subcutaneous - Macrophages, Adipose visceral - Macrophages, Adrenal gland - Macrophages, Breast - Macrophages, Heart muscle - Macrophages, Lung - Neutrophils_2, Minor Salivary Gland - Macrophages, Skel	Low region specificity	Detected in all
		Low region specificity	Detected in all
Neuroblastoma: 27.0	Colon - Enteric glia cells, Minor Salivary Gland - Minor salivary glandular cells	Low region specificity	Detected in all
	Adrenal gland - Fibroblasts, Prostate - Endothelial cells, Testis - Early spermatids, Testis - Late spermatids	Low region specificity	Detected in all
liver cancer: 99.3	Adipose subcutaneous - Macrophages, Breast - Macrophages, Liver - Hepatocytes, Lung - Neutrophils_2, Skin - Keratinocyte (granular), Spleen - Neutrophils_2	Not detected	Not detected
		Low region specificity	Detected in all
	Adipose subcutaneous - Adipose progenitor cells, Liver - Hepatic stellate cells, Skeletal muscle - Fibroblasts, Stomach - Fibroblasts, Thyroid gland - Fibroblasts	Low region specificity	Detected in all
	Adrenal gland - Adrenal cortex cells, Testis - Early spermatids, Thyroid gland - Thyroid glandular cells	Low region specificity	Detected in all
		Not detected	Not detected
testis cancer: 4.4	Lung - Respiratory ciliated cells	Not detected	Not detected
	Testis - Late spermatids	Low region specificity	Detected in all
	Adrenal gland - Adrenal cortex cells, Liver - Hepatocytes, Testis - Early spermatids	Low region specificity	Detected in all
	Adipose subcutaneous - Adipose progenitor cells	Low region specificity	Detected in all
Bone cancer: 4.9;Neuroblastoma: 9.1	Adrenal gland - Adrenal cortex cells, Colon - Colon enteroendocrine cells, Pituitary gland - Gonadotropes	Low region specificity	Detected in all
testis cancer: 204.8	Prostate - Smooth muscle cells, Skin - Keratinocyte (other)	Low region specificity	Detected in all
		Low region specificity	Detected in all
	Heart muscle - Cardiomyocytes, Thyroid gland - Thyroid glandular cells	Low region specificity	Detected in all
prostate cancer: 5.2	Colon - Colon enteroendocrine cells, Skin - Outer root sheath cells, Testis - Early spermatids	Low region specificity	Detected in all
	Skeletal muscle - Fibroblasts, Thyroid gland - Thyroid glandular cells	Low region specificity	Detected in many
Myeloma: 105.7	Adipose visceral - Adipose progenitor cells, Adrenal gland - Fibroblasts, Breast - Fibroblasts, Prostate - Smooth muscle cells, Testis - Sertoli cells	Low region specificity	Detected in all
Adrenocortical cancer: 812.7	Testis - Sertoli cells	Low region specificity	Detected in all
	Skeletal muscle - Endothelial cells, Thyroid gland - T-cells	Low region specificity	Detected in all
	Adipose subcutaneous - Adipose progenitor cells, Kidney - Fibroblasts, Pancreas - Exocrine glandular cells	Low region specificity	Detected in all
	Adipose visceral - Mesothelial cells, Breast - Breast glandular cells, Minor Salivary Gland - Minor salivary gland basal cells, Prostate - Prostate glandular cells, Skin - Keratinocyte (other), Testis - Spermatogonia	Low region specificity	Detected in all
	Adipose subcutaneous - Endothelial cells, Minor Salivary Gland - Minor salivary glandular cells, Prostate - Prostate glandular cells, Stomach - Gastric mucous cells	Low region specificity	Detected in some
Adrenocortical cancer: 573.1	Adipose subcutaneous - Adipose progenitor cells, Prostate - Prostate glandular cells, Spleen - Neutrophils_2	Low region specificity	Detected in all
cervical cancer: 18.2		Not detected	Not detected
	Skeletal muscle - Fibroblasts, Skin - Outer root sheath cells, Testis - Spermatogonia, Thyroid gland - Thyroid glandular cells	Low region specificity	Detected in many
Leukemia: 55.4;Myeloma: 48.7	Adrenal gland - Macrophages, Lung - Neutrophils_2, Spleen - Neutrophils_2, Testis - Early spermatids, Thyroid gland - T-cells	Low region specificity	Detected in all
Leukemia: 18.6;lymphoma: 53.4	Adrenal gland - Macrophages, Spleen - B-cells (Spleen), Stomach - Gastric mucous cells	Low region specificity	Detected in many
	Adipose visceral - Endothelial cells, Lung - Endothelial cells, Skeletal muscle - Endothelial cells, Stomach - Endothelial cells, Thyroid gland - Endothelial cells	Low region specificity	Detected in all
cervical cancer: 2.6;lung cancer: 4.6;prostate cancer: 2.0;skin cancer	Breast - Endothelial cells, Colon - Endothelial cells, Minor Salivary Gland - Endothelial cells, Prostate - Endothelial cells, Skeletal muscle - Endothelial cells, Spleen - Neutrophils_2, Stomach - Endothelial cells	Low region specificity	Detected in many
	Adrenal gland - Fibroblasts, Heart muscle - Fibroblasts, Lung - Fibroblasts_2, Skeletal muscle - Fibroblasts, Spleen - Fibroblasts_1, Thyroid gland - Fibroblasts	Low region specificity	Detected in all
	Pancreas - Endothelial cells, Testis - Early spermatids, Testis - Spermatocytes	Low region specificity	Detected in all
Leukemia: 79.9;lymphoma: 84.3;Myeloma: 21.2	Adipose subcutaneous - Macrophages, Adrenal gland - Macrophages, Breast - Macrophages, Liver - Kupffer cells, Skeletal muscle - Macrophages, Spleen - B-cells (Spleen)	Low region specificity	Detected in many
Adrenocortical cancer: 16.5	Adipose subcutaneous - Smooth muscle cells, Prostate - Smooth muscle cells, Stomach - Chief cells, Testis - Early spermatids	Low region specificity	Detected in some
	Adipose visceral - Mesothelial cells, Breast - Breast glandular cells, Heart muscle - Macrophages, Liver - Hepatocytes, Minor Salivary Gland - Minor salivary glandular cells, Prostate - Prostate glandular cells, Testis - Early spe	Low region specificity	Detected in all
prostate cancer: 57.3	Colon - Colon enterocytes, Liver - Cholangiocytes, Minor Salivary Gland - Minor salivary gland basal cells, Pituitary gland - Corticotropes, Prostate - Prostate glandular cells, Stomach - Gastric mucous cells, Testis - Early spe	Low region specificity	Detected in all
	Stomach - Mitotic cells (Stomach)	Low region specificity	Detected in all
		Low region specificity	Detected in all
	Liver - Hepatocytes	Not detected	Not detected

Kidney cancer: 114.9	Adipose visceral - Mesothelial cells, Liver - Cholangiocytes, Prostate - Smooth muscle cells, Testis - Late spermatids	Region enhanced	Detected in many
Brain cancer: 3.9	Prostate - Smooth muscle cells, Testis - Early spermatids, Testis - Late spermatids, Thyroid gland - Smooth muscle cells	Low region specificity	Detected in all
	Adrenal gland - Adrenal cortex cells, Colon - Enteric glia cells, Lung - Alveolar cells type 1, Minor Salivary Gland - Minor salivary glandular cells	Low region specificity	Detected in all
Rhabdoid: 12.7;Sarcoma: 23.1	Adrenal gland - Endothelial cells, Colon - Endothelial cells, Lung - Endothelial cells, Prostate - Endothelial cells, Skin - Endothelial cells, Stomach - Endothelial cells	Low region specificity	Detected in all
	Adipose subcutaneous - Smooth muscle cells, Adrenal gland - Fibroblasts, Kidney - Fibroblasts, Lung - Smooth muscle cells, Prostate - Smooth muscle cells, Spleen - Fibroblasts_2	Low region specificity	Detected in all
	Testis - Sertoli cells	Low region specificity	Detected in all
	Breast - Endothelial cells, Lung - Endothelial cells, Minor Salivary Gland - Endothelial cells, Prostate - Endothelial cells	Low region specificity	Detected in all
	Testis - Early spermatids	Low region specificity	Detected in all
	Adrenal gland - Fibroblasts, Colon - Smooth muscle cells, Minor Salivary Gland - Endothelial cells, Prostate - Smooth muscle cells	Low region specificity	Detected in all
cervical cancer: 65.0	Adrenal gland - Endothelial cells, Breast - Endothelial cells, Kidney - Endothelial cells, Liver - Sinusoid Endothelial cells, Spleen - Endothelial cells	Low region specificity	Detected in all
Neuroblastoma: 6.6	Heart muscle - Cardiomyocytes, Skeletal muscle - Skeletal myocytes, Testis - Early spermatids, Testis - Late spermatids	Not detected	Not detected
		Low region specificity	Detected in all
		Low region specificity	Detected in all
		Low region specificity	Detected in all
	Testis - Early spermatids	Low region specificity	Detected in all
Myeloma: 50.1	Colon - T-cells, Spleen - B-cells (Spleen), Testis - Spermatogonia, Thyroid gland - T-cells	Low region specificity	Detected in all
	Heart muscle - Cardiomyocytes, Liver - Vascular endothelial cells	Low region specificity	Detected in all
Bone cancer: 4.3;liver cancer: 2.0;Neuroblastoma: 7.5	Stomach - Chief cells, Testis - Late spermatids	Region enhanced	Detected in many
Rhabdoid: 2.5;testis cancer: 9.7	Adipose visceral - Mesothelial cells, Stomach - Parietal cells	Low region specificity	Detected in many
	Breast - Adipocytes (Breast), Lung - Alveolar cells type 2, Testis - Spermatocytes	Low region specificity	Detected in all
Myeloma: 29.9	Breast - Plasma cells, Minor Salivary Gland - Plasma cells	Not detected	Not detected
lymphoma: 18.7	Adipose subcutaneous - T-cells, Skin - T-cells	Not detected	Not detected
	Breast - Endothelial cells, Heart muscle - Endothelial cells, Kidney - Endothelial cells, Prostate - Endothelial cells, Skin - Endothelial cells	Low region specificity	Detected in many
	Adrenal gland - Endothelial cells, Liver - Hepatic stellate cells, Testis - Early spermatids, Testis - Late spermatids, Thyroid gland - Fibroblasts	Low region specificity	Detected in many
lymphoma: 6.1	Adipose subcutaneous - Macrophages, Adipose visceral - Macrophages, Adrenal gland - Macrophages, Breast - Macrophages, Colon - Macrophages, Heart muscle - Macrophages, Kidney - Macrophages, Lung - Macropha	Low region specificity	Detected in single
lymphoma: 19.2;Myeloma: 11.7	Adipose subcutaneous - Macrophages, Adipose visceral - Macrophages, Breast - Plasma cells, Colon - Macrophages, Heart muscle - Macrophages, Liver - Kupffer cells, Lung - Macrophages, Pancreas - Macrophages, Skel	Low region specificity	Detected in many
Leukemia: 11.8;lymphoma: 12.5	Adipose subcutaneous - T-cells, Adipose visceral - T-cells, Adrenal gland - T-cells, Breast - T-cells, Colon - T-cells, Kidney - T-cells, Liver - T-cells, Lung - T-cells, Minor Salivary Gland - T-cells, Pancreas - T-cells, Pituitary glan	Not detected	Not detected
cervical cancer: 10.0	Adrenal gland - Macrophages		
Leukemia: 8.2;lymphoma: 10.3	Adipose subcutaneous - T-cells, Adipose visceral - T-cells, Colon - T-cells, Lung - T-cells, Minor Salivary Gland - T-cells, Prostate - T-cells, Skeletal muscle - Fibroblasts, Stomach - T-cells, Thyroid gland - T-cells	Not detected	Not detected
	Kidney - Endothelial cells	Low region specificity	Detected in many
Myeloma: 98.6	Adipose visceral - T-cells, Breast - T-cells, Colon - T-cells, Kidney - T-cells, Thyroid gland - T-cells	Region enhanced	Detected in single
	Adipose subcutaneous - Endothelial cells, Thyroid gland - Thyroid glandular cells	Low region specificity	Detected in all
Leukemia: 53.6	Adipose subcutaneous - Macrophages, Adipose visceral - Macrophages, Adrenal gland - Macrophages, Breast - Macrophages, Colon - Macrophages, Heart muscle - Macrophages, Kidney - Macrophages, Liver - Kupffer cells, Lung - Macrophages, Minor Salivary Gland - Macrophages, Pancreas - Macropha		
Leukemia: 25.9;lymphoma: 43.3;Myeloma: 35.3	Lung - Alveolar cells type 2, Prostate - Prostate glandular cells, Skeletal muscle - Skeletal myocytes, Skeletal muscle - Skeletal myocytes, Stomach - Plasma cells	Low region specificity	Detected in all
Leukemia: 125.7	Adipose subcutaneous - T-cells, Adipose visceral - T-cells, Adrenal gland - T-cells, Breast - T-cells, Colon - T-cells, Heart muscle - T-cells, Kidney - T-cells, Liver - T-cells, Lung - T-cells, Minor Salivary Gland - T-cells, Pancreas	Not detected	Not detected
Leukemia: 19.9	Adipose subcutaneous - T-cells, Adipose visceral - T-cells, Adrenal gland - T-cells, Colon - T-cells, Kidney - T-cells, Liver - T-cells, Lung - T-cells, Minor Salivary Gland - T-cells, Pancreas - T-cells, Pituitary gland - T-cells, Prost	Not detected	Not detected
Leukemia: 40.6;lymphoma: 27.6;Myeloma: 23.9	Adrenal gland - Macrophages, Minor Salivary Gland - Macrophages, Skeletal muscle - Macrophages, Spleen - Macrophages, Stomach - Macrophages	Region enriched	Detected in many
lymphoma: 97.7	Spleen - B-cells (Spleen)	Low region specificity	Detected in many
Leukemia: 2.6	Adipose subcutaneous - T-cells, Adipose visceral - T-cells, Adrenal gland - T-cells, Breast - T-cells, Colon - T-cells, Lung - T-cells, Minor Salivary Gland - T-cells, Prostate - T-cells, Skin - T-cells, Spleen - T-cells	Not detected	Not detected
Adrenocortical cancer: 1400.7	Adipose subcutaneous - Adipose progenitor cells, Prostate - Urothelial cells	Low region specificity	Detected in all
	Adipose subcutaneous - Adipose progenitor cells	Low region specificity	Detected in all
lymphoma: 657.0	Pituitary gland - Somatotropes, Spleen - B-cells (Spleen)	Not detected	Not detected
Leukemia: 11.4;lymphoma: 8.2	Adipose subcutaneous - Macrophages, Adipose visceral - Macrophages, Adrenal gland - Macrophages, Liver - Kupffer cells, Minor Salivary Gland - Macrophages, Skeletal muscle - Macrophages	Low region specificity	Detected in all
lymphoma: 54.5	Adipose subcutaneous - Macrophages, Adipose visceral - Macrophages, Adrenal gland - Macrophages, Breast - Macrophages, Colon - Macrophages, Heart muscle - Macrophages, Kidney - Macrophages, Minor Salivary Gla	Low region specificity	Detected in many
Leukemia: 4.0;lymphoma: 5.1	Adipose subcutaneous - T-cells, Adipose visceral - T-cells, Adrenal gland - T-cells, Breast - T-cells, Colon - T-cells, Kidney - T-cells, Liver - T-cells, Lung - T-cells, Minor Salivary Gland - T-cells, Pancreas - T-cells, Pituitary glan	Not detected	Not detected
Leukemia: 8.7;Myeloma: 33.2	Adipose subcutaneous - T-cells, Adipose visceral - T-cells, Adrenal gland - T-cells, Breast - T-cells, Colon - T-cells, Kidney - T-cells, Liver - T-cells, Lung - T-cells, Minor Salivary Gland - T-cells, Pancreas - T-cells, Pituitary gland - Corticotropes, Prostate - T-cells, Skin - T-cells, Spleen - T-cells, Stomach - T-cells		
		Low region specificity	Detected in all
		Low region specificity	Detected in all
	Minor Salivary Gland - Minor salivary glandular cells, Spleen - Fibroblasts_1	Low region specificity	Detected in all
	Breast - Endothelial cells, Prostate - Smooth muscle cells, Testis - Early spermatids	Low region specificity	Detected in all
		Low region specificity	Detected in all
	Adipose visceral - Mesothelial cells, Colon - Colon enterocytes, Liver - Hepatocytes, Lung - Alveolar cells type 2, Prostate - Prostate glandular cells, Thyroid gland - Thyroid glandular cells	Low region specificity	Detected in many
Brain cancer: 66.5;testis cancer: 95.3	Adipose visceral - Endothelial cells, Lung - Fibroblasts_1, Pituitary gland - Fibroblasts, Testis - Early spermatids, Testis - Late spermatids	Low region specificity	Detected in all
Sarcoma: 32.4		Region enriched	Detected in many
Bile duct cancer: 61.7;colorectal cancer: 158.4;Gastric cancer: 75.7;p	Colon - Colon enterocytes	Not detected	Not detected
	Adrenal gland - Adrenal cortex cells, Colon - Enteric glia cells, Heart muscle - Cardiomyocytes, Kidney - Proximal tubular cells, Pituitary gland - Undifferentiated cells (Pituitary gland), Prostate - Smooth muscle cells, Stomach	Low region specificity	Detected in all
prostate cancer: 8.9	Prostate - Prostate glandular cells	Low region specificity	Detected in some
head and neck cancer: 157.4	Adipose visceral - Mesothelial cells, Spleen - Fibroblasts_1	Region enhanced	Detected in single
Brain cancer: 9.6	Minor Salivary Gland - Smooth muscle cells, Skin - Keratinocyte (granular), Spleen - Endothelial cells	Low region specificity	Detected in all
ovarian cancer: 12.0;pancreatic cancer: 9.6;Rhabdoid: 12.8	Adipose subcutaneous - Endothelial cells, Adipose visceral - Endothelial cells, Adrenal gland - Endothelial cells, Breast - Endothelial cells, Colon - Endothelial cells, Heart muscle - Endothelial cells, Kidney - Endothelial cells,	Low region specificity	Detected in all
Kidney cancer: 53.6;ovarian cancer: 81.5;Uterine cancer: 44.9	Breast - Endothelial cells, Prostate - Endothelial cells, Thyroid gland - Fibroblasts	Low region specificity	Detected in many
	Testis - Sertoli cells	Low region specificity	Detected in all
	Lung - Macrophages	Low region specificity	Detected in all
colorectal cancer: 196.8;Gallbladder cancer: 484.8	Colon - Colon enterocytes, Minor Salivary Gland - Minor salivary gland basal cells, Prostate - Urothelial cells, Skin - Eccrine sweat gland cells, Stomach - Gastric mucous cells, Thyroid gland - Parafollicular cells		
Bladder cancer: 2.3;colorectal cancer: 3.4;ovarian cancer: 3.2	Colon - Colon enterocytes		
	Breast - Breast glandular cells, Minor Salivary Gland - Minor salivary gland basal cells, Pituitary gland - Corticotropes, Spleen - B-cells (Spleen)	Low region specificity	Detected in many
	Heart muscle - Endothelial cells, Minor Salivary Gland - Minor salivary glandular cells	Low region specificity	Detected in all
		Low region specificity	Detected in all
	Adipose visceral - Mesothelial cells, Colon - Colon enterocytes, Kidney - Macrophages, Minor Salivary Gland - Minor salivary glandular cells, Skin - Keratinocyte (granular), Stomach - Gastric mucous cells	Low region specificity	Detected in many
testis cancer: 34.7	Adipose subcutaneous - Smooth muscle cells, Adipose visceral - Mesothelial cells, Lung - Alveolar cells type 1, Minor Salivary Gland - Minor salivary glandular cells, Stomach - Chief cells, Thyroid gland - Thyroid glandular ce	Low region specificity	Detected in all
	Testis - Spermatocytes	Low region specificity	Detected in all
Myeloma: 8.4	Heart muscle - Smooth muscle cells, Minor Salivary Gland - Minor salivary glandular cells, Skin - Eccrine sweat gland cells	Low region specificity	Detected in many
	Adrenal gland - Adrenal medulla cells, Colon - Colon enteroendocrine cells, Skin - Keratinocyte (granular), Testis - Early spermatids, Testis - Late spermatids	Low region specificity	Detected in many
Sarcoma: 107.0	Pituitary gland - Gonadotropes, Testis - Early spermatids, Testis - Late spermatids	Not detected	Not detected
prostate cancer: 26.6	Pituitary gland - Corticotropes, Prostate - Prostate glandular cells, Skin - Eccrine sweat gland cells	Low region specificity	Detected in some
Neuroblastoma: 170.3	Adrenal gland - Adrenal medulla cells, Colon - Colon enteroendocrine cells	Low region specificity	Detected in many
	Adrenal gland - Adrenal medulla cells, Colon - Colon enteroendocrine cells, Prostate - Prostate glandular cells, Testis - Spermatocytes, Testis - Spermatogonia	Low region specificity	Detected in many
Neuroblastoma: 34.5	Adrenal gland - Adrenal medulla cells, Colon - Colon enteroendocrine cells, Skin - Inner root sheath cells, Skin - Outer root sheath cells, Testis - Early spermatids, Testis - Late spermatids	Group enriched	Detected in some
Sarcoma: 23.7		Not detected	Not detected
	Lung - Respiratory ciliated cells, Prostate - Prostate glandular cells, Stomach - Gastric mucous cells, Testis - Early spermatids, Testis - Late spermatids	Low region specificity	Detected in all
breast cancer: 24.6;Esophageal cancer: 13.5;Gallbladder cancer: 13.:	Lung - Respiratory ciliated cells, Prostate - Urothelial cells, Skin - Mitotic cells (Skin), Testis - Early spermatids, Testis - Late spermatids	Not detected	Not detected
	Kidney - Ascending Loop of Henle cells, Liver - Hepatocytes	Low region specificity	Detected in all
ovarian cancer: 29.3	Adrenal gland - Fibroblasts, Pituitary gland - Fibroblasts, Prostate - Fibroblasts, Stomach - Fibroblasts, Testis - Sertoli cells	Region enriched	Detected in all
liver cancer: 2.9;testis cancer: 3.2	Breast - Breast myoepithelial cells, Kidney - Ascending Loop of Henle cells, Skin - Inner root sheath cells, Skin - Outer root sheath cells	Region enhanced	Detected in many
Bile duct cancer: 127.7;Kidney cancer: 98.9	Adipose subcutaneous - Adipocytes (Subcutaneous), Adipose visceral - Adipocytes (Visceral), Adrenal gland - Adrenal cortex cells, Breast - Adipocytes (Breast), Kidney - Proximal tubular cells	Group enriched	Detected in many
	Skin - Outer root sheath cells		
colorectal cancer: 169.1	Breast - Breast glandular cells, Colon - Colon enterocytes, Minor Salivary Gland - Minor salivary glandular cells, Prostate - Prostate glandular cells, Skin - Eccrine sweat gland cells, Stomach - Gastric enteroendocrine cells	Region enhanced	Detected in single
Gallbladder cancer: 992.2	Colon - Colon enterocytes, Liver - Cholangiocytes, Lung - Alveolar cells type 2, Minor Salivary Gland - Minor salivary glandular cells, Pancreas - Ductal cells, Skin - Keratinocyte (granular)	Not detected	Not detected
	Breast - Breast glandular cells, Colon - Colon enterocytes, Lung - Alveolar cells type 2, Pancreas - Ductal cells, Skin - Eccrine sweat gland cells	Not detected	Not detected
	Pancreas - Ductal cells, Spleen - Neutrophils_2	Not detected	Not detected
Leukemia: 12.7	Kidney - Macrophages, Prostate - Macrophages	Low region specificity	Detected in all
Myeloma: 33.4	Breast - Endothelial cells, Kidney - Endothelial cells, Pituitary gland - Endothelial cells, Skeletal muscle - Endothelial cells, Spleen - Macrophages		
	Prostate - Smooth muscle cells, Skeletal muscle - Endothelial cells, Spleen - Fibroblasts_1	Low region specificity	Detected in all
colorectal cancer: 11.2;ovarian cancer: 13.8	Breast - Endothelial cells, Heart muscle - Cardiomyocytes, Kidney - Podocytes, Lung - Alveolar cells type 1, Pituitary gland - Undifferentiated cells (Pituitary gland), Skeletal muscle - Skeletal myocytes, Spleen - Fibroblasts_1	Low region specificity	Detected in all
		Low region specificity	Detected in all
	Adrenal gland - Adrenal cortex cells, Prostate - Smooth muscle cells, Stomach - Gastric mucous cells	Low region specificity	Detected in all
	Adrenal gland - Adrenal cortex cells, Colon - Fibroblasts, Kidney - Endothelial cells	Low region specificity	Detected in some
	Liver - Hepatocytes	Region enhanced	Detected in single
	Heart muscle - Cardiomyocytes, Lung - Alveolar cells type 1, Minor Salivary Gland - Minor salivary glandular cells, Skeletal muscle - Skeletal myocytes, Stomach - Gastric mucous cells, Testis - Early spermatids	Low region specificity	Detected in all
Brain cancer: 9.9;lymphoma: 15.1;thyroid cancer: 4.0	Kidney - Macrophages, Pituitary gland - Corticotropes, Testis - Endothelial cells	Low region specificity	Detected in all
		Low region specificity	Detected in all
cervical cancer: 200.6;head and neck cancer: 440.5	Breast - Breast myoepithelial cells, Colon - Colon enterocytes, Prostate - Prostate basal glandular cells, Skin - Keratinocyte (other), Spleen - Neutrophils_1, Stomach - Gastric mucous cells	Region enriched	Detected in single
Brain cancer: 574.1	Adipose subcutaneous - Adipose progenitor cells, Adipose visceral - Adipose progenitor cells, Adrenal gland - Fibroblasts, Breast - Fibroblasts, Heart muscle - Fibroblasts, Kidney - Fibroblasts, Liver - Hepatic stellate cells, S	Low region specificity	Detected in all
	Colon - Macrophages, Lung - Macrophages, Prostate - Prostate glandular cells, Spleen - Macrophages	Low region specificity	Detected in all
	Adrenal gland - Adrenal cortex cells, Testis - Sertoli cells	Low region specificity	Detected in all
Leukemia: 3.6;lymphoma: 1.0	Adipose subcutaneous - Macrophages, Adipose visceral - Macrophages, Adrenal gland - Macrophages, Breast - Macrophages, Colon - Macrophages, Kidney - Podocytes, Lung - Neutrophils_2, Minor Salivary Gland - Macro	Low region specificity	Detected in all
	Adipose visceral - Mesothelial cells, Breast - Breast glandular cells, Breast - Breast glandular cells, Lung - Alveolar cells type 2, Pituitary gland - Somatotropes, Stomach - Gastric mucous cells, Thyroid gland - Thyroid glandu	Low region specificity	Detected in many
thyroid cancer: 5.5	Pituitary gland - Thyrotropes	Region enhanced	Detected in many
Kidney cancer: 236.2;ovarian cancer: 273.3	Adipose subcutaneous - Adipocytes (Subcutaneous), Colon - Enteric glia cells, Prostate - Smooth muscle cells, Spleen - Fibroblasts_2	Low region specificity	Detected in all
		Low region specificity	Detected in all
Leukemia: 13.3	Adipose subcutaneous - Macrophages, Adipose visceral - Macrophages, Adrenal gland - Macrophages, Breast - Macrophages, Colon - Macrophages, Heart muscle - Macrophages, Kidney - Macrophages, Liver - Kupffer cel	Low region specificity	Detected in all
	Minor Salivary Gland - Minor salivary glandular cells	Low region specificity	Detected in all
		Low region specificity	Detected in all
		Low region specificity	Detected in all
	Adipose subcutaneous - Endothelial cells, Adrenal gland - Adrenal cortex cells, Prostate - Prostate basal glandular cells, Skin - Keratinocyte (other), Spleen - Endothelial cells, Stomach - Gastric mucous cells	Low region specificity	Detected in all
	Minor Salivary Gland - Minor salivary glandular cells, Spleen - Endothelial cells	Low region specificity	Detected in all
	Adipose subcutaneous - Endothelial cells, Minor Salivary Gland - Minor salivary glandular cells	Low region specificity	Detected in all
Myeloma: 16.7	Heart muscle - T-cells	Low region specificity	Detected in all
	Adipose visceral - Mesothelial cells, Minor Salivary Gland - Minor salivary glandular cells, Testis - Sertoli cells, Thyroid gland - Thyroid glandular cells	Low region specificity	Detected in all
Leukemia: 6.9	Adipose visceral - Neutrophils, Colon - Neutrophils, Heart muscle - Neutrophils, Lung - Neutrophils_2, Prostate - Urothelial cells, Skeletal muscle - Neutrophils, Spleen - Neutrophils_2, Stomach - Neutrophils, Thyroid gland -	Not detected	Not detected
	Breast - Fibroblasts, Heart muscle - Fibroblasts, Kidney - Fibroblasts, Liver - Hepatic stellate cells, Minor Salivary Gland - Macrophages, Pancreas - Fibroblasts, Prostate - Fibroblasts	Low region specificity	Detected in many
	Breast - Breast glandular cells, Breast - Breast glandular cells, Minor Salivary Gland - Minor salivary glandular cells, Testis - Late spermatids	Low region specificity	Detected in all
Leukemia: 24.3;lymphoma: 25.1;Myeloma: 12.2	Adipose visceral - Macrophages, Adrenal gland - Macrophages, Breast - Macrophages, Skeletal muscle - Macrophages, Spleen - Neutrophils_2, Testis - Macrophages	Low region specificity	Detected in all
	Kidney - Proximal tubular cells	Low region specificity	Detected in all
	Adrenal gland - Adrenal cortex cells, Prostate - Smooth muscle cells, Thyroid gland - Thyroid glandular cells	Low region specificity	Detected in all
Adrenocortical cancer: 88.5		Low region specificity	Detected in all
	Spleen - B-cells (Spleen)	Low region specificity	Detected in all
skin cancer: 361.4	Liver - Hepatocytes, Skin - Melanocytes	Low region specificity	Detected in some
	Adrenal gland - Adrenal cortex cells, Breast - Breast glandular cells, Minor Salivary Gland - Minor salivary glandular cells, Thyroid gland - Thyroid glandular cells	Low region specificity	Detected in all

Neuroblastoma: 44.3	Adrenal gland - Adrenal cortex cells, Lung - Fibroblasts_1, Skeletal muscle - Fibroblasts, Spleen - Fibroblasts_1	Low region specificity	Detected in all
	Skeletal muscle - Endothelial cells	Low region specificity	Detected in all
	Adrenal gland - Mitotic cells (Adrenal gland), Colon - Mitotic cells (Colon), Heart muscle - Mitotic cells (Heart), Minor Salivary Gland - Mitotic cells (Minor salivary gland), Spleen - Mitotic cells (Spleen), Testis - Early spermatids	Low region specificity	Detected in all
		Low region specificity	Detected in all
	Thyroid gland - Thyroid glandular cells	Low region specificity	Detected in all
	Adipose subcutaneous - Adipose progenitor cells, Adipose visceral - Adipose progenitor cells, Pituitary gland - Lactotropes, Testis - Early spermatids	Low region specificity	Detected in all
	Breast - Breast glandular cells, Prostate - Prostate basal glandular cells	Low region specificity	Detected in all
	Spleen - Endothelial cells, Thyroid gland - Fibroblasts	Low region specificity	Detected in all
	Stomach - Mitotic cells (Stomach)	Low region specificity	Detected in all
	Adipose subcutaneous - Endothelial cells, Heart muscle - Endothelial cells, Kidney - Endothelial cells, Stomach - Endothelial cells, Thyroid gland - Endothelial cells	Low region specificity	Detected in all
Leukemia: 46.2;lymphoma: 39.0;Myeloma: 19.4	Adrenal gland - Adrenal cortex cells, Thyroid gland - T-cells	Low region specificity	Detected in many
Leukemia: 36.3;lymphoma: 9.8;Myeloma: 11.1	Adipose subcutaneous - Macrophages, Adipose visceral - Macrophages, Adrenal gland - Macrophages, Breast - Macrophages, Colon - Macrophages, Heart muscle - Macrophages, Liver - Kupffer cells, Minor Salivary Gland	Not detected	Not detected
Leukemia: 72.6;lymphoma: 95.9;Myeloma: 24.7	Adipose subcutaneous - Macrophages, Adrenal gland - Macrophages, Breast - Macrophages, Kidney - Macrophages, Lung - Neutrophils_2, Spleen - Neutrophils_2, Stomach - Macrophages, Thyroid gland - Macrophages	Low region specificity	Detected in many
colorectal cancer: 134.1	Kidney - Proximal tubular cells, Kidney - Proximal tubular cells, Minor Salivary Gland - Plasma cells, Skin - Hair cortex cells, Skin - Inner root sheath cells, Spleen - Plasma cells, Stomach - Gastric enteroendocrine cells	Not detected	Not detected
	Colon - Enteric glia cells	Not detected	Not detected
	Prostate - Prostate glandular cells, Skin - Outer root sheath cells, Stomach - Gastric mucous cells	Not detected	Not detected
Bladder cancer: 21.2;cervical cancer: 33.3;Esophageal cancer: 44.7;(	Adipose visceral - Mesothelial cells, Breast - Breast myoepithelial cells, Skin - Keratinocyte (other)	Region enhanced	Detected in some
	Lung - Alveolar cells type 2, Skeletal muscle - Endothelial cells, Stomach - Gastric mucous cells, Testis - Spermatocytes, Testis - Spermatogonia, Thyroid gland - Thyroid glandular cells	Low region specificity	Detected in many
	Adipose visceral - Mesothelial cells, Adrenal gland - Adrenal cortex cells, Heart muscle - Cardiomyocytes, Pituitary gland - Thyrotropes, Prostate - Prostate basal glandular cells, Skin - Keratinocyte (other), Stomach - Gastric	Region enriched	Detected in single
	Adipose subcutaneous - Smooth muscle cells, Minor Salivary Gland - Minor salivary glandular cells, Prostate - Smooth muscle cells, Skeletal muscle - Skeletal myocytes, Testis - Sertoli cells, Thyroid gland - Thyroid glandula	Low region specificity	Detected in all
	Adipose subcutaneous - Endothelial cells, Adipose visceral - Endothelial cells, Adrenal gland - Endothelial cells, Breast - Endothelial cells, Kidney - Endothelial cells, Lung - Neutrophils_2, Minor Salivary Gland - Endothelial ce	Low region specificity	Detected in many
ovarian cancer: 15.8;pancreatic cancer: 11.4	Adipose visceral - Endothelial cells, Adrenal gland - Endothelial cells, Breast - Endothelial cells, Colon - Endothelial cells, Heart muscle - Endothelial cells, Kidney - Endothelial cells, Liver - Vascular endothelial cells, Lung - En	Low region specificity	Detected in all
	Adipose subcutaneous - Smooth muscle cells, Liver - Hepatic stellate cells, Minor Salivary Gland - Fibroblasts, Prostate - Smooth muscle cells, Spleen - Fibroblasts_1, Testis - Peritubular cells, Thyroid gland - Fibroblasts	Low region specificity	Detected in all
Myeloma: 84.2;skin cancer: 130.7	Adrenal gland - Fibroblasts, Liver - Hepatic stellate cells, Testis - Endothelial cells	Low region specificity	Detected in all
	Adipose subcutaneous - Adipocytes (Subcutaneous), Breast - Adipocytes (Breast), Kidney - Endothelial cells, Liver - Vascular endothelial cells	Low region specificity	Detected in all
	Adrenal gland - Adrenal cortex cells, Testis - Spermatocytes, Testis - Spermatogonia, Thyroid gland - Thyroid glandular cells	Low region specificity	Detected in all
	Colon - Enteric glia cells, Lung - Alveolar cells type 1	Low region specificity	Detected in all
	Prostate - Prostate basal glandular cells	Low region specificity	Detected in all
	Adrenal gland - Fibroblasts, Testis - Early spermatids, Testis - Late spermatids, Thyroid gland - Fibroblasts	Low region specificity	Detected in all
	Adrenal gland - Adrenal cortex cells, Stomach - Fibroblasts, Testis - Late spermatids	Low region specificity	Detected in all
	Adrenal gland - Adrenal cortex cells, Minor Salivary Gland - Endothelial cells, Prostate - Smooth muscle cells	Low region specificity	Detected in all
	Liver - Sinusoid Endothelial cells, Prostate - Fibroblasts, Skin - Hair cortex cells, Skin - Inner root sheath cells, Skin - Outer root sheath cells	Low region specificity	Detected in all
breast cancer: 34.8	Breast - Breast glandular cells, Colon - Colon enterocytes, Lung - Respiratory ciliated cells, Minor Salivary Gland - Minor salivary glandular cells, Prostate - Prostate glandular cells, Skin - Eccrine sweat gland cells, Stomach -	Low region specificity	Detected in many
	Adrenal gland - Adrenal cortex cells, Prostate - Smooth muscle cells	Low region specificity	Detected in all
	Adipose visceral - Endothelial cells, Breast - Endothelial cells, Colon - Endothelial cells, Heart muscle - Endothelial cells, Liver - Vascular endothelial cells, Minor Salivary Gland - Endothelial cells, Prostate - Endothelial cells, S	Low region specificity	Detected in all
		Low region specificity	Detected in all
	Spleen - B-cells (Spleen), Stomach - Fibroblasts	Low region specificity	Detected in all
	Heart muscle - Cardiomyocytes, Skeletal muscle - Skeletal myocytes	Low region specificity	Detected in all
Neuroblastoma: 13.8	Adipose visceral - Mesothelial cells, Adrenal gland - Fibroblasts, Testis - Early spermatids, Testis - Late spermatids, Thyroid gland - Fibroblasts	Low region specificity	Detected in all
	Adrenal gland - Adrenal cortex cells	Low region specificity	Detected in all
	Adrenal gland - Adrenal cortex cells, Prostate - Fibroblasts, Skeletal muscle - Fibroblasts	Low region specificity	Detected in all
	Adrenal gland - Fibroblasts, Colon - Enteric glia cells, Kidney - Fibroblasts, Prostate - Fibroblasts, Skeletal muscle - Fibroblasts, Stomach - Fibroblasts, Thyroid gland - Fibroblasts	Low region specificity	Detected in all
	Adipose subcutaneous - Macrophages, Kidney - Proximal tubular cells, Spleen - Macrophages, Testis - Early spermatids, Testis - Late spermatids	Low region specificity	Detected in all
	Adrenal gland - Adrenal cortex cells, Breast - Breast glandular cells, Liver - Hepatocytes, Lung - Alveolar cells type 1, Minor Salivary Gland - Minor salivary glandular cells, Testis - Spermatocytes, Testis - Spermatogonia	Low region specificity	Detected in all
colorectal cancer: 762.9	Breast - Breast glandular cells, Colon - Colon enterocytes, Liver - Cholangiocytes, Lung - Alveolar cells type 2, Minor Salivary Gland - Minor salivary glandular cells, Skin - Eccrine sweat gland cells, Testis - Spermatogonia, T	Low region specificity	Detected in some
	Adipose subcutaneous - Endothelial cells, Colon - Endothelial cells, Minor Salivary Gland - Minor salivary gland basal cells, Pituitary gland - Endothelial cells, Prostate - Urothelial cells, Spleen - Fibroblasts_1	Low region specificity	Detected in single
skin cancer: 12.8	Adipose visceral - Endothelial cells, Breast - Endothelial cells, Heart muscle - Fibroblasts, Kidney - Fibroblasts, Liver - Hepatic stellate cells, Lung - Fibroblasts_2, Prostate - Fibroblasts, Skeletal muscle - Fibroblasts, Thyroid	Low region specificity	Detected in all
Rhabdoid: 10.7;Sarcoma: 21.2	Adipose subcutaneous - Smooth muscle cells, Adipose visceral - Mesothelial cells, Adrenal gland - Adrenal medulla cells, Minor Salivary Gland - Minor salivary glandular cells, Prostate - Prostate glandular cells, Stomach - C	Low region specificity	Detected in all
lymphoma: 10.5	Breast - Breast myoepithelial cells, Kidney - Fibroblasts, Skin - Keratinocyte (other)	Low region specificity	Detected in all
	Skin - Outer root sheath cells, Spleen - Fibroblasts_1, Stomach - Fibroblasts	Low region specificity	Detected in all
	Skin - Endothelial cells	Low region specificity	Detected in all
breast cancer: 705.7;Esophageal cancer: 445.4;Gastric cancer: 199.3	Adrenal gland - Fibroblasts, Breast - Breast glandular cells, Lung - Alveolar cells type 1, Pituitary gland - Undifferentiated cells (Pituitary gland), Spleen - Fibroblasts_1	Low region specificity	Detected in many
	Adipose subcutaneous - Smooth muscle cells, Adipose visceral - Mesothelial cells, Adrenal gland - Adrenal medulla cells, Breast - Breast glandular cells, Colon - Enteric glia cells, Lung - Alveolar cells type 2, Minor Salivary G	Low region specificity	Detected in all
	Testis - Spermatocytes, Thyroid gland - Thyroid glandular cells	Low region specificity	Detected in all
	Prostate - Smooth muscle cells	Low region specificity	Detected in all
Leukemia: 30.0;Myeloma: 68.8	Adipose subcutaneous - Macrophages, Adrenal gland - Macrophages, Minor Salivary Gland - Macrophages, Skeletal muscle - Macrophages	Low region specificity	Detected in all
	Adipose visceral - Mesothelial cells, Adrenal gland - Adrenal cortex cells, Skin - Keratinocyte (other), Spleen - B-cells (Spleen), Thyroid gland - Thyroid glandular cells	Low region specificity	Detected in all
testis cancer: 214.6	Heart muscle - Endothelial cells, Skeletal muscle - Endothelial cells, Stomach - Gastric mucous cells	Low region specificity	Detected in all
Leukemia: 10.6	Testis - Sertoli cells	Not detected	Not detected
	Adipose subcutaneous - Adipose progenitor cells	Low region specificity	Detected in all
	Adipose subcutaneous - Adipocytes (Subcutaneous), Adipose visceral - Adipocytes (Visceral), Breast - Adipocytes (Breast), Liver - Hepatocytes, Lung - Alveolar cells type 2, Pituitary gland - Corticotropes, Prostate - Prostate	Low region specificity	Detected in all
	Adipose subcutaneous - Smooth muscle cells, Adrenal gland - Fibroblasts, Lung - Smooth muscle cells, Minor Salivary Gland - Minor salivary glandular cells, Spleen - Fibroblasts_1, Stomach - Gastric mucous cells	Low region specificity	Detected in many
	Liver - Hepatic stellate cells, Lung - Respiratory ciliated cells, Minor Salivary Gland - Minor salivary glandular cells, Testis - Early spermatids	Low region specificity	Detected in all
	Adipose visceral - Neutrophils, Colon - Neutrophils, Heart muscle - Neutrophils, Liver - Neutrophils, Lung - Neutrophils_2, Pituitary gland - Neutrophils, Skeletal muscle - Neutrophils, Spleen - Neutrophils_2, Stomach - Neutrophils, Thyroid gland - Neutrophils		
Leukemia: 25.7	Adipose visceral - Macrophages, Adrenal gland - Macrophages, Heart muscle - Macrophages, Kidney - Macrophages, Lung - Macrophages, Pituitary gland - Macrophages, Prostate - Macrophages, Stomach - Macrophages,	Low region specificity	Detected in all
Leukemia: 40.8	Adipose subcutaneous - Macrophages, Adipose visceral - Macrophages, Adrenal gland - Macrophages, Breast - Macrophages, Heart muscle - Macrophages, Kidney - Macrophages, Liver - Neutrophils, Lung - Neutrophils_2, Pancreas - Macrophages, Pituitary gland - Macrophages, Prostate - Macrophages,		
	Adipose subcutaneous - Endothelial cells, Adrenal gland - Fibroblasts, Breast - Endothelial cells, Skeletal muscle - Endothelial cells, Skin - Endothelial cells	Low region specificity	Detected in all
Leukemia: 23.9;lymphoma: 55.8;Myeloma: 45.1	Spleen - B-cells (Spleen)	Not detected	Not detected
lymphoma: 1.3	Pancreas - Beta cells	Not detected	Not detected
colorectal cancer: 4.3	Adipose subcutaneous - Adipocytes (Subcutaneous), Adipose visceral - Adipocytes (Visceral), Adrenal gland - Macrophages, Breast - Adipocytes (Breast), Colon - Colon enterocytes, Pituitary gland - Corticotropes, Testis - La	Not detected	Not detected
	Adipose subcutaneous - Adipose progenitor cells, Adipose visceral - Adipose progenitor cells, Testis - Spermatogonia	Low region specificity	Detected in all
Sarcoma: 6.2;testis cancer: 6.1	Adipose subcutaneous - Endothelial cells, Adipose visceral - Endothelial cells, Adrenal gland - Endothelial cells, Breast - Endothelial cells, Heart muscle - Endothelial cells, Liver - Vascular endothelial cells, Pancreas - Endoth	Low region specificity	Detected in all
	Liver - Hepatic stellate cells, Prostate - Smooth muscle cells, Skeletal muscle - Skeletal myocytes, Skeletal muscle - Skeletal myocytes, Thyroid gland - Fibroblasts	Low region specificity	Detected in all
	Adipose subcutaneous - Endothelial cells, Adipose visceral - Endothelial cells, Breast - Breast glandular cells	Low region specificity	Detected in all
	Skin - Endothelial cells	Low region specificity	Detected in all
	Spleen - Neutrophils_2	Low region specificity	Detected in all
	Adipose subcutaneous - Smooth muscle cells, Adipose visceral - Mesothelial cells, Adrenal gland - Adrenal medulla cells, Lung - Alveolar cells type 2, Prostate - Prostate basal glandular cells, Testis - Sertoli cells	Low region specificity	Detected in all
skin cancer: 15.1;testis cancer: 39.8	Adipose visceral - Endothelial cells, Breast - Endothelial cells, Colon - Endothelial cells, Heart muscle - Endothelial cells, Liver - Vascular endothelial cells, Minor Salivary Gland - Endothelial cells, Pituitary gland - Endothelial c	Low region specificity	Detected in all
	Colon - Endothelial cells, Liver - Vascular endothelial cells, Minor Salivary Gland - Endothelial cells, Pancreas - Endothelial cells, Prostate - Endothelial cells, Skeletal muscle - Endothelial cells, Spleen - Endothelial cells	Low region specificity	Detected in many
	Adipose subcutaneous - Endothelial cells, Adipose visceral - Endothelial cells, Colon - Enteric glia cells, Liver - Vascular endothelial cells, Testis - Sertoli cells	Low region specificity	Detected in all
	Prostate - Smooth muscle cells, Spleen - B-cells (Spleen)	Low region specificity	Detected in all
Gastric cancer: 6.7	Adipose subcutaneous - Macrophages, Adipose visceral - Macrophages, Adrenal gland - Macrophages, Breast - Macrophages, Heart muscle - Macrophages, Kidney - Macrophages, Liver - Kupffer cells, Lung - Macrophages, Minor Salivary Gland - Macrophages, Pancreas - Macrophages, Pituitary gland - M		
	Heart muscle - Endothelial cells, Lung - Endothelial cells, Pituitary gland - Endothelial cells, Testis - Sertoli cells	Low region specificity	Detected in all
	Adrenal gland - Endothelial cells, Breast - Endothelial cells, Lung - Endothelial cells	Low region specificity	Detected in all
	Adrenal gland - Fibroblasts, Kidney - Fibroblasts, Lung - Fibroblasts_1, Skin - Eccrine sweat gland cells, Spleen - Fibroblasts_1, Thyroid gland - Thyroid glandular cells	Low region specificity	Detected in all
	Adipose subcutaneous - Adipocytes (Subcutaneous), Adipose visceral - Adipocytes (Visceral), Breast - Adipocytes (Breast), Prostate - Endothelial cells, Spleen - Endothelial cells	Low region specificity	Detected in all
	Adipose subcutaneous - Endothelial cells, Liver - Vascular endothelial cells, Skeletal muscle - Endothelial cells	Low region specificity	Detected in all
		Low region specificity	Detected in all
	Colon - Enteric glia cells	Low region specificity	Detected in all
breast cancer: 3.7	Adrenal gland - Adrenal medulla cells, Skin - Inner root sheath cells, Stomach - Gastric enteroendocrine cells, Testis - Early spermatids, Testis - Late spermatids	Low region specificity	Detected in all
Rhabdoid: 22.0	Pituitary gland - Corticotropes	Low region specificity	Detected in all
Adrenocortical cancer: 1.4;Myeloma: 2.3		Low region specificity	Detected in all
thyroid cancer: 3.1	Adrenal gland - Adrenal medulla cells, Minor Salivary Gland - Minor salivary glandular cells, Prostate - Prostate glandular cells, Skin - Eccrine sweat gland cells, Testis - Late spermatids	Low region specificity	Detected in many
Neuroblastoma: 1.4	Adrenal gland - Adrenal medulla cells, Colon - Colon enteroendocrine cells, Testis - Early spermatids, Testis - Late spermatids	Region enhanced	Detected in some
Neuroblastoma: 120.6	Adrenal gland - Adrenal medulla cells, Colon - Colon enteroendocrine cells, Minor Salivary Gland - Fibroblasts	Low region specificity	Detected in all
	Heart muscle - Cardiomyocytes, Prostate - Smooth muscle cells, Skeletal muscle - Skeletal myocytes	Low region specificity	Detected in all
Leukemia: 16.2;lymphoma: 5.2	Spleen - B-cells (Spleen)	Not detected	Not detected
	Testis - Early spermatids, Testis - Spermatocytes, Thyroid gland - Thyroid glandular cells	Low region specificity	Detected in all
	Heart muscle - Cardiomyocytes, Lung - Respiratory ciliated cells, Minor Salivary Gland - Minor salivary glandular cells, Spleen - Endothelial cells, Testis - Early spermatids, Testis - Late spermatids	Low region specificity	Detected in all
Neuroblastoma: 14.9	Adipose subcutaneous - Smooth muscle cells, Colon - Enteric glia cells, Minor Salivary Gland - Fibroblasts, Skeletal muscle - Fibroblasts, Stomach - Gastric enteroendocrine cells, Thyroid gland - Fibroblasts	Not detected	Not detected
		Not detected	Not detected
skin cancer: 6.9	Adipose subcutaneous - Adipocytes (Subcutaneous), Adipose visceral - Adipocytes (Visceral), Breast - Adipocytes (Breast), Liver - Hepatocytes	Low region specificity	Detected in all
	Thyroid gland - Thyroid glandular cells	Low region specificity	Detected in all
Rhabdoid: 382.5;testis cancer: 417.8	Adipose visceral - Mesothelial cells, Adrenal gland - Adrenal cortex cells, Heart muscle - Cardiomyocytes	Not detected	Not detected
		Not detected	Not detected
head and neck cancer: 10.9;pancreatic cancer: 11.1	Testis - Late spermatids	Not detected	Not detected
Bladder cancer: 34.8;cervical cancer: 81.6;Esophageal cancer: 26.2;(	Skin - Hair cortex cells, Skin - Inner root sheath cells, Skin - Outer root sheath cells, Testis - Early spermatids, Testis - Late spermatids	Low region specificity	Detected in all
	Adipose subcutaneous - Endothelial cells, Adrenal gland - Endothelial cells, Minor Salivary Gland - Endothelial cells, Prostate - Smooth muscle cells, Skeletal muscle - Endothelial cells, Testis - Spermatocytes, Testis - Sperm	Low region specificity	Detected in all
	Kidney - Fibroblasts, Skeletal muscle - Fibroblasts, Spleen - Fibroblasts_1, Stomach - Fibroblasts, Thyroid gland - Fibroblasts	Region enhanced	Detected in all
	Adrenal gland - Macrophages, Heart muscle - Cardiomyocytes, Thyroid gland - Smooth muscle cells	Low region specificity	Detected in some
	Adrenal gland - Adrenal cortex cells, Liver - Hepatocytes, Prostate - Prostate glandular cells	Low region specificity	Detected in all
Leukemia: 1.9	Heart muscle - Cardiomyocytes, Skin - Eccrine sweat gland cells, Testis - Early spermatids, Testis - Late spermatids	Region enriched	Detected in many
cervical cancer: 17.9;liver cancer: 9.1;Rhabdoid: 8.6	Adipose subcutaneous - Adipocytes (Subcutaneous), Breast - Adipocytes (Breast), Liver - Hepatic stellate cells, Lung - Fibroblasts_2, Pancreas - Fibroblasts, Stomach - Fibroblasts	Group enriched	Detected in some
	Adrenal gland - Adrenal medulla cells, Pancreas - Beta cells	Group enriched	Detected in many
	Pituitary gland - Gonadotropes, Skin - Hair cortex cells, Skin - Inner root sheath cells	Low region specificity	Detected in all
lymphoma: 1.4	Adrenal gland - Adrenal medulla cells, Colon - Colon enteroendocrine cells, Pancreas - Beta cells, Pituitary gland - Thyrotropes, Testis - Early spermatids, Testis - Late spermatids	Low region specificity	Detected in many
	Breast - Endothelial cells, Lung - Endothelial cells, Skeletal muscle - Endothelial cells	Low region specificity	Detected in all
	Adrenal gland - Adrenal cortex cells, Spleen - Neutrophils_2		
	Minor Salivary Gland - Minor salivary glandular cells	Low region specificity	Detected in all
	Skin - Keratinocyte (other)	Low region specificity	Detected in all
Leukemia: 1.3	Testis - Early spermatids, Testis - Late spermatids	Not detected	Not detected
Gallbladder cancer: 382.6	Colon - Enteric glia cells, Minor Salivary Gland - Minor salivary gland basal cells, Pituitary gland - Corticotropes, Spleen - Fibroblasts_1, Testis - Sertoli cells	Low region specificity	Detected in all
Gastric cancer: 115.2;liver cancer: 376.5;Rhabdoid: 143.8;testis canc	Adrenal gland - Fibroblasts, Kidney - Fibroblasts, Lung - Fibroblasts_1, Minor Salivary Gland - Fibroblasts, Pancreas - Fibroblasts, Spleen - Smooth muscle cells, Thyroid gland - Fibroblasts	Low region specificity	Detected in all
skin cancer: 74.2	Adipose visceral - Mesothelial cells, Skin - Melanocytes	Not detected	Not detected
	Skin - Inner root sheath cells, Testis - Early spermatids, Testis - Late spermatids	Not detected	Not detected
	Adipose subcutaneous - Endothelial cells, Adipose visceral - Endothelial cells, Liver - Vascular endothelial cells, Prostate - Prostate glandular cells, Skeletal muscle - Endothelial cells, Testis - Early spermatids	Low region specificity	Detected in many
Leukemia: 11.2;lymphoma: 3.2	Adipose subcutaneous - T-cells, Adipose visceral - T-cells, Adrenal gland - T-cells, Breast - T-cells, Colon - T-cells, Heart muscle - T-cells, Kidney - T-cells, Minor Salivary Gland - T-cells, Pancreas - T-cells, Pituitary gland - T-c	Not detected	Not detected
Leukemia: 14.9;lymphoma: 23.7;thyroid cancer: 22.6	Adipose subcutaneous - Macrophages, Adipose visceral - Macrophages, Skeletal muscle - Macrophages, Spleen - B-cells (Spleen), Thyroid gland - T-cells	Low region specificity	Detected in many

	Adipose subcutaneous - Smooth muscle cells, Adipose visceral - Smooth muscle cells, Heart muscle - Smooth muscle cells	Not detected	Not detected
	Colon - Enteric glia cells	Low region specificity	Detected in all
Neuroblastoma: 23.7		Group enriched	Detected in many
	Adrenal gland - Adrenal cortex cells, Minor Salivary Gland - Minor salivary glandular cells	Low region specificity	Detected in all
	Lung - Alveolar cells type 1, Prostate - Urothelial cells	Not detected	Not detected
	Breast - Breast glandular cells, Breast - Breast glandular cells, Liver - Hepatocytes, Thyroid gland - Thyroid glandular cells	Region enriched	Detected in all
	Colon - Colon enteroendocrine cells, Kidney - Macrophages, Minor Salivary Gland - Mitotic cells (Minor salivary gland), Pituitary gland - Lactotropes, Testis - Spermatogonia	Low region specificity	Detected in all
	Minor Salivary Gland - Minor salivary glandular cells, Skin - Endothelial cells	Low region specificity	Detected in all
Adrenocortical cancer: 69.7	Adrenal gland - Adrenal cortex cells, Kidney - Proximal tubular cells, Liver - Cholangiocytes, Pituitary gland - Lactotropes	Low region specificity	Detected in all
breast cancer: 130.5;Esophageal cancer: 160.3	Adipose visceral - Mesothelial cells, Breast - Breast glandular cells, Pituitary gland - Somatotropes	Region enhanced	Detected in many
	Adrenal gland - Adrenal medulla cells, Breast - Breast glandular cells, Stomach - Fibroblasts, Testis - Early spermatids, Testis - Late spermatids	Low region specificity	Detected in all
Leukemia: 9.9;Neuroblastoma: 19.3	Adipose subcutaneous - Smooth muscle cells, Adipose visceral - Mesothelial cells, Adrenal gland - Adrenal medulla cells, Breast - Breast glandular cells, Lung - Smooth muscle cells, Spleen - Fibroblasts_1, Thyroid gland - T	Low region specificity	Detected in all
colorectal cancer: 1.5	Adrenal gland - Adrenal medulla cells, Liver - Hepatocytes, Pituitary gland - Undifferentiated cells (Pituitary gland), Testis - Early spermatids, Testis - Late spermatids	Low region specificity	Detected in many
	Breast - Endothelial cells, Minor Salivary Gland - Endothelial cells, Spleen - Endothelial cells	Low region specificity	Detected in all
prostate cancer: 12.1;Rhabdoid: 7.9		Region enhanced	Detected in single
breast cancer: 3.9;colorectal cancer: 4.3	Heart muscle - Macrophages, Skin - Keratinocyte (granular), Thyroid gland - Macrophages	Not detected	Not detected
	Adrenal gland - Mitotic cells (Adrenal gland), Colon - Mitotic cells (Colon), Heart muscle - Mitotic cells (Heart), Lung - Mitotic cells (Lung), Minor Salivary Gland - Mitotic cells (Minor salivary gland), Skin - Mitotic cells (Skin), Sp	Low region specificity	Detected in single
Leukemia: 32.6	Kidney - Proximal tubular cells, Liver - Erythroid cells, Lung - Neutrophils_1, Spleen - Erythrocytes		
Leukemia: 222.8;skin cancer: 319.3	Liver - Vascular endothelial cells, Pituitary gland - Endothelial cells	Low region specificity	Detected in all
	Adrenal gland - Adrenal cortex cells, Testis - Early spermatids, Testis - Late spermatids	Low region specificity	Detected in all
Neuroblastoma: 3.4		Low region specificity	Detected in some
Bladder cancer: 21.1;Esophageal cancer: 21.1;head and neck cancer	Adipose subcutaneous - Adipocytes (Subcutaneous), Adipose visceral - Adipocytes (Visceral), Breast - Adipocytes (Breast), Skin - Keratinocyte (other), Testis - Late spermatids		
Leukemia: 25.5;lymphoma: 43.9	Adipose subcutaneous - Macrophages, Adipose visceral - Macrophages, Adrenal gland - Macrophages, Breast - Macrophages, Colon - Macrophages, Heart muscle - Macrophages, Kidney - Macrophages, Lung - Neutrophil	Low region specificity	Detected in many
Leukemia: 188.6;lymphoma: 241.0;Myeloma: 214.2	Adipose subcutaneous - Macrophages, Adipose visceral - Macrophages, Adrenal gland - Macrophages, Breast - Macrophages, Colon - Macrophages, Heart muscle - Macrophages, Lung - Neutrophils_2, Minor Salivary Glan	Low region specificity	Detected in all
cervical cancer: 1.0	Pituitary gland - Gonadotropes	Low region specificity	Detected in many
	Adrenal gland - Adrenal cortex cells, Skeletal muscle - Fibroblasts	Low region specificity	Detected in all
	Thyroid gland - T-cells		
Leukemia: 4.4;lymphoma: 14.2;skin cancer: 3.7	Adipose visceral - T-cells, Thyroid gland - T-cells		
	Adipose subcutaneous - Endothelial cells, Breast - Endothelial cells, Colon - Endothelial cells, Kidney - Endothelial cells, Pituitary gland - Endothelial cells, Skeletal muscle - Endothelial cells, Testis - Endothelial cells		
	Adrenal gland - Adrenal cortex cells, Lung - Alveolar cells type 2, Minor Salivary Gland - Minor salivary gland basal cells	Low region specificity	Detected in all
	Adipose subcutaneous - Adipose progenitor cells, Liver - Vascular endothelial cells	Region enriched	Detected in all
	Liver - Kupffer cells, Minor Salivary Gland - Endothelial cells, Spleen - Macrophages, Testis - Spermatogonia	Low region specificity	Detected in all
	Adrenal gland - Adrenal cortex cells, Testis - Spermatogonia	Low region specificity	Detected in all
	Colon - Smooth muscle cells, Prostate - Smooth muscle cells, Skin - Keratinocyte (other), Spleen - Fibroblasts_1, Stomach - Fibroblasts	Low region specificity	Detected in all
	Adrenal gland - Macrophages, Testis - Early spermatids	Low region specificity	Detected in many
	Adrenal gland - Fibroblasts, Breast - Fibroblasts, Kidney - Podocytes, Lung - Fibroblasts_2, Prostate - Fibroblasts, Skeletal muscle - Fibroblasts, Spleen - Fibroblasts_1, Spleen - Fibroblasts_2, Stomach - Fibroblasts, Thyroid	Low region specificity	Detected in all
	Prostate - Fibroblasts, Skin - Endothelial cells, Testis - Early spermatids	Low region specificity	Detected in all
		Low region specificity	Detected in all
		Low region specificity	Detected in all
	Skeletal muscle - Endothelial cells		
	Colon - Endothelial cells, Minor Salivary Gland - Endothelial cells, Skeletal muscle - Endothelial cells		
		Not detected	Not detected
	Liver - Neutrophils, Spleen - Macrophages	Low region specificity	Detected in all
	Adipose subcutaneous - Smooth muscle cells, Liver - Vascular endothelial cells, Minor Salivary Gland - Minor salivary glandular cells	Low region specificity	Detected in all
Myeloma: 17.3	Adipose subcutaneous - Macrophages, Adipose visceral - Macrophages, Heart muscle - Macrophages, Spleen - T-cells	Low region specificity	Detected in single
lymphoma: 28.7;Myeloma: 13.1	Adipose subcutaneous - Macrophages, Adrenal gland - Macrophages, Breast - Macrophages, Skeletal muscle - Macrophages, Skin - T-cells, Testis - Macrophages	Low region specificity	Detected in all
	Kidney - Endothelial cells, Liver - Neutrophils, Pituitary gland - Endothelial cells, Prostate - Fibroblasts, Testis - Sertoli cells	Low region specificity	Detected in all
	Breast - Breast glandular cells, Breast - Breast glandular cells, Kidney - Proximal tubular cells, Minor Salivary Gland - Minor salivary glandular cells, Stomach - Mitotic cells (Stomach), Testis - Sertoli cells	Low region specificity	Detected in many
Esophageal cancer: 24.0;pancreatic cancer: 13.6		Not detected	Not detected
Leukemia: 207.1;lymphoma: 145.1;Myeloma: 354.9	Adipose subcutaneous - T-cells, Adipose visceral - T-cells, Adrenal gland - T-cells, Colon - T-cells, Liver - T-cells, Minor Salivary Gland - Plasma cells, Pancreas - T-cells, Pituitary gland - T-cells, Prostate - T-cells, Spleen - T-c	Low region specificity	Detected in many
Gallbladder cancer: 139.2	Kidney - Endothelial cells, Liver - Neutrophils, Skeletal muscle - Endothelial cells	Low region specificity	Detected in many
	Stomach - Endothelial cells	Low region specificity	Detected in all
	Adipose subcutaneous - T-cells, Colon - T-cells, Prostate - T-cells, Skin - T-cells, Stomach - T-cells	Not detected	Not detected
	Adrenal gland - Adrenal cortex cells	Low region specificity	Detected in all
		Low region specificity	Detected in all
	Breast - Breast myoepithelial cells, Kidney - Endothelial cells, Lung - Alveolar cells type 1, Spleen - Fibroblasts_1	Low region specificity	Detected in all
Leukemia: 7.5;lymphoma: 7.8	Adipose visceral - Neutrophils, Heart muscle - Neutrophils, Kidney - T-cells, Liver - Neutrophils, Skeletal muscle - Neutrophils, Spleen - Neutrophils_2, Thyroid gland - T-cells	Not detected	Not detected
		Low region specificity	Detected in all
Bone cancer: 64.2;Leukemia: 194.5;lymphoma: 74.0	Adipose subcutaneous - Macrophages, Adipose visceral - Macrophages, Adrenal gland - Macrophages, Breast - Macrophages, Colon - Macrophages, Heart muscle - Macrophages, Kidney - Macrophages, Minor Salivary Gla	Low region specificity	Detected in all
Kidney cancer: 99.0;skin cancer: 125.1	Liver - Vascular endothelial cells, Pituitary gland - Endothelial cells, Prostate - Smooth muscle cells	Low region specificity	Detected in many
	Adipose subcutaneous - Smooth muscle cells, Breast - Breast glandular cells, Minor Salivary Gland - Minor salivary glandular cells, Pituitary gland - Endothelial cells, Spleen - Endothelial cells, Stomach - Gastric mucous cell	Low region specificity	Detected in all
	Adipose subcutaneous - Adipocytes (Subcutaneous), Adipose visceral - Adipocytes (Visceral), Adrenal gland - Adrenal cortex cells, Breast - Adipocytes (Breast)	Low region specificity	Detected in all
colorectal cancer: 2.8;Gastric cancer: 2.2	Colon - Colon enterocytes, Kidney - Proximal tubular cells, Liver - Hepatocytes, Prostate - Prostate glandular cells, Skin - Sebaceous gland cells, Thyroid gland - Thyroid glandular cells	Group enriched	Detected in some
Neuroblastoma: 9.7;Rhabdoid: 8.5	Adipose subcutaneous - Endothelial cells, Adrenal gland - Adrenal cortex cells, Colon - Enteric glia cells, Liver - Vascular endothelial cells, Skeletal muscle - Fibroblasts, Testis - Sertoli cells	Low region specificity	Detected in all
Leukemia: 7.5;lymphoma: 18.0	Adipose subcutaneous - T-cells, Breast - T-cells, Colon - T-cells, Kidney - T-cells, Prostate - T-cells, Skin - T-cells, Spleen - Neutrophils_2, Stomach - T-cells, Thyroid gland - T-cells		
	Heart muscle - Cardiomyocytes, Liver - Hepatocytes, Lung - Alveolar cells type 1, Prostate - Prostate basal glandular cells, Skin - Keratinocyte (other), Thyroid gland - Thyroid glandular cells	Low region specificity	Detected in all
	Adipose visceral - Adipocytes (Visceral), Heart muscle - Cardiomyocytes, Prostate - Smooth muscle cells, Spleen - Fibroblasts_2	Low region specificity	Detected in all
		Low region specificity	Detected in all
	Testis - Early spermatids	Low region specificity	Detected in all
Leukemia: 2.3;testis cancer: 7.8	Adrenal gland - Adrenal medulla cells, Breast - Fibroblasts, Colon - Colon enteroendocrine cells, Minor Salivary Gland - Fibroblasts, Pancreas - Beta cells, Pituitary gland - Corticotropes, Skeletal muscle - Fibroblasts	Low region specificity	Detected in many
Neuroblastoma: 6.1;thyroid cancer: 5.0	Adipose subcutaneous - Smooth muscle cells, Breast - Endothelial cells, Colon - Colon enteroendocrine cells, Heart muscle - Smooth muscle cells, Kidney - Fibroblasts, Skeletal muscle - Smooth muscle cells, Testis - Sperm	Low region specificity	Detected in all
Bone cancer: 68.1;cervical cancer: 61.1	Adipose subcutaneous - Macrophages, Adrenal gland - Macrophages, Prostate - Fibroblasts	Not detected	Not detected
	Adrenal gland - Adrenal medulla cells, Minor Salivary Gland - Minor salivary glandular cells	Group enriched	Detected in all
Gallbladder cancer: 4.0;Neuroblastoma: 2.4	Colon - Enteric glia cells, Skin - Outer root sheath cells, Spleen - B-cells (Spleen)	Low region specificity	Detected in many
Leukemia: 5.6;thyroid cancer: 3.4	Colon - Colon enteroendocrine cells		
	Breast - Breast glandular cells (progenitors), Minor Salivary Gland - Minor salivary glandular cells, Skin - Eccrine sweat gland cells, Spleen - B-cells (Spleen), Stomach - Gastric mucous cells	Region enhanced	Detected in single
colorectal cancer: 32.2;Gastric cancer: 20.3	Adipose subcutaneous - Macrophages, Adipose visceral - Macrophages, Heart muscle - Cardiomyocytes, Minor Salivary Gland - Minor salivary glandular cells, Skeletal muscle - Macrophages, Spleen - Macrophages, Stoma	Not detected	Not detected
cervical cancer: 11.4;thyroid cancer: 4.0	Colon - Enteric glia cells, Pituitary gland - Gonadotropes, Skin - Keratinocyte (other), Testis - Spermatogonia	Low region specificity	Detected in many
	Minor Salivary Gland - Minor salivary glandular cells, Skeletal muscle - Skeletal myocytes, Spleen - Fibroblasts_1, Testis - Sertoli cells	Low region specificity	Detected in many
Neuroblastoma: 5.6	Adipose subcutaneous - Smooth muscle cells, Heart muscle - Cardiomyocytes, Stomach - Gastric enteroendocrine cells	Low region specificity	Detected in all
colorectal cancer: 2.5	Pituitary gland - Lactotropes	Low region specificity	Detected in many
Leukemia: 10.4	Colon - Enteric glia cells, Minor Salivary Gland - Minor salivary glandular cells, Testis - Sertoli cells	Not detected	Not detected
	Adipose visceral - Mesothelial cells, Breast - Breast glandular cells, Lung - Alveolar cells type 2, Minor Salivary Gland - Minor salivary glandular cells, Skin - Keratinocyte (granular), Stomach - Gastric mucous cells	Low region specificity	Detected in all
Myeloma: 13.1		Low region specificity	Detected in all
Leukemia: 28.3	Adipose subcutaneous - Mast cells, Lung - Mast cells, Testis - Spermatogonia, Thyroid gland - Thyroid glandular cells	Low region specificity	Detected in all
	Adrenal gland - Adrenal cortex cells	Low region specificity	Detected in all
Bile duct cancer: 63.9;colorectal cancer: 23.3;Gastric cancer: 17.6;he	Adipose visceral - Mesothelial cells, Kidney - Podocytes	Not detected	Not detected
Leukemia: 9.9;lymphoma: 31.2	Adipose subcutaneous - T-cells, Adipose visceral - T-cells, Adrenal gland - T-cells, Breast - T-cells, Colon - T-cells, Heart muscle - T-cells, Kidney - T-cells, Liver - NK-cells (Liver), Minor Salivary Gland - T-cells, Pancreas - T-ce	Not detected	Not detected
Adrenocortical cancer: 288.6	Adipose subcutaneous - Smooth muscle cells, Adipose visceral - Smooth muscle cells, Adrenal gland - Adrenal medulla cells, Colon - Enteric glia cells, Skeletal muscle - Fibroblasts, Testis - Endothelial cells	Low region specificity	Detected in all
Leukemia: 4.3;Myeloma: 2.3	Adipose subcutaneous - Macrophages, Adrenal gland - Macrophages, Colon - Macrophages, Heart muscle - Macrophages, Kidney - Macrophages, Lung - Macrophages, Minor Salivary Gland - Macrophages, Pituitary gland	Low region specificity	Detected in all
	Adrenal gland - Adrenal cortex cells, Thyroid gland - Thyroid glandular cells	Low region specificity	Detected in all
	Thyroid gland - Thyroid glandular cells	Low region specificity	Detected in all
Leukemia: 87.4	Adipose visceral - T-cells, Spleen - T-cells, Stomach - Fibroblasts	Not detected	Not detected
Leukemia: 200.3;lymphoma: 102.9	Adipose subcutaneous - Macrophages, Adrenal gland - Macrophages, Lung - Neutrophils_2	Low region specificity	Detected in all
Myeloma: 40.5	Adipose subcutaneous - Mast cells, Breast - Plasma cells, Colon - Mast cells, Minor Salivary Gland - Plasma cells	Not detected	Not detected
Leukemia: 582.6;lymphoma: 986.4	Adipose subcutaneous - Macrophages, Adipose visceral - Macrophages, Breast - Macrophages, Colon - Macrophages, Heart muscle - Macrophages, Lung - Neutrophils_2, Minor Salivary Gland - Macrophages, Pituitary glar	Low region specificity	Detected in all
	Prostate - Endothelial cells, Stomach - Chief cells	Low region specificity	Detected in many
	Breast - Adipocytes (Breast)	Not detected	Not detected
Bone cancer: 65.3	Skin - Inner root sheath cells, Skin - Outer root sheath cells	Low region specificity	Detected in all
Bone cancer: 22.4		Not detected	Not detected
	Minor Salivary Gland - Minor salivary glandular cells, Pituitary gland - Endothelial cells	Low region specificity	Detected in all
	Testis - Early spermatids, Testis - Late spermatids, Thyroid gland - Thyroid glandular cells	Low region specificity	Detected in all
prostate cancer: 21.4	Heart muscle - Cardiomyocytes, Lung - Respiratory ciliated cells, Prostate - Prostate glandular cells, Testis - Sertoli cells	Low region specificity	Detected in some
Bone cancer: 2.0;testis cancer: 2.5	Kidney - Fibroblasts, Lung - Fibroblasts_2, Prostate - Fibroblasts, Skeletal muscle - Fibroblasts, Stomach - Fibroblasts, Thyroid gland - Fibroblasts	Low region specificity	Detected in many
	Breast - T-cells, Testis - Early spermatids, Testis - Late spermatids	Low region specificity	Detected in all
	Adipose subcutaneous - Smooth muscle cells, Lung - Smooth muscle cells, Prostate - Smooth muscle cells, Skin - Endothelial cells, Thyroid gland - Smooth muscle cells	Low region specificity	Detected in many
Bone cancer: 2.1;Neuroblastoma: 4.2	Adrenal gland - Adrenal medulla cells, Colon - Colon enteroendocrine cells, Pancreas - Beta cells, Testis - Early spermatids	Low region specificity	Detected in many
	Adrenal gland - Fibroblasts, Kidney - Fibroblasts, Liver - Hepatocytes, Lung - Fibroblasts_2, Prostate - Fibroblasts, Stomach - Fibroblasts	Low region specificity	Detected in all
breast cancer: 19.6;prostate cancer: 35.9	Colon - Colon enterocytes, Lung - Respiratory ciliated cells, Minor Salivary Gland - Minor salivary glandular cells, Prostate - Prostate glandular cells, Skin - Eccrine sweat gland cells	Not detected	Not detected
	Adipose visceral - Mesothelial cells, Breast - Breast glandular cells, Lung - Alveolar cells type 2, Prostate - Prostate basal glandular cells, Skin - Keratinocyte (other), Testis - Sertoli cells	Low region specificity	Detected in all
Leukemia: 45.7	Adipose visceral - Macrophages, Adrenal gland - Macrophages, Liver - Kupffer cells, Minor Salivary Gland - Macrophages, Skeletal muscle - Macrophages, Skin - Langerhans cells, Spleen - Macrophages, Stomach - Macrop	Low region specificity	Detected in all
		Not detected	Not detected
	Lung - Neutrophils_2, Minor Salivary Gland - Minor salivary glandular cells	Low region specificity	Detected in all
	Adrenal gland - Adrenal cortex cells, Liver - Vascular endothelial cells, Prostate - Smooth muscle cells	Low region specificity	Detected in all
	Pituitary gland - Lactotropes, Testis - Spermatocytes, Testis - Spermatogonia	Low region specificity	Detected in all
	Adipose subcutaneous - Endothelial cells, Adipose visceral - Endothelial cells, Liver - Hepatocytes, Minor Salivary Gland - Minor salivary glandular cells, Spleen - Endothelial cells, Testis - Early spermatids, Testis - Late sperm	Low region specificity	Detected in all
	Liver - Hepatocytes, Lung - Alveolar cells type 1, Spleen - Endothelial cells, Stomach - Gastric mucous cells, Testis - Early spermatids	Low region specificity	Detected in all
	Testis - Spermatogonia	Low region specificity	Detected in all
	Spleen - Endothelial cells	Low region specificity	Detected in all
	Colon - Colon enteroendocrine cells, Testis - Early spermatids, Testis - Late spermatids, Thyroid gland - Thyroid glandular cells	Low region specificity	Detected in all
Bone cancer: 46.4;Neuroblastoma: 13.7	Adipose subcutaneous - Adipocytes (Subcutaneous), Adipose visceral - Adipocytes (Visceral), Adrenal gland - Adrenal medulla cells, Heart muscle - Cardiomyocytes, Kidney - Proximal tubular cells, Prostate - Smooth muscle	Low region specificity	Detected in all
	Colon - Enteric glia cells, Skeletal muscle - Fibroblasts, Spleen - Fibroblasts_1, Thyroid gland - Fibroblasts	Low region specificity	Detected in all
	Skin - Keratinocyte (other)	Low region specificity	Detected in all
	Adipose visceral - Mesothelial cells, Breast - Breast glandular cells, Colon - Colon enterocytes, Liver - Hepatocytes, Testis - Early spermatids, Thyroid gland - Thyroid glandular cells	Region enriched	Detected in some
	Adipose visceral - Adipocytes (Visceral), Skin - Sebaceous gland cells	Low region specificity	Detected in some
skin cancer: 283.7	Colon - Smooth muscle cells, Liver - Vascular endothelial cells, Pituitary gland - Endothelial cells, Prostate - Smooth muscle cells, Testis - Endothelial cells, Thyroid gland - Smooth muscle cells	Low region specificity	Detected in all
Leukemia: 17.5	Adipose visceral - Neutrophils, Colon - Neutrophils, Heart muscle - Neutrophils, Kidney - Macrophages, Liver - Neutrophils, Pituitary gland - Neutrophils, Prostate - Macrophages, Skeletal muscle - Neutrophils, Spleen - Neut	Region enhanced	Detected in single
	Adrenal gland - Adrenal cortex cells, Skin - Sebaceous gland cells, Testis - Sertoli cells	Low region specificity	Detected in all

	Adipose visceral - Mesothelial cells, Testis - Early spermatids		
	Adipose subcutaneous - Macrophages, Adrenal gland - Adrenal cortex cells, Lung - Macrophages, Prostate - Macrophages, Spleen - Macrophages	Low region specificity	Detected in all
	Kidney - Fibroblasts, Pituitary gland - Undifferentiated cells (Pituitary gland), Skeletal muscle - Fibroblasts, Stomach - Fibroblasts	Low region specificity	Detected in all
Leukemia: 1.0	Adipose subcutaneous - Macrophages, Adipose visceral - Neutrophils, Adrenal gland - Macrophages, Breast - Macrophages, Heart muscle - Neutrophils, Liver - Neutrophils, Skin - Macrophages, Spleen - Neutrophils_2, Thy	Not detected	Not detected
Sarcoma: 10.0;skin cancer: 6.9	Adrenal gland - Adrenal cortex cells, Breast - Breast myoepithelial cells, Colon - Enteric glia cells, Liver - Hepatocytes, Minor Salivary Gland - Smooth muscle cells, Pituitary gland - Undifferentiated cells (Pituitary gland), Skin	Low region specificity	Detected in all
	Adipose visceral - Macrophages, Heart muscle - Macrophages, Minor Salivary Gland - Macrophages, Spleen - Macrophages	Low region specificity	Detected in all
	Adipose visceral - Mesothelial cells, Colon - Enteric glia cells, Liver - Hepatocytes, Testis - Early spermatids, Testis - Late spermatids	Low region specificity	Detected in all
	Adrenal gland - Adrenal medulla cells, Colon - Enteric glia cells, Lung - Alveolar cells type 2, Minor Salivary Gland - Minor salivary gland basal cells, Skin - Keratinocyte (other), Testis - Sertoli cells	Low region specificity	Detected in all
	Colon - Smooth muscle cells, Prostate - Smooth muscle cells, Skin - Keratinocyte (granular), Stomach - Parietal cells, Testis - Early spermatids	Low region specificity	Detected in all
breast cancer: 293.7	Breast - Breast glandular cells, Prostate - Prostate glandular cells, Testis - Early spermatids, Testis - Late spermatids	Low region specificity	Detected in all
colorectal cancer: 104.4	Adipose visceral - Mesothelial cells, Breast - Breast glandular cells, Colon - Colon enterocytes, Kidney - Proximal tubular cells, Minor Salivary Gland - Minor salivary glandular cells, Prostate - Prostate glandular cells, Stomac	Not detected	Not detected
	Breast - Endothelial cells, Pituitary gland - Endothelial cells, Skeletal muscle - Endothelial cells, Testis - Endothelial cells	Not detected	Not detected
	Adipose visceral - Adipocytes (Visceral), Kidney - Podocytes, Liver - Hepatocytes, Prostate - Prostate glandular cells	Region enhanced	Detected in many
Leukemia: 2.6	Adipose visceral - Neutrophils, Heart muscle - Neutrophils, Liver - Neutrophils, Lung - Neutrophils_2, Minor Salivary Gland - Minor salivary glandular cells, Skeletal muscle - Neutrophils, Spleen - Neutrophils_2, Stomach - Ne	Not detected	Not detected
Leukemia: 5.9	Heart muscle - Cardiomyocytes, Testis - Early spermatids	Region enhanced	Detected in single
Leukemia: 109.3	Adipose subcutaneous - Macrophages, Adipose visceral - Macrophages, Breast - Macrophages, Lung - Neutrophils_2, Prostate - Smooth muscle cells, Thyroid gland - Fibroblasts	Low region specificity	Detected in all
	Adipose subcutaneous - Smooth muscle cells, Adrenal gland - Adrenal cortex cells, Lung - Alveolar cells type 2, Skeletal muscle - Skeletal myocytes, Skin - Keratinocyte (other), Testis - Early spermatids, Testis - Late sperma	Low region specificity	Detected in all
	Adipose subcutaneous - Adipose progenitor cells, Adipose visceral - Adipose progenitor cells, Adrenal gland - Fibroblasts	Low region specificity	Detected in all
	Adipose subcutaneous - Adipose progenitor cells, Adipose visceral - Adipose progenitor cells, Adrenal gland - Adrenal cortex cells, Kidney - Fibroblasts, Lung - Macrophages, Skin - Langerhans cells, Thyroid gland - Thyroid	Low region specificity	Detected in all
Brain cancer: 7.4	Breast - Endothelial cells, Colon - Smooth muscle cells, Prostate - Smooth muscle cells, Spleen - Fibroblasts_1, Stomach - Fibroblasts, Thyroid gland - Smooth muscle cells	Region enriched	Detected in many
lymphoma: 397.8	Adrenal gland - T-cells, Lung - B-cells (Lung), Spleen - B-cells (Spleen), Thyroid gland - T-cells	Not detected	Not detected
ovarian cancer: 100.1;pancreatic cancer: 97.1	Adipose visceral - Mesothelial cells, Pancreas - Ductal cells, Prostate - Urothelial cells, Skin - Eccrine sweat gland cells	Low region specificity	Detected in many
	Breast - Endothelial cells, Pituitary gland - Endothelial cells, Skeletal muscle - Endothelial cells	Low region specificity	Detected in all
	Adrenal gland - Adrenal cortex cells	Low region specificity	Detected in all
breast cancer: 211.1;pancreatic cancer: 218.2	Adipose visceral - Mesothelial cells, Breast - Breast glandular cells, Lung - Alveolar cells type 2, Minor Salivary Gland - Minor salivary glandular cells, Skin - Sebaceous gland cells, Testis - Early spermatids, Testis - Late spermatids		
lymphoma: 3.4;Rhabdoid: 1.7	Adipose subcutaneous - Smooth muscle cells, Adipose visceral - Smooth muscle cells, Adrenal gland - Smooth muscle cells, Breast - Breast myoepithelial cells, Colon - Smooth muscle cells, Heart muscle - Smooth muscle	Low region specificity	Detected in all
Brain cancer: 92.9	Adipose subcutaneous - Smooth muscle cells, Adipose visceral - Smooth muscle cells, Breast - Breast myoepithelial cells, Colon - Smooth muscle cells, Heart muscle - Smooth muscle cells, Kidney - Fibroblasts, Minor Saliv	Low region specificity	Detected in all
	Adipose visceral - Endothelial cells, Minor Salivary Gland - Minor salivary glandular cells, Spleen - Endothelial cells, Testis - Sertoli cells, Thyroid gland - Thyroid glandular cells	Low region specificity	Detected in all
	Adipose subcutaneous - Endothelial cells, Breast - Endothelial cells, Colon - Endothelial cells, Liver - Hepatocytes, Skeletal muscle - Endothelial cells, Testis - Spermatogonia	Low region specificity	Detected in all
Adrenocortical cancer: 449.8	Adipose subcutaneous - Adipocytes (Subcutaneous), Adipose visceral - Adipocytes (Visceral), Breast - Adipocytes (Breast), Prostate - Smooth muscle cells, Spleen - Fibroblasts_1	Low region specificity	Detected in all
Leukemia: 88.2;lymphoma: 96.1	Adipose subcutaneous - T-cells, Breast - T-cells, Kidney - T-cells, Prostate - T-cells, Spleen - T-cells, Thyroid gland - T-cells	Not detected	Not detected
	Adipose subcutaneous - Adipose progenitor cells, Adipose visceral - Adipose progenitor cells	Low region specificity	Detected in many
	Adipose subcutaneous - Smooth muscle cells, Colon - Colon enteroendocrine cells, Heart muscle - Cardiomyocytes, Liver - Hepatic stellate cells, Skin - Eccrine sweat gland cells, Spleen - Smooth muscle cells, Stomach - G	Low region specificity	Detected in all
Myeloma: 9.2	Adipose subcutaneous - Smooth muscle cells, Skeletal muscle - Fibroblasts, Thyroid gland - Thyroid glandular cells	Low region specificity	Detected in all
		Low region specificity	Detected in all
Neuroblastoma: 126.4	Adipose subcutaneous - Smooth muscle cells, Kidney - Fibroblasts, Liver - Hepatic stellate cells, Prostate - Smooth muscle cells	Low region specificity	Detected in all
prostate cancer: 45.3;Rhabdoid: 16.5	Skeletal muscle - Fibroblasts, Thyroid gland - Fibroblasts	Low region specificity	Detected in all
Bone cancer: 54.5;Leukemia: 63.4;lymphoma: 53.7	Adipose subcutaneous - Macrophages, Adipose visceral - Macrophages, Adrenal gland - Macrophages, Breast - Macrophages, Heart muscle - Macrophages, Liver - Kupffer cells, Minor Salivary Gland - Macrophages, Pancr	Low region specificity	Detected in all
	Adipose subcutaneous - Smooth muscle cells, Adipose visceral - Smooth muscle cells, Lung - Smooth muscle cells, Prostate - Smooth muscle cells, Skin - Keratinocyte (granular), Spleen - Smooth muscle cells, Testis - Spe	Low region specificity	Detected in all
breast cancer: 27.9	Lung - Alveolar cells type 2, Skin - Keratinocyte (other), Stomach - Gastric mucous cells	Low region specificity	Detected in all
	Adipose visceral - Mesothelial cells, Testis - Spermatogonia	Low region specificity	Detected in all
	Minor Salivary Gland - Minor salivary glandular cells, Prostate - Prostate glandular cells, Thyroid gland - Thyroid glandular cells	Low region specificity	Detected in all
	Adipose visceral - Adipose progenitor cells, Adrenal gland - Adrenal cortex cells, Spleen - Endothelial cells, Testis - Spermatocytes, Thyroid gland - Thyroid glandular cells	Low region specificity	Detected in all
	Testis - Early spermatids, Testis - Late spermatids	Low region specificity	Detected in all
Sarcoma: 69.2;skin cancer: 133.4	Adrenal gland - Fibroblasts, Lung - Fibroblasts_2, Pituitary gland - Fibroblasts, Skeletal muscle - Fibroblasts	Low region specificity	Detected in many
	Kidney - Macrophages	Low region specificity	Detected in many
	Thyroid gland - Thyroid glandular cells	Low region specificity	Detected in all
ovarian cancer: 50.3	Kidney - Fibroblasts, Liver - Hepatic stellate cells, Prostate - Fibroblasts, Skeletal muscle - Fibroblasts, Spleen - Fibroblasts_1	Low region specificity	Detected in many
	Skeletal muscle - Endothelial cells	Low region specificity	Detected in all
Rhabdoid: 5.5	Adrenal gland - Adrenal cortex cells, Colon - Enteric glia cells, Stomach - Fibroblasts, Testis - Peritubular cells, Thyroid gland - Fibroblasts	Low region specificity	Detected in all
	Testis - Early spermatids	Low region specificity	Detected in all
	Adipose subcutaneous - Adipocytes (Subcutaneous), Prostate - Fibroblasts, Testis - Sertoli cells, Thyroid gland - Fibroblasts	Low region specificity	Detected in all
Gastric cancer: 4.5;pancreatic cancer: 8.1	Testis - Sertoli cells	Region enhanced	Detected in single
Esophageal cancer: 4.5;Sarcoma: 4.2	Adipose visceral - Smooth muscle cells, Colon - Colon enteroendocrine cells, Pituitary gland - Corticotropes, Prostate - Prostate basal glandular cells, Testis - Spermatogonia	Low region specificity	Detected in all
		Low region specificity	Detected in all
Gallbladder cancer: 85.0	Breast - Endothelial cells, Prostate - Endothelial cells, Spleen - Macrophages	Low region specificity	Detected in all
Rhabdoid: 14.7	Adipose visceral - Mesothelial cells	Region enriched	Detected in some
	Skeletal muscle - Endothelial cells	Low region specificity	Detected in all
Adrenocortical cancer: 320.3	Adipose subcutaneous - Adipose progenitor cells, Adipose visceral - Adipose progenitor cells, Breast - Fibroblasts, Heart muscle - Fibroblasts, Lung - Fibroblasts_2, Prostate - Fibroblasts, Skeletal muscle - Fibroblasts	Low region specificity	Detected in all
Bone cancer: 10.1	Adipose visceral - Mesothelial cells, Adrenal gland - Adrenal medulla cells, Kidney - Podocytes, Skeletal muscle - Fibroblasts, Stomach - Fibroblasts	Low region specificity	Detected in all
Leukemia: 4.7	Prostate - Prostate glandular cells, Skin - Fibroblasts_2, Spleen - Neutrophils_2, Stomach - Fibroblasts	Low region specificity	Detected in all
colorectal cancer: 8.8;Esophageal cancer: 9.1	Breast - Breast myoepithelial cells, Liver - Hepatic stellate cells, Minor Salivary Gland - Fibroblasts	Low region specificity	Detected in all
colorectal cancer: 11.8;Gastric cancer: 13.5;pancreatic cancer: 19.5	Kidney - Ascending Loop of Henle cells, Lung - Neutrophils_2, Spleen - Neutrophils_2	Low region specificity	Detected in many
		Low region specificity	Detected in all
		Low region specificity	Detected in all
		Low region specificity	Detected in all
colorectal cancer: 123.7;Gastric cancer: 42.1	Colon - Colon enterocytes, Lung - Neutrophils_1, Prostate - Urothelial cells, Spleen - Neutrophils_1	Low region specificity	Detected in many
cervical cancer: 116.7;head and neck cancer: 80.2	Testis - Early spermatids, Testis - Late spermatids, Thyroid gland - T-cells	Not detected	Not detected
	Adipose subcutaneous - Macrophages, Pituitary gland - Somatotropes, Testis - Early spermatids, Testis - Late spermatids	Low region specificity	Detected in all
Leukemia: 2.1;prostate cancer: 3.8	Colon - Colon enteroendocrine cells, Minor Salivary Gland - Adipocytes (Minor salivary gland), Prostate - Prostate glandular cells	Low region specificity	Detected in many
		Not detected	Not detected
	Prostate - Prostate glandular cells, Testis - Early spermatids		
		Not detected	Not detected
	Adrenal gland - Adrenal medulla cells, Kidney - Proximal tubular cells, Kidney - Proximal tubular cells, Lung - Respiratory ciliated cells, Skin - Keratinocyte (other), Thyroid gland - Fibroblasts	Low region specificity	Detected in all
	Stomach - Gastric mucous cells	Low region specificity	Detected in all
colorectal cancer: 2.9;Rhabdoid: 5.3	Adipose subcutaneous - Smooth muscle cells, Minor Salivary Gland - Minor salivary glandular cells, Pancreas - Beta cells, Skin - Eccrine sweat gland cells	Not detected	Not detected
	Adipose subcutaneous - Smooth muscle cells, Pituitary gland - Endothelial cells, Testis - Spermatogonia	Low region specificity	Detected in all
lymphoma: 25.9	Minor Salivary Gland - Minor salivary glandular cells, Skeletal muscle - Endothelial cells	Low region specificity	Detected in all
	Adrenal gland - Adrenal cortex cells, Testis - Spermatocytes, Thyroid gland - Thyroid glandular cells	Low region specificity	Detected in all
	Adipose subcutaneous - Adipose progenitor cells, Skeletal muscle - Skeletal myocytes, Skin - Outer root sheath cells, Stomach - Gastric mucous cells, Thyroid gland - Thyroid glandular cells	Low region specificity	Detected in all
	Prostate - Prostate glandular cells, Thyroid gland - Thyroid glandular cells	Low region specificity	Detected in all
Neuroblastoma: 29.4	Adipose subcutaneous - Adipocytes (Subcutaneous), Breast - Adipocytes (Breast), Liver - Vascular endothelial cells, Prostate - Fibroblasts	Low region specificity	Detected in all
	Breast - Endothelial cells, Kidney - Endothelial cells, Lung - Endothelial cells, Pancreas - Endothelial cells, Skin - Endothelial cells, Stomach - Endothelial cells	Low region specificity	Detected in all
	Adrenal gland - Adrenal cortex cells, Testis - Spermatogonia, Thyroid gland - Thyroid glandular cells	Low region specificity	Detected in all
		Low region specificity	Detected in all
	Adipose visceral - Mesothelial cells, Breast - Breast glandular cells, Breast - Breast glandular cells, Minor Salivary Gland - Minor salivary glandular cells, Spleen - Neutrophils_2, Testis - Spermatogonia	Low region specificity	Detected in all
testis cancer: 31.6	Prostate - Smooth muscle cells, Stomach - Gastric mucous cells	Low region specificity	Detected in all
	Lung - Alveolar cells type 1, Testis - Spermatogonia	Low region specificity	Detected in all
	Adrenal gland - Fibroblasts, Breast - Fibroblasts, Liver - Hepatic stellate cells, Lung - Fibroblasts_1, Thyroid gland - Fibroblasts	Low region specificity	Detected in all
	Testis - Late spermatids	Low region specificity	Detected in all
	Heart muscle - Cardiomyocytes, Minor Salivary Gland - Endothelial cells	Low region specificity	Detected in all
	Adipose visceral - Mesothelial cells, Heart muscle - Cardiomyocytes, Lung - Alveolar cells type 1, Minor Salivary Gland - Minor salivary glandular cells, Prostate - Prostate basal glandular cells	Low region specificity	Detected in all
Gallbladder cancer: 76.0	Kidney - Macrophages, Minor Salivary Gland - Minor salivary glandular cells, Skeletal muscle - Endothelial cells, Skin - Keratinocyte (granular), Testis - Endothelial cells	Low region specificity	Detected in all
	Adipose subcutaneous - Smooth muscle cells, Adipose visceral - Smooth muscle cells, Breast - Breast myoepithelial cells, Heart muscle - Cardiomyocytes, Skeletal muscle - Fibroblasts, Skin - Mitotic cells (Skin), Spleen - Er	Low region specificity	Detected in all
Gallbladder cancer: 5.9	Testis - Early spermatids	Low region specificity	Detected in all
	Lung - Fibroblasts_1	Low region specificity	Detected in all
	Skin - Inner root sheath cells, Testis - Spermatogonia	Low region specificity	Detected in all
	Testis - Late spermatids	Low region specificity	Detected in all
	Prostate - Smooth muscle cells, Testis - Spermatogonia	Low region specificity	Detected in all
prostate cancer: 40.8	Adipose subcutaneous - Endothelial cells, Minor Salivary Gland - Minor salivary gland ductal cells, Prostate - Prostate glandular cells, Skin - Eccrine sweat gland cells, Spleen - Endothelial cells	Low region specificity	Detected in all
	Spleen - Macrophages, Thyroid gland - Thyroid glandular cells	Low region specificity	Detected in many
Rhabdoid: 78.7;thyroid cancer: 38.5	Adrenal gland - Fibroblasts, Breast - Fibroblasts, Heart muscle - Fibroblasts, Kidney - Fibroblasts, Minor Salivary Gland - Macrophages, Pituitary gland - Fibroblasts, Prostate - Fibroblasts, Spleen - Fibroblasts_2	Low region specificity	Detected in all
testis cancer: 109.6;thyroid cancer: 97.5	Kidney - Podocytes	Low region specificity	Detected in all
	Adipose subcutaneous - Adipocytes (Subcutaneous), Colon - Enteric glia cells, Heart muscle - Cardiomyocytes, Lung - Alveolar cells type 1	Low region specificity	Detected in all
Leukemia: 41.9	Adipose subcutaneous - Endothelial cells, Adipose visceral - Endothelial cells, Adrenal gland - Endothelial cells, Breast - Endothelial cells, Colon - Endothelial cells, Heart muscle - Endothelial cells, Kidney - Endothelial cells,	Low region specificity	Detected in all
		Low region specificity	Detected in some
	Breast - Endothelial cells, Prostate - Smooth muscle cells, Spleen - Endothelial cells	Low region specificity	Detected in all
	Stomach - Parietal cells, Testis - Early spermatids, Testis - Spermatocytes	Low region specificity	Detected in all
Leukemia: 18.8;lymphoma: 26.1;Myeloma: 22.5	Adrenal gland - Macrophages, Testis - Early spermatids, Testis - Spermatocytes	Low region specificity	Detected in many
	Testis - Spermatocytes	Low region specificity	Detected in all
	Adipose visceral - Mesothelial cells	Low region specificity	Detected in all
testis cancer: 164.7	Adipose subcutaneous - Adipocytes (Subcutaneous), Adipose visceral - Mesothelial cells, Breast - Breast glandular cells, Heart muscle - Cardiomyocytes, Prostate - Prostate basal glandular cells	Region enriched	Detected in many
	Colon - Colon enterocytes, Lung - Alveolar cells type 2, Pituitary gland - Corticotropes	Region enriched	Detected in single
	Adipose subcutaneous - Endothelial cells, Adipose visceral - Endothelial cells	Low region specificity	Detected in all
Leukemia: 27.8	Adipose subcutaneous - Adipocytes (Subcutaneous), Testis - Early spermatids	Low region specificity	Detected in all
	Adrenal gland - Adrenal cortex cells, Testis - Spermatocytes, Testis - Spermatogonia	Low region specificity	Detected in all
	Minor Salivary Gland - Minor salivary glandular cells, Thyroid gland - Neutrophils	Low region specificity	Detected in some
Kidney cancer: 60.0	Adipose subcutaneous - Smooth muscle cells, Liver - Hepatic stellate cells	Low region specificity	Detected in all
		Low region specificity	Detected in all
	Lung - Alveolar cells type 2, Prostate - Urothelial cells, Skeletal muscle - Endothelial cells, Stomach - Gastric mucous cells	Low region specificity	Detected in all
	Adipose subcutaneous - Adipocytes (Subcutaneous), Adipose visceral - Adipocytes (Visceral), Colon - Colon enterocytes, Stomach - Gastric mucous cells	Not detected	Not detected
Gallbladder cancer: 24.7	Prostate - Urothelial cells	Low region specificity	Detected in many
liver cancer: 4.6	Adipose subcutaneous - Adipocytes (Subcutaneous), Adipose visceral - Adipocytes (Visceral), Breast - Adipocytes (Breast), Heart muscle - Cardiomyocytes, Minor Salivary Gland - Adipocytes (Minor salivary gland), Prostate	Low region specificity	Detected in many
prostate cancer: 117.5	Breast - Endothelial cells, Prostate - Prostate glandular cells, Spleen - Fibroblasts_2, Stomach - Endothelial cells	Low region specificity	Detected in all
	Breast - Breast glandular cells, Liver - Cholangiocytes, Lung - Respiratory ciliated cells, Minor Salivary Gland - Minor salivary glandular cells, Skin - Keratinocyte (other), Testis - Spermatogonia	Low region specificity	Detected in all
	Heart muscle - Endothelial cells, Kidney - Endothelial cells, Minor Salivary Gland - Minor salivary glandular cells, Skeletal muscle - Endothelial cells, Skin - Endothelial cells	Low region specificity	Detected in all

testis cancer: 590.4	Adrenal gland - Endothelial cells, Colon - Endothelial cells, Heart muscle - Endothelial cells, Kidney - Podocytes, Liver - Vascular endothelial cells, Minor Salivary Gland - Minor salivary glandular cells, Prostate - Endothelial ce	Low region specificity	Detected in all
		Low region specificity	Detected in single
	Spleen - Endothelial cells	Low region specificity	Detected in all
	Spleen - B-cells (Spleen)	Low region specificity	Detected in all
Leukemia: 18.2;lymphoma: 46.7;Myeloma: 27.4;Neuroblastoma: 15.9	Breast - Adipocytes (Breast), Kidney - Proximal tubular cells, Kidney - Proximal tubular cells, Prostate - Endothelial cells, Spleen - B-cells (Spleen)	Low region specificity	Detected in all
		Low region specificity	Detected in all
	Testis - Spermatogonia	Low region specificity	Detected in all
skin cancer: 136.9			
	Adipose visceral - Adipose progenitor cells, Adrenal gland - Adrenal cortex cells	Low region specificity	Detected in all
	Lung - Endothelial cells, Testis - Late spermatids	Low region specificity	Detected in all
prostate cancer: 16.0	Adipose visceral - Mesothelial cells, Spleen - Fibroblasts_2	Low region specificity	Detected in all
	Adrenal gland - Adrenal cortex cells, Prostate - Fibroblasts, Testis - Early spermatids, Testis - Spermatocytes	Low region specificity	Detected in all
		Low region specificity	Detected in many
Neuroblastoma: 37.5	Colon - Enteric glia cells, Kidney - Fibroblasts, Minor Salivary Gland - Fibroblasts, Pituitary gland - Lactotropes, Skeletal muscle - Fibroblasts, Stomach - Fibroblasts	Low region specificity	Detected in all
pancreatic cancer: 205.3	Lung - Respiratory ciliated cells, Skin - Keratinocyte (granular)	Not detected	Not detected
	Adipose visceral - Adipocytes (Visceral), Minor Salivary Gland - Minor salivary glandular cells, Prostate - Smooth muscle cells, Skin - Macrophages, Testis - Early spermatids	Low region specificity	Detected in all
	Testis - Early spermatids, Testis - Late spermatids	Low region specificity	Detected in all
Leukemia: 14.9;lymphoma: 11.2	Adipose subcutaneous - Macrophages, Adrenal gland - Macrophages, Heart muscle - Macrophages, Kidney - T-cells, Liver - T-cells, Prostate - T-cells, Skeletal muscle - Macrophages, Thyroid gland - T-cells	Low region specificity	Detected in many
Gallbladder cancer: 22.4	Adipose subcutaneous - Macrophages, Adipose visceral - Macrophages, Adrenal gland - Macrophages, Colon - Macrophages, Heart muscle - Macrophages, Lung - Macrophages, Minor Salivary Gland - Macrophages, Panc	Low region specificity	Detected in many
	Adipose subcutaneous - Adipose progenitor cells, Minor Salivary Gland - Minor salivary glandular cells, Stomach - Fibroblasts, Testis - Peritubular cells	Not detected	Not detected
testis cancer: 4.8	Breast - Adipocytes (Breast), Spleen - Fibroblasts_2	Low region specificity	Detected in all
Leukemia: 122.9;lymphoma: 76.7;Myeloma: 126.4;prostate cancer: 3	Heart muscle - Macrophages, Skin - Keratinocyte (granular)	Low region specificity	Detected in all
	Adipose subcutaneous - Adipose progenitor cells, Heart muscle - Fibroblasts, Skeletal muscle - Fibroblasts, Testis - Late spermatids	Low region specificity	Detected in all
	Adrenal gland - Adrenal cortex cells, Pituitary gland - Gonadotropes, Prostate - Urothelial cells, Spleen - Endothelial cells, Stomach - Gastric mucous cells	Not detected	Not detected
	Adipose visceral - Macrophages, Adrenal gland - Macrophages, Lung - Neutrophils_2, Prostate - Prostate glandular cells, Testis - Spermatogonia, Thyroid gland - Thyroid glandular cells	Low region specificity	Detected in all
	Liver - Hepatocytes, Skeletal muscle - Endothelial cells	Low region specificity	Detected in all
		Low region specificity	Detected in all
Neuroblastoma: 12.2;ovarian cancer: 10.4	Adrenal gland - Adrenal medulla cells, Colon - Colon enteroendocrine cells, Stomach - Gastric mucous cells	Region enriched	Detected in all
	Adipose subcutaneous - Adipocytes (Subcutaneous)	Low region specificity	Detected in all
	Adrenal gland - Adrenal cortex cells, Minor Salivary Gland - Minor salivary glandular cells, Spleen - Fibroblasts_1	Low region specificity	Detected in all
Leukemia: 73.5	Adrenal gland - Adrenal cortex cells, Kidney - Fibroblasts, Lung - Fibroblasts_1, Prostate - Fibroblasts, Skin - Endothelial cells, Thyroid gland - Thyroid glandular cells	Low region specificity	Detected in all
	Colon - Endothelial cells, Pituitary gland - Endothelial cells	Low region specificity	Detected in all
	Prostate - Smooth muscle cells	Low region specificity	Detected in all
	Breast - Breast glandular cells, Colon - Colon enterocytes, Liver - Cholangiocytes, Lung - Alveolar cells type 2, Skin - Keratinocyte (other), Thyroid gland - Thyroid glandular cells	Not detected	Not detected
	Adrenal gland - Adrenal cortex cells, Colon - Macrophages, Skin - Keratinocyte (other), Testis - Spermatocytes	Low region specificity	Detected in all
cervical cancer: 23.7	Adipose visceral - Mesothelial cells, Breast - T-cells		
cervical cancer: 14.0;Gallbladder cancer: 20.6	Pancreas - Ductal cells		
	Adrenal gland - Adrenal cortex cells, Liver - Hepatocytes	Low region specificity	Detected in all
	Lung - Endothelial cells, Testis - Spermatogonia	Low region specificity	Detected in all
	Adrenal gland - Adrenal cortex cells	Low region specificity	Detected in all
Bone cancer: 12.3;breast cancer: 4.4	Adrenal gland - Endothelial cells, Breast - Endothelial cells, Colon - Endothelial cells, Heart muscle - Endothelial cells, Kidney - Endothelial cells, Liver - Sinusoid Endothelial cells, Minor Salivary Gland - Endothelial cells, Pituit	Group enriched	Detected in all
	Adipose subcutaneous - Endothelial cells, Spleen - Endothelial cells, Testis - Spermatogonia, Thyroid gland - Thyroid glandular cells	Low region specificity	Detected in all
	Adrenal gland - Fibroblasts, Breast - Breast glandular cells, Liver - Hepatocytes, Testis - Spermatocytes	Low region specificity	Detected in all
Leukemia: 25.6;lymphoma: 20.9;Myeloma: 29.9	Adipose subcutaneous - T-cells, Adipose visceral - T-cells, Adrenal gland - T-cells, Breast - T-cells, Colon - T-cells, Kidney - T-cells, Liver - T-cells, Minor Salivary Gland - T-cells, Pituitary gland - T-cells, Prostate - T-cells, Stom	Low region specificity	Detected in some
Leukemia: 9.4;lymphoma: 17.1;Myeloma: 6.6	Adipose subcutaneous - T-cells, Adipose visceral - T-cells, Adrenal gland - T-cells, Breast - T-cells, Colon - T-cells, Liver - T-cells, Prostate - T-cells, Thyroid gland - T-cells	Low region specificity	Detected in all
Leukemia: 109.2;lymphoma: 43.8	Adrenal gland - Fibroblasts, Prostate - Smooth muscle cells, Spleen - B-cells (Spleen)	Low region specificity	Detected in all
Leukemia: 7.5	Adipose subcutaneous - Macrophages, Adrenal gland - Macrophages, Breast - Macrophages, Colon - Macrophages, Kidney - Macrophages, Lung - Neutrophils_2, Minor Salivary Gland - Macrophages, Pituitary gland - Macr	Not detected	Not detected
		Low region specificity	Detected in all
		Low region specificity	Detected in many
	Adrenal gland - Adrenal cortex cells, Breast - Endothelial cells, Skin - Endothelial cells, Thyroid gland - Thyroid glandular cells	Low region specificity	Detected in all
	Adrenal gland - Fibroblasts, Breast - Fibroblasts, Colon - Fibroblasts, Lung - Fibroblasts_1, Prostate - Fibroblasts, Thyroid gland - Fibroblasts	Low region specificity	Detected in all
	Breast - Endothelial cells, Kidney - Endothelial cells, Minor Salivary Gland - Endothelial cells	Low region specificity	Detected in all
		Low region specificity	Detected in all
Neuroblastoma: 97.7;thyroid cancer: 31.6	Adrenal gland - Adrenal medulla cells, Colon - Colon enteroendocrine cells, Pituitary gland - Corticotropes, Testis - Spermatogonia	Low region specificity	Detected in all
	Adipose subcutaneous - Macrophages, Adipose visceral - Macrophages, Adrenal gland - Macrophages, Colon - Macrophages	Low region specificity	Detected in all
	Adipose visceral - Adipocytes (Visceral), Minor Salivary Gland - Endothelial cells, Prostate - Fibroblasts	Low region specificity	Detected in all
Leukemia: 58.2	Adipose subcutaneous - Mast cells, Adipose visceral - Mast cells, Colon - Mast cells, Minor Salivary Gland - Mast cells, Pituitary gland - Corticotropes		
	Spleen - Macrophages	Low region specificity	Detected in all
		Low region specificity	Detected in many
Bile duct cancer: 19.3		Low region specificity	Detected in all
		Low region specificity	Detected in all
	Adipose subcutaneous - Adipose progenitor cells, Heart muscle - Fibroblasts, Prostate - Urothelial cells, Thyroid gland - Fibroblasts	Low region specificity	Detected in all
	Adrenal gland - Adrenal cortex cells	Low region specificity	Detected in all
	Spleen - B-cells (Spleen)	Low region specificity	Detected in all
	Adrenal gland - Adrenal cortex cells	Low region specificity	Detected in all
		Low region specificity	Detected in all
	Adipose visceral - Mesothelial cells, Skeletal muscle - Fibroblasts, Skin - Keratinocyte (other), Spleen - Fibroblasts_1	Low region specificity	Detected in all
breast cancer: 8.7	Adipose subcutaneous - Adipocytes (Subcutaneous), Adipose visceral - Adipocytes (Visceral), Skin - Keratinocyte (other)	Low region specificity	Detected in all
	Skin - Hair cortex cells, Skin - Inner root sheath cells	Low region specificity	Detected in many
		Low region specificity	Detected in all
breast cancer: 536.0;head and neck cancer: 1157.4	Colon - Neutrophils, Kidney - Macrophages, Liver - Neutrophils, Lung - Neutrophils_2, Pituitary gland - Neutrophils, Prostate - Macrophages, Spleen - Neutrophils_2, Thyroid gland - Neutrophils	Low region specificity	Detected in many
Gallbladder cancer: 54.0;pancreatic cancer: 51.0	Adipose visceral - Mesothelial cells, Lung - Alveolar cells type 1	Low region specificity	Detected in many
	Adrenal gland - Adrenal medulla cells, Colon - Colon enteroendocrine cells, Heart muscle - Cardiomyocytes, Lung - Alveolar cells type 2	Low region specificity	Detected in all
Neuroblastoma: 18.0	Adipose subcutaneous - Smooth muscle cells, Adrenal gland - Adrenal medulla cells, Pituitary gland - Lactotropes	Low region specificity	Detected in all
Neuroblastoma: 5.5;Sarcoma: 4.2	Breast - Breast myoepithelial cells, Colon - Colon enteroendocrine cells, Heart muscle - Cardiomyocytes, Pituitary gland - Undifferentiated cells (Pituitary gland)	Group enriched	Detected in many
	Testis - Spermatogonia	Low region specificity	Detected in all
	Colon - Enteric glia cells, Skeletal muscle - Fibroblasts		
Bone cancer: 13.8	Breast - Breast glandular cells, Lung - Alveolar cells type 1, Minor Salivary Gland - Minor salivary gland ductal cells, Pancreas - Ductal cells, Prostate - Prostate basal glandular cells, Stomach - Gastric mucous cells	Region enriched	Detected in single
		Low region specificity	Detected in all
Brain cancer: 11.1;testis cancer: 9.4	Adipose subcutaneous - Smooth muscle cells, Adipose visceral - Smooth muscle cells, Lung - Smooth muscle cells, Minor Salivary Gland - Smooth muscle cells, Prostate - Smooth muscle cells, Skeletal muscle - Smooth m	Low region specificity	Detected in many
	Colon - Fibroblasts, Liver - Hepatocytes, Prostate - Smooth muscle cells, Skeletal muscle - Fibroblasts, Spleen - Fibroblasts_1, Thyroid gland - Thyroid glandular cells	Low region specificity	Detected in all
	Testis - Early spermatids	Low region specificity	Detected in all
Leukemia: 47.6	Colon - T-cells, Skin - Keratinocyte (granular), Testis - Spermatogonia	Low region specificity	Detected in all
	Testis - Sertoli cells	Low region specificity	Detected in all
Bone cancer: 6.4;Neuroblastoma: 8.3	Adipose visceral - Adipose progenitor cells, Colon - Enteric glia cells, Stomach - Fibroblasts	Low region specificity	Detected in all
Leukemia: 10.6;lymphoma: 31.2	Adrenal gland - Macrophages, Colon - Macrophages, Heart muscle - Macrophages, Kidney - Macrophages, Pancreas - Macrophages, Pituitary gland - Macrophages, Prostate - Macrophages, Skin - Inner root sheath cells, Stomach - Macrophages, Thyroid gland - Macrophages		
Leukemia: 3.2	Adipose subcutaneous - Macrophages, Adrenal gland - Macrophages, Colon - Macrophages, Kidney - Macrophages, Pancreas - Macrophages, Pituitary gland - Macrophages, Prostate - Macrophages, Skeletal muscle - Macrophages, Stomach - Macrophages, Thyroid gland - Macrophages		
	Minor Salivary Gland - Minor salivary glandular cells, Prostate - Smooth muscle cells, Spleen - Endothelial cells, Testis - Spermatocytes, Testis - Spermatogonia	Low region specificity	Detected in all
	Breast - Breast glandular cells, Spleen - B-cells (Spleen), Testis - Spermatogonia	Low region specificity	Detected in all
	Adipose subcutaneous - Adipose progenitor cells, Kidney - Proximal tubular cells, Minor Salivary Gland - Minor salivary glandular cells, Prostate - Prostate glandular cells, Stomach - Chief cells, Testis - Early spermatids, Test	Low region specificity	Detected in all
Bile duct cancer: 2.9;liver cancer: 6.5	Pituitary gland - Undifferentiated cells (Pituitary gland)	Low region specificity	Detected in all
Brain cancer: 35.3;thyroid cancer: 34.9	Skin - Eccrine sweat gland cells	Low region specificity	Detected in all
	Adipose visceral - Mesothelial cells	Low region specificity	Detected in all
	Adipose subcutaneous - Macrophages, Adipose visceral - Macrophages, Kidney - Proximal tubular cells, Skin - Keratinocyte (other), Spleen - Macrophages, Stomach - Chief cells, Testis - Spermatocytes	Low region specificity	Detected in many
	Kidney - Macrophages, Lung - Neutrophils_2, Spleen - Neutrophils_2	Low region specificity	Detected in many
	Adipose subcutaneous - Adipocytes (Subcutaneous), Adipose visceral - Adipocytes (Visceral), Adrenal gland - Adrenal cortex cells, Breast - Adipocytes (Breast), Heart muscle - Cardiomyocytes, Stomach - Parietal cells, Testi	Low region specificity	Detected in all
	Adrenal gland - Adrenal cortex cells, Minor Salivary Gland - Minor salivary glandular cells, Testis - Early spermatids, Thyroid gland - Thyroid glandular cells	Low region specificity	Detected in all
	Adipose subcutaneous - Adipocytes (Subcutaneous), Adipose visceral - Adipocytes (Visceral), Minor Salivary Gland - Minor salivary gland ductal cells, Prostate - Prostate basal glandular cells, Skin - Eccrine sweat gland cells	Low region specificity	Detected in many
Bladder cancer: 8.1;breast cancer: 8.4	Pituitary gland - Gonadotropes, Skin - Hair cortex cells, Skin - Inner root sheath cells, Skin - Outer root sheath cells, Testis - Sertoli cells	Region enriched	Detected in all
		Low region specificity	Detected in all
Bone cancer: 1.0	Adrenal gland - Adrenal medulla cells, Colon - Colon enteroendocrine cells, Skin - Hair cortex cells, Skin - Inner root sheath cells, Skin - Outer root sheath cells, Testis - Spermatogonia	Low region specificity	Detected in all
		Low region specificity	Detected in all
Kidney cancer: 3.9	Heart muscle - Cardiomyocytes, Kidney - Proximal tubular cells, Skeletal muscle - Skeletal myocytes, Skeletal muscle - Skeletal myocytes	Not detected	Not detected
	Minor Salivary Gland - Minor salivary gland basal cells	Low region specificity	Detected in all
testis cancer: 19.9	Adipose subcutaneous - Smooth muscle cells, Breast - Breast myoepithelial cells, Heart muscle - Cardiomyocytes, Liver - Hepatocytes, Stomach - Parietal cells	Low region specificity	Detected in all
testis cancer: 32.7	Skin - Sebaceous gland cells, Testis - Early spermatids		
Bladder cancer: 4.6;Kidney cancer: 6.4;liver cancer: 4.0	Kidney - Proximal tubular cells, Liver - Hepatocytes	Not detected	Not detected
testis cancer: 1031.5			
Leukemia: 40.5;lymphoma: 53.5;Myeloma: 32.2	Adrenal gland - Macrophages, Kidney - Proximal tubular cells, Kidney - Proximal tubular cells, Pituitary gland - Macrophages, Testis - Early spermatids	Low region specificity	Detected in many
Adrenocortical cancer: 79.1	Testis - Early spermatids	Low region specificity	Detected in all
head and neck cancer: 18.5;Sarcoma: 16.7	Kidney - Proximal tubular cells, Pancreas - Alpha cells, Pancreas - Beta cells	Not detected	Not detected
	Kidney - Proximal tubular cells, Kidney - Proximal tubular cells, Liver - Hepatocytes	Not detected	Not detected
Kidney cancer: 53.2;ovarian cancer: 115.1;pancreatic cancer: 54.6;U	Kidney - Macrophages, Lung - Alveolar cells type 2, Minor Salivary Gland - Minor salivary glandular cells, Testis - Early spermatids, Testis - Late spermatids	Not detected	Not detected
	Adrenal gland - Adrenal cortex cells, Breast - Endothelial cells, Heart muscle - Endothelial cells, Kidney - Endothelial cells, Skeletal muscle - Endothelial cells, Skin - Endothelial cells	Low region specificity	Detected in all
		Low region specificity	Detected in all
	Adrenal gland - Adrenal cortex cells, Breast - Breast glandular cells, Prostate - Prostate glandular cells, Thyroid gland - Thyroid glandular cells	Low region specificity	Detected in all
	Breast - Breast glandular cells, Kidney - Proximal tubular cells, Testis - Sertoli cells	Low region specificity	Detected in all
Leukemia: 75.6;liver cancer: 50.9	Adrenal gland - Adrenal cortex cells, Skin - Hair cortex cells, Skin - Inner root sheath cells, Skin - Outer root sheath cells, Spleen - Macrophages	Low region specificity	Detected in all
	Adrenal gland - Adrenal cortex cells, Testis - Sertoli cells	Low region specificity	Detected in all
	Spleen - Macrophages	Low region specificity	Detected in all
	Stomach - Parietal cells	Low region specificity	Detected in all
Kidney cancer: 21.0;prostate cancer: 13.6	Adipose visceral - Mesothelial cells, Kidney - Proximal tubular cells, Testis - Early spermatids, Thyroid gland - Thyroid glandular cells	Low region specificity	Detected in all
	Prostate - Smooth muscle cells	Low region specificity	Detected in all
Bile duct cancer: 3.3;colorectal cancer: 8.3;Gastric cancer: 2.7	Colon - Colon enterocytes, Heart muscle - Cardiomyocytes, Kidney - Ascending Loop of Henle cells, Liver - Cholangiocytes, Minor Salivary Gland - Minor salivary glandular cells, Testis - Early spermatids, Testis - Late sperma	Region enriched	Detected in single
testis cancer: 3.0	Kidney - Proximal tubular cells, Kidney - Proximal tubular cells, Liver - Hepatocytes	Not detected	Not detected
	Testis - Sertoli cells	Low region specificity	Detected in all
	Colon - Endothelial cells	Low region specificity	Detected in all
Adrenocortical cancer: 1339.5	Testis - Sertoli cells	Low region specificity	Detected in all
	Adipose subcutaneous - Adipocytes (Subcutaneous), Adipose visceral - Adipocytes (Visceral), Breast - Adipocytes (Breast), Spleen - B-cells (Spleen), Thyroid gland - Thyroid glandular cells	Low region specificity	Detected in all

	Adipose subcutaneous - Smooth muscle cells, Colon - Smooth muscle cells, Heart muscle - Cardiomyocytes, Kidney - Intercalated cells, Pituitary gland - Thyrotropes, Prostate - Smooth muscle cells, Spleen - Macrophages,	Low region specificity	Detected in all
	Adipose subcutaneous - Adipose progenitor cells, Minor Salivary Gland - Minor salivary glandular cells, Skeletal muscle - Fibroblasts, Stomach - Gastric mucous cells, Testis - Late spermatids	Low region specificity	Detected in all
	Colon - Colon enterocytes, Minor Salivary Gland - Minor salivary gland ductal cells, Prostate - Prostate glandular cells, Testis - Early spermatids	Low region specificity	Detected in many
	Stomach - Parietal cells	Region enriched	Detected in single
	Liver - Hepatocytes, Prostate - Prostate glandular cells	Low region specificity	Detected in all
	Adrenal gland - Adrenal cortex cells, Colon - Colon enteroendocrine cells, Pituitary gland - Undifferentiated cells (Pituitary gland), Thyroid gland - Thyroid glandular cells	Low region specificity	Detected in all
	Adipose visceral - Mesothelial cells, Lung - Respiratory ciliated cells, Pituitary gland - Undifferentiated cells (Pituitary gland), Testis - Early spermatids		
	Liver - Hepatocytes, Lung - Alveolar cells type 1, Spleen - B-cells (Spleen)	Low region specificity	Detected in some
testis cancer: 2.0	Liver - Hepatocytes		
Bile duct cancer: 53.7	Liver - Hepatocytes, Minor Salivary Gland - Minor salivary gland ductal cells		
		Region enriched	Detected in some
Bone cancer: 19.5	Pituitary gland - Corticotropes, Prostate - Prostate glandular cells, Skeletal muscle - Skeletal myocytes, Skin - Hair cortex cells, Skin - Inner root sheath cells, Skin - Outer root sheath cells	Group enriched	Detected in some
Neuroblastoma: 66.3	Adipose subcutaneous - Adipose progenitor cells, Adrenal gland - Fibroblasts, Breast - Fibroblasts, Colon - Fibroblasts, Kidney - Fibroblasts, Liver - Hepatic stellate cells, Lung - Fibroblasts_1, Minor Salivary Gland - Fibrobla	Low region specificity	Detected in all
Neuroblastoma: 40.6	Breast - Breast glandular cells, Lung - Respiratory ciliated cells, Minor Salivary Gland - Minor salivary glandular cells, Thyroid gland - Fibroblasts	Region enhanced	Detected in many
	Adipose visceral - Endothelial cells, Heart muscle - Endothelial cells, Skeletal muscle - Endothelial cells, Thyroid gland - Endothelial cells	Low region specificity	Detected in all
	Adrenal gland - Adrenal cortex cells, Colon - Macrophages	Low region specificity	Detected in all
		Low region specificity	Detected in all
thyroid cancer: 1.2	Colon - Endothelial cells, Pituitary gland - Corticotropes, Skeletal muscle - Endothelial cells, Testis - Early spermatids, Testis - Late spermatids	Low region specificity	Detected in many
	Adipose subcutaneous - Adipose progenitor cells, Prostate - Smooth muscle cells	Low region specificity	Detected in all
	Adipose subcutaneous - Adipocytes (Subcutaneous), Adipose visceral - Adipocytes (Visceral), Adrenal gland - Smooth muscle cells, Breast - Adipocytes (Breast), Heart muscle - Cardiomyocytes, Lung - Smooth muscle cells,	Low region specificity	Detected in all
	Adipose subcutaneous - Adipocytes (Subcutaneous), Adipose visceral - Adipocytes (Visceral), Adrenal gland - Adrenal cortex cells, Breast - Adipocytes (Breast), Prostate - Smooth muscle cells	Low region specificity	Detected in all
	Adrenal gland - Adrenal cortex cells, Testis - Spermatocytes, Thyroid gland - Thyroid glandular cells	Low region specificity	Detected in all
	Colon - Endothelial cells, Skeletal muscle - Endothelial cells	Low region specificity	Detected in many
	Adipose visceral - Mesothelial cells, Breast - Breast glandular cells, Colon - Colon enterocytes, Liver - Cholangiocytes, Lung - Alveolar cells type 2, Minor Salivary Gland - Minor salivary glandular cells, Stomach - Gastric muc	Low region specificity	Detected in many
Adrenocortical cancer: 83.4	Adrenal gland - Adrenal cortex cells, Testis - Early spermatids, Testis - Late spermatids	Low region specificity	Detected in all
	Spleen - B-cells (Spleen), Stomach - Gastric mucous cells, Testis - Early spermatids	Low region specificity	Detected in all
	Skin - Keratinocyte (other), Spleen - Endothelial cells, Testis - Early spermatids, Thyroid gland - Thyroid glandular cells	Low region specificity	Detected in all
	Spleen - Macrophages, Stomach - Gastric mucous cells, Testis - Sertoli cells	Low region specificity	Detected in all
Gastric cancer: 5.6	Adipose visceral - Mesothelial cells, Heart muscle - Cardiomyocytes, Pancreas - Beta cells, Prostate - Prostate basal glandular cells	Group enriched	Detected in some
		Low region specificity	Detected in all
cervical cancer: 90.3	Breast - Endothelial cells, Colon - Endothelial cells, Skeletal muscle - Endothelial cells, Spleen - Neutrophils_2, Stomach - Endothelial cells	Not detected	Not detected
	Breast - Endothelial cells, Colon - Endothelial cells, Heart muscle - Endothelial cells, Skin - Keratinocyte (granular), Spleen - T-cells, Testis - Sertoli cells	Low region specificity	Detected in all
	Breast - Endothelial cells, Colon - Neutrophils, Heart muscle - Endothelial cells, Lung - Endothelial cells, Pancreas - Endothelial cells, Pituitary gland - Endothelial cells, Skeletal muscle - Endothelial cells	Low region specificity	Detected in all
		Low region specificity	Detected in all
	Lung - Respiratory ciliated cells	Region enriched	Detected in many
	Testis - Early spermatids	Low region specificity	Detected in all
	Adipose subcutaneous - Smooth muscle cells, Heart muscle - Cardiomyocytes, Prostate - Smooth muscle cells	Low region specificity	Detected in all
lymphoma: 134.6	Adipose visceral - Endothelial cells, Colon - Endothelial cells, Prostate - Endothelial cells, Skeletal muscle - Endothelial cells, Stomach - Endothelial cells, Thyroid gland - Endothelial cells	Low region specificity	Detected in all
	Spleen - B-cells (Spleen), Testis - Early spermatids, Testis - Late spermatids	Low region specificity	Detected in all
Kidney cancer: 85.8	Adrenal gland - Adrenal cortex cells, Liver - Hepatic stellate cells, Minor Salivary Gland - Minor salivary glandular cells, Skeletal muscle - Fibroblasts, Stomach - Gastric mucous cells	Low region specificity	Detected in all
	Adrenal gland - Adrenal cortex cells	Low region specificity	Detected in all
Brain cancer: 2.9;ovarian cancer: 1.2	Breast - Endothelial cells, Minor Salivary Gland - Endothelial cells, Prostate - Endothelial cells, Skeletal muscle - Endothelial cells	Low region specificity	Detected in many
	Prostate - Smooth muscle cells	Not detected	Not detected
Gallbladder cancer: 603.0;head and neck cancer: 792.6	Liver - Cholangiocytes, Pancreas - Ductal cells, Skin - Keratinocyte (other)	Not detected	Not detected
Myeloma: 7.0	Breast - Plasma cells, Minor Salivary Gland - Plasma cells, Spleen - Plasma cells	Not detected	Not detected
		Low region specificity	Detected in all
	Minor Salivary Gland - Minor salivary glandular cells	Low region specificity	Detected in all
	Adipose visceral - Endothelial cells, Heart muscle - Endothelial cells, Kidney - Endothelial cells, Minor Salivary Gland - Minor salivary glandular cells, Skeletal muscle - Endothelial cells, Spleen - Macrophages, Stomach - Chie	Low region specificity	Detected in all
	Adipose subcutaneous - Smooth muscle cells, Adipose visceral - Smooth muscle cells	Low region specificity	Detected in single
	Breast - Breast glandular cells, Kidney - Endothelial cells, Lung - Fibroblasts_1		
Myeloma: 12.4	Adipose subcutaneous - Macrophages, Testis - Sertoli cells	Low region specificity	Detected in all
Rhabdoid: 17.5;testis cancer: 16.9	Adipose subcutaneous - Endothelial cells, Adipose visceral - Endothelial cells, Adrenal gland - Endothelial cells, Breast - Endothelial cells, Colon - Endothelial cells, Heart muscle - Endothelial cells, Kidney - Endothelial cells,	Low region specificity	Detected in all
Adrenocortical cancer: 123.2	Thyroid gland - Fibroblasts	Low region specificity	Detected in all
	Heart muscle - Macrophages, Kidney - Endothelial cells, Pancreas - Endothelial cells, Pituitary gland - Endothelial cells, Stomach - Endothelial cells, Testis - Endothelial cells, Thyroid gland - Endothelial cells	Low region specificity	Detected in all
	Adrenal gland - Adrenal medulla cells, Pituitary gland - Corticotropes	Low region specificity	Detected in all
	Adrenal gland - Fibroblasts, Breast - Endothelial cells, Spleen - Endothelial cells	Low region specificity	Detected in all
		Low region specificity	Detected in all
	Adipose visceral - Mesothelial cells, Breast - Breast glandular cells, Colon - Colon enterocytes, Lung - Alveolar cells type 2, Prostate - Urothelial cells, Stomach - Gastric mucous cells, Testis - Early spermatids, Testis - Late s	Low region specificity	Detected in single
	Colon - Smooth muscle cells, Prostate - Smooth muscle cells	Low region specificity	Detected in all
head and neck cancer: 11.0;Leukemia: 10.6	Adipose subcutaneous - Macrophages, Adrenal gland - Macrophages, Kidney - Macrophages, Liver - Neutrophils, Lung - Neutrophils_2, Minor Salivary Gland - Minor salivary glandular cells, Spleen - Neutrophils_2	Low region specificity	Detected in many
Brain cancer: 11.7	Heart muscle - Endothelial cells, Kidney - Endothelial cells, Lung - Neutrophils_2	Low region specificity	Detected in many
colorectal cancer: 3.0;Gastric cancer: 2.0	Testis - Early spermatids, Testis - Late spermatids	Not detected	Not detected
	Lung - Respiratory ciliated cells, Testis - Early spermatids, Testis - Late spermatids	Low region specificity	Detected in many
	Kidney - Podocytes, Stomach - Gastric mucous cells	Region enriched	Detected in all
	Lung - Alveolar cells type 2, Skin - Keratinocyte (other), Stomach - Gastric mucous cells	Low region specificity	Detected in all
	Heart muscle - Cardiomyocytes, Liver - Vascular endothelial cells	Low region specificity	Detected in all
prostate cancer: 267.6	Colon - Colon enterocytes, Kidney - Intercalated cells, Lung - Alveolar cells type 2, Prostate - Prostate glandular cells, Skin - Eccrine sweat gland cells, Stomach - Gastric mucous cells	Not detected	Not detected
Gastric cancer: 9.7	Adipose subcutaneous - Macrophages, Adrenal gland - Macrophages, Kidney - Macrophages, Minor Salivary Gland - Minor salivary glandular cells, Pituitary gland - Corticotropes, Skeletal muscle - Macrophages, Testis - Spe	Low region specificity	Detected in many
skin cancer: 17.5			
	Prostate - Urothelial cells, Spleen - Macrophages, Testis - Early spermatids, Testis - Late spermatids	Low region specificity	Detected in all
lymphoma: 49.3	Adipose subcutaneous - Endothelial cells, Breast - Endothelial cells, Colon - Endothelial cells, Minor Salivary Gland - Endothelial cells	Region enhanced	Detected in many
Leukemia: 21.6	Breast - T-cells	Low region specificity	Detected in many
	Breast - Breast glandular cells, Colon - Enteric glia cells, Stomach - Fibroblasts	Low region specificity	Detected in all
		Low region specificity	Detected in all
Kidney cancer: 4.2	Adipose subcutaneous - Smooth muscle cells, Colon - Enteric glia cells, Spleen - Fibroblasts_2, Thyroid gland - Smooth muscle cells	Low region specificity	Detected in many
	Adipose subcutaneous - Adipose progenitor cells, Adrenal gland - Mitotic cells (Adrenal gland), Kidney - Fibroblasts, Spleen - Mitotic cells (Spleen), Stomach - Mitotic cells (Stomach), Testis - Early spermatids	Low region specificity	Detected in all
Leukemia: 10.3;Myeloma: 2.8	Adipose subcutaneous - T-cells, Adipose visceral - T-cells, Adrenal gland - T-cells, Breast - T-cells, Kidney - T-cells, Liver - T-cells, Minor Salivary Gland - T-cells, Prostate - T-cells, Skin - T-cells, Thyroid gland - T-cells	Not detected	Not detected
Rhabdoid: 16.0	Heart muscle - Cardiomyocytes, Skeletal muscle - Skeletal myocytes, Testis - Early spermatids, Testis - Late spermatids	Region enriched	Detected in some
		Not detected	Not detected
	Prostate - Prostate glandular cells, Skin - Keratinocyte (other)	Low region specificity	Detected in all
skin cancer: 81.4	Breast - Endothelial cells, Prostate - Fibroblasts, Skeletal muscle - Fibroblasts	Low region specificity	Detected in all
	Adipose subcutaneous - Adipocytes (Subcutaneous)	Low region specificity	Detected in all
Leukemia: 2.0	Spleen - Erythrocytes, Testis - Early spermatids, Testis - Late spermatids	Not detected	Not detected
	Spleen - Endothelial cells	Low region specificity	Detected in all
	Liver - Vascular endothelial cells, Spleen - Endothelial cells, Testis - Spermatogonia	Low region specificity	Detected in all
	Adipose visceral - T-cells, Liver - NK-cells (Liver), Lung - NK-cells (Lung), Prostate - T-cells, Spleen - NK-cells (Spleen)	Not detected	Not detected
Gallbladder cancer: 119.2;Myeloma: 175.3	Breast - Plasma cells, Prostate - Prostate glandular cells, Thyroid gland - Thyroid glandular cells	Low region specificity	Detected in all
Leukemia: 111.0	Adipose subcutaneous - Macrophages, Adipose visceral - Macrophages, Adrenal gland - Macrophages, Breast - Macrophages, Colon - Macrophages, Heart muscle - Macrophages, Kidney - Macrophages, Liver - Kupffer cel	Low region specificity	Detected in all
	Adrenal gland - Mitotic cells (Adrenal gland), Colon - Mitotic cells (Colon), Heart muscle - Mitotic cells (Heart), Lung - Mitotic cells (Lung), Minor Salivary Gland - Mitotic cells (Minor salivary gland), Skin - Mitotic cells (Skin), Sp	Group enriched	Detected in many
		Low region specificity	Detected in all
	Heart muscle - Endothelial cells, Kidney - Endothelial cells, Liver - Vascular endothelial cells		
	Testis - Spermatogonia	Low region specificity	Detected in all
	Testis - Early spermatids	Low region specificity	Detected in all
	Adipose subcutaneous - Endothelial cells, Adipose visceral - Endothelial cells, Lung - Alveolar cells type 1, Skeletal muscle - Endothelial cells, Testis - Early spermatids, Testis - Late spermatids	Low region specificity	Detected in all
	Adipose visceral - Endothelial cells, Colon - Smooth muscle cells, Prostate - Smooth muscle cells, Spleen - Neutrophils_2	Low region specificity	Detected in all
Bile duct cancer: 128.5;colorectal cancer: 290.7;Gastric cancer: 138.	Colon - Colon enterocytes, Kidney - Proximal tubular cells, Kidney - Proximal tubular cells, Skin - Keratinocyte (granular), Testis - Late spermatids	Low region specificity	Detected in single
		Low region specificity	Detected in all
	Adipose subcutaneous - Smooth muscle cells, Prostate - Smooth muscle cells, Testis - Late spermatids	Low region specificity	Detected in all
	Adrenal gland - Adrenal cortex cells, Testis - Spermatogonia	Low region specificity	Detected in all
	Testis - Spermatocytes	Low region specificity	Detected in all
	Adipose visceral - Endothelial cells, Heart muscle - Endothelial cells, Kidney - Endothelial cells, Lung - Endothelial cells, Skeletal muscle - Endothelial cells, Spleen - Endothelial cells	Low region specificity	Detected in all
		Low region specificity	Detected in all
	Adrenal gland - Adrenal cortex cells	Low region specificity	Detected in all
	Testis - Early spermatids, Thyroid gland - Thyroid glandular cells	Low region specificity	Detected in all
	Testis - Early spermatids	Low region specificity	Detected in all
Neuroblastoma: 8.6	Adrenal gland - Adrenal medulla cells, Colon - Colon enteroendocrine cells	Low region specificity	Detected in all
	Skin - Keratinocyte (other), Testis - Early spermatids	Low region specificity	Detected in all
	Adipose visceral - Mesothelial cells, Adrenal gland - Adrenal cortex cells, Lung - Alveolar cells type 1, Skeletal muscle - Fibroblasts, Testis - Spermatogonia	Low region specificity	Detected in many
		Low region specificity	Detected in all

RNA mouse brain regional specificity score	RNA mouse brain regional specific nTPM	RNA pig brain regional specificity	RNA pig brain regional distribution	RNA pig brain regional specificity score	RNA pig brain regional specific nTPM	Antibody	Reliability (IH)
		Low region specificity	Detected in all			HPA017931, HPA020289	Enhanced
		Low region specificity	Detected in all			CAB017717, HPA031868	Approved
		Low region specificity	Detected in all			CAB069889, HPA075201	Approved
		Low region specificity	Detected in all			HPA041564	Enhanced
		Low region specificity	Detected in all			CAB001716, HPA002199, HPA074614	Enhanced
						HPA049395, HPA053288	Enhanced
		Low region specificity	Detected in all			HPA046723, HPA058011	Approved
		Low region specificity	Detected in all			HPA002380, HPA076011	Uncertain
		Low region specificity	Detected in many			CAB037136, HPA048483	Enhanced
		Low region specificity	Detected in all			HPA002476	Enhanced
		Not detected	Not detected			HPA038105	
		Low region specificity	Detected in all			CAB037299, HPA054719	Enhanced
		Low region specificity	Detected in all			HPA032003, HPA049718	Approved
		Low region specificity	Detected in all			HPA001399, CAB004307, HPA023884, CAB080432	Enhanced
		Low region specificity	Detected in all			CAB001709, HPA050670	Approved
		Low region specificity	Detected in many			HPA030866, HPA030867, HPA030868	Enhanced
		Low region specificity	Detected in all			HPA074034	Enhanced
		Low region specificity	Detected in all			CAB010223, HPA068616	Approved
		Low region specificity	Detected in all			CAB005070, HPA048344	Approved
		Low region specificity	Detected in all			HPA041328, HPA044225	Approved
		Low region specificity	Detected in all			CAB009794, HPA035873, HPA035874	Enhanced
		Low region specificity	Detected in all			CAB009796, HPA034509, HPA034510	Supported
		Low region specificity	Detected in all			CAB009797, HPA035696	Supported
		Low region specificity	Detected in all			HPA008984, HPA012393	Uncertain
		Low region specificity	Detected in all			HPA069175	
	basal ganglia: 5.1	Low region specificity	Detected in all			HPA042395, HPA076279	Uncertain
		Low region specificity	Detected in some			HPA001478, HPA050029	Enhanced
		Low region specificity	Detected in all			HPA044383, CAB080543	Approved
		Low region specificity	Detected in all			HPA073747	
		Low region specificity	Detected in many			HPA074416	
		Low region specificity	Detected in all			HPA075322	
		Low region specificity	Detected in all			CAB013496, HPA030212, HPA030213, HPA030214, HPA030215, HPA049868	Approved
		Not detected	Not detected			CAB011682, HPA064436, HPA069474	Enhanced
		Region enhanced	Detected in all		spinal cord: 219.0	HPA019010, HPA019070, HPA026643	Uncertain
		Group enriched	Detected in many	7	medulla oblongata: 16.4;midbrain: 15.1;pons: 18.8;spinal cord: 56.8;thalamus: 21.4	HPA000878, HPA002940, HPA004145, HPA020111	Enhanced
		Low region specificity	Detected in all			HPA006344, CAB026379, HPA056230	Enhanced
						HPA020280, CAB070163, HPA073633	Enhanced
						HPA020280, HPA073633	Uncertain
		Low region specificity	Detected in all			HPA041794, HPA046300	Approved
		Low region specificity	Detected in many			HPA010694	Approved
		Low region specificity	Detected in some			HPA010562	Enhanced
		Low region specificity	Detected in all			HPA065214, HPA065265	Approved
		Low region specificity	Detected in all			HPA039458	Enhanced
		Low region specificity	Detected in all			HPA067290, HPA067853	Enhanced
		Low region specificity	Detected in all			HPA008007, CAB015178, HPA035970	Enhanced
		Low region specificity	Detected in all			CAB013249, CAB015179, HPA038455	Enhanced
		Low region specificity	Detected in all			HPA019358, HPA021780	Uncertain
		Low region specificity	Detected in all			HPA003968, HPA065263	Approved
		Low region specificity	Detected in all			HPA039488, HPA043218	Enhanced
		Low region specificity	Detected in all			HPA051886, HPA063748	Uncertain
		Region enhanced	Detected in many		spinal cord: 8.8	HPA032148, HPA057356	Enhanced
		Low region specificity	Detected in all			HPA016624, HPA051569	Approved
4	basal ganglia: 19.6					HPA036276, HPA057499	Enhanced
		Low region specificity	Detected in all			HPA038958	Approved
		Region enriched	Detected in many	5	spinal cord: 19.5	CAB002417, HPA004625	Enhanced
		Low region specificity	Detected in all			HPA011271, HPA011272, CAB013023, CAB035987, CAB058693, CAB080415	Enhanced
		Low region specificity	Detected in many			HPA018535, HPA019569, HPA019650, CAB025135	Enhanced
						HPA013398, HPA013431	Enhanced
		Low region specificity	Detected in all			HPA036849, HPA069870	Uncertain
		Low region specificity	Detected in all			HPA038521, HPA038522	Approved
		Low region specificity	Detected in all			HPA017009, HPA063903	Enhanced
		Low region specificity	Detected in all			HPA013349, HPA013362	Supported
		Low region specificity	Detected in many			HPA041667, HPA042093, HPA079642	Enhanced
4	basal ganglia: 5.7;olfactory bulb: 20.1	Low region specificity	Detected in all			HPA052462, HPA058882	Approved
		Low region specificity	Detected in all			HPA011138	Approved
5	olfactory bulb: 46.6	Region enriched	Detected in many	4	spinal cord: 29.0	CAB001707, HPA019206	Enhanced
		Not detected	Not detected			HPA062621, HPA065947	Enhanced
		Low region specificity	Detected in all			HPA077460	
		Low region specificity	Detected in all			HPA014924	Enhanced
		Low region specificity	Detected in all			CAB005079, HPA014784, CAB058689	Enhanced
		Low region specificity	Detected in all			HPA066326, CAB080050, CAB080051	Approved
		Low region specificity	Detected in all			HPA032126	
		Low region specificity	Detected in all			HPA041703, HPA075891	Approved
		Low region specificity	Detected in all			HPA035777, HPA043231	Uncertain
		Low region specificity	Detected in all			HPA036610, HPA057861	Approved
		Low region specificity	Detected in all			CAB020696, HPA040391, HPA064439	Uncertain
		Low region specificity	Detected in all			HPA019816, HPA079252	Enhanced
		Low region specificity	Detected in all			CAB009502, HPA012924, HPA060784	Enhanced
		Low region specificity	Detected in all			HPA011026, HPA012037, HPA014658	Enhanced
		Low region specificity	Detected in all			HPA044443, HPA063495	
		Low region specificity	Detected in all			HPA042689, HPA049151, HPA071867	Approved
		Low region specificity	Detected in all			HPA017722, HPA049195	Approved
		Not detected	Not detected			HPA020980, HPA029504, HPA044188, HPA045619, HPA045699	Enhanced
		Low region specificity	Detected in all			HPA028927	Uncertain
		Low region specificity	Detected in all			HPA034769, HPA034770	Uncertain
		Low region specificity	Detected in all			HPA075177	
		Low region specificity	Detected in all			HPA039934, HPA042109	Approved
		Low region specificity	Detected in all			CAB016162, HPA055264, HPA063675	Enhanced
		Low region specificity	Detected in all			CAB018615, HPA068383	Approved
		Not detected	Not detected			HPA014899, HPA015998	Enhanced
		Low region specificity	Detected in all			HPA042289	Approved
		Low region specificity	Detected in all			HPA058870	
		Low region specificity	Detected in all			CAB037260, HPA040471	Enhanced
		Low region specificity	Detected in all			HPA050808	Approved
		Not detected	Not detected			HPA039526	Approved
		Low region specificity	Detected in all			CAB020697, HPA048963	Enhanced
		Low region specificity	Detected in all			HPA035804, HPA043204	Uncertain
		Low region specificity	Detected in all			CAB005605, HPA011166, HPA012945	Enhanced
		Low region specificity	Detected in all			HPA001583, HPA064779	Enhanced
		Low region specificity	Detected in all			CAB016118, HPA040431	Enhanced
		Low region specificity	Detected in all			HPA044279	Approved
		Low region specificity	Detected in all			HPA031515, HPA057316	Enhanced
		Low region specificity	Detected in all			HPA039926, HPA040033	Enhanced
		Low region specificity	Detected in all			HPA018673, HPA018674	Enhanced
		Low region specificity	Detected in all			HPA046680, HPA061103	Approved
		Low region specificity	Detected in all			CAB032501, HPA037422, HPA037423, HPA075217	Uncertain
		Low region specificity	Detected in all			CAB002572, HPA006361	Enhanced
		Low region specificity	Detected in all			CAB016358	Approved
		Low region specificity	Detected in all			HPA023310, HPA027421	Enhanced
		Low region specificity	Detected in many			HPA019484, HPA021257, HPA023874, HPA029503	Enhanced
		Low region specificity	Detected in all			HPA045218, HPA050333	Enhanced
		Low region specificity	Detected in many			HPA003551, HPA050841	Approved
		Group enriched	Detected in some	5	amygdala: 3.2;cerebral cortex: 4.0;hippocampal formation: 4.5	HPA040748, HPA042038	Enhanced
		Low region specificity	Detected in all			HPA038666, HPA038667	Enhanced
		Low region specificity	Detected in all			CAB009333, CAB016291, HPA038309, HPA038310	Enhanced
		Low region specificity	Detected in all			HPA017385, HPA049014	Approved
		Low region specificity	Detected in all			HPA001048, CAB032495	Enhanced
		Low region specificity	Detected in all			CAB025806	Uncertain
		Low region specificity	Detected in all			HPA001328, CAB004552, HPA071048, CAB080447, CAB080451, CAB080452,	Supported
		Low region specificity	Detected in many			HPA001198, HPA002028, CAB016689, CAB080300	Enhanced
		Low region specificity	Detected in all			HPA014788, HPA018176	Enhanced
		Low region specificity	Detected in all			HPA038387, HPA038388	Uncertain
		Not detected	Not detected			HPA011888, HPA011889	Enhanced
		Low region specificity	Detected in all			HPA026483	Supported
		Low region specificity	Detected in all			HPA011044, HPA075369	Approved
		Not detected	Not detected			HPA077621	

	cerebellum: 12.3	Low region specificity	Detected in all			CAB005100, CAB017107, HPA055207	Enhanced
		Low region specificity	Detected in all			HPA039796, CAB079031	Enhanced
		Low region specificity	Detected in all			CAB037266	Enhanced
		Low region specificity	Detected in all			HPA008070, HPA046515	Uncertain
		Low region specificity	Detected in all			CAB000006, HPA008066, HPA017330	Enhanced
		Low region specificity	Detected in all			HPA029038, HPA029039	Enhanced
		Low region specificity	Detected in all			HPA071906, HPA075184	Enhanced
		Low region specificity	Detected in all			CAB009491, HPA036881, HPA036882, HPA075906	Enhanced
		Low region specificity	Detected in all			HPA049838, HPA074213	Supported
		Low region specificity	Detected in all			HPA039325, HPA062122	Enhanced
		Low region specificity	Detected in all			HPA020973, HPA020987, HPA021021	Enhanced
		Low region specificity	Detected in all			HPA034598	Approved
		Low region specificity	Detected in all			HPA041708, HPA046765	Supported
		Low region specificity	Detected in all			HPA058346	Uncertain
		Low region specificity	Detected in all			HPA038101, HPA038102	Enhanced
		Region enriched	Detected in all	5	basal ganglia: 42.5	HPA002726, HPA005832, HPA064745	Approved
		Low region specificity	Detected in all			HPA054183	Approved
	hypothalamus: 7.2	Region enhanced	Detected in many		midbrain: 16.5	HPA073900	
						HPA079548	
		Low region specificity	Detected in all			HPA046825	Approved
		Low region specificity	Detected in many			CAB003793, HPA063255	Approved
		Low region specificity	Detected in many			HPA031613	Approved
		Low region specificity	Detected in many			HPA043238	Approved
		Low region specificity	Detected in all			HPA008992, HPA015723	Enhanced
		Low region specificity	Detected in all			CAB002432, HPA046404, HPA051974	Enhanced
		Low region specificity	Detected in many			HPA003740	Enhanced
		Low region specificity	Detected in many			CAB002430, HPA003883	Enhanced
						HPA068512	
		Low region specificity	Detected in all			HPA015715, HPA015865	Supported
		Low region specificity	Detected in all			HPA003528, CAB076385, CAB080537	Enhanced
	olfactory bulb: 1.0	Low region specificity	Detected in all			HPA041233, HPA070003	Enhanced
		Low region specificity	Detected in all			HPA003267, HPA003326, CAB004352	Enhanced
						CAB011442, HPA035832, CAB079996, CAB080305	Enhanced
		Low region specificity	Detected in all			CAB002493, HPA022132, CAB025255, HPA052381	Enhanced
		Low region specificity	Detected in all			CAB000010, HPA040957, HPA043955, CAB072863, CAB072864	Enhanced
		Low region specificity	Detected in all			CAB017520, HPA038494	Enhanced
14	basal ganglia: 46.2	Low region specificity	Detected in many			CAB000011, HPA004252, HPA004472, CAB068180	Enhanced
		Low region specificity	Detected in all			CAB002495, HPA031567, HPA031568, CAB072868, CAB080468	Enhanced
		Not detected	Not detected			HPA045827, HPA072480	Enhanced
		Low region specificity	Detected in all			CAB000112, CAB000316, HPA005785	Enhanced
		Low region specificity	Detected in all			CAB016055, HPA044659	Approved
		Not detected	Not detected			HPA009178, CAB009751, HPA044107	Enhanced
		Low region specificity	Detected in all			HPA063316, HPA068020, HPA070502	Enhanced
		Low region specificity	Detected in all			CAB004319, HPA026802	Approved
		Low region specificity	Detected in some			CAB000012, HPA037756, CAB075722	Enhanced
						CAB004353, HPA029164	Enhanced
		Low region specificity	Detected in all			CAB002490	Supported
		Low region specificity	Detected in all			HPA039404, HPA040787	Supported
		Low region specificity	Detected in all			HPA006379, HPA076536	Approved
		Low region specificity	Detected in all			HPA034986, HPA061792	Approved
		Low region specificity	Detected in all			HPA027634	Approved
		Low region specificity	Detected in all			CAB000087, HPA004812, CAB028364, CAB072855, CAB072856, CAB072857	Enhanced
		Low region specificity	Detected in all			HPA001380, CAB025863	Enhanced
7	cerebellum: 15.7	Region enriched	Detected in some	19	cerebellum: 24.8	HPA009139, HPA070961	Enhanced
		Not detected	Not detected			HPA023614, HPA023616, CAB025143, HPA026556	Enhanced
		Low region specificity	Detected in all			CAB000141, CAB018580, HPA058574, CAB078686, CAB080169	Enhanced
						HPA015722, HPA047578	Uncertain
	olfactory bulb: 1.7	Low region specificity	Detected in all			HPA001767, CAB002487, HPA002974	Enhanced
		Low region specificity	Detected in all			HPA015613	
		Low region specificity	Detected in all			CAB028366, HPA075875	Approved
		Low region specificity	Detected in all			HPA007047, HPA007403, HPA007456, CAB025274	Enhanced
		Low region specificity	Detected in all			HPA000974, HPA001366, CAB016535	Approved
						CAB008909, HPA064535	Enhanced
						CAB000021, CAB000022, HPA011041, HPA019758	Enhanced
						HPA069621, HPA073910	Enhanced
		Low region specificity	Detected in all			HPA052976, HPA060521	
		Low region specificity	Detected in all			HPA017080, HPA044889	Approved
		Low region specificity	Detected in all			HPA019044, CAB022157, CAB025216	Uncertain
						CAB017193, HPA027586, HPA027587, HPA027657	Enhanced
		Low region specificity	Detected in all			CAB013500, HPA056911, HPA069214	Enhanced
		Low region specificity	Detected in all			HPA026782, HPA026784, HPA076603	Uncertain
		Low region specificity	Detected in many			HPA024106, HPA048036	Approved
		Region enhanced	Detected in many		basal ganglia: 22.1	CAB022444, HPA072083	Enhanced
		Low region specificity	Detected in all			HPA071554	Enhanced
		Region enhanced	Detected in many		thalamus: 4.9	HPA048348	
		Low region specificity	Detected in all			HPA029430	Enhanced
		Low region specificity	Detected in many			HPA054381	Approved
5	olfactory bulb: 5.3;pons and medulla: 1.4;thalamus: 4.2					HPA059484	
		Low region specificity	Detected in all			HPA065404	
		Low region specificity	Detected in all			CAB012991, HPA042413, HPA048825, HPA074310	Approved
		Low region specificity	Detected in all			HPA047192	Enhanced
		Low region specificity	Detected in all			HPA000401, HPA003213	Uncertain
5	white matter: 2085.3	Low region specificity	Detected in all			CAB009789, HPA013166, HPA013657	Enhanced
	olfactory bulb: 5.4	Region enriched	Detected in all	5	spinal cord: 89.7	HPA014412	
4	basal ganglia: 4.5;hippocampal formation: 9.	Low region specificity	Detected in many			CAB002609, HPA051548, HPA074528	Enhanced
		Low region specificity	Detected in all			HPA073105	
	basal ganglia: 1.5					HPA014361	Enhanced
		Not detected	Not detected			CAB002610	Enhanced
		Low region specificity	Detected in all			CAB013063, HPA014703, HPA073662	Enhanced
		Region enhanced	Detected in single		basal ganglia: 1.3	HPA076613	
		Low region specificity	Detected in all			HPA054137	
		Low region specificity	Detected in all			HPA047381, HPA060101	Uncertain
		Low region specificity	Detected in all			HPA008019, HPA060804	Enhanced
		Low region specificity	Detected in all			HPA074371	
		Low region specificity	Detected in all			CAB002665, HPA050918, HPA054667	Enhanced
		Low region specificity	Detected in all			CAB003692, HPA036458	Approved
		Low region specificity	Detected in all			HPA010525	Uncertain
	basal ganglia: 1.1	Not detected	Not detected			HPA076168	
		Low region specificity	Detected in all			HPA017732	Enhanced
		Low region specificity	Detected in all			HPA042256, HPA069945	Enhanced
		Low region specificity	Detected in all			HPA040353, HPA054470	Approved
4	cerebellum: 1.0	Low region specificity	Detected in all			HPA043673, HPA052963	Enhanced
		Region enriched	Detected in all		spinal cord: 64.8	HPA007029, HPA030920, HPA038799	Enhanced
		Low region specificity	Detected in all			CAB017616, HPA040113, HPA070456	Enhanced
		Low region specificity	Detected in all			HPA040938, HPA041970	Uncertain
		Not detected	Not detected			CAB002491, CAB016271, HPA042455, HPA043579, HPA049348	Enhanced
		Not detected	Not detected			HPA013835	Enhanced
	basal ganglia: 8.1	Low region specificity	Detected in many			HPA073345	
		Low region specificity	Detected in all			CAB002053, CAB040560, HPA057100	Enhanced
		Low region specificity	Detected in all			HPA076624	
		Low region specificity	Detected in all			CAB008970, HPA012323	Approved
		Low region specificity	Detected in all			CAB015410, HPA070888, HPA072593	Approved
		Low region specificity	Detected in all			CAB021089, HPA046119, HPA063535	Supported
		Low region specificity	Detected in all			CAB000108, CAB001950, HPA029159, HPA029160	Supported
		Low region specificity	Detected in all			CAB003837, HPA015954, HPA015955	Enhanced
		Low region specificity	Detected in all			CAB011235, HPA057242	Supported
		Low region specificity	Detected in all			CAB026192, HPA040361, CAB080311	Approved
		Low region specificity	Detected in all			CAB032478, HPA046587	Approved
		Low region specificity	Detected in all			HPA003342, CAB005103, HPA030411, HPA030412	Enhanced
		Low region specificity	Detected in many			CAB016268, HPA031999, HPA032017, CAB080545	Enhanced
		Low region specificity	Detected in all			HPA014757	Approved
		Low region specificity	Detected in all			CAB014872, HPA060662	Uncertain
		Low region specificity	Detected in all			HPA004065, HPA071573	Approved
		Low region specificity	Detected in all			CAB009314, HPA028888	Enhanced
		Low region specificity	Detected in all			CAB001960, CAB016353, HPA070563	Enhanced
		Low region specificity	Detected in all			HPA069377	
		Low region specificity	Detected in all			HPA020265, HPA027735	Enhanced
		Low region specificity	Detected in all			HPA010742, HPA010743, HPA010800, CAB017634	Enhanced
		Low region specificity	Detected in all			CAB010162, CAB025656, HPA074209	Approved

		Low region specificity	Detected in all			HPA070112	
		Low region specificity	Detected in all			HPA039533, HPA040355, HPA061538	Uncertain
		Low region specificity	Detected in all			HPA032151	
		Low region specificity	Detected in all			HPA044692, HPA052516	Uncertain
		Low region specificity	Detected in all			CAB016307, HPA069593	Supported
		Low region specificity	Detected in all			CAB001999, CAB002000, HPA010122, HPA071006	Enhanced
		Low region specificity	Detected in all			HPA001746	
		Low region specificity	Detected in all			CAB004995, HPA028383, HPA028385	Approved
		Low region specificity	Detected in all			HPA035215, HPA035216	Supported
		Low region specificity	Detected in all			HPA071516	
		Low region specificity	Detected in all			HPA003218, HPA003219, HPA073349	Enhanced
		Low region specificity	Detected in all			HPA002862, CAB004379, HPA066571, HPA069121	Enhanced
		Low region specificity	Detected in many			HPA077312	Enhanced
		Not detected	Not detected			HPA009426, HPA012783	Enhanced
		Not detected	Not detected			HPA062682	
		Low region specificity	Detected in all			HPA011911, HPA012615	Enhanced
	hippocampal formation: 3.3	Low region specificity	Detected in many			CAB037328, HPA049265, HPA073937	Enhanced
		Low region specificity	Detected in all			HPA004896, CAB025122	Enhanced
23	hippocampal formation: 11.6	Region enhanced	Detected in many		spinal cord: 12.7	CAB037324, HPA045840, HPA054950	Enhanced
		Low region specificity	Detected in all			CAB015196, HPA071177	Enhanced
		Low region specificity	Detected in all			CAB002510, HPA017071, HPA021945	Enhanced
		Low region specificity	Detected in many			HPA063337	
		Low region specificity	Detected in all			HPA013774, CAB018957	Uncertain
		Low region specificity	Detected in all			CAB017842, HPA027546	Approved
		Low region specificity	Detected in all			CAB031489, HPA067188	Uncertain
		Low region specificity	Detected in all			HPA022859, HPA023092, HPA023402	Enhanced
		Low region specificity	Detected in all			HPA038089, HPA071831	Uncertain
		Low region specificity	Detected in all			CAB000035, HPA001200, HPA018530, CAB068186, CAB073534, CAB080313,	Enhanced
		Low region specificity	Detected in all			HPA035468, HPA035469	Enhanced
		Low region specificity	Detected in all			HPA049890, HPA049986, HPA066751, HPA067747	Approved
		Low region specificity	Detected in all			HPA047394, HPA049890, HPA049986, HPA066751	Approved
		Low region specificity	Detected in all			HPA049890, HPA049986, HPA066751	Approved
		Low region specificity	Detected in all			HPA029869, HPA070749	Enhanced
		Low region specificity	Detected in all			HPA028448, HPA028696	Enhanced
		Low region specificity	Detected in all			CAB000096, HPA011862, HPA067440, CAB072873	Enhanced
		Low region specificity	Detected in all			CAB018614, HPA068284, HPA068721, CAB069394, CAB080034	Supported
		Low region specificity	Detected in all			CAB000063, HPA068284, HPA068721, CAB073539, CAB079023	Enhanced
		Low region specificity	Detected in all			HPA000793, HPA068284, HPA068721	Enhanced
		Low region specificity	Detected in all			HPA038355	Approved
		Low region specificity	Detected in all			HPA000514, HPA003027	Uncertain
		Low region specificity	Detected in all			HPA054104, HPA056817	Enhanced
		Low region specificity	Detected in all			HPA005730, HPA006642	Supported
		Low region specificity	Detected in all			HPA028605	Enhanced
		Low region specificity	Detected in all			HPA037563, HPA037564	Uncertain
		Low region specificity	Detected in all			CAB003809, HPA026761, CAB030012, CAB055098, HPA067463	Enhanced
		Low region specificity	Detected in all			CAB010464	
		Low region specificity	Detected in all			CAB010462, HPA069390	Approved
		Low region specificity	Detected in all			HPA034528	
		Low region specificity	Detected in all			HPA067740	Enhanced
		Low region specificity	Detected in many			CAB013647, HPA071200	Approved
		Low region specificity	Detected in all			HPA077654	
		Low region specificity	Detected in all			CAB000043, HPA001060, HPA001338, HPA001383, HPA007983, CAB020416,	Enhanced
		Low region specificity	Detected in all			CAB025331, HPA045396, HPA070524	Approved
		Low region specificity	Detected in all			HPA048606, HPA059863	Approved
		Low region specificity	Detected in all			HPA002132, HPA070823	Approved
		Low region specificity	Detected in all			HPA045033	Uncertain
		Low region specificity	Detected in all			CAB004035, HPA021616, CAB047324, CAB075739	Enhanced
		Low region specificity	Detected in all			CAB004671, HPA043616, HPA061700	Enhanced
		Not detected	Not detected			HPA031401, HPA042340	
		Low region specificity	Detected in all			HPA027444	Uncertain
		Low region specificity	Detected in all			CAB005192, HPA006461, CAB015417, HPA056108	Enhanced
		Low region specificity	Detected in all			HPA025287, HPA072750	Approved
		Low region specificity	Detected in all			HPA003240, HPA061783	Approved
		Not detected	Not detected			HPA014050	Approved
		Low region specificity	Detected in all			HPA026910	
						HPA010718, HPA010776, HPA014730	Supported
		Low region specificity	Detected in all			HPA038545, HPA038546	Uncertain
		Low region specificity	Detected in all			HPA003910	Approved
						HPA048933	
		Low region specificity	Detected in all			HPA042563	
		Low region specificity	Detected in all			HPA000911, HPA076516	Approved
		Low region specificity	Detected in all			HPA014436, HPA014536, HPA019191	Uncertain
		Low region specificity	Detected in all			HPA001040, HPA001391, CAB020817	Enhanced
		Low region specificity	Detected in all			HPA004747, HPA004886, CAB019322	Enhanced
		Low region specificity	Detected in all			HPA001393, CAB007766	Enhanced
		Low region specificity	Detected in all			HPA001396, CAB004395, HPA071038	Supported
		Low region specificity	Detected in all			HPA056033	Enhanced
		Low region specificity	Detected in all			HPA011740, HPA014290, CAB068189, CAB068190	Enhanced
		Not detected	Not detected			HPA067906, HPA074389	Approved
		Low region specificity	Detected in all			HPA002552, HPA023201	Uncertain
		Low region specificity	Detected in all			HPA019635, HPA019691, HPA022119, HPA067742	Enhanced
						HPA059452	
		Low region specificity	Detected in all			HPA002861	Approved
		Low region specificity	Detected in all			CAB005034, CAB018387, HPA023887, HPA063770	Approved
		Low region specificity	Detected in all			CAB013008	Approved
		Low region specificity	Detected in all			HPA042328, HPA074833	Approved
		Low region specificity	Detected in all			HPA017991	Approved
		Low region specificity	Detected in all			HPA000271, HPA001368, CAB022159	Enhanced
		Low region specificity	Detected in all			HPA050483	Uncertain
		Low region specificity	Detected in all			CAB022502, HPA029363, HPA055746	Enhanced
		Low region specificity	Detected in many			HPA029363, HPA077031, CAB079022	
		Low region specificity	Detected in all			HPA035622, HPA058102	Approved
		Low region specificity	Detected in all			HPA054010	Approved
	hypothalamus: 5.2	Low region specificity	Detected in all			HPA074584	
		Low region specificity	Detected in all			CAB004417, HPA013392, HPA015600, CAB078783	Enhanced
		Low region specificity	Detected in all			CAB005197, CAB016392, HPA040067, HPA061280, CAB079968	Supported
		Low region specificity	Detected in many			HPA011048, HPA011070	Uncertain
		Low region specificity	Detected in all			HPA029386, HPA029387, HPA074882	Uncertain
		Low region specificity	Detected in all			HPA041311, HPA048238	Approved
		Region enriched	Detected in all	6	spinal cord: 41.1	HPA020731	Approved
		Low region specificity	Detected in many			HPA047372	Uncertain
		Low region specificity	Detected in all			HPA045339, HPA057705	Enhanced
		Low region specificity	Detected in all			HPA049929, HPA055047	Approved
		Low region specificity	Detected in all			CAB010753, HPA035097, HPA047551, HPA069245	Enhanced
		Not detected	Not detected			HPA074472	
		Not detected	Not detected			HPA055112, HPA073904	Enhanced
		Low region specificity	Detected in all			HPA014846	
		Low region specificity	Detected in all			HPA014005	
	white matter: 105.5	Low region specificity	Detected in all			HPA069619	
		Low region specificity	Detected in all			HPA050873, HPA059441	Uncertain
		Low region specificity	Detected in all			CAB040541, CAB040542, HPA059791	Enhanced
4	basal ganglia: 20.7	Low region specificity	Detected in some			CAB080541	
5	cerebral cortex: 2.1;hippocampal formation:	Low region specificity	Detected in all			CAB022690, HPA049244	Approved
5	hypothalamus: 9.4;midbrain: 15.1;pons and r	Low region specificity	Detected in all			HPA078885, CAB079042	
		Low region specificity	Detected in all			CAB070422, HPA078885	Approved
		Low region specificity	Detected in many			HPA078885	
		Low region specificity	Detected in all			HPA007704	Approved
		Low region specificity	Detected in all			CAB010036, HPA048886	Uncertain
		Low region specificity	Detected in all			CAB010337, HPA018122, HPA027478, HPA028386	Uncertain
		Low region specificity	Detected in all			CAB010032, HPA040736, HPA074924	Approved
		Low region specificity	Detected in some			HPA047611, HPA066482	Approved
		Low region specificity	Detected in all			HPA030571	
		Low region specificity	Detected in all			HPA006316	
		Low region specificity	Detected in all			HPA003648	Enhanced
						HPA046655, HPA060527	Uncertain
		Low region specificity	Detected in all			HPA028642, HPA028644	Uncertain
		Low region specificity	Detected in all			HPA006970	Approved
		Low region specificity	Detected in many			HPA041025, HPA058306	Enhanced
		Low region specificity	Detected in all			CAB033690, HPA075590	Approved

		Region enhanced	Detected in single		midbrain: 1.1	HPA071337	
		Low region specificity	Detected in all			HPA061629, HPA064454	Enhanced
4	hypothalamus: 14.4;white matter: 5.0	Region enriched	Detected in single	12	hypothalamus: 6.6	HPA049862, HPA054678	Enhanced
		Low region specificity	Detected in all			HPA056032	
		Low region specificity	Detected in some			HPA007928, HPA046526	Enhanced
9	cerebellum: 42.5	Low region specificity	Detected in all			HPA029776	Enhanced
		Low region specificity	Detected in all			HPA036478, HPA070892	Enhanced
		Low region specificity	Detected in all			HPA008852, HPA012570	Approved
		Low region specificity	Detected in all			HPA008557, HPA075204	Approved
	cerebellum: 8.3	Low region specificity	Detected in all			CAB005226, HPA057084, CAB073538	Approved
		Low region specificity	Detected in all			HPA014623, HPA073879	Uncertain
		Low region specificity	Detected in all			HPA074001	
		Low region specificity	Detected in all			HPA052160, HPA069762	Approved
		Low region specificity	Detected in all			CAB025579, HPA046838	Approved
	hypothalamus: 2.0	Low region specificity	Detected in all			HPA069604	
		Low region specificity	Detected in all			HPA023313, HPA064826	Enhanced
		Not detected	Not detected			HPA029457	
						CAB002658, HPA014811	Enhanced
		Low region specificity	Detected in all			HPA008965, CAB009445	Enhanced
		Low region specificity	Detected in all			HPA038671, HPA045457	Approved
		Low region specificity	Detected in all			HPA067602	Approved
						HPA028660	Approved
		Low region specificity	Detected in all			CAB005195, HPA035534, HPA063768	Approved
		Low region specificity	Detected in all			HPA019143	Enhanced
		Low region specificity	Detected in all			HPA054516, HPA070481	Enhanced
		Low region specificity	Detected in all			HPA017276, HPA053470	Approved
						CAB078470	
						HPA011078, HPA058509	Enhanced
						CAB024589, HPA031454, CAB080329	Enhanced
		Low region specificity	Detected in all			CAB032907, HPA036913, HPA036914	Approved
6	cerebellum: 189.2	Low region specificity	Detected in all			CAB032656, HPA040999, CAB079015	Enhanced
		Low region specificity	Detected in all			HPA029740	Uncertain
		Low region specificity	Detected in all			CAB005230, HPA072606, HPA074351	Enhanced
		Low region specificity	Detected in all			HPA000497, CAB004439, CAB047330, CAB047331, CAB047332	Enhanced
		Low region specificity	Detected in many			HPA065367, HPA073617, CAB080587	Approved
		Low region specificity	Detected in all			HPA036655	Approved
		Low region specificity	Detected in all			HPA042873	Approved
		Low region specificity	Detected in all			HPA036166, HPA036167, HPA067307	Uncertain
		Low region specificity	Detected in all			CAB026037, HPA029179, CAB079997	Approved
		Low region specificity	Detected in all			HPA037957, HPA062180	Uncertain
						HPA004810, CAB017615	Enhanced
		Low region specificity	Detected in many			HPA039258	
		Low region specificity	Detected in all			CAB025889, HPA029213, HPA063871, CAB080493, CAB080494, CAB080495	Supported
		Low region specificity	Detected in all			HPA004822, CAB010268, HPA045563	Approved
		Not detected	Not detected			HPA012369, HPA070778	Uncertain
		Low region specificity	Detected in all			HPA065647, HPA069086	
						CAB026215, HPA036245	Approved
		Low region specificity	Detected in all			HPA005482	Enhanced
		Low region specificity	Detected in all			HPA007406, HPA007917	Uncertain
		Low region specificity	Detected in all			HPA046641, CAB080341	Enhanced
		Low region specificity	Detected in many			CAB004451, HPA050124, HPA070380	Enhanced
		Low region specificity	Detected in all			HPA010558, CAB025784	Approved
		Low region specificity	Detected in single			CAB010215, HPA067550, CAB080603	Supported
		Low region specificity	Detected in all			CAB013302, HPA014055	Enhanced
		Low region specificity	Detected in all			HPA030813, CAB032306, HPA057327	Supported
		Low region specificity	Detected in all			HPA008572, CAB018594	Enhanced
						CAB004458, HPA004723, CAB072871, CAB080214	Enhanced
		Low region specificity	Detected in all			CAB003434, HPA059297, HPA069003	Enhanced
		Low region specificity	Detected in all			HPA008877, HPA016894	Enhanced
		Low region specificity	Detected in all			HPA027852	Approved
		Region enhanced	Detected in all		spinal cord: 49.5	CAB002422, CAB005258, HPA036348, HPA036349	Enhanced
		Low region specificity	Detected in all			HPA018007	Approved
13	hippocampal formation: 7.4;thalamus: 2.4	Low region specificity	Detected in all			HPA059627	Enhanced
		Low region specificity	Detected in all			HPA028789, HPA077346	
		Region enriched	Detected in many	11	cerebellum: 63.6	HPA005854, HPA047919	Approved
		Low region specificity	Detected in all			CAB002139, HPA032047	Supported
		Low region specificity	Detected in all			HPA005539, HPA056090	Approved
		Low region specificity	Detected in all			HPA038497, HPA038498	Uncertain
		Low region specificity	Detected in all			HPA041345, HPA041550	Enhanced
		Low region specificity	Detected in all			HPA076475	
		Low region specificity	Detected in all			HPA036110, HPA044550	Approved
		Low region specificity	Detected in all			HPA014849	Approved
4	cerebellum: 90.5;pons and medulla: 27.0	Low region specificity	Detected in many			HPA063516	
		Low region specificity	Detected in many			HPA016586, HPA077537, HPA077773	Approved
		Not detected	Not detected			HPA043892	Uncertain
	olfactory bulb: 1.9	Not detected	Not detected			HPA013367, HPA053841, HPA059622	Approved
		Low region specificity	Detected in all			HPA011306, HPA048553, HPA071107	Enhanced
		Low region specificity	Detected in many			HPA043351	
		Low region specificity	Detected in all			HPA014864	Approved
		Region enhanced	Detected in all		cerebellum: 56.2	HPA059880, HPA060577	Uncertain
		Region enhanced	Detected in many		cerebral cortex: 48.0	HPA067233	
		Low region specificity	Detected in single			HPA064442	
		Low region specificity	Detected in all			HPA032050, HPA032051, HPA064839	Approved
		Low region specificity	Detected in some			HPA007156, HPA071913	Approved
		Low region specificity	Detected in all			CAB003288, HPA004471, CAB068253, CAB072867, HPA073252	Enhanced
		Low region specificity	Detected in all			HPA040416, HPA040434	Approved
		Not detected	Not detected			HPA018994, CAB026342, HPA062126	Enhanced
		Low region specificity	Detected in single			CAB021896, HPA068877	Enhanced
		Low region specificity	Detected in all			CAB010896	Enhanced
						HPA011155	Enhanced
		Low region specificity	Detected in all			CAB005272, HPA029100	Supported
		Low region specificity	Detected in all			HPA002997	Approved
		Low region specificity	Detected in all			CAB002223, HPA011157, CAB012978, CAB079960	Enhanced
		Low region specificity	Detected in all			HPA003462	Enhanced
		Low region specificity	Detected in many			HPA002461, HPA003887, CAB025358	Enhanced
		Low region specificity	Detected in all			HPA000895, HPA019493, CAB020673	Enhanced
		Low region specificity	Detected in all			HPA030899	Approved
		Low region specificity	Detected in many			CAB009814, HPA073668	Uncertain
		Low region specificity	Detected in all			HPA074653	Enhanced
		Low region specificity	Detected in some			HPA049665	
						HPA006960	Approved
		Low region specificity	Detected in all			HPA002235, HPA071091	Approved
		Low region specificity	Detected in some			HPA013421	
		Low region specificity	Detected in some			HPA046563, HPA054951, HPA055571	Uncertain
		Low region specificity	Detected in all			HPA028934, HPA076273	Approved
		Low region specificity	Detected in all			HPA011133, HPA017342	Enhanced
		Low region specificity	Detected in many			HPA076660	Enhanced
		Low region specificity	Detected in all			HPA004182, CAB018621, HPA022903	Supported
		Not detected	Not detected			HPA056312	Approved
		Low region specificity	Detected in all			HPA007270, HPA075309	Approved
						HPA079451	
		Low region specificity	Detected in many			CAB022689, HPA029680, HPA072921	Uncertain
		Low region specificity	Detected in all			HPA001231, CAB004492	Enhanced
		Low region specificity	Detected in all			HPA013713, HPA068064, HPA068103	Approved
		Low region specificity	Detected in all			HPA038568	Uncertain
		Low region specificity	Detected in all			HPA031851, HPA031853, HPA077002	Supported
		Low region specificity	Detected in all			HPA008444	
		Low region specificity	Detected in all			CAB003834, HPA026430, CAB080093	Approved
		Low region specificity	Detected in all			HPA007633, HPA075335	Approved
		Low region specificity	Detected in all			HPA058921, HPA070332, HPA079657	Approved
		Low region specificity	Detected in all			CAB000151, HPA048895, HPA069524, HPA069570, CAB072344	Enhanced
		Low region specificity	Detected in all			CAB022062, HPA067595, HPA069443	Enhanced
		Low region specificity	Detected in all			HPA038790, HPA074905	Approved
8	cerebellum: 7.9	Low region specificity	Detected in all			HPA018119, HPA061726	Uncertain
						HPA017983	Uncertain
		Not detected	Not detected			HPA042365, HPA058088	Approved
		Low region specificity	Detected in all			CAB002147, HPA008848	Enhanced
	cerebellum: 1.3	Not detected	Not detected			HPA014731	Enhanced
		Low region specificity	Detected in all			CAB010423	

		Low region specificity	Detected in many			HPA030115	
		Low region specificity	Detected in all			HPA031763, CAB032637	Uncertain
		Low region specificity	Detected in all			HPA031235, HPA050382	Enhanced
						HPA066867, HPA067626	
		Low region specificity	Detected in all			HPA011401	
		Low region specificity	Detected in all			HPA036196, HPA075622	Enhanced
		Low region specificity	Detected in all			HPA017986, HPA062306, HPA077490	Enhanced
		Low region specificity	Detected in all			HPA043107	Uncertain
		Low region specificity	Detected in all			HPA043480, HPA051956	Approved
		Low region specificity	Detected in all			HPA056650, HPA061344	Approved
		Low region specificity	Detected in many			HPA049511, HPA062232	Enhanced
		Low region specificity	Detected in all			HPA078638	
		Not detected	Not detected			HPA070324	
	basal ganglia: 28.1	Region enhanced	Detected in all		basal ganglia: 43.8	CAB000013, HPA052583, HPA056072	Enhanced
		Low region specificity	Detected in many			CAB025177, HPA055640	Uncertain
	cerebral cortex: 1.5	Low region specificity	Detected in many			HPA007619	
						HPA076675	Enhanced
		Low region specificity	Detected in all			HPA037597, HPA037598	Approved
		Low region specificity	Detected in all			HPA026966, HPA063538	Approved
		Low region specificity	Detected in all			HPA048304	
5	cerebellum: 14.8	Region enhanced	Detected in many		spinal cord: 6.4	HPA028811	Approved
		Not detected	Not detected			CAB000015, HPA014341, HPA014391	Enhanced
		Region enriched	Detected in single	9	spinal cord: 3.8	CAB002216, HPA017172, CAB080356, CAB080357	Enhanced
		Low region specificity	Detected in all			HPA000263, CAB010898, HPA011135, HPA011227, CAB047336, CAB047337,	Enhanced
		Low region specificity	Detected in all			HPA010008, HPA010665	Approved
		Low region specificity	Detected in many			CAB000036, CAB001986, HPA004179, HPA007235, HPA008855, CAB080102,	Enhanced
		Region enhanced	Detected in all		midbrain: 52.0	CAB002302, HPA014539, HPA015310	Enhanced
		Low region specificity	Detected in all			CAB020789, HPA031677, HPA048055	Enhanced
		Low region specificity	Detected in all			CAB015224, HPA024223	Enhanced
		Low region specificity	Detected in all			HPA013607, HPA060144	Approved
		Low region specificity	Detected in all			HPA001768, HPA005924, CAB037023	Enhanced
		Low region specificity	Detected in single			HPA021252	Enhanced
		Low region specificity	Detected in all			HPA014245	Enhanced
		Low region specificity	Detected in all			HPA031889, HPA031890, HPA031958	Approved
		Low region specificity	Detected in all			HPA054050, HPA057065	
		Low region specificity	Detected in all			HPA008208, HPA011389	Enhanced
		Low region specificity	Detected in all			CAB000142, CAB018071, HPA039835	Enhanced
		Low region specificity	Detected in all			HPA030900, HPA030901	Enhanced
		Low region specificity	Detected in all			HPA039490, HPA040772	Approved
		Low region specificity	Detected in all			CAB018587, HPA019713	Enhanced
		Low region specificity	Detected in many			HPA010775	Enhanced
		Low region specificity	Detected in all			CAB009320, HPA027804, HPA027805, HPA027806	Uncertain
		Low region specificity	Detected in all			HPA015634, HPA021506	Approved
		Low region specificity	Detected in all			HPA045502, HPA075627, CAB080363, CAB080365	Uncertain
		Low region specificity	Detected in all			HPA003097, CAB005385	Supported
		Region enhanced	Detected in many		basal ganglia: 13.5	CAB000143, CAB001995, HPA004765	Enhanced
		Low region specificity	Detected in many			HPA031497, HPA076257	Uncertain
		Low region specificity	Detected in all			HPA021284, HPA021417, HPA023261, HPA024312	Uncertain
		Low region specificity	Detected in all			HPA052460, HPA058772	Enhanced
		Low region specificity	Detected in all			HPA022120, HPA051576	Approved
		Low region specificity	Detected in all			HPA003183, HPA004066	Enhanced
		Low region specificity	Detected in many			HPA026665	
		Low region specificity	Detected in all			HPA001303	Approved
	hypothalamus: 1.7	Low region specificity	Detected in all			HPA027800, HPA045836	Enhanced
		Low region specificity	Detected in all			CAB002167, HPA058312, HPA069509	Enhanced
		Low region specificity	Detected in all			HPA048743	Approved
		Low region specificity	Detected in all			CAB005393, HPA044392, HPA067424	Approved
4	olfactory bulb: 4.3	Low region specificity	Detected in all			HPA026825	
		Low region specificity	Detected in all			CAB010157, HPA049830, HPA072761, CAB079912	Uncertain
		Low region specificity	Detected in all			HPA017357	Approved
		Group enriched	Detected in all	4	hippocampal formation: 26.0;midbrain: 79.9;pons: 71.8;thalamus: 99.3	HPA065954	
		Low region specificity	Detected in all			HPA024306, HPA071290	Approved
		Low region specificity	Detected in all			HPA007637, CAB010346, HPA074873	Enhanced
		Low region specificity	Detected in all			HPA050299	
		Low region specificity	Detected in all			HPA002874, CAB011478	Approved
		Low region specificity	Detected in all			HPA005933, CAB013075, CAB068212, CAB068213, CAB068214	Enhanced
		Low region specificity	Detected in all			HPA030751, HPA030752, HPA030753, HPA030754, CAB034099	Approved
		Low region specificity	Detected in some			HPA077718	Enhanced
		Low region specificity	Detected in all			HPA050798	
						CAB013682, HPA049243	Uncertain
		Low region specificity	Detected in many			HPA067549	Enhanced
		Not detected	Not detected			HPA074114	
						HPA078297	
						HPA078297	
		Low region specificity	Detected in all			HPA058578	
		Low region specificity	Detected in many			HPA051090	Uncertain
		Low region specificity	Detected in all			HPA032131	Enhanced
		Low region specificity	Detected in all			HPA036076, HPA053083, HPA073967	Uncertain
						HPA067673	
		Low region specificity	Detected in all			HPA049854, HPA056520	Enhanced
		Low region specificity	Detected in all			HPA001632, HPA066527	Enhanced
		Low region specificity	Detected in all			HPA073653, HPA076187	Approved
		Low region specificity	Detected in all			HPA003565, CAB005312	Enhanced
		Low region specificity	Detected in all			CAB025747, CAB044670, HPA070175, HPA072220	Uncertain
		Low region specificity	Detected in all			HPA041713, HPA049578	Approved
		Low region specificity	Detected in all			HPA058905, HPA074153, HPA076866	Uncertain
		Low region specificity	Detected in all			HPA000993, HPA063890	Enhanced
		Low region specificity	Detected in all			HPA016930	Uncertain
		Low region specificity	Detected in all			HPA004624, HPA039117	Enhanced
		Low region specificity	Detected in all			HPA064625	
		Low region specificity	Detected in all			HPA030443	Approved
		Low region specificity	Detected in all			HPA035283	Approved
		Low region specificity	Detected in all			CAB009733, HPA041551	Approved
		Low region specificity	Detected in all			HPA003568, HPA072217	Enhanced
		Region enhanced	Detected in all		cerebellum: 93.4	HPA066352, HPA066960, HPA069079	Approved
		Low region specificity	Detected in all			HPA047720, HPA050538	Enhanced
		Low region specificity	Detected in all			HPA011866, HPA046521	Uncertain
						HPA043180, HPA073478	Approved
						HPA008755, HPA010580, HPA077526	Uncertain
		Low region specificity	Detected in all			CAB021103, HPA050294, HPA060836	Approved
		Low region specificity	Detected in all			CAB017632, HPA043310, HPA076091	Uncertain
		Low region specificity	Detected in all			HPA045895	Uncertain
		Low region specificity	Detected in all			HPA011380, HPA011978	Enhanced
		Low region specificity	Detected in all			HPA009134	Approved
		Low region specificity	Detected in all			HPA004947, CAB018143	Supported
		Low region specificity	Detected in all			HPA007534, HPA007949, CAB008376	Enhanced
						CAB020784, HPA036503, HPA036504, HPA064387, HPA076072	Uncertain
		Low region specificity	Detected in all			HPA004690, HPA004871	Enhanced
		Low region specificity	Detected in many			HPA078754	
		Low region specificity	Detected in all			HPA035146, HPA035147	Approved
		Low region specificity	Detected in all			HPA039922, HPA040374	Approved
		Low region specificity	Detected in all			HPA069976	Enhanced
		Low region specificity	Detected in all			HPA029366	Approved
		Low region specificity	Detected in all			CAB004544, HPA015794, HPA055297	Approved
4	hippocampal formation: 20.2	Low region specificity	Detected in all			HPA014314, CAB037336, HPA056908	Enhanced
37	cerebellum: 8.9	Low region specificity	Detected in all			CAB012993, HPA062937	Enhanced
		Low region specificity	Detected in all			CAB037334, HPA066647, HPA070648	Approved
		Low region specificity	Detected in all			HPA000825, HPA001171	Approved
		Low region specificity	Detected in all			CAB005035, HPA020994, HPA020996	Approved
		Low region specificity	Detected in all			HPA050843, CAB073533	Uncertain
		Low region specificity	Detected in all			HPA007951	Approved
		Low region specificity	Detected in all			CAB004277, HPA036681, HPA036682	Approved
		Low region specificity	Detected in all			HPA038610, HPA066103	Approved
		Low region specificity	Detected in all			HPA041459, HPA058230	Uncertain
		Low region specificity	Detected in some			HPA031741, HPA031742	Uncertain
		Low region specificity	Detected in single			HPA044682, HPA054657	Enhanced
		Low region specificity	Detected in all			CAB033331, HPA047815	Enhanced
		Low region specificity	Detected in all			HPA055540, HPA058428	Uncertain
		Low region specificity	Detected in all			HPA068923, CAB070421	Approved

		Low region specificity	Detected in all			HPA002110, CAB016169, HPA045507, CAB062558, CAB068219, CAB068220	Enhanced
		Not detected	Not detected			HPA058210, HPA067385	
		Low region specificity	Detected in all			HPA001924, HPA071162	Uncertain
		Low region specificity	Detected in all			HPA039380	Approved
		Low region specificity	Detected in all			HPA049571, HPA079512	Approved
		Low region specificity	Detected in all			CAB005610, HPA047157	Approved
		Low region specificity	Detected in all			CAB017183, HPA038467, HPA038468	Supported
		Not detected	Not detected			HPA045153, HPA068562, CAB080138	Enhanced
		Low region specificity	Detected in all			CAB003844, HPA006563, HPA006564, CAB016290	Enhanced
		Low region specificity	Detected in all			CAB001948, HPA008236, HPA044496	Enhanced
		Low region specificity	Detected in all			CAB018367, HPA029834	Uncertain
		Low region specificity	Detected in all			HPA029529	Uncertain
		Low region specificity	Detected in all			HPA046636	Uncertain
		Low region specificity	Detected in all			HPA014447, HPA019203, HPA048045	Enhanced
		Region enriched	Detected in many	8	spinal cord: 50.6	HPA030783, HPA056418	Approved
		Low region specificity	Detected in all			HPA053927, HPA062763	Approved
		Low region specificity	Detected in all			HPA017871, HPA064293	Enhanced
		Low region specificity	Detected in many			HPA010116, HPA010600, HPA072744	Enhanced
		Low region specificity	Detected in all			HPA027543	Approved
		Low region specificity	Detected in many			HPA046839	
		Low region specificity	Detected in all			HPA007491, HPA075879	Enhanced
		Low region specificity	Detected in all			CAB003850, HPA026091, HPA026276	Approved
		Low region specificity	Detected in all			HPA003222	Approved
		Low region specificity	Detected in all			HPA008708	
		Low region specificity	Detected in all			HPA006026	Approved
		Low region specificity	Detected in all			HPA054056, HPA054822	Uncertain
		Low region specificity	Detected in all			HPA003891, CAB022442, CAB022443	Supported
5	cerebellum: 89.6	Low region specificity	Detected in all			CAB011461, HPA011851, HPA071067	Uncertain
		Low region specificity	Detected in all			HPA054747	
		Low region specificity	Detected in all			HPA012568, HPA064739	Approved
		Low region specificity	Detected in all			HPA024068	Supported
		Low region specificity	Detected in all			HPA003996	Approved
		Low region specificity	Detected in all			HPA029135, HPA029136	Enhanced
		Not detected	Not detected			HPA010940, CAB033645	Enhanced
		Low region specificity	Detected in all			HPA041945	
						HPA062673, HPA066741	
						HPA066741	
		Low region specificity	Detected in all			HPA002639, HPA002640, CAB019291, CAB080384	Approved
		Low region specificity	Detected in all			HPA047037, HPA065232	Approved
		Low region specificity	Detected in all			HPA007622, CAB046010, HPA046651	Approved
4	cerebellum: 62.2;cerebral cortex: 49.2;thalam	Low region specificity	Detected in all			HPA075527	
		Low region specificity	Detected in all			HPA068798	
		Low region specificity	Detected in all			HPA016744, HPA020027	Approved
		Low region specificity	Detected in many			HPA043417	Enhanced
		Low region specificity	Detected in all			HPA039389	
		Low region specificity	Detected in all			HPA015635, HPA015667	Approved
		Low region specificity	Detected in all			HPA041929	
		Low region specificity	Detected in all			HPA027428, HPA027434, HPA027448	Approved
		Low region specificity	Detected in all			HPA039683, HPA040776	Enhanced
		Low region specificity	Detected in all			HPA000263, HPA000763, CAB037312	Enhanced
		Low region specificity	Detected in all			CAB025109, HPA071729	Supported
		Low region specificity	Detected in all			HPA013377	Approved
		Low region specificity	Detected in all			HPA029961, HPA029962, HPA029963	Approved
		Group enriched	Detected in many	5	midbrain: 86.5;spinal cord: 33.3	CAB002581, HPA008356, HPA008495	Approved
		Low region specificity	Detected in all			HPA056384, HPA069984	Enhanced
						HPA044234	
		Low region specificity	Detected in all			HPA037859	
		Low region specificity	Detected in all			CAB005052	Approved
		Low region specificity	Detected in all			CAB012643, HPA047193, HPA073217	Approved
		Low region specificity	Detected in all			HPA077800	
		Low region specificity	Detected in all			HPA027158, HPA027209	Approved
		Low region specificity	Detected in all			CAB013524, HPA052968, HPA074461	Uncertain
		Low region specificity	Detected in all			HPA000234, HPA000909	Uncertain
						CAB009561	Approved
		Low region specificity	Detected in all			HPA049945, HPA074403	
		Low region specificity	Detected in all			HPA027813, HPA028676	Approved
		Low region specificity	Detected in all			CAB012443	
		Low region specificity	Detected in all			HPA044428	Uncertain
		Region enhanced	Detected in many		spinal cord: 20.9	HPA042674, HPA056140	Enhanced
		Low region specificity	Detected in all			CAB002601, HPA007575, CAB040549, CAB047354	Enhanced
		Not detected	Not detected			HPA004193, CAB009441, CAB080262	Enhanced
		Not detected	Not detected			HPA039737, HPA040154	Enhanced
		Low region specificity	Detected in all			HPA078664	
		Low region specificity	Detected in all			HPA078632	
5	amygdala: 3.1;basal ganglia: 10.1;hypothalar	Region enhanced	Detected in many		basal ganglia: 6.6	HPA063346	
		Low region specificity	Detected in all			CAB022169, HPA043937, HPA073590	
		Low region specificity	Detected in all			HPA026884	Enhanced
13	cerebellum: 2.5	Not detected	Not detected			HPA071194	
		Low region specificity	Detected in all			CAB015463, CAB022081, HPA023557, HPA064312	Supported
		Low region specificity	Detected in all			HPA040606	Uncertain
		Low region specificity	Detected in all			CAB025123, HPA068532, HPA074427	Uncertain
		Low region specificity	Detected in all			HPA028467, HPA031255, HPA031256	Approved
		Low region specificity	Detected in all			HPA015662, HPA023277	Approved
		Low region specificity	Detected in all			HPA037898, HPA037899	Supported
		Low region specificity	Detected in all			HPA032129, HPA032130	Uncertain
						CAB025807, HPA027093	Enhanced
						HPA014377, HPA014428	Uncertain
		Low region specificity	Detected in all			HPA002875, HPA077689	Approved
		Low region specificity	Detected in all			HPA042072, HPA045480, HPA074988	Uncertain
		Low region specificity	Detected in all			HPA014333, HPA014353, HPA014736	Enhanced
		Low region specificity	Detected in all			HPA044343	Approved
		Low region specificity	Detected in all			HPA059570, HPA077233	Approved
		Low region specificity	Detected in all			HPA003324, CAB017489, HPA071055, CAB079035	Enhanced
		Low region specificity	Detected in all			HPA016860	Approved
		Low region specificity	Detected in many			CAB017490, HPA021451	Approved
		Region enriched	Detected in all	5	cerebellum: 30.2	HPA005911	Approved
		Low region specificity	Detected in all			HPA044479	
		Low region specificity	Detected in all			HPA035239, HPA035240	Enhanced
6	cerebellum: 136.3	Region enhanced	Detected in all		cerebellum: 70.8	HPA041505, HPA044066	Enhanced
		Low region specificity	Detected in all			CAB019279, HPA035834, HPA064386	Approved
		Low region specificity	Detected in all			HPA079359	
		Low region specificity	Detected in all			HPA048363, HPA072381	Approved
		Low region specificity	Detected in all			HPA019551, HPA056247	Enhanced
		Low region specificity	Detected in all			CAB002759, HPA031345, HPA058494	Enhanced
		Low region specificity	Detected in all			HPA031593, HPA031594	Approved
						HPA006539	Supported
		Low region specificity	Detected in many			CAB010444, HPA028997, HPA069490	Enhanced
						CAB002763, HPA006539	Approved
		Low region specificity	Detected in all			HPA005449, HPA075145	Enhanced
		Low region specificity	Detected in all			HPA015275, HPA049000	Uncertain
		Region enhanced	Detected in some		hippocampal formation: 5.5	HPA065953, HPA076165	Enhanced
		Not detected	Not detected			HPA051255, HPA077175	Approved
		Low region specificity	Detected in all			HPA037989, HPA066474	Enhanced
		Low region specificity	Detected in all			HPA052054, HPA064728	Approved
		Low region specificity	Detected in all			HPA035180, HPA069672	Approved
		Low region specificity	Detected in all			HPA042377	Approved
		Low region specificity	Detected in all			CAB010455, HPA017980	Enhanced
		Low region specificity	Detected in all			HPA065634	Approved
		Low region specificity	Detected in all			HPA045847	Approved
		Low region specificity	Detected in all			CAB011614	Approved
		Low region specificity	Detected in all			HPA019339	Enhanced
		Low region specificity	Detected in all			CAB022493, HPA035628, HPA035629, HPA079220	Enhanced
		Low region specificity	Detected in all			CAB022494, HPA035857	Enhanced
26	cerebellum: 7.6	Low region specificity	Detected in many			CAB015467, HPA051805, HPA055106	Enhanced
		Not detected	Not detected			HPA060904	Enhanced
		Low region specificity	Detected in all			HPA029791	
		Low region specificity	Detected in all			HPA009277	
		Low region specificity	Detected in all			HPA052673, HPA056077	Approved
		Low region specificity	Detected in all			HPA050713	Uncertain

		Low region specificity	Detected in all			CAB022694, HPA070007	Uncertain
		Low region specificity	Detected in all			CAB022371, HPA048532, HPA052891	Enhanced
		Low region specificity	Detected in many			HPA035121, HPA035122	Approved
14	cerebellum: 4.1	Low region specificity	Detected in many			HPA036493, HPA036669	Enhanced
		Low region specificity	Detected in all			HPA001672, HPA066520	Enhanced
		Low region specificity	Detected in all			HPA059445, HPA059590	Enhanced
		Low region specificity	Detected in all			HPA059122, HPA079529	Enhanced
		Low region specificity	Detected in all			HPA071152	
						HPA050892, HPA073093	Supported
						HPA004943, HPA050892	Enhanced
5	cerebellum: 6.7	Low region specificity	Detected in all			HPA030669, HPA030670	Uncertain
7	basal ganglia: 2.3;olfactory bulb: 6.7	Region enriched	Detected in many	7	olfactory bulb: 17.5	HPA025062	Uncertain
		Low region specificity	Detected in all			HPA051630	Approved
	thalamus: 22.6	Low region specificity	Detected in many			HPA014491, HPA014513	Uncertain
		Low region specificity	Detected in all			HPA039711	Enhanced
		Low region specificity	Detected in all			HPA001214, CAB037083, CAB037317	Approved
		Low region specificity	Detected in all			HPA001394	
						HPA065258, HPA077904	Approved
		Low region specificity	Detected in all			HPA031410, HPA057203	Approved
		Low region specificity	Detected in all			HPA027559, CAB032850, HPA043084	Enhanced
		Low region specificity	Detected in all			HPA006889, CAB011498	Enhanced
		Low region specificity	Detected in all			HPA040412, HPA040947	Approved
		Region enhanced	Detected in many		olfactory bulb: 7.6	HPA022829, HPA028761	Uncertain
		Not detected	Not detected			HPA006903, HPA031178	Uncertain
		Low region specificity	Detected in all			HPA040737, HPA040942	Uncertain
		Low region specificity	Detected in all			CAB005592, HPA039292	Approved
		Low region specificity	Detected in all			HPA004194, HPA059958	Uncertain
		Low region specificity	Detected in all			CAB004023, HPA030875	Approved
5	basal ganglia: 4.1;hypothalamus: 2.3	Low region specificity	Detected in many			HPA066503	
		Low region specificity	Detected in all			HPA035800, HPA035801	Uncertain
		Low region specificity	Detected in single			HPA065201	
		Low region specificity	Detected in all			HPA015083, HPA015500, CAB020840	Enhanced
		Low region specificity	Detected in all			HPA010961, HPA011419	Approved
		Low region specificity	Detected in all			CAB015944, HPA062016	Supported
6	olfactory bulb: 23.6	Low region specificity	Detected in all			HPA012892, HPA071011	Enhanced
		Low region specificity	Detected in all			HPA003019	Approved
		Low region specificity	Detected in all			CAB010878, HPA020095, HPA020138, CAB080402	Supported
		Low region specificity	Detected in all			HPA006810, HPA006929	Supported
		Low region specificity	Detected in all			HPA041756, HPA042449	Approved
		Low region specificity	Detected in all			HPA039651	Approved
		Low region specificity	Detected in all			HPA005480, HPA024300, CAB037030	Enhanced
		Low region specificity	Detected in many			HPA055565, HPA074573	Enhanced
		Low region specificity	Detected in all			HPA076573	
		Low region specificity	Detected in some			HPA043104, HPA055067, CAB072852	Enhanced
		Low region specificity	Detected in all			HPA053439	Uncertain
		Low region specificity	Detected in all			HPA007725, HPA008142, HPA076041	Approved
		Low region specificity	Detected in all			HPA044712, HPA068080	Uncertain
		Low region specificity	Detected in all			HPA039772, HPA040043	Approved
		Low region specificity	Detected in all			HPA077366	
						HPA038756, HPA038758	Uncertain
						HPA038756, HPA038758	Uncertain
		Low region specificity	Detected in many			HPA005733, HPA024657	Approved
		Low region specificity	Detected in all			CAB010359, HPA073265	Approved
		Low region specificity	Detected in all			CAB002441, CAB031481, HPA056473	Uncertain
		Low region specificity	Detected in all			CAB073537, HPA074821	
		Region enhanced	Detected in all		cerebellum: 363.0	CAB010416, HPA034853	Enhanced
		Low region specificity	Detected in all			HPA041357, HPA041474	Uncertain
		Low region specificity	Detected in all			HPA001636, HPA001637, CAB010822	Enhanced
		Low region specificity	Detected in all			HPA001813, CAB009228, HPA070714	Supported
		Low region specificity	Detected in single			CAB013244, HPA046863, HPA053337	Enhanced
		Low region specificity	Detected in all			CAB002006, HPA004748	Supported
		Low region specificity	Detected in all			HPA078321	
		Low region specificity	Detected in all			CAB004025, HPA049174	Approved
		Low region specificity	Detected in all			HPA051838, HPA063184, HPA065531	Uncertain
		Low region specificity	Detected in all			HPA056530, HPA064017	Enhanced
6	cerebellum: 58.7	Region enriched	Detected in all	5	cerebellum: 29.6	HPA041921, HPA049425	Enhanced
		Low region specificity	Detected in all			HPA029249, HPA029250, HPA029251	Uncertain
		Low region specificity	Detected in all			HPA007108, HPA025020	Uncertain
		Low region specificity	Detected in some			HPA034493, HPA035787	Enhanced
		Low region specificity	Detected in all			CAB010391, HPA047976	Approved
						HPA006404, CAB026150, CAB030007, CAB080525, CAB080526	Approved
		Low region specificity	Detected in all			HPA006746, CAB009805	Approved
	hypothalamus: 5.5	Low region specificity	Detected in all			CAB000016, HPA014823, HPA032081, HPA032082	Approved
		Low region specificity	Detected in many			CAB009188, HPA030526	Enhanced
		Low region specificity	Detected in all			HPA041954, HPA069561	Approved
		Low region specificity	Detected in all			HPA021603, HPA050429	Supported
		Low region specificity	Detected in some			HPA045817, HPA074725	Approved
		Region enhanced	Detected in many		basal ganglia: 14.2	HPA036261, HPA036262	Enhanced
		Not detected	Not detected			HPA002356, CAB026130, HPA072760	Enhanced
10	olfactory bulb: 11.0	Region enriched	Detected in some	24	olfactory bulb: 40.1	HPA038226, HPA058226	Enhanced
		Not detected	Not detected			HPA016700, HPA017860	Enhanced
		Low region specificity	Detected in all			HPA041169	Enhanced
		Low region specificity	Detected in all			HPA044993	Uncertain
		Low region specificity	Detected in all			HPA044657, HPA071160	Approved
		Low region specificity	Detected in all			HPA060691, HPA066996	
		Low region specificity	Detected in all			HPA038508	Approved
		Low region specificity	Detected in all			HPA015285, HPA018496	Uncertain
		Low region specificity	Detected in all			HPA078636	
		Low region specificity	Detected in all			HPA041174, HPA041390	Supported
		Low region specificity	Detected in all			CAB009493, HPA041899	Enhanced
5	basal ganglia: 4.3;white matter: 11.5	Low region specificity	Detected in many			CAB011464, HPA034569, CAB035990, HPA054975, CAB080407	Approved
		Low region specificity	Detected in all			HPA003920	Uncertain
						HPA007547, HPA071005	Enhanced
						CAB018974, HPA066741	Approved
		Low region specificity	Detected in all			HPA001308	Approved
		Not detected	Not detected			HPA076515	Enhanced
		Low region specificity	Detected in all			CAB016348, HPA018894	Enhanced
		Low region specificity	Detected in all			CAB004612, HPA005724	Enhanced
		Not detected	Not detected			CAB002452, HPA006884, HPA006885	Enhanced
		Low region specificity	Detected in all			HPA039734, HPA043947	Approved
		Low region specificity	Detected in all			CAB020804, HPA036434, HPA070293	Approved
		Low region specificity	Detected in all			CAB009932, HPA035707, HPA069673	Supported
		Low region specificity	Detected in all			HPA035005, HPA035006	Approved
		Low region specificity	Detected in all			CAB004370, HPA026480	Approved
		Low region specificity	Detected in all			HPA028400, HPA028439	Uncertain
		Low region specificity	Detected in all			HPA000755, CAB001980, HPA005732	Approved
		Low region specificity	Detected in all			HPA023331, HPA023334	Uncertain
		Low region specificity	Detected in all			HPA014483, HPA014702	Approved
		Low region specificity	Detected in all			HPA049471, HPA072213	
		Low region specificity	Detected in all			HPA014670	Approved
		Low region specificity	Detected in all			HPA000400, HPA016438	Enhanced
		Low region specificity	Detected in all			HPA029534, HPA050750	Uncertain

Reliability (Mouse Brain)	Reliability (IF)	Subcellular location	Secretome location	Secretome function	CCD Protein	CCD Transcript	Blood concentration - Conc. blood IM [pg/L]	Blood concentration - Conc. blood MS [pg/L]	Blood expression cluster
	Supported	Vesicles,Plasma membrane,Cytosol			No	No			Cluster 11: T-cells - Unknown function
	Approved	Plasma membrane,Microtubules,Cytokinetic bridge,Primary cilium,Primary cilium tip,Cytosol			Yes	NA			Cluster 33: Non-specific - ATP binding
	Supported	Golgi apparatus,Plasma membrane			NA	NA			Cluster 22: Eosinophils - Transcription
	Supported	Golgi apparatus,Plasma membrane,Cell Junctions			NA	NA			Cluster 29: Non-specific - Unknown function
	Supported	Plasma membrane			NA	NA			Cluster 24: Non-specific - Transcription
	Approved	Nucleoplasm,Plasma membrane,Focal adhesion sites,Cytosol			NA	NA			Cluster 34: B-cells - Unknown function
	Supported	Nucleoplasm,Golgi apparatus,Plasma membrane,Mitochondria,Cytosol,Mid piece,Principal piece	Intracellular and membrane	Transport	NA	NA			Cluster 8: Plasmacytoid DCs - Unknown function
	Supported	Plasma membrane,Cell Junctions			NA	NA			
	Supported	Plasma membrane,Basal body			NA	NA			Cluster 40: Monocytes - Innate immune response
	Supported	Golgi apparatus,Vesicles,Plasma membrane			NA	NA			Cluster 12: Non-specific - Mitochondrial translation
	Supported	Nucleoplasm,Plasma membrane			NA	NA			Cluster 38: Monocytes & Neutrophils - Degranulation
	Supported	Nucleoplasm,Plasma membrane			NA	NA			Cluster 15: NK-cells - Unknown function
	Supported	Vesicles,Plasma membrane			NA	NA			Cluster 24: Non-specific - Transcription
	Supported	Plasma membrane,Cytosol			No	No		210000	Cluster 10: Non-specific - Membrane trafficking
	Supported	Vesicles,Plasma membrane	Intracellular and membrane	Enzyme	NA	NA		950000	Cluster 38: Monocytes & Neutrophils - Degranulation
	Supported	Plasma membrane	Secreted in female reproductive system	Cell adhesion	NA	NA		14000000	Cluster 1: T-cells - Unknown function
	Approved	Plasma membrane,Mitochondria	Secreted to blood	Enzyme	NA	NA	3720000		Cluster 32: Basophils - Transcription
	Uncertain	Nucleoplasm,Nucleoli,Golgi apparatus,Plasma membrane,Primary cilium,Primary cilium transition zone,Basal body			NA	NA			Cluster 13: Non-specific - Mitochondria
	Approved	Nucleoplasm,Plasma membrane,Mitochondria			NA	NA			
	Approved	Plasma membrane,Primary cilium,Basal body,Cytosol			NA	NA			
	Enhanced	Nucleoplasm,Plasma membrane			NA	NA		720000	Cluster 14: Non-specific - Unknown function
	Approved	Nucleoplasm,Plasma membrane,Cytosol			NA	NA		47000	Cluster 14: Non-specific - Unknown function
	Supported	Plasma membrane			NA	NA		120000	Cluster 42: Non-specific - Vesicular transport
	Supported	Vesicles,Plasma membrane			NA	NA		73000	
	Supported	Plasma membrane	Intracellular and membrane	Receptor	NA	NA		13000	
	Approved	Nucleoplasm,Nuclear speckles,Plasma membrane			NA	NA		170000	Cluster 26: Monocytes - Plasma membrane proteins
	Approved	Plasma membrane,Cytosol			NA	NA		2200000	
	Supported	Plasma membrane,Primary cilium			NA	NA			
	Supported	Vesicles,Plasma membrane,Primary cilium tip			NA	Yes			Cluster 26: Monocytes - Plasma membrane proteins
	Supported	Plasma membrane			NA	NA			
	Supported	Plasma membrane,Microtubules,Cytokinetic bridge,Mitotic spindle,Primary cilium,Basal body			Yes	NA			Cluster 25: Non-specific - Unknown function
	Enhanced	Nucleoplasm,Plasma membrane,Cell Junctions			NA	NA			Cluster 20: Non-specific - DNA binding
	Approved	Nucleoli fibrillar center,Plasma membrane,Cell Junctions	Secreted in other tissues	Receptor	NA	NA		72000	
	Enhanced	Plasma membrane,Cytosol			NA	NA		48000000	Cluster 38: Monocytes & Neutrophils - Degranulation
	Enhanced	Plasma membrane,Cytosol			No	No		400000	
	Enhanced	Plasma membrane,Cytosol			No	No		3700000	Cluster 46: Basophils - Proteolysis
	Supported	Plasma membrane,Cytosol	Secreted to digestive system	Enzyme	NA	NA		250000	
	Supported	Plasma membrane,Cytosol			NA	NA			
	Approved	Nucleoli,Golgi apparatus,Plasma membrane,Basal body,Cytosol			NA	NA			Cluster 11: T-cells - Unknown function
	Supported	Plasma membrane			NA	NA			
	Supported	Plasma membrane,Cytosol			NA	NA			Cluster 26: Monocytes - Plasma membrane proteins
	Supported	Nuclear bodies,Vesicles,Plasma membrane			NA	NA			
	Supported	Plasma membrane			NA	NA			
	Supported	Nucleoplasm,Cell Junctions			NA	NA			
Approved	Supported	Plasma membrane,Acrosome			NA	NA			
	Supported	Plasma membrane			NA	NA			Cluster 24: Non-specific - Transcription
	Supported	Plasma membrane,Cytosol			NA	NA			Cluster 14: Non-specific - Unknown function
	Supported	Nucleoplasm,Plasma membrane,Cell Junctions			NA	NA			Cluster 5: Non-specific - Cell proliferation
	Supported	Vesicles,Plasma membrane,Primary cilium,Primary cilium tip,Primary cilium transition zone			NA	NA			Cluster 22: Eosinophils - Transcription
	Uncertain	Vesicles,Plasma membrane			NA	NA			
	Supported	Nucleoplasm,Plasma membrane			NA	NA			
	Supported	Vesicles,Plasma membrane,Primary cilium			NA	NA			Cluster 38: Monocytes & Neutrophils - Degranulation
	Supported	Nucleoplasm,Plasma membrane			NA	NA			
	Approved	Plasma membrane,Cytosol			NA	NA		41000	Cluster 10: Non-specific - Membrane trafficking
	Supported	Plasma membrane			NA	NA		420000000	Cluster 38: Monocytes & Neutrophils - Degranulation
	Supported	Nucleoplasm,Plasma membrane,Cytosol	Secreted to blood	Immunity	NA	Yes	17600000	24000000	Cluster 42: Non-specific - Vesicular transport
	Supported	Nucleoplasm,Plasma membrane			NA	NA			
	Supported	Plasma membrane			NA	NA		5000000	Cluster 4: Neutrophils - Degranulation
	Supported	Plasma membrane			NA	NA		6500	Cluster 29: Non-specific - Unknown function
	Supported	Endoplasmic reticulum,Plasma membrane			NA	NA			Cluster 39: T-cells - T-cell receptor
	Enhanced	Plasma membrane,Cytosol			No	NA			Cluster 35: Neutrophils - Protein binding
	Supported	Golgi apparatus,Plasma membrane			NA	NA			Cluster 35: Neutrophils - Protein binding
	Supported	Plasma membrane,Centrosome			NA	NA		270000	Cluster 21: Neutrophils - Unknown function
	Supported	Nucleoplasm,Plasma membrane			No	No			
	Supported	Vesicles,Plasma membrane,Actin filaments,Cytosol			NA	No		11000	Cluster 34: B-cells - Unknown function
	Supported	Plasma membrane			NA	NA		1600000	
	Supported	Vesicles,Plasma membrane			NA	NA			
	Supported	Vesicles,Plasma membrane			NA	NA			
	Supported	Plasma membrane			NA	NA			Cluster 39: T-cells - T-cell receptor
Supported	Supported	Plasma membrane,Cell Junctions			NA	NA			
	Supported	Plasma membrane			NA	NA			Cluster 42: Non-specific - Vesicular transport
	Supported	Plasma membrane			NA	NA			Cluster 21: Neutrophils - Unknown function
	Supported	Plasma membrane,Cytosol			NA	NA		5100	Cluster 23: B-cells - Immunoglobulins
	Enhanced	Plasma membrane			NA	NA			Cluster 45: Eosinophils - Unknown function
	Approved	Plasma membrane,Cell Junctions,Actin filaments			NA	NA			Cluster 31: T-cells - T-cell receptor
	Supported	Nucleoplasm,Plasma membrane,Cytosol			NA	NA			Cluster 2: Dendritic cells - Unknown function
	Supported	Plasma membrane,Cytosol			NA	NA		610000	Cluster 43: Non-specific - Transcription & Translation
	Enhanced	Plasma membrane,Cytosol			NA	NA			Cluster 33: Non-specific - ATP binding
	Supported	Nucleoplasm,Plasma membrane			NA	NA			Cluster 37: Monocytes & Neutrophils - Innate immune
	Supported	Plasma membrane			NA	NA			
	Supported	Plasma membrane,Cytosol			NA	NA			Cluster 22: Eosinophils - Transcription
	Supported	Nucleoplasm,Plasma membrane,Cytosol			NA	NA			
	Supported	Nucleoplasm,Plasma membrane,Cytosol			NA	NA			Cluster 43: Non-specific - Transcription & Translation
	Supported	Plasma membrane,Cytosol			NA	NA			Cluster 3: Non-specific - Nuclear processes
	Enhanced	Plasma membrane,Cytosol			NA	NA			Cluster 10: Non-specific - Membrane trafficking
	Supported	Nucleoplasm,Plasma membrane,Cytosol			NA	NA			Cluster 25: Non-specific - Unknown function
	Supported	Vesicles,Plasma membrane			NA	NA			Cluster 29: Non-specific - Unknown function
	Enhanced	Plasma membrane,Cell Junctions			NA	NA			
	Supported	Plasma membrane,Cytosol			NA	NA		100000	Cluster 41: Basophils - Unknown function
	Supported	Endoplasmic reticulum,Plasma membrane			NA	NA		23000000	Cluster 26: Monocytes - Plasma membrane proteins
	Supported	Nucleoplasm,Plasma membrane			Yes	No			Cluster 37: Monocytes & Neutrophils - Innate immune
	Supported	Golgi apparatus,Plasma membrane			NA	NA			
	Supported	Plasma membrane,Cytosol			NA	NA		90000	Cluster 40: Monocytes - Innate immune response
	Supported	Nucleoplasm,Plasma membrane			NA	NA			Cluster 32: Basophils - Transcription
	Approved	Plasma membrane,Actin filaments,Cytosol			NA	NA			
	Supported	Plasma membrane			NA	NA		52000	Cluster 33: Non-specific - ATP binding
	Enhanced	Vesicles,Plasma membrane,Cell Junctions,Cytosol			NA	NA			Cluster 20: Non-specific - DNA binding
Supported	Supported	Plasma membrane			NA	NA			Cluster 2: Dendritic cells - Unknown function
Supported	Approved	Nucleoplasm,Plasma membrane			NA	NA			
	Supported	Plasma membrane,Primary cilium,Mitochondria,Basal body			NA	NA		58000	Cluster 33: Non-specific - ATP binding
	Supported	Vesicles,Plasma membrane,Focal adhesion sites,Connecting piece,Mid piece,Principal piece,End piece			NA	NA			Cluster 22: Eosinophils - Transcription
	Approved	Nucleoplasm,Plasma membrane			NA	No		11000	Cluster 37: Monocytes & Neutrophils - Innate immune
	Supported	Nucleoplasm,Plasma membrane,Acrosome,Mid piece,Annulus			NA	NA			
	Supported	Nucleoplasm,Nuclear bodies,Plasma membrane,Cytosol			NA	NA			
	Supported	Nucleoli,Plasma membrane,Cytosol			NA	NA			Cluster 3: Non-specific - Nuclear processes
	Supported	Vesicles,Plasma membrane,Actin filaments			NA	Yes		6700000	Cluster 8: Plasmacytoid DCs - Unknown function
	Enhanced	Golgi apparatus,Plasma membrane,Cytosol	Secreted to blood	Immunity	NA	NA	1790000000	1300000000	Cluster 21: Neutrophils - Unknown function
	Supported	Plasma membrane			NA	NA			
	Enhanced	Plasma membrane,Cytosol			NA	NA		7900	Cluster 38: Monocytes & Neutrophils - Degranulation
	Enhanced	Plasma membrane,Cytosol			NA	NA			Cluster 8: Plasmacytoid DCs - Unknown function
	Supported	Plasma membrane			NA	NA		49000000	Cluster 21: Neutrophils - Unknown function
	Supported	Vesicles,Plasma membrane,Cytokinetic bridge			Yes	NA			
	Supported	Plasma membrane,Cytosol			No	No			Cluster 1: T-cells - Unknown function
	Enhanced	Plasma membrane			NA	NA		20000000	Cluster 35: Neutrophils - Protein binding
	Supported	Vesicles,Plasma membrane			NA	NA			Cluster 12: Non-specific - Mitochondrial translation
	Supported	Nucleoplasm,Plasma membrane			NA	NA			
	Supported	Nucleoplasm,Plasma membrane			NA	NA			Cluster 35: Neutrophils - Protein binding
	Supported	Nucleoplasm,Nuclear bodies,Plasma membrane	Intracellular and membrane	Cell adhesion	NA	No		1500000	
	Supported	Vesicles,Plasma membrane,Primary cilium,Centriolar satellite,Basal body,Cytosol,Mid piece,Principal piece,End piece			No	No			Cluster 20: Non-specific - DNA binding
	Supported	Plasma membrane,Cytosol			NA	NA		1400000	Cluster 32: Basophils - Transcription
	Supported	Plasma membrane,Cell Junctions			Yes	NA			
	Enhanced	Cell Junctions			NA	NA			
	Enhanced	Plasma membrane,Cytosol			NA	NA		62000	
	Supported	Plasma membrane,Mitochondria	Secreted to blood	Complement pathway	NA	NA		1500000	Cluster 13: Non-specific - Mitochondria
	Supported	Plasma membrane			NA	NA			Cluster 36: Eosinophils - Protein ubiquitination
	Supported	Plasma membrane	Secreted to blood	Complement pathway	NA	NA		15000000000	

	Supported	Plasma membrane			NA	NA			
Approved	Approved	Nucleoplasm,Plasma membrane,Primary cilium			NA	NA			
	Supported	Plasma membrane			NA	NA		35000000	Cluster 33: Non-specific - ATP binding
	Supported	Plasma membrane			NA	NA			Cluster 2: Dendritic cells - Unknown function
	Enhanced	Plasma membrane,Actin filaments			Yes	No		46000000	Cluster 4: Neutrophils - Degranulation
	Supported	Nuclear speckles,Plasma membrane,Cytosol			NA	NA			Cluster 30: Non-specific - DNA binding
	Supported	Plasma membrane			NA	NA			
	Enhanced	Endoplasmic reticulum,Plasma membrane,Cytosol			NA	NA		7900000	Cluster 38: Monocytes & Neutrophils - Degranulation
	Supported	Vesicles,Plasma membrane			NA	NA		300000	
	Enhanced	Plasma membrane,Cytosol			Yes	No		110000000	Cluster 21: Neutrophils - Unknown function
	Supported	Nucleoplasm,Golgi apparatus,Plasma membrane			NA	NA			
	Supported	Vesicles,Plasma membrane			NA	NA			Cluster 25: Non-specific - Unknown function
	Enhanced	Plasma membrane,Cytosol			NA	NA			Cluster 29: Non-specific - Unknown function
	Approved	Nuclear speckles,Cell Junctions			NA	No			
	Supported	Nucleoplasm,Plasma membrane,Centrosome,Basal body			NA	NA			Cluster 2: Dendritic cells - Unknown function
	Enhanced	Cell Junctions			NA	NA			Cluster 33: Non-specific - ATP binding
	Enhanced	Vesicles,Plasma membrane			NA	NA			Cluster 24: Non-specific - Transcription
	Approved	Nucleoplasm,Plasma membrane,Cytosol			NA	NA			
	Uncertain	Vesicles,Plasma membrane			NA	NA			
	Supported	Plasma membrane			Yes	No			Cluster 20: Non-specific - DNA binding
	Supported	Nucleoli fibrillar center,Plasma membrane			NA	NA			Cluster 41: Basophils - Unknown function
	Supported	Plasma membrane			NA	NA			Cluster 9: T-regs - Cell cycle regulation
	Supported	Plasma membrane,Primary cilium,Primary cilium tip			NA	NA			Cluster 37: Monocytes & Neutrophils - Innate immune
	Supported	Plasma membrane,Cytosol			NA	NA		130000000	
	Supported	Plasma membrane	Secreted to blood	Acute phase	NA	NA	903000000	340000000	Cluster 26: Monocytes - Plasma membrane proteins
	Approved	Nucleoplasm,Nucleoli,Plasma membrane,Mitotic spindle			Yes	NA			Cluster 23: B-cells - Immunoglobulins
	Supported	Nucleoplasm,Golgi apparatus,Plasma membrane			NA	NA			Cluster 1: T-cells - Unknown function
	Supported	Plasma membrane	Intracellular and membrane	Receptor	NA	NA		110000	Cluster 46: Basophils - Proteolysis
	Supported	Nucleoplasm,Plasma membrane,Centriolar satellite			NA	No		830000	Cluster 15: NK-cells - Unknown function
	Supported	Nucleoplasm,Nuclear speckles,Plasma membrane	Secreted - unknown location	Receptor	NA	NA			Cluster 35: Neutrophils - Protein binding
	Supported	Plasma membrane			NA	NA			Cluster 31: T-cells - T-cell receptor
	Supported	Plasma membrane,Centriolar satellite			NA	NA		21000	Cluster 8: Plasmacytoid DCs - Unknown function
	Supported	Plasma membrane,Cytosol			NA	NA		730000	Cluster 26: Monocytes - Plasma membrane proteins
	Supported	Plasma membrane			NA	NA			Cluster 14: Non-specific - Unknown function
	Supported	Endoplasmic reticulum,Golgi apparatus,Plasma membrane			NA	NA			Cluster 11: T-cells - Unknown function
	Supported	Plasma membrane,Cytosol			NA	NA			Cluster 11: T-cells - Unknown function
	Supported	Plasma membrane			NA	NA			Cluster 2: Dendritic cells - Unknown function
	Approved	Vesicles,Plasma membrane	Secreted to blood	Receptor	NA	NA	705000		Cluster 23: B-cells - Immunoglobulins
	Approved	Golgi apparatus,Plasma membrane	Secreted to blood	Cytokine	NA	NA	776000	14000	Cluster 11: T-cells - Unknown function
	Supported	Golgi apparatus,Plasma membrane	Secreted to blood	Receptor	NA	NA	260000000	200000000	Cluster 38: Monocytes & Neutrophils - Degranulation
	Supported	Vesicles,Plasma membrane			NA	NA		210000	Cluster 12: Non-specific - Mitochondrial translation
	Supported	Nucleoplasm,Plasma membrane,Cytosol			NA	NA			Cluster 23: B-cells - Immunoglobulins
	Enhanced	Vesicles,Plasma membrane			NA	NA		3400000	Cluster 42: Non-specific - Vesicular transport
	Supported	Plasma membrane,Centriolar satellite			NA	NA		130000	Cluster 38: Monocytes & Neutrophils - Degranulation
	Supported	Plasma membrane	Intracellular and membrane	Immunity	NA	NA		72000	Cluster 11: T-cells - Unknown function
	Supported	Plasma membrane	Intracellular and membrane	Immunity	NA	NA			Cluster 43: Non-specific - Transcription & Translation
	Supported	Plasma membrane	Intracellular and membrane	Cell adhesion	NA	NA		4200000	Cluster 41: Basophils - Unknown function
	Enhanced	Plasma membrane,Cytosol			NA	NA			Cluster 36: Eosinophils - Protein ubiquitination
	Supported	Nucleoplasm,Plasma membrane,Actin filaments			NA	NA			Cluster 38: Monocytes & Neutrophils - Degranulation
	Supported	Plasma membrane,Actin filaments			NA	NA			Cluster 28: Neutrophils - Unknown function
	Supported	Cell Junctions			NA	NA			Cluster 35: Neutrophils - Protein binding
	Supported	Golgi apparatus,Plasma membrane,Cell Junctions			NA	NA		130000000	Cluster 13: Non-specific - Mitochondria
	Supported	Plasma membrane	Intracellular and membrane	Cell adhesion	NA	NA		160000000	Cluster 40: Monocytes - Innate immune response
Supported	Supported	Golgi apparatus,Plasma membrane			NA	NA		370000	
	Supported	Nucleoplasm,Cell Junctions			NA	NA		4700000	Cluster 35: Neutrophils - Protein binding
	Supported	Plasma membrane,Cell Junctions			NA	NA		71000000	Cluster 35: Neutrophils - Protein binding
	Supported	Plasma membrane,Microtubules			NA	NA			
	Supported	Plasma membrane,Cell Junctions			NA	NA		1800000	
	Supported	Plasma membrane			NA	NA		59000	
	Supported	Nucleoplasm,Nuclear membrane,Plasma membrane			NA	NA		960000000	
	Supported	Plasma membrane,Cell Junctions			NA	NA		16000000	
	Supported	Plasma membrane,Microtubules,Cytosol			NA	NA			Cluster 10: Non-specific - Membrane trafficking
	Supported	Nucleoplasm,Plasma membrane,Cell Junctions			NA	NA			Cluster 10: Non-specific - Membrane trafficking
	Supported	Plasma membrane			NA	NA		400000	
	Supported	Plasma membrane,Cytosol			NA	NA			
	Enhanced	Nucleoplasm,Plasma membrane	Intracellular and membrane	Receptor	NA	NA		260000	Cluster 34: B-cells - Unknown function
	Supported	Vesicles,Plasma membrane,Cytosol			NA	NA		130000	Cluster 43: Non-specific - Transcription & Translation
	Supported	Nucleoplasm,Nuclear bodies,Plasma membrane,Cytosol			NA	Yes			Cluster 35: Neutrophils - Protein binding
	Supported	Plasma membrane,Cell Junctions			NA	NA			
	Supported	Nuclear bodies,Cell Junctions			No	No			
	Enhanced	Vesicles,Plasma membrane			NA	Yes			Cluster 35: Neutrophils - Protein binding
	Supported	Nucleoli,Vesicles,Plasma membrane,Primary cilium tip			NA	NA			
	Uncertain	Nucleoplasm,Golgi apparatus,Plasma membrane,Primary cilium,Primary cilium tip			NA	NA			
	Supported	Plasma membrane			NA	NA			
	Supported	Nucleoplasm,Plasma membrane,Cytokinetic bridge			Yes	NA			
	Approved	Nuclear speckles,Plasma membrane,Primary cilium tip,Primary cilium transition zone,Basal body,Cytosol			NA	NA			
	Approved	Plasma membrane,Focal adhesion sites			NA	NA			
	Approved	Endoplasmic reticulum,Cell Junctions			NA	NA			
	Supported	Plasma membrane			NA	NA			
	Supported	Nucleoplasm,Nuclear bodies,Plasma membrane			NA	NA			Cluster 25: Non-specific - Unknown function
	Supported	Nucleoplasm,Plasma membrane,Cell Junctions	Intracellular and membrane	Transport	NA	NA		54000	
	Supported	Golgi apparatus,Plasma membrane,Cytosol			NA	NA			Cluster 8: Plasmacytoid DCs - Unknown function
	Approved	Lipid droplets,Cell Junctions			NA	NA			
	Supported	Plasma membrane			NA	NA			
	Supported	Nucleoplasm,Nucleoli,Vesicles,Plasma membrane,Cell Junctions			NA	NA			
	Approved	Golgi apparatus,Plasma membrane,Cytosol			NA	NA			
	Supported	Cell Junctions			NA	NA			
	Supported	Plasma membrane			NA	NA			
	Approved	Vesicles,Cell Junctions			NA	NA			Cluster 24: Non-specific - Transcription
	Supported	Vesicles,Cell Junctions			NA	NA			
	Supported	Plasma membrane,Cytosol			NA	NA			Cluster 38: Monocytes & Neutrophils - Degranulation
	Supported	Nucleoplasm,Plasma membrane,Cytosol			NA	NA		49000	Cluster 26: Monocytes - Plasma membrane proteins
	Supported	Plasma membrane,Centrosome,Calyx,Connecting piece,Principal piece			NA	NA		3800000	Cluster 23: B-cells - Immunoglobulins
	Supported	Nuclear speckles,Plasma membrane			NA	NA		750000	Cluster 1: T-cells - Unknown function
	Supported	Golgi apparatus,Endosomes,Lysosomes,Plasma membrane,Mitotic spindle,Primary cilium			Yes	NA		630000	Cluster 10: Non-specific - Membrane trafficking
	Supported	Vesicles,Plasma membrane,Cytosol			NA	NA		12000	Cluster 45: Eosinophils - Unknown function
	Supported	Nucleoplasm,Vesicles,Plasma membrane			NA	NA			
	Uncertain	Nucleoplasm,Vesicles,Plasma membrane,Mitotic spindle,Primary cilium,Primary cilium tip,Mid piece,Principal piece			Yes	NA			
	Uncertain	Plasma membrane,Actin filaments			NA	NA			Cluster 23: B-cells - Immunoglobulins
Supported	Supported	Plasma membrane,Actin filaments			NA	NA			
	Supported	Nucleoplasm,Golgi apparatus,Plasma membrane,Cytosol			NA	NA			Cluster 41: Basophils - Unknown function
	Supported	Golgi apparatus,Plasma membrane	Secreted to extracellular matrix	No annotated function	NA	NA		24000	
	Uncertain	Plasma membrane,Cytosol	Secreted to extracellular matrix	Cell adhesion	NA	NA		8700000	Cluster 1: T-cells - Unknown function
	Supported	Plasma membrane,Cytosol			NA	NA		1000000	Cluster 40: Monocytes - Innate immune response
	Supported	Plasma membrane,Cytosol			No	No		580000	Cluster 37: Monocytes & Neutrophils - Innate immune
	Supported	Plasma membrane			NA	NA		19000000	Cluster 20: Non-specific - DNA binding
	Supported	Cell Junctions			NA	NA			Cluster 43: Non-specific - Transcription & Translation
	Supported	Plasma membrane	Secreted - unknown location	Receptor	NA	NA			
	Enhanced	Plasma membrane,Cytosol	Intracellular and membrane	Other	NA	NA		510000	
	Supported	Nuclear bodies,Plasma membrane	Secreted to blood	Cytokine	NA	NA	270000	8800000	Cluster 19: Eosinophils - Unknown function
	Supported	Vesicles,Plasma membrane	Intracellular and membrane	Receptor	NA	NA		220000000	Cluster 40: Monocytes - Innate immune response
	Supported	Plasma membrane,Actin filaments,Primary cilium,Cytosol,Principal piece,End piece			NA	NA			Cluster 7: Non-specific - Innate immune response
	Supported	Golgi apparatus,Vesicles,Plasma membrane,Cell Junctions,Connecting piece,Mid piece,Principal piece			NA	NA		41000	Cluster 38: Monocytes & Neutrophils - Degranulation
	Enhanced	Plasma membrane,Cell Junctions,Primary cilium,Basal body			NA	NA		3200000	Cluster 33: Non-specific - ATP binding
	Enhanced	Plasma membrane,Basal body			NA	NA		20000	Cluster 38: Monocytes & Neutrophils - Degranulation
	Supported	Golgi apparatus,Vesicles,Plasma membrane			NA	NA		19000000	Cluster 35: Neutrophils - Protein binding
	Supported	Plasma membrane	Secreted to blood	Chemokine	NA	NA	1870000		
	Supported	Plasma membrane			NA	NA			Cluster 37: Monocytes & Neutrophils - Innate immune
	Supported	Nucleoplasm,Plasma membrane,Cell Junctions	Secreted - unknown location	Receptor	NA	NA		12000	
	Approved	Vesicles,Plasma membrane,Microtubules,Mitotic spindle,Primary cilium,Primary cilium tip,Basal body			Yes	NA			Cluster 35: Neutrophils - Protein binding
	Supported	Plasma membrane			NA	NA			Cluster 38: Monocytes & Neutrophils - Degranulation
	Supported	Golgi apparatus,Plasma membrane,Cytosol			NA	NA			Cluster 38: Monocytes & Neutrophils - Degranulation
	Supported	Plasma membrane,Cytokinetic bridge,Centriolar satellite,Cytosol			Yes	NA			Cluster 38: Monocytes & Neutrophils - Degranulation
	Enhanced	Nucleoli fibrillar center,Vesicles,Plasma membrane			NA	NA		53000	Cluster 8: Plasmacytoid DCs - Unknown function
	Supported	Nucleoplasm,Vesicles,Plasma membrane	Intracellular and membrane	Receptor	NA	NA		42000000	Cluster 29: Non-specific - Unknown function
	Supported	Nucleoplasm,Plasma membrane			No	No			Cluster 29: Non-specific - Unknown function
	Supported	Vesicles,Plasma membrane			NA	NA		6400000	Cluster 10: Non-specific - Membrane trafficking
	Supported	Vesicles,Plasma membrane			NA	NA			
	Supported	Nucleoplasm,Plasma membrane,Cytosol	Intracellular and membrane	Receptor	NA	NA		15000000	

	Approved	Plasma membrane,Actin filaments			Yes	No		490000	
	Supported	Vesicles,Plasma membrane			NA	NA			
	Supported	Plasma membrane,Microtubules			NA	Yes			
	Uncertain	Nucleoplasm,Plasma membrane,Actin filaments,Focal adhesion sites,Mitochondria			NA	NA			Cluster 3: Non-specific - Nuclear processes
	Approved	Vesicles,Plasma membrane,Basal body			NA	NA		9500	Cluster 24: Non-specific - Transcription
	Uncertain	Nucleoplasm,Plasma membrane,Primary cilium,Primary cilium tip,Centrosome,Basal body,Cytosol			NA	NA			
	Supported	Cell Junctions			NA	NA			
	Enhanced	Nucleoplasm,Plasma membrane,Cytosol			NA	NA		8100	Cluster 30: Non-specific - DNA binding
	Supported	Nucleoplasm,Plasma membrane,Cytosol			NA	NA		89000	Cluster 5: Non-specific - Cell proliferation
	Approved	Nucleoli,Golgi apparatus,Plasma membrane,Cytosol			NA	NA			Cluster 28: Neutrophils - Unknown function
	Supported	Plasma membrane,Cytosol			NA	NA			Cluster 38: Monocytes & Neutrophils - Degranulation
	Enhanced	Plasma membrane			NA	NA		130000	Cluster 25: Non-specific - Unknown function
	Supported	Nucleoplasm,Plasma membrane,Cytokinetic bridge,Cytosol			Yes	NA		130000	Cluster 37: Monocytes & Neutrophils - Innate immune
	Approved	Nucleoplasm,Cell Junctions	Intracellular and membrane	Enzyme	NA	NA		240000	
	Uncertain	Plasma membrane,Centrosome			NA	NA			Cluster 44: Plasmacytoid DCs - Plasma membrane pr
	Supported	Endoplasmic reticulum,Plasma membrane			NA	NA		18000000	Cluster 36: Eosinophils - Protein ubiquitination
	Enhanced	Plasma membrane,Cell Junctions			NA	NA		8900000	
	Supported	Vesicles,Plasma membrane,Cell Junctions			NA	NA		94000000	Cluster 41: Basophils - Unknown function
	Supported	Cell Junctions			NA	NA		130000000	Cluster 34: B-cells - Unknown function
	Approved	Nucleoplasm,Cell Junctions,Intermediate filaments			NA	NA			Cluster 33: Non-specific - ATP binding
	Approved	Plasma membrane,Centriolar satellite,Mid piece			Yes	No			Cluster 21: Neutrophils - Unknown function
	Supported	Nucleoplasm,Vesicles,Plasma membrane			NA	NA			
	Approved	Plasma membrane,Cytosol			NA	NA			
	Supported	Plasma membrane,Cytosol			NA	NA			
	Supported	Plasma membrane			NA	NA		990000	Cluster 37: Monocytes & Neutrophils - Innate immune
	Supported	Plasma membrane,Cytosol			NA	NA			Cluster 33: Non-specific - ATP binding
	Supported	Plasma membrane,Actin filaments,Cytosol			NA	NA			Cluster 32: Basophils - Transcription
	Supported	Golgi apparatus,Plasma membrane,Cell Junctions,Primary cilium,Basal body,Mid piece,Principal piece,End piece	Secreted to blood	Receptor	NA	NA	4740000	19000000	
	Enhanced	Plasma membrane,Cytosol			NA	NA			Cluster 33: Non-specific - ATP binding
	Supported	Plasma membrane,Primary cilium,Primary cilium transition zone			NA	NA		6100000	Cluster 7: Non-specific - Innate immune response
	Supported	Plasma membrane,Primary cilium,Primary cilium transition zone			NA	NA		740000	
	Supported	Plasma membrane,Primary cilium,Primary cilium transition zone			NA	NA		8800000	Cluster 23: B-cells - Immunoglobulins
	Supported	Plasma membrane			NA	NA			Cluster 4: Neutrophils - Degranulation
	Enhanced	Plasma membrane,Focal adhesion sites,Cytosol			NA	NA			
	Supported	Plasma membrane			NA	NA		43000000	Cluster 40: Monocytes - Innate immune response
	Enhanced	Plasma membrane,Cytosol			NA	NA		280000000	Cluster 33: Non-specific - ATP binding
Supported	Enhanced	Plasma membrane,Cytosol			NA	NA		47000000	Cluster 43: Non-specific - Transcription & Translation
	Enhanced	Plasma membrane,Cytosol			Yes	No		65000000	Cluster 29: Non-specific - Unknown function
	Supported	Plasma membrane	Intracellular and membrane	Transport	NA	NA			
	Approved	Plasma membrane,Cytosol	Intracellular and membrane	Transport	NA	NA			Cluster 41: Basophils - Unknown function
	Supported	Plasma membrane			NA	NA			Cluster 44: Plasmacytoid DCs - Plasma membrane pr
	Enhanced	Nucleoplasm,Plasma membrane,Cell Junctions			NA	NA		160000	Cluster 23: B-cells - Immunoglobulins
	Supported	Plasma membrane,Cell Junctions,Primary cilium,Basal body			NA	NA		380000	Cluster 40: Monocytes - Innate immune response
	Enhanced	Plasma membrane			NA	NA			Cluster 43: Non-specific - Transcription & Translation
	Supported	Plasma membrane			NA	NA		8800	
	Supported	Nuclear speckles,Golgi apparatus,Plasma membrane,Cell Junctions			NA	Yes			Cluster 44: Plasmacytoid DCs - Plasma membrane pr
	Supported	Nucleoplasm,Nuclear membrane,Plasma membrane,Actin filaments,Cytosol	Intracellular and membrane	Receptor	NA	NA			
	Approved	Golgi apparatus,Vesicles,Plasma membrane			NA	NA		98000	
	Supported	Endoplasmic reticulum,Plasma membrane,Cytosol			NA	NA			Cluster 2: Dendritic cells - Unknown function
	Supported	Nucleoplasm,Plasma membrane	Intracellular and membrane	Receptor	No	No		520000	
	Supported	Nuclear speckles,Plasma membrane	Intracellular and membrane	Receptor	NA	Yes			Cluster 35: Neutrophils - Protein binding
	Enhanced	Nucleoplasm,Plasma membrane,Cytosol			NA	NA		360000	Cluster 24: Non-specific - Transcription
	Supported	Plasma membrane,Actin filaments	Secreted - unknown location	Receptor	NA	NA		240000	
	Supported	Plasma membrane,Cell Junctions			NA	NA		16000	Cluster 35: Neutrophils - Protein binding
	Approved	Plasma membrane,Cytosol			NA	NA		6300	Cluster 1: T-cells - Unknown function
	Supported	Golgi apparatus,Vesicles,Plasma membrane,Primary cilium,Primary cilium transition zone,Cytosol			NA	NA			Cluster 21: Neutrophils - Unknown function
	Supported	Plasma membrane			NA	NA		33000000	Cluster 16: Non-specific - Cell proliferation
	Supported	Cell Junctions,Microtubules			NA	NA		1800000	Cluster 21: Neutrophils - Unknown function
	Supported	Plasma membrane			NA	NA			Cluster 20: Non-specific - DNA binding
	Supported	Nuclear bodies,Plasma membrane,Cytosol	Secreted to blood	Receptor	NA	NA	11900000	560000	Cluster 35: Neutrophils - Protein binding
	Enhanced	Plasma membrane,Cytosol			No	No		3300000	
	Enhanced	Nucleoli fibrillar center,Cell Junctions,Focal adhesion sites			NA	NA			Cluster 20: Non-specific - DNA binding
	Approved	Nucleoplasm,Nucleoli,Plasma membrane			NA	NA			
	Supported	Plasma membrane	Intracellular and membrane	Receptor	NA	NA			Cluster 21: Neutrophils - Unknown function
	Uncertain	Golgi apparatus,Vesicles,Plasma membrane			NA	NA			Cluster 38: Monocytes & Neutrophils - Degranulation
	Supported	Golgi apparatus,Plasma membrane			NA	NA		34000000	Cluster 21: Neutrophils - Unknown function
	Approved	Nuclear speckles,Plasma membrane,Actin filaments,Focal adhesion sites			Yes	No			Cluster 32: Basophils - Transcription
	Approved	Nucleoplasm,Golgi apparatus,Plasma membrane,Centrosome	Secreted to blood	Receptor	NA	NA		2500000	Cluster 43: Non-specific - Transcription & Translation
	Supported	Plasma membrane			NA	NA			
	Supported	Vesicles,Plasma membrane,Basal body			NA	NA			Cluster 38: Monocytes & Neutrophils - Degranulation
	Enhanced	Plasma membrane,Cytosol			NA	NA			
	Supported	Plasma membrane			NA	NA			
	Uncertain	Plasma membrane,Cytosol			NA	NA		11000000	Cluster 5: Non-specific - Cell proliferation
	Supported	Golgi apparatus,Plasma membrane,Actin filaments,Cytosol			NA	NA		19000000	Cluster 5: Non-specific - Cell proliferation
	Uncertain	Golgi apparatus,Vesicles,Plasma membrane			NA	NA		58000	Cluster 35: Neutrophils - Protein binding
	Supported	Vesicles,Plasma membrane			NA	NA		140000	Cluster 21: Neutrophils - Unknown function
	Supported	Vesicles,Plasma membrane,Cell Junctions	Intracellular and membrane	Developmental protei	NA	NA		210000	
	Supported	Plasma membrane,Actin filaments,Cytosol	Secreted to blood	Receptor	NA	NA	876000	300000	
	Supported	Plasma membrane,Centrosome,Cytosol	Secreted to blood	Receptor	NA	NA	20000000	43000000	Cluster 1: T-cells - Unknown function
	Supported	Golgi apparatus,Vesicles,Plasma membrane,Cytosol			NA	NA			Cluster 12: Non-specific - Mitochondrial translation
	Supported	Vesicles,Plasma membrane			NA	NA			Cluster 42: Non-specific - Vesicular transport
	Approved	Plasma membrane,Primary cilium			NA	NA			Cluster 10: Non-specific - Membrane trafficking
	Approved	Nucleoplasm,Plasma membrane,Centriolar satellite,Cytosol			NA	NA			Cluster 7: Non-specific - Innate immune response
	Supported	Plasma membrane,Cytosol			NA	NA		290000	Cluster 1: T-cells - Unknown function
	Supported	Plasma membrane			NA	NA			Cluster 40: Monocytes - Innate immune response
	Supported	Nucleoplasm,Plasma membrane,Primary cilium			NA	NA			
	Supported	Plasma membrane,Primary cilium,Basal body			NA	NA			Cluster 14: Non-specific - Unknown function
	Supported	Plasma membrane			NA	NA			Cluster 35: Neutrophils - Protein binding
	Approved	Vesicles,Plasma membrane,Primary cilium tip,Basal body	Intracellular and membrane	Receptor	NA	NA			
Supported	Supported	Nucleoplasm,Plasma membrane			NA	NA			
Supported	Supported	Nucleoplasm,Plasma membrane,Actin filaments,Cytosol			NA	NA			
	Supported	Vesicles,Plasma membrane			NA	NA			
	Approved	Nucleoli,Plasma membrane,Microtubules			NA	NA			
	Supported	Plasma membrane			NA	NA			
Supported	Enhanced	Plasma membrane			NA	NA		29000	
	Enhanced	Nuclear membrane,Vesicles,Plasma membrane,Cytosol			No	No		810000000	Cluster 26: Monocytes - Plasma membrane proteins
	Supported	Golgi apparatus,Vesicles,Plasma membrane			NA	NA			Cluster 29: Non-specific - Unknown function
	Supported	Plasma membrane,Cytosol			NA	NA		40000	Cluster 35: Neutrophils - Protein binding
	Supported	Golgi apparatus,Vesicles,Plasma membrane,Primary cilium,Cytosol,Mid piece,Principal piece,End piece			NA	NA			Cluster 10: Non-specific - Membrane trafficking
	Supported	Plasma membrane,Cytosol			NA	NA		120000	
	Approved	Nucleoplasm,Plasma membrane,Actin filaments,Focal adhesion sites			NA	NA			
	Supported	Plasma membrane,Cytosol,Cytoplasmic bodies	Secreted to blood	Receptor	NA	NA	2200000	3600000	
	Supported	Nucleoplasm,Cell Junctions			NA	NA			Cluster 21: Neutrophils - Unknown function
Supported	Approved	Nucleoplasm,Vesicles,Cell Junctions			NA	NA			
	Supported	Vesicles,Cell Junctions			NA	NA			
	Supported	Cell Junctions			NA	NA			
	Supported	Cell Junctions			NA	NA			Cluster 43: Non-specific - Transcription & Translation
	Supported	Nucleoplasm,Endoplasmic reticulum,Plasma membrane			NA	NA			
	Supported	Plasma membrane,Cytosol			NA	NA			
	Supported	Vesicles,Plasma membrane	Secreted to extracellular matrix	Developmental protei	NA	NA		400000	
	Supported	Nucleoplasm,Plasma membrane,Cytosol			No	No		2000000	Cluster 8: Plasmacytoid DCs - Unknown function
	Approved	Nuclear bodies,Golgi apparatus,Plasma membrane,Centrosome			NA	NA			
	Supported	Plasma membrane			NA	NA			
Supported	Approved	Vesicles,Plasma membrane			NA	NA			
	Approved	Vesicles,Plasma membrane			NA	NA			
	Approved	Vesicles,Plasma membrane			NA	NA			
	Supported	Nucleoplasm,Plasma membrane,Basal body,Cytosol			NA	NA		7300000	Cluster 7: Non-specific - Innate immune response
	Approved	Nuclear speckles,Plasma membrane,Cytosol			NA	NA		460000	Cluster 41: Basophils - Unknown function
	Uncertain	Nucleoplasm,Plasma membrane,Cytosol			NA	NA			Cluster 10: Non-specific - Membrane trafficking
	Supported	Plasma membrane			NA	NA		3200000	Cluster 21: Neutrophils - Unknown function
	Supported	Plasma membrane	Intracellular and membrane	Blood coagulation	NA	NA		8300000	Cluster 35: Neutrophils - Protein binding
	Supported	Plasma membrane,Cytosol	Intracellular and membrane	No annotated function	NA	NA		5400000	
	Supported	Plasma membrane			NA	NA		89000	Cluster 43: Non-specific - Transcription & Translation
	Supported	Nucleoplasm,Nuclear bodies,Golgi apparatus,Plasma membrane			NA	NA			
	Supported	Plasma membrane			NA	NA			
	Supported	Plasma membrane			NA	NA			
	Supported	Plasma membrane			NA	NA			Cluster 24: Non-specific - Transcription
	Approved	Vesicles,Plasma membrane,Centriolar satellite			NA	NA			Cluster 1: T-cells - Unknown function
	Supported	Nucleoplasm,Plasma membrane,Primary cilium,Primary cilium tip			NA	NA			Cluster 5: Non-specific - Cell proliferation

	Supported	Plasma membrane			NA	NA			Cluster 40: Monocytes - Innate immune response
	Approved	Nucleoplasm,Vesicles,Plasma membrane,Cell Junctions			NA	NA			
	Supported	Nucleoplasm,Plasma membrane,Primary cilium,Primary cilium tip			NA	NA			
	Supported	Vesicles,Plasma membrane,Primary cilium			NA	NA			
	Supported	Vesicles,Plasma membrane,Primary cilium,Primary cilium tip			NA	Yes			
	Supported	Nucleoplasm,Vesicles,Plasma membrane,Primary cilium,Primary cilium transition zone			NA	Yes		98000	Cluster 44: Plasmacytoid DCs - Plasma membrane pr
	Supported	Vesicles,Plasma membrane			NA	NA			
	Supported	Plasma membrane,Cytosol			NA	NA			Cluster 28: Neutrophils - Unknown function
	Supported	Endoplasmic reticulum,Vesicles,Plasma membrane			NA	NA			Cluster 8: Plasmacytoid DCs - Unknown function
	Supported	Plasma membrane,Cytosol			NA	NA			
	Supported	Vesicles,Plasma membrane			NA	NA			
	Supported	Nucleoplasm,Plasma membrane			NA	NA			
	Supported	Nucleoplasm,Plasma membrane,Primary cilium,Primary cilium tip,Primary cilium transition zone,Basal body			NA	NA			
	Supported	Nuclear membrane,Nuclear speckles,Plasma membrane			NA	NA			Cluster 3: Non-specific - Nuclear processes
	Supported	Plasma membrane			NA	NA			
	Supported	Nucleoplasm,Nuclear bodies,Plasma membrane,Cytosol			NA	NA		1500000	
	Supported	Nucleoplasm,Plasma membrane,Primary cilium,Primary cilium tip,Centrosome,Basal body,Cytosol			Yes	Yes			Cluster 15: NK-cells - Unknown function
	Approved	Nucleoplasm,Plasma membrane,Cytosol			NA	NA		2300000	
	Supported	Plasma membrane			NA	NA		760000	Cluster 43: Non-specific - Transcription & Translation
	Enhanced	Plasma membrane,Cytosol			NA	NA			Cluster 32: Basophils - Transcription
	Supported	Nucleoplasm,Plasma membrane			NA	NA			
	Supported	Cell Junctions			NA	NA			Cluster 28: Neutrophils - Unknown function
	Supported	Vesicles,Plasma membrane,Cytosol			NA	NA		69000	Cluster 37: Monocytes & Neutrophils - Innate immune
	Supported	Plasma membrane,Cytosol			NA	NA		880000	Cluster 7: Non-specific - Innate immune response
	Supported	Nucleoplasm,Plasma membrane			NA	NA			
	Supported	Nucleoplasm,Plasma membrane			No	No			Cluster 37: Monocytes & Neutrophils - Innate immune
	Supported	Golgi apparatus,Plasma membrane			NA	NA		67000000	Cluster 21: Neutrophils - Unknown function
	Approved	Nucleoplasm,Cell Junctions			NA	NA		420000	Cluster 2: Dendritic cells - Unknown function
	Supported	Vesicles,Plasma membrane	Secreted to blood	Immunity	NA	NA		9300000	Cluster 35: Neutrophils - Protein binding
	Supported	Nucleoplasm,Plasma membrane,Cytosol			No	No		660000	Cluster 29: Non-specific - Unknown function
Supported	Supported	Plasma membrane,Cytosol,Cytoplasmic bodies			NA	NA			Cluster 38: Monocytes & Neutrophils - Degranulation
	Supported	Plasma membrane,Cytosol			NA	NA			
	Supported	Plasma membrane,Cytosol	Intracellular and membrane	Other	NA	Yes		96000000	Cluster 43: Non-specific - Transcription & Translation
	Enhanced	Plasma membrane,Cytosol			NA	NA		25000000	Cluster 9: T-regs - Cell cycle regulation
Supported	Supported	Nucleoplasm,Vesicles,Plasma membrane,Primary cilium			NA	NA			Cluster 26: Monocytes - Plasma membrane proteins
	Supported	Plasma membrane	Secreted to blood	Enzyme	NA	NA	24800000	4200000	
	Supported	Plasma membrane,Cytosol			NA	NA			Cluster 29: Non-specific - Unknown function
	Supported	Nucleoplasm,Cell Junctions			NA	NA			Cluster 35: Neutrophils - Protein binding
	Approved	Vesicles,Plasma membrane			NA	NA		32000000	Cluster 23: B-cells - Immunoglobulins
	Supported	Plasma membrane			NA	NA			Cluster 37: Monocytes & Neutrophils - Innate immune
	Approved	Golgi apparatus,Plasma membrane			NA	NA			Cluster 24: Non-specific - Transcription
	Supported	Vesicles,Plasma membrane,Cytosol			NA	NA			
	Supported	Plasma membrane			NA	NA			Cluster 38: Monocytes & Neutrophils - Degranulation
	Approved	Nucleoli,Nucleoli rim,Plasma membrane,Primary cilium			NA	NA		6800	Cluster 35: Neutrophils - Protein binding
	Approved	Nucleoplasm,Plasma membrane	Intracellular and membrane	Receptor	NA	NA			Cluster 10: Non-specific - Membrane trafficking
	Supported	Plasma membrane,Primary cilium,Cytosol			NA	NA			Cluster 33: Non-specific - ATP binding
	Supported	Vesicles,Plasma membrane,Cytosol	Secreted to blood	Receptor	NA	NA			Cluster 42: Non-specific - Vesicular transport
	Approved	Vesicles,Plasma membrane	Intracellular and membrane	Receptor	NA	NA			Cluster 5: Non-specific - Cell proliferation
	Supported	Plasma membrane,Focal adhesion sites,Cytosol	Secreted to blood	Receptor	NA	NA	19100000	6900000	Cluster 41: Basophils - Unknown function
	Supported	Nucleoplasm,Plasma membrane			NA	NA		14000	Cluster 1: T-cells - Unknown function
	Supported	Nucleoplasm,Plasma membrane,Centriolar satellite	Secreted to blood	Receptor	NA	NA	38500	10000	Cluster 34: B-cells - Unknown function
	Supported	Golgi apparatus,Plasma membrane	Secreted to blood	Receptor	NA	NA		48000000	Cluster 33: Non-specific - ATP binding
	Supported	Plasma membrane	Intracellular and membrane	Receptor	NA	NA		290000	Cluster 27: T-cells - Unknown function
	Supported	Plasma membrane,Cell Junctions			NA	NA		1900000	Cluster 38: Monocytes & Neutrophils - Degranulation
	Enhanced	Nucleoplasm,Cell Junctions,Cytosol			NA	NA			Cluster 35: Neutrophils - Protein binding
	Supported	Plasma membrane			NA	NA		47000	
	Supported	Vesicles,Plasma membrane			NA	NA			Cluster 37: Monocytes & Neutrophils - Innate immune
	Supported	Endoplasmic reticulum,Plasma membrane,Focal adhesion sites			NA	NA		69000000	Cluster 16: Non-specific - Cell proliferation
	Supported	Plasma membrane,Rods & Rings			NA	NA		5100000	Cluster 38: Monocytes & Neutrophils - Degranulation
	Supported	Nucleoplasm,Plasma membrane	Intracellular and membrane	Receptor	NA	NA		74000000	Cluster 35: Neutrophils - Protein binding
	Supported	Plasma membrane,Cell Junctions			NA	NA		160000	Cluster 30: Non-specific - DNA binding
	Supported	Nucleoplasm,Plasma membrane			NA	NA			Cluster 25: Non-specific - Unknown function
	Supported	Plasma membrane			NA	NA			Cluster 35: Neutrophils - Protein binding
	Supported	Nucleoplasm,Plasma membrane			NA	NA		32000	Cluster 24: Non-specific - Transcription
	Supported	Nucleoplasm,Plasma membrane			NA	NA		1000000	Cluster 7: Non-specific - Innate immune response
	Supported	Vesicles,Plasma membrane,Cell Junctions			NA	NA		61000000	Cluster 23: B-cells - Immunoglobulins
	Supported	Plasma membrane			NA	NA			Cluster 13: Non-specific - Mitochondria
	Supported	Nucleoplasm,Plasma membrane,Cytosol	Intracellular and membrane	Other	NA	NA			Cluster 5: Non-specific - Cell proliferation
	Supported	Plasma membrane,Cytosol	Intracellular and membrane	Enzyme	NA	NA		74000	Cluster 3: Non-specific - Nuclear processes
	Approved	Vesicles,Plasma membrane,Cytosol			NA	NA			
	Approved	Nucleoplasm,Vesicles,Plasma membrane			NA	NA			Cluster 5: Non-specific - Cell proliferation
	Supported	Plasma membrane			NA	NA		10000	Cluster 38: Monocytes & Neutrophils - Degranulation
	Supported	Nucleoplasm,Plasma membrane			NA	NA			
	Supported	Plasma membrane			NA	NA			Cluster 23: B-cells - Immunoglobulins
	Approved	Nucleoplasm,Plasma membrane,Cytosol			NA	NA			Cluster 8: Plasmacytoid DCs - Unknown function
	Approved	Plasma membrane,Cytosol			NA	NA			Cluster 14: Non-specific - Unknown function
	Enhanced	Endoplasmic reticulum,Plasma membrane,Cytosol			NA	NA			Cluster 2: Dendritic cells - Unknown function
	Approved	Nucleoplasm,Vesicles,Plasma membrane			NA	NA			
	Supported	Golgi apparatus,Plasma membrane,Cytosol			NA	NA			
	Supported	Nucleoplasm,Plasma membrane			NA	NA			
	Supported	Plasma membrane,Cytokinetic bridge			Yes	NA			
	Approved	Nucleoplasm,Golgi apparatus,Plasma membrane,Cytosol			NA	NA			
	Supported	Nucleoplasm,Plasma membrane,Cell Junctions			NA	NA			
	Supported	Vesicles,Plasma membrane			NA	NA			
Supported	Supported	Nucleoli fibrillar center,Plasma membrane			NA	NA		33000000	Cluster 32: Basophils - Transcription
	Supported	Nucleoplasm,Plasma membrane,Mitochondria,Cytosol			NA	NA			Cluster 24: Non-specific - Transcription
	Supported	Nuclear membrane,Plasma membrane	Secreted in other tissues	Enzyme	NA	NA		7300000	
	Supported	Plasma membrane			NA	NA		41000	Cluster 1: T-cells - Unknown function
	Approved	Nucleoplasm,Plasma membrane			NA	NA		13000000	
	Supported	Plasma membrane			NA	NA		220000	Cluster 29: Non-specific - Unknown function
	Supported	Endoplasmic reticulum,Vesicles,Plasma membrane			NA	NA		37000000	Cluster 7: Non-specific - Innate immune response
	Uncertain	Golgi apparatus,Vesicles,Plasma membrane			NA	NA		59000	Cluster 2: Dendritic cells - Unknown function
	Supported	Golgi apparatus,Plasma membrane			NA	NA		93000	Cluster 5: Non-specific - Cell proliferation
	Supported	Plasma membrane			NA	NA			Cluster 20: Non-specific - DNA binding
	Enhanced	Golgi apparatus,Plasma membrane,Cytosol			NA	NA			Cluster 24: Non-specific - Transcription
	Supported	Plasma membrane,Actin filaments,Cytosol			NA	NA		770000000	Cluster 38: Monocytes & Neutrophils - Degranulation
Supported	Approved	Vesicles,Plasma membrane,Primary cilium,Basal body,Cytosol	Intracellular and membrane	Receptor	NA	NA		3300000	Cluster 33: Non-specific - ATP binding
	Supported	Plasma membrane,Centriolar satellite			NA	NA			
	Supported	Plasma membrane			NA	NA		10000	
	Supported	Plasma membrane	Secreted in male reproductive system	Enzyme	NA	NA			
	Supported	Nucleoplasm,Nuclear speckles,Golgi apparatus,Vesicles,Plasma membrane			NA	NA			Cluster 35: Neutrophils - Protein binding
	Supported	Cell Junctions,Cytosol	Intracellular and membrane	Enzyme	NA	NA			
	Supported	Plasma membrane,Primary cilium			NA	NA			
	Supported	Nuclear bodies,Vesicles,Plasma membrane			NA	NA			Cluster 42: Non-specific - Vesicular transport
	Supported	Vesicles,Plasma membrane			NA	NA			Cluster 33: Non-specific - ATP binding
	Supported	Plasma membrane,Cell Junctions,Focal adhesion sites,Cytosol			NA	NA		230000	Cluster 2: Dendritic cells - Unknown function
	Supported	Plasma membrane			NA	NA			
	Supported	Nucleoli,Plasma membrane,Cytosol	Intracellular and membrane	Receptor	NA	NA		220000000	Cluster 26: Monocytes - Plasma membrane proteins
	Supported	Nucleoli fibrillar center,Plasma membrane			NA	NA			Cluster 44: Plasmacytoid DCs - Plasma membrane pr
	Enhanced	Plasma membrane,Cell Junctions			NA	NA		3500000	Cluster 43: Non-specific - Transcription & Translation
	Supported	Plasma membrane,Cytosol			NA	NA			Cluster 37: Monocytes & Neutrophils - Innate immune
	Supported	Nucleoplasm,Plasma membrane			NA	NA			Cluster 33: Non-specific - ATP binding
	Supported	Golgi apparatus,Vesicles,Plasma membrane			NA	NA		350000	Cluster 37: Monocytes & Neutrophils - Innate immune
	Supported	Plasma membrane,Cell Junctions,Actin filaments,Basal body			NA	NA		3500	Cluster 3: Non-specific - Nuclear processes
	Supported	Plasma membrane,Cytosol			NA	NA			Cluster 32: Basophils - Transcription
	Supported	Nucleoplasm,Cell Junctions			NA	NA			
	Supported	Cell Junctions			NA	NA			
	Supported	Plasma membrane,Basal body,Cytosol			NA	NA		110000	Cluster 44: Plasmacytoid DCs - Plasma membrane pr
	Supported	Nuclear speckles,Plasma membrane,Cytosol			NA	NA			Cluster 33: Non-specific - ATP binding
	Enhanced	Plasma membrane			NA	NA			
	Supported	Nuclear speckles,Plasma membrane,End piece	Secreted to blood	No annotated function	NA	NA		82000	
	Supported	Nucleoplasm,Plasma membrane,Primary cilium,Cytosol			NA	NA		8500000	Cluster 40: Monocytes - Innate immune response
	Supported	Nucleoplasm,Plasma membrane,Cytosol			NA	NA			Cluster 21: Neutrophils - Unknown function
	Supported	Cell Junctions			NA	NA			
	Supported	Nucleoplasm,Plasma membrane,Cytosol			NA	NA			
	Supported	Plasma membrane			NA	NA			
	Supported	Plasma membrane			NA	NA		200000000	
	Supported	Plasma membrane			NA	NA			Cluster 38: Monocytes & Neutrophils - Degranulation
	Supported	Nucleoplasm,Plasma membrane,Cytosol			NA	NA			

	Supported	Nucleoplasm,Vesicles,Plasma membrane,Primary cilium tip,Basal body			NA	NA			
	Supported	Plasma membrane,Cytosol			NA	NA			Cluster 42: Non-specific - Vesicular transport
	Supported	Golgi apparatus,Plasma membrane	Intracellular and membrane	Developmental protei	NA	NA		250000	
	Enhanced	Plasma membrane			NA	NA			Cluster 38: Monocytes & Neutrophils - Degranulation
	Supported	Plasma membrane	Intracellular and membrane	Cell adhesion	NA	NA		8100000	
	Supported	Plasma membrane,Midbody,Cytosol,Acrosome,Mid piece,Principal piece			Yes	NA		8400000	Cluster 40: Monocytes - Innate immune response
	Supported	Nucleoplasm,Cell Junctions	Intracellular and membrane	No annotated function	NA	NA			Cluster 41: Basophils - Unknown function
	Approved	Plasma membrane,Cytosol,Cytoplasmic bodies			NA	NA			Cluster 2: Dendritic cells - Unknown function
	Supported	Cell Junctions,Centriolar satellite,Cytosol			NA	NA			Cluster 30: Non-specific - DNA binding
	Supported	Plasma membrane,Cytosol			NA	NA		65000	Cluster 42: Non-specific - Vesicular transport
	Enhanced	Plasma membrane,Focal adhesion sites			No	No			
	Supported	Plasma membrane,Cell Junctions,Cytosol			NA	NA			Cluster 38: Monocytes & Neutrophils - Degranulation
	Approved	Plasma membrane,Cytosol			NA	NA			
	Approved	Plasma membrane,Centrosome,Basal body,Cytosol			NA	NA		470000	Cluster 35: Neutrophils - Protein binding
	Supported	Plasma membrane	Secreted to blood	Enzyme	NA	NA		5000	Cluster 35: Neutrophils - Protein binding
	Supported	Nuclear membrane,Plasma membrane			NA	NA			Cluster 35: Neutrophils - Protein binding
	Supported	Plasma membrane,Centriolar satellite			NA	NA		370000	Cluster 7: Non-specific - Innate immune response
	Supported	Nucleoplasm,Cell Junctions			NA	NA			Cluster 3: Non-specific - Nuclear processes
	Supported	Vesicles,Plasma membrane			NA	NA			Cluster 38: Monocytes & Neutrophils - Degranulation
	Supported	Plasma membrane	Intracellular and membrane	Immunity	NA	NA			Cluster 42: Non-specific - Vesicular transport
	Approved	Nucleoplasm,Nuclear membrane,Vesicles,Plasma membrane,Primary cilium transition zone			NA	NA			
	Supported	Plasma membrane			NA	NA			Cluster 23: B-cells - Immunoglobulins
	Supported	Vesicles,Plasma membrane	Intracellular and membrane	Cell adhesion	NA	NA		9400000	Cluster 40: Monocytes - Innate immune response
	Enhanced	Plasma membrane			NA	NA		130000000	Cluster 38: Monocytes & Neutrophils - Degranulation
	Supported	Plasma membrane			NA	NA			Cluster 42: Non-specific - Vesicular transport
	Supported	Plasma membrane	Secreted in other tissues	No annotated function	NA	NA		2100000	Cluster 5: Non-specific - Cell proliferation
	Enhanced	Plasma membrane,Cytosol			NA	NA		37000000	
	Enhanced	Plasma membrane,Actin filaments			NA	NA		2300000	Cluster 21: Neutrophils - Unknown function
	Supported	Nucleoli rim,Plasma membrane,Cytosol			NA	NA			Cluster 37: Monocytes & Neutrophils - Innate immune
	Enhanced	Plasma membrane			NA	NA		13000	
	Enhanced	Nuclear bodies,Plasma membrane			NA	NA		72000	Cluster 14: Non-specific - Unknown function
	Supported	Nucleoplasm,Plasma membrane			NA	NA		37000	Cluster 33: Non-specific - ATP binding
	Supported	Vesicles,Plasma membrane,Primary cilium,Primary cilium tip,Centriolar satellite,Basal body			NA	NA		62000	Cluster 40: Monocytes - Innate immune response
	Uncertain	Nucleoli,Plasma membrane,Microtubules,Cytokinetic bridge,Mitotic spindle,Primary cilium,Cytosol			Yes	NA			Cluster 32: Basophils - Transcription
	Enhanced	Plasma membrane,Cytosol			NA	NA			Cluster 43: Non-specific - Transcription & Translation
	Supported	Plasma membrane			NA	NA			Cluster 45: Eosinophils - Unknown function
	Enhanced	Plasma membrane,Cytosol	Secreted in other tissues	Receptor	NA	NA		300000000	Cluster 15: NK-cells - Unknown function
Supported	Supported	Nuclear bodies,Plasma membrane			No	No		30000000	
	Supported	Plasma membrane,Cytosol			NA	NA		22000	Cluster 10: Non-specific - Membrane trafficking
Supported	Supported	Vesicles,Plasma membrane,Cytosol			NA	NA		17000	
	Supported	Plasma membrane	Secreted in other tissues	Receptor	No	No		2300000	Cluster 22: Eosinophils - Transcription
	Approved	Nucleoplasm,Golgi apparatus,Plasma membrane			NA	NA		65000000	
	Supported	Vesicles,Cell Junctions			NA	NA		180000	Cluster 20: Non-specific - DNA binding
	Supported	Nucleoplasm,Plasma membrane,Microtubules			NA	No			Cluster 5: Non-specific - Cell proliferation
	Enhanced	Nucleoplasm,Plasma membrane,Cytosol			NA	NA			Cluster 33: Non-specific - ATP binding
	Approved	Nucleoplasm,Plasma membrane			NA	NA		4600000	Cluster 8: Plasmacytoid DCs - Unknown function
	Supported	Cell Junctions			NA	NA			
	Enhanced	Nucleoplasm,Plasma membrane,Cytosol			NA	NA		980000	Cluster 26: Monocytes - Plasma membrane proteins
	Enhanced	Plasma membrane	Secreted to extracellular matrix	Cell adhesion	NA	NA		6900000	
	Supported	Nucleoplasm,Plasma membrane,Cytosol			No	No			Cluster 21: Neutrophils - Unknown function
	Supported	Golgi apparatus,Cell Junctions	Intracellular and membrane	Cell adhesion	NA	NA			
	Supported	Plasma membrane,Cytosol			NA	NA			
	Supported	Golgi apparatus,Plasma membrane,Cytosol			NA	NA			Cluster 3: Non-specific - Nuclear processes
	Supported	Plasma membrane			NA	NA			
	Supported	Nucleoplasm,Plasma membrane			NA	NA			
	Supported	Nucleoplasm,Golgi apparatus,Plasma membrane			NA	NA		26000000	Cluster 2: Dendritic cells - Unknown function
	Supported	Nucleoplasm,Plasma membrane			NA	NA		15000000	Cluster 8: Plasmacytoid DCs - Unknown function
	Approved	Plasma membrane,Actin filaments			NA	NA			
	Supported	Plasma membrane,Cytosol			NA	NA			Cluster 5: Non-specific - Cell proliferation
	Supported	Nucleoplasm,Plasma membrane,Cytosol	Intracellular and membrane	Enzyme	NA	NA		960000	Cluster 34: B-cells - Unknown function
	Supported	Plasma membrane	Intracellular and membrane	Developmental protei	NA	NA			
	Uncertain	Vesicles,Plasma membrane	Intracellular and membrane	Developmental protei	NA	NA			
	Supported	Vesicles,Plasma membrane,Primary cilium tip			NA	NA		1700000	
	Supported	Plasma membrane			NA	NA			
	Uncertain	Plasma membrane,Cell Junctions,Cytosol			NA	NA			Cluster 38: Monocytes & Neutrophils - Degranulation
	Supported	Plasma membrane,Cell Junctions			NA	NA			Cluster 20: Non-specific - DNA binding
	Supported	Nucleoplasm,Plasma membrane			Yes	No		3600	Cluster 14: Non-specific - Unknown function
	Supported	Nucleoplasm,Plasma membrane,Cytokinetic bridge,Cytosol	Secreted to digestive system	Cell adhesion	Yes	NA		840000	
	Approved	Nucleoplasm,Vesicles,Plasma membrane	Intracellular and membrane	Receptor	NA	NA			Cluster 38: Monocytes & Neutrophils - Degranulation
	Approved	Nucleoplasm,Plasma membrane,Primary cilium,Basal body,Cytosol			NA	NA			Cluster 44: Plasmacytoid DCs - Plasma membrane pr
	Supported	Nucleoplasm,Plasma membrane,Cytosol			NA	NA			
	Supported	Plasma membrane			NA	NA			
	Supported	Nucleoplasm,Plasma membrane			NA	NA			
	Supported	Nucleoplasm,Plasma membrane			NA	NA			
	Supported	Plasma membrane			NA	NA			Cluster 41: Basophils - Unknown function
	Approved	Lipid droplets,Plasma membrane			NA	NA			
	Supported	Plasma membrane			NA	NA			
	Approved	Nucleoplasm,Plasma membrane,Cytosol			NA	NA			Cluster 13: Non-specific - Mitochondria
	Supported	Plasma membrane			NA	NA			
	Approved	Nuclear speckles,Vesicles,Plasma membrane,Primary cilium,Centriolar satellite			NA	NA		1400000	Cluster 7: Non-specific - Innate immune response
	Supported	Vesicles,Plasma membrane			NA	NA			Cluster 42: Non-specific - Vesicular transport
	Enhanced	Plasma membrane,Cytosol			No	No		95000	Cluster 8: Plasmacytoid DCs - Unknown function
Supported	Supported	Plasma membrane,Cytosol			NA	NA		320000	Cluster 7: Non-specific - Innate immune response
	Supported	Plasma membrane,Cell Junctions			NA	Yes			Cluster 21: Neutrophils - Unknown function
	Enhanced	Nucleoplasm,Plasma membrane			NA	NA			
	Supported	Plasma membrane			NA	NA			Cluster 17: B-cells - Immunoglobulins
	Approved	Nucleoplasm,Plasma membrane,Cell Junctions,Cytosol			NA	NA			Cluster 21: Neutrophils - Unknown function
	Enhanced	Plasma membrane			NA	NA			Cluster 33: Non-specific - ATP binding
	Supported	Plasma membrane			NA	NA			Cluster 35: Neutrophils - Protein binding
	Supported	Golgi apparatus,Plasma membrane			NA	NA			Cluster 11: T-cells - Unknown function
	Supported	Plasma membrane,Cell Junctions			NA	NA			
	Supported	Cell Junctions			NA	NA			
	Approved	Plasma membrane,Cell Junctions,Actin filaments,Cytosol			No	No			Cluster 43: Non-specific - Transcription & Translation
	Supported	Plasma membrane,Actin filaments			NA	NA		26000000	Cluster 10: Non-specific - Membrane trafficking
	Supported	Cell Junctions,Centriolar satellite,Cytosol			NA	NA			Cluster 3: Non-specific - Nuclear processes
	Approved	Nucleoplasm,Nucleoli,Cell Junctions			NA	NA		3000000	
	Enhanced	Plasma membrane,Cell Junctions			NA	NA		190000	
	Supported	Nucleoplasm,Plasma membrane,Cytosol			NA	NA			
	Supported	Nucleoplasm,Vesicles,Plasma membrane,Cytosol			NA	NA			
	Enhanced	Plasma membrane,Cytosol			NA	NA		510000	Cluster 21: Neutrophils - Unknown function
	Supported	Plasma membrane			NA	NA			Cluster 25: Non-specific - Unknown function
	Approved	Nucleoplasm,Nuclear membrane,Plasma membrane,Primary cilium,Cytosol			NA	NA			Cluster 22: Eosinophils - Transcription
	Supported	Nucleoplasm,Plasma membrane,Cytosol			NA	NA			Cluster 39: T-cells - T-cell receptor
	Supported	Plasma membrane,Cytosol	Secreted to blood	Growth factor	NA	NA		48000	
	Approved	Nucleoplasm,Nuclear bodies,Plasma membrane,Cell Junctions,Primary cilium,Cytosol	Intracellular and membrane	Receptor	NA	NA		310000	
	Supported	Plasma membrane,Cell Junctions,Mitochondria			NA	NA			
	Supported	Plasma membrane,Cytosol	Intracellular and membrane	Cell adhesion	NA	NA			
	Approved	Nucleoli,Plasma membrane			NA	NA		16000000	Cluster 37: Monocytes & Neutrophils - Innate immune
	Approved	Vesicles,Plasma membrane			NA	NA			Cluster 44: Plasmacytoid DCs - Plasma membrane pr
	Supported	Plasma membrane,Cytosol			NA	NA			Cluster 24: Non-specific - Transcription
	Enhanced	Plasma membrane,Cytosol			NA	NA			Cluster 24: Non-specific - Transcription
	Supported	Plasma membrane,Cytosol			NA	NA			Cluster 40: Monocytes - Innate immune response
	Supported	Nucleoplasm,Plasma membrane,Cytosol			NA	NA			Cluster 7: Non-specific - Innate immune response
	Supported	Endoplasmic reticulum,Plasma membrane,Cytosol	Secreted - unknown location	Transport	NA	NA			
	Enhanced	Nucleoplasm,Cell Junctions			NA	NA			Cluster 45: Eosinophils - Unknown function
	Enhanced	Nucleoplasm,Cell Junctions			NA	NA		5700	
	Enhanced	Plasma membrane,Cell Junctions			NA	NA			
	Supported	Nuclear speckles,Plasma membrane,Microtubules			NA	NA			Cluster 8: Plasmacytoid DCs - Unknown function
	Supported	Nucleoplasm,Plasma membrane,Cytosol			NA	NA		20000	Cluster 42: Non-specific - Vesicular transport
	Supported	Plasma membrane	Intracellular and membrane	Receptor	NA	NA		620000	Cluster 7: Non-specific - Innate immune response
	Uncertain	Nucleoplasm,Plasma membrane,Actin filaments,Microtubules,Cytokinetic bridge,Primary cilium,Centrosome,Basal body,Acrosome,Equatorial segment,Mid piece,Principal piece,End			Yes	NA		2900	Cluster 2: Dendritic cells - Unknown function
	Supported	Plasma membrane,Basal body,Cytosol			NA	NA		10000	Cluster 1: T-cells - Unknown function
	Enhanced	Nucleoplasm,Cell Junctions,Cytosol			NA	NA			Cluster 1: T-cells - Unknown function
	Supported	Cell Junctions,Centrosome			NA	NA			Cluster 35: Neutrophils - Protein binding
	Supported	Plasma membrane,Cytosol			NA	NA			
	Enhanced	Lipid droplets,Plasma membrane,Cytosol			NA	NA		130000	
	Supported	Plasma membrane			NA	NA			Cluster 3: Non-specific - Nuclear processes
	Supported	Plasma membrane			NA	NA			
	Supported	Nucleoplasm,Golgi apparatus,Plasma membrane			NA	Yes		47000	Cluster 38: Monocytes & Neutrophils - Degranulation

	Approved	Nucleoli,Endoplasmic reticulum,Vesicles,Plasma membrane,Centriolar satellite			NA	NA		9400000	
	Enhanced	Nucleoplasm,Plasma membrane			NA	NA			
	Enhanced	Plasma membrane			NA	NA			Cluster 18: Non-specific - Translation
	Supported	Plasma membrane,Cytosol			NA	NA			Cluster 14: Non-specific - Unknown function
	Supported	Nuclear speckles,Plasma membrane			NA	NA			Cluster 12: Non-specific - Mitochondrial translation
	Supported	Plasma membrane,Primary cilium,Cytosol			NA	NA		53000	Cluster 35: Neutrophils - Protein binding
	Enhanced	Nucleoplasm,Plasma membrane,Cytosol			NA	NA		3500	Cluster 5: Non-specific - Cell proliferation
	Approved	Nucleoplasm,Plasma membrane			NA	NA			
Approved	Supported	Vesicles,Plasma membrane,Cytosol			NA	NA		170000	Cluster 11: T-cells - Unknown function
Supported	Supported	Vesicles,Plasma membrane,Intermediate filaments,Cytosol			NA	NA			Cluster 23: B-cells - Immunoglobulins
	Supported	Plasma membrane,Cytosol			NA	NA			
	Supported	Nucleoplasm,Vesicles,Plasma membrane,Basal body,Cytosol			NA	NA			Cluster 22: Eosinophils - Transcription
	Supported	Nucleoplasm,Plasma membrane,Cytosol			NA	NA			Cluster 45: Eosinophils - Unknown function
	Supported	Plasma membrane			NA	NA			
	Supported	Plasma membrane			NA	NA			
	Supported	Nuclear speckles,Plasma membrane,Primary cilium,Cytosol			NA	NA			Cluster 33: Non-specific - ATP binding
	Supported	Plasma membrane,Cytosol			NA	NA		83000	Cluster 5: Non-specific - Cell proliferation
	Supported	Plasma membrane,Cytosol			NA	NA			Cluster 21: Neutrophils - Unknown function
	Supported	Nucleoplasm,Vesicles,Plasma membrane			NA	NA			Cluster 21: Neutrophils - Unknown function
	Supported	Nucleoli,Plasma membrane			NA	NA			Cluster 30: Non-specific - DNA binding
	Supported	Nucleoli,Plasma membrane,Cytosol			NA	NA			
	Supported	Plasma membrane,Cytosol			NA	NA			Cluster 2: Dendritic cells - Unknown function
	Approved	Vesicles,Plasma membrane,Cytosol	Secreted to blood	Receptor	NA	NA		5600000	Cluster 8: Plasmacytoid DCs - Unknown function
	Approved	Nuclear speckles,Plasma membrane,Cytosol			NA	NA			
	Approved	Nucleoplasm,Nucleoli,Nuclear bodies,Cell Junctions	Secreted to blood	Enzyme	NA	NA		90000000	Cluster 38: Monocytes & Neutrophils - Degranulation
	Supported	Vesicles,Plasma membrane,Cell Junctions,Connecting piece,Flagellar centriole			NA	NA		4800000	Cluster 18: Non-specific - Translation
	Enhanced	Plasma membrane,Primary cilium,Primary cilium tip			NA	Yes		17000000	Cluster 30: Non-specific - DNA binding
	Supported	Plasma membrane,Cell Junctions,Cytosol	Secreted - unknown location	Receptor	NA	NA			
	Approved	Plasma membrane,Cytosol	Secreted - unknown location	Receptor	NA	NA		41000000	Cluster 44: Plasmacytoid DCs - Plasma membrane pr
	Supported	Nucleoplasm,Vesicles,Plasma membrane	Secreted - unknown location	Receptor	NA	NA		160000000	Cluster 38: Monocytes & Neutrophils - Degranulation
	Supported	Plasma membrane,Centriolar satellite,Cytosol			NA	NA			Cluster 2: Dendritic cells - Unknown function
	Supported	Plasma membrane,Cytosol			NA	NA			Cluster 2: Dendritic cells - Unknown function
	Supported	Plasma membrane,Primary cilium,Centrosome,Basal body,Cytosol			NA	NA			
	Supported	Plasma membrane			NA	NA			Cluster 43: Non-specific - Transcription & Translation
	Approved	Nucleoplasm,Nucleoli,Golgi apparatus,Plasma membrane,Primary cilium,Centriolar satellite,Basal body,Cytosol			No	No		5300000	Cluster 37: Monocytes & Neutrophils - Innate immune
	Supported	Vesicles,Plasma membrane	Secreted - unknown location	Immunity	NA	NA			
	Supported	Vesicles,Plasma membrane			NA	NA			
	Supported	Plasma membrane,Cytosol			NA	NA			Cluster 35: Neutrophils - Protein binding
	Supported	Plasma membrane,Focal adhesion sites			NA	NA			Cluster 11: T-cells - Unknown function
	Supported	Nucleoplasm,Nuclear bodies,Plasma membrane			NA	NA			Cluster 35: Neutrophils - Protein binding
	Supported	Plasma membrane			NA	NA			
	Supported	Plasma membrane,Cytosol			NA	NA			Cluster 35: Neutrophils - Protein binding
	Approved	Nuclear bodies,Plasma membrane,Cytosol			NA	NA		34000000	Cluster 33: Non-specific - ATP binding
	Approved	Nucleoplasm,Plasma membrane			NA	NA			Cluster 3: Non-specific - Nuclear processes
	Supported	Plasma membrane,Cytosol			NA	NA			Cluster 1: T-cells - Unknown function
	Supported	Plasma membrane			NA	NA		700000	Cluster 29: Non-specific - Unknown function
	Supported	Nucleoplasm,Plasma membrane,Cytosol			NA	NA			Cluster 7: Non-specific - Innate immune response
	Supported	Golgi apparatus,Plasma membrane,Cytosol			NA	NA			Cluster 35: Neutrophils - Protein binding
	Enhanced	Plasma membrane,Cytosol			NA	Yes			Cluster 33: Non-specific - ATP binding
	Supported	Nucleoplasm,Plasma membrane			NA	NA		31000000	Cluster 5: Non-specific - Cell proliferation
	Supported	Plasma membrane			NA	NA		23000000	Cluster 33: Non-specific - ATP binding
	Supported	Plasma membrane,Microtubules			NA	NA			Cluster 21: Neutrophils - Unknown function
	Supported	Vesicles,Plasma membrane,Cytosol			NA	NA			Cluster 29: Non-specific - Unknown function
Supported	Approved	Golgi apparatus,Plasma membrane,Cytosol			No	NA		14000000	
	Supported	Nucleoli fibrillar center,Cell Junctions			NA	NA		5200	Cluster 25: Non-specific - Unknown function
	Supported	Plasma membrane			NA	NA			Cluster 33: Non-specific - ATP binding
	Supported	Plasma membrane			NA	NA			Cluster 46: Basophils - Proteolysis
	Supported	Plasma membrane,Cytosol			NA	NA		1600000	Cluster 38: Monocytes & Neutrophils - Degranulation
	Supported	Plasma membrane,Cytosol			No	No			Cluster 24: Non-specific - Transcription
	Supported	Vesicles,Plasma membrane,Actin filaments			NA	NA			
	Supported	Nucleoplasm,Plasma membrane,Cytosol			No	No			Cluster 35: Neutrophils - Protein binding
	Supported	Plasma membrane,Primary cilium			NA	NA		7300000	
	Approved	Nucleoplasm,Nuclear bodies,Vesicles,Plasma membrane,Cytosol,Acrosome,Mid piece,Principal piece,End piece			NA	NA		6700	Cluster 40: Monocytes - Innate immune response
	Supported	Plasma membrane,Cytosol			NA	NA		750000	Cluster 24: Non-specific - Transcription
	Supported	Nucleoli,Vesicles,Plasma membrane,Cytosol			No	No		4300	Cluster 37: Monocytes & Neutrophils - Innate immune
	Supported	Nucleoplasm,Nuclear bodies,Golgi apparatus,Vesicles,Plasma membrane,Primary cilium,Primary cilium tip,Basal body			NA	NA			Cluster 15: NK-cells - Unknown function
	Approved	Plasma membrane,Actin filaments,Focal adhesion sites			NA	NA		780000	Cluster 11: T-cells - Unknown function
	Approved	Nucleoplasm,Plasma membrane,Actin filaments,Midbody	Intracellular and membrane	Receptor	Yes	NA		19000	
	Enhanced	Golgi apparatus,Plasma membrane,Cytosol			NA	NA		52000	Cluster 10: Non-specific - Membrane trafficking
	Enhanced	Plasma membrane,Cytosol			NA	NA		38000000	Cluster 38: Monocytes & Neutrophils - Degranulation
	Supported	Plasma membrane,Intermediate filaments,Cytosol	Secreted to blood	Immunity	NA	NA	10600000	1100000000	Cluster 38: Monocytes & Neutrophils - Degranulation
	Supported	Nucleoplasm,Plasma membrane,Cytosol			No	No		6000	
	Supported	Nucleoplasm,Nuclear bodies,Plasma membrane			NA	NA			
	Approved	Vesicles,Plasma membrane,Cytosol			NA	NA			
	Supported	Nucleoplasm,Nucleoli,Plasma membrane			NA	NA			
	Approved	Vesicles,Plasma membrane,Cell Junctions			NA	NA			
	Supported	Plasma membrane,Actin filaments			NA	NA			
	Supported	Nucleoplasm,Plasma membrane,Primary cilium,Primary cilium transition zone			NA	NA			
	Enhanced	Nucleoplasm,Plasma membrane,Cell Junctions,Rods & Rings			NA	NA			
	Supported	Plasma membrane	Secreted to blood	No annotated function	NA	NA	1890000	600000	
	Enhanced	Plasma membrane			NA	NA			Cluster 21: Neutrophils - Unknown function
	Supported	Nuclear speckles,Golgi apparatus,Plasma membrane,Cytosol			NA	NA			Cluster 35: Neutrophils - Protein binding
	Supported	Nucleoplasm,Vesicles,Plasma membrane,Primary cilium transition zone,Centrosome,Basal body			NA	NA		2800000	Cluster 21: Neutrophils - Unknown function
	Supported	Vesicles,Plasma membrane,Centrosome,Cytosol			NA	NA			Cluster 33: Non-specific - ATP binding
	Supported	Plasma membrane			NA	NA			
	Supported	Plasma membrane,Actin filaments,Cytosol	Intracellular and membrane	Immunity	NA	NA			Cluster 25: Non-specific - Unknown function
	Uncertain	Vesicles,Plasma membrane			NA	NA		9700000	Cluster 38: Monocytes & Neutrophils - Degranulation
	Enhanced	Plasma membrane,Actin filaments			NA	NA			Cluster 2: Dendritic cells - Unknown function
	Supported	Nucleoplasm,Golgi apparatus,Plasma membrane			NA	NA			Cluster 29: Non-specific - Unknown function
	Supported	Plasma membrane			NA	NA			
	Approved	Nucleoplasm,Plasma membrane			NA	NA			
	Supported	Plasma membrane			NA	NA			Cluster 9: T-regs - Cell cycle regulation
Supported	Enhanced	Plasma membrane,Cell Junctions			NA	NA		15000	Cluster 9: T-regs - Cell cycle regulation
	Supported	Vesicles,Cell Junctions			NA	NA			Cluster 31: T-cells - T-cell receptor
	Supported	Nuclear membrane,Plasma membrane			NA	NA		28000	Cluster 21: Neutrophils - Unknown function
	Supported	Nucleoplasm,Plasma membrane			NA	NA			Cluster 33: Non-specific - ATP binding
	Supported	Plasma membrane,Cytosol			NA	NA			Cluster 13: Non-specific - Mitochondria
	Supported	Plasma membrane,Centriolar satellite,Basal body			NA	NA		27000	Cluster 10: Non-specific - Membrane trafficking
Supported	Supported	Plasma membrane,Intermediate filaments			NA	NA			
	Supported	Plasma membrane			NA	NA			Cluster 37: Monocytes & Neutrophils - Innate immune
	Supported	Plasma membrane			NA	NA			
	Supported	Plasma membrane,Cytosol			NA	NA			Cluster 10: Non-specific - Membrane trafficking
	Approved	Nuclear speckles,Plasma membrane,Cytosol			NA	NA			
Supported	Enhanced	Plasma membrane,Centriolar satellite			No	No		2500000	Cluster 14: Non-specific - Unknown function
	Supported	Plasma membrane,Cell Junctions			NA	NA			
	Supported	Plasma membrane			NA	NA			Cluster 41: Basophils - Unknown function
	Supported	Plasma membrane,Mid piece,Principal piece			NA	NA			
	Supported	Plasma membrane			NA	NA		6800000	Cluster 35: Neutrophils - Protein binding
	Supported	Plasma membrane,Mid piece,Principal piece			NA	NA			Cluster 34: B-cells - Unknown function
	Supported	Vesicles,Plasma membrane			NA	NA			Cluster 42: Non-specific - Vesicular transport
	Supported	Vesicles,Plasma membrane			NA	NA			
	Approved	Nucleoplasm,Nuclear speckles,Plasma membrane,Mitotic spindle,Cytosol			Yes	NA			
	Supported	Nucleoplasm,Plasma membrane,Cytosol			NA	NA			
	Supported	Plasma membrane			NA	NA			
	Supported	Plasma membrane			NA	NA			Cluster 30: Non-specific - DNA binding
	Uncertain	Nucleoplasm,Plasma membrane,Cytosol			NA	NA			Cluster 10: Non-specific - Membrane trafficking
	Approved	Nucleoplasm,Plasma membrane			NA	NA		61000000	Cluster 44: Plasmacytoid DCs - Plasma membrane pr
	Approved	Nucleoplasm,Plasma membrane,Cytosol			NA	NA			Cluster 21: Neutrophils - Unknown function
	Supported	Plasma membrane			NA	NA			Cluster 5: Non-specific - Cell proliferation
	Supported	Plasma membrane,Cytosol			NA	NA			Cluster 38: Monocytes & Neutrophils - Degranulation
	Uncertain	Nuclear speckles,Plasma membrane,Cytosol			NA	NA			Cluster 13: Non-specific - Mitochondria
	Approved	Plasma membrane,Cytosol			NA	NA		4500	
	Supported	Plasma membrane,Focal adhesion sites,Cytosol			NA	NA			Cluster 33: Non-specific - ATP binding
	Approved	Nuclear membrane,Golgi apparatus,Plasma membrane			NA	NA			
	Supported	Nucleoplasm,Plasma membrane			NA	NA			
	Supported	Plasma membrane			NA	NA			Cluster 30: Non-specific - DNA binding
	Supported	Plasma membrane,Cell Junctions			NA	NA			
	Supported	Vesicles,Plasma membrane,Cytosol			NA	NA		76000	Cluster 44: Plasmacytoid DCs - Plasma membrane pr
	Uncertain	Vesicles,Plasma membrane			NA	NA			Cluster 30: Non-specific - DNA binding

	Supported	Nucleoplasm,Plasma membrane	Intracellular and membrane	Transport	NA	NA			Cluster 38: Monocytes & Neutrophils - Degranulation
	Supported	Nucleoplasm,Plasma membrane			NA	NA		8400	Cluster 35: Neutrophils - Protein binding
	Supported	Cell Junctions			NA	NA			
	Supported	Plasma membrane			NA	NA			Cluster 43: Non-specific - Transcription & Translation
	Supported	Plasma membrane			NA	NA			Cluster 2: Dendritic cells - Unknown function
	Supported	Vesicles,Plasma membrane			NA	NA			Cluster 33: Non-specific - ATP binding
	Supported	Nuclear speckles,Plasma membrane			NA	NA			
	Supported	Plasma membrane			No	No			Cluster 21: Neutrophils - Unknown function
	Supported	Plasma membrane			NA	NA			
	Supported	Plasma membrane			NA	NA			
	Supported	Cell Junctions			NA	NA			Cluster 16: Non-specific - Cell proliferation
	Supported	Vesicles,Plasma membrane			NA	NA			
	Supported	Cell Junctions	Secreted in other tissues	Developmental protei	NA	NA			
	Supported	Plasma membrane			NA	NA		16000	
	Supported	Nucleoplasm,Plasma membrane,Cell Junctions			NA	NA			Cluster 24: Non-specific - Transcription
	Supported	Plasma membrane,Primary cilium			NA	NA		2000000	Cluster 35: Neutrophils - Protein binding
	Approved	Nucleoplasm,Golgi apparatus,Plasma membrane,Primary cilium transition zone,Centriolar satellite,Basal body			NA	NA			Cluster 33: Non-specific - ATP binding
	Supported	Nucleoplasm,Plasma membrane			NA	NA			
	Supported	Plasma membrane,Cytosol			No	No		120000	Cluster 44: Plasmacytoid DCs - Plasma membrane pr
	Supported	Plasma membrane,Focal adhesion sites,Centrosome			NA	NA			Cluster 41: Basophils - Unknown function
	Supported	Golgi apparatus,Plasma membrane,Cytosol			NA	NA		120000	Cluster 25: Non-specific - Unknown function
	Supported	Nucleoli,Plasma membrane,Cytosol			NA	NA			Cluster 25: Non-specific - Unknown function
	Supported	Vesicles,Plasma membrane,Primary cilium,Primary cilium transition zone			NA	NA			Cluster 2: Dendritic cells - Unknown function
	Supported	Nucleoplasm,Plasma membrane,Cytosol	Secreted to blood	Enzyme inhibitor	NA	NA		10000000	Cluster 7: Non-specific - Innate immune response
	Approved	Nucleoplasm,Plasma membrane,Cytosol			NA	NA			
	Approved	Nucleoplasm,Plasma membrane,Centrosome			NA	NA			Cluster 22: Eosinophils - Transcription
	Supported	Vesicles,Plasma membrane			NA	NA			Cluster 35: Neutrophils - Protein binding
	Supported	Vesicles,Plasma membrane,Cell Junctions			NA	NA		1700000	Cluster 10: Non-specific - Membrane trafficking
	Supported	Plasma membrane			NA	NA			
	Supported	Nucleoplasm,Plasma membrane,Cytosol			NA	NA			Cluster 42: Non-specific - Vesicular transport
	Approved	Nucleoplasm,Plasma membrane			NA	NA			Cluster 21: Neutrophils - Unknown function
	Supported	Nucleoplasm,Nuclear bodies,Plasma membrane			NA	NA		35000	Cluster 37: Monocytes & Neutrophils - Innate immune
	Supported	Vesicles,Plasma membrane,Cytosol			NA	NA		23000000	Cluster 33: Non-specific - ATP binding
	Supported	Plasma membrane,Cytosol			NA	NA		33000	Cluster 13: Non-specific - Mitochondria
	Supported	Plasma membrane			NA	NA			
	Supported	Endoplasmic reticulum,Plasma membrane			NA	NA			Cluster 13: Non-specific - Mitochondria
	Supported	Plasma membrane,Actin filaments,Cytosol			NA	NA		120000	Cluster 21: Neutrophils - Unknown function
	Supported	Plasma membrane,Actin filaments			NA	NA		32000	Cluster 29: Non-specific - Unknown function
	Supported	Nucleoplasm,Plasma membrane,Cytosol			NA	NA			Cluster 14: Non-specific - Unknown function
	Approved	Nucleoplasm,Golgi apparatus,Plasma membrane			NA	NA			Cluster 1: T-cells - Unknown function
	Supported	Nucleoplasm,Endoplasmic reticulum,Plasma membrane			NA	NA			Cluster 41: Basophils - Unknown function
	Approved	Plasma membrane,Centrosome,Basal body			NA	NA			
	Supported	Plasma membrane			NA	NA			
	Supported	Nucleoli,Vesicles,Plasma membrane			NA	NA			Cluster 7: Non-specific - Innate immune response
	Supported	Golgi apparatus,Plasma membrane			NA	NA			Cluster 17: B-cells - Immunoglobulins
	Enhanced	Plasma membrane			NA	NA			Cluster 3: Non-specific - Nuclear processes
	Supported	Plasma membrane,Cell Junctions			NA	NA			Cluster 30: Non-specific - DNA binding
	Supported	Plasma membrane,Mid piece,Principal piece			NA	NA			
	Supported	Nuclear speckles,Plasma membrane			NA	NA			Cluster 34: B-cells - Unknown function
	Supported	Plasma membrane,Cell Junctions			NA	NA			Cluster 5: Non-specific - Cell proliferation
	Supported	Plasma membrane,Cell Junctions			NA	NA			Cluster 5: Non-specific - Cell proliferation
	Supported	Plasma membrane			NA	NA			Cluster 32: Basophils - Transcription
	Supported	Plasma membrane,Centrosome,Basal body	Secreted to blood	Receptor	NA	NA	7750000	3100000	
	Approved	Plasma membrane,Primary cilium,Primary cilium tip	Intracellular and membrane	Receptor	NA	NA			Cluster 42: Non-specific - Vesicular transport
	Enhanced	Plasma membrane	Secreted to blood	Receptor	NA	NA		550000	Cluster 20: Non-specific - DNA binding
	Supported	Nucleoplasm,Nuclear membrane,Cell Junctions,Cytokinetic bridge,Cytosol			Yes	NA			Cluster 9: T-regs - Cell cycle regulation
	Supported	Plasma membrane,Cytosol			NA	NA			Cluster 43: Non-specific - Transcription & Translation
	Enhanced	Cell Junctions			NA	NA			
	Enhanced	Nucleoplasm,Plasma membrane,Cell Junctions			NA	NA		1100000	Cluster 40: Monocytes - Innate immune response
	Enhanced	Nucleoplasm,Plasma membrane,Cell Junctions			NA	NA			Cluster 11: T-cells - Unknown function
	Supported	Plasma membrane,Focal adhesion sites,Centriolar satellite,Cytosol			NA	NA		1300000000	Cluster 25: Non-specific - Unknown function
	Supported	Nucleoplasm,Vesicles,Plasma membrane,Cytosol			NA	NA			Cluster 38: Monocytes & Neutrophils - Degranulation
	Approved	Golgi apparatus,Plasma membrane	Intracellular and membrane	Receptor	NA	NA			Cluster 38: Monocytes & Neutrophils - Degranulation
	Supported	Plasma membrane,Focal adhesion sites			NA	NA			
	Approved	Plasma membrane,Cytosol			NA	NA			
	Supported	Plasma membrane,Cytosol			NA	NA			
	Enhanced	Plasma membrane,Actin filaments			NA	NA			
	Supported	Vesicles,Plasma membrane			NA	NA			Cluster 36: Eosinophils - Protein ubiquitination
	Supported	Plasma membrane	Secreted in other tissues	Enzyme	NA	NA			
	Supported	Plasma membrane,Cytosol	Intracellular and membrane	Receptor	NA	NA			Cluster 15: NK-cells - Unknown function
	Supported	Plasma membrane,Cytosol			NA	NA		150000	Cluster 33: Non-specific - ATP binding
	Supported	Plasma membrane,Cytosol			NA	NA		350000	Cluster 44: Plasmacytoid DCs - Plasma membrane pr
	Supported	Plasma membrane			NA	NA			Cluster 37: Monocytes & Neutrophils - Innate immune
	Supported	Vesicles,Plasma membrane,Focal adhesion sites	Secreted to blood	Cytokine	NA	NA	393000	480000	Cluster 38: Monocytes & Neutrophils - Degranulation
	Supported	Vesicles,Plasma membrane			NA	NA			Cluster 20: Non-specific - DNA binding
	Enhanced	Cell Junctions,Cytosol			NA	NA			Cluster 30: Non-specific - DNA binding
	Supported	Nucleoplasm,Plasma membrane	Intracellular and membrane	Enzyme	NA	NA		58000	
	Supported	Plasma membrane,Cytosol			NA	NA			Cluster 33: Non-specific - ATP binding
	Enhanced	Plasma membrane,Mitotic spindle,Centriolar satellite			Yes	NA			Cluster 5: Non-specific - Cell proliferation
	Approved	Nucleoplasm,Plasma membrane,Cytosol			NA	NA			
	Uncertain	Nuclear speckles,Golgi apparatus,Plasma membrane,Cytosol			NA	NA		41000000	Cluster 21: Neutrophils - Unknown function
	Supported	Nucleoplasm,Plasma membrane			NA	NA			Cluster 38: Monocytes & Neutrophils - Degranulation
	Supported	Plasma membrane			NA	NA			Cluster 42: Non-specific - Vesicular transport
	Approved	Nuclear bodies,Vesicles,Plasma membrane,Cytosol			NA	NA			Cluster 1: T-cells - Unknown function
	Enhanced	Plasma membrane			NA	NA			
	Supported	Plasma membrane,Cytosol	Intracellular and membrane	Other	NA	NA			Cluster 10: Non-specific - Membrane trafficking
	Supported	Nucleoplasm,Nucleoli,Plasma membrane,Mitotic spindle,Primary cilium,Primary cilium transition zone,Basal body	Intracellular and membrane	Developmental protei	Yes	NA			Cluster 14: Non-specific - Unknown function
	Supported	Nucleoplasm,Nucleoli,Plasma membrane,Cytosol			NA	NA			Cluster 24: Non-specific - Transcription
	Supported	Plasma membrane,Cytosol			No	No			Cluster 45: Eosinophils - Unknown function
	Supported	Plasma membrane			NA	NA			Cluster 38: Monocytes & Neutrophils - Degranulation
	Supported	Plasma membrane,Cytosol			Yes	Yes			Cluster 16: Non-specific - Cell proliferation
	Approved	Plasma membrane,Cytosol			NA	NA			Cluster 35: Neutrophils - Protein binding
	Supported	Plasma membrane,Cytosol			NA	NA			
	Supported	Vesicles,Plasma membrane	Secreted in other tissues	Immunity	NA	NA			Cluster 1: T-cells - Unknown function
	Supported	Endoplasmic reticulum,Plasma membrane,Cytosol			NA	NA		4400000	Cluster 33: Non-specific - ATP binding
	Supported	Plasma membrane,Cytosol			NA	NA			Cluster 20: Non-specific - DNA binding
	Supported	Nucleoplasm,Plasma membrane,Primary cilium,Basal body			NA	NA		16000	Cluster 40: Monocytes - Innate immune response
	Supported	Plasma membrane,Cell Junctions,Focal adhesion sites			NA	NA		28000000	Cluster 45: Eosinophils - Unknown function
	Enhanced	Plasma membrane			NA	NA			Cluster 38: Monocytes & Neutrophils - Degranulation
	Supported	Vesicles,Lysosomes,Plasma membrane,Cell Junctions			NA	NA			Cluster 5: Non-specific - Cell proliferation
	Supported	Plasma membrane,Cell Junctions,Cytosol			NA	NA		66000000	Cluster 37: Monocytes & Neutrophils - Innate immune
	Enhanced	Nucleoplasm,Plasma membrane			NA	NA			Cluster 21: Neutrophils - Unknown function
	Supported	Plasma membrane,Cytosol			NA	NA			Cluster 22: Eosinophils - Transcription
	Supported	Plasma membrane,Cytosol			NA	NA			Cluster 41: Basophils - Unknown function
	Supported	Nucleoplasm,Plasma membrane,Cytosol			NA	NA		100000	Cluster 35: Neutrophils - Protein binding
	Enhanced	Nucleoplasm,Plasma membrane,Cytosol			NA	NA			Cluster 5: Non-specific - Cell proliferation
	Supported	Plasma membrane			NA	NA			Cluster 3: Non-specific - Nuclear processes
	Supported	Vesicles,Plasma membrane			NA	NA			Cluster 33: Non-specific - ATP binding
	Enhanced	Plasma membrane			NA	NA			
	Approved	Nucleoplasm,Plasma membrane			NA	NA			Cluster 41: Basophils - Unknown function
	Supported	Plasma membrane,Actin filaments,Focal adhesion sites,Cytosol			NA	NA		64000	Cluster 42: Non-specific - Vesicular transport
	Supported	Nucleoplasm,Plasma membrane			NA	NA			Cluster 42: Non-specific - Vesicular transport

Tissue expression cluster	Brain expression cluster	Cell line expression cluster	Single cell expression cluster	Interactions	Subcellular main location	Subcellular additional location
Cluster 69: Parathyroid gland - Mixed function	Cluster 52: Neurons & Synapses - Synaptic function	Cluster 59: Leukemia - T-cell receptor	Cluster 9: T-cells - Adaptive immunity: Regulation	28	Plasma membrane	Vesicles, Cytosol
Cluster 70: Non-specific - Protein processing	Cluster 27: Non-specific - Mixed function	Cluster 26: Non-specific - Mitochondria & RNA binding	Cluster 15: Non-specific - Protein processing	23	Microtubules	Plasma membrane, Cytokinetic bridge, Primary cilium, Primary cilium tip, Cytosol
Cluster 76: Non-specific - Metabolism	Cluster 39: Astrocytes - Mixed function	Cluster 15: Kidney cancer - Membrane components	Cluster 31: Neutrophils - Chemotaxis & Apoptosis	16	Plasma membrane	Golgi apparatus
Cluster 64: Non-specific - Transcription	Cluster 44: Non-specific - Mixed function	Cluster 55: Lymphoma - Immune response	Cluster 86: Retinal bipolar cells - Synaptic signal transduction		Golgi apparatus, Plasma membrane	Cell Junctions
Cluster 4: Adrenal gland - Steroid metabolism	Cluster 47: Endothelial cells - Vasculature	Cluster 70: DU4475 - Unknown function	Cluster 104: Brain endothelial cells - Blood brain barrier	10	Plasma membrane	
Cluster 45: Liver - Metabolism & Coagulation	Cluster 49: Hindbrain - Mixed function	Cluster 69: Skin cancer - ECM organization	Cluster 93: Hepatocytes - Bile production & excretion		Nucleoplasm, Plasma membrane	Focal adhesion sites, Cytosol
Cluster 24: Non-specific - Transcription	Cluster 27: Non-specific - Mixed function	Cluster 7: Liver cancer - Metabolism	Cluster 35: Neurons - Synaptic function & neurogenesis		Golgi apparatus	Nucleoplasm, Plasma membrane, Mitochondria, Cytosol, Mid piece, Principal piece
Cluster 12: Smooth muscle tissue - ECM organization	Cluster 21: Non-specific - Metabolism	Cluster 7: Liver cancer - Metabolism	Cluster 102: Non-specific - Mixed function	13	Plasma membrane	Cell Junctions
Cluster 28: Liver & Adrenal gland - Secretion	Cluster 39: Astrocytes - Mixed function	Cluster 7: Liver cancer - Metabolism	Cluster 18: Adrenal cortex cells - Steroidogensis		Plasma membrane	Basal body
Cluster 38: Non-specific - Basic cellular processes	Cluster 21: Non-specific - Metabolism	Cluster 49: HMC-1 - Innate immune response	Cluster 12: Endothelial cells - Angiogenesis & vascular homeostasis	7	Plasma membrane	Golgi apparatus, Vesicles
Cluster 31: Liver - Metabolism & Coagulation	Cluster 3: Choroid plexus - Cilium	Cluster 18: Liver cancer - Metabolism	Cluster 88: Proximal tubular cells - Transmembrane transport	1	Plasma membrane	Nucleoplasm
Cluster 77: Intestine - Digestion	Cluster 47: Endothelial cells - Vasculature	Cluster 53: Squamous epithelial cells - Keratinization	Cluster 104: Brain endothelial cells - Blood brain barrier	6	Plasma membrane	Nucleoplasm
Cluster 14: Adipose tissue - Angiogenesis	Cluster 39: Astrocytes - Mixed function	Cluster 15: Kidney cancer - Membrane components	Cluster 108: Fibroblasts - ECM organization	6	Vesicles, Plasma membrane	
Cluster 77: Intestine - Digestion	Cluster 34: Non-specific - Mixed function	Cluster 59: Leukemia - T-cell receptor	Cluster 40: Monocytes & Neutrophils - Inflammatory signaling	4	Plasma membrane	Cytosol
Cluster 61: Non-specific - Transcription	Cluster 13: Oligodendrocytes - Mixed function	Cluster 58: Non-specific - Nuclear processes	Cluster 24: Neutrophils - Innate immunity signal integration	41	Vesicles, Plasma membrane	
Cluster 43: Placenta - Pregnancy hormone signaling	Cluster 38: Sub-cortical - Mixed function	Cluster 64: Connective tissue cells - ECM organization	Cluster 21: Extravillous trophoblasts - Placental invasion & endocrine regulatio	10	Plasma membrane	
Cluster 50: Epididymis - Male reproductive secretion	Cluster 20: Macrophages & Microglia - Immune response	Cluster 2: Mucus-secreting cells - Unknown function	Cluster 87: B-cells - Adaptive immunity: Humoral response	1	Plasma membrane	Mitochondria
Cluster 75: Ovary - Mixed function	Cluster 33: Neurons - Mixed function	Cluster 26: Non-specific - Mitochondria & RNA binding	Cluster 110: Mural cells - ECM organization	14	Nucleoplasm, Primary cilium	Nucleoli, Golgi apparatus, Plasma membrane, Primary cilium transition zone, Basal body
Cluster 14: Adipose tissue - Angiogenesis	Cluster 47: Endothelial cells - Vasculature	Cluster 64: Connective tissue cells - ECM organization	Cluster 104: Brain endothelial cells - Blood brain barrier	3	Mitochondria	Nucleoplasm, Plasma membrane
Cluster 19: Skeletal muscle - Striated muscle contraction	Cluster 23: Choroid plexus - Mixed function	Cluster 64: Connective tissue cells - ECM organization	Cluster 101: Choroid plexus epithelial cells - Ion transport	7	Plasma membrane, Cytosol	Primary cilium, Basal body
Cluster 25: Non-specific - Basic cellular processes	Cluster 4: Non-specific - mRNA splicing & Cell cycle	Cluster 22: Non-specific - mRNA splicing & Cell cycle	Cluster 79: Photoreceptor cells - Phototransduction	17	Plasma membrane	Nucleoplasm
Cluster 46: Brain & retina - Neuronal signaling	Cluster 24: Neurons - Mixed function	Cluster 47: Leukemia - Hemostasis	Cluster 109: Neurons - Neuronal signaling	8	Nucleoplasm, Cytosol	Plasma membrane
Cluster 36: Brain & Liver - Signaling	Cluster 39: Astrocytes - Mixed function	Cluster 44: Connective tissue cells - ECM organization	Cluster 48: Neutrophils - Phagocytosis & degranulation	9	Plasma membrane	
Cluster 75: Ovary - Mixed function	Cluster 32: Cerebral cortex - Mixed function	Cluster 14: Myeloid leukemia - Innate immune response	Cluster 110: Mural cells - ECM organization	4	Plasma membrane	Vesicles
Cluster 10: Brain - Synaptic signal transduction	Cluster 51: Neurons - Mixed function	Cluster 72: Neuronal - Signal transduction	Cluster 35: Neurons - Synaptic function & neurogenesis	1	Plasma membrane	
Cluster 14: Adipose tissue - Angiogenesis	Cluster 38: Sub-cortical - Mixed function	Cluster 64: Connective tissue cells - ECM organization	Cluster 108: Fibroblasts - ECM organization	1	Plasma membrane	Nucleoplasm, Nuclear speckles
Cluster 50: Epididymis - Male reproductive secretion	Cluster 29: Forebrain - Mixed function	Cluster 12: Bone cancer - Neuronal signaling	Cluster 10: Epididymal principal cells - Sperm maturation microenvironment		Plasma membrane, Cytosol	
Cluster 3: Oligodendrocytes - Myelination	Cluster 49: Hindbrain - Mixed function	Cluster 15: Kidney cancer - Membrane components	Cluster 57: Podocytes - Glomerular filtration	9	Plasma membrane	Primary cilium
Cluster 1: Choroid plexus - Transmembrane transport	Cluster 40: Astrocytes - Astrocyte-neuron interactions	Cluster 49: HMC-1 - Innate immune response	Cluster 26: Keratinocytes - Keratinization	28	Plasma membrane	Vesicles, Primary cilium tip
Cluster 5: Spleen - Immune response	Cluster 14: Neurons - Synaptic function	Cluster 15: Kidney cancer - Membrane components	Cluster 35: Neurons - Synaptic function & neurogenesis	3	Plasma membrane	
Cluster 58: Skin - Keratinization	Cluster 20: Macrophages & Microglia - Immune response	Cluster 53: Squamous epithelial cells - Keratinization	Cluster 26: Keratinocytes - Keratinization	88	Plasma membrane	Microtubules, Cytokinetic bridge, Mitotic spindle, Primary cilium, Basal body
Cluster 82: Salivary gland - Secretion	Cluster 6: Non-specific - Immune response	Cluster 72: Neuronal - Signal transduction	Cluster 51: Secretory epithelial cells - Mixed function	56	Plasma membrane, Cell Junctions	Nucleoplasm
Cluster 13: Lung - Secretion		Cluster 15: Kidney cancer - Membrane components	Cluster 81: Alveolar cells - Lung function	13	Plasma membrane, Cell Junctions	Nucleoli fibrillar center
Cluster 58: Skin - Keratinization	Cluster 39: Astrocytes - Mixed function	Cluster 72: Neuronal - Signal transduction	Cluster 49: Apical squamous epithelium - Epithelial barrier & keratinization	45	Plasma membrane, Cytosol	
Cluster 58: Skin - Keratinization	Cluster 45: Non-specific - Mixed function	Cluster 53: Squamous epithelial cells - Keratinization	Cluster 85: Squamous epithelial cells - Keratinization	3	Cytosol	Plasma membrane
Cluster 47: Testis - Mixed function	Cluster 52: Neurons & Synapses - Synaptic function	Cluster 72: Neuronal - Signal transduction	Cluster 12: Endothelial cells - Angiogenesis & vascular homeostasis	29	Plasma membrane, Cytosol	
Cluster 77: Intestine - Digestion	Cluster 48: Non-specific - Mixed function	Cluster 7: Liver cancer - Metabolism	Cluster 85: Squamous epithelial cells - Keratinization	13	Cytosol	Plasma membrane
Cluster 55: Breast - Lactation	Cluster 13: Oligodendrocytes - Mixed function	Cluster 7: Liver cancer - Metabolism	Cluster 39: Syncytiotrophoblasts - Placental homeostasis & endocrine signal	1	Cytosol	Plasma membrane
Cluster 25: Non-specific - Basic cellular processes	Cluster 45: Non-specific - Mixed function	Cluster 53: Squamous epithelial cells - Keratinization	Cluster 63: Cytotrophoblasts - Basic cellular functions	28	Nucleoli, Golgi apparatus, Basal body	Plasma membrane, Cytosol
Cluster 73: Brain - Neuronal signaling	Cluster 41: Monoamines - Neurotransmitter signalling	Cluster 17: Neuroblastoma - Neuronal signaling	Cluster 59: Pituitary endocrine cells - Neuroendocrine signaling	109	Plasma membrane	
Cluster 81: Squamous epithelium - Keratinization	Cluster 30: White matter - Myelination	Cluster 27: Breast cancer - Unknown function	Cluster 81: Alveolar cells - Lung function	7	Plasma membrane, Cytosol	
Cluster 63: Non-specific - Basic cellular processes	Cluster 1: Neurons - Mixed function	Cluster 36: Non-specific - mRNA processing	Cluster 62: Smooth muscle cells - Muscle contraction	19	Nuclear bodies	Vesicles, Plasma membrane
Cluster 3: Oligodendrocytes - Myelination	Cluster 11: White matter - Signal transduction	Cluster 12: Bone cancer - Neuronal signaling	Cluster 11: Muller glia cells - Retinal homeostasis	1	Plasma membrane	
Cluster 22: Epididymis - Mixed function	Cluster 39: Astrocytes - Mixed function	Cluster 70: DU4475 - Unknown function	Cluster 30: Epididymal principal cells - Sperm storage microenvironment	81	Cell Junctions	Nucleoplasm
Cluster 25: Non-specific - Basic cellular processes	Cluster 14: Neurons - Synaptic function	Cluster 17: Neuroblastoma - Neuronal signaling	Cluster 28: Astrocytes - Homeostasis & neuron support	40	Plasma membrane, Acrosome	
Cluster 41: Striated muscle - Muscle contraction	Cluster 37: Neurons - Mixed function	Cluster 64: Connective tissue cells - ECM organization	Cluster 86: Retinal bipolar cells - Synaptic signal transduction	16	Plasma membrane	
Cluster 3: Oligodendrocytes - Myelination	Cluster 13: Oligodendrocytes - Mixed function	Cluster 39: Non-specific - Transcription	Cluster 80: Oligodendrocytes - Myelination	2	Plasma membrane, Cytosol	
Cluster 61: Non-specific - Transcription	Cluster 55: Non-specific - Transcription	Cluster 3: Rhabdoid - Membrane components	Cluster 18: Adrenal cortex cells - Steroidogensis	17	Plasma membrane	Nucleoplasm, Cell Junctions
Cluster 34: Lymphoid tissue - Adaptive immune response	Cluster 11: White matter - Signal transduction	Cluster 46: Lymphoma - Humoral immune response	Cluster 29: Neutrophils - Innate immune cellular maintenance	41	Plasma membrane	Vesicles, Primary cilium, Primary cilium tip, Primary cilium transition zone
Cluster 71: Cerebellum - Nervous system development	Cluster 34: Non-specific - Mixed function	Cluster 17: Neuroblastoma - Neuronal signaling	Cluster 73: Late spermatids - Spermiogenesis: Maturation	2	Vesicles	Plasma membrane
Cluster 22: Epididymis - Mixed function	Cluster 37: Neurons - Mixed function	Cluster 53: Squamous epithelial cells - Keratinization	Cluster 110: Mural cells - ECM organization	5	Plasma membrane	Nucleoplasm
Cluster 77: Intestine - Digestion	Cluster 25: Non-specific - Transcription	Cluster 64: Connective tissue cells - ECM organization	Cluster 37: Neutrophil progenitors - Granulopoiesis	1	Plasma membrane	Vesicles, Primary cilium
Cluster 42: Retina - Visual perception	Cluster 47: Endothelial cells - Vasculature	Cluster 1: Neuronal - Visual perception	Cluster 12: Endothelial cells - Angiogenesis & vascular homeostasis		Plasma membrane	Nucleoplasm
Cluster 75: Ovary - Mixed function	Cluster 39: Astrocytes - Mixed function	Cluster 53: Squamous epithelial cells - Keratinization	Cluster 89: Adipocytes - Lipid metabolism	16	Plasma membrane, Cytosol	
Cluster 77: Intestine - Digestion	Cluster 12: Non-specific - Vasculature	Cluster 44: Connective tissue cells - ECM organization	Cluster 1: Enterocytes - Absorption and Digestion	15	Plasma membrane	
Cluster 81: Squamous epithelium - Keratinization	Cluster 39: Astrocytes - Mixed function	Cluster 5: Rhabdoid - Unknown function	Cluster 49: Apical squamous epithelium - Epithelial barrier & keratinization	35	Plasma membrane, Cytosol	Nucleoplasm
Cluster 7: Small intestine - Absorption and Digestion	Cluster 30: White matter - Myelination	Cluster 10: Ciliated cells - Ion channel activity	Cluster 95: Tuft cells - Chemosensory signaling	1	Plasma membrane	Nucleoplasm
Cluster 29: Lymphoid tissue & Bone marrow - Innate immune response	Cluster 49: Hindbrain - Mixed function	Cluster 53: Squamous epithelial cells - Keratinization	Cluster 37: Neutrophil progenitors - Granulopoiesis		Plasma membrane	
Cluster 36: Brain & Liver - Signaling	Cluster 14: Neurons - Synaptic function	Cluster 63: Non-specific - Protein processing	Cluster 97: Non-specific - Cellular respiration	132	Plasma membrane	
Cluster 27: Cerebellum - Synaptic function	Cluster 30: White matter - Myelination	Cluster 17: Neuroblastoma - Neuronal signaling	Cluster 80: Oligodendrocytes - Myelination	32	Plasma membrane	Endoplasmic reticulum
Cluster 51: Lymphoid tissue - Adaptive immune response	Cluster 20: Macrophages & Microglia - Immune response	Cluster 46: Lymphoma - Humoral immune response	Cluster 24: Neutrophils - Innate immunity signal integration	4	Plasma membrane, Cytosol	
Cluster 73: Brain - Neuronal signaling	Cluster 38: Sub-cortical - Mixed function	Cluster 22: Non-specific - mRNA splicing & Cell cycle	Cluster 28: Astrocytes - Homeostasis & neuron support	94	Plasma membrane	Golgi apparatus
Cluster 66: Lymphoid tissue - Immune response	Cluster 6: Non-specific - Immune response	Cluster 49: HMC-1 - Innate immune response	Cluster 48: Neutrophils - Phagocytosis & degranulation		Plasma membrane	Centrosome
Cluster 14: Adipose tissue - Angiogenesis	Cluster 47: Endothelial cells - Vasculature	Cluster 9: Skin cancer - Melanin biosynthesis	Cluster 103: Non-specific - Basic cellular processes		Nucleoplasm, Plasma membrane	
Cluster 63: Non-specific - Basic cellular processes	Cluster 55: Non-specific - Transcription	Cluster 8: Non-specific - Basic cellular processes	Cluster 82: Dendritic cells - Antigen presentation	108	Vesicles, Plasma membrane	Actin filaments, Cytosol
Cluster 1: Choroid plexus - Transmembrane transport	Cluster 22: Non-specific - Mixed function	Cluster 65: Non-specific - Unknown function	Cluster 32: Endothelial cells - Angiogenesis & vascular immunity	109	Plasma membrane	
Cluster 7: Small intestine - Absorption and Digestion		Cluster 47: Leukemia - Hemostasis	Cluster 47: Platelets - Hemostasis	17	Plasma membrane	Vesicles
Cluster 7: Small intestine - Absorption and Digestion	Cluster 1: Neurons - Mixed function	Cluster 3: Rhabdoid - Membrane components	Cluster 71: Non-specific - Cardiac endocrine signaling & maintenanc		Plasma membrane	Vesicles
Cluster 81: Squamous epithelium - Keratinization	Cluster 49: Hindbrain - Mixed function	Cluster 53: Squamous epithelial cells - Keratinization	Cluster 85: Squamous epithelial cells - Keratinization	23	Plasma membrane	
Cluster 73: Brain - Neuronal signaling	Cluster 39: Astrocytes - Mixed function	Cluster 72: Neuronal - Signal transduction	Cluster 28: Astrocytes - Homeostasis & neuron support	4	Plasma membrane	Cell Junctions
Cluster 63: Non-specific - Basic cellular processes	Cluster 25: Non-specific - Transcription	Cluster 36: Non-specific - mRNA processing	Cluster 15: Non-specific - Protein processing	115	Plasma membrane	
Cluster 51: Lymphoid tissue - Adaptive immune response	Cluster 50: Non-specific - Nucleic acid binding	Cluster 71: Lymphoma - Inflammatory response	Cluster 24: Neutrophils - Innate immunity signal integration	2	Plasma membrane	
Cluster 5: Spleen - Immune response	Cluster 30: White matter - Myelination	Cluster 55: Lymphoma - Immune response	Cluster 102: Non-specific - Mixed function	30	Plasma membrane, Cytosol	
Cluster 59: Lymphoid tissue & Bone marrow - Cell proliferation	Cluster 13: Oligodendrocytes - Mixed function	Cluster 6: Leukemia - Signal transduction	Cluster 66: Erythroid cells - Erythroid differentiation and maturation	10	Plasma membrane	
Cluster 3: Oligodendrocytes - Myelination	Cluster 11: White matter - Signal transduction	Cluster 52: Non-specific - Mitochondria	Cluster 80: Oligodendrocytes - Myelination	24	Plasma membrane, Cell Junctions	Actin filaments
Cluster 3: Oligodendrocytes - Myelination	Cluster 30: White matter - Myelination	Cluster 14: Myeloid leukemia - Innate immune response	Cluster 74: Microglia - Neuroimmunity & neuroinflammation	8	Nucleoplasm, Cytosol	Plasma membrane
Cluster 66: Lymphoid tissue - Immune response	Cluster 20: Macrophages & Microglia - Immune response	Cluster 14: Myeloid leukemia - Innate immune response	Cluster 92: NK-cells & T-cells - Adaptive immunity: Cytotoxicity	8	Plasma membrane, Cytosol	
Cluster 66: Lymphoid tissue - Immune response	Cluster 11: White matter - Signal transduction	Cluster 46: Lymphoma - Humoral immune response	Cluster 92: NK-cells & T-cells - Adaptive immunity: Cytotoxicity	13	Plasma membrane, Cytosol	
Cluster 46: Brain & retina - Neuronal signaling	Cluster 52: Neurons & Synapses - Synaptic function	Cluster 69: Skin cancer - ECM organization	Cluster 86: Retinal bipolar cells - Synaptic signal transduction	16	Plasma membrane	Nucleoplasm
Cluster 14: Adipose tissue - Angiogenesis	Cluster 47: Endothelial cells - Vasculature	Cluster 48: Adipocytes & Endothelial cells - Angiogenesis	Cluster 32: Endothelial cells - Angiogenesis & vascular immunity	4	Plasma membrane	
Cluster 66: Lymphoid tissue - Immune response	Cluster 11: White matter - Signal transduction	Cluster 23: Non-specific - Transcription	Cluster 87: B-cells - Adaptive immunity: Humoral response	3	Cytosol	Plasma membrane
Cluster 74: Non-specific - Signal transduction	Cluster 39: Astrocytes - Mixed function	Cluster 3: Rhabdoid - Membrane components	Cluster 28: Astrocytes - Homeostasis & neuron support	5	Plasma membrane	Nucleoplasm, Cytosol
Cluster 58: Skin - Keratinization	Cluster 42: Choroid plexus - Mitochondria	Cluster 15: Kidney cancer - Membrane components	Cluster 100: Stratified epithelial cells - Barrier functions	30	Plasma membrane, Cytosol	Nucleoplasm
Cluster 44: Bone marrow - Nuclear processes	Cluster 23: Choroid plexus - Mixed function	Cluster 15: Kidney cancer - Membrane components	Cluster 92: NK-cells & T-cells - Adaptive immunity: Cytotoxicity	8	Plasma membrane	Cytosol
Cluster 48: Ciliated tissues - Cilium organization	Cluster 3: Choroid plexus - Cilium	Cluster 14: Myeloid leukemia - Innate immune response	Cluster 34: Ciliated cells - Cilia assembly & function		Plasma membrane, Cytosol	
Cluster 29: Lymphoid tissue & Bone marrow - Innate immune response	Cluster 20: Macrophages & Microglia - Immune response	Cluster 14: Myeloid leukemia - Innate immune response	Cluster 40: Monocytes & Neutrophils - Inflammatory signaling	114	Plasma membrane, Cytosol	Nucleoplasm
Cluster 3: Oligodendrocytes - Myelination	Cluster 22: Non-specific - Mixed function	Cluster 53: Squamous epithelial cells - Keratinization	Cluster 45: Colonocytes & Enterocytes - Gastrointestinal absorption & barrier	4	Plasma membrane	Vesicles
Cluster 64: Non-specific - Transcription	Cluster 35: Non-specific - DNA binding	Cluster 54: Non-specific - Transcription	Cluster 10: Epididymal principal cells - Sperm maturation microenvironment	4	Plasma membrane, Cell Junctions	
Cluster 26: Kidney & Intestine - Metabolism	Cluster 37: Neurons - Mixed function	Cluster 53: Squamous epithelial cells - Keratinization	Cluster 59: Pituitary endocrine cells - Neuroendocrine signaling	11	Cytosol	Plasma membrane
Cluster 53: Liver - Plasma proteins	Cluster 12: Non-specific - Vasculature	Cluster 18: Liver cancer - Metabolism	Cluster 93: Hepatocytes - Bile production & excretion	8	Endoplasmic reticulum, Plasma membrane	
Cluster 61: Non-specific - Transcription	Cluster 14: Neurons - Synaptic function	Cluster 40: Non-specific - Unknown function	Cluster 94: Gonadal somatic cells - Mixed function	84	Nucleoplasm	Plasma membrane
Cluster 10: Brain - Synaptic signal transduction	Cluster 40: Astrocytes - Astrocyte-neuron interactions	Cluster 71: Lymphoma - Inflammatory response	Cluster 14: Oligodendrocyte progenitor cells - Development & myelination	5	Golgi apparatus, Plasma membrane	
Cluster 3: Oligodendrocytes - Myelination	Cluster 28: Oligodendrocytes - Mixed function	Cluster 63: Non-specific - Protein processing	Cluster 31: Neutrophils - Chemotaxis & Apoptosis	35	Plasma membrane, Cytosol	
Cluster 61: Non-specific - Transcription	Cluster 36: Choroid plexus - Mixed function	Cluster 64: Connective tissue cells - ECM organization	Cluster 39: Syncytiotrophoblasts - Placental homeostasis & endocrine signal		Nucleoplasm, Plasma membrane	
Cluster 81: Squamous epithelium - Keratinization		Cluster 55: Lymphoma - Immune response	Cluster 38: Secretory epithelial cells - Exocrine secretion		Plasma membrane, Actin filaments	Cytosol
Cluster 28: Liver & Adrenal gland - Secretion	Cluster 44: Non-specific - Mixed function	Cluster 53: Squamous epithelial cells - Keratinization	Cluster 103: Non-specific - Basic cellular processes	39	Plasma membrane	
Cluster 67: Testis - Mixed function	Cluster 40: Astrocytes - Astrocyte-neuron interactions	Cluster 56: Non-specific - Sensory perception	Cluster 53: Late primary spermatocytes - Meiotic division & transition	24	Vesicles, Plasma membrane	Cell Junctions, Cytosol
Cluster 73: Brain - Neuronal signaling	Cluster 33: Neurons - Mixed function	Cluster 64: Connective tissue cells - ECM organization	Cluster 40: Monocytes & Neutrophils - Inflammatory signaling	36	Plasma membrane	
Cluster 1: Choroid plexus - Transmembrane transport	Cluster 23: Choroid plexus - Mixed function	Cluster 1: Neuronal - Visual perception	Cluster 101: Choroid plexus epithelial cells - Ion transport	3	Nucleoplasm, Plasma membrane	
Cluster 75: Ovary - Mixed function	Cluster 51: Neurons - Mixed function	Cluster 64: Connective tissue cells - ECM organization	Cluster 26: Keratinocytes - Keratinization	19	Plasma membrane, Primary cilium	Mitochondria, Basal body
Cluster 49: Non-specific - Transcription	Cluster 6: Non-specific - Immune response	Cluster 49: HMC-1 - Innate immune response	Cluster 58: Mast cells - Allergic inflammatory responses	73	Plasma membrane, Connecting piece,	Vesicles, Focal adhesion sites, Principal piece, End piece
Cluster 76: Non-specific - Metabolism	Cluster 14: Neurons - Synaptic function	Cluster 30: Non-specific - Gene expression regulation	Cluster 29: Neutrophils - Innate immune cellular maintenance	63	Plasma membrane	Nucleoplasm
Cluster 27: Cerebellum - Synaptic function	Cluster 52: Neurons & Synapses - Synaptic function	Cluster 24: Non-specific - Unknown function	Cluster 59: Pituitary endocrine cells - Neuroendocrine signaling	1	Plasma membrane	Nucleoplasm, Acrosome, Mid piece, Annulus
Cluster 17: Intestine - Digestion	Cluster 48: Non-specific - Mixed function	Cluster 64: Connective tissue cells - ECM organization	Cluster 12: Endothelial cells - Angiogenesis & vascular homeostasis	2	Plasma membrane, Cytosol	Nucleoplasm, Nuclear bodies
Cluster 12: Smooth muscle tissue - ECM organization	Cluster 23: Choroid plexus - Mixed function	Cluster 51: Myeloma - Immunoglobulins	Cluster 9: T-cells - Adaptive immunity: Regulation	3	Plasma membrane	Nucleoli, Cytosol
Cluster 12: Smooth muscle tissue - ECM organization	Cluster 19: Astrocytes - Mixed function	Cluster 72: Neuronal - Signal transduction	Cluster 103: Non-specific - Basic cellular processes	26	Plasma membrane	Vesicles, Actin filaments
Cluster 66: Lymphoid tissue - Immune response	Cluster 8: Immune cells - Immune response	Cluster 31: Non-specific - Antiviral immune response	Cluster 67: Plasma cells - Plasma cell differentiation	30	Golgi apparatus, Plasma membrane	Cytosol
Cluster 8: Pancreas - Proteolysis	Cluster 30: White matter - Myelination	Cluster 72: Neuronal - Signal transduction	Cluster 23: Elongating spermatids - Sperm cell metabolism & fertilization	22	Plasma membrane	
Cluster 2: Non-specific - Mixed function	Cluster 51: Neurons - Mixed function	Cluster 27: Breast cancer - Unknown function	Cluster 49: Apical squamous epithelium - Epithelial barrier & keratinization	108	Plasma membrane, Cytosol	
Cluster 17: Intestine - Digestion	Cluster 42: Choroid plexus - Mitochondria	Cluster 39: Non-specific - Transcription	Cluster 68: Foveolar cells - Mucosal defense	30	Plasma membrane	Cytosol
Cluster 22: Epididymis - Mixed function	Cluster 53: Neurons - Mixed function	Cluster 7: Liver cancer - Metabolism	Cluster 31: Neutrophils - Chemotaxis & Apoptosis	18	Plasma membrane	
Cluster 11: Esophagus - Basic cellular processes	Cluster 49: Hindbrain - Mixed function	Cluster 64: Connective tissue cells - ECM organization	Cluster 2: Cerebellar granular cells - Neuronal signaling	2	Plasma membrane	Vesicles, Cytokinetic bridge
Cluster 3: Oligodendrocytes - Myelination	Cluster 33: Neurons - Mixed function	Cluster 69: Skin cancer - ECM organization	Cluster 81: Alveolar cells - Lung function	7	Plasma membrane, Cytosol	
Cluster 29: Lymphoid tissue & Bone marrow - Innate immune response	Cluster 20: Macrophages & Microglia - Immune response	Cluster 49: HMC-1 - Innate immune response	Cluster 92: NK-cells & T-cells - Adaptive immunity: Cytotoxicity	4	Plasma membrane	
Cluster 34: Lymphoid tissue - Adaptive immune response	Cluster 20: Macrophages & Microglia - Immune response	Cluster 55: Lymphoma - Immune response	Cluster 74: Microglia - Neuroimmunity & neuroinflammation	20	Plasma membrane	Vesicles
Cluster 43: Placenta - Pregnancy hormone signaling	Cluster 1: Neurons - Mixed function	Cluster 9: Skin cancer - Melanin biosynthesis	Cluster 12: Endothelial cells - Angiogenesis & vascular homeostasis	14	Nucleoplasm, Plasma membrane	
Cluster 50: Epididymis - Male reproductive secretion	Cluster 42: Choroid plexus - Mitochondria	Cluster 24: Non-specific - Unknown function	Cluster 48: Neutrophils - Phagocytosis & degranulation	14	Plasma membrane	Nucleoplasm
Cluster 75: Ovary - Mixed function	Cluster 44: Non-specific - Mixed function	Cluster 44: Connective tissue cells - ECM organization	Cluster 91: Pituicytes/FSC - Homeostasis & neuron support	2	Nuclear bodies, Plasma membrane	Nucleoplasm
Cluster 44: Bone marrow - Nuclear processes	Cluster 31: Non-specific - Transcription	Cluster 39: Non-specific - Transcription	Cluster 31: Neutrophils - Chemotaxis & Apoptosis	174	Vesicles, Cytosol	Plasma membrane, Primary cilium, Centriolar satellite, Basal body, Mid piece, Principal pi
Cluster 18: Lymphoid tissue - Immune response	Cluster 20: Macrophages & Microglia - Immune response	Cluster 49: HMC-1 - Innate immune response	Cluster 58: Mast cells - Allergic inflammatory responses	41	Plasma membrane, Cytosol	
Cluster 41: Striated muscle - Muscle contraction	Cluster 30: White matter - Myelination	Cluster 33: Sarcoma - Muscle contraction	Cluster 36: Skeletal myocytes - Muscle excitation & contraction	2	Plasma membrane, Cell Junctions	
Cluster 72: Kidney - Transmembrane transport	Cluster 25: Non-specific - Transcription	Cluster 27: Breast cancer - Unknown function	Cluster 101: Choroid plexus epithelial cells - Ion transport	1	Cell Junctions	
Cluster 11: Esophagus - Basic cellular processes		Cluster 53: Squamous epithelial cells - Keratinization	Cluster 100: Stratified epithelial cells - Barrier functions	4	Plasma membrane, Cytosol	
Cluster 63: Non-specific - Basic cellular processes	Cluster 21: Non-specific - Metabolism	Cluster 42: Non-specific - Unknown function	Cluster 97: Non-specific - Cellular respiration	179	Plasma membrane	Mitochondria
Cluster 10: Brain - Synaptic signal transduction	Cluster 33: Neurons - Mixed function	Cluster 24: Non-specific - Unknown function	Cluster 45: Colonocytes & Enterocytes - Gastrointestinal absorption & barrier	36	Plasma membrane	
Cluster 53: Liver - Plasma proteins		Cluster 18: Liver cancer - Metabolism	Cluster 25: Hepatocytes - Plasma protein synthesis & secretion	7	Plasma membrane	

Cluster 60: Stomach - Digestion	Cluster 35: Non-specific - DNA binding	Cluster 15: Kidney cancer - Membrane components	Cluster 42: Early spermatids - Spermiogenesis: Acrosomal phase	14	Plasma membrane	
Cluster 12: Smooth muscle tissue - ECM organization	Cluster 40: Astrocytes - Astrocyte-neuron interactions	Cluster 21: Lymphoma - Adaptive immune response	Cluster 105: Non-specific - Mixed function	27	Nucleoplasm, Plasma membrane	Primary cilium
Cluster 46: Brain & retina - Neuronal signaling	Cluster 10: Cerebellum - Nucleic acid binding	Cluster 69: Skin cancer - ECM organization	Cluster 79: Photoreceptor cells - Phototransduction	5	Plasma membrane	
Cluster 13: Lung - Secretion	Cluster 43: Hindbrain - Mixed function	Cluster 48: Adipocytes & Endothelial cells - Angiogenesis	Cluster 12: Endothelial cells - Angiogenesis & vascular homeostasis	5	Plasma membrane	
Cluster 12: Smooth muscle tissue - ECM organization	Cluster 42: Choroid plexus - Mitochondria	Cluster 64: Connective tissue cells - ECM organization	Cluster 62: Smooth muscle cells - Muscle contraction	108	Plasma membrane, Actin filaments	
Cluster 7: Small intestine - Absorption and Digestion	Cluster 40: Astrocytes - Astrocyte-neuron interactions	Cluster 15: Kidney cancer - Membrane components	Cluster 71: Non-specific - Cardiac endocrine signaling & maintenanc	7	Plasma membrane, Cytosol	Nuclear speckles
Cluster 14: Adipose tissue - Angiogenesis	Cluster 13: Oligodendrocytes - Mixed function	Cluster 27: Breast cancer - Unknown function	Cluster 32: Endothelial cells - Angiogenesis & vascular immunity		Plasma membrane	
Cluster 41: Striated muscle - Muscle contraction	Cluster 49: Hindbrain - Mixed function	Cluster 53: Squamous epithelial cells - Keratinization	Cluster 100: Stratified epithelial cells - Barrier functions	5	Endoplasmic reticulum, Cytosol	Plasma membrane
Cluster 12: Smooth muscle tissue - ECM organization	Cluster 30: White matter - Myelination	Cluster 72: Neuronal - Signal transduction	Cluster 62: Smooth muscle cells - Muscle contraction	45	Plasma membrane	Vesicles
Cluster 14: Adipose tissue - Angiogenesis	Cluster 47: Endothelial cells - Vasculature	Cluster 48: Adipocytes & Endothelial cells - Angiogenesis	Cluster 47: Platelets - Hemostasis	9	Plasma membrane	Cytosol
Cluster 30: Skeletal muscle - Striated muscle contraction	Cluster 6: Non-specific - Immune response	Cluster 17: Neuroblastoma - Neuronal signaling	Cluster 46: Skeletal myocytes - Muscle structure & formation	2	Golgi apparatus, Plasma membrane	Nucleoplasm
Cluster 74: Non-specific - Signal transduction	Cluster 52: Neurons & Synapses - Synaptic function	Cluster 26: Non-specific - Mitochondria & RNA binding	Cluster 15: Non-specific - Protein processing	19	Plasma membrane	Vesicles
Cluster 68: Non-specific - Basic cellular processes	Cluster 4: Non-specific - mRNA splicing & Cell cycle	Cluster 23: Non-specific - Transcription	Cluster 97: Non-specific - Cellular respiration	35	Plasma membrane, Cytosol	
Cluster 27: Cerebellum - Synaptic function	Cluster 15: Non-specific - Transcription	Cluster 72: Neuronal - Signal transduction	Cluster 51: Secretory epithelial cells - Mixed function	13	Nuclear speckles, Cell Junctions	
Cluster 3: Oligodendrocytes - Myelination	Cluster 11: White matter - Signal transduction	Cluster 26: Non-specific - Mitochondria & RNA binding	Cluster 80: Oligodendrocytes - Myelination	21	Nucleoplasm, Centrosome, Basal body	Plasma membrane
Cluster 66: Lymphoid tissue - Immune response	Cluster 33: Neurons - Mixed function	Cluster 59: Leukemia - T-cell receptor	Cluster 92: NK-cells & T-cells - Adaptive immunity: Cytotoxicity	5	Cell Junctions	
Cluster 41: Striated muscle - Muscle contraction	Cluster 42: Choroid plexus - Mitochondria	Cluster 26: Non-specific - Mitochondria & RNA binding	Cluster 31: Neutrophils - Chemotaxis & Apoptosis	74	Vesicles	Plasma membrane
Cluster 60: Stomach - Digestion	Cluster 41: Monoamines - Neurotransmitter signalling	Cluster 64: Connective tissue cells - ECM organization	Cluster 97: Non-specific - Cellular respiration		Plasma membrane, Cytosol	Nucleoplasm
Cluster 70: Non-specific - Protein processing	Cluster 53: Neurons - Mixed function	Cluster 34: Testis cancer - Unknown function	Cluster 97: Non-specific - Cellular respiration	7	Vesicles	Plasma membrane
Cluster 11: Esophagus - Basic cellular processes	Cluster 53: Neurons - Mixed function	Cluster 3: Rhabdoid - Membrane components	Cluster 20: Non-specific - Transcription regulation	6	Plasma membrane	
Cluster 18: Lymphoid tissue - Immune response		Cluster 20: Myeloma - Immune response	Cluster 40: Monocytes & Neutrophils - Inflammatory signaling	1	Plasma membrane	Nucleoli fibrillar center
Cluster 51: Lymphoid tissue - Adaptive immune response	Cluster 48: Non-specific - Mixed function	Cluster 59: Leukemia - T-cell receptor	Cluster 9: T-cells - Adaptive immunity: Regulation	1	Plasma membrane	
Cluster 66: Lymphoid tissue - Immune response	Cluster 12: Non-specific - Vasculature	Cluster 14: Myeloid leukemia - Innate immune response	Cluster 40: Monocytes & Neutrophils - Inflammatory signaling		Plasma membrane	Primary cilium, Primary cilium tip
Cluster 69: Parathyroid gland - Mixed function	Cluster 39: Astrocytes - Mixed function	Cluster 69: Skin cancer - ECM organization	Cluster 40: Monocytes & Neutrophils - Inflammatory signaling	5	Plasma membrane	Cytosol
Cluster 66: Lymphoid tissue - Immune response	Cluster 12: Non-specific - Vasculature	Cluster 71: Lymphoma - Inflammatory response	Cluster 72: Macrophages - Phagocytosis & lysosomal degradation	2	Plasma membrane	
Cluster 18: Lymphoid tissue - Immune response	Cluster 20: Macrophages & Microglia - Immune response	Cluster 46: Lymphoma - Humoral immune response	Cluster 87: B-cells - Adaptive immunity: Humoral response	3	Plasma membrane	Nucleoplasm, Nucleoli, Mitotic spindle
Cluster 51: Lymphoid tissue - Adaptive immune response	Cluster 8: Immune cells - Immune response	Cluster 59: Leukemia - T-cell receptor	Cluster 9: T-cells - Adaptive immunity: Regulation	15	Plasma membrane	Nucleoplasm, Golgi apparatus
Cluster 66: Lymphoid tissue - Immune response	Cluster 23: Choroid plexus - Mixed function	Cluster 47: Leukemia - Hemostasis	Cluster 72: Macrophages - Phagocytosis & lysosomal degradation	1	Plasma membrane	
Cluster 69: Parathyroid gland - Mixed function	Cluster 38: Sub-cortical - Mixed function	Cluster 59: Leukemia - T-cell receptor	Cluster 47: Platelets - Hemostasis	2	Plasma membrane	Nucleoplasm, Centriolar satellite
Cluster 66: Lymphoid tissue - Immune response	Cluster 56: Hypothalamus - Neuropeptide signaling	Cluster 71: Lymphoma - Inflammatory response	Cluster 82: Dendritic cells - Antigen presentation	60	Nucleoplasm, Nuclear speckles, Plasma membrane	
Cluster 51: Lymphoid tissue - Adaptive immune response	Cluster 20: Macrophages & Microglia - Immune response	Cluster 20: Myeloma - Immune response	Cluster 9: T-cells - Adaptive immunity: Regulation	5	Plasma membrane	
Cluster 77: Intestine - Digestion	Cluster 50: Non-specific - Nucleic acid binding	Cluster 57: Stomach & Colon cancer - Digestion	Cluster 51: Secretory epithelial cells - Mixed function	62	Plasma membrane	Centriolar satellite
Cluster 29: Lymphoid tissue & Bone marrow - Innate immune response	Cluster 20: Macrophages & Microglia - Immune response	Cluster 14: Myeloid leukemia - Innate immune response	Cluster 40: Monocytes & Neutrophils - Inflammatory signaling	24	Plasma membrane	Cytosol
Cluster 51: Lymphoid tissue - Adaptive immune response	Cluster 46: Brainstem - Mixed function	Cluster 46: Lymphoma - Humoral immune response	Cluster 44: Plasma cells - Antibody production & secretion	3	Plasma membrane	
Cluster 80: Thymus - Adaptive immune response	Cluster 8: Immune cells - Immune response	Cluster 59: Leukemia - T-cell receptor	Cluster 9: T-cells - Adaptive immunity: Regulation	38	Endoplasmic reticulum, Plasma memb	Golgi apparatus
Cluster 80: Thymus - Adaptive immune response	Cluster 6: Non-specific - Immune response	Cluster 59: Leukemia - T-cell receptor	Cluster 9: T-cells - Adaptive immunity: Regulation	10	Plasma membrane, Cytosol	
Cluster 51: Lymphoid tissue - Adaptive immune response	Cluster 20: Macrophages & Microglia - Immune response	Cluster 51: Myeloma - Immunoglobulins	Cluster 72: Macrophages - Phagocytosis & lysosomal degradation	10	Plasma membrane	
Cluster 66: Lymphoid tissue - Immune response	Cluster 42: Choroid plexus - Mitochondria	Cluster 55: Lymphoma - Immune response	Cluster 82: Dendritic cells - Antigen presentation	76	Vesicles	Plasma membrane
Cluster 51: Lymphoid tissue - Adaptive immune response	Cluster 6: Non-specific - Immune response	Cluster 59: Leukemia - T-cell receptor	Cluster 9: T-cells - Adaptive immunity: Regulation	1	Golgi apparatus, Plasma membrane	
Cluster 58: Skin - Keratinization	Cluster 39: Astrocytes - Mixed function	Cluster 72: Neuronal - Signal transduction	Cluster 40: Monocytes & Neutrophils - Inflammatory signaling	27	Plasma membrane	Golgi apparatus
Cluster 32: Retina & Lymphoid tissues - Signaling	Cluster 34: Non-specific - Mixed function	Cluster 30: Non-specific - Gene expression regulation	Cluster 51: Secretory epithelial cells - Mixed function	6	Plasma membrane	Vesicles
Cluster 18: Lymphoid tissue - Immune response		Cluster 46: Lymphoma - Humoral immune response	Cluster 102: Non-specific - Mixed function	4	Plasma membrane	Nucleoplasm, Cytosol
Cluster 18: Lymphoid tissue - Immune response	Cluster 20: Macrophages & Microglia - Immune response	Cluster 49: HMC-1 - Innate immune response	Cluster 72: Macrophages - Phagocytosis & lysosomal degradation	1	Plasma membrane	Vesicles
Cluster 66: Lymphoid tissue - Immune response	Cluster 20: Macrophages & Microglia - Immune response	Cluster 21: Lymphoma - Adaptive immune response	Cluster 82: Dendritic cells - Antigen presentation	3	Plasma membrane	Centriolar satellite
Cluster 51: Lymphoid tissue - Adaptive immune response	Cluster 8: Immune cells - Immune response	Cluster 59: Leukemia - T-cell receptor	Cluster 92: NK-cells & T-cells - Adaptive immunity: Cytotoxicity	6	Plasma membrane	
Cluster 80: Thymus - Adaptive immune response	Cluster 49: Hindbrain - Mixed function	Cluster 59: Leukemia - T-cell receptor	Cluster 92: NK-cells & T-cells - Adaptive immunity: Cytotoxicity		Plasma membrane	
Cluster 11: Esophagus - Basic cellular processes	Cluster 11: White matter - Signal transduction	Cluster 48: Adipocytes & Endothelial cells - Angiogenesis	Cluster 45: Colonocytes & Enterocytes - Gastrointestinal absorption & barrier	20	Plasma membrane	
Cluster 75: Ovary - Mixed function	Cluster 48: Non-specific - Mixed function	Cluster 58: Non-specific - Nuclear processes	Cluster 71: Non-specific - Cardiac endocrine signaling & maintenanc	8	Cytosol	Plasma membrane
Cluster 70: Non-specific - Protein processing	Cluster 11: White matter - Signal transduction	Cluster 57: Stomach & Colon cancer - Digestion	Cluster 63: Cytotrophoblasts - Basic cellular functions	20	Plasma membrane	Nucleoplasm, Actin filaments
Cluster 12: Smooth muscle tissue - ECM organization	Cluster 23: Choroid plexus - Mixed function	Cluster 53: Squamous epithelial cells - Keratinization	Cluster 31: Neutrophils - Chemotaxis & Apoptosis	4	Plasma membrane, Actin filaments	
Cluster 11: Esophagus - Basic cellular processes	Cluster 20: Macrophages & Microglia - Immune response	Cluster 45: Non-specific - Nuclear processes	Cluster 29: Neutrophils - Innate immune cellular maintenance	3	Cell Junctions	
Cluster 17: Intestine - Digestion	Cluster 36: Choroid plexus - Mixed function	Cluster 53: Squamous epithelial cells - Keratinization	Cluster 100: Stratified epithelial cells - Barrier functions	110	Plasma membrane, Cell Junctions	Golgi apparatus
Cluster 12: Smooth muscle tissue - ECM organization	Cluster 14: Neurons - Synaptic function	Cluster 64: Connective tissue cells - ECM organization	Cluster 46: Skeletal myocytes - Muscle structure & formation	3	Plasma membrane	
Cluster 19: Skeletal muscle - Striated muscle contraction	Cluster 35: Non-specific - DNA binding	Cluster 33: Sarcoma - Muscle contraction	Cluster 46: Skeletal myocytes - Muscle structure & formation	5	Golgi apparatus, Plasma membrane	
Cluster 77: Intestine - Digestion	Cluster 48: Non-specific - Mixed function	Cluster 57: Stomach & Colon cancer - Digestion	Cluster 45: Colonocytes & Enterocytes - Gastrointestinal absorption & barrier	1	Cell Junctions	Nucleoplasm
Cluster 35: Non-specific - Mixed function	Cluster 40: Astrocytes - Astrocyte-neuron interactions	Cluster 15: Kidney cancer - Membrane components	Cluster 60: Cardiomyocytes - Cardiac muscle contraction & structure	30	Plasma membrane	Cell Junctions
Cluster 11: Esophagus - Basic cellular processes	Cluster 11: White matter - Signal transduction	Cluster 66: Non-specific - Mitochondria	Cluster 38: Secretory epithelial cells - Exocrine secretion		Plasma membrane	Microtubules
Cluster 44: Bone marrow - Nuclear processes	Cluster 36: Choroid plexus - Mixed function	Cluster 53: Squamous epithelial cells - Keratinization	Cluster 26: Keratinocytes - Keratinization	5	Plasma membrane, Cell Junctions	
Cluster 78: Brain - Neuronal signaling	Cluster 56: Hypothalamus - Neuropeptide signaling	Cluster 72: Neuronal - Signal transduction	Cluster 35: Neurons - Synaptic function & neurogenesis	2	Plasma membrane	
Cluster 14: Adipose tissue - Angiogenesis	Cluster 47: Endothelial cells - Vasculature	Cluster 48: Adipocytes & Endothelial cells - Angiogenesis	Cluster 32: Endothelial cells - Angiogenesis & vascular immunity	19	Plasma membrane	Nucleoplasm, Nuclear membrane
Cluster 72: Kidney - Transmembrane transport	Cluster 38: Sub-cortical - Mixed function	Cluster 15: Kidney cancer - Membrane components	Cluster 88: Proximal tubular cells - Transmembrane transport	1	Plasma membrane, Cell Junctions	
Cluster 19: Skeletal muscle - Striated muscle contraction	Cluster 32: Cerebral cortex - Mixed function	Cluster 36: Non-specific - mRNA processing	Cluster 65: Non-specific - Cell growth & division	35	Microtubules, Cytosol	Plasma membrane
Cluster 78: Brain - Neuronal signaling	Cluster 14: Neurons - Synaptic function	Cluster 39: Non-specific - Transcription	Cluster 98: Neurons - Neurotransmission & signaling	91	Nucleoplasm, Plasma membrane	Cell Junctions
Cluster 17: Intestine - Digestion	Cluster 48: Non-specific - Mixed function	Cluster 16: Thyroid cancer - Neuronal signaling	Cluster 45: Colonocytes & Enterocytes - Gastrointestinal absorption & barrier	1	Plasma membrane	
Cluster 17: Intestine - Digestion		Cluster 57: Stomach & Colon cancer - Digestion	Cluster 45: Colonocytes & Enterocytes - Gastrointestinal absorption & barrier	1	Plasma membrane	Cytosol
Cluster 58: Skin - Keratinization	Cluster 46: Brainstem - Mixed function	Cluster 15: Kidney cancer - Membrane components	Cluster 100: Stratified epithelial cells - Barrier functions	1	Plasma membrane	Nucleoplasm
Cluster 14: Adipose tissue - Angiogenesis	Cluster 29: Forebrain - Mixed function	Cluster 57: Stomach & Colon cancer - Digestion	Cluster 92: NK-cells & T-cells - Adaptive immunity: Cytotoxicity	7	Plasma membrane	Vesicles, Cytosol
Cluster 14: Adipose tissue - Angiogenesis	Cluster 25: Non-specific - Transcription	Cluster 55: Lymphoma - Immune response	Cluster 24: Neutrophils - Innate immunity signal integration	34	Nucleoplasm, Plasma membrane, Cytc	Nuclear bodies
Cluster 77: Intestine - Digestion	Cluster 26: Oligodendrocytes - Mixed function	Cluster 53: Squamous epithelial cells - Keratinization	Cluster 45: Colonocytes & Enterocytes - Gastrointestinal absorption & barrier	28	Plasma membrane, Cell Junctions	
Cluster 76: Non-specific - Metabolism	Cluster 42: Choroid plexus - Mitochondria	Cluster 15: Kidney cancer - Membrane components	Cluster 50: Renal epithelial cells - Ion transport and pH regulation	10	Cell Junctions	Nuclear bodies
Cluster 39: Testis - Nuclear processes	Cluster 10: Cerebellum - Nucleic acid binding	Cluster 64: Connective tissue cells - ECM organization	Cluster 12: Endothelial cells - Angiogenesis & vascular homeostasis	53	Vesicles, Plasma membrane	
Cluster 82: Salivary gland - Secretion	Cluster 40: Astrocytes - Astrocyte-neuron interactions	Cluster 56: Non-specific - Sensory perception	Cluster 38: Secretory epithelial cells - Exocrine secretion	4	Plasma membrane	Nucleoli, Vesicles, Primary cilium tip
Cluster 34: Lymphoid tissue - Adaptive immune response	Cluster 33: Neurons - Mixed function	Cluster 10: Ciliated cells - Ion channel activity	Cluster 70: Intestinal goblet cells - Mucin production	2	Golgi apparatus	Nucleoplasm, Plasma membrane, Primary cilium, Primary cilium tip
Cluster 19: Skeletal muscle - Striated muscle contraction	Cluster 29: Forebrain - Mixed function	Cluster 33: Sarcoma - Muscle contraction	Cluster 46: Skeletal myocytes - Muscle structure & formation	1	Plasma membrane	
Cluster 38: Non-specific - Basic cellular processes	Cluster 38: Sub-cortical - Mixed function	Cluster 60: Prostate cancer - Unknown function	Cluster 16: Prostatic glandular cells - Androgen response & seminal fluid		Plasma membrane	Nucleoplasm, Cytokinetic bridge
Cluster 32: Retina & Lymphoid tissues - Signaling	Cluster 38: Sub-cortical - Mixed function	Cluster 17: Neuroblastoma - Neuronal signaling	Cluster 99: Retinal pigment epithelial cells - Retinol metabolism	1	Nuclear speckles, Plasma membrane	Primary cilium tip, Primary cilium transition zone, Basal body, Cytosol
Cluster 21: Testis - Mixed function	Cluster 38: Sub-cortical - Mixed function	Cluster 58: Non-specific - Nuclear processes	Cluster 41: Oocytes - Oogenesis	26	Focal adhesion sites	Plasma membrane
Cluster 39: Testis - Nuclear processes	Cluster 46: Brainstem - Mixed function	Cluster 17: Neuroblastoma - Neuronal signaling	Cluster 33: Early spermatids - Spermiogenesis: Cap phase		Endoplasmic reticulum	Cell Junctions
Cluster 19: Skeletal muscle - Striated muscle contraction		Cluster 33: Sarcoma - Muscle contraction	Cluster 46: Skeletal myocytes - Muscle structure & formation	7	Plasma membrane	
Cluster 70: Non-specific - Protein processing	Cluster 8: Immune cells - Immune response	Cluster 15: Kidney cancer - Membrane components	Cluster 23: Elongating spermatids - Sperm cell metabolism & fertilization	45	Nucleoplasm, Plasma membrane	Nuclear bodies
Cluster 81: Squamous epithelium - Keratinization	Cluster 28: Oligodendrocytes - Mixed function	Cluster 53: Squamous epithelial cells - Keratinization	Cluster 85: Squamous epithelial cells - Keratinization		Plasma membrane, Cell Junctions	Nucleoplasm
Cluster 76: Non-specific - Metabolism	Cluster 41: Monoamines - Neurotransmitter signalling	Cluster 33: Sarcoma - Muscle contraction	Cluster 50: Renal epithelial cells - Ion transport and pH regulation	9	Golgi apparatus, Plasma membrane	Cytosol
Cluster 3: Oligodendrocytes - Myelination	Cluster 30: White matter - Myelination	Cluster 48: Adipocytes & Endothelial cells - Angiogenesis	Cluster 94: Gonadal somatic cells - Mixed function	9	Lipid droplets, Cell Junctions	
Cluster 72: Kidney - Transmembrane transport	Cluster 36: Choroid plexus - Mixed function	Cluster 18: Liver cancer - Metabolism	Cluster 21: Extravillous trophoblasts - Placental invasion & endocrine regulatio		Plasma membrane	
Cluster 1: Choroid plexus - Transmembrane transport	Cluster 36: Choroid plexus - Mixed function	Cluster 15: Kidney cancer - Membrane components	Cluster 38: Secretory epithelial cells - Exocrine secretion	20	Plasma membrane, Cell Junctions	Nucleoplasm, Nucleoli, Vesicles
Cluster 40: Non-specific - Mixed function	Cluster 6: Non-specific - Immune response	Cluster 56: Non-specific - Sensory perception	Cluster 60: Cardiomyocytes - Cardiac muscle contraction & structure	4	Golgi apparatus, Plasma membrane	Cytosol
Cluster 17: Intestine - Digestion	Cluster 36: Choroid plexus - Mixed function	Cluster 57: Stomach & Colon cancer - Digestion	Cluster 45: Colonocytes & Enterocytes - Gastrointestinal absorption & barrier		Cell Junctions	
Cluster 11: Esophagus - Basic cellular processes	Cluster 17: Choroid plexus - Cilium	Cluster 53: Squamous epithelial cells - Keratinization	Cluster 100: Stratified epithelial cells - Barrier functions	14	Plasma membrane	
Cluster 11: Esophagus - Basic cellular processes	Cluster 6: Non-specific - Immune response	Cluster 53: Squamous epithelial cells - Keratinization	Cluster 100: Stratified epithelial cells - Barrier functions	61	Vesicles	Cell Junctions
Cluster 71: Cerebellum - Nervous system development	Cluster 37: Neurons - Mixed function	Cluster 10: Ciliated cells - Ion channel activity	Cluster 48: Neutrophils - Phagocytosis & degranulation	17	Cell Junctions	Vesicles
Cluster 15: Bone marrow - Innate immune response	Cluster 12: Non-specific - Vasculature	Cluster 14: Myeloid leukemia - Innate immune response	Cluster 82: Dendritic cells - Antigen presentation		Plasma membrane, Cytosol	
Cluster 66: Lymphoid tissue - Immune response	Cluster 42: Choroid plexus - Mitochondria	Cluster 51: Myeloma - Immunoglobulins	Cluster 82: Dendritic cells - Antigen presentation	2	Plasma membrane, Cytosol	Nucleoplasm
Cluster 12: Smooth muscle tissue - ECM organization	Cluster 13: Oligodendrocytes - Mixed function	Cluster 64: Connective tissue cells - ECM organization	Cluster 12: Endothelial cells - Angiogenesis & vascular homeostasis	7	Plasma membrane, Centrosome	Calyx, Connecting piece, Principal piece
Cluster 62: Skeletal muscle - Muscle contraction	Cluster 40: Astrocytes - Astrocyte-neuron interactions	Cluster 15: Kidney cancer - Membrane components	Cluster 57: Podocytes - Glomerular filtration	1	Plasma membrane	Nuclear speckles
Cluster 68: Non-specific - Basic cellular processes	Cluster 27: Non-specific - Mixed function	Cluster 53: Squamous epithelial cells - Keratinization	Cluster 63: Cytotrophoblasts - Basic cellular functions	208	Endosomes, Lysosomes	Golgi apparatus, Plasma membrane, Mitotic spindle, Primary cilium
Cluster 68: Non-specific - Basic cellular processes	Cluster 52: Neurons & Synapses - Synaptic function	Cluster 53: Squamous epithelial cells - Keratinization	Cluster 85: Squamous epithelial cells - Keratinization	79	Vesicles	Plasma membrane, Cytosol
Cluster 1: Choroid plexus - Transmembrane transport	Cluster 36: Choroid plexus - Mixed function	Cluster 64: Connective tissue cells - ECM organization	Cluster 101: Choroid plexus epithelial cells - Ion transport		Plasma membrane	Nucleoplasm, Vesicles
Cluster 42: Retina - Visual perception	Cluster 10: Cerebellum - Nucleic acid binding	Cluster 53: Squamous epithelial cells - Keratinization	Cluster 79: Photoreceptor cells - Phototransduction		Nucleoplasm, Vesicles, Mid piece, Prin	Plasma membrane, Mitotic spindle, Primary cilium, Primary cilium tip
Cluster 25: Non-specific - Basic cellular processes	Cluster 15: Non-specific - Transcription	Cluster 57: Stomach & Colon cancer - Digestion	Cluster 79: Photoreceptor cells - Phototransduction	29	Actin filaments	Plasma membrane
Cluster 74: Non-specific - Signal transduction	Cluster 51: Neurons - Mixed function	Cluster 46: Lymphoma - Humoral immune response	Cluster 110: Mural cells - ECM organization	3	Plasma membrane	Actin filaments
Cluster 70: Non-specific - Protein processing	Cluster 31: Non-specific - Transcription	Cluster 19: Non-specific - Unknown function	Cluster 8: Non-specific - Secretory pathway	22	Golgi apparatus, Plasma membrane	Nucleoplasm, Cytosol
Cluster 37: Skin - Keratinization	Cluster 15: Non-specific - Transcription	Cluster 53: Squamous epithelial cells - Keratinization	Cluster 100: Stratified epithelial cells - Barrier functions	14	Plasma membrane	Golgi apparatus
Cluster 12: Smooth muscle tissue - ECM organization	Cluster 3: Choroid plexus - Cilium	Cluster 64: Connective tissue cells - ECM organization	Cluster 108: Fibroblasts - ECM organization		Cytosol	Plasma membrane
Cluster 11: Esophagus - Basic cellular processes	Cluster 20: Macrophages & Microglia - Immune response	Cluster 24: Non-specific - Unknown function	Cluster 45: Colonocytes & Enterocytes - Gastrointestinal absorption & barrier	25	Plasma membrane, Cytosol	
Cluster 76: Non-specific - Metabolism	Cluster 30: White matter - Myelination	Cluster 49: HMC-1 - Innate immune response	Cluster 24: Neutrophils - Innate immunity signal integration	2	Cytosol	Plasma membrane
Cluster 66: Lymphoid tissue - Immune response	Cluster 11: White matter - Signal transduction	Cluster 55: Lymphoma - Immune response	Cluster 24: Neutrophils - Innate immunity signal integration	1	Plasma membrane	
Cluster 60: Stomach - Digestion	Cluster 5: Non-specific - Mixed function	Cluster 53: Squamous epithelial cells - Keratinization	Cluster 45: Colonocytes & Enterocytes - Gastrointestinal absorption & barrier	7	Cell Junctions	
Cluster 1: Choroid plexus - Transmembrane transport	Cluster 23: Choroid plexus - Mixed function	Cluster 16: Thyroid cancer - Neuronal signaling	Cluster 71: Non-specific - Cardiac endocrine signaling & maintenanc		Plasma membrane	
Cluster 41: Striated muscle - Muscle contraction	Cluster 28: Oligodendrocytes - Mixed function	Cluster 64: Connective tissue cells - ECM organization	Cluster 61: Schwann cells & Melanocytes - Mixed function	74	Plasma membrane, Cytosol	
Cluster 14: Adipose tissue - Angiogenesis	Cluster 13: Oligodendrocytes - Mixed function	Cluster 7: Liver cancer - Metabolism	Cluster 58: Mast cells - Allergic inflammatory responses	7	Plasma membrane	Nuclear bodies
Cluster 66: Lymphoid tissue - Immune response	Cluster 20: Macrophages & Microglia - Immune response	Cluster 14: Myeloid leukemia - Innate immune response	Cluster 72: Macrophages - Phagocytosis & lysosomal degradation	19	Vesicles, Plasma membrane	
Cluster 24: Non-specific - Transcription	Cluster 1: Neurons - Mixed function	Cluster 27: Breast cancer - Unknown function	Cluster 29: Neutrophils - Innate immune cellular maintenance	80	Plasma membrane, Cytosol	Actin filaments, Primary cilium, Principal piece, End piece
Cluster 11: Esophagus - Basic cellular processes	Cluster 11: White matter - Signal transduction	Cluster 22: Non-specific - mRNA splicing & Cell cycle	Cluster 51: Secretory epithelial cells - Mixed function	73	Plasma membrane, Cell Junctions	Golgi apparatus, Vesicles, Connecting piece, Mid piece, Principal piece
Cluster 75: Ovary - Mixed function	Cluster 44: Non-specific - Mixed function	Cluster 9: Skin cancer - Melanin biosynthesis	Cluster 40: Monocytes & Neutrophils - Inflammatory signaling	443	Plasma membrane	Cell Junctions, Primary cilium, Basal body
Cluster 11: Esophagus - Basic cellular processes	Cluster 8: Immune cells - Immune response	Cluster 53: Squamous epithelial cells - Keratinization	Cluster 102: Non-specific - Mixed function	67	Plasma membrane	Basal body
Cluster 74: Non-specific - Signal transduction	Cluster 27: Non-specific - Mixed function	Cluster 24: Non-specific - Unknown function	Cluster 47: Platelets - Hemostasis	154	Vesicles, Plasma membrane	Golgi apparatus
Cluster 74: Non-specific - Signal transduction	Cluster 51: Neurons - Mixed function	Cluster 18: Liver cancer - Metabolism	Cluster 32: Endothelial cells - Angiogenesis & vascular immunity	9	Plasma membrane	
Cluster 73: Brain - Neuronal signaling	Cluster 20: Macrophages & Microglia - Immune response	Cluster 51: Myeloma - Immunoglobulins	Cluster 74: Microglia - Neuroimmunity & neuroinflammation		Plasma membrane	
Cluster 77: Intestine - Digestion	Cluster 30: White matter - Myelination	Cluster 4: Non-specific - Transcription	Cluster 69: Urothelial cells - Basic cellular functions	11	Plasma membrane, Cell Junctions	Nucleoplasm
Cluster 66: Lymphoid tissue - Immune response	Cluster 12: Non-specific - Vasculature	Cluster 14: Myeloid leukemia - Innate immune response	Cluster 24: Neutrophils - Innate immunity signal integration	13	Plasma membrane	Vesicles, Microtubules, Mitotic spindle, Primary cilium, Primary cilium tip, Basal body
Cluster 75: Ovary - Mixed function	Cluster 39: Astrocytes - Mixed function	Cluster 44: Connective tissue cells - ECM organization	Cluster 108: Fibroblasts - ECM organization	22	Plasma membrane	
Cluster 47: Testis - Mixed function	Cluster 6: Non-specific - Immune response	Cluster 56: Non-specific - Sensory perception	Cluster 42: Early spermatids - Spermiogenesis: Acrosomal phase	23	Cytosol	Golgi apparatus, Plasma membrane
Cluster 66: Lymphoid tissue - Immune response	Cluster 20: Macrophages & Microglia - Immune response	Cluster 71: Lymphoma - Inflammatory response	Cluster 24: Neutrophils - Innate immunity signal integration	21	Plasma membrane	Cytokinetic bridge, Centriolar satellite, Cytosol
Cluster 4: Adrenal gland - Steroid metabolism	Cluster 20: Macrophages & Microglia - Immune response	Cluster 9: Skin cancer - Melanin biosynthesis	Cluster 72: Macrophages - Phagocytosis & lysosomal degradation	33	Vesicles, Plasma membrane	Nucleoli fibrillar center
Cluster 63: Non-specific - Basic cellular processes	Cluster 39: Astrocytes - Mixed function	Cluster 69: Skin cancer - ECM organization	Cluster 36: Skeletal myocytes - Muscle excitation & contraction	39	Vesicles, Plasma membrane	Nucleoplasm
Cluster 24: Non-specific - Transcription	Cluster 51: Neurons - Mixed function	Cluster 6: Leukemia - Signal transduction	Cluster 74: Microglia - Neuroimmunity & neuroinflammation	2	Plasma membrane	Nucleoplasm
Cluster 81: Squamous epithelium - Keratinization	Cluster 41: Monoamines - Neurotransmitter signalling	Cluster 67: Non-specific - Unknown function	Cluster 47: Platelets - Hemostasis	36	Vesicles, Plasma membrane	
Cluster 37: Skin - Keratinization	Cluster 33: Neurons - Mixed function	Cluster 9: Skin cancer - Melanin biosynthesis	Cluster 61: Schwann cells & Melanocytes - Mixed function	7	Plasma membrane	Vesicles
Cluster 68: Non-specific - Basic cellular processes	Cluster 19: Astrocytes - Mixed function	Cluster 53: Squamous epithelial cells - Keratinization	Cluster 64: Renal epithelial cells - Ion transport & homeostasis	13	Plasma membrane	Nucleoplasm, Cytosol

Cluster 12: Smooth muscle tissue - ECM organization	Cluster 39: Astrocytes - Mixed function	Cluster 44: Connective tissue cells - ECM organization	Cluster 103: Non-specific - Basic cellular processes	37	Plasma membrane, Actin filaments	
Cluster 74: Non-specific - Signal transduction	Cluster 33: Neurons - Mixed function	Cluster 53: Squamous epithelial cells - Keratinization	Cluster 69: Urothelial cells - Basic cellular functions	1	Plasma membrane	Vesicles
Cluster 39: Testis - Nuclear processes		Cluster 19: Non-specific - Unknown function	Cluster 27: Non-specific - Cell proliferation	8	Plasma membrane, Microtubules	
Cluster 32: Retina & Lymphoid tissues - Signaling	Cluster 6: Non-specific - Immune response	Cluster 47: Leukemia - Hemostasis	Cluster 92: NK-cells & T-cells - Adaptive immunity: Cytotoxicity	8	Nucleoplasm	Plasma membrane, Actin filaments, Focal adhesion sites, Mitochondria
Cluster 3: Oligodendrocytes - Myelination	Cluster 11: White matter - Signal transduction	Cluster 72: Neuronal - Signal transduction	Cluster 14: Oligodendrocyte progenitor cells - Development & myelination	75	Vesicles	Plasma membrane, Basal body
Cluster 46: Brain & retina - Neuronal signaling	Cluster 51: Neurons - Mixed function	Cluster 5: Rhabdoid - Unknown function	Cluster 79: Photoreceptor cells - Phototransduction	78	Nucleoplasm, Centrosome, Basal body	Plasma membrane, Primary cilium, Primary cilium tip, Cytosol
Cluster 4: Adrenal gland - Steroid metabolism	Cluster 44: Non-specific - Mixed function	Cluster 40: Non-specific - Unknown function	Cluster 102: Non-specific - Mixed function	18	Cell Junctions	
Cluster 35: Non-specific - Mixed function	Cluster 28: Oligodendrocytes - Mixed function	Cluster 64: Connective tissue cells - ECM organization	Cluster 33: Early spermatids - Spermiogenesis: Cap phase	60	Nucleoplasm	Plasma membrane, Cytosol
Cluster 44: Bone marrow - Nuclear processes	Cluster 37: Neurons - Mixed function	Cluster 52: Non-specific - Mitochondria	Cluster 27: Non-specific - Cell proliferation	47	Nucleoplasm	Plasma membrane, Cytosol
Cluster 36: Brain & Liver - Signaling	Cluster 51: Neurons - Mixed function	Cluster 48: Adipocytes & Endothelial cells - Angiogenesis	Cluster 74: Microglia - Neuroimmunity & neuroinflammation	9	Nucleoli, Plasma membrane	Golgi apparatus, Cytosol
Cluster 51: Lymphoid tissue - Adaptive immune response	Cluster 20: Macrophages & Microglia - Immune response	Cluster 46: Lymphoma - Humoral immune response	Cluster 74: Microglia - Neuroimmunity & neuroinflammation	19	Plasma membrane, Cytosol	
Cluster 66: Lymphoid tissue - Immune response	Cluster 12: Non-specific - Vasculature	Cluster 14: Myeloid leukemia - Innate immune response	Cluster 92: NK-cells & T-cells - Adaptive immunity: Cytotoxicity	17	Plasma membrane	
Cluster 66: Lymphoid tissue - Immune response	Cluster 49: Hindbrain - Mixed function	Cluster 6: Leukemia - Signal transduction	Cluster 29: Neutrophils - Innate immune cellular maintenance	8	Plasma membrane	Nucleoplasm, Cytokinetic bridge, Cytosol
Cluster 60: Stomach - Digestion	Cluster 46: Brainstem - Mixed function	Cluster 57: Stomach & Colon cancer - Digestion	Cluster 1: Enterocytes - Absorption and Digestion	21	Nucleoplasm, Cell Junctions	
Cluster 64: Non-specific - Transcription	Cluster 37: Neurons - Mixed function	Cluster 51: Myeloma - Immunoglobulins	Cluster 79: Photoreceptor cells - Phototransduction	10	Plasma membrane	Centrosome
Cluster 11: Esophagus - Basic cellular processes	Cluster 39: Astrocytes - Mixed function	Cluster 53: Squamous epithelial cells - Keratinization	Cluster 85: Squamous epithelial cells - Keratinization	9	Plasma membrane	Endoplasmic reticulum
Cluster 58: Skin - Keratinization	Cluster 23: Choroid plexus - Mixed function	Cluster 53: Squamous epithelial cells - Keratinization	Cluster 100: Stratified epithelial cells - Barrier functions	10	Plasma membrane, Cell Junctions	
Cluster 77: Intestine - Digestion	Cluster 36: Choroid plexus - Mixed function	Cluster 53: Squamous epithelial cells - Keratinization	Cluster 69: Urothelial cells - Basic cellular functions	29	Cell Junctions	Vesicles, Plasma membrane
Cluster 58: Skin - Keratinization	Cluster 42: Choroid plexus - Mitochondria	Cluster 53: Squamous epithelial cells - Keratinization	Cluster 85: Squamous epithelial cells - Keratinization	45	Cell Junctions	
Cluster 25: Non-specific - Basic cellular processes	Cluster 39: Astrocytes - Mixed function	Cluster 16: Thyroid cancer - Neuronal signaling	Cluster 28: Astrocytes - Homeostasis & neuron support	21	Cell Junctions, Intermediate filaments	Nucleoplasm
Cluster 63: Non-specific - Basic cellular processes	Cluster 11: White matter - Signal transduction	Cluster 72: Neuronal - Signal transduction	Cluster 48: Neutrophils - Phagocytosis & degranulation	47	Plasma membrane, Centriolar satellite, Mid piece	
Cluster 14: Adipose tissue - Angiogenesis	Cluster 47: Endothelial cells - Vasculature	Cluster 48: Adipocytes & Endothelial cells - Angiogenesis	Cluster 32: Endothelial cells - Angiogenesis & vascular immunity	1	Vesicles, Plasma membrane	Nucleoplasm
Cluster 75: Ovary - Mixed function	Cluster 47: Endothelial cells - Vasculature	Cluster 33: Sarcoma - Muscle contraction	Cluster 110: Mural cells - ECM organization	27	Plasma membrane, Cytosol	
Cluster 43: Placenta - Pregnancy hormone signaling	Cluster 19: Astrocytes - Mixed function	Cluster 51: Myeloma - Immunoglobulins	Cluster 17: Non-specific - RNA processing	1	Plasma membrane	Cytosol
Cluster 58: Skin - Keratinization	Cluster 40: Astrocytes - Astrocyte-neuron interactions	Cluster 53: Squamous epithelial cells - Keratinization	Cluster 32: Endothelial cells - Angiogenesis & vascular immunity	30	Plasma membrane	
Cluster 42: Retina - Visual perception	Cluster 24: Neurons - Mixed function	Cluster 40: Non-specific - Unknown function	Cluster 86: Retinal bipolar cells - Synaptic signal transduction	5	Plasma membrane, Cytosol	
Cluster 10: Brain - Synaptic signal transduction	Cluster 24: Neurons - Mixed function	Cluster 10: Ciliated cells - Ion channel activity	Cluster 99: Retinal pigment epithelial cells - Retinol metabolism	4	Plasma membrane	Actin filaments, Cytosol
Cluster 11: Esophagus - Basic cellular processes	Cluster 19: Astrocytes - Mixed function	Cluster 53: Squamous epithelial cells - Keratinization	Cluster 108: Fibroblasts - ECM organization	926	Golgi apparatus, Plasma membrane	Cell Junctions, Primary cilium, Basal body, Mid piece, Principal piece, End piece
Cluster 75: Ovary - Mixed function	Cluster 38: Sub-cortical - Mixed function	Cluster 3: Rhabdoid - Membrane components	Cluster 89: Adipocytes - Lipid metabolism	5	Plasma membrane	Cytosol
Cluster 39: Testis - Nuclear processes	Cluster 25: Non-specific - Transcription	Cluster 55: Lymphoma - Immune response	Cluster 31: Neutrophils - Chemotaxis & Apoptosis	46	Plasma membrane	Primary cilium, Primary cilium transition zone
Cluster 12: Smooth muscle tissue - ECM organization	Cluster 19: Astrocytes - Mixed function	Cluster 64: Connective tissue cells - ECM organization	Cluster 110: Mural cells - ECM organization	4	Plasma membrane	Primary cilium, Primary cilium transition zone
Cluster 10: Brain - Synaptic signal transduction	Cluster 51: Neurons - Mixed function	Cluster 20: Myeloma - Immune response	Cluster 32: Endothelial cells - Angiogenesis & vascular immunity	14	Plasma membrane	Primary cilium, Primary cilium transition zone
Cluster 82: Salivary gland - Secretion	Cluster 56: Hypothalamus - Neuropeptide signaling	Cluster 10: Ciliated cells - Ion channel activity	Cluster 70: Intestinal goblet cells - Mucin production		Plasma membrane	
Cluster 12: Smooth muscle tissue - ECM organization	Cluster 19: Astrocytes - Mixed function	Cluster 5: Rhabdoid - Unknown function	Cluster 62: Smooth muscle cells - Muscle contraction	31	Plasma membrane, Focal adhesion sites, Cytosol	
Cluster 14: Adipose tissue - Angiogenesis	Cluster 47: Endothelial cells - Vasculature	Cluster 64: Connective tissue cells - ECM organization	Cluster 32: Endothelial cells - Angiogenesis & vascular immunity	11	Plasma membrane	
Cluster 68: Non-specific - Basic cellular processes	Cluster 21: Non-specific - Metabolism	Cluster 15: Kidney cancer - Membrane components	Cluster 11: Muller glia cells - Retinal homeostasis	95	Plasma membrane, Cytosol	
Cluster 46: Brain & retina - Neuronal signaling	Cluster 52: Neurons & Synapses - Synaptic function	Cluster 15: Kidney cancer - Membrane components	Cluster 79: Photoreceptor cells - Phototransduction	12	Plasma membrane, Cytosol	
Cluster 30: Skeletal muscle - Striated muscle contraction	Cluster 25: Non-specific - Transcription	Cluster 33: Sarcoma - Muscle contraction	Cluster 71: Non-specific - Cardiac endocrine signaling & maintenanc	11	Plasma membrane, Cytosol	
Cluster 61: Non-specific - Transcription	Cluster 28: Oligodendrocytes - Mixed function	Cluster 33: Sarcoma - Muscle contraction	Cluster 82: Dendritic cells - Antigen presentation	6	Plasma membrane	
Cluster 35: Non-specific - Mixed function	Cluster 11: White matter - Signal transduction	Cluster 36: Non-specific - mRNA processing	Cluster 82: Dendritic cells - Antigen presentation		Cytosol	Plasma membrane
Cluster 36: Brain & Liver - Signaling	Cluster 1: Neurons - Mixed function	Cluster 53: Squamous epithelial cells - Keratinization	Cluster 50: Renal epithelial cells - Ion transport and pH regulation	19	Plasma membrane	
Cluster 46: Brain & retina - Neuronal signaling	Cluster 30: White matter - Myelination	Cluster 57: Stomach & Colon cancer - Digestion	Cluster 74: Microglia - Neuroimmunity & neuroinflammation	46	Plasma membrane	Nucleoplasm, Cell Junctions
Cluster 3: Oligodendrocytes - Myelination	Cluster 52: Neurons & Synapses - Synaptic function	Cluster 48: Adipocytes & Endothelial cells - Angiogenesis	Cluster 40: Monocytes & Neutrophils - Inflammatory signaling	59	Plasma membrane, Cell Junctions	Primary cilium, Basal body
Cluster 76: Non-specific - Metabolism	Cluster 2: Neurons - Nucleosome	Cluster 15: Kidney cancer - Membrane components	Cluster 57: Podocytes - Glomerular filtration	22	Plasma membrane	
Cluster 77: Intestine - Digestion	Cluster 56: Hypothalamus - Neuropeptide signaling	Cluster 57: Stomach & Colon cancer - Digestion	Cluster 45: Colonocytes & Enterocytes - Gastrointestinal absorption & barrier	7	Plasma membrane	
Cluster 81: Squamous epithelium - Keratinization	Cluster 42: Choroid plexus - Mitochondria	Cluster 53: Squamous epithelial cells - Keratinization	Cluster 100: Stratified epithelial cells - Barrier functions	128	Plasma membrane, Cell Junctions	Nuclear speckles, Golgi apparatus
Cluster 14: Adipose tissue - Angiogenesis	Cluster 24: Neurons - Mixed function	Cluster 72: Neuronal - Signal transduction	Cluster 86: Retinal bipolar cells - Synaptic signal transduction	17	Nuclear membrane, Plasma membrane	Nucleoplasm, Actin filaments
Cluster 73: Brain - Neuronal signaling	Cluster 51: Neurons - Mixed function	Cluster 5: Rhabdoid - Unknown function	Cluster 35: Neurons - Synaptic function & neurogenesis	13	Golgi apparatus	Vesicles, Plasma membrane
Cluster 27: Cerebellum - Synaptic function	Cluster 2: Neurons - Nucleosome	Cluster 50: Lymphoma - Unknown function	Cluster 14: Oligodendrocyte progenitor cells - Development & myelination	10	Endoplasmic reticulum, Plasma memb	Cytosol
Cluster 77: Intestine - Digestion	Cluster 30: White matter - Myelination	Cluster 72: Neuronal - Signal transduction	Cluster 54: Intestinal epithelial cells - Intestinal epi homeostasis & respiration	25	Plasma membrane	Nucleoplasm
Cluster 4: Adrenal gland - Steroid metabolism	Cluster 16: Non-specific - Transcription	Cluster 47: Leukemia - Hemostasis	Cluster 66: Erythroid cells - Erythroid differentiation and maturation	20	Plasma membrane	Nuclear speckles
Cluster 11: Esophagus - Basic cellular processes	Cluster 39: Astrocytes - Mixed function	Cluster 27: Breast cancer - Unknown function	Cluster 64: Renal epithelial cells - Ion transport & homeostasis	202	Plasma membrane	Nucleoplasm, Cytosol
Cluster 77: Intestine - Digestion	Cluster 11: White matter - Signal transduction	Cluster 69: Skin cancer - ECM organization	Cluster 80: Oligodendrocytes - Myelination	102	Plasma membrane	Actin filaments
Cluster 3: Oligodendrocytes - Myelination	Cluster 13: Oligodendrocytes - Mixed function	Cluster 22: Non-specific - mRNA splicing & Cell cycle	Cluster 31: Neutrophils - Chemotaxis & Apoptosis	42	Plasma membrane, Cell Junctions	
Cluster 12: Smooth muscle tissue - ECM organization	Cluster 1: Neurons - Mixed function	Cluster 39: Non-specific - Transcription	Cluster 103: Non-specific - Basic cellular processes	54	Plasma membrane, Cytosol	
Cluster 3: Oligodendrocytes - Myelination	Cluster 28: Oligodendrocytes - Mixed function	Cluster 51: Myeloma - Immunoglobulins	Cluster 9: T-cells - Adaptive immunity: Regulation	8	Golgi apparatus, Vesicles, Plasma men	Primary cilium transition zone, Cytosol
Cluster 11: Esophagus - Basic cellular processes	Cluster 8: Immune cells - Immune response	Cluster 53: Squamous epithelial cells - Keratinization	Cluster 100: Stratified epithelial cells - Barrier functions	124	Plasma membrane	
Cluster 11: Esophagus - Basic cellular processes	Cluster 12: Non-specific - Vasculature	Cluster 53: Squamous epithelial cells - Keratinization	Cluster 100: Stratified epithelial cells - Barrier functions	30	Microtubules	Cell Junctions
Cluster 14: Adipose tissue - Angiogenesis	Cluster 48: Non-specific - Mixed function	Cluster 47: Leukemia - Hemostasis	Cluster 32: Endothelial cells - Angiogenesis & vascular immunity		Plasma membrane	
Cluster 49: Non-specific - Transcription	Cluster 48: Non-specific - Mixed function	Cluster 12: Bone cancer - Neuronal signaling	Cluster 9: T-cells - Adaptive immunity: Regulation	34	Plasma membrane	Nuclear bodies, Cytosol
Cluster 14: Adipose tissue - Angiogenesis	Cluster 6: Non-specific - Immune response	Cluster 27: Breast cancer - Unknown function	Cluster 4: Breast lactating luminal cells - Lactation	60	Plasma membrane, Cytosol	
Cluster 12: Smooth muscle tissue - ECM organization	Cluster 23: Choroid plexus - Mixed function	Cluster 48: Adipocytes & Endothelial cells - Angiogenesis	Cluster 45: Colonocytes & Enterocytes - Gastrointestinal absorption & barrier	23	Cell Junctions, Focal adhesion sites	Nucleoli fibrillar center
Cluster 48: Ciliated tissues - Cilium organization	Cluster 1: Neurons - Mixed function	Cluster 56: Non-specific - Sensory perception	Cluster 51: Secretory epithelial cells - Mixed function	20	Plasma membrane	Nucleoplasm, Nucleoli
Cluster 29: Lymphoid tissue & Bone marrow - Innate immune response		Cluster 14: Myeloid leukemia - Innate immune response	Cluster 48: Neutrophils - Phagocytosis & degranulation	1	Plasma membrane	
Cluster 22: Epididymis - Mixed function	Cluster 20: Macrophages & Microglia - Immune response	Cluster 14: Myeloid leukemia - Innate immune response	Cluster 72: Macrophages - Phagocytosis & lysosomal degradation	21	Vesicles	Golgi apparatus, Plasma membrane
Cluster 14: Adipose tissue - Angiogenesis	Cluster 20: Macrophages & Microglia - Immune response	Cluster 47: Leukemia - Hemostasis	Cluster 24: Neutrophils - Innate immunity signal integration	4	Plasma membrane	Golgi apparatus
Cluster 18: Lymphoid tissue - Immune response	Cluster 7: Non-specific - Ribosome	Cluster 55: Lymphoma - Immune response	Cluster 6: Plasmacytoid dendritic cells - Viral sensing & interferon response	11	Plasma membrane, Actin filaments	Nuclear speckles, Focal adhesion sites
Cluster 18: Lymphoid tissue - Immune response	Cluster 10: Cerebellum - Nucleic acid binding	Cluster 46: Lymphoma - Humoral immune response	Cluster 87: B-cells - Adaptive immunity: Humoral response	1	Nucleoplasm, Plasma membrane	Golgi apparatus, Centrosome
Cluster 68: Non-specific - Basic cellular processes	Cluster 11: White matter - Signal transduction	Cluster 46: Lymphoma - Humoral immune response	Cluster 76: Pancreatic endocrine cells - Endocrine signaling		Plasma membrane	
Cluster 17: Intestine - Digestion	Cluster 56: Hypothalamus - Neuropeptide signaling	Cluster 57: Stomach & Colon cancer - Digestion	Cluster 70: Intestinal goblet cells - Mucin production		Plasma membrane	Vesicles, Basal body
Cluster 75: Ovary - Mixed function	Cluster 29: Forebrain - Mixed function	Cluster 36: Non-specific - mRNA processing	Cluster 35: Neurons - Synaptic function & neurogenesis	2	Plasma membrane, Cytosol	
Cluster 14: Adipose tissue - Angiogenesis	Cluster 47: Endothelial cells - Vasculature	Cluster 48: Adipocytes & Endothelial cells - Angiogenesis	Cluster 12: Endothelial cells - Angiogenesis & vascular homeostasis	11	Plasma membrane	
Cluster 30: Skeletal muscle - Striated muscle contraction	Cluster 14: Neurons - Synaptic function	Cluster 64: Connective tissue cells - ECM organization	Cluster 62: Smooth muscle cells - Muscle contraction	34	Cytosol	Plasma membrane
Cluster 77: Intestine - Digestion	Cluster 6: Non-specific - Immune response	Cluster 53: Squamous epithelial cells - Keratinization	Cluster 12: Endothelial cells - Angiogenesis & vascular homeostasis	49	Plasma membrane	Golgi apparatus, Actin filaments, Cytosol
Cluster 68: Non-specific - Basic cellular processes	Cluster 23: Choroid plexus - Mixed function	Cluster 71: Lymphoma - Inflammatory response	Cluster 31: Neutrophils - Chemotaxis & Apoptosis	62	Golgi apparatus	Vesicles, Plasma membrane
Cluster 66: Lymphoid tissue - Immune response	Cluster 24: Neurons - Mixed function	Cluster 71: Lymphoma - Inflammatory response	Cluster 24: Neutrophils - Innate immunity signal integration	49	Plasma membrane	Vesicles
Cluster 72: Kidney - Transmembrane transport	Cluster 31: Non-specific - Transcription	Cluster 15: Kidney cancer - Membrane components	Cluster 81: Alveolar cells - Lung function	3	Plasma membrane	Vesicles, Cell Junctions
Cluster 43: Placenta - Pregnancy hormone signaling	Cluster 47: Endothelial cells - Vasculature	Cluster 49: HMC-1 - Innate immune response	Cluster 12: Endothelial cells - Angiogenesis & vascular homeostasis	21	Plasma membrane	Actin filaments, Cytosol
Cluster 14: Adipose tissue - Angiogenesis	Cluster 47: Endothelial cells - Vasculature	Cluster 54: Non-specific - Transcription	Cluster 32: Endothelial cells - Angiogenesis & vascular immunity	24	Cytosol	Plasma membrane, Centrosome
Cluster 66: Lymphoid tissue - Immune response	Cluster 20: Macrophages & Microglia - Immune response	Cluster 55: Lymphoma - Immune response	Cluster 74: Microglia - Neuroimmunity & neuroinflammation	2	Golgi apparatus, Vesicles, Plasma men	Cytosol
Cluster 51: Lymphoid tissue - Adaptive immune response	Cluster 6: Non-specific - Immune response	Cluster 55: Lymphoma - Immune response	Cluster 82: Dendritic cells - Antigen presentation	26	Plasma membrane	Vesicles
Cluster 66: Lymphoid tissue - Immune response	Cluster 12: Non-specific - Vasculature	Cluster 32: Non-specific - Cell proliferation	Cluster 82: Dendritic cells - Antigen presentation		Plasma membrane, Primary cilium	
Cluster 11: Esophagus - Basic cellular processes	Cluster 11: White matter - Signal transduction	Cluster 69: Skin cancer - ECM organization	Cluster 69: Urothelial cells - Basic cellular functions	5	Plasma membrane, Centriolar satellite	Nucleoplasm, Cytosol
Cluster 73: Brain - Neuronal signaling	Cluster 39: Astrocytes - Mixed function	Cluster 59: Leukemia - T-cell receptor	Cluster 92: NK-cells & T-cells - Adaptive immunity: Cytotoxicity	141	Plasma membrane	Cytosol
Cluster 12: Smooth muscle tissue - ECM organization	Cluster 44: Non-specific - Mixed function	Cluster 64: Connective tissue cells - ECM organization	Cluster 108: Fibroblasts - ECM organization		Plasma membrane	
Cluster 14: Adipose tissue - Angiogenesis	Cluster 32: Cerebral cortex - Mixed function	Cluster 48: Adipocytes & Endothelial cells - Angiogenesis	Cluster 89: Adipocytes - Lipid metabolism	7	Plasma membrane	Nucleoplasm, Primary cilium
Cluster 69: Parathyroid gland - Mixed function	Cluster 44: Non-specific - Mixed function	Cluster 53: Squamous epithelial cells - Keratinization	Cluster 12: Endothelial cells - Angiogenesis & vascular homeostasis	14	Plasma membrane	Primary cilium, Basal body
Cluster 3: Oligodendrocytes - Myelination	Cluster 11: White matter - Signal transduction	Cluster 47: Leukemia - Hemostasis	Cluster 29: Neutrophils - Innate immune cellular maintenance	37	Plasma membrane	
Cluster 10: Brain - Synaptic signal transduction	Cluster 38: Sub-cortical - Mixed function	Cluster 47: Leukemia - Hemostasis	Cluster 98: Neurons - Neurotransmission & signaling	13	Vesicles, Plasma membrane	Primary cilium tip, Basal body
Cluster 27: Cerebellum - Synaptic function	Cluster 53: Neurons - Mixed function	Cluster 70: DU4475 - Unknown function	Cluster 109: Neurons - Neuronal signaling	14	Plasma membrane	Nucleoplasm
Cluster 78: Brain - Neuronal signaling	Cluster 51: Neurons - Mixed function	Cluster 1: Neuronal - Visual perception	Cluster 86: Retinal bipolar cells - Synaptic signal transduction		Plasma membrane	Nucleoplasm, Actin filaments, Cytosol
Cluster 78: Brain - Neuronal signaling	Cluster 56: Hypothalamus - Neuropeptide signaling	Cluster 11: Neuronal - Sensory perception	Cluster 34: Ciliated cells - Cilia assembly & function	1	Plasma membrane	Vesicles
Cluster 42: Retina - Visual perception	Cluster 56: Hypothalamus - Neuropeptide signaling	Cluster 12: Bone cancer - Neuronal signaling	Cluster 109: Neurons - Neuronal signaling		Plasma membrane, Microtubules	Nucleoli
Cluster 46: Brain & retina - Neuronal signaling	Cluster 56: Hypothalamus - Neuropeptide signaling	Cluster 66: Non-specific - Mitochondria	Cluster 96: Enteroendocrine cells - Neuropeptide signaling		Plasma membrane	
Cluster 10: Brain - Synaptic signal transduction	Cluster 38: Sub-cortical - Mixed function	Cluster 17: Neuroblastoma - Neuronal signaling	Cluster 98: Neurons - Neurotransmission & signaling	3	Plasma membrane	
Cluster 25: Non-specific - Basic cellular processes	Cluster 27: Non-specific - Mixed function	Cluster 38: Non-specific - Transcription	Cluster 97: Non-specific - Cellular respiration	148	Plasma membrane, Cytosol	Nuclear membrane, Vesicles
Cluster 18: Lymphoid tissue - Immune response	Cluster 20: Macrophages & Microglia - Immune response	Cluster 14: Myeloid leukemia - Innate immune response	Cluster 48: Neutrophils - Phagocytosis & degranulation		Plasma membrane	Golgi apparatus, Vesicles
Cluster 61: Non-specific - Transcription	Cluster 50: Non-specific - Nucleic acid binding	Cluster 43: Non-specific - Basic cellular processes	Cluster 7: Non-specific - Transcription	31	Plasma membrane	Cytosol
Cluster 21: Testis - Mixed function	Cluster 23: Choroid plexus - Mixed function	Cluster 68: Non-specific - Cell cycle regulation	Cluster 90: Elongating spermatids - Spermiogenesis: Elongation	99	Golgi apparatus, Plasma membrane, P	Vesicles
Cluster 12: Smooth muscle tissue - ECM organization	Cluster 35: Non-specific - DNA binding	Cluster 17: Neuroblastoma - Neuronal signaling	Cluster 96: Enteroendocrine cells - Neuropeptide signaling	1	Plasma membrane	Cytosol
	Cluster 15: Non-specific - Transcription		Cluster 71: Non-specific - Cardiac endocrine signaling & maintenanc	1	Nucleoplasm, Plasma membrane	Actin filaments, Focal adhesion sites
Cluster 45: Liver - Metabolism & Coagulation	Cluster 56: Hypothalamus - Neuropeptide signaling	Cluster 3: Rhabdoid - Membrane components	Cluster 89: Adipocytes - Lipid metabolism	31	Plasma membrane, Cytosol, Cytoplasmic bodies	
Cluster 54: Non-specific - Ribosome	Cluster 40: Astrocytes - Astrocyte-neuron interactions	Cluster 62: Non-specific - Plasma proteins	Cluster 84: Apical squamous epithelium - Cornification	83	Nucleoplasm	Cell Junctions
Cluster 73: Brain - Neuronal signaling	Cluster 39: Astrocytes - Mixed function	Cluster 5: Rhabdoid - Unknown function	Cluster 32: Endothelial cells - Angiogenesis & vascular immunity	35	Vesicles, Cell Junctions	Nucleoplasm
Cluster 58: Skin - Keratinization	Cluster 15: Non-specific - Transcription	Cluster 53: Squamous epithelial cells - Keratinization	Cluster 85: Squamous epithelial cells - Keratinization	13	Vesicles, Cell Junctions	
Cluster 37: Skin - Keratinization	Cluster 51: Neurons - Mixed function	Cluster 53: Squamous epithelial cells - Keratinization	Cluster 100: Stratified epithelial cells - Barrier functions	8	Cell Junctions	
Cluster 81: Squamous epithelium - Keratinization	Cluster 40: Astrocytes - Astrocyte-neuron interactions	Cluster 53: Squamous epithelial cells - Keratinization	Cluster 49: Apical squamous epithelium - Epithelial barrier & keratinization	11	Cell Junctions	
Cluster 14: Adipose tissue - Angiogenesis	Cluster 15: Non-specific - Transcription	Cluster 72: Neuronal - Signal transduction	Cluster 110: Mural cells - ECM organization	5	Plasma membrane	Nucleoplasm, Endoplasmic reticulum
Cluster 3: Oligodendrocytes - Myelination	Cluster 30: White matter - Myelination	Cluster 57: Stomach & Colon cancer - Digestion	Cluster 32: Endothelial cells - Angiogenesis & vascular immunity		Plasma membrane, Cytosol	
Cluster 3: Oligodendrocytes - Myelination	Cluster 11: White matter - Signal transduction	Cluster 10: Ciliated cells - Ion channel activity	Cluster 80: Oligodendrocytes - Myelination		Vesicles, Plasma membrane	
Cluster 76: Non-specific - Metabolism	Cluster 21: Non-specific - Metabolism	Cluster 49: HMC-1 - Innate immune response	Cluster 87: B-cells - Adaptive immunity: Humoral response	6	Nucleoplasm, Plasma membrane, Cytosol	
Cluster 8: Pancreas - Proteolysis	Cluster 56: Hypothalamus - Neuropeptide signaling	Cluster 59: Leukemia - T-cell receptor	Cluster 76: Pancreatic endocrine cells - Endocrine signaling	52	Nuclear bodies, Plasma membrane	Golgi apparatus, Centrosome
Cluster 77: Intestine - Digestion	Cluster 51: Neurons - Mixed function	Cluster 3: Rhabdoid - Membrane components	Cluster 103: Non-specific - Basic cellular processes		Plasma membrane	
Cluster 46: Brain & retina - Neuronal signaling	Cluster 43: Hindbrain - Mixed function	Cluster 1: Neuronal - Visual perception	Cluster 86: Retinal bipolar cells - Synaptic signal transduction	2	Vesicles, Plasma membrane	
Cluster 27: Cerebellum - Synaptic function	Cluster 56: Hypothalamus - Neuropeptide signaling	Cluster 40: Non-specific - Unknown function	Cluster 109: Neurons - Neuronal signaling		Vesicles, Plasma membrane	
Cluster 73: Brain - Neuronal signaling	Cluster 56: Hypothalamus - Neuropeptide signaling	Cluster 46: Lymphoma - Humoral immune response	Cluster 109: Neurons - Neuronal signaling	1	Vesicles, Plasma membrane	
Cluster 5: Spleen - Immune response	Cluster 39: Astrocytes - Mixed function	Cluster 48: Adipocytes & Endothelial cells - Angiogenesis	Cluster 40: Monocytes & Neutrophils - Inflammatory signaling	38	Cytosol	Nucleoplasm, Plasma membrane, Basal body
Cluster 73: Brain - Neuronal signaling	Cluster 51: Neurons - Mixed function	Cluster 3: Rhabdoid - Membrane components	Cluster 48: Neutrophils - Phagocytosis & degranulation	20	Nuclear speckles, Plasma membrane	Cytosol
Cluster 38: Non-specific - Basic cellular processes	Cluster 1: Neurons - Mixed function	Cluster 62: Non-specific - Plasma proteins	Cluster 76: Pancreatic endocrine cells - Endocrine signaling	30	Nucleoplasm	Plasma membrane, Cytosol
Cluster 68: Non-specific - Basic cellular processes	Cluster 33: Neurons - Mixed function	Cluster 39: Non-specific - Transcription	Cluster 48: Neutrophils - Phagocytosis & degranulation	161	Plasma membrane	
Cluster 67: Testis - Mixed function	Cluster 37: Neurons - Mixed function	Cluster 47: Leukemia - Hemostasis	Cluster 102: Non-specific - Mixed function	5	Plasma membrane	
Cluster 24: Non-specific - Transcription	Cluster 32: Cerebral cortex - Mixed function	Cluster 64: Connective tissue cells - ECM organization	Cluster 21: Extravillous trophoblasts - Placental invasion & endocrine regulatio	32	Plasma membrane, Cytosol	
Cluster 43: Placenta - Pregnancy hormone signaling	Cluster 56: Hypothalamus - Neuropeptide signaling	Cluster 18: Liver cancer - Metabolism	Cluster 108: Fibroblasts - ECM organization	1	Plasma membrane	
Cluster 1: Choroid plexus - Transmembrane transport	Cluster 41: Monoamines - Neurotransmitter signalling	Cluster 9: Skin cancer - Melanin biosynthesis	Cluster 61: Schwann cells & Melanocytes - Mixed function	4	Plasma membrane	Nucleoplasm, Nuclear bodies, Golgi apparatus
	Cluster 52: Neurons & Synapses - Synaptic function	Cluster 10: Ciliated cells - Ion channel activity	Cluster 109: Neurons - Neuronal signaling		Plasma membrane	
Cluster 75: Ovary - Mixed function	Cluster 39: Astrocytes - Mixed function	Cluster 11: Neuronal - Sensory perception	Cluster 109: Neurons - Neuronal signaling		Plasma membrane	
Cluster 42: Retina - Visual perception	Cluster 55: Non-specific - Transcription	Cluster 57: Stomach & Colon cancer - Digestion	Cluster 79: Photoreceptor cells - Phototransduction	2	Plasma membrane	
Cluster 51: Lymphoid tissue - Adaptive immune response	Cluster 6: Non-specific - Immune response	Cluster 59: Leukemia - T-cell receptor	Cluster 9: T-cells - Adaptive immunity: Regulation		Plasma membrane	Vesicles, Centriolar satellite
Cluster 29: Lymphoid tissue & Bone marrow - Innate immune response	Cluster 20: Macrophages & Microglia - Immune response	Cluster 55: Lymphoma - Immune response	Cluster 40: Monocytes & Neutrophils - Inflammatory signaling	7	Plasma membrane	Nucleoplasm, Primary cilium, Primary cilium tip

Cluster 12: Smooth muscle tissue - ECM organization	Cluster 52: Neurons & Synapses - Synaptic function	Cluster 15: Kidney cancer - Membrane components	Cluster 22: Non-specific - Basic cellular functions		Plasma membrane	
Cluster 78: Brain - Neuronal signaling	Cluster 19: Astrocytes - Mixed function	Cluster 27: Breast cancer - Unknown function	Cluster 14: Oligodendrocyte progenitor cells - Development & myelination	49	Nucleoplasm, Cell Junctions	Vesicles, Plasma membrane
Cluster 20: Pituitary gland - Hormone signaling	Cluster 56: Hypothalamus - Neuropeptide signaling	Cluster 17: Neuroblastoma - Neuronal signaling	Cluster 59: Pituitary endocrine cells - Neuroendocrine signaling	3	Plasma membrane	Nucleoplasm, Primary cilium, Primary cilium tip
Cluster 46: Brain & retina - Neuronal signaling	Cluster 19: Astrocytes - Mixed function	Cluster 51: Myeloma - Immunoglobulins			Plasma membrane	Vesicles, Primary cilium
Cluster 13: Lung - Secretion	Cluster 42: Choroid plexus - Mitochondria	Cluster 53: Squamous epithelial cells - Keratinization	Cluster 69: Urothelial cells - Basic cellular functions	6	Vesicles	Plasma membrane, Primary cilium, Primary cilium tip
Cluster 70: Non-specific - Protein processing	Cluster 25: Non-specific - Transcription	Cluster 27: Breast cancer - Unknown function	Cluster 103: Non-specific - Basic cellular processes	5	Vesicles, Plasma membrane	Nucleoplasm, Primary cilium, Primary cilium transition zone
Cluster 27: Cerebellum - Synaptic function	Cluster 51: Neurons - Mixed function	Cluster 17: Neuroblastoma - Neuronal signaling	Cluster 86: Retinal bipolar cells - Synaptic signal transduction	3	Plasma membrane	Vesicles
Cluster 24: Non-specific - Transcription	Cluster 18: Non-specific - Mixed function	Cluster 4: Non-specific - Transcription	Cluster 31: Neutrophils - Chemotaxis & Apoptosis	16	Plasma membrane	Cytosol
Cluster 4: Adrenal gland - Steroid metabolism	Cluster 5: Non-specific - Mixed function	Cluster 7: Liver cancer - Metabolism	Cluster 79: Photoreceptor cells - Phototransduction	7	Endoplasmic reticulum, Plasma memb	Vesicles
Cluster 11: Esophagus - Basic cellular processes	Cluster 5: Non-specific - Mixed function	Cluster 27: Breast cancer - Unknown function	Cluster 26: Keratinocytes - Keratinization	41	Plasma membrane, Cytosol	
Cluster 46: Brain & retina - Neuronal signaling	Cluster 49: Hindbrain - Mixed function	Cluster 10: Ciliated cells - Ion channel activity	Cluster 86: Retinal bipolar cells - Synaptic signal transduction	3	Plasma membrane	Vesicles
Cluster 65: Brain - Transcription	Cluster 29: Forebrain - Mixed function	Cluster 14: Myeloid leukemia - Innate immune response	Cluster 35: Neurons - Synaptic function & neurogenesis	2	Plasma membrane	Nucleoplasm
Cluster 73: Brain - Neuronal signaling	Cluster 51: Neurons - Mixed function	Cluster 57: Stomach & Colon cancer - Digestion	Cluster 35: Neurons - Synaptic function & neurogenesis	26	Plasma membrane	Nucleoplasm, Primary cilium, Primary cilium tip, Primary cilium transition zone, Basal bod
Cluster 12: Smooth muscle tissue - ECM organization	Cluster 40: Astrocytes - Astrocyte-neuron interactions	Cluster 64: Connective tissue cells - ECM organization	Cluster 12: Endothelial cells - Angiogenesis & vascular homeostasis	4	Nuclear membrane, Nuclear speckles	Plasma membrane
Cluster 8: Pancreas - Proteolysis	Cluster 14: Neurons - Synaptic function	Cluster 3: Rhabdoid - Membrane components	Cluster 109: Neurons - Neuronal signaling		Plasma membrane	
Cluster 37: Skin - Keratinization	Cluster 48: Non-specific - Mixed function	Cluster 70: DU4475 - Unknown function	Cluster 49: Apical squamous epithelium - Epithelial barrier & keratinization	27	Plasma membrane, Cytosol	Nucleoplasm, Nuclear bodies
Cluster 59: Lymphoid tissue & Bone marrow - Cell proliferation	Cluster 6: Non-specific - Immune response	Cluster 66: Non-specific - Mitochondria	Cluster 27: Non-specific - Cell proliferation	21	Plasma membrane, Centrosome	Nucleoplasm, Primary cilium, Primary cilium tip, Basal body, Cytosol
Cluster 15: Bone marrow - Innate immune response	Cluster 53: Neurons - Mixed function	Cluster 47: Leukemia - Hemostasis	Cluster 66: Erythroid cells - Erythroid differentiation and maturation	27	Nucleoplasm, Plasma membrane, Cytosol	
Cluster 41: Striated muscle - Muscle contraction	Cluster 20: Macrophages & Microglia - Immune response	Cluster 9: Skin cancer - Melanin biosynthesis	Cluster 66: Erythroid cells - Erythroid differentiation and maturation	11	Plasma membrane	
Cluster 39: Testis - Nuclear processes	Cluster 11: White matter - Signal transduction	Cluster 24: Non-specific - Unknown function	Cluster 71: Non-specific - Cardiac endocrine signaling & maintenanc	1	Plasma membrane, Cytosol	
Cluster 14: Adipose tissue - Angiogenesis	Cluster 1: Neurons - Mixed function	Cluster 64: Connective tissue cells - ECM organization	Cluster 103: Non-specific - Basic cellular processes	2	Plasma membrane	Nucleoplasm
Cluster 81: Squamous epithelium - Keratinization	Cluster 12: Non-specific - Vasculature	Cluster 53: Squamous epithelial cells - Keratinization	Cluster 84: Apical squamous epithelium - Cornification		Cell Junctions	
Cluster 66: Lymphoid tissue - Immune response	Cluster 20: Macrophages & Microglia - Immune response	Cluster 55: Lymphoma - Immune response	Cluster 31: Neutrophils - Chemotaxis & Apoptosis	47	Vesicles	Plasma membrane, Cytosol
Cluster 66: Lymphoid tissue - Immune response	Cluster 20: Macrophages & Microglia - Immune response	Cluster 55: Lymphoma - Immune response	Cluster 72: Macrophages - Phagocytosis & lysosomal degradation	5	Plasma membrane, Cytosol	
	Cluster 49: Hindbrain - Mixed function	Cluster 5: Rhabdoid - Unknown function	Cluster 109: Neurons - Neuronal signaling	1	Plasma membrane	Nucleoplasm
Cluster 22: Epididymis - Mixed function	Cluster 36: Choroid plexus - Mixed function	Cluster 31: Non-specific - Antiviral immune response	Cluster 72: Macrophages - Phagocytosis & lysosomal degradation	6	Plasma membrane	Nucleoplasm
Cluster 5: Spleen - Immune response	Cluster 12: Non-specific - Vasculature	Cluster 31: Non-specific - Antiviral immune response	Cluster 9: T-cells - Adaptive immunity: Regulation	55	Plasma membrane	Golgi apparatus
Cluster 5: Spleen - Immune response	Cluster 25: Non-specific - Transcription	Cluster 55: Lymphoma - Immune response	Cluster 82: Dendritic cells - Antigen presentation	2	Cell Junctions	Nucleoplasm
Cluster 5: Spleen - Immune response	Cluster 20: Macrophages & Microglia - Immune response	Cluster 46: Lymphoma - Humoral immune response	Cluster 104: Brain endothelial cells - Blood brain barrier	17	Vesicles, Plasma membrane	
Cluster 45: Liver - Metabolism & Coagulation	Cluster 49: Hindbrain - Mixed function	Cluster 66: Non-specific - Mitochondria	Cluster 45: Colonocytes & Enterocytes - Gastrointestinal absorption & barrier	15	Nucleoplasm, Cytosol	Plasma membrane
Cluster 24: Non-specific - Transcription	Cluster 25: Non-specific - Transcription	Cluster 51: Myeloma - Immunoglobulins	Cluster 67: Plasma cells - Plasma cell differentiation	85	Cytosol	Plasma membrane, Cytoplasmic bodies
Cluster 12: Smooth muscle tissue - ECM organization	Cluster 51: Neurons - Mixed function	Cluster 72: Neuronal - Signal transduction	Cluster 72: Macrophages - Phagocytosis & lysosomal degradation	1	Plasma membrane	Cytosol
Cluster 25: Non-specific - Basic cellular processes	Cluster 27: Non-specific - Mixed function	Cluster 28: Non-specific - Nuclear processes	Cluster 75: Non-specific - Chaperones and protein folding	439	Plasma membrane	Cytosol
Cluster 81: Squamous epithelium - Keratinization	Cluster 39: Astrocytes - Mixed function	Cluster 35: RS4;11 - Unknown function	Cluster 85: Squamous epithelial cells - Keratinization	281	Plasma membrane, Cytosol	
Cluster 69: Parathyroid gland - Mixed function	Cluster 38: Sub-cortical - Mixed function	Cluster 72: Neuronal - Signal transduction	Cluster 109: Neurons - Neuronal signaling	1	Nucleoplasm, Plasma membrane	Vesicles, Primary cilium
Cluster 65: Brain - Transcription	Cluster 19: Astrocytes - Mixed function	Cluster 44: Connective tissue cells - ECM organization	Cluster 108: Fibroblasts - ECM organization	4	Plasma membrane	
Cluster 63: Non-specific - Basic cellular processes	Cluster 13: Oligodendrocytes - Mixed function	Cluster 72: Neuronal - Signal transduction	Cluster 91: Pituicytes/FSC - Homeostasis & neuron support	12	Plasma membrane, Cytosol	
Cluster 44: Bone marrow - Nuclear processes	Cluster 16: Non-specific - Transcription	Cluster 20: Myeloma - Immune response	Cluster 24: Neutrophils - Innate immunity signal integration	6	Cell Junctions	Nucleoplasm
Cluster 3: Oligodendrocytes - Myelination	Cluster 11: White matter - Signal transduction	Cluster 6: Leukemia - Signal transduction	Cluster 82: Dendritic cells - Antigen presentation	3	Vesicles, Plasma membrane	
Cluster 61: Non-specific - Transcription	Cluster 50: Non-specific - Nucleic acid binding	Cluster 31: Non-specific - Antiviral immune response	Cluster 71: Non-specific - Cardiac endocrine signaling & maintenanc	11	Plasma membrane	
Cluster 22: Epididymis - Mixed function	Cluster 47: Endothelial cells - Vasculature	Cluster 31: Non-specific - Antiviral immune response	Cluster 32: Endothelial cells - Angiogenesis & vascular immunity	36	Golgi apparatus, Plasma membrane	
Cluster 8: Pancreas - Proteolysis	Cluster 12: Non-specific - Vasculature	Cluster 51: Myeloma - Immunoglobulins	Cluster 92: NK-cells & T-cells - Adaptive immunity: Cytotoxicity		Plasma membrane	Vesicles, Cytosol
Cluster 66: Lymphoid tissue - Immune response	Cluster 30: White matter - Myelination	Cluster 71: Lymphoma - Inflammatory response	Cluster 40: Monocytes & Neutrophils - Inflammatory signaling	24	Plasma membrane	
Cluster 8: Pancreas - Proteolysis	Cluster 42: Choroid plexus - Mitochondria	Cluster 58: Non-specific - Nuclear processes	Cluster 48: Neutrophils - Phagocytosis & degranulation	128	Nucleoli, Plasma membrane	Nucleoli rim, Primary cilium
Cluster 66: Lymphoid tissue - Immune response	Cluster 44: Non-specific - Mixed function	Cluster 55: Lymphoma - Immune response	Cluster 67: Plasma cells - Plasma cell differentiation	1	Nucleoplasm, Plasma membrane	
Cluster 66: Lymphoid tissue - Immune response	Cluster 22: Non-specific - Mixed function	Cluster 55: Lymphoma - Immune response	Cluster 40: Monocytes & Neutrophils - Inflammatory signaling	30	Plasma membrane, Cytosol	Primary cilium
Cluster 13: Lung - Secretion	Cluster 47: Endothelial cells - Vasculature	Cluster 31: Non-specific - Antiviral immune response	Cluster 12: Endothelial cells - Angiogenesis & vascular homeostasis	3	Vesicles, Plasma membrane	Cytosol
Cluster 76: Non-specific - Metabolism	Cluster 40: Astrocytes - Astrocyte-neuron interactions	Cluster 18: Liver cancer - Metabolism	Cluster 95: Tuft cells - Chemosensory signaling	2	Vesicles, Plasma membrane	
Cluster 14: Adipose tissue - Angiogenesis	Cluster 42: Choroid plexus - Mitochondria	Cluster 49: HMC-1 - Innate immune response	Cluster 58: Mast cells - Allergic inflammatory responses	2	Plasma membrane	Focal adhesion sites, Cytosol
Cluster 51: Lymphoid tissue - Adaptive immune response	Cluster 42: Choroid plexus - Mitochondria	Cluster 51: Myeloma - Immunoglobulins	Cluster 78: Plasma cells - Antibody production & ER-stress response	9	Plasma membrane	Nucleoplasm
Cluster 66: Lymphoid tissue - Immune response	Cluster 20: Macrophages & Microglia - Immune response	Cluster 46: Lymphoma - Humoral immune response	Cluster 24: Neutrophils - Innate immunity signal integration	12	Plasma membrane	Nucleoplasm, Centriolar satellite
Cluster 43: Placenta - Pregnancy hormone signaling	Cluster 28: Oligodendrocytes - Mixed function	Cluster 64: Connective tissue cells - ECM organization	Cluster 51: Secretory epithelial cells - Mixed function	22	Plasma membrane	Golgi apparatus
Cluster 51: Lymphoid tissue - Adaptive immune response	Cluster 48: Non-specific - Mixed function	Cluster 6: Leukemia - Signal transduction	Cluster 9: T-cells - Adaptive immunity: Regulation	13	Plasma membrane	
Cluster 13: Lung - Secretion	Cluster 11: White matter - Signal transduction	Cluster 58: Non-specific - Nuclear processes	Cluster 24: Neutrophils - Innate immunity signal integration	116	Plasma membrane, Cell Junctions	
Cluster 66: Lymphoid tissue - Immune response	Cluster 25: Non-specific - Transcription	Cluster 55: Lymphoma - Immune response	Cluster 24: Neutrophils - Innate immunity signal integration	24	Nucleoplasm	Cell Junctions, Cytosol
Cluster 14: Adipose tissue - Angiogenesis	Cluster 1: Neurons - Mixed function	Cluster 72: Neuronal - Signal transduction	Cluster 69: Urothelial cells - Basic cellular functions	15	Plasma membrane	
Cluster 66: Lymphoid tissue - Immune response	Cluster 12: Non-specific - Vasculature	Cluster 49: HMC-1 - Innate immune response	Cluster 31: Neutrophils - Chemotaxis & Apoptosis	2	Vesicles, Plasma membrane	
Cluster 12: Smooth muscle tissue - ECM organization	Cluster 47: Endothelial cells - Vasculature	Cluster 15: Kidney cancer - Membrane components	Cluster 62: Smooth muscle cells - Muscle contraction	97	Plasma membrane, Focal adhesion sit	Endoplasmic reticulum
Cluster 29: Lymphoid tissue & Bone marrow - Innate immune response	Cluster 20: Macrophages & Microglia - Immune response	Cluster 14: Myeloid leukemia - Innate immune response	Cluster 40: Monocytes & Neutrophils - Inflammatory signaling	34	Plasma membrane	Rods & Rings
Cluster 12: Smooth muscle tissue - ECM organization	Cluster 12: Non-specific - Vasculature	Cluster 69: Skin cancer - ECM organization	Cluster 47: Platelets - Hemostasis	26	Plasma membrane	Nucleoplasm
Cluster 2: Non-specific - Mixed function	Cluster 39: Astrocytes - Mixed function	Cluster 53: Squamous epithelial cells - Keratinization	Cluster 61: Schwann cells & Melanocytes - Mixed function	14	Plasma membrane, Cell Junctions	
Cluster 14: Adipose tissue - Angiogenesis	Cluster 23: Choroid plexus - Mixed function	Cluster 72: Neuronal - Signal transduction	Cluster 72: Macrophages - Phagocytosis & lysosomal degradation	68	Plasma membrane	Nucleoplasm
Cluster 33: Thyroid gland - Tissue development	Cluster 48: Non-specific - Mixed function	Cluster 57: Stomach & Colon cancer - Digestion	Cluster 1: Enterocytes - Absorption and Digestion	1	Plasma membrane	
Cluster 43: Placenta - Pregnancy hormone signaling	Cluster 19: Astrocytes - Mixed function	Cluster 5: Rhabdoid - Unknown function	Cluster 12: Endothelial cells - Angiogenesis & vascular homeostasis	2	Plasma membrane	Nucleoplasm
Cluster 66: Lymphoid tissue - Immune response	Cluster 25: Non-specific - Transcription	Cluster 59: Leukemia - T-cell receptor	Cluster 24: Neutrophils - Innate immunity signal integration		Plasma membrane	Nucleoplasm
Cluster 81: Squamous epithelium - Keratinization	Cluster 11: White matter - Signal transduction	Cluster 53: Squamous epithelial cells - Keratinization	Cluster 85: Squamous epithelial cells - Keratinization	78	Plasma membrane, Cell Junctions	Vesicles
Cluster 76: Non-specific - Metabolism	Cluster 8: Immune cells - Immune response	Cluster 46: Lymphoma - Humoral immune response	Cluster 57: Podocytes - Glomerular filtration	17	Plasma membrane	
Cluster 63: Non-specific - Basic cellular processes	Cluster 55: Non-specific - Transcription	Cluster 38: Non-specific - Transcription	Cluster 20: Non-specific - Transcription regulation	13	Nucleoplasm, Plasma membrane, Cytosol	
Cluster 63: Non-specific - Basic cellular processes	Cluster 21: Non-specific - Metabolism	Cluster 68: Non-specific - Cell cycle regulation	Cluster 71: Non-specific - Cardiac endocrine signaling & maintenanc	46	Cytosol	Plasma membrane
Cluster 73: Brain - Neuronal signaling	Cluster 56: Hypothalamus - Neuropeptide signaling	Cluster 59: Leukemia - T-cell receptor		8	Plasma membrane, Cytosol	Vesicles
Cluster 74: Non-specific - Signal transduction	Cluster 33: Neurons - Mixed function	Cluster 17: Neuroblastoma - Neuronal signaling	Cluster 105: Non-specific - Mixed function	2	Vesicles	Nucleoplasm, Plasma membrane
Cluster 77: Intestine - Digestion	Cluster 20: Macrophages & Microglia - Immune response	Cluster 32: Non-specific - Cell proliferation	Cluster 48: Neutrophils - Phagocytosis & degranulation	72	Plasma membrane	
Cluster 46: Brain & retina - Neuronal signaling	Cluster 43: Hindbrain - Mixed function	Cluster 1: Neuronal - Visual perception	Cluster 86: Retinal bipolar cells - Synaptic signal transduction	2	Plasma membrane	Nucleoplasm
Cluster 3: Oligodendrocytes - Myelination	Cluster 11: White matter - Signal transduction	Cluster 10: Ciliated cells - Ion channel activity	Cluster 59: Pituitary endocrine cells - Neuroendocrine signaling		Plasma membrane	
Cluster 12: Smooth muscle tissue - ECM organization	Cluster 40: Astrocytes - Astrocyte-neuron interactions	Cluster 14: Myeloid leukemia - Innate immune response	Cluster 110: Mural cells - ECM organization		Plasma membrane, Cytosol	Nucleoplasm
Cluster 82: Salivary gland - Secretion	Cluster 20: Macrophages & Microglia - Immune response	Cluster 69: Skin cancer - ECM organization	Cluster 39: Syncytiotrophoblasts - Placental homeostasis & endocrine signal	39	Plasma membrane, Cytosol	
Cluster 4: Adrenal gland - Steroid metabolism	Cluster 20: Macrophages & Microglia - Immune response	Cluster 57: Stomach & Colon cancer - Digestion	Cluster 83: Epicardial mesothelial cells - Basic cellular functions	9	Endoplasmic reticulum	Plasma membrane, Cytosol
Cluster 73: Brain - Neuronal signaling	Cluster 53: Neurons - Mixed function	Cluster 4: Non-specific - Transcription	Cluster 35: Neurons - Synaptic function & neurogenesis		Vesicles, Plasma membrane	Nucleoplasm
Cluster 30: Skeletal muscle - Striated muscle contraction	Cluster 49: Hindbrain - Mixed function	Cluster 53: Squamous epithelial cells - Keratinization	Cluster 36: Skeletal myocytes - Muscle excitation & contraction		Cytosol	Golgi apparatus, Plasma membrane
Cluster 27: Cerebellum - Synaptic function	Cluster 53: Neurons - Mixed function	Cluster 17: Neuroblastoma - Neuronal signaling	Cluster 35: Neurons - Synaptic function & neurogenesis		Plasma membrane	Nucleoplasm
Cluster 73: Brain - Neuronal signaling	Cluster 53: Neurons - Mixed function	Cluster 11: Neuronal - Sensory perception	Cluster 35: Neurons - Synaptic function & neurogenesis		Plasma membrane, Cytokinetic bridge	
Cluster 44: Bone marrow - Nuclear processes	Cluster 6: Non-specific - Immune response	Cluster 47: Leukemia - Hemostasis	Cluster 80: Oligodendrocytes - Myelination	8	Golgi apparatus, Plasma membrane	Nucleoplasm, Cytosol
Cluster 60: Stomach - Digestion	Cluster 23: Choroid plexus - Mixed function	Cluster 53: Squamous epithelial cells - Keratinization	Cluster 38: Secretory epithelial cells - Exocrine secretion	28	Plasma membrane, Cell Junctions	Nucleoplasm
Cluster 78: Brain - Neuronal signaling	Cluster 24: Neurons - Mixed function	Cluster 51: Myeloma - Immunoglobulins	Cluster 21: Extravillous trophoblasts - Placental invasion & endocrine regulatio	3	Plasma membrane	Vesicles
Cluster 55: Breast - Lactation	Cluster 35: Non-specific - DNA binding	Cluster 49: HMC-1 - Innate immune response	Cluster 58: Mast cells - Allergic inflammatory responses	42	Plasma membrane	Nucleoli fibrillar center
Cluster 27: Cerebellum - Synaptic function	Cluster 52: Neurons & Synapses - Synaptic function	Cluster 24: Non-specific - Unknown function	Cluster 96: Enteroendocrine cells - Neuropeptide signaling	50	Plasma membrane, Cytosol	Nucleoplasm, Mitochondria
Cluster 58: Skin - Keratinization	Cluster 24: Neurons - Mixed function	Cluster 53: Squamous epithelial cells - Keratinization	Cluster 84: Apical squamous epithelium - Cornification		Plasma membrane	Nuclear membrane
Cluster 66: Lymphoid tissue - Immune response	Cluster 13: Oligodendrocytes - Mixed function	Cluster 46: Lymphoma - Humoral immune response	Cluster 102: Non-specific - Mixed function	5	Plasma membrane	
Cluster 27: Cerebellum - Synaptic function	Cluster 1: Neurons - Mixed function	Cluster 15: Kidney cancer - Membrane components	Cluster 61: Schwann cells & Melanocytes - Mixed function	8	Nucleoplasm, Plasma membrane	
Cluster 5: Spleen - Immune response	Cluster 6: Non-specific - Immune response	Cluster 14: Myeloid leukemia - Innate immune response	Cluster 74: Microglia - Neuroimmunity & neuroinflammation	6	Plasma membrane	
Cluster 3: Oligodendrocytes - Myelination	Cluster 11: White matter - Signal transduction	Cluster 36: Non-specific - mRNA processing	Cluster 72: Macrophages - Phagocytosis & lysosomal degradation	29	Endoplasmic reticulum, Vesicles, Plasma membrane	
Cluster 68: Non-specific - Basic cellular processes	Cluster 19: Astrocytes - Mixed function	Cluster 24: Non-specific - Unknown function	Cluster 47: Platelets - Hemostasis	109	Golgi apparatus	Vesicles, Plasma membrane
Cluster 80: Thymus - Adaptive immune response	Cluster 15: Non-specific - Transcription	Cluster 59: Leukemia - T-cell receptor	Cluster 102: Non-specific - Mixed function	29	Plasma membrane	Golgi apparatus
Cluster 18: Lymphoid tissue - Immune response	Cluster 20: Macrophages & Microglia - Immune response	Cluster 6: Leukemia - Signal transduction	Cluster 40: Monocytes & Neutrophils - Inflammatory signaling	1	Plasma membrane	
Cluster 18: Lymphoid tissue - Immune response	Cluster 30: White matter - Myelination	Cluster 51: Myeloma - Immunoglobulins	Cluster 58: Mast cells - Allergic inflammatory responses	4	Plasma membrane, Cytosol	Golgi apparatus
Cluster 18: Lymphoid tissue - Immune response	Cluster 12: Non-specific - Vasculature	Cluster 55: Lymphoma - Immune response	Cluster 31: Neutrophils - Chemotaxis & Apoptosis	20	Plasma membrane	Actin filaments, Cytosol
Cluster 45: Liver - Metabolism & Coagulation	Cluster 36: Choroid plexus - Mixed function	Cluster 47: Leukemia - Hemostasis	Cluster 108: Fibroblasts - ECM organization	7	Vesicles	Plasma membrane, Primary cilium, Basal body, Cytosol
Cluster 75: Ovary - Mixed function	Cluster 44: Non-specific - Mixed function	Cluster 72: Neuronal - Signal transduction	Cluster 89: Adipocytes - Lipid metabolism	1	Plasma membrane	Centriolar satellite
Cluster 73: Brain - Neuronal signaling	Cluster 51: Neurons - Mixed function	Cluster 12: Bone cancer - Neuronal signaling	Cluster 67: Plasma cells - Plasma cell differentiation	12	Plasma membrane	
Cluster 50: Epididymis - Male reproductive secretion		Cluster 12: Bone cancer - Neuronal signaling	Cluster 10: Epididymal principal cells - Sperm maturation microenvironment		Plasma membrane	
Cluster 14: Adipose tissue - Angiogenesis	Cluster 30: White matter - Myelination	Cluster 15: Kidney cancer - Membrane components	Cluster 31: Neutrophils - Chemotaxis & Apoptosis	62	Nucleoplasm	Nuclear speckles, Golgi apparatus, Vesicles, Plasma membrane
Cluster 63: Non-specific - Basic cellular processes	Cluster 53: Neurons - Mixed function	Cluster 55: Lymphoma - Immune response	Cluster 51: Secretory epithelial cells - Mixed function	380	Cell Junctions	Cytosol
Cluster 56: Ciliated tissues - Cilium organization	Cluster 53: Neurons - Mixed function	Cluster 64: Connective tissue cells - ECM organization	Cluster 52: Glandular & Luminal cells - Estrogen-regulated epithelial secretion	46	Plasma membrane	Primary cilium
Cluster 75: Ovary - Mixed function	Cluster 55: Non-specific - Transcription	Cluster 14: Myeloid leukemia - Innate immune response	Cluster 94: Gonadal somatic cells - Mixed function		Plasma membrane	Nuclear bodies, Vesicles
Cluster 11: Esophagus - Basic cellular processes	Cluster 20: Macrophages & Microglia - Immune response	Cluster 6: Leukemia - Signal transduction	Cluster 74: Microglia - Neuroimmunity & neuroinflammation	9	Plasma membrane	Vesicles
Cluster 12: Smooth muscle tissue - ECM organization	Cluster 12: Non-specific - Vasculature	Cluster 46: Lymphoma - Humoral immune response	Cluster 62: Smooth muscle cells - Muscle contraction	10	Focal adhesion sites, Cytosol	Plasma membrane, Cell Junctions
Cluster 46: Brain & retina - Neuronal signaling	Cluster 53: Neurons - Mixed function	Cluster 10: Ciliated cells - Ion channel activity	Cluster 79: Photoreceptor cells - Phototransduction		Plasma membrane	
Cluster 12: Smooth muscle tissue - ECM organization	Cluster 44: Non-specific - Mixed function	Cluster 64: Connective tissue cells - ECM organization	Cluster 108: Fibroblasts - ECM organization	31	Plasma membrane	Nucleoli, Cytosol
Cluster 82: Salivary gland - Secretion	Cluster 15: Non-specific - Transcription	Cluster 60: Prostate cancer - Unknown function	Cluster 70: Intestinal goblet cells - Mucin production		Plasma membrane	Nucleoli fibrillar center
Cluster 60: Stomach - Digestion	Cluster 42: Choroid plexus - Mitochondria	Cluster 53: Squamous epithelial cells - Keratinization	Cluster 45: Colonocytes & Enterocytes - Gastrointestinal absorption & barrier	17	Plasma membrane, Cell Junctions	
Cluster 66: Lymphoid tissue - Immune response	Cluster 20: Macrophages & Microglia - Immune response	Cluster 14: Myeloid leukemia - Innate immune response	Cluster 40: Monocytes & Neutrophils - Inflammatory signaling		Plasma membrane, Cytosol	
Cluster 58: Skin - Keratinization	Cluster 6: Non-specific - Immune response	Cluster 56: Non-specific - Sensory perception	Cluster 71: Non-specific - Cardiac endocrine signaling & maintenanc	1	Plasma membrane	Nucleoplasm
Cluster 66: Lymphoid tissue - Immune response	Cluster 6: Non-specific - Immune response	Cluster 55: Lymphoma - Immune response	Cluster 24: Neutrophils - Innate immunity signal integration	112	Plasma membrane	Golgi apparatus, Vesicles
Cluster 3: Oligodendrocytes - Myelination	Cluster 19: Astrocytes - Mixed function	Cluster 37: Non-specific - mRNA processing	Cluster 100: Stratified epithelial cells - Barrier functions	19	Plasma membrane, Actin filaments	Cell Junctions, Basal body
Cluster 27: Cerebellum - Synaptic function	Cluster 34: Non-specific - Mixed function	Cluster 24: Non-specific - Unknown function	Cluster 59: Pituitary endocrine cells - Neuroendocrine signaling	8	Plasma membrane, Cytosol	
Cluster 64: Non-specific - Transcription	Cluster 10: Cerebellum - Nucleic acid binding	Cluster 47: Leukemia - Hemostasis	Cluster 50: Renal epithelial cells - Ion transport and pH regulation	31	Cell Junctions	Nucleoplasm
Cluster 42: Retina - Visual perception	Cluster 52: Neurons & Synapses - Synaptic function	Cluster 17: Neuroblastoma - Neuronal signaling	Cluster 50: Renal epithelial cells - Ion transport and pH regulation	15	Cell Junctions	
Cluster 25: Non-specific - Basic cellular processes	Cluster 51: Neurons - Mixed function	Cluster 46: Lymphoma - Humoral immune response	Cluster 40: Monocytes & Neutrophils - Inflammatory signaling	69	Plasma membrane, Cytosol	Basal body
Cluster 63: Non-specific - Basic cellular processes	Cluster 28: Oligodendrocytes - Mixed function	Cluster 5: Rhabdoid - Unknown function	Cluster 7: Non-specific - Transcription	108	Cytosol	Nuclear speckles, Plasma membrane
Cluster 3: Oligodendrocytes - Myelination	Cluster 20: Macrophages & Microglia - Immune response	Cluster 17: Neuroblastoma - Neuronal signaling	Cluster 80: Oligodendrocytes - Myelination	32	Plasma membrane	
Cluster 10: Brain - Synaptic signal transduction	Cluster 14: Neurons - Synaptic function	Cluster 12: Bone cancer - Neuronal signaling	Cluster 109: Neurons - Neuronal signaling	218	Plasma membrane, End piece	Nuclear speckles
Cluster 10: Brain - Synaptic signal transduction	Cluster 22: Non-specific - Mixed function	Cluster 28: Non-specific - Nuclear processes	Cluster 31: Neutrophils - Chemotaxis & Apoptosis	21	Plasma membrane	Nucleoplasm, Primary cilium, Cytosol
Cluster 11: Esophagus - Basic cellular processes	Cluster 34: Non-specific - Mixed function	Cluster 24: Non-specific - Unknown function	Cluster 24: Neutrophils - Innate immunity signal integration	71	Plasma membrane	Nucleoplasm, Cytosol
Cluster 60: Stomach - Digestion	Cluster 42: Choroid plexus - Mitochondria	Cluster 53: Squamous epithelial cells - Keratinization	Cluster 50: Renal epithelial cells - Ion transport and pH regulation	4	Cell Junctions	
Cluster 38: Non-specific - Basic cellular processes		Cluster 47: Leukemia - Hemostasis		1	Plasma membrane	Nucleoplasm, Cytosol
Cluster 22: Epididymis - Mixed function	Cluster 48: Non-specific - Mixed function	Cluster 59: Leukemia - T-cell receptor	Cluster 33: Early spermatids - Spermiogenesis: Cap phase		Plasma membrane	
Cluster 12: Smooth muscle tissue - ECM organization	Cluster 6: Non-specific - Immune response	Cluster 69: Skin cancer - ECM organization	Cluster 62: Smooth muscle cells - Muscle contraction	28	Plasma membrane	
Cluster 29: Lymphoid tissue & Bone marrow - Innate immune response	Cluster 12: Non-specific - Vasculature	Cluster 14: Myeloid leukemia - Innate immune response	Cluster 48: Neutrophils - Phagocytosis & degranulation	9	Plasma membrane	
Cluster 4: Adrenal gland - Steroid metabolism	Cluster 52: Neurons & Synapses - Synaptic function	Cluster 7: Liver cancer - Metabolism	Cluster 43: Sertoli cells - Spermatogenesis microenvironment	6	Plasma membrane, Cytosol	Nucleoplasm

Cluster 73: Brain - Neuronal signaling	Cluster 53: Neurons - Mixed function	Cluster 21: Lymphoma - Adaptive immune response	Cluster 35: Neurons - Synaptic function & neurogenesis		Plasma membrane	Nucleoplasm, Vesicles, Primary cilium tip, Basal body
Cluster 5: Spleen - Immune response	Cluster 27: Non-specific - Mixed function	Cluster 9: Skin cancer - Melanin biosynthesis	Cluster 72: Macrophages - Phagocytosis & lysosomal degradation	7	Plasma membrane	Cytosol
Cluster 71: Cerebellum - Nervous system development	Cluster 35: Non-specific - DNA binding	Cluster 3: Rhabdoid - Membrane components	Cluster 2: Cerebellar granular cells - Neuronal signaling		Plasma membrane	Golgi apparatus
Cluster 66: Lymphoid tissue - Immune response	Cluster 48: Non-specific - Mixed function	Cluster 14: Myeloid leukemia - Innate immune response	Cluster 31: Neutrophils - Chemotaxis & Apoptosis	7	Plasma membrane	
Cluster 3: Oligodendrocytes - Myelination	Cluster 13: Oligodendrocytes - Mixed function	Cluster 33: Sarcoma - Muscle contraction	Cluster 80: Oligodendrocytes - Myelination	1	Plasma membrane	
Cluster 1: Choroid plexus - Transmembrane transport	Cluster 23: Choroid plexus - Mixed function	Cluster 15: Kidney cancer - Membrane components	Cluster 72: Macrophages - Phagocytosis & lysosomal degradation	11	Plasma membrane	Midbody, Cytosol, Acrosome, Mid piece, Principal piece
Cluster 48: Ciliated tissues - Cilium organization	Cluster 3: Choroid plexus - Cilium	Cluster 27: Breast cancer - Unknown function	Cluster 99: Retinal pigment epithelial cells - Retinol metabolism		Nucleoplasm	Cell Junctions
Cluster 31: Liver - Metabolism & Coagulation	Cluster 47: Endothelial cells - Vasculature	Cluster 10: Ciliated cells - Ion channel activity	Cluster 93: Hepatocytes - Bile production & excretion		Plasma membrane, Cytosol	Cytoplasmic bodies
Cluster 11: Esophagus - Basic cellular processes	Cluster 30: White matter - Myelination	Cluster 53: Squamous epithelial cells - Keratinization	Cluster 63: Cytotrophoblasts - Basic cellular functions	28	Cell Junctions, Centriolar satellite, Cytosol	
Cluster 68: Non-specific - Basic cellular processes	Cluster 27: Non-specific - Mixed function	Cluster 27: Breast cancer - Unknown function	Cluster 45: Colonocytes & Enterocytes - Gastrointestinal absorption & barrier	11	Plasma membrane, Cytosol	
Cluster 77: Intestine - Digestion		Cluster 57: Stomach & Colon cancer - Digestion	Cluster 45: Colonocytes & Enterocytes - Gastrointestinal absorption & barrier	77	Plasma membrane, Focal adhesion sites	
Cluster 66: Lymphoid tissue - Immune response	Cluster 47: Endothelial cells - Vasculature	Cluster 71: Lymphoma - Inflammatory response	Cluster 32: Endothelial cells - Angiogenesis & vascular immunity	4	Plasma membrane	Cell Junctions, Cytosol
Cluster 44: Bone marrow - Nuclear processes	Cluster 19: Astrocytes - Mixed function	Cluster 4: Non-specific - Transcription	Cluster 37: Neutrophil progenitors - Granulopoiesis		Plasma membrane, Cytosol	
Cluster 26: Kidney & Intestine - Metabolism	Cluster 49: Hindbrain - Mixed function	Cluster 44: Connective tissue cells - ECM organization	Cluster 24: Neutrophils - Innate immunity signal integration	9	Plasma membrane, Centrosome, Basa	Cytosol
Cluster 66: Lymphoid tissue - Immune response	Cluster 47: Endothelial cells - Vasculature	Cluster 49: HMC-1 - Innate immune response	Cluster 24: Neutrophils - Innate immunity signal integration		Plasma membrane	
Cluster 40: Non-specific - Mixed function	Cluster 11: White matter - Signal transduction	Cluster 47: Leukemia - Hemostasis	Cluster 47: Platelets - Hemostasis	3	Plasma membrane	Nuclear membrane
Cluster 44: Bone marrow - Nuclear processes	Cluster 52: Neurons & Synapses - Synaptic function	Cluster 47: Leukemia - Hemostasis	Cluster 47: Platelets - Hemostasis	28	Plasma membrane	Centriolar satellite
Cluster 63: Non-specific - Basic cellular processes	Cluster 2: Neurons - Nucleosome	Cluster 53: Squamous epithelial cells - Keratinization	Cluster 29: Neutrophils - Innate immune cellular maintenance	27	Cell Junctions	Nucleoplasm
Cluster 75: Ovary - Mixed function	Cluster 41: Monoamines - Neurotransmitter signalling	Cluster 45: Non-specific - Nuclear processes	Cluster 24: Neutrophils - Innate immunity signal integration	9	Vesicles, Plasma membrane	
Cluster 69: Parathyroid gland - Mixed function	Cluster 20: Macrophages & Microglia - Immune response	Cluster 64: Connective tissue cells - ECM organization	Cluster 72: Macrophages - Phagocytosis & lysosomal degradation	1	Plasma membrane	
Cluster 12: Smooth muscle tissue - ECM organization	Cluster 35: Non-specific - DNA binding	Cluster 64: Connective tissue cells - ECM organization	Cluster 62: Smooth muscle cells - Muscle contraction		Vesicles, Plasma membrane	Nucleoplasm, Nuclear membrane, Primary cilium transition zone
Cluster 18: Lymphoid tissue - Immune response		Cluster 55: Lymphoma - Immune response	Cluster 87: B-cells - Adaptive immunity: Humoral response	34	Plasma membrane	
Cluster 48: Ciliated tissues - Cilium organization	Cluster 18: Non-specific - Mixed function	Cluster 72: Neuronal - Signal transduction	Cluster 38: Secretory epithelial cells - Exocrine secretion		Vesicles, Plasma membrane	
Cluster 14: Adipose tissue - Angiogenesis	Cluster 44: Non-specific - Mixed function	Cluster 48: Adipocytes & Endothelial cells - Angiogenesis	Cluster 32: Endothelial cells - Angiogenesis & vascular immunity	45	Plasma membrane	
Cluster 34: Lymphoid tissue - Adaptive immune response	Cluster 39: Astrocytes - Mixed function	Cluster 36: Non-specific - mRNA processing	Cluster 7: Non-specific - Transcription	11	Plasma membrane	
Cluster 60: Stomach - Digestion	Cluster 44: Non-specific - Mixed function	Cluster 53: Squamous epithelial cells - Keratinization	Cluster 68: Foveolar cells - Mucosal defense	29	Plasma membrane	
Cluster 12: Smooth muscle tissue - ECM organization	Cluster 42: Choroid plexus - Mitochondria	Cluster 55: Lymphoma - Immune response	Cluster 62: Smooth muscle cells - Muscle contraction	8	Plasma membrane, Cytosol	
Cluster 75: Ovary - Mixed function	Cluster 30: White matter - Myelination	Cluster 64: Connective tissue cells - ECM organization	Cluster 62: Smooth muscle cells - Muscle contraction	4	Actin filaments	Plasma membrane
Cluster 36: Brain & Liver - Signaling	Cluster 53: Neurons - Mixed function	Cluster 9: Skin cancer - Melanin biosynthesis	Cluster 52: Glandular & Luminal cells - Estrogen-regulated epithelial secretion	11	Plasma membrane, Cytosol	Nucleoli rim
Cluster 45: Liver - Metabolism & Coagulation	Cluster 38: Sub-cortical - Mixed function	Cluster 64: Connective tissue cells - ECM organization	Cluster 110: Mural cells - ECM organization	44	Plasma membrane	
Cluster 12: Smooth muscle tissue - ECM organization	Cluster 47: Endothelial cells - Vasculature	Cluster 42: Non-specific - Unknown function	Cluster 32: Endothelial cells - Angiogenesis & vascular immunity	50	Nuclear bodies, Plasma membrane	
Cluster 66: Lymphoid tissue - Immune response	Cluster 12: Non-specific - Vasculature	Cluster 71: Lymphoma - Inflammatory response	Cluster 40: Monocytes & Neutrophils - Inflammatory signaling		Plasma membrane	Nucleoplasm
Cluster 11: Esophagus - Basic cellular processes	Cluster 8: Immune cells - Immune response	Cluster 53: Squamous epithelial cells - Keratinization	Cluster 51: Secretory epithelial cells - Mixed function	5	Vesicles, Plasma membrane, Centriolar	Primary cilium, Primary cilium tip, Basal body
Cluster 3: Oligodendrocytes - Myelination	Cluster 34: Non-specific - Mixed function	Cluster 72: Neuronal - Signal transduction	Cluster 59: Pituitary endocrine cells - Neuroendocrine signaling	1	Microtubules, Primary cilium, Cytosol	Nucleoli, Plasma membrane, Cytokinetic bridge, Mitotic spindle
Cluster 27: Cerebellum - Synaptic function	Cluster 52: Neurons & Synapses - Synaptic function	Cluster 16: Thyroid cancer - Neuronal signaling	Cluster 98: Neurons - Neurotransmission & signaling	13	Plasma membrane, Cytosol	
Cluster 27: Cerebellum - Synaptic function	Cluster 49: Hindbrain - Mixed function	Cluster 13: Non-specific - Mixed function	Cluster 35: Neurons - Synaptic function & neurogenesis	44	Plasma membrane	
Cluster 35: Non-specific - Mixed function	Cluster 28: Oligodendrocytes - Mixed function	Cluster 17: Neuroblastoma - Neuronal signaling	Cluster 28: Astrocytes - Homeostasis & neuron support	5	Plasma membrane	Cytosol
Cluster 73: Brain - Neuronal signaling	Cluster 14: Neurons - Synaptic function	Cluster 60: Prostate cancer - Unknown function	Cluster 35: Neurons - Synaptic function & neurogenesis		Nuclear bodies, Plasma membrane	
Cluster 66: Lymphoid tissue - Immune response	Cluster 20: Macrophages & Microglia - Immune response	Cluster 12: Bone cancer - Neuronal signaling	Cluster 40: Monocytes & Neutrophils - Inflammatory signaling		Cytosol	Plasma membrane
Cluster 78: Brain - Neuronal signaling	Cluster 51: Neurons - Mixed function	Cluster 43: Non-specific - Basic cellular processes	Cluster 62: Smooth muscle cells - Muscle contraction	36	Plasma membrane	Vesicles, Cytosol
Cluster 81: Squamous epithelium - Keratinization	Cluster 48: Non-specific - Mixed function	Cluster 53: Squamous epithelial cells - Keratinization	Cluster 39: Syncytiotrophoblasts - Placental homeostasis & endocrine signal	6	Plasma membrane	
Cluster 77: Intestine - Digestion	Cluster 13: Oligodendrocytes - Mixed function	Cluster 47: Leukemia - Hemostasis	Cluster 36: Skeletal myocytes - Muscle excitation & contraction	3	Nucleoplasm, Plasma membrane	Golgi apparatus
Cluster 68: Non-specific - Basic cellular processes	Cluster 15: Non-specific - Transcription	Cluster 20: Myeloma - Immune response	Cluster 98: Neurons - Neurotransmission & signaling	28	Vesicles	Cell Junctions
Cluster 61: Non-specific - Transcription	Cluster 34: Non-specific - Mixed function	Cluster 27: Breast cancer - Unknown function	Cluster 12: Endothelial cells - Angiogenesis & vascular homeostasis	25	Plasma membrane	Nucleoplasm, Microtubules
Cluster 52: Non-specific - Cellular respiration	Cluster 32: Cerebral cortex - Mixed function	Cluster 15: Kidney cancer - Membrane components	Cluster 17: Non-specific - RNA processing	64	Plasma membrane	Nucleoplasm, Cytosol
Cluster 64: Non-specific - Transcription	Cluster 6: Non-specific - Immune response	Cluster 69: Skin cancer - ECM organization	Cluster 11: Muller glia cells - Retinal homeostasis	35	Nucleoplasm, Plasma membrane	
Cluster 74: Non-specific - Signal transduction	Cluster 1: Neurons - Mixed function	Cluster 15: Kidney cancer - Membrane components	Cluster 88: Proximal tubular cells - Transmembrane transport	4	Cell Junctions	
Cluster 11: Esophagus - Basic cellular processes	Cluster 44: Non-specific - Mixed function	Cluster 43: Non-specific - Basic cellular processes	Cluster 49: Apical squamous epithelium - Epithelial barrier & keratinization	19	Plasma membrane, Cytosol	Nucleoplasm
Cluster 43: Placenta - Pregnancy hormone signaling	Cluster 36: Choroid plexus - Mixed function	Cluster 15: Kidney cancer - Membrane components	Cluster 108: Fibroblasts - ECM organization	6	Plasma membrane	
Cluster 64: Non-specific - Transcription	Cluster 11: White matter - Signal transduction	Cluster 56: Non-specific - Sensory perception	Cluster 12: Endothelial cells - Angiogenesis & vascular homeostasis	16	Nucleoplasm, Plasma membrane, Cytosol	
Cluster 73: Brain - Neuronal signaling	Cluster 51: Neurons - Mixed function	Cluster 5: Rhabdoid - Unknown function	Cluster 14: Oligodendrocyte progenitor cells - Development & myelination	6	Golgi apparatus, Cell Junctions	
	Cluster 41: Monoamines - Neurotransmitter signalling	Cluster 11: Neuronal - Sensory perception	Cluster 109: Neurons - Neuronal signaling		Plasma membrane, Cytosol	
Cluster 61: Non-specific - Transcription	Cluster 15: Non-specific - Transcription	Cluster 72: Neuronal - Signal transduction	Cluster 58: Mast cells - Allergic inflammatory responses	29	Golgi apparatus, Plasma membrane, Cytosol	
Cluster 67: Testis - Mixed function	Cluster 26: Oligodendrocytes - Mixed function	Cluster 53: Squamous epithelial cells - Keratinization	Cluster 55: Myocytes - Oxidative muscle function	4	Plasma membrane	
Cluster 30: Skeletal muscle - Striated muscle contraction	Cluster 24: Neurons - Mixed function	Cluster 33: Sarcoma - Muscle contraction	Cluster 36: Skeletal myocytes - Muscle excitation & contraction	8	Plasma membrane	Nucleoplasm
Cluster 75: Ovary - Mixed function	Cluster 39: Astrocytes - Mixed function	Cluster 45: Non-specific - Nuclear processes	Cluster 24: Neutrophils - Innate immunity signal integration	49	Nucleoplasm, Plasma membrane	Golgi apparatus
Cluster 14: Adipose tissue - Angiogenesis	Cluster 47: Endothelial cells - Vasculature	Cluster 64: Connective tissue cells - ECM organization	Cluster 110: Mural cells - ECM organization	12	Nucleoplasm, Plasma membrane	
Cluster 80: Thymus - Adaptive immune response	Cluster 14: Neurons - Synaptic function	Cluster 3: Rhabdoid - Membrane components	Cluster 4: Breast lactating luminal cells - Lactation		Plasma membrane	Actin filaments
Cluster 44: Bone marrow - Nuclear processes	Cluster 27: Non-specific - Mixed function	Cluster 37: Non-specific - mRNA processing	Cluster 17: Non-specific - RNA processing	151	Plasma membrane	Cytosol
Cluster 75: Ovary - Mixed function	Cluster 44: Non-specific - Mixed function	Cluster 72: Neuronal - Signal transduction	Cluster 79: Photoreceptor cells - Phototransduction	7	Plasma membrane	Nucleoplasm, Cytosol
Cluster 46: Brain & retina - Neuronal signaling	Cluster 38: Sub-cortical - Mixed function	Cluster 72: Neuronal - Signal transduction	Cluster 57: Podocytes - Glomerular filtration	5	Plasma membrane	
Cluster 46: Brain & retina - Neuronal signaling	Cluster 51: Neurons - Mixed function	Cluster 12: Bone cancer - Neuronal signaling	Cluster 48: Neutrophils - Phagocytosis & degranulation	1	Vesicles	Plasma membrane
Cluster 73: Brain - Neuronal signaling	Cluster 40: Astrocytes - Astrocyte-neuron interactions	Cluster 7: Liver cancer - Metabolism	Cluster 28: Astrocytes - Homeostasis & neuron support	25	Plasma membrane	Vesicles, Primary cilium tip
Cluster 77: Intestine - Digestion	Cluster 41: Monoamines - Neurotransmitter signalling	Cluster 53: Squamous epithelial cells - Keratinization	Cluster 31: Neutrophils - Chemotaxis & Apoptosis	22	Plasma membrane	
Cluster 11: Esophagus - Basic cellular processes	Cluster 40: Astrocytes - Astrocyte-neuron interactions	Cluster 30: Non-specific - Gene expression regulation	Cluster 24: Neutrophils - Innate immunity signal integration	68	Cell Junctions	Plasma membrane, Cytosol
Cluster 60: Stomach - Digestion	Cluster 42: Choroid plexus - Mitochondria	Cluster 30: Non-specific - Gene expression regulation	Cluster 50: Renal epithelial cells - Ion transport and pH regulation	53	Plasma membrane, Cell Junctions	
Cluster 64: Non-specific - Transcription	Cluster 53: Neurons - Mixed function	Cluster 36: Non-specific - mRNA processing	Cluster 9: T-cells - Adaptive immunity: Regulation	124	Nucleoplasm	Plasma membrane
Cluster 77: Intestine - Digestion		Cluster 57: Stomach & Colon cancer - Digestion	Cluster 37: Neutrophil progenitors - Granulopoiesis	27	Nucleoplasm, Plasma membrane	Cytokinetic bridge, Cytosol
Cluster 43: Placenta - Pregnancy hormone signaling	Cluster 20: Macrophages & Microglia - Immune response	Cluster 53: Squamous epithelial cells - Keratinization	Cluster 40: Monocytes & Neutrophils - Inflammatory signaling	4	Nucleoplasm, Plasma membrane	Vesicles
Cluster 14: Adipose tissue - Angiogenesis	Cluster 53: Neurons - Mixed function	Cluster 7: Liver cancer - Metabolism	Cluster 21: Extravillous trophoblasts - Placental invasion & endocrine regulatio	1	Nucleoplasm, Cytosol	Plasma membrane, Primary cilium, Basal body
Cluster 73: Brain - Neuronal signaling	Cluster 24: Neurons - Mixed function	Cluster 14: Myeloid leukemia - Innate immune response	Cluster 35: Neurons - Synaptic function & neurogenesis	6	Plasma membrane	Nucleoplasm, Cytosol
		Cluster 50: Lymphoma - Unknown function			Plasma membrane	
	Cluster 49: Hindbrain - Mixed function	Cluster 51: Myeloma - Immunoglobulins			Plasma membrane	Nucleoplasm
	Cluster 49: Hindbrain - Mixed function	Cluster 51: Myeloma - Immunoglobulins	Cluster 56: Late spermatids - Spermiogenesis: Maturation		Plasma membrane	Nucleoplasm
	Cluster 10: Cerebellum - Nucleic acid binding	Cluster 66: Non-specific - Mitochondria	Cluster 3: Early primary spermatocytes - Early meiosis		Plasma membrane	
	Cluster 48: Non-specific - Mixed function		Cluster 103: Non-specific - Basic cellular processes		Lipid droplets, Plasma membrane	
Cluster 25: Non-specific - Basic cellular processes	Cluster 52: Neurons & Synapses - Synaptic function	Cluster 71: Lymphoma - Inflammatory response	Cluster 46: Skeletal myocytes - Muscle structure & formation	15	Plasma membrane	
Cluster 77: Intestine - Digestion	Cluster 32: Cerebral cortex - Mixed function	Cluster 27: Breast cancer - Unknown function	Cluster 68: Foveolar cells - Mucosal defense	1	Nucleoplasm, Cytosol	Plasma membrane
Cluster 72: Kidney - Transmembrane transport	Cluster 38: Sub-cortical - Mixed function	Cluster 5: Rhabdoid - Unknown function	Cluster 21: Extravillous trophoblasts - Placental invasion & endocrine regulatio		Plasma membrane	
Cluster 8: Pancreas - Proteolysis	Cluster 49: Hindbrain - Mixed function	Cluster 9: Skin cancer - Melanin biosynthesis	Cluster 24: Neutrophils - Innate immunity signal integration	48	Nuclear speckles, Plasma membrane	Vesicles, Primary cilium, Centriolar satellite
Cluster 61: Non-specific - Transcription	Cluster 25: Non-specific - Transcription	Cluster 46: Lymphoma - Humoral immune response	Cluster 48: Neutrophils - Phagocytosis & degranulation	15	Vesicles, Plasma membrane	
Cluster 63: Non-specific - Basic cellular processes	Cluster 31: Non-specific - Transcription	Cluster 29: Non-specific - Unknown function	Cluster 41: Oocytes - Oogenesis	26	Plasma membrane, Cytosol	
Cluster 10: Brain - Synaptic signal transduction	Cluster 14: Neurons - Synaptic function	Cluster 55: Lymphoma - Immune response	Cluster 31: Neutrophils - Chemotaxis & Apoptosis	80	Plasma membrane, Cytosol	
Cluster 72: Kidney - Transmembrane transport	Cluster 19: Astrocytes - Mixed function	Cluster 4: Non-specific - Transcription	Cluster 54: Intestinal epithelial cells - Intestinal epi homeostasis & respiration	55	Plasma membrane, Cell Junctions	
Cluster 78: Brain - Neuronal signaling	Cluster 51: Neurons - Mixed function	Cluster 72: Neuronal - Signal transduction	Cluster 32: Endothelial cells - Angiogenesis & vascular immunity	7	Nucleoplasm, Plasma membrane	
Cluster 14: Adipose tissue - Angiogenesis	Cluster 38: Sub-cortical - Mixed function	Cluster 72: Neuronal - Signal transduction	Cluster 32: Endothelial cells - Angiogenesis & vascular immunity	5	Plasma membrane	
Cluster 76: Non-specific - Metabolism	Cluster 28: Oligodendrocytes - Mixed function	Cluster 30: Non-specific - Gene expression regulation	Cluster 51: Secretory epithelial cells - Mixed function	47	Nucleoplasm, Plasma membrane	Cell Junctions, Cytosol
Cluster 75: Ovary - Mixed function	Cluster 14: Neurons - Synaptic function	Cluster 24: Non-specific - Unknown function	Cluster 103: Non-specific - Basic cellular processes	21	Plasma membrane	
Cluster 27: Cerebellum - Synaptic function	Cluster 24: Neurons - Mixed function	Cluster 7: Liver cancer - Metabolism	Cluster 98: Neurons - Neurotransmission & signaling		Plasma membrane	
Cluster 3: Oligodendrocytes - Myelination	Cluster 30: White matter - Myelination	Cluster 57: Stomach & Colon cancer - Digestion	Cluster 1: Enterocytes - Absorption and Digestion	3	Plasma membrane	Golgi apparatus
Cluster 54: Non-specific - Ribosome	Cluster 46: Brainstem - Mixed function	Cluster 15: Kidney cancer - Membrane components	Cluster 51: Secretory epithelial cells - Mixed function	74	Plasma membrane, Cell Junctions	
Cluster 12: Smooth muscle tissue - ECM organization	Cluster 46: Brainstem - Mixed function	Cluster 64: Connective tissue cells - ECM organization	Cluster 57: Podocytes - Glomerular filtration	10	Cell Junctions	
Cluster 70: Non-specific - Protein processing	Cluster 32: Cerebral cortex - Mixed function	Cluster 68: Non-specific - Cell cycle regulation	Cluster 23: Elongating spermatids - Sperm cell metabolism & fertilization	30	Actin filaments	Plasma membrane, Cell Junctions, Cytosol
Cluster 62: Skeletal muscle - Muscle contraction	Cluster 40: Astrocytes - Astrocyte-neuron interactions	Cluster 14: Myeloid leukemia - Innate immune response	Cluster 47: Platelets - Hemostasis	8	Plasma membrane, Actin filaments	
Cluster 41: Striated muscle - Muscle contraction	Cluster 25: Non-specific - Transcription	Cluster 53: Squamous epithelial cells - Keratinization	Cluster 50: Renal epithelial cells - Ion transport and pH regulation	16	Cell Junctions	Centriolar satellite, Cytosol
Cluster 11: Esophagus - Basic cellular processes	Cluster 51: Neurons - Mixed function	Cluster 53: Squamous epithelial cells - Keratinization	Cluster 32: Endothelial cells - Angiogenesis & vascular immunity		Nucleoplasm, Cell Junctions	Nucleoli
Cluster 12: Smooth muscle tissue - ECM organization	Cluster 56: Hypothalamus - Neuropeptide signaling	Cluster 33: Sarcoma - Muscle contraction	Cluster 109: Neurons - Neuronal signaling	10	Plasma membrane, Cell Junctions	
Cluster 27: Cerebellum - Synaptic function	Cluster 10: Cerebellum - Nucleic acid binding	Cluster 15: Kidney cancer - Membrane components	Cluster 14: Oligodendrocyte progenitor cells - Development & myelination	1	Nucleoplasm, Plasma membrane	Cytosol
Cluster 73: Brain - Neuronal signaling	Cluster 15: Non-specific - Transcription	Cluster 15: Kidney cancer - Membrane components	Cluster 28: Astrocytes - Homeostasis & neuron support		Vesicles, Plasma membrane, Cytosol	Nucleoplasm
Cluster 25: Non-specific - Basic cellular processes	Cluster 45: Non-specific - Mixed function	Cluster 39: Non-specific - Transcription	Cluster 97: Non-specific - Cellular respiration	9	Cytosol	Plasma membrane
Cluster 25: Non-specific - Basic cellular processes	Cluster 52: Neurons & Synapses - Synaptic function	Cluster 47: Leukemia - Hemostasis	Cluster 92: NK-cells & T-cells - Adaptive immunity: Cytotoxicity		Plasma membrane	
Cluster 63: Non-specific - Basic cellular processes	Cluster 40: Astrocytes - Astrocyte-neuron interactions	Cluster 7: Liver cancer - Metabolism	Cluster 36: Skeletal myocytes - Muscle excitation & contraction	19	Plasma membrane, Cytosol	Nucleoplasm, Nuclear membrane, Primary cilium
Cluster 77: Intestine - Digestion	Cluster 19: Astrocytes - Mixed function	Cluster 60: Prostate cancer - Unknown function	Cluster 1: Enterocytes - Absorption and Digestion	17	Cytosol	Nucleoplasm, Plasma membrane
Cluster 69: Parathyroid gland - Mixed function	Cluster 40: Astrocytes - Astrocyte-neuron interactions	Cluster 5: Rhabdoid - Unknown function	Cluster 72: Macrophages - Phagocytosis & lysosomal degradation	3	Plasma membrane, Cytosol	
Cluster 75: Ovary - Mixed function	Cluster 20: Macrophages & Microglia - Immune response	Cluster 44: Connective tissue cells - ECM organization	Cluster 108: Fibroblasts - ECM organization	36	Nucleoplasm, Plasma membrane, Cell	Nuclear bodies, Primary cilium, Cytosol
Cluster 14: Adipose tissue - Angiogenesis	Cluster 12: Non-specific - Vasculature	Cluster 15: Kidney cancer - Membrane components	Cluster 103: Non-specific - Basic cellular processes	1	Plasma membrane	Cell Junctions, Mitochondria
Cluster 35: Non-specific - Mixed function	Cluster 53: Neurons - Mixed function	Cluster 71: Lymphoma - Inflammatory response	Cluster 101: Choroid plexus epithelial cells - Ion transport	2	Plasma membrane, Cytosol	
Cluster 14: Adipose tissue - Angiogenesis	Cluster 47: Endothelial cells - Vasculature	Cluster 48: Adipocytes & Endothelial cells - Angiogenesis	Cluster 12: Endothelial cells - Angiogenesis & vascular homeostasis	66	Nucleoli, Plasma membrane	
Cluster 75: Ovary - Mixed function	Cluster 39: Astrocytes - Mixed function	Cluster 27: Breast cancer - Unknown function	Cluster 6: Plasmacytoid dendritic cells - Viral sensing & interferon response	1	Vesicles, Plasma membrane	
Cluster 14: Adipose tissue - Angiogenesis	Cluster 23: Choroid plexus - Mixed function	Cluster 53: Squamous epithelial cells - Keratinization	Cluster 103: Non-specific - Basic cellular processes	29	Plasma membrane, Cytosol	
Cluster 42: Retina - Visual perception	Cluster 9: Non-specific - Receptor signaling	Cluster 13: Non-specific - Mixed function	Cluster 7: Non-specific - Transcription	14	Plasma membrane, Cytosol	
Cluster 44: Bone marrow - Nuclear processes	Cluster 20: Macrophages & Microglia - Immune response	Cluster 46: Lymphoma - Humoral immune response	Cluster 48: Neutrophils - Phagocytosis & degranulation	10	Plasma membrane	Cytosol
Cluster 39: Testis - Nuclear processes	Cluster 48: Non-specific - Mixed function	Cluster 45: Non-specific - Nuclear processes	Cluster 100: Stratified epithelial cells - Barrier functions	9	Plasma membrane, Cytosol	Nucleoplasm
Cluster 12: Smooth muscle tissue - ECM organization	Cluster 11: White matter - Signal transduction	Cluster 64: Connective tissue cells - ECM organization	Cluster 64: Renal epithelial cells - Ion transport & homeostasis	25	Endoplasmic reticulum, Plasma membr	Cytosol
Cluster 35: Non-specific - Mixed function	Cluster 29: Forebrain - Mixed function	Cluster 53: Squamous epithelial cells - Keratinization	Cluster 60: Cardiomyocytes - Cardiac muscle contraction & structure	41	Cell Junctions	Nucleoplasm
Cluster 81: Squamous epithelium - Keratinization	Cluster 24: Neurons - Mixed function	Cluster 53: Squamous epithelial cells - Keratinization	Cluster 85: Squamous epithelial cells - Keratinization	19	Cell Junctions	Nucleoplasm
Cluster 3: Oligodendrocytes - Myelination	Cluster 30: White matter - Myelination	Cluster 72: Neuronal - Signal transduction	Cluster 12: Endothelial cells - Angiogenesis & vascular homeostasis	23	Cell Junctions	Plasma membrane
Cluster 21: Testis - Mixed function	Cluster 28: Oligodendrocytes - Mixed function	Cluster 47: Leukemia - Hemostasis	Cluster 83: Epicardial mesothelial cells - Basic cellular functions	9	Plasma membrane	Nuclear speckles, Microtubules
Cluster 63: Non-specific - Basic cellular processes	Cluster 31: Non-specific - Transcription	Cluster 43: Non-specific - Basic cellular processes	Cluster 29: Neutrophils - Innate immune cellular maintenance	23	Plasma membrane, Cytosol	Nucleoplasm
Cluster 15: Bone marrow - Innate immune response	Cluster 12: Non-specific - Vasculature	Cluster 72: Neuronal - Signal transduction	Cluster 31: Neutrophils - Chemotaxis & Apoptosis	5	Plasma membrane	
Cluster 60: Stomach - Digestion	Cluster 25: Non-specific - Transcription	Cluster 15: Kidney cancer - Membrane components	Cluster 12: Endothelial cells - Angiogenesis & vascular homeostasis	1	Plasma membrane, Microtubules, Prim	Nucleoplasm, Actin filaments, Cytokinetic bridge, Centrosome, Acrosome, Equatorial seg
Cluster 64: Non-specific - Transcription	Cluster 37: Neurons - Mixed function	Cluster 62: Non-specific - Plasma proteins	Cluster 71: Non-specific - Cardiac endocrine signaling & maintenanc	85	Plasma membrane	Basal body, Cytosol
Cluster 11: Esophagus - Basic cellular processes	Cluster 42: Choroid plexus - Mitochondria	Cluster 53: Squamous epithelial cells - Keratinization	Cluster 88: Proximal tubular cells - Transmembrane transport	25	Nucleoplasm, Cell Junctions	Cytosol
Cluster 17: Intestine - Digestion	Cluster 19: Astrocytes - Mixed function	Cluster 34: Testis cancer - Unknown function	Cluster 45: Colonocytes & Enterocytes - Gastrointestinal absorption & barrier	1	Cell Junctions	Centrosome
Cluster 81: Squamous epithelium - Keratinization		Cluster 53: Squamous epithelial cells - Keratinization	Cluster 85: Squamous epithelial cells - Keratinization	11	Plasma membrane	Cytosol
Cluster 14: Adipose tissue - Angiogenesis	Cluster 19: Astrocytes - Mixed function	Cluster 44: Connective tissue cells - ECM organization	Cluster 89: Adipocytes - Lipid metabolism		Plasma membrane, Cytosol	Lipid droplets
Cluster 12: Smooth muscle tissue - ECM organization	Cluster 30: White matter - Myelination	Cluster 44: Connective tissue cells - ECM organization	Cluster 16: Prostatic glandular cells - Androgen response & seminal fluid	7	Plasma membrane	
Cluster 60: Stomach - Digestion	Cluster 11: White matter - Signal transduction	Cluster 2: Mucus-secreting cells - Unknown function	Cluster 38: Secretory epithelial cells - Exocrine secretion	6	Plasma membrane	
Cluster 14: Adipose tissue - Angiogenesis	Cluster 43: Hindbrain - Mixed function	Cluster 31: Non-specific - Antiviral immune response	Cluster 37: Neutrophil progenitors - Granulopoiesis	81	Nucleoplasm, Golgi apparatus, Plasma membrane	

Cluster 72: Kidney - Transmembrane transport	Cluster 47: Endothelial cells - Vasculature	Cluster 34: Testis cancer - Unknown function	Cluster 57: Podocytes - Glomerular filtration	10	Endoplasmic reticulum, Plasma memb	Nucleoli, Vesicles, Centriolar satellite
Cluster 44: Bone marrow - Nuclear processes	Cluster 15: Non-specific - Transcription	Cluster 54: Non-specific - Transcription	Cluster 37: Neutrophil progenitors - Granulopoiesis	43	Nucleoplasm	Plasma membrane
Cluster 35: Non-specific - Mixed function	Cluster 40: Astrocytes - Astrocyte-neuron interactions	Cluster 15: Kidney cancer - Membrane components	Cluster 32: Endothelial cells - Angiogenesis & vascular immunity	47	Plasma membrane	
Cluster 46: Brain & retina - Neuronal signaling	Cluster 35: Non-specific - DNA binding	Cluster 58: Non-specific - Nuclear processes	Cluster 79: Photoreceptor cells - Phototransduction		Plasma membrane, Cytosol	
Cluster 10: Brain - Synaptic signal transduction	Cluster 53: Neurons - Mixed function	Cluster 55: Lymphoma - Immune response	Cluster 92: NK-cells & T-cells - Adaptive immunity: Cytotoxicity	43	Plasma membrane	Nuclear speckles
Cluster 46: Brain & retina - Neuronal signaling	Cluster 51: Neurons - Mixed function	Cluster 26: Non-specific - Mitochondria & RNA binding	Cluster 47: Platelets - Hemostasis	21	Plasma membrane	Primary cilium, Cytosol
Cluster 63: Non-specific - Basic cellular processes	Cluster 55: Non-specific - Transcription	Cluster 24: Non-specific - Unknown function	Cluster 7: Non-specific - Transcription	30	Nucleoplasm, Plasma membrane, Cytosol	
Cluster 79: Testis - Spermatogenesis		Cluster 5: Rhabdoid - Unknown function	Cluster 106: Spermatogonia - Spermatogonial differentiation	27	Nucleoplasm	Plasma membrane
Cluster 46: Brain & retina - Neuronal signaling	Cluster 29: Forebrain - Mixed function	Cluster 72: Neuronal - Signal transduction	Cluster 86: Retinal bipolar cells - Synaptic signal transduction	121	Cytosol	Vesicles, Plasma membrane
Cluster 27: Cerebellum - Synaptic function	Cluster 51: Neurons - Mixed function	Cluster 6: Leukemia - Signal transduction	Cluster 35: Neurons - Synaptic function & neurogenesis	30	Intermediate filaments, Cytosol	Vesicles, Plasma membrane
Cluster 21: Testis - Mixed function	Cluster 39: Astrocytes - Mixed function	Cluster 60: Prostate cancer - Unknown function	Cluster 16: Prostatic glandular cells - Androgen response & seminal fluid	30	Plasma membrane	Cytosol
Cluster 32: Retina & Lymphoid tissues - Signaling	Cluster 44: Non-specific - Mixed function	Cluster 9: Skin cancer - Melanin biosynthesis	Cluster 61: Schwann cells & Melanocytes - Mixed function	4	Plasma membrane, Cytosol	Nucleoplasm, Vesicles, Basal body
Cluster 78: Brain - Neuronal signaling	Cluster 29: Forebrain - Mixed function	Cluster 69: Skin cancer - ECM organization	Cluster 92: NK-cells & T-cells - Adaptive immunity: Cytotoxicity	3	Nucleoplasm, Plasma membrane, Cytosol	
Cluster 64: Non-specific - Transcription	Cluster 37: Neurons - Mixed function	Cluster 17: Neuroblastoma - Neuronal signaling	Cluster 2: Cerebellar granular cells - Neuronal signaling	6	Plasma membrane	
Cluster 60: Stomach - Digestion	Cluster 40: Astrocytes - Astrocyte-neuron interactions	Cluster 53: Squamous epithelial cells - Keratinization	Cluster 84: Apical squamous epithelium - Cornification	11	Plasma membrane	
Cluster 70: Non-specific - Protein processing	Cluster 42: Choroid plexus - Mitochondria	Cluster 68: Non-specific - Cell cycle regulation	Cluster 36: Skeletal myocytes - Muscle excitation & contraction	4	Nuclear speckles	Plasma membrane, Primary cilium, Cytosol
Cluster 25: Non-specific - Basic cellular processes	Cluster 45: Non-specific - Mixed function	Cluster 27: Breast cancer - Unknown function	Cluster 56: Late spermatids - Spermiogenesis: Maturation	235	Plasma membrane, Cytosol	
Cluster 29: Lymphoid tissue & Bone marrow - Innate immune response	Cluster 12: Non-specific - Vasculature	Cluster 14: Myeloid leukemia - Innate immune response	Cluster 92: NK-cells & T-cells - Adaptive immunity: Cytotoxicity	53	Plasma membrane, Cytosol	
Cluster 29: Lymphoid tissue & Bone marrow - Innate immune response	Cluster 20: Macrophages & Microglia - Immune response	Cluster 49: HMC-1 - Innate immune response	Cluster 29: Neutrophils - Innate immune cellular maintenance	26	Plasma membrane	Nucleoplasm, Vesicles
Cluster 69: Parathyroid gland - Mixed function	Cluster 40: Astrocytes - Astrocyte-neuron interactions	Cluster 41: Non-specific - Nucleosome assembly	Cluster 94: Gonadal somatic cells - Mixed function		Plasma membrane	Nucleoli
Cluster 72: Kidney - Transmembrane transport	Cluster 47: Endothelial cells - Vasculature	Cluster 15: Kidney cancer - Membrane components	Cluster 88: Proximal tubular cells - Transmembrane transport	16	Plasma membrane, Cytosol	Nucleoli
Cluster 27: Cerebellum - Synaptic function	Cluster 51: Neurons - Mixed function	Cluster 55: Lymphoma - Immune response	Cluster 40: Monocytes & Neutrophils - Inflammatory signaling	37	Plasma membrane, Cytosol	
Cluster 75: Ovary - Mixed function	Cluster 36: Choroid plexus - Mixed function	Cluster 32: Non-specific - Cell proliferation	Cluster 33: Early spermatids - Spermiogenesis: Cap phase	22	Plasma membrane, Cytosol	Vesicles
Cluster 7: Small intestine - Absorption and Digestion	Cluster 30: White matter - Myelination	Cluster 2: Mucus-secreting cells - Unknown function	Cluster 45: Colonocytes & Enterocytes - Gastrointestinal absorption & barrier	10	Nuclear speckles, Cytosol	Plasma membrane
Cluster 66: Lymphoid tissue - Immune response	Cluster 12: Non-specific - Vasculature	Cluster 15: Kidney cancer - Membrane components	Cluster 24: Neutrophils - Innate immunity signal integration	21	Nucleoplasm, Cell Junctions	Nucleoli, Nuclear bodies
Cluster 60: Stomach - Digestion	Cluster 11: White matter - Signal transduction	Cluster 53: Squamous epithelial cells - Keratinization	Cluster 22: Non-specific - Basic cellular functions	10	Cell Junctions	Vesicles, Plasma membrane, Connecting piece, Flagellar centriole
Cluster 14: Adipose tissue - Angiogenesis	Cluster 38: Sub-cortical - Mixed function	Cluster 15: Kidney cancer - Membrane components	Cluster 12: Endothelial cells - Angiogenesis & vascular homeostasis	3	Plasma membrane	Primary cilium, Primary cilium tip
Cluster 27: Cerebellum - Synaptic function	Cluster 35: Non-specific - DNA binding	Cluster 15: Kidney cancer - Membrane components	Cluster 86: Retinal bipolar cells - Synaptic signal transduction	19	Plasma membrane, Cell Junctions, Cytosol	
Cluster 64: Non-specific - Transcription	Cluster 32: Cerebral cortex - Mixed function	Cluster 23: Non-specific - Transcription	Cluster 108: Fibroblasts - ECM organization	9	Plasma membrane, Cytosol	
Cluster 70: Non-specific - Protein processing	Cluster 56: Hypothalamus - Neuropeptide signaling	Cluster 15: Kidney cancer - Membrane components	Cluster 60: Cardiomyocytes - Cardiac muscle contraction & structure	58	Nucleoplasm, Vesicles, Plasma membrane	
Cluster 3: Oligodendrocytes - Myelination	Cluster 28: Oligodendrocytes - Mixed function	Cluster 14: Myeloid leukemia - Innate immune response	Cluster 80: Oligodendrocytes - Myelination	3	Cytosol	Plasma membrane, Centriolar satellite
Cluster 1: Choroid plexus - Transmembrane transport	Cluster 44: Non-specific - Mixed function	Cluster 45: Non-specific - Nuclear processes	Cluster 64: Renal epithelial cells - Ion transport & homeostasis	30	Plasma membrane, Cytosol	
Cluster 12: Smooth muscle tissue - ECM organization	Cluster 55: Non-specific - Transcription	Cluster 5: Rhabdoid - Unknown function	Cluster 62: Smooth muscle cells - Muscle contraction	8	Plasma membrane, Cytosol	Primary cilium, Centrosome, Basal body
Cluster 11: Esophagus - Basic cellular processes		Cluster 53: Squamous epithelial cells - Keratinization	Cluster 85: Squamous epithelial cells - Keratinization	17	Plasma membrane	
Cluster 77: Intestine - Digestion	Cluster 21: Non-specific - Metabolism	Cluster 23: Non-specific - Transcription	Cluster 47: Platelets - Hemostasis	52	Nucleoplasm, Nucleoli, Plasma membr	Golgi apparatus, Primary cilium, Centriolar satellite, Basal body, Cytosol
Cluster 81: Squamous epithelium - Keratinization	Cluster 32: Cerebral cortex - Mixed function	Cluster 53: Squamous epithelial cells - Keratinization	Cluster 85: Squamous epithelial cells - Keratinization	1	Vesicles, Plasma membrane	
Cluster 81: Squamous epithelium - Keratinization		Cluster 53: Squamous epithelial cells - Keratinization	Cluster 84: Apical squamous epithelium - Cornification	2	Vesicles, Plasma membrane	
Cluster 63: Non-specific - Basic cellular processes	Cluster 25: Non-specific - Transcription	Cluster 8: Non-specific - Basic cellular processes	Cluster 24: Neutrophils - Innate immunity signal integration	284	Plasma membrane, Cytosol	
Cluster 11: Esophagus - Basic cellular processes	Cluster 7: Non-specific - Ribosome	Cluster 67: Non-specific - Unknown function	Cluster 51: Secretory epithelial cells - Mixed function	27	Plasma membrane	Focal adhesion sites
Cluster 58: Skin - Keratinization	Cluster 55: Non-specific - Transcription	Cluster 13: Non-specific - Mixed function	Cluster 64: Renal epithelial cells - Ion transport & homeostasis	104	Nucleoplasm, Plasma membrane	Nuclear bodies
Cluster 14: Adipose tissue - Angiogenesis	Cluster 30: White matter - Myelination	Cluster 12: Bone cancer - Neuronal signaling	Cluster 32: Endothelial cells - Angiogenesis & vascular immunity	107	Plasma membrane	
Cluster 73: Brain - Neuronal signaling	Cluster 53: Neurons - Mixed function	Cluster 5: Rhabdoid - Unknown function	Cluster 104: Brain endothelial cells - Blood brain barrier	20	Plasma membrane, Cytosol	
Cluster 45: Liver - Metabolism & Coagulation	Cluster 14: Neurons - Synaptic function	Cluster 27: Breast cancer - Unknown function	Cluster 72: Macrophages - Phagocytosis & lysosomal degradation	9	Nuclear bodies, Plasma membrane	Cytosol
Cluster 66: Lymphoid tissue - Immune response	Cluster 20: Macrophages & Microglia - Immune response	Cluster 14: Myeloid leukemia - Innate immune response	Cluster 92: NK-cells & T-cells - Adaptive immunity: Cytotoxicity	15	Nucleoplasm, Plasma membrane	
Cluster 61: Non-specific - Transcription	Cluster 37: Neurons - Mixed function	Cluster 50: Lymphoma - Unknown function	Cluster 9: T-cells - Adaptive immunity: Regulation	3	Cytosol	Plasma membrane
Cluster 66: Lymphoid tissue - Immune response	Cluster 33: Neurons - Mixed function	Cluster 6: Leukemia - Signal transduction	Cluster 47: Platelets - Hemostasis		Plasma membrane	
Cluster 29: Lymphoid tissue & Bone marrow - Innate immune response	Cluster 6: Non-specific - Immune response	Cluster 14: Myeloid leukemia - Innate immune response	Cluster 48: Neutrophils - Phagocytosis & degranulation		Nucleoplasm, Cytosol	Plasma membrane
Cluster 63: Non-specific - Basic cellular processes	Cluster 5: Non-specific - Mixed function	Cluster 45: Non-specific - Nuclear processes	Cluster 24: Neutrophils - Innate immunity signal integration	135	Cytosol	Golgi apparatus, Plasma membrane
Cluster 64: Non-specific - Transcription	Cluster 5: Non-specific - Mixed function	Cluster 58: Non-specific - Nuclear processes	Cluster 27: Non-specific - Cell proliferation	28	Plasma membrane, Cytosol	
Cluster 4: Adrenal gland - Steroid metabolism	Cluster 11: White matter - Signal transduction	Cluster 24: Non-specific - Unknown function	Cluster 18: Adrenal cortex cells - Steroidogensis	26	Plasma membrane	Nucleoplasm
Cluster 75: Ovary - Mixed function	Cluster 11: White matter - Signal transduction	Cluster 64: Connective tissue cells - ECM organization	Cluster 108: Fibroblasts - ECM organization	2	Plasma membrane	
Cluster 3: Oligodendrocytes - Myelination	Cluster 30: White matter - Myelination	Cluster 32: Non-specific - Cell proliferation	Cluster 29: Neutrophils - Innate immune cellular maintenance	5	Plasma membrane, Microtubules	
Cluster 22: Epididymis - Mixed function	Cluster 40: Astrocytes - Astrocyte-neuron interactions	Cluster 28: Non-specific - Nuclear processes	Cluster 59: Pituitary endocrine cells - Neuroendocrine signaling	11	Plasma membrane	Vesicles, Cytosol
Cluster 69: Parathyroid gland - Mixed function	Cluster 41: Monoamines - Neurotransmitter signalling	Cluster 16: Thyroid cancer - Neuronal signaling	Cluster 109: Neurons - Neuronal signaling	53	Golgi apparatus, Cytosol	Plasma membrane
Cluster 66: Lymphoid tissue - Immune response	Cluster 20: Macrophages & Microglia - Immune response	Cluster 14: Myeloid leukemia - Innate immune response	Cluster 40: Monocytes & Neutrophils - Inflammatory signaling	15	Cell Junctions	Nucleoli fibrillar center
Cluster 1: Choroid plexus - Transmembrane transport	Cluster 38: Sub-cortical - Mixed function	Cluster 72: Neuronal - Signal transduction	Cluster 32: Endothelial cells - Angiogenesis & vascular immunity	4	Plasma membrane	
Cluster 22: Epididymis - Mixed function		Cluster 49: HMC-1 - Innate immune response	Cluster 51: Secretory epithelial cells - Mixed function	6	Plasma membrane	
Cluster 12: Smooth muscle tissue - ECM organization	Cluster 28: Oligodendrocytes - Mixed function	Cluster 8: Non-specific - Basic cellular processes	Cluster 102: Non-specific - Mixed function	188	Plasma membrane, Cytosol	
Cluster 51: Lymphoid tissue - Adaptive immune response	Cluster 8: Immune cells - Immune response	Cluster 31: Non-specific - Antiviral immune response	Cluster 102: Non-specific - Mixed function	47	Plasma membrane, Cytosol	
Cluster 36: Brain & Liver - Signaling	Cluster 53: Neurons - Mixed function	Cluster 53: Squamous epithelial cells - Keratinization	Cluster 32: Endothelial cells - Angiogenesis & vascular immunity	15	Vesicles, Plasma membrane, Actin filaments	
Cluster 19: Skeletal muscle - Striated muscle contraction	Cluster 25: Non-specific - Transcription	Cluster 28: Non-specific - Nuclear processes	Cluster 24: Neutrophils - Innate immunity signal integration	41	Plasma membrane, Cytosol	Nucleoplasm
Cluster 61: Non-specific - Transcription	Cluster 38: Sub-cortical - Mixed function	Cluster 5: Rhabdoid - Unknown function	Cluster 59: Pituitary endocrine cells - Neuroendocrine signaling	6	Plasma membrane	Primary cilium
Cluster 44: Bone marrow - Nuclear processes	Cluster 22: Non-specific - Mixed function	Cluster 36: Non-specific - mRNA processing	Cluster 48: Neutrophils - Phagocytosis & degranulation	39	Plasma membrane, Mid piece, Principa	Nucleoplasm, Nuclear bodies, Vesicles, Cytosol, Acrosome
Cluster 54: Non-specific - Ribosome	Cluster 7: Non-specific - Ribosome	Cluster 25: Non-specific - Ribosome	Cluster 15: Non-specific - Protein processing	257	Plasma membrane, Cytosol	
Cluster 63: Non-specific - Basic cellular processes	Cluster 52: Neurons & Synapses - Synaptic function	Cluster 52: Non-specific - Mitochondria	Cluster 29: Neutrophils - Innate immune cellular maintenance	43	Nucleoli	Vesicles, Plasma membrane, Cytosol
Cluster 16: Liver & Kidney - Metabolism	Cluster 34: Non-specific - Mixed function	Cluster 37: Non-specific - mRNA processing	Cluster 22: Non-specific - Basic cellular functions	3	Golgi apparatus, Vesicles, Plasma men	Nucleoplasm, Nuclear bodies, Primary cilium, Primary cilium tip, Basal body
Cluster 27: Cerebellum - Synaptic function	Cluster 34: Non-specific - Mixed function	Cluster 35: RS4;11 - Unknown function	Cluster 2: Cerebellar granular cells - Neuronal signaling	18	Plasma membrane, Focal adhesion site	Actin filaments
Cluster 2: Non-specific - Mixed function	Cluster 51: Neurons - Mixed function	Cluster 27: Breast cancer - Unknown function	Cluster 56: Late spermatids - Spermiogenesis: Maturation	5	Actin filaments	Nucleoplasm, Plasma membrane, Midbody
Cluster 9: Skin - Hair follicle development	Cluster 39: Astrocytes - Mixed function	Cluster 53: Squamous epithelial cells - Keratinization	Cluster 108: Fibroblasts - ECM organization	21	Cytosol	Golgi apparatus, Plasma membrane
Cluster 12: Smooth muscle tissue - ECM organization	Cluster 12: Non-specific - Vasculature	Cluster 2: Mucus-secreting cells - Unknown function	Cluster 45: Colonocytes & Enterocytes - Gastrointestinal absorption & barrier	27	Plasma membrane, Cytosol	
Cluster 11: Esophagus - Basic cellular processes	Cluster 12: Non-specific - Vasculature	Cluster 53: Squamous epithelial cells - Keratinization	Cluster 85: Squamous epithelial cells - Keratinization	44	Plasma membrane, Intermediate filaments, Cytosol	
Cluster 81: Squamous epithelium - Keratinization		Cluster 53: Squamous epithelial cells - Keratinization	Cluster 49: Apical squamous epithelium - Epithelial barrier & keratinization	6	Plasma membrane	Nucleoplasm, Cytosol
Cluster 10: Brain - Synaptic signal transduction	Cluster 52: Neurons & Synapses - Synaptic function	Cluster 10: Ciliated cells - Ion channel activity	Cluster 109: Neurons - Neuronal signaling	1	Nucleoplasm, Plasma membrane	Nuclear bodies
Cluster 73: Brain - Neuronal signaling	Cluster 51: Neurons - Mixed function	Cluster 17: Neuroblastoma - Neuronal signaling	Cluster 32: Endothelial cells - Angiogenesis & vascular immunity	128	Plasma membrane, Cytosol	Vesicles
Cluster 35: Non-specific - Mixed function	Cluster 56: Hypothalamus - Neuropeptide signaling	Cluster 33: Sarcoma - Muscle contraction	Cluster 60: Cardiomyocytes - Cardiac muscle contraction & structure	10	Plasma membrane	Nucleoplasm, Nucleoli
Cluster 27: Cerebellum - Synaptic function	Cluster 52: Neurons & Synapses - Synaptic function	Cluster 64: Connective tissue cells - ECM organization	Cluster 35: Neurons - Synaptic function & neurogenesis	1	Plasma membrane	Vesicles, Cell Junctions
Cluster 67: Testis - Mixed function	Cluster 37: Neurons - Mixed function	Cluster 27: Breast cancer - Unknown function	Cluster 88: Proximal tubular cells - Transmembrane transport	3	Plasma membrane, Actin filaments	
Cluster 72: Kidney - Transmembrane transport	Cluster 35: Non-specific - DNA binding	Cluster 12: Bone cancer - Neuronal signaling	Cluster 38: Secretory epithelial cells - Exocrine secretion	6	Plasma membrane	Nucleoplasm, Primary cilium, Primary cilium transition zone
Cluster 68: Non-specific - Basic cellular processes	Cluster 13: Oligodendrocytes - Mixed function	Cluster 40: Non-specific - Unknown function	Cluster 28: Astrocytes - Homeostasis & neuron support	72	Plasma membrane, Cell Junctions	Nucleoplasm, Rods & Rings
Cluster 12: Smooth muscle tissue - ECM organization	Cluster 48: Non-specific - Mixed function	Cluster 64: Connective tissue cells - ECM organization	Cluster 83: Epicardial mesothelial cells - Basic cellular functions	1	Plasma membrane	
Cluster 45: Liver - Metabolism & Coagulation	Cluster 21: Non-specific - Metabolism	Cluster 64: Connective tissue cells - ECM organization	Cluster 108: Fibroblasts - ECM organization	55	Plasma membrane	
Cluster 44: Bone marrow - Nuclear processes	Cluster 28: Oligodendrocytes - Mixed function	Cluster 47: Leukemia - Hemostasis	Cluster 17: Non-specific - RNA processing	1	Nuclear speckles	Golgi apparatus, Plasma membrane, Cytosol
Cluster 3: Oligodendrocytes - Myelination	Cluster 30: White matter - Myelination	Cluster 59: Leukemia - T-cell receptor	Cluster 24: Neutrophils - Innate immunity signal integration	7	Plasma membrane	Nucleoplasm, Vesicles, Primary cilium transition zone, Centrosome, Basal body
Cluster 76: Non-specific - Metabolism	Cluster 11: White matter - Signal transduction	Cluster 57: Stomach & Colon cancer - Digestion	Cluster 51: Secretory epithelial cells - Mixed function	3	Plasma membrane, Cytosol	Vesicles, Centrosome
Cluster 10: Brain - Synaptic signal transduction	Cluster 51: Neurons - Mixed function	Cluster 6: Leukemia - Signal transduction	Cluster 19: Cerebellar purkinje cells - Neurotransmission & signaling	11	Plasma membrane	
Cluster 66: Lymphoid tissue - Immune response	Cluster 30: White matter - Myelination	Cluster 46: Lymphoma - Humoral immune response	Cluster 40: Monocytes & Neutrophils - Inflammatory signaling		Cytosol	Plasma membrane, Actin filaments
Cluster 66: Lymphoid tissue - Immune response	Cluster 6: Non-specific - Immune response	Cluster 49: HMC-1 - Innate immune response	Cluster 31: Neutrophils - Chemotaxis & Apoptosis		Vesicles	Plasma membrane
Cluster 10: Brain - Synaptic signal transduction	Cluster 23: Choroid plexus - Mixed function	Cluster 71: Lymphoma - Inflammatory response	Cluster 40: Monocytes & Neutrophils - Inflammatory signaling	26	Plasma membrane	Actin filaments
Cluster 77: Intestine - Digestion	Cluster 29: Forebrain - Mixed function	Cluster 4: Non-specific - Transcription	Cluster 87: B-cells - Adaptive immunity: Humoral response	26	Plasma membrane	Nucleoplasm, Golgi apparatus
Cluster 72: Kidney - Transmembrane transport	Cluster 13: Oligodendrocytes - Mixed function	Cluster 10: Ciliated cells - Ion channel activity	Cluster 88: Proximal tubular cells - Transmembrane transport	9	Plasma membrane	
Cluster 31: Liver - Metabolism & Coagulation	Cluster 40: Astrocytes - Astrocyte-neuron interactions	Cluster 18: Liver cancer - Metabolism	Cluster 38: Secretory epithelial cells - Exocrine secretion	17	Nucleoplasm, Plasma membrane	
Cluster 3: Oligodendrocytes - Myelination	Cluster 44: Non-specific - Mixed function	Cluster 72: Neuronal - Signal transduction	Cluster 28: Astrocytes - Homeostasis & neuron support	22	Plasma membrane	
Cluster 52: Non-specific - Cellular respiration	Cluster 47: Endothelial cells - Vasculature	Cluster 34: Testis cancer - Unknown function	Cluster 104: Brain endothelial cells - Blood brain barrier	27	Plasma membrane, Cell Junctions	
Cluster 63: Non-specific - Basic cellular processes	Cluster 23: Choroid plexus - Mixed function	Cluster 32: Non-specific - Cell proliferation	Cluster 40: Monocytes & Neutrophils - Inflammatory signaling	4	Vesicles, Cell Junctions	
Cluster 30: Skeletal muscle - Striated muscle contraction	Cluster 20: Macrophages & Microglia - Immune response	Cluster 15: Kidney cancer - Membrane components	Cluster 21: Extravillous trophoblasts - Placental invasion & endocrine regulatio	10	Plasma membrane	Nuclear membrane
Cluster 35: Non-specific - Mixed function	Cluster 37: Neurons - Mixed function	Cluster 71: Lymphoma - Inflammatory response	Cluster 103: Non-specific - Basic cellular processes	13	Plasma membrane	Nucleoplasm
Cluster 69: Parathyroid gland - Mixed function	Cluster 45: Non-specific - Mixed function	Cluster 64: Connective tissue cells - ECM organization	Cluster 72: Macrophages - Phagocytosis & lysosomal degradation	2	Plasma membrane, Cytosol	
Cluster 11: Esophagus - Basic cellular processes	Cluster 49: Hindbrain - Mixed function	Cluster 32: Non-specific - Cell proliferation	Cluster 103: Non-specific - Basic cellular processes	33	Plasma membrane	Centriolar satellite, Basal body
Cluster 71: Cerebellum - Nervous system development	Cluster 35: Non-specific - DNA binding	Cluster 53: Squamous epithelial cells - Keratinization	Cluster 19: Cerebellar purkinje cells - Neurotransmission & signaling		Plasma membrane	Intermediate filaments
Cluster 29: Lymphoid tissue & Bone marrow - Innate immune response	Cluster 37: Neurons - Mixed function	Cluster 72: Neuronal - Signal transduction	Cluster 100: Stratified epithelial cells - Barrier functions	31	Plasma membrane	
Cluster 3: Oligodendrocytes - Myelination	Cluster 34: Non-specific - Mixed function	Cluster 12: Bone cancer - Neuronal signaling	Cluster 80: Oligodendrocytes - Myelination		Plasma membrane	
Cluster 77: Intestine - Digestion	Cluster 6: Non-specific - Immune response	Cluster 57: Stomach & Colon cancer - Digestion	Cluster 45: Colonocytes & Enterocytes - Gastrointestinal absorption & barrier	2	Plasma membrane	Cytosol
Cluster 16: Liver & Kidney - Metabolism	Cluster 30: White matter - Myelination	Cluster 15: Kidney cancer - Membrane components	Cluster 88: Proximal tubular cells - Transmembrane transport		Plasma membrane	Nuclear speckles, Cytosol
Cluster 11: Esophagus - Basic cellular processes	Cluster 47: Endothelial cells - Vasculature	Cluster 15: Kidney cancer - Membrane components	Cluster 39: Syncytiotrophoblasts - Placental homeostasis & endocrine signal	90	Plasma membrane	Centriolar satellite
Cluster 1: Choroid plexus - Transmembrane transport	Cluster 36: Choroid plexus - Mixed function	Cluster 72: Neuronal - Signal transduction	Cluster 101: Choroid plexus epithelial cells - Ion transport	1	Plasma membrane	Cell Junctions
Cluster 57: Testis - Spermatogenesis	Cluster 10: Cerebellum - Nucleic acid binding	Cluster 34: Testis cancer - Unknown function	Cluster 53: Late primary spermatocytes - Meiotic division & transition		Plasma membrane	
Cluster 45: Liver - Metabolism & Coagulation	Cluster 11: White matter - Signal transduction	Cluster 15: Kidney cancer - Membrane components	Cluster 93: Hepatocytes - Bile production & excretion	3	Plasma membrane, Mid piece, Principal piece	
Cluster 15: Bone marrow - Innate immune response	Cluster 1: Neurons - Mixed function	Cluster 34: Testis cancer - Unknown function	Cluster 48: Neutrophils - Phagocytosis & degranulation	3	Plasma membrane	
Cluster 63: Non-specific - Basic cellular processes	Cluster 20: Macrophages & Microglia - Immune response	Cluster 14: Myeloid leukemia - Innate immune response	Cluster 53: Late primary spermatocytes - Meiotic division & transition	12	Plasma membrane	Mid piece, Principal piece
Cluster 45: Liver - Metabolism & Coagulation	Cluster 42: Choroid plexus - Mitochondria	Cluster 47: Leukemia - Hemostasis	Cluster 104: Brain endothelial cells - Blood brain barrier	21	Plasma membrane	Vesicles
Cluster 8: Pancreas - Proteolysis	Cluster 37: Neurons - Mixed function	Cluster 65: Non-specific - Unknown function	Cluster 76: Pancreatic endocrine cells - Endocrine signaling	66	Vesicles, Plasma membrane	
Cluster 72: Kidney - Transmembrane transport		Cluster 47: Leukemia - Hemostasis	Cluster 88: Proximal tubular cells - Transmembrane transport	1	Nuclear speckles, Plasma membrane	Nucleoplasm, Mitotic spindle, Cytosol
Cluster 13: Lung - Secretion	Cluster 5: Non-specific - Mixed function	Cluster 15: Kidney cancer - Membrane components	Cluster 81: Alveolar cells - Lung function	13	Plasma membrane	Nucleoplasm, Cytosol
Cluster 36: Brain & Liver - Signaling	Cluster 41: Monoamines - Neurotransmitter signalling	Cluster 64: Connective tissue cells - ECM organization	Cluster 32: Endothelial cells - Angiogenesis & vascular immunity	8	Plasma membrane	
Cluster 76: Non-specific - Metabolism	Cluster 28: Oligodendrocytes - Mixed function	Cluster 64: Connective tissue cells - ECM organization	Cluster 26: Keratinocytes - Keratinization	10	Plasma membrane	
Cluster 55: Breast - Lactation	Cluster 50: Non-specific - Nucleic acid binding	Cluster 27: Breast cancer - Unknown function	Cluster 16: Prostatic glandular cells - Androgen response & seminal fluid	26	Cytosol	Nucleoplasm, Plasma membrane
Cluster 68: Non-specific - Basic cellular processes	Cluster 19: Astrocytes - Mixed function	Cluster 53: Squamous epithelial cells - Keratinization	Cluster 13: Syncytiotrophoblasts - Placental remodeling & endocrine func.	58	Nucleoplasm, Plasma membrane	
Cluster 4: Adrenal gland - Steroid metabolism	Cluster 44: Non-specific - Mixed function	Cluster 47: Leukemia - Hemostasis	Cluster 39: Syncytiotrophoblasts - Placental homeostasis & endocrine signal	3	Plasma membrane, Cytosol	Nucleoplasm
Cluster 25: Non-specific - Basic cellular processes	Cluster 52: Neurons & Synapses - Synaptic function	Cluster 63: Non-specific - Protein processing	Cluster 32: Endothelial cells - Angiogenesis & vascular immunity	9	Plasma membrane	
Cluster 7: Small intestine - Absorption and Digestion	Cluster 25: Non-specific - Transcription	Cluster 34: Testis cancer - Unknown function	Cluster 72: Macrophages - Phagocytosis & lysosomal degradation	1	Plasma membrane, Cytosol	
Cluster 1: Choroid plexus - Transmembrane transport	Cluster 36: Choroid plexus - Mixed function	Cluster 39: Non-specific - Transcription	Cluster 99: Retinal pigment epithelial cells - Retinol metabolism	8	Nuclear speckles, Plasma membrane	Cytosol
Cluster 8: Pancreas - Proteolysis	Cluster 40: Astrocytes - Astrocyte-neuron interactions	Cluster 15: Kidney cancer - Membrane components	Cluster 28: Astrocytes - Homeostasis & neuron support	4	Plasma membrane	Cytosol
Cluster 42: Retina - Visual perception	Cluster 29: Forebrain - Mixed function	Cluster 72: Neuronal - Signal transduction	Cluster 79: Photoreceptor cells - Phototransduction	34	Plasma membrane	Focal adhesion sites, Cytosol
Cluster 7: Small intestine - Absorption and Digestion	Cluster 9: Non-specific - Receptor signaling	Cluster 57: Stomach & Colon cancer - Digestion	Cluster 1: Enterocytes - Absorption and Digestion	1	Golgi apparatus, Plasma membrane	Nuclear membrane
Cluster 72: Kidney - Transmembrane transport	Cluster 9: Non-specific - Receptor signaling	Cluster 56: Non-specific - Sensory perception	Cluster 88: Proximal tubular cells - Transmembrane transport		Plasma membrane	Nucleoplasm
Cluster 72: Kidney - Transmembrane transport	Cluster 36: Choroid plexus - Mixed function	Cluster 69: Skin cancer - ECM organization	Cluster 72: Macrophages - Phagocytosis & lysosomal degradation	13	Plasma membrane	
Cluster 76: Non-specific - Metabolism	Cluster 8: Immune cells - Immune response	Cluster 10: Ciliated cells - Ion channel activity	Cluster 51: Secretory epithelial cells - Mixed function	5	Plasma membrane	Cell Junctions
Cluster 3: Oligodendrocytes - Myelination	Cluster 19: Astrocytes - Mixed function	Cluster 53: Squamous epithelial cells - Keratinization	Cluster 104: Brain endothelial cells - Blood brain barrier	21	Plasma membrane, Cytosol	Vesicles
Cluster 63: Non-specific - Basic cellular processes	Cluster 23: Choroid plexus - Mixed function	Cluster 68: Non-specific - Cell cycle regulation	Cluster 46: Skeletal myocytes - Muscle structure & formation	31	Vesicles	Plasma membrane

Cluster 35: Non-specific - Mixed function	Cluster 38: Sub-cortical - Mixed function	Cluster 64: Connective tissue cells - ECM organization	Cluster 24: Neutrophils - Innate immunity signal integration	3	Nucleoplasm, Plasma membrane	
Cluster 60: Stomach - Digestion	Cluster 52: Neurons & Synapses - Synaptic function	Cluster 53: Squamous epithelial cells - Keratinization	Cluster 29: Neutrophils - Innate immune cellular maintenance	20	Plasma membrane	Nucleoplasm
Cluster 17: Intestine - Digestion	Cluster 52: Neurons & Synapses - Synaptic function	Cluster 32: Non-specific - Cell proliferation	Cluster 54: Intestinal epithelial cells - Intestinal epi homeostasis & respiration	2	Cell Junctions	
Cluster 26: Kidney & Intestine - Metabolism	Cluster 37: Neurons - Mixed function	Cluster 57: Stomach & Colon cancer - Digestion	Cluster 50: Renal epithelial cells - Ion transport and pH regulation	7	Plasma membrane	
Cluster 70: Non-specific - Protein processing	Cluster 47: Endothelial cells - Vasculature	Cluster 72: Neuronal - Signal transduction	Cluster 32: Endothelial cells - Angiogenesis & vascular immunity	67	Plasma membrane	
Cluster 10: Brain - Synaptic signal transduction	Cluster 14: Neurons - Synaptic function	Cluster 36: Non-specific - mRNA processing	Cluster 91: Pituicytes/FSC - Homeostasis & neuron support	17	Vesicles	Plasma membrane
Cluster 56: Ciliated tissues - Cilium organization	Cluster 17: Choroid plexus - Cilium	Cluster 47: Leukemia - Hemostasis	Cluster 34: Ciliated cells - Cilia assembly & function		Plasma membrane	Nuclear speckles
Cluster 36: Brain & Liver - Signaling	Cluster 11: White matter - Signal transduction	Cluster 56: Non-specific - Sensory perception	Cluster 104: Brain endothelial cells - Blood brain barrier		Plasma membrane	
Cluster 45: Liver - Metabolism & Coagulation	Cluster 28: Oligodendrocytes - Mixed function	Cluster 7: Liver cancer - Metabolism	Cluster 25: Hepatocytes - Plasma protein synthesis & secretion	2	Plasma membrane	
Cluster 45: Liver - Metabolism & Coagulation	Cluster 36: Choroid plexus - Mixed function	Cluster 57: Stomach & Colon cancer - Digestion	Cluster 93: Hepatocytes - Bile production & excretion	3	Plasma membrane	
Cluster 14: Adipose tissue - Angiogenesis	Cluster 47: Endothelial cells - Vasculature	Cluster 15: Kidney cancer - Membrane components	Cluster 21: Extravillous trophoblasts - Placental invasion & endocrine regulatio	2	Cell Junctions	
Cluster 63: Non-specific - Basic cellular processes	Cluster 56: Hypothalamus - Neuropeptide signaling	Cluster 12: Bone cancer - Neuronal signaling	Cluster 55: Myocytes - Oxidative muscle function	1	Plasma membrane	Vesicles
Cluster 14: Adipose tissue - Angiogenesis	Cluster 24: Neurons - Mixed function	Cluster 64: Connective tissue cells - ECM organization	Cluster 62: Smooth muscle cells - Muscle contraction		Cell Junctions	
Cluster 60: Stomach - Digestion	Cluster 33: Neurons - Mixed function	Cluster 27: Breast cancer - Unknown function	Cluster 38: Secretory epithelial cells - Exocrine secretion		Plasma membrane	
Cluster 11: Esophagus - Basic cellular processes	Cluster 25: Non-specific - Transcription	Cluster 53: Squamous epithelial cells - Keratinization	Cluster 63: Cytotrophoblasts - Basic cellular functions	35	Plasma membrane	Nucleoplasm, Cell Junctions
Cluster 66: Lymphoid tissue - Immune response	Cluster 11: White matter - Signal transduction	Cluster 58: Non-specific - Nuclear processes	Cluster 47: Platelets - Hemostasis	57	Plasma membrane	Primary cilium
Cluster 47: Testis - Mixed function	Cluster 23: Choroid plexus - Mixed function	Cluster 68: Non-specific - Cell cycle regulation	Cluster 12: Endothelial cells - Angiogenesis & vascular homeostasis	28	Golgi apparatus, Plasma membrane, C	Nucleoplasm, Primary cilium transition zone
Cluster 64: Non-specific - Transcription	Cluster 49: Hindbrain - Mixed function	Cluster 16: Thyroid cancer - Neuronal signaling	Cluster 32: Endothelial cells - Angiogenesis & vascular immunity	10	Plasma membrane	Nucleoplasm
Cluster 12: Smooth muscle tissue - ECM organization	Cluster 28: Oligodendrocytes - Mixed function	Cluster 9: Skin cancer - Melanin biosynthesis	Cluster 51: Secretory epithelial cells - Mixed function	53	Plasma membrane	Cytosol
Cluster 12: Smooth muscle tissue - ECM organization	Cluster 40: Astrocytes - Astrocyte-neuron interactions	Cluster 32: Non-specific - Cell proliferation	Cluster 62: Smooth muscle cells - Muscle contraction	22	Focal adhesion sites	Plasma membrane, Centrosome
Cluster 3: Oligodendrocytes - Myelination	Cluster 11: White matter - Signal transduction	Cluster 9: Skin cancer - Melanin biosynthesis	Cluster 37: Neutrophil progenitors - Granulopoiesis	58	Golgi apparatus, Cytosol	Plasma membrane
Cluster 70: Non-specific - Protein processing	Cluster 11: White matter - Signal transduction	Cluster 58: Non-specific - Nuclear processes	Cluster 24: Neutrophils - Innate immunity signal integration	16	Plasma membrane, Cytosol	Nucleoli
Cluster 11: Esophagus - Basic cellular processes	Cluster 38: Sub-cortical - Mixed function	Cluster 72: Neuronal - Signal transduction	Cluster 32: Endothelial cells - Angiogenesis & vascular immunity	6	Plasma membrane	Vesicles, Primary cilium, Primary cilium transition zone
Cluster 11: Esophagus - Basic cellular processes	Cluster 50: Non-specific - Nucleic acid binding	Cluster 53: Squamous epithelial cells - Keratinization	Cluster 84: Apical squamous epithelium - Cornification	17	Plasma membrane, Cytosol	Nucleoplasm
Cluster 25: Non-specific - Basic cellular processes	Cluster 34: Non-specific - Mixed function	Cluster 9: Skin cancer - Melanin biosynthesis	Cluster 50: Renal epithelial cells - Ion transport and pH regulation	17	Nucleoplasm, Cytosol	Plasma membrane
Cluster 67: Testis - Mixed function	Cluster 20: Macrophages & Microglia - Immune response	Cluster 23: Non-specific - Transcription	Cluster 54: Intestinal epithelial cells - Intestinal epi homeostasis & respiration	3	Nucleoplasm, Plasma membrane	Centrosome
Cluster 61: Non-specific - Transcription	Cluster 13: Oligodendrocytes - Mixed function	Cluster 38: Non-specific - Transcription	Cluster 85: Squamous epithelial cells - Keratinization	2	Vesicles, Plasma membrane	
Cluster 17: Intestine - Digestion	Cluster 29: Forebrain - Mixed function	Cluster 53: Squamous epithelial cells - Keratinization	Cluster 69: Urothelial cells - Basic cellular functions	220	Vesicles, Plasma membrane, Cell Junctions	
Cluster 4: Adrenal gland - Steroid metabolism	Cluster 56: Hypothalamus - Neuropeptide signaling	Cluster 2: Mucus-secreting cells - Unknown function	Cluster 96: Enteroendocrine cells - Neuropeptide signaling	4	Plasma membrane	
Cluster 63: Non-specific - Basic cellular processes	Cluster 28: Oligodendrocytes - Mixed function	Cluster 26: Non-specific - Mitochondria & RNA binding	Cluster 73: Late spermatids - Spermiogenesis: Maturation	59	Nucleoplasm	Plasma membrane, Cytosol
Cluster 14: Adipose tissue - Angiogenesis	Cluster 23: Choroid plexus - Mixed function	Cluster 7: Liver cancer - Metabolism	Cluster 110: Mural cells - ECM organization		Nucleoplasm, Plasma membrane	
Cluster 66: Lymphoid tissue - Immune response	Cluster 25: Non-specific - Transcription	Cluster 14: Myeloid leukemia - Innate immune response	Cluster 31: Neutrophils - Chemotaxis & Apoptosis	8	Plasma membrane	Nucleoplasm, Nuclear bodies
Cluster 14: Adipose tissue - Angiogenesis	Cluster 39: Astrocytes - Mixed function	Cluster 64: Connective tissue cells - ECM organization	Cluster 37: Neutrophil progenitors - Granulopoiesis	162	Vesicles, Plasma membrane	Cytosol
Cluster 63: Non-specific - Basic cellular processes	Cluster 21: Non-specific - Metabolism	Cluster 43: Non-specific - Basic cellular processes	Cluster 97: Non-specific - Cellular respiration	18	Plasma membrane, Cytosol	
Cluster 48: Ciliated tissues - Cilium organization	Cluster 46: Brainstem - Mixed function	Cluster 38: Non-specific - Transcription	Cluster 107: Ciliated cells - Cilia assembly & function	4	Plasma membrane	
Cluster 48: Ciliated tissues - Cilium organization	Cluster 36: Choroid plexus - Mixed function	Cluster 3: Rhabdoid - Membrane components	Cluster 52: Glandular & Luminal cells - Estrogen-regulated epithelial secretion	72	Endoplasmic reticulum, Plasma membrane	
Cluster 25: Non-specific - Basic cellular processes	Cluster 44: Non-specific - Mixed function	Cluster 15: Kidney cancer - Membrane components	Cluster 62: Smooth muscle cells - Muscle contraction	26	Plasma membrane	Actin filaments, Cytosol
Cluster 66: Lymphoid tissue - Immune response	Cluster 20: Macrophages & Microglia - Immune response	Cluster 46: Lymphoma - Humoral immune response	Cluster 87: B-cells - Adaptive immunity: Humoral response	6	Plasma membrane	Actin filaments
Cluster 67: Testis - Mixed function	Cluster 48: Non-specific - Mixed function	Cluster 4: Non-specific - Transcription	Cluster 82: Dendritic cells - Antigen presentation	54	Nucleoplasm	Plasma membrane, Cytosol
Cluster 17: Intestine - Digestion	Cluster 53: Neurons - Mixed function	Cluster 15: Kidney cancer - Membrane components	Cluster 68: Foveolar cells - Mucosal defense	3	Nucleoplasm, Golgi apparatus	Plasma membrane
Cluster 70: Non-specific - Protein processing	Cluster 6: Non-specific - Immune response	Cluster 51: Myeloma - Immunoglobulins	Cluster 44: Plasma cells - Antibody production & secretion	74	Nucleoplasm	Endoplasmic reticulum, Plasma membrane
Cluster 74: Non-specific - Signal transduction	Cluster 41: Monoamines - Neurotransmitter signalling	Cluster 72: Neuronal - Signal transduction	Cluster 109: Neurons - Neuronal signaling	14	Plasma membrane, Centrosome, Basal body	
Cluster 12: Smooth muscle tissue - ECM organization	Cluster 51: Neurons - Mixed function	Cluster 2: Mucus-secreting cells - Unknown function	Cluster 62: Smooth muscle cells - Muscle contraction	1	Plasma membrane	
Cluster 81: Squamous epithelium - Keratinization	Cluster 36: Choroid plexus - Mixed function	Cluster 53: Squamous epithelial cells - Keratinization	Cluster 100: Stratified epithelial cells - Barrier functions	6	Plasma membrane	Nucleoli, Vesicles
Cluster 22: Epididymis - Mixed function	Cluster 6: Non-specific - Immune response	Cluster 51: Myeloma - Immunoglobulins	Cluster 30: Epididymal principal cells - Sperm storage microenvironment		Plasma membrane	Golgi apparatus
Cluster 81: Squamous epithelium - Keratinization	Cluster 40: Astrocytes - Astrocyte-neuron interactions	Cluster 66: Non-specific - Mitochondria	Cluster 91: Pituicytes/FSC - Homeostasis & neuron support	10	Plasma membrane	
Cluster 70: Non-specific - Protein processing	Cluster 14: Neurons - Synaptic function	Cluster 10: Ciliated cells - Ion channel activity	Cluster 2: Cerebellar granular cells - Neuronal signaling	1	Plasma membrane, Cell Junctions	
Cluster 70: Non-specific - Protein processing	Cluster 52: Neurons & Synapses - Synaptic function	Cluster 17: Neuroblastoma - Neuronal signaling	Cluster 101: Choroid plexus epithelial cells - Ion transport	1	Plasma membrane	Mid piece, Principal piece
Cluster 12: Smooth muscle tissue - ECM organization	Cluster 6: Non-specific - Immune response	Cluster 14: Myeloid leukemia - Innate immune response	Cluster 47: Platelets - Hemostasis	20	Plasma membrane	Nuclear speckles
Cluster 81: Squamous epithelium - Keratinization	Cluster 37: Neurons - Mixed function	Cluster 55: Lymphoma - Immune response	Cluster 105: Non-specific - Mixed function	1	Plasma membrane, Cell Junctions	
		Cluster 55: Lymphoma - Immune response	Cluster 39: Syncytiotrophoblasts - Placental homeostasis & endocrine signal		Plasma membrane, Cell Junctions	
Cluster 58: Skin - Keratinization	Cluster 49: Hindbrain - Mixed function	Cluster 6: Leukemia - Signal transduction	Cluster 92: NK-cells & T-cells - Adaptive immunity: Cytotoxicity	7	Plasma membrane	
Cluster 14: Adipose tissue - Angiogenesis	Cluster 47: Endothelial cells - Vasculature	Cluster 48: Adipocytes & Endothelial cells - Angiogenesis	Cluster 12: Endothelial cells - Angiogenesis & vascular homeostasis	14	Plasma membrane	Centrosome, Basal body
Cluster 14: Adipose tissue - Angiogenesis	Cluster 20: Macrophages & Microglia - Immune response	Cluster 33: Sarcoma - Muscle contraction	Cluster 74: Microglia - Neuroimmunity & neuroinflammation	49	Plasma membrane, Primary cilium	Primary cilium tip
Cluster 14: Adipose tissue - Angiogenesis	Cluster 42: Choroid plexus - Mitochondria	Cluster 44: Connective tissue cells - ECM organization	Cluster 12: Endothelial cells - Angiogenesis & vascular homeostasis	94	Plasma membrane	
Cluster 71: Cerebellum - Nervous system development	Cluster 35: Non-specific - DNA binding	Cluster 69: Skin cancer - ECM organization	Cluster 2: Cerebellar granular cells - Neuronal signaling	27	Nucleoplasm, Cell Junctions	Nuclear membrane, Cytokinetic bridge, Cytosol
Cluster 21: Testis - Mixed function	Cluster 50: Non-specific - Nucleic acid binding	Cluster 4: Non-specific - Transcription	Cluster 60: Cardiomyocytes - Cardiac muscle contraction & structure	1	Plasma membrane, Cytosol	
Cluster 74: Non-specific - Signal transduction	Cluster 11: White matter - Signal transduction	Cluster 53: Squamous epithelial cells - Keratinization	Cluster 104: Brain endothelial cells - Blood brain barrier	44	Cell Junctions	
Cluster 42: Retina - Visual perception	Cluster 13: Oligodendrocytes - Mixed function	Cluster 53: Squamous epithelial cells - Keratinization	Cluster 28: Astrocytes - Homeostasis & neuron support	53	Plasma membrane, Cell Junctions	Nucleoplasm
Cluster 17: Intestine - Digestion	Cluster 36: Choroid plexus - Mixed function	Cluster 27: Breast cancer - Unknown function	Cluster 45: Colonocytes & Enterocytes - Gastrointestinal absorption & barrier	1	Nucleoplasm, Cell Junctions	Plasma membrane
Cluster 12: Smooth muscle tissue - ECM organization	Cluster 20: Macrophages & Microglia - Immune response	Cluster 53: Squamous epithelial cells - Keratinization	Cluster 47: Platelets - Hemostasis	42	Focal adhesion sites, Cytosol	Plasma membrane, Centriolar satellite
Cluster 29: Lymphoid tissue & Bone marrow - Innate immune response	Cluster 20: Macrophages & Microglia - Immune response	Cluster 14: Myeloid leukemia - Innate immune response	Cluster 31: Neutrophils - Chemotaxis & Apoptosis	14	Plasma membrane	Nucleoplasm, Vesicles, Cytosol
Cluster 29: Lymphoid tissue & Bone marrow - Innate immune response	Cluster 39: Astrocytes - Mixed function	Cluster 72: Neuronal - Signal transduction	Cluster 29: Neutrophils - Innate immune cellular maintenance	25	Golgi apparatus, Plasma membrane	
Cluster 7: Small intestine - Absorption and Digestion	Cluster 10: Cerebellum - Nucleic acid binding	Cluster 57: Stomach & Colon cancer - Digestion	Cluster 1: Enterocytes - Absorption and Digestion	6	Plasma membrane, Focal adhesion sites	
Cluster 6: Testis - Spermatogenesis	Cluster 18: Non-specific - Mixed function	Cluster 65: Non-specific - Unknown function	Cluster 56: Late spermatids - Spermiogenesis: Maturation	5	Plasma membrane, Cytosol	
Cluster 27: Cerebellum - Synaptic function	Cluster 35: Non-specific - DNA binding	Cluster 11: Neuronal - Sensory perception	Cluster 2: Cerebellar granular cells - Neuronal signaling	3	Plasma membrane, Cytosol	
Cluster 24: Non-specific - Transcription	Cluster 52: Neurons & Synapses - Synaptic function	Cluster 28: Non-specific - Nuclear processes	Cluster 81: Alveolar cells - Lung function	9	Plasma membrane, Actin filaments	
Cluster 41: Striated muscle - Muscle contraction	Cluster 38: Sub-cortical - Mixed function	Cluster 40: Non-specific - Unknown function	Cluster 55: Myocytes - Oxidative muscle function	29	Vesicles, Plasma membrane	
Cluster 60: Stomach - Digestion	Cluster 23: Choroid plexus - Mixed function	Cluster 60: Prostate cancer - Unknown function	Cluster 16: Prostatic glandular cells - Androgen response & seminal fluid	71	Plasma membrane	
Cluster 77: Intestine - Digestion	Cluster 40: Astrocytes - Astrocyte-neuron interactions	Cluster 50: Lymphoma - Unknown function	Cluster 54: Intestinal epithelial cells - Intestinal epi homeostasis & respiration	9	Plasma membrane	Cytosol
Cluster 68: Non-specific - Basic cellular processes	Cluster 12: Non-specific - Vasculature	Cluster 9: Skin cancer - Melanin biosynthesis	Cluster 88: Proximal tubular cells - Transmembrane transport	12	Plasma membrane, Cytosol	
Cluster 11: Esophagus - Basic cellular processes	Cluster 30: White matter - Myelination	Cluster 53: Squamous epithelial cells - Keratinization	Cluster 69: Urothelial cells - Basic cellular functions	4	Plasma membrane, Cytosol	
Cluster 14: Adipose tissue - Angiogenesis	Cluster 33: Neurons - Mixed function	Cluster 21: Lymphoma - Adaptive immune response	Cluster 83: Epicardial mesothelial cells - Basic cellular functions	6	Plasma membrane	
Cluster 66: Lymphoid tissue - Immune response	Cluster 8: Immune cells - Immune response	Cluster 14: Myeloid leukemia - Innate immune response	Cluster 40: Monocytes & Neutrophils - Inflammatory signaling	27	Plasma membrane	Vesicles, Focal adhesion sites
Cluster 78: Brain - Neuronal signaling	Cluster 32: Cerebral cortex - Mixed function	Cluster 53: Squamous epithelial cells - Keratinization	Cluster 14: Oligodendrocyte progenitor cells - Development & myelination	56	Plasma membrane	Vesicles
Cluster 78: Brain - Neuronal signaling	Cluster 39: Astrocytes - Mixed function	Cluster 45: Non-specific - Nuclear processes	Cluster 51: Secretory epithelial cells - Mixed function	66	Cell Junctions, Cytosol	
Cluster 12: Smooth muscle tissue - ECM organization	Cluster 35: Non-specific - DNA binding	Cluster 15: Kidney cancer - Membrane components	Cluster 88: Proximal tubular cells - Transmembrane transport	3	Plasma membrane	Nucleoplasm
Cluster 59: Lymphoid tissue & Bone marrow - Cell proliferation	Cluster 33: Neurons - Mixed function	Cluster 8: Non-specific - Basic cellular processes	Cluster 27: Non-specific - Cell proliferation	20	Plasma membrane, Cytosol	
Cluster 80: Thymus - Adaptive immune response		Cluster 59: Leukemia - T-cell receptor	Cluster 9: T-cells - Adaptive immunity: Regulation	2	Plasma membrane	Mitotic spindle, Centriolar satellite
Cluster 30: Skeletal muscle - Striated muscle contraction	Cluster 39: Astrocytes - Mixed function	Cluster 5: Rhabdoid - Unknown function	Cluster 60: Cardiomyocytes - Cardiac muscle contraction & structure	17	Plasma membrane, Cytosol	Nucleoplasm
Cluster 66: Lymphoid tissue - Immune response	Cluster 20: Macrophages & Microglia - Immune response	Cluster 47: Leukemia - Hemostasis	Cluster 47: Platelets - Hemostasis	4	Nuclear speckles, Cytosol	Golgi apparatus, Plasma membrane
Cluster 77: Intestine - Digestion	Cluster 12: Non-specific - Vasculature	Cluster 57: Stomach & Colon cancer - Digestion	Cluster 65: Non-specific - Cell growth & division	9	Nucleoplasm, Plasma membrane	
Cluster 66: Lymphoid tissue - Immune response	Cluster 41: Monoamines - Neurotransmitter signalling	Cluster 64: Connective tissue cells - ECM organization	Cluster 72: Macrophages - Phagocytosis & lysosomal degradation		Plasma membrane	
Cluster 74: Non-specific - Signal transduction	Cluster 30: White matter - Myelination	Cluster 53: Squamous epithelial cells - Keratinization	Cluster 69: Urothelial cells - Basic cellular functions	131	Nuclear bodies, Plasma membrane	Vesicles, Cytosol
Cluster 15: Bone marrow - Innate immune response	Cluster 6: Non-specific - Immune response	Cluster 47: Leukemia - Hemostasis	Cluster 66: Erythroid cells - Erythroid differentiation and maturation	49	Plasma membrane	
Cluster 60: Stomach - Digestion	Cluster 6: Non-specific - Immune response	Cluster 51: Myeloma - Immunoglobulins	Cluster 7: Non-specific - Transcription	31	Plasma membrane	Cytosol
Cluster 75: Ovary - Mixed function	Cluster 55: Non-specific - Transcription	Cluster 38: Non-specific - Transcription	Cluster 94: Gonadal somatic cells - Mixed function	70	Nucleoplasm, Primary cilium	Nucleoli, Plasma membrane, Mitotic spindle, Primary cilium transition zone, Basal body
Cluster 18: Lymphoid tissue - Immune response	Cluster 49: Hindbrain - Mixed function	Cluster 14: Myeloid leukemia - Innate immune response	Cluster 92: NK-cells & T-cells - Adaptive immunity: Cytotoxicity	18	Nucleoplasm, Cytosol	Nucleoli, Plasma membrane
Cluster 17: Intestine - Digestion	Cluster 42: Choroid plexus - Mitochondria	Cluster 51: Myeloma - Immunoglobulins	Cluster 44: Plasma cells - Antibody production & secretion	8	Plasma membrane, Cytosol	
Cluster 29: Lymphoid tissue & Bone marrow - Innate immune response	Cluster 20: Macrophages & Microglia - Immune response	Cluster 49: HMC-1 - Innate immune response	Cluster 40: Monocytes & Neutrophils - Inflammatory signaling	7	Plasma membrane	
Cluster 59: Lymphoid tissue & Bone marrow - Cell proliferation	Cluster 48: Non-specific - Mixed function	Cluster 62: Non-specific - Plasma proteins	Cluster 27: Non-specific - Cell proliferation	15	Plasma membrane, Cytosol	
Cluster 63: Non-specific - Basic cellular processes	Cluster 55: Non-specific - Transcription	Cluster 59: Leukemia - T-cell receptor	Cluster 29: Neutrophils - Innate immune cellular maintenance	170	Plasma membrane, Cytosol	
Cluster 71: Cerebellum - Nervous system development	Cluster 35: Non-specific - DNA binding	Cluster 13: Non-specific - Mixed function	Cluster 100: Stratified epithelial cells - Barrier functions	3	Plasma membrane, Cytosol	
Cluster 81: Squamous epithelium - Keratinization	Cluster 51: Neurons - Mixed function	Cluster 53: Squamous epithelial cells - Keratinization	Cluster 100: Stratified epithelial cells - Barrier functions	5	Vesicles, Plasma membrane	
Cluster 63: Non-specific - Basic cellular processes	Cluster 49: Hindbrain - Mixed function	Cluster 13: Non-specific - Mixed function	Cluster 66: Erythroid cells - Erythroid differentiation and maturation	94	Plasma membrane, Cytosol	Endoplasmic reticulum
Cluster 39: Testis - Nuclear processes	Cluster 25: Non-specific - Transcription	Cluster 56: Non-specific - Sensory perception	Cluster 53: Late primary spermatocytes - Meiotic division & transition	6	Plasma membrane	Cytosol
Cluster 61: Non-specific - Transcription	Cluster 49: Hindbrain - Mixed function	Cluster 28: Non-specific - Nuclear processes	Cluster 12: Endothelial cells - Angiogenesis & vascular homeostasis	57	Plasma membrane	Nucleoplasm, Primary cilium, Basal body
Cluster 12: Smooth muscle tissue - ECM organization	Cluster 12: Non-specific - Vasculature	Cluster 4: Non-specific - Transcription	Cluster 31: Neutrophils - Chemotaxis & Apoptosis	68	Plasma membrane, Cell Junctions, Focal adhesion sites	
Cluster 77: Intestine - Digestion	Cluster 15: Non-specific - Transcription	Cluster 57: Stomach & Colon cancer - Digestion	Cluster 1: Enterocytes - Absorption and Digestion	3	Plasma membrane	
Cluster 61: Non-specific - Transcription	Cluster 37: Neurons - Mixed function	Cluster 19: Non-specific - Unknown function	Cluster 79: Photoreceptor cells - Phototransduction	17	Vesicles, Lysosomes	Plasma membrane, Cell Junctions
Cluster 55: Breast - Lactation	Cluster 21: Non-specific - Metabolism	Cluster 22: Non-specific - mRNA splicing & Cell cycle	Cluster 62: Smooth muscle cells - Muscle contraction	34	Plasma membrane, Cell Junctions, Cytosol	
Cluster 49: Non-specific - Transcription	Cluster 26: Oligodendrocytes - Mixed function	Cluster 8: Non-specific - Basic cellular processes	Cluster 29: Neutrophils - Innate immune cellular maintenance	60	Nucleoplasm	Plasma membrane
Cluster 44: Bone marrow - Nuclear processes	Cluster 31: Non-specific - Transcription	Cluster 33: Sarcoma - Muscle contraction	Cluster 37: Neutrophil progenitors - Granulopoiesis	26	Plasma membrane, Cytosol	
Cluster 14: Adipose tissue - Angiogenesis	Cluster 44: Non-specific - Mixed function	Cluster 13: Non-specific - Mixed function	Cluster 92: NK-cells & T-cells - Adaptive immunity: Cytotoxicity	112	Plasma membrane, Cytosol	
Cluster 11: Esophagus - Basic cellular processes	Cluster 28: Oligodendrocytes - Mixed function	Cluster 47: Leukemia - Hemostasis	Cluster 84: Apical squamous epithelium - Cornification	31	Nucleoplasm, Plasma membrane, Cytosol	
Cluster 61: Non-specific - Transcription	Cluster 21: Non-specific - Metabolism	Cluster 36: Non-specific - mRNA processing	Cluster 17: Non-specific - RNA processing	28	Nucleoplasm, Plasma membrane, Cytosol	
Cluster 42: Retina - Visual perception	Cluster 25: Non-specific - Transcription	Cluster 5: Rhabdoid - Unknown function	Cluster 86: Retinal bipolar cells - Synaptic signal transduction		Plasma membrane	
Cluster 59: Lymphoid tissue & Bone marrow - Cell proliferation	Cluster 30: White matter - Myelination	Cluster 19: Non-specific - Unknown function	Cluster 24: Neutrophils - Innate immunity signal integration	2	Vesicles, Plasma membrane	
Cluster 73: Brain - Neuronal signaling	Cluster 38: Sub-cortical - Mixed function	Cluster 1: Neuronal - Visual perception	Cluster 86: Retinal bipolar cells - Synaptic signal transduction	16	Plasma membrane	
Cluster 63: Non-specific - Basic cellular processes	Cluster 38: Sub-cortical - Mixed function	Cluster 24: Non-specific - Unknown function	Cluster 100: Stratified epithelial cells - Barrier functions	8	Nucleoplasm, Plasma membrane	
Cluster 81: Squamous epithelium - Keratinization	Cluster 36: Choroid plexus - Mixed function	Cluster 53: Squamous epithelial cells - Keratinization	Cluster 85: Squamous epithelial cells - Keratinization	8	Plasma membrane, Cytosol	Actin filaments, Focal adhesion sites
Cluster 11: Esophagus - Basic cellular processes	Cluster 34: Non-specific - Mixed function	Cluster 39: Non-specific - Transcription	Cluster 17: Non-specific - RNA processing	127	Nucleoplasm	Plasma membrane

Antibody RRID	Cancer prognostics - Bladder Urothelial Carcinoma (TCGA)	Cancer prognostics - Breast Invasive Carcinoma (TCGA)	Cancer prognostics - Breast Invasive Carcinoma (validation)	Cancer prognostics - Cervical Squamous Cell Carcinoma and Endocervical Adenocarcinoma (TCGA)
HPA017931: , HPA020289:	unprognostic (1.64e-1)	unprognostic (2.95e-3)	unprognostic (1.48e-1)	unprognostic (2.32e-1)
CAB017717: , HPA031868:	unprognostic (9.46e-2)	unprognostic (7.23e-2)	unprognostic (1.01e-1)	unprognostic (4.68e-1)
CAB069889: , HPA075201:	unprognostic (1.04e-2)	unprognostic (2.61e-2)	unprognostic (9.75e-2)	unprognostic (2.19e-1)
HPA041564:	unprognostic (3.96e-2)	unprognostic (2.00e-1)	unprognostic (3.49e-1)	unprognostic (5.60e-2)
CAB001716: , HPA002199: , HPA074614:		unprognostic (5.03e-2)	unprognostic (1.00e-1)	
HPA049395: , HPA053288:			unprognostic (5.25e-2)	
HPA046723: , HPA058011:	unprognostic (4.36e-2)	unprognostic (8.72e-3)	unprognostic (1.88e-1)	unprognostic (3.59e-2)
HPA002380: , HPA076011:	unprognostic (9.49e-2)	unprognostic (3.68e-1)	unprognostic (1.30e-1)	unprognostic (9.60e-2)
CAB037136: , HPA048483:	unprognostic (1.23e-2)	unprognostic (3.14e-3)	unprognostic (1.03e-1)	unprognostic (8.03e-2)
HPA002476:	unprognostic (4.59e-3)	unprognostic (2.05e-1)	unprognostic (7.27e-2)	unprognostic (2.72e-2)
HPA038105:		unprognostic (8.02e-2)	unprognostic (3.70e-2)	unprognostic (3.81e-2)
CAB037299: , HPA054719:	unprognostic (9.74e-2)	unprognostic (1.82e-1)	unprognostic (5.75e-2)	
HPA032003: , HPA049718:	unprognostic (1.51e-1)	unprognostic (1.62e-3)		unprognostic (2.01e-3)
CAB004307: , CAB080432: , HPA001399: , HPA023884:	unprognostic (2.37e-2)	unprognostic (1.78e-1)	unprognostic (2.18e-2)	unprognostic (8.81e-2)
CAB001709: , HPA050670:	unprognostic (4.08e-2)	unprognostic (5.03e-2)	unprognostic (5.15e-2)	potential prognostic unfavorable (3.94e-4)
HPA030866: , HPA030867: , HPA030868:	unprognostic (5.38e-2)	unprognostic (2.73e-1)	unprognostic (1.02e-1)	unprognostic (2.42e-3)
HPA074034:	unprognostic (2.46e-1)	unprognostic (5.80e-2)	unprognostic (7.96e-2)	unprognostic (8.55e-2)
CAB010223: , HPA068616:	unprognostic (8.72e-3)	unprognostic (1.13e-1)	unprognostic (1.99e-1)	unprognostic (4.97e-2)
CAB005070: , HPA048344:		unprognostic (2.90e-1)	unprognostic (4.31e-3)	
HPA041328: , HPA044225:	unprognostic (4.22e-2)	unprognostic (1.72e-1)	unprognostic (1.60e-1)	unprognostic (1.30e-1)
CAB009794: , HPA035873: , HPA035874:	unprognostic (7.79e-3)	unprognostic (6.30e-2)	unprognostic (7.88e-2)	unprognostic (2.84e-3)
CAB009796: , HPA034509: , HPA034510:	unprognostic (1.35e-1)	unprognostic (1.25e-1)	unprognostic (2.21e-1)	unprognostic (2.93e-3)
CAB009797: , HPA035696:	unprognostic (2.34e-2)	unprognostic (1.01e-1)	unprognostic (1.09e-1)	unprognostic (1.46e-1)
HPA008984: , HPA012393:	unprognostic (1.16e-3)	unprognostic (6.20e-2)		unprognostic (5.00e-3)
HPA069175:	unprognostic (1.32e-2)	unprognostic (1.16e-2)		unprognostic (4.45e-2)
HPA042395: , HPA076279:	unprognostic (1.33e-1)	unprognostic (9.78e-2)		
HPA001478: , HPA050029:	unprognostic (1.62e-1)	unprognostic (3.32e-3)		unprognostic (2.23e-1)
CAB080543: , HPA044383:	unprognostic (1.90e-2)	unprognostic (7.77e-3)	unprognostic (6.77e-2)	unprognostic (6.62e-2)
HPA073747:	unprognostic (7.71e-2)	unprognostic (7.31e-2)	unprognostic (1.90e-2)	unprognostic (1.93e-2)
HPA074416:			unprognostic (1.98e-1)	
HPA075322:	unprognostic (4.34e-2)	unprognostic (4.20e-2)	unprognostic (1.49e-1)	unprognostic (5.93e-2)
CAB013496: , HPA030212: , HPA030213: , HPA030214: , HPA030215: , HPA049868:	unprognostic (8.10e-2)	potential prognostic unfavorable (4.57e-5)		potential prognostic unfavorable (1.36e-4)
CAB011682: , HPA064436: , HPA069474:			unprognostic (1.43e-1)	
HPA019010: , HPA019070: , HPA026643:	unprognostic (3.17e-1)	unprognostic (2.62e-1)	unprognostic (2.45e-2)	unprognostic (3.05e-2)
HPA000878: , HPA002940: , HPA004145: , HPA020111:	unprognostic (3.01e-2)	unprognostic (2.17e-1)	unprognostic (5.53e-2)	unprognostic (7.60e-2)
CAB026379: , HPA006344: , HPA056230:	unprognostic (2.32e-1)	unprognostic (5.50e-2)	unprognostic (8.87e-3)	unprognostic (2.09e-2)
CAB070163: , HPA020280: , HPA073633:	unprognostic (5.66e-1)	unprognostic (6.98e-3)	unprognostic (2.01e-1)	unprognostic (1.45e-2)
HPA020280: , HPA073633:	unprognostic (6.11e-3)	unprognostic (1.97e-2)	unprognostic (5.12e-1)	unprognostic (1.39e-3)
HPA041794: , HPA046300:	unprognostic (1.48e-2)	unprognostic (5.56e-2)	unprognostic (1.46e-1)	unprognostic (3.27e-2)
HPA010694:				
HPA010562:	unprognostic (9.30e-3)	unprognostic (3.06e-2)	unprognostic (7.88e-2)	unprognostic (1.46e-1)
HPA065214: , HPA065265:	unprognostic (1.03e-1)	unprognostic (3.02e-2)		unprognostic (2.42e-1)
HPA039458:				
HPA067290: , HPA067853:	unprognostic (1.44e-2)	unprognostic (1.57e-2)	unprognostic (1.23e-1)	unprognostic (4.66e-2)
CAB015178: , HPA008007: , HPA035970:	unprognostic (8.16e-2)	unprognostic (1.58e-1)	unprognostic (1.54e-3)	potential prognostic favorable (7.08e-5)
CAB013249: , CAB015179: , HPA038455:	unprognostic (2.14e-1)	unprognostic (1.36e-1)	unprognostic (3.27e-2)	unprognostic (2.74e-1)
HPA019358: , HPA021780:	unprognostic (1.64e-2)	unprognostic (2.94e-2)	unprognostic (2.34e-1)	unprognostic (1.69e-3)
HPA003968: , HPA065263:	unprognostic (3.13e-1)	unprognostic (3.49e-1)	unprognostic (4.92e-2)	unprognostic (1.04e-1)
HPA039488: , HPA043218:	unprognostic (1.48e-1)	unprognostic (9.99e-3)	unprognostic (8.13e-2)	unprognostic (1.11e-1)
HPA051886: , HPA063748:	unprognostic (3.42e-2)	unprognostic (1.55e-1)	unprognostic (3.64e-2)	unprognostic (1.52e-1)
HPA032148: , HPA057356:	unprognostic (1.13e-1)	unprognostic (2.70e-1)	unprognostic (3.35e-1)	unprognostic (5.07e-2)
HPA016624: , HPA051569:	unprognostic (2.10e-3)	unprognostic (2.67e-3)	unprognostic (1.23e-1)	unprognostic (1.83e-2)
HPA036276: , HPA057499:		unprognostic (1.67e-1)	unprognostic (4.05e-1)	
HPA038958:	unprognostic (1.31e-2)	potential prognostic unfavorable (7.82e-5)	unprognostic (3.28e-1)	potential prognostic unfavorable (3.96e-4)
CAB002417: , HPA004625:	unprognostic (9.29e-2)	unprognostic (3.34e-2)	unprognostic (1.53e-2)	unprognostic (1.16e-2)
CAB013023: , CAB035987: , CAB058693: , CAB080415: , HPA011271: , HPA011272:	unprognostic (2.41e-3)	unprognostic (9.20e-3)	unprognostic (1.75e-1)	unprognostic (1.32e-1)
CAB025135: , HPA018535: , HPA019569: , HPA019650:			unprognostic (3.08e-2)	
HPA013398: , HPA013431:	unprognostic (3.22e-1)	unprognostic (4.15e-1)	unprognostic (1.09e-1)	unprognostic (1.71e-2)
HPA036849: , HPA069870:	unprognostic (1.13e-1)	unprognostic (3.39e-2)	unprognostic (4.11e-2)	unprognostic (1.00e-1)
HPA038521: , HPA038522:	unprognostic (1.55e-1)	unprognostic (1.95e-1)	unprognostic (3.80e-2)	unprognostic (1.15e-1)
HPA017009: , HPA063903:	unprognostic (3.10e-1)	unprognostic (2.32e-2)	unprognostic (1.06e-1)	unprognostic (6.05e-2)
HPA013349: , HPA013362:	unprognostic (9.53e-2)	unprognostic (7.72e-2)	unprognostic (2.61e-1)	unprognostic (4.76e-2)
HPA041667: , HPA042093: , HPA079642:	unprognostic (2.65e-2)	unprognostic (3.50e-1)		unprognostic (4.47e-2)
HPA052462: , HPA058882:	unprognostic (5.17e-3)	unprognostic (3.19e-1)	unprognostic (1.54e-2)	unprognostic (2.03e-2)
HPA011138:	unprognostic (6.26e-2)	unprognostic (1.54e-1)	unprognostic (2.65e-2)	unprognostic (3.12e-2)
CAB001707: , HPA019206:	unprognostic (2.23e-1)	unprognostic (1.62e-1)	unprognostic (1.17e-2)	unprognostic (1.98e-1)
HPA062621: , HPA065947:				
HPA077460:		unprognostic (3.08e-1)	unprognostic (2.52e-1)	
HPA014924:	unprognostic (1.80e-3)	unprognostic (7.60e-2)	unprognostic (5.02e-2)	unprognostic (1.02e-3)
CAB005079: , CAB058689: , HPA014784:				
CAB080050: , CAB080051: , HPA066326:	unprognostic (2.66e-1)	unprognostic (3.40e-2)	unprognostic (6.52e-2)	unprognostic (5.01e-2)
HPA032126:	unprognostic (1.12e-1)	unprognostic (1.29e-2)	unprognostic (7.65e-3)	potential prognostic favorable (4.24e-4)
HPA041703: , HPA075891:	unprognostic (1.46e-1)	unprognostic (2.63e-2)	potential prognostic favorable (1.38e-5)	unprognostic (2.40e-1)
HPA035777: , HPA043231:	unprognostic (1.68e-2)	unprognostic (4.45e-1)	unprognostic (5.90e-2)	unprognostic (1.85e-1)
HPA036610: , HPA057861:	unprognostic (6.64e-2)	unprognostic (4.08e-2)	unprognostic (2.25e-1)	unprognostic (1.02e-3)
CAB020696: , HPA040391: , HPA064439:	unprognostic (9.02e-2)	unprognostic (1.42e-1)	unprognostic (4.32e-2)	unprognostic (4.50e-3)
HPA019816: , HPA079252:	unprognostic (6.65e-3)	unprognostic (1.81e-1)		unprognostic (5.90e-2)
CAB009502: , HPA012924: , HPA060784:	unprognostic (1.07e-1)	unprognostic (7.06e-3)	unprognostic (1.08e-1)	unprognostic (1.94e-1)
HPA011026: , HPA012037: , HPA014658:	unprognostic (1.28e-1)	unprognostic (4.34e-2)	unprognostic (8.45e-2)	unprognostic (2.02e-2)
HPA044443: , HPA063495:	unprognostic (1.44e-1)	unprognostic (2.19e-1)	unprognostic (3.16e-2)	unprognostic (6.77e-3)
HPA042689: , HPA049151: , HPA071867:	unprognostic (1.45e-3)	unprognostic (2.40e-1)	unprognostic (1.47e-1)	unprognostic (1.49e-3)
HPA017722: , HPA049195:	unprognostic (8.42e-3)	unprognostic (1.27e-1)		unprognostic (1.07e-1)
HPA020980: , HPA029504: , HPA044188: , HPA045619: , HPA045699:	unprognostic (5.02e-2)	unprognostic (1.24e-1)	unprognostic (2.84e-1)	unprognostic (8.43e-3)
HPA028927:	unprognostic (2.68e-1)	unprognostic (6.04e-2)	unprognostic (8.23e-2)	unprognostic (7.95e-2)
HPA034769: , HPA034770:				
HPA075177:	unprognostic (9.10e-3)	unprognostic (1.21e-1)	unprognostic (1.18e-1)	unprognostic (2.58e-2)
HPA039934: , HPA042109:	unprognostic (3.64e-2)	unprognostic (7.71e-2)	unprognostic (2.82e-3)	unprognostic (2.14e-3)
CAB016162: , HPA055264: , HPA063675:	unprognostic (8.66e-2)	unprognostic (1.42e-1)	unprognostic (1.91e-2)	unprognostic (1.80e-2)
CAB018615: , HPA068383:	unprognostic (1.00e-1)	unprognostic (7.36e-2)	unprognostic (1.99e-1)	unprognostic (1.94e-2)
HPA014899: , HPA015998:			potential prognostic favorable (1.27e-4)	
HPA042289:	unprognostic (1.27e-3)	unprognostic (4.22e-3)	potential prognostic unfavorable (2.09e-4)	unprognostic (3.78e-2)
HPA058870:	unprognostic (1.04e-1)	unprognostic (4.18e-2)		unprognostic (1.09e-1)
CAB037260: , HPA040471:	unprognostic (3.04e-1)	unprognostic (1.35e-2)	unprognostic (1.20e-2)	unprognostic (5.78e-2)
HPA050808:	unprognostic (1.53e-1)	unprognostic (6.67e-2)	unprognostic (7.05e-2)	unprognostic (2.29e-1)
HPA039526:	unprognostic (7.98e-2)			unprognostic (9.28e-3)
CAB020697: , HPA048963:	unprognostic (9.23e-2)	unprognostic (4.56e-1)	unprognostic (1.77e-2)	unprognostic (1.47e-3)
HPA035804: , HPA043204:				
CAB005605: , HPA011166: , HPA012945:	unprognostic (1.24e-1)	unprognostic (4.71e-2)	unprognostic (6.53e-2)	unprognostic (2.85e-3)
HPA001583: , HPA064779:				
CAB016118: , HPA040431:	unprognostic (3.94e-2)	unprognostic (2.47e-1)	unprognostic (1.57e-1)	unprognostic (2.30e-2)
HPA044279:	unprognostic (2.45e-1)	unprognostic (3.01e-1)	unprognostic (2.05e-1)	unprognostic (2.74e-1)
HPA031515: , HPA057316:	unprognostic (1.28e-1)	unprognostic (4.04e-2)	unprognostic (7.77e-2)	unprognostic (8.70e-3)
HPA039926: , HPA040033:		unprognostic (3.12e-2)	unprognostic (1.99e-1)	
HPA018673: , HPA018674:	unprognostic (1.79e-1)	unprognostic (9.13e-2)	unprognostic (7.27e-2)	unprognostic (4.08e-2)
HPA046680: , HPA061103:	unprognostic (1.46e-1)	unprognostic (1.30e-1)	unprognostic (2.50e-1)	unprognostic (2.09e-2)
CAB032501: , HPA037422: , HPA037423: , HPA075217:	unprognostic (1.99e-1)	unprognostic (1.61e-1)	unprognostic (2.47e-1)	unprognostic (3.47e-2)
CAB002572: , HPA006361:	unprognostic (6.33e-2)	unprognostic (1.23e-1)	unprognostic (1.58e-1)	unprognostic (1.73e-1)
CAB016358:	unprognostic (1.33e-1)	unprognostic (1.38e-3)	unprognostic (2.66e-1)	potential prognostic unfavorable (7.73e-5)
HPA023310: , HPA027421:	unprognostic (1.85e-1)	unprognostic (1.96e-1)	unprognostic (2.33e-1)	unprognostic (1.01e-1)
HPA019484: , HPA021257: , HPA023874: , HPA029503:	unprognostic (1.67e-1)	unprognostic (1.39e-1)	unprognostic (7.53e-2)	potential prognostic unfavorable (1.37e-4)
HPA045218: , HPA050333:	unprognostic (2.73e-1)	unprognostic (9.10e-3)	unprognostic (1.81e-1)	unprognostic (2.28e-2)
HPA003551: , HPA050841:	unprognostic (9.71e-2)	unprognostic (1.28e-1)	unprognostic (6.67e-2)	unprognostic (1.82e-2)
HPA040748: , HPA042038:	unprognostic (9.03e-3)	potential prognostic unfavorable (9.99e-5)	unprognostic (1.03e-1)	unprognostic (8.38e-2)
HPA038666: , HPA038667:	unprognostic (1.16e-1)	unprognostic (2.38e-2)	unprognostic (2.11e-3)	unprognostic (1.29e-3)
CAB009333: , CAB016291: , HPA038309: , HPA038310:	unprognostic (1.04e-1)	unprognostic (4.21e-2)	unprognostic (2.09e-1)	unprognostic (9.07e-2)
HPA017385: , HPA049014:	unprognostic (3.74e-2)	unprognostic (1.56e-1)	unprognostic (2.13e-1)	unprognostic (3.85e-2)
CAB032495: , HPA001048:	unprognostic (1.96e-2)		unprognostic (5.64e-2)	
CAB025806:	unprognostic (2.79e-2)	unprognostic (1.73e-2)	unprognostic (2.37e-1)	unprognostic (1.31e-2)
CAB004552: , CAB080447: , CAB080451: , CAB080452: , CAB080531: , HPA001328:	potential prognostic favorable (4.52e-4)	unprognostic (8.04e-3)	unprognostic (2.25e-1)	unprognostic (1.00e-1)
CAB016689: , CAB080300: , HPA001198: , HPA002028:	unprognostic (2.58e-1)	unprognostic (3.91e-2)	unprognostic (2.72e-1)	potential prognostic favorable (3.57e-4)
HPA014788: , HPA018176:	unprognostic (1.30e-1)	unprognostic (2.02e-1)	unprognostic (2.35e-1)	unprognostic (3.16e-1)
HPA038387: , HPA038388:	unprognostic (2.33e-1)	unprognostic (1.72e-1)	unprognostic (2.42e-1)	unprognostic (4.69e-2)
HPA011888: , HPA011889:	unprognostic (1.58e-2)	unprognostic (2.54e-2)	unprognostic (4.46e-2)	unprognostic (1.19e-2)
HPA026483:	unprognostic (1.74e-2)	unprognostic (3.81e-2)	unprognostic (2.67e-2)	unprognostic (3.18e-1)
HPA011044: , HPA075369:	unprognostic (2.78e-1)	unprognostic (3.20e-3)	unprognostic (4.86e-2)	unprognostic (4.31e-1)
HPA077621:				

CAB005100: , CAB017107: , HPA055207:	unprognostic (2.26e-2)	unprognostic (3.73e-2)	unprognostic (4.90e-1)	potential prognostic unfavorable (9.05e-4)
CAB079031: , HPA039796:	unprognostic (3.73e-2)	unprognostic (9.56e-2)	unprognostic (7.20e-2)	
CAB037266:	unprognostic (6.41e-2)	unprognostic (1.23e-1)	unprognostic (1.21e-2)	unprognostic (3.84e-1)
HPA008070: , HPA046515:	unprognostic (2.35e-1)	unprognostic (4.28e-2)	unprognostic (1.31e-1)	unprognostic (3.87e-2)
CAB000006: , HPA008066: , HPA017330:	unprognostic (3.34e-2)	unprognostic (1.63e-1)	unprognostic (1.72e-1)	unprognostic (1.82e-2)
HPA029038: , HPA029039:	unprognostic (1.71e-1)	unprognostic (1.09e-2)		unprognostic (2.44e-1)
HPA071906: , HPA075184:	unprognostic (7.46e-3)	unprognostic (1.71e-1)	unprognostic (2.58e-2)	unprognostic (9.24e-2)
CAB009491: , HPA036881: , HPA036882: , HPA075906:	unprognostic (1.94e-1)	unprognostic (1.81e-1)	unprognostic (2.15e-1)	unprognostic (1.25e-2)
HPA049838: , HPA074213:	unprognostic (1.38e-1)	unprognostic (1.48e-1)		unprognostic (4.13e-2)
HPA039325: , HPA062122:	unprognostic (2.07e-2)	unprognostic (8.24e-3)		unprognostic (1.25e-1)
HPA020973: , HPA020987: , HPA021021:				
HPA034598:	unprognostic (1.47e-2)	unprognostic (8.39e-2)	unprognostic (2.39e-2)	unprognostic (1.45e-1)
HPA041708: , HPA046765:	unprognostic (1.86e-1)	unprognostic (1.03e-1)	unprognostic (2.71e-1)	unprognostic (4.33e-1)
HPA058346:	unprognostic (1.94e-1)	unprognostic (2.27e-2)	unprognostic (3.55e-2)	unprognostic (1.24e-2)
HPA038101: , HPA038102:	unprognostic (2.43e-2)	unprognostic (9.29e-2)	unprognostic (2.49e-2)	unprognostic (6.88e-2)
HPA002726: , HPA005832: , HPA064745:	unprognostic (1.46e-2)	unprognostic (1.27e-1)	unprognostic (1.23e-1)	unprognostic (7.03e-3)
HPA054183:	unprognostic (7.47e-3)	unprognostic (1.66e-1)	unprognostic (5.06e-3)	unprognostic (3.12e-2)
HPA073900:				
HPA079548:	unprognostic (2.47e-1)		unprognostic (3.00e-2)	
HPA046825:	unprognostic (1.60e-1)	unprognostic (2.87e-2)	unprognostic (1.60e-1)	unprognostic (9.31e-2)
CAB003793: , HPA063255:	unprognostic (1.86e-1)	unprognostic (1.05e-2)	unprognostic (7.54e-2)	unprognostic (2.92e-3)
HPA031613:	unprognostic (4.81e-3)	unprognostic (5.27e-3)	unprognostic (3.82e-3)	unprognostic (2.33e-2)
HPA043238:	unprognostic (8.87e-2)	unprognostic (3.06e-1)	unprognostic (3.77e-2)	unprognostic (1.76e-1)
HPA008992: , HPA015723:	unprognostic (1.68e-3)	unprognostic (1.66e-1)	unprognostic (2.46e-1)	unprognostic (1.58e-1)
CAB002432: , HPA046404: , HPA051974:	unprognostic (3.36e-1)	unprognostic (3.58e-1)	unprognostic (2.24e-1)	unprognostic (7.01e-2)
HPA003740:	unprognostic (1.08e-1)	unprognostic (4.66e-2)	unprognostic (1.85e-1)	unprognostic (3.27e-2)
CAB002430: , HPA003883:	unprognostic (7.62e-3)	unprognostic (1.57e-3)	unprognostic (3.33e-2)	unprognostic (3.23e-3)
HPA068512:			unprognostic (1.20e-1)	unprognostic (5.98e-3)
HPA015715: , HPA015865:		unprognostic (1.93e-3)	unprognostic (2.18e-1)	unprognostic (5.26e-2)
CAB076385: , CAB080537: , HPA003528:	unprognostic (1.57e-1)	unprognostic (6.40e-2)	unprognostic (9.65e-3)	unprognostic (1.01e-1)
HPA041233: , HPA070003:	unprognostic (2.55e-1)	unprognostic (1.55e-1)	unprognostic (6.70e-2)	
CAB004352: , HPA003267: , HPA003326:	potential prognostic favorable (9.26e-6)	unprognostic (7.09e-3)	unprognostic (2.46e-1)	unprognostic (2.58e-2)
CAB011442: , CAB079996: , CAB080305: , HPA035832:	unprognostic (1.63e-1)	unprognostic (1.82e-2)	unprognostic (5.32e-2)	unprognostic (2.66e-1)
CAB002493: , CAB025255: , HPA022132: , HPA052381:	unprognostic (1.95e-1)	unprognostic (1.10e-2)	unprognostic (2.37e-1)	unprognostic (1.11e-2)
CAB000010: , CAB072863: , CAB072864: , HPA040957: , HPA043955:	unprognostic (2.34e-2)	potential prognostic favorable (1.61e-4)	unprognostic (2.89e-2)	potential prognostic favorable (2.43e-4)
CAB017520: , HPA038494:	unprognostic (3.30e-2)	unprognostic (1.87e-2)	unprognostic (4.46e-1)	unprognostic (3.75e-3)
CAB000011: , CAB068180: , HPA004252: , HPA004472:	unprognostic (2.67e-3)	unprognostic (8.77e-3)	unprognostic (1.16e-1)	unprognostic (6.22e-2)
CAB002495: , CAB072868: , CAB080468: , HPA031567: , HPA031568:	unprognostic (3.20e-2)	unprognostic (1.68e-2)	unprognostic (1.28e-1)	unprognostic (1.24e-1)
HPA045827: , HPA072480:		potential prognostic favorable (2.15e-4)	unprognostic (7.50e-3)	
CAB000112: , CAB000316: , HPA005785:	unprognostic (1.93e-3)	unprognostic (1.85e-1)	unprognostic (3.84e-1)	unprognostic (7.75e-2)
CAB016055: , HPA044659:	unprognostic (2.73e-2)	unprognostic (5.32e-2)	unprognostic (1.84e-1)	unprognostic (1.19e-1)
CAB009751: , HPA009178: , HPA044107:	unprognostic (1.46e-1)	unprognostic (1.55e-2)	potential prognostic favorable (2.92e-5)	potential prognostic favorable (3.09e-4)
HPA063316: , HPA068020: , HPA070502:	unprognostic (4.21e-2)	unprognostic (2.15e-2)	unprognostic (4.87e-1)	unprognostic (2.31e-1)
CAB004319: , HPA026802:	unprognostic (1.96e-1)	unprognostic (1.97e-1)	unprognostic (3.93e-1)	unprognostic (6.14e-2)
CAB000012: , CAB075722: , HPA037756:	unprognostic (5.62e-2)	unprognostic (2.24e-3)	unprognostic (8.45e-2)	unprognostic (1.66e-2)
CAB004353: , HPA029164:	unprognostic (5.53e-3)	unprognostic (1.52e-3)	unprognostic (6.39e-2)	unprognostic (1.32e-3)
CAB002490:	unprognostic (2.47e-1)	unprognostic (1.03e-1)	unprognostic (5.66e-2)	unprognostic (7.09e-2)
HPA039404: , HPA040787:	unprognostic (1.39e-3)	unprognostic (1.93e-2)	unprognostic (2.75e-1)	unprognostic (8.41e-2)
HPA006379: , HPA076536:	unprognostic (1.84e-2)	unprognostic (1.18e-1)	unprognostic (2.93e-1)	unprognostic (2.50e-2)
HPA034986: , HPA061792:	unprognostic (9.31e-3)	unprognostic (1.01e-1)	unprognostic (2.95e-2)	unprognostic (1.07e-2)
HPA027634:	unprognostic (5.57e-2)	unprognostic (3.05e-2)	unprognostic (2.20e-2)	unprognostic (1.95e-1)
CAB000087: , CAB028364: , CAB072855: , CAB072856: , CAB072857: , HPA004812:	unprognostic (2.74e-1)	unprognostic (3.08e-2)	unprognostic (8.05e-3)	unprognostic (6.64e-2)
CAB025863: , HPA001380:	unprognostic (1.26e-1)	potential prognostic unfavorable (9.77e-4)	unprognostic (2.46e-1)	unprognostic (2.64e-2)
HPA009139: , HPA070961:				
CAB025143: , HPA023614: , HPA023616: , HPA026556:	unprognostic (1.59e-1)			unprognostic (3.13e-2)
CAB000141: , CAB018580: , CAB078686: , CAB080169: , HPA058574:	unprognostic (2.90e-1)	unprognostic (1.34e-1)	unprognostic (7.28e-2)	unprognostic (2.13e-2)
HPA015722: , HPA047578:	unprognostic (2.66e-1)		unprognostic (4.07e-2)	unprognostic (1.79e-3)
CAB002487: , HPA001767: , HPA002974:	unprognostic (6.93e-3)	unprognostic (2.18e-1)	unprognostic (3.26e-1)	unprognostic (1.03e-2)
HPA015613:				
CAB028366: , HPA075875:	unprognostic (9.32e-2)	unprognostic (1.51e-1)	unprognostic (1.01e-1)	unprognostic (1.68e-2)
CAB025274: , HPA007047: , HPA007403: , HPA007456:	unprognostic (1.07e-1)	unprognostic (3.08e-2)	unprognostic (4.47e-1)	unprognostic (1.56e-2)
CAB016535: , HPA000974: , HPA001366:	unprognostic (2.67e-2)	unprognostic (1.72e-3)	unprognostic (1.95e-1)	unprognostic (1.05e-1)
CAB008909: , HPA064535:	unprognostic (1.69e-1)	unprognostic (2.79e-2)	unprognostic (1.61e-1)	unprognostic (2.53e-2)
CAB000021: , CAB000022: , HPA011041: , HPA019758:	unprognostic (1.28e-1)	unprognostic (1.24e-2)	unprognostic (3.30e-1)	unprognostic (1.37e-1)
HPA069621: , HPA073910:	unprognostic (1.56e-1)		unprognostic (4.49e-2)	unprognostic (1.62e-1)
HPA052976: , HPA060521:	unprognostic (5.85e-2)	unprognostic (1.46e-1)	unprognostic (1.31e-1)	unprognostic (1.68e-1)
HPA017080: , HPA044889:	unprognostic (1.28e-1)	unprognostic (2.31e-1)		unprognostic (3.43e-2)
CAB022157: , CAB025216: , HPA019044:	unprognostic (7.43e-2)	unprognostic (3.75e-1)	potential prognostic favorable (2.39e-4)	unprognostic (2.25e-2)
CAB017193: , HPA027586: , HPA027587: , HPA027657:	unprognostic (2.51e-1)	unprognostic (7.18e-2)	potential prognostic favorable (2.84e-4)	unprognostic (6.86e-2)
CAB013500: , HPA056911: , HPA069214:	unprognostic (3.53e-3)	unprognostic (2.39e-2)	unprognostic (1.67e-2)	unprognostic (2.34e-1)
HPA026782: , HPA026784: , HPA076603:	unprognostic (3.50e-1)	unprognostic (1.62e-1)	unprognostic (1.95e-2)	unprognostic (1.34e-2)
HPA024106: , HPA048036:	unprognostic (2.47e-1)	unprognostic (7.72e-2)	unprognostic (2.81e-1)	
CAB022444: , HPA072083:			unprognostic (2.48e-1)	
HPA071554:	unprognostic (1.62e-1)	unprognostic (2.58e-1)	unprognostic (3.15e-3)	unprognostic (1.30e-1)
HPA048348:				
HPA029430:				unprognostic (2.28e-1)
HPA054381:	unprognostic (4.42e-2)	unprognostic (1.93e-1)	unprognostic (1.91e-1)	unprognostic (1.13e-1)
HPA059484:				unprognostic (1.45e-1)
HPA065404:				
CAB012991: , HPA042413: , HPA048825: , HPA074310:	unprognostic (3.21e-2)	unprognostic (2.44e-1)	unprognostic (6.97e-2)	unprognostic (1.05e-1)
HPA047192:	unprognostic (1.31e-1)	unprognostic (8.10e-3)	unprognostic (5.03e-1)	unprognostic (9.65e-2)
HPA000401: , HPA003213:	unprognostic (1.38e-2)	unprognostic (2.92e-3)	unprognostic (1.86e-2)	unprognostic (1.42e-1)
CAB009789: , HPA013166: , HPA013657:			unprognostic (2.95e-1)	
HPA014412:			unprognostic (2.19e-1)	
CAB002609: , HPA051548: , HPA074528:			unprognostic (1.12e-1)	unprognostic (2.45e-2)
HPA073105:			unprognostic (3.37e-1)	
HPA014361:	unprognostic (1.55e-1)	unprognostic (2.30e-1)	unprognostic (1.79e-1)	potential prognostic favorable (6.55e-4)
CAB002610:	unprognostic (2.09e-1)	unprognostic (2.59e-1)	unprognostic (7.79e-2)	unprognostic (1.65e-1)
CAB013063: , HPA014703: , HPA073662:	unprognostic (4.18e-2)	unprognostic (2.01e-2)	unprognostic (2.39e-1)	unprognostic (8.84e-2)
HPA076613:	unprognostic (8.56e-2)	unprognostic (4.38e-2)	unprognostic (1.98e-1)	unprognostic (8.67e-2)
HPA054137:	unprognostic (7.73e-2)	unprognostic (1.68e-2)	unprognostic (7.06e-2)	unprognostic (3.89e-2)
HPA047381: , HPA060101:	unprognostic (6.48e-3)	unprognostic (1.88e-2)	unprognostic (1.13e-1)	unprognostic (1.10e-1)
HPA008019: , HPA060804:	unprognostic (2.90e-1)	unprognostic (5.65e-2)	unprognostic (1.58e-3)	unprognostic (4.17e-3)
HPA074371:	unprognostic (6.11e-3)	unprognostic (1.04e-1)	potential prognostic favorable (5.59e-4)	unprognostic (3.55e-2)
CAB002665: , HPA050918: , HPA054667:	unprognostic (5.09e-3)	unprognostic (4.59e-3)	unprognostic (2.76e-2)	unprognostic (3.51e-1)
CAB003692: , HPA036458:	unprognostic (5.51e-2)	unprognostic (1.76e-1)	unprognostic (7.25e-2)	unprognostic (1.11e-1)
HPA010525:	unprognostic (2.85e-2)		unprognostic (1.16e-1)	
HPA076168:	unprognostic (1.03e-1)	unprognostic (7.73e-2)	unprognostic (1.14e-1)	unprognostic (6.01e-3)
HPA017732:	unprognostic (4.69e-2)	unprognostic (2.13e-3)	unprognostic (1.09e-1)	unprognostic (1.17e-2)
HPA042256: , HPA069945:		unprognostic (8.60e-2)	unprognostic (1.58e-1)	
HPA040353: , HPA054470:	unprognostic (2.80e-2)	unprognostic (1.88e-2)	unprognostic (2.40e-1)	potential prognostic unfavorable (4.29e-4)
HPA043673: , HPA052963:	unprognostic (1.00e-1)	potential prognostic favorable (1.86e-5)	unprognostic (3.71e-1)	unprognostic (7.85e-2)
HPA007029: , HPA030920: , HPA038799:	unprognostic (6.73e-2)	unprognostic (2.43e-1)	unprognostic (5.51e-2)	unprognostic (5.87e-3)
CAB017616: , HPA040113: , HPA070456:	unprognostic (6.53e-2)	unprognostic (8.95e-3)	unprognostic (6.05e-2)	unprognostic (1.55e-2)
HPA040938: , HPA041970:	unprognostic (1.50e-1)	potential prognostic unfavorable (1.14e-4)	unprognostic (2.29e-1)	unprognostic (1.03e-1)
CAB002491: , CAB016271: , HPA042455: , HPA043579: , HPA049348:	unprognostic (3.19e-1)		unprognostic (6.73e-3)	
HPA013835:	unprognostic (3.69e-2)	unprognostic (3.69e-2)	unprognostic (5.87e-2)	unprognostic (3.55e-1)
HPA073345:				
CAB002053: , CAB040560: , HPA057100:	unprognostic (9.83e-2)	unprognostic (2.70e-2)	unprognostic (3.07e-1)	unprognostic (1.02e-1)
HPA076624:	unprognostic (1.23e-1)	unprognostic (8.11e-3)	unprognostic (1.84e-1)	unprognostic (7.29e-2)
CAB008970: , HPA012323:	unprognostic (1.28e-1)	unprognostic (6.19e-2)	unprognostic (2.37e-1)	unprognostic (1.20e-1)
CAB015410: , HPA070888: , HPA072593:	unprognostic (1.36e-1)	unprognostic (6.98e-2)	unprognostic (1.83e-1)	unprognostic (9.43e-3)
CAB021089: , HPA046119: , HPA063535:	unprognostic (9.90e-2)	unprognostic (7.56e-2)	unprognostic (4.19e-1)	unprognostic (1.99e-2)
CAB000108: , CAB001950: , HPA029159: , HPA029160:	unprognostic (1.79e-2)	unprognostic (8.56e-2)	unprognostic (1.15e-1)	unprognostic (5.61e-3)
CAB003837: , HPA015954: , HPA015955:	unprognostic (1.97e-1)	unprognostic (2.00e-2)	unprognostic (9.18e-2)	unprognostic (1.02e-2)
CAB011235: , HPA057242:	unprognostic (3.50e-1)	unprognostic (6.86e-2)	unprognostic (1.76e-2)	unprognostic (5.63e-3)
CAB026192: , CAB080311: , HPA040361:	unprognostic (1.06e-1)	unprognostic (8.24e-3)	unprognostic (2.57e-1)	unprognostic (1.32e-3)
CAB032478: , HPA046587:		unprognostic (8.51e-2)	unprognostic (1.09e-1)	
CAB005103: , HPA003342: , HPA030411: , HPA030412:	unprognostic (7.24e-3)	unprognostic (2.20e-1)	unprognostic (1.24e-3)	unprognostic (1.24e-1)
CAB016268: , CAB080545: , HPA031999: , HPA032017:	unprognostic (1.85e-2)		unprognostic (2.82e-3)	unprognostic (2.27e-2)
HPA014757:	unprognostic (2.31e-2)	unprognostic (2.72e-1)	unprognostic (6.85e-2)	unprognostic (1.64e-2)
CAB014872: , HPA060662:	unprognostic (1.04e-2)	unprognostic (5.06e-2)	unprognostic (1.84e-1)	unprognostic (1.85e-1)
HPA004065: , HPA071573:	unprognostic (1.26e-2)	unprognostic (2.20e-2)	unprognostic (3.74e-3)	unprognostic (8.09e-2)
CAB009314: , HPA028888:	unprognostic (5.29e-2)	unprognostic (1.85e-1)	unprognostic (8.24e-2)	unprognostic (2.36e-2)
CAB001960: , CAB016353: , HPA070563:	unprognostic (8.32e-2)	unprognostic (1.10e-1)	unprognostic (7.72e-2)	unprognostic (2.01e-2)
HPA069377:	unprognostic (3.75e-1)	unprognostic (1.27e-2)	unprognostic (2.33e-1)	unprognostic (1.39e-2)
HPA020265: , HPA027735:	unprognostic (7.80e-2)	unprognostic (1.40e-1)	unprognostic (3.37e-1)	unprognostic (3.37e-1)
CAB017634: , HPA010742: , HPA010743: , HPA010800:				
CAB010162: , CAB025656: , HPA074209:	unprognostic (1.77e-1)	unprognostic (6.79e-2)	unprognostic (1.26e-1)	potential prognostic unfavorable (8.28e-4)

HPA070112:	unprognostic (4.93e-3)	unprognostic (5.52e-2)	unprognostic (1.10e-2)	unprognostic (6.72e-3)
HPA039533: , HPA040355: , HPA061538:	unprognostic (7.07e-2)	unprognostic (1.74e-1)	unprognostic (1.19e-1)	unprognostic (8.86e-3)
HPA032151:	unprognostic (5.77e-2)	unprognostic (1.35e-1)	unprognostic (1.43e-2)	potential prognostic unfavorable (3.05e-4)
HPA044692: , HPA052516:	unprognostic (1.00e-1)	unprognostic (1.41e-2)	unprognostic (1.40e-2)	unprognostic (7.64e-3)
CAB016307: , HPA069593:	unprognostic (6.62e-2)	unprognostic (2.29e-2)	unprognostic (2.42e-2)	unprognostic (4.40e-3)
CAB001999: , CAB002000: , HPA010122: , HPA071006:	unprognostic (1.24e-1)	unprognostic (5.94e-2)	unprognostic (2.91e-1)	unprognostic (4.86e-2)
HPA001746:	unprognostic (2.49e-2)	unprognostic (9.68e-2)	unprognostic (2.40e-1)	unprognostic (5.97e-3)
CAB004995: , HPA028383: , HPA028385:	unprognostic (7.98e-2)	unprognostic (7.32e-2)	unprognostic (3.09e-1)	unprognostic (8.77e-3)
HPA035215: , HPA035216:	unprognostic (1.22e-1)	unprognostic (1.92e-2)	unprognostic (2.62e-3)	unprognostic (1.06e-3)
HPA071516:	unprognostic (1.13e-1)	unprognostic (2.18e-1)	unprognostic (4.28e-1)	unprognostic (2.56e-1)
HPA003218: , HPA003219: , HPA073349:	unprognostic (5.68e-2)	unprognostic (1.18e-1)	potential prognostic favorable (6.03e-4)	unprognostic (1.03e-1)
CAB004379: , HPA002862: , HPA066571: , HPA069121:	unprognostic (1.37e-1)	unprognostic (6.10e-2)	unprognostic (1.15e-3)	unprognostic (5.51e-3)
HPA077312:	unprognostic (2.77e-3)	unprognostic (7.49e-2)	unprognostic (6.01e-2)	unprognostic (2.17e-1)
HPA009426: , HPA012783:	unprognostic (7.53e-2)	unprognostic (3.14e-2)	unprognostic (2.54e-1)	unprognostic (1.94e-1)
HPA062682:			unprognostic (1.24e-1)	
HPA011911: , HPA012615:	unprognostic (1.77e-2)	unprognostic (1.48e-2)	unprognostic (3.79e-1)	unprognostic (1.95e-1)
CAB037328: , HPA049265: , HPA073937:	unprognostic (2.85e-2)	unprognostic (1.09e-2)	unprognostic (1.76e-1)	unprognostic (1.41e-1)
CAB025122: , HPA004896:	unprognostic (7.88e-2)	unprognostic (2.01e-2)	unprognostic (4.97e-1)	potential prognostic unfavorable (1.72e-4)
CAB037324: , HPA045840: , HPA054950:	unprognostic (5.26e-2)	unprognostic (8.88e-2)	unprognostic (2.63e-1)	unprognostic (9.03e-2)
CAB015196: , HPA071177:	unprognostic (1.75e-1)	unprognostic (6.17e-2)	unprognostic (1.32e-1)	unprognostic (6.70e-2)
CAB002510: , HPA017071: , HPA021945:	unprognostic (9.90e-2)	unprognostic (1.32e-1)	unprognostic (1.34e-1)	unprognostic (1.21e-2)
HPA063337:	unprognostic (3.62e-1)	unprognostic (2.93e-1)	unprognostic (1.39e-1)	unprognostic (2.83e-2)
CAB018957: , HPA013774:	unprognostic (1.06e-1)	unprognostic (8.17e-2)	unprognostic (3.08e-2)	unprognostic (5.06e-2)
CAB017842: , HPA027546:	unprognostic (2.40e-1)	unprognostic (1.63e-1)	unprognostic (1.29e-2)	unprognostic (2.14e-1)
CAB031489: , HPA067188:	unprognostic (2.86e-1)	unprognostic (1.97e-2)	unprognostic (4.18e-3)	unprognostic (1.81e-2)
HPA022859: , HPA023092: , HPA023402:	unprognostic (1.65e-1)	unprognostic (4.36e-2)	unprognostic (1.30e-1)	unprognostic (6.48e-3)
HPA038089: , HPA071831:		unprognostic (6.40e-2)	unprognostic (4.57e-1)	
CAB000035: , CAB068186: , CAB073534: , CAB080313: , CAB080314: , CAB080473:	unprognostic (7.14e-2)	unprognostic (2.28e-2)	unprognostic (9.66e-2)	unprognostic (2.14e-3)
HPA035468: , HPA035469:	unprognostic (3.78e-2)	unprognostic (1.10e-1)	unprognostic (2.53e-1)	unprognostic (6.34e-3)
HPA049890: , HPA049986: , HPA066751: , HPA067747:	unprognostic (2.37e-1)	unprognostic (1.86e-1)	unprognostic (2.83e-3)	unprognostic (1.60e-2)
HPA047394: , HPA049890: , HPA049986: , HPA066751:	unprognostic (1.87e-1)	unprognostic (1.21e-1)	unprognostic (1.18e-1)	unprognostic (7.23e-3)
HPA049890: , HPA049986: , HPA066751:	unprognostic (1.77e-1)	unprognostic (7.39e-2)	unprognostic (2.23e-1)	unprognostic (7.85e-2)
HPA029869: , HPA070749:		unprognostic (3.13e-1)		unprognostic (6.59e-2)
HPA028448: , HPA028696:	unprognostic (1.44e-1)	unprognostic (2.64e-2)	unprognostic (9.41e-2)	unprognostic (2.23e-3)
CAB000096: , CAB072873: , HPA011862: , HPA067440:	unprognostic (1.19e-1)	unprognostic (1.93e-1)	unprognostic (3.89e-3)	unprognostic (7.97e-3)
CAB018614: , CAB069394: , CAB080034: , HPA068284: , HPA068721:	potential prognostic unfavorable (1.29e-4)	potential prognostic unfavorable (6.67e-4)	unprognostic (1.73e-1)	unprognostic (5.66e-3)
CAB000063: , CAB073539: , CAB079023: , HPA068284: , HPA068721:	potential prognostic favorable (6.50e-4)	unprognostic (2.31e-1)	unprognostic (1.22e-1)	unprognostic (4.55e-2)
HPA000793: , HPA068284: , HPA068721:	unprognostic (4.90e-3)	unprognostic (1.07e-1)	unprognostic (1.45e-1)	unprognostic (2.83e-2)
HPA038355:	unprognostic (7.66e-2)	unprognostic (8.65e-2)	unprognostic (1.22e-1)	
HPA000514: , HPA003027:	unprognostic (5.19e-1)	unprognostic (9.77e-3)	unprognostic (2.19e-1)	unprognostic (1.38e-1)
HPA054104: , HPA056817:	potential prognostic favorable (6.57e-5)	unprognostic (1.36e-1)	unprognostic (9.41e-2)	unprognostic (1.49e-2)
HPA005730: , HPA006642:	unprognostic (9.57e-3)	unprognostic (7.85e-2)	unprognostic (6.65e-2)	unprognostic (1.60e-2)
HPA028605:	unprognostic (1.77e-1)	unprognostic (1.37e-2)	unprognostic (9.32e-2)	unprognostic (2.51e-1)
HPA037563: , HPA037564:	unprognostic (1.83e-2)	unprognostic (7.96e-3)	unprognostic (2.26e-1)	unprognostic (7.22e-2)
CAB003809: , CAB030012: , CAB055098: , HPA026761: , HPA067463:	unprognostic (3.41e-2)	unprognostic (1.52e-2)	unprognostic (8.29e-3)	unprognostic (2.22e-1)
CAB010464:	unprognostic (1.53e-2)	unprognostic (7.88e-2)	unprognostic (2.56e-1)	unprognostic (5.74e-2)
CAB010462: , HPA069390:	unprognostic (3.91e-3)	unprognostic (3.10e-3)	unprognostic (2.82e-1)	unprognostic (5.48e-2)
HPA034528:				
HPA067740:		unprognostic (1.66e-1)	unprognostic (3.12e-1)	unprognostic (2.54e-2)
CAB013647: , HPA071200:	unprognostic (8.42e-2)	unprognostic (1.03e-2)	unprognostic (8.41e-2)	unprognostic (6.28e-3)
HPA077654:	unprognostic (6.99e-2)	unprognostic (2.81e-1)	unprognostic (1.64e-1)	unprognostic (4.71e-2)
CAB000043: , CAB020416: , CAB062555: , HPA001060: , HPA001338: , HPA001383: ,	unprognostic (6.10e-2)	unprognostic (5.53e-2)	unprognostic (4.02e-2)	unprognostic (2.28e-1)
CAB025331: , HPA045396: , HPA070524:	unprognostic (7.09e-2)	unprognostic (1.97e-1)	unprognostic (5.39e-2)	unprognostic (3.58e-1)
HPA048606: , HPA059863:	potential prognostic favorable (4.28e-4)	unprognostic (2.52e-1)		unprognostic (4.15e-2)
HPA002132: , HPA070823:	unprognostic (2.24e-2)	unprognostic (5.02e-2)	unprognostic (4.04e-1)	unprognostic (6.49e-3)
HPA045033:	unprognostic (3.34e-1)	unprognostic (1.21e-1)	unprognostic (2.23e-1)	unprognostic (1.44e-2)
CAB004035: , CAB047324: , CAB075739: , HPA021616:	unprognostic (3.75e-1)	unprognostic (2.88e-3)	unprognostic (1.63e-1)	unprognostic (2.90e-2)
CAB004671: , HPA043616: , HPA061700:	unprognostic (2.88e-1)	unprognostic (1.56e-3)	unprognostic (5.01e-2)	unprognostic (1.28e-1)
HPA031401: , HPA042340:	unprognostic (2.84e-1)	unprognostic (3.48e-1)	unprognostic (5.83e-2)	unprognostic (7.16e-2)
HPA027444:	unprognostic (4.87e-2)	unprognostic (4.15e-2)	unprognostic (3.99e-2)	unprognostic (1.23e-1)
CAB005192: , CAB015417: , HPA006461: , HPA056108:	unprognostic (2.78e-2)	unprognostic (6.23e-2)	unprognostic (8.08e-2)	potential prognostic unfavorable (8.58e-6)
HPA025287: , HPA072750:	unprognostic (5.39e-2)	unprognostic (1.25e-1)	unprognostic (3.36e-1)	unprognostic (3.68e-3)
HPA003240: , HPA061783:		unprognostic (1.97e-1)	unprognostic (1.38e-1)	unprognostic (1.54e-2)
HPA014050:			unprognostic (1.77e-1)	
HPA026910:	unprognostic (5.28e-2)	unprognostic (5.91e-2)	unprognostic (1.22e-1)	unprognostic (1.68e-2)
HPA010718: , HPA010776: , HPA014730:	unprognostic (2.04e-1)	unprognostic (1.41e-2)	unprognostic (1.23e-1)	unprognostic (5.86e-2)
HPA038545: , HPA038546:	unprognostic (1.76e-3)	unprognostic (4.06e-1)	unprognostic (6.82e-2)	unprognostic (4.10e-2)
HPA003910:	unprognostic (4.08e-2)	unprognostic (3.91e-3)		unprognostic (7.05e-3)
HPA048933:				
HPA042563:				
HPA000911: , HPA076516:	unprognostic (4.63e-2)	unprognostic (2.92e-1)	unprognostic (8.95e-2)	unprognostic (1.57e-1)
HPA014436: , HPA014536: , HPA019191:	unprognostic (3.03e-1)	unprognostic (1.58e-1)	unprognostic (1.03e-1)	unprognostic (9.08e-2)
CAB020817: , HPA001040: , HPA001391:	unprognostic (1.10e-1)	unprognostic (2.90e-2)	unprognostic (2.95e-1)	unprognostic (5.24e-2)
CAB019322: , HPA004747: , HPA004886:	unprognostic (2.33e-1)	unprognostic (7.50e-2)	unprognostic (2.44e-1)	unprognostic (5.62e-3)
CAB007766: , HPA001393:	unprognostic (2.74e-1)	unprognostic (3.06e-2)	unprognostic (9.26e-3)	potential prognostic unfavorable (1.29e-4)
CAB004395: , HPA001396: , HPA071038:	unprognostic (3.02e-1)	unprognostic (2.06e-1)	unprognostic (3.69e-1)	unprognostic (2.36e-3)
HPA056033:	unprognostic (3.38e-3)	unprognostic (2.91e-1)	unprognostic (1.88e-1)	unprognostic (1.58e-1)
CAB068189: , CAB068190: , HPA011740: , HPA014290:	unprognostic (1.82e-1)	unprognostic (2.40e-2)	unprognostic (2.03e-1)	unprognostic (3.42e-3)
HPA067906: , HPA074389:	unprognostic (4.13e-2)	unprognostic (1.18e-1)	unprognostic (7.15e-2)	unprognostic (1.96e-1)
HPA002552: , HPA023201:	unprognostic (1.38e-1)	unprognostic (1.05e-1)	unprognostic (3.44e-1)	unprognostic (2.68e-1)
HPA019635: , HPA019691: , HPA022119: , HPA067742:	unprognostic (1.56e-1)	unprognostic (5.18e-2)	unprognostic (1.65e-2)	unprognostic (1.17e-3)
HPA059452:	unprognostic (1.33e-1)	unprognostic (4.30e-2)	unprognostic (3.10e-1)	unprognostic (7.69e-2)
HPA002861:	unprognostic (1.39e-1)	unprognostic (4.78e-2)	unprognostic (1.29e-1)	unprognostic (1.94e-1)
CAB005034: , CAB018387: , HPA023887: , HPA063770:	unprognostic (3.58e-1)	unprognostic (1.91e-2)	unprognostic (9.86e-2)	unprognostic (5.15e-2)
CAB013008:	unprognostic (2.07e-2)	unprognostic (1.98e-1)	unprognostic (4.30e-2)	unprognostic (1.14e-1)
HPA042328: , HPA074833:	unprognostic (5.42e-2)	unprognostic (1.25e-1)	unprognostic (6.24e-3)	unprognostic (7.78e-3)
HPA017991:	unprognostic (1.98e-2)	unprognostic (1.87e-2)	unprognostic (8.83e-2)	unprognostic (1.63e-1)
CAB022159: , HPA000271: , HPA001368:	unprognostic (3.10e-3)	unprognostic (1.27e-1)	unprognostic (4.57e-2)	unprognostic (2.32e-2)
HPA050483:	unprognostic (1.47e-1)	unprognostic (1.21e-1)	unprognostic (3.41e-1)	unprognostic (3.21e-1)
CAB022502: , HPA029363: , HPA055746:				
CAB079022: , HPA029363: , HPA077031:				
HPA035622: , HPA058102:				
HPA054010:				
HPA074584:				
CAB004417: , CAB078783: , HPA013392: , HPA015600:	unprognostic (1.02e-1)		unprognostic (5.11e-2)	
CAB005197: , CAB016392: , CAB079968: , HPA040067: , HPA061280:	unprognostic (2.72e-2)	unprognostic (2.17e-1)	unprognostic (1.38e-1)	unprognostic (2.95e-1)
HPA011048: , HPA011070:		unprognostic (7.72e-2)	unprognostic (8.89e-3)	
HPA029386: , HPA029387: , HPA074882:	unprognostic (1.57e-1)	unprognostic (2.24e-3)	unprognostic (2.76e-1)	unprognostic (2.99e-1)
HPA041311: , HPA048238:	unprognostic (7.22e-2)	unprognostic (9.38e-2)	unprognostic (1.42e-1)	unprognostic (2.50e-2)
HPA020731:	unprognostic (5.68e-2)	unprognostic (1.29e-1)	unprognostic (1.38e-1)	unprognostic (5.09e-2)
HPA047372:				
HPA045339: , HPA057705:	unprognostic (6.47e-2)	unprognostic (1.26e-2)	unprognostic (8.47e-2)	unprognostic (1.11e-2)
HPA049929: , HPA055047:	unprognostic (9.47e-2)	unprognostic (7.07e-2)		unprognostic (2.28e-1)
CAB010753: , HPA035097: , HPA047551: , HPA069245:	unprognostic (7.85e-3)	unprognostic (4.02e-2)	unprognostic (1.95e-2)	unprognostic (1.57e-2)
HPA074472:	unprognostic (1.48e-1)	potential prognostic favorable (7.25e-4)	unprognostic (2.14e-1)	unprognostic (8.27e-2)
HPA055112: , HPA073904:	unprognostic (2.85e-1)		unprognostic (3.43e-2)	unprognostic (1.12e-2)
HPA014846:	unprognostic (8.76e-2)	unprognostic (1.07e-1)	unprognostic (1.24e-1)	unprognostic (5.95e-2)
HPA014005:	unprognostic (1.56e-1)	unprognostic (7.54e-3)	unprognostic (2.57e-1)	unprognostic (8.76e-2)
HPA069619:	unprognostic (1.77e-1)	unprognostic (1.02e-1)	unprognostic (1.12e-1)	unprognostic (3.20e-3)
HPA050873: , HPA059441:	unprognostic (3.45e-1)	unprognostic (4.40e-2)	unprognostic (2.94e-1)	
CAB040541: , CAB040542: , HPA059791:	unprognostic (1.94e-1)	unprognostic (1.15e-2)	unprognostic (1.59e-2)	unprognostic (4.12e-1)
CAB080541:				
CAB022690: , HPA049244:				
CAB079042: , HPA078885:				
CAB070422: , HPA078885:				
HPA078885:		unprognostic (1.57e-2)	unprognostic (2.44e-1)	
HPA007704:	unprognostic (3.03e-2)	unprognostic (1.47e-2)	unprognostic (2.01e-1)	unprognostic (9.59e-2)
CAB010036: , HPA048886:	unprognostic (1.80e-1)	unprognostic (1.48e-1)	unprognostic (5.74e-2)	unprognostic (3.79e-2)
CAB010337: , HPA018122: , HPA027478: , HPA028386:	unprognostic (4.49e-2)	unprognostic (7.56e-2)	unprognostic (5.38e-2)	unprognostic (3.67e-2)
CAB010032: , HPA040736: , HPA074924:	unprognostic (6.88e-2)	unprognostic (2.78e-1)	unprognostic (7.53e-2)	unprognostic (6.48e-2)
HPA047611: , HPA066482:			unprognostic (8.92e-3)	
HPA030571:	unprognostic (5.34e-2)	unprognostic (7.75e-2)	unprognostic (1.94e-2)	unprognostic (8.84e-2)
HPA006316:	unprognostic (1.06e-2)	unprognostic (1.56e-1)	unprognostic (1.61e-1)	unprognostic (2.35e-1)
HPA003648:	unprognostic (3.23e-2)	unprognostic (9.14e-3)	unprognostic (1.08e-1)	unprognostic (8.79e-2)
HPA046655: , HPA060527:				
HPA028642: , HPA028644:				
HPA006970:	potential prognostic favorable (4.25e-5)	unprognostic (5.67e-2)	unprognostic (2.17e-1)	unprognostic (7.78e-2)
HPA041025: , HPA058306:		unprognostic (5.83e-3)	unprognostic (4.60e-2)	unprognostic (3.41e-3)
CAB033690: , HPA075590:	unprognostic (1.37e-1)	unprognostic (1.17e-2)	unprognostic (9.70e-2)	unprognostic (8.63e-3)

HPA071337:			unprognostic (3.00e-3)	
HPA061629: , HPA064454:			unprognostic (3.14e-2)	
HPA049862: , HPA054678:				
HPA056032:		unprognostic (1.37e-1)	unprognostic (4.79e-2)	
HPA007928: , HPA046526:	unprognostic (6.04e-3)	unprognostic (4.75e-3)	unprognostic (2.45e-2)	unprognostic (1.77e-2)
HPA029776:	unprognostic (4.47e-2)	unprognostic (3.00e-2)	unprognostic (1.54e-1)	unprognostic (3.14e-1)
HPA036478: , HPA070892:	unprognostic (3.90e-1)	unprognostic (5.15e-2)	unprognostic (1.25e-1)	unprognostic (2.61e-1)
HPA008852: , HPA012570:	unprognostic (3.07e-1)	unprognostic (2.13e-1)	unprognostic (2.52e-2)	potential prognostic unfavorable (5.08e-4)
HPA008557: , HPA075204:	unprognostic (5.34e-2)	unprognostic (2.07e-1)	unprognostic (3.76e-2)	unprognostic (2.33e-2)
CAB005226: , CAB073538: , HPA057084:	unprognostic (6.98e-2)	unprognostic (6.82e-2)	unprognostic (1.78e-1)	unprognostic (1.82e-1)
HPA014623: , HPA073879:			unprognostic (2.10e-1)	
HPA074001:			unprognostic (9.31e-2)	
HPA052160: , HPA069762:				
CAB025579: , HPA046838:	unprognostic (5.34e-2)	unprognostic (1.74e-2)	unprognostic (2.48e-2)	unprognostic (1.20e-1)
HPA069604:		unprognostic (8.22e-2)	unprognostic (4.73e-1)	
HPA023313: , HPA064826:	unprognostic (1.06e-1)		unprognostic (5.36e-2)	unprognostic (1.54e-1)
HPA029457:	unprognostic (1.25e-2)	unprognostic (2.89e-1)	potential prognostic favorable (7.05e-5)	unprognostic (2.74e-1)
CAB002658: , HPA014811:				
CAB009445: , HPA008965:	unprognostic (8.65e-3)	unprognostic (1.11e-2)	unprognostic (7.03e-2)	unprognostic (9.19e-2)
HPA038671: , HPA045457:	unprognostic (9.03e-2)	unprognostic (2.00e-3)	unprognostic (5.43e-2)	unprognostic (4.73e-2)
HPA067602:	unprognostic (1.11e-1)	unprognostic (6.43e-2)	unprognostic (4.83e-3)	
HPA028660:	unprognostic (1.47e-2)	unprognostic (3.36e-2)		unprognostic (6.83e-2)
CAB005195: , HPA035534: , HPA063768:	unprognostic (2.79e-2)	unprognostic (5.35e-2)	unprognostic (1.17e-2)	unprognostic (1.40e-2)
HPA019143:	unprognostic (1.19e-1)	unprognostic (2.61e-2)	unprognostic (2.89e-2)	unprognostic (7.39e-3)
HPA054516: , HPA070481:				
HPA017276: , HPA053470:	unprognostic (3.17e-2)	unprognostic (6.59e-2)	unprognostic (2.24e-1)	unprognostic (1.36e-2)
CAB078470:	unprognostic (1.22e-2)	unprognostic (9.01e-3)		unprognostic (1.46e-1)
HPA011078: , HPA058509:	unprognostic (1.26e-1)	unprognostic (1.10e-1)		unprognostic (4.25e-2)
CAB024589: , CAB080329: , HPA031454:	unprognostic (8.84e-2)	unprognostic (2.32e-1)		unprognostic (1.55e-2)
CAB032907: , HPA036913: , HPA036914:	unprognostic (3.64e-1)	unprognostic (1.28e-1)	unprognostic (5.13e-2)	unprognostic (9.33e-3)
CAB032656: , CAB079015: , HPA040999:	unprognostic (5.02e-2)	unprognostic (1.89e-1)	unprognostic (5.91e-3)	unprognostic (3.14e-1)
HPA029740:	unprognostic (1.64e-1)	unprognostic (1.81e-2)	unprognostic (1.87e-1)	unprognostic (4.47e-2)
CAB005230: , HPA072606: , HPA074351:	unprognostic (1.00e-2)	unprognostic (5.04e-3)	unprognostic (4.49e-2)	unprognostic (3.54e-2)
CAB004439: , CAB047330: , CAB047331: , CAB047332: , HPA000497:	unprognostic (3.29e-2)	unprognostic (1.15e-1)	unprognostic (2.75e-2)	unprognostic (3.05e-2)
CAB080587: , HPA065367: , HPA073617:	unprognostic (1.43e-1)		unprognostic (3.78e-2)	unprognostic (1.54e-3)
HPA036655:	unprognostic (1.60e-2)	unprognostic (3.01e-1)	unprognostic (2.79e-1)	unprognostic (2.21e-2)
HPA042873:	unprognostic (2.22e-3)	unprognostic (6.70e-2)	unprognostic (1.42e-1)	unprognostic (8.24e-2)
HPA036166: , HPA036167: , HPA067307:	unprognostic (1.29e-2)	unprognostic (3.48e-3)	unprognostic (1.28e-1)	unprognostic (1.87e-1)
CAB026037: , CAB079997: , HPA029179:	unprognostic (2.07e-1)	potential prognostic unfavorable (6.31e-4)	unprognostic (2.83e-3)	unprognostic (7.04e-3)
HPA037957: , HPA062180:	unprognostic (1.29e-1)	unprognostic (3.52e-1)	unprognostic (1.53e-1)	unprognostic (8.48e-2)
CAB017615: , HPA004810:	unprognostic (2.90e-1)	unprognostic (2.71e-3)	unprognostic (7.60e-2)	unprognostic (8.33e-2)
HPA039258:		unprognostic (1.46e-1)	unprognostic (4.53e-2)	
CAB025889: , CAB080493: , CAB080494: , CAB080495: , HPA029213: , HPA063871:	unprognostic (1.31e-1)	unprognostic (2.66e-1)	unprognostic (9.31e-2)	unprognostic (3.35e-2)
CAB010268: , HPA004822: , HPA045563:	unprognostic (1.45e-2)	unprognostic (2.28e-2)	unprognostic (2.15e-1)	potential prognostic unfavorable (7.78e-4)
HPA012369: , HPA070778:	unprognostic (4.61e-2)	potential prognostic favorable (6.28e-4)		unprognostic (8.26e-3)
HPA065647: , HPA069086:	unprognostic (4.85e-2)	unprognostic (3.46e-2)	unprognostic (3.32e-2)	unprognostic (1.34e-2)
CAB026215: , HPA036245:	unprognostic (2.77e-1)	unprognostic (4.63e-2)	unprognostic (2.69e-3)	unprognostic (9.21e-2)
HPA005482:	unprognostic (8.21e-3)	unprognostic (3.24e-2)	unprognostic (3.63e-1)	unprognostic (1.89e-1)
HPA007406: , HPA007917:			unprognostic (7.84e-2)	
CAB080341: , HPA046641:	unprognostic (1.11e-2)	unprognostic (4.75e-3)	unprognostic (4.55e-3)	unprognostic (6.80e-2)
CAB004451: , HPA050124: , HPA070380:	unprognostic (1.22e-2)	unprognostic (2.18e-1)	unprognostic (2.80e-3)	unprognostic (2.44e-3)
CAB025784: , HPA010558:	unprognostic (3.34e-2)	unprognostic (9.54e-3)	unprognostic (6.35e-2)	unprognostic (4.58e-2)
CAB010215: , CAB080603: , HPA067550:	unprognostic (2.81e-1)	unprognostic (5.36e-3)	unprognostic (4.45e-2)	unprognostic (6.91e-2)
CAB013302: , HPA014055:	unprognostic (1.02e-1)	unprognostic (3.37e-1)	potential prognostic favorable (6.28e-5)	unprognostic (7.95e-3)
CAB032306: , HPA030813: , HPA057327:	unprognostic (2.52e-3)	unprognostic (8.08e-3)	unprognostic (2.27e-1)	unprognostic (2.37e-1)
CAB018594: , HPA008572:	unprognostic (7.22e-2)	unprognostic (1.21e-1)	unprognostic (2.78e-1)	unprognostic (1.78e-2)
CAB004458: , CAB072871: , CAB080214: , HPA004723:	unprognostic (1.92e-1)	unprognostic (5.48e-3)	unprognostic (1.24e-2)	unprognostic (3.52e-1)
CAB003434: , HPA059297: , HPA069003:	unprognostic (4.54e-2)	unprognostic (6.31e-2)	unprognostic (2.88e-1)	unprognostic (5.64e-3)
HPA008877: , HPA016894:	unprognostic (4.81e-2)	unprognostic (4.76e-2)	unprognostic (2.34e-2)	unprognostic (5.75e-2)
HPA027852:	unprognostic (3.07e-1)	unprognostic (5.14e-2)	unprognostic (1.97e-1)	potential prognostic unfavorable (1.29e-5)
CAB002422: , CAB005258: , HPA036348: , HPA036349:	unprognostic (3.96e-2)	unprognostic (3.58e-3)	unprognostic (2.19e-2)	unprognostic (1.14e-1)
HPA018007:	unprognostic (1.02e-1)	unprognostic (4.43e-2)	unprognostic (1.13e-3)	unprognostic (2.11e-2)
HPA059627:			unprognostic (3.11e-2)	unprognostic (1.34e-1)
HPA028789: , HPA077346:	unprognostic (1.17e-2)	unprognostic (2.08e-1)	unprognostic (2.05e-1)	unprognostic (1.59e-1)
HPA005854: , HPA047919:	unprognostic (7.91e-3)	unprognostic (2.20e-2)		unprognostic (2.63e-3)
CAB002139: , HPA032047:	unprognostic (1.20e-1)	unprognostic (3.23e-3)	unprognostic (5.99e-2)	unprognostic (1.03e-1)
HPA005539: , HPA056090:	unprognostic (1.62e-1)	unprognostic (1.55e-1)	unprognostic (1.00e-1)	unprognostic (1.19e-1)
HPA038497: , HPA038498:	unprognostic (2.86e-1)	unprognostic (7.62e-3)		unprognostic (6.81e-2)
HPA041345: , HPA041550:	unprognostic (1.23e-3)	unprognostic (5.25e-2)		unprognostic (9.31e-2)
HPA076475:			unprognostic (2.00e-1)	
HPA036110: , HPA044550:	unprognostic (3.86e-2)	unprognostic (4.79e-1)	unprognostic (1.62e-2)	unprognostic (7.58e-2)
HPA014849:	unprognostic (7.59e-2)	unprognostic (1.99e-2)	unprognostic (2.20e-3)	unprognostic (4.99e-1)
HPA063516:				
HPA016586: , HPA077537: , HPA077773:			unprognostic (6.89e-2)	
HPA043892:		unprognostic (1.22e-2)	unprognostic (2.36e-1)	
HPA013367: , HPA053841: , HPA059622:	unprognostic (4.27e-3)	unprognostic (4.76e-3)	unprognostic (5.18e-2)	unprognostic (1.70e-3)
HPA011306: , HPA048553: , HPA071107:	unprognostic (1.69e-3)	unprognostic (3.84e-1)	unprognostic (2.52e-1)	unprognostic (1.35e-1)
HPA043351:	unprognostic (5.30e-2)			unprognostic (5.09e-3)
HPA014864:	potential prognostic favorable (6.40e-4)	unprognostic (1.18e-2)	unprognostic (3.18e-1)	unprognostic (8.01e-2)
HPA059880: , HPA060577:				
HPA067233:				
HPA064442:	unprognostic (9.70e-2)		unprognostic (2.99e-2)	potential prognostic favorable (2.19e-4)
HPA032050: , HPA032051: , HPA064839:	unprognostic (2.85e-2)	unprognostic (1.35e-1)	unprognostic (4.87e-1)	unprognostic (3.46e-2)
HPA007156: , HPA071913:	unprognostic (2.20e-1)	unprognostic (3.24e-1)	unprognostic (4.25e-1)	
CAB003288: , CAB068253: , CAB072867: , HPA004471: , HPA073252:	unprognostic (7.25e-2)	unprognostic (1.24e-2)	unprognostic (1.08e-1)	unprognostic (3.57e-1)
HPA040416: , HPA040434:	unprognostic (1.05e-1)	unprognostic (3.80e-2)	unprognostic (1.81e-1)	unprognostic (6.82e-3)
CAB026342: , HPA018994: , HPA062126:	unprognostic (9.81e-2)	unprognostic (2.24e-2)	unprognostic (6.01e-2)	unprognostic (1.19e-1)
CAB021896: , HPA068877:	unprognostic (4.38e-2)	potential prognostic favorable (2.53e-4)	unprognostic (6.76e-3)	unprognostic (2.28e-3)
CAB010896:	unprognostic (5.60e-2)	unprognostic (3.02e-3)	unprognostic (1.12e-1)	unprognostic (2.95e-2)
HPA011155:	unprognostic (1.88e-2)	unprognostic (4.83e-2)	unprognostic (1.82e-1)	unprognostic (4.84e-3)
CAB005272: , HPA029100:	unprognostic (2.90e-1)	unprognostic (2.21e-2)	unprognostic (1.51e-1)	unprognostic (2.55e-1)
HPA002997:	unprognostic (1.84e-1)	unprognostic (1.58e-1)		unprognostic (4.37e-2)
CAB002223: , CAB012978: , CAB079960: , HPA011157:	unprognostic (3.79e-1)	unprognostic (4.51e-3)	unprognostic (1.38e-2)	potential prognostic favorable (9.44e-4)
HPA003462:	unprognostic (1.81e-1)	unprognostic (9.74e-2)	unprognostic (3.69e-1)	unprognostic (1.10e-3)
CAB025358: , HPA002461: , HPA003887:		unprognostic (3.54e-2)	unprognostic (9.46e-2)	
CAB020673: , HPA000895: , HPA019493:	unprognostic (6.48e-2)	unprognostic (1.11e-2)	unprognostic (5.99e-3)	unprognostic (6.13e-2)
HPA030899:	unprognostic (4.19e-1)	unprognostic (4.96e-2)	unprognostic (1.36e-1)	unprognostic (1.50e-3)
CAB009814: , HPA073668:				
HPA074653:	unprognostic (3.02e-1)	unprognostic (9.16e-2)	unprognostic (7.64e-2)	unprognostic (4.52e-2)
HPA049665:				
HPA006960:	unprognostic (3.18e-2)	unprognostic (1.23e-1)	unprognostic (1.31e-3)	unprognostic (3.25e-1)
HPA002235: , HPA071091:	unprognostic (2.04e-1)	unprognostic (1.89e-2)	unprognostic (1.92e-1)	unprognostic (2.78e-1)
HPA013421:	potential prognostic unfavorable (9.11e-4)	unprognostic (4.44e-2)	unprognostic (6.10e-3)	unprognostic (1.80e-1)
HPA046563: , HPA054951: , HPA055571:			unprognostic (6.05e-2)	
HPA028934: , HPA076273:	unprognostic (1.76e-1)	unprognostic (1.28e-1)	unprognostic (1.31e-2)	unprognostic (4.68e-2)
HPA011133: , HPA017342:	unprognostic (9.67e-3)	unprognostic (2.34e-1)	unprognostic (2.19e-1)	potential prognostic unfavorable (1.26e-4)
HPA076660:			unprognostic (6.02e-3)	
CAB018621: , HPA004182: , HPA022903:	unprognostic (3.21e-1)	unprognostic (2.13e-1)	unprognostic (1.61e-2)	unprognostic (6.96e-2)
HPA056312:		unprognostic (2.41e-1)	unprognostic (3.61e-2)	unprognostic (6.69e-2)
HPA007270: , HPA075309:	unprognostic (1.42e-2)	unprognostic (1.36e-2)	unprognostic (1.42e-1)	unprognostic (2.03e-3)
HPA079451:	unprognostic (1.49e-2)	unprognostic (1.76e-3)	unprognostic (4.62e-3)	unprognostic (3.89e-3)
CAB022689: , HPA029680: , HPA072921:	unprognostic (4.50e-2)		unprognostic (1.18e-2)	unprognostic (1.46e-1)
CAB004492: , HPA001231:	unprognostic (2.94e-1)	unprognostic (2.93e-2)	unprognostic (1.28e-1)	unprognostic (1.78e-1)
HPA013713: , HPA068064: , HPA068103:	unprognostic (2.46e-2)	unprognostic (6.12e-2)	unprognostic (5.98e-2)	unprognostic (2.65e-1)
HPA038568:	unprognostic (2.00e-1)	unprognostic (5.95e-2)	unprognostic (9.57e-2)	unprognostic (2.43e-2)
HPA031851: , HPA031853: , HPA077002:	unprognostic (5.66e-3)	unprognostic (1.84e-1)	unprognostic (9.52e-2)	unprognostic (1.63e-1)
HPA008444:	unprognostic (3.26e-1)	unprognostic (8.92e-2)	unprognostic (9.05e-3)	unprognostic (2.86e-1)
CAB003834: , CAB080093: , HPA026430:	unprognostic (1.16e-2)	unprognostic (4.36e-3)	unprognostic (9.52e-2)	unprognostic (5.73e-3)
HPA007633: , HPA075335:	unprognostic (1.28e-1)	unprognostic (1.26e-2)	unprognostic (1.16e-3)	unprognostic (6.96e-3)
HPA058921: , HPA070332: , HPA079657:	unprognostic (4.55e-2)	unprognostic (1.91e-1)	unprognostic (2.62e-2)	unprognostic (3.36e-2)
CAB000151: , CAB072344: , HPA048895: , HPA069524: , HPA069570:	unprognostic (1.23e-1)	unprognostic (8.49e-3)	unprognostic (1.19e-1)	
CAB022062: , HPA067595: , HPA069443:	unprognostic (6.09e-3)	unprognostic (1.12e-1)	unprognostic (2.67e-1)	potential prognostic unfavorable (3.52e-4)
HPA038790: , HPA074905:	unprognostic (1.82e-1)	unprognostic (8.14e-3)	unprognostic (1.90e-2)	unprognostic (7.82e-3)
HPA018119: , HPA061726:	unprognostic (5.13e-2)	unprognostic (2.25e-1)	unprognostic (1.32e-1)	unprognostic (1.44e-1)
HPA017983:				
HPA042365: , HPA058088:				
CAB002147: , HPA008848:	unprognostic (5.46e-2)	unprognostic (3.20e-2)	unprognostic (3.13e-1)	unprognostic (3.08e-3)
HPA014731:				
CAB010423:				

HPA030115:				
CAB032637: , HPA031763:	unprognostic (8.33e-2)	unprognostic (3.06e-2)	unprognostic (1.47e-1)	unprognostic (2.74e-3)
HPA031235: , HPA050382:	unprognostic (3.03e-2)	unprognostic (4.45e-1)	unprognostic (3.36e-2)	unprognostic (8.17e-3)
HPA066867: , HPA067626:	unprognostic (8.58e-2)		unprognostic (1.78e-1)	
HPA011401:		unprognostic (6.64e-2)	unprognostic (1.87e-1)	
HPA036196: , HPA075622:	unprognostic (3.26e-1)	unprognostic (2.03e-2)	unprognostic (1.92e-1)	potential prognostic favorable (8.83e-4)
HPA017986: , HPA062306: , HPA077490:	unprognostic (6.06e-3)	unprognostic (5.83e-3)	unprognostic (1.83e-1)	unprognostic (8.59e-3)
HPA043107:	unprognostic (5.01e-2)	unprognostic (1.60e-1)	unprognostic (2.81e-1)	unprognostic (1.85e-2)
HPA043480: , HPA051956:	unprognostic (2.88e-1)	unprognostic (7.26e-2)	unprognostic (2.81e-1)	unprognostic (1.77e-1)
HPA056650: , HPA061344:	unprognostic (1.02e-1)	unprognostic (3.01e-1)		unprognostic (4.12e-2)
HPA049511: , HPA062232:	unprognostic (1.62e-2)	unprognostic (5.85e-2)		unprognostic (9.27e-2)
HPA078638:	unprognostic (1.15e-1)	unprognostic (2.76e-1)	potential prognostic favorable (5.44e-4)	unprognostic (5.85e-3)
HPA070324:				
CAB000013: , HPA052583: , HPA056072:	unprognostic (2.30e-1)	unprognostic (2.49e-1)	unprognostic (5.64e-2)	unprognostic (5.09e-3)
CAB025177: , HPA055640:	unprognostic (1.81e-1)	unprognostic (2.81e-2)	unprognostic (3.82e-2)	unprognostic (8.63e-2)
HPA007619:			unprognostic (1.08e-2)	
HPA076675:	unprognostic (7.40e-2)	unprognostic (6.25e-2)	unprognostic (3.47e-1)	unprognostic (9.64e-2)
HPA037597: , HPA037598:	unprognostic (8.65e-2)	unprognostic (1.79e-1)	unprognostic (2.48e-1)	unprognostic (1.45e-1)
HPA026966: , HPA063538:	unprognostic (3.36e-1)	unprognostic (1.73e-1)	unprognostic (7.49e-3)	potential prognostic unfavorable (3.51e-5)
HPA048304:	unprognostic (2.35e-1)	unprognostic (3.46e-1)	unprognostic (1.75e-1)	unprognostic (6.26e-3)
HPA028811:	unprognostic (2.06e-2)	unprognostic (2.68e-2)	unprognostic (6.83e-2)	unprognostic (3.06e-2)
CAB000015: , HPA014341: , HPA014391:	unprognostic (1.71e-1)	validated prognostic favorable (3.10e-4)	validated prognostic favorable (2.75e-5)	potential prognostic favorable (4.40e-5)
CAB002216: , CAB080356: , CAB080357: , HPA017172:	unprognostic (5.68e-3)	unprognostic (1.17e-1)	unprognostic (2.01e-1)	unprognostic (2.65e-1)
CAB010898: , CAB047336: , CAB047337: , CAB047338: , HPA000263: , HPA011135:	unprognostic (2.17e-1)	unprognostic (1.56e-1)	unprognostic (9.72e-2)	unprognostic (3.16e-2)
HPA010008: , HPA010665:	unprognostic (8.14e-3)	unprognostic (1.41e-2)	unprognostic (2.82e-1)	unprognostic (2.12e-2)
CAB000036: , CAB001986: , CAB080102: , CAB080103: , HPA004179: , HPA007235:	unprognostic (6.27e-2)	unprognostic (1.12e-1)	unprognostic (1.67e-1)	unprognostic (1.37e-1)
CAB002302: , HPA014539: , HPA015310:	potential prognostic favorable (8.33e-4)	unprognostic (1.07e-1)	unprognostic (1.38e-1)	unprognostic (4.24e-2)
CAB020789: , HPA031677: , HPA048055:	unprognostic (3.26e-3)	unprognostic (5.13e-2)	unprognostic (1.06e-1)	unprognostic (3.17e-2)
CAB015224: , HPA024223:	potential prognostic unfavorable (2.05e-4)	unprognostic (7.98e-2)	unprognostic (7.87e-2)	unprognostic (1.98e-3)
HPA013607: , HPA060144:	unprognostic (9.53e-2)	unprognostic (1.72e-2)	unprognostic (2.32e-1)	unprognostic (1.54e-3)
CAB037023: , HPA001768: , HPA005924:	unprognostic (6.60e-2)	unprognostic (1.13e-1)	unprognostic (4.56e-1)	unprognostic (3.80e-3)
HPA021252:	unprognostic (3.21e-2)	unprognostic (1.22e-1)	unprognostic (2.80e-2)	unprognostic (2.10e-3)
HPA014245:	unprognostic (1.13e-1)	unprognostic (2.50e-1)	unprognostic (1.25e-1)	unprognostic (1.34e-1)
HPA031889: , HPA031890: , HPA031958:		unprognostic (1.20e-1)	unprognostic (1.37e-1)	
HPA054050: , HPA057065:	unprognostic (3.10e-1)	unprognostic (3.76e-2)	unprognostic (1.00e-1)	
HPA008208: , HPA011389:	unprognostic (2.19e-3)	unprognostic (3.74e-2)	unprognostic (2.01e-1)	unprognostic (3.64e-2)
CAB000142: , CAB018071: , HPA039835:	unprognostic (9.56e-2)	unprognostic (1.04e-1)	unprognostic (1.54e-1)	unprognostic (7.30e-2)
HPA030900: , HPA030901:		unprognostic (5.71e-2)	unprognostic (3.28e-2)	
HPA039490: , HPA040772:	unprognostic (2.50e-2)	unprognostic (5.44e-3)	unprognostic (1.32e-1)	unprognostic (1.06e-2)
CAB018587: , HPA019713:	unprognostic (6.33e-2)	unprognostic (2.04e-3)	unprognostic (3.19e-2)	unprognostic (1.35e-1)
HPA010775:	unprognostic (1.86e-1)	potential prognostic unfavorable (3.01e-4)		unprognostic (4.27e-1)
CAB009320: , HPA027804: , HPA027805: , HPA027806:	unprognostic (1.40e-1)	unprognostic (1.67e-1)	unprognostic (1.26e-1)	unprognostic (2.38e-2)
HPA015634: , HPA021506:			unprognostic (6.33e-2)	
CAB080363: , CAB080365: , HPA045502: , HPA075627:	unprognostic (1.75e-1)	unprognostic (4.11e-2)	unprognostic (2.46e-1)	unprognostic (4.14e-3)
CAB005385: , HPA003097:	unprognostic (4.47e-1)	unprognostic (1.10e-1)	unprognostic (9.08e-2)	unprognostic (1.45e-1)
CAB000143: , CAB001995: , HPA004765:	unprognostic (4.27e-1)	unprognostic (5.70e-3)	unprognostic (1.33e-1)	unprognostic (6.45e-3)
HPA031497: , HPA076257:	unprognostic (1.32e-3)	unprognostic (2.18e-2)	unprognostic (1.53e-1)	unprognostic (2.45e-1)
HPA021284: , HPA021417: , HPA023261: , HPA024312:	unprognostic (1.37e-1)	unprognostic (1.34e-3)		unprognostic (1.91e-2)
HPA052460: , HPA058772:	unprognostic (1.03e-1)	unprognostic (7.81e-2)	unprognostic (7.41e-2)	potential prognostic unfavorable (1.09e-4)
HPA022120: , HPA051576:	potential prognostic favorable (5.56e-5)	unprognostic (7.25e-2)	unprognostic (2.72e-1)	unprognostic (8.63e-2)
HPA003183: , HPA004066:			unprognostic (5.53e-2)	
HPA026665:				
HPA001303:	unprognostic (4.36e-1)	unprognostic (1.36e-1)	unprognostic (1.23e-1)	unprognostic (1.27e-1)
HPA027800: , HPA045836:				
CAB002167: , HPA058312: , HPA069509:				
HPA048743:	unprognostic (4.09e-2)	unprognostic (2.32e-1)	unprognostic (2.27e-1)	unprognostic (2.00e-2)
CAB005393: , HPA044392: , HPA067424:	unprognostic (1.01e-1)	unprognostic (5.13e-2)	unprognostic (1.90e-1)	unprognostic (4.22e-2)
HPA026825:	unprognostic (5.14e-3)		unprognostic (2.47e-1)	
CAB010157: , CAB079912: , HPA049830: , HPA072761:	unprognostic (1.56e-2)	unprognostic (1.83e-1)	unprognostic (2.21e-2)	unprognostic (5.90e-2)
HPA017357:	unprognostic (7.12e-3)	unprognostic (1.13e-1)	unprognostic (9.12e-2)	unprognostic (3.44e-3)
HPA065954:		unprognostic (4.68e-2)	unprognostic (2.19e-2)	
HPA024306: , HPA071290:			unprognostic (2.56e-1)	
CAB010346: , HPA007637: , HPA074873:	unprognostic (1.74e-1)	unprognostic (1.65e-3)	unprognostic (4.55e-2)	unprognostic (9.27e-2)
HPA050299:				
CAB011478: , HPA002874:	unprognostic (1.25e-1)	unprognostic (2.96e-1)	unprognostic (4.34e-2)	unprognostic (2.37e-2)
CAB013075: , CAB068212: , CAB068213: , CAB068214: , HPA005933:	unprognostic (2.16e-1)	unprognostic (3.24e-2)	unprognostic (9.66e-2)	unprognostic (1.65e-1)
CAB034099: , HPA030751: , HPA030752: , HPA030753: , HPA030754:	potential prognostic favorable (4.12e-4)	unprognostic (9.34e-3)	unprognostic (1.25e-1)	unprognostic (9.57e-2)
HPA077718:	unprognostic (2.03e-1)	unprognostic (8.08e-3)	unprognostic (3.62e-3)	unprognostic (8.93e-2)
HPA050798:	unprognostic (1.09e-1)	unprognostic (2.12e-1)	unprognostic (6.10e-2)	unprognostic (2.56e-2)
CAB013682: , HPA049243:	unprognostic (4.16e-3)	unprognostic (9.55e-2)	unprognostic (3.63e-1)	unprognostic (9.63e-3)
HPA067549:				
HPA074114:				
HPA078297:				
HPA078297:				
HPA058578:				
HPA051090:				
HPA032131:	unprognostic (9.47e-2)	unprognostic (1.50e-1)	unprognostic (1.50e-2)	unprognostic (2.29e-1)
HPA036076: , HPA053083: , HPA073967:	unprognostic (8.47e-3)	unprognostic (4.60e-1)	unprognostic (2.75e-1)	unprognostic (2.05e-1)
HPA067673:	unprognostic (1.25e-1)	unprognostic (1.67e-1)	unprognostic (2.81e-1)	unprognostic (7.82e-2)
HPA049854: , HPA056520:	unprognostic (2.67e-3)	unprognostic (4.28e-2)	unprognostic (9.66e-2)	unprognostic (2.74e-2)
HPA001632: , HPA066527:	unprognostic (9.22e-2)	unprognostic (1.59e-1)	unprognostic (1.16e-1)	unprognostic (2.09e-3)
HPA073653: , HPA076187:	unprognostic (1.90e-1)	unprognostic (1.25e-2)	potential prognostic unfavorable (7.11e-4)	unprognostic (3.37e-2)
CAB005312: , HPA003565:	unprognostic (1.37e-1)	unprognostic (6.91e-2)	potential prognostic favorable (7.03e-4)	unprognostic (2.40e-1)
CAB025747: , CAB044670: , HPA070175: , HPA072220:	unprognostic (2.08e-1)	unprognostic (2.86e-2)	unprognostic (1.37e-1)	unprognostic (2.08e-2)
HPA041713: , HPA049578:	unprognostic (6.17e-3)	unprognostic (5.73e-2)	unprognostic (3.21e-1)	unprognostic (1.25e-2)
HPA058905: , HPA074153: , HPA076866:	unprognostic (1.47e-1)	unprognostic (8.54e-2)		potential prognostic unfavorable (1.88e-5)
HPA000993: , HPA063890:	unprognostic (4.20e-3)	unprognostic (3.35e-1)	unprognostic (3.60e-1)	unprognostic (6.94e-3)
HPA016930:	unprognostic (7.40e-3)	potential prognostic unfavorable (5.48e-4)	unprognostic (3.77e-2)	unprognostic (6.18e-3)
HPA004624: , HPA039117:	unprognostic (1.30e-1)	unprognostic (3.66e-2)	unprognostic (2.97e-1)	unprognostic (4.67e-2)
HPA064625:	unprognostic (2.08e-1)	unprognostic (1.62e-2)	unprognostic (4.27e-2)	unprognostic (7.25e-2)
HPA030443:	unprognostic (2.87e-2)	unprognostic (5.92e-3)	unprognostic (2.50e-1)	unprognostic (2.35e-1)
HPA035283:	unprognostic (1.77e-3)	unprognostic (4.85e-2)	unprognostic (1.00e-1)	unprognostic (2.01e-1)
CAB009733: , HPA041551:	unprognostic (1.37e-2)	unprognostic (6.03e-3)	unprognostic (2.14e-2)	unprognostic (1.68e-1)
HPA003568: , HPA072217:	unprognostic (1.18e-1)	unprognostic (1.05e-1)	unprognostic (4.17e-2)	unprognostic (1.42e-3)
HPA066352: , HPA066960: , HPA069079:	unprognostic (2.76e-3)	unprognostic (2.93e-1)		potential prognostic unfavorable (1.80e-4)
HPA047720: , HPA050538:	unprognostic (1.75e-1)	unprognostic (1.98e-2)	unprognostic (1.83e-1)	unprognostic (2.83e-2)
HPA011866: , HPA046521:	unprognostic (1.43e-3)	unprognostic (3.08e-1)	unprognostic (1.43e-1)	unprognostic (6.36e-2)
HPA043180: , HPA073478:		unprognostic (3.07e-3)	unprognostic (3.12e-1)	
HPA008755: , HPA010580: , HPA077526:		unprognostic (1.09e-2)	unprognostic (2.12e-1)	
CAB021103: , HPA050294: , HPA060836:	unprognostic (1.55e-2)	unprognostic (1.20e-1)	unprognostic (6.95e-3)	unprognostic (2.10e-2)
CAB017632: , HPA043310: , HPA076091:	unprognostic (6.10e-3)	unprognostic (1.21e-3)	unprognostic (5.89e-2)	unprognostic (1.37e-1)
HPA045895:	unprognostic (2.73e-2)	unprognostic (1.77e-1)	unprognostic (7.47e-2)	potential prognostic unfavorable (4.94e-4)
HPA011380: , HPA011978:	unprognostic (9.94e-2)	unprognostic (2.11e-2)	unprognostic (2.61e-1)	unprognostic (1.92e-2)
HPA009134:	unprognostic (2.48e-2)	unprognostic (3.18e-1)	unprognostic (5.06e-2)	unprognostic (5.24e-2)
CAB018143: , HPA004947:	unprognostic (1.39e-1)	unprognostic (2.75e-1)	unprognostic (4.29e-1)	unprognostic (3.15e-2)
CAB008376: , HPA007534: , HPA007949:	unprognostic (1.31e-2)	unprognostic (2.88e-1)	unprognostic (5.46e-2)	unprognostic (4.03e-2)
CAB020784: , HPA036503: , HPA036504: , HPA064387: , HPA076072:		unprognostic (1.13e-1)	unprognostic (1.21e-1)	unprognostic (1.28e-2)
HPA004690: , HPA004871:	unprognostic (1.07e-1)	unprognostic (3.74e-1)	unprognostic (1.71e-1)	unprognostic (6.38e-2)
HPA078754:		unprognostic (3.32e-2)	unprognostic (3.63e-1)	unprognostic (1.20e-1)
HPA035146: , HPA035147:	unprognostic (9.99e-2)	unprognostic (1.35e-2)	unprognostic (5.14e-2)	unprognostic (7.34e-3)
HPA039922: , HPA040374:	unprognostic (1.02e-1)	unprognostic (6.78e-3)	unprognostic (1.18e-1)	unprognostic (1.04e-1)
HPA069976:	unprognostic (2.79e-2)	unprognostic (7.74e-2)	unprognostic (8.79e-3)	unprognostic (3.05e-1)
HPA029366:	unprognostic (1.20e-1)	unprognostic (9.12e-3)	unprognostic (1.59e-1)	unprognostic (7.64e-2)
CAB004544: , HPA015794: , HPA055297:	unprognostic (6.97e-2)	unprognostic (1.91e-1)	unprognostic (1.67e-1)	unprognostic (1.43e-1)
CAB037336: , HPA014314: , HPA056908:	unprognostic (9.77e-2)	unprognostic (4.13e-1)	unprognostic (2.45e-1)	unprognostic (1.51e-2)
CAB012993: , HPA062937:	unprognostic (7.27e-3)	unprognostic (6.36e-2)	unprognostic (1.46e-2)	unprognostic (5.92e-2)
CAB037334: , HPA066647: , HPA070648:	unprognostic (4.08e-1)	unprognostic (8.68e-2)	unprognostic (2.15e-1)	unprognostic (5.35e-2)
HPA000825: , HPA001171:	unprognostic (5.65e-2)	unprognostic (5.72e-2)	unprognostic (4.84e-2)	unprognostic (1.64e-3)
CAB005035: , HPA020994: , HPA020996:	unprognostic (9.06e-2)	unprognostic (7.44e-2)	unprognostic (2.36e-1)	potential prognostic unfavorable (4.18e-4)
CAB073533: , HPA050843:	unprognostic (1.01e-1)	unprognostic (8.30e-2)	unprognostic (2.27e-1)	unprognostic (3.73e-3)
HPA007951:	unprognostic (6.71e-2)	unprognostic (6.96e-2)	unprognostic (9.74e-3)	unprognostic (3.02e-1)
CAB004277: , HPA036681: , HPA036682:	unprognostic (1.77e-3)	unprognostic (3.60e-2)	unprognostic (8.47e-2)	unprognostic (7.77e-3)
HPA038610: , HPA066103:	unprognostic (5.02e-3)	unprognostic (2.89e-1)	unprognostic (5.12e-2)	unprognostic (1.54e-2)
HPA041459: , HPA058230:	unprognostic (1.08e-1)	unprognostic (2.03e-2)	unprognostic (3.26e-2)	unprognostic (7.56e-2)
HPA031741: , HPA031742:	unprognostic (1.08e-1)	unprognostic (1.58e-2)	unprognostic (6.13e-2)	unprognostic (2.85e-2)
HPA044682: , HPA054657:	unprognostic (6.31e-2)	unprognostic (4.31e-2)	potential prognostic favorable (3.46e-4)	unprognostic (2.70e-1)
CAB033331: , HPA047815:	unprognostic (5.58e-3)	unprognostic (1.26e-1)		unprognostic (2.62e-1)
HPA055540: , HPA058428:	unprognostic (4.63e-2)	potential prognostic unfavorable (5.64e-4)		unprognostic (4.43e-2)
CAB070421: , HPA068923:	unprognostic (8.19e-2)	unprognostic (1.57e-1)	unprognostic (4.39e-1)	unprognostic (7.66e-2)

CAB016169: , CAB062558: , CAB068219: , CAB068220: , HPA002110: , HPA045507:	unprognostic (6.59e-3)	unprognostic (1.12e-1)	unprognostic (9.25e-3)	unprognostic (1.25e-1)
HPA058210: , HPA067385:	unprognostic (2.75e-2)	unprognostic (9.47e-2)	unprognostic (1.33e-1)	unprognostic (1.97e-3)
HPA001924: , HPA071162:	unprognostic (1.76e-1)	unprognostic (1.63e-1)	unprognostic (3.30e-1)	unprognostic (5.49e-3)
HPA039380:	unprognostic (1.26e-1)	unprognostic (4.40e-2)	potential prognostic favorable (9.51e-4)	unprognostic (1.93e-1)
HPA049571: , HPA079512:	unprognostic (6.37e-2)	unprognostic (2.27e-2)	unprognostic (2.04e-2)	unprognostic (1.44e-2)
CAB005610: , HPA047157:	unprognostic (2.60e-2)	unprognostic (1.17e-2)	unprognostic (2.40e-1)	unprognostic (3.51e-2)
CAB017183: , HPA038467: , HPA038468:	unprognostic (2.17e-1)	unprognostic (1.53e-2)		unprognostic (2.47e-3)
CAB080138: , HPA045153: , HPA068562:	unprognostic (2.76e-3)	unprognostic (5.22e-3)	unprognostic (1.11e-1)	unprognostic (1.39e-1)
CAB003844: , CAB016290: , HPA006563: , HPA006564:	unprognostic (2.13e-2)	unprognostic (5.59e-1)	unprognostic (3.81e-2)	potential prognostic unfavorable (3.97e-4)
CAB001948: , HPA008236: , HPA044496:	unprognostic (4.97e-2)	unprognostic (8.67e-2)	unprognostic (7.77e-2)	unprognostic (8.60e-2)
CAB018367: , HPA029834:	unprognostic (1.35e-2)	unprognostic (9.63e-2)	unprognostic (1.91e-1)	
HPA029529:	unprognostic (1.68e-2)	unprognostic (1.45e-1)	unprognostic (1.50e-2)	potential prognostic unfavorable (7.12e-4)
HPA046636:	unprognostic (1.60e-1)	unprognostic (2.25e-2)	unprognostic (3.21e-2)	unprognostic (6.29e-3)
HPA014447: , HPA019203: , HPA048045:	unprognostic (3.31e-1)	unprognostic (8.65e-2)	unprognostic (3.15e-1)	unprognostic (5.15e-2)
HPA030783: , HPA056418:	unprognostic (5.86e-3)	potential prognostic unfavorable (7.14e-4)	unprognostic (5.41e-3)	unprognostic (9.27e-3)
HPA053927: , HPA062763:	unprognostic (2.14e-1)	unprognostic (1.42e-2)	unprognostic (2.42e-1)	unprognostic (6.76e-2)
HPA017871: , HPA064293:	unprognostic (1.31e-1)	unprognostic (3.43e-2)	unprognostic (4.45e-1)	unprognostic (1.76e-2)
HPA010116: , HPA010600: , HPA072744:	unprognostic (3.19e-2)	unprognostic (2.10e-2)	unprognostic (5.36e-3)	potential prognostic favorable (3.83e-5)
HPA027543:	unprognostic (1.18e-1)	unprognostic (1.95e-2)	unprognostic (3.10e-1)	unprognostic (1.54e-1)
HPA046839:	unprognostic (4.14e-3)	potential prognostic favorable (3.51e-4)	unprognostic (9.51e-2)	
HPA007491: , HPA075879:	unprognostic (2.48e-2)	unprognostic (2.20e-1)	unprognostic (1.06e-1)	unprognostic (4.40e-2)
CAB003850: , HPA026091: , HPA026276:	unprognostic (4.51e-3)	unprognostic (2.35e-1)	potential prognostic favorable (4.22e-4)	unprognostic (2.95e-2)
HPA003222:	unprognostic (2.72e-1)	unprognostic (1.79e-1)	unprognostic (4.56e-1)	potential prognostic unfavorable (2.16e-4)
HPA008708:	unprognostic (5.48e-2)		unprognostic (7.99e-3)	unprognostic (2.30e-1)
HPA006026:	unprognostic (2.18e-1)	unprognostic (1.40e-1)	unprognostic (6.25e-2)	unprognostic (2.64e-1)
HPA054056: , HPA054822:	unprognostic (7.09e-2)	unprognostic (2.33e-1)	unprognostic (2.49e-1)	unprognostic (3.10e-2)
CAB022442: , CAB022443: , HPA003891:	unprognostic (2.99e-3)	unprognostic (1.85e-1)	unprognostic (2.03e-1)	potential prognostic unfavorable (1.42e-4)
CAB011461: , HPA011851: , HPA071067:	unprognostic (7.24e-2)		unprognostic (3.85e-2)	
HPA054747:	unprognostic (9.17e-2)	unprognostic (1.03e-1)	unprognostic (3.81e-3)	unprognostic (2.85e-1)
HPA012568: , HPA064739:	unprognostic (2.97e-2)	unprognostic (2.42e-3)	unprognostic (1.30e-1)	potential prognostic unfavorable (1.01e-4)
HPA024068:	unprognostic (6.49e-2)	unprognostic (1.80e-2)	unprognostic (1.92e-2)	unprognostic (1.54e-1)
HPA003996:	unprognostic (3.62e-3)	unprognostic (2.20e-2)	potential prognostic favorable (7.81e-4)	unprognostic (5.06e-2)
HPA029135: , HPA029136:	unprognostic (1.83e-3)	unprognostic (8.57e-2)	unprognostic (1.84e-2)	unprognostic (2.05e-1)
CAB033645: , HPA010940:	unprognostic (1.60e-1)	unprognostic (6.48e-2)	unprognostic (3.83e-1)	unprognostic (1.74e-1)
HPA041945:	unprognostic (6.59e-2)	unprognostic (1.61e-1)	unprognostic (2.93e-1)	unprognostic (2.29e-1)
HPA062673: , HPA066741:	unprognostic (1.90e-2)		unprognostic (8.20e-2)	unprognostic (1.45e-1)
HPA066741:	unprognostic (3.65e-3)	unprognostic (1.31e-1)	unprognostic (2.04e-1)	unprognostic (1.11e-1)
CAB019291: , CAB080384: , HPA002639: , HPA002640:	unprognostic (9.22e-2)	unprognostic (2.30e-2)	unprognostic (3.02e-1)	unprognostic (3.44e-1)
HPA047037: , HPA065232:	unprognostic (2.38e-1)	unprognostic (2.56e-2)	unprognostic (2.58e-3)	potential prognostic unfavorable (3.66e-4)
CAB046010: , HPA007622: , HPA046651:	unprognostic (3.71e-2)	unprognostic (2.23e-1)	unprognostic (3.38e-1)	unprognostic (1.52e-3)
HPA075527:	unprognostic (1.46e-2)	unprognostic (1.16e-2)	unprognostic (1.06e-1)	unprognostic (9.01e-3)
HPA068798:	unprognostic (3.80e-2)	unprognostic (2.27e-1)	unprognostic (7.51e-2)	unprognostic (1.20e-2)
HPA016744: , HPA020027:	unprognostic (5.48e-2)	unprognostic (7.51e-2)	unprognostic (2.67e-1)	unprognostic (4.36e-2)
HPA043417:	unprognostic (9.30e-3)	unprognostic (2.01e-2)	unprognostic (1.01e-3)	unprognostic (2.68e-3)
HPA039389:	unprognostic (2.48e-1)	unprognostic (7.85e-3)	unprognostic (2.81e-1)	unprognostic (2.13e-2)
HPA015635: , HPA015667:	unprognostic (5.99e-2)	unprognostic (9.43e-2)	unprognostic (2.31e-2)	unprognostic (1.57e-2)
HPA041929:			unprognostic (2.79e-2)	
HPA027428: , HPA027434: , HPA027448:	unprognostic (1.08e-1)	unprognostic (4.47e-2)	unprognostic (4.03e-2)	unprognostic (2.08e-2)
HPA039683: , HPA040776:	unprognostic (1.02e-3)	unprognostic (8.91e-2)	unprognostic (1.42e-1)	unprognostic (1.21e-1)
CAB037312: , HPA000263: , HPA000763:	unprognostic (1.97e-1)	unprognostic (7.14e-3)	unprognostic (2.22e-1)	unprognostic (1.64e-2)
CAB025109: , HPA071729:	unprognostic (8.95e-2)	unprognostic (5.11e-1)	unprognostic (1.78e-1)	unprognostic (1.56e-2)
HPA013377:	unprognostic (2.17e-1)	unprognostic (1.41e-1)	unprognostic (1.42e-2)	unprognostic (3.21e-2)
HPA029961: , HPA029962: , HPA029963:	unprognostic (1.91e-1)	potential prognostic unfavorable (2.84e-5)	unprognostic (2.17e-1)	unprognostic (1.25e-1)
CAB002581: , HPA008356: , HPA008495:		unprognostic (7.24e-2)	unprognostic (1.86e-1)	
HPA056384: , HPA069984:	unprognostic (1.12e-1)	unprognostic (2.07e-1)	unprognostic (2.47e-1)	unprognostic (5.96e-2)
HPA044234:	unprognostic (1.39e-1)	potential prognostic unfavorable (2.68e-4)	unprognostic (3.29e-1)	unprognostic (1.50e-3)
HPA037859:	unprognostic (3.71e-2)	unprognostic (9.18e-2)		unprognostic (2.39e-1)
CAB005052:	unprognostic (3.25e-2)	unprognostic (9.84e-2)	unprognostic (3.95e-1)	unprognostic (8.00e-2)
CAB012643: , HPA047193: , HPA073217:	unprognostic (6.06e-2)	unprognostic (3.45e-1)	unprognostic (1.40e-1)	unprognostic (2.52e-1)
HPA077800:	unprognostic (5.59e-2)	unprognostic (3.18e-1)	unprognostic (3.00e-1)	potential prognostic unfavorable (2.89e-4)
HPA027158: , HPA027209:	unprognostic (1.10e-1)	unprognostic (4.18e-3)	unprognostic (3.44e-2)	unprognostic (7.48e-3)
CAB013524: , HPA052968: , HPA074461:	unprognostic (2.83e-1)	unprognostic (4.14e-1)	unprognostic (4.24e-2)	unprognostic (1.22e-2)
HPA000234: , HPA000909:	unprognostic (1.64e-1)	unprognostic (6.29e-2)	unprognostic (1.36e-1)	unprognostic (1.77e-2)
CAB009561:	unprognostic (6.18e-3)	unprognostic (1.47e-2)	unprognostic (1.56e-1)	potential prognostic favorable (3.57e-4)
HPA049945: , HPA074403:	unprognostic (2.85e-1)	unprognostic (4.86e-2)	unprognostic (5.33e-2)	unprognostic (2.65e-3)
HPA027813: , HPA028676:		unprognostic (4.48e-1)	unprognostic (4.54e-2)	
CAB012443:	unprognostic (1.40e-1)	unprognostic (2.35e-1)	unprognostic (3.69e-1)	unprognostic (2.58e-3)
HPA044428:	unprognostic (8.26e-2)	unprognostic (1.24e-1)	unprognostic (3.56e-1)	unprognostic (6.15e-3)
HPA042674: , HPA056140:	unprognostic (2.33e-1)	unprognostic (1.15e-1)	unprognostic (1.33e-1)	unprognostic (3.19e-2)
CAB002601: , CAB040549: , CAB047354: , HPA007575:	unprognostic (2.02e-1)	unprognostic (2.54e-2)	unprognostic (1.00e-1)	unprognostic (1.97e-1)
CAB009441: , CAB080262: , HPA004193:	unprognostic (8.79e-2)	unprognostic (1.28e-1)	unprognostic (1.23e-1)	unprognostic (1.50e-2)
HPA039737: , HPA040154:	unprognostic (1.53e-1)			unprognostic (9.94e-2)
HPA078664:				
HPA078632:			unprognostic (7.64e-3)	
HPA063346:	unprognostic (9.16e-2)			
CAB022169: , HPA043937: , HPA073590:			unprognostic (1.37e-1)	
HPA026884:	unprognostic (1.54e-3)	unprognostic (4.77e-2)	unprognostic (1.33e-1)	unprognostic (1.32e-1)
HPA071194:	unprognostic (6.04e-3)	unprognostic (1.00e-1)	unprognostic (2.17e-3)	unprognostic (4.60e-3)
CAB015463: , CAB022081: , HPA023557: , HPA064312:	unprognostic (8.67e-2)	unprognostic (1.08e-1)	unprognostic (2.13e-1)	potential prognostic unfavorable (2.73e-5)
HPA040606:	unprognostic (4.59e-2)	unprognostic (6.40e-2)	unprognostic (2.09e-1)	unprognostic (1.27e-1)
CAB025123: , HPA068532: , HPA074427:	unprognostic (6.47e-3)	unprognostic (2.22e-1)	unprognostic (2.37e-3)	unprognostic (1.43e-2)
HPA028467: , HPA031255: , HPA031256:	unprognostic (4.85e-1)	unprognostic (7.20e-3)		unprognostic (1.26e-1)
HPA015662: , HPA023277:	unprognostic (3.04e-2)	unprognostic (1.12e-1)	unprognostic (5.62e-2)	unprognostic (2.60e-1)
HPA037898: , HPA037899:	unprognostic (6.38e-2)	unprognostic (1.76e-1)	unprognostic (3.75e-1)	unprognostic (6.52e-2)
HPA032129: , HPA032130:			unprognostic (2.19e-1)	
CAB025807: , HPA027093:	unprognostic (2.88e-2)	unprognostic (6.70e-2)	unprognostic (1.70e-1)	unprognostic (1.12e-2)
HPA014377: , HPA014428:	unprognostic (1.77e-3)	unprognostic (9.34e-2)	unprognostic (1.13e-1)	unprognostic (2.51e-1)
HPA002875: , HPA077689:	unprognostic (7.77e-2)	unprognostic (1.00e-1)	unprognostic (1.37e-1)	unprognostic (2.85e-2)
HPA042072: , HPA045480: , HPA074988:	unprognostic (1.50e-2)	unprognostic (8.56e-2)	unprognostic (3.13e-1)	unprognostic (3.05e-2)
HPA014333: , HPA014353: , HPA014736:	unprognostic (3.25e-1)	unprognostic (2.33e-3)	unprognostic (2.38e-1)	unprognostic (6.64e-2)
HPA044343:			unprognostic (6.76e-2)	unprognostic (2.26e-1)
HPA059570: , HPA077233:	unprognostic (2.48e-2)	unprognostic (8.13e-2)	unprognostic (1.85e-1)	unprognostic (6.19e-2)
CAB017489: , CAB079035: , HPA003324: , HPA071055:	unprognostic (1.30e-2)	unprognostic (1.01e-1)	unprognostic (7.38e-2)	unprognostic (5.28e-3)
HPA016860:	unprognostic (5.80e-2)	unprognostic (1.40e-1)	unprognostic (2.87e-1)	unprognostic (3.16e-1)
CAB017490: , HPA021451:	unprognostic (1.07e-2)	unprognostic (1.95e-1)	unprognostic (2.01e-1)	potential prognostic unfavorable (4.92e-4)
HPA005911:	unprognostic (3.18e-2)	unprognostic (5.87e-2)	unprognostic (1.89e-1)	unprognostic (2.35e-2)
HPA044479:	unprognostic (1.55e-3)	unprognostic (4.27e-3)	unprognostic (5.80e-2)	unprognostic (9.50e-3)
HPA035239: , HPA035240:	unprognostic (1.16e-1)	unprognostic (1.19e-3)	unprognostic (1.32e-1)	unprognostic (1.23e-1)
HPA041505: , HPA044066:	unprognostic (1.05e-3)	unprognostic (7.21e-2)	unprognostic (4.29e-1)	unprognostic (3.63e-1)
CAB019279: , HPA035834: , HPA064386:	unprognostic (8.26e-2)	unprognostic (2.88e-1)	unprognostic (3.72e-1)	unprognostic (2.82e-2)
HPA079359:		unprognostic (7.47e-2)	unprognostic (4.82e-2)	
HPA048363: , HPA072381:	unprognostic (3.94e-2)	unprognostic (8.05e-2)	unprognostic (9.99e-2)	unprognostic (1.31e-2)
HPA019551: , HPA056247:				
CAB002759: , HPA031345: , HPA058494:	unprognostic (1.47e-1)	unprognostic (1.82e-1)	unprognostic (6.77e-3)	unprognostic (2.23e-3)
HPA031593: , HPA031594:	unprognostic (2.81e-3)	unprognostic (3.97e-1)	unprognostic (4.97e-2)	unprognostic (8.07e-3)
HPA006539:	unprognostic (5.04e-3)	unprognostic (3.35e-2)	unprognostic (5.84e-2)	unprognostic (1.90e-1)
CAB010444: , HPA028997: , HPA069490:				
CAB002763: , HPA006539:	unprognostic (3.56e-2)	unprognostic (1.71e-1)	unprognostic (7.30e-2)	unprognostic (7.41e-2)
HPA005449: , HPA075145:	unprognostic (1.66e-1)	unprognostic (2.67e-1)	unprognostic (2.25e-1)	unprognostic (1.30e-1)
HPA015275: , HPA049000:	unprognostic (4.74e-2)	unprognostic (2.27e-1)	unprognostic (3.86e-2)	potential prognostic unfavorable (1.48e-4)
HPA065953: , HPA076165:		unprognostic (2.74e-2)	unprognostic (1.64e-2)	
HPA051255: , HPA077175:				
HPA037989: , HPA066474:	unprognostic (1.01e-2)	unprognostic (5.82e-2)	unprognostic (1.21e-1)	unprognostic (1.64e-3)
HPA052054: , HPA064728:	unprognostic (1.25e-1)	unprognostic (6.33e-3)		unprognostic (1.31e-2)
HPA035180: , HPA069672:	unprognostic (3.25e-1)	unprognostic (2.34e-3)	unprognostic (1.36e-2)	unprognostic (2.68e-3)
HPA042377:	unprognostic (1.40e-2)	unprognostic (4.00e-1)	unprognostic (7.10e-2)	unprognostic (4.57e-3)
CAB010455: , HPA017980:	unprognostic (2.19e-2)	unprognostic (5.47e-2)	unprognostic (1.54e-1)	unprognostic (1.51e-1)
HPA065634:	unprognostic (9.99e-2)	unprognostic (2.41e-2)	unprognostic (2.35e-1)	unprognostic (1.10e-1)
HPA045847:	unprognostic (1.97e-3)	unprognostic (2.50e-2)	unprognostic (2.59e-1)	unprognostic (1.34e-1)
CAB011614:	unprognostic (3.47e-1)	unprognostic (1.64e-1)	unprognostic (4.41e-2)	unprognostic (3.51e-2)
HPA019339:	unprognostic (8.69e-3)	unprognostic (3.41e-1)	unprognostic (4.51e-1)	unprognostic (4.66e-3)
CAB022493: , HPA035628: , HPA035629: , HPA079220:	unprognostic (2.46e-1)	unprognostic (2.84e-2)	unprognostic (3.41e-2)	unprognostic (1.01e-1)
CAB022494: , HPA035857:	unprognostic (2.76e-2)	unprognostic (7.34e-2)	unprognostic (9.22e-2)	unprognostic (1.21e-1)
CAB015467: , HPA051805: , HPA055106:		unprognostic (5.41e-2)	unprognostic (1.39e-1)	unprognostic (1.57e-1)
HPA060904:				
HPA029791:	unprognostic (6.74e-2)	unprognostic (1.22e-1)	unprognostic (2.00e-2)	unprognostic (3.21e-2)
HPA009277:	unprognostic (2.64e-2)	unprognostic (1.34e-1)	unprognostic (1.10e-2)	unprognostic (1.59e-1)
HPA052673: , HPA056077:	unprognostic (1.29e-1)	unprognostic (1.70e-3)	unprognostic (2.80e-1)	unprognostic (2.60e-2)
HPA050713:	unprognostic (7.85e-2)	unprognostic (5.67e-3)	unprognostic (5.86e-3)	unprognostic (1.30e-1)

CAB022694: , HPA070007:	unprognostic (2.25e-1)	unprognostic (3.47e-1)	unprognostic (1.84e-1)	
CAB022371: , HPA048532: , HPA052891:	unprognostic (2.30e-1)	unprognostic (2.95e-3)	unprognostic (9.51e-3)	unprognostic (1.44e-2)
HPA035121: , HPA035122:	unprognostic (6.17e-2)	unprognostic (1.45e-2)	unprognostic (9.22e-2)	unprognostic (5.34e-3)
HPA036493: , HPA036669:	unprognostic (2.49e-2)	unprognostic (2.24e-2)		unprognostic (8.23e-2)
HPA001672: , HPA066520:	unprognostic (3.40e-2)	unprognostic (4.46e-2)	unprognostic (1.83e-1)	unprognostic (1.08e-1)
HPA059445: , HPA059590:	unprognostic (5.88e-3)	unprognostic (1.95e-1)	unprognostic (3.69e-1)	unprognostic (1.87e-1)
HPA059122: , HPA079529:				
HPA071152:	unprognostic (3.27e-1)			unprognostic (3.32e-3)
HPA050892: , HPA073093:				
HPA004943: , HPA050892:	unprognostic (7.86e-2)			unprognostic (9.05e-2)
HPA030669: , HPA030670:	unprognostic (1.73e-1)	unprognostic (1.10e-1)	unprognostic (6.50e-2)	unprognostic (2.23e-1)
HPA025062:			unprognostic (2.16e-2)	
HPA051630:	unprognostic (5.43e-2)	unprognostic (7.01e-2)	unprognostic (1.53e-1)	unprognostic (1.18e-1)
HPA014491: , HPA014513:	potential prognostic favorable (9.27e-4)	unprognostic (5.92e-2)	unprognostic (1.91e-1)	unprognostic (1.09e-2)
HPA039711:	unprognostic (1.38e-1)	unprognostic (5.49e-3)	unprognostic (2.98e-2)	unprognostic (1.15e-1)
CAB037083: , CAB037317: , HPA001214:	unprognostic (2.23e-2)	unprognostic (9.35e-2)	unprognostic (2.00e-1)	unprognostic (2.91e-2)
HPA001394:	unprognostic (2.87e-2)	unprognostic (4.05e-1)	unprognostic (1.22e-1)	unprognostic (1.48e-1)
HPA065258: , HPA077904:			unprognostic (7.77e-2)	
HPA031410: , HPA057203:	unprognostic (4.25e-3)	potential prognostic unfavorable (8.10e-4)	unprognostic (5.58e-3)	unprognostic (2.42e-3)
CAB032850: , HPA027559: , HPA043084:	unprognostic (7.27e-3)	unprognostic (4.37e-3)	unprognostic (1.04e-1)	unprognostic (4.30e-1)
CAB011498: , HPA006889:	unprognostic (1.92e-1)	unprognostic (5.29e-2)	unprognostic (1.03e-2)	unprognostic (3.61e-1)
HPA040412: , HPA040947:	unprognostic (2.82e-2)	unprognostic (2.10e-1)	unprognostic (9.49e-3)	unprognostic (1.00e-1)
HPA022829: , HPA028761:	unprognostic (8.79e-2)	unprognostic (3.30e-2)	unprognostic (2.90e-1)	unprognostic (1.43e-1)
HPA006903: , HPA031178:	unprognostic (6.42e-2)	unprognostic (2.17e-3)	unprognostic (1.32e-1)	potential prognostic unfavorable (6.56e-4)
HPA040737: , HPA040942:	unprognostic (1.06e-2)	unprognostic (1.80e-1)	unprognostic (5.70e-2)	unprognostic (1.13e-1)
CAB005592: , HPA039292:	unprognostic (9.64e-2)	unprognostic (3.72e-2)	unprognostic (8.78e-2)	unprognostic (4.50e-1)
HPA004194: , HPA059958:	unprognostic (1.19e-1)	unprognostic (5.99e-3)	unprognostic (1.18e-1)	unprognostic (2.77e-1)
CAB004023: , HPA030875:	unprognostic (1.20e-1)	unprognostic (3.66e-2)	unprognostic (6.36e-2)	unprognostic (1.40e-3)
HPA066503:				unprognostic (2.15e-2)
HPA035800: , HPA035801:	unprognostic (1.01e-2)	unprognostic (3.54e-2)	unprognostic (5.43e-2)	unprognostic (1.77e-2)
HPA065201:	unprognostic (1.02e-1)	unprognostic (9.18e-2)	unprognostic (1.94e-1)	unprognostic (2.28e-3)
CAB020840: , HPA015083: , HPA015500:	unprognostic (1.55e-1)	unprognostic (7.85e-2)	unprognostic (3.21e-2)	unprognostic (2.70e-2)
HPA010961: , HPA011419:	unprognostic (9.61e-2)	unprognostic (1.44e-1)	unprognostic (8.98e-2)	unprognostic (4.29e-2)
CAB015944: , HPA062016:	unprognostic (2.28e-2)	unprognostic (1.78e-1)	potential prognostic unfavorable (4.46e-4)	unprognostic (1.82e-1)
HPA012892: , HPA071011:				
HPA003019:	unprognostic (2.27e-2)	unprognostic (1.80e-1)	unprognostic (7.64e-2)	unprognostic (9.54e-2)
CAB010878: , CAB080402: , HPA020095: , HPA020138:	unprognostic (2.26e-1)	unprognostic (1.68e-1)	unprognostic (1.22e-1)	unprognostic (2.17e-1)
HPA006810: , HPA006929:	unprognostic (9.08e-2)	unprognostic (1.12e-1)	unprognostic (1.28e-1)	unprognostic (3.09e-3)
HPA041756: , HPA042449:	unprognostic (1.19e-2)	unprognostic (1.16e-1)	unprognostic (1.58e-1)	unprognostic (1.83e-2)
HPA039651:	unprognostic (2.45e-2)	potential prognostic unfavorable (2.77e-4)	unprognostic (8.75e-2)	unprognostic (1.99e-1)
CAB037030: , HPA005480: , HPA024300:	unprognostic (1.76e-1)	unprognostic (2.31e-2)	unprognostic (2.15e-1)	unprognostic (3.66e-3)
HPA055565: , HPA074573:		potential prognostic favorable (7.18e-4)	unprognostic (4.16e-2)	
HPA076573:	unprognostic (1.38e-1)	unprognostic (5.33e-2)	unprognostic (1.60e-1)	potential prognostic favorable (3.82e-4)
CAB072852: , HPA043104: , HPA055067:	unprognostic (1.45e-1)	unprognostic (5.10e-2)	unprognostic (7.09e-2)	unprognostic (2.30e-1)
HPA053439:	unprognostic (5.59e-3)	unprognostic (6.29e-2)	unprognostic (1.29e-1)	
HPA007725: , HPA008142: , HPA076041:	unprognostic (7.40e-2)	unprognostic (4.36e-1)	unprognostic (2.55e-2)	unprognostic (1.51e-1)
HPA044712: , HPA068080:	unprognostic (6.07e-2)	unprognostic (1.89e-2)	unprognostic (1.56e-1)	unprognostic (9.20e-2)
HPA039772: , HPA040043:	unprognostic (1.75e-1)	unprognostic (1.80e-2)		unprognostic (3.01e-1)
HPA077366:	unprognostic (6.48e-2)	unprognostic (9.85e-2)	unprognostic (7.09e-2)	unprognostic (2.63e-1)
HPA038756: , HPA038758:	unprognostic (2.15e-2)	unprognostic (1.29e-1)		unprognostic (1.73e-1)
HPA038756: , HPA038758:				
HPA005733: , HPA024657:			unprognostic (2.81e-2)	
CAB010359: , HPA073265:	unprognostic (2.08e-1)	unprognostic (3.02e-1)	unprognostic (7.64e-3)	unprognostic (4.21e-2)
CAB002441: , CAB031481: , HPA056473:	unprognostic (4.08e-1)	unprognostic (1.58e-2)	unprognostic (1.85e-3)	unprognostic (2.01e-3)
CAB073537: , HPA074821:	unprognostic (1.13e-1)	unprognostic (4.37e-1)	unprognostic (4.60e-2)	unprognostic (4.96e-3)
CAB010416: , HPA034853:	potential prognostic favorable (4.77e-5)	unprognostic (1.52e-1)	unprognostic (6.85e-2)	unprognostic (1.92e-2)
HPA041357: , HPA041474:	unprognostic (1.52e-1)	unprognostic (4.12e-3)	unprognostic (3.88e-1)	unprognostic (3.04e-2)
CAB010822: , HPA001636: , HPA001637:	unprognostic (1.59e-1)	unprognostic (2.89e-2)	unprognostic (2.99e-1)	unprognostic (2.80e-2)
CAB009228: , HPA001813: , HPA070714:	unprognostic (1.11e-1)	unprognostic (1.16e-1)	unprognostic (2.27e-1)	unprognostic (1.54e-1)
CAB013244: , HPA046863: , HPA053337:	unprognostic (5.50e-3)	unprognostic (6.04e-2)	unprognostic (6.58e-2)	unprognostic (8.16e-2)
CAB002006: , HPA004748:	unprognostic (1.06e-1)	unprognostic (2.23e-1)	unprognostic (2.96e-1)	potential prognostic unfavorable (7.71e-4)
HPA078321:	unprognostic (1.11e-1)	unprognostic (1.35e-1)	unprognostic (1.12e-1)	unprognostic (7.84e-2)
CAB004025: , HPA049174:	unprognostic (1.71e-1)	unprognostic (6.55e-2)	unprognostic (1.07e-1)	unprognostic (7.44e-2)
HPA051838: , HPA063184: , HPA065531:				
HPA056530: , HPA064017:				
HPA041921: , HPA049425:				
HPA029249: , HPA029250: , HPA029251:	unprognostic (1.85e-1)	unprognostic (6.08e-3)	unprognostic (8.99e-2)	unprognostic (1.65e-1)
HPA007108: , HPA025020:	unprognostic (2.94e-2)	unprognostic (2.17e-3)	unprognostic (2.55e-2)	unprognostic (7.42e-3)
HPA034493: , HPA035787:	unprognostic (3.93e-3)	unprognostic (2.10e-3)	unprognostic (7.93e-2)	unprognostic (1.09e-1)
CAB010391: , HPA047976:	unprognostic (2.06e-2)	unprognostic (1.98e-1)	unprognostic (1.07e-1)	unprognostic (2.17e-1)
CAB026150: , CAB030007: , CAB080525: , CAB080526: , HPA006404:	unprognostic (1.19e-1)	unprognostic (4.43e-2)	unprognostic (1.44e-2)	unprognostic (2.03e-1)
CAB009805: , HPA006746:	unprognostic (1.06e-1)	unprognostic (7.93e-3)	unprognostic (3.92e-1)	unprognostic (5.90e-2)
CAB000016: , HPA014823: , HPA032081: , HPA032082:			potential prognostic favorable (9.62e-4)	
CAB009188: , HPA030526:	unprognostic (5.03e-3)	unprognostic (1.57e-1)	unprognostic (1.19e-1)	unprognostic (1.57e-1)
HPA041954: , HPA069561:	unprognostic (1.69e-1)	unprognostic (4.78e-2)	unprognostic (1.98e-1)	unprognostic (2.05e-1)
HPA021603: , HPA050429:	unprognostic (2.06e-2)	unprognostic (1.45e-1)	unprognostic (8.44e-3)	unprognostic (6.64e-2)
HPA045817: , HPA074725:		unprognostic (1.79e-2)		
HPA036261: , HPA036262:	unprognostic (8.61e-3)	unprognostic (7.57e-2)	unprognostic (7.05e-2)	unprognostic (6.68e-2)
CAB026130: , HPA002356: , HPA072760:		unprognostic (1.02e-3)	unprognostic (6.87e-2)	potential prognostic favorable (5.68e-4)
HPA038226: , HPA058226:				
HPA016700: , HPA017860:			unprognostic (4.47e-2)	
HPA041169:	unprognostic (4.49e-2)	unprognostic (7.14e-2)	unprognostic (1.37e-1)	unprognostic (3.19e-1)
HPA044993:	unprognostic (5.80e-2)	unprognostic (3.86e-2)	unprognostic (1.28e-1)	unprognostic (3.04e-2)
HPA044657: , HPA071160:	unprognostic (1.90e-1)	unprognostic (2.36e-1)	unprognostic (1.64e-1)	unprognostic (1.97e-2)
HPA060691: , HPA066996:				
HPA038508:	unprognostic (3.26e-1)	unprognostic (8.01e-2)	unprognostic (2.25e-1)	unprognostic (8.55e-3)
HPA015285: , HPA018496:	unprognostic (1.58e-2)	unprognostic (1.34e-1)	unprognostic (1.27e-1)	unprognostic (5.39e-2)
HPA078636:	unprognostic (2.83e-1)		unprognostic (6.19e-2)	
HPA041174: , HPA041390:	unprognostic (6.23e-1)	unprognostic (9.28e-2)	unprognostic (1.75e-1)	unprognostic (1.64e-2)
CAB009493: , HPA041899:	unprognostic (8.04e-2)	unprognostic (3.48e-2)	unprognostic (2.39e-1)	unprognostic (5.28e-2)
CAB011464: , CAB035990: , CAB080407: , HPA034569: , HPA054975:	unprognostic (3.63e-2)	unprognostic (1.90e-2)	unprognostic (1.01e-1)	unprognostic (1.32e-1)
HPA003920:	unprognostic (6.66e-2)	unprognostic (1.78e-2)	unprognostic (4.84e-1)	unprognostic (9.42e-2)
HPA007547: , HPA071005:			unprognostic (8.66e-2)	unprognostic (3.00e-1)
CAB018974: , HPA066741:	unprognostic (4.46e-2)	potential prognostic unfavorable (1.75e-5)	unprognostic (4.82e-3)	unprognostic (5.29e-2)
HPA001308:	unprognostic (3.79e-3)	unprognostic (3.80e-1)	unprognostic (1.44e-2)	unprognostic (3.95e-2)
HPA076515:	unprognostic (6.97e-3)			
CAB016348: , HPA018894:	unprognostic (3.61e-2)	unprognostic (1.22e-1)	unprognostic (6.66e-2)	unprognostic (6.57e-2)
CAB004612: , HPA005724:	unprognostic (1.14e-1)	unprognostic (2.94e-1)	unprognostic (1.74e-1)	unprognostic (4.79e-2)
CAB002452: , HPA006884: , HPA006885:	unprognostic (1.41e-1)			potential prognostic favorable (2.94e-4)
HPA039734: , HPA043947:	unprognostic (1.58e-1)	unprognostic (1.96e-1)	unprognostic (1.15e-2)	unprognostic (5.53e-2)
CAB020804: , HPA036434: , HPA070293:	unprognostic (1.56e-2)	unprognostic (3.07e-1)	unprognostic (1.60e-1)	unprognostic (4.43e-2)
CAB009932: , HPA035707: , HPA069673:	unprognostic (1.66e-2)	unprognostic (5.63e-2)	unprognostic (5.04e-2)	unprognostic (5.07e-1)
HPA035005: , HPA035006:	unprognostic (1.86e-2)	unprognostic (2.56e-1)	unprognostic (8.61e-2)	unprognostic (1.44e-1)
CAB004370: , HPA026480:	unprognostic (4.13e-1)	unprognostic (1.85e-1)	unprognostic (5.76e-2)	unprognostic (4.79e-3)
HPA028400: , HPA028439:	unprognostic (3.33e-1)	unprognostic (2.97e-2)	unprognostic (1.19e-1)	unprognostic (1.30e-1)
CAB001980: , HPA000755: , HPA005732:	unprognostic (2.44e-1)	unprognostic (8.55e-3)	unprognostic (1.19e-1)	unprognostic (1.82e-1)
HPA023331: , HPA023334:	potential prognostic favorable (2.66e-4)	unprognostic (8.84e-2)	unprognostic (1.33e-1)	unprognostic (4.53e-1)
HPA014483: , HPA014702:	unprognostic (5.38e-2)	unprognostic (3.18e-1)	unprognostic (2.62e-1)	potential prognostic unfavorable (2.99e-4)
HPA049471: , HPA072213:			unprognostic (3.07e-1)	
HPA014670:	unprognostic (3.98e-1)	unprognostic (3.93e-2)	unprognostic (1.29e-1)	unprognostic (3.02e-3)
HPA000400: , HPA016438:	unprognostic (6.23e-2)	unprognostic (1.45e-1)	unprognostic (9.35e-2)	unprognostic (6.76e-2)
HPA029534: , HPA050750:	unprognostic (1.03e-3)	unprognostic (3.53e-2)	unprognostic (5.91e-2)	unprognostic (1.69e-3)

Cancer prognostics - Colon Adenocarcinoma (TCGA)	Cancer prognostics - Colon Adenocarcinoma (validation)	Cancer prognostics - Glioblastoma Multiforme (TCGA)	Cancer prognostics - Glioblastoma Multiforme (validation)	Cancer prognostics - Head and Neck Squamous Cell Carcinoma (TCGA)	Cancer prognostics - Kidney Chromophobe (TCGA)
unprognostic (2.02e-1)	unprognostic (2.48e-3)	unprognostic (1.07e-1)	unprognostic (1.15e-1)	unprognostic (4.66e-2)	potential prognostic unfavorable (6.13e-4)
unprognostic (4.95e-2)	unprognostic (1.07e-1)	unprognostic (3.88e-3)	unprognostic (6.15e-2)	unprognostic (3.67e-2)	unprognostic (1.25e-3)
unprognostic (2.28e-1)	unprognostic (8.23e-2)	unprognostic (7.72e-2)	unprognostic (8.66e-2)	unprognostic (2.61e-1)	unprognostic (1.87e-2)
unprognostic (5.72e-3)	unprognostic (2.86e-1)	unprognostic (3.89e-2)	unprognostic (1.79e-1)	unprognostic (6.69e-2)	unprognostic (2.19e-1)
unprognostic (8.89e-2)	unprognostic (7.32e-3)	unprognostic (8.80e-3)	unprognostic (1.58e-1)		
	unprognostic (1.31e-1)	unprognostic (4.84e-2)	unprognostic (1.73e-1)		
unprognostic (4.22e-1)	unprognostic (5.58e-3)	unprognostic (5.85e-2)	potential prognostic favorable (8.68e-4)	unprognostic (2.17e-1)	unprognostic (6.76e-2)
unprognostic (6.27e-2)	unprognostic (7.13e-2)		unprognostic (1.71e-1)	unprognostic (1.96e-1)	unprognostic (2.04e-1)
unprognostic (5.45e-2)	unprognostic (8.98e-2)	unprognostic (5.60e-3)	unprognostic (1.16e-2)	unprognostic (1.86e-3)	
unprognostic (1.30e-1)	unprognostic (7.23e-2)	unprognostic (9.68e-2)	unprognostic (1.39e-2)	unprognostic (1.69e-1)	unprognostic (2.89e-1)
unprognostic (1.42e-2)	unprognostic (9.04e-3)				
unprognostic (4.47e-1)	unprognostic (1.39e-1)	potential prognostic favorable (2.42e-4)	unprognostic (1.22e-1)	unprognostic (1.71e-1)	unprognostic (5.98e-3)
unprognostic (2.34e-1)	unprognostic (1.30e-2)	unprognostic (2.57e-1)	unprognostic (1.91e-3)	unprognostic (2.56e-1)	unprognostic (3.88e-2)
unprognostic (1.42e-1)	unprognostic (1.95e-2)	unprognostic (7.39e-2)	unprognostic (1.70e-2)	unprognostic (1.50e-2)	unprognostic (3.97e-1)
unprognostic (1.00e-1)	unprognostic (1.57e-1)	unprognostic (3.96e-1)	unprognostic (9.59e-2)	unprognostic (7.17e-3)	unprognostic (1.11e-1)
unprognostic (1.13e-2)	unprognostic (6.50e-2)	unprognostic (8.50e-2)	unprognostic (1.11e-3)	unprognostic (1.27e-1)	unprognostic (1.80e-1)
unprognostic (1.91e-2)	unprognostic (2.81e-2)	unprognostic (1.77e-1)	unprognostic (4.38e-2)	unprognostic (2.88e-2)	unprognostic (3.49e-1)
unprognostic (1.49e-1)	unprognostic (2.50e-2)	unprognostic (7.79e-3)	unprognostic (1.17e-1)	unprognostic (1.20e-1)	unprognostic (3.37e-2)
	unprognostic (2.32e-1)		unprognostic (1.74e-1)		unprognostic (1.37e-2)
unprognostic (3.18e-1)	unprognostic (1.68e-3)	unprognostic (7.40e-2)	unprognostic (2.14e-2)	unprognostic (2.28e-2)	unprognostic (2.35e-2)
unprognostic (1.40e-1)	unprognostic (2.27e-1)	unprognostic (7.26e-2)	unprognostic (1.14e-3)	unprognostic (7.65e-2)	unprognostic (6.98e-2)
		unprognostic (2.13e-1)	potential prognostic favorable (1.33e-4)	unprognostic (3.54e-2)	
unprognostic (1.08e-1)	unprognostic (1.07e-2)	unprognostic (1.49e-1)	unprognostic (8.10e-2)	unprognostic (1.30e-2)	unprognostic (3.74e-2)
unprognostic (2.18e-2)	unprognostic (5.00e-2)	unprognostic (1.60e-1)	unprognostic (6.18e-2)	unprognostic (9.96e-2)	unprognostic (1.07e-2)
	unprognostic (3.88e-2)	unprognostic (1.47e-1)	unprognostic (2.18e-1)	unprognostic (1.03e-1)	unprognostic (3.50e-2)
unprognostic (4.33e-2)	unprognostic (4.17e-2)	unprognostic (8.47e-3)	unprognostic (2.29e-1)		
unprognostic (1.57e-1)	unprognostic (6.55e-2)	unprognostic (5.86e-2)	unprognostic (1.60e-3)	unprognostic (2.40e-1)	unprognostic (1.03e-1)
unprognostic (1.24e-1)	unprognostic (1.65e-2)	unprognostic (2.97e-2)	unprognostic (1.31e-1)	unprognostic (9.21e-3)	
		unprognostic (2.28e-3)	unprognostic (2.30e-1)		unprognostic (1.79e-2)
	unprognostic (1.66e-2)	unprognostic (2.46e-1)	unprognostic (1.31e-2)	unprognostic (2.35e-1)	unprognostic (5.14e-3)
unprognostic (1.17e-1)	unprognostic (3.78e-1)	unprognostic (1.51e-1)	unprognostic (8.44e-2)	unprognostic (1.21e-1)	unprognostic (7.86e-2)
			unprognostic (3.52e-2)		
unprognostic (2.43e-2)	unprognostic (9.49e-2)	unprognostic (3.36e-2)	unprognostic (3.79e-2)	unprognostic (1.37e-1)	unprognostic (5.16e-2)
unprognostic (6.54e-3)	unprognostic (7.43e-2)	potential prognostic unfavorable (2.93e-4)	unprognostic (1.10e-2)	unprognostic (4.88e-2)	unprognostic (3.45e-1)
unprognostic (1.93e-2)	unprognostic (4.09e-2)	unprognostic (1.19e-1)	unprognostic (1.30e-1)	unprognostic (1.00e-1)	unprognostic (8.12e-2)
unprognostic (2.27e-2)	unprognostic (2.45e-1)		unprognostic (1.80e-1)	unprognostic (2.72e-1)	
	unprognostic (9.75e-3)			unprognostic (3.52e-2)	
unprognostic (1.24e-1)	unprognostic (2.60e-2)	unprognostic (2.15e-1)	unprognostic (9.78e-2)	unprognostic (1.16e-1)	unprognostic (1.17e-1)
		unprognostic (8.98e-2)	unprognostic (9.10e-2)		
unprognostic (3.38e-1)	unprognostic (4.02e-2)	unprognostic (8.18e-3)	unprognostic (2.34e-3)	unprognostic (5.57e-2)	
unprognostic (5.44e-2)	unprognostic (1.76e-1)	unprognostic (1.98e-2)	potential prognostic favorable (3.75e-6)	unprognostic (4.65e-2)	unprognostic (2.11e-1)
		unprognostic (1.40e-1)	unprognostic (2.07e-2)		
unprognostic (1.07e-1)	unprognostic (4.60e-1)	unprognostic (2.66e-1)	unprognostic (1.08e-1)	unprognostic (6.24e-2)	unprognostic (6.21e-3)
unprognostic (7.10e-2)	unprognostic (3.36e-2)	unprognostic (1.63e-1)	unprognostic (1.97e-1)	unprognostic (3.34e-1)	unprognostic (3.43e-1)
unprognostic (8.25e-3)	unprognostic (3.53e-1)	unprognostic (3.84e-1)	unprognostic (3.08e-3)	unprognostic (3.43e-2)	unprognostic (3.96e-2)
unprognostic (4.13e-1)	unprognostic (1.86e-1)	unprognostic (4.37e-1)	unprognostic (1.02e-1)	unprognostic (1.78e-1)	potential prognostic unfavorable (2.96e-6)
unprognostic (2.50e-2)	unprognostic (2.53e-1)	unprognostic (6.97e-3)	unprognostic (1.65e-1)	unprognostic (1.04e-1)	unprognostic (1.64e-1)
unprognostic (5.70e-2)	unprognostic (3.02e-1)	unprognostic (1.89e-1)	unprognostic (1.49e-3)	unprognostic (6.64e-3)	potential prognostic unfavorable (6.22e-4)
unprognostic (6.42e-2)	unprognostic (2.80e-1)	unprognostic (1.73e-1)	unprognostic (1.26e-3)	unprognostic (1.49e-1)	
unprognostic (2.41e-2)	unprognostic (4.50e-3)	unprognostic (1.60e-1)	unprognostic (3.32e-1)	potential prognostic unfavorable (6.93e-5)	unprognostic (9.17e-2)
unprognostic (5.86e-3)	unprognostic (2.63e-1)	unprognostic (1.00e-1)	unprognostic (3.09e-1)	unprognostic (1.56e-1)	unprognostic (5.56e-2)
	unprognostic (1.25e-1)		unprognostic (1.85e-1)		
unprognostic (1.77e-1)	unprognostic (1.90e-2)	unprognostic (4.44e-1)	unprognostic (5.54e-2)	unprognostic (2.50e-2)	unprognostic (3.18e-1)
unprognostic (2.46e-2)	unprognostic (2.82e-1)	unprognostic (7.64e-2)	unprognostic (1.54e-2)	unprognostic (4.31e-1)	unprognostic (1.97e-2)
unprognostic (1.30e-1)	unprognostic (6.39e-2)	unprognostic (2.17e-2)	unprognostic (5.11e-3)	unprognostic (8.49e-2)	unprognostic (4.02e-2)
unprognostic (1.54e-1)	unprognostic (9.49e-2)				
unprognostic (2.26e-3)	unprognostic (1.44e-1)	unprognostic (3.50e-1)	unprognostic (7.04e-3)	unprognostic (4.83e-3)	unprognostic (7.43e-2)
unprognostic (1.78e-1)	unprognostic (3.99e-2)	unprognostic (3.84e-1)	potential prognostic favorable (7.49e-4)	unprognostic (7.77e-3)	unprognostic (2.51e-3)
unprognostic (4.00e-3)	unprognostic (1.65e-1)	unprognostic (2.01e-1)	unprognostic (4.07e-2)	unprognostic (1.33e-1)	unprognostic (4.69e-2)
unprognostic (5.30e-2)	unprognostic (2.66e-1)	unprognostic (2.73e-1)	unprognostic (2.02e-1)	unprognostic (2.92e-2)	unprognostic (1.47e-1)
unprognostic (1.47e-1)	unprognostic (1.77e-1)	unprognostic (1.33e-1)	unprognostic (1.87e-1)	unprognostic (1.13e-2)	unprognostic (1.55e-2)
unprognostic (4.42e-2)	unprognostic (4.53e-3)	unprognostic (1.28e-1)	unprognostic (1.97e-3)	unprognostic (9.85e-3)	unprognostic (9.86e-2)
unprognostic (1.09e-1)	unprognostic (2.31e-1)	unprognostic (1.51e-1)	unprognostic (2.09e-1)	unprognostic (2.57e-2)	unprognostic (1.65e-1)
unprognostic (1.57e-1)	unprognostic (1.34e-1)	unprognostic (5.21e-2)	unprognostic (5.45e-2)	unprognostic (6.61e-3)	unprognostic (6.59e-3)
unprognostic (3.33e-3)	unprognostic (8.98e-2)	unprognostic (1.40e-1)	unprognostic (2.27e-2)	potential prognostic favorable (1.63e-4)	unprognostic (8.39e-2)
unprognostic (1.54e-2)	unprognostic (7.07e-2)	unprognostic (1.12e-1)	unprognostic (1.10e-1)		
unprognostic (4.94e-2)	unprognostic (3.32e-2)	unprognostic (5.08e-3)	unprognostic (1.60e-1)	unprognostic (1.49e-2)	unprognostic (1.88e-1)
		unprognostic (3.25e-1)	unprognostic (6.75e-2)		unprognostic (1.84e-1)
unprognostic (1.41e-1)	unprognostic (1.81e-1)	unprognostic (1.80e-1)	unprognostic (8.95e-2)	unprognostic (2.45e-1)	unprognostic (5.24e-2)
unprognostic (5.04e-2)	unprognostic (6.96e-2)	unprognostic (2.58e-2)	unprognostic (2.05e-1)	unprognostic (1.33e-2)	unprognostic (1.40e-1)
unprognostic (1.58e-1)	unprognostic (1.33e-1)	unprognostic (1.25e-1)	unprognostic (9.62e-2)	unprognostic (8.53e-2)	unprognostic (4.87e-1)
unprognostic (3.81e-1)	unprognostic (3.41e-2)	unprognostic (8.53e-2)	unprognostic (1.69e-1)	unprognostic (1.34e-2)	
unprognostic (1.23e-1)	unprognostic (3.94e-2)	unprognostic (4.18e-1)	potential prognostic favorable (2.73e-4)	unprognostic (3.19e-2)	unprognostic (1.65e-3)
unprognostic (3.75e-2)	unprognostic (2.30e-3)	unprognostic (9.55e-2)	potential prognostic favorable (6.69e-4)	unprognostic (3.10e-2)	unprognostic (2.35e-1)
unprognostic (1.63e-1)	unprognostic (1.90e-2)	unprognostic (1.10e-1)	unprognostic (1.16e-1)	potential prognostic favorable (3.35e-4)	unprognostic (2.15e-1)
unprognostic (9.82e-2)	unprognostic (7.47e-3)	unprognostic (1.26e-1)	unprognostic (6.82e-2)	unprognostic (3.19e-1)	unprognostic (5.00e-2)
unprognostic (1.51e-1)	unprognostic (2.75e-2)	unprognostic (7.87e-2)	unprognostic (5.82e-3)	unprognostic (1.96e-2)	unprognostic (6.03e-2)
unprognostic (6.74e-2)	unprognostic (4.89e-2)	unprognostic (2.01e-1)	unprognostic (7.48e-2)	unprognostic (3.18e-3)	unprognostic (2.93e-2)
unprognostic (1.29e-1)	unprognostic (3.49e-1)	unprognostic (1.03e-1)	unprognostic (6.81e-3)	unprognostic (2.91e-3)	unprognostic (4.11e-1)
	unprognostic (8.44e-2)	unprognostic (2.13e-1)	unprognostic (9.65e-3)	unprognostic (1.72e-2)	
unprognostic (2.15e-1)	unprognostic (1.09e-1)			unprognostic (4.82e-2)	unprognostic (2.71e-2)
unprognostic (9.99e-3)	unprognostic (6.66e-2)	unprognostic (1.26e-2)	potential prognostic unfavorable (3.00e-4)	unprognostic (9.28e-2)	unprognostic (3.31e-2)
		unprognostic (7.21e-2)	unprognostic (1.76e-1)		
unprognostic (3.11e-2)	unprognostic (1.23e-1)	unprognostic (1.03e-2)	unprognostic (5.93e-2)	unprognostic (4.29e-2)	unprognostic (1.50e-2)
unprognostic (2.81e-2)	unprognostic (7.53e-2)	unprognostic (1.98e-3)	potential prognostic unfavorable (5.16e-4)	unprognostic (9.77e-2)	unprognostic (9.36e-2)
unprognostic (7.27e-2)	unprognostic (1.35e-3)	unprognostic (2.61e-1)	potential prognostic favorable (7.22e-4)	unprognostic (7.34e-2)	unprognostic (3.57e-2)
unprognostic (1.34e-1)	unprognostic (5.13e-2)	unprognostic (2.63e-1)	unprognostic (7.39e-2)	unprognostic (1.39e-2)	unprognostic (1.73e-2)
		unprognostic (1.53e-2)			
unprognostic (1.62e-3)	unprognostic (8.52e-2)	unprognostic (2.75e-1)	unprognostic (1.27e-3)	unprognostic (7.33e-2)	unprognostic (2.62e-3)
unprognostic (1.77e-2)	unprognostic (5.50e-2)	unprognostic (1.88e-1)	unprognostic (4.87e-2)	potential prognostic unfavorable (7.90e-4)	unprognostic (2.86e-2)
unprognostic (7.37e-2)	unprognostic (2.64e-1)	unprognostic (3.64e-1)	unprognostic (1.06e-1)	unprognostic (1.45e-1)	unprognostic (4.34e-2)
unprognostic (5.43e-2)	unprognostic (2.27e-1)	unprognostic (1.82e-1)	unprognostic (1.29e-1)	unprognostic (1.51e-1)	unprognostic (2.08e-2)
				unprognostic (4.50e-2)	
unprognostic (1.50e-1)	unprognostic (1.56e-1)	potential prognostic unfavorable (1.99e-4)	unprognostic (3.59e-1)	unprognostic (3.45e-2)	unprognostic (1.09e-2)
	unprognostic (1.03e-1)		unprognostic (2.77e-1)		
unprognostic (3.56e-2)	unprognostic (7.93e-2)	unprognostic (2.88e-1)	unprognostic (1.30e-2)	unprognostic (2.75e-2)	unprognostic (6.47e-2)
			unprognostic (1.63e-2)		
unprognostic (3.38e-1)	unprognostic (1.10e-1)	unprognostic (1.31e-1)	unprognostic (2.92e-1)	unprognostic (1.35e-2)	unprognostic (1.92e-1)
unprognostic (4.70e-2)	unprognostic (3.28e-1)	unprognostic (1.69e-1)	unprognostic (2.48e-1)	unprognostic (3.83e-2)	unprognostic (1.76e-3)
unprognostic (2.21e-1)	unprognostic (3.79e-2)	unprognostic (2.11e-1)	unprognostic (2.41e-1)	unprognostic (8.49e-3)	unprognostic (2.19e-3)
			unprognostic (4.28e-2)		
potential prognostic favorable (8.40e-4)	unprognostic (1.06e-3)	unprognostic (1.20e-1)	unprognostic (1.94e-3)	unprognostic (2.70e-1)	unprognostic (2.81e-1)
unprognostic (2.56e-2)	unprognostic (7.23e-2)	unprognostic (4.83e-1)	potential prognostic favorable (8.02e-4)	unprognostic (7.29e-2)	unprognostic (3.84e-2)
unprognostic (9.12e-3)	unprognostic (1.19e-2)	unprognostic (4.30e-1)	unprognostic (1.15e-1)	unprognostic (8.92e-3)	unprognostic (1.85e-1)
unprognostic (7.87e-2)	unprognostic (2.69e-2)	unprognostic (3.85e-1)	unprognostic (2.75e-1)	unprognostic (2.00e-1)	unprognostic (2.22e-1)
unprognostic (1.97e-1)	unprognostic (6.10e-2)	unprognostic (2.92e-1)	unprognostic (1.67e-1)	unprognostic (2.84e-1)	unprognostic (7.17e-3)
unprognostic (3.84e-2)	unprognostic (1.88e-1)	unprognostic (6.16e-2)	unprognostic (2.97e-1)	unprognostic (1.03e-1)	unprognostic (7.19e-2)
unprognostic (9.02e-2)	unprognostic (1.16e-1)			unprognostic (1.38e-2)	unprognostic (1.26e-2)
unprognostic (2.97e-1)	unprognostic (1.47e-1)	unprognostic (1.31e-1)	unprognostic (7.33e-3)	unprognostic (1.16e-3)	unprognostic (1.97e-1)
unprognostic (6.10e-2)	unprognostic (1.38e-1)	unprognostic (2.47e-3)	unprognostic (5.08e-2)	unprognostic (2.77e-2)	potential prognostic favorable (3.26e-4)
unprognostic (1.09e-1)	unprognostic (4.68e-2)		unprognostic (2.82e-1)	unprognostic (8.15e-2)	
unprognostic (8.32e-2)	unprognostic (3.12e-1)	unprognostic (1.52e-1)	unprognostic (6.28e-2)	unprognostic (5.63e-3)	unprognostic (2.83e-1)
unprognostic (7.83e-2)	unprognostic (1.32e-1)	unprognostic (3.05e-1)	unprognostic (7.24e-2)	unprognostic (4.68e-3)	unprognostic (3.70e-2)
unprognostic (2.23e-1)	unprognostic (1.78e-2)	unprognostic (2.74e-1)	unprognostic (9.97e-2)	unprognostic (2.05e-1)	unprognostic (2.24e-2)
	unprognostic (3.20e-1)				
unprognostic (2.67e-2)	unprognostic (1.87e-2)	unprognostic (1.71e-1)	unprognostic (8.29e-2)	potential prognostic favorable (9.96e-4)	unprognostic (1.45e-1)
unprognostic (6.81e-2)	unprognostic (1.39e-1)	unprognostic (1.64e-1)	unprognostic (1.79e-2)	unprognostic (2.07e-2)	unprognostic (4.01e-2)
unprognostic (9.42e-2)	unprognostic (1.68e-1)	unprognostic (2.52e-2)	unprognostic (6.50e-2)	unprognostic (1.15e-2)	unprognostic (5.15e-2)
	unprognostic (1.19e-1)	unprognostic (1.16e-1)	unprognostic (4.26e-1)	unprognostic (7.36e-2)	
unprognostic (1.58e-2)	unprognostic (2.99e-1)			unprognostic (2.83e-1)	unprognostic (5.96e-2)
unprognostic (2.66e-1)	potential prognostic unfavorable (1.93e-4)			unprognostic (1.12e-1)	unprognostic (2.60e-2)
unprognostic (8.18e-2)	unprognostic (2.70e-2)	unprognostic (1.31e-1)	unprognostic (3.13e-1)	unprognostic (4.46e-2)	potential prognostic unfavorable (1.15e-4)
unprognostic (1.20e-3)	unprognostic (7.15e-2)	unprognostic (1.42e-1)	unprognostic (4.23e-3)	unprognostic (4.28e-2)	unprognostic (2.30e-2)

unprognostic (1.70e-1)	unprognostic (1.22e-2)	unprognostic (2.80e-3)	unprognostic (2.67e-1)	unprognostic (3.99e-2)	
	unprognostic (8.22e-2)		unprognostic (1.22e-3)		
unprognostic (1.35e-2)	unprognostic (4.70e-1)	unprognostic (4.29e-2)	potential prognostic unfavorable (1.18e-4)	unprognostic (1.39e-1)	unprognostic (3.38e-1)
unprognostic (4.64e-2)	unprognostic (1.07e-1)	unprognostic (7.80e-3)	potential prognostic favorable (6.86e-6)	unprognostic (4.96e-2)	unprognostic (2.21e-1)
unprognostic (2.38e-3)	unprognostic (8.10e-2)	unprognostic (1.33e-1)	potential prognostic unfavorable (3.64e-4)	unprognostic (1.29e-1)	potential prognostic unfavorable (2.70e-4)
unprognostic (2.07e-2)	unprognostic (2.47e-2)	unprognostic (2.31e-1)	unprognostic (1.79e-3)	unprognostic (1.08e-1)	unprognostic (3.39e-3)
unprognostic (3.91e-2)	unprognostic (2.28e-1)	unprognostic (4.91e-2)	unprognostic (7.71e-3)	unprognostic (1.10e-1)	unprognostic (3.65e-1)
unprognostic (1.73e-2)	unprognostic (1.21e-1)	unprognostic (1.11e-1)	potential prognostic unfavorable (8.12e-5)	unprognostic (1.86e-1)	unprognostic (1.85e-2)
unprognostic (3.56e-3)	unprognostic (4.72e-2)	unprognostic (1.08e-2)	potential prognostic unfavorable (3.04e-5)	unprognostic (1.11e-3)	unprognostic (3.61e-2)
unprognostic (1.66e-1)	unprognostic (5.34e-2)	unprognostic (9.46e-2)	unprognostic (1.45e-1)	unprognostic (5.41e-2)	unprognostic (7.79e-3)
			potential prognostic favorable (5.15e-4)	unprognostic (8.67e-3)	
unprognostic (2.20e-2)	unprognostic (7.67e-2)	unprognostic (4.66e-1)	unprognostic (1.18e-1)	unprognostic (7.55e-2)	unprognostic (3.36e-2)
unprognostic (2.93e-2)	unprognostic (4.95e-2)	unprognostic (3.55e-1)	unprognostic (1.67e-1)	unprognostic (1.12e-1)	unprognostic (1.31e-2)
unprognostic (6.66e-2)	unprognostic (1.24e-1)	unprognostic (7.00e-2)	unprognostic (3.94e-2)	unprognostic (1.87e-1)	unprognostic (1.56e-1)
unprognostic (1.91e-1)	unprognostic (5.13e-3)	unprognostic (1.89e-2)	unprognostic (2.09e-3)	unprognostic (1.43e-1)	unprognostic (1.63e-2)
unprognostic (1.52e-1)	unprognostic (2.76e-1)	unprognostic (5.31e-2)	unprognostic (3.61e-3)	unprognostic (1.06e-3)	unprognostic (1.15e-1)
unprognostic (1.75e-1)	unprognostic (1.86e-2)	unprognostic (5.54e-2)	unprognostic (2.64e-2)	unprognostic (7.38e-3)	potential prognostic unfavorable (1.02e-4)
			unprognostic (3.21e-3)		unprognostic (2.28e-1)
unprognostic (2.33e-2)	unprognostic (2.77e-2)	unprognostic (2.25e-1)	unprognostic (2.74e-3)	unprognostic (3.86e-1)	unprognostic (1.74e-2)
	unprognostic (1.45e-1)		unprognostic (2.66e-2)		
unprognostic (2.76e-1)	unprognostic (1.34e-1)			potential prognostic favorable (7.29e-7)	
unprognostic (1.41e-2)	unprognostic (4.57e-2)	unprognostic (3.34e-1)	unprognostic (1.56e-1)	unprognostic (1.14e-1)	unprognostic (1.02e-1)
unprognostic (3.81e-2)	unprognostic (6.52e-3)	unprognostic (1.34e-2)	potential prognostic unfavorable (3.99e-4)	unprognostic (2.36e-1)	unprognostic (2.64e-1)
unprognostic (2.11e-1)	unprognostic (4.46e-2)	unprognostic (2.02e-3)	unprognostic (3.39e-2)	unprognostic (1.37e-1)	unprognostic (2.19e-1)
	unprognostic (3.61e-1)	unprognostic (2.06e-2)	unprognostic (1.15e-1)		
unprognostic (8.33e-2)	unprognostic (9.51e-2)	unprognostic (3.03e-2)	unprognostic (9.52e-2)	potential prognostic favorable (3.95e-4)	unprognostic (3.29e-1)
	unprognostic (2.48e-1)		unprognostic (1.57e-1)		
	unprognostic (1.02e-1)	unprognostic (2.67e-1)	unprognostic (1.15e-1)		
unprognostic (1.98e-2)	unprognostic (4.77e-2)	unprognostic (9.07e-2)	unprognostic (8.18e-2)	unprognostic (4.37e-2)	unprognostic (1.06e-1)
	unprognostic (3.29e-1)		unprognostic (4.51e-2)	potential prognostic favorable (6.76e-4)	
unprognostic (1.39e-1)	unprognostic (1.53e-2)	unprognostic (2.61e-1)	unprognostic (1.21e-1)	unprognostic (8.05e-2)	unprognostic (4.11e-2)
unprognostic (5.81e-2)	unprognostic (1.90e-1)	unprognostic (1.16e-1)	unprognostic (1.50e-2)	unprognostic (1.57e-1)	unprognostic (1.52e-1)
unprognostic (2.57e-2)	unprognostic (1.85e-1)	unprognostic (1.00e-1)	unprognostic (2.40e-2)	potential prognostic favorable (1.51e-4)	
unprognostic (1.49e-1)	unprognostic (1.11e-1)	unprognostic (3.05e-3)	unprognostic (8.41e-2)	potential prognostic favorable (3.77e-4)	unprognostic (2.18e-1)
unprognostic (1.58e-1)	unprognostic (1.84e-1)		unprognostic (4.89e-2)	unprognostic (2.41e-3)	
unprognostic (1.11e-1)	unprognostic (7.78e-2)	unprognostic (4.32e-2)	unprognostic (2.37e-1)	unprognostic (1.82e-2)	unprognostic (2.86e-1)
unprognostic (3.59e-2)	unprognostic (5.71e-2)	potential prognostic unfavorable (6.62e-4)	unprognostic (7.05e-2)	unprognostic (1.50e-1)	unprognostic (2.11e-2)
	unprognostic (2.87e-1)				
unprognostic (1.18e-1)	unprognostic (1.07e-1)	unprognostic (1.02e-3)	unprognostic (1.90e-2)	unprognostic (3.00e-2)	unprognostic (3.19e-1)
unprognostic (6.14e-2)	unprognostic (9.01e-2)	unprognostic (2.18e-2)	unprognostic (4.95e-2)	unprognostic (1.14e-1)	unprognostic (3.25e-2)
unprognostic (1.18e-1)	unprognostic (1.67e-1)	potential prognostic unfavorable (4.18e-4)	unprognostic (1.78e-1)	potential prognostic favorable (4.85e-4)	unprognostic (1.39e-1)
unprognostic (1.94e-1)	unprognostic (2.85e-1)	unprognostic (1.48e-1)	unprognostic (8.10e-2)	unprognostic (2.40e-2)	unprognostic (9.86e-2)
unprognostic (2.12e-1)	unprognostic (1.99e-1)	unprognostic (1.55e-1)	unprognostic (2.78e-1)	unprognostic (2.07e-1)	unprognostic (1.57e-1)
unprognostic (1.05e-1)	unprognostic (6.79e-2)	unprognostic (2.99e-2)	unprognostic (2.63e-1)	unprognostic (1.42e-3)	unprognostic (2.91e-1)
unprognostic (1.51e-1)	unprognostic (1.76e-1)	potential prognostic unfavorable (2.85e-4)	unprognostic (1.16e-1)	unprognostic (1.03e-3)	
unprognostic (1.31e-3)	unprognostic (1.39e-1)	unprognostic (2.40e-1)	potential prognostic unfavorable (4.96e-4)	unprognostic (5.92e-2)	unprognostic (1.15e-1)
unprognostic (1.08e-1)	unprognostic (6.95e-2)	unprognostic (8.69e-2)	unprognostic (2.88e-3)	unprognostic (1.26e-1)	unprognostic (1.67e-2)
unprognostic (2.51e-1)	unprognostic (4.29e-1)	unprognostic (1.77e-1)	unprognostic (2.56e-1)	unprognostic (2.47e-1)	unprognostic (1.06e-1)
unprognostic (9.34e-3)	unprognostic (6.23e-2)	unprognostic (1.52e-2)	unprognostic (1.84e-2)	unprognostic (2.51e-3)	unprognostic (4.12e-2)
unprognostic (1.67e-1)	unprognostic (1.64e-1)	unprognostic (2.36e-1)	unprognostic (4.53e-3)	unprognostic (3.30e-1)	unprognostic (7.30e-2)
unprognostic (1.28e-1)	unprognostic (3.43e-1)	unprognostic (2.77e-1)	unprognostic (9.33e-2)	unprognostic (2.21e-1)	unprognostic (9.84e-2)
unprognostic (4.60e-1)	unprognostic (9.07e-2)	unprognostic (1.70e-1)	unprognostic (1.50e-2)	unprognostic (6.47e-3)	unprognostic (2.93e-2)
		unprognostic (5.62e-2)	unprognostic (2.29e-2)		
unprognostic (3.89e-2)	unprognostic (1.53e-1)				
unprognostic (1.19e-2)	unprognostic (2.48e-1)	unprognostic (2.32e-1)	unprognostic (2.66e-2)	unprognostic (7.64e-2)	
			unprognostic (3.88e-2)	unprognostic (2.11e-3)	
unprognostic (6.62e-2)	unprognostic (1.27e-2)	unprognostic (1.04e-1)	unprognostic (3.91e-2)	unprognostic (1.17e-2)	unprognostic (2.08e-2)
		unprognostic (1.80e-2)	unprognostic (3.71e-2)		
unprognostic (8.62e-2)	unprognostic (3.66e-1)	unprognostic (9.58e-2)	unprognostic (5.26e-2)	unprognostic (6.52e-3)	unprognostic (1.55e-1)
unprognostic (9.49e-2)	unprognostic (4.81e-2)	unprognostic (2.68e-2)	unprognostic (2.09e-3)	unprognostic (2.12e-2)	unprognostic (2.57e-1)
unprognostic (1.42e-1)	unprognostic (4.29e-3)	unprognostic (1.84e-2)	unprognostic (7.22e-2)	unprognostic (4.82e-3)	unprognostic (3.71e-2)
unprognostic (3.80e-2)	unprognostic (2.17e-1)	potential prognostic unfavorable (4.08e-5)	unprognostic (2.33e-1)	unprognostic (1.96e-1)	potential prognostic unfavorable (7.70e-5)
unprognostic (1.59e-2)	unprognostic (8.80e-3)			unprognostic (4.29e-3)	
unprognostic (9.68e-2)	unprognostic (1.47e-2)			unprognostic (2.14e-2)	
unprognostic (6.21e-2)	unprognostic (6.04e-2)	unprognostic (4.44e-1)	unprognostic (3.76e-2)	unprognostic (5.35e-2)	unprognostic (5.78e-2)
unprognostic (1.42e-1)	unprognostic (8.38e-2)	unprognostic (8.69e-2)	unprognostic (1.65e-2)	unprognostic (6.40e-2)	unprognostic (1.13e-1)
unprognostic (2.73e-1)	unprognostic (7.45e-2)	unprognostic (5.78e-2)	unprognostic (9.01e-2)	unprognostic (2.44e-2)	unprognostic (8.71e-3)
unprognostic (9.41e-3)	unprognostic (2.16e-2)	unprognostic (1.80e-1)	unprognostic (1.85e-2)	unprognostic (2.74e-1)	unprognostic (1.99e-1)
unprognostic (4.13e-2)	unprognostic (1.73e-1)	unprognostic (1.98e-1)	potential prognostic unfavorable (6.97e-7)	unprognostic (1.79e-3)	unprognostic (4.67e-1)
unprognostic (9.48e-2)	unprognostic (3.30e-2)	unprognostic (1.87e-1)	unprognostic (1.37e-2)	unprognostic (7.23e-3)	unprognostic (1.25e-2)
unprognostic (9.58e-2)	unprognostic (3.20e-1)	unprognostic (1.46e-2)	unprognostic (8.47e-2)	unprognostic (3.94e-2)	
		unprognostic (4.43e-2)	unprognostic (1.10e-2)		
	unprognostic (3.48e-3)	unprognostic (1.88e-1)	unprognostic (2.58e-3)	unprognostic (1.40e-2)	
	unprognostic (2.40e-1)				
unprognostic (4.23e-2)	unprognostic (2.44e-1)		unprognostic (3.19e-2)		
unprognostic (4.33e-2)	unprognostic (1.89e-1)	unprognostic (1.78e-1)	unprognostic (3.36e-1)	unprognostic (2.08e-3)	potential prognostic unfavorable (1.36e-4)
				unprognostic (2.40e-2)	
				unprognostic (3.31e-3)	
unprognostic (4.15e-2)	unprognostic (1.22e-2)	unprognostic (1.20e-2)	unprognostic (5.28e-1)	unprognostic (1.30e-1)	unprognostic (1.47e-3)
				unprognostic (1.13e-1)	
unprognostic (7.92e-2)	unprognostic (1.95e-1)	unprognostic (7.77e-2)	unprognostic (8.92e-2)	unprognostic (1.21e-1)	unprognostic (1.19e-1)
		unprognostic (1.45e-2)	unprognostic (5.49e-2)		
				unprognostic (1.94e-2)	
unprognostic (3.34e-1)	unprognostic (3.64e-1)				
	unprognostic (3.52e-2)				
unprognostic (2.58e-1)	unprognostic (8.50e-3)			unprognostic (8.09e-2)	potential prognostic favorable (8.51e-6)
unprognostic (6.37e-2)	unprognostic (7.83e-2)			unprognostic (2.09e-1)	unprognostic (1.40e-1)
unprognostic (4.33e-2)	unprognostic (4.21e-3)	unprognostic (2.83e-2)	unprognostic (9.94e-2)	unprognostic (1.72e-1)	unprognostic (1.77e-1)
unprognostic (5.76e-3)	unprognostic (1.04e-1)				unprognostic (9.70e-2)
unprognostic (8.70e-2)	unprognostic (2.08e-1)	unprognostic (8.42e-3)	potential prognostic unfavorable (1.43e-4)	unprognostic (9.22e-2)	
unprognostic (8.91e-2)	unprognostic (5.06e-3)	unprognostic (3.03e-1)	unprognostic (9.49e-3)	unprognostic (3.56e-2)	unprognostic (3.46e-1)
unprognostic (2.82e-1)	unprognostic (1.90e-1)	unprognostic (9.82e-2)	unprognostic (1.84e-1)	unprognostic (1.86e-1)	unprognostic (5.81e-3)
unprognostic (2.20e-1)	unprognostic (6.02e-2)		unprognostic (2.99e-2)	unprognostic (7.39e-2)	
unprognostic (2.37e-2)	unprognostic (3.34e-2)	unprognostic (2.99e-1)	unprognostic (1.26e-2)	unprognostic (4.94e-2)	unprognostic (1.39e-1)
unprognostic (1.95e-1)	unprognostic (1.09e-3)	unprognostic (8.99e-3)	unprognostic (3.26e-1)	unprognostic (1.25e-1)	unprognostic (1.46e-2)
			unprognostic (1.58e-2)	unprognostic (1.01e-1)	
unprognostic (4.58e-2)	unprognostic (1.79e-1)			unprognostic (1.59e-2)	unprognostic (4.25e-2)
unprognostic (3.77e-1)	unprognostic (1.90e-3)	unprognostic (4.82e-2)	unprognostic (3.07e-1)	unprognostic (2.74e-2)	potential prognostic unfavorable (6.12e-4)
	unprognostic (1.22e-1)	unprognostic (3.74e-1)	unprognostic (1.77e-1)		unprognostic (2.45e-1)
unprognostic (1.08e-1)	unprognostic (2.63e-1)	unprognostic (1.09e-1)	unprognostic (8.97e-2)	unprognostic (1.43e-1)	unprognostic (7.85e-2)
unprognostic (1.75e-1)	unprognostic (2.60e-1)			unprognostic (2.52e-1)	
unprognostic (1.44e-1)	unprognostic (2.56e-1)	unprognostic (3.76e-2)	unprognostic (1.66e-3)	unprognostic (2.05e-1)	unprognostic (2.63e-2)
unprognostic (3.07e-1)	unprognostic (1.85e-1)	unprognostic (2.06e-1)	unprognostic (8.74e-2)	unprognostic (1.29e-2)	unprognostic (2.23e-2)
unprognostic (1.40e-2)	unprognostic (5.59e-2)	potential prognostic unfavorable (1.87e-4)	unprognostic (3.11e-3)	unprognostic (1.03e-1)	unprognostic (2.59e-1)
	unprognostic (2.40e-1)		potential prognostic unfavorable (3.09e-4)		
unprognostic (6.42e-3)	unprognostic (1.25e-1)			unprognostic (6.74e-2)	unprognostic (8.47e-2)
potential prognostic unfavorable (6.57e-4)	unprognostic (5.21e-2)	unprognostic (3.00e-2)	unprognostic (9.22e-3)	unprognostic (1.66e-3)	unprognostic (3.72e-3)
unprognostic (6.91e-2)	unprognostic (5.69e-2)	unprognostic (3.33e-1)	unprognostic (3.65e-3)	unprognostic (5.63e-2)	unprognostic (1.70e-1)
unprognostic (2.53e-2)	unprognostic (1.16e-1)	unprognostic (9.78e-2)	unprognostic (1.01e-1)	unprognostic (8.10e-2)	unprognostic (3.68e-1)
unprognostic (2.18e-2)	unprognostic (6.54e-2)	unprognostic (5.32e-2)	unprognostic (7.78e-2)	unprognostic (1.92e-2)	unprognostic (1.13e-3)
unprognostic (5.48e-3)	unprognostic (9.48e-2)	unprognostic (8.35e-2)	potential prognostic unfavorable (2.84e-8)	unprognostic (1.74e-2)	unprognostic (7.22e-3)
unprognostic (3.56e-2)	unprognostic (3.47e-1)	unprognostic (1.20e-1)	unprognostic (5.18e-2)	unprognostic (3.77e-2)	potential prognostic unfavorable (4.23e-4)
unprognostic (5.64e-2)	unprognostic (2.10e-1)	unprognostic (6.01e-2)	unprognostic (2.52e-1)	unprognostic (3.83e-1)	unprognostic (1.05e-1)
unprognostic (2.15e-1)	unprognostic (1.41e-1)	unprognostic (1.71e-1)	unprognostic (9.11e-3)	potential prognostic unfavorable (9.69e-5)	potential prognostic unfavorable (4.20e-4)
unprognostic (3.67e-2)	unprognostic (2.90e-2)	unprognostic (3.06e-1)	unprognostic (1.49e-1)	unprognostic (2.53e-1)	unprognostic (2.03e-3)
	unprognostic (9.40e-2)	unprognostic (6.10e-2)	unprognostic (3.66e-2)		unprognostic (2.09e-1)
unprognostic (7.47e-2)	unprognostic (7.62e-2)	unprognostic (3.78e-2)	unprognostic (1.25e-1)	unprognostic (2.35e-3)	unprognostic (1.25e-1)
unprognostic (3.32e-2)	unprognostic (9.35e-3)	unprognostic (7.78e-2)	unprognostic (3.08e-1)	unprognostic (1.12e-1)	
unprognostic (5.77e-2)	unprognostic (2.22e-2)	unprognostic (3.87e-2)	potential prognostic unfavorable (1.98e-4)	unprognostic (1.91e-2)	unprognostic (6.46e-2)
unprognostic (4.64e-2)	unprognostic (6.07e-2)	unprognostic (1.42e-1)	unprognostic (2.26e-2)	unprognostic (3.62e-1)	unprognostic (5.77e-2)
unprognostic (8.00e-2)	unprognostic (8.95e-2)	unprognostic (1.33e-2)	unprognostic (1.33e-1)	unprognostic (1.17e-2)	unprognostic (2.13e-1)
unprognostic (4.38e-1)	unprognostic (1.37e-1)	unprognostic (4.15e-3)	unprognostic (1.60e-1)	unprognostic (5.95e-2)	unprognostic (3.28e-3)
unprognostic (2.78e-2)	unprognostic (1.98e-1)	unprognostic (2.61e-1)	unprognostic (2.67e-3)	unprognostic (1.14e-1)	unprognostic (5.21e-2)
unprognostic (1.62e-1)	unprognostic (2.36e-1)	unprognostic (2.06e-2)	unprognostic (1.05e-1)	unprognostic (2.89e-2)	unprognostic (5.54e-3)
unprognostic (1.09e-1)	unprognostic (3.34e-2)	potential prognostic unfavorable (3.16e-5)	unprognostic (3.34e-2)	unprognostic (9.43e-2)	unprognostic (1.03e-1)
		unprognostic (3.25e-2)	unprognostic (4.05e-3)		
unprognostic (2.87e-1)	unprognostic (3.71e-2)	unprognostic (2.62e-1)	unprognostic (1.55e-1)	unprognostic (2.91e-1)	unprognostic (1.92e-1)

unprognostic (5.10e-2)	unprognostic (1.04e-1)	unprognostic (6.48e-2)	unprognostic (3.25e-2)	unprognostic (1.76e-1)	unprognostic (1.71e-1)
unprognostic (7.78e-2)	unprognostic (3.47e-2)	unprognostic (2.65e-3)	unprognostic (4.52e-3)	unprognostic (3.32e-3)	unprognostic (1.72e-2)
unprognostic (4.93e-2)	unprognostic (6.59e-2)	unprognostic (3.94e-1)	unprognostic (3.46e-1)	unprognostic (5.85e-2)	potential prognostic unfavorable (7.37e-5)
unprognostic (2.27e-1)	unprognostic (1.48e-1)	unprognostic (2.48e-1)	unprognostic (8.14e-3)	unprognostic (8.87e-3)	unprognostic (3.41e-2)
unprognostic (3.00e-1)	unprognostic (4.57e-3)	unprognostic (8.65e-2)	unprognostic (3.52e-1)	unprognostic (1.69e-1)	unprognostic (1.21e-3)
unprognostic (7.69e-2)	unprognostic (1.07e-1)	unprognostic (2.09e-2)	unprognostic (8.02e-2)	unprognostic (1.32e-1)	unprognostic (2.80e-2)
unprognostic (5.18e-1)	unprognostic (2.14e-1)	unprognostic (8.98e-2)	potential prognostic favorable (5.06e-6)	unprognostic (5.20e-2)	unprognostic (5.98e-3)
unprognostic (2.94e-1)	unprognostic (1.40e-1)	unprognostic (5.92e-2)	unprognostic (3.78e-1)	unprognostic (1.64e-3)	unprognostic (3.53e-1)
unprognostic (3.34e-1)	unprognostic (3.78e-1)	unprognostic (1.17e-1)	potential prognostic favorable (1.98e-6)	unprognostic (1.19e-1)	unprognostic (3.77e-1)
unprognostic (9.58e-2)	unprognostic (7.74e-2)	unprognostic (7.09e-2)	unprognostic (1.14e-1)	unprognostic (4.11e-2)	potential prognostic unfavorable (2.16e-4)
unprognostic (1.90e-1)	unprognostic (2.94e-1)	unprognostic (2.62e-1)	unprognostic (2.71e-2)	unprognostic (6.05e-3)	unprognostic (1.55e-1)
unprognostic (5.18e-2)	unprognostic (2.27e-2)	potential prognostic unfavorable (2.49e-4)	unprognostic (8.09e-3)	unprognostic (3.10e-3)	unprognostic (2.67e-2)
unprognostic (7.50e-2)	unprognostic (9.46e-2)	unprognostic (2.30e-3)	unprognostic (3.60e-1)	unprognostic (1.56e-3)	unprognostic (1.19e-2)
unprognostic (3.09e-1)	unprognostic (2.17e-2)	unprognostic (3.61e-2)	unprognostic (2.43e-1)		
validated prognostic favorable (6.39e-4)	validated prognostic favorable (6.93e-4)	unprognostic (5.42e-2)	unprognostic (2.80e-1)	unprognostic (5.56e-2)	unprognostic (1.23e-2)
unprognostic (8.41e-3)	unprognostic (1.43e-2)			unprognostic (3.71e-3)	
unprognostic (7.61e-3)	unprognostic (9.20e-2)	potential prognostic unfavorable (8.44e-5)	unprognostic (2.32e-3)	unprognostic (4.89e-3)	unprognostic (6.83e-3)
unprognostic (2.22e-2)	unprognostic (5.96e-2)		unprognostic (1.08e-2)	unprognostic (2.24e-2)	unprognostic (3.10e-2)
unprognostic (3.41e-2)	unprognostic (1.98e-1)	unprognostic (1.82e-1)	unprognostic (3.13e-2)	unprognostic (6.77e-3)	unprognostic (1.15e-1)
unprognostic (3.84e-2)	unprognostic (2.45e-1)	unprognostic (3.29e-1)	unprognostic (1.01e-1)	unprognostic (3.32e-1)	unprognostic (4.09e-1)
unprognostic (1.33e-2)	unprognostic (1.18e-1)	unprognostic (1.30e-1)	unprognostic (4.55e-2)	unprognostic (3.36e-2)	unprognostic (1.89e-1)
unprognostic (1.01e-1)	unprognostic (4.26e-2)	unprognostic (9.72e-2)	potential prognostic favorable (1.72e-4)	unprognostic (4.08e-1)	unprognostic (7.02e-2)
unprognostic (1.03e-2)	unprognostic (1.26e-1)	unprognostic (2.97e-2)	unprognostic (5.30e-2)	unprognostic (1.58e-2)	unprognostic (1.66e-1)
unprognostic (2.23e-1)	unprognostic (2.06e-1)	unprognostic (2.95e-1)	unprognostic (1.11e-1)	unprognostic (1.00e-2)	unprognostic (1.15e-1)
unprognostic (2.84e-1)	unprognostic (6.63e-2)	unprognostic (1.19e-1)	unprognostic (2.08e-1)	unprognostic (2.48e-1)	unprognostic (1.13e-3)
	unprognostic (1.65e-2)	unprognostic (2.89e-1)	unprognostic (2.10e-2)		unprognostic (1.52e-2)
unprognostic (9.48e-2)	unprognostic (9.42e-2)	unprognostic (1.44e-1)	unprognostic (2.07e-2)	unprognostic (3.73e-2)	unprognostic (2.68e-3)
unprognostic (2.50e-1)	unprognostic (1.83e-1)	unprognostic (5.47e-2)	unprognostic (2.76e-2)	unprognostic (3.29e-2)	unprognostic (6.84e-2)
unprognostic (1.21e-1)	unprognostic (8.89e-2)	unprognostic (2.12e-1)	unprognostic (1.09e-2)	unprognostic (3.00e-1)	unprognostic (3.57e-3)
unprognostic (2.27e-2)	unprognostic (5.12e-2)	unprognostic (6.52e-2)	potential prognostic unfavorable (1.88e-4)	unprognostic (2.01e-2)	unprognostic (2.71e-2)
unprognostic (2.18e-1)	unprognostic (5.98e-2)	unprognostic (1.83e-1)	unprognostic (6.04e-3)	unprognostic (1.58e-1)	unprognostic (2.82e-2)
unprognostic (5.86e-2)	unprognostic (1.33e-2)	unprognostic (1.73e-1)	unprognostic (1.21e-1)	unprognostic (3.98e-2)	
unprognostic (1.20e-1)	unprognostic (2.44e-2)	unprognostic (3.52e-3)	potential prognostic favorable (2.55e-4)	unprognostic (1.70e-1)	potential prognostic unfavorable (6.45e-4)
unprognostic (2.94e-2)	unprognostic (2.27e-2)	unprognostic (4.13e-2)	unprognostic (1.87e-2)	unprognostic (5.99e-3)	unprognostic (1.19e-1)
unprognostic (2.31e-3)	unprognostic (2.32e-1)	potential prognostic unfavorable (4.46e-4)	unprognostic (3.80e-2)	unprognostic (1.03e-2)	potential prognostic unfavorable (7.17e-7)
unprognostic (1.57e-3)	unprognostic (7.86e-2)	potential prognostic unfavorable (9.52e-4)	unprognostic (2.33e-1)	unprognostic (1.73e-2)	unprognostic (4.55e-3)
unprognostic (3.41e-3)	unprognostic (2.84e-3)	unprognostic (5.13e-2)	unprognostic (1.93e-1)	unprognostic (1.05e-1)	unprognostic (5.96e-2)
	unprognostic (7.76e-3)	unprognostic (7.10e-2)	potential prognostic favorable (7.21e-4)		
unprognostic (2.49e-1)	unprognostic (5.46e-2)	unprognostic (3.08e-1)	unprognostic (1.16e-2)	unprognostic (2.04e-1)	potential prognostic unfavorable (7.48e-6)
unprognostic (1.94e-2)	unprognostic (3.52e-2)	unprognostic (1.84e-2)	unprognostic (1.63e-2)	unprognostic (3.64e-1)	unprognostic (1.70e-1)
unprognostic (1.86e-2)	potential prognostic favorable (2.67e-4)	unprognostic (1.08e-1)	unprognostic (4.76e-3)	unprognostic (8.70e-2)	unprognostic (1.44e-2)
unprognostic (1.95e-1)	unprognostic (5.93e-2)	unprognostic (2.30e-1)	unprognostic (5.00e-2)	unprognostic (8.30e-3)	unprognostic (4.56e-2)
unprognostic (2.81e-2)	unprognostic (2.01e-1)	unprognostic (7.77e-2)	unprognostic (6.11e-3)	unprognostic (1.62e-2)	unprognostic (7.37e-2)
unprognostic (4.13e-1)	unprognostic (8.83e-3)		unprognostic (9.22e-3)	unprognostic (4.51e-2)	unprognostic (2.73e-1)
unprognostic (4.10e-1)	unprognostic (1.92e-1)	unprognostic (4.20e-2)	unprognostic (1.30e-3)	unprognostic (7.53e-2)	unprognostic (1.91e-1)
unprognostic (1.80e-1)	unprognostic (2.61e-1)	unprognostic (1.98e-2)	unprognostic (4.14e-3)	unprognostic (1.14e-2)	
		unprognostic (4.73e-3)	unprognostic (3.34e-1)		
unprognostic (3.70e-1)	unprognostic (1.46e-1)	unprognostic (6.53e-2)	unprognostic (1.49e-3)	unprognostic (2.89e-1)	unprognostic (2.52e-1)
unprognostic (1.64e-3)	unprognostic (6.59e-3)	unprognostic (9.17e-2)	unprognostic (6.57e-2)	unprognostic (4.09e-2)	unprognostic (1.04e-2)
unprognostic (1.28e-1)	unprognostic (4.01e-1)	unprognostic (1.01e-1)	unprognostic (7.42e-2)	unprognostic (4.23e-2)	unprognostic (1.17e-1)
unprognostic (5.11e-3)	unprognostic (2.48e-1)	unprognostic (7.78e-2)	potential prognostic unfavorable (7.20e-5)	unprognostic (2.40e-2)	unprognostic (1.18e-1)
unprognostic (2.12e-2)	unprognostic (9.74e-3)	unprognostic (4.38e-1)	unprognostic (2.72e-3)	unprognostic (2.11e-2)	unprognostic (5.50e-2)
unprognostic (2.70e-1)	unprognostic (2.86e-2)	unprognostic (3.80e-1)	unprognostic (1.62e-2)	unprognostic (1.31e-2)	unprognostic (6.03e-2)
unprognostic (1.51e-1)	unprognostic (1.53e-2)	unprognostic (7.51e-2)	unprognostic (2.69e-2)	unprognostic (3.06e-2)	unprognostic (1.17e-2)
unprognostic (1.29e-1)	unprognostic (2.22e-1)	unprognostic (1.24e-1)	unprognostic (1.87e-1)	unprognostic (6.70e-2)	unprognostic (2.86e-1)
unprognostic (4.11e-2)	unprognostic (2.76e-1)	unprognostic (4.30e-2)	unprognostic (2.82e-1)	unprognostic (6.87e-3)	unprognostic (8.98e-2)
unprognostic (5.52e-2)	unprognostic (2.86e-1)	unprognostic (7.60e-2)	unprognostic (2.79e-3)	unprognostic (1.32e-1)	unprognostic (4.69e-2)
unprognostic (1.49e-1)	unprognostic (2.26e-1)	unprognostic (2.69e-1)	unprognostic (1.80e-1)	unprognostic (1.95e-2)	unprognostic (4.14e-1)
unprognostic (4.12e-3)	unprognostic (4.53e-2)	unprognostic (1.05e-2)	potential prognostic unfavorable (6.97e-4)	unprognostic (1.90e-1)	unprognostic (2.22e-1)
unprognostic (6.36e-2)	unprognostic (8.71e-2)	unprognostic (4.76e-2)	unprognostic (1.46e-1)	unprognostic (5.38e-2)	unprognostic (7.40e-3)
unprognostic (2.57e-1)	unprognostic (1.38e-1)	unprognostic (3.87e-2)	unprognostic (1.13e-1)	unprognostic (6.11e-2)	unprognostic (1.17e-2)
	unprognostic (1.13e-1)	unprognostic (1.19e-1)	unprognostic (1.12e-1)		unprognostic (8.97e-3)
			unprognostic (2.25e-2)		
unprognostic (3.77e-2)	unprognostic (3.53e-2)	unprognostic (1.74e-2)	unprognostic (3.24e-2)	unprognostic (8.62e-2)	unprognostic (3.13e-1)
unprognostic (2.23e-1)	unprognostic (1.57e-2)	unprognostic (6.11e-2)	unprognostic (5.06e-3)	unprognostic (3.20e-2)	unprognostic (2.79e-1)
unprognostic (3.97e-1)	unprognostic (3.36e-1)	unprognostic (7.84e-2)	unprognostic (8.27e-3)	potential prognostic favorable (3.74e-4)	unprognostic (2.22e-2)
unprognostic (6.12e-2)	unprognostic (2.23e-1)	unprognostic (3.95e-1)	unprognostic (2.99e-1)	unprognostic (1.57e-3)	unprognostic (3.38e-1)
unprognostic (8.43e-2)	unprognostic (2.07e-3)	unprognostic (1.68e-1)	unprognostic (4.03e-1)		
unprognostic (1.81e-2)	unprognostic (1.92e-2)	unprognostic (3.29e-1)	unprognostic (2.31e-3)	unprognostic (1.59e-1)	unprognostic (5.30e-3)
unprognostic (1.02e-1)	unprognostic (1.68e-1)	unprognostic (1.63e-1)	unprognostic (2.99e-3)	unprognostic (1.98e-2)	unprognostic (1.80e-2)
unprognostic (4.82e-2)	unprognostic (1.32e-1)	unprognostic (5.71e-2)	unprognostic (7.24e-3)	unprognostic (1.96e-2)	unprognostic (1.45e-2)
unprognostic (2.15e-1)	unprognostic (1.10e-3)	unprognostic (3.24e-1)	unprognostic (5.70e-2)	unprognostic (1.58e-1)	unprognostic (3.54e-1)
unprognostic (1.47e-1)	unprognostic (6.65e-2)	unprognostic (1.45e-1)	unprognostic (2.06e-1)	unprognostic (3.39e-2)	unprognostic (1.18e-1)
unprognostic (7.53e-2)	unprognostic (2.46e-2)	unprognostic (2.62e-2)	unprognostic (2.10e-1)	unprognostic (3.09e-2)	unprognostic (3.06e-1)
unprognostic (2.67e-2)	unprognostic (6.24e-2)	unprognostic (3.42e-1)	unprognostic (2.00e-1)	unprognostic (3.83e-2)	
unprognostic (4.86e-3)	unprognostic (3.34e-2)	unprognostic (2.12e-1)	unprognostic (5.01e-3)	unprognostic (6.22e-2)	unprognostic (1.45e-1)
unprognostic (1.14e-2)	unprognostic (7.99e-2)	unprognostic (5.76e-2)	unprognostic (1.29e-1)	unprognostic (3.46e-2)	unprognostic (2.09e-2)
unprognostic (5.67e-2)	unprognostic (3.74e-2)	unprognostic (1.39e-1)	unprognostic (3.78e-2)	unprognostic (4.09e-3)	unprognostic (1.13e-3)
unprognostic (3.52e-2)	unprognostic (5.53e-2)	unprognostic (1.92e-1)	unprognostic (5.30e-2)	unprognostic (3.23e-2)	unprognostic (2.63e-2)
unprognostic (3.26e-1)	unprognostic (1.46e-1)	unprognostic (7.03e-3)	unprognostic (3.65e-1)	unprognostic (2.22e-1)	unprognostic (6.54e-2)
unprognostic (1.47e-1)	unprognostic (2.91e-2)	unprognostic (1.21e-1)	unprognostic (3.12e-2)	unprognostic (2.47e-2)	unprognostic (1.32e-1)
unprognostic (1.91e-1)	unprognostic (3.16e-2)	unprognostic (1.09e-1)	unprognostic (2.68e-2)	unprognostic (2.72e-2)	unprognostic (8.15e-2)
unprognostic (1.01e-1)	unprognostic (6.80e-3)	unprognostic (7.21e-3)	potential prognostic unfavorable (1.57e-4)	unprognostic (3.04e-1)	unprognostic (2.26e-2)
unprognostic (2.34e-1)	unprognostic (1.51e-2)	unprognostic (1.76e-1)	unprognostic (9.70e-3)	unprognostic (1.11e-1)	unprognostic (9.00e-2)
unprognostic (9.73e-2)	unprognostic (7.50e-2)	unprognostic (1.51e-2)	unprognostic (4.25e-3)	unprognostic (1.63e-2)	unprognostic (1.77e-2)
unprognostic (1.98e-1)	unprognostic (3.41e-2)	unprognostic (1.18e-2)	unprognostic (2.77e-3)	unprognostic (7.64e-2)	unprognostic (3.85e-2)
unprognostic (1.85e-2)	unprognostic (2.21e-1)	unprognostic (1.33e-1)	potential prognostic favorable (5.87e-5)	unprognostic (3.27e-2)	
		unprognostic (1.08e-1)	unprognostic (1.27e-1)		
		unprognostic (1.45e-1)	unprognostic (5.29e-2)		
			unprognostic (1.42e-2)		
			unprognostic (1.76e-1)		
		unprognostic (2.32e-2)	unprognostic (3.23e-2)		unprognostic (1.65e-1)
	unprognostic (1.05e-3)	unprognostic (1.36e-2)	unprognostic (1.28e-1)	unprognostic (1.12e-2)	
unprognostic (1.41e-1)	unprognostic (1.03e-1)	unprognostic (1.26e-2)	unprognostic (5.08e-2)	unprognostic (1.00e-3)	unprognostic (2.31e-3)
	unprognostic (5.66e-2)	unprognostic (6.17e-3)	unprognostic (2.93e-1)		
unprognostic (3.38e-1)	unprognostic (7.83e-3)	unprognostic (6.46e-2)	unprognostic (2.36e-2)	unprognostic (1.40e-1)	unprognostic (9.51e-3)
unprognostic (3.01e-1)	unprognostic (5.22e-2)	unprognostic (1.20e-1)	unprognostic (2.87e-1)	unprognostic (1.37e-1)	unprognostic (2.39e-2)
				unprognostic (6.00e-2)	
unprognostic (3.23e-1)	unprognostic (4.88e-2)	unprognostic (2.95e-1)	potential prognostic favorable (4.80e-5)	unprognostic (3.73e-2)	unprognostic (6.78e-2)
unprognostic (3.84e-1)	unprognostic (7.08e-2)	unprognostic (1.34e-1)	unprognostic (9.78e-2)	unprognostic (2.22e-1)	unprognostic (1.60e-1)
unprognostic (1.04e-1)	unprognostic (1.52e-1)	unprognostic (1.87e-2)	potential prognostic unfavorable (6.63e-4)	unprognostic (5.77e-2)	unprognostic (1.08e-1)
unprognostic (1.95e-1)	unprognostic (1.89e-1)			unprognostic (1.52e-2)	
unprognostic (1.23e-1)	unprognostic (2.43e-1)			unprognostic (1.45e-1)	
		unprognostic (2.25e-1)	unprognostic (1.95e-1)	unprognostic (1.60e-1)	
unprognostic (3.62e-2)	unprognostic (1.62e-1)	unprognostic (1.31e-1)	unprognostic (7.05e-2)	unprognostic (1.14e-1)	unprognostic (6.98e-2)
unprognostic (1.63e-1)	unprognostic (7.88e-2)	unprognostic (2.78e-1)	unprognostic (1.11e-3)		unprognostic (1.75e-1)
	unprognostic (7.91e-2)	unprognostic (1.10e-2)	unprognostic (2.92e-1)		unprognostic (1.30e-1)
unprognostic (8.69e-2)	unprognostic (3.25e-1)	unprognostic (3.57e-3)	unprognostic (2.86e-1)	unprognostic (5.56e-2)	unprognostic (5.67e-3)
	unprognostic (9.87e-2)				
unprognostic (1.18e-1)	unprognostic (1.69e-1)	unprognostic (2.88e-2)	unprognostic (1.20e-1)		
			unprognostic (4.32e-3)		
unprognostic (2.19e-1)	unprognostic (1.26e-1)	unprognostic (4.03e-1)	unprognostic (2.88e-2)	unprognostic (1.08e-1)	unprognostic (4.76e-2)
unprognostic (2.50e-1)	unprognostic (3.48e-3)	unprognostic (4.05e-1)	unprognostic (2.15e-1)	unprognostic (1.18e-1)	unprognostic (3.61e-1)
unprognostic (1.80e-1)	unprognostic (1.24e-2)	unprognostic (5.21e-2)	unprognostic (6.88e-2)	unprognostic (1.40e-2)	unprognostic (1.93e-1)
unprognostic (2.27e-1)	unprognostic (8.08e-3)	unprognostic (1.21e-2)	unprognostic (7.78e-2)	unprognostic (2.78e-1)	unprognostic (2.10e-2)
unprognostic (1.06e-2)	unprognostic (1.74e-2)	unprognostic (5.50e-2)	unprognostic (9.31e-2)	unprognostic (2.33e-2)	unprognostic (2.24e-1)
unprognostic (1.32e-1)	unprognostic (3.91e-2)	unprognostic (3.97e-1)	unprognostic (3.56e-1)	unprognostic (1.45e-1)	unprognostic (1.42e-2)
unprognostic (1.70e-1)	unprognostic (7.17e-2)	unprognostic (2.94e-3)	unprognostic (4.24e-2)	unprognostic (8.59e-3)	unprognostic (1.61e-1)
			unprognostic (4.16e-3)		
unprognostic (6.28e-2)	unprognostic (6.02e-2)	unprognostic (7.42e-2)	unprognostic (3.55e-1)	unprognostic (2.58e-1)	potential prognostic unfavorable (1.18e-5)
	unprognostic (2.31e-1)		unprognostic (1.80e-1)		
unprognostic (9.49e-2)	unprognostic (6.11e-2)	unprognostic (1.81e-1)	unprognostic (1.54e-1)	unprognostic (3.52e-2)	unprognostic (1.62e-1)

	unprognostic (1.09e-1)				
	unprognostic (7.16e-2)	unprognostic (1.55e-1)	unprognostic (8.65e-3)		
				unprognostic (3.88e-2)	
	unprognostic (2.55e-2)	unprognostic (5.93e-2)	unprognostic (4.50e-1)		
unprognostic (7.09e-2)	unprognostic (4.25e-2)	unprognostic (7.05e-3)	unprognostic (9.99e-2)	unprognostic (2.45e-1)	unprognostic (3.42e-2)
unprognostic (1.53e-1)	unprognostic (3.51e-1)	unprognostic (1.86e-2)	unprognostic (8.47e-3)	unprognostic (2.85e-2)	unprognostic (2.59e-2)
unprognostic (7.09e-2)	unprognostic (1.28e-1)	unprognostic (3.53e-2)	potential prognostic favorable (7.78e-5)	unprognostic (2.50e-1)	
unprognostic (1.93e-1)	unprognostic (1.37e-1)	unprognostic (1.04e-1)	unprognostic (2.44e-1)	unprognostic (2.08e-1)	unprognostic (3.31e-2)
unprognostic (8.97e-2)	unprognostic (7.37e-2)	unprognostic (1.87e-1)	unprognostic (1.23e-3)	unprognostic (8.09e-3)	unprognostic (1.73e-2)
unprognostic (5.01e-2)	unprognostic (7.86e-2)			unprognostic (3.53e-2)	unprognostic (8.56e-2)
		unprognostic (1.08e-3)	unprognostic (3.99e-3)		
		unprognostic (1.41e-1)	unprognostic (1.94e-2)		unprognostic (1.59e-1)
unprognostic (1.63e-1)	unprognostic (5.01e-2)		unprognostic (9.12e-2)		
unprognostic (3.32e-1)	potential prognostic unfavorable (2.36e-4)	unprognostic (1.65e-1)	unprognostic (2.02e-2)	unprognostic (9.99e-2)	unprognostic (4.81e-2)
unprognostic (1.45e-1)	unprognostic (1.15e-1)		unprognostic (1.19e-1)		
unprognostic (1.98e-1)	unprognostic (1.00e-2)	unprognostic (2.04e-1)	unprognostic (1.23e-1)	unprognostic (1.86e-1)	
unprognostic (2.10e-2)	unprognostic (9.93e-2)	unprognostic (1.19e-1)	unprognostic (3.71e-2)	unprognostic (6.60e-2)	potential prognostic unfavorable (1.69e-4)
unprognostic (5.64e-2)	unprognostic (7.49e-2)	unprognostic (9.16e-3)	unprognostic (2.81e-3)	unprognostic (3.00e-2)	unprognostic (2.40e-1)
unprognostic (3.34e-2)	unprognostic (6.31e-2)	unprognostic (5.50e-2)	unprognostic (4.36e-3)	unprognostic (6.77e-2)	unprognostic (1.03e-2)
				unprognostic (1.96e-1)	
unprognostic (2.60e-1)	unprognostic (3.55e-3)		unprognostic (6.03e-2)	unprognostic (2.11e-1)	unprognostic (3.73e-2)
unprognostic (1.32e-1)	unprognostic (5.35e-2)	unprognostic (4.34e-2)	unprognostic (1.70e-1)	unprognostic (1.86e-1)	unprognostic (4.12e-2)
unprognostic (1.24e-1)	unprognostic (1.74e-1)	unprognostic (1.80e-2)	unprognostic (3.99e-1)	unprognostic (8.08e-3)	unprognostic (6.90e-2)
unprognostic (6.18e-2)	unprognostic (1.08e-1)	unprognostic (6.94e-3)	potential prognostic unfavorable (7.65e-7)	unprognostic (6.50e-2)	unprognostic (9.88e-2)
unprognostic (1.90e-1)	unprognostic (2.55e-2)	unprognostic (3.43e-2)	unprognostic (3.00e-1)	unprognostic (2.25e-1)	unprognostic (2.70e-3)
unprognostic (1.64e-1)	unprognostic (4.34e-1)	unprognostic (5.96e-2)	unprognostic (1.65e-1)	unprognostic (9.00e-2)	unprognostic (2.62e-1)
unprognostic (1.02e-2)	unprognostic (1.80e-1)	unprognostic (3.49e-2)	unprognostic (1.12e-1)	unprognostic (8.91e-2)	unprognostic (3.55e-2)
unprognostic (5.20e-2)	unprognostic (1.14e-1)	unprognostic (5.08e-2)	unprognostic (7.53e-2)	unprognostic (2.88e-2)	unprognostic (9.11e-3)
unprognostic (4.00e-3)	unprognostic (4.85e-2)	unprognostic (3.42e-1)	unprognostic (2.15e-2)	unprognostic (3.93e-2)	unprognostic (1.28e-1)
unprognostic (4.87e-2)	unprognostic (8.40e-2)	unprognostic (1.89e-2)	unprognostic (1.07e-2)	unprognostic (3.55e-2)	unprognostic (1.74e-3)
unprognostic (3.94e-2)	unprognostic (1.18e-2)	unprognostic (1.39e-1)	unprognostic (3.45e-2)	potential prognostic unfavorable (2.78e-5)	unprognostic (4.53e-1)
unprognostic (2.39e-2)	unprognostic (1.28e-2)	unprognostic (3.57e-3)	potential prognostic unfavorable (8.42e-4)	unprognostic (1.37e-2)	unprognostic (1.43e-2)
			unprognostic (8.63e-2)	unprognostic (1.48e-2)	
unprognostic (2.62e-2)	unprognostic (2.07e-2)	unprognostic (2.71e-2)	unprognostic (8.97e-2)	unprognostic (3.88e-3)	unprognostic (2.44e-2)
unprognostic (5.82e-3)	unprognostic (1.88e-1)	unprognostic (6.47e-2)	potential prognostic unfavorable (1.65e-4)	unprognostic (1.72e-2)	unprognostic (2.71e-3)
unprognostic (1.14e-1)	unprognostic (6.36e-2)	unprognostic (2.51e-1)	unprognostic (1.03e-2)	unprognostic (8.44e-3)	unprognostic (3.95e-2)
unprognostic (3.61e-2)	unprognostic (4.54e-2)	unprognostic (1.20e-2)	unprognostic (3.54e-2)	unprognostic (2.70e-1)	unprognostic (1.21e-1)
unprognostic (7.64e-2)	unprognostic (1.14e-1)	unprognostic (3.86e-2)	unprognostic (3.67e-3)	unprognostic (2.52e-2)	unprognostic (2.72e-1)
unprognostic (1.38e-1)	unprognostic (7.76e-2)	unprognostic (7.24e-2)	unprognostic (9.56e-2)	unprognostic (3.54e-1)	unprognostic (8.98e-2)
unprognostic (2.36e-2)	unprognostic (8.76e-2)	unprognostic (2.36e-1)	unprognostic (3.26e-1)	unprognostic (5.61e-2)	unprognostic (8.62e-3)
unprognostic (2.57e-2)	potential prognostic unfavorable (4.03e-5)	unprognostic (1.43e-1)	unprognostic (9.27e-3)	unprognostic (4.46e-2)	unprognostic (3.08e-1)
unprognostic (2.65e-1)	unprognostic (1.90e-1)	unprognostic (1.55e-3)	unprognostic (3.56e-3)	potential prognostic favorable (6.66e-5)	unprognostic (2.49e-2)
unprognostic (1.01e-1)	unprognostic (3.13e-1)	unprognostic (6.19e-2)	unprognostic (2.84e-2)	unprognostic (5.03e-3)	unprognostic (3.33e-1)
unprognostic (1.58e-2)	unprognostic (1.23e-1)	unprognostic (1.63e-2)	unprognostic (1.89e-2)	unprognostic (5.20e-2)	unprognostic (2.27e-1)
unprognostic (4.95e-3)	unprognostic (3.54e-2)	unprognostic (8.45e-3)	unprognostic (6.06e-3)	unprognostic (3.28e-2)	unprognostic (1.49e-1)
	unprognostic (4.38e-2)		unprognostic (7.39e-3)	unprognostic (1.09e-2)	
unprognostic (2.87e-1)	unprognostic (4.46e-2)	unprognostic (1.29e-2)	unprognostic (1.05e-1)	unprognostic (1.13e-3)	unprognostic (3.08e-1)
unprognostic (2.15e-1)	unprognostic (6.86e-2)	unprognostic (1.02e-2)	unprognostic (3.53e-1)	unprognostic (6.16e-2)	unprognostic (1.86e-1)
unprognostic (3.44e-1)	unprognostic (5.93e-3)	unprognostic (1.49e-1)	potential prognostic unfavorable (3.79e-5)	unprognostic (1.62e-2)	unprognostic (1.27e-2)
unprognostic (9.97e-2)	unprognostic (3.73e-1)	unprognostic (3.58e-2)	unprognostic (1.17e-2)	unprognostic (2.53e-1)	unprognostic (1.38e-1)
unprognostic (1.17e-1)	unprognostic (2.26e-1)	unprognostic (6.83e-2)	unprognostic (2.73e-3)	unprognostic (1.50e-1)	unprognostic (2.12e-3)
unprognostic (2.90e-1)	unprognostic (1.21e-1)	unprognostic (2.35e-1)	unprognostic (3.65e-1)	unprognostic (7.32e-2)	unprognostic (7.23e-2)
unprognostic (5.74e-3)	unprognostic (1.35e-1)	unprognostic (4.84e-3)	unprognostic (2.05e-3)	potential prognostic unfavorable (1.60e-4)	unprognostic (2.19e-1)
unprognostic (8.23e-2)	unprognostic (5.35e-2)	unprognostic (9.77e-2)	unprognostic (9.55e-2)	unprognostic (8.97e-2)	unprognostic (1.44e-1)
unprognostic (2.25e-1)	unprognostic (3.28e-2)	unprognostic (7.99e-2)	unprognostic (3.49e-2)	unprognostic (1.17e-2)	unprognostic (2.98e-3)
unprognostic (8.94e-2)	unprognostic (1.28e-1)	unprognostic (5.59e-3)	unprognostic (2.22e-1)	unprognostic (8.19e-2)	unprognostic (3.35e-1)
unprognostic (2.03e-1)	unprognostic (1.00e-1)	unprognostic (1.08e-2)	unprognostic (2.19e-2)	unprognostic (6.47e-3)	potential prognostic unfavorable (6.40e-4)
unprognostic (1.69e-1)	unprognostic (3.28e-1)	unprognostic (9.55e-3)	unprognostic (2.98e-2)	unprognostic (2.32e-2)	unprognostic (1.44e-1)
unprognostic (1.62e-1)	unprognostic (5.71e-2)	unprognostic (1.42e-1)	unprognostic (1.51e-1)	unprognostic (9.98e-2)	unprognostic (2.27e-2)
unprognostic (9.88e-2)	unprognostic (1.73e-1)				unprognostic (1.14e-1)
unprognostic (5.18e-2)	unprognostic (1.88e-1)	unprognostic (1.16e-1)	unprognostic (4.36e-1)	unprognostic (2.46e-2)	unprognostic (5.39e-2)
unprognostic (8.83e-2)	unprognostic (1.54e-1)	potential prognostic unfavorable (3.75e-5)	unprognostic (2.31e-1)	potential prognostic favorable (6.22e-4)	unprognostic (4.34e-2)
unprognostic (1.12e-1)	unprognostic (6.25e-2)	unprognostic (4.77e-2)	unprognostic (2.88e-2)	unprognostic (9.80e-2)	unprognostic (1.36e-1)
unprognostic (6.86e-2)	unprognostic (1.08e-1)	unprognostic (2.15e-1)	unprognostic (1.11e-1)	unprognostic (1.26e-1)	unprognostic (3.47e-1)
unprognostic (1.22e-1)	unprognostic (1.44e-1)	unprognostic (9.16e-2)	unprognostic (5.67e-2)	unprognostic (2.24e-1)	unprognostic (1.90e-3)
unprognostic (1.63e-1)	unprognostic (1.99e-1)	unprognostic (4.85e-2)	unprognostic (2.25e-2)	potential prognostic unfavorable (1.85e-5)	unprognostic (1.54e-2)
		unprognostic (2.27e-1)	unprognostic (4.51e-2)		
	unprognostic (1.75e-1)	unprognostic (3.96e-2)	unprognostic (1.24e-1)		unprognostic (6.26e-2)
unprognostic (2.87e-2)	unprognostic (1.96e-2)	unprognostic (1.27e-1)	unprognostic (2.31e-1)	unprognostic (1.58e-1)	unprognostic (3.71e-2)
unprognostic (2.26e-2)	unprognostic (5.30e-2)	unprognostic (1.29e-1)	unprognostic (1.63e-3)		
unprognostic (7.19e-2)	unprognostic (2.16e-1)	unprognostic (3.79e-3)	unprognostic (2.44e-1)	unprognostic (1.30e-2)	unprognostic (9.87e-2)
unprognostic (1.75e-1)	unprognostic (4.83e-2)	unprognostic (2.19e-1)	unprognostic (1.57e-1)	unprognostic (1.59e-1)	unprognostic (9.76e-2)
		unprognostic (6.35e-3)	unprognostic (2.08e-3)	unprognostic (1.14e-2)	
unprognostic (3.59e-1)	unprognostic (8.00e-3)	unprognostic (3.61e-1)	unprognostic (2.67e-2)	unprognostic (1.78e-2)	unprognostic (1.59e-2)
			unprognostic (1.44e-1)		
	unprognostic (4.31e-3)		unprognostic (7.87e-2)		
			unprognostic (1.98e-2)		
unprognostic (6.35e-2)	unprognostic (1.48e-2)	unprognostic (1.85e-1)	unprognostic (4.77e-2)	unprognostic (2.53e-2)	unprognostic (6.75e-2)
		unprognostic (8.51e-3)			
unprognostic (2.82e-1)	unprognostic (4.48e-2)	unprognostic (1.51e-1)	unprognostic (1.08e-1)	unprognostic (4.25e-2)	unprognostic (1.60e-1)
unprognostic (1.95e-1)	unprognostic (1.00e-2)	unprognostic (2.35e-1)	unprognostic (6.31e-2)	potential prognostic unfavorable (5.87e-4)	unprognostic (1.85e-3)
unprognostic (8.42e-2)	potential prognostic favorable (1.42e-4)			unprognostic (1.94e-1)	
unprognostic (8.77e-2)	unprognostic (1.17e-1)	unprognostic (4.78e-2)	unprognostic (1.87e-3)	unprognostic (4.21e-3)	unprognostic (2.92e-1)
unprognostic (1.88e-1)	unprognostic (5.01e-2)	unprognostic (3.49e-2)	unprognostic (3.43e-2)	unprognostic (8.82e-3)	unprognostic (3.16e-2)
	unprognostic (6.56e-3)	unprognostic (1.05e-1)	unprognostic (2.84e-1)		unprognostic (1.31e-1)
unprognostic (3.07e-3)	unprognostic (1.31e-3)	unprognostic (4.73e-2)	potential prognostic unfavorable (2.14e-4)	unprognostic (3.30e-2)	potential prognostic unfavorable (8.29e-4)
unprognostic (1.09e-1)	unprognostic (2.94e-1)	unprognostic (4.67e-2)	unprognostic (1.17e-1)	unprognostic (6.45e-3)	unprognostic (6.22e-2)
unprognostic (1.67e-2)	unprognostic (1.77e-1)	unprognostic (1.48e-1)		potential prognostic favorable (6.26e-4)	unprognostic (1.89e-2)
unprognostic (1.19e-2)	unprognostic (4.13e-1)	unprognostic (4.48e-1)	unprognostic (7.11e-2)	unprognostic (1.36e-2)	unprognostic (4.28e-1)
unprognostic (2.67e-1)	unprognostic (5.91e-2)		unprognostic (6.64e-2)	potential prognostic favorable (1.45e-4)	
unprognostic (1.71e-2)	unprognostic (1.20e-1)	unprognostic (5.10e-2)	unprognostic (1.14e-1)	unprognostic (5.92e-2)	unprognostic (5.42e-2)
unprognostic (1.17e-2)	unprognostic (4.71e-2)	unprognostic (2.98e-1)	unprognostic (1.97e-2)	unprognostic (1.83e-1)	unprognostic (5.06e-2)
unprognostic (9.97e-2)	unprognostic (6.54e-2)	unprognostic (2.06e-1)	unprognostic (1.57e-3)	unprognostic (2.60e-1)	unprognostic (1.38e-1)
unprognostic (4.19e-2)	unprognostic (1.46e-1)	unprognostic (1.19e-3)	unprognostic (3.25e-3)	unprognostic (3.52e-2)	unprognostic (1.77e-1)
unprognostic (7.21e-2)	unprognostic (1.32e-1)	unprognostic (4.98e-1)	unprognostic (2.15e-1)	unprognostic (2.45e-1)	unprognostic (1.44e-2)
	unprognostic (2.35e-2)			unprognostic (5.63e-3)	
		unprognostic (1.11e-1)	unprognostic (4.56e-3)		
unprognostic (1.84e-1)	unprognostic (7.77e-2)	unprognostic (4.04e-2)	unprognostic (1.78e-1)	unprognostic (2.33e-2)	unprognostic (4.72e-1)
unprognostic (3.77e-1)	unprognostic (1.12e-2)	unprognostic (2.13e-1)	unprognostic (1.37e-1)	unprognostic (1.97e-1)	unprognostic (7.63e-3)
			unprognostic (3.88e-3)		
unprognostic (1.36e-1)	unprognostic (2.56e-1)	unprognostic (2.17e-1)	unprognostic (5.13e-3)	unprognostic (1.74e-1)	unprognostic (1.05e-1)
unprognostic (1.70e-2)	unprognostic (9.35e-3)				
unprognostic (5.78e-3)	unprognostic (4.23e-2)	unprognostic (1.42e-1)	unprognostic (1.19e-1)	unprognostic (3.05e-2)	unprognostic (3.24e-1)
unprognostic (1.97e-1)	unprognostic (7.29e-2)	unprognostic (1.87e-1)	unprognostic (3.94e-2)	unprognostic (1.20e-2)	unprognostic (1.45e-1)
	unprognostic (2.15e-1)			unprognostic (5.72e-2)	
unprognostic (5.89e-2)	unprognostic (1.59e-1)	unprognostic (3.06e-1)	unprognostic (5.10e-2)	unprognostic (2.39e-1)	unprognostic (2.74e-3)
unprognostic (2.75e-1)	unprognostic (3.92e-1)	unprognostic (7.24e-2)	unprognostic (1.59e-1)	unprognostic (2.13e-1)	unprognostic (2.04e-1)
unprognostic (2.32e-2)	unprognostic (1.15e-2)	unprognostic (1.42e-1)	unprognostic (2.38e-2)	unprognostic (1.62e-1)	potential prognostic unfavorable (1.45e-4)
unprognostic (6.51e-2)	unprognostic (1.35e-1)	unprognostic (2.13e-1)	unprognostic (1.18e-2)	unprognostic (1.67e-2)	unprognostic (9.66e-2)
potential prognostic favorable (8.00e-4)	unprognostic (1.86e-1)	unprognostic (4.20e-2)	unprognostic (3.31e-3)	unprognostic (2.79e-3)	unprognostic (3.26e-2)
unprognostic (6.40e-2)	unprognostic (3.65e-1)	unprognostic (6.05e-3)	unprognostic (1.65e-1)	unprognostic (1.96e-3)	unprognostic (4.30e-2)
unprognostic (7.22e-2)	unprognostic (4.36e-2)	unprognostic (1.62e-1)	unprognostic (3.48e-2)	unprognostic (3.03e-1)	unprognostic (7.73e-3)
unprognostic (1.49e-1)	unprognostic (3.97e-3)	unprognostic (8.00e-2)	unprognostic (3.19e-3)	unprognostic (2.90e-1)	unprognostic (6.06e-2)
		unprognostic (2.85e-1)	potential prognostic favorable (6.46e-4)		unprognostic (1.10e-1)
unprognostic (7.71e-2)	unprognostic (2.32e-1)	unprognostic (5.78e-2)		unprognostic (1.94e-2)	unprognostic (9.95e-3)
unprognostic (1.81e-1)	unprognostic (4.25e-2)	unprognostic (1.24e-1)	unprognostic (1.88e-1)	unprognostic (1.80e-1)	unprognostic (5.27e-2)
unprognostic (5.19e-2)	unprognostic (7.34e-2)		unprognostic (2.40e-2)	unprognostic (2.21e-1)	unprognostic (1.03e-1)
			unprognostic (6.07e-2)		
unprognostic (4.26e-2)	unprognostic (1.25e-1)	unprognostic (1.22e-1)	unprognostic (2.05e-1)	unprognostic (1.52e-1)	unprognostic (2.98e-2)
unprognostic (2.73e-1)	unprognostic (2.53e-1)	potential prognostic unfavorable (5.33e-4)			
			potential prognostic favorable (1.68e-4)		

unprognostic (1.18e-1)	unprognostic (6.11e-2)	unprognostic (1.65e-1)	unprognostic (6.66e-2)	unprognostic (1.17e-1)	potential prognostic unfavorable (4.86e-4)
	unprognostic (3.46e-1)	unprognostic (6.81e-3)	unprognostic (2.04e-1)	unprognostic (2.59e-1)	unprognostic (1.27e-2)
	unprognostic (7.84e-2)		unprognostic (1.07e-1)		
		unprognostic (5.66e-2)	potential prognostic unfavorable (6.09e-4)	unprognostic (6.15e-2)	
unprognostic (1.92e-2)	unprognostic (1.05e-1)	unprognostic (6.18e-2)	unprognostic (6.39e-2)	unprognostic (1.50e-1)	unprognostic (1.20e-3)
unprognostic (1.99e-2)	unprognostic (1.38e-2)	unprognostic (1.52e-1)	unprognostic (1.15e-1)	unprognostic (4.79e-2)	unprognostic (2.13e-1)
potential prognostic favorable (1.75e-4)	unprognostic (7.18e-2)	unprognostic (1.14e-1)	unprognostic (2.27e-1)	unprognostic (5.91e-2)	
unprognostic (5.76e-2)	unprognostic (1.88e-1)	unprognostic (3.92e-1)	unprognostic (1.57e-1)	unprognostic (9.41e-2)	unprognostic (1.20e-2)
unprognostic (3.98e-1)	unprognostic (4.10e-2)	unprognostic (2.71e-2)	unprognostic (1.08e-1)	unprognostic (3.25e-2)	unprognostic (6.63e-2)
unprognostic (1.02e-1)	unprognostic (1.89e-1)			unprognostic (6.83e-2)	
unprognostic (1.84e-1)	unprognostic (4.88e-2)	unprognostic (6.53e-3)	unprognostic (1.11e-1)	unprognostic (4.82e-1)	unprognostic (1.39e-1)
unprognostic (8.46e-2)	unprognostic (2.60e-2)		unprognostic (3.20e-2)	unprognostic (1.01e-2)	unprognostic (1.31e-2)
unprognostic (1.00e-1)	unprognostic (1.21e-1)	unprognostic (3.16e-1)	unprognostic (6.46e-2)	potential prognostic favorable (9.34e-5)	
	unprognostic (6.27e-2)				
unprognostic (9.94e-2)	unprognostic (2.23e-1)	unprognostic (8.35e-2)	unprognostic (2.52e-1)	unprognostic (3.34e-3)	unprognostic (1.78e-1)
unprognostic (7.48e-2)	potential prognostic favorable (3.01e-4)		unprognostic (1.80e-1)	unprognostic (7.99e-2)	unprognostic (2.21e-2)
unprognostic (5.92e-2)	unprognostic (2.29e-1)	unprognostic (1.69e-2)	unprognostic (3.54e-1)	unprognostic (2.07e-1)	unprognostic (4.77e-3)
unprognostic (2.36e-1)	unprognostic (1.43e-1)	unprognostic (1.56e-2)	unprognostic (1.52e-2)	unprognostic (1.35e-2)	unprognostic (4.72e-3)
unprognostic (8.22e-2)	unprognostic (1.04e-1)	unprognostic (3.68e-2)	unprognostic (8.10e-2)	unprognostic (8.21e-3)	unprognostic (1.65e-1)
unprognostic (1.25e-1)	unprognostic (9.81e-2)			potential prognostic favorable (9.96e-7)	
unprognostic (9.28e-2)	unprognostic (1.09e-1)	unprognostic (4.96e-2)		unprognostic (4.27e-2)	unprognostic (1.84e-1)
unprognostic (2.03e-1)	unprognostic (4.24e-2)	unprognostic (1.31e-2)	potential prognostic unfavorable (1.26e-5)	unprognostic (1.18e-1)	unprognostic (2.12e-1)
unprognostic (2.16e-2)	unprognostic (2.81e-1)	unprognostic (1.96e-1)	unprognostic (1.74e-1)	unprognostic (5.41e-2)	unprognostic (2.35e-2)
unprognostic (1.99e-1)	unprognostic (9.53e-3)	unprognostic (5.63e-2)	unprognostic (9.41e-2)	unprognostic (9.23e-2)	potential prognostic favorable (8.45e-4)
unprognostic (1.49e-1)	unprognostic (2.04e-2)	unprognostic (9.34e-2)	unprognostic (2.72e-2)	unprognostic (1.40e-2)	unprognostic (9.54e-2)
unprognostic (5.76e-2)	unprognostic (3.12e-2)	unprognostic (1.31e-1)	unprognostic (1.58e-1)	unprognostic (4.12e-2)	unprognostic (2.72e-3)
unprognostic (3.24e-1)	unprognostic (9.87e-2)	unprognostic (3.28e-2)	unprognostic (7.08e-2)	unprognostic (1.15e-1)	unprognostic (1.40e-2)
unprognostic (1.06e-1)	unprognostic (8.94e-2)	unprognostic (4.13e-1)	unprognostic (9.79e-2)	potential prognostic unfavorable (4.45e-4)	unprognostic (1.65e-1)
unprognostic (1.20e-1)	unprognostic (9.54e-2)	unprognostic (1.06e-2)	unprognostic (1.06e-2)	unprognostic (4.00e-2)	unprognostic (3.74e-2)
unprognostic (2.21e-1)	unprognostic (4.31e-1)	unprognostic (2.97e-3)	unprognostic (2.68e-1)	potential prognostic favorable (5.46e-4)	
unprognostic (3.56e-1)	unprognostic (3.64e-2)	unprognostic (1.12e-1)	unprognostic (6.23e-3)	unprognostic (9.08e-2)	unprognostic (4.73e-1)
	unprognostic (7.49e-3)	unprognostic (4.22e-2)	unprognostic (3.31e-3)		unprognostic (5.87e-2)
		unprognostic (2.26e-1)	unprognostic (1.79e-1)	unprognostic (2.77e-3)	unprognostic (9.70e-2)
unprognostic (4.02e-3)	unprognostic (1.29e-2)	unprognostic (3.04e-1)	unprognostic (2.13e-3)	unprognostic (2.22e-2)	unprognostic (2.83e-1)
unprognostic (3.55e-2)	unprognostic (8.34e-2)	unprognostic (2.63e-1)	potential prognostic favorable (1.51e-4)	unprognostic (5.08e-3)	unprognostic (3.00e-1)
	unprognostic (1.38e-1)	unprognostic (1.41e-1)	potential prognostic favorable (6.43e-4)		
unprognostic (4.53e-2)	unprognostic (2.32e-1)	unprognostic (1.10e-2)	unprognostic (1.07e-1)	unprognostic (5.83e-3)	unprognostic (2.90e-1)
unprognostic (9.55e-3)	unprognostic (1.83e-1)	unprognostic (2.70e-1)	unprognostic (2.50e-3)	unprognostic (1.02e-1)	unprognostic (1.71e-1)
unprognostic (1.77e-1)	unprognostic (9.91e-2)			unprognostic (8.36e-2)	unprognostic (1.83e-1)
unprognostic (1.59e-2)	unprognostic (2.77e-1)	unprognostic (1.97e-1)	unprognostic (1.12e-1)	unprognostic (3.34e-2)	unprognostic (1.58e-1)
	unprognostic (6.98e-2)		unprognostic (1.27e-1)		unprognostic (4.91e-3)
unprognostic (6.12e-2)	unprognostic (1.98e-1)	unprognostic (2.38e-2)	unprognostic (1.38e-1)	unprognostic (5.80e-2)	unprognostic (1.44e-2)
unprognostic (1.60e-2)	unprognostic (3.34e-1)	unprognostic (2.26e-1)	unprognostic (1.17e-2)	unprognostic (1.60e-1)	unprognostic (9.40e-2)
unprognostic (2.03e-2)	unprognostic (4.75e-2)	unprognostic (1.21e-2)	unprognostic (9.19e-2)	unprognostic (1.91e-1)	unprognostic (1.65e-1)
unprognostic (2.78e-2)	unprognostic (3.57e-1)	unprognostic (2.78e-1)	unprognostic (2.79e-2)	unprognostic (9.77e-2)	unprognostic (1.53e-1)
unprognostic (1.05e-1)	unprognostic (1.20e-3)	unprognostic (2.69e-2)	unprognostic (1.51e-1)	unprognostic (1.52e-1)	unprognostic (1.98e-1)
unprognostic (1.57e-1)	unprognostic (2.15e-1)	unprognostic (9.68e-2)	unprognostic (1.49e-1)	unprognostic (2.50e-1)	unprognostic (6.38e-2)
unprognostic (5.77e-2)	unprognostic (1.14e-1)	unprognostic (6.53e-3)	unprognostic (3.31e-2)	potential prognostic favorable (6.80e-4)	unprognostic (1.02e-2)
		unprognostic (9.34e-2)	unprognostic (1.69e-1)		
			unprognostic (2.53e-1)		
unprognostic (5.93e-2)	unprognostic (3.99e-1)	unprognostic (3.98e-2)	potential prognostic favorable (3.13e-5)	unprognostic (3.53e-1)	unprognostic (1.87e-1)
	unprognostic (3.24e-2)				
			unprognostic (8.60e-2)	unprognostic (2.45e-1)	
unprognostic (1.24e-1)	unprognostic (4.68e-2)	unprognostic (7.39e-2)	unprognostic (2.84e-1)	unprognostic (5.64e-2)	unprognostic (4.21e-2)
unprognostic (1.58e-3)	unprognostic (8.11e-2)	unprognostic (1.38e-1)	unprognostic (6.16e-3)	unprognostic (4.17e-3)	unprognostic (1.10e-2)
unprognostic (9.31e-3)	unprognostic (3.69e-1)	unprognostic (9.12e-2)	unprognostic (4.56e-1)	unprognostic (3.75e-1)	potential prognostic unfavorable (1.11e-6)
unprognostic (6.64e-2)	unprognostic (2.23e-2)	unprognostic (3.71e-1)	unprognostic (1.15e-2)	unprognostic (3.75e-3)	unprognostic (8.83e-2)
		unprognostic (7.07e-3)	unprognostic (3.22e-1)		
	unprognostic (1.68e-1)	unprognostic (2.85e-1)	potential prognostic favorable (1.34e-4)		
unprognostic (4.97e-2)	unprognostic (6.57e-2)	unprognostic (1.93e-1)	unprognostic (6.15e-2)	unprognostic (3.51e-2)	
	unprognostic (1.42e-1)				
unprognostic (7.06e-2)	unprognostic (4.25e-2)	unprognostic (2.96e-1)	unprognostic (1.02e-1)	unprognostic (2.90e-1)	unprognostic (7.76e-3)
unprognostic (5.38e-2)	unprognostic (4.71e-2)	unprognostic (6.53e-2)	unprognostic (2.35e-1)	unprognostic (3.16e-2)	unprognostic (2.11e-1)
unprognostic (6.48e-2)	unprognostic (2.73e-2)	unprognostic (1.47e-2)	unprognostic (4.18e-3)	potential prognostic favorable (1.00e-3)	unprognostic (7.74e-3)
unprognostic (8.87e-2)	unprognostic (8.59e-3)		unprognostic (5.80e-2)	unprognostic (1.47e-1)	
unprognostic (1.32e-2)	unprognostic (1.80e-1)	unprognostic (8.42e-2)	unprognostic (8.80e-2)	potential prognostic unfavorable (3.53e-4)	unprognostic (2.82e-1)
unprognostic (4.10e-2)	unprognostic (9.94e-2)	potential prognostic unfavorable (3.04e-4)	unprognostic (5.57e-2)	unprognostic (4.05e-2)	unprognostic (2.82e-1)
			unprognostic (2.87e-1)		
	unprognostic (7.17e-2)	unprognostic (4.48e-2)	unprognostic (1.82e-1)	unprognostic (4.14e-2)	unprognostic (1.65e-2)
unprognostic (1.64e-1)	unprognostic (3.03e-1)	unprognostic (2.95e-2)	unprognostic (1.45e-3)	unprognostic (3.30e-2)	unprognostic (3.30e-3)
unprognostic (3.13e-1)	unprognostic (7.17e-2)				unprognostic (1.85e-2)
unprognostic (2.92e-2)	unprognostic (1.66e-3)	unprognostic (1.11e-2)	unprognostic (3.95e-2)	unprognostic (1.82e-1)	unprognostic (4.21e-1)
unprognostic (8.08e-2)	unprognostic (1.92e-1)	unprognostic (1.91e-1)	unprognostic (5.23e-2)	unprognostic (3.38e-2)	unprognostic (6.91e-3)
unprognostic (2.81e-1)	unprognostic (2.95e-1)	unprognostic (1.02e-1)	unprognostic (5.30e-2)	unprognostic (2.99e-1)	potential prognostic unfavorable (1.80e-6)
unprognostic (3.92e-2)	unprognostic (2.59e-1)	potential prognostic unfavorable (4.26e-4)	unprognostic (1.35e-1)	unprognostic (2.81e-1)	unprognostic (3.79e-3)
unprognostic (1.28e-1)	unprognostic (1.52e-1)	unprognostic (2.53e-1)	unprognostic (8.15e-2)	unprognostic (6.93e-2)	unprognostic (1.94e-1)
unprognostic (3.90e-2)	unprognostic (6.92e-2)	unprognostic (8.48e-2)	unprognostic (2.12e-2)	unprognostic (1.34e-2)	unprognostic (1.06e-3)
unprognostic (2.22e-1)	unprognostic (2.25e-2)	unprognostic (1.63e-1)		unprognostic (6.42e-2)	unprognostic (4.18e-2)
unprognostic (3.01e-2)	unprognostic (4.52e-2)	unprognostic (1.19e-1)	unprognostic (2.73e-1)	unprognostic (5.81e-2)	unprognostic (2.08e-2)
unprognostic (5.24e-2)	unprognostic (3.15e-1)	unprognostic (1.82e-1)	unprognostic (8.61e-3)	unprognostic (4.72e-2)	unprognostic (7.59e-2)
		unprognostic (7.46e-3)	unprognostic (2.90e-2)	unprognostic (1.60e-2)	unprognostic (3.71e-1)
unprognostic (1.05e-2)	unprognostic (6.76e-2)	unprognostic (2.94e-1)	unprognostic (2.20e-1)	unprognostic (4.50e-3)	unprognostic (6.79e-3)
unprognostic (8.35e-2)	unprognostic (4.63e-2)	unprognostic (1.74e-2)	potential prognostic favorable (2.23e-6)	unprognostic (1.20e-1)	unprognostic (5.70e-3)
unprognostic (4.85e-2)	unprognostic (4.97e-2)	unprognostic (2.42e-2)	unprognostic (1.79e-2)	unprognostic (1.77e-1)	unprognostic (8.06e-2)
unprognostic (1.92e-2)	unprognostic (1.13e-1)	unprognostic (6.88e-2)	unprognostic (1.60e-2)	unprognostic (4.76e-3)	unprognostic (9.84e-2)
unprognostic (1.56e-1)	unprognostic (1.30e-2)	unprognostic (2.67e-3)	potential prognostic unfavorable (9.06e-4)	unprognostic (3.54e-3)	unprognostic (1.70e-1)
unprognostic (3.12e-1)	unprognostic (4.59e-1)	unprognostic (2.10e-1)	unprognostic (9.60e-3)	unprognostic (6.40e-2)	unprognostic (5.91e-2)
unprognostic (6.34e-2)	unprognostic (5.67e-2)	unprognostic (7.04e-2)	unprognostic (1.06e-1)	unprognostic (6.31e-2)	unprognostic (2.25e-1)
unprognostic (3.98e-2)	unprognostic (1.42e-1)	unprognostic (9.88e-2)	potential prognostic favorable (2.32e-5)	unprognostic (1.96e-1)	unprognostic (7.15e-2)
	unprognostic (6.56e-2)	unprognostic (3.76e-1)	unprognostic (1.52e-1)		
unprognostic (2.27e-1)	unprognostic (1.61e-2)	unprognostic (4.34e-3)	unprognostic (1.66e-1)	unprognostic (1.61e-2)	
unprognostic (7.59e-2)	unprognostic (1.35e-2)	unprognostic (1.28e-2)	unprognostic (3.33e-2)	unprognostic (3.15e-2)	potential prognostic unfavorable (4.71e-5)
unprognostic (6.44e-2)	unprognostic (1.88e-2)	unprognostic (5.99e-2)	unprognostic (5.61e-2)	unprognostic (5.69e-2)	unprognostic (8.71e-2)
unprognostic (2.29e-2)	unprognostic (5.48e-2)	unprognostic (3.21e-2)	unprognostic (1.51e-1)	unprognostic (9.15e-2)	potential prognostic unfavorable (4.19e-4)
unprognostic (2.84e-2)	unprognostic (1.10e-1)	unprognostic (1.69e-1)	unprognostic (1.43e-1)	unprognostic (1.39e-1)	unprognostic (3.17e-1)
unprognostic (3.65e-1)	unprognostic (1.20e-1)	unprognostic (3.12e-1)	unprognostic (2.81e-2)	unprognostic (2.62e-1)	unprognostic (2.36e-1)
unprognostic (5.76e-2)	unprognostic (9.07e-2)	unprognostic (3.95e-1)	unprognostic (8.34e-3)	potential prognostic favorable (6.22e-5)	unprognostic (2.31e-1)
unprognostic (3.82e-2)	unprognostic (1.83e-1)	unprognostic (3.41e-2)	potential prognostic unfavorable (4.86e-7)	unprognostic (3.34e-3)	unprognostic (5.73e-2)
	unprognostic (3.32e-2)	unprognostic (9.98e-2)	unprognostic (2.59e-3)	unprognostic (4.70e-3)	unprognostic (7.44e-3)
unprognostic (5.71e-2)	unprognostic (1.12e-1)	unprognostic (2.42e-2)		unprognostic (2.23e-2)	unprognostic (1.01e-1)
		unprognostic (6.20e-2)	unprognostic (5.55e-2)		
unprognostic (4.95e-2)	unprognostic (2.03e-2)	unprognostic (2.96e-1)	unprognostic (9.90e-2)	unprognostic (3.95e-2)	unprognostic (1.55e-1)
unprognostic (9.36e-3)	unprognostic (1.35e-1)	unprognostic (3.14e-1)	unprognostic (6.56e-2)	unprognostic (2.75e-1)	potential prognostic unfavorable (9.07e-7)
unprognostic (4.81e-2)	unprognostic (3.92e-1)	unprognostic (8.55e-2)	unprognostic (6.57e-2)	unprognostic (1.51e-3)	
unprognostic (8.85e-2)	unprognostic (1.27e-1)	unprognostic (2.36e-1)	unprognostic (4.13e-2)	unprognostic (4.41e-2)	unprognostic (3.14e-2)
unprognostic (3.41e-2)	unprognostic (1.46e-1)	unprognostic (3.29e-1)	unprognostic (1.09e-3)	unprognostic (1.57e-1)	unprognostic (9.98e-3)
unprognostic (1.45e-2)	unprognostic (7.36e-2)		unprognostic (5.39e-3)	unprognostic (5.62e-2)	potential prognostic unfavorable (7.45e-4)
unprognostic (1.78e-1)	unprognostic (1.08e-1)			unprognostic (2.15e-1)	unprognostic (7.99e-2)
unprognostic (1.53e-1)	unprognostic (5.15e-2)	unprognostic (1.04e-1)	unprognostic (2.86e-2)	unprognostic (1.79e-1)	unprognostic (1.31e-1)
unprognostic (1.84e-1)	unprognostic (7.26e-2)	unprognostic (1.39e-1)	unprognostic (5.23e-2)	unprognostic (3.27e-3)	unprognostic (3.51e-2)
unprognostic (1.03e-1)	unprognostic (5.88e-3)	unprognostic (7.11e-2)	unprognostic (1.44e-1)	unprognostic (1.79e-1)	unprognostic (1.88e-1)
unprognostic (1.43e-1)	unprognostic (9.42e-2)	unprognostic (1.67e-3)	unprognostic (7.01e-3)	unprognostic (9.80e-3)	unprognostic (1.78e-1)
unprognostic (1.08e-2)	unprognostic (1.19e-1)	unprognostic (1.49e-1)	potential prognostic favorable (6.41e-4)	unprognostic (1.65e-2)	unprognostic (1.37e-2)
unprognostic (1.45e-1)	unprognostic (3.03e-1)	unprognostic (8.87e-2)	unprognostic (2.11e-1)	unprognostic (4.87e-2)	unprognostic (3.66e-3)
unprognostic (3.17e-1)	unprognostic (2.82e-1)	unprognostic (3.90e-2)	unprognostic (8.23e-2)	unprognostic (6.32e-3)	unprognostic (8.90e-2)
unprognostic (1.84e-1)	unprognostic (9.55e-2)			unprognostic (1.18e-1)	unprognostic (2.31e-1)
unprognostic (1.92e-1)	unprognostic (1.09e-1)			unprognostic (3.40e-2)	
unprognostic (7.75e-2)	unprognostic (2.96e-1)				unprognostic (1.35e-1)
unprognostic (3.53e-1)	unprognostic (1.01e-1)	unprognostic (1.34e-1)	unprognostic (7.53e-3)	unprognostic (7.48e-2)	unprognostic (4.43e-1)
unprognostic (4.18e-2)	unprognostic (1.37e-1)	unprognostic (2.19e-1)	unprognostic (4.07e-3)	unprognostic (2.59e-1)	unprognostic (2.60e-2)
unprognostic (1.23e-1)	unprognostic (1.03e-1)	unprognostic (1.00e-1)	unprognostic (4.68e-3)	unprognostic (2.75e-1)	unprognostic (2.97e-2)

unprognostic (1.59e-1)	unprognostic (3.06e-2)	unprognostic (4.54e-2)	unprognostic (7.88e-3)	unprognostic (6.86e-2)	unprognostic (4.48e-1)
unprognostic (1.19e-1)	unprognostic (1.25e-1)	unprognostic (4.30e-1)	unprognostic (5.05e-2)	unprognostic (2.09e-2)	unprognostic (6.35e-3)
unprognostic (6.75e-2)	unprognostic (6.21e-3)	unprognostic (1.53e-1)	unprognostic (6.90e-2)	unprognostic (7.11e-2)	unprognostic (3.09e-3)
unprognostic (4.27e-1)	unprognostic (4.37e-2)	unprognostic (2.02e-1)	unprognostic (4.74e-3)	unprognostic (6.83e-3)	unprognostic (2.94e-2)
unprognostic (1.46e-2)	unprognostic (1.38e-1)	unprognostic (3.18e-1)	unprognostic (6.40e-2)	potential prognostic favorable (1.40e-4)	unprognostic (6.68e-2)
unprognostic (1.14e-1)	unprognostic (5.23e-3)	unprognostic (2.84e-1)	unprognostic (7.49e-3)	unprognostic (2.20e-1)	unprognostic (1.66e-3)
unprognostic (7.65e-2)	unprognostic (7.41e-2)	unprognostic (6.66e-2)	potential prognostic favorable (2.66e-4)	unprognostic (6.32e-2)	unprognostic (5.57e-3)
unprognostic (4.00e-2)		unprognostic (2.34e-2)	unprognostic (7.12e-2)	unprognostic (2.05e-3)	
unprognostic (1.32e-1)	unprognostic (7.67e-2)	unprognostic (1.26e-1)	unprognostic (3.98e-1)	unprognostic (1.31e-2)	unprognostic (2.33e-1)
unprognostic (7.20e-2)	unprognostic (1.93e-1)	unprognostic (6.74e-2)	unprognostic (2.76e-3)	unprognostic (1.84e-1)	unprognostic (1.91e-1)
	unprognostic (5.91e-2)	unprognostic (1.20e-1)	unprognostic (1.40e-2)		unprognostic (8.79e-2)
unprognostic (7.31e-2)	unprognostic (1.72e-2)	unprognostic (6.10e-2)	unprognostic (1.31e-1)	unprognostic (6.72e-2)	potential prognostic unfavorable (4.50e-6)
unprognostic (1.16e-1)	unprognostic (2.18e-2)	unprognostic (1.68e-2)	unprognostic (1.74e-1)	unprognostic (4.92e-2)	unprognostic (1.25e-2)
unprognostic (5.47e-3)	unprognostic (8.32e-2)	unprognostic (4.69e-2)	unprognostic (9.89e-2)		
unprognostic (1.61e-1)	unprognostic (4.75e-2)			unprognostic (9.31e-2)	unprognostic (3.31e-2)
unprognostic (1.08e-1)	unprognostic (2.03e-1)	unprognostic (1.38e-1)	unprognostic (1.17e-3)	unprognostic (1.57e-1)	unprognostic (2.80e-2)
unprognostic (1.15e-1)	unprognostic (9.28e-2)	unprognostic (1.39e-1)	unprognostic (2.51e-1)	unprognostic (4.26e-2)	unprognostic (1.22e-2)
unprognostic (3.52e-1)	unprognostic (1.98e-1)	unprognostic (9.02e-3)	unprognostic (2.18e-2)	unprognostic (2.75e-2)	
unprognostic (2.33e-1)	unprognostic (3.20e-1)	unprognostic (1.88e-1)	unprognostic (1.41e-1)	unprognostic (2.33e-2)	unprognostic (6.16e-2)
unprognostic (9.40e-3)	unprognostic (1.35e-1)	unprognostic (2.95e-1)	unprognostic (8.80e-2)		
		unprognostic (2.32e-1)	unprognostic (2.94e-1)	unprognostic (2.00e-2)	unprognostic (1.03e-1)
unprognostic (1.47e-2)	unprognostic (6.36e-2)	unprognostic (1.15e-1)	unprognostic (2.90e-2)	unprognostic (2.61e-2)	unprognostic (8.43e-2)
unprognostic (2.54e-1)	unprognostic (2.23e-1)	unprognostic (1.39e-1)	unprognostic (8.45e-2)	unprognostic (1.47e-2)	unprognostic (9.87e-3)
unprognostic (1.47e-2)	unprognostic (7.12e-2)	unprognostic (1.98e-3)	unprognostic (1.61e-3)	unprognostic (1.33e-2)	
unprognostic (1.02e-1)	unprognostic (1.44e-3)	unprognostic (8.84e-2)	unprognostic (1.30e-1)	unprognostic (5.98e-2)	unprognostic (1.61e-1)
unprognostic (4.87e-3)	unprognostic (2.23e-2)	unprognostic (1.07e-1)	unprognostic (2.14e-2)	unprognostic (8.02e-2)	unprognostic (6.36e-2)
unprognostic (8.76e-2)	unprognostic (3.26e-2)	unprognostic (1.83e-1)	unprognostic (7.55e-3)	unprognostic (2.19e-1)	unprognostic (4.95e-2)
unprognostic (6.65e-2)	unprognostic (2.29e-1)		unprognostic (1.61e-1)		
unprognostic (2.44e-1)	unprognostic (1.84e-1)	unprognostic (2.03e-1)	unprognostic (2.97e-3)	unprognostic (4.25e-2)	unprognostic (5.69e-2)
unprognostic (6.93e-2)	unprognostic (3.70e-2)	unprognostic (1.51e-1)	unprognostic (1.34e-1)	unprognostic (1.77e-3)	unprognostic (4.32e-1)
unprognostic (5.08e-2)	unprognostic (2.17e-1)	unprognostic (1.30e-1)	unprognostic (4.36e-2)	unprognostic (7.54e-3)	unprognostic (9.41e-3)
unprognostic (1.33e-1)	unprognostic (1.27e-1)	unprognostic (2.92e-2)	unprognostic (5.49e-2)	unprognostic (1.34e-2)	unprognostic (2.89e-2)
unprognostic (2.06e-1)	unprognostic (8.28e-2)	unprognostic (2.01e-2)	unprognostic (2.78e-1)	unprognostic (3.70e-2)	unprognostic (1.12e-3)
unprognostic (4.56e-2)	unprognostic (1.10e-1)			unprognostic (2.74e-1)	unprognostic (2.38e-1)
unprognostic (1.01e-1)	unprognostic (1.68e-1)	unprognostic (4.35e-1)	unprognostic (2.75e-1)	unprognostic (2.95e-1)	unprognostic (2.37e-2)
				unprognostic (1.04e-1)	
unprognostic (3.04e-1)	unprognostic (9.96e-2)			unprognostic (4.66e-2)	
unprognostic (3.49e-1)	unprognostic (1.52e-1)	unprognostic (3.23e-1)	potential prognostic favorable (3.08e-5)	unprognostic (1.08e-2)	potential prognostic unfavorable (4.06e-4)
unprognostic (8.24e-2)	unprognostic (4.30e-2)	unprognostic (1.17e-1)	unprognostic (1.62e-3)	unprognostic (1.37e-1)	unprognostic (3.26e-3)
unprognostic (2.40e-1)	unprognostic (4.77e-3)	unprognostic (1.85e-1)	unprognostic (1.69e-1)	unprognostic (1.78e-1)	unprognostic (4.79e-2)
unprognostic (8.51e-2)	unprognostic (2.05e-2)	unprognostic (2.32e-2)	unprognostic (3.35e-2)	potential prognostic favorable (3.96e-4)	unprognostic (8.54e-2)
unprognostic (1.12e-1)	unprognostic (1.44e-1)	unprognostic (1.28e-1)	unprognostic (7.01e-3)	unprognostic (2.84e-2)	unprognostic (7.66e-3)
unprognostic (1.36e-1)	unprognostic (7.83e-2)	unprognostic (2.12e-1)	unprognostic (2.33e-1)	unprognostic (1.37e-1)	unprognostic (3.84e-2)
unprognostic (8.80e-2)	unprognostic (6.17e-2)	unprognostic (1.79e-1)	unprognostic (6.95e-2)	potential prognostic favorable (7.56e-4)	unprognostic (5.72e-2)
unprognostic (4.66e-2)	unprognostic (4.35e-1)	unprognostic (3.66e-2)	unprognostic (1.98e-1)	unprognostic (3.21e-3)	unprognostic (4.36e-2)
unprognostic (1.05e-1)	unprognostic (2.00e-1)	unprognostic (9.30e-3)	unprognostic (6.79e-3)	unprognostic (1.91e-3)	unprognostic (1.65e-2)
	unprognostic (1.80e-1)	unprognostic (9.14e-2)	unprognostic (4.31e-1)		
unprognostic (1.16e-1)	unprognostic (5.01e-2)	unprognostic (2.43e-1)	unprognostic (6.22e-2)	unprognostic (1.35e-2)	unprognostic (6.27e-2)
unprognostic (5.95e-3)	unprognostic (3.62e-1)	unprognostic (9.87e-3)	unprognostic (2.52e-3)	unprognostic (6.15e-2)	unprognostic (5.71e-3)
unprognostic (2.71e-1)	unprognostic (6.69e-2)	unprognostic (2.88e-2)	potential prognostic unfavorable (8.44e-4)	unprognostic (2.65e-1)	unprognostic (1.79e-2)
unprognostic (1.68e-1)	unprognostic (1.37e-1)	unprognostic (1.02e-1)	unprognostic (2.02e-1)	unprognostic (1.23e-1)	unprognostic (8.56e-3)
unprognostic (2.31e-1)	unprognostic (5.92e-3)	unprognostic (3.03e-1)	unprognostic (6.85e-2)	unprognostic (6.07e-2)	unprognostic (1.37e-2)
unprognostic (7.91e-3)	unprognostic (4.72e-2)	unprognostic (2.59e-3)	unprognostic (7.11e-2)	unprognostic (6.22e-2)	unprognostic (4.15e-2)
	unprognostic (3.21e-1)	unprognostic (1.61e-1)	unprognostic (5.47e-2)	unprognostic (6.93e-3)	
unprognostic (4.59e-2)	unprognostic (2.13e-1)	unprognostic (2.29e-2)	unprognostic (3.18e-1)	unprognostic (5.54e-2)	unprognostic (1.65e-1)
unprognostic (5.96e-2)	unprognostic (4.42e-3)	unprognostic (1.54e-2)	unprognostic (4.75e-2)	unprognostic (1.19e-2)	unprognostic (1.59e-3)
	unprognostic (2.58e-1)			unprognostic (1.29e-2)	
unprognostic (2.18e-2)	unprognostic (3.32e-1)	unprognostic (2.57e-1)	unprognostic (6.23e-2)	unprognostic (2.10e-1)	potential prognostic unfavorable (2.40e-4)
unprognostic (9.67e-2)	unprognostic (2.64e-1)	unprognostic (1.95e-1)	unprognostic (1.42e-1)	unprognostic (8.33e-2)	unprognostic (1.57e-1)
unprognostic (1.48e-1)	unprognostic (1.29e-1)	unprognostic (7.90e-2)	unprognostic (9.69e-2)	unprognostic (1.66e-2)	unprognostic (7.73e-2)
unprognostic (5.52e-2)	unprognostic (7.11e-2)	unprognostic (5.67e-2)	unprognostic (8.36e-2)	unprognostic (2.68e-2)	unprognostic (4.38e-2)
unprognostic (1.41e-1)	unprognostic (2.65e-2)	unprognostic (4.46e-1)	unprognostic (2.89e-1)	potential prognostic favorable (1.50e-4)	unprognostic (5.29e-2)
unprognostic (1.37e-2)	unprognostic (7.54e-2)	unprognostic (2.58e-1)	unprognostic (8.19e-2)	unprognostic (3.56e-1)	unprognostic (4.88e-2)
unprognostic (1.38e-1)	unprognostic (2.03e-1)	unprognostic (1.89e-1)	potential prognostic favorable (1.46e-4)	unprognostic (8.15e-2)	unprognostic (6.55e-2)
unprognostic (1.80e-2)	unprognostic (8.62e-2)	unprognostic (2.60e-1)	unprognostic (1.45e-3)	unprognostic (6.96e-2)	unprognostic (1.24e-1)
	unprognostic (6.22e-2)			unprognostic (3.24e-1)	
unprognostic (2.59e-1)	unprognostic (1.94e-2)	unprognostic (7.66e-2)		unprognostic (1.01e-2)	unprognostic (1.14e-1)
				unprognostic (3.66e-2)	
unprognostic (2.72e-2)	unprognostic (3.55e-2)	unprognostic (9.71e-2)	unprognostic (1.35e-1)	unprognostic (1.37e-2)	unprognostic (2.32e-1)
unprognostic (3.00e-1)	unprognostic (3.71e-1)	unprognostic (7.02e-2)	unprognostic (1.98e-2)	unprognostic (1.08e-1)	unprognostic (5.44e-2)
unprognostic (3.50e-1)	unprognostic (4.57e-1)	potential prognostic unfavorable (2.06e-4)	unprognostic (3.56e-2)	unprognostic (4.25e-3)	unprognostic (8.25e-2)
unprognostic (3.01e-2)	unprognostic (2.33e-2)			unprognostic (1.12e-1)	
		unprognostic (4.38e-1)	unprognostic (7.03e-3)		
		unprognostic (3.89e-2)	unprognostic (1.77e-3)		
	unprognostic (2.40e-1)	unprognostic (7.00e-2)	potential prognostic favorable (2.44e-4)		
	unprognostic (9.07e-3)	unprognostic (1.41e-1)	unprognostic (1.01e-1)	potential prognostic favorable (1.56e-4)	unprognostic (2.23e-2)
	unprognostic (5.29e-2)		unprognostic (2.15e-2)	unprognostic (7.73e-3)	
unprognostic (1.34e-1)	unprognostic (2.25e-1)	unprognostic (1.40e-1)	unprognostic (1.73e-3)	unprognostic (2.32e-2)	unprognostic (1.16e-1)
unprognostic (2.40e-1)	unprognostic (2.79e-1)		unprognostic (1.11e-2)	unprognostic (1.71e-1)	
unprognostic (3.00e-2)	unprognostic (2.01e-1)	unprognostic (5.02e-3)	potential prognostic unfavorable (8.09e-4)	unprognostic (3.09e-2)	unprognostic (5.69e-3)
unprognostic (9.84e-2)	unprognostic (7.92e-2)	unprognostic (1.87e-1)	unprognostic (1.31e-1)	unprognostic (6.21e-2)	unprognostic (8.90e-2)
unprognostic (7.72e-2)	unprognostic (1.91e-1)	unprognostic (2.01e-1)	unprognostic (1.12e-1)	unprognostic (1.88e-2)	unprognostic (2.51e-2)
unprognostic (2.39e-1)	unprognostic (1.36e-1)	unprognostic (5.46e-2)	potential prognostic favorable (7.03e-4)	unprognostic (4.57e-2)	unprognostic (4.12e-2)
		unprognostic (3.74e-2)	unprognostic (6.16e-2)		
unprognostic (2.44e-1)	unprognostic (4.89e-2)	unprognostic (1.14e-2)	unprognostic (2.87e-2)	unprognostic (4.45e-2)	unprognostic (1.27e-1)
unprognostic (4.69e-3)	unprognostic (1.70e-1)	unprognostic (5.12e-3)	unprognostic (1.90e-1)	unprognostic (7.86e-3)	unprognostic (3.25e-1)
unprognostic (5.26e-2)	unprognostic (4.72e-1)	unprognostic (2.92e-1)	unprognostic (2.12e-2)	unprognostic (1.99e-1)	unprognostic (3.95e-2)
unprognostic (1.02e-1)	unprognostic (2.09e-2)	unprognostic (3.85e-2)	unprognostic (1.25e-1)	unprognostic (1.99e-2)	unprognostic (5.30e-2)
unprognostic (1.11e-1)	unprognostic (8.38e-2)	unprognostic (3.28e-1)	unprognostic (3.43e-2)	unprognostic (4.18e-2)	unprognostic (8.47e-2)
		unprognostic (1.95e-1)	unprognostic (1.18e-1)	unprognostic (1.09e-1)	
unprognostic (5.15e-2)	unprognostic (3.98e-2)	unprognostic (8.36e-2)	unprognostic (3.44e-1)		unprognostic (8.71e-3)
unprognostic (1.44e-1)	unprognostic (3.94e-1)	unprognostic (4.66e-2)	unprognostic (3.48e-2)	unprognostic (1.59e-2)	potential prognostic unfavorable (5.73e-6)
unprognostic (1.75e-1)	unprognostic (2.61e-1)	unprognostic (6.11e-2)	unprognostic (6.01e-2)	unprognostic (2.59e-1)	unprognostic (5.95e-3)
unprognostic (6.84e-2)	unprognostic (2.56e-1)	unprognostic (1.31e-3)	unprognostic (1.76e-3)	unprognostic (1.40e-2)	unprognostic (4.71e-1)
	unprognostic (9.50e-2)	unprognostic (2.32e-2)	potential prognostic favorable (2.31e-4)	unprognostic (4.37e-2)	unprognostic (2.95e-2)
unprognostic (1.83e-1)	unprognostic (7.14e-2)	unprognostic (2.00e-1)	unprognostic (1.12e-1)	unprognostic (3.59e-2)	unprognostic (2.65e-1)
unprognostic (1.79e-2)	unprognostic (9.91e-3)	unprognostic (1.30e-1)	unprognostic (1.88e-2)	unprognostic (6.83e-2)	unprognostic (2.76e-1)
	unprognostic (1.53e-1)	unprognostic (1.83e-1)	potential prognostic favorable (9.69e-5)		
unprognostic (6.63e-2)	unprognostic (1.18e-1)	unprognostic (2.48e-1)	unprognostic (1.34e-1)	unprognostic (2.11e-3)	unprognostic (3.65e-1)
		unprognostic (1.74e-1)	unprognostic (1.81e-1)		unprognostic (1.97e-1)
unprognostic (1.19e-1)	unprognostic (1.21e-1)	unprognostic (2.29e-2)	unprognostic (4.87e-1)	unprognostic (2.94e-2)	potential prognostic unfavorable (4.14e-5)
			unprognostic (3.46e-2)		
unprognostic (3.13e-2)	unprognostic (1.92e-2)	unprognostic (4.46e-2)	unprognostic (2.70e-3)	unprognostic (2.41e-1)	potential prognostic unfavorable (7.39e-4)
unprognostic (2.19e-1)	unprognostic (1.28e-1)	unprognostic (1.13e-1)	unprognostic (3.18e-1)	unprognostic (9.21e-2)	unprognostic (1.94e-1)
	unprognostic (2.00e-2)		unprognostic (4.07e-2)		
unprognostic (2.48e-2)	unprognostic (3.61e-1)	potential prognostic unfavorable (4.47e-4)	unprognostic (2.60e-2)	unprognostic (1.11e-3)	unprognostic (3.42e-1)
unprognostic (2.19e-1)	unprognostic (2.27e-1)	unprognostic (2.66e-2)	unprognostic (6.38e-2)	unprognostic (7.38e-2)	
unprognostic (1.90e-1)	unprognostic (6.39e-2)	unprognostic (1.84e-1)	unprognostic (4.15e-3)	unprognostic (1.90e-1)	unprognostic (1.85e-1)
		unprognostic (2.55e-1)	unprognostic (5.37e-2)	unprognostic (1.79e-1)	
unprognostic (1.83e-3)	unprognostic (1.63e-1)	unprognostic (3.29e-2)	potential prognostic unfavorable (6.98e-5)	unprognostic (8.71e-2)	unprognostic (1.45e-2)
unprognostic (2.81e-1)	unprognostic (2.52e-1)	unprognostic (1.78e-1)	unprognostic (4.27e-1)	unprognostic (8.47e-2)	unprognostic (2.64e-2)
unprognostic (1.23e-1)	unprognostic (4.23e-2)	unprognostic (1.04e-1)	unprognostic (3.70e-2)	unprognostic (2.30e-2)	unprognostic (1.95e-2)
unprognostic (8.33e-2)	unprognostic (3.01e-2)	unprognostic (5.63e-2)	unprognostic (1.12e-1)	unprognostic (1.49e-3)	unprognostic (2.57e-1)
unprognostic (4.95e-2)	unprognostic (4.78e-3)	unprognostic (1.87e-2)	unprognostic (8.33e-2)	unprognostic (4.26e-2)	unprognostic (7.36e-2)
unprognostic (5.82e-2)	unprognostic (2.55e-2)	unprognostic (1.99e-1)	potential prognostic favorable (5.85e-4)	unprognostic (1.13e-1)	unprognostic (7.84e-2)
unprognostic (3.01e-1)	unprognostic (1.51e-2)	unprognostic (6.06e-2)	unprognostic (1.42e-1)	unprognostic (2.67e-1)	unprognostic (6.88e-3)
unprognostic (1.59e-1)	unprognostic (1.60e-1)	unprognostic (1.30e-1)	unprognostic (8.92e-2)	unprognostic (2.02e-1)	unprognostic (3.14e-2)
potential prognostic favorable (9.95e-4)	unprognostic (2.90e-1)	unprognostic (1.97e-1)	potential prognostic unfavorable (7.31e-4)		unprognostic (3.54e-2)
unprognostic (1.28e-1)	unprognostic (4.18e-1)	unprognostic (1.06e-1)	unprognostic (1.00e-1)	unprognostic (3.52e-1)	unprognostic (2.13e-3)
unprognostic (7.52e-2)	unprognostic (5.85e-2)			unprognostic (5.91e-2)	unprognostic (2.96e-1)
	unprognostic (6.79e-2)		unprognostic (3.11e-2)		
unprognostic (3.68e-1)	unprognostic (6.12e-2)	unprognostic (2.07e-1)		unprognostic (3.34e-1)	unprognostic (3.87e-1)
	unprognostic (6.90e-3)	unprognostic (1.99e-1)	unprognostic (1.21e-1)	unprognostic (2.14e-2)	unprognostic (4.34e-3)
unprognostic (8.42e-2)	unprognostic (1.56e-2)	unprognostic (4.18e-2)	unprognostic (1.40e-1)	unprognostic (1.15e-3)	unprognostic (9.11e-2)
unprognostic (3.20e-1)	potential prognostic favorable (1.44e-4)	unprognostic (2.12e-1)	unprognostic (6.74e-3)	unprognostic (1.05e-1)	unprognostic (3.31e-1)

unprognostic (7.73e-2)	unprognostic (1.04e-2)	unprognostic (1.92e-1)	unprognostic (1.60e-1)		unprognostic (1.89e-1)
unprognostic (1.85e-3)	unprognostic (9.67e-2)	unprognostic (1.47e-2)	unprognostic (2.80e-2)	unprognostic (7.15e-2)	unprognostic (3.99e-2)
unprognostic (1.80e-1)	unprognostic (2.88e-3)			unprognostic (1.05e-1)	unprognostic (3.62e-2)
unprognostic (3.71e-1)	unprognostic (2.05e-1)	unprognostic (9.80e-2)		unprognostic (3.33e-1)	unprognostic (1.06e-3)
unprognostic (1.57e-2)	unprognostic (1.51e-1)	unprognostic (2.29e-3)	unprognostic (4.48e-2)	unprognostic (2.29e-1)	unprognostic (1.34e-1)
unprognostic (1.71e-1)	unprognostic (1.90e-1)	unprognostic (6.49e-2)	potential prognostic favorable (8.20e-4)	unprognostic (5.93e-2)	potential prognostic unfavorable (3.25e-4)
			unprognostic (1.40e-2)		
unprognostic (6.03e-2)	unprognostic (6.78e-2)	unprognostic (4.54e-2)	potential prognostic favorable (5.43e-4)	unprognostic (5.50e-2)	
unprognostic (1.27e-1)	unprognostic (5.82e-3)			unprognostic (2.56e-3)	
unprognostic (1.83e-2)	unprognostic (2.87e-1)	potential prognostic unfavorable (8.87e-4)	unprognostic (8.07e-2)	unprognostic (1.05e-1)	unprognostic (5.65e-2)
	unprognostic (1.30e-2)		unprognostic (1.39e-3)		
unprognostic (1.08e-1)	unprognostic (4.55e-2)	unprognostic (2.73e-2)	unprognostic (2.14e-1)	unprognostic (1.22e-1)	unprognostic (5.76e-2)
unprognostic (3.99e-1)	unprognostic (1.08e-1)		unprognostic (7.75e-2)	unprognostic (1.25e-3)	
unprognostic (7.82e-2)	unprognostic (6.51e-3)	unprognostic (1.98e-3)	potential prognostic unfavorable (7.00e-4)	unprognostic (1.03e-1)	unprognostic (4.27e-2)
unprognostic (6.42e-2)	unprognostic (3.03e-1)	unprognostic (6.93e-2)	unprognostic (3.12e-1)	unprognostic (2.12e-1)	unprognostic (1.48e-3)
unprognostic (2.81e-1)	potential prognostic unfavorable (2.33e-5)	unprognostic (4.12e-1)	unprognostic (1.08e-1)	unprognostic (9.29e-2)	unprognostic (1.14e-1)
unprognostic (7.14e-2)	unprognostic (2.25e-2)	unprognostic (9.60e-2)	unprognostic (3.03e-3)	unprognostic (6.28e-3)	unprognostic (1.59e-2)
unprognostic (2.54e-2)	unprognostic (2.09e-1)	unprognostic (1.58e-1)	unprognostic (2.84e-3)	unprognostic (1.94e-1)	unprognostic (2.63e-2)
potential prognostic favorable (2.02e-5)	unprognostic (5.63e-2)	unprognostic (2.17e-1)	unprognostic (2.51e-2)	unprognostic (1.75e-1)	unprognostic (1.31e-3)
unprognostic (2.19e-1)	unprognostic (1.81e-1)	unprognostic (8.18e-2)	unprognostic (3.76e-2)	unprognostic (2.73e-1)	unprognostic (4.02e-2)
unprognostic (7.15e-3)	unprognostic (4.47e-2)	potential prognostic unfavorable (9.54e-4)	unprognostic (2.81e-1)	unprognostic (1.79e-1)	unprognostic (1.35e-2)
unprognostic (4.43e-1)	unprognostic (5.38e-2)	unprognostic (4.18e-2)	unprognostic (3.45e-3)	unprognostic (1.06e-1)	unprognostic (1.21e-1)
unprognostic (2.34e-1)	unprognostic (2.05e-1)	unprognostic (1.81e-2)	unprognostic (4.31e-3)	unprognostic (2.34e-2)	potential prognostic unfavorable (3.47e-4)
unprognostic (5.66e-2)	unprognostic (2.60e-2)	unprognostic (2.15e-1)	unprognostic (4.66e-1)	unprognostic (1.20e-2)	unprognostic (3.07e-2)
unprognostic (1.96e-1)	unprognostic (1.71e-1)	unprognostic (1.92e-1)	unprognostic (2.25e-1)	unprognostic (1.61e-2)	unprognostic (2.09e-1)
unprognostic (2.16e-1)	unprognostic (2.57e-1)	unprognostic (4.56e-1)	unprognostic (1.45e-2)	unprognostic (2.42e-1)	unprognostic (3.26e-1)
unprognostic (2.14e-1)	unprognostic (5.25e-2)				unprognostic (4.57e-2)
unprognostic (4.14e-2)	unprognostic (4.00e-1)	unprognostic (3.62e-2)	unprognostic (1.14e-2)	unprognostic (2.44e-2)	unprognostic (4.65e-2)
unprognostic (2.70e-1)	unprognostic (1.75e-1)		unprognostic (5.61e-2)	unprognostic (1.79e-1)	unprognostic (6.02e-2)
unprognostic (3.68e-1)	unprognostic (2.95e-1)	unprognostic (5.34e-2)	unprognostic (3.29e-1)	unprognostic (1.30e-1)	unprognostic (1.09e-1)
unprognostic (9.72e-2)	unprognostic (6.01e-2)	unprognostic (1.93e-3)	unprognostic (4.80e-3)	unprognostic (4.08e-2)	potential prognostic unfavorable (3.59e-4)
unprognostic (1.24e-3)	unprognostic (2.34e-1)	unprognostic (1.20e-1)	unprognostic (2.32e-3)	unprognostic (8.49e-2)	unprognostic (3.40e-2)
			unprognostic (9.81e-3)		
unprognostic (1.45e-2)	unprognostic (8.82e-2)	unprognostic (3.13e-1)	unprognostic (5.55e-3)	unprognostic (4.86e-2)	unprognostic (2.13e-1)
unprognostic (1.69e-1)	unprognostic (6.00e-2)	unprognostic (1.19e-1)	unprognostic (1.40e-1)	unprognostic (2.82e-2)	unprognostic (1.64e-2)
unprognostic (1.21e-1)	unprognostic (2.92e-2)	unprognostic (2.27e-1)	potential prognostic unfavorable (1.73e-5)	unprognostic (3.21e-2)	unprognostic (1.16e-2)
unprognostic (2.05e-1)	unprognostic (5.39e-2)	unprognostic (2.46e-2)	unprognostic (1.57e-2)	unprognostic (1.11e-1)	unprognostic (2.09e-1)
unprognostic (8.78e-2)	unprognostic (9.91e-2)	unprognostic (2.15e-2)	unprognostic (1.47e-1)	unprognostic (1.75e-3)	unprognostic (2.11e-1)
unprognostic (9.28e-2)	unprognostic (3.61e-1)	unprognostic (1.65e-1)	unprognostic (5.95e-2)	unprognostic (7.70e-2)	unprognostic (3.34e-3)
		unprognostic (7.10e-2)	potential prognostic favorable (1.96e-4)		
unprognostic (4.01e-1)	unprognostic (2.36e-2)				unprognostic (8.34e-3)
unprognostic (1.42e-1)	unprognostic (1.24e-1)			unprognostic (6.81e-2)	potential prognostic favorable (7.73e-5)
unprognostic (8.35e-2)	unprognostic (2.21e-2)				
unprognostic (5.65e-2)	unprognostic (2.31e-2)	unprognostic (3.94e-3)	unprognostic (7.10e-3)	unprognostic (4.27e-2)	unprognostic (1.14e-1)
unprognostic (2.03e-1)	unprognostic (5.66e-2)	unprognostic (3.18e-1)	unprognostic (4.48e-3)	unprognostic (3.78e-2)	unprognostic (7.09e-2)
potential prognostic favorable (3.68e-4)	unprognostic (2.99e-2)	unprognostic (3.35e-1)	potential prognostic favorable (2.10e-5)	unprognostic (9.05e-2)	unprognostic (4.97e-2)
unprognostic (1.49e-2)	unprognostic (2.38e-1)	unprognostic (7.24e-2)	unprognostic (1.91e-1)	unprognostic (2.67e-1)	unprognostic (3.28e-2)
unprognostic (7.30e-2)	unprognostic (1.10e-1)	potential prognostic unfavorable (5.99e-4)	unprognostic (1.08e-1)	unprognostic (9.26e-2)	unprognostic (1.11e-1)
unprognostic (6.87e-2)	unprognostic (1.06e-1)		unprognostic (7.79e-2)		unprognostic (9.69e-3)
unprognostic (3.16e-2)	unprognostic (5.78e-2)	unprognostic (2.56e-1)	unprognostic (8.83e-3)	unprognostic (4.71e-3)	unprognostic (3.04e-1)
unprognostic (2.31e-2)	unprognostic (2.50e-2)	unprognostic (6.29e-2)	unprognostic (1.33e-1)	unprognostic (4.54e-2)	unprognostic (1.20e-1)
unprognostic (6.38e-2)	unprognostic (1.72e-1)	unprognostic (1.67e-1)	unprognostic (2.78e-2)	unprognostic (2.10e-1)	unprognostic (2.92e-3)
unprognostic (4.54e-2)	unprognostic (3.26e-1)	unprognostic (3.03e-1)	unprognostic (5.15e-3)	unprognostic (3.86e-3)	unprognostic (4.42e-2)
	potential prognostic favorable (2.32e-4)	unprognostic (1.35e-1)	unprognostic (2.23e-2)	unprognostic (5.54e-2)	unprognostic (2.37e-1)
unprognostic (2.84e-1)	unprognostic (2.68e-2)	unprognostic (5.18e-2)	unprognostic (1.30e-1)	unprognostic (2.82e-1)	unprognostic (5.04e-2)
unprognostic (1.17e-1)	unprognostic (1.03e-1)	unprognostic (2.31e-1)	unprognostic (2.32e-2)	unprognostic (9.98e-2)	unprognostic (9.50e-2)
unprognostic (1.14e-1)	unprognostic (3.67e-2)			unprognostic (1.18e-2)	unprognostic (7.44e-2)
unprognostic (1.13e-1)	unprognostic (4.83e-3)	unprognostic (9.12e-2)	unprognostic (5.58e-2)	unprognostic (2.64e-1)	unprognostic (4.84e-2)
unprognostic (2.17e-1)	unprognostic (3.22e-1)	unprognostic (6.40e-2)	unprognostic (3.80e-1)	unprognostic (1.38e-2)	unprognostic (1.39e-1)
unprognostic (2.56e-1)	unprognostic (2.34e-1)	unprognostic (2.87e-1)	unprognostic (2.10e-2)	unprognostic (9.23e-2)	unprognostic (1.14e-1)
unprognostic (1.93e-1)	unprognostic (3.05e-1)				
unprognostic (1.24e-1)	unprognostic (5.88e-2)	unprognostic (3.98e-1)	unprognostic (2.91e-1)	unprognostic (1.95e-1)	unprognostic (1.14e-1)
unprognostic (2.52e-1)	unprognostic (1.87e-3)	unprognostic (1.31e-1)	unprognostic (2.40e-1)	unprognostic (7.20e-2)	unprognostic (4.42e-2)
unprognostic (6.55e-2)	unprognostic (1.88e-2)			unprognostic (1.95e-2)	unprognostic (8.31e-2)
unprognostic (1.11e-2)	unprognostic (1.43e-3)	unprognostic (1.98e-2)	unprognostic (1.26e-1)	unprognostic (7.99e-2)	
unprognostic (1.76e-2)	unprognostic (1.99e-3)	unprognostic (1.81e-2)	potential prognostic unfavorable (1.70e-4)	unprognostic (3.17e-2)	unprognostic (1.28e-1)
unprognostic (1.04e-1)	unprognostic (1.22e-1)	unprognostic (2.89e-1)	unprognostic (6.99e-2)	unprognostic (7.14e-2)	unprognostic (1.92e-2)
unprognostic (1.05e-1)	unprognostic (1.04e-1)	unprognostic (2.11e-2)	unprognostic (2.12e-1)	unprognostic (1.26e-1)	unprognostic (5.72e-2)
potential prognostic unfavorable (5.66e-4)	unprognostic (2.54e-2)	unprognostic (1.20e-1)	potential prognostic favorable (9.49e-4)	unprognostic (1.25e-1)	unprognostic (2.30e-1)
unprognostic (1.07e-1)	unprognostic (1.16e-2)	unprognostic (7.91e-3)	unprognostic (3.22e-1)	unprognostic (2.06e-1)	unprognostic (5.95e-3)
					unprognostic (2.39e-1)
unprognostic (1.42e-1)	unprognostic (1.54e-1)	unprognostic (1.23e-1)	unprognostic (3.81e-2)	unprognostic (1.78e-1)	unprognostic (7.55e-2)
	unprognostic (2.66e-1)				
		unprognostic (9.96e-2)	unprognostic (2.12e-2)	unprognostic (1.21e-2)	
		unprognostic (8.86e-2)	unprognostic (3.37e-2)		
unprognostic (9.51e-2)	unprognostic (3.59e-2)	unprognostic (9.12e-2)	unprognostic (1.02e-3)	unprognostic (5.64e-2)	unprognostic (4.27e-2)
unprognostic (1.70e-1)	unprognostic (1.70e-1)	unprognostic (1.84e-2)	unprognostic (1.15e-1)	unprognostic (4.35e-3)	unprognostic (4.29e-3)
unprognostic (2.09e-1)	unprognostic (6.78e-2)	unprognostic (1.59e-1)	unprognostic (1.13e-2)	unprognostic (2.33e-2)	unprognostic (1.89e-1)
	unprognostic (1.42e-1)				
unprognostic (1.34e-1)	unprognostic (1.76e-2)	unprognostic (3.47e-1)	unprognostic (5.34e-3)	unprognostic (3.36e-1)	unprognostic (9.35e-2)
unprognostic (1.38e-1)	unprognostic (1.88e-3)	unprognostic (1.67e-1)	unprognostic (1.27e-1)	unprognostic (1.94e-2)	unprognostic (6.20e-3)
	unprognostic (5.35e-2)		unprognostic (5.38e-2)		unprognostic (2.44e-1)
unprognostic (6.02e-2)	unprognostic (1.86e-1)	unprognostic (1.01e-1)	unprognostic (3.73e-2)	unprognostic (4.05e-2)	unprognostic (6.48e-2)
unprognostic (3.68e-2)	unprognostic (2.76e-1)	unprognostic (7.87e-2)	unprognostic (1.18e-1)	unprognostic (2.56e-1)	unprognostic (1.03e-1)
unprognostic (2.25e-1)	unprognostic (3.33e-2)	unprognostic (2.60e-1)	unprognostic (1.29e-3)	unprognostic (1.07e-1)	potential prognostic unfavorable (5.60e-6)
unprognostic (1.66e-2)	unprognostic (2.68e-1)	unprognostic (3.92e-2)	unprognostic (2.72e-1)	unprognostic (9.66e-2)	unprognostic (2.42e-1)
	unprognostic (2.30e-3)	unprognostic (3.34e-3)	potential prognostic unfavorable (8.05e-4)	unprognostic (1.90e-2)	
unprognostic (1.75e-3)	unprognostic (1.59e-1)	potential prognostic unfavorable (4.93e-4)	unprognostic (6.34e-2)	unprognostic (1.53e-3)	
unprognostic (1.03e-1)	unprognostic (6.55e-2)	unprognostic (1.89e-1)	unprognostic (4.51e-2)	potential prognostic unfavorable (1.61e-4)	unprognostic (4.59e-2)
	unprognostic (1.51e-1)				
unprognostic (1.19e-1)	unprognostic (7.02e-2)	unprognostic (5.53e-2)	unprognostic (1.54e-1)	unprognostic (7.61e-2)	unprognostic (4.24e-3)
unprognostic (1.42e-2)	unprognostic (7.32e-2)	unprognostic (2.13e-1)	unprognostic (3.62e-2)	unprognostic (1.23e-2)	unprognostic (1.20e-1)
unprognostic (6.10e-2)	unprognostic (4.35e-2)				
unprognostic (3.54e-1)	unprognostic (1.34e-2)	unprognostic (4.94e-1)	unprognostic (8.25e-2)	unprognostic (2.20e-1)	unprognostic (5.70e-2)
potential prognostic favorable (3.18e-4)	unprognostic (2.44e-2)	unprognostic (2.39e-1)	unprognostic (5.30e-1)	unprognostic (6.37e-2)	unprognostic (3.32e-3)
unprognostic (1.28e-1)	unprognostic (9.67e-2)	unprognostic (3.68e-1)	unprognostic (1.66e-1)	unprognostic (2.23e-2)	unprognostic (1.44e-2)
unprognostic (2.66e-1)	unprognostic (3.20e-2)	unprognostic (5.02e-2)	unprognostic (1.49e-1)	unprognostic (1.04e-2)	unprognostic (1.34e-2)
unprognostic (6.11e-2)	unprognostic (2.28e-1)	unprognostic (3.04e-2)	unprognostic (3.79e-1)	unprognostic (1.26e-1)	potential prognostic unfavorable (2.71e-5)
unprognostic (2.01e-1)	unprognostic (6.74e-2)	unprognostic (2.75e-2)	unprognostic (1.17e-3)	unprognostic (2.62e-2)	unprognostic (2.09e-1)
unprognostic (3.64e-1)	unprognostic (3.01e-1)	unprognostic (2.90e-2)	unprognostic (1.79e-2)	unprognostic (7.64e-2)	unprognostic (1.55e-3)
unprognostic (5.05e-3)	unprognostic (2.24e-1)	unprognostic (1.85e-2)	unprognostic (5.60e-3)	unprognostic (1.92e-1)	unprognostic (3.62e-2)
unprognostic (1.36e-1)	unprognostic (6.27e-3)	unprognostic (1.27e-1)	unprognostic (4.15e-2)	unprognostic (8.90e-2)	unprognostic (3.34e-2)
		unprognostic (5.99e-2)	potential prognostic favorable (9.77e-5)		
unprognostic (7.02e-2)	unprognostic (1.10e-2)	unprognostic (9.67e-2)	unprognostic (4.84e-2)	unprognostic (2.79e-2)	unprognostic (3.32e-3)
unprognostic (1.33e-1)	unprognostic (6.70e-3)	unprognostic (5.00e-2)	unprognostic (2.39e-2)	unprognostic (3.57e-1)	unprognostic (4.39e-2)
unprognostic (2.62e-1)	unprognostic (7.27e-3)	unprognostic (6.08e-2)	unprognostic (1.67e-1)	unprognostic (1.56e-1)	unprognostic (1.90e-3)

Cancer prognostics - Kidney Renal Clear Cell Carcinoma (TCGA)	Cancer prognostics - Kidney Renal Clear Cell Carcinoma (validation)	Cancer prognostics - Kidney Renal Papillary Cell Carcinoma (TCGA)	Cancer prognostics - Liver Hepatocellular Carcinoma (TCGA)	Cancer prognostics - Liver Hepatocellular Carcinoma (validation)	Cancer prognostics - Lung Adenocarcinoma (TCGA)
unprognostic (6.46e-3)	unprognostic (7.86e-2)	unprognostic (5.57e-2)	unprognostic (2.42e-2)	unprognostic (8.05e-2)	unprognostic (2.48e-1)
potential prognostic favorable (1.15e-7)	unprognostic (5.58e-3)	potential prognostic unfavorable (7.10e-4)	validated prognostic unfavorable (4.27e-7)	validated prognostic unfavorable (9.15e-8)	unprognostic (8.83e-3)
potential prognostic favorable (4.66e-4)	unprognostic (6.71e-3)	unprognostic (9.94e-3)	unprognostic (7.19e-2)	unprognostic (1.03e-1)	unprognostic (2.68e-1)
potential prognostic unfavorable (4.16e-6)	unprognostic (1.28e-1)	unprognostic (1.42e-2)	unprognostic (1.12e-1)	unprognostic (4.91e-2)	unprognostic (6.63e-2)
potential prognostic favorable (2.30e-8)	unprognostic (9.76e-3)	unprognostic (1.75e-1)	unprognostic (1.06e-2)	unprognostic (7.36e-2)	
potential prognostic favorable (2.60e-5)	unprognostic (1.32e-2)	unprognostic (9.38e-2)	unprognostic (2.06e-3)	potential prognostic favorable (9.66e-7)	
potential prognostic favorable (1.37e-7)	unprognostic (3.26e-2)	potential prognostic unfavorable (5.98e-4)	unprognostic (4.80e-2)	potential prognostic unfavorable (1.89e-4)	unprognostic (1.65e-3)
potential prognostic favorable (1.30e-5)	unprognostic (3.17e-1)	unprognostic (1.47e-1)		unprognostic (1.12e-1)	unprognostic (9.51e-2)
potential prognostic favorable (6.36e-4)	unprognostic (5.22e-2)	unprognostic (1.38e-1)	potential prognostic unfavorable (2.43e-4)	unprognostic (1.75e-1)	unprognostic (1.20e-1)
potential prognostic favorable (4.22e-13)	unprognostic (5.65e-2)	unprognostic (3.11e-2)	unprognostic (6.52e-3)	unprognostic (6.35e-3)	unprognostic (1.31e-1)
potential prognostic favorable (2.34e-4)	unprognostic (3.38e-2)	unprognostic (8.57e-3)	unprognostic (3.39e-3)	unprognostic (4.75e-3)	unprognostic (1.89e-3)
validated prognostic favorable (3.55e-15)	validated prognostic favorable (5.94e-5)	unprognostic (1.76e-1)	unprognostic (1.22e-1)	unprognostic (2.82e-2)	unprognostic (7.57e-2)
unprognostic (3.68e-1)	unprognostic (2.00e-2)	unprognostic (5.10e-2)	unprognostic (1.83e-3)		unprognostic (2.98e-2)
potential prognostic unfavorable (1.73e-5)	unprognostic (3.05e-2)	potential prognostic unfavorable (5.82e-5)	potential prognostic unfavorable (1.35e-6)	unprognostic (2.20e-2)	potential prognostic unfavorable (6.28e-6)
potential prognostic favorable (5.99e-6)	unprognostic (1.15e-2)	unprognostic (3.35e-1)	unprognostic (1.48e-1)	unprognostic (2.23e-1)	potential prognostic unfavorable (9.44e-4)
validated prognostic unfavorable (2.02e-5)	validated prognostic unfavorable (3.63e-5)	potential prognostic unfavorable (6.38e-4)			unprognostic (1.69e-3)
unprognostic (9.03e-2)	unprognostic (2.28e-2)	unprognostic (2.14e-2)	unprognostic (7.99e-2)	unprognostic (6.11e-2)	unprognostic (2.10e-1)
unprognostic (3.38e-2)	unprognostic (4.86e-2)	unprognostic (1.91e-3)	unprognostic (1.50e-1)	unprognostic (2.53e-1)	unprognostic (7.56e-2)
potential prognostic favorable (1.99e-7)	unprognostic (1.18e-3)			potential prognostic favorable (1.64e-4)	unprognostic (1.68e-1)
potential prognostic favorable (3.63e-11)	unprognostic (1.36e-2)	unprognostic (1.01e-2)	unprognostic (3.92e-1)	unprognostic (2.47e-1)	unprognostic (2.43e-3)
validated prognostic favorable (4.82e-8)	validated prognostic favorable (4.23e-6)	unprognostic (6.24e-3)	unprognostic (3.21e-3)	unprognostic (8.48e-3)	unprognostic (1.96e-1)
	unprognostic (1.17e-1)				unprognostic (2.06e-2)
potential prognostic favorable (2.65e-9)	unprognostic (1.27e-3)	unprognostic (1.05e-1)	unprognostic (3.18e-3)	unprognostic (1.73e-1)	unprognostic (3.00e-1)
unprognostic (3.64e-3)	potential prognostic favorable (9.02e-4)	potential prognostic unfavorable (2.00e-5)	unprognostic (7.04e-2)		unprognostic (7.48e-2)
	unprognostic (1.07e-3)	unprognostic (6.46e-2)			unprognostic (1.06e-3)
			potential prognostic unfavorable (6.49e-5)		potential prognostic favorable (3.40e-4)
unprognostic (1.07e-1)	unprognostic (2.68e-1)	potential prognostic unfavorable (5.69e-9)	potential prognostic unfavorable (9.30e-4)		unprognostic (2.11e-1)
unprognostic (5.16e-3)	unprognostic (8.88e-2)	potential prognostic unfavorable (4.46e-4)	unprognostic (3.10e-2)	unprognostic (2.84e-1)	unprognostic (5.65e-3)
potential prognostic unfavorable (3.29e-11)	unprognostic (1.43e-2)			unprognostic (5.82e-2)	unprognostic (1.14e-1)
unprognostic (3.25e-3)	unprognostic (1.17e-2)	unprognostic (9.60e-3)	unprognostic (1.26e-3)	unprognostic (5.80e-3)	unprognostic (5.16e-2)
potential prognostic favorable (6.64e-7)	unprognostic (3.59e-2)	unprognostic (1.34e-3)	unprognostic (1.11e-2)	potential prognostic favorable (8.63e-4)	unprognostic (4.60e-3)
potential prognostic favorable (1.65e-5)	unprognostic (3.47e-3)	unprognostic (2.02e-2)	unprognostic (2.56e-1)		unprognostic (1.77e-1)
				unprognostic (1.68e-2)	unprognostic (7.88e-3)
potential prognostic favorable (4.52e-6)	unprognostic (4.85e-3)	unprognostic (3.53e-2)	unprognostic (1.47e-1)	unprognostic (5.32e-2)	unprognostic (2.07e-2)
unprognostic (5.12e-2)	unprognostic (8.28e-2)	unprognostic (1.76e-1)	unprognostic (7.65e-2)	unprognostic (8.66e-3)	potential prognostic unfavorable (1.13e-8)
potential prognostic favorable (1.30e-6)	unprognostic (1.10e-3)	unprognostic (6.30e-2)	unprognostic (8.48e-2)	unprognostic (9.48e-3)	potential prognostic unfavorable (2.30e-4)
validated prognostic unfavorable (5.83e-8)	validated prognostic unfavorable (5.94e-4)	potential prognostic unfavorable (1.35e-5)	unprognostic (1.67e-3)	unprognostic (1.36e-1)	unprognostic (2.15e-2)
	unprognostic (1.35e-1)	potential prognostic unfavorable (1.57e-5)	potential prognostic unfavorable (3.14e-4)	unprognostic (2.19e-1)	unprognostic (4.85e-2)
potential prognostic favorable (9.77e-14)	unprognostic (3.15e-3)	unprognostic (1.14e-1)	unprognostic (3.30e-2)	unprognostic (1.04e-3)	unprognostic (9.81e-3)
unprognostic (1.07e-1)	unprognostic (4.32e-2)	potential prognostic favorable (2.15e-5)	unprognostic (1.75e-1)	unprognostic (1.08e-1)	unprognostic (3.62e-2)
potential prognostic favorable (2.77e-4)	unprognostic (2.52e-2)	unprognostic (2.32e-2)	unprognostic (5.06e-2)		unprognostic (2.55e-1)
potential prognostic favorable (4.58e-11)	unprognostic (5.33e-3)	unprognostic (9.82e-3)	unprognostic (5.04e-2)	unprognostic (6.29e-2)	unprognostic (4.82e-3)
unprognostic (1.17e-1)	unprognostic (9.71e-2)	unprognostic (4.03e-3)		unprognostic (1.78e-1)	unprognostic (1.22e-3)
validated prognostic favorable (1.47e-11)	validated prognostic favorable (4.15e-4)	unprognostic (8.65e-2)	unprognostic (2.99e-1)	unprognostic (9.38e-2)	unprognostic (2.10e-1)
potential prognostic favorable (8.50e-6)	unprognostic (1.71e-1)	unprognostic (3.76e-2)	potential prognostic unfavorable (1.23e-4)	unprognostic (5.01e-2)	unprognostic (2.62e-2)
unprognostic (1.20e-3)	unprognostic (2.33e-2)	unprognostic (6.40e-2)	unprognostic (1.20e-1)	unprognostic (2.81e-1)	unprognostic (3.91e-2)
potential prognostic favorable (4.43e-8)	unprognostic (3.40e-2)	unprognostic (2.03e-2)	unprognostic (2.43e-1)	unprognostic (9.95e-2)	unprognostic (8.33e-3)
unprognostic (4.03e-3)	unprognostic (1.26e-1)	unprognostic (2.38e-2)		unprognostic (5.86e-3)	unprognostic (1.47e-1)
unprognostic (8.82e-2)	potential prognostic favorable (2.19e-4)	potential prognostic unfavorable (2.26e-5)	unprognostic (2.09e-3)	potential prognostic favorable (4.08e-6)	unprognostic (3.70e-3)
unprognostic (4.39e-3)	unprognostic (5.60e-2)	potential prognostic favorable (1.31e-4)	potential prognostic unfavorable (1.88e-5)	unprognostic (2.63e-1)	unprognostic (2.51e-2)
unprognostic (2.72e-2)	potential prognostic favorable (3.64e-4)				
potential prognostic favorable (2.41e-4)	unprognostic (2.41e-2)	unprognostic (1.37e-1)	unprognostic (1.79e-2)	unprognostic (1.48e-1)	unprognostic (1.23e-2)
potential prognostic favorable (5.54e-7)	unprognostic (1.25e-2)	unprognostic (3.78e-3)	unprognostic (2.63e-2)	unprognostic (1.56e-1)	unprognostic (2.45e-1)
unprognostic (2.83e-2)	unprognostic (2.00e-1)	unprognostic (2.07e-1)	unprognostic (2.02e-2)	unprognostic (1.28e-1)	unprognostic (1.11e-2)
potential prognostic favorable (1.29e-4)	unprognostic (6.23e-3)	unprognostic (1.56e-1)	unprognostic (5.56e-2)	unprognostic (1.48e-1)	unprognostic (1.03e-3)
unprognostic (1.32e-3)	unprognostic (2.68e-2)	unprognostic (8.09e-2)	unprognostic (1.59e-2)	unprognostic (1.01e-3)	unprognostic (2.32e-1)
unprognostic (2.58e-2)	potential prognostic unfavorable (4.34e-4)	unprognostic (1.15e-1)	validated prognostic unfavorable (2.84e-5)	validated prognostic unfavorable (9.34e-5)	potential prognostic unfavorable (5.10e-4)
validated prognostic favorable (1.30e-8)	validated prognostic favorable (5.58e-4)	unprognostic (1.33e-3)	unprognostic (2.23e-2)	unprognostic (5.96e-2)	unprognostic (3.78e-2)
potential prognostic favorable (1.72e-4)	unprognostic (2.75e-1)	potential prognostic favorable (9.92e-6)	unprognostic (1.16e-2)	unprognostic (1.46e-3)	unprognostic (5.95e-2)
potential prognostic favorable (2.40e-6)	unprognostic (3.11e-3)	unprognostic (1.78e-1)	unprognostic (2.57e-1)	unprognostic (4.04e-2)	unprognostic (3.48e-2)
unprognostic (2.51e-2)	unprognostic (3.76e-1)	unprognostic (3.87e-3)	unprognostic (1.13e-1)	unprognostic (2.09e-2)	unprognostic (1.51e-2)
validated prognostic favorable (2.12e-11)	validated prognostic favorable (2.52e-5)	unprognostic (4.42e-3)	unprognostic (2.43e-2)	unprognostic (7.68e-3)	unprognostic (7.85e-2)
potential prognostic favorable (4.28e-7)	unprognostic (5.07e-3)	unprognostic (3.45e-1)	unprognostic (2.58e-3)	unprognostic (7.58e-3)	unprognostic (6.57e-2)
validated prognostic favorable (6.00e-14)	validated prognostic favorable (1.30e-6)	potential prognostic favorable (3.21e-5)	unprognostic (1.78e-2)	unprognostic (2.19e-3)	unprognostic (2.40e-2)
unprognostic (4.81e-2)	unprognostic (4.41e-2)	unprognostic (7.56e-2)	unprognostic (2.07e-1)	unprognostic (2.22e-1)	unprognostic (2.36e-1)
unprognostic (4.49e-3)	unprognostic (3.47e-3)	unprognostic (1.28e-1)	unprognostic (8.21e-2)	unprognostic (2.75e-1)	unprognostic (4.24e-3)
potential prognostic favorable (3.94e-7)	unprognostic (1.34e-3)				unprognostic (7.21e-3)
potential prognostic favorable (3.98e-5)	unprognostic (4.88e-2)	unprognostic (1.72e-1)	unprognostic (2.08e-2)	unprognostic (2.65e-1)	unprognostic (9.02e-2)
unprognostic (2.53e-2)	unprognostic (8.00e-2)	unprognostic (7.28e-2)	unprognostic (1.08e-1)	unprognostic (2.21e-2)	potential prognostic favorable (2.69e-5)
unprognostic (3.39e-3)	unprognostic (3.06e-3)	unprognostic (1.29e-1)	potential prognostic unfavorable (6.83e-6)	unprognostic (8.69e-2)	unprognostic (8.86e-2)
potential prognostic favorable (1.60e-5)	unprognostic (2.70e-3)	unprognostic (1.40e-1)		unprognostic (1.45e-1)	unprognostic (2.40e-1)
potential prognostic favorable (3.65e-7)	unprognostic (1.98e-2)	unprognostic (3.61e-1)	unprognostic (1.28e-1)	unprognostic (2.09e-1)	unprognostic (2.14e-1)
potential prognostic unfavorable (1.84e-4)	unprognostic (3.00e-3)	unprognostic (1.39e-1)		unprognostic (2.55e-1)	unprognostic (2.53e-1)
unprognostic (6.89e-2)	unprognostic (1.97e-1)	unprognostic (8.88e-2)	unprognostic (2.64e-2)		unprognostic (4.99e-3)
unprognostic (6.02e-2)	unprognostic (2.35e-1)	unprognostic (9.76e-2)	unprognostic (4.25e-2)	unprognostic (3.79e-2)	unprognostic (7.42e-2)
potential prognostic favorable (3.67e-4)	unprognostic (3.87e-2)	unprognostic (2.25e-1)	unprognostic (6.02e-2)	unprognostic (2.18e-1)	unprognostic (1.26e-1)
validated prognostic favorable (3.14e-7)	validated prognostic favorable (1.72e-4)	unprognostic (9.51e-3)	unprognostic (7.04e-2)	potential prognostic favorable (1.13e-4)	unprognostic (3.45e-2)
potential prognostic favorable (2.29e-5)	unprognostic (1.64e-1)	unprognostic (3.41e-2)	unprognostic (4.26e-2)	unprognostic (6.40e-2)	unprognostic (7.46e-2)
			unprognostic (1.95e-2)	unprognostic (3.14e-3)	unprognostic (1.33e-1)
unprognostic (2.52e-2)	unprognostic (2.04e-1)	unprognostic (1.84e-1)	unprognostic (6.01e-2)	unprognostic (2.73e-1)	unprognostic (9.35e-3)
potential prognostic unfavorable (6.03e-4)	unprognostic (2.23e-3)	unprognostic (1.13e-3)	unprognostic (9.08e-2)	unprognostic (9.89e-2)	unprognostic (1.71e-2)
		unprognostic (1.00e-1)			unprognostic (1.73e-1)
unprognostic (4.34e-2)	unprognostic (3.14e-1)	unprognostic (1.39e-2)	potential prognostic unfavorable (1.10e-4)	unprognostic (2.71e-1)	unprognostic (1.34e-3)
unprognostic (5.45e-3)	unprognostic (4.10e-1)	unprognostic (7.71e-2)	unprognostic (1.11e-2)	potential prognostic favorable (5.98e-8)	unprognostic (3.07e-1)
potential prognostic favorable (1.91e-7)	unprognostic (4.38e-3)	potential prognostic favorable (8.97e-5)	unprognostic (6.33e-2)	unprognostic (7.63e-2)	potential prognostic favorable (8.29e-4)
potential prognostic favorable (5.17e-7)	unprognostic (1.01e-2)	unprognostic (1.26e-1)	unprognostic (2.26e-2)	unprognostic (6.11e-2)	unprognostic (1.22e-3)
unprognostic (1.10e-1)	unprognostic (9.40e-3)	unprognostic (1.33e-1)	potential prognostic favorable (6.90e-4)	unprognostic (1.27e-3)	unprognostic (1.03e-1)
potential prognostic favorable (1.81e-6)	unprognostic (3.74e-3)	unprognostic (1.29e-1)	unprognostic (6.55e-2)	unprognostic (1.72e-1)	unprognostic (6.16e-2)
potential prognostic favorable (7.50e-6)	unprognostic (1.42e-1)	unprognostic (4.73e-2)	potential prognostic unfavorable (3.90e-8)	unprognostic (7.38e-3)	unprognostic (2.98e-2)
validated prognostic favorable (1.32e-8)	validated prognostic favorable (2.64e-4)	unprognostic (3.30e-1)		unprognostic (1.77e-1)	unprognostic (9.13e-2)
					unprognostic (3.30e-2)
unprognostic (4.11e-2)	unprognostic (1.32e-1)	unprognostic (4.96e-3)	validated prognostic unfavorable (6.92e-6)	validated prognostic unfavorable (1.04e-6)	unprognostic (3.01e-2)
potential prognostic favorable (5.04e-4)	unprognostic (7.25e-2)	unprognostic (2.93e-1)	unprognostic (1.01e-3)	unprognostic (4.45e-3)	unprognostic (3.47e-3)
potential prognostic favorable (2.76e-5)	unprognostic (3.87e-2)	unprognostic (4.62e-2)	unprognostic (5.16e-2)	unprognostic (1.95e-1)	unprognostic (2.91e-1)
unprognostic (2.68e-1)	unprognostic (8.66e-2)	unprognostic (1.24e-1)	potential prognostic unfavorable (2.75e-5)	unprognostic (1.37e-2)	unprognostic (7.01e-2)
potential prognostic favorable (5.58e-9)	unprognostic (1.81e-3)	potential prognostic favorable (2.39e-4)	unprognostic (8.45e-3)	unprognostic (1.45e-2)	unprognostic (4.30e-3)
validated prognostic favorable (4.75e-4)	validated prognostic favorable (7.06e-6)	unprognostic (1.41e-1)	unprognostic (1.90e-1)	unprognostic (3.14e-3)	unprognostic (3.44e-2)
unprognostic (4.38e-2)	unprognostic (8.30e-3)	unprognostic (2.24e-3)	unprognostic (1.08e-2)	unprognostic (7.41e-2)	unprognostic (1.81e-3)
unprognostic (9.14e-2)	unprognostic (8.04e-2)	unprognostic (6.26e-3)	unprognostic (1.99e-1)	unprognostic (8.38e-3)	unprognostic (1.08e-1)
potential prognostic favorable (2.28e-4)	unprognostic (8.66e-2)	unprognostic (1.10e-1)	unprognostic (6.29e-2)	unprognostic (2.34e-2)	unprognostic (1.01e-1)
potential prognostic favorable (2.18e-5)	unprognostic (5.53e-2)	unprognostic (1.59e-2)	unprognostic (5.19e-1)	unprognostic (7.21e-3)	unprognostic (8.41e-2)
unprognostic (6.47e-3)	unprognostic (1.15e-2)	unprognostic (7.98e-2)	unprognostic (6.45e-3)	unprognostic (8.32e-3)	unprognostic (1.42e-1)
validated prognostic unfavorable (1.69e-6)	validated prognostic unfavorable (1.02e-4)	unprognostic (8.24e-2)	unprognostic (1.59e-2)	unprognostic (9.20e-3)	unprognostic (2.81e-2)
potential prognostic unfavorable (2.38e-8)	unprognostic (1.11e-2)	unprognostic (2.36e-2)	unprognostic (1.94e-1)	unprognostic (1.51e-2)	unprognostic (5.34e-2)
unprognostic (2.19e-1)	unprognostic (6.58e-2)	unprognostic (1.39e-2)	unprognostic (2.44e-1)	unprognostic (3.76e-2)	unprognostic (2.33e-1)
unprognostic (2.29e-1)	unprognostic (1.71e-1)	unprognostic (7.26e-2)		unprognostic (1.71e-3)	unprognostic (1.39e-1)
unprognostic (1.68e-1)	unprognostic (1.63e-2)	unprognostic (1.70e-2)	unprognostic (1.33e-1)	unprognostic (4.13e-2)	unprognostic (4.92e-3)
potential prognostic favorable (4.34e-5)	unprognostic (2.09e-1)	unprognostic (4.28e-3)	unprognostic (4.32e-2)	potential prognostic favorable (4.44e-4)	unprognostic (1.47e-2)
validated prognostic favorable (4.24e-6)	validated prognostic favorable (2.41e-4)	unprognostic (7.09e-2)	unprognostic (1.21e-2)	unprognostic (3.84e-1)	unprognostic (3.54e-1)
potential prognostic favorable (4.88e-9)	unprognostic (1.66e-3)				unprognostic (1.07e-2)
potential prognostic favorable (3.45e-6)	unprognostic (1.43e-3)	unprognostic (3.14e-2)		unprognostic (1.90e-2)	unprognostic (1.78e-1)
validated prognostic favorable (1.04e-4)	validated prognostic favorable (2.24e-4)	unprognostic (1.26e-1)	unprognostic (3.93e-2)	unprognostic (1.48e-1)	unprognostic (7.31e-2)
unprognostic (8.47e-2)	unprognostic (1.57e-1)	unprognostic (1.07e-1)	unprognostic (1.28e-1)	unprognostic (1.20e-1)	potential prognostic favorable (1.03e-5)
unprognostic (1.80e-2)					
potential prognostic favorable (3.24e-12)	unprognostic (1.82e-3)	unprognostic (5.17e-3)	unprognostic (5.67e-3)	unprognostic (2.08e-1)	unprognostic (6.72e-2)
unprognostic (2.41e-3)		unprognostic (2.02e-1)	unprognostic (3.03e-1)	unprognostic (6.38e-2)	unprognostic (8.80e-3)
unprognostic (5.19e-3)	unprognostic (2.39e-1)	unprognostic (3.65e-1)	unprognostic (2.86e-3)	unprognostic (2.88e-3)	potential prognostic unfavorable (7.40e-4)
unprognostic (5.44e-3)	unprognostic (1.37e-1)	potential prognostic favorable (7.99e-4)	unprognostic (1.31e-3)	unprognostic (3.22e-2)	unprognostic (2.28e-2)
			unprognostic (9.39e-3)	potential prognostic favorable (1.71e-6)	

potential prognostic favorable (5.22e-8)	unprognostic (3.45e-2)	unprognostic (1.28e-2)	validated prognostic unfavorable (5.71e-6)	validated prognostic unfavorable (7.41e-4)	unprognostic (2.38e-3)
validated prognostic favorable (1.04e-4)	validated prognostic favorable (4.50e-4)				
unprognostic (8.91e-3)	unprognostic (4.27e-2)	unprognostic (1.13e-1)	unprognostic (1.74e-2)	potential prognostic favorable (8.77e-5)	unprognostic (1.14e-3)
validated prognostic favorable (1.58e-10)	validated prognostic favorable (8.04e-4)	unprognostic (2.84e-3)	unprognostic (2.66e-2)	unprognostic (4.10e-3)	unprognostic (1.44e-2)
potential prognostic favorable (1.21e-6)	unprognostic (3.57e-2)	unprognostic (4.66e-2)	unprognostic (1.29e-1)	unprognostic (1.33e-1)	unprognostic (4.32e-2)
potential prognostic favorable (2.03e-6)	unprognostic (6.39e-2)	unprognostic (3.56e-1)	unprognostic (6.99e-3)		unprognostic (2.03e-1)
validated prognostic favorable (2.84e-8)	validated prognostic favorable (4.96e-5)	unprognostic (7.71e-3)	unprognostic (7.42e-2)	unprognostic (1.53e-2)	unprognostic (2.44e-2)
potential prognostic favorable (3.50e-6)	unprognostic (1.63e-1)	unprognostic (8.21e-2)	unprognostic (2.26e-1)	unprognostic (5.14e-2)	unprognostic (3.16e-3)
unprognostic (1.31e-3)	unprognostic (1.07e-3)	unprognostic (7.67e-3)	unprognostic (2.59e-2)		unprognostic (5.45e-2)
potential prognostic favorable (4.16e-11)	unprognostic (1.10e-3)	unprognostic (1.09e-1)	unprognostic (5.63e-2)		unprognostic (7.23e-3)
potential prognostic favorable (5.60e-8)	unprognostic (1.01e-2)	unprognostic (6.54e-2)	unprognostic (7.12e-2)	unprognostic (2.71e-2)	unprognostic (1.47e-1)
unprognostic (3.43e-1)	unprognostic (3.93e-1)	unprognostic (1.93e-1)	potential prognostic unfavorable (3.84e-6)	unprognostic (1.40e-3)	unprognostic (3.89e-2)
potential prognostic favorable (2.42e-6)	unprognostic (4.99e-3)	unprognostic (2.62e-1)	unprognostic (1.12e-3)	unprognostic (5.62e-2)	potential prognostic unfavorable (3.28e-4)
unprognostic (1.41e-2)	unprognostic (1.80e-1)	unprognostic (6.73e-3)	validated prognostic unfavorable (1.60e-5)	validated prognostic unfavorable (4.05e-4)	unprognostic (6.99e-3)
unprognostic (2.33e-1)	unprognostic (1.67e-1)	unprognostic (3.81e-2)	unprognostic (3.19e-2)	unprognostic (1.45e-1)	unprognostic (1.53e-3)
unprognostic (5.74e-3)	unprognostic (2.13e-2)	unprognostic (2.63e-1)	potential prognostic unfavorable (8.35e-6)	unprognostic (3.04e-3)	unprognostic (2.11e-1)
potential prognostic favorable (6.09e-7)	unprognostic (9.30e-2)	unprognostic (4.65e-2)	unprognostic (1.49e-3)	potential prognostic unfavorable (9.65e-4)	unprognostic (1.53e-1)
unprognostic (6.41e-3)	unprognostic (1.23e-1)	unprognostic (2.21e-2)		unprognostic (8.66e-3)	potential prognostic favorable (5.21e-6)
unprognostic (6.70e-3)	unprognostic (6.17e-2)				potential prognostic favorable (7.41e-4)
unprognostic (3.90e-3)	unprognostic (1.09e-2)	unprognostic (7.25e-2)	unprognostic (8.77e-2)	unprognostic (1.57e-1)	unprognostic (2.92e-1)
unprognostic (9.11e-3)	unprognostic (1.08e-1)	unprognostic (1.20e-1)	unprognostic (2.29e-3)	unprognostic (2.23e-2)	potential prognostic unfavorable (1.21e-6)
unprognostic (2.67e-2)	unprognostic (2.50e-1)	unprognostic (5.02e-1)	unprognostic (1.84e-1)	unprognostic (3.71e-2)	unprognostic (8.19e-2)
unprognostic (6.05e-3)	unprognostic (1.29e-1)	unprognostic (1.91e-1)		unprognostic (3.30e-1)	unprognostic (1.16e-2)
unprognostic (1.17e-1)	unprognostic (1.18e-1)	unprognostic (3.54e-2)	unprognostic (8.61e-3)	unprognostic (4.64e-3)	unprognostic (3.01e-3)
unprognostic (1.14e-1)	unprognostic (4.85e-2)			unprognostic (4.43e-2)	potential prognostic favorable (6.71e-7)
potential prognostic favorable (1.08e-4)	unprognostic (1.85e-1)				unprognostic (2.08e-3)
potential prognostic favorable (5.33e-4)	unprognostic (9.06e-2)	unprognostic (1.18e-1)		unprognostic (3.02e-3)	unprognostic (1.32e-1)
unprognostic (2.28e-1)	unprognostic (2.26e-1)	unprognostic (5.95e-2)		unprognostic (9.28e-3)	unprognostic (5.33e-3)
potential prognostic favorable (5.79e-7)	unprognostic (1.57e-2)	unprognostic (1.91e-2)	unprognostic (1.91e-3)	unprognostic (2.08e-2)	potential prognostic unfavorable (7.96e-4)
unprognostic (5.05e-3)	unprognostic (3.40e-1)	unprognostic (1.40e-1)	unprognostic (8.17e-2)	unprognostic (7.35e-3)	unprognostic (1.02e-2)
unprognostic (1.18e-3)	unprognostic (1.31e-1)	unprognostic (6.14e-3)	unprognostic (1.00e-1)	unprognostic (8.73e-2)	unprognostic (4.05e-2)
unprognostic (1.70e-1)	unprognostic (2.67e-2)	unprognostic (4.50e-2)	unprognostic (1.28e-2)	unprognostic (3.27e-2)	unprognostic (2.30e-2)
unprognostic (3.93e-2)	unprognostic (9.65e-2)	unprognostic (1.55e-2)		unprognostic (4.77e-3)	unprognostic (9.31e-2)
unprognostic (1.01e-1)	unprognostic (2.56e-1)	unprognostic (1.24e-1)	unprognostic (7.83e-2)	unprognostic (5.34e-2)	unprognostic (1.44e-2)
unprognostic (2.74e-2)	unprognostic (1.71e-1)	unprognostic (6.55e-2)	unprognostic (8.70e-3)	unprognostic (3.46e-1)	unprognostic (2.31e-2)
unprognostic (2.42e-1)	unprognostic (2.12e-1)			unprognostic (4.01e-3)	unprognostic (1.10e-3)
potential prognostic unfavorable (1.71e-4)	unprognostic (1.76e-2)	unprognostic (1.32e-3)	unprognostic (3.08e-3)	unprognostic (1.73e-1)	unprognostic (5.04e-2)
unprognostic (8.57e-3)	unprognostic (4.16e-1)	unprognostic (2.95e-2)	potential prognostic unfavorable (1.03e-5)	unprognostic (1.09e-1)	unprognostic (5.42e-2)
potential prognostic favorable (2.74e-4)	unprognostic (1.70e-2)	potential prognostic unfavorable (3.33e-4)	unprognostic (1.82e-1)	unprognostic (2.64e-2)	potential prognostic favorable (2.91e-4)
unprognostic (2.09e-2)	unprognostic (1.97e-1)	unprognostic (1.23e-1)		unprognostic (6.70e-2)	unprognostic (2.64e-3)
unprognostic (5.50e-2)	unprognostic (3.54e-1)	unprognostic (1.10e-1)	unprognostic (1.49e-2)	unprognostic (2.22e-1)	unprognostic (2.98e-2)
unprognostic (5.56e-2)	unprognostic (1.13e-1)	unprognostic (1.28e-3)	unprognostic (1.73e-3)	unprognostic (3.28e-3)	unprognostic (9.10e-3)
unprognostic (4.71e-2)	unprognostic (1.78e-1)	unprognostic (1.27e-2)	unprognostic (4.54e-3)	unprognostic (9.97e-2)	unprognostic (1.79e-2)
validated prognostic favorable (1.87e-6)	validated prognostic favorable (3.24e-5)	unprognostic (1.34e-1)	unprognostic (7.21e-2)	unprognostic (3.13e-2)	unprognostic (8.25e-2)
unprognostic (3.17e-3)	unprognostic (2.31e-2)	unprognostic (5.11e-1)	potential prognostic unfavorable (2.11e-4)	unprognostic (2.56e-2)	unprognostic (8.26e-2)
unprognostic (9.23e-2)	unprognostic (1.74e-2)	unprognostic (1.31e-1)	unprognostic (4.14e-2)	unprognostic (3.54e-2)	unprognostic (6.89e-2)
potential prognostic favorable (4.20e-5)	unprognostic (1.61e-3)	unprognostic (9.67e-2)	unprognostic (1.16e-1)	unprognostic (2.37e-2)	unprognostic (4.04e-2)
unprognostic (2.53e-1)	unprognostic (1.71e-1)	unprognostic (4.53e-3)	unprognostic (7.95e-3)	unprognostic (1.61e-1)	unprognostic (4.21e-2)
potential prognostic favorable (3.08e-8)	unprognostic (2.11e-3)	unprognostic (1.17e-2)	unprognostic (2.04e-2)	unprognostic (9.35e-2)	unprognostic (1.57e-1)
validated prognostic favorable (1.93e-9)	validated prognostic favorable (6.97e-5)	unprognostic (6.80e-3)	unprognostic (1.34e-1)	unprognostic (8.41e-3)	unprognostic (1.43e-1)
			unprognostic (1.52e-3)	unprognostic (1.71e-2)	unprognostic (5.17e-3)
potential prognostic unfavorable (9.37e-4)	unprognostic (9.73e-2)	unprognostic (2.67e-3)	unprognostic (1.02e-1)	unprognostic (1.31e-2)	unprognostic (1.68e-3)
potential prognostic favorable (7.16e-5)	unprognostic (2.92e-3)	unprognostic (5.18e-2)	unprognostic (4.40e-2)	unprognostic (1.11e-2)	unprognostic (2.64e-2)
					potential prognostic favorable (3.80e-5)
unprognostic (6.92e-3)		potential prognostic unfavorable (7.77e-4)		unprognostic (1.41e-1)	unprognostic (2.75e-2)
potential prognostic favorable (1.64e-4)	unprognostic (2.26e-2)	unprognostic (3.77e-3)			
validated prognostic favorable (1.37e-7)	validated prognostic favorable (9.91e-4)	potential prognostic unfavorable (9.22e-5)	unprognostic (1.37e-1)	unprognostic (8.39e-3)	unprognostic (1.59e-1)
potential prognostic favorable (2.60e-5)	unprognostic (6.26e-3)	unprognostic (1.05e-3)	unprognostic (2.02e-1)	unprognostic (1.99e-1)	unprognostic (6.91e-2)
unprognostic (2.00e-1)	unprognostic (2.71e-2)	unprognostic (1.39e-1)	potential prognostic unfavorable (4.41e-6)	unprognostic (1.71e-3)	unprognostic (5.28e-3)
unprognostic (2.92e-3)	potential prognostic unfavorable (5.91e-4)	unprognostic (5.38e-2)	potential prognostic unfavorable (7.49e-5)	unprognostic (5.36e-3)	unprognostic (1.78e-1)
			potential prognostic unfavorable (2.31e-4)	unprognostic (2.01e-1)	unprognostic (4.91e-1)
				unprognostic (7.51e-2)	unprognostic (1.54e-3)
unprognostic (4.08e-3)	unprognostic (2.97e-2)	unprognostic (1.39e-3)	unprognostic (3.28e-1)	potential prognostic favorable (9.21e-5)	unprognostic (5.96e-2)
potential prognostic favorable (5.78e-7)	unprognostic (7.72e-3)	unprognostic (1.22e-2)	unprognostic (5.45e-2)		unprognostic (2.94e-1)
potential prognostic favorable (9.32e-4)	unprognostic (1.47e-3)	unprognostic (2.71e-1)	unprognostic (2.21e-2)	unprognostic (1.70e-1)	unprognostic (9.02e-2)
potential prognostic favorable (2.92e-11)	unprognostic (3.91e-3)	unprognostic (1.19e-1)	unprognostic (1.30e-1)	unprognostic (7.25e-2)	unprognostic (5.76e-2)
potential prognostic favorable (2.83e-6)	unprognostic (1.43e-2)	unprognostic (5.21e-2)	unprognostic (1.46e-3)	unprognostic (3.37e-3)	unprognostic (9.15e-3)
unprognostic (1.53e-3)	potential prognostic favorable (3.34e-4)	unprognostic (5.18e-2)	unprognostic (2.72e-3)	unprognostic (5.89e-2)	unprognostic (1.39e-1)
validated prognostic favorable (2.76e-8)	validated prognostic favorable (8.41e-4)				
	unprognostic (3.38e-1)	unprognostic (6.42e-3)			unprognostic (1.07e-3)
	unprognostic (1.82e-1)	potential prognostic unfavorable (1.49e-6)			potential prognostic unfavorable (9.12e-5)
potential prognostic unfavorable (8.40e-5)	unprognostic (3.32e-3)	unprognostic (1.10e-1)	unprognostic (9.42e-3)	potential prognostic unfavorable (5.34e-4)	unprognostic (1.46e-2)
		unprognostic (2.29e-1)			unprognostic (1.20e-1)
validated prognostic favorable (1.11e-10)	validated prognostic favorable (3.80e-4)	unprognostic (1.41e-2)	unprognostic (2.08e-1)	unprognostic (8.47e-2)	unprognostic (1.45e-1)
				unprognostic (2.69e-3)	
unprognostic (4.50e-2)	unprognostic (1.98e-1)	unprognostic (3.59e-2)			
potential prognostic favorable (8.01e-6)	unprognostic (7.56e-3)	unprognostic (9.58e-3)	unprognostic (7.70e-2)	unprognostic (8.67e-3)	potential prognostic favorable (6.58e-5)
potential prognostic favorable (6.29e-5)	unprognostic (7.53e-3)	potential prognostic favorable (3.39e-5)	unprognostic (3.00e-2)	unprognostic (1.61e-1)	unprognostic (1.70e-1)
validated prognostic favorable (1.95e-9)	validated prognostic favorable (5.05e-5)	unprognostic (1.34e-1)	unprognostic (8.54e-3)	unprognostic (2.87e-2)	unprognostic (7.40e-2)
unprognostic (2.89e-2)	unprognostic (1.11e-2)	potential prognostic favorable (9.52e-7)	unprognostic (5.73e-2)	unprognostic (5.49e-3)	unprognostic (1.52e-2)
		unprognostic (4.23e-3)	unprognostic (1.04e-1)	unprognostic (1.19e-2)	unprognostic (1.50e-1)
unprognostic (1.83e-1)	unprognostic (2.86e-2)	unprognostic (2.99e-3)		unprognostic (2.06e-2)	unprognostic (1.03e-1)
potential prognostic favorable (7.63e-5)	unprognostic (5.69e-3)	potential prognostic unfavorable (2.41e-4)	unprognostic (2.54e-1)	unprognostic (1.45e-2)	unprognostic (2.95e-2)
potential prognostic favorable (2.33e-7)	unprognostic (2.67e-2)	potential prognostic favorable (4.05e-4)	unprognostic (6.48e-3)	unprognostic (3.78e-2)	unprognostic (3.03e-2)
potential prognostic favorable (8.40e-8)	unprognostic (2.69e-3)	unprognostic (2.37e-1)		unprognostic (1.23e-2)	unprognostic (1.19e-2)
potential prognostic favorable (1.60e-6)	unprognostic (1.00e-2)	unprognostic (1.00e-1)	validated prognostic unfavorable (2.99e-6)	validated prognostic unfavorable (2.68e-5)	unprognostic (7.50e-2)
unprognostic (9.93e-3)	unprognostic (1.33e-2)	unprognostic (6.38e-2)	unprognostic (4.25e-3)	unprognostic (2.06e-1)	potential prognostic unfavorable (3.51e-4)
potential prognostic favorable (5.64e-5)			unprognostic (6.55e-2)	unprognostic (5.75e-3)	unprognostic (9.08e-3)
unprognostic (3.41e-2)	unprognostic (2.73e-1)	unprognostic (1.27e-1)	unprognostic (3.57e-3)	unprognostic (1.48e-2)	unprognostic (3.23e-2)
	unprognostic (3.48e-2)	unprognostic (1.33e-1)			unprognostic (1.07e-2)
potential prognostic favorable (2.37e-5)	unprognostic (4.00e-3)	unprognostic (1.12e-1)	unprognostic (1.78e-2)	unprognostic (5.86e-2)	unprognostic (1.47e-2)
unprognostic (3.46e-1)		unprognostic (2.25e-1)			unprognostic (5.44e-2)
potential prognostic unfavorable (1.52e-4)	unprognostic (1.23e-2)	potential prognostic unfavorable (2.45e-4)	unprognostic (2.49e-1)	unprognostic (9.53e-3)	unprognostic (2.08e-2)
unprognostic (1.26e-1)	unprognostic (3.65e-2)	unprognostic (8.04e-2)	unprognostic (5.38e-3)	unprognostic (8.87e-2)	unprognostic (1.21e-1)
potential prognostic favorable (3.84e-9)	unprognostic (7.26e-2)	unprognostic (3.16e-1)	unprognostic (3.29e-1)	unprognostic (1.94e-1)	unprognostic (6.75e-2)
	unprognostic (6.59e-2)				unprognostic (1.20e-3)
potential prognostic favorable (3.33e-16)	unprognostic (1.83e-3)	unprognostic (1.81e-3)	unprognostic (1.47e-1)	unprognostic (1.53e-1)	unprognostic (2.25e-2)
unprognostic (1.01e-3)	unprognostic (1.22e-1)	unprognostic (6.98e-3)	unprognostic (3.96e-3)	unprognostic (3.30e-1)	unprognostic (1.45e-1)
unprognostic (2.94e-3)	unprognostic (1.33e-1)	unprognostic (8.42e-2)	potential prognostic unfavorable (2.14e-5)	unprognostic (1.09e-1)	unprognostic (1.45e-1)
unprognostic (1.41e-2)	unprognostic (9.98e-2)	unprognostic (6.89e-2)	unprognostic (5.10e-2)	unprognostic (8.87e-2)	unprognostic (2.73e-3)
unprognostic (2.39e-2)	unprognostic (7.58e-2)	unprognostic (6.12e-2)	validated prognostic unfavorable (1.10e-5)	validated prognostic unfavorable (5.43e-4)	unprognostic (1.11e-1)
potential prognostic favorable (3.83e-8)	unprognostic (4.99e-3)	unprognostic (8.96e-2)	unprognostic (3.40e-3)	unprognostic (6.43e-2)	unprognostic (1.02e-2)
validated prognostic favorable (6.88e-8)	validated prognostic favorable (1.81e-5)	unprognostic (3.52e-2)	potential prognostic unfavorable (6.51e-4)	unprognostic (1.47e-2)	unprognostic (2.35e-1)
validated prognostic favorable (2.97e-9)	validated prognostic favorable (8.63e-5)	unprognostic (2.37e-1)	unprognostic (1.85e-3)	unprognostic (9.42e-2)	unprognostic (1.31e-2)
unprognostic (1.43e-3)	unprognostic (1.86e-1)	unprognostic (1.60e-3)	potential prognostic unfavorable (8.33e-4)	unprognostic (8.81e-3)	unprognostic (4.40e-2)
validated prognostic favorable (1.08e-8)	validated prognostic favorable (1.92e-5)	potential prognostic favorable (7.97e-6)	unprognostic (4.94e-2)	unprognostic (2.59e-1)	unprognostic (2.83e-3)
potential prognostic favorable (7.39e-6)	unprognostic (1.11e-3)	unprognostic (3.88e-3)		unprognostic (7.23e-2)	unprognostic (1.82e-3)
unprognostic (5.11e-2)	unprognostic (9.38e-3)	unprognostic (1.04e-1)	unprognostic (1.14e-1)	unprognostic (3.71e-2)	unprognostic (6.35e-2)
unprognostic (1.16e-2)	unprognostic (1.71e-1)				unprognostic (7.00e-3)
unprognostic (2.91e-2)	unprognostic (1.09e-1)	unprognostic (3.31e-3)	unprognostic (2.13e-1)	unprognostic (6.00e-3)	unprognostic (4.43e-2)
potential prognostic unfavorable (2.05e-4)	unprognostic (1.42e-1)	unprognostic (2.18e-3)	potential prognostic unfavorable (3.64e-4)	unprognostic (1.25e-1)	unprognostic (1.88e-2)
unprognostic (4.69e-2)	unprognostic (2.47e-1)	unprognostic (3.48e-1)	unprognostic (3.33e-2)	unprognostic (8.04e-2)	unprognostic (8.39e-3)
potential prognostic favorable (4.08e-7)	unprognostic (8.64e-3)	unprognostic (4.01e-2)	potential prognostic unfavorable (3.80e-7)	unprognostic (1.69e-1)	unprognostic (1.55e-1)
unprognostic (1.20e-2)	unprognostic (1.70e-1)	unprognostic (8.76e-3)	unprognostic (1.19e-1)	unprognostic (6.25e-3)	unprognostic (4.55e-2)
potential prognostic favorable (4.82e-4)	unprognostic (1.46e-1)	unprognostic (5.02e-2)	potential prognostic unfavorable (1.22e-4)	unprognostic (1.85e-1)	unprognostic (9.90e-2)
unprognostic (5.43e-3)	unprognostic (6.32e-2)	unprognostic (1.34e-3)	potential prognostic unfavorable (7.01e-4)	unprognostic (1.02e-2)	potential prognostic unfavorable (5.48e-4)
potential prognostic favorable (3.80e-6)	unprognostic (6.51e-3)	unprognostic (1.57e-1)	unprognostic (4.83e-2)	unprognostic (5.38e-3)	unprognostic (2.42e-1)

unprognostic (4.29e-3)	unprognostic (5.27e-2)	potential prognostic unfavorable (5.22e-7)	unprognostic (2.74e-1)	unprognostic (3.36e-2)	unprognostic (1.89e-2)
potential prognostic favorable (8.65e-5)	unprognostic (1.46e-3)	unprognostic (3.90e-3)			unprognostic (6.81e-2)
unprognostic (4.00e-3)	unprognostic (3.67e-2)	unprognostic (2.30e-2)		potential prognostic unfavorable (1.52e-7)	validated prognostic unfavorable (6.38e-5)
potential prognostic favorable (1.05e-5)	unprognostic (7.86e-3)	unprognostic (1.80e-1)	unprognostic (1.72e-1)	unprognostic (1.84e-1)	unprognostic (5.90e-2)
potential prognostic favorable (4.02e-5)	unprognostic (2.43e-1)	unprognostic (1.48e-1)	unprognostic (7.17e-2)	unprognostic (6.79e-2)	unprognostic (1.13e-1)
unprognostic (2.31e-2)	unprognostic (9.33e-2)	unprognostic (7.07e-2)	unprognostic (3.59e-3)	unprognostic (5.72e-3)	unprognostic (3.67e-2)
potential prognostic unfavorable (2.33e-9)	unprognostic (5.03e-2)	unprognostic (2.16e-2)	potential prognostic unfavorable (1.91e-5)	unprognostic (1.30e-2)	unprognostic (2.91e-3)
validated prognostic favorable (1.94e-6)	validated prognostic favorable (5.20e-4)	unprognostic (4.00e-2)	unprognostic (1.81e-3)	unprognostic (7.94e-2)	potential prognostic unfavorable (2.47e-4)
unprognostic (1.50e-2)	unprognostic (8.61e-2)	unprognostic (4.07e-2)	validated prognostic unfavorable (6.90e-4)	validated prognostic unfavorable (4.33e-4)	potential prognostic unfavorable (1.12e-4)
potential prognostic favorable (2.05e-6)	unprognostic (2.44e-3)	unprognostic (1.58e-1)	unprognostic (1.71e-1)	unprognostic (2.86e-1)	unprognostic (6.74e-3)
validated prognostic favorable (2.87e-9)	validated prognostic favorable (8.14e-4)	potential prognostic favorable (1.76e-5)	unprognostic (1.64e-1)	unprognostic (4.16e-2)	unprognostic (1.24e-2)
unprognostic (7.23e-2)	unprognostic (2.70e-1)	unprognostic (2.17e-1)	unprognostic (1.60e-1)	unprognostic (1.20e-1)	unprognostic (5.37e-3)
potential prognostic unfavorable (8.04e-5)	unprognostic (6.49e-2)	unprognostic (1.71e-1)	unprognostic (2.12e-2)	unprognostic (2.94e-2)	unprognostic (4.48e-2)
potential prognostic favorable (7.23e-5)	unprognostic (5.49e-3)	unprognostic (3.41e-2)	unprognostic (9.61e-2)	unprognostic (2.73e-1)	unprognostic (2.16e-1)
				unprognostic (1.25e-1)	
potential prognostic favorable (3.24e-4)	unprognostic (1.17e-2)	unprognostic (1.02e-2)	unprognostic (6.58e-3)	potential prognostic unfavorable (3.82e-4)	potential prognostic unfavorable (1.05e-4)
					unprognostic (1.35e-1)
potential prognostic favorable (2.68e-7)	unprognostic (1.77e-2)	unprognostic (2.05e-2)	unprognostic (1.57e-3)	unprognostic (1.06e-3)	potential prognostic unfavorable (4.07e-6)
validated prognostic favorable (3.33e-5)	validated prognostic favorable (9.83e-4)	unprognostic (1.29e-1)	unprognostic (1.11e-1)	unprognostic (7.94e-2)	unprognostic (2.91e-3)
unprognostic (1.98e-3)	unprognostic (2.47e-2)	potential prognostic favorable (2.10e-5)	unprognostic (3.81e-1)	unprognostic (1.20e-1)	unprognostic (2.59e-2)
validated prognostic favorable (2.13e-11)	validated prognostic favorable (7.59e-4)	unprognostic (3.10e-1)	unprognostic (2.80e-1)	unprognostic (5.39e-3)	unprognostic (7.17e-2)
potential prognostic favorable (3.19e-6)	unprognostic (1.78e-3)	potential prognostic unfavorable (3.64e-5)	unprognostic (4.91e-2)	unprognostic (1.63e-3)	unprognostic (1.00e-1)
unprognostic (4.67e-3)	potential prognostic favorable (9.23e-4)	potential prognostic unfavorable (4.71e-5)	unprognostic (5.16e-2)	unprognostic (2.76e-1)	unprognostic (2.27e-1)
validated prognostic favorable (7.14e-12)	validated prognostic favorable (1.07e-4)	unprognostic (1.35e-3)	unprognostic (3.71e-3)	unprognostic (3.39e-3)	unprognostic (2.64e-1)
potential prognostic favorable (3.51e-4)	unprognostic (3.49e-1)	unprognostic (4.76e-3)	unprognostic (1.16e-2)	unprognostic (4.71e-2)	unprognostic (1.98e-1)
potential prognostic favorable (3.24e-4)	unprognostic (2.10e-2)	unprognostic (1.61e-1)	unprognostic (8.75e-2)	unprognostic (1.08e-1)	unprognostic (8.06e-2)
					unprognostic (7.70e-2)
potential prognostic favorable (8.23e-5)	unprognostic (1.24e-2)	unprognostic (1.11e-1)	unprognostic (1.35e-1)	unprognostic (1.01e-2)	unprognostic (4.78e-2)
potential prognostic favorable (1.95e-7)	unprognostic (5.73e-3)	unprognostic (1.96e-1)	unprognostic (1.61e-1)	unprognostic (1.40e-1)	potential prognostic unfavorable (1.52e-4)
potential prognostic favorable (2.48e-6)	unprognostic (7.85e-2)	unprognostic (4.58e-2)	potential prognostic unfavorable (3.82e-8)	unprognostic (3.20e-2)	unprognostic (6.10e-2)
potential prognostic favorable (1.59e-4)	unprognostic (3.81e-2)	unprognostic (8.37e-2)	unprognostic (1.40e-2)	unprognostic (1.49e-1)	unprognostic (1.78e-2)
validated prognostic favorable (5.01e-6)	validated prognostic favorable (5.45e-4)	unprognostic (2.35e-1)	unprognostic (9.24e-2)	unprognostic (2.37e-2)	unprognostic (8.92e-2)
					potential prognostic favorable (2.59e-4)
unprognostic (1.45e-2)	unprognostic (1.18e-2)	unprognostic (1.26e-2)	unprognostic (4.99e-3)	unprognostic (2.68e-2)	unprognostic (2.80e-1)
validated prognostic favorable (2.31e-7)	validated prognostic favorable (7.87e-5)	unprognostic (1.34e-1)	validated prognostic favorable (1.53e-5)	validated prognostic favorable (2.80e-4)	unprognostic (7.58e-2)
potential prognostic favorable (1.76e-5)	unprognostic (1.36e-2)	unprognostic (1.91e-2)	validated prognostic unfavorable (8.23e-8)	validated prognostic unfavorable (1.25e-8)	potential prognostic unfavorable (4.92e-4)
potential prognostic unfavorable (6.20e-4)	unprognostic (3.11e-3)	unprognostic (1.61e-2)	unprognostic (1.03e-3)	unprognostic (1.45e-1)	unprognostic (8.18e-2)
unprognostic (4.88e-3)	unprognostic (6.38e-2)	unprognostic (1.72e-2)	unprognostic (5.00e-2)	unprognostic (9.82e-2)	unprognostic (1.01e-2)
		unprognostic (1.83e-1)			
potential prognostic favorable (9.99e-7)	unprognostic (4.92e-2)	unprognostic (5.50e-2)	unprognostic (3.08e-2)	unprognostic (1.37e-1)	unprognostic (4.10e-1)
validated prognostic favorable (4.67e-10)	validated prognostic favorable (3.24e-5)	unprognostic (2.14e-2)	unprognostic (5.27e-2)	unprognostic (6.44e-2)	unprognostic (4.63e-2)
potential prognostic favorable (1.92e-6)	unprognostic (1.59e-2)	unprognostic (7.03e-2)	unprognostic (1.01e-3)	unprognostic (1.89e-2)	unprognostic (1.07e-1)
potential prognostic favorable (2.61e-5)	unprognostic (4.08e-2)	unprognostic (1.48e-2)		unprognostic (1.00e-1)	unprognostic (9.99e-2)
potential prognostic favorable (1.82e-10)	unprognostic (3.32e-3)	unprognostic (2.16e-2)	unprognostic (2.87e-1)	unprognostic (9.15e-2)	potential prognostic favorable (6.54e-4)
potential prognostic favorable (3.91e-4)	unprognostic (4.55e-2)	unprognostic (3.08e-2)	unprognostic (1.83e-1)	unprognostic (5.00e-2)	unprognostic (9.92e-2)
validated prognostic favorable (3.23e-5)	validated prognostic favorable (6.42e-5)	unprognostic (5.34e-2)	unprognostic (4.66e-2)	unprognostic (3.51e-2)	unprognostic (3.45e-2)
unprognostic (7.47e-2)	unprognostic (6.37e-3)	potential prognostic unfavorable (4.79e-6)	unprognostic (2.15e-1)	unprognostic (2.23e-2)	unprognostic (7.61e-3)
unprognostic (5.84e-2)	unprognostic (1.27e-1)	unprognostic (8.00e-2)			unprognostic (1.50e-1)
unprognostic (2.24e-2)	unprognostic (9.05e-3)	unprognostic (6.22e-3)	unprognostic (1.39e-1)	unprognostic (6.17e-2)	potential prognostic unfavorable (3.18e-4)
potential prognostic unfavorable (1.87e-4)	unprognostic (1.99e-1)	unprognostic (4.77e-1)	unprognostic (6.64e-2)	unprognostic (5.72e-2)	unprognostic (7.62e-2)
potential prognostic favorable (1.76e-8)	unprognostic (1.16e-3)	unprognostic (5.15e-3)	unprognostic (8.44e-2)	unprognostic (5.43e-2)	unprognostic (3.03e-1)
validated prognostic favorable (6.64e-5)	validated prognostic favorable (4.22e-4)	unprognostic (4.51e-2)	potential prognostic unfavorable (2.29e-4)	unprognostic (3.71e-3)	unprognostic (1.76e-1)
potential prognostic favorable (8.24e-7)	unprognostic (4.09e-2)	unprognostic (2.08e-1)	unprognostic (1.22e-1)		unprognostic (1.95e-2)
potential prognostic favorable (1.05e-5)	unprognostic (3.21e-1)	unprognostic (1.00e-1)	unprognostic (8.50e-2)	unprognostic (5.44e-3)	unprognostic (1.59e-2)
unprognostic (4.76e-2)	unprognostic (1.82e-1)	unprognostic (2.34e-1)	unprognostic (1.23e-1)	unprognostic (1.59e-1)	unprognostic (1.71e-2)
potential prognostic favorable (1.21e-10)	unprognostic (1.02e-3)	potential prognostic favorable (1.01e-4)	unprognostic (9.77e-3)	unprognostic (6.42e-2)	unprognostic (5.78e-2)
potential prognostic favorable (8.87e-6)	unprognostic (5.87e-2)	unprognostic (1.95e-1)	unprognostic (1.82e-1)	unprognostic (1.52e-1)	unprognostic (1.59e-1)
validated prognostic favorable (4.41e-8)	validated prognostic favorable (2.50e-6)	unprognostic (3.23e-2)	unprognostic (1.72e-1)	unprognostic (6.54e-2)	unprognostic (1.38e-2)
potential prognostic favorable (1.96e-5)	unprognostic (1.11e-1)	unprognostic (2.77e-1)	unprognostic (1.16e-1)	unprognostic (2.03e-3)	unprognostic (4.06e-2)
potential prognostic unfavorable (2.84e-4)	unprognostic (8.13e-3)	potential prognostic unfavorable (3.88e-4)	unprognostic (3.83e-2)	unprognostic (2.13e-1)	unprognostic (1.89e-1)
unprognostic (2.58e-1)	unprognostic (6.62e-2)	unprognostic (4.27e-2)	unprognostic (6.08e-2)	unprognostic (2.31e-2)	unprognostic (4.52e-2)
potential prognostic unfavorable (3.55e-4)	unprognostic (5.06e-2)	unprognostic (1.09e-2)		unprognostic (8.01e-2)	unprognostic (5.38e-2)
unprognostic (5.95e-3)	unprognostic (1.16e-1)	unprognostic (1.87e-1)	unprognostic (2.51e-2)	unprognostic (5.11e-2)	unprognostic (1.49e-1)
unprognostic (1.68e-2)	unprognostic (2.65e-1)	unprognostic (9.49e-2)	unprognostic (1.31e-2)	unprognostic (1.51e-1)	unprognostic (8.99e-2)
potential prognostic favorable (3.15e-5)	unprognostic (4.52e-2)	unprognostic (8.58e-2)	unprognostic (3.69e-2)	unprognostic (1.32e-2)	unprognostic (2.17e-2)
potential prognostic favorable (7.92e-4)	unprognostic (1.38e-1)	unprognostic (1.03e-2)	potential prognostic favorable (3.00e-4)		potential prognostic favorable (8.47e-4)
	unprognostic (2.93e-1)	unprognostic (7.64e-2)			unprognostic (5.70e-3)
unprognostic (5.54e-2)	unprognostic (2.09e-3)	potential prognostic unfavorable (6.91e-4)	unprognostic (8.40e-3)	unprognostic (4.53e-2)	unprognostic (1.96e-2)
validated prognostic favorable (2.60e-9)	validated prognostic favorable (6.45e-5)	unprognostic (1.26e-1)	unprognostic (2.04e-1)	unprognostic (3.24e-2)	unprognostic (2.05e-3)
potential prognostic favorable (2.27e-4)	unprognostic (7.78e-2)	potential prognostic favorable (8.31e-4)	unprognostic (1.51e-1)	potential prognostic favorable (9.08e-4)	unprognostic (2.66e-3)
unprognostic (3.52e-3)	unprognostic (3.59e-2)	potential prognostic favorable (2.52e-4)	unprognostic (6.24e-3)	unprognostic (6.68e-2)	unprognostic (6.47e-2)
potential prognostic favorable (1.77e-4)	unprognostic (4.01e-1)	unprognostic (2.26e-1)	validated prognostic unfavorable (7.93e-4)	validated prognostic unfavorable (8.07e-4)	unprognostic (2.46e-2)
potential prognostic favorable (2.58e-7)	unprognostic (7.55e-2)	unprognostic (2.41e-1)	unprognostic (1.81e-3)	potential prognostic unfavorable (1.14e-4)	unprognostic (1.97e-1)
validated prognostic favorable (2.15e-10)	validated prognostic favorable (4.93e-4)	unprognostic (1.29e-3)	unprognostic (2.84e-2)	unprognostic (4.20e-2)	unprognostic (2.31e-2)
validated prognostic favorable (2.61e-8)	validated prognostic favorable (3.90e-4)	unprognostic (1.78e-3)	unprognostic (7.66e-2)	unprognostic (1.43e-1)	unprognostic (4.42e-3)
validated prognostic favorable (5.61e-6)	validated prognostic favorable (8.45e-5)	unprognostic (8.98e-2)	unprognostic (4.80e-2)	potential prognostic favorable (2.55e-4)	unprognostic (1.80e-1)
validated prognostic favorable (3.50e-4)	validated prognostic favorable (6.55e-4)	unprognostic (1.24e-1)	unprognostic (1.64e-2)	unprognostic (8.82e-2)	unprognostic (3.85e-3)
unprognostic (4.44e-3)	unprognostic (4.86e-2)	unprognostic (5.72e-2)	unprognostic (3.54e-2)	unprognostic (1.36e-1)	unprognostic (2.36e-2)
unprognostic (9.26e-3)	unprognostic (4.23e-1)	unprognostic (1.79e-1)	unprognostic (1.41e-1)	unprognostic (1.19e-1)	unprognostic (2.69e-2)
unprognostic (1.80e-3)	unprognostic (3.95e-2)	unprognostic (9.97e-2)	potential prognostic unfavorable (4.34e-6)	unprognostic (8.16e-2)	unprognostic (4.56e-3)
validated prognostic favorable (1.08e-5)	validated prognostic favorable (3.70e-5)	unprognostic (8.27e-3)	validated prognostic favorable (7.38e-4)	validated prognostic favorable (5.08e-5)	unprognostic (8.88e-3)
potential prognostic favorable (5.19e-12)	unprognostic (7.92e-3)	unprognostic (5.22e-3)	unprognostic (1.48e-2)	unprognostic (3.37e-1)	unprognostic (6.45e-3)
validated prognostic favorable (1.58e-8)	validated prognostic favorable (2.01e-4)	potential prognostic favorable (5.49e-4)	unprognostic (2.17e-1)	unprognostic (1.96e-2)	unprognostic (7.14e-3)
unprognostic (4.62e-2)	unprognostic (1.77e-1)	unprognostic (1.18e-1)	unprognostic (7.09e-3)	unprognostic (4.79e-3)	unprognostic (2.77e-1)
validated prognostic favorable (6.27e-7)	validated prognostic favorable (5.86e-4)	unprognostic (2.46e-1)	unprognostic (1.69e-2)	unprognostic (2.62e-1)	potential prognostic favorable (8.37e-4)
potential prognostic unfavorable (7.31e-7)	unprognostic (2.47e-2)	unprognostic (2.53e-1)		potential prognostic favorable (5.06e-5)	unprognostic (1.22e-2)
potential prognostic unfavorable (3.31e-5)	unprognostic (2.74e-3)	unprognostic (2.57e-2)	validated prognostic unfavorable (3.95e-6)	validated prognostic unfavorable (8.80e-10)	validated prognostic unfavorable (1.61e-7)
potential prognostic favorable (3.38e-4)	unprognostic (6.28e-2)	unprognostic (1.77e-1)			potential prognostic favorable (9.05e-4)
validated prognostic favorable (5.72e-7)	validated prognostic favorable (1.32e-4)	unprognostic (5.55e-2)	unprognostic (9.55e-2)	unprognostic (7.68e-2)	unprognostic (4.03e-2)
potential prognostic favorable (6.37e-7)	unprognostic (4.25e-2)	potential prognostic favorable (1.79e-7)	unprognostic (1.70e-1)	unprognostic (1.84e-1)	unprognostic (1.78e-1)
					potential prognostic favorable (2.00e-4)
validated prognostic favorable (2.53e-10)	validated prognostic favorable (1.24e-4)	unprognostic (2.09e-1)	validated prognostic favorable (3.06e-6)	validated prognostic favorable (1.71e-4)	
validated prognostic favorable (9.07e-5)	validated prognostic favorable (4.00e-4)	unprognostic (1.63e-1)	unprognostic (1.83e-2)		unprognostic (1.23e-2)
potential prognostic favorable (3.97e-5)	unprognostic (3.94e-3)	unprognostic (2.09e-1)	unprognostic (7.45e-2)	unprognostic (1.93e-1)	unprognostic (4.58e-2)
		unprognostic (2.35e-2)		unprognostic (3.26e-1)	potential prognostic unfavorable (6.18e-8)
		unprognostic (9.41e-3)			unprognostic (2.42e-3)
	unprognostic (1.56e-2)	unprognostic (4.44e-3)		unprognostic (3.34e-3)	unprognostic (1.90e-2)
unprognostic (5.96e-3)	potential prognostic favorable (2.88e-4)	potential prognostic unfavorable (5.78e-6)	unprognostic (3.46e-2)	unprognostic (2.51e-1)	unprognostic (2.20e-2)
unprognostic (1.46e-1)	unprognostic (1.57e-1)	unprognostic (1.53e-1)			unprognostic (3.81e-2)
unprognostic (1.46e-1)		unprognostic (1.66e-2)	potential prognostic unfavorable (2.59e-4)	unprognostic (2.08e-2)	unprognostic (5.66e-2)
validated prognostic favorable (1.37e-5)	validated prognostic favorable (6.28e-4)	unprognostic (3.19e-2)	validated prognostic unfavorable (4.65e-4)	validated prognostic unfavorable (2.84e-5)	unprognostic (1.94e-1)
potential prognostic favorable (7.04e-4)	unprognostic (5.58e-3)	unprognostic (3.41e-1)	unprognostic (3.18e-3)	unprognostic (1.05e-1)	unprognostic (1.62e-1)
validated prognostic favorable (6.35e-7)	validated prognostic favorable (2.30e-4)	unprognostic (1.34e-1)	unprognostic (1.02e-1)	unprognostic (8.43e-2)	unprognostic (2.27e-2)
unprognostic (7.49e-3)	unprognostic (3.03e-2)	unprognostic (5.53e-2)	unprognostic (4.92e-3)	unprognostic (3.84e-3)	unprognostic (2.11e-2)
potential prognostic favorable (1.72e-5)	unprognostic (6.26e-2)	unprognostic (3.55e-2)	unprognostic (4.62e-3)	potential prognostic unfavorable (1.71e-5)	unprognostic (1.16e-1)
unprognostic (1.27e-2)	potential prognostic unfavorable (3.26e-4)	unprognostic (1.28e-2)	potential prognostic unfavorable (3.79e-6)	unprognostic (1.44e-2)	potential prognostic unfavorable (2.87e-4)
unprognostic (3.76e-2)	unprognostic (3.55e-2)	unprognostic (2.81e-3)	unprognostic (6.59e-2)	unprognostic (4.97e-3)	unprognostic (2.25e-3)
unprognostic (7.28e-2)	unprognostic (6.95e-3)	unprognostic (1.63e-1)	unprognostic (1.36e-1)	potential prognostic favorable (5.45e-4)	unprognostic (2.03e-2)
potential prognostic favorable (1.23e-8)	unprognostic (1.17e-2)	unprognostic (9.73e-3)	unprognostic (1.06e-2)	unprognostic (6.81e-2)	unprognostic (1.40e-2)
unprognostic (1.60e-1)	unprognostic (4.41e-1)			unprognostic (2.23e-2)	unprognostic (1.26e-3)
unprognostic (3.18e-1)	unprognostic (2.84e-1)	unprognostic (9.90e-2)	unprognostic (1.07e-1)	unprognostic (1.88e-2)	unprognostic (5.47e-3)

unprognostic (1.68e-2)	potential prognostic favorable (2.33e-5)				
				unprognostic (7.27e-2)	
potential prognostic unfavorable (8.40e-10)	unprognostic (1.65e-3)	potential prognostic unfavorable (1.57e-5)		unprognostic (1.68e-1)	unprognostic (1.67e-2)
validated prognostic favorable (6.79e-6)	validated prognostic favorable (7.97e-5)	unprognostic (5.85e-3)	unprognostic (2.66e-3)	unprognostic (7.99e-2)	unprognostic (1.22e-2)
unprognostic (1.34e-3)	unprognostic (3.73e-3)	unprognostic (1.07e-3)	validated prognostic unfavorable (9.75e-6)	validated prognostic unfavorable (7.18e-4)	potential prognostic unfavorable (1.55e-5)
potential prognostic unfavorable (5.73e-4)	unprognostic (1.63e-1)	potential prognostic unfavorable (2.57e-4)	potential prognostic unfavorable (1.40e-4)	unprognostic (3.97e-2)	unprognostic (2.05e-3)
potential prognostic unfavorable (9.44e-4)	unprognostic (8.42e-2)	unprognostic (4.10e-3)	unprognostic (1.79e-3)	unprognostic (3.36e-1)	unprognostic (2.80e-3)
potential prognostic favorable (2.10e-4)	unprognostic (1.49e-2)	unprognostic (2.98e-1)	unprognostic (6.68e-2)	unprognostic (5.05e-3)	unprognostic (3.43e-1)
		unprognostic (5.06e-3)			unprognostic (1.66e-1)
unprognostic (1.59e-3)	potential prognostic favorable (6.97e-5)	potential prognostic unfavorable (9.27e-4)	unprognostic (2.98e-2)	unprognostic (8.99e-2)	unprognostic (1.51e-1)
				unprognostic (4.27e-1)	
unprognostic (3.76e-2)					unprognostic (1.75e-2)
validated prognostic unfavorable (1.96e-8)	validated prognostic unfavorable (8.71e-7)	potential prognostic unfavorable (1.77e-8)	validated prognostic unfavorable (2.08e-8)	validated prognostic unfavorable (1.20e-5)	validated prognostic unfavorable (4.82e-6)
potential prognostic favorable (2.09e-4)	unprognostic (7.37e-3)	unprognostic (1.90e-1)	unprognostic (1.25e-1)	unprognostic (3.16e-2)	unprognostic (2.35e-2)
unprognostic (1.96e-1)	unprognostic (2.75e-1)	unprognostic (4.18e-2)	potential prognostic unfavorable (1.20e-8)	unprognostic (1.71e-2)	unprognostic (4.93e-1)
unprognostic (1.91e-3)	unprognostic (6.78e-2)			unprognostic (2.23e-1)	unprognostic (1.61e-2)
unprognostic (2.97e-2)	unprognostic (4.77e-1)	unprognostic (4.09e-2)	unprognostic (1.99e-2)	unprognostic (1.94e-1)	unprognostic (1.44e-2)
unprognostic (9.19e-3)	unprognostic (5.49e-2)	unprognostic (1.54e-1)	unprognostic (4.94e-1)	unprognostic (2.80e-2)	unprognostic (9.90e-3)
potential prognostic favorable (1.21e-5)	unprognostic (9.40e-3)	unprognostic (2.48e-1)	unprognostic (2.91e-1)	unprognostic (2.46e-1)	unprognostic (9.41e-2)
unprognostic (1.51e-1)	unprognostic (1.73e-1)	unprognostic (1.38e-1)	unprognostic (3.38e-1)		unprognostic (4.62e-2)
potential prognostic favorable (4.84e-4)	unprognostic (2.45e-1)	unprognostic (7.35e-2)	potential prognostic unfavorable (6.90e-4)		unprognostic (2.10e-3)
unprognostic (2.67e-3)	unprognostic (3.62e-2)	unprognostic (4.22e-2)	unprognostic (3.06e-2)		unprognostic (1.12e-2)
potential prognostic favorable (1.29e-6)	unprognostic (4.52e-2)	unprognostic (9.29e-2)	unprognostic (5.59e-2)	unprognostic (9.38e-2)	unprognostic (3.51e-2)
unprognostic (2.38e-3)	unprognostic (8.86e-2)	unprognostic (2.88e-2)	potential prognostic unfavorable (2.20e-5)	unprognostic (1.05e-1)	unprognostic (8.22e-2)
unprognostic (2.30e-1)	unprognostic (2.80e-1)	unprognostic (2.02e-1)		unprognostic (2.39e-1)	unprognostic (1.43e-3)
potential prognostic favorable (3.74e-6)	unprognostic (9.79e-3)	potential prognostic unfavorable (4.28e-4)	validated prognostic unfavorable (3.93e-4)	validated prognostic unfavorable (2.93e-4)	unprognostic (3.90e-2)
unprognostic (7.75e-2)	potential prognostic unfavorable (8.89e-4)	unprognostic (1.24e-2)	unprognostic (6.90e-3)	unprognostic (1.07e-3)	unprognostic (2.91e-1)
	unprognostic (9.57e-2)				
unprognostic (1.05e-1)	unprognostic (1.16e-1)	unprognostic (5.87e-2)	unprognostic (7.90e-2)	unprognostic (1.83e-3)	unprognostic (2.44e-1)
unprognostic (2.13e-1)	unprognostic (1.36e-1)	unprognostic (9.36e-2)	potential prognostic unfavorable (6.47e-5)	unprognostic (3.04e-2)	unprognostic (1.97e-2)
potential prognostic favorable (2.14e-4)	unprognostic (3.51e-3)	unprognostic (3.88e-2)	unprognostic (1.89e-2)	unprognostic (3.74e-3)	unprognostic (3.06e-3)
unprognostic (8.81e-2)	unprognostic (7.04e-3)	unprognostic (6.07e-2)	unprognostic (1.83e-1)	unprognostic (1.46e-2)	unprognostic (1.61e-2)
potential prognostic favorable (9.32e-8)	unprognostic (1.85e-3)	unprognostic (5.93e-2)	unprognostic (1.37e-2)	unprognostic (5.30e-3)	unprognostic (2.03e-1)
unprognostic (2.04e-3)	unprognostic (3.38e-1)	potential prognostic unfavorable (2.43e-5)	unprognostic (1.06e-1)	unprognostic (8.64e-3)	unprognostic (9.45e-2)
potential prognostic favorable (4.69e-4)	unprognostic (1.87e-1)	unprognostic (2.40e-1)	unprognostic (1.32e-1)	unprognostic (4.70e-2)	unprognostic (1.54e-1)
validated prognostic favorable (3.89e-9)	validated prognostic favorable (2.90e-4)	unprognostic (1.95e-1)		unprognostic (4.72e-2)	unprognostic (5.61e-2)
potential prognostic unfavorable (1.02e-5)	unprognostic (1.09e-1)	unprognostic (6.83e-2)	unprognostic (1.90e-1)	unprognostic (1.55e-1)	unprognostic (6.53e-3)
unprognostic (9.06e-2)	unprognostic (3.28e-1)	unprognostic (1.79e-1)	unprognostic (1.26e-1)	unprognostic (2.16e-2)	unprognostic (3.93e-3)
potential prognostic unfavorable (1.75e-5)	unprognostic (4.15e-1)	potential prognostic unfavorable (8.57e-6)	validated prognostic unfavorable (7.22e-6)	validated prognostic unfavorable (1.91e-4)	unprognostic (5.30e-2)
potential prognostic favorable (2.03e-6)	unprognostic (2.56e-3)	unprognostic (3.84e-3)	unprognostic (9.72e-3)	unprognostic (3.47e-1)	unprognostic (2.13e-2)
potential prognostic favorable (1.56e-5)	unprognostic (5.69e-3)	unprognostic (1.45e-1)			unprognostic (5.93e-2)
unprognostic (1.62e-2)	unprognostic (1.23e-1)	unprognostic (1.33e-2)	unprognostic (6.30e-2)	unprognostic (2.15e-1)	unprognostic (1.15e-1)
unprognostic (1.97e-1)	unprognostic (8.28e-2)	unprognostic (2.76e-1)	unprognostic (7.42e-2)	potential prognostic favorable (2.13e-4)	unprognostic (7.35e-2)
potential prognostic favorable (3.47e-5)	unprognostic (6.75e-3)	unprognostic (1.20e-1)	unprognostic (7.66e-2)	unprognostic (2.13e-3)	unprognostic (4.31e-3)
unprognostic (1.25e-1)	unprognostic (1.04e-2)	unprognostic (1.22e-1)	unprognostic (6.18e-3)	unprognostic (2.22e-2)	unprognostic (2.20e-3)
potential prognostic favorable (3.08e-7)	unprognostic (2.16e-2)	unprognostic (5.52e-2)	unprognostic (7.88e-2)	unprognostic (3.19e-1)	unprognostic (3.42e-1)
unprognostic (1.10e-3)	unprognostic (2.05e-2)	unprognostic (2.30e-1)	unprognostic (4.04e-3)	unprognostic (2.43e-2)	unprognostic (4.23e-1)
potential prognostic favorable (6.07e-4)	unprognostic (5.51e-2)	unprognostic (7.36e-2)	unprognostic (3.01e-2)	unprognostic (3.32e-3)	unprognostic (4.09e-2)
unprognostic (6.02e-2)	unprognostic (3.16e-1)	unprognostic (3.37e-1)	unprognostic (4.36e-2)	unprognostic (8.16e-2)	unprognostic (3.47e-2)
potential prognostic favorable (4.33e-4)	unprognostic (1.02e-1)	unprognostic (1.79e-2)	unprognostic (2.23e-3)	unprognostic (8.77e-2)	validated prognostic unfavorable (1.05e-5)
unprognostic (1.78e-3)	unprognostic (2.33e-1)	unprognostic (3.55e-1)	unprognostic (1.18e-1)	unprognostic (2.03e-1)	unprognostic (1.74e-1)
unprognostic (1.20e-3)	unprognostic (2.55e-1)	unprognostic (1.55e-1)	unprognostic (4.08e-1)	unprognostic (8.90e-2)	unprognostic (1.38e-1)
potential prognostic unfavorable (6.61e-6)	unprognostic (1.89e-1)	unprognostic (3.45e-2)	unprognostic (4.45e-3)	unprognostic (6.51e-2)	potential prognostic unfavorable (1.75e-5)
unprognostic (5.71e-2)	unprognostic (1.12e-1)	unprognostic (2.24e-2)	unprognostic (4.19e-2)	unprognostic (3.16e-2)	unprognostic (4.52e-3)
	potential prognostic favorable (9.84e-6)	unprognostic (2.10e-2)	potential prognostic favorable (3.74e-4)	unprognostic (1.04e-3)	unprognostic (1.12e-1)
potential prognostic favorable (3.68e-6)	unprognostic (1.41e-3)	unprognostic (2.49e-3)	potential prognostic favorable (5.83e-4)	unprognostic (2.26e-3)	unprognostic (1.71e-2)
unprognostic (1.46e-2)	unprognostic (1.05e-1)	unprognostic (3.54e-2)	unprognostic (1.14e-1)		potential prognostic favorable (6.64e-4)
validated prognostic favorable (3.52e-8)	validated prognostic favorable (3.75e-4)	unprognostic (3.24e-2)	unprognostic (2.80e-1)	potential prognostic unfavorable (4.87e-5)	unprognostic (1.41e-2)
unprognostic (1.01e-3)	unprognostic (7.10e-2)	unprognostic (3.66e-2)	unprognostic (4.05e-1)	unprognostic (2.35e-1)	unprognostic (9.02e-3)
potential prognostic favorable (1.35e-4)	unprognostic (1.54e-2)	unprognostic (4.87e-1)	unprognostic (2.53e-2)		unprognostic (3.28e-2)
potential prognostic favorable (5.75e-4)	unprognostic (9.69e-2)	unprognostic (1.48e-1)	unprognostic (8.27e-3)		potential prognostic unfavorable (6.35e-5)
validated prognostic favorable (1.20e-8)	validated prognostic favorable (4.39e-5)	unprognostic (1.50e-2)	unprognostic (3.82e-2)	unprognostic (2.37e-2)	unprognostic (1.35e-3)
unprognostic (1.20e-3)	potential prognostic favorable (3.11e-4)	unprognostic (2.58e-1)	unprognostic (1.74e-3)	unprognostic (1.86e-2)	unprognostic (1.93e-2)
potential prognostic unfavorable (1.22e-4)	unprognostic (2.30e-2)			potential prognostic favorable (4.33e-4)	potential prognostic favorable (4.67e-5)
validated prognostic unfavorable (1.54e-5)	validated prognostic unfavorable (1.44e-4)	unprognostic (1.87e-3)	unprognostic (2.28e-1)	unprognostic (1.72e-1)	unprognostic (4.97e-3)
potential prognostic favorable (9.37e-5)	unprognostic (1.16e-3)	unprognostic (9.03e-2)	unprognostic (1.29e-1)	unprognostic (1.49e-1)	unprognostic (3.50e-3)
potential prognostic unfavorable (2.09e-8)	unprognostic (2.30e-3)	unprognostic (1.21e-3)			
unprognostic (2.91e-1)	unprognostic (1.17e-1)	unprognostic (1.91e-2)	unprognostic (2.31e-1)	unprognostic (4.21e-2)	unprognostic (3.73e-1)
	unprognostic (1.51e-2)	unprognostic (2.90e-1)			
			unprognostic (1.49e-2)	unprognostic (1.35e-2)	
potential prognostic favorable (9.29e-4)	unprognostic (1.20e-2)	unprognostic (2.92e-2)	validated prognostic unfavorable (4.78e-6)	validated prognostic unfavorable (1.17e-4)	unprognostic (1.79e-2)
unprognostic (5.59e-2)	unprognostic (6.43e-3)				unprognostic (3.67e-2)
validated prognostic favorable (7.36e-5)	validated prognostic favorable (6.08e-5)	unprognostic (2.94e-1)		unprognostic (7.19e-3)	unprognostic (2.19e-3)
unprognostic (1.26e-1)	unprognostic (2.52e-2)	unprognostic (1.07e-1)	unprognostic (1.57e-3)	unprognostic (2.73e-1)	unprognostic (1.50e-2)
					unprognostic (1.29e-2)
unprognostic (1.39e-2)	unprognostic (3.41e-1)	potential prognostic unfavorable (9.13e-4)	unprognostic (5.88e-3)	unprognostic (1.87e-3)	unprognostic (2.92e-2)
validated prognostic unfavorable (7.43e-6)	validated prognostic unfavorable (1.01e-4)	unprognostic (1.68e-3)		unprognostic (1.34e-1)	potential prognostic unfavorable (5.33e-4)
unprognostic (1.24e-2)	unprognostic (9.98e-2)	unprognostic (2.97e-2)		unprognostic (1.16e-1)	unprognostic (8.67e-3)
potential prognostic favorable (1.14e-5)	unprognostic (1.56e-2)	unprognostic (6.74e-2)	unprognostic (4.02e-1)	unprognostic (7.73e-2)	unprognostic (1.33e-1)
potential prognostic favorable (1.35e-5)	unprognostic (2.99e-2)	potential prognostic favorable (6.09e-4)	validated prognostic unfavorable (5.38e-4)	validated prognostic unfavorable (5.49e-4)	unprognostic (1.84e-2)
potential prognostic unfavorable (1.29e-5)	unprognostic (8.78e-2)	unprognostic (6.20e-2)	unprognostic (9.10e-2)	unprognostic (3.13e-2)	unprognostic (1.81e-2)
unprognostic (3.78e-2)	unprognostic (2.17e-1)	unprognostic (2.04e-1)	unprognostic (2.84e-3)	unprognostic (2.53e-1)	unprognostic (1.63e-2)
unprognostic (1.70e-1)	unprognostic (3.83e-2)				unprognostic (7.12e-3)
unprognostic (6.51e-2)	unprognostic (2.73e-1)	unprognostic (5.43e-2)	unprognostic (1.82e-1)	unprognostic (2.44e-1)	unprognostic (3.43e-3)
potential prognostic favorable (5.17e-7)	unprognostic (3.92e-3)	unprognostic (1.75e-1)	unprognostic (2.26e-1)	unprognostic (3.74e-2)	unprognostic (1.92e-2)
unprognostic (1.01e-1)	potential prognostic favorable (3.55e-4)	potential prognostic unfavorable (1.96e-5)	unprognostic (4.98e-2)	unprognostic (1.48e-1)	unprognostic (5.65e-2)
unprognostic (1.90e-3)	unprognostic (1.28e-1)	unprognostic (2.49e-2)	unprognostic (3.61e-2)	unprognostic (1.86e-1)	unprognostic (5.74e-2)
potential prognostic favorable (7.08e-8)	unprognostic (5.70e-3)	unprognostic (1.88e-2)	unprognostic (1.21e-1)	unprognostic (7.06e-3)	unprognostic (7.69e-2)
					unprognostic (2.94e-3)
unprognostic (1.64e-1)	unprognostic (2.21e-2)	unprognostic (9.35e-2)	unprognostic (1.36e-2)	unprognostic (4.95e-3)	potential prognostic favorable (2.44e-5)
potential prognostic favorable (3.76e-6)	unprognostic (1.15e-2)	unprognostic (2.20e-1)	unprognostic (5.24e-3)	unprognostic (2.86e-1)	unprognostic (6.97e-2)
potential prognostic favorable (4.04e-4)	unprognostic (1.95e-1)	unprognostic (6.28e-2)	unprognostic (1.21e-1)	unprognostic (1.94e-3)	unprognostic (1.07e-1)
					unprognostic (1.99e-2)
unprognostic (6.72e-3)	unprognostic (1.51e-1)	unprognostic (1.15e-1)	unprognostic (1.58e-1)	unprognostic (1.59e-1)	unprognostic (6.32e-3)
unprognostic (9.16e-3)	unprognostic (1.74e-1)	unprognostic (2.87e-2)	unprognostic (9.19e-2)	unprognostic (9.82e-2)	unprognostic (5.09e-3)
unprognostic (8.91e-3)	unprognostic (1.92e-1)			unprognostic (4.56e-2)	
potential prognostic favorable (6.64e-4)	unprognostic (1.79e-1)	unprognostic (9.35e-3)	unprognostic (9.32e-3)	unprognostic (1.05e-1)	unprognostic (3.29e-1)
potential prognostic favorable (1.67e-5)	unprognostic (1.84e-2)	unprognostic (1.68e-2)	unprognostic (5.64e-3)	unprognostic (1.02e-1)	unprognostic (1.20e-1)
unprognostic (2.88e-3)	unprognostic (3.08e-3)	unprognostic (2.91e-2)	unprognostic (4.23e-2)	unprognostic (2.66e-1)	unprognostic (3.12e-1)
validated prognostic favorable (9.05e-6)	validated prognostic favorable (1.41e-4)	unprognostic (6.81e-3)	unprognostic (9.25e-3)	unprognostic (5.95e-3)	unprognostic (1.09e-2)
validated prognostic favorable (3.99e-7)	validated prognostic favorable (8.74e-5)	unprognostic (4.21e-1)	unprognostic (2.54e-1)	unprognostic (8.74e-2)	unprognostic (7.87e-3)
potential prognostic favorable (8.43e-4)	unprognostic (1.38e-3)	unprognostic (2.21e-1)	unprognostic (4.82e-2)	unprognostic (3.44e-2)	unprognostic (1.10e-2)
unprognostic (6.42e-2)	unprognostic (1.22e-1)	unprognostic (1.82e-1)	potential prognostic unfavorable (6.81e-5)	unprognostic (4.18e-2)	unprognostic (2.31e-2)
potential prognostic favorable (6.59e-8)	unprognostic (9.65e-2)	unprognostic (9.76e-3)	unprognostic (1.53e-2)	unprognostic (4.54e-2)	unprognostic (7.82e-3)
potential prognostic favorable (6.36e-9)	unprognostic (1.27e-3)	unprognostic (7.13e-2)	potential prognostic unfavorable (6.92e-5)	unprognostic (5.22e-2)	
potential prognostic favorable (3.38e-4)	unprognostic (9.98e-2)	unprognostic (8.23e-2)	validated prognostic unfavorable (1.23e-9)	validated prognostic unfavorable (8.92e-5)	unprognostic (6.66e-3)
potential prognostic favorable (2.47e-6)	unprognostic (3.83e-2)	unprognostic (3.47e-2)	potential prognostic unfavorable (2.43e-4)	unprognostic (2.48e-1)	unprognostic (2.33e-2)
validated prognostic favorable (1.21e-8)	validated prognostic favorable (2.86e-5)	unprognostic (2.11e-1)	unprognostic (3.88e-1)	unprognostic (3.36e-1)	unprognostic (2.64e-2)
unprognostic (1.81e-3)	potential prognostic favorable (7.26e-4)	unprognostic (2.45e-1)	unprognostic (6.80e-3)	unprognostic (9.00e-2)	unprognostic (7.24e-2)
					unprognostic (4.41e-3)

potential prognostic favorable (1.04e-6)	unprognostic (1.65e-1)	unprognostic (6.02e-2)	unprognostic (1.85e-2)	unprognostic (2.23e-2)	unprognostic (1.84e-1)
potential prognostic unfavorable (5.88e-4)	unprognostic (5.76e-2)	unprognostic (2.99e-1)	unprognostic (1.10e-1)	unprognostic (8.82e-3)	unprognostic (1.17e-1)
potential prognostic favorable (2.36e-6)	unprognostic (6.69e-3)	unprognostic (3.02e-1)	unprognostic (7.89e-3)	unprognostic (6.29e-3)	unprognostic (1.14e-2)
validated prognostic favorable (1.02e-8)	validated prognostic favorable (1.37e-4)	potential prognostic favorable (1.23e-4)	unprognostic (4.51e-2)	unprognostic (4.81e-2)	unprognostic (7.19e-3)
potential prognostic unfavorable (1.87e-5)	unprognostic (5.12e-3)	unprognostic (3.93e-1)	unprognostic (5.25e-2)	unprognostic (2.11e-3)	unprognostic (1.19e-3)
unprognostic (2.20e-3)	unprognostic (2.19e-2)	unprognostic (3.49e-1)	potential prognostic unfavorable (1.05e-4)	unprognostic (7.05e-2)	unprognostic (1.92e-2)
unprognostic (5.09e-2)	unprognostic (1.82e-2)	unprognostic (9.45e-2)	unprognostic (6.43e-2)		unprognostic (3.90e-1)
unprognostic (7.81e-3)	unprognostic (2.94e-1)	unprognostic (2.24e-1)	unprognostic (1.57e-2)		potential prognostic unfavorable (2.69e-4)
unprognostic (7.01e-3)	unprognostic (7.01e-2)	unprognostic (1.75e-2)	unprognostic (3.67e-2)	unprognostic (1.70e-1)	potential prognostic unfavorable (5.61e-4)
potential prognostic favorable (3.40e-4)	unprognostic (2.64e-2)	unprognostic (2.59e-2)	unprognostic (1.41e-1)	unprognostic (2.37e-2)	unprognostic (3.78e-1)
unprognostic (6.11e-3)	unprognostic (3.71e-1)			unprognostic (3.49e-2)	unprognostic (1.78e-1)
		potential prognostic favorable (1.69e-4)			
potential prognostic favorable (1.82e-9)	unprognostic (7.18e-2)	unprognostic (7.43e-2)	unprognostic (6.94e-2)	unprognostic (1.55e-1)	unprognostic (8.79e-2)
potential prognostic favorable (7.31e-8)	unprognostic (4.01e-2)	unprognostic (1.02e-1)	unprognostic (1.43e-3)	unprognostic (4.27e-2)	unprognostic (3.45e-3)
unprognostic (3.62e-3)	potential prognostic unfavorable (3.30e-6)	unprognostic (8.00e-2)	potential prognostic unfavorable (7.77e-5)	unprognostic (5.46e-3)	unprognostic (2.49e-3)
unprognostic (1.84e-2)	unprognostic (2.91e-1)	unprognostic (2.26e-1)	unprognostic (6.11e-2)	unprognostic (2.55e-1)	unprognostic (4.22e-2)
unprognostic (1.99e-1)	unprognostic (1.86e-1)	unprognostic (2.96e-3)	unprognostic (2.41e-1)	potential prognostic favorable (5.99e-4)	unprognostic (1.57e-1)
unprognostic (6.54e-3)	unprognostic (9.61e-2)			unprognostic (1.54e-2)	potential prognostic favorable (1.29e-4)
unprognostic (1.45e-2)	unprognostic (1.86e-1)	potential prognostic favorable (6.79e-4)	unprognostic (8.03e-2)	unprognostic (1.33e-1)	unprognostic (3.22e-2)
validated prognostic favorable (2.34e-5)	validated prognostic favorable (6.25e-4)	unprognostic (2.52e-2)	unprognostic (1.26e-2)	unprognostic (9.30e-2)	unprognostic (1.51e-1)
potential prognostic favorable (1.58e-7)	unprognostic (6.22e-2)	unprognostic (5.71e-2)	unprognostic (2.20e-1)	unprognostic (2.27e-1)	unprognostic (1.30e-1)
potential prognostic unfavorable (6.35e-4)	unprognostic (2.30e-2)	unprognostic (1.09e-1)	unprognostic (3.90e-3)	unprognostic (2.60e-2)	unprognostic (3.67e-1)
unprognostic (3.54e-3)	potential prognostic favorable (1.59e-4)	potential prognostic unfavorable (5.83e-4)	potential prognostic favorable (2.96e-6)	unprognostic (8.18e-2)	unprognostic (5.12e-2)
potential prognostic favorable (2.06e-6)	unprognostic (1.38e-2)	unprognostic (1.09e-3)	unprognostic (4.40e-2)	unprognostic (1.15e-3)	unprognostic (9.43e-2)
potential prognostic favorable (6.33e-4)	unprognostic (1.88e-3)	unprognostic (2.98e-1)	unprognostic (5.56e-2)	potential prognostic favorable (3.92e-4)	unprognostic (2.86e-1)
unprognostic (1.16e-3)	unprognostic (1.49e-3)	potential prognostic unfavorable (6.30e-4)	unprognostic (4.48e-2)	potential prognostic favorable (1.97e-7)	unprognostic (1.79e-1)
potential prognostic favorable (6.17e-6)	unprognostic (8.69e-3)	unprognostic (1.47e-1)	unprognostic (2.29e-1)	unprognostic (1.13e-1)	unprognostic (1.26e-2)
unprognostic (1.93e-3)	unprognostic (2.31e-1)	unprognostic (1.45e-1)		unprognostic (3.70e-2)	unprognostic (2.24e-1)
potential prognostic favorable (4.25e-5)	unprognostic (1.51e-1)	unprognostic (1.38e-1)	unprognostic (2.67e-2)	unprognostic (1.89e-1)	unprognostic (5.30e-3)
					unprognostic (8.02e-2)
unprognostic (4.20e-2)	unprognostic (2.29e-1)	unprognostic (1.32e-2)	unprognostic (8.61e-2)	unprognostic (9.03e-3)	unprognostic (2.56e-2)
potential prognostic favorable (3.55e-8)	unprognostic (6.95e-2)	unprognostic (2.48e-1)	unprognostic (1.73e-1)	unprognostic (9.32e-3)	unprognostic (2.89e-1)
unprognostic (9.30e-3)	unprognostic (1.74e-1)	unprognostic (2.21e-1)	unprognostic (7.41e-2)		unprognostic (3.92e-3)
					unprognostic (1.27e-1)
unprognostic (4.28e-2)	unprognostic (2.64e-1)	unprognostic (1.80e-1)	unprognostic (5.04e-2)	unprognostic (7.94e-2)	unprognostic (7.68e-3)
unprognostic (1.55e-1)	potential prognostic unfavorable (7.13e-4)	unprognostic (2.50e-1)	unprognostic (3.77e-3)	unprognostic (4.11e-3)	unprognostic (7.68e-3)
		unprognostic (1.55e-2)			unprognostic (1.01e-1)
potential prognostic favorable (2.83e-8)	unprognostic (7.53e-3)	unprognostic (2.03e-1)	unprognostic (2.05e-2)	unprognostic (1.05e-1)	unprognostic (2.02e-1)
potential prognostic favorable (4.57e-5)	unprognostic (1.22e-3)	unprognostic (2.14e-1)		potential prognostic unfavorable (4.89e-5)	
potential prognostic favorable (2.97e-4)	unprognostic (1.54e-2)	unprognostic (1.82e-1)	unprognostic (1.46e-1)	unprognostic (2.67e-2)	unprognostic (6.20e-2)
potential prognostic favorable (5.60e-4)	unprognostic (4.02e-3)	potential prognostic favorable (1.21e-4)	unprognostic (3.49e-3)	unprognostic (4.81e-2)	unprognostic (2.10e-1)
unprognostic (1.26e-3)	potential prognostic favorable (3.07e-6)	unprognostic (2.19e-2)	unprognostic (1.16e-1)	potential prognostic favorable (6.12e-4)	unprognostic (4.25e-2)
unprognostic (1.90e-2)	unprognostic (3.27e-1)	potential prognostic favorable (5.60e-4)			unprognostic (1.21e-1)
unprognostic (4.89e-3)	unprognostic (9.03e-2)	unprognostic (1.41e-1)	unprognostic (5.37e-2)		unprognostic (4.14e-3)
unprognostic (4.65e-3)	unprognostic (7.27e-3)	unprognostic (1.56e-2)	unprognostic (5.96e-2)	unprognostic (8.47e-2)	unprognostic (1.02e-3)
potential prognostic unfavorable (9.72e-4)	unprognostic (1.18e-1)	unprognostic (1.93e-2)	potential prognostic unfavorable (5.34e-4)	unprognostic (2.67e-2)	unprognostic (1.32e-1)
potential prognostic favorable (1.12e-7)	unprognostic (8.11e-2)	unprognostic (1.70e-2)	unprognostic (2.97e-2)	unprognostic (1.29e-1)	unprognostic (5.63e-2)
		unprognostic (2.71e-1)			
potential prognostic favorable (7.88e-4)	unprognostic (6.60e-2)	unprognostic (1.11e-1)	unprognostic (1.26e-1)	unprognostic (4.48e-1)	unprognostic (1.08e-1)
unprognostic (3.65e-3)	unprognostic (5.08e-3)	potential prognostic unfavorable (9.86e-7)	unprognostic (2.63e-1)	unprognostic (2.34e-1)	unprognostic (3.57e-2)
				unprognostic (2.11e-2)	
potential prognostic favorable (3.94e-7)	unprognostic (6.93e-2)	unprognostic (7.26e-2)	validated prognostic unfavorable (1.92e-6)	validated prognostic unfavorable (2.91e-4)	potential prognostic unfavorable (5.75e-4)
potential prognostic favorable (2.14e-5)	unprognostic (2.03e-2)	unprognostic (1.14e-1)	unprognostic (1.36e-2)	unprognostic (2.09e-2)	unprognostic (3.67e-3)
	unprognostic (1.02e-1)				unprognostic (6.62e-2)
unprognostic (1.48e-3)	potential prognostic favorable (1.81e-4)	unprognostic (9.59e-2)			unprognostic (2.95e-2)
validated prognostic favorable (3.41e-7)	validated prognostic favorable (3.44e-5)	potential prognostic favorable (6.75e-4)	unprognostic (1.69e-1)	unprognostic (8.83e-3)	unprognostic (9.25e-3)
unprognostic (3.75e-3)	unprognostic (1.17e-2)	unprognostic (7.05e-3)	unprognostic (1.13e-1)	unprognostic (8.98e-3)	potential prognostic favorable (2.01e-4)
unprognostic (1.36e-3)	unprognostic (1.76e-1)	unprognostic (3.16e-1)	potential prognostic unfavorable (1.02e-4)	unprognostic (3.54e-1)	unprognostic (1.49e-3)
		unprognostic (1.67e-3)	unprognostic (3.29e-2)	unprognostic (1.67e-1)	unprognostic (8.54e-2)
unprognostic (2.10e-1)	unprognostic (1.38e-1)	unprognostic (3.69e-2)	unprognostic (4.98e-2)	unprognostic (1.81e-1)	unprognostic (8.86e-3)
unprognostic (2.51e-2)	unprognostic (4.55e-3)	unprognostic (2.27e-1)	potential prognostic unfavorable (1.11e-4)	unprognostic (2.00e-1)	potential prognostic unfavorable (3.66e-4)
			unprognostic (1.27e-2)	unprognostic (7.34e-2)	unprognostic (4.40e-3)
potential prognostic unfavorable (3.52e-4)	unprognostic (1.34e-1)	unprognostic (8.73e-3)	unprognostic (4.46e-3)	unprognostic (1.47e-2)	unprognostic (2.33e-1)
validated prognostic favorable (3.95e-7)	validated prognostic favorable (2.02e-5)	unprognostic (1.99e-3)	potential prognostic unfavorable (2.88e-4)	unprognostic (2.34e-2)	unprognostic (3.03e-1)
unprognostic (7.77e-3)	unprognostic (1.47e-1)	unprognostic (4.54e-2)	unprognostic (1.35e-2)	unprognostic (1.51e-1)	unprognostic (2.29e-2)
unprognostic (1.23e-2)	unprognostic (1.74e-1)	unprognostic (2.99e-1)	potential prognostic unfavorable (9.33e-7)	unprognostic (2.33e-2)	unprognostic (1.84e-2)
potential prognostic favorable (4.36e-6)	unprognostic (3.34e-1)	unprognostic (2.92e-1)	unprognostic (8.46e-3)	unprognostic (4.09e-2)	unprognostic (4.40e-3)
unprognostic (2.66e-2)	unprognostic (2.60e-1)	unprognostic (6.01e-2)	potential prognostic unfavorable (3.46e-4)	unprognostic (7.84e-3)	unprognostic (2.17e-3)
unprognostic (5.30e-3)	unprognostic (3.85e-2)	unprognostic (9.68e-2)	unprognostic (1.70e-1)	unprognostic (5.04e-2)	unprognostic (1.30e-1)
validated prognostic favorable (3.03e-5)	validated prognostic favorable (5.78e-5)	potential prognostic unfavorable (7.73e-4)	unprognostic (1.53e-1)		unprognostic (3.83e-2)
potential prognostic favorable (5.80e-10)	unprognostic (4.46e-3)	unprognostic (1.75e-1)	unprognostic (7.61e-3)	unprognostic (8.89e-2)	unprognostic (2.96e-2)
potential prognostic favorable (2.96e-4)	unprognostic (2.18e-2)	unprognostic (7.39e-3)	unprognostic (2.60e-2)	unprognostic (2.36e-1)	potential prognostic unfavorable (2.09e-4)
	potential prognostic unfavorable (9.15e-5)	potential prognostic unfavorable (7.90e-5)	unprognostic (5.42e-2)	unprognostic (7.80e-2)	unprognostic (1.35e-1)
unprognostic (1.81e-2)	unprognostic (1.78e-2)	unprognostic (7.54e-2)	unprognostic (2.16e-2)	unprognostic (6.16e-2)	unprognostic (1.49e-1)
potential prognostic favorable (9.59e-4)	unprognostic (2.33e-2)	unprognostic (2.31e-2)	validated prognostic unfavorable (1.07e-4)	validated prognostic unfavorable (8.27e-4)	unprognostic (1.08e-1)
validated prognostic favorable (1.51e-9)	validated prognostic favorable (2.29e-4)	unprognostic (8.01e-2)	unprognostic (1.68e-1)	unprognostic (4.29e-2)	unprognostic (2.67e-3)
unprognostic (9.24e-3)	unprognostic (1.27e-2)	unprognostic (5.35e-3)	unprognostic (1.67e-3)	unprognostic (1.89e-2)	unprognostic (2.14e-1)
unprognostic (5.94e-3)	unprognostic (3.67e-2)	unprognostic (8.94e-2)	unprognostic (2.93e-2)	unprognostic (1.86e-1)	unprognostic (1.05e-2)
validated prognostic favorable (1.46e-5)	validated prognostic favorable (2.34e-4)	unprognostic (1.45e-1)	unprognostic (2.43e-3)		unprognostic (1.73e-1)
validated prognostic favorable (1.87e-6)	validated prognostic favorable (2.56e-4)	unprognostic (1.03e-3)	validated prognostic unfavorable (2.07e-4)	validated prognostic unfavorable (7.57e-4)	unprognostic (4.91e-2)
unprognostic (2.17e-2)					potential prognostic unfavorable (1.29e-4)
unprognostic (8.83e-2)	unprognostic (2.43e-2)	unprognostic (4.58e-3)		unprognostic (3.18e-2)	unprognostic (5.89e-2)
unprognostic (3.08e-3)	unprognostic (5.09e-1)	unprognostic (3.69e-2)		unprognostic (1.62e-2)	unprognostic (7.89e-2)
potential prognostic unfavorable (2.11e-4)	unprognostic (6.66e-3)	unprognostic (3.56e-1)	validated prognostic unfavorable (1.72e-4)	validated prognostic unfavorable (2.06e-6)	unprognostic (7.15e-3)
unprognostic (1.96e-3)	unprognostic (2.05e-2)	unprognostic (1.94e-1)	unprognostic (1.44e-2)	unprognostic (1.71e-2)	unprognostic (2.72e-1)
potential prognostic favorable (2.53e-8)	unprognostic (2.01e-2)	unprognostic (6.58e-2)	unprognostic (2.29e-3)	unprognostic (9.25e-2)	unprognostic (1.01e-1)
validated prognostic favorable (5.66e-7)	validated prognostic favorable (8.31e-6)	unprognostic (2.06e-2)	unprognostic (3.00e-2)	unprognostic (7.19e-3)	unprognostic (1.03e-1)
validated prognostic favorable (5.03e-10)	validated prognostic favorable (5.33e-5)	unprognostic (2.16e-1)		unprognostic (5.23e-2)	unprognostic (1.29e-1)
unprognostic (3.55e-2)	unprognostic (3.37e-1)	unprognostic (1.09e-3)	unprognostic (7.37e-2)	unprognostic (4.97e-3)	unprognostic (1.86e-1)
unprognostic (4.78e-3)	unprognostic (1.93e-2)	unprognostic (1.83e-1)	unprognostic (2.23e-2)	unprognostic (1.70e-1)	unprognostic (1.50e-1)
potential prognostic favorable (2.26e-8)	unprognostic (3.60e-3)				unprognostic (1.33e-2)
validated prognostic favorable (1.17e-10)	validated prognostic favorable (6.17e-5)	potential prognostic unfavorable (7.07e-4)	unprognostic (2.02e-1)	unprognostic (8.94e-2)	unprognostic (1.09e-3)
potential prognostic favorable (5.25e-10)	unprognostic (1.53e-1)	unprognostic (2.89e-1)	unprognostic (3.11e-1)	unprognostic (2.21e-2)	unprognostic (2.28e-2)
unprognostic (7.05e-2)	unprognostic (1.52e-2)	unprognostic (1.82e-1)	unprognostic (7.48e-2)	unprognostic (8.51e-2)	unprognostic (8.26e-2)
unprognostic (1.61e-2)	unprognostic (6.55e-2)	unprognostic (2.01e-1)			potential prognostic favorable (9.44e-4)
unprognostic (2.14e-1)	unprognostic (4.47e-3)	unprognostic (7.85e-3)	unprognostic (4.14e-3)	unprognostic (2.20e-1)	unprognostic (9.03e-2)
validated prognostic favorable (1.63e-7)	validated prognostic favorable (4.33e-4)	unprognostic (3.04e-3)	unprognostic (3.83e-2)	unprognostic (1.01e-1)	unprognostic (1.49e-1)
potential prognostic favorable (1.56e-7)	unprognostic (1.13e-1)	unprognostic (1.28e-2)	unprognostic (7.17e-2)	unprognostic (2.39e-1)	potential prognostic unfavorable (1.01e-6)
validated prognostic unfavorable (3.40e-9)	validated prognostic unfavorable (7.04e-4)	unprognostic (3.64e-1)	unprognostic (2.28e-2)	unprognostic (2.28e-2)	unprognostic (3.81e-3)
validated prognostic favorable (1.39e-9)	validated prognostic favorable (2.74e-4)	unprognostic (5.43e-2)	unprognostic (2.26e-2)	unprognostic (2.81e-2)	unprognostic (1.93e-2)
potential prognostic unfavorable (2.40e-4)	unprognostic (5.50e-2)	unprognostic (7.05e-2)	unprognostic (1.61e-2)	unprognostic (6.32e-2)	unprognostic (1.58e-1)
potential prognostic favorable (1.11e-5)	unprognostic (6.63e-2)	unprognostic (9.93e-2)	unprognostic (4.10e-2)	unprognostic (5.74e-2)	unprognostic (4.00e-2)
potential prognostic unfavorable (1.24e-6)	unprognostic (2.64e-2)	unprognostic (2.51e-1)	potential prognostic unfavorable (4.24e-5)	unprognostic (1.64e-1)	potential prognostic unfavorable (4.67e-4)
unprognostic (7.69e-2)	unprognostic (3.02e-2)	unprognostic (3.16e-3)		unprognostic (1.00e-1)	unprognostic (3.55e-1)
unprognostic (1.72e-2)	unprognostic (9.73e-2)	unprognostic (5.83e-2)	potential prognostic unfavorable (8.79e-4)	unprognostic (7.29e-2)	unprognostic (6.43e-3)
validated prognostic favorable (6.02e-9)	validated prognostic favorable (2.58e-4)	potential prognostic favorable (5.92e-7)	unprognostic (1.10e-2)	unprognostic (4.58e-2)	unprognostic (1.33e-1)
unprognostic (9.40e-2)	unprognostic (3.68e-1)	unprognostic (1.31e-2)	unprognostic (1.16e-1)	potential prognostic unfavorable (4.02e-5)	unprognostic (8.92e-3)
potential prognostic unfavorable (1.17e-6)	unprognostic (1.88e-1)	unprognostic (7.21e-2)		unprognostic (1.13e-2)	unprognostic (1.37e-1)
	unprognostic (1.36e-1)	potential prognostic favorable (8.34e-4)	unprognostic (1.14e-1)	unprognostic (8.14e-3)	
validated prognostic favorable (3.17e-11)	validated prognostic favorable (5.46e-5)	unprognostic (2.41e-3)	potential prognostic favorable (6.49e-4)		unprognostic (3.33e-1)
unprognostic (4.40e-3)	unprognostic (9.52e-3)	unprognostic (6.69e-2)	unprognostic (2.71e-3)		unprognostic (2.92e-1)
potential prognostic favorable (7.04e-6)	unprognostic (2.53e-2)	unprognostic (2.14e-1)	unprognostic (5.61e-2)	unprognostic (2.99e-2)	potential prognostic unfavorable (2.96e-4)

validated prognostic favorable (2.81e-13)	validated prognostic favorable (1.16e-4)	unprognostic (1.43e-3)	unprognostic (1.34e-2)	unprognostic (3.38e-2)	unprognostic (3.27e-1)
unprognostic (9.94e-3)	unprognostic (1.69e-1)	unprognostic (1.98e-2)	unprognostic (6.50e-2)	unprognostic (8.61e-2)	unprognostic (1.24e-1)
validated prognostic favorable (1.72e-7)	validated prognostic favorable (3.78e-4)	unprognostic (3.53e-3)	unprognostic (9.94e-2)	unprognostic (4.86e-2)	potential prognostic unfavorable (8.35e-4)
potential prognostic favorable (1.74e-4)	unprognostic (2.41e-2)	potential prognostic favorable (6.77e-5)	unprognostic (1.41e-1)	unprognostic (5.58e-2)	unprognostic (8.51e-3)
validated prognostic favorable (2.70e-7)	validated prognostic favorable (2.46e-4)	unprognostic (3.78e-1)	unprognostic (2.25e-1)	potential prognostic favorable (3.61e-4)	unprognostic (5.25e-3)
potential prognostic favorable (2.48e-5)	unprognostic (6.02e-2)	potential prognostic unfavorable (3.65e-4)	unprognostic (6.85e-2)	unprognostic (2.52e-2)	unprognostic (1.35e-1)
potential prognostic favorable (1.90e-4)	unprognostic (4.21e-3)	unprognostic (6.52e-3)	unprognostic (5.64e-2)	unprognostic (1.97e-1)	unprognostic (1.84e-2)
validated prognostic unfavorable (4.96e-7)	validated prognostic unfavorable (5.60e-4)	unprognostic (2.60e-1)	unprognostic (2.32e-2)	unprognostic (5.29e-3)	unprognostic (1.28e-2)
unprognostic (1.76e-1)	unprognostic (1.99e-2)	unprognostic (1.80e-1)	unprognostic (5.83e-2)	potential prognostic unfavorable (5.02e-4)	unprognostic (3.50e-3)
potential prognostic favorable (1.41e-10)	unprognostic (5.19e-2)	unprognostic (6.57e-2)	unprognostic (1.65e-1)	unprognostic (7.12e-2)	unprognostic (1.49e-3)
validated prognostic favorable (3.42e-8)	validated prognostic favorable (2.18e-4)	unprognostic (1.14e-1)		unprognostic (6.67e-3)	unprognostic (3.02e-1)
unprognostic (1.10e-3)	unprognostic (1.89e-1)	unprognostic (1.40e-1)	unprognostic (3.51e-3)	unprognostic (3.91e-2)	unprognostic (4.11e-2)
unprognostic (6.31e-3)	unprognostic (5.47e-3)	unprognostic (5.97e-2)	validated prognostic unfavorable (3.63e-4)	validated prognostic unfavorable (9.43e-4)	unprognostic (3.61e-2)
potential prognostic unfavorable (1.81e-4)	unprognostic (2.07e-1)	unprognostic (2.37e-2)		unprognostic (4.61e-2)	unprognostic (3.75e-1)
potential prognostic unfavorable (3.38e-5)		unprognostic (5.27e-2)	unprognostic (5.44e-3)	unprognostic (1.45e-1)	unprognostic (4.36e-3)
potential prognostic favorable (7.28e-7)	unprognostic (3.28e-1)	potential prognostic favorable (8.05e-6)	unprognostic (3.08e-2)	unprognostic (2.18e-2)	unprognostic (3.27e-2)
unprognostic (3.69e-2)	unprognostic (1.65e-2)	unprognostic (1.40e-1)	validated prognostic unfavorable (7.56e-4)	validated prognostic unfavorable (4.97e-4)	validated prognostic unfavorable (6.49e-4)
unprognostic (9.75e-3)	unprognostic (7.24e-2)	unprognostic (2.68e-1)	unprognostic (1.16e-1)	unprognostic (2.96e-3)	potential prognostic favorable (3.16e-4)
unprognostic (6.90e-2)	unprognostic (2.18e-1)	unprognostic (1.48e-2)	unprognostic (2.50e-2)	unprognostic (2.64e-1)	unprognostic (1.13e-1)
potential prognostic unfavorable (8.83e-4)	unprognostic (9.92e-2)			unprognostic (5.10e-3)	potential prognostic favorable (5.89e-4)
validated prognostic favorable (2.29e-5)	validated prognostic favorable (3.76e-4)	potential prognostic favorable (4.34e-4)	unprognostic (1.08e-2)	potential prognostic favorable (5.61e-4)	unprognostic (7.69e-2)
unprognostic (1.17e-2)	unprognostic (8.52e-2)	unprognostic (6.87e-2)	unprognostic (1.00e-1)	potential prognostic favorable (9.18e-4)	unprognostic (4.80e-3)
unprognostic (1.83e-1)	unprognostic (2.81e-2)	unprognostic (1.53e-1)	unprognostic (1.57e-2)	unprognostic (3.60e-1)	unprognostic (4.86e-2)
validated prognostic unfavorable (2.53e-11)	validated prognostic unfavorable (9.40e-4)	unprognostic (4.08e-2)	unprognostic (8.55e-2)	unprognostic (4.68e-1)	potential prognostic unfavorable (3.21e-5)
potential prognostic favorable (2.50e-4)	unprognostic (1.91e-2)	unprognostic (2.12e-1)	unprognostic (6.02e-2)	unprognostic (2.39e-1)	unprognostic (2.70e-1)
validated prognostic favorable (5.57e-9)	validated prognostic favorable (9.10e-5)	unprognostic (2.55e-1)	unprognostic (5.64e-1)	unprognostic (5.82e-3)	unprognostic (1.23e-1)
validated prognostic favorable (1.92e-9)	validated prognostic favorable (3.76e-5)	unprognostic (7.14e-2)	unprognostic (2.15e-1)	potential prognostic favorable (6.55e-4)	unprognostic (2.44e-1)
unprognostic (3.19e-2)	potential prognostic favorable (5.70e-4)	unprognostic (9.04e-2)	unprognostic (1.89e-2)	unprognostic (1.20e-2)	unprognostic (1.81e-1)
unprognostic (4.41e-2)	unprognostic (9.65e-2)	unprognostic (4.48e-2)	unprognostic (1.40e-2)	unprognostic (4.44e-2)	potential prognostic unfavorable (5.78e-6)
unprognostic (2.96e-2)	unprognostic (8.40e-2)	unprognostic (1.21e-1)	unprognostic (5.36e-3)	unprognostic (6.04e-2)	unprognostic (1.46e-3)
unprognostic (3.82e-1)	unprognostic (9.91e-2)	unprognostic (5.59e-2)	potential prognostic unfavorable (1.98e-4)	unprognostic (4.47e-3)	potential prognostic unfavorable (5.01e-4)
unprognostic (1.65e-2)	unprognostic (4.84e-2)	unprognostic (1.16e-3)	unprognostic (1.78e-2)	potential prognostic unfavorable (4.91e-6)	unprognostic (4.11e-3)
unprognostic (2.83e-2)	unprognostic (2.10e-2)	unprognostic (1.59e-1)	unprognostic (3.91e-1)	unprognostic (1.48e-1)	unprognostic (2.73e-1)
unprognostic (2.05e-3)	unprognostic (1.83e-1)	unprognostic (1.47e-1)	unprognostic (1.96e-2)	unprognostic (1.86e-2)	unprognostic (6.36e-3)
					unprognostic (5.57e-2)
potential prognostic favorable (2.76e-5)	unprognostic (8.03e-2)	unprognostic (1.57e-2)	unprognostic (5.91e-3)	unprognostic (5.31e-2)	unprognostic (1.15e-1)
potential prognostic favorable (2.01e-5)	unprognostic (2.55e-2)	unprognostic (2.11e-1)	validated prognostic unfavorable (2.74e-5)	validated prognostic unfavorable (2.23e-5)	unprognostic (3.06e-2)
validated prognostic favorable (5.92e-6)	validated prognostic favorable (2.07e-4)	unprognostic (2.52e-1)	unprognostic (2.52e-2)	unprognostic (3.69e-3)	unprognostic (2.09e-3)
validated prognostic favorable (1.47e-12)	validated prognostic favorable (5.33e-5)	unprognostic (2.21e-2)	validated prognostic favorable (3.56e-7)	validated prognostic favorable (3.93e-8)	unprognostic (9.46e-2)
validated prognostic favorable (4.54e-8)	validated prognostic favorable (2.48e-6)	unprognostic (1.85e-1)	unprognostic (2.70e-2)	potential prognostic favorable (6.68e-5)	unprognostic (6.42e-2)
validated prognostic favorable (1.59e-4)	validated prognostic favorable (1.85e-4)	unprognostic (2.60e-2)	unprognostic (2.08e-2)	unprognostic (8.90e-2)	unprognostic (4.42e-2)
unprognostic (6.64e-2)	unprognostic (1.44e-1)	unprognostic (1.43e-1)	unprognostic (7.63e-2)	unprognostic (4.31e-3)	unprognostic (1.01e-2)
validated prognostic favorable (1.36e-4)	validated prognostic favorable (1.54e-4)	unprognostic (1.29e-2)		unprognostic (2.40e-2)	unprognostic (1.22e-1)
unprognostic (2.65e-2)	unprognostic (5.02e-2)	unprognostic (1.17e-1)	unprognostic (2.80e-2)	potential prognostic favorable (7.55e-5)	potential prognostic favorable (2.57e-4)
potential prognostic favorable (1.86e-4)	unprognostic (1.14e-1)			unprognostic (1.27e-1)	unprognostic (4.41e-2)
potential prognostic favorable (7.00e-5)	unprognostic (2.22e-3)	unprognostic (1.66e-1)	unprognostic (3.74e-2)	unprognostic (1.05e-1)	unprognostic (2.59e-1)
unprognostic (1.24e-1)	unprognostic (8.08e-2)	unprognostic (4.53e-2)	unprognostic (5.43e-2)	potential prognostic unfavorable (2.26e-4)	unprognostic (1.29e-3)
potential prognostic favorable (2.99e-8)	unprognostic (1.41e-2)	unprognostic (5.91e-2)	unprognostic (1.03e-1)	unprognostic (1.24e-1)	potential prognostic unfavorable (4.98e-5)
validated prognostic favorable (4.86e-6)	validated prognostic favorable (2.02e-4)	unprognostic (3.27e-2)	unprognostic (3.61e-1)	unprognostic (1.12e-2)	unprognostic (1.88e-1)
unprognostic (1.21e-1)	potential prognostic favorable (6.82e-6)	unprognostic (2.73e-3)	unprognostic (2.86e-2)	unprognostic (5.74e-2)	unprognostic (7.94e-3)
potential prognostic favorable (2.50e-4)	unprognostic (4.67e-2)	unprognostic (1.85e-1)	unprognostic (1.12e-3)	unprognostic (1.57e-1)	unprognostic (2.54e-1)
					unprognostic (1.24e-1)
unprognostic (1.06e-3)	unprognostic (1.19e-2)	unprognostic (4.30e-2)	potential prognostic unfavorable (6.12e-4)	unprognostic (3.86e-1)	unprognostic (3.24e-1)
unprognostic (1.37e-1)	unprognostic (1.34e-2)	unprognostic (5.99e-2)	potential prognostic unfavorable (2.68e-4)	unprognostic (1.88e-2)	unprognostic (3.69e-2)
unprognostic (1.76e-2)	unprognostic (2.50e-2)	unprognostic (1.61e-3)	unprognostic (1.29e-1)		potential prognostic favorable (2.67e-5)
potential prognostic favorable (2.75e-7)	unprognostic (9.18e-3)	potential prognostic favorable (3.33e-4)	validated prognostic unfavorable (2.85e-4)	validated prognostic unfavorable (2.59e-4)	unprognostic (1.05e-1)
potential prognostic favorable (1.20e-4)	unprognostic (4.02e-2)	unprognostic (2.50e-1)	unprognostic (6.67e-2)	unprognostic (1.33e-2)	unprognostic (4.34e-2)
unprognostic (2.65e-3)	unprognostic (7.93e-3)	unprognostic (3.95e-2)	unprognostic (1.95e-2)	unprognostic (1.93e-1)	unprognostic (1.07e-2)
potential prognostic favorable (1.26e-6)	unprognostic (7.72e-3)	unprognostic (2.07e-1)	unprognostic (5.89e-3)	unprognostic (9.05e-2)	unprognostic (2.32e-2)
potential prognostic favorable (5.71e-5)	unprognostic (2.26e-1)	unprognostic (1.57e-1)	potential prognostic unfavorable (5.19e-4)	unprognostic (9.62e-2)	unprognostic (3.60e-1)
potential prognostic favorable (1.87e-7)	unprognostic (2.20e-2)	unprognostic (2.68e-1)	potential prognostic unfavorable (6.72e-4)	unprognostic (2.80e-1)	unprognostic (3.31e-2)
unprognostic (5.16e-3)	unprognostic (2.38e-3)	unprognostic (2.41e-1)	unprognostic (1.12e-3)	potential prognostic unfavorable (2.27e-10)	unprognostic (4.93e-2)
unprognostic (5.74e-2)	unprognostic (6.68e-3)	unprognostic (2.37e-1)	potential prognostic unfavorable (8.08e-9)	unprognostic (1.28e-3)	unprognostic (7.72e-2)
	unprognostic (1.08e-2)			unprognostic (1.63e-1)	
unprognostic (1.24e-3)	unprognostic (6.72e-2)	unprognostic (1.05e-1)	unprognostic (2.61e-1)	unprognostic (7.10e-2)	unprognostic (1.03e-1)
unprognostic (2.37e-2)	unprognostic (7.65e-2)	unprognostic (1.82e-1)	unprognostic (2.30e-3)	unprognostic (6.08e-3)	unprognostic (1.89e-2)
unprognostic (2.62e-1)	unprognostic (1.26e-2)	unprognostic (1.45e-2)	unprognostic (1.74e-1)	unprognostic (1.40e-1)	unprognostic (1.84e-2)
unprognostic (1.66e-3)	unprognostic (1.44e-3)	unprognostic (1.93e-1)	potential prognostic unfavorable (5.14e-4)	unprognostic (1.40e-1)	unprognostic (9.10e-3)
unprognostic (5.24e-2)	unprognostic (3.07e-1)	unprognostic (2.03e-1)	potential prognostic unfavorable (6.94e-6)	unprognostic (1.29e-1)	unprognostic (6.18e-3)
		potential prognostic favorable (6.10e-4)			unprognostic (8.53e-2)
	unprognostic (3.80e-1)				
		unprognostic (7.21e-2)		unprognostic (8.57e-3)	
potential prognostic unfavorable (2.99e-5)	unprognostic (1.70e-1)	unprognostic (2.17e-1)	unprognostic (2.31e-2)	unprognostic (2.56e-1)	unprognostic (1.82e-1)
potential prognostic unfavorable (7.37e-6)	unprognostic (3.87e-2)	unprognostic (1.44e-1)			unprognostic (9.68e-2)
unprognostic (1.55e-1)	unprognostic (3.85e-2)	unprognostic (4.93e-2)	unprognostic (7.79e-3)	unprognostic (1.28e-2)	unprognostic (1.28e-1)
unprognostic (2.09e-2)					unprognostic (2.64e-1)
potential prognostic favorable (1.36e-6)	unprognostic (2.12e-2)	unprognostic (2.54e-1)	unprognostic (1.36e-1)	unprognostic (2.25e-3)	unprognostic (1.52e-1)
potential prognostic favorable (1.57e-5)	unprognostic (6.24e-2)	unprognostic (1.55e-1)	unprognostic (8.26e-2)		unprognostic (1.01e-1)
potential prognostic favorable (7.28e-5)	unprognostic (1.15e-2)	unprognostic (6.21e-2)	unprognostic (1.20e-1)	unprognostic (3.53e-2)	unprognostic (2.02e-1)
validated prognostic favorable (1.10e-4)	validated prognostic favorable (4.77e-4)	unprognostic (3.59e-3)	unprognostic (6.15e-2)	unprognostic (2.02e-1)	unprognostic (5.32e-2)
unprognostic (5.38e-2)	unprognostic (1.64e-1)	unprognostic (6.60e-2)	unprognostic (4.26e-2)	unprognostic (5.13e-2)	unprognostic (1.08e-1)
unprognostic (2.51e-2)	unprognostic (2.11e-3)	unprognostic (9.99e-2)		unprognostic (5.02e-1)	unprognostic (5.31e-2)
potential prognostic favorable (5.04e-8)	unprognostic (2.22e-2)	unprognostic (6.02e-2)	unprognostic (2.33e-2)	unprognostic (2.56e-1)	unprognostic (1.13e-1)
unprognostic (1.25e-1)	unprognostic (3.69e-2)	unprognostic (2.47e-1)	unprognostic (1.30e-2)	unprognostic (3.41e-2)	unprognostic (2.89e-1)
unprognostic (2.74e-2)	unprognostic (3.42e-2)	unprognostic (7.13e-2)	unprognostic (1.64e-1)	unprognostic (6.59e-3)	unprognostic (1.99e-2)
			unprognostic (1.59e-2)	potential prognostic favorable (7.33e-4)	
potential prognostic favorable (2.98e-5)	unprognostic (3.99e-3)	unprognostic (7.52e-2)		potential prognostic favorable (1.08e-5)	
unprognostic (8.11e-3)	unprognostic (1.60e-1)	unprognostic (4.02e-2)	unprognostic (6.52e-3)	unprognostic (2.50e-3)	potential prognostic unfavorable (2.39e-6)
unprognostic (1.33e-1)	unprognostic (8.11e-2)	unprognostic (1.88e-2)	unprognostic (1.15e-1)	unprognostic (1.01e-1)	unprognostic (3.17e-2)
unprognostic (1.74e-2)	unprognostic (1.59e-2)	unprognostic (2.33e-1)	potential prognostic unfavorable (1.05e-7)	unprognostic (9.31e-2)	validated prognostic unfavorable (9.21e-7)
unprognostic (2.39e-1)	unprognostic (4.35e-2)	unprognostic (7.55e-2)	unprognostic (4.17e-2)	unprognostic (3.95e-1)	unprognostic (1.09e-1)
potential prognostic favorable (5.00e-6)	unprognostic (5.74e-2)	unprognostic (5.52e-2)	unprognostic (5.65e-3)	unprognostic (2.43e-2)	unprognostic (2.98e-1)
potential prognostic unfavorable (2.20e-7)	unprognostic (1.12e-2)	unprognostic (1.24e-1)	potential prognostic unfavorable (2.14e-10)	unprognostic (4.21e-3)	unprognostic (1.46e-3)
unprognostic (7.24e-3)		unprognostic (2.47e-1)			
unprognostic (1.43e-2)	unprognostic (2.99e-2)	unprognostic (5.01e-2)	potential prognostic unfavorable (7.36e-4)	unprognostic (3.37e-2)	unprognostic (1.01e-3)
potential prognostic unfavorable (8.94e-7)	unprognostic (3.96e-1)	unprognostic (2.66e-2)	potential prognostic unfavorable (4.27e-4)	unprognostic (9.74e-3)	unprognostic (1.24e-3)
validated prognostic favorable (4.02e-5)	validated prognostic favorable (4.28e-4)	potential prognostic favorable (2.14e-4)	unprognostic (2.84e-2)	potential prognostic favorable (7.20e-5)	
unprognostic (7.25e-3)	unprognostic (3.93e-1)	potential prognostic unfavorable (7.22e-4)	validated prognostic unfavorable (2.32e-6)	validated prognostic unfavorable (9.77e-4)	validated prognostic unfavorable (5.40e-9)
			unprognostic (6.52e-3)	unprognostic (1.01e-2)	unprognostic (1.30e-1)
potential prognostic unfavorable (3.62e-7)	unprognostic (1.52e-1)	unprognostic (1.15e-1)	unprognostic (1.61e-1)		unprognostic (4.18e-2)
potential prognostic favorable (1.10e-5)	unprognostic (7.97e-2)	unprognostic (7.92e-2)	potential prognostic favorable (1.70e-5)	unprognostic (1.46e-2)	
unprognostic (7.00e-2)	unprognostic (1.81e-2)	unprognostic (1.81e-1)	unprognostic (6.61e-2)	unprognostic (1.95e-1)	unprognostic (2.34e-1)
potential prognostic favorable (5.25e-4)	unprognostic (4.50e-1)	unprognostic (3.61e-1)	unprognostic (2.66e-3)	unprognostic (1.30e-1)	unprognostic (2.01e-1)
potential prognostic favorable (5.14e-6)	unprognostic (1.97e-2)	unprognostic (5.42e-2)	unprognostic (4.78e-2)	unprognostic (1.72e-2)	unprognostic (1.28e-2)
potential prognostic favorable (1.78e-6)	unprognostic (1.13e-3)	unprognostic (1.23e-1)			
unprognostic (3.23e-3)	unprognostic (2.50e-1)	potential prognostic favorable (1.65e-6)	unprognostic (2.50e-2)	unprognostic (1.22e-2)	potential prognostic favorable (1.82e-4)
unprognostic (3.29e-1)	unprognostic (1.06e-1)	unprognostic (1.03e-1)	potential prognostic unfavorable (3.17e-5)		potential prognostic unfavorable (9.03e-6)
unprognostic (1.90e-3)	unprognostic (1.92e-1)	unprognostic (2.69e-1)	unprognostic (9.02e-2)	unprognostic (8.21e-2)	potential prognostic unfavorable (1.52e-4)
unprognostic (1.56e-3)	unprognostic (2.68e-2)	unprognostic (1.28e-1)	unprognostic (2.85e-3)	unprognostic (2.26e-2)	unprognostic (7.63e-2)
unprognostic (1.14e-3)	unprognostic (5.59e-2)	unprognostic (1.40e-1)	unprognostic (1.45e-2)	unprognostic (4.98e-2)	potential prognostic unfavorable (7.08e-5)
validated prognostic favorable (1.92e-12)	validated prognostic favorable (6.11e-4)	unprognostic (3.51e-1)	unprognostic (1.66e-1)	potential prognostic favorable (7.30e-5)	unprognostic (1.24e-1)
potential prognostic favorable (1.15e-4)	unprognostic (7.32e-2)	potential prognostic favorable (4.76e-5)	potential prognostic unfavorable (9.97e-8)	unprognostic (1.40e-3)	unprognostic (1.15e-1)
potential prognostic favorable (4.83e-6)	unprognostic (1.14e-2)	unprognostic (2.95e-3)	unprognostic (8.49e-2)	unprognostic (4.74e-2)	unprognostic (3.07e-2)
potential prognostic favorable (1.09e-5)	unprognostic (5.90e-2)	unprognostic (6.67e-2)	validated prognostic unfavorable (4.42e-5)	validated prognostic unfavorable (8.83e-4)	unprognostic (1.13e-2)
validated prognostic favorable (1.95e-8)	validated prognostic favorable (2.79e-4)	unprognostic (3.00e-1)	unprognostic (7.36e-2)	unprognostic (8.53e-3)	unprognostic (7.54e-2)
unprognostic (1.42e-1)	unprognostic (1.18e-1)	unprognostic (1.77e-1)	potential prognostic unfavorable (8.41e-4)	unprognostic (2.69e-1)	unprognostic (2.38e-1)
validated prognostic favorable (4.06e-9)	validated prognostic favorable (4.70e-4)	potential prognostic favorable (1.39e-4)		unprognostic (1.61e-2)	unprognostic (6.50e-2)
validated prognostic favorable (1.88e-8)	validated prognostic favorable (2.35e-4)	unprognostic (5.04e-2)			
unprognostic (5.56e-2)	unprognostic (1.92e-1)	unprognostic (3.29e-2)	unprognostic (1.04e-2)	unprognostic (1.34e-2)	unprognostic (2.40e-1)
unprognostic (4.32e-2)	unprognostic (1.08e-2)	unprognostic (1.29e-2)	unprognostic (1.27e-2)	unprognostic (1.17e-1)	unprognostic (2.72e-1)
validated prognostic unfavorable (9.58e-9)	validated prognostic unfavorable (6.51e-5)	potential prognostic unfavorable (6.71e-4)	unprognostic (5.22e-2)	unprognostic (2.87e-1)	unprognostic (4.76e-3)
unprognostic (5.27e-2)	unprognostic (1.23e-1)	unprognostic (1.28e-1)	potential prognostic unfavorable (5.33e-4)	unprognostic (1.62e-2)	unprognostic (1.58e-1)

potential prognostic favorable (3.46e-8)	unprognostic (1.15e-2)	unprognostic (3.74e-1)		unprognostic (1.25e-1)	unprognostic (6.08e-2)
unprognostic (1.28e-3)	unprognostic (2.61e-2)	unprognostic (1.59e-1)	unprognostic (7.39e-3)	unprognostic (1.87e-2)	unprognostic (6.92e-2)
		unprognostic (1.16e-1)			
unprognostic (2.15e-2)	unprognostic (1.32e-2)	unprognostic (1.77e-2)	unprognostic (7.25e-2)	potential prognostic favorable (7.69e-4)	unprognostic (2.50e-2)
validated prognostic favorable (2.53e-7)	validated prognostic favorable (2.67e-5)	unprognostic (8.20e-2)	unprognostic (5.91e-3)	potential prognostic favorable (2.67e-4)	unprognostic (1.72e-1)
potential prognostic favorable (1.72e-7)	unprognostic (8.06e-2)	unprognostic (2.45e-1)	unprognostic (1.53e-1)	unprognostic (1.72e-3)	unprognostic (1.18e-1)
unprognostic (3.28e-2)	unprognostic (5.09e-2)	unprognostic (9.29e-2)	unprognostic (1.31e-2)	unprognostic (2.64e-3)	unprognostic (1.62e-2)
			unprognostic (2.85e-3)	unprognostic (1.43e-3)	
			unprognostic (2.10e-2)	potential prognostic favorable (3.79e-4)	unprognostic (2.25e-3)
unprognostic (6.49e-2)	unprognostic (1.11e-1)	unprognostic (1.72e-1)	unprognostic (2.19e-2)	unprognostic (2.44e-2)	potential prognostic unfavorable (6.68e-5)
unprognostic (4.29e-3)	unprognostic (1.85e-3)	unprognostic (1.04e-3)	unprognostic (3.52e-2)	unprognostic (4.27e-3)	unprognostic (2.89e-3)
		potential prognostic unfavorable (2.46e-5)			unprognostic (1.84e-2)
potential prognostic favorable (8.89e-6)	unprognostic (3.39e-1)	potential prognostic favorable (3.15e-4)	unprognostic (2.66e-3)	unprognostic (7.31e-3)	unprognostic (1.44e-2)
unprognostic (1.06e-3)	unprognostic (3.85e-2)	unprognostic (2.58e-1)	unprognostic (1.58e-3)	unprognostic (2.42e-2)	unprognostic (9.14e-2)
unprognostic (1.26e-3)	unprognostic (1.76e-2)	unprognostic (1.25e-1)	unprognostic (9.13e-3)	unprognostic (2.39e-1)	unprognostic (5.38e-2)
potential prognostic favorable (3.89e-6)	unprognostic (2.61e-3)	unprognostic (3.97e-1)	unprognostic (6.22e-2)	unprognostic (2.13e-1)	unprognostic (1.02e-3)
potential prognostic favorable (4.07e-7)	unprognostic (4.03e-3)	unprognostic (3.51e-1)	unprognostic (3.63e-2)	unprognostic (2.66e-3)	unprognostic (5.56e-2)
validated prognostic favorable (2.94e-4)	validated prognostic favorable (2.80e-4)	unprognostic (4.33e-2)	unprognostic (1.99e-3)	unprognostic (8.20e-2)	unprognostic (1.21e-2)
unprognostic (1.31e-2)	unprognostic (1.03e-2)	potential prognostic favorable (7.29e-4)	unprognostic (2.57e-2)	unprognostic (1.53e-1)	unprognostic (4.31e-1)
potential prognostic unfavorable (1.77e-5)	unprognostic (3.26e-1)	unprognostic (5.94e-2)	unprognostic (9.57e-3)	unprognostic (1.51e-2)	potential prognostic unfavorable (6.51e-5)
potential prognostic favorable (3.32e-7)	unprognostic (7.89e-2)	unprognostic (2.84e-2)	unprognostic (2.36e-2)	unprognostic (2.03e-1)	unprognostic (2.24e-2)
potential prognostic favorable (1.18e-4)	unprognostic (1.11e-3)	unprognostic (2.89e-1)	unprognostic (2.05e-1)	unprognostic (2.08e-2)	unprognostic (2.86e-1)
unprognostic (4.24e-2)	unprognostic (6.47e-2)	unprognostic (1.67e-2)	unprognostic (3.14e-1)	unprognostic (2.52e-1)	unprognostic (1.04e-1)
potential prognostic favorable (6.02e-6)	unprognostic (1.10e-2)	unprognostic (8.93e-2)	validated prognostic unfavorable (4.69e-5)	validated prognostic unfavorable (1.86e-4)	unprognostic (1.10e-2)
unprognostic (1.08e-2)	unprognostic (6.32e-3)	unprognostic (1.48e-1)	validated prognostic unfavorable (2.14e-4)	validated prognostic unfavorable (3.05e-5)	unprognostic (3.55e-2)
		unprognostic (2.19e-1)	unprognostic (3.40e-1)	unprognostic (9.68e-2)	
potential prognostic favorable (7.87e-6)	unprognostic (4.22e-1)	unprognostic (1.38e-1)	unprognostic (2.21e-2)	potential prognostic unfavorable (3.43e-4)	unprognostic (7.74e-3)
unprognostic (2.42e-3)	unprognostic (9.47e-3)	unprognostic (1.48e-2)	unprognostic (1.24e-3)	potential prognostic favorable (1.91e-4)	unprognostic (4.99e-2)
potential prognostic favorable (3.04e-4)	unprognostic (3.24e-2)	unprognostic (1.12e-1)	unprognostic (1.18e-3)	unprognostic (1.20e-2)	unprognostic (2.11e-1)
validated prognostic favorable (5.47e-7)	validated prognostic favorable (8.29e-5)	potential prognostic favorable (1.84e-4)	unprognostic (1.11e-2)	unprognostic (2.94e-3)	unprognostic (3.80e-1)
potential prognostic favorable (2.68e-9)	unprognostic (5.63e-3)	unprognostic (1.80e-1)	validated prognostic unfavorable (2.78e-4)	validated prognostic unfavorable (5.12e-5)	unprognostic (2.69e-2)
					unprognostic (1.54e-1)
potential prognostic favorable (6.21e-6)	unprognostic (2.60e-3)	unprognostic (5.54e-2)	potential prognostic unfavorable (1.48e-6)	unprognostic (1.88e-1)	potential prognostic unfavorable (1.79e-4)
validated prognostic favorable (1.58e-5)	validated prognostic favorable (5.65e-5)	unprognostic (1.27e-1)	unprognostic (1.09e-1)	unprognostic (1.65e-1)	unprognostic (6.08e-2)
validated prognostic favorable (4.51e-8)	validated prognostic favorable (8.08e-5)	unprognostic (1.36e-1)	unprognostic (7.46e-3)	unprognostic (1.34e-1)	unprognostic (8.14e-2)
potential prognostic favorable (3.90e-4)	unprognostic (1.17e-2)	unprognostic (1.17e-1)	potential prognostic unfavorable (8.09e-4)	unprognostic (1.43e-1)	potential prognostic unfavorable (8.35e-5)
unprognostic (3.26e-1)	unprognostic (9.60e-2)	unprognostic (2.07e-2)	unprognostic (2.44e-1)	unprognostic (1.06e-1)	unprognostic (2.41e-1)
potential prognostic favorable (4.70e-4)	unprognostic (3.89e-1)	unprognostic (3.48e-1)	unprognostic (1.37e-1)	unprognostic (2.23e-2)	unprognostic (2.58e-1)
	unprognostic (8.39e-3)				unprognostic (9.09e-2)
unprognostic (8.69e-2)	unprognostic (1.52e-1)	unprognostic (4.31e-2)	unprognostic (1.37e-1)	unprognostic (1.21e-2)	unprognostic (5.84e-2)
					unprognostic (1.45e-1)
potential prognostic favorable (2.56e-6)	unprognostic (6.55e-3)	unprognostic (3.97e-1)	unprognostic (1.12e-1)	unprognostic (3.43e-2)	unprognostic (1.49e-1)
unprognostic (3.17e-3)	unprognostic (5.80e-2)	unprognostic (1.80e-1)	unprognostic (2.18e-1)	potential prognostic favorable (2.93e-4)	unprognostic (5.71e-2)
unprognostic (2.19e-2)	unprognostic (2.65e-3)	unprognostic (2.42e-2)		unprognostic (1.58e-3)	unprognostic (1.85e-1)
validated prognostic favorable (7.55e-5)	validated prognostic favorable (9.61e-6)	unprognostic (1.22e-2)	unprognostic (3.24e-2)	potential prognostic favorable (7.80e-6)	unprognostic (5.37e-2)
unprognostic (3.98e-3)	unprognostic (2.82e-2)	unprognostic (5.83e-2)	unprognostic (3.38e-2)		unprognostic (3.87e-1)
potential prognostic favorable (6.15e-5)	unprognostic (1.88e-1)	unprognostic (8.38e-2)		unprognostic (1.06e-1)	unprognostic (8.04e-2)
validated prognostic favorable (3.45e-13)	validated prognostic favorable (5.48e-5)	unprognostic (4.39e-3)	validated prognostic favorable (1.01e-4)	validated prognostic favorable (8.49e-4)	unprognostic (2.27e-2)
potential prognostic favorable (1.07e-6)	unprognostic (2.16e-3)	unprognostic (4.45e-2)	unprognostic (2.98e-2)	unprognostic (2.70e-2)	unprognostic (1.49e-2)
validated prognostic favorable (2.35e-7)	validated prognostic favorable (3.61e-4)	unprognostic (2.97e-1)	unprognostic (2.96e-1)	unprognostic (3.33e-2)	unprognostic (8.78e-2)
unprognostic (1.87e-1)	unprognostic (7.47e-2)	unprognostic (5.05e-2)	unprognostic (9.16e-2)	potential prognostic favorable (1.51e-5)	unprognostic (3.62e-1)
unprognostic (1.05e-3)	unprognostic (4.10e-3)	unprognostic (4.33e-2)	unprognostic (5.97e-3)	unprognostic (5.06e-2)	unprognostic (1.90e-2)
validated prognostic favorable (5.38e-5)	validated prognostic favorable (3.00e-4)	unprognostic (8.66e-2)	unprognostic (1.53e-1)	unprognostic (3.60e-3)	unprognostic (6.42e-3)
validated prognostic favorable (1.97e-12)	validated prognostic favorable (1.52e-5)	unprognostic (1.45e-2)	unprognostic (7.33e-3)	unprognostic (5.82e-2)	unprognostic (5.35e-2)
		unprognostic (3.44e-2)	unprognostic (6.07e-2)	unprognostic (4.91e-2)	unprognostic (7.44e-2)
potential prognostic favorable (9.27e-7)	unprognostic (1.35e-3)	unprognostic (2.09e-2)	unprognostic (2.76e-2)	unprognostic (1.69e-2)	unprognostic (1.15e-1)
unprognostic (8.77e-3)	unprognostic (1.72e-1)	unprognostic (1.08e-2)	unprognostic (2.05e-2)	unprognostic (3.58e-3)	potential prognostic favorable (5.70e-4)
potential prognostic favorable (1.51e-6)	unprognostic (1.28e-2)	unprognostic (9.68e-2)	unprognostic (6.78e-2)	unprognostic (2.14e-3)	unprognostic (2.99e-3)
			unprognostic (3.57e-1)	unprognostic (1.19e-1)	unprognostic (6.93e-2)
unprognostic (5.13e-2)	unprognostic (1.11e-1)	unprognostic (1.67e-1)	unprognostic (7.74e-3)	unprognostic (7.26e-2)	unprognostic (6.99e-3)
unprognostic (2.48e-3)	unprognostic (4.41e-2)	unprognostic (1.92e-1)	potential prognostic unfavorable (8.33e-5)	unprognostic (9.46e-3)	unprognostic (1.85e-3)
unprognostic (4.58e-1)		unprognostic (7.00e-3)	unprognostic (4.06e-2)	potential prognostic favorable (9.91e-5)	potential prognostic favorable (6.80e-4)
unprognostic (3.58e-3)	unprognostic (3.19e-2)			potential prognostic unfavorable (6.59e-4)	potential prognostic unfavorable (8.41e-4)
potential prognostic favorable (5.65e-6)	unprognostic (4.32e-3)	unprognostic (1.79e-1)	unprognostic (3.05e-1)	unprognostic (9.33e-2)	unprognostic (7.65e-2)
potential prognostic favorable (1.79e-7)	unprognostic (4.11e-2)	unprognostic (7.09e-2)	unprognostic (1.33e-2)	unprognostic (1.16e-2)	unprognostic (8.33e-2)
potential prognostic unfavorable (5.23e-4)	unprognostic (1.28e-1)	unprognostic (3.10e-2)	unprognostic (9.44e-2)	unprognostic (1.68e-2)	unprognostic (9.78e-2)
unprognostic (1.59e-2)	unprognostic (1.71e-1)	unprognostic (2.08e-1)	unprognostic (1.38e-1)	unprognostic (3.86e-2)	unprognostic (2.85e-1)
unprognostic (2.43e-3)	unprognostic (9.02e-2)	unprognostic (1.06e-1)	unprognostic (4.61e-2)	unprognostic (2.65e-1)	unprognostic (1.09e-1)
validated prognostic favorable (4.03e-5)	validated prognostic favorable (6.59e-4)	unprognostic (6.36e-3)	unprognostic (1.05e-2)		
validated prognostic unfavorable (6.86e-7)	validated prognostic unfavorable (6.61e-4)	unprognostic (5.88e-2)	validated prognostic unfavorable (6.33e-8)	validated prognostic unfavorable (9.26e-7)	potential prognostic unfavorable (2.97e-4)
unprognostic (1.01e-1)	unprognostic (8.96e-2)			unprognostic (1.92e-2)	unprognostic (1.26e-2)
	unprognostic (9.02e-3)				
unprognostic (1.26e-1)	unprognostic (4.45e-3)	potential prognostic favorable (4.08e-4)	unprognostic (1.77e-1)	unprognostic (4.52e-2)	unprognostic (7.02e-2)
potential prognostic unfavorable (6.43e-4)	unprognostic (1.06e-2)	unprognostic (1.07e-1)	unprognostic (1.19e-2)	unprognostic (2.82e-1)	unprognostic (2.56e-1)
unprognostic (2.67e-2)	unprognostic (3.52e-1)	unprognostic (1.02e-1)	potential prognostic unfavorable (2.83e-4)	unprognostic (3.51e-1)	unprognostic (1.81e-1)
			unprognostic (2.85e-3)	unprognostic (1.06e-1)	
unprognostic (7.11e-3)	unprognostic (4.16e-3)	unprognostic (5.67e-3)	unprognostic (1.03e-1)	unprognostic (3.17e-1)	unprognostic (2.46e-1)
unprognostic (2.28e-2)	unprognostic (6.01e-2)	unprognostic (3.61e-1)	potential prognostic unfavorable (4.20e-5)	unprognostic (3.16e-2)	unprognostic (4.20e-2)
unprognostic (1.86e-2)	unprognostic (4.33e-3)				
potential prognostic favorable (1.24e-6)	unprognostic (2.45e-1)	unprognostic (1.04e-1)	unprognostic (2.22e-1)	unprognostic (2.39e-1)	unprognostic (1.02e-3)
unprognostic (3.40e-3)	unprognostic (3.56e-1)	unprognostic (7.16e-2)	unprognostic (6.71e-2)	unprognostic (2.06e-1)	unprognostic (6.02e-2)
validated prognostic unfavorable (1.67e-13)	validated prognostic unfavorable (2.92e-7)	potential prognostic unfavorable (2.33e-12)	validated prognostic unfavorable (5.02e-6)	validated prognostic unfavorable (1.88e-8)	validated prognostic unfavorable (4.61e-5)
validated prognostic favorable (1.94e-9)	validated prognostic favorable (3.70e-4)	unprognostic (3.04e-1)	unprognostic (1.59e-2)	unprognostic (7.43e-2)	unprognostic (2.51e-3)
unprognostic (4.64e-2)	unprognostic (1.36e-2)	unprognostic (1.57e-1)		unprognostic (1.15e-1)	unprognostic (4.56e-2)
potential prognostic favorable (3.82e-7)	unprognostic (2.32e-1)	unprognostic (2.38e-1)	validated prognostic unfavorable (4.69e-4)	validated prognostic unfavorable (1.61e-5)	potential prognostic unfavorable (1.41e-4)
validated prognostic favorable (4.36e-7)	validated prognostic favorable (5.33e-5)	unprognostic (2.12e-1)	unprognostic (1.57e-3)	unprognostic (3.77e-1)	unprognostic (7.65e-2)
unprognostic (5.85e-3)	unprognostic (6.40e-2)	unprognostic (2.66e-1)	potential prognostic unfavorable (1.48e-6)	unprognostic (3.84e-2)	unprognostic (4.02e-2)
validated prognostic favorable (1.25e-6)	validated prognostic favorable (1.75e-4)	unprognostic (2.43e-2)	unprognostic (1.16e-1)	potential prognostic unfavorable (4.14e-4)	unprognostic (3.14e-1)
potential prognostic favorable (4.82e-8)	unprognostic (4.62e-3)	unprognostic (1.11e-2)	unprognostic (2.25e-1)	unprognostic (3.00e-1)	unprognostic (5.78e-2)
potential prognostic favorable (1.77e-6)	unprognostic (3.19e-2)	unprognostic (5.94e-3)	unprognostic (3.70e-3)	unprognostic (2.10e-2)	validated prognostic unfavorable (8.01e-6)
validated prognostic favorable (1.12e-7)	validated prognostic favorable (1.69e-4)	unprognostic (4.26e-3)	unprognostic (1.31e-1)	unprognostic (6.60e-2)	unprognostic (3.11e-2)
unprognostic (2.25e-3)	unprognostic (6.41e-3)	unprognostic (1.31e-1)	unprognostic (2.16e-1)	unprognostic (2.49e-3)	unprognostic (1.97e-1)
potential prognostic favorable (5.57e-6)	unprognostic (1.27e-2)	unprognostic (6.19e-2)	unprognostic (1.57e-2)	unprognostic (5.31e-3)	unprognostic (2.17e-3)
potential prognostic favorable (3.98e-4)	unprognostic (1.71e-2)	unprognostic (9.75e-2)	unprognostic (2.80e-3)	unprognostic (4.61e-2)	unprognostic (2.98e-2)
potential prognostic favorable (2.84e-5)	unprognostic (4.56e-2)	unprognostic (7.22e-2)	unprognostic (9.84e-2)	unprognostic (4.08e-1)	unprognostic (1.64e-1)
unprognostic (2.33e-3)	unprognostic (7.40e-2)	unprognostic (3.70e-2)	unprognostic (1.79e-1)	unprognostic (1.40e-1)	unprognostic (1.25e-2)
potential prognostic favorable (1.11e-4)	unprognostic (3.02e-3)	unprognostic (3.98e-2)	unprognostic (2.35e-2)	unprognostic (2.95e-2)	unprognostic (9.55e-2)
potential prognostic favorable (2.64e-7)	unprognostic (4.09e-3)	unprognostic (4.76e-1)	unprognostic (1.05e-1)	unprognostic (2.07e-1)	unprognostic (9.97e-3)
potential prognostic favorable (9.35e-4)	unprognostic (4.98e-3)	unprognostic (7.52e-2)	unprognostic (2.45e-2)	unprognostic (1.03e-1)	potential prognostic unfavorable (2.20e-5)
potential prognostic favorable (3.60e-6)	unprognostic (6.15e-2)	unprognostic (1.37e-2)	unprognostic (2.03e-2)	unprognostic (4.03e-2)	unprognostic (2.25e-1)

Cancer prognostics - Lung Adenocarcinoma (validation)	Cancer prognostics - Lung Squamous Cell Carcinoma (TCGA)	Cancer prognostics - Lung Squamous Cell Carcinoma (validation)	Cancer prognostics - Ovary Serous Cystadenocarcinoma (TCGA)	Cancer prognostics - Ovary Serous Cystadenocarcinoma (validation)	Cancer prognostics - Pancreatic Adenocarcinoma (TCGA)
unprognostic (1.53e-2)	unprognostic (2.27e-1)	unprognostic (9.91e-2)	potential prognostic unfavorable (1.45e-4)	unprognostic (1.43e-3)	unprognostic (2.71e-1)
unprognostic (1.26e-1)	unprognostic (8.16e-2)	unprognostic (1.12e-1)	unprognostic (1.05e-1)	unprognostic (1.16e-1)	unprognostic (3.75e-2)
unprognostic (8.55e-3)	unprognostic (1.50e-1)	unprognostic (1.65e-1)	unprognostic (4.09e-3)	potential prognostic unfavorable (1.19e-4)	unprognostic (3.06e-1)
unprognostic (4.78e-2)	unprognostic (1.38e-1)	unprognostic (1.61e-1)	unprognostic (3.22e-2)	unprognostic (6.87e-2)	unprognostic (7.72e-3)
unprognostic (2.74e-2)		unprognostic (7.12e-2)			unprognostic (3.61e-3)
unprognostic (2.49e-1)					unprognostic (1.67e-3)
unprognostic (7.42e-2)	unprognostic (2.09e-1)	unprognostic (1.93e-2)	unprognostic (3.19e-1)	unprognostic (2.12e-2)	unprognostic (6.36e-3)
unprognostic (1.13e-1)	unprognostic (1.36e-1)	unprognostic (2.77e-2)	unprognostic (4.25e-2)	unprognostic (3.61e-2)	unprognostic (9.51e-3)
unprognostic (1.33e-1)	unprognostic (1.48e-2)	unprognostic (1.69e-1)	unprognostic (8.19e-3)	unprognostic (2.14e-1)	unprognostic (1.05e-3)
unprognostic (3.11e-1)	unprognostic (1.90e-1)	unprognostic (1.62e-2)	unprognostic (1.40e-1)	unprognostic (1.24e-1)	unprognostic (7.59e-2)
unprognostic (7.25e-2)	unprognostic (7.14e-2)	unprognostic (4.12e-1)	unprognostic (1.54e-2)	unprognostic (1.76e-1)	unprognostic (2.86e-1)
unprognostic (4.10e-1)	unprognostic (2.17e-1)	unprognostic (3.04e-1)			unprognostic (4.54e-3)
unprognostic (5.82e-3)	unprognostic (1.60e-1)	unprognostic (3.56e-2)	unprognostic (6.38e-2)	unprognostic (1.57e-1)	unprognostic (1.48e-3)
unprognostic (1.13e-2)	unprognostic (2.01e-2)	unprognostic (1.29e-1)	unprognostic (1.90e-1)	unprognostic (6.99e-2)	unprognostic (3.57e-3)
unprognostic (2.43e-3)	unprognostic (5.70e-2)	unprognostic (3.20e-1)	unprognostic (2.74e-2)	unprognostic (5.46e-3)	potential prognostic unfavorable (2.06e-4)
potential prognostic unfavorable (7.32e-4)	unprognostic (2.04e-1)	unprognostic (1.99e-1)	unprognostic (4.71e-3)	potential prognostic unfavorable (5.36e-5)	unprognostic (4.10e-2)
unprognostic (1.42e-1)	unprognostic (2.45e-1)	unprognostic (1.11e-1)	unprognostic (1.12e-1)	unprognostic (6.00e-2)	unprognostic (1.19e-2)
unprognostic (2.49e-2)	unprognostic (2.64e-3)	unprognostic (3.36e-2)	unprognostic (1.78e-1)	unprognostic (6.48e-3)	unprognostic (1.42e-2)
				unprognostic (9.01e-2)	unprognostic (2.98e-2)
unprognostic (7.95e-3)	unprognostic (9.43e-2)	unprognostic (1.28e-1)	unprognostic (6.35e-3)	unprognostic (1.39e-1)	unprognostic (1.70e-1)
unprognostic (9.69e-3)	unprognostic (4.36e-3)	unprognostic (3.47e-2)	unprognostic (1.30e-1)	unprognostic (6.23e-2)	unprognostic (1.48e-1)
unprognostic (3.73e-2)	unprognostic (1.02e-1)	unprognostic (1.46e-2)	unprognostic (4.25e-2)	unprognostic (8.25e-2)	
unprognostic (2.52e-2)	unprognostic (1.76e-2)	unprognostic (1.25e-1)	unprognostic (9.51e-3)	potential prognostic unfavorable (5.32e-4)	unprognostic (1.02e-2)
unprognostic (7.98e-3)	unprognostic (3.06e-2)	unprognostic (2.93e-1)	unprognostic (1.21e-2)	unprognostic (3.07e-2)	unprognostic (1.64e-1)
unprognostic (3.96e-2)	unprognostic (2.74e-3)	unprognostic (5.70e-2)	unprognostic (7.03e-2)	unprognostic (1.08e-1)	unprognostic (7.30e-2)
unprognostic (2.75e-2)	unprognostic (4.86e-3)	unprognostic (2.19e-1)	unprognostic (1.35e-1)		unprognostic (4.84e-2)
unprognostic (4.34e-2)		unprognostic (3.35e-1)	unprognostic (2.64e-1)	unprognostic (3.63e-2)	unprognostic (2.45e-2)
unprognostic (1.94e-3)	potential prognostic unfavorable (8.41e-4)	unprognostic (8.47e-2)	unprognostic (1.06e-3)	unprognostic (5.04e-2)	unprognostic (3.75e-2)
unprognostic (8.63e-3)	unprognostic (6.40e-2)	unprognostic (9.42e-2)			unprognostic (1.31e-3)
			unprognostic (7.14e-3)	unprognostic (1.11e-1)	unprognostic (1.88e-1)
unprognostic (1.35e-1)	unprognostic (4.01e-2)	unprognostic (1.19e-1)			unprognostic (2.08e-2)
unprognostic (1.08e-1)	unprognostic (1.05e-1)	unprognostic (2.19e-2)	potential prognostic unfavorable (1.79e-4)	unprognostic (3.06e-1)	unprognostic (1.26e-1)
unprognostic (9.72e-2)	unprognostic (6.00e-2)	unprognostic (3.45e-1)		potential prognostic favorable (8.62e-4)	
unprognostic (2.20e-1)	unprognostic (2.44e-1)	unprognostic (1.40e-1)	unprognostic (2.16e-2)	unprognostic (1.89e-2)	potential prognostic unfavorable (6.65e-4)
unprognostic (4.82e-2)	unprognostic (2.08e-1)	unprognostic (1.48e-2)	unprognostic (1.21e-2)	unprognostic (5.76e-3)	potential prognostic unfavorable (6.60e-5)
unprognostic (2.54e-1)	unprognostic (8.35e-3)	unprognostic (1.07e-1)	potential prognostic unfavorable (9.92e-4)	unprognostic (2.23e-2)	unprognostic (1.57e-1)
unprognostic (1.12e-1)	unprognostic (1.29e-1)	unprognostic (1.70e-1)	unprognostic (3.97e-2)	unprognostic (2.39e-1)	unprognostic (3.43e-3)
potential prognostic unfavorable (8.94e-4)	unprognostic (1.22e-1)	unprognostic (1.09e-1)	unprognostic (2.71e-1)	unprognostic (5.99e-2)	
unprognostic (1.83e-1)	unprognostic (1.85e-2)	unprognostic (2.28e-1)	unprognostic (1.15e-1)	unprognostic (4.42e-2)	unprognostic (6.33e-3)
			unprognostic (1.61e-1)	unprognostic (2.25e-1)	
unprognostic (2.08e-1)	unprognostic (3.79e-2)	unprognostic (8.89e-2)	unprognostic (5.58e-2)	unprognostic (1.59e-3)	unprognostic (4.96e-2)
unprognostic (4.04e-2)	unprognostic (3.57e-2)	potential prognostic favorable (2.64e-4)	unprognostic (1.42e-1)	unprognostic (1.99e-1)	unprognostic (1.05e-1)
unprognostic (7.91e-2)	unprognostic (2.05e-2)	unprognostic (4.27e-3)	unprognostic (1.37e-1)	potential prognostic unfavorable (1.38e-4)	unprognostic (3.80e-1)
unprognostic (6.01e-2)	potential prognostic unfavorable (3.78e-4)	unprognostic (2.16e-1)	unprognostic (6.28e-2)	unprognostic (1.64e-1)	unprognostic (1.46e-3)
unprognostic (6.76e-2)	unprognostic (1.25e-1)	unprognostic (1.08e-1)	unprognostic (3.03e-2)	unprognostic (1.73e-1)	unprognostic (1.72e-1)
unprognostic (2.58e-2)	unprognostic (4.10e-2)	unprognostic (3.34e-1)	unprognostic (5.97e-2)	unprognostic (2.70e-3)	unprognostic (2.89e-3)
unprognostic (1.84e-2)	unprognostic (6.81e-2)	unprognostic (3.80e-2)	unprognostic (5.07e-2)	unprognostic (3.62e-1)	unprognostic (5.63e-2)
unprognostic (7.25e-2)	unprognostic (1.35e-3)	unprognostic (2.85e-1)	unprognostic (6.02e-3)	unprognostic (1.47e-2)	unprognostic (5.75e-2)
unprognostic (7.60e-2)	unprognostic (2.78e-1)	unprognostic (6.70e-3)	unprognostic (2.86e-2)	unprognostic (4.61e-2)	unprognostic (1.06e-1)
potential prognostic unfavorable (6.93e-4)	unprognostic (2.07e-2)	potential prognostic unfavorable (8.69e-5)	unprognostic (9.53e-2)	unprognostic (1.17e-2)	potential prognostic unfavorable (6.07e-5)
unprognostic (1.62e-2)	unprognostic (7.20e-2)	unprognostic (1.61e-1)	unprognostic (6.24e-2)	unprognostic (1.69e-2)	unprognostic (8.38e-2)
			unprognostic (1.19e-1)	unprognostic (2.84e-1)	
unprognostic (2.44e-3)	unprognostic (1.25e-3)	potential prognostic unfavorable (5.93e-4)	unprognostic (1.06e-3)	unprognostic (3.96e-1)	unprognostic (2.69e-3)
potential prognostic unfavorable (4.98e-4)	unprognostic (7.80e-3)	unprognostic (4.92e-2)	unprognostic (7.48e-2)	unprognostic (3.83e-1)	unprognostic (7.37e-3)
unprognostic (3.05e-2)	unprognostic (1.69e-1)	unprognostic (1.17e-1)	unprognostic (4.63e-2)	unprognostic (1.64e-1)	unprognostic (1.86e-3)
unprognostic (1.84e-1)	unprognostic (8.90e-2)		unprognostic (2.91e-1)	unprognostic (2.48e-1)	unprognostic (1.77e-2)
unprognostic (4.47e-1)	unprognostic (3.11e-2)	unprognostic (2.14e-3)	unprognostic (5.09e-3)	unprognostic (3.34e-1)	potential prognostic unfavorable (2.29e-4)
unprognostic (1.57e-3)	unprognostic (1.45e-2)	unprognostic (7.43e-3)	unprognostic (1.58e-1)	unprognostic (2.19e-1)	unprognostic (7.93e-2)
unprognostic (1.20e-1)	unprognostic (6.69e-3)	unprognostic (8.66e-3)	unprognostic (2.57e-1)	unprognostic (5.40e-2)	potential prognostic favorable (5.98e-5)
unprognostic (3.45e-2)	unprognostic (6.95e-2)	unprognostic (3.60e-1)	unprognostic (2.55e-1)	unprognostic (5.32e-1)	unprognostic (3.27e-2)
unprognostic (2.78e-1)	unprognostic (1.33e-1)	unprognostic (1.23e-1)	unprognostic (8.62e-3)	potential prognostic unfavorable (2.79e-4)	unprognostic (2.17e-1)
unprognostic (4.92e-2)	unprognostic (3.63e-1)	unprognostic (8.86e-2)	unprognostic (2.51e-2)	unprognostic (1.71e-1)	unprognostic (2.37e-2)
unprognostic (2.55e-2)	unprognostic (1.25e-1)	unprognostic (2.61e-1)	unprognostic (6.55e-3)	unprognostic (2.69e-3)	unprognostic (5.51e-2)
unprognostic (2.33e-2)	unprognostic (1.14e-1)	unprognostic (7.84e-2)	unprognostic (1.54e-1)	unprognostic (1.06e-2)	unprognostic (7.31e-3)
unprognostic (9.25e-2)	unprognostic (4.21e-3)	unprognostic (2.90e-1)	potential prognostic unfavorable (2.53e-4)	unprognostic (1.47e-1)	unprognostic (2.67e-1)
unprognostic (1.19e-1)	unprognostic (2.39e-1)	potential prognostic favorable (2.37e-4)	unprognostic (6.81e-3)	unprognostic (1.37e-1)	unprognostic (1.83e-1)
unprognostic (2.18e-2)	unprognostic (7.24e-3)	unprognostic (2.74e-1)	unprognostic (1.42e-1)	unprognostic (3.46e-2)	unprognostic (1.60e-2)
unprognostic (8.03e-2)	unprognostic (4.87e-3)	unprognostic (3.68e-1)			
unprognostic (1.19e-1)	unprognostic (2.13e-2)	unprognostic (7.89e-2)	unprognostic (1.16e-1)	unprognostic (1.27e-1)	unprognostic (3.83e-2)
unprognostic (1.28e-1)	unprognostic (1.06e-1)	unprognostic (3.79e-1)	unprognostic (7.77e-2)	unprognostic (2.99e-1)	unprognostic (1.62e-1)
unprognostic (3.26e-3)	unprognostic (4.69e-2)	unprognostic (3.40e-1)	unprognostic (9.76e-2)	unprognostic (5.59e-3)	unprognostic (2.57e-2)
unprognostic (7.72e-3)	unprognostic (3.08e-1)	unprognostic (2.95e-2)	unprognostic (2.24e-2)	unprognostic (3.43e-2)	unprognostic (2.50e-2)
unprognostic (4.95e-2)	unprognostic (5.02e-2)	unprognostic (7.75e-2)	unprognostic (4.01e-2)	unprognostic (5.84e-2)	potential prognostic unfavorable (2.68e-4)
unprognostic (1.24e-2)	unprognostic (8.10e-2)	unprognostic (9.17e-3)	unprognostic (2.18e-1)	unprognostic (2.12e-1)	unprognostic (1.53e-1)
unprognostic (2.25e-1)	unprognostic (2.36e-2)	unprognostic (3.70e-1)	unprognostic (3.15e-2)	unprognostic (3.27e-2)	unprognostic (2.25e-1)
unprognostic (9.91e-2)	unprognostic (1.40e-1)	unprognostic (9.47e-2)	unprognostic (1.85e-2)	unprognostic (2.08e-2)	unprognostic (1.47e-1)
unprognostic (6.19e-2)	unprognostic (2.00e-2)	unprognostic (6.34e-2)	unprognostic (4.64e-2)	unprognostic (2.23e-1)	unprognostic (1.26e-1)
unprognostic (6.21e-2)	unprognostic (1.49e-2)	unprognostic (1.45e-1)	unprognostic (2.76e-1)	unprognostic (1.20e-1)	unprognostic (1.89e-2)
unprognostic (2.09e-1)	unprognostic (1.17e-2)	unprognostic (1.82e-1)	unprognostic (1.62e-1)	unprognostic (3.03e-1)	unprognostic (1.27e-1)
unprognostic (8.35e-2)	unprognostic (1.41e-1)	potential prognostic favorable (4.92e-4)		unprognostic (1.79e-2)	unprognostic (5.15e-3)
unprognostic (1.44e-1)	unprognostic (1.81e-1)	unprognostic (1.27e-2)	unprognostic (9.99e-2)	potential prognostic unfavorable (9.53e-4)	unprognostic (5.40e-2)
unprognostic (1.95e-3)	unprognostic (1.32e-1)	unprognostic (9.03e-2)	unprognostic (9.59e-3)	unprognostic (1.98e-1)	unprognostic (4.33e-3)
unprognostic (2.05e-2)		unprognostic (1.15e-1)		unprognostic (8.00e-3)	
unprognostic (1.66e-1)	unprognostic (2.16e-2)	unprognostic (2.76e-1)	unprognostic (1.36e-1)	unprognostic (9.51e-2)	unprognostic (1.80e-2)
unprognostic (1.45e-1)	unprognostic (1.48e-1)	unprognostic (1.40e-1)	unprognostic (1.44e-1)	unprognostic (1.14e-1)	unprognostic (2.38e-2)
	unprognostic (1.41e-2)		unprognostic (3.12e-2)	unprognostic (7.35e-2)	unprognostic (1.32e-3)
unprognostic (1.56e-2)	unprognostic (1.72e-1)	unprognostic (1.27e-1)	unprognostic (1.24e-1)	unprognostic (1.85e-2)	validated prognostic unfavorable (9.24e-5)
	unprognostic (5.05e-2)				unprognostic (7.20e-2)
unprognostic (1.37e-1)	unprognostic (2.17e-3)	unprognostic (1.78e-1)	unprognostic (5.65e-2)	unprognostic (2.37e-2)	unprognostic (8.61e-2)
unprognostic (5.38e-2)	unprognostic (1.97e-2)	unprognostic (1.28e-2)	unprognostic (1.80e-1)		
unprognostic (7.74e-3)	unprognostic (1.17e-1)	unprognostic (1.85e-1)	unprognostic (7.73e-2)	unprognostic (2.32e-1)	unprognostic (1.78e-3)
unprognostic (9.70e-3)	unprognostic (1.12e-1)	unprognostic (1.44e-1)	unprognostic (1.46e-2)	unprognostic (1.22e-1)	unprognostic (1.13e-1)
potential prognostic favorable (5.59e-4)	unprognostic (1.98e-2)	unprognostic (1.72e-1)			
unprognostic (2.73e-3)	unprognostic (2.03e-1)	unprognostic (8.78e-2)	unprognostic (3.55e-2)	unprognostic (6.63e-2)	unprognostic (5.87e-2)
				unprognostic (3.40e-1)	
unprognostic (2.10e-3)	unprognostic (2.62e-2)	unprognostic (4.16e-3)	unprognostic (7.60e-3)	unprognostic (7.71e-3)	unprognostic (1.85e-1)
unprognostic (6.45e-2)	unprognostic (2.55e-2)	unprognostic (3.33e-1)	unprognostic (4.89e-2)	unprognostic (3.97e-2)	unprognostic (3.61e-2)
unprognostic (2.86e-1)	unprognostic (3.35e-2)	unprognostic (1.16e-1)	unprognostic (1.72e-1)	unprognostic (5.19e-2)	unprognostic (4.66e-3)
unprognostic (1.04e-1)	unprognostic (1.17e-1)	unprognostic (4.59e-2)	unprognostic (1.05e-1)	unprognostic (3.39e-3)	unprognostic (7.33e-2)
unprognostic (2.34e-1)					
unprognostic (7.25e-2)	unprognostic (1.71e-1)	unprognostic (9.68e-2)	unprognostic (1.66e-1)	unprognostic (7.26e-2)	unprognostic (6.87e-3)
unprognostic (5.27e-2)	unprognostic (1.00e-1)	unprognostic (9.30e-2)	unprognostic (6.64e-2)	unprognostic (2.16e-1)	unprognostic (2.99e-3)
unprognostic (9.13e-3)	unprognostic (6.41e-2)	unprognostic (6.31e-2)	unprognostic (2.05e-2)	potential prognostic unfavorable (1.07e-5)	unprognostic (9.85e-3)
unprognostic (1.29e-1)	unprognostic (2.75e-1)	unprognostic (2.34e-1)	unprognostic (3.02e-1)	unprognostic (1.35e-1)	unprognostic (6.76e-3)
unprognostic (7.63e-2)	unprognostic (2.46e-1)	unprognostic (4.56e-2)	unprognostic (2.88e-1)	unprognostic (5.67e-2)	unprognostic (8.09e-2)
unprognostic (4.63e-1)	unprognostic (6.49e-2)	unprognostic (2.63e-1)	unprognostic (1.56e-2)	unprognostic (5.09e-2)	unprognostic (1.79e-1)
unprognostic (1.25e-3)	unprognostic (2.56e-1)	unprognostic (6.78e-2)	unprognostic (1.32e-1)	unprognostic (2.24e-1)	unprognostic (1.00e-3)
unprognostic (3.61e-1)	unprognostic (1.26e-2)	unprognostic (1.55e-2)	unprognostic (1.70e-1)	unprognostic (2.11e-1)	unprognostic (1.49e-1)
potential prognostic unfavorable (3.28e-4)	unprognostic (6.00e-2)	unprognostic (3.79e-2)	unprognostic (6.90e-3)	unprognostic (1.80e-1)	unprognostic (1.99e-1)
unprognostic (4.74e-2)	unprognostic (4.97e-2)	unprognostic (2.26e-1)			unprognostic (2.83e-1)
unprognostic (1.97e-1)	unprognostic (2.23e-2)	unprognostic (2.98e-1)	unprognostic (1.87e-1)	unprognostic (9.30e-2)	unprognostic (8.16e-2)
unprognostic (1.05e-3)	unprognostic (7.35e-2)	unprognostic (2.54e-2)	unprognostic (2.44e-1)	unprognostic (1.07e-1)	unprognostic (1.99e-1)
unprognostic (6.34e-2)	unprognostic (6.62e-2)	unprognostic (3.82e-1)	unprognostic (2.33e-2)	unprognostic (2.15e-2)	unprognostic (1.19e-3)
unprognostic (2.35e-1)		unprognostic (1.92e-1)			unprognostic (2.24e-1)
unprognostic (2.58e-1)	unprognostic (7.69e-2)	unprognostic (7.96e-2)	unprognostic (1.42e-3)	unprognostic (1.65e-1)	unprognostic (6.64e-2)
unprognostic (7.33e-3)	unprognostic (1.95e-1)	unprognostic (1.82e-1)	unprognostic (7.86e-2)	potential prognostic unfavorable (1.29e-4)	unprognostic (2.08e-1)
unprognostic (3.35e-2)	unprognostic (1.21e-1)	unprognostic (2.83e-1)	unprognostic (3.52e-1)	unprognostic (4.26e-1)	unprognostic (1.33e-1)
unprognostic (2.41e-1)	unprognostic (2.61e-2)	unprognostic (7.33e-3)	unprognostic (3.88e-2)		unprognostic (2.78e-2)
unprognostic (2.61e-1)	unprognostic (9.06e-2)	unprognostic (4.82e-1)	unprognostic (5.82e-2)	unprognostic (2.01e-1)	unprognostic (2.66e-1)
unprognostic (7.19e-2)	unprognostic (4.28e-2)	unprognostic (1.29e-1)	unprognostic (2.92e-2)	unprognostic (1.42e-2)	potential prognostic unfavorable (4.11e-4)
unprognostic (7.88e-2)	unprognostic (1.67e-1)	unprognostic (2.17e-1)	unprognostic (6.68e-2)	unprognostic (3.27e-1)	unprognostic (1.24e-1)
unprognostic (1.48e-1)	unprognostic (1.09e-1)	unprognostic (1.97e-1)	unprognostic (4.53e-1)	unprognostic (4.15e-1)	unprognostic (4.84e-2)

potential prognostic unfavorable (4.33e-5)	unprognostic (3.49e-1)	unprognostic (1.64e-1)	unprognostic (3.82e-1)	unprognostic (2.79e-1)	unprognostic (1.89e-3)
unprognostic (3.90e-1)		unprognostic (2.10e-2)			unprognostic (1.43e-2)
unprognostic (6.86e-3)	unprognostic (1.50e-1)	unprognostic (5.52e-2)	unprognostic (7.08e-2)	unprognostic (3.89e-3)	unprognostic (1.02e-2)
unprognostic (3.73e-1)	unprognostic (1.86e-1)	unprognostic (1.17e-1)	unprognostic (1.41e-1)	unprognostic (1.37e-3)	unprognostic (5.78e-2)
unprognostic (5.50e-2)	unprognostic (1.58e-2)	unprognostic (4.28e-2)	unprognostic (2.44e-2)	potential prognostic unfavorable (1.30e-5)	unprognostic (7.91e-2)
unprognostic (4.20e-1)	unprognostic (2.41e-1)	unprognostic (9.50e-2)	unprognostic (1.17e-1)	unprognostic (2.50e-1)	unprognostic (3.48e-3)
unprognostic (1.76e-1)	unprognostic (8.96e-2)	unprognostic (1.25e-2)	unprognostic (2.15e-1)	unprognostic (1.66e-1)	potential prognostic favorable (6.55e-5)
unprognostic (3.28e-3)	unprognostic (1.59e-2)	unprognostic (1.14e-2)	unprognostic (5.36e-3)	unprognostic (4.24e-3)	potential prognostic unfavorable (3.07e-5)
unprognostic (1.94e-2)	unprognostic (4.72e-2)	unprognostic (3.26e-1)	unprognostic (2.05e-3)	unprognostic (1.23e-2)	unprognostic (3.66e-2)
unprognostic (1.28e-1)	unprognostic (9.33e-2)	unprognostic (4.55e-2)	unprognostic (1.30e-1)	unprognostic (1.62e-1)	unprognostic (4.76e-2)
unprognostic (8.60e-2)	unprognostic (1.31e-2)	unprognostic (2.83e-1)	unprognostic (8.29e-2)	unprognostic (1.56e-1)	unprognostic (1.52e-1)
unprognostic (1.55e-1)	unprognostic (2.20e-3)	unprognostic (1.11e-1)	unprognostic (2.59e-1)	unprognostic (1.10e-1)	unprognostic (4.25e-2)
unprognostic (3.02e-1)	unprognostic (2.90e-1)	unprognostic (1.19e-1)	unprognostic (2.69e-2)	unprognostic (1.46e-1)	unprognostic (1.81e-1)
unprognostic (7.66e-2)	unprognostic (7.26e-2)	unprognostic (1.39e-1)	unprognostic (3.59e-1)	unprognostic (3.41e-1)	unprognostic (5.54e-2)
unprognostic (2.64e-1)	unprognostic (6.92e-2)	unprognostic (1.88e-1)	unprognostic (3.31e-1)	unprognostic (4.63e-2)	unprognostic (1.78e-2)
unprognostic (1.02e-1)	unprognostic (3.04e-1)	unprognostic (1.50e-1)	unprognostic (8.81e-2)	unprognostic (5.12e-2)	unprognostic (1.58e-2)
			unprognostic (1.56e-1)		unprognostic (2.61e-2)
unprognostic (6.12e-3)	unprognostic (1.25e-2)	unprognostic (3.65e-2)	unprognostic (3.85e-2)	unprognostic (3.32e-1)	unprognostic (1.95e-2)
unprognostic (1.77e-1)	unprognostic (8.59e-2)	unprognostic (1.19e-1)		unprognostic (4.11e-1)	unprognostic (1.97e-1)
unprognostic (4.98e-1)	unprognostic (1.61e-2)	unprognostic (4.97e-2)			unprognostic (1.63e-1)
unprognostic (2.89e-2)	unprognostic (5.84e-2)	unprognostic (1.57e-1)	unprognostic (3.40e-2)	unprognostic (2.23e-1)	unprognostic (9.76e-2)
unprognostic (6.69e-3)	unprognostic (7.59e-2)	unprognostic (1.43e-1)	unprognostic (3.05e-1)	unprognostic (2.11e-2)	potential prognostic unfavorable (8.91e-4)
potential prognostic unfavorable (7.35e-5)	unprognostic (2.18e-2)	unprognostic (1.23e-1)	unprognostic (5.10e-2)	unprognostic (3.43e-1)	unprognostic (8.41e-2)
unprognostic (4.80e-2)	unprognostic (3.87e-2)	unprognostic (9.02e-2)	unprognostic (1.06e-1)	unprognostic (3.29e-1)	unprognostic (2.07e-1)
unprognostic (6.71e-2)	unprognostic (3.71e-1)	unprognostic (1.95e-1)	unprognostic (1.74e-2)	unprognostic (4.93e-2)	unprognostic (4.31e-2)
unprognostic (1.79e-1)	unprognostic (2.56e-1)	unprognostic (2.79e-1)			
unprognostic (2.37e-1)	unprognostic (1.83e-2)	unprognostic (1.06e-1)			
unprognostic (3.70e-3)	unprognostic (3.49e-1)	unprognostic (1.55e-1)	unprognostic (4.51e-3)	unprognostic (3.35e-3)	unprognostic (9.49e-3)
unprognostic (1.67e-1)	unprognostic (7.67e-2)	unprognostic (1.38e-1)			unprognostic (1.10e-1)
unprognostic (6.08e-2)	unprognostic (3.37e-1)	unprognostic (4.69e-2)	unprognostic (2.86e-1)	unprognostic (1.43e-1)	potential prognostic unfavorable (2.28e-4)
unprognostic (9.04e-3)	unprognostic (8.70e-2)	unprognostic (1.20e-1)	unprognostic (2.96e-2)	unprognostic (1.18e-1)	unprognostic (1.58e-1)
unprognostic (5.33e-3)	unprognostic (4.45e-1)	unprognostic (2.37e-1)	potential prognostic favorable (3.86e-5)	unprognostic (1.97e-2)	unprognostic (3.98e-1)
unprognostic (2.25e-1)	unprognostic (5.00e-1)	unprognostic (1.44e-1)	unprognostic (8.79e-2)	unprognostic (7.73e-2)	unprognostic (1.22e-1)
unprognostic (1.88e-1)	unprognostic (1.98e-1)	unprognostic (5.39e-2)	unprognostic (5.38e-3)		unprognostic (7.48e-2)
unprognostic (1.29e-2)	unprognostic (1.98e-2)	unprognostic (3.00e-2)	unprognostic (2.27e-1)	unprognostic (2.71e-1)	unprognostic (2.49e-1)
unprognostic (3.07e-2)	unprognostic (1.57e-1)	unprognostic (2.17e-2)	unprognostic (1.51e-1)	unprognostic (2.00e-1)	unprognostic (9.95e-3)
unprognostic (1.70e-2)		unprognostic (2.76e-1)			unprognostic (1.26e-1)
unprognostic (1.25e-2)	unprognostic (2.49e-1)	unprognostic (1.76e-1)	unprognostic (1.03e-1)	unprognostic (1.27e-1)	unprognostic (1.77e-3)
unprognostic (1.44e-1)	potential prognostic unfavorable (9.98e-5)	unprognostic (1.61e-1)	unprognostic (1.36e-1)	unprognostic (2.95e-2)	unprognostic (2.77e-3)
unprognostic (1.02e-1)	unprognostic (7.09e-2)	unprognostic (8.64e-2)	unprognostic (1.95e-3)	unprognostic (1.56e-1)	unprognostic (1.37e-1)
unprognostic (1.61e-2)	unprognostic (9.69e-2)	unprognostic (7.25e-2)	unprognostic (7.67e-2)	unprognostic (3.15e-1)	unprognostic (6.20e-2)
unprognostic (4.94e-3)	unprognostic (1.01e-1)	unprognostic (1.75e-1)	unprognostic (8.71e-2)	unprognostic (3.25e-1)	unprognostic (8.71e-2)
unprognostic (2.97e-1)	unprognostic (1.39e-1)	unprognostic (2.16e-1)	unprognostic (4.58e-2)	unprognostic (8.39e-2)	unprognostic (1.74e-2)
unprognostic (5.63e-2)	unprognostic (2.43e-1)	unprognostic (2.09e-1)	unprognostic (1.31e-1)	unprognostic (1.21e-1)	unprognostic (7.60e-2)
unprognostic (3.86e-2)	potential prognostic favorable (5.09e-4)	unprognostic (4.27e-1)	unprognostic (2.59e-2)	unprognostic (3.02e-2)	potential prognostic unfavorable (1.07e-4)
unprognostic (5.60e-3)	unprognostic (1.41e-1)	unprognostic (1.86e-2)	unprognostic (5.80e-2)	unprognostic (1.37e-1)	unprognostic (1.17e-1)
unprognostic (1.02e-2)	unprognostic (2.65e-2)	unprognostic (1.91e-1)	unprognostic (1.19e-1)	unprognostic (1.20e-1)	unprognostic (2.52e-2)
unprognostic (2.11e-2)	unprognostic (2.13e-1)	unprognostic (8.02e-2)	unprognostic (7.77e-2)	unprognostic (1.69e-2)	unprognostic (2.05e-2)
unprognostic (5.87e-2)	unprognostic (1.60e-1)	unprognostic (3.47e-1)	unprognostic (2.30e-1)	unprognostic (1.33e-2)	unprognostic (4.80e-2)
unprognostic (1.61e-2)	unprognostic (5.62e-2)	unprognostic (3.69e-3)	unprognostic (9.81e-2)	unprognostic (4.14e-1)	unprognostic (1.66e-2)
unprognostic (3.30e-2)	unprognostic (4.46e-2)	unprognostic (1.19e-1)	unprognostic (1.04e-1)	unprognostic (2.13e-3)	unprognostic (7.78e-2)
unprognostic (1.25e-1)		unprognostic (1.62e-1)			unprognostic (1.19e-1)
unprognostic (7.69e-2)	unprognostic (2.48e-2)	unprognostic (7.27e-2)	unprognostic (4.31e-2)	unprognostic (1.22e-1)	unprognostic (2.90e-2)
unprognostic (3.86e-2)	unprognostic (1.88e-1)	unprognostic (2.81e-1)			
unprognostic (8.83e-2)	unprognostic (1.11e-2)	unprognostic (4.34e-2)	unprognostic (1.62e-2)	unprognostic (6.46e-2)	unprognostic (1.63e-3)
unprognostic (2.09e-1)	unprognostic (2.55e-2)	unprognostic (1.95e-1)	unprognostic (8.25e-2)	unprognostic (5.76e-2)	unprognostic (4.29e-2)
unprognostic (9.86e-2)	unprognostic (1.52e-2)	unprognostic (3.62e-2)	unprognostic (3.50e-1)	unprognostic (1.28e-1)	unprognostic (1.56e-1)
potential prognostic unfavorable (2.79e-5)	unprognostic (2.48e-1)	unprognostic (9.15e-3)	unprognostic (2.60e-2)	unprognostic (1.18e-1)	unprognostic (1.88e-2)
unprognostic (1.57e-2)	unprognostic (7.11e-3)	unprognostic (2.30e-1)	unprognostic (1.88e-3)	unprognostic (1.72e-1)	unprognostic (6.34e-2)
unprognostic (8.67e-2)	unprognostic (1.84e-1)	unprognostic (1.11e-1)		unprognostic (4.32e-2)	unprognostic (2.67e-2)
unprognostic (1.48e-1)	unprognostic (2.18e-1)	unprognostic (1.79e-1)			unprognostic (1.83e-2)
unprognostic (2.11e-2)	unprognostic (1.36e-1)	unprognostic (2.38e-1)	unprognostic (7.38e-3)	unprognostic (6.76e-2)	unprognostic (1.98e-2)
unprognostic (3.07e-2)	unprognostic (6.16e-2)	unprognostic (2.60e-1)	unprognostic (5.22e-2)	unprognostic (2.24e-2)	potential prognostic unfavorable (6.07e-4)
unprognostic (1.12e-2)	unprognostic (2.94e-3)	unprognostic (1.70e-1)	unprognostic (1.62e-1)	unprognostic (7.84e-2)	unprognostic (3.92e-2)
unprognostic (3.59e-3)	unprognostic (1.44e-1)	unprognostic (1.10e-1)	unprognostic (6.34e-3)	unprognostic (1.32e-1)	potential prognostic unfavorable (7.10e-4)
unprognostic (2.72e-1)	unprognostic (2.27e-1)	unprognostic (2.37e-1)	unprognostic (3.73e-2)	unprognostic (1.19e-1)	unprognostic (4.53e-2)
unprognostic (7.08e-2)	unprognostic (3.18e-1)	unprognostic (7.55e-2)	unprognostic (1.45e-1)	unprognostic (9.37e-2)	unprognostic (5.60e-3)
unprognostic (4.06e-1)	unprognostic (6.82e-2)	unprognostic (1.99e-2)			unprognostic (9.97e-2)
unprognostic (8.13e-2)	unprognostic (7.43e-2)	unprognostic (2.49e-1)	unprognostic (8.10e-2)	unprognostic (2.41e-2)	
					unprognostic (2.23e-1)
unprognostic (7.28e-3)	unprognostic (1.24e-1)	unprognostic (4.34e-2)	unprognostic (6.47e-2)	unprognostic (2.67e-2)	unprognostic (4.42e-2)
	unprognostic (2.24e-1)	unprognostic (2.03e-2)			
potential prognostic unfavorable (8.69e-5)	unprognostic (1.07e-1)	unprognostic (2.25e-2)	unprognostic (4.28e-2)	unprognostic (6.80e-2)	potential prognostic unfavorable (5.07e-4)
	potential prognostic favorable (4.12e-4)	unprognostic (2.65e-1)			
unprognostic (9.85e-2)	unprognostic (1.57e-2)	unprognostic (1.68e-2)	unprognostic (1.30e-1)	unprognostic (3.70e-1)	unprognostic (2.40e-3)
	unprognostic (5.09e-2)		unprognostic (1.16e-1)	unprognostic (1.14e-1)	unprognostic (2.54e-2)
	unprognostic (1.19e-2)		unprognostic (2.75e-1)	unprognostic (1.85e-1)	
unprognostic (8.02e-2)	unprognostic (1.23e-2)	unprognostic (1.78e-1)			unprognostic (5.45e-2)
	unprognostic (1.66e-2)	unprognostic (1.31e-3)			
unprognostic (2.77e-1)	unprognostic (9.94e-3)	unprognostic (2.77e-2)	unprognostic (1.11e-2)	unprognostic (2.80e-1)	unprognostic (1.86e-3)
unprognostic (5.73e-3)	unprognostic (8.48e-2)	unprognostic (6.19e-2)	potential prognostic unfavorable (2.30e-4)	unprognostic (5.77e-2)	unprognostic (3.90e-3)
unprognostic (6.70e-2)	unprognostic (2.03e-1)	unprognostic (1.27e-1)	unprognostic (8.56e-2)	unprognostic (9.55e-2)	unprognostic (3.35e-3)
unprognostic (6.23e-2)	unprognostic (2.32e-3)		unprognostic (3.76e-2)	unprognostic (5.04e-2)	unprognostic (4.56e-3)
unprognostic (3.94e-3)	unprognostic (7.88e-2)	unprognostic (3.77e-2)	potential prognostic unfavorable (2.59e-4)	unprognostic (1.19e-2)	unprognostic (3.53e-3)
unprognostic (3.53e-2)	unprognostic (1.48e-1)	unprognostic (2.04e-1)	unprognostic (4.26e-1)	unprognostic (6.12e-2)	unprognostic (2.52e-1)
potential prognostic unfavorable (6.26e-4)	unprognostic (9.51e-3)	unprognostic (1.82e-1)	unprognostic (2.03e-3)	unprognostic (1.65e-2)	unprognostic (6.28e-3)
unprognostic (9.39e-3)	unprognostic (4.74e-3)	unprognostic (3.02e-1)	unprognostic (1.16e-2)	unprognostic (3.00e-3)	unprognostic (1.30e-1)
unprognostic (4.20e-3)	unprognostic (2.98e-1)	unprognostic (1.18e-1)	unprognostic (5.05e-3)	unprognostic (3.37e-1)	unprognostic (4.44e-2)
unprognostic (7.32e-2)	unprognostic (1.08e-1)	unprognostic (1.20e-1)	unprognostic (2.26e-1)	unprognostic (3.51e-3)	unprognostic (2.58e-3)
		unprognostic (2.02e-3)			
unprognostic (6.53e-2)	unprognostic (3.24e-2)	unprognostic (2.85e-1)	unprognostic (1.91e-2)		unprognostic (6.61e-2)
potential prognostic unfavorable (7.27e-4)	unprognostic (7.09e-3)	unprognostic (2.40e-2)	potential prognostic unfavorable (4.80e-4)	unprognostic (1.08e-1)	unprognostic (8.18e-3)
unprognostic (9.39e-2)	unprognostic (1.04e-1)	unprognostic (1.12e-2)			unprognostic (1.70e-1)
unprognostic (5.16e-2)	unprognostic (2.19e-1)	unprognostic (7.96e-2)	unprognostic (1.66e-2)	unprognostic (1.93e-2)	unprognostic (2.34e-1)
unprognostic (8.05e-2)	unprognostic (5.98e-2)	unprognostic (2.48e-3)			potential prognostic unfavorable (3.96e-7)
unprognostic (2.58e-2)	unprognostic (1.23e-1)	unprognostic (1.96e-1)	unprognostic (6.79e-3)	unprognostic (1.14e-1)	unprognostic (5.00e-2)
unprognostic (4.57e-2)	unprognostic (6.71e-2)	unprognostic (3.25e-1)	unprognostic (1.19e-3)	unprognostic (1.71e-1)	unprognostic (8.79e-3)
unprognostic (1.21e-1)	unprognostic (8.34e-2)	unprognostic (1.41e-1)	unprognostic (1.41e-1)	unprognostic (7.43e-3)	unprognostic (3.06e-2)
unprognostic (6.21e-2)	unprognostic (3.54e-2)	unprognostic (3.00e-1)			unprognostic (4.72e-2)
unprognostic (2.21e-1)	unprognostic (2.08e-1)	unprognostic (1.65e-1)	unprognostic (6.08e-2)	unprognostic (5.85e-3)	unprognostic (2.41e-1)
unprognostic (9.35e-2)	unprognostic (3.00e-1)	unprognostic (1.44e-1)	unprognostic (9.18e-3)	unprognostic (5.91e-2)	unprognostic (5.27e-2)
unprognostic (6.52e-3)	unprognostic (1.59e-1)	unprognostic (1.09e-1)	unprognostic (4.91e-2)	unprognostic (4.26e-1)	unprognostic (3.59e-1)
unprognostic (1.93e-2)	unprognostic (3.91e-2)	unprognostic (7.55e-2)	unprognostic (2.76e-2)	unprognostic (9.96e-2)	unprognostic (2.65e-1)
unprognostic (8.86e-2)	unprognostic (6.25e-3)	unprognostic (1.92e-2)	unprognostic (1.60e-1)	unprognostic (2.54e-1)	unprognostic (1.31e-2)
unprognostic (1.69e-2)	unprognostic (2.57e-2)	unprognostic (1.24e-1)	unprognostic (1.12e-1)	unprognostic (1.21e-2)	potential prognostic unfavorable (2.44e-4)
unprognostic (1.78e-2)	unprognostic (1.00e-1)	unprognostic (2.01e-1)	unprognostic (4.61e-2)	unprognostic (8.64e-3)	unprognostic (1.26e-2)
unprognostic (1.45e-2)	unprognostic (1.49e-1)	unprognostic (6.43e-2)	unprognostic (3.41e-1)	potential prognostic unfavorable (1.25e-5)	potential prognostic unfavorable (9.04e-6)
potential prognostic unfavorable (7.98e-5)	unprognostic (3.77e-2)	unprognostic (2.55e-1)	unprognostic (4.82e-2)	unprognostic (1.81e-1)	potential prognostic unfavorable (4.54e-4)
unprognostic (1.03e-1)	unprognostic (2.16e-1)	unprognostic (2.94e-1)	unprognostic (1.63e-1)	unprognostic (4.14e-2)	unprognostic (3.66e-2)
unprognostic (7.50e-2)		unprognostic (3.23e-1)	unprognostic (1.15e-3)	unprognostic (4.55e-3)	unprognostic (4.18e-2)
unprognostic (6.59e-2)	unprognostic (1.79e-1)	unprognostic (1.03e-1)	unprognostic (5.57e-2)	unprognostic (1.24e-1)	unprognostic (1.18e-2)
unprognostic (8.57e-2)	unprognostic (1.35e-1)	unprognostic (4.02e-1)			unprognostic (1.98e-1)
unprognostic (3.28e-2)	unprognostic (1.19e-2)	unprognostic (9.05e-2)	unprognostic (1.14e-3)	unprognostic (4.90e-3)	unprognostic (1.25e-1)
unprognostic (1.74e-1)	unprognostic (8.16e-2)	unprognostic (4.05e-2)	unprognostic (1.50e-1)	unprognostic (3.16e-1)	unprognostic (1.06e-1)
unprognostic (2.01e-1)	unprognostic (1.45e-2)	unprognostic (2.45e-1)	unprognostic (3.22e-2)	unprognostic (2.80e-1)	unprognostic (3.11e-2)
potential prognostic unfavorable (3.84e-4)	unprognostic (5.20e-2)	unprognostic (1.32e-2)	unprognostic (5.60e-3)	unprognostic (7.29e-3)	unprognostic (1.03e-1)
unprognostic (4.39e-2)	unprognostic (8.21e-2)	unprognostic (1.52e-1)	unprognostic (5.13e-2)	unprognostic (5.92e-2)	unprognostic (6.52e-3)
unprognostic (1.78e-1)	unprognostic (1.09e-1)	unprognostic (1.37e-1)	unprognostic (6.40e-3)	unprognostic (1.24e-2)	unprognostic (1.98e-1)
unprognostic (1.80e-2)	unprognostic (1.96e-1)	unprognostic (1.05e-1)	unprognostic (6.86e-3)	unprognostic (1.62e-1)	unprognostic (1.16e-1)
unprognostic (2.06e-1)	unprognostic (1.57e-1)	unprognostic (4.65e-2)	unprognostic (8.30e-2)	unprognostic (2.82e-1)	unprognostic (3.47e-2)

unprognostic (8.95e-2)	unprognostic (1.63e-1)	unprognostic (2.07e-1)	unprognostic (5.31e-3)	potential prognostic unfavorable (9.83e-6)	unprognostic (1.68e-1)
potential prognostic unfavorable (2.26e-4)	unprognostic (2.10e-1)	unprognostic (4.82e-2)	unprognostic (8.01e-2)		unprognostic (1.35e-1)
validated prognostic unfavorable (6.75e-5)	unprognostic (2.16e-1)	unprognostic (1.47e-1)	unprognostic (3.43e-2)	unprognostic (2.53e-1)	potential prognostic unfavorable (6.87e-5)
unprognostic (7.41e-2)	unprognostic (3.37e-1)	unprognostic (6.43e-2)	unprognostic (3.16e-3)	unprognostic (4.86e-2)	unprognostic (2.20e-1)
unprognostic (8.54e-3)	unprognostic (1.46e-1)	unprognostic (7.96e-2)	unprognostic (3.08e-2)	unprognostic (7.32e-3)	unprognostic (2.94e-3)
unprognostic (6.83e-3)	unprognostic (2.66e-1)	unprognostic (1.76e-1)	unprognostic (2.05e-2)	unprognostic (3.85e-1)	potential prognostic favorable (3.85e-4)
unprognostic (9.47e-3)	unprognostic (4.11e-2)	unprognostic (2.07e-1)	unprognostic (2.28e-2)	unprognostic (1.59e-1)	unprognostic (8.58e-2)
unprognostic (8.56e-2)	unprognostic (3.67e-1)	unprognostic (5.64e-2)	unprognostic (3.49e-2)	unprognostic (1.11e-3)	unprognostic (2.62e-1)
unprognostic (4.38e-2)	unprognostic (2.18e-1)	unprognostic (2.72e-1)	unprognostic (2.61e-1)	unprognostic (1.72e-1)	unprognostic (1.80e-2)
unprognostic (8.01e-2)	unprognostic (2.48e-1)	unprognostic (4.57e-3)	unprognostic (1.37e-2)	unprognostic (5.76e-3)	unprognostic (1.11e-1)
unprognostic (1.08e-1)	unprognostic (1.40e-1)	unprognostic (2.62e-1)	unprognostic (8.86e-2)	unprognostic (1.51e-1)	unprognostic (2.03e-1)
unprognostic (2.82e-1)	unprognostic (1.01e-1)	unprognostic (3.91e-1)	unprognostic (3.47e-1)	unprognostic (4.01e-2)	unprognostic (8.88e-2)
unprognostic (7.96e-2)	unprognostic (1.87e-1)	unprognostic (1.59e-1)	unprognostic (3.20e-1)	unprognostic (6.09e-2)	unprognostic (1.12e-2)
unprognostic (7.48e-2)	unprognostic (1.52e-2)	unprognostic (8.28e-3)	unprognostic (3.01e-1)		unprognostic (6.14e-2)
			unprognostic (3.92e-3)	unprognostic (2.59e-2)	
unprognostic (1.28e-2)	unprognostic (8.34e-2)	unprognostic (1.85e-1)	unprognostic (1.59e-1)	unprognostic (1.12e-1)	unprognostic (2.92e-3)
	unprognostic (2.73e-3)	unprognostic (2.26e-1)	unprognostic (3.02e-2)	unprognostic (2.63e-1)	unprognostic (3.82e-3)
unprognostic (1.43e-1)	unprognostic (2.47e-1)	unprognostic (2.74e-2)	unprognostic (9.21e-2)	unprognostic (4.76e-1)	potential prognostic unfavorable (4.26e-4)
unprognostic (1.64e-2)	unprognostic (3.23e-2)	unprognostic (2.13e-1)	unprognostic (9.11e-2)	unprognostic (3.22e-3)	unprognostic (1.19e-1)
unprognostic (3.58e-1)	unprognostic (2.93e-1)	unprognostic (2.47e-2)	unprognostic (9.38e-2)	unprognostic (1.16e-1)	potential prognostic favorable (2.17e-4)
unprognostic (1.45e-2)	unprognostic (7.23e-2)	unprognostic (5.53e-2)	unprognostic (4.52e-2)	unprognostic (2.84e-1)	unprognostic (2.53e-2)
unprognostic (2.45e-1)	unprognostic (7.31e-3)	unprognostic (8.20e-3)	unprognostic (2.17e-1)	unprognostic (6.04e-3)	unprognostic (7.50e-2)
unprognostic (1.79e-3)	unprognostic (2.34e-2)	unprognostic (1.62e-1)	unprognostic (2.98e-3)	potential prognostic unfavorable (1.36e-5)	unprognostic (8.61e-3)
unprognostic (1.57e-2)	potential prognostic unfavorable (7.94e-4)	unprognostic (1.01e-1)	unprognostic (6.55e-2)	unprognostic (7.17e-2)	unprognostic (1.32e-1)
unprognostic (9.84e-3)	unprognostic (2.24e-1)	unprognostic (2.06e-1)	unprognostic (7.40e-2)	unprognostic (1.46e-1)	potential prognostic unfavorable (5.49e-4)
unprognostic (4.15e-2)	unprognostic (3.08e-1)	unprognostic (8.76e-2)	unprognostic (6.58e-2)	unprognostic (5.48e-3)	unprognostic (4.64e-3)
unprognostic (2.60e-2)					potential prognostic favorable (1.07e-5)
unprognostic (1.38e-1)	unprognostic (6.90e-2)	unprognostic (2.25e-1)	unprognostic (3.35e-3)	unprognostic (2.91e-1)	unprognostic (5.73e-3)
unprognostic (2.23e-1)	unprognostic (1.31e-1)	unprognostic (3.67e-2)	unprognostic (1.90e-2)	unprognostic (1.24e-1)	unprognostic (2.15e-3)
potential prognostic unfavorable (9.09e-4)	unprognostic (1.86e-3)	unprognostic (2.79e-1)	unprognostic (2.02e-2)	unprognostic (4.69e-1)	potential prognostic unfavorable (7.67e-4)
unprognostic (3.16e-2)	unprognostic (1.28e-2)	unprognostic (3.80e-3)	unprognostic (1.40e-3)	unprognostic (2.05e-1)	unprognostic (7.77e-3)
unprognostic (1.57e-3)	unprognostic (5.09e-2)	unprognostic (1.74e-1)	unprognostic (7.71e-3)	unprognostic (3.85e-2)	unprognostic (4.76e-2)
unprognostic (2.47e-1)	unprognostic (5.54e-2)	unprognostic (3.67e-3)	unprognostic (8.38e-3)	unprognostic (8.89e-2)	unprognostic (4.25e-2)
unprognostic (2.56e-2)	unprognostic (1.06e-1)	unprognostic (9.34e-3)	unprognostic (2.38e-1)	unprognostic (2.78e-3)	unprognostic (5.45e-2)
unprognostic (4.91e-2)	unprognostic (4.32e-3)	unprognostic (2.33e-1)	unprognostic (4.71e-2)	unprognostic (2.13e-1)	unprognostic (2.84e-2)
unprognostic (1.23e-3)	unprognostic (3.51e-1)	unprognostic (1.16e-2)	unprognostic (7.15e-3)	unprognostic (2.84e-2)	unprognostic (6.59e-3)
unprognostic (3.68e-2)	unprognostic (2.06e-1)	unprognostic (2.21e-1)	unprognostic (1.71e-1)	unprognostic (1.32e-1)	unprognostic (3.16e-1)
unprognostic (6.83e-2)	unprognostic (1.53e-2)	unprognostic (2.48e-2)	unprognostic (1.84e-1)	unprognostic (8.99e-3)	unprognostic (2.52e-3)
unprognostic (2.93e-3)	unprognostic (4.25e-1)	unprognostic (2.36e-1)	unprognostic (5.76e-3)	unprognostic (3.92e-2)	unprognostic (1.58e-1)
unprognostic (6.53e-3)	unprognostic (2.49e-1)	unprognostic (7.58e-2)	unprognostic (3.21e-2)	unprognostic (1.30e-1)	unprognostic (1.43e-1)
unprognostic (2.04e-1)	unprognostic (1.25e-1)	unprognostic (1.84e-3)	unprognostic (6.19e-3)	unprognostic (1.67e-1)	potential prognostic unfavorable (8.33e-4)
unprognostic (4.53e-3)	unprognostic (1.59e-1)	unprognostic (3.54e-1)	unprognostic (1.72e-3)	unprognostic (7.64e-2)	unprognostic (1.65e-1)
unprognostic (1.83e-1)	unprognostic (1.07e-1)	unprognostic (1.65e-1)	unprognostic (2.18e-3)	unprognostic (1.95e-1)	unprognostic (2.51e-2)
unprognostic (4.60e-2)	unprognostic (4.09e-1)	unprognostic (7.65e-3)	unprognostic (2.74e-1)	unprognostic (7.77e-2)	unprognostic (1.04e-1)
unprognostic (2.74e-2)	unprognostic (5.14e-2)	unprognostic (4.88e-3)	unprognostic (2.48e-2)	unprognostic (2.18e-1)	unprognostic (3.62e-2)
unprognostic (2.64e-1)	unprognostic (2.90e-2)	unprognostic (1.46e-1)	unprognostic (6.79e-3)	unprognostic (1.76e-1)	potential prognostic unfavorable (1.75e-4)
unprognostic (1.21e-1)	unprognostic (4.25e-2)	unprognostic (6.80e-3)			unprognostic (2.09e-1)
unprognostic (1.05e-1)	unprognostic (6.67e-3)	unprognostic (8.32e-2)	unprognostic (9.50e-2)	unprognostic (6.14e-2)	
unprognostic (6.93e-3)	unprognostic (5.85e-3)	unprognostic (6.96e-3)	unprognostic (3.58e-3)	unprognostic (2.53e-2)	unprognostic (2.23e-2)
unprognostic (5.87e-2)	unprognostic (4.53e-3)	unprognostic (1.86e-1)	unprognostic (1.37e-1)	unprognostic (7.58e-2)	unprognostic (5.95e-3)
unprognostic (1.06e-2)	unprognostic (1.99e-1)	unprognostic (1.94e-1)	potential prognostic unfavorable (6.98e-4)	unprognostic (5.27e-1)	potential prognostic unfavorable (6.95e-4)
unprognostic (1.37e-1)	unprognostic (3.94e-1)	unprognostic (6.19e-2)	unprognostic (2.13e-2)	unprognostic (5.93e-2)	unprognostic (1.13e-2)
unprognostic (2.53e-2)	unprognostic (2.86e-1)	unprognostic (3.70e-1)	unprognostic (9.77e-2)	unprognostic (4.60e-2)	unprognostic (2.85e-3)
unprognostic (1.07e-3)	unprognostic (2.92e-3)	unprognostic (5.64e-2)	unprognostic (2.15e-2)	unprognostic (1.27e-1)	unprognostic (1.11e-3)
unprognostic (8.55e-2)	unprognostic (1.94e-2)	unprognostic (1.54e-1)	unprognostic (1.34e-1)	unprognostic (1.09e-2)	unprognostic (7.14e-2)
unprognostic (2.01e-1)	unprognostic (2.92e-1)	unprognostic (2.44e-1)	unprognostic (4.17e-2)	unprognostic (2.71e-1)	potential prognostic unfavorable (1.85e-4)
unprognostic (3.71e-2)	unprognostic (1.82e-1)	unprognostic (1.42e-1)	unprognostic (1.59e-1)	unprognostic (3.65e-2)	potential prognostic unfavorable (4.26e-4)
potential prognostic unfavorable (7.64e-4)	unprognostic (2.13e-2)	unprognostic (1.17e-1)	unprognostic (2.38e-1)		unprognostic (5.08e-2)
unprognostic (1.60e-2)	unprognostic (6.50e-2)	unprognostic (4.36e-3)	unprognostic (6.94e-3)	unprognostic (6.86e-2)	potential prognostic unfavorable (1.39e-4)
unprognostic (1.90e-2)	unprognostic (1.11e-2)	unprognostic (1.98e-2)	unprognostic (1.51e-1)	unprognostic (4.17e-3)	unprognostic (7.36e-2)
unprognostic (1.85e-2)	unprognostic (6.92e-3)	potential prognostic unfavorable (2.72e-4)	unprognostic (2.98e-2)	unprognostic (1.77e-2)	unprognostic (2.58e-2)
unprognostic (1.74e-1)		unprognostic (1.90e-2)	unprognostic (5.61e-2)	unprognostic (3.56e-2)	unprognostic (1.77e-3)
unprognostic (1.57e-2)	unprognostic (4.07e-2)	unprognostic (2.05e-1)	unprognostic (9.91e-2)	unprognostic (3.07e-1)	unprognostic (1.57e-1)
unprognostic (2.18e-3)	unprognostic (3.85e-3)	unprognostic (9.64e-2)	unprognostic (1.02e-3)	unprognostic (1.90e-2)	unprognostic (3.68e-2)
unprognostic (5.58e-2)	unprognostic (5.80e-2)	unprognostic (3.00e-1)	unprognostic (7.84e-2)	unprognostic (6.35e-2)	unprognostic (3.19e-2)
unprognostic (1.16e-1)	unprognostic (1.27e-1)	unprognostic (2.22e-1)	unprognostic (1.19e-1)	unprognostic (1.05e-1)	potential prognostic favorable (8.26e-4)
					unprognostic (4.73e-2)
unprognostic (1.56e-1)					unprognostic (3.24e-1)
potential prognostic unfavorable (8.38e-4)	unprognostic (6.62e-2)	unprognostic (1.97e-2)	unprognostic (1.22e-1)	unprognostic (7.19e-2)	potential prognostic favorable (5.58e-4)
unprognostic (2.62e-2)	unprognostic (2.87e-2)	unprognostic (2.08e-1)	unprognostic (9.67e-2)	unprognostic (1.46e-1)	unprognostic (1.90e-1)
unprognostic (4.81e-2)	unprognostic (1.33e-2)	unprognostic (2.55e-1)	unprognostic (1.25e-1)	unprognostic (8.39e-2)	unprognostic (1.58e-3)
unprognostic (8.29e-3)	unprognostic (1.70e-1)	unprognostic (1.74e-2)	unprognostic (1.07e-1)	unprognostic (3.58e-1)	potential prognostic unfavorable (2.37e-4)
unprognostic (1.31e-1)	potential prognostic unfavorable (9.13e-4)	unprognostic (9.78e-2)	unprognostic (2.50e-2)	unprognostic (2.84e-1)	unprognostic (1.05e-1)
unprognostic (1.70e-1)	unprognostic (1.38e-1)	unprognostic (2.23e-1)	unprognostic (4.83e-2)	unprognostic (4.62e-1)	unprognostic (3.01e-2)
unprognostic (5.01e-3)	potential prognostic unfavorable (1.33e-4)	unprognostic (5.01e-2)	unprognostic (5.80e-2)	unprognostic (2.68e-1)	potential prognostic unfavorable (4.11e-4)
unprognostic (9.15e-3)	unprognostic (2.65e-3)	unprognostic (2.15e-1)	unprognostic (3.53e-1)	potential prognostic unfavorable (8.13e-4)	unprognostic (3.90e-1)
unprognostic (1.98e-2)	potential prognostic unfavorable (9.96e-4)	unprognostic (6.93e-2)	unprognostic (2.98e-2)	unprognostic (1.34e-1)	unprognostic (9.09e-3)
unprognostic (1.16e-2)	unprognostic (3.01e-1)	unprognostic (3.39e-1)	unprognostic (1.59e-2)	unprognostic (1.12e-3)	unprognostic (2.99e-1)
unprognostic (1.19e-3)	unprognostic (1.64e-2)	unprognostic (6.71e-2)	unprognostic (2.52e-2)	unprognostic (1.97e-1)	unprognostic (1.96e-1)
potential prognostic unfavorable (2.57e-4)	unprognostic (7.49e-2)	unprognostic (1.32e-1)	unprognostic (1.17e-2)	unprognostic (1.78e-1)	unprognostic (2.80e-1)
potential prognostic unfavorable (6.99e-4)	unprognostic (9.37e-2)	unprognostic (1.49e-1)	unprognostic (1.30e-3)	unprognostic (3.43e-1)	unprognostic (4.95e-3)
unprognostic (1.04e-1)	unprognostic (8.18e-3)	unprognostic (7.02e-2)	unprognostic (1.45e-2)	unprognostic (6.71e-2)	unprognostic (3.54e-3)
unprognostic (2.27e-2)	unprognostic (6.32e-2)	unprognostic (1.13e-1)	unprognostic (4.23e-3)	unprognostic (1.49e-3)	unprognostic (1.98e-1)
unprognostic (2.49e-1)	unprognostic (1.33e-2)	unprognostic (2.56e-1)	unprognostic (6.91e-2)	unprognostic (2.83e-2)	unprognostic (4.35e-2)
unprognostic (1.32e-1)	unprognostic (8.83e-2)	unprognostic (2.47e-1)	unprognostic (5.72e-2)	unprognostic (6.81e-3)	validated prognostic unfavorable (6.43e-5)
unprognostic (3.11e-1)	potential prognostic unfavorable (5.94e-5)	unprognostic (1.39e-1)	unprognostic (5.81e-1)	unprognostic (2.03e-1)	unprognostic (1.75e-1)
	unprognostic (1.02e-1)	unprognostic (1.54e-2)	unprognostic (9.51e-2)	unprognostic (4.62e-2)	unprognostic (1.15e-2)
	unprognostic (9.13e-2)				
	unprognostic (9.49e-2)	unprognostic (2.54e-2)	unprognostic (2.74e-1)		
	unprognostic (2.91e-1)	unprognostic (1.91e-1)			unprognostic (6.80e-2)
validated prognostic unfavorable (1.15e-5)	unprognostic (8.44e-2)	unprognostic (2.50e-1)	unprognostic (6.92e-2)	unprognostic (1.08e-1)	unprognostic (4.63e-3)
unprognostic (3.13e-1)	unprognostic (8.89e-2)	unprognostic (6.11e-2)	unprognostic (7.80e-2)	unprognostic (1.53e-2)	unprognostic (9.68e-2)
unprognostic (8.39e-2)	unprognostic (1.62e-1)	unprognostic (4.57e-2)	unprognostic (1.06e-1)	unprognostic (1.18e-2)	unprognostic (3.89e-1)
unprognostic (2.51e-1)	unprognostic (4.10e-2)	unprognostic (3.96e-2)	unprognostic (1.38e-1)	unprognostic (4.64e-3)	unprognostic (4.16e-2)
unprognostic (3.01e-2)	unprognostic (3.36e-1)	unprognostic (6.53e-2)	unprognostic (5.40e-2)		unprognostic (7.03e-2)
unprognostic (2.92e-2)	unprognostic (1.51e-1)	unprognostic (8.98e-2)	unprognostic (5.90e-2)		unprognostic (2.97e-3)
unprognostic (5.76e-2)	unprognostic (1.21e-1)	unprognostic (1.43e-1)	unprognostic (6.98e-2)	unprognostic (2.57e-1)	unprognostic (1.39e-1)
unprognostic (3.00e-2)	unprognostic (2.82e-1)	unprognostic (2.40e-1)	unprognostic (1.43e-1)	unprognostic (4.59e-2)	unprognostic (5.51e-2)
unprognostic (2.72e-2)	unprognostic (3.13e-1)	unprognostic (1.76e-2)	unprognostic (9.99e-2)	unprognostic (1.12e-1)	potential prognostic unfavorable (1.22e-4)
	unprognostic (3.07e-1)	unprognostic (1.98e-1)			potential prognostic unfavorable (1.04e-4)
unprognostic (1.02e-1)	unprognostic (1.14e-2)	unprognostic (3.08e-1)		unprognostic (3.27e-1)	unprognostic (1.28e-2)
potential prognostic unfavorable (1.35e-5)	unprognostic (6.08e-2)	unprognostic (2.89e-3)	unprognostic (2.56e-2)	unprognostic (1.34e-1)	unprognostic (3.36e-1)
			unprognostic (6.68e-2)	unprognostic (9.89e-2)	unprognostic (2.26e-2)
unprognostic (1.44e-1)	unprognostic (2.19e-3)	unprognostic (1.23e-1)	unprognostic (1.47e-2)	unprognostic (1.77e-1)	unprognostic (3.85e-1)
unprognostic (1.15e-1)	unprognostic (2.54e-2)	unprognostic (1.29e-1)	unprognostic (3.41e-3)	unprognostic (1.16e-1)	unprognostic (2.47e-2)
					unprognostic (7.38e-2)
					unprognostic (2.87e-1)
unprognostic (1.36e-1)					
unprognostic (5.28e-3)	unprognostic (7.12e-2)	unprognostic (8.62e-2)	unprognostic (1.50e-1)	unprognostic (5.47e-1)	unprognostic (1.58e-1)
unprognostic (3.99e-2)	unprognostic (5.08e-2)	unprognostic (3.05e-1)	unprognostic (2.51e-1)	unprognostic (1.64e-2)	unprognostic (2.17e-1)
potential prognostic unfavorable (3.14e-4)	unprognostic (2.57e-1)	unprognostic (2.36e-2)	unprognostic (9.63e-2)	unprognostic (2.53e-1)	unprognostic (5.12e-2)
potential prognostic unfavorable (8.45e-4)	unprognostic (4.42e-1)	unprognostic (4.35e-1)	unprognostic (1.64e-1)	unprognostic (1.27e-1)	unprognostic (1.09e-2)
unprognostic (2.80e-1)	unprognostic (2.94e-1)	unprognostic (5.63e-2)	unprognostic (1.79e-2)	unprognostic (2.50e-2)	unprognostic (6.25e-2)
unprognostic (1.44e-1)	unprognostic (3.29e-2)	unprognostic (6.43e-3)	unprognostic (1.86e-2)	unprognostic (1.24e-1)	unprognostic (3.61e-2)
unprognostic (2.38e-1)	unprognostic (5.35e-2)	unprognostic (1.47e-1)	unprognostic (2.58e-1)	unprognostic (4.94e-1)	unprognostic (8.72e-2)
unprognostic (4.91e-2)	unprognostic (2.55e-3)	unprognostic (9.29e-2)	unprognostic (2.54e-1)	unprognostic (1.54e-1)	unprognostic (2.14e-2)
unprognostic (2.78e-1)		unprognostic (1.34e-1)			
unprognostic (1.89e-1)	unprognostic (1.04e-1)	unprognostic (3.21e-2)	unprognostic (6.80e-2)	unprognostic (1.65e-1)	unprognostic (7.41e-2)

					unprognostic (1.29e-1)
	unprognostic (1.18e-2)	unprognostic (4.59e-2)			
unprognostic (1.53e-1)	unprognostic (8.54e-2)	unprognostic (1.85e-2)	unprognostic (2.02e-1)		
unprognostic (1.50e-1)	potential prognostic unfavorable (3.60e-4)	unprognostic (1.04e-1)	unprognostic (5.34e-3)	unprognostic (1.64e-1)	potential prognostic unfavorable (9.31e-5)
unprognostic (1.74e-2)	unprognostic (1.21e-2)	unprognostic (2.13e-1)	unprognostic (1.44e-3)	unprognostic (4.26e-1)	unprognostic (4.36e-2)
unprognostic (3.65e-2)	unprognostic (1.39e-3)	unprognostic (2.48e-1)	unprognostic (1.98e-1)	unprognostic (1.03e-1)	unprognostic (1.56e-2)
unprognostic (2.83e-1)	unprognostic (1.67e-2)	unprognostic (4.76e-1)	unprognostic (3.50e-3)	unprognostic (9.85e-2)	unprognostic (1.48e-1)
unprognostic (9.67e-2)	unprognostic (3.31e-2)	unprognostic (4.66e-2)	unprognostic (2.68e-1)	unprognostic (1.01e-3)	unprognostic (1.13e-1)
unprognostic (8.08e-2)	unprognostic (1.34e-1)	unprognostic (2.28e-1)	unprognostic (1.24e-3)	unprognostic (3.41e-1)	potential prognostic unfavorable (7.07e-4)
	unprognostic (8.59e-2)		unprognostic (1.32e-3)	unprognostic (8.04e-3)	potential prognostic favorable (4.37e-4)
unprognostic (2.21e-1)	unprognostic (2.85e-2)	unprognostic (3.51e-1)		unprognostic (1.63e-2)	unprognostic (4.45e-1)
unprognostic (4.03e-2)	unprognostic (1.27e-1)	unprognostic (1.12e-1)			unprognostic (2.05e-1)
validated prognostic unfavorable (4.23e-4)	unprognostic (1.10e-1)	unprognostic (1.09e-3)	unprognostic (5.38e-2)	unprognostic (3.05e-2)	potential prognostic unfavorable (2.28e-5)
unprognostic (5.42e-2)	unprognostic (4.80e-2)	unprognostic (9.28e-2)	unprognostic (3.17e-2)	unprognostic (1.61e-1)	unprognostic (6.07e-2)
unprognostic (1.13e-1)	unprognostic (2.05e-1)	unprognostic (1.14e-1)	unprognostic (2.97e-1)	unprognostic (2.71e-2)	unprognostic (1.20e-1)
	unprognostic (1.17e-2)	unprognostic (1.00e-1)			unprognostic (2.99e-1)
unprognostic (3.02e-1)	unprognostic (3.80e-1)	unprognostic (7.52e-2)			unprognostic (4.97e-2)
unprognostic (2.35e-2)	unprognostic (3.97e-1)	unprognostic (1.58e-1)	unprognostic (6.78e-2)	unprognostic (4.63e-1)	unprognostic (5.84e-2)
unprognostic (6.00e-2)	unprognostic (8.21e-2)	unprognostic (5.60e-2)	unprognostic (1.18e-1)	unprognostic (2.73e-1)	unprognostic (2.19e-2)
unprognostic (5.08e-2)	unprognostic (3.75e-1)	unprognostic (1.30e-1)	unprognostic (1.86e-1)	unprognostic (2.77e-2)	unprognostic (2.01e-3)
unprognostic (1.53e-1)	unprognostic (1.35e-3)	unprognostic (8.98e-2)	unprognostic (2.76e-1)	unprognostic (1.79e-2)	unprognostic (2.29e-1)
unprognostic (3.11e-2)	unprognostic (2.73e-2)	unprognostic (3.40e-1)	unprognostic (9.58e-2)	unprognostic (1.13e-1)	unprognostic (3.94e-2)
unprognostic (8.08e-2)	unprognostic (7.44e-2)	unprognostic (2.09e-1)	unprognostic (3.59e-1)	unprognostic (2.14e-1)	unprognostic (1.95e-1)
unprognostic (5.75e-2)	unprognostic (2.01e-1)	unprognostic (2.28e-3)	unprognostic (1.59e-1)	unprognostic (1.10e-1)	unprognostic (6.91e-3)
unprognostic (2.61e-3)	unprognostic (1.67e-3)	unprognostic (1.70e-1)	unprognostic (2.13e-1)	unprognostic (4.66e-2)	unprognostic (5.38e-2)
potential prognostic unfavorable (1.54e-5)	unprognostic (6.03e-2)	unprognostic (8.71e-2)	unprognostic (7.41e-3)	unprognostic (1.81e-2)	potential prognostic unfavorable (1.21e-4)
unprognostic (4.65e-3)	unprognostic (8.69e-2)	unprognostic (1.12e-1)	unprognostic (3.98e-2)	unprognostic (1.90e-1)	unprognostic (7.34e-2)
unprognostic (2.65e-2)	unprognostic (1.60e-1)	unprognostic (1.04e-1)	unprognostic (1.24e-1)	unprognostic (3.00e-1)	unprognostic (3.59e-2)
	unprognostic (1.76e-1)	unprognostic (1.58e-2)			
unprognostic (3.59e-2)	unprognostic (1.54e-1)	unprognostic (5.65e-2)	unprognostic (1.03e-2)	unprognostic (5.16e-3)	unprognostic (9.94e-2)
potential prognostic unfavorable (8.27e-4)	unprognostic (2.66e-1)	unprognostic (1.55e-1)	unprognostic (1.27e-1)	unprognostic (1.80e-2)	unprognostic (2.85e-1)
unprognostic (3.47e-2)	unprognostic (2.52e-1)	unprognostic (1.77e-2)	unprognostic (6.49e-2)	potential prognostic unfavorable (5.17e-4)	unprognostic (2.60e-3)
unprognostic (7.53e-2)	unprognostic (3.97e-1)	unprognostic (2.86e-1)	unprognostic (3.03e-2)	potential prognostic unfavorable (3.63e-4)	unprognostic (1.26e-2)
unprognostic (5.40e-2)	unprognostic (3.10e-1)	unprognostic (1.83e-1)	unprognostic (2.21e-1)	unprognostic (7.40e-2)	unprognostic (4.41e-2)
unprognostic (4.53e-1)	unprognostic (2.86e-1)	unprognostic (3.81e-1)	unprognostic (2.23e-1)	unprognostic (1.15e-1)	unprognostic (2.78e-3)
unprognostic (2.77e-1)	unprognostic (1.99e-2)	unprognostic (1.53e-1)	unprognostic (4.12e-2)	unprognostic (4.87e-3)	unprognostic (4.63e-2)
potential prognostic unfavorable (3.73e-4)	unprognostic (1.75e-1)	unprognostic (1.07e-1)	unprognostic (1.66e-2)	unprognostic (3.49e-1)	unprognostic (8.00e-2)
unprognostic (1.36e-1)	unprognostic (3.40e-1)	unprognostic (2.78e-1)	unprognostic (5.71e-2)	unprognostic (2.00e-2)	unprognostic (4.13e-3)
unprognostic (8.50e-2)	unprognostic (1.17e-1)	unprognostic (4.22e-1)	unprognostic (5.63e-2)	unprognostic (1.57e-1)	unprognostic (2.80e-2)
unprognostic (7.50e-3)	unprognostic (3.62e-2)	unprognostic (3.01e-2)	unprognostic (1.16e-1)	unprognostic (7.53e-2)	potential prognostic unfavorable (1.80e-4)
unprognostic (3.99e-2)	unprognostic (1.36e-1)	unprognostic (1.23e-2)	unprognostic (6.15e-2)	unprognostic (5.48e-2)	unprognostic (2.77e-1)
unprognostic (4.68e-1)	unprognostic (5.90e-2)	unprognostic (1.55e-1)			unprognostic (1.04e-1)
unprognostic (9.48e-2)	unprognostic (3.91e-1)	unprognostic (9.50e-2)	unprognostic (5.57e-2)	unprognostic (8.45e-2)	unprognostic (1.66e-1)
unprognostic (2.36e-2)	unprognostic (2.99e-3)	potential prognostic unfavorable (3.26e-4)	unprognostic (4.67e-2)	unprognostic (1.67e-1)	unprognostic (9.43e-2)
unprognostic (2.61e-1)	unprognostic (5.52e-2)	unprognostic (2.62e-1)	unprognostic (2.74e-1)	unprognostic (5.83e-3)	unprognostic (1.16e-2)
unprognostic (6.89e-2)	unprognostic (6.82e-2)	unprognostic (1.79e-1)	unprognostic (8.32e-3)	unprognostic (2.31e-1)	unprognostic (1.92e-1)
unprognostic (1.30e-2)	unprognostic (1.28e-1)	unprognostic (2.10e-2)	unprognostic (4.01e-3)	unprognostic (7.80e-2)	potential prognostic unfavorable (1.08e-5)
unprognostic (5.69e-2)	unprognostic (1.87e-1)	unprognostic (1.25e-1)	unprognostic (7.20e-2)	unprognostic (2.17e-1)	unprognostic (1.45e-2)
unprognostic (8.18e-3)	potential prognostic unfavorable (9.31e-5)	unprognostic (3.58e-2)	unprognostic (3.57e-2)	unprognostic (2.72e-1)	validated prognostic unfavorable (1.33e-4)
unprognostic (1.31e-2)	unprognostic (4.74e-2)	unprognostic (2.19e-1)	unprognostic (1.84e-1)	unprognostic (1.98e-1)	unprognostic (7.07e-2)
validated prognostic unfavorable (7.17e-4)	potential prognostic unfavorable (2.85e-4)	unprognostic (2.00e-2)	unprognostic (1.71e-2)	potential prognostic unfavorable (7.34e-4)	unprognostic (1.55e-3)
unprognostic (2.15e-2)	unprognostic (2.83e-1)	unprognostic (1.03e-1)	unprognostic (5.88e-2)	unprognostic (4.43e-1)	unprognostic (1.60e-1)
potential prognostic unfavorable (1.51e-4)	unprognostic (6.12e-2)	potential prognostic unfavorable (8.56e-4)	unprognostic (3.74e-1)	unprognostic (2.11e-1)	unprognostic (4.34e-2)
unprognostic (2.89e-1)	unprognostic (3.27e-1)	unprognostic (1.34e-1)	unprognostic (8.15e-2)	unprognostic (1.49e-2)	unprognostic (1.62e-2)
unprognostic (7.80e-3)	unprognostic (8.97e-2)	unprognostic (1.61e-1)	unprognostic (4.69e-2)	unprognostic (7.79e-3)	unprognostic (7.66e-2)
unprognostic (2.22e-1)					unprognostic (1.15e-1)
unprognostic (2.46e-3)	unprognostic (1.12e-2)	unprognostic (2.88e-1)	unprognostic (1.72e-2)	unprognostic (1.47e-1)	unprognostic (4.92e-2)
unprognostic (8.17e-2)	unprognostic (1.02e-1)	unprognostic (2.19e-1)	unprognostic (3.94e-1)	unprognostic (6.06e-2)	unprognostic (8.68e-2)
unprognostic (1.72e-1)	unprognostic (2.02e-1)	unprognostic (2.60e-1)	unprognostic (4.75e-3)	unprognostic (1.29e-1)	unprognostic (2.49e-3)
unprognostic (5.63e-2)	unprognostic (1.97e-1)	unprognostic (5.16e-2)	unprognostic (9.73e-2)	unprognostic (4.50e-2)	unprognostic (5.68e-3)
unprognostic (6.39e-3)	unprognostic (1.55e-1)	unprognostic (6.33e-2)	unprognostic (4.57e-1)	unprognostic (9.52e-2)	unprognostic (3.01e-1)
unprognostic (5.33e-3)	unprognostic (1.74e-1)	unprognostic (2.69e-1)	unprognostic (2.02e-1)	unprognostic (2.24e-2)	unprognostic (3.75e-3)
unprognostic (2.12e-1)	unprognostic (2.43e-1)	unprognostic (2.60e-2)	unprognostic (1.53e-1)		unprognostic (1.55e-2)
unprognostic (3.30e-2)	unprognostic (5.58e-2)	unprognostic (2.25e-2)	unprognostic (1.55e-1)	unprognostic (1.76e-1)	unprognostic (3.96e-2)
					unprognostic (1.38e-3)
unprognostic (2.58e-1)	unprognostic (2.76e-2)	unprognostic (2.08e-2)			unprognostic (8.90e-3)
unprognostic (4.10e-2)	unprognostic (1.28e-2)	unprognostic (3.02e-2)	unprognostic (9.67e-2)	unprognostic (3.70e-1)	potential prognostic unfavorable (9.06e-5)
unprognostic (3.22e-2)	unprognostic (2.05e-3)	unprognostic (4.64e-1)	unprognostic (6.94e-2)	unprognostic (1.78e-1)	unprognostic (6.88e-2)
			potential prognostic unfavorable (3.45e-4)	unprognostic (1.59e-1)	unprognostic (5.57e-2)
unprognostic (1.26e-1)	unprognostic (3.12e-2)	unprognostic (1.16e-1)	unprognostic (1.51e-1)	unprognostic (1.92e-1)	unprognostic (4.80e-2)
			potential prognostic unfavorable (1.39e-4)	unprognostic (1.99e-2)	
	unprognostic (4.43e-2)	unprognostic (5.55e-2)			
unprognostic (2.78e-2)	unprognostic (1.58e-1)	unprognostic (3.13e-1)	unprognostic (8.60e-2)	unprognostic (6.14e-2)	potential prognostic unfavorable (3.67e-4)
			unprognostic (1.07e-2)		
unprognostic (1.04e-2)	unprognostic (3.27e-2)	unprognostic (1.38e-1)			unprognostic (2.74e-1)
unprognostic (7.82e-2)	unprognostic (1.01e-1)	unprognostic (4.58e-3)	unprognostic (3.82e-1)	unprognostic (3.57e-1)	unprognostic (2.88e-2)
unprognostic (3.03e-1)	unprognostic (1.30e-2)	unprognostic (8.15e-2)	unprognostic (2.55e-2)	unprognostic (1.96e-1)	potential prognostic unfavorable (7.47e-5)
unprognostic (2.23e-1)	unprognostic (4.23e-2)	unprognostic (2.57e-2)	unprognostic (4.22e-2)	unprognostic (9.51e-3)	unprognostic (9.90e-2)
unprognostic (1.98e-1)	unprognostic (1.16e-3)	unprognostic (1.83e-1)	unprognostic (1.87e-1)	unprognostic (6.44e-2)	unprognostic (2.94e-2)
unprognostic (3.81e-1)		unprognostic (8.78e-2)		unprognostic (3.53e-1)	unprognostic (2.04e-1)
unprognostic (4.82e-3)	unprognostic (1.32e-1)	unprognostic (1.91e-1)	unprognostic (4.65e-3)	unprognostic (1.41e-1)	unprognostic (2.33e-1)
unprognostic (8.46e-3)	unprognostic (1.51e-1)	unprognostic (3.88e-1)	unprognostic (1.19e-1)	unprognostic (1.99e-1)	unprognostic (1.54e-1)
unprognostic (5.39e-2)	unprognostic (1.58e-1)	unprognostic (1.39e-1)	unprognostic (6.42e-2)	potential prognostic favorable (4.17e-4)	unprognostic (2.93e-2)
unprognostic (4.78e-2)	unprognostic (7.32e-2)	unprognostic (6.52e-2)	unprognostic (3.92e-2)	unprognostic (3.82e-1)	unprognostic (2.93e-2)
unprognostic (1.31e-3)	unprognostic (1.38e-2)	unprognostic (2.17e-1)			unprognostic (9.72e-2)
unprognostic (3.44e-2)	unprognostic (7.19e-2)	unprognostic (2.97e-1)	unprognostic (3.18e-1)	unprognostic (1.71e-1)	unprognostic (2.74e-2)
unprognostic (2.10e-1)	unprognostic (4.85e-2)	unprognostic (4.63e-1)	unprognostic (1.54e-2)	unprognostic (2.57e-2)	unprognostic (2.16e-1)
unprognostic (3.58e-2)	unprognostic (1.27e-1)	potential prognostic favorable (5.29e-4)	unprognostic (2.07e-1)	unprognostic (9.24e-2)	unprognostic (1.34e-1)
unprognostic (1.23e-1)	unprognostic (6.87e-2)	unprognostic (4.16e-1)	unprognostic (2.91e-3)	unprognostic (4.80e-3)	unprognostic (1.03e-1)
unprognostic (3.27e-1)	unprognostic (5.23e-2)	unprognostic (9.56e-2)	unprognostic (1.89e-1)	unprognostic (1.77e-1)	unprognostic (5.15e-2)
unprognostic (1.73e-1)	unprognostic (2.23e-1)	unprognostic (3.15e-1)	potential prognostic favorable (4.89e-4)	unprognostic (2.48e-2)	
unprognostic (1.68e-2)	unprognostic (1.35e-1)	unprognostic (1.08e-1)	unprognostic (8.39e-3)	unprognostic (1.17e-1)	unprognostic (1.15e-1)
potential prognostic unfavorable (9.24e-4)	unprognostic (1.06e-1)	unprognostic (5.37e-2)	unprognostic (8.35e-2)	potential prognostic unfavorable (1.34e-4)	unprognostic (1.08e-2)
unprognostic (1.90e-2)	unprognostic (2.41e-2)	unprognostic (4.01e-1)	unprognostic (4.34e-3)	unprognostic (2.26e-1)	unprognostic (1.77e-1)
			unprognostic (3.30e-1)	unprognostic (4.84e-2)	unprognostic (6.38e-2)
unprognostic (7.38e-3)	unprognostic (1.92e-1)	unprognostic (2.39e-1)	unprognostic (5.11e-3)	unprognostic (7.56e-2)	unprognostic (6.36e-3)
unprognostic (1.16e-1)	unprognostic (1.94e-1)	unprognostic (2.23e-1)	unprognostic (1.79e-1)	unprognostic (1.82e-1)	unprognostic (4.19e-2)
unprognostic (1.12e-1)	unprognostic (1.39e-1)	unprognostic (1.75e-2)		unprognostic (1.83e-1)	
unprognostic (1.49e-2)	unprognostic (1.73e-1)	unprognostic (3.22e-1)	unprognostic (8.22e-2)	unprognostic (9.27e-2)	unprognostic (1.69e-2)
unprognostic (3.07e-2)	unprognostic (8.56e-2)	unprognostic (4.14e-2)	unprognostic (3.14e-1)	unprognostic (1.82e-1)	unprognostic (1.13e-1)
unprognostic (2.27e-1)	unprognostic (1.84e-1)	unprognostic (2.18e-1)	unprognostic (1.10e-2)	unprognostic (1.82e-1)	potential prognostic favorable (2.76e-5)
unprognostic (2.37e-2)	unprognostic (1.35e-1)	unprognostic (1.22e-2)	unprognostic (1.98e-1)	unprognostic (7.04e-3)	unprognostic (2.14e-1)
unprognostic (2.90e-1)	unprognostic (1.80e-1)	unprognostic (5.98e-2)	unprognostic (2.14e-2)	unprognostic (3.64e-1)	unprognostic (2.19e-2)
potential prognostic unfavorable (1.17e-4)	unprognostic (2.52e-1)	unprognostic (3.18e-1)	unprognostic (1.06e-1)	unprognostic (3.21e-1)	unprognostic (1.55e-2)
unprognostic (1.06e-1)	unprognostic (1.67e-1)	unprognostic (1.17e-1)	unprognostic (2.32e-1)	unprognostic (3.44e-1)	unprognostic (7.60e-2)
unprognostic (3.60e-1)	unprognostic (1.47e-1)	unprognostic (3.04e-1)	unprognostic (1.31e-1)	unprognostic (7.08e-2)	unprognostic (6.75e-3)
					unprognostic (2.30e-2)
unprognostic (3.11e-3)	unprognostic (1.91e-1)	unprognostic (5.11e-2)	unprognostic (1.41e-2)		unprognostic (3.61e-1)
unprognostic (3.46e-3)	unprognostic (2.68e-2)	unprognostic (8.02e-2)	unprognostic (2.19e-1)	unprognostic (4.07e-2)	unprognostic (4.25e-3)
unprognostic (2.44e-1)	unprognostic (7.89e-2)	unprognostic (2.88e-1)	unprognostic (2.50e-2)	unprognostic (1.16e-1)	unprognostic (1.62e-1)
potential prognostic unfavorable (4.77e-5)	unprognostic (3.52e-1)	unprognostic (1.91e-2)	unprognostic (2.55e-1)	unprognostic (1.74e-1)	unprognostic (1.73e-1)
unprognostic (1.27e-1)	potential prognostic unfavorable (2.08e-5)	unprognostic (3.08e-2)			unprognostic (1.76e-2)

unprognostic (8.75e-2)	unprognostic (1.59e-1)	unprognostic (1.46e-1)	unprognostic (3.87e-1)	unprognostic (1.43e-1)	potential prognostic favorable (4.11e-4)
unprognostic (3.13e-2)	unprognostic (2.24e-1)	unprognostic (4.65e-2)	unprognostic (2.73e-1)	unprognostic (2.10e-1)	unprognostic (5.12e-2)
		unprognostic (2.29e-1)			
	unprognostic (1.35e-3)	unprognostic (4.93e-2)			
unprognostic (3.75e-2)	unprognostic (1.59e-2)	unprognostic (2.33e-2)	unprognostic (1.12e-1)	unprognostic (1.14e-1)	unprognostic (1.42e-1)
unprognostic (4.85e-2)	unprognostic (8.22e-2)	unprognostic (3.48e-2)	unprognostic (2.72e-1)	unprognostic (8.98e-2)	unprognostic (2.55e-1)
unprognostic (3.06e-1)	unprognostic (3.93e-2)	unprognostic (4.47e-2)	unprognostic (1.14e-1)	unprognostic (3.65e-2)	unprognostic (3.45e-1)
unprognostic (5.56e-2)	unprognostic (1.20e-1)	unprognostic (2.55e-1)	unprognostic (2.07e-1)	unprognostic (1.63e-1)	unprognostic (3.30e-3)
unprognostic (1.63e-1)	unprognostic (1.21e-1)	unprognostic (2.52e-1)	unprognostic (6.29e-2)	unprognostic (3.41e-1)	unprognostic (1.58e-1)
unprognostic (7.58e-2)	unprognostic (1.37e-3)	unprognostic (1.42e-1)	unprognostic (1.07e-2)	unprognostic (8.40e-2)	unprognostic (2.15e-3)
unprognostic (7.30e-2)	unprognostic (5.03e-2)	unprognostic (1.29e-1)	unprognostic (1.40e-1)	unprognostic (1.05e-1)	unprognostic (5.94e-2)
unprognostic (1.31e-1)	potential prognostic unfavorable (2.61e-4)	unprognostic (1.59e-1)	unprognostic (1.64e-1)	unprognostic (9.93e-2)	unprognostic (6.95e-3)
unprognostic (1.06e-1)	unprognostic (3.56e-2)	unprognostic (3.42e-1)			
unprognostic (2.64e-2)	unprognostic (1.96e-1)	unprognostic (1.93e-1)	unprognostic (2.18e-3)	unprognostic (3.47e-2)	unprognostic (7.24e-3)
unprognostic (1.63e-2)	unprognostic (6.67e-2)	unprognostic (2.78e-1)	unprognostic (1.41e-1)	unprognostic (3.31e-2)	unprognostic (9.31e-2)
unprognostic (3.29e-3)	unprognostic (7.58e-3)	unprognostic (3.31e-2)	unprognostic (4.13e-1)	unprognostic (7.45e-2)	unprognostic (1.62e-2)
unprognostic (3.76e-1)	unprognostic (2.68e-1)	unprognostic (3.29e-3)	unprognostic (3.15e-1)	unprognostic (1.62e-3)	unprognostic (5.02e-2)
unprognostic (3.63e-1)	unprognostic (2.80e-2)	unprognostic (9.05e-2)	unprognostic (7.16e-2)	unprognostic (1.13e-2)	unprognostic (2.78e-1)
unprognostic (3.75e-2)	unprognostic (1.21e-1)	unprognostic (1.31e-1)		unprognostic (6.53e-2)	unprognostic (8.62e-2)
unprognostic (2.51e-2)	unprognostic (4.61e-2)	unprognostic (2.41e-1)	unprognostic (1.59e-2)	unprognostic (2.73e-2)	unprognostic (1.24e-3)
unprognostic (4.99e-3)	unprognostic (7.58e-3)	unprognostic (1.43e-2)	unprognostic (2.13e-1)	unprognostic (5.60e-2)	unprognostic (1.46e-2)
unprognostic (5.52e-2)	unprognostic (1.96e-1)	unprognostic (1.74e-1)	unprognostic (1.89e-1)	unprognostic (5.71e-2)	unprognostic (1.37e-2)
unprognostic (2.24e-1)	unprognostic (5.09e-3)	unprognostic (9.30e-2)	unprognostic (1.85e-1)	unprognostic (1.04e-1)	potential prognostic unfavorable (4.90e-4)
unprognostic (2.08e-1)	unprognostic (4.69e-2)	unprognostic (1.33e-1)	unprognostic (9.29e-2)	unprognostic (6.13e-3)	unprognostic (1.92e-1)
unprognostic (2.01e-1)	unprognostic (2.62e-3)	unprognostic (3.78e-2)	unprognostic (1.07e-2)	unprognostic (8.72e-3)	unprognostic (6.68e-2)
unprognostic (1.03e-3)	unprognostic (3.87e-2)	unprognostic (6.84e-2)	unprognostic (3.68e-1)	unprognostic (1.57e-1)	unprognostic (1.36e-1)
potential prognostic unfavorable (9.87e-4)	unprognostic (5.17e-3)	unprognostic (1.04e-2)	unprognostic (6.00e-2)	unprognostic (6.61e-2)	unprognostic (2.51e-1)
unprognostic (1.93e-1)	unprognostic (5.11e-3)	unprognostic (9.66e-2)	unprognostic (2.99e-2)	unprognostic (2.60e-1)	unprognostic (8.04e-2)
unprognostic (8.57e-2)	unprognostic (8.25e-3)	unprognostic (2.75e-1)	unprognostic (8.48e-2)	unprognostic (4.98e-2)	unprognostic (2.82e-2)
unprognostic (4.18e-2)	unprognostic (2.64e-2)	unprognostic (2.76e-3)	unprognostic (6.93e-3)	unprognostic (4.41e-3)	potential prognostic unfavorable (4.95e-4)
unprognostic (3.71e-1)		unprognostic (3.67e-2)			unprognostic (3.12e-1)
unprognostic (1.12e-2)	unprognostic (5.53e-2)	unprognostic (2.40e-2)			potential prognostic favorable (2.99e-4)
potential prognostic favorable (8.49e-4)	unprognostic (1.33e-1)	unprognostic (7.99e-2)	unprognostic (5.76e-2)	unprognostic (1.34e-1)	unprognostic (2.54e-3)
unprognostic (2.09e-2)	unprognostic (7.56e-2)	unprognostic (4.00e-2)	unprognostic (3.93e-2)	unprognostic (8.16e-2)	potential prognostic favorable (1.08e-4)
unprognostic (3.59e-2)	unprognostic (6.73e-3)	unprognostic (1.15e-1)			
unprognostic (1.70e-1)	unprognostic (8.60e-2)	unprognostic (2.15e-1)	unprognostic (2.74e-1)	unprognostic (2.57e-1)	unprognostic (1.13e-1)
unprognostic (5.67e-3)	unprognostic (2.51e-2)	unprognostic (7.46e-2)	unprognostic (3.28e-3)	unprognostic (1.72e-1)	unprognostic (2.21e-1)
unprognostic (1.35e-3)	unprognostic (1.44e-1)	unprognostic (2.56e-1)	unprognostic (1.92e-1)	unprognostic (2.21e-2)	unprognostic (1.17e-2)
unprognostic (1.34e-1)	unprognostic (1.28e-1)	unprognostic (1.88e-3)	unprognostic (5.96e-2)	unprognostic (1.85e-1)	unprognostic (1.04e-1)
				unprognostic (3.09e-2)	
unprognostic (4.16e-2)	unprognostic (1.35e-1)	unprognostic (1.37e-2)	unprognostic (1.65e-2)	unprognostic (7.41e-2)	unprognostic (1.17e-2)
unprognostic (7.44e-2)	unprognostic (1.16e-1)	unprognostic (1.47e-2)	unprognostic (1.18e-2)	unprognostic (2.69e-1)	unprognostic (1.91e-2)
unprognostic (1.95e-1)	unprognostic (1.38e-2)	unprognostic (2.02e-1)	unprognostic (2.15e-2)	unprognostic (1.36e-1)	unprognostic (3.37e-2)
unprognostic (5.51e-2)	unprognostic (2.44e-3)	unprognostic (1.48e-2)	unprognostic (4.65e-2)		unprognostic (1.74e-3)
potential prognostic unfavorable (8.43e-4)	unprognostic (5.40e-3)	unprognostic (6.67e-2)	unprognostic (8.11e-3)	unprognostic (1.28e-1)	unprognostic (2.55e-3)
potential prognostic unfavorable (2.36e-4)	unprognostic (5.80e-2)	unprognostic (1.14e-1)	unprognostic (5.49e-2)	unprognostic (1.30e-2)	unprognostic (4.61e-2)
unprognostic (4.49e-3)	unprognostic (1.15e-1)	potential prognostic favorable (3.52e-4)	unprognostic (3.71e-1)	unprognostic (1.23e-1)	unprognostic (1.42e-2)
unprognostic (2.90e-1)	unprognostic (4.87e-2)	potential prognostic unfavorable (8.50e-4)	unprognostic (6.01e-2)	unprognostic (3.07e-2)	unprognostic (9.06e-2)
					unprognostic (3.92e-2)
unprognostic (6.84e-3)	unprognostic (5.58e-2)				
unprognostic (1.69e-3)	unprognostic (4.58e-1)	unprognostic (1.45e-1)	unprognostic (6.54e-3)	unprognostic (1.35e-2)	unprognostic (1.01e-3)
potential prognostic unfavorable (1.06e-4)	unprognostic (2.28e-1)	unprognostic (6.40e-2)	unprognostic (3.03e-1)	unprognostic (3.36e-1)	unprognostic (1.94e-1)
unprognostic (4.63e-3)	unprognostic (1.46e-1)	unprognostic (1.50e-1)	unprognostic (1.36e-3)	unprognostic (2.96e-2)	unprognostic (1.76e-3)
unprognostic (1.03e-2)	unprognostic (2.46e-3)	unprognostic (2.28e-2)	unprognostic (4.88e-2)	unprognostic (1.74e-2)	potential prognostic unfavorable (1.51e-7)
					unprognostic (5.02e-2)
unprognostic (3.52e-2)	unprognostic (1.51e-2)	unprognostic (1.90e-2)	unprognostic (2.15e-1)		unprognostic (5.05e-2)
					unprognostic (8.27e-3)
unprognostic (6.15e-2)	unprognostic (1.05e-2)	unprognostic (7.78e-2)	unprognostic (1.09e-3)	unprognostic (1.38e-3)	potential prognostic unfavorable (1.06e-4)
unprognostic (3.45e-2)	unprognostic (1.12e-1)	unprognostic (4.59e-1)	unprognostic (2.28e-1)	unprognostic (3.07e-2)	unprognostic (1.90e-1)
unprognostic (1.45e-1)	unprognostic (2.48e-2)	unprognostic (3.01e-2)	unprognostic (8.39e-2)	unprognostic (9.67e-2)	unprognostic (1.37e-1)
unprognostic (3.22e-1)	unprognostic (6.21e-3)	unprognostic (1.55e-1)	unprognostic (6.87e-2)		unprognostic (5.05e-2)
unprognostic (8.27e-2)	unprognostic (1.26e-2)	unprognostic (1.02e-1)	unprognostic (4.49e-2)	unprognostic (2.66e-1)	unprognostic (1.38e-2)
unprognostic (1.57e-1)	unprognostic (2.98e-2)	unprognostic (6.93e-2)	unprognostic (2.19e-1)	unprognostic (5.79e-2)	unprognostic (2.01e-2)
unprognostic (1.14e-1)	unprognostic (1.30e-1)	unprognostic (1.36e-1)			unprognostic (2.21e-3)
unprognostic (1.98e-1)	unprognostic (4.44e-1)	unprognostic (1.26e-1)	unprognostic (9.92e-2)	unprognostic (2.52e-1)	unprognostic (4.31e-2)
	unprognostic (2.31e-1)	unprognostic (5.01e-1)	unprognostic (1.78e-1)	unprognostic (4.88e-1)	
unprognostic (1.25e-1)	unprognostic (8.54e-2)	unprognostic (2.17e-2)	unprognostic (5.80e-2)	unprognostic (1.92e-1)	unprognostic (2.20e-2)
unprognostic (2.42e-1)	unprognostic (2.09e-2)	unprognostic (5.25e-1)	unprognostic (3.01e-1)	unprognostic (1.07e-1)	unprognostic (2.59e-1)
unprognostic (1.06e-2)	unprognostic (4.47e-1)	unprognostic (2.76e-1)	unprognostic (1.24e-1)	unprognostic (3.16e-2)	potential prognostic unfavorable (6.01e-4)
unprognostic (2.34e-3)	unprognostic (6.30e-3)	unprognostic (4.04e-3)	unprognostic (3.25e-2)	unprognostic (8.18e-2)	unprognostic (5.66e-3)
unprognostic (1.15e-1)	unprognostic (1.09e-2)	unprognostic (1.70e-1)	unprognostic (3.44e-2)	unprognostic (2.39e-1)	unprognostic (2.07e-1)
unprognostic (1.06e-1)	unprognostic (2.93e-1)	unprognostic (5.87e-2)	unprognostic (1.82e-2)	unprognostic (1.72e-1)	unprognostic (1.24e-3)
unprognostic (3.28e-2)	unprognostic (7.13e-2)	unprognostic (3.84e-3)	unprognostic (2.77e-3)		unprognostic (2.94e-2)
unprognostic (2.04e-1)	unprognostic (1.74e-1)	unprognostic (1.98e-1)	unprognostic (5.46e-2)	potential prognostic unfavorable (9.08e-5)	unprognostic (7.09e-3)
unprognostic (1.66e-3)	unprognostic (1.66e-2)	unprognostic (1.32e-1)	unprognostic (3.97e-2)	unprognostic (8.29e-3)	unprognostic (2.64e-3)
unprognostic (7.87e-3)	unprognostic (3.66e-1)	unprognostic (7.46e-2)	unprognostic (1.08e-1)	unprognostic (3.66e-1)	unprognostic (2.64e-1)
unprognostic (4.14e-2)	unprognostic (1.90e-2)	unprognostic (1.07e-1)	unprognostic (2.26e-2)	unprognostic (1.73e-1)	unprognostic (3.78e-1)
unprognostic (1.45e-3)	unprognostic (1.32e-1)	unprognostic (3.28e-2)	potential prognostic unfavorable (1.22e-4)	unprognostic (2.65e-2)	unprognostic (1.41e-3)
unprognostic (2.95e-1)	unprognostic (6.67e-2)	unprognostic (2.54e-1)	unprognostic (2.06e-1)	unprognostic (3.11e-2)	unprognostic (2.42e-1)
unprognostic (9.85e-2)	unprognostic (5.59e-2)	unprognostic (1.64e-1)	unprognostic (8.89e-2)	unprognostic (2.27e-1)	potential prognostic favorable (7.34e-5)
unprognostic (6.73e-2)	unprognostic (1.48e-1)	unprognostic (1.17e-2)	unprognostic (1.56e-1)	unprognostic (2.97e-2)	unprognostic (6.05e-3)
unprognostic (5.82e-3)	unprognostic (1.02e-1)	unprognostic (2.15e-1)	unprognostic (1.18e-1)	unprognostic (1.11e-1)	potential prognostic unfavorable (3.89e-4)
unprognostic (8.77e-2)	potential prognostic unfavorable (2.51e-4)	unprognostic (3.98e-3)	unprognostic (2.09e-2)	unprognostic (3.02e-2)	potential prognostic unfavorable (4.25e-4)
unprognostic (1.57e-2)	unprognostic (2.89e-2)	unprognostic (1.49e-2)	unprognostic (9.49e-3)	unprognostic (1.07e-1)	unprognostic (1.41e-2)
unprognostic (7.87e-2)			unprognostic (2.15e-1)	unprognostic (7.16e-3)	unprognostic (3.28e-3)
unprognostic (3.56e-1)	unprognostic (2.08e-1)	unprognostic (1.61e-1)	unprognostic (1.10e-1)		unprognostic (7.15e-2)
unprognostic (4.63e-3)	unprognostic (1.15e-3)	unprognostic (4.96e-2)	unprognostic (8.35e-2)	unprognostic (2.34e-2)	unprognostic (2.70e-1)
unprognostic (1.93e-2)	unprognostic (3.51e-2)	unprognostic (8.93e-2)	potential prognostic unfavorable (8.66e-4)	unprognostic (3.94e-2)	unprognostic (8.68e-3)
unprognostic (2.94e-2)	unprognostic (1.38e-1)	unprognostic (6.87e-2)	unprognostic (1.27e-1)	unprognostic (2.81e-1)	unprognostic (6.46e-2)
unprognostic (8.61e-2)	unprognostic (3.25e-1)	unprognostic (3.05e-2)	unprognostic (2.24e-1)	unprognostic (1.10e-3)	unprognostic (1.62e-2)
unprognostic (6.17e-2)	unprognostic (6.40e-3)	unprognostic (9.99e-2)	unprognostic (1.69e-2)	unprognostic (3.40e-3)	potential prognostic unfavorable (3.94e-4)
unprognostic (2.27e-1)	unprognostic (1.51e-2)	unprognostic (1.12e-1)	unprognostic (1.17e-2)	unprognostic (3.70e-3)	unprognostic (9.86e-2)
unprognostic (4.54e-2)	unprognostic (6.44e-2)	unprognostic (1.29e-1)	unprognostic (5.75e-2)	unprognostic (2.17e-2)	unprognostic (1.07e-2)
unprognostic (3.61e-2)	unprognostic (1.28e-2)	unprognostic (3.54e-1)	unprognostic (8.11e-2)	unprognostic (1.16e-2)	unprognostic (3.25e-1)
unprognostic (6.36e-2)	unprognostic (5.17e-3)	unprognostic (1.02e-1)	unprognostic (3.52e-2)		unprognostic (2.20e-1)
unprognostic (1.38e-1)	unprognostic (8.40e-2)	unprognostic (3.05e-2)			
unprognostic (1.27e-1)	unprognostic (1.13e-1)	unprognostic (5.77e-1)	unprognostic (9.49e-3)	unprognostic (2.77e-3)	unprognostic (1.33e-1)
unprognostic (2.78e-1)	unprognostic (8.81e-2)	unprognostic (1.36e-1)	unprognostic (2.37e-1)	unprognostic (2.40e-3)	unprognostic (2.39e-2)
unprognostic (4.14e-2)	unprognostic (7.30e-2)	unprognostic (1.51e-1)	unprognostic (1.06e-2)		unprognostic (4.51e-2)
unprognostic (1.36e-1)	unprognostic (1.40e-1)	unprognostic (3.17e-1)	unprognostic (9.35e-2)	unprognostic (2.11e-1)	unprognostic (1.34e-1)
unprognostic (1.07e-2)	unprognostic (1.15e-1)	unprognostic (1.69e-1)	unprognostic (1.13e-1)	unprognostic (1.56e-2)	unprognostic (2.18e-1)
unprognostic (2.81e-2)	unprognostic (2.03e-1)	unprognostic (2.06e-1)	unprognostic (2.02e-1)	unprognostic (1.89e-1)	unprognostic (7.85e-3)
unprognostic (5.78e-3)	potential prognostic unfavorable (9.74e-4)	unprognostic (1.96e-1)	unprognostic (2.24e-3)	unprognostic (4.00e-1)	unprognostic (1.19e-3)
unprognostic (1.32e-1)	unprognostic (1.54e-1)	unprognostic (1.78e-2)	unprognostic (2.18e-1)	unprognostic (1.22e-1)	unprognostic (5.53e-3)
unprognostic (3.68e-1)	unprognostic (2.31e-1)	unprognostic (4.41e-3)	unprognostic (1.62e-1)	unprognostic (9.42e-3)	unprognostic (1.53e-3)
unprognostic (2.59e-2)	unprognostic (4.40e-1)	unprognostic (1.02e-1)	unprognostic (1.27e-2)	unprognostic (2.92e-2)	unprognostic (2.60e-2)
unprognostic (3.24e-3)	unprognostic (2.28e-2)	unprognostic (7.06e-2)	unprognostic (2.28e-3)	unprognostic (1.57e-1)	unprognostic (2.37e-3)
unprognostic (3.13e-2)	unprognostic (8.98e-2)	potential prognostic favorable (9.82e-4)	unprognostic (2.83e-1)	unprognostic (2.43e-2)	unprognostic (3.54e-2)
unprognostic (2.14e-2)	unprognostic (1.05e-1)	unprognostic (1.61e-2)	unprognostic (1.01e-1)	unprognostic (2.99e-1)	unprognostic (1.43e-3)
unprognostic (3.61e-2)	unprognostic (8.82e-2)	unprognostic (2.42e-1)	unprognostic (2.31e-1)	unprognostic (2.18e-1)	unprognostic (2.81e-3)
unprognostic (8.58e-2)	unprognostic (4.12e-1)	unprognostic (1.11e-1)	unprognostic (3.17e-1)	unprognostic (1.60e-1)	unprognostic (5.88e-2)
unprognostic (6.72e-2)	unprognostic (2.03e-1)	unprognostic (1.87e-1)	unprognostic (2.90e-3)	unprognostic (9.90e-2)	unprognostic (1.05e-3)
			unprognostic (5.45e-2)	unprognostic (1.81e-1)	unprognostic (1.89e-2)
unprognostic (1.21e-1)	unprognostic (9.96e-3)	unprognostic (3.60e-3)	unprognostic (6.92e-2)	unprognostic (1.45e-2)	unprognostic (1.38e-2)
unprognostic (1.27e-2)	unprognostic (1.72e-1)	unprognostic (9.50e-2)	unprognostic (1.55e-1)	unprognostic (1.98e-2)	potential prognostic unfavorable (6.14e-4)
unprognostic (5.24e-3)	unprognostic (1.04e-1)	unprognostic (4.32e-2)	unprognostic (1.15e-1)	unprognostic (1.50e-1)	unprognostic (4.65e-3)

unprognostic (1.52e-2)	unprognostic (2.86e-2)	unprognostic (8.87e-2)	unprognostic (9.12e-2)	unprognostic (1.73e-1)	potential prognostic unfavorable (2.77e-4)
unprognostic (1.41e-3)	unprognostic (1.46e-1)	unprognostic (3.07e-3)	unprognostic (2.71e-1)	unprognostic (1.73e-3)	unprognostic (4.77e-2)
unprognostic (2.24e-2)	unprognostic (8.92e-3)	unprognostic (3.65e-2)	unprognostic (2.49e-3)	unprognostic (4.77e-2)	unprognostic (2.22e-3)
unprognostic (1.78e-1)	unprognostic (2.49e-1)	unprognostic (1.13e-1)	unprognostic (2.78e-2)	unprognostic (7.52e-2)	unprognostic (2.19e-1)
unprognostic (1.30e-1)	unprognostic (2.53e-1)	unprognostic (2.92e-1)	unprognostic (2.58e-2)	unprognostic (1.81e-1)	unprognostic (7.38e-3)
unprognostic (3.97e-2)	unprognostic (1.43e-1)	unprognostic (5.47e-2)	unprognostic (7.72e-2)	unprognostic (4.43e-2)	potential prognostic unfavorable (6.20e-4)
potential prognostic unfavorable (1.06e-4)	unprognostic (8.80e-2)	unprognostic (2.39e-2)	unprognostic (5.90e-3)	unprognostic (5.79e-3)	unprognostic (2.12e-2)
unprognostic (2.07e-1)	unprognostic (2.42e-2)	unprognostic (2.15e-2)	unprognostic (4.45e-3)	unprognostic (2.65e-2)	
unprognostic (7.80e-2)	unprognostic (3.38e-2)	unprognostic (3.81e-2)	unprognostic (8.91e-2)	unprognostic (4.01e-1)	unprognostic (1.77e-1)
unprognostic (2.04e-1)	unprognostic (2.23e-1)	unprognostic (2.72e-2)	unprognostic (1.14e-1)	unprognostic (1.73e-2)	unprognostic (3.44e-2)
unprognostic (3.32e-2)	unprognostic (1.42e-1)	unprognostic (2.86e-1)	unprognostic (3.61e-2)	unprognostic (2.03e-1)	unprognostic (9.68e-2)
unprognostic (1.94e-2)	unprognostic (2.12e-1)	unprognostic (2.93e-1)	unprognostic (4.10e-1)	unprognostic (4.13e-2)	unprognostic (2.05e-1)
	unprognostic (8.78e-2)		unprognostic (4.54e-2)	unprognostic (4.93e-2)	unprognostic (4.71e-2)
unprognostic (9.55e-2)	unprognostic (1.17e-1)	unprognostic (6.05e-2)	unprognostic (2.69e-1)	potential prognostic favorable (7.59e-4)	unprognostic (2.43e-2)
unprognostic (7.08e-2)	unprognostic (2.68e-2)	unprognostic (3.79e-2)	unprognostic (2.02e-1)	unprognostic (2.42e-1)	potential prognostic unfavorable (3.08e-5)
unprognostic (1.29e-1)	unprognostic (1.99e-2)	unprognostic (1.08e-1)	unprognostic (1.49e-1)	unprognostic (4.26e-2)	unprognostic (3.59e-2)
validated prognostic unfavorable (2.11e-5)	unprognostic (3.59e-1)	unprognostic (9.70e-2)	unprognostic (3.13e-1)	unprognostic (1.97e-2)	unprognostic (7.31e-2)
unprognostic (7.50e-2)	unprognostic (1.16e-1)	unprognostic (1.03e-1)	unprognostic (7.22e-2)	unprognostic (1.95e-2)	unprognostic (6.76e-2)
unprognostic (1.33e-2)	unprognostic (1.11e-1)	unprognostic (3.80e-2)	unprognostic (4.87e-2)	unprognostic (1.22e-2)	unprognostic (1.21e-1)
	unprognostic (2.81e-1)	unprognostic (1.62e-2)	unprognostic (7.37e-2)		potential prognostic favorable (3.67e-5)
unprognostic (5.46e-2)	unprognostic (2.25e-2)	unprognostic (2.42e-1)	unprognostic (2.97e-2)	unprognostic (3.60e-2)	unprognostic (8.41e-2)
unprognostic (1.02e-1)	unprognostic (1.70e-1)	unprognostic (8.71e-2)	unprognostic (5.89e-2)	unprognostic (2.65e-2)	unprognostic (1.53e-2)
unprognostic (1.77e-1)	unprognostic (1.12e-1)	unprognostic (8.48e-2)	unprognostic (1.01e-1)	unprognostic (1.69e-1)	unprognostic (2.23e-2)
unprognostic (2.77e-3)	unprognostic (2.44e-1)	unprognostic (2.08e-1)			unprognostic (6.79e-2)
unprognostic (1.94e-2)	unprognostic (1.54e-1)	unprognostic (1.51e-1)	unprognostic (1.04e-2)	unprognostic (2.07e-2)	unprognostic (1.19e-1)
unprognostic (1.68e-2)	unprognostic (9.56e-2)	unprognostic (5.09e-2)	unprognostic (7.76e-3)	unprognostic (1.15e-3)	potential prognostic unfavorable (3.10e-4)
unprognostic (1.17e-1)	unprognostic (4.61e-2)	unprognostic (1.58e-1)	unprognostic (8.29e-2)	unprognostic (9.36e-2)	unprognostic (4.17e-2)
			unprognostic (4.07e-2)	unprognostic (1.90e-2)	potential prognostic unfavorable (8.04e-4)
unprognostic (3.42e-2)	unprognostic (1.46e-1)	unprognostic (1.14e-3)	unprognostic (6.01e-2)	unprognostic (1.47e-1)	unprognostic (4.95e-3)
unprognostic (1.01e-1)	unprognostic (1.39e-2)	unprognostic (1.09e-1)	unprognostic (4.05e-2)	unprognostic (1.29e-1)	unprognostic (8.75e-3)
unprognostic (1.70e-2)	unprognostic (3.96e-1)	unprognostic (2.56e-1)	unprognostic (4.33e-2)	unprognostic (2.00e-2)	unprognostic (1.05e-1)
unprognostic (7.03e-2)	unprognostic (1.18e-1)	unprognostic (1.75e-2)	unprognostic (2.14e-1)	unprognostic (6.19e-2)	unprognostic (1.42e-1)
unprognostic (8.55e-2)	unprognostic (1.64e-1)	unprognostic (3.22e-1)	unprognostic (8.65e-2)	unprognostic (1.21e-2)	unprognostic (1.61e-1)
unprognostic (3.33e-1)	unprognostic (1.89e-1)	unprognostic (1.25e-1)	unprognostic (2.17e-2)	unprognostic (1.54e-1)	unprognostic (5.75e-3)
unprognostic (8.19e-2)	unprognostic (2.04e-1)	unprognostic (2.42e-3)	unprognostic (3.26e-1)	unprognostic (5.66e-2)	unprognostic (4.53e-3)
	unprognostic (3.56e-1)	unprognostic (1.80e-1)			
	unprognostic (5.99e-2)	unprognostic (7.33e-2)			unprognostic (8.73e-3)
unprognostic (9.58e-2)	unprognostic (2.15e-1)	unprognostic (1.22e-1)	unprognostic (4.47e-2)	unprognostic (2.75e-2)	unprognostic (5.12e-3)
unprognostic (1.06e-2)	unprognostic (1.26e-1)	unprognostic (5.18e-2)	unprognostic (1.13e-1)	unprognostic (7.36e-2)	unprognostic (2.18e-3)
unprognostic (4.38e-1)	unprognostic (1.09e-1)	unprognostic (7.73e-2)	unprognostic (2.53e-2)	unprognostic (1.34e-1)	unprognostic (1.30e-3)
unprognostic (1.82e-2)	unprognostic (1.82e-1)	unprognostic (2.41e-1)	unprognostic (2.93e-1)	unprognostic (1.17e-1)	unprognostic (3.10e-2)
unprognostic (1.15e-1)	unprognostic (9.04e-3)	unprognostic (1.56e-1)	unprognostic (5.30e-3)	unprognostic (1.25e-1)	unprognostic (9.57e-3)
unprognostic (2.17e-2)	unprognostic (2.19e-2)	unprognostic (4.49e-1)	unprognostic (7.50e-2)	unprognostic (2.20e-1)	potential prognostic unfavorable (4.45e-5)
unprognostic (5.68e-2)	unprognostic (1.47e-1)	unprognostic (2.19e-1)	unprognostic (3.91e-1)	unprognostic (5.46e-2)	unprognostic (2.59e-2)
unprognostic (4.44e-2)	unprognostic (4.80e-1)	unprognostic (1.70e-1)	unprognostic (1.08e-1)	unprognostic (1.21e-1)	unprognostic (2.73e-1)
unprognostic (3.81e-2)	unprognostic (9.56e-3)	unprognostic (3.06e-1)	unprognostic (2.88e-1)	unprognostic (8.57e-2)	unprognostic (7.79e-2)
unprognostic (1.39e-1)					
unprognostic (5.98e-2)	unprognostic (6.28e-2)	unprognostic (3.76e-2)	unprognostic (1.70e-1)	unprognostic (3.38e-1)	unprognostic (5.44e-2)
unprognostic (2.25e-2)	unprognostic (6.79e-2)	unprognostic (5.15e-3)	unprognostic (1.16e-2)	unprognostic (1.05e-2)	unprognostic (1.44e-2)
unprognostic (2.00e-3)	unprognostic (1.82e-1)	unprognostic (2.98e-1)	unprognostic (1.79e-1)	unprognostic (7.76e-2)	unprognostic (2.41e-3)
unprognostic (3.24e-1)	unprognostic (1.52e-2)	unprognostic (3.44e-1)	unprognostic (3.05e-3)	unprognostic (1.33e-1)	unprognostic (1.34e-1)
unprognostic (2.66e-1)	unprognostic (1.28e-1)	unprognostic (1.25e-2)	unprognostic (7.04e-2)	unprognostic (2.99e-1)	unprognostic (2.20e-1)
unprognostic (1.13e-1)	unprognostic (4.91e-2)	unprognostic (1.29e-1)	unprognostic (1.12e-2)	unprognostic (9.86e-2)	unprognostic (2.05e-2)
unprognostic (2.00e-2)	unprognostic (2.72e-2)	unprognostic (1.05e-1)	unprognostic (3.68e-2)	unprognostic (1.31e-2)	
unprognostic (2.80e-1)	unprognostic (5.62e-3)	unprognostic (1.86e-1)	unprognostic (1.92e-2)	unprognostic (1.16e-1)	unprognostic (2.25e-1)
unprognostic (1.57e-2)	unprognostic (1.32e-3)	unprognostic (1.79e-1)	unprognostic (2.27e-1)	unprognostic (2.79e-2)	unprognostic (1.13e-1)
unprognostic (9.00e-2)	unprognostic (2.65e-1)	unprognostic (7.30e-2)	unprognostic (7.60e-2)		unprognostic (1.74e-1)
unprognostic (1.53e-3)	unprognostic (8.79e-2)	unprognostic (2.55e-2)	unprognostic (1.25e-2)	unprognostic (1.12e-1)	unprognostic (8.62e-3)
unprognostic (7.43e-2)	unprognostic (2.53e-1)	unprognostic (3.71e-2)	unprognostic (1.43e-2)	unprognostic (7.97e-2)	unprognostic (4.46e-3)
unprognostic (2.38e-2)	unprognostic (4.23e-2)	unprognostic (9.66e-2)	unprognostic (1.65e-1)	unprognostic (4.02e-1)	unprognostic (1.27e-1)
unprognostic (3.71e-1)	unprognostic (9.35e-2)	unprognostic (2.48e-1)	unprognostic (2.00e-2)	unprognostic (4.47e-2)	unprognostic (1.89e-3)
unprognostic (1.59e-1)	unprognostic (9.87e-2)	unprognostic (2.94e-1)	unprognostic (1.36e-1)	unprognostic (1.79e-1)	unprognostic (8.14e-2)
unprognostic (9.49e-2)	unprognostic (8.98e-2)	unprognostic (4.42e-1)	unprognostic (1.53e-1)	unprognostic (1.21e-2)	unprognostic (3.15e-2)
unprognostic (2.93e-2)	unprognostic (4.94e-2)	unprognostic (6.36e-2)	unprognostic (4.97e-2)	unprognostic (1.02e-1)	unprognostic (2.09e-3)
unprognostic (1.71e-2)	unprognostic (3.35e-1)	unprognostic (1.83e-1)	unprognostic (5.74e-2)	unprognostic (2.76e-1)	unprognostic (1.51e-1)
unprognostic (1.88e-2)	unprognostic (9.25e-2)	unprognostic (3.46e-2)	unprognostic (1.89e-2)		
	unprognostic (2.37e-1)		unprognostic (1.45e-2)	unprognostic (1.73e-1)	unprognostic (9.35e-3)
	unprognostic (4.11e-2)		unprognostic (3.49e-2)		unprognostic (1.80e-3)
unprognostic (1.12e-1)	unprognostic (6.99e-2)	unprognostic (1.58e-2)	unprognostic (6.17e-2)	unprognostic (3.47e-2)	unprognostic (3.73e-3)
unprognostic (1.53e-2)	unprognostic (1.34e-1)	unprognostic (5.98e-2)	unprognostic (1.35e-1)	unprognostic (2.72e-1)	unprognostic (4.93e-3)
unprognostic (2.51e-1)	unprognostic (1.85e-1)	potential prognostic unfavorable (2.89e-4)	unprognostic (1.78e-1)	unprognostic (1.59e-1)	unprognostic (8.06e-3)
unprognostic (5.68e-2)	unprognostic (3.85e-2)	unprognostic (1.12e-1)	unprognostic (4.71e-2)	unprognostic (2.20e-1)	validated prognostic unfavorable (1.22e-4)
			unprognostic (4.31e-2)		unprognostic (1.94e-3)
unprognostic (3.78e-1)		potential prognostic favorable (3.92e-4)	unprognostic (1.68e-2)		
	unprognostic (1.39e-1)		unprognostic (6.73e-2)	unprognostic (6.71e-2)	potential prognostic favorable (5.55e-4)
unprognostic (2.06e-1)	unprognostic (2.32e-2)	unprognostic (1.99e-1)	unprognostic (1.85e-2)		unprognostic (1.65e-1)
unprognostic (1.08e-1)	unprognostic (3.26e-2)	unprognostic (6.80e-2)	unprognostic (2.92e-1)	unprognostic (9.97e-2)	unprognostic (7.49e-2)
unprognostic (2.12e-2)	unprognostic (2.38e-1)	unprognostic (4.06e-2)	unprognostic (1.52e-1)		unprognostic (2.17e-1)
unprognostic (7.45e-2)	unprognostic (1.43e-2)	unprognostic (6.05e-3)	unprognostic (8.86e-2)	unprognostic (2.07e-2)	unprognostic (9.98e-2)
unprognostic (1.47e-2)	unprognostic (9.23e-2)	unprognostic (7.38e-2)	unprognostic (1.73e-1)	unprognostic (3.91e-3)	unprognostic (1.47e-2)
unprognostic (3.13e-2)	unprognostic (2.13e-2)	unprognostic (1.35e-2)	unprognostic (2.82e-1)	unprognostic (5.16e-2)	unprognostic (4.96e-2)
unprognostic (7.21e-2)	unprognostic (7.28e-2)	unprognostic (2.80e-2)	unprognostic (1.40e-1)	unprognostic (1.32e-2)	unprognostic (5.44e-2)
			unprognostic (8.57e-2)	unprognostic (2.22e-2)	
unprognostic (1.39e-1)	unprognostic (8.96e-2)	unprognostic (2.28e-1)	unprognostic (2.51e-1)	unprognostic (5.03e-1)	unprognostic (1.66e-1)
unprognostic (9.59e-3)	unprognostic (6.81e-2)	unprognostic (2.32e-1)	unprognostic (3.46e-1)	unprognostic (1.74e-1)	unprognostic (2.44e-2)
unprognostic (1.04e-1)	unprognostic (3.71e-2)	unprognostic (9.49e-2)	unprognostic (8.39e-2)	unprognostic (2.54e-1)	unprognostic (6.16e-2)
unprognostic (6.65e-2)	unprognostic (1.78e-1)	unprognostic (1.15e-1)	unprognostic (6.30e-2)	unprognostic (1.91e-1)	unprognostic (1.63e-1)
unprognostic (2.31e-1)	unprognostic (8.68e-2)	unprognostic (1.96e-1)	unprognostic (9.82e-2)	unprognostic (3.71e-2)	unprognostic (1.09e-2)
	unprognostic (1.99e-1)	unprognostic (1.21e-2)	unprognostic (3.78e-1)	unprognostic (6.33e-2)	unprognostic (2.71e-3)
unprognostic (1.96e-2)	unprognostic (1.50e-2)	unprognostic (2.56e-2)	unprognostic (4.85e-2)		unprognostic (2.80e-1)
unprognostic (1.76e-2)	unprognostic (7.00e-2)	unprognostic (1.40e-1)	unprognostic (2.87e-2)	unprognostic (2.24e-1)	potential prognostic unfavorable (4.85e-4)
unprognostic (2.75e-2)	unprognostic (2.47e-1)	unprognostic (3.34e-2)	unprognostic (1.66e-1)	unprognostic (7.75e-2)	unprognostic (6.29e-2)
validated prognostic unfavorable (7.01e-4)	unprognostic (1.74e-2)	unprognostic (1.74e-1)	unprognostic (1.35e-2)	unprognostic (1.53e-1)	unprognostic (8.14e-3)
unprognostic (1.60e-1)	unprognostic (1.24e-1)	unprognostic (2.82e-3)			unprognostic (7.65e-2)
unprognostic (9.79e-2)	unprognostic (4.20e-1)	unprognostic (1.82e-1)	unprognostic (1.13e-1)	unprognostic (2.65e-2)	unprognostic (1.54e-2)
unprognostic (1.06e-2)	unprognostic (6.65e-2)	unprognostic (1.39e-1)	unprognostic (8.07e-3)	unprognostic (2.91e-2)	unprognostic (2.71e-2)
	unprognostic (5.07e-2)	unprognostic (4.62e-2)			
unprognostic (1.91e-2)	unprognostic (1.75e-1)	unprognostic (1.69e-1)	unprognostic (5.41e-2)	unprognostic (1.52e-2)	unprognostic (2.50e-2)
unprognostic (1.06e-2)	unprognostic (1.22e-1)	unprognostic (1.17e-1)	unprognostic (3.16e-1)	unprognostic (1.04e-1)	unprognostic (4.36e-1)
validated prognostic unfavorable (3.54e-4)	unprognostic (5.29e-3)	unprognostic (3.59e-2)	unprognostic (2.92e-2)	unprognostic (5.33e-2)	potential prognostic unfavorable (8.01e-4)
unprognostic (1.35e-2)	unprognostic (2.70e-2)	unprognostic (1.84e-1)	unprognostic (4.04e-2)	unprognostic (6.22e-2)	unprognostic (3.34e-2)
unprognostic (3.42e-1)	unprognostic (3.52e-2)	unprognostic (3.92e-2)	unprognostic (9.44e-2)		
					unprognostic (3.32e-1)
unprognostic (2.28e-3)	unprognostic (1.01e-1)	unprognostic (4.53e-1)	unprognostic (3.32e-3)	unprognostic (3.78e-1)	unprognostic (2.08e-2)
unprognostic (6.03e-2)	unprognostic (1.08e-2)	unprognostic (3.49e-1)	unprognostic (5.42e-2)	unprognostic (3.82e-1)	unprognostic (3.62e-2)
unprognostic (1.03e-2)	unprognostic (4.28e-3)	unprognostic (1.06e-1)	unprognostic (7.70e-2)	potential prognostic unfavorable (1.39e-4)	potential prognostic unfavorable (2.22e-4)
					unprognostic (2.31e-1)
unprognostic (6.89e-2)	unprognostic (1.63e-3)	unprognostic (1.05e-1)	unprognostic (4.29e-3)	unprognostic (1.16e-2)	unprognostic (2.91e-2)
unprognostic (3.30e-2)	unprognostic (7.87e-2)	unprognostic (2.87e-2)	unprognostic (3.42e-2)	unprognostic (3.59e-2)	unprognostic (4.16e-2)
unprognostic (2.58e-2)	unprognostic (2.74e-1)	unprognostic (7.78e-2)	unprognostic (2.48e-1)	unprognostic (1.68e-1)	unprognostic (2.10e-2)
unprognostic (1.71e-2)	unprognostic (5.97e-2)	unprognostic (1.18e-3)	unprognostic (1.68e-1)	unprognostic (6.52e-2)	unprognostic (1.77e-1)
unprognostic (1.61e-2)	unprognostic (4.15e-1)	unprognostic (2.73e-1)	unprognostic (7.89e-2)	unprognostic (6.26e-2)	unprognostic (5.35e-2)
unprognostic (1.49e-1)	unprognostic (2.84e-1)	unprognostic (2.39e-2)	unprognostic (1.18e-1)	unprognostic (1.78e-1)	unprognostic (8.66e-2)
unprognostic (1.93e-2)	unprognostic (1.40e-1)	unprognostic (1.15e-1)	potential prognostic unfavorable (8.21e-5)	unprognostic (7.57e-2)	unprognostic (1.81e-1)
unprognostic (7.64e-2)	unprognostic (2.99e-1)	unprognostic (3.75e-1)	unprognostic (2.31e-1)	unprognostic (7.98e-2)	unprognostic (3.33e-3)
unprognostic (1.54e-1)	unprognostic (7.88e-2)	unprognostic (1.99e-1)	unprognostic (2.04e-1)	unprognostic (6.88e-3)	unprognostic (1.27e-1)
unprognostic (1.58e-1)	unprognostic (1.54e-3)	unprognostic (7.39e-2)			unprognostic (5.57e-2)
potential prognostic unfavorable (7.49e-5)	unprognostic (4.00e-2)	unprognostic (5.71e-2)	unprognostic (4.45e-1)	unprognostic (3.13e-2)	unprognostic (1.28e-1)
unprognostic (5.70e-3)	unprognostic (3.20e-3)	unprognostic (1.36e-1)	unprognostic (1.53e-1)	unprognostic (1.45e-1)	unprognostic (5.68e-2)
		unprognostic (1.07e-2)			
potential prognostic unfavorable (2.36e-4)	unprognostic (1.55e-1)	unprognostic (4.06e-2)	unprognostic (2.20e-1)	unprognostic (1.87e-1)	potential prognostic unfavorable (1.26e-4)
unprognostic (4.98e-2)	unprognostic (6.61e-2)	unprognostic (8.49e-2)	unprognostic (2.96e-1)	unprognostic (3.73e-1)	unprognostic (1.26e-2)
unprognostic (3.57e-3)	unprognostic (2.18e-2)	unprognostic (3.06e-1)	unprognostic (6.38e-2)	unprognostic (2.81e-1)	unprognostic (9.59e-2)
unprognostic (1.21e-1)	unprognostic (8.77e-2)	unprognostic (4.79e-2)	unprognostic (4.93e-2)	unprognostic (4.21e-3)	unprognostic (9.81e-2)

unprognostic (2.97e-2)	unprognostic (1.10e-2)	unprognostic (5.82e-2)	unprognostic (2.80e-2)	unprognostic (1.49e-3)	unprognostic (5.72e-2)
unprognostic (8.64e-3)	unprognostic (9.06e-2)	unprognostic (1.12e-1)	unprognostic (4.12e-3)	unprognostic (4.52e-2)	unprognostic (2.87e-2)
	unprognostic (1.46e-1)	unprognostic (2.50e-1)	unprognostic (5.65e-2)	unprognostic (8.90e-3)	unprognostic (2.52e-2)
unprognostic (4.05e-2)	unprognostic (1.40e-1)	unprognostic (4.69e-2)	unprognostic (1.45e-1)		unprognostic (1.14e-2)
unprognostic (2.32e-1)	potential prognostic unfavorable (8.15e-4)	unprognostic (3.90e-1)	unprognostic (6.08e-3)	unprognostic (6.10e-2)	unprognostic (1.46e-1)
unprognostic (3.40e-2)	unprognostic (1.51e-1)	unprognostic (1.61e-1)	unprognostic (6.01e-2)	unprognostic (1.47e-1)	unprognostic (2.69e-2)
				unprognostic (3.17e-1)	
unprognostic (6.77e-2)	unprognostic (3.19e-2)	unprognostic (3.67e-1)	unprognostic (8.11e-2)	unprognostic (1.15e-1)	
unprognostic (1.58e-1)	unprognostic (4.28e-2)	unprognostic (1.81e-1)		unprognostic (2.68e-1)	unprognostic (1.87e-2)
unprognostic (1.92e-2)	unprognostic (2.94e-2)	unprognostic (1.59e-1)	unprognostic (5.31e-3)	unprognostic (2.55e-1)	unprognostic (4.50e-2)
unprognostic (3.24e-2)					
unprognostic (1.52e-1)	unprognostic (1.31e-2)	unprognostic (8.81e-2)	unprognostic (1.42e-2)	unprognostic (8.89e-2)	unprognostic (2.23e-1)
unprognostic (8.54e-2)	unprognostic (7.53e-3)	unprognostic (2.32e-3)	unprognostic (1.12e-1)		unprognostic (1.09e-1)
unprognostic (7.54e-3)	unprognostic (4.39e-2)	unprognostic (9.60e-2)	unprognostic (4.82e-2)	unprognostic (6.07e-2)	potential prognostic unfavorable (6.27e-5)
unprognostic (2.28e-2)	unprognostic (8.19e-2)	unprognostic (3.43e-3)	unprognostic (4.06e-2)	unprognostic (3.11e-3)	unprognostic (1.82e-2)
unprognostic (1.04e-1)	unprognostic (2.79e-1)	unprognostic (2.22e-2)	unprognostic (1.49e-2)	unprognostic (6.49e-3)	unprognostic (3.10e-2)
unprognostic (1.77e-2)	unprognostic (2.82e-2)	unprognostic (1.21e-1)	unprognostic (3.55e-3)	unprognostic (1.07e-2)	unprognostic (7.23e-2)
unprognostic (2.79e-1)	unprognostic (6.91e-3)	unprognostic (4.73e-2)	unprognostic (4.72e-3)	potential prognostic unfavorable (4.91e-5)	unprognostic (1.26e-2)
unprognostic (4.21e-2)	unprognostic (2.50e-1)	unprognostic (2.09e-1)	unprognostic (2.51e-1)	unprognostic (9.90e-2)	unprognostic (9.34e-2)
unprognostic (4.55e-2)	unprognostic (3.64e-1)	unprognostic (2.50e-1)	unprognostic (3.47e-3)	unprognostic (3.28e-3)	unprognostic (1.14e-1)
unprognostic (6.52e-3)	unprognostic (1.02e-1)	unprognostic (1.79e-1)	unprognostic (2.22e-2)	unprognostic (2.28e-1)	unprognostic (8.84e-3)
potential prognostic unfavorable (6.63e-5)	unprognostic (3.79e-1)	unprognostic (3.09e-2)	unprognostic (8.23e-2)	unprognostic (2.53e-2)	unprognostic (2.72e-3)
unprognostic (8.94e-2)	unprognostic (1.94e-2)	unprognostic (7.25e-2)	unprognostic (1.45e-1)	unprognostic (1.11e-1)	unprognostic (4.14e-1)
unprognostic (3.90e-1)	unprognostic (3.38e-2)	unprognostic (4.68e-2)	unprognostic (2.61e-2)	unprognostic (1.09e-2)	unprognostic (3.96e-3)
unprognostic (1.56e-1)	unprognostic (7.77e-2)	unprognostic (7.90e-2)	unprognostic (2.66e-2)	unprognostic (2.30e-3)	unprognostic (1.92e-1)
unprognostic (1.19e-2)	unprognostic (3.20e-2)	unprognostic (6.00e-2)	unprognostic (8.52e-3)	unprognostic (3.78e-1)	unprognostic (1.82e-2)
unprognostic (1.41e-2)	unprognostic (9.15e-2)	unprognostic (1.59e-1)	unprognostic (5.39e-2)	unprognostic (2.76e-2)	potential prognostic unfavorable (8.03e-4)
unprognostic (2.75e-1)	unprognostic (1.86e-1)	unprognostic (1.75e-1)	unprognostic (1.00e-2)	unprognostic (2.28e-1)	unprognostic (2.60e-1)
unprognostic (5.99e-2)	potential prognostic unfavorable (5.57e-4)	unprognostic (4.14e-1)	unprognostic (1.04e-2)	unprognostic (4.77e-2)	unprognostic (4.00e-1)
unprognostic (1.90e-1)	unprognostic (7.79e-3)	unprognostic (5.31e-2)	unprognostic (2.89e-3)	unprognostic (6.19e-2)	unprognostic (2.00e-1)
unprognostic (1.77e-3)	unprognostic (9.57e-2)	unprognostic (2.11e-1)	unprognostic (3.47e-2)	unprognostic (9.52e-3)	unprognostic (2.74e-2)
unprognostic (2.75e-1)					
unprognostic (2.51e-3)	unprognostic (5.95e-2)	unprognostic (1.40e-1)	unprognostic (6.58e-2)	unprognostic (4.42e-3)	unprognostic (5.32e-2)
unprognostic (4.54e-3)	unprognostic (1.15e-1)	unprognostic (2.87e-1)	unprognostic (6.60e-3)	unprognostic (5.81e-2)	unprognostic (7.52e-2)
unprognostic (3.28e-1)	unprognostic (1.94e-2)	unprognostic (1.37e-1)	unprognostic (1.90e-1)	potential prognostic unfavorable (1.09e-4)	unprognostic (2.34e-1)
unprognostic (3.21e-1)	unprognostic (2.23e-1)	unprognostic (5.56e-2)	unprognostic (1.18e-2)	unprognostic (1.40e-1)	unprognostic (7.79e-2)
unprognostic (5.30e-3)	unprognostic (6.59e-2)	unprognostic (1.02e-2)	unprognostic (3.88e-3)	unprognostic (6.22e-3)	unprognostic (3.68e-2)
unprognostic (2.96e-1)	unprognostic (2.16e-2)	unprognostic (9.80e-2)	unprognostic (1.28e-1)	unprognostic (1.55e-2)	unprognostic (1.86e-1)
		unprognostic (4.07e-1)	unprognostic (1.18e-1)	unprognostic (1.84e-3)	
			unprognostic (3.03e-2)	unprognostic (9.78e-2)	unprognostic (9.89e-3)
unprognostic (2.56e-2)	unprognostic (6.29e-2)	unprognostic (1.62e-3)	unprognostic (1.61e-2)	unprognostic (9.31e-2)	unprognostic (1.34e-2)
	unprognostic (2.08e-2)		unprognostic (3.43e-1)		
unprognostic (3.23e-2)	unprognostic (7.33e-2)	unprognostic (1.55e-1)	unprognostic (6.92e-2)	unprognostic (3.70e-1)	unprognostic (1.04e-1)
unprognostic (2.55e-1)	potential prognostic unfavorable (2.82e-4)	unprognostic (6.29e-3)	unprognostic (5.74e-2)	unprognostic (8.89e-2)	unprognostic (1.19e-1)
unprognostic (1.59e-1)	unprognostic (4.02e-2)	unprognostic (4.41e-1)		unprognostic (1.42e-1)	unprognostic (1.34e-1)
	unprognostic (5.59e-2)		unprognostic (1.98e-2)		unprognostic (4.04e-3)
unprognostic (2.22e-3)	unprognostic (1.51e-2)	unprognostic (3.22e-1)	unprognostic (1.01e-1)		unprognostic (2.42e-1)
unprognostic (3.29e-1)		unprognostic (2.54e-3)	unprognostic (7.59e-3)	unprognostic (4.69e-2)	
unprognostic (3.43e-2)	unprognostic (6.68e-2)	unprognostic (1.65e-1)	unprognostic (1.82e-1)		unprognostic (7.71e-2)
unprognostic (4.97e-2)	unprognostic (6.75e-3)	unprognostic (1.61e-1)	unprognostic (4.40e-2)	potential prognostic unfavorable (6.87e-4)	unprognostic (3.21e-3)
unprognostic (2.21e-1)	unprognostic (4.04e-2)	unprognostic (5.62e-2)	unprognostic (5.82e-3)	unprognostic (1.32e-3)	unprognostic (3.90e-2)
unprognostic (3.31e-2)	unprognostic (3.55e-1)	unprognostic (2.64e-2)	unprognostic (1.24e-2)	unprognostic (3.87e-1)	unprognostic (4.23e-3)
unprognostic (3.31e-2)	unprognostic (3.01e-1)	unprognostic (8.90e-2)	unprognostic (1.60e-1)	unprognostic (9.66e-2)	unprognostic (5.98e-3)
unprognostic (2.90e-3)	unprognostic (6.07e-2)	unprognostic (2.17e-1)	unprognostic (1.09e-1)	unprognostic (9.01e-2)	unprognostic (2.38e-3)
unprognostic (1.07e-1)	unprognostic (4.13e-2)	unprognostic (2.79e-2)	unprognostic (3.52e-2)	unprognostic (3.41e-1)	unprognostic (1.51e-3)
unprognostic (8.16e-2)	unprognostic (1.47e-3)	unprognostic (1.51e-1)	unprognostic (5.09e-2)	unprognostic (2.99e-1)	unprognostic (3.43e-3)
unprognostic (1.86e-3)	unprognostic (1.76e-1)	unprognostic (4.17e-1)	unprognostic (3.27e-2)	unprognostic (5.87e-2)	unprognostic (4.00e-1)
unprognostic (6.79e-2)	unprognostic (2.18e-1)	unprognostic (3.61e-2)	unprognostic (5.71e-2)	unprognostic (2.39e-1)	unprognostic (4.22e-1)
unprognostic (5.26e-2)	unprognostic (4.44e-3)	unprognostic (2.25e-1)	unprognostic (1.26e-2)	unprognostic (2.79e-2)	unprognostic (1.18e-1)
					unprognostic (5.78e-2)
unprognostic (6.66e-2)	unprognostic (7.84e-2)	unprognostic (2.58e-2)	unprognostic (3.18e-1)	unprognostic (2.26e-1)	unprognostic (3.31e-2)
unprognostic (1.22e-2)	unprognostic (3.52e-2)	unprognostic (1.14e-1)	unprognostic (2.61e-1)	unprognostic (3.87e-2)	unprognostic (4.80e-2)
unprognostic (2.58e-1)	unprognostic (2.22e-1)	unprognostic (1.10e-1)	unprognostic (6.10e-2)	unprognostic (2.12e-3)	unprognostic (3.63e-2)
unprognostic (1.87e-2)	unprognostic (1.57e-1)	unprognostic (2.65e-2)	unprognostic (2.56e-2)		unprognostic (1.84e-2)
unprognostic (4.34e-2)	unprognostic (7.40e-2)	unprognostic (2.37e-1)	unprognostic (1.26e-1)	unprognostic (8.82e-3)	unprognostic (2.01e-2)
unprognostic (2.84e-2)	unprognostic (2.55e-1)	unprognostic (2.88e-2)	unprognostic (2.58e-1)	potential prognostic unfavorable (1.66e-4)	unprognostic (2.27e-3)
			unprognostic (1.65e-2)		
unprognostic (1.24e-1)	unprognostic (1.10e-2)	unprognostic (3.48e-1)	unprognostic (1.31e-1)	unprognostic (4.85e-2)	unprognostic (2.99e-2)
unprognostic (1.10e-2)	unprognostic (1.39e-1)	unprognostic (2.54e-2)	unprognostic (5.68e-2)	unprognostic (2.62e-2)	unprognostic (2.74e-3)
potential prognostic unfavorable (2.79e-4)	unprognostic (1.54e-1)	unprognostic (9.05e-2)	unprognostic (1.79e-2)	unprognostic (9.78e-2)	unprognostic (2.76e-2)
					unprognostic (1.94e-1)
unprognostic (8.29e-3)	unprognostic (1.04e-2)	unprognostic (1.03e-2)	unprognostic (6.46e-2)	unprognostic (1.65e-1)	unprognostic (8.60e-3)
unprognostic (8.25e-2)	unprognostic (2.34e-1)	unprognostic (1.78e-1)			unprognostic (6.86e-2)
unprognostic (2.16e-3)					
unprognostic (9.86e-2)	unprognostic (7.60e-2)	unprognostic (9.68e-3)	unprognostic (2.58e-2)	unprognostic (1.42e-1)	unprognostic (4.30e-3)
unprognostic (4.42e-3)	unprognostic (1.65e-2)	unprognostic (1.49e-1)	unprognostic (2.50e-1)	unprognostic (1.51e-1)	unprognostic (5.15e-3)
unprognostic (4.90e-2)	unprognostic (8.90e-2)	unprognostic (2.65e-1)	unprognostic (1.70e-1)	unprognostic (6.96e-3)	unprognostic (9.42e-3)
potential prognostic unfavorable (8.03e-4)	unprognostic (1.61e-1)	unprognostic (2.60e-1)	unprognostic (7.54e-2)	unprognostic (2.16e-1)	unprognostic (2.74e-1)
potential prognostic unfavorable (6.73e-4)	unprognostic (8.01e-2)	unprognostic (5.19e-2)	unprognostic (1.04e-1)	unprognostic (1.30e-1)	unprognostic (4.09e-2)
unprognostic (3.68e-1)		unprognostic (3.32e-1)	unprognostic (3.45e-2)	unprognostic (9.78e-2)	
unprognostic (2.69e-2)	potential prognostic unfavorable (6.48e-4)	unprognostic (2.06e-1)	unprognostic (1.86e-1)	unprognostic (2.41e-1)	unprognostic (6.37e-2)
unprognostic (4.25e-2)	unprognostic (5.87e-2)	unprognostic (3.05e-1)	unprognostic (3.66e-1)	unprognostic (2.97e-1)	unprognostic (1.22e-1)
validated prognostic unfavorable (1.59e-4)	unprognostic (4.54e-2)	unprognostic (3.98e-1)	unprognostic (1.64e-1)	unprognostic (6.45e-3)	validated prognostic unfavorable (3.28e-4)
unprognostic (1.37e-2)	unprognostic (8.82e-2)	unprognostic (1.88e-2)	unprognostic (1.20e-1)	unprognostic (4.69e-2)	unprognostic (2.18e-2)
unprognostic (7.41e-2)	unprognostic (6.32e-2)	unprognostic (6.13e-2)			
unprognostic (5.59e-2)	unprognostic (2.88e-1)	unprognostic (6.36e-2)			unprognostic (2.67e-1)
unprognostic (2.35e-1)	unprognostic (7.60e-2)	unprognostic (1.55e-1)	unprognostic (1.43e-2)	unprognostic (3.38e-1)	unprognostic (6.90e-2)
unprognostic (2.82e-1)	unprognostic (2.57e-2)	unprognostic (1.36e-1)	unprognostic (7.01e-2)	unprognostic (2.11e-1)	unprognostic (1.28e-1)
unprognostic (8.00e-2)	unprognostic (1.30e-3)	unprognostic (2.70e-1)	unprognostic (1.09e-2)	unprognostic (1.14e-1)	unprognostic (7.38e-3)
unprognostic (2.58e-2)	unprognostic (2.98e-2)	unprognostic (7.84e-2)	unprognostic (2.29e-1)	unprognostic (6.39e-2)	unprognostic (2.86e-1)
unprognostic (9.97e-2)	unprognostic (4.32e-1)	unprognostic (1.16e-1)	unprognostic (7.74e-3)	unprognostic (2.10e-2)	unprognostic (4.87e-2)
validated prognostic unfavorable (6.17e-4)	unprognostic (4.04e-2)	unprognostic (2.24e-2)	unprognostic (9.87e-2)	unprognostic (3.43e-1)	unprognostic (4.46e-2)
unprognostic (4.15e-2)	unprognostic (2.18e-1)	unprognostic (2.89e-1)	unprognostic (8.15e-3)	unprognostic (6.29e-2)	unprognostic (5.76e-2)
unprognostic (4.60e-2)	unprognostic (4.18e-1)	unprognostic (6.01e-2)	unprognostic (1.40e-1)	unprognostic (4.58e-2)	unprognostic (1.50e-3)
unprognostic (2.13e-1)	unprognostic (1.08e-1)	unprognostic (2.85e-1)	unprognostic (3.43e-3)	unprognostic (3.58e-2)	potential prognostic unfavorable (2.08e-4)
unprognostic (4.21e-2)	unprognostic (2.46e-1)	unprognostic (2.74e-1)	unprognostic (9.44e-2)	unprognostic (1.17e-2)	unprognostic (6.01e-3)
unprognostic (1.23e-2)	unprognostic (1.71e-1)	unprognostic (1.52e-1)	unprognostic (4.82e-1)	unprognostic (8.27e-2)	unprognostic (1.89e-1)
unprognostic (9.27e-2)	unprognostic (8.93e-2)	unprognostic (4.76e-2)	unprognostic (6.91e-2)	unprognostic (2.46e-2)	unprognostic (2.83e-2)
unprognostic (2.85e-2)	unprognostic (4.40e-2)	unprognostic (1.35e-1)	unprognostic (7.17e-3)	potential prognostic unfavorable (6.11e-4)	potential prognostic unfavorable (1.40e-4)
potential prognostic unfavorable (2.65e-4)	unprognostic (2.19e-1)	unprognostic (2.57e-1)	unprognostic (4.95e-2)	unprognostic (7.66e-2)	potential prognostic unfavorable (2.06e-4)
unprognostic (1.30e-2)	unprognostic (3.14e-1)	unprognostic (4.04e-1)	unprognostic (5.20e-2)	unprognostic (2.26e-1)	potential prognostic unfavorable (1.42e-5)
unprognostic (3.90e-3)	unprognostic (6.48e-2)	unprognostic (2.37e-1)	unprognostic (1.32e-1)	unprognostic (1.15e-1)	unprognostic (3.12e-3)

Cancer prognostics - Pancreatic Adenocarcinoma (validation)	Cancer prognostics - Prostate Adenocarcinoma (TCGA)	Cancer prognostics - Rectum Adenocarcinoma (TCGA)	Cancer prognostics - Rectum Adenocarcinoma (validation)	Cancer prognostics - Skin Cutaneous Melanoma (TCGA)	Cancer prognostics - Stomach Adenocarcinoma (TCGA)
potential prognostic unfavorable (9.11e-4)	unprognostic (1.14e-1)	unprognostic (6.14e-3)	unprognostic (1.30e-2)	unprognostic (5.20e-1)	unprognostic (9.10e-2)
unprognostic (9.14e-2)	unprognostic (1.69e-1)	unprognostic (5.30e-1)	unprognostic (9.79e-2)	unprognostic (2.08e-1)	unprognostic (8.84e-3)
unprognostic (5.37e-2)	unprognostic (1.45e-1)	unprognostic (1.99e-1)	unprognostic (1.36e-2)	unprognostic (2.73e-1)	potential prognostic unfavorable (7.46e-4)
potential prognostic unfavorable (3.23e-5)	unprognostic (3.26e-2)	unprognostic (9.74e-3)	unprognostic (3.68e-1)	unprognostic (1.46e-1)	unprognostic (3.11e-2)
unprognostic (1.10e-1)	unprognostic (3.54e-1)	unprognostic (2.71e-2)	unprognostic (1.33e-2)		unprognostic (1.21e-1)
unprognostic (7.84e-3)			unprognostic (2.42e-1)	unprognostic (2.60e-1)	
unprognostic (1.75e-1)	unprognostic (1.25e-1)	unprognostic (1.16e-1)	unprognostic (7.91e-2)	unprognostic (4.57e-1)	unprognostic (9.85e-2)
unprognostic (1.99e-1)	unprognostic (1.63e-1)	unprognostic (5.58e-3)	unprognostic (4.55e-2)	unprognostic (2.40e-1)	unprognostic (3.35e-1)
unprognostic (3.53e-1)	unprognostic (3.24e-1)	unprognostic (1.78e-1)	unprognostic (2.13e-1)		unprognostic (1.89e-2)
unprognostic (1.08e-2)	unprognostic (3.36e-2)	unprognostic (1.16e-1)	unprognostic (1.33e-3)	unprognostic (7.63e-2)	unprognostic (3.24e-3)
unprognostic (3.99e-2)	unprognostic (5.07e-2)	unprognostic (7.73e-2)	unprognostic (9.51e-3)		unprognostic (2.08e-1)
unprognostic (4.03e-2)	unprognostic (7.26e-2)	unprognostic (2.56e-1)	unprognostic (4.62e-2)	unprognostic (1.84e-1)	unprognostic (2.73e-2)
unprognostic (6.04e-2)	unprognostic (3.76e-2)	unprognostic (1.21e-1)	unprognostic (3.07e-3)	unprognostic (6.33e-3)	potential prognostic unfavorable (1.37e-4)
unprognostic (1.22e-3)	unprognostic (7.92e-2)	unprognostic (2.84e-1)	unprognostic (4.38e-2)	unprognostic (3.72e-2)	unprognostic (1.30e-1)
unprognostic (1.13e-2)	unprognostic (1.18e-1)	unprognostic (1.52e-1)	unprognostic (1.05e-1)	unprognostic (8.53e-2)	unprognostic (1.51e-2)
unprognostic (1.41e-1)		unprognostic (9.85e-2)	unprognostic (6.35e-3)	unprognostic (9.98e-2)	unprognostic (2.78e-3)
unprognostic (4.70e-2)	unprognostic (6.13e-2)	unprognostic (6.88e-2)	unprognostic (9.57e-2)	unprognostic (2.45e-1)	unprognostic (9.09e-2)
unprognostic (4.03e-1)	unprognostic (2.09e-1)	unprognostic (3.85e-2)	potential prognostic favorable (6.24e-4)	unprognostic (4.23e-1)	unprognostic (1.63e-1)
unprognostic (1.12e-1)	unprognostic (9.06e-2)		unprognostic (2.38e-2)		unprognostic (5.71e-2)
unprognostic (1.78e-1)	unprognostic (4.51e-1)	unprognostic (1.03e-1)	unprognostic (1.11e-1)	unprognostic (2.61e-1)	unprognostic (9.43e-2)
unprognostic (8.19e-2)	unprognostic (1.19e-1)	unprognostic (7.96e-2)	unprognostic (8.29e-2)	unprognostic (2.63e-1)	unprognostic (4.41e-2)
unprognostic (1.43e-1)	unprognostic (2.93e-2)				
unprognostic (1.69e-1)	unprognostic (3.09e-1)	unprognostic (7.61e-3)	unprognostic (4.68e-2)	unprognostic (6.66e-2)	unprognostic (2.74e-1)
unprognostic (1.90e-2)	unprognostic (3.39e-2)	unprognostic (2.57e-1)	unprognostic (1.58e-2)	potential prognostic unfavorable (2.34e-4)	unprognostic (2.09e-2)
unprognostic (1.48e-1)	unprognostic (4.67e-2)		unprognostic (1.58e-1)	unprognostic (5.70e-2)	unprognostic (2.19e-1)
unprognostic (8.06e-2)	unprognostic (1.01e-1)	unprognostic (4.72e-2)	unprognostic (4.16e-3)	unprognostic (1.47e-2)	unprognostic (1.14e-2)
unprognostic (1.79e-1)	unprognostic (2.84e-1)		unprognostic (1.81e-1)		
unprognostic (9.22e-2)		unprognostic (1.41e-1)	unprognostic (1.31e-1)	unprognostic (1.20e-1)	unprognostic (3.51e-2)
unprognostic (1.98e-2)		unprognostic (1.83e-1)	unprognostic (5.14e-1)	unprognostic (2.00e-1)	unprognostic (8.92e-2)
unprognostic (6.17e-3)				unprognostic (9.64e-3)	
unprognostic (7.70e-3)	unprognostic (2.20e-1)		unprognostic (2.45e-2)	unprognostic (1.48e-1)	unprognostic (3.00e-2)
unprognostic (5.74e-3)	unprognostic (1.39e-1)	unprognostic (1.64e-2)	unprognostic (6.35e-2)	unprognostic (8.20e-2)	unprognostic (9.55e-3)
potential prognostic unfavorable (5.01e-4)					
unprognostic (6.09e-2)	unprognostic (3.88e-1)	unprognostic (4.50e-2)	unprognostic (5.10e-3)	unprognostic (1.15e-1)	unprognostic (4.35e-2)
unprognostic (8.33e-3)	unprognostic (3.99e-2)	unprognostic (1.02e-1)	unprognostic (4.27e-3)	unprognostic (3.07e-1)	unprognostic (8.62e-3)
unprognostic (3.33e-2)	unprognostic (5.75e-2)	unprognostic (2.03e-1)	unprognostic (2.14e-2)	unprognostic (1.43e-1)	unprognostic (4.18e-3)
unprognostic (4.07e-3)		unprognostic (9.07e-3)	unprognostic (2.07e-1)	unprognostic (1.83e-1)	unprognostic (2.36e-1)
unprognostic (1.54e-1)			unprognostic (1.24e-1)	unprognostic (2.58e-1)	unprognostic (1.57e-1)
unprognostic (1.00e-1)	unprognostic (3.61e-1)	unprognostic (1.71e-1)	unprognostic (3.04e-2)	unprognostic (4.29e-1)	unprognostic (7.61e-3)
				unprognostic (2.65e-3)	
unprognostic (2.76e-2)	unprognostic (3.74e-1)	unprognostic (4.94e-2)	unprognostic (2.71e-2)	unprognostic (9.23e-2)	unprognostic (3.50e-2)
unprognostic (1.19e-1)	unprognostic (1.12e-1)	potential prognostic favorable (1.14e-5)	unprognostic (2.14e-2)	unprognostic (1.24e-1)	unprognostic (3.43e-1)
potential prognostic favorable (8.11e-4)	unprognostic (1.11e-1)	unprognostic (6.17e-2)	unprognostic (8.26e-2)	unprognostic (7.18e-2)	unprognostic (2.41e-1)
unprognostic (2.10e-1)	unprognostic (4.04e-2)	unprognostic (5.85e-2)	unprognostic (3.74e-2)	unprognostic (5.89e-2)	unprognostic (3.48e-3)
unprognostic (3.56e-3)	unprognostic (6.21e-2)	unprognostic (3.11e-2)	unprognostic (2.16e-2)	unprognostic (1.85e-1)	unprognostic (9.27e-2)
potential prognostic unfavorable (2.04e-4)	unprognostic (3.85e-2)	unprognostic (3.23e-3)	unprognostic (4.17e-2)	unprognostic (1.01e-1)	unprognostic (5.62e-3)
unprognostic (1.77e-2)	unprognostic (1.30e-1)	unprognostic (1.29e-3)	unprognostic (2.23e-1)	unprognostic (4.86e-1)	unprognostic (1.79e-1)
unprognostic (1.71e-1)	unprognostic (3.76e-2)	unprognostic (1.24e-1)	unprognostic (4.99e-2)	unprognostic (3.30e-1)	unprognostic (1.99e-1)
unprognostic (7.33e-2)	unprognostic (4.26e-2)	unprognostic (4.18e-3)	potential prognostic favorable (5.88e-4)	unprognostic (1.42e-1)	unprognostic (7.64e-2)
unprognostic (1.33e-2)	unprognostic (1.43e-1)	unprognostic (8.19e-2)	unprognostic (1.35e-1)	unprognostic (3.84e-2)	unprognostic (1.73e-1)
unprognostic (1.03e-1)	unprognostic (2.13e-1)	unprognostic (2.65e-1)	unprognostic (1.60e-1)	unprognostic (2.33e-2)	unprognostic (4.87e-2)
unprognostic (9.12e-2)			unprognostic (2.36e-1)		
unprognostic (1.90e-1)	unprognostic (2.63e-1)	unprognostic (8.28e-2)	unprognostic (2.89e-1)	unprognostic (1.15e-1)	unprognostic (1.32e-2)
unprognostic (7.63e-2)	unprognostic (4.32e-1)	unprognostic (1.49e-1)	unprognostic (6.25e-2)	unprognostic (1.35e-1)	unprognostic (2.58e-2)
unprognostic (3.66e-3)	unprognostic (3.30e-1)	unprognostic (1.30e-1)	unprognostic (4.76e-3)	unprognostic (1.51e-1)	unprognostic (3.59e-2)
unprognostic (2.06e-2)		unprognostic (1.13e-1)	unprognostic (2.58e-2)		unprognostic (6.59e-2)
unprognostic (4.35e-2)	unprognostic (2.65e-1)	unprognostic (7.54e-3)	unprognostic (5.99e-2)	unprognostic (1.83e-1)	unprognostic (2.88e-1)
unprognostic (1.59e-1)	unprognostic (4.12e-3)	unprognostic (6.00e-2)	unprognostic (3.64e-1)	unprognostic (1.45e-1)	unprognostic (1.21e-1)
unprognostic (1.92e-1)	unprognostic (3.38e-1)	unprognostic (2.75e-1)	unprognostic (3.64e-3)	unprognostic (1.72e-2)	unprognostic (1.67e-2)
unprognostic (6.56e-2)	unprognostic (1.17e-1)	unprognostic (2.96e-2)	unprognostic (2.47e-1)	unprognostic (7.70e-3)	unprognostic (7.37e-3)
unprognostic (1.87e-1)	unprognostic (1.12e-1)	unprognostic (7.56e-2)	unprognostic (3.37e-3)	unprognostic (4.45e-1)	unprognostic (1.04e-1)
unprognostic (2.59e-2)	unprognostic (9.50e-2)	unprognostic (3.34e-1)	potential prognostic unfavorable (3.84e-4)	unprognostic (3.05e-3)	unprognostic (1.01e-1)
unprognostic (2.25e-1)	unprognostic (4.48e-2)	unprognostic (2.04e-2)	unprognostic (1.41e-3)	unprognostic (3.28e-1)	unprognostic (6.88e-3)
unprognostic (1.49e-1)	unprognostic (1.07e-2)	unprognostic (1.46e-2)	unprognostic (2.87e-1)	unprognostic (1.65e-1)	unprognostic (1.17e-1)
unprognostic (3.40e-2)	unprognostic (1.56e-2)	unprognostic (2.01e-2)	unprognostic (2.32e-2)	unprognostic (1.53e-1)	unprognostic (1.21e-1)
unprognostic (7.40e-3)	unprognostic (1.58e-1)	unprognostic (6.31e-2)	unprognostic (6.58e-2)	unprognostic (9.33e-2)	unprognostic (1.63e-1)
unprognostic (1.59e-1)	unprognostic (1.23e-1)	unprognostic (7.32e-2)	unprognostic (2.92e-3)	unprognostic (2.70e-1)	unprognostic (2.77e-2)
unprognostic (2.22e-1)	unprognostic (7.05e-2)	unprognostic (2.89e-2)	unprognostic (4.77e-2)	unprognostic (3.79e-1)	unprognostic (1.43e-1)
unprognostic (6.34e-2)	unprognostic (1.81e-1)	unprognostic (3.19e-1)	unprognostic (8.43e-3)	unprognostic (2.45e-1)	unprognostic (3.26e-2)
potential prognostic unfavorable (5.66e-5)	unprognostic (2.24e-1)	unprognostic (1.99e-1)	unprognostic (1.22e-1)	unprognostic (1.56e-1)	unprognostic (9.36e-2)
potential prognostic unfavorable (1.63e-4)	unprognostic (2.43e-1)	unprognostic (1.83e-2)	unprognostic (1.32e-2)	unprognostic (3.12e-1)	unprognostic (7.96e-2)
unprognostic (3.13e-2)	unprognostic (1.49e-1)	unprognostic (2.04e-2)	unprognostic (7.50e-2)	unprognostic (2.27e-1)	unprognostic (1.33e-1)
unprognostic (1.02e-2)	unprognostic (2.68e-1)	unprognostic (3.16e-1)	unprognostic (6.07e-3)	unprognostic (4.01e-2)	unprognostic (9.75e-2)
unprognostic (1.57e-2)	unprognostic (2.56e-1)	unprognostic (1.54e-1)	unprognostic (2.64e-2)	unprognostic (8.39e-2)	unprognostic (3.97e-1)
unprognostic (1.43e-1)	unprognostic (4.53e-2)	unprognostic (3.08e-1)	unprognostic (4.42e-3)	unprognostic (2.10e-2)	unprognostic (2.60e-1)
unprognostic (3.55e-2)	unprognostic (2.28e-1)	unprognostic (2.73e-2)	unprognostic (2.35e-1)	unprognostic (2.69e-1)	unprognostic (1.14e-1)
unprognostic (4.36e-2)	unprognostic (2.85e-2)	unprognostic (1.97e-2)	unprognostic (2.68e-1)	unprognostic (2.50e-1)	unprognostic (1.29e-2)
unprognostic (5.93e-2)	unprognostic (1.05e-1)	unprognostic (5.04e-2)	unprognostic (5.62e-2)	unprognostic (2.33e-3)	unprognostic (3.79e-1)
unprognostic (3.88e-1)	unprognostic (5.87e-2)	unprognostic (1.07e-1)	unprognostic (1.64e-1)	unprognostic (5.91e-2)	unprognostic (1.52e-2)
unprognostic (1.33e-1)	unprognostic (3.40e-1)	unprognostic (1.87e-1)	unprognostic (2.09e-1)	unprognostic (5.69e-2)	unprognostic (2.04e-2)
unprognostic (9.73e-3)	unprognostic (1.30e-1)	unprognostic (1.33e-3)	unprognostic (2.05e-2)	unprognostic (4.24e-1)	unprognostic (7.00e-2)
unprognostic (1.70e-3)	unprognostic (1.10e-1)		unprognostic (2.86e-1)	unprognostic (2.36e-2)	
unprognostic (7.87e-2)	unprognostic (5.52e-3)	unprognostic (4.48e-1)	unprognostic (8.80e-2)	unprognostic (7.63e-2)	unprognostic (8.79e-2)
unprognostic (2.67e-2)	unprognostic (7.26e-3)	unprognostic (1.55e-1)	unprognostic (7.93e-2)	unprognostic (8.56e-2)	unprognostic (4.83e-2)
unprognostic (2.74e-3)	unprognostic (1.27e-1)	potential prognostic favorable (5.17e-4)	unprognostic (1.03e-3)	unprognostic (2.53e-1)	unprognostic (9.93e-3)
validated prognostic unfavorable (1.81e-5)	unprognostic (1.39e-1)	unprognostic (1.83e-2)	unprognostic (2.16e-3)	unprognostic (2.04e-1)	unprognostic (1.28e-1)
					unprognostic (1.63e-3)
unprognostic (2.70e-1)	unprognostic (3.22e-2)	unprognostic (1.47e-1)	unprognostic (9.42e-2)	unprognostic (4.76e-2)	unprognostic (1.96e-1)
unprognostic (2.17e-1)	unprognostic (1.13e-2)	unprognostic (5.43e-3)	unprognostic (2.01e-2)	unprognostic (1.16e-1)	unprognostic (1.20e-1)
unprognostic (1.41e-2)	unprognostic (3.17e-2)	unprognostic (3.65e-2)	unprognostic (4.48e-2)	unprognostic (1.56e-1)	unprognostic (5.17e-2)
unprognostic (3.77e-2)	unprognostic (1.34e-1)	unprognostic (1.49e-1)	unprognostic (2.37e-1)	unprognostic (1.08e-2)	unprognostic (9.00e-2)
	unprognostic (3.59e-1)		unprognostic (1.27e-1)	unprognostic (2.41e-1)	
unprognostic (2.10e-1)	unprognostic (2.27e-1)	unprognostic (8.81e-2)	unprognostic (2.39e-1)	unprognostic (3.29e-3)	unprognostic (3.63e-2)
unprognostic (5.81e-2)			unprognostic (2.30e-2)		unprognostic (2.78e-2)
unprognostic (1.29e-2)	unprognostic (2.85e-1)	unprognostic (1.04e-1)	unprognostic (5.50e-2)	unprognostic (1.56e-2)	unprognostic (2.65e-2)
unprognostic (1.38e-1)	unprognostic (1.01e-1)	unprognostic (7.18e-2)	unprognostic (1.78e-2)	unprognostic (2.33e-1)	unprognostic (1.47e-2)
unprognostic (1.85e-1)	unprognostic (2.14e-1)	unprognostic (8.81e-3)	unprognostic (1.18e-2)	unprognostic (1.05e-3)	unprognostic (2.22e-2)
potential prognostic unfavorable (7.58e-4)	unprognostic (1.03e-1)	unprognostic (1.95e-1)	unprognostic (1.08e-1)	unprognostic (1.83e-1)	unprognostic (1.33e-1)
	unprognostic (1.50e-1)			unprognostic (1.37e-1)	
unprognostic (3.91e-1)	unprognostic (1.18e-1)	unprognostic (1.36e-2)	unprognostic (4.13e-3)	unprognostic (1.60e-1)	unprognostic (2.49e-1)
unprognostic (2.03e-1)	unprognostic (1.91e-1)	unprognostic (1.27e-1)	unprognostic (4.62e-2)	unprognostic (2.42e-1)	unprognostic (5.68e-3)
unprognostic (1.20e-2)	unprognostic (1.90e-1)	unprognostic (1.83e-1)	unprognostic (1.06e-3)	unprognostic (3.69e-2)	unprognostic (3.56e-3)
unprognostic (7.86e-2)	unprognostic (4.18e-2)	unprognostic (4.20e-1)	unprognostic (1.68e-1)	unprognostic (6.18e-2)	unprognostic (1.86e-1)
unprognostic (2.96e-1)	unprognostic (1.09e-1)	unprognostic (1.44e-1)	unprognostic (3.90e-2)	unprognostic (3.72e-3)	unprognostic (3.13e-2)
unprognostic (1.18e-2)	unprognostic (3.75e-1)	unprognostic (2.12e-1)	unprognostic (4.13e-2)	unprognostic (1.15e-1)	unprognostic (9.83e-2)
unprognostic (2.37e-2)	unprognostic (7.22e-2)	unprognostic (1.63e-1)	unprognostic (9.10e-2)	unprognostic (1.43e-1)	unprognostic (5.22e-2)
unprognostic (7.98e-3)	unprognostic (1.44e-1)	unprognostic (3.12e-3)	unprognostic (3.57e-1)	unprognostic (4.97e-1)	potential prognostic unfavorable (8.18e-4)
unprognostic (5.16e-2)	unprognostic (2.84e-1)	unprognostic (2.07e-1)	unprognostic (9.48e-2)	unprognostic (5.34e-2)	unprognostic (1.04e-2)
unprognostic (3.16e-1)	unprognostic (1.16e-1)	unprognostic (1.42e-1)	unprognostic (5.20e-3)	unprognostic (2.58e-2)	unprognostic (5.14e-2)
unprognostic (9.34e-2)	unprognostic (1.34e-1)	unprognostic (1.60e-2)	unprognostic (1.63e-1)	unprognostic (2.45e-2)	unprognostic (3.18e-1)
unprognostic (7.07e-3)	unprognostic (1.32e-1)	unprognostic (3.89e-2)	unprognostic (3.46e-1)	unprognostic (2.77e-1)	unprognostic (1.52e-1)
unprognostic (2.72e-1)	unprognostic (1.91e-1)	unprognostic (2.25e-2)	unprognostic (5.83e-2)	unprognostic (2.50e-2)	unprognostic (2.45e-2)
unprognostic (9.69e-2)	unprognostic (1.10e-1)	unprognostic (1.69e-1)	unprognostic (6.53e-2)	unprognostic (2.23e-1)	
unprognostic (6.74e-2)	unprognostic (5.88e-2)	unprognostic (1.01e-2)	unprognostic (3.51e-2)	unprognostic (7.61e-2)	unprognostic (1.23e-2)
unprognostic (2.47e-2)	unprognostic (1.21e-1)	unprognostic (8.43e-2)	unprognostic (2.20e-3)	unprognostic (5.50e-2)	unprognostic (2.47e-1)
unprognostic (2.66e-3)	unprognostic (2.23e-1)	unprognostic (2.12e-2)	unprognostic (1.36e-1)	unprognostic (3.19e-2)	unprognostic (1.20e-1)
unprognostic (1.37e-1)	unprognostic (7.02e-2)		unprognostic (4.27e-2)	unprognostic (2.56e-1)	unprognostic (3.76e-2)
unprognostic (3.75e-1)	unprognostic (2.62e-1)	unprognostic (2.09e-1)	unprognostic (1.85e-2)		unprognostic (1.28e-1)
unprognostic (1.02e-1)	unprognostic (1.36e-1)	unprognostic (7.84e-2)	unprognostic (1.14e-1)	unprognostic (1.72e-2)	unprognostic (2.10e-1)
unprognostic (6.43e-2)	unprognostic (1.59e-2)	unprognostic (4.79e-2)	unprognostic (2.25e-1)	potential prognostic unfavorable (9.85e-4)	unprognostic (9.75e-2)
unprognostic (2.84e-1)	unprognostic (5.03e-3)	unprognostic (1.73e-1)	unprognostic (1.02e-1)	unprognostic (1.09e-2)	unprognostic (4.08e-2)

unprognostic (5.25e-3)		unprognostic (8.55e-2)	unprognostic (1.34e-3)	unprognostic (1.00e-1)	unprognostic (6.38e-2)
unprognostic (2.39e-1)	unprognostic (6.39e-2)		unprognostic (8.77e-3)		unprognostic (2.07e-2)
unprognostic (1.30e-1)	unprognostic (1.54e-1)	unprognostic (1.68e-2)	unprognostic (2.43e-2)	unprognostic (9.40e-2)	unprognostic (2.86e-2)
unprognostic (5.19e-2)	unprognostic (2.41e-1)	unprognostic (1.18e-1)	unprognostic (4.34e-2)	potential prognostic unfavorable (1.64e-4)	unprognostic (1.54e-2)
unprognostic (2.62e-1)	unprognostic (5.27e-2)	unprognostic (3.23e-2)	unprognostic (1.16e-2)	unprognostic (2.50e-2)	unprognostic (1.48e-3)
unprognostic (2.28e-2)	unprognostic (2.17e-1)	unprognostic (2.68e-2)	unprognostic (2.82e-2)	unprognostic (8.78e-2)	unprognostic (1.20e-1)
unprognostic (9.90e-2)	unprognostic (5.95e-2)	unprognostic (3.12e-1)	unprognostic (3.15e-2)	unprognostic (1.03e-1)	unprognostic (1.92e-1)
unprognostic (5.23e-2)	unprognostic (3.97e-2)	unprognostic (1.81e-2)	unprognostic (1.38e-2)	unprognostic (9.78e-3)	unprognostic (1.34e-2)
unprognostic (2.15e-1)	unprognostic (1.24e-1)	unprognostic (9.15e-2)	unprognostic (5.06e-3)	unprognostic (4.33e-2)	unprognostic (2.86e-3)
unprognostic (3.06e-1)	unprognostic (2.36e-1)	unprognostic (2.51e-1)	unprognostic (7.86e-3)	unprognostic (9.38e-3)	unprognostic (6.55e-3)
unprognostic (2.49e-1)	unprognostic (4.82e-1)	unprognostic (1.18e-1)	unprognostic (6.76e-2)	unprognostic (1.22e-1)	unprognostic (4.20e-2)
unprognostic (1.79e-2)	unprognostic (9.26e-3)	unprognostic (7.81e-2)	unprognostic (5.86e-3)	unprognostic (3.21e-1)	unprognostic (2.63e-1)
unprognostic (7.56e-2)	potential prognostic unfavorable (7.22e-4)	unprognostic (3.11e-2)	unprognostic (8.08e-3)	unprognostic (1.18e-2)	unprognostic (5.86e-2)
potential prognostic unfavorable (3.26e-4)	unprognostic (1.14e-1)	unprognostic (7.66e-2)	unprognostic (2.92e-2)	unprognostic (2.97e-1)	unprognostic (2.67e-2)
unprognostic (1.55e-2)	unprognostic (3.00e-1)	unprognostic (3.32e-1)	unprognostic (1.41e-1)	unprognostic (8.09e-2)	unprognostic (1.53e-1)
potential prognostic unfavorable (4.46e-4)	unprognostic (1.30e-1)	unprognostic (2.54e-3)	unprognostic (1.09e-1)	unprognostic (7.63e-3)	unprognostic (9.13e-2)
unprognostic (5.11e-2)					
potential prognostic unfavorable (2.85e-5)	unprognostic (1.04e-1)	unprognostic (4.90e-2)	unprognostic (2.24e-1)	unprognostic (2.72e-1)	unprognostic (2.64e-2)
unprognostic (3.18e-1)			unprognostic (7.17e-2)	unprognostic (1.09e-2)	unprognostic (4.69e-2)
unprognostic (7.34e-3)	unprognostic (9.45e-2)		unprognostic (2.11e-2)		unprognostic (1.23e-1)
unprognostic (1.40e-2)	unprognostic (5.83e-2)	unprognostic (1.34e-2)	unprognostic (1.07e-1)	potential prognostic favorable (3.87e-4)	unprognostic (7.30e-2)
unprognostic (1.52e-3)	unprognostic (7.55e-2)	unprognostic (2.45e-2)	unprognostic (3.70e-3)	unprognostic (1.88e-1)	potential prognostic unfavorable (2.81e-4)
unprognostic (1.31e-1)	unprognostic (3.06e-2)	unprognostic (5.84e-3)	unprognostic (1.95e-2)	unprognostic (9.01e-2)	unprognostic (2.05e-1)
unprognostic (5.95e-2)			unprognostic (2.19e-2)		unprognostic (1.21e-1)
unprognostic (1.94e-1)	unprognostic (3.87e-1)	unprognostic (1.35e-2)	unprognostic (1.49e-1)	potential prognostic favorable (1.87e-4)	unprognostic (1.35e-1)
unprognostic (8.54e-3)			unprognostic (9.44e-3)		unprognostic (3.76e-1)
unprognostic (3.96e-2)			unprognostic (8.63e-2)		unprognostic (2.82e-1)
unprognostic (3.27e-3)		unprognostic (3.46e-2)	unprognostic (3.48e-2)	unprognostic (5.88e-2)	unprognostic (6.88e-2)
unprognostic (4.10e-3)	unprognostic (4.00e-2)		unprognostic (9.82e-2)		unprognostic (1.11e-1)
unprognostic (1.06e-2)	unprognostic (1.90e-1)	unprognostic (1.72e-2)	unprognostic (2.08e-2)	unprognostic (2.99e-2)	unprognostic (4.33e-2)
unprognostic (3.68e-2)	unprognostic (6.54e-2)	unprognostic (2.02e-2)	unprognostic (5.50e-2)	unprognostic (4.78e-2)	unprognostic (2.20e-1)
unprognostic (3.16e-2)	unprognostic (3.03e-1)	unprognostic (6.26e-3)	unprognostic (2.70e-2)	unprognostic (2.34e-1)	unprognostic (1.37e-1)
unprognostic (1.47e-1)	unprognostic (1.82e-1)	unprognostic (2.31e-2)	unprognostic (2.26e-1)	unprognostic (8.54e-3)	unprognostic (1.20e-1)
unprognostic (2.21e-2)	unprognostic (1.41e-1)	unprognostic (4.90e-2)	unprognostic (2.59e-1)	unprognostic (5.30e-2)	unprognostic (8.32e-2)
unprognostic (5.33e-2)	unprognostic (4.32e-1)	unprognostic (1.50e-1)	unprognostic (1.59e-2)	unprognostic (5.76e-2)	unprognostic (1.17e-1)
unprognostic (3.31e-2)	unprognostic (1.12e-1)	unprognostic (3.58e-2)	unprognostic (1.59e-3)	unprognostic (5.10e-2)	unprognostic (3.74e-2)
			unprognostic (1.73e-1)		unprognostic (6.40e-2)
unprognostic (1.44e-2)	unprognostic (8.40e-2)	unprognostic (1.58e-2)	unprognostic (5.89e-3)	unprognostic (8.97e-2)	unprognostic (7.15e-2)
unprognostic (1.51e-1)	unprognostic (2.04e-1)	unprognostic (1.41e-1)	unprognostic (1.16e-1)	unprognostic (3.32e-2)	unprognostic (6.42e-2)
unprognostic (2.07e-1)	unprognostic (3.06e-2)	unprognostic (5.15e-2)	unprognostic (5.97e-2)	unprognostic (4.42e-3)	unprognostic (1.85e-1)
unprognostic (4.36e-2)	unprognostic (2.79e-1)	unprognostic (5.92e-2)	unprognostic (4.61e-2)	unprognostic (2.80e-1)	unprognostic (1.35e-1)
unprognostic (1.02e-3)	unprognostic (3.57e-1)	unprognostic (7.46e-2)	unprognostic (4.25e-2)	unprognostic (1.27e-3)	unprognostic (1.59e-1)
unprognostic (2.48e-1)	unprognostic (2.87e-1)	unprognostic (1.09e-2)	unprognostic (1.50e-1)	unprognostic (7.09e-3)	unprognostic (1.32e-1)
unprognostic (7.58e-2)	unprognostic (4.21e-1)	unprognostic (1.43e-2)	unprognostic (1.27e-2)	unprognostic (6.30e-3)	unprognostic (1.21e-1)
unprognostic (4.92e-2)	unprognostic (1.09e-1)	unprognostic (1.36e-1)	unprognostic (2.43e-1)	unprognostic (1.93e-1)	unprognostic (3.40e-2)
unprognostic (1.78e-2)	unprognostic (4.58e-1)	unprognostic (7.47e-3)	unprognostic (5.88e-2)	unprognostic (1.20e-1)	unprognostic (1.51e-1)
unprognostic (6.24e-2)	unprognostic (1.35e-1)	unprognostic (1.78e-1)	unprognostic (4.62e-2)	unprognostic (3.87e-2)	unprognostic (2.22e-2)
unprognostic (1.22e-1)	unprognostic (3.10e-1)	unprognostic (1.29e-1)	unprognostic (3.99e-1)	unprognostic (2.56e-1)	unprognostic (1.20e-2)
unprognostic (6.09e-2)	unprognostic (2.47e-1)	unprognostic (4.78e-2)	unprognostic (1.93e-1)	unprognostic (9.27e-2)	unprognostic (2.23e-1)
unprognostic (9.66e-3)	unprognostic (1.57e-1)	unprognostic (8.58e-3)	unprognostic (7.03e-2)	unprognostic (1.23e-1)	unprognostic (1.87e-1)
unprognostic (6.16e-2)	unprognostic (5.57e-1)	unprognostic (4.24e-1)	unprognostic (1.50e-3)	unprognostic (1.15e-2)	unprognostic (8.98e-3)
				unprognostic (3.83e-3)	
unprognostic (2.35e-1)	unprognostic (6.41e-2)	unprognostic (1.25e-1)	unprognostic (4.46e-2)		unprognostic (2.59e-1)
unprognostic (3.81e-2)	unprognostic (7.90e-2)		unprognostic (1.71e-1)	unprognostic (1.08e-3)	unprognostic (5.44e-3)
unprognostic (5.63e-3)	unprognostic (1.51e-2)		unprognostic (2.39e-2)		
unprognostic (1.01e-2)	unprognostic (3.13e-2)	unprognostic (2.88e-1)	unprognostic (9.37e-2)	unprognostic (1.31e-1)	unprognostic (1.49e-1)
unprognostic (1.25e-1)	unprognostic (1.07e-1)	unprognostic (2.05e-2)	unprognostic (1.19e-1)	unprognostic (2.12e-1)	unprognostic (6.53e-3)
unprognostic (6.71e-2)		unprognostic (1.44e-1)	unprognostic (3.97e-1)		potential prognostic unfavorable (8.87e-5)
unprognostic (2.74e-1)	unprognostic (9.14e-2)	unprognostic (1.22e-1)	unprognostic (2.08e-1)	unprognostic (2.02e-1)	unprognostic (7.47e-2)
unprognostic (3.30e-1)	unprognostic (5.34e-2)	unprognostic (1.63e-1)	unprognostic (9.40e-2)	unprognostic (2.67e-1)	unprognostic (9.98e-3)
unprognostic (1.37e-2)	unprognostic (3.76e-1)	unprognostic (8.05e-2)	unprognostic (2.00e-2)	unprognostic (2.82e-1)	unprognostic (4.32e-1)
unprognostic (2.37e-1)		unprognostic (2.01e-2)	unprognostic (1.21e-1)		unprognostic (1.49e-1)
potential prognostic unfavorable (1.28e-5)	unprognostic (3.41e-2)	unprognostic (2.91e-1)	unprognostic (2.15e-1)	unprognostic (3.22e-1)	unprognostic (3.13e-2)
unprognostic (1.01e-1)	unprognostic (6.48e-2)	unprognostic (7.24e-2)	unprognostic (1.01e-1)	unprognostic (3.08e-1)	unprognostic (1.03e-1)
unprognostic (1.38e-3)	unprognostic (6.65e-2)	unprognostic (6.20e-3)	unprognostic (1.26e-2)	unprognostic (2.10e-1)	unprognostic (2.53e-1)
unprognostic (4.72e-2)	unprognostic (2.21e-1)	unprognostic (8.25e-2)	unprognostic (1.13e-1)	unprognostic (3.58e-1)	unprognostic (2.18e-1)
unprognostic (1.44e-1)	unprognostic (5.77e-2)	unprognostic (1.21e-2)	unprognostic (2.16e-1)	unprognostic (2.25e-1)	unprognostic (1.51e-2)
unprognostic (1.09e-2)	unprognostic (2.25e-1)	unprognostic (1.38e-2)	unprognostic (3.10e-1)	unprognostic (4.67e-2)	unprognostic (1.67e-2)
unprognostic (9.67e-2)	unprognostic (6.00e-2)	unprognostic (3.09e-1)	unprognostic (2.29e-1)		unprognostic (5.62e-2)
			unprognostic (2.14e-1)	unprognostic (9.49e-2)	
	unprognostic (1.76e-1)		unprognostic (1.39e-2)		
unprognostic (2.20e-1)		unprognostic (1.65e-2)	unprognostic (2.25e-1)		unprognostic (2.63e-3)
unprognostic (2.05e-1)	unprognostic (1.58e-2)	unprognostic (8.42e-2)	unprognostic (9.05e-2)	unprognostic (1.18e-2)	unprognostic (2.35e-1)
unprognostic (1.72e-1)	unprognostic (1.08e-1)	unprognostic (1.42e-2)	unprognostic (5.60e-2)	unprognostic (1.30e-1)	unprognostic (2.82e-1)
unprognostic (6.91e-2)				unprognostic (8.69e-2)	unprognostic (2.12e-1)
unprognostic (3.46e-1)	unprognostic (6.24e-2)	unprognostic (4.88e-2)	unprognostic (5.24e-3)	unprognostic (1.69e-2)	unprognostic (9.88e-2)
unprognostic (2.14e-1)					
unprognostic (1.43e-2)		unprognostic (1.06e-1)	potential prognostic unfavorable (8.77e-4)		unprognostic (2.09e-1)
			unprognostic (1.76e-2)		
unprognostic (1.97e-2)	unprognostic (2.95e-1)	unprognostic (2.53e-1)	unprognostic (2.58e-1)		unprognostic (3.00e-2)
unprognostic (3.49e-1)	unprognostic (2.66e-1)	unprognostic (4.43e-2)	unprognostic (1.26e-1)	unprognostic (1.72e-1)	unprognostic (9.81e-2)
unprognostic (1.89e-1)	unprognostic (3.76e-2)	unprognostic (2.50e-2)	unprognostic (2.82e-2)	potential prognostic unfavorable (8.39e-4)	unprognostic (1.04e-3)
unprognostic (1.13e-2)		unprognostic (1.07e-1)	unprognostic (2.40e-1)		unprognostic (2.33e-3)
unprognostic (9.39e-2)		unprognostic (1.14e-3)	unprognostic (8.90e-2)	unprognostic (6.49e-2)	unprognostic (8.09e-3)
unprognostic (4.00e-1)	unprognostic (2.47e-1)	unprognostic (1.57e-1)	unprognostic (4.99e-2)	unprognostic (1.91e-1)	unprognostic (8.34e-3)
unprognostic (1.09e-1)	unprognostic (1.11e-1)	unprognostic (1.25e-1)	unprognostic (7.53e-2)	unprognostic (3.16e-2)	unprognostic (1.50e-3)
unprognostic (2.85e-1)	unprognostic (1.41e-1)	unprognostic (2.12e-2)	unprognostic (1.79e-1)		unprognostic (1.75e-1)
unprognostic (2.70e-1)	unprognostic (3.97e-1)	unprognostic (1.15e-1)	unprognostic (3.08e-1)	unprognostic (2.13e-1)	unprognostic (7.18e-2)
unprognostic (8.58e-2)	unprognostic (2.61e-1)	unprognostic (4.80e-1)	unprognostic (6.16e-2)	unprognostic (3.68e-1)	unprognostic (2.46e-2)
potential prognostic unfavorable (8.87e-4)					
unprognostic (2.51e-1)	unprognostic (1.58e-1)	unprognostic (1.26e-2)	unprognostic (6.36e-2)		unprognostic (2.19e-1)
unprognostic (1.17e-1)	unprognostic (2.60e-1)	potential prognostic favorable (9.58e-4)	unprognostic (3.88e-1)	unprognostic (3.10e-1)	unprognostic (1.11e-1)
unprognostic (9.62e-2)			unprognostic (1.06e-1)	unprognostic (2.89e-1)	unprognostic (4.13e-3)
unprognostic (2.10e-2)	unprognostic (6.89e-2)	unprognostic (5.96e-2)	unprognostic (1.27e-1)	unprognostic (4.05e-2)	unprognostic (3.37e-2)
unprognostic (5.57e-3)	unprognostic (6.09e-2)	unprognostic (1.51e-1)	unprognostic (2.41e-2)	unprognostic (8.04e-2)	unprognostic (1.13e-1)
unprognostic (2.19e-1)	unprognostic (1.75e-1)	unprognostic (8.50e-2)	unprognostic (5.62e-3)	unprognostic (2.26e-2)	unprognostic (2.49e-2)
unprognostic (1.13e-1)	unprognostic (2.59e-2)	unprognostic (2.34e-1)	unprognostic (2.38e-1)	unprognostic (1.07e-1)	unprognostic (2.33e-1)
unprognostic (1.65e-1)	unprognostic (4.10e-1)	unprognostic (2.13e-1)	unprognostic (2.83e-1)	unprognostic (2.80e-1)	unprognostic (9.30e-2)
unprognostic (3.66e-2)			unprognostic (1.23e-1)		unprognostic (5.13e-2)
unprognostic (5.83e-3)	unprognostic (4.66e-1)	unprognostic (4.50e-2)	unprognostic (2.27e-1)	unprognostic (1.45e-1)	unprognostic (1.96e-2)
unprognostic (3.81e-1)	unprognostic (4.84e-2)	potential prognostic unfavorable (9.71e-4)	unprognostic (5.66e-2)	unprognostic (2.25e-1)	unprognostic (4.54e-2)
unprognostic (1.83e-2)	unprognostic (7.50e-2)	unprognostic (9.71e-2)	unprognostic (5.82e-3)	unprognostic (8.95e-3)	unprognostic (8.24e-2)
unprognostic (1.50e-1)	unprognostic (1.44e-1)	unprognostic (1.88e-2)	unprognostic (2.01e-2)	unprognostic (9.85e-2)	unprognostic (3.02e-2)
potential prognostic unfavorable (1.34e-5)	unprognostic (1.17e-2)	unprognostic (3.63e-2)	unprognostic (2.96e-1)	unprognostic (2.54e-1)	unprognostic (1.16e-1)
unprognostic (9.23e-2)	unprognostic (2.83e-1)	unprognostic (1.06e-1)	unprognostic (3.80e-2)	unprognostic (2.13e-1)	unprognostic (3.76e-1)
unprognostic (2.00e-1)	unprognostic (1.63e-1)	unprognostic (3.63e-3)	unprognostic (8.27e-2)	unprognostic (2.22e-1)	unprognostic (7.32e-3)
unprognostic (1.58e-1)	unprognostic (1.21e-1)	unprognostic (2.22e-3)	unprognostic (1.97e-1)	unprognostic (3.76e-1)	unprognostic (8.77e-2)
unprognostic (6.55e-3)	unprognostic (1.32e-1)	unprognostic (1.15e-1)	unprognostic (1.14e-1)	unprognostic (3.01e-1)	unprognostic (6.59e-2)
unprognostic (1.91e-2)	unprognostic (3.05e-3)	unprognostic (8.93e-2)	unprognostic (8.37e-2)	unprognostic (4.94e-2)	unprognostic (1.45e-2)
unprognostic (7.69e-2)	unprognostic (9.11e-2)		unprognostic (1.77e-2)		unprognostic (3.25e-2)
unprognostic (2.26e-1)	unprognostic (7.23e-2)	unprognostic (1.43e-2)	unprognostic (1.12e-1)	unprognostic (4.21e-1)	unprognostic (4.62e-3)
unprognostic (2.39e-2)			unprognostic (1.39e-1)		unprognostic (5.72e-2)
unprognostic (9.58e-2)	unprognostic (3.72e-1)	unprognostic (8.67e-2)	unprognostic (4.14e-3)	unprognostic (1.92e-2)	unprognostic (2.46e-2)
unprognostic (6.39e-2)	unprognostic (1.48e-3)	unprognostic (2.23e-1)	unprognostic (3.54e-1)	unprognostic (8.42e-2)	unprognostic (3.52e-1)
potential prognostic unfavorable (8.15e-5)	unprognostic (2.29e-1)	unprognostic (1.96e-2)	unprognostic (8.07e-2)	unprognostic (4.44e-3)	unprognostic (1.64e-1)
unprognostic (5.94e-2)	unprognostic (2.06e-1)	unprognostic (4.86e-2)	unprognostic (1.44e-3)	unprognostic (9.04e-2)	potential prognostic unfavorable (3.90e-4)
potential prognostic favorable (6.76e-6)	unprognostic (2.62e-1)	unprognostic (8.35e-2)	unprognostic (8.33e-2)	unprognostic (1.26e-1)	unprognostic (9.82e-2)
unprognostic (4.34e-3)	unprognostic (8.67e-2)	unprognostic (1.21e-1)	unprognostic (5.59e-2)	unprognostic (1.12e-1)	unprognostic (1.49e-1)
unprognostic (1.66e-1)	unprognostic (7.20e-2)	unprognostic (2.98e-1)	unprognostic (2.56e-1)	unprognostic (6.22e-2)	unprognostic (2.22e-1)
				unprognostic (1.94e-2)	
unprognostic (3.66e-2)	unprognostic (1.72e-1)	unprognostic (3.57e-2)	unprognostic (5.69e-3)	unprognostic (3.57e-2)	unprognostic (2.37e-1)

unprognostic (4.52e-2)	unprognostic (1.59e-1)	unprognostic (5.70e-2)	unprognostic (1.48e-2)	unprognostic (6.21e-2)	unprognostic (3.94e-3)
unprognostic (2.17e-3)	unprognostic (3.68e-1)	unprognostic (2.10e-1)	unprognostic (1.25e-1)	unprognostic (1.11e-1)	unprognostic (2.60e-1)
unprognostic (8.11e-3)		unprognostic (1.58e-2)	unprognostic (4.29e-3)	unprognostic (4.30e-2)	unprognostic (1.21e-1)
unprognostic (3.67e-3)	unprognostic (1.58e-1)	unprognostic (7.25e-2)	unprognostic (1.48e-1)	unprognostic (1.27e-1)	unprognostic (2.15e-1)
unprognostic (3.42e-3)	unprognostic (6.12e-2)	unprognostic (4.72e-2)	unprognostic (1.18e-2)	unprognostic (3.90e-2)	unprognostic (3.01e-1)
unprognostic (7.64e-3)	unprognostic (1.24e-1)	unprognostic (9.42e-2)	unprognostic (7.68e-2)	unprognostic (4.41e-1)	unprognostic (1.71e-1)
potential prognostic unfavorable (5.49e-5)	unprognostic (2.30e-2)	potential prognostic unfavorable (7.40e-4)	unprognostic (3.62e-3)	unprognostic (3.95e-1)	unprognostic (1.01e-1)
unprognostic (1.59e-2)	unprognostic (2.66e-1)	unprognostic (3.54e-1)	unprognostic (5.74e-2)	unprognostic (1.33e-1)	unprognostic (2.57e-3)
unprognostic (1.72e-3)	unprognostic (1.40e-3)	unprognostic (1.55e-1)	unprognostic (4.45e-2)	unprognostic (1.39e-1)	unprognostic (1.32e-2)
unprognostic (2.38e-2)	unprognostic (1.99e-1)	unprognostic (2.94e-2)	unprognostic (7.93e-2)	unprognostic (5.23e-2)	unprognostic (1.82e-2)
unprognostic (8.79e-2)	unprognostic (7.72e-2)	unprognostic (4.22e-2)	unprognostic (6.63e-2)	unprognostic (2.64e-2)	unprognostic (2.47e-1)
unprognostic (8.80e-3)	unprognostic (6.47e-2)	unprognostic (7.08e-2)	unprognostic (6.62e-2)	potential prognostic favorable (5.77e-5)	unprognostic (3.14e-1)
unprognostic (2.27e-2)	unprognostic (7.14e-2)	unprognostic (2.44e-2)	unprognostic (2.27e-1)	unprognostic (2.04e-2)	unprognostic (1.74e-1)
unprognostic (3.09e-2)	unprognostic (8.77e-3)	unprognostic (6.57e-2)	unprognostic (3.45e-1)		unprognostic (1.23e-2)
unprognostic (5.03e-3)	unprognostic (2.26e-1)	unprognostic (1.21e-1)	unprognostic (5.45e-2)	unprognostic (5.65e-2)	unprognostic (3.54e-1)
potential prognostic unfavorable (1.10e-4)	unprognostic (2.03e-1)	unprognostic (2.38e-2)	unprognostic (3.33e-1)	unprognostic (1.78e-1)	unprognostic (2.29e-2)
unprognostic (9.83e-3)	unprognostic (1.47e-1)	unprognostic (3.84e-2)	unprognostic (5.30e-2)	unprognostic (8.51e-2)	unprognostic (1.70e-1)
unprognostic (3.51e-2)	unprognostic (2.22e-2)	unprognostic (2.86e-2)	unprognostic (4.10e-1)	unprognostic (5.16e-2)	unprognostic (2.81e-1)
unprognostic (1.21e-2)	unprognostic (1.18e-1)	unprognostic (2.68e-1)	unprognostic (3.15e-2)	unprognostic (1.68e-1)	unprognostic (4.14e-3)
unprognostic (8.42e-2)	unprognostic (7.85e-2)	unprognostic (3.80e-2)	unprognostic (6.45e-2)	unprognostic (1.90e-2)	unprognostic (4.23e-2)
unprognostic (3.80e-2)	unprognostic (2.85e-2)	unprognostic (3.26e-2)	unprognostic (1.37e-1)	unprognostic (8.32e-2)	unprognostic (1.05e-3)
unprognostic (1.43e-1)	unprognostic (2.19e-1)	unprognostic (2.53e-1)	unprognostic (3.86e-2)	unprognostic (4.74e-3)	unprognostic (2.35e-3)
unprognostic (4.97e-2)	unprognostic (4.04e-2)	unprognostic (1.70e-1)	unprognostic (9.80e-2)	unprognostic (7.73e-2)	unprognostic (3.67e-3)
unprognostic (9.94e-2)	unprognostic (2.24e-2)	unprognostic (2.65e-1)	unprognostic (1.09e-1)	unprognostic (1.68e-3)	unprognostic (3.51e-2)
unprognostic (1.42e-2)	unprognostic (1.60e-1)	unprognostic (1.18e-1)	unprognostic (3.24e-1)	unprognostic (2.35e-1)	unprognostic (8.77e-2)
			unprognostic (1.82e-1)	unprognostic (3.12e-1)	unprognostic (8.48e-2)
potential prognostic unfavorable (4.42e-5)	unprognostic (1.11e-1)	unprognostic (3.51e-2)	unprognostic (1.84e-1)	unprognostic (1.92e-2)	unprognostic (4.08e-2)
unprognostic (1.93e-3)	unprognostic (1.20e-1)	unprognostic (1.65e-1)	unprognostic (4.03e-2)	unprognostic (3.04e-1)	unprognostic (3.67e-2)
unprognostic (1.66e-1)	unprognostic (1.20e-1)	unprognostic (6.53e-2)	unprognostic (8.27e-2)	unprognostic (2.10e-2)	unprognostic (2.64e-1)
unprognostic (1.06e-2)	unprognostic (7.60e-2)	unprognostic (1.73e-2)	unprognostic (5.22e-2)	unprognostic (4.28e-2)	unprognostic (7.65e-3)
unprognostic (3.02e-1)	unprognostic (2.87e-1)	unprognostic (4.43e-1)	unprognostic (7.41e-2)	unprognostic (2.51e-1)	unprognostic (2.24e-3)
unprognostic (1.33e-2)	unprognostic (2.53e-1)	unprognostic (3.98e-3)	potential prognostic unfavorable (7.10e-4)		unprognostic (1.79e-1)
unprognostic (2.78e-1)	unprognostic (1.02e-1)	unprognostic (1.13e-2)	unprognostic (2.27e-2)	unprognostic (4.57e-2)	unprognostic (1.57e-1)
unprognostic (2.71e-1)	unprognostic (2.64e-1)	unprognostic (7.10e-2)	unprognostic (8.68e-2)	unprognostic (2.03e-1)	potential prognostic unfavorable (7.45e-4)
unprognostic (4.28e-2)	unprognostic (9.51e-2)	unprognostic (1.93e-1)	unprognostic (1.21e-2)	unprognostic (7.46e-2)	unprognostic (7.70e-2)
potential prognostic unfavorable (6.28e-4)	unprognostic (5.19e-2)	unprognostic (1.25e-1)	unprognostic (1.82e-1)	unprognostic (1.05e-1)	unprognostic (9.28e-2)
unprognostic (1.23e-3)	unprognostic (1.44e-1)	unprognostic (2.04e-2)	unprognostic (1.85e-1)	unprognostic (4.59e-2)	unprognostic (1.69e-2)
unprognostic (1.58e-1)	unprognostic (1.41e-1)		unprognostic (5.19e-3)	unprognostic (2.25e-1)	unprognostic (1.49e-3)
unprognostic (2.23e-2)	unprognostic (3.63e-1)	unprognostic (4.35e-2)	unprognostic (4.13e-2)	unprognostic (2.08e-3)	unprognostic (6.51e-2)
unprognostic (5.34e-2)	unprognostic (3.05e-1)	unprognostic (1.78e-1)	unprognostic (1.65e-1)	unprognostic (2.29e-3)	unprognostic (1.62e-1)
unprognostic (3.02e-2)	unprognostic (1.66e-1)	unprognostic (1.81e-2)	unprognostic (1.42e-2)	unprognostic (4.14e-1)	unprognostic (5.39e-2)
unprognostic (7.71e-2)	unprognostic (8.21e-2)	unprognostic (2.88e-2)	unprognostic (2.58e-1)	unprognostic (1.62e-1)	unprognostic (2.01e-3)
unprognostic (7.74e-2)	unprognostic (7.37e-2)	unprognostic (1.50e-2)	unprognostic (6.00e-2)	unprognostic (4.79e-2)	unprognostic (8.29e-2)
unprognostic (1.84e-1)	unprognostic (2.81e-2)	unprognostic (3.65e-1)	unprognostic (1.42e-2)		unprognostic (6.94e-3)
unprognostic (6.78e-2)	unprognostic (2.73e-1)	unprognostic (1.99e-1)	unprognostic (1.46e-2)	unprognostic (9.96e-3)	unprognostic (1.27e-1)
unprognostic (6.53e-2)	unprognostic (1.51e-1)	unprognostic (3.21e-1)	unprognostic (2.17e-2)	unprognostic (1.67e-2)	unprognostic (2.39e-2)
	unprognostic (1.44e-1)				
unprognostic (1.25e-1)		unprognostic (2.36e-1)	unprognostic (5.80e-3)	unprognostic (1.80e-1)	unprognostic (8.12e-2)
unprognostic (4.43e-2)	unprognostic (1.49e-1)	unprognostic (1.87e-3)	unprognostic (3.99e-1)	unprognostic (1.28e-1)	unprognostic (1.26e-1)
potential prognostic unfavorable (5.13e-4)	unprognostic (1.26e-2)	unprognostic (1.61e-1)	unprognostic (3.74e-1)	unprognostic (1.46e-2)	unprognostic (1.23e-1)
unprognostic (1.50e-1)	unprognostic (1.40e-1)	unprognostic (1.56e-2)	unprognostic (1.78e-1)	unprognostic (2.07e-2)	unprognostic (8.31e-2)
unprognostic (4.00e-2)	unprognostic (2.44e-1)	unprognostic (3.75e-2)	unprognostic (1.42e-1)	unprognostic (1.47e-1)	unprognostic (9.29e-2)
unprognostic (6.85e-2)	unprognostic (2.27e-1)	unprognostic (4.12e-1)	unprognostic (8.89e-2)	unprognostic (7.79e-2)	unprognostic (2.66e-2)
unprognostic (7.54e-3)	unprognostic (5.02e-1)	unprognostic (1.19e-2)	unprognostic (6.72e-2)	unprognostic (3.56e-1)	unprognostic (1.09e-1)
unprognostic (9.03e-2)	unprognostic (2.05e-2)	unprognostic (7.12e-2)	unprognostic (2.06e-1)	unprognostic (1.89e-3)	unprognostic (6.39e-2)
unprognostic (2.15e-3)	unprognostic (1.57e-1)	unprognostic (1.28e-2)	unprognostic (5.45e-2)	unprognostic (2.22e-1)	unprognostic (1.54e-1)
unprognostic (1.38e-1)	unprognostic (4.38e-2)	unprognostic (4.10e-3)	unprognostic (1.35e-2)	unprognostic (3.68e-1)	unprognostic (9.89e-2)
unprognostic (1.05e-1)	unprognostic (9.91e-2)	unprognostic (2.67e-1)	potential prognostic favorable (1.80e-5)		unprognostic (6.66e-3)
unprognostic (1.20e-1)	unprognostic (3.30e-1)	unprognostic (1.44e-1)	unprognostic (2.14e-1)	unprognostic (2.38e-1)	unprognostic (1.96e-1)
unprognostic (1.94e-1)	unprognostic (1.70e-1)	unprognostic (7.55e-2)	unprognostic (7.18e-2)	unprognostic (8.07e-2)	unprognostic (5.28e-2)
unprognostic (1.36e-2)	unprognostic (1.94e-1)	unprognostic (1.40e-1)	unprognostic (5.08e-2)	potential prognostic unfavorable (6.73e-4)	unprognostic (8.81e-2)
potential prognostic unfavorable (2.86e-5)	unprognostic (3.78e-2)		unprognostic (1.51e-1)	unprognostic (1.01e-1)	
unprognostic (2.14e-2)					
unprognostic (4.21e-1)	unprognostic (1.21e-1)	unprognostic (2.77e-2)	unprognostic (2.69e-2)	unprognostic (6.35e-2)	unprognostic (3.73e-2)
unprognostic (7.81e-2)	unprognostic (1.77e-1)	unprognostic (4.83e-3)	unprognostic (9.63e-2)	unprognostic (1.18e-1)	unprognostic (2.83e-2)
unprognostic (6.21e-3)	unprognostic (8.92e-2)	unprognostic (2.19e-2)	unprognostic (1.72e-2)	unprognostic (5.51e-3)	unprognostic (5.12e-2)
unprognostic (9.73e-2)	unprognostic (3.18e-1)	unprognostic (2.50e-1)	unprognostic (3.09e-2)	unprognostic (2.55e-3)	unprognostic (6.59e-2)
unprognostic (1.88e-1)		unprognostic (1.29e-1)	unprognostic (7.93e-2)		unprognostic (8.04e-2)
unprognostic (1.55e-2)	unprognostic (2.54e-1)	unprognostic (2.04e-1)	unprognostic (1.14e-2)	unprognostic (5.71e-2)	unprognostic (6.32e-3)
unprognostic (2.91e-1)	unprognostic (2.05e-2)	unprognostic (3.28e-1)	unprognostic (2.46e-3)	unprognostic (7.39e-2)	unprognostic (2.82e-2)
unprognostic (9.11e-2)	unprognostic (2.52e-1)	unprognostic (1.96e-2)	unprognostic (7.69e-2)	unprognostic (4.69e-2)	unprognostic (3.01e-3)
unprognostic (6.14e-2)	unprognostic (1.82e-1)	unprognostic (8.77e-2)	unprognostic (2.03e-2)	unprognostic (2.05e-1)	unprognostic (1.68e-1)
potential prognostic unfavorable (3.20e-5)	unprognostic (3.77e-1)	unprognostic (2.69e-2)	unprognostic (1.39e-1)	unprognostic (2.07e-1)	unprognostic (1.92e-1)
unprognostic (2.39e-1)	unprognostic (4.57e-2)	unprognostic (1.11e-2)	unprognostic (3.59e-2)	unprognostic (1.45e-1)	unprognostic (4.65e-2)
unprognostic (1.30e-3)	unprognostic (1.88e-1)	unprognostic (4.54e-2)	unprognostic (1.92e-1)	unprognostic (2.48e-1)	unprognostic (3.25e-2)
unprognostic (2.96e-3)	unprognostic (2.92e-1)	unprognostic (1.41e-2)	unprognostic (9.98e-2)	unprognostic (4.56e-2)	unprognostic (1.28e-2)
unprognostic (4.82e-2)	unprognostic (9.24e-2)	unprognostic (7.37e-2)	unprognostic (6.89e-3)	unprognostic (1.65e-1)	unprognostic (1.52e-2)
unprognostic (1.06e-1)	unprognostic (2.23e-1)	unprognostic (4.42e-1)	unprognostic (1.11e-3)	unprognostic (3.00e-1)	unprognostic (6.47e-2)
unprognostic (1.81e-2)	unprognostic (3.87e-1)	unprognostic (1.80e-3)	unprognostic (7.78e-2)	unprognostic (1.86e-1)	unprognostic (1.40e-2)
unprognostic (9.45e-2)	unprognostic (1.81e-1)	unprognostic (3.16e-2)	unprognostic (8.25e-2)	unprognostic (3.31e-2)	unprognostic (1.89e-1)
unprognostic (1.41e-2)	unprognostic (8.47e-3)	unprognostic (1.49e-1)	potential prognostic favorable (2.60e-4)	unprognostic (1.98e-2)	unprognostic (1.86e-2)
unprognostic (1.36e-2)	unprognostic (3.55e-1)	unprognostic (4.15e-2)	unprognostic (2.37e-1)	unprognostic (4.90e-2)	unprognostic (3.47e-2)
unprognostic (2.20e-1)	unprognostic (5.13e-3)	unprognostic (3.08e-2)	unprognostic (6.34e-2)	unprognostic (7.20e-3)	unprognostic (1.18e-2)
unprognostic (1.49e-1)	unprognostic (1.94e-1)	unprognostic (4.97e-2)	unprognostic (9.58e-2)	unprognostic (3.70e-2)	unprognostic (3.98e-3)
validated prognostic unfavorable (3.59e-6)	unprognostic (8.02e-3)	unprognostic (8.21e-2)	unprognostic (8.80e-2)	unprognostic (1.58e-1)	unprognostic (1.28e-1)
unprognostic (1.05e-1)	unprognostic (1.77e-1)	unprognostic (9.51e-2)	unprognostic (5.27e-2)	unprognostic (1.11e-2)	unprognostic (6.22e-2)
unprognostic (1.97e-3)	unprognostic (1.35e-2)	unprognostic (2.53e-1)	unprognostic (1.57e-1)	unprognostic (8.89e-2)	unprognostic (2.78e-1)
				unprognostic (1.46e-1)	
	unprognostic (1.06e-1)				
unprognostic (3.98e-3)			unprognostic (4.18e-2)	unprognostic (3.42e-2)	unprognostic (4.79e-2)
unprognostic (4.52e-2)	unprognostic (2.01e-1)	unprognostic (3.57e-1)	unprognostic (1.46e-1)	unprognostic (2.78e-1)	unprognostic (9.28e-2)
unprognostic (2.81e-1)	unprognostic (1.15e-1)		unprognostic (1.03e-1)		unprognostic (4.60e-2)
unprognostic (4.18e-2)	unprognostic (9.41e-2)	potential prognostic favorable (7.06e-4)	unprognostic (6.26e-2)	unprognostic (1.90e-1)	unprognostic (2.42e-1)
unprognostic (4.51e-3)	unprognostic (1.47e-1)	unprognostic (3.16e-1)	unprognostic (1.34e-1)	unprognostic (4.23e-1)	unprognostic (6.06e-2)
	unprognostic (9.01e-3)		unprognostic (8.23e-2)	unprognostic (1.65e-1)	unprognostic (1.04e-2)
unprognostic (3.57e-2)	unprognostic (8.96e-2)	unprognostic (1.13e-2)	unprognostic (1.27e-1)		unprognostic (5.69e-3)
unprognostic (1.27e-2)	unprognostic (1.74e-1)	unprognostic (8.40e-3)	unprognostic (5.17e-2)	unprognostic (2.47e-1)	unprognostic (2.98e-1)
unprognostic (1.40e-2)	unprognostic (4.80e-2)	unprognostic (4.13e-2)	unprognostic (1.13e-1)	unprognostic (5.55e-2)	potential prognostic unfavorable (1.44e-5)
unprognostic (2.62e-3)	unprognostic (6.53e-2)	unprognostic (4.15e-2)	unprognostic (9.69e-2)	unprognostic (8.32e-2)	unprognostic (9.09e-2)
unprognostic (1.67e-2)		unprognostic (1.04e-1)	unprognostic (1.58e-1)	unprognostic (1.80e-1)	unprognostic (2.40e-1)
potential prognostic unfavorable (1.13e-4)				unprognostic (8.57e-2)	unprognostic (2.17e-1)
unprognostic (1.07e-2)	unprognostic (2.84e-1)	unprognostic (9.53e-2)	unprognostic (1.63e-2)	unprognostic (4.36e-2)	unprognostic (8.00e-3)
unprognostic (2.79e-2)	unprognostic (1.52e-1)	unprognostic (2.33e-1)	unprognostic (9.17e-2)		unprognostic (5.56e-2)
unprognostic (6.22e-2)			unprognostic (1.27e-1)		unprognostic (4.77e-3)
unprognostic (4.38e-1)	unprognostic (1.08e-1)	unprognostic (6.00e-3)	unprognostic (8.08e-2)	unprognostic (1.06e-1)	unprognostic (9.13e-3)
unprognostic (3.11e-1)					
unprognostic (3.49e-3)			unprognostic (6.02e-2)		
unprognostic (4.02e-2)					
		unprognostic (2.49e-1)	unprognostic (9.84e-2)		
unprognostic (4.70e-2)	unprognostic (3.07e-1)	unprognostic (1.94e-2)	unprognostic (6.37e-3)	unprognostic (4.49e-2)	unprognostic (8.58e-2)
unprognostic (1.22e-2)	unprognostic (9.63e-2)	unprognostic (4.12e-2)	unprognostic (3.96e-1)	unprognostic (7.59e-2)	unprognostic (9.97e-2)
unprognostic (7.06e-3)	unprognostic (1.46e-1)	unprognostic (4.64e-2)	unprognostic (5.81e-2)	unprognostic (5.11e-2)	unprognostic (1.02e-3)
unprognostic (9.18e-2)	unprognostic (1.66e-1)	unprognostic (1.43e-2)	unprognostic (1.24e-1)	unprognostic (4.94e-2)	unprognostic (7.98e-2)
unprognostic (2.98e-1)	unprognostic (3.93e-1)	unprognostic (5.75e-2)	unprognostic (7.93e-3)	unprognostic (8.22e-2)	unprognostic (3.47e-2)
unprognostic (1.16e-1)	unprognostic (1.17e-1)	unprognostic (2.44e-1)	unprognostic (1.91e-1)	unprognostic (1.34e-1)	potential prognostic unfavorable (1.01e-4)
unprognostic (7.08e-2)	unprognostic (1.20e-1)	unprognostic (6.73e-3)	unprognostic (4.78e-3)	unprognostic (3.18e-1)	unprognostic (5.29e-2)
unprognostic (4.24e-2)	unprognostic (5.51e-2)	potential prognostic favorable (5.75e-4)	unprognostic (2.08e-1)	unprognostic (1.18e-1)	unprognostic (4.17e-1)
unprognostic (1.66e-1)			unprognostic (1.00e-1)		unprognostic (1.44e-2)
unprognostic (2.60e-1)	unprognostic (2.77e-1)	unprognostic (1.51e-1)	unprognostic (4.95e-3)	unprognostic (2.46e-1)	unprognostic (3.13e-3)

			unprognostic (1.99e-1)		unprognostic (1.21e-1)
unprognostic (2.14e-1)			unprognostic (8.24e-2)		
unprognostic (2.11e-2)			unprognostic (1.53e-1)		unprognostic (8.99e-2)
unprognostic (1.17e-1)	unprognostic (3.55e-1)	unprognostic (1.96e-1)	unprognostic (4.85e-3)	unprognostic (2.15e-1)	unprognostic (1.60e-1)
unprognostic (5.43e-1)	unprognostic (6.09e-2)	unprognostic (6.39e-2)	unprognostic (1.24e-1)	unprognostic (4.88e-2)	unprognostic (1.89e-2)
unprognostic (3.68e-3)		unprognostic (3.19e-1)	unprognostic (6.72e-2)	unprognostic (1.47e-1)	unprognostic (1.72e-2)
unprognostic (1.25e-1)	unprognostic (4.72e-2)	unprognostic (3.79e-2)	unprognostic (3.13e-1)	unprognostic (2.03e-1)	unprognostic (1.32e-1)
unprognostic (2.08e-1)		unprognostic (2.57e-1)	unprognostic (2.73e-1)	unprognostic (2.79e-1)	unprognostic (4.09e-2)
unprognostic (1.43e-2)	unprognostic (2.93e-1)	unprognostic (6.09e-3)	unprognostic (7.17e-3)	unprognostic (2.01e-1)	unprognostic (2.05e-2)
	unprognostic (4.72e-3)				
	unprognostic (1.06e-1)		unprognostic (6.18e-2)		unprognostic (1.44e-2)
		unprognostic (5.18e-2)	unprognostic (1.15e-1)		
unprognostic (2.01e-1)	unprognostic (4.15e-2)	unprognostic (3.07e-1)	unprognostic (1.03e-1)	unprognostic (3.26e-1)	unprognostic (1.61e-1)
	unprognostic (8.23e-2)	unprognostic (2.45e-1)	unprognostic (1.92e-3)		
unprognostic (3.14e-2)		unprognostic (1.22e-1)	unprognostic (1.69e-1)	unprognostic (1.26e-1)	unprognostic (5.20e-2)
unprognostic (1.39e-2)	unprognostic (3.12e-3)	unprognostic (1.84e-2)	unprognostic (1.09e-1)	unprognostic (1.03e-1)	unprognostic (3.20e-2)
unprognostic (4.32e-1)	unprognostic (2.61e-1)	unprognostic (6.10e-2)	unprognostic (8.84e-2)	unprognostic (1.11e-1)	unprognostic (1.18e-2)
unprognostic (2.17e-1)	unprognostic (2.43e-1)	unprognostic (2.47e-2)	unprognostic (4.31e-2)	unprognostic (8.25e-2)	unprognostic (2.70e-1)
			unprognostic (1.96e-1)		
unprognostic (5.14e-3)	unprognostic (1.36e-1)	unprognostic (1.52e-1)	unprognostic (7.29e-2)	unprognostic (2.25e-1)	unprognostic (5.47e-2)
unprognostic (3.56e-2)	unprognostic (2.71e-2)	unprognostic (1.93e-2)	unprognostic (1.32e-2)	unprognostic (7.90e-2)	unprognostic (7.14e-2)
unprognostic (4.59e-2)	unprognostic (3.49e-1)	unprognostic (5.70e-2)	unprognostic (6.67e-2)	unprognostic (4.31e-3)	unprognostic (2.81e-1)
unprognostic (1.33e-1)	unprognostic (2.69e-1)	unprognostic (1.90e-1)	unprognostic (2.60e-1)	unprognostic (8.32e-3)	unprognostic (1.12e-1)
unprognostic (3.94e-1)	unprognostic (6.79e-2)	unprognostic (9.77e-2)	unprognostic (7.55e-2)	unprognostic (1.14e-2)	unprognostic (8.64e-2)
unprognostic (4.04e-1)	unprognostic (9.54e-2)	unprognostic (1.40e-1)	unprognostic (1.76e-1)	unprognostic (1.88e-2)	unprognostic (1.23e-1)
unprognostic (2.92e-2)	unprognostic (7.15e-2)	unprognostic (1.69e-1)	unprognostic (9.26e-2)	unprognostic (6.09e-2)	unprognostic (4.04e-2)
unprognostic (2.87e-3)	unprognostic (9.60e-2)	unprognostic (5.34e-2)	unprognostic (6.27e-2)	unprognostic (2.35e-2)	unprognostic (3.50e-1)
unprognostic (1.50e-3)	unprognostic (1.49e-2)	unprognostic (5.79e-2)	unprognostic (1.36e-2)	unprognostic (8.11e-3)	unprognostic (1.84e-2)
unprognostic (7.38e-3)	unprognostic (1.32e-1)	unprognostic (2.02e-3)	unprognostic (5.31e-2)	unprognostic (2.04e-1)	unprognostic (7.64e-2)
unprognostic (5.62e-2)	unprognostic (2.37e-2)	unprognostic (1.66e-2)	unprognostic (2.86e-1)	unprognostic (7.07e-2)	unprognostic (1.53e-1)
unprognostic (3.08e-1)	unprognostic (3.18e-1)	potential prognostic unfavorable (8.01e-4)	unprognostic (9.54e-2)	unprognostic (1.22e-1)	unprognostic (3.89e-2)
unprognostic (3.13e-2)					
unprognostic (1.00e-1)	unprognostic (1.56e-1)	unprognostic (1.90e-1)	unprognostic (2.31e-2)	unprognostic (3.01e-2)	potential prognostic unfavorable (5.15e-4)
unprognostic (5.35e-2)	unprognostic (1.52e-1)	unprognostic (1.81e-1)	unprognostic (3.14e-3)	unprognostic (1.07e-2)	unprognostic (3.78e-3)
unprognostic (9.10e-2)	unprognostic (2.59e-2)	unprognostic (1.03e-2)	unprognostic (1.80e-1)	unprognostic (8.68e-3)	unprognostic (5.32e-2)
unprognostic (2.65e-3)	unprognostic (1.24e-1)	unprognostic (2.28e-1)	unprognostic (2.44e-2)	unprognostic (1.47e-1)	unprognostic (6.24e-2)
unprognostic (6.32e-3)	unprognostic (8.19e-2)	unprognostic (6.20e-2)	unprognostic (1.85e-2)	unprognostic (1.86e-1)	unprognostic (1.35e-1)
unprognostic (1.55e-3)	unprognostic (5.94e-2)	unprognostic (2.46e-2)	unprognostic (2.38e-1)	unprognostic (6.09e-2)	unprognostic (2.02e-1)
			unprognostic (1.40e-1)		
unprognostic (1.16e-2)	unprognostic (2.54e-1)	unprognostic (3.98e-2)	unprognostic (1.26e-2)	unprognostic (1.28e-1)	unprognostic (2.15e-1)
unprognostic (2.97e-2)	unprognostic (2.84e-1)	unprognostic (1.29e-2)	unprognostic (1.20e-2)	unprognostic (5.71e-2)	unprognostic (2.20e-1)
unprognostic (6.25e-2)	unprognostic (3.06e-1)	unprognostic (2.28e-1)	unprognostic (4.77e-2)	unprognostic (7.10e-2)	unprognostic (3.27e-1)
unprognostic (1.32e-1)	unprognostic (2.97e-1)	unprognostic (1.14e-2)	unprognostic (8.27e-2)	unprognostic (1.04e-2)	unprognostic (2.11e-1)
unprognostic (8.30e-2)	unprognostic (2.61e-1)	unprognostic (7.75e-2)	unprognostic (3.28e-3)	unprognostic (2.79e-2)	unprognostic (2.66e-2)
unprognostic (2.66e-1)	unprognostic (6.28e-2)	unprognostic (1.10e-2)	unprognostic (1.94e-2)	unprognostic (3.59e-1)	unprognostic (8.28e-2)
unprognostic (2.05e-2)			potential prognostic favorable (1.43e-4)		unprognostic (1.13e-2)
unprognostic (1.01e-2)	unprognostic (2.24e-1)	unprognostic (2.20e-1)	unprognostic (1.56e-2)	potential prognostic favorable (2.33e-4)	unprognostic (1.70e-1)
unprognostic (1.50e-3)	unprognostic (1.59e-1)	unprognostic (1.54e-2)	unprognostic (2.65e-1)	unprognostic (3.40e-1)	unprognostic (1.21e-2)
unprognostic (4.67e-2)	unprognostic (7.33e-2)	unprognostic (6.47e-3)	unprognostic (1.68e-1)	unprognostic (2.75e-2)	unprognostic (5.11e-3)
unprognostic (1.94e-2)	unprognostic (3.92e-1)	unprognostic (1.72e-2)	unprognostic (2.01e-1)	unprognostic (1.22e-1)	unprognostic (1.68e-2)
unprognostic (1.79e-2)	unprognostic (1.36e-1)	unprognostic (3.62e-3)	unprognostic (1.39e-1)	unprognostic (2.02e-1)	unprognostic (1.52e-1)
unprognostic (2.14e-1)	unprognostic (1.17e-1)	unprognostic (4.33e-2)	unprognostic (2.18e-1)	unprognostic (5.20e-2)	unprognostic (1.18e-2)
validated prognostic unfavorable (2.27e-5)	unprognostic (1.48e-2)	unprognostic (2.27e-1)	unprognostic (6.81e-2)	potential prognostic favorable (8.80e-4)	unprognostic (1.84e-1)
unprognostic (1.04e-2)	unprognostic (3.64e-1)	unprognostic (3.27e-2)	unprognostic (2.77e-3)	unprognostic (2.49e-2)	unprognostic (3.41e-2)
unprognostic (1.99e-2)	unprognostic (2.03e-1)	unprognostic (2.51e-1)	unprognostic (5.64e-2)	unprognostic (2.77e-1)	unprognostic (4.34e-3)
unprognostic (9.67e-2)	unprognostic (1.04e-1)	unprognostic (8.46e-2)	unprognostic (1.94e-2)	unprognostic (1.12e-1)	unprognostic (8.14e-2)
unprognostic (1.34e-2)	unprognostic (2.33e-2)	unprognostic (8.62e-2)	unprognostic (8.26e-2)	unprognostic (5.73e-2)	unprognostic (6.30e-3)
unprognostic (1.28e-1)	unprognostic (3.41e-2)	unprognostic (1.03e-1)	unprognostic (5.67e-2)	unprognostic (8.89e-2)	unprognostic (4.26e-2)
unprognostic (4.88e-2)	unprognostic (1.88e-2)	unprognostic (8.27e-2)	unprognostic (3.17e-3)	unprognostic (4.58e-2)	unprognostic (1.36e-2)
unprognostic (1.23e-1)		unprognostic (1.83e-2)	unprognostic (3.79e-2)		unprognostic (3.67e-2)
unprognostic (4.39e-1)	unprognostic (1.38e-1)	unprognostic (5.08e-2)	unprognostic (4.91e-2)	unprognostic (8.14e-2)	unprognostic (3.26e-3)
unprognostic (3.52e-2)	unprognostic (4.46e-1)	unprognostic (9.35e-2)	unprognostic (1.22e-2)	unprognostic (6.91e-2)	unprognostic (4.65e-2)
unprognostic (1.47e-2)	unprognostic (1.83e-1)	unprognostic (2.17e-2)	unprognostic (1.70e-2)	unprognostic (1.20e-2)	unprognostic (1.35e-1)
unprognostic (1.76e-1)	unprognostic (1.91e-1)	unprognostic (2.47e-1)	unprognostic (3.14e-1)	unprognostic (1.39e-1)	unprognostic (1.99e-1)
potential prognostic unfavorable (8.02e-5)	unprognostic (1.81e-2)	unprognostic (8.69e-3)	unprognostic (2.84e-1)	unprognostic (6.33e-3)	unprognostic (2.81e-1)
unprognostic (1.43e-1)	unprognostic (3.36e-2)	unprognostic (5.11e-2)	unprognostic (3.80e-1)	unprognostic (1.67e-1)	unprognostic (1.05e-1)
unprognostic (1.68e-1)	unprognostic (2.76e-2)		unprognostic (2.31e-1)	unprognostic (1.20e-2)	unprognostic (6.55e-2)
potential prognostic favorable (7.95e-4)	unprognostic (2.22e-2)	unprognostic (2.26e-1)	unprognostic (4.59e-3)	potential prognostic unfavorable (1.73e-4)	unprognostic (3.25e-2)
unprognostic (1.42e-1)	unprognostic (7.77e-2)	unprognostic (1.99e-1)	unprognostic (3.07e-2)		
unprognostic (7.89e-2)			unprognostic (3.70e-2)		
unprognostic (4.22e-1)	unprognostic (8.41e-3)	unprognostic (1.72e-1)	unprognostic (2.20e-1)	unprognostic (4.69e-3)	unprognostic (3.00e-2)
unprognostic (9.32e-2)	unprognostic (1.53e-1)	unprognostic (1.22e-1)	unprognostic (7.87e-3)	unprognostic (8.78e-3)	unprognostic (4.42e-3)
unprognostic (2.90e-1)				unprognostic (2.09e-1)	
unprognostic (7.69e-2)	unprognostic (1.95e-1)	unprognostic (3.63e-1)	unprognostic (6.31e-2)	unprognostic (2.47e-2)	unprognostic (8.32e-3)
			unprognostic (1.67e-1)		
unprognostic (7.60e-2)	unprognostic (8.56e-2)	unprognostic (2.82e-2)	unprognostic (3.98e-2)	unprognostic (4.12e-2)	unprognostic (3.35e-2)
unprognostic (1.04e-1)	unprognostic (9.24e-2)	potential prognostic favorable (3.33e-4)	unprognostic (1.39e-2)	unprognostic (8.07e-2)	unprognostic (2.38e-3)
unprognostic (8.58e-3)	unprognostic (1.32e-3)	unprognostic (1.80e-2)	unprognostic (1.70e-1)	unprognostic (2.84e-2)	unprognostic (1.69e-1)
unprognostic (2.27e-1)		unprognostic (2.18e-1)	unprognostic (1.61e-2)	unprognostic (1.72e-1)	unprognostic (1.88e-1)
unprognostic (3.06e-3)	unprognostic (3.34e-1)	unprognostic (1.75e-1)	unprognostic (6.80e-2)	unprognostic (1.94e-2)	unprognostic (2.37e-1)
unprognostic (2.60e-3)	unprognostic (8.42e-2)	unprognostic (9.50e-2)	unprognostic (7.11e-3)	unprognostic (1.03e-1)	unprognostic (3.05e-1)
unprognostic (2.66e-3)			unprognostic (4.00e-2)	potential prognostic favorable (8.76e-4)	unprognostic (1.39e-2)
unprognostic (1.42e-1)	unprognostic (6.14e-2)	unprognostic (8.71e-2)	unprognostic (1.18e-1)	unprognostic (3.02e-2)	unprognostic (3.81e-2)
unprognostic (3.67e-1)	unprognostic (8.27e-2)	unprognostic (2.02e-1)	unprognostic (3.85e-1)	unprognostic (1.27e-2)	unprognostic (1.27e-1)
potential prognostic unfavorable (1.30e-5)	unprognostic (5.81e-2)	unprognostic (1.66e-1)	unprognostic (1.94e-2)	unprognostic (4.66e-2)	unprognostic (3.76e-2)
unprognostic (3.48e-3)	unprognostic (1.59e-1)	unprognostic (4.33e-2)	unprognostic (2.67e-2)	unprognostic (1.00e-1)	unprognostic (7.24e-2)
unprognostic (2.64e-1)			unprognostic (3.15e-2)		unprognostic (1.11e-1)
potential prognostic unfavorable (7.47e-4)	unprognostic (1.24e-1)	unprognostic (5.28e-2)	unprognostic (3.77e-2)	unprognostic (4.14e-3)	unprognostic (1.74e-1)
unprognostic (1.76e-1)	unprognostic (1.14e-1)	unprognostic (2.05e-1)	unprognostic (1.66e-1)		unprognostic (7.42e-2)
unprognostic (1.45e-2)					
unprognostic (5.20e-2)	unprognostic (5.22e-2)	unprognostic (5.95e-2)	unprognostic (6.60e-2)	unprognostic (1.04e-1)	unprognostic (8.59e-2)
unprognostic (1.71e-1)	unprognostic (3.03e-1)	unprognostic (3.29e-3)	unprognostic (1.70e-1)	unprognostic (2.22e-1)	unprognostic (8.78e-2)
unprognostic (8.37e-2)	unprognostic (3.70e-2)	unprognostic (2.54e-3)	potential prognostic unfavorable (9.89e-4)	unprognostic (4.86e-2)	unprognostic (2.83e-1)
unprognostic (1.28e-3)	unprognostic (7.69e-2)		unprognostic (6.67e-2)	unprognostic (1.12e-1)	
unprognostic (2.04e-1)	unprognostic (1.58e-1)	unprognostic (2.83e-1)	unprognostic (2.39e-1)	unprognostic (1.76e-2)	unprognostic (1.09e-2)
unprognostic (2.37e-1)	unprognostic (8.60e-2)	unprognostic (6.72e-2)	unprognostic (1.91e-2)	unprognostic (1.68e-1)	unprognostic (3.22e-2)
unprognostic (2.78e-2)	unprognostic (3.18e-1)	unprognostic (3.51e-1)	unprognostic (2.25e-3)	unprognostic (1.33e-1)	unprognostic (4.14e-3)
unprognostic (1.46e-3)	unprognostic (1.20e-1)	unprognostic (7.22e-2)	unprognostic (4.24e-3)		potential prognostic favorable (1.91e-4)
unprognostic (2.88e-2)	unprognostic (7.32e-2)	unprognostic (4.39e-2)	unprognostic (1.20e-1)	unprognostic (1.68e-1)	unprognostic (1.29e-1)
unprognostic (3.15e-2)	unprognostic (6.89e-2)	unprognostic (2.58e-1)	potential prognostic favorable (3.06e-4)	unprognostic (1.94e-1)	unprognostic (1.45e-1)
unprognostic (1.06e-1)			unprognostic (8.49e-2)	unprognostic (2.82e-1)	unprognostic (1.13e-1)
unprognostic (3.42e-1)	unprognostic (3.00e-1)	unprognostic (8.31e-2)	unprognostic (4.61e-1)	unprognostic (1.01e-1)	unprognostic (3.31e-1)
unprognostic (3.51e-3)	unprognostic (4.16e-1)	unprognostic (9.16e-2)	unprognostic (1.82e-2)	unprognostic (1.10e-1)	unprognostic (2.89e-1)
unprognostic (7.41e-2)	unprognostic (2.90e-1)	unprognostic (3.37e-2)	unprognostic (2.37e-2)	unprognostic (9.40e-2)	unprognostic (2.90e-1)
unprognostic (6.90e-2)	unprognostic (1.55e-2)	unprognostic (3.65e-3)	unprognostic (1.39e-1)		unprognostic (4.64e-2)
unprognostic (1.57e-1)	unprognostic (1.81e-1)	unprognostic (4.82e-3)	unprognostic (1.34e-2)	unprognostic (2.23e-2)	unprognostic (8.06e-2)
unprognostic (1.31e-2)	unprognostic (2.73e-2)	unprognostic (5.36e-3)	unprognostic (3.28e-1)	unprognostic (3.53e-2)	unprognostic (2.09e-1)
unprognostic (1.02e-2)	unprognostic (3.75e-1)	unprognostic (1.74e-2)	unprognostic (7.38e-2)	unprognostic (3.01e-2)	unprognostic (7.14e-2)
unprognostic (4.92e-2)	unprognostic (4.56e-2)	unprognostic (2.68e-1)	unprognostic (1.23e-2)	unprognostic (2.14e-2)	unprognostic (5.93e-2)
	unprognostic (3.42e-2)			unprognostic (9.86e-2)	
	unprognostic (2.51e-2)	unprognostic (8.02e-2)	unprognostic (6.57e-2)	unprognostic (1.64e-1)	potential prognostic unfavorable (8.43e-4)
unprognostic (1.22e-2)	unprognostic (3.45e-1)	unprognostic (6.75e-2)	unprognostic (5.00e-3)	unprognostic (2.67e-1)	unprognostic (2.07e-2)
unprognostic (5.94e-2)	unprognostic (8.04e-2)	unprognostic (6.28e-3)	unprognostic (2.43e-1)		unprognostic (3.94e-1)
unprognostic (2.66e-1)	unprognostic (2.70e-2)	unprognostic (1.95e-2)	unprognostic (1.78e-2)	unprognostic (3.32e-2)	unprognostic (1.07e-2)
unprognostic (2.22e-2)		unprognostic (8.57e-3)	unprognostic (1.12e-1)		unprognostic (2.05e-3)

unprognostic (1.02e-1)	unprognostic (3.06e-1)	unprognostic (2.86e-3)	unprognostic (1.07e-2)	unprognostic (2.77e-1)	unprognostic (2.78e-1)
unprognostic (1.74e-1)	unprognostic (1.56e-1)		unprognostic (5.71e-2)	unprognostic (2.04e-2)	unprognostic (6.59e-3)
unprognostic (2.02e-2)			unprognostic (1.22e-1)		
				unprognostic (1.12e-1)	
unprognostic (6.60e-2)	unprognostic (4.06e-1)	unprognostic (1.06e-1)	unprognostic (5.20e-2)	unprognostic (2.60e-1)	unprognostic (1.63e-2)
unprognostic (1.80e-1)	unprognostic (1.32e-1)	unprognostic (4.67e-1)	unprognostic (1.15e-1)	unprognostic (3.53e-1)	potential prognostic unfavorable (4.66e-4)
unprognostic (3.53e-1)		unprognostic (2.69e-2)	unprognostic (2.54e-1)	unprognostic (1.87e-1)	unprognostic (9.29e-2)
potential prognostic unfavorable (6.89e-4)	unprognostic (8.71e-2)	unprognostic (6.81e-2)	unprognostic (4.83e-3)	unprognostic (7.28e-2)	unprognostic (2.05e-2)
unprognostic (3.19e-1)	unprognostic (1.27e-1)	unprognostic (3.66e-1)	unprognostic (8.54e-2)	unprognostic (6.39e-2)	unprognostic (3.19e-2)
unprognostic (3.26e-2)	unprognostic (2.35e-1)	unprognostic (8.03e-3)	unprognostic (5.34e-2)		unprognostic (6.51e-3)
potential prognostic unfavorable (3.47e-5)	unprognostic (3.29e-1)	unprognostic (1.37e-2)	unprognostic (1.37e-1)	unprognostic (2.53e-1)	unprognostic (2.52e-1)
unprognostic (1.39e-2)	unprognostic (1.07e-1)	unprognostic (6.95e-2)	unprognostic (1.87e-1)	unprognostic (2.21e-1)	unprognostic (1.51e-1)
unprognostic (6.93e-2)		unprognostic (2.42e-2)	unprognostic (3.12e-1)	unprognostic (6.18e-2)	unprognostic (1.16e-1)
			unprognostic (1.46e-1)		
unprognostic (6.33e-2)	unprognostic (2.40e-2)	unprognostic (5.65e-2)	unprognostic (9.04e-2)	unprognostic (2.58e-1)	unprognostic (1.51e-1)
unprognostic (2.82e-1)	unprognostic (2.46e-1)	unprognostic (5.89e-3)	unprognostic (7.93e-2)		unprognostic (2.48e-1)
unprognostic (3.48e-2)	unprognostic (3.68e-1)	unprognostic (1.32e-1)	unprognostic (1.07e-1)	unprognostic (2.04e-2)	unprognostic (2.39e-1)
unprognostic (7.80e-2)	unprognostic (7.06e-2)	unprognostic (1.15e-2)	unprognostic (1.01e-1)	unprognostic (1.38e-1)	unprognostic (6.73e-3)
unprognostic (8.17e-2)	unprognostic (7.65e-2)	unprognostic (7.11e-3)	unprognostic (3.24e-2)	unprognostic (1.71e-1)	unprognostic (1.54e-2)
unprognostic (3.32e-1)	unprognostic (3.46e-2)	unprognostic (4.36e-2)	unprognostic (3.36e-3)	unprognostic (2.04e-1)	unprognostic (1.93e-1)
unprognostic (7.87e-3)	unprognostic (1.18e-1)	unprognostic (9.57e-3)	unprognostic (3.20e-1)		unprognostic (5.29e-3)
unprognostic (1.28e-1)	unprognostic (8.65e-2)	unprognostic (3.77e-2)	unprognostic (1.37e-1)	unprognostic (3.18e-2)	unprognostic (7.62e-3)
unprognostic (2.94e-1)	unprognostic (1.19e-1)	unprognostic (1.70e-2)	unprognostic (1.14e-1)	unprognostic (3.00e-1)	unprognostic (9.11e-2)
unprognostic (1.65e-1)	unprognostic (4.41e-1)	unprognostic (5.51e-3)	unprognostic (3.73e-3)	unprognostic (2.61e-1)	unprognostic (1.91e-1)
potential prognostic favorable (4.81e-4)	unprognostic (8.72e-2)	unprognostic (6.70e-2)	unprognostic (2.05e-2)	unprognostic (6.42e-2)	unprognostic (5.12e-2)
unprognostic (5.13e-2)	unprognostic (1.27e-1)	unprognostic (5.89e-3)	unprognostic (2.26e-2)	unprognostic (1.38e-1)	unprognostic (1.94e-2)
unprognostic (6.84e-2)	unprognostic (1.05e-1)	unprognostic (5.33e-2)	unprognostic (3.94e-2)	unprognostic (8.68e-3)	unprognostic (1.06e-1)
unprognostic (2.01e-2)	unprognostic (1.16e-1)	unprognostic (1.30e-1)	unprognostic (1.52e-1)	unprognostic (3.19e-3)	unprognostic (7.46e-2)
unprognostic (3.08e-1)	unprognostic (3.90e-2)	unprognostic (2.00e-1)	unprognostic (2.87e-2)	unprognostic (6.65e-2)	unprognostic (1.83e-1)
unprognostic (4.46e-3)	unprognostic (8.50e-2)	unprognostic (3.41e-2)	unprognostic (9.33e-2)	unprognostic (1.51e-1)	unprognostic (2.01e-1)
unprognostic (2.69e-2)	unprognostic (1.37e-1)	unprognostic (6.53e-2)	unprognostic (7.82e-2)	unprognostic (3.01e-2)	unprognostic (3.40e-1)
unprognostic (1.07e-1)			unprognostic (1.94e-3)	unprognostic (2.19e-2)	potential prognostic unfavorable (5.39e-5)
unprognostic (2.29e-2)	unprognostic (3.72e-2)				
unprognostic (1.49e-3)	unprognostic (6.83e-2)	unprognostic (1.72e-2)	unprognostic (3.73e-3)	unprognostic (6.46e-2)	unprognostic (1.10e-1)
unprognostic (7.84e-2)	unprognostic (7.01e-2)	unprognostic (2.56e-1)	unprognostic (1.64e-2)	unprognostic (3.33e-1)	unprognostic (1.16e-2)
unprognostic (7.35e-3)	unprognostic (2.12e-1)		unprognostic (1.11e-2)	unprognostic (2.05e-2)	
unprognostic (2.45e-2)	unprognostic (2.42e-1)	unprognostic (2.02e-1)	unprognostic (2.95e-2)	unprognostic (2.27e-2)	unprognostic (3.65e-1)
unprognostic (4.84e-1)	unprognostic (2.63e-1)	unprognostic (2.18e-1)	unprognostic (2.78e-2)	unprognostic (5.41e-1)	unprognostic (1.97e-1)
unprognostic (3.19e-1)	unprognostic (2.24e-2)	unprognostic (3.37e-2)	unprognostic (2.62e-2)	unprognostic (4.56e-3)	unprognostic (5.30e-2)
unprognostic (1.76e-3)	unprognostic (1.54e-1)	unprognostic (4.96e-2)	unprognostic (5.61e-3)	unprognostic (4.36e-1)	unprognostic (1.53e-1)
unprognostic (1.93e-1)	unprognostic (4.64e-2)		unprognostic (1.25e-1)	unprognostic (1.42e-1)	unprognostic (1.83e-1)
unprognostic (2.53e-3)	unprognostic (6.64e-2)	unprognostic (6.89e-3)	unprognostic (5.77e-2)	unprognostic (1.63e-1)	unprognostic (6.77e-2)
unprognostic (1.43e-2)	unprognostic (4.09e-1)	unprognostic (3.27e-2)	unprognostic (1.23e-1)	unprognostic (1.82e-1)	unprognostic (6.50e-2)
unprognostic (4.98e-2)	unprognostic (4.38e-2)	unprognostic (1.91e-2)	unprognostic (4.31e-2)	unprognostic (1.73e-2)	unprognostic (2.79e-2)
unprognostic (5.85e-2)		unprognostic (3.91e-1)	unprognostic (2.89e-1)		unprognostic (5.02e-2)
unprognostic (2.11e-2)	unprognostic (8.13e-2)	unprognostic (5.21e-2)	unprognostic (2.87e-1)	unprognostic (2.97e-1)	unprognostic (1.58e-2)
unprognostic (9.09e-2)	unprognostic (9.55e-2)	unprognostic (1.98e-1)	unprognostic (1.53e-2)	unprognostic (1.61e-2)	unprognostic (2.27e-3)
unprognostic (7.23e-3)	unprognostic (1.11e-2)	unprognostic (1.94e-2)	unprognostic (7.83e-2)	unprognostic (1.05e-1)	unprognostic (1.22e-1)
unprognostic (6.18e-2)	unprognostic (9.79e-2)			unprognostic (6.61e-2)	
unprognostic (1.48e-1)	unprognostic (3.07e-1)	unprognostic (3.05e-2)	unprognostic (1.44e-3)	unprognostic (2.02e-1)	unprognostic (7.02e-3)
unprognostic (1.78e-1)			unprognostic (3.62e-2)		unprognostic (1.64e-2)
unprognostic (1.01e-1)	unprognostic (1.15e-1)	unprognostic (1.74e-1)	unprognostic (1.19e-1)	unprognostic (9.53e-3)	unprognostic (5.59e-2)
unprognostic (1.78e-1)	unprognostic (3.73e-1)	unprognostic (1.23e-1)	unprognostic (1.11e-2)	unprognostic (3.57e-2)	unprognostic (3.81e-2)
unprognostic (2.68e-2)	unprognostic (2.27e-3)	unprognostic (1.88e-3)	unprognostic (4.84e-2)	unprognostic (6.42e-2)	unprognostic (4.99e-1)
unprognostic (2.37e-3)	unprognostic (1.28e-1)	unprognostic (3.66e-1)	potential prognostic unfavorable (2.06e-5)	unprognostic (3.77e-2)	potential prognostic unfavorable (5.50e-5)
	unprognostic (1.82e-1)			unprognostic (7.66e-2)	
unprognostic (9.72e-2)	potential prognostic favorable (1.98e-4)		potential prognostic favorable (5.53e-4)		
unprognostic (2.15e-1)	unprognostic (2.23e-2)	unprognostic (2.71e-1)	unprognostic (5.08e-3)	unprognostic (8.42e-3)	unprognostic (1.99e-2)
unprognostic (8.53e-3)					unprognostic (2.49e-1)
unprognostic (2.45e-3)	unprognostic (2.04e-1)	unprognostic (1.66e-1)	unprognostic (1.23e-2)	unprognostic (4.72e-3)	unprognostic (7.21e-2)
unprognostic (2.39e-1)	unprognostic (1.88e-1)	unprognostic (2.41e-1)	unprognostic (2.39e-1)	unprognostic (9.97e-2)	unprognostic (2.09e-1)
unprognostic (1.85e-2)	unprognostic (1.17e-1)	unprognostic (4.94e-2)	unprognostic (6.59e-3)	unprognostic (4.64e-2)	unprognostic (4.00e-2)
unprognostic (1.80e-3)	unprognostic (6.71e-2)	unprognostic (1.37e-2)	unprognostic (1.64e-3)		unprognostic (4.90e-2)
unprognostic (1.91e-2)	unprognostic (2.62e-1)	unprognostic (3.95e-3)	unprognostic (2.16e-3)	unprognostic (3.60e-2)	unprognostic (2.14e-2)
unprognostic (6.20e-2)	unprognostic (2.05e-1)	unprognostic (1.06e-1)	unprognostic (1.01e-1)	unprognostic (1.59e-1)	potential prognostic unfavorable (6.37e-4)
	unprognostic (2.15e-2)				
unprognostic (7.60e-2)	unprognostic (1.17e-1)		unprognostic (2.08e-2)	unprognostic (6.47e-2)	unprognostic (7.45e-3)
unprognostic (7.99e-2)	unprognostic (1.88e-1)	unprognostic (1.96e-3)	unprognostic (4.99e-1)	unprognostic (1.27e-2)	unprognostic (2.42e-1)
	unprognostic (2.65e-2)	unprognostic (2.45e-1)	unprognostic (1.82e-2)		
unprognostic (2.56e-1)	unprognostic (1.50e-1)	unprognostic (3.27e-2)	unprognostic (3.43e-2)	unprognostic (2.24e-1)	unprognostic (1.58e-1)
unprognostic (2.12e-2)	unprognostic (2.82e-2)	unprognostic (1.19e-2)	unprognostic (1.13e-2)	unprognostic (1.44e-1)	unprognostic (1.99e-1)
unprognostic (7.42e-3)	unprognostic (6.37e-2)	unprognostic (1.24e-2)	unprognostic (3.61e-1)	unprognostic (7.54e-2)	unprognostic (1.70e-2)
unprognostic (9.10e-2)	unprognostic (1.52e-1)	unprognostic (2.32e-3)	unprognostic (9.11e-2)	unprognostic (2.54e-1)	unprognostic (3.38e-1)
potential prognostic unfavorable (4.10e-4)	unprognostic (6.99e-3)	unprognostic (4.35e-1)	unprognostic (3.12e-1)	unprognostic (1.55e-2)	unprognostic (3.09e-1)
unprognostic (8.53e-2)	unprognostic (2.30e-2)	unprognostic (3.63e-2)	unprognostic (9.07e-2)	unprognostic (2.11e-2)	unprognostic (4.48e-3)
unprognostic (4.11e-1)	unprognostic (1.10e-1)	unprognostic (1.43e-1)	unprognostic (6.99e-3)	unprognostic (8.08e-2)	unprognostic (2.61e-2)
unprognostic (1.69e-3)	unprognostic (6.51e-2)	unprognostic (3.13e-2)	unprognostic (9.46e-3)	unprognostic (3.64e-1)	unprognostic (1.93e-1)
unprognostic (7.14e-2)	unprognostic (1.45e-1)	unprognostic (7.82e-3)	unprognostic (8.11e-2)	unprognostic (1.70e-1)	unprognostic (2.45e-2)
unprognostic (1.59e-3)	unprognostic (8.36e-3)			unprognostic (2.80e-2)	unprognostic (1.94e-1)
unprognostic (5.32e-3)	unprognostic (1.37e-1)	unprognostic (1.45e-2)	unprognostic (2.08e-2)	unprognostic (1.61e-1)	unprognostic (1.00e-1)
unprognostic (2.29e-2)	unprognostic (1.66e-1)	unprognostic (1.07e-2)	unprognostic (2.36e-1)	unprognostic (3.45e-1)	unprognostic (5.04e-2)
unprognostic (1.09e-1)	unprognostic (7.28e-2)	unprognostic (1.28e-2)	unprognostic (3.14e-2)	unprognostic (2.34e-1)	unprognostic (1.63e-1)
unprognostic (1.65e-1)	unprognostic (5.69e-2)	unprognostic (1.02e-2)	unprognostic (2.72e-2)	unprognostic (1.80e-1)	unprognostic (1.24e-2)
unprognostic (5.04e-3)	unprognostic (1.32e-1)	unprognostic (1.51e-1)	unprognostic (1.18e-2)	unprognostic (1.43e-1)	unprognostic (4.60e-2)
unprognostic (1.26e-1)	unprognostic (5.60e-2)	unprognostic (1.11e-1)	unprognostic (3.24e-1)	unprognostic (5.03e-2)	potential prognostic favorable (9.95e-4)
unprognostic (5.84e-2)	unprognostic (3.50e-1)	unprognostic (2.93e-1)	potential prognostic unfavorable (2.09e-4)	unprognostic (1.03e-1)	unprognostic (1.67e-1)
unprognostic (1.47e-1)	unprognostic (7.96e-2)	unprognostic (1.09e-1)	unprognostic (1.54e-3)	unprognostic (8.12e-2)	potential prognostic unfavorable (1.57e-4)
unprognostic (2.42e-1)	potential prognostic unfavorable (3.33e-5)		unprognostic (7.80e-3)		
unprognostic (8.02e-3)	unprognostic (3.06e-3)		unprognostic (3.07e-2)	unprognostic (2.57e-1)	unprognostic (1.91e-1)
potential prognostic unfavorable (2.04e-4)	unprognostic (7.75e-2)	unprognostic (1.08e-2)	unprognostic (4.62e-2)	unprognostic (4.04e-2)	unprognostic (2.79e-1)
unprognostic (2.10e-1)	unprognostic (2.12e-1)	unprognostic (3.75e-2)	unprognostic (1.63e-1)	unprognostic (1.35e-2)	unprognostic (7.77e-2)
unprognostic (1.62e-1)	unprognostic (1.11e-1)	unprognostic (6.08e-2)	unprognostic (2.54e-1)	unprognostic (1.82e-1)	unprognostic (1.42e-1)
unprognostic (3.11e-2)	unprognostic (5.90e-2)	unprognostic (2.23e-1)	potential prognostic unfavorable (7.06e-5)	unprognostic (1.89e-2)	unprognostic (1.94e-3)
unprognostic (2.26e-1)	unprognostic (2.31e-1)	unprognostic (2.08e-1)	unprognostic (5.33e-3)		unprognostic (3.38e-2)
unprognostic (6.95e-2)	unprognostic (8.35e-2)	unprognostic (2.92e-1)	unprognostic (3.93e-2)	unprognostic (1.56e-2)	unprognostic (6.53e-3)
unprognostic (2.60e-1)	unprognostic (6.07e-2)	unprognostic (3.95e-1)	unprognostic (5.39e-3)	unprognostic (1.12e-1)	potential prognostic unfavorable (5.24e-4)
unprognostic (3.42e-2)			unprognostic (1.06e-1)		unprognostic (2.07e-2)
	unprognostic (1.32e-1)	unprognostic (3.53e-2)	unprognostic (1.57e-1)	unprognostic (2.10e-1)	unprognostic (2.35e-3)
unprognostic (2.89e-2)					
unprognostic (1.72e-3)	unprognostic (2.80e-1)	unprognostic (1.36e-1)	unprognostic (1.02e-1)	unprognostic (6.05e-2)	unprognostic (1.82e-3)
unprognostic (2.59e-1)	unprognostic (2.18e-1)	unprognostic (1.11e-2)	unprognostic (4.01e-2)	unprognostic (7.90e-2)	unprognostic (9.36e-2)
unprognostic (1.75e-1)			unprognostic (8.38e-3)		unprognostic (5.03e-2)
unprognostic (2.80e-2)	unprognostic (6.93e-2)	unprognostic (5.80e-2)	unprognostic (2.36e-1)	unprognostic (2.17e-1)	unprognostic (2.66e-1)
unprognostic (3.11e-1)	unprognostic (2.07e-1)	unprognostic (2.24e-2)	unprognostic (8.06e-3)	unprognostic (1.42e-1)	unprognostic (1.00e-2)
unprognostic (1.51e-1)	unprognostic (1.41e-1)	unprognostic (1.32e-2)	unprognostic (3.13e-2)		unprognostic (8.97e-2)
unprognostic (1.73e-2)	unprognostic (2.65e-2)	unprognostic (3.39e-1)	unprognostic (3.64e-2)	unprognostic (1.33e-1)	unprognostic (2.18e-2)
unprognostic (1.83e-1)	unprognostic (2.17e-1)	unprognostic (2.33e-2)	unprognostic (9.89e-2)	unprognostic (1.48e-1)	unprognostic (1.70e-1)
unprognostic (1.14e-2)	unprognostic (2.72e-1)	unprognostic (5.02e-3)	potential prognostic favorable (2.31e-7)	unprognostic (1.27e-1)	unprognostic (1.20e-1)
unprognostic (8.01e-2)	unprognostic (3.17e-2)	unprognostic (3.56e-2)	unprognostic (2.80e-1)	unprognostic (2.64e-2)	unprognostic (2.05e-1)
unprognostic (9.71e-3)	unprognostic (2.65e-1)	unprognostic (1.10e-1)	unprognostic (5.75e-3)	unprognostic (1.52e-2)	unprognostic (8.71e-2)
unprognostic (2.28e-3)	unprognostic (2.21e-1)	unprognostic (2.01e-3)	unprognostic (1.76e-1)	unprognostic (2.95e-1)	unprognostic (6.28e-2)
unprognostic (5.79e-3)	unprognostic (3.41e-1)	unprognostic (3.67e-2)	unprognostic (6.36e-3)	unprognostic (6.26e-3)	unprognostic (2.24e-1)
unprognostic (4.67e-3)	unprognostic (2.39e-2)	unprognostic (3.96e-3)	unprognostic (2.84e-1)	unprognostic (2.36e-1)	unprognostic (1.14e-1)
unprognostic (5.15e-3)	unprognostic (5.09e-2)	unprognostic (4.82e-2)	unprognostic (2.43e-2)	unprognostic (5.69e-2)	unprognostic (1.83e-1)
unprognostic (5.75e-2)	unprognostic (3.76e-1)	unprognostic (1.32e-1)	unprognostic (3.77e-3)	unprognostic (2.63e-1)	unprognostic (1.25e-1)
unprognostic (1.71e-2)	unprognostic (4.13e-2)	unprognostic (2.07e-2)	unprognostic (1.37e-2)	unprognostic (1.52e-1)	unprognostic (7.04e-3)
unprognostic (2.10e-2)	unprognostic (1.07e-1)	unprognostic (1.22e-2)	unprognostic (8.70e-3)	unprognostic (3.76e-2)	potential prognostic unfavorable (9.17e-4)
unprognostic (8.63e-2)	unprognostic (2.86e-1)	unprognostic (8.48e-2)	unprognostic (8.31e-2)	unprognostic (2.79e-1)	unprognostic (1.02e-2)
unprognostic (1.43e-2)	unprognostic (1.95e-1)	unprognostic (5.68e-2)	unprognostic (1.58e-1)	unprognostic (1.03e-1)	unprognostic (2.01e-1)

unprognostic (9.45e-3)	unprognostic (1.61e-1)	unprognostic (3.08e-1)	unprognostic (1.02e-1)	potential prognostic unfavorable (5.09e-4)	unprognostic (1.42e-1)
unprognostic (5.60e-3)	unprognostic (1.70e-1)	unprognostic (1.67e-3)	unprognostic (7.50e-3)	potential prognostic unfavorable (6.82e-4)	unprognostic (4.00e-1)
unprognostic (3.35e-3)	unprognostic (6.98e-2)	unprognostic (1.86e-1)	unprognostic (9.36e-2)	unprognostic (2.52e-2)	unprognostic (1.90e-1)
unprognostic (6.11e-3)	unprognostic (4.86e-2)	unprognostic (2.19e-2)	unprognostic (1.95e-1)	unprognostic (4.98e-3)	unprognostic (9.50e-2)
unprognostic (9.14e-2)	unprognostic (3.19e-1)	unprognostic (5.56e-2)	unprognostic (4.42e-2)	unprognostic (1.01e-1)	unprognostic (1.66e-1)
unprognostic (1.40e-2)	unprognostic (9.26e-2)	unprognostic (2.19e-1)	unprognostic (3.13e-2)	unprognostic (1.31e-1)	unprognostic (1.01e-1)
potential prognostic unfavorable (3.46e-4)	unprognostic (6.96e-2)	unprognostic (1.57e-2)	unprognostic (7.58e-3)	unprognostic (1.04e-1)	unprognostic (2.96e-1)
unprognostic (8.39e-3)		unprognostic (2.01e-1)	unprognostic (1.70e-1)	unprognostic (7.60e-2)	unprognostic (2.60e-1)
unprognostic (6.46e-3)	unprognostic (7.15e-2)	unprognostic (9.55e-2)	unprognostic (2.61e-1)	unprognostic (3.73e-2)	unprognostic (2.50e-1)
unprognostic (2.90e-2)	unprognostic (9.64e-2)	potential prognostic unfavorable (8.47e-4)	unprognostic (1.20e-2)	unprognostic (2.28e-2)	unprognostic (9.93e-2)
unprognostic (3.10e-1)	unprognostic (1.23e-2)	unprognostic (3.78e-2)	unprognostic (1.75e-2)	unprognostic (7.71e-2)	unprognostic (1.67e-3)
unprognostic (1.20e-1)	unprognostic (1.19e-1)	unprognostic (4.57e-2)	unprognostic (8.80e-2)	unprognostic (1.00e-1)	unprognostic (4.52e-2)
unprognostic (1.09e-2)	unprognostic (1.02e-2)	unprognostic (1.13e-1)	unprognostic (7.74e-3)	unprognostic (3.29e-2)	unprognostic (3.74e-2)
unprognostic (2.83e-2)	unprognostic (2.05e-2)	unprognostic (1.70e-1)	unprognostic (2.00e-1)	unprognostic (2.49e-1)	unprognostic (3.03e-2)
unprognostic (2.00e-2)	unprognostic (2.18e-2)	unprognostic (1.52e-2)	unprognostic (8.66e-2)	unprognostic (1.33e-2)	unprognostic (1.23e-1)
unprognostic (1.26e-3)	unprognostic (4.44e-2)	unprognostic (2.68e-1)	unprognostic (3.25e-2)	unprognostic (4.22e-1)	unprognostic (2.45e-1)
unprognostic (1.17e-1)	unprognostic (4.66e-2)	unprognostic (1.66e-1)	unprognostic (1.29e-2)	unprognostic (1.80e-1)	unprognostic (1.63e-1)
unprognostic (3.18e-1)	unprognostic (3.51e-2)	unprognostic (5.74e-2)	unprognostic (7.83e-2)	unprognostic (4.78e-2)	unprognostic (1.30e-1)
unprognostic (3.87e-3)	unprognostic (4.20e-1)	unprognostic (1.59e-1)	unprognostic (1.16e-1)	unprognostic (7.46e-2)	unprognostic (1.64e-1)
unprognostic (5.16e-2)	unprognostic (2.48e-1)		unprognostic (8.01e-3)		unprognostic (1.12e-2)
unprognostic (3.15e-1)	unprognostic (1.59e-2)			unprognostic (4.15e-2)	
unprognostic (5.51e-3)	unprognostic (3.45e-1)	unprognostic (7.39e-2)	unprognostic (2.10e-2)	unprognostic (2.04e-1)	unprognostic (2.08e-1)
unprognostic (1.25e-2)	unprognostic (8.50e-2)	unprognostic (2.00e-1)	unprognostic (4.57e-3)	unprognostic (1.83e-3)	unprognostic (1.10e-1)
unprognostic (1.21e-1)		unprognostic (1.54e-1)	unprognostic (1.26e-2)	unprognostic (2.21e-2)	unprognostic (1.06e-2)
unprognostic (4.01e-1)	unprognostic (1.37e-1)	unprognostic (5.64e-2)	unprognostic (3.10e-1)	unprognostic (3.31e-2)	unprognostic (4.55e-2)
unprognostic (1.49e-1)	unprognostic (3.71e-1)	unprognostic (2.81e-3)	unprognostic (2.99e-1)	unprognostic (9.52e-2)	unprognostic (9.47e-2)
unprognostic (1.86e-2)	unprognostic (7.57e-2)	unprognostic (1.30e-1)	unprognostic (2.33e-2)	unprognostic (2.48e-1)	unprognostic (1.31e-3)
unprognostic (4.19e-1)	unprognostic (4.83e-1)	unprognostic (1.58e-1)	unprognostic (2.95e-1)		unprognostic (7.70e-2)
unprognostic (1.22e-1)	unprognostic (1.49e-1)	unprognostic (1.38e-1)	unprognostic (2.96e-2)	unprognostic (5.48e-2)	unprognostic (4.30e-1)
unprognostic (2.53e-3)	unprognostic (7.85e-2)	unprognostic (1.38e-2)	unprognostic (5.42e-2)	unprognostic (4.78e-2)	unprognostic (1.60e-1)
unprognostic (3.00e-1)	unprognostic (1.45e-1)	unprognostic (1.28e-1)	unprognostic (8.97e-3)	unprognostic (4.54e-2)	unprognostic (1.01e-1)
unprognostic (6.58e-2)	unprognostic (2.40e-1)	unprognostic (9.07e-3)	unprognostic (2.62e-1)	unprognostic (1.80e-1)	unprognostic (6.37e-2)
unprognostic (1.69e-1)	unprognostic (1.23e-2)	unprognostic (4.40e-1)	unprognostic (3.17e-2)	unprognostic (9.13e-2)	unprognostic (1.28e-2)
unprognostic (3.74e-2)	unprognostic (5.00e-2)	unprognostic (5.88e-2)	unprognostic (2.38e-3)	unprognostic (1.13e-1)	unprognostic (6.14e-3)
unprognostic (1.81e-1)	unprognostic (1.63e-3)	unprognostic (3.11e-1)	unprognostic (8.96e-2)	unprognostic (1.93e-1)	unprognostic (2.15e-1)
unprognostic (6.54e-3)				unprognostic (1.69e-1)	
unprognostic (2.05e-2)		unprognostic (2.92e-1)	unprognostic (1.10e-1)	unprognostic (3.20e-1)	unprognostic (1.41e-1)
unprognostic (8.51e-3)	unprognostic (1.22e-1)	unprognostic (1.24e-1)	unprognostic (3.08e-2)	unprognostic (1.97e-1)	unprognostic (1.62e-1)
unprognostic (4.55e-3)	unprognostic (2.03e-1)	unprognostic (1.34e-1)	unprognostic (3.21e-1)	unprognostic (6.79e-2)	unprognostic (7.94e-2)
unprognostic (8.32e-2)	unprognostic (2.12e-1)	unprognostic (1.58e-1)	unprognostic (4.01e-1)	unprognostic (1.55e-1)	unprognostic (1.50e-1)
unprognostic (1.35e-1)	unprognostic (2.43e-2)	unprognostic (1.87e-1)	unprognostic (1.23e-1)	unprognostic (1.22e-1)	unprognostic (2.18e-3)
unprognostic (4.70e-2)	unprognostic (2.10e-1)	unprognostic (2.09e-2)	potential prognostic favorable (6.31e-4)	unprognostic (2.13e-1)	unprognostic (3.50e-1)
unprognostic (3.49e-2)	unprognostic (7.76e-2)	unprognostic (5.56e-2)	unprognostic (1.64e-1)	unprognostic (2.87e-1)	unprognostic (1.03e-1)
unprognostic (1.55e-1)	unprognostic (2.23e-1)	unprognostic (1.27e-1)	unprognostic (4.08e-2)	unprognostic (8.99e-3)	unprognostic (3.66e-1)
unprognostic (1.61e-1)			unprognostic (4.33e-2)	unprognostic (4.60e-2)	unprognostic (3.75e-2)
unprognostic (2.14e-2)	unprognostic (1.41e-2)	unprognostic (2.36e-1)	unprognostic (5.58e-2)	unprognostic (3.45e-1)	unprognostic (1.49e-2)
unprognostic (3.96e-2)			unprognostic (2.20e-1)		
unprognostic (1.34e-2)	unprognostic (5.92e-2)	unprognostic (2.98e-2)	unprognostic (5.18e-2)	unprognostic (6.93e-2)	unprognostic (2.67e-1)
unprognostic (1.27e-1)	unprognostic (1.50e-1)	unprognostic (3.87e-3)	unprognostic (2.27e-1)	unprognostic (9.08e-3)	unprognostic (2.54e-3)
potential prognostic unfavorable (4.36e-8)	unprognostic (1.14e-1)	unprognostic (2.12e-1)	unprognostic (4.38e-2)	unprognostic (2.76e-1)	unprognostic (3.81e-2)
unprognostic (8.82e-2)	unprognostic (2.27e-1)	unprognostic (1.43e-1)	unprognostic (2.95e-2)	unprognostic (7.02e-3)	unprognostic (4.35e-3)
unprognostic (1.28e-1)	unprognostic (2.71e-1)	unprognostic (5.94e-3)	unprognostic (3.17e-2)	unprognostic (1.45e-1)	unprognostic (1.18e-1)
unprognostic (7.33e-3)	unprognostic (2.54e-1)	unprognostic (9.53e-3)	unprognostic (4.16e-2)	unprognostic (2.71e-1)	unprognostic (1.47e-1)
	unprognostic (4.72e-1)		unprognostic (9.61e-2)		unprognostic (1.17e-3)
potential prognostic unfavorable (6.46e-4)	unprognostic (3.04e-1)	unprognostic (1.07e-3)	unprognostic (5.18e-2)	unprognostic (2.31e-1)	unprognostic (4.07e-2)
unprognostic (3.17e-1)	unprognostic (3.20e-1)	unprognostic (2.82e-2)	unprognostic (1.10e-1)	unprognostic (5.47e-2)	unprognostic (3.41e-1)
	unprognostic (1.08e-2)		unprognostic (2.27e-1)		potential prognostic unfavorable (4.61e-4)
unprognostic (7.88e-2)	unprognostic (1.68e-1)	unprognostic (7.61e-2)	unprognostic (1.91e-1)	unprognostic (1.07e-1)	unprognostic (7.86e-2)
unprognostic (2.18e-3)	unprognostic (2.94e-1)	unprognostic (1.03e-1)	unprognostic (1.11e-3)	unprognostic (1.25e-1)	unprognostic (2.78e-3)
unprognostic (1.02e-1)	unprognostic (1.50e-1)	unprognostic (7.60e-2)	unprognostic (1.01e-1)		unprognostic (4.06e-3)
unprognostic (1.06e-1)	unprognostic (1.84e-1)	unprognostic (1.16e-1)	unprognostic (2.02e-2)	unprognostic (1.52e-1)	unprognostic (2.25e-2)
unprognostic (1.60e-1)	unprognostic (1.12e-1)	unprognostic (1.12e-2)	potential prognostic favorable (7.36e-4)	unprognostic (8.65e-2)	unprognostic (1.48e-2)
unprognostic (3.76e-1)	unprognostic (1.19e-1)	unprognostic (4.88e-2)	unprognostic (2.52e-1)	unprognostic (3.18e-2)	unprognostic (4.25e-2)
unprognostic (2.43e-1)	unprognostic (4.30e-2)	unprognostic (1.70e-2)	unprognostic (4.20e-2)	unprognostic (2.91e-2)	unprognostic (1.74e-1)
unprognostic (1.26e-1)	unprognostic (7.69e-3)	unprognostic (3.30e-2)	unprognostic (1.98e-2)	unprognostic (8.18e-2)	unprognostic (2.43e-2)
unprognostic (1.75e-1)			potential prognostic favorable (7.50e-4)		unprognostic (9.29e-2)
unprognostic (9.43e-2)	unprognostic (7.04e-2)	unprognostic (3.01e-1)	unprognostic (4.18e-2)	unprognostic (1.57e-1)	unprognostic (1.68e-3)
unprognostic (7.04e-3)	unprognostic (5.20e-2)			unprognostic (3.01e-2)	
unprognostic (2.36e-2)	unprognostic (1.46e-3)	unprognostic (1.57e-1)	unprognostic (6.74e-2)	unprognostic (2.38e-1)	unprognostic (9.15e-2)
unprognostic (1.82e-1)	unprognostic (2.02e-1)	unprognostic (6.67e-2)	unprognostic (9.45e-2)	unprognostic (4.79e-2)	unprognostic (1.80e-1)
unprognostic (1.70e-3)	unprognostic (3.30e-2)	unprognostic (5.43e-2)	unprognostic (3.16e-1)	unprognostic (2.19e-1)	unprognostic (8.19e-2)
validated prognostic unfavorable (5.97e-4)		unprognostic (9.40e-2)	unprognostic (1.07e-1)	unprognostic (1.59e-1)	unprognostic (3.42e-1)
unprognostic (2.49e-1)			unprognostic (2.75e-1)		unprognostic (2.10e-1)
unprognostic (1.18e-3)	unprognostic (1.01e-1)		unprognostic (9.91e-2)	unprognostic (9.99e-2)	unprognostic (1.55e-1)
	unprognostic (9.55e-2)		unprognostic (5.08e-2)		
unprognostic (9.48e-3)	unprognostic (3.06e-2)	unprognostic (2.75e-1)	unprognostic (2.50e-1)	unprognostic (2.31e-2)	unprognostic (1.10e-2)
unprognostic (1.00e-1)		unprognostic (3.08e-1)	unprognostic (1.91e-1)	unprognostic (5.48e-2)	unprognostic (6.50e-2)
unprognostic (5.76e-2)	unprognostic (7.68e-2)	unprognostic (1.01e-1)	unprognostic (7.51e-2)	unprognostic (1.81e-2)	unprognostic (1.14e-3)
unprognostic (1.06e-1)	unprognostic (1.39e-1)	unprognostic (1.99e-1)	unprognostic (1.06e-1)	unprognostic (9.29e-2)	unprognostic (2.67e-1)
unprognostic (2.94e-2)	unprognostic (6.56e-2)	unprognostic (6.53e-2)	unprognostic (4.42e-3)	unprognostic (2.93e-2)	unprognostic (6.61e-2)
unprognostic (6.05e-2)	unprognostic (1.04e-1)	unprognostic (1.98e-1)	unprognostic (2.27e-1)	unprognostic (1.35e-1)	unprognostic (2.04e-1)
unprognostic (1.61e-3)	unprognostic (2.69e-1)	unprognostic (1.34e-1)	unprognostic (1.55e-1)	unprognostic (3.62e-1)	unprognostic (2.08e-1)
unprognostic (1.16e-1)		unprognostic (1.88e-3)	unprognostic (8.73e-2)	unprognostic (2.28e-2)	unprognostic (2.99e-1)
unprognostic (5.67e-2)	unprognostic (3.29e-1)	unprognostic (7.14e-2)	unprognostic (6.45e-2)	unprognostic (2.12e-1)	unprognostic (1.03e-1)
unprognostic (3.36e-2)	unprognostic (4.08e-2)	unprognostic (6.50e-3)	unprognostic (1.23e-1)	unprognostic (1.46e-1)	unprognostic (2.08e-1)
unprognostic (2.20e-1)	unprognostic (3.85e-2)	unprognostic (9.89e-2)	unprognostic (3.69e-2)	potential prognostic unfavorable (5.51e-4)	unprognostic (5.89e-2)
unprognostic (5.15e-2)					unprognostic (6.06e-2)
unprognostic (3.45e-1)	unprognostic (1.85e-1)	unprognostic (2.30e-1)	unprognostic (1.13e-1)		unprognostic (1.47e-1)
unprognostic (2.50e-1)	unprognostic (1.53e-2)	unprognostic (2.59e-2)	unprognostic (2.35e-1)	unprognostic (8.65e-2)	unprognostic (1.06e-1)
unprognostic (3.46e-2)	unprognostic (1.67e-1)	unprognostic (3.04e-1)	unprognostic (2.98e-1)	unprognostic (3.93e-2)	unprognostic (6.42e-2)
unprognostic (1.27e-2)	unprognostic (1.25e-1)	unprognostic (4.37e-2)	unprognostic (2.43e-1)	unprognostic (6.79e-2)	unprognostic (1.77e-1)
unprognostic (3.84e-1)			unprognostic (2.84e-1)	unprognostic (4.45e-3)	unprognostic (9.00e-3)
unprognostic (1.05e-1)	unprognostic (1.29e-1)	unprognostic (1.45e-2)	unprognostic (1.69e-1)	unprognostic (1.76e-1)	unprognostic (1.94e-1)
unprognostic (1.27e-1)	unprognostic (2.53e-2)	unprognostic (1.03e-1)	unprognostic (3.04e-1)	unprognostic (8.69e-3)	unprognostic (1.52e-2)
			potential prognostic favorable (4.47e-4)		
potential prognostic unfavorable (2.89e-4)	unprognostic (5.54e-2)	unprognostic (8.83e-2)	potential prognostic favorable (6.73e-4)	unprognostic (4.76e-2)	unprognostic (1.73e-1)
unprognostic (2.66e-1)					
unprognostic (1.60e-2)	unprognostic (3.86e-2)	unprognostic (7.37e-2)	unprognostic (2.19e-1)	unprognostic (2.49e-2)	unprognostic (4.54e-3)
unprognostic (2.14e-3)	unprognostic (1.90e-1)	unprognostic (2.44e-1)	unprognostic (8.35e-2)	unprognostic (8.37e-3)	unprognostic (2.89e-2)
unprognostic (2.20e-2)	unprognostic (1.65e-1)	unprognostic (2.07e-1)	potential prognostic favorable (2.64e-4)		unprognostic (2.84e-1)
unprognostic (1.46e-1)	unprognostic (2.70e-1)		unprognostic (1.19e-1)		unprognostic (1.16e-1)
unprognostic (2.02e-2)					
unprognostic (2.13e-3)	unprognostic (8.10e-2)	unprognostic (1.25e-1)	unprognostic (8.13e-3)	unprognostic (3.01e-1)	potential prognostic unfavorable (1.31e-4)
unprognostic (7.44e-2)	unprognostic (9.17e-2)	unprognostic (3.29e-3)	unprognostic (2.65e-3)	unprognostic (2.52e-1)	unprognostic (2.78e-2)
unprognostic (9.64e-2)	unprognostic (1.16e-1)	unprognostic (1.27e-1)	unprognostic (1.49e-1)	unprognostic (4.21e-1)	unprognostic (1.50e-1)
unprognostic (5.42e-2)					
unprognostic (6.27e-2)	unprognostic (9.30e-2)				unprognostic (1.60e-1)
potential prognostic unfavorable (3.60e-4)	unprognostic (2.07e-1)	unprognostic (1.31e-1)	potential prognostic favorable (3.28e-4)	unprognostic (2.53e-1)	unprognostic (5.81e-3)
potential prognostic unfavorable (2.02e-4)	unprognostic (7.07e-2)	unprognostic (1.25e-1)	unprognostic (2.27e-1)	unprognostic (4.40e-2)	unprognostic (1.83e-1)
unprognostic (3.16e-1)	unprognostic (1.33e-1)	unprognostic (3.10e-1)	unprognostic (2.73e-1)	unprognostic (2.40e-1)	unprognostic (3.81e-1)
unprognostic (1.10e-2)	unprognostic (1.61e-1)	unprognostic (2.71e-1)	unprognostic (2.46e-2)	unprognostic (2.71e-1)	unprognostic (6.16e-2)
potential prognostic favorable (2.70e-4)	unprognostic (1.18e-1)	unprognostic (2.39e-2)	unprognostic (3.23e-2)	unprognostic (4.88e-2)	unprognostic (3.65e-1)
unprognostic (1.33e-1)	unprognostic (2.23e-1)	unprognostic (1.65e-1)	unprognostic (4.53e-3)	unprognostic (1.97e-1)	unprognostic (4.04e-1)
unprognostic (8.71e-2)	unprognostic (5.48e-2)	unprognostic (9.12e-2)	unprognostic (1.22e-1)	unprognostic (2.25e-1)	unprognostic (4.16e-2)
unprognostic (3.11e-1)	unprognostic (1.27e-1)	unprognostic (1.71e-1)	unprognostic (2.22e-1)	unprognostic (2.42e-1)	unprognostic (1.10e-1)
unprognostic (1.29e-1)	unprognostic (1.16e-1)	unprognostic (1.82e-1)	unprognostic (8.54e-3)	unprognostic (2.09e-1)	unprognostic (1.19e-1)
unprognostic (1.63e-1)	unprognostic (3.91e-2)	unprognostic (2.17e-2)	unprognostic (6.67e-3)	unprognostic (1.54e-1)	unprognostic (5.10e-2)
unprognostic (6.23e-2)	unprognostic (1.15e-1)	unprognostic (9.00e-3)	unprognostic (1.10e-1)		unprognostic (2.61e-3)
			unprognostic (2.39e-1)		
unprognostic (1.56e-2)	unprognostic (1.49e-1)	unprognostic (2.49e-3)	unprognostic (9.88e-2)	unprognostic (4.31e-2)	unprognostic (1.58e-1)
unprognostic (8.72e-2)	unprognostic (2.27e-2)	unprognostic (1.19e-2)	unprognostic (9.94e-2)	unprognostic (3.59e-2)	potential prognostic unfavorable (1.16e-4)
potential prognostic unfavorable (7.45e-4)	unprognostic (3.97e-1)	unprognostic (2.46e-1)	unprognostic (1.14e-1)	unprognostic (2.05e-1)	unprognostic (6.07e-2)
unprognostic (4.66e-3)	unprognostic (2.25e-2)	unprognostic (2.20e-2)	unprognostic (1.60e-1)	unprognostic (1.74e-1)	unprognostic (1.59e-1)

unprognostic (1.08e-1)	unprognostic (1.12e-1)	unprognostic (3.00e-1)	unprognostic (8.96e-3)		unprognostic (1.65e-2)
unprognostic (1.29e-1)	unprognostic (2.27e-1)	unprognostic (1.35e-2)	unprognostic (4.53e-1)	unprognostic (3.02e-1)	unprognostic (1.02e-1)
unprognostic (5.64e-3)	unprognostic (1.19e-1)	unprognostic (2.41e-3)	unprognostic (1.64e-3)		unprognostic (1.41e-1)
unprognostic (2.60e-3)	unprognostic (2.51e-1)	unprognostic (2.42e-2)	unprognostic (1.28e-2)	unprognostic (6.95e-2)	unprognostic (1.07e-1)
unprognostic (1.02e-3)	unprognostic (8.02e-2)	unprognostic (1.78e-1)	unprognostic (3.41e-1)	unprognostic (1.10e-2)	unprognostic (5.69e-3)
unprognostic (3.57e-1)	unprognostic (3.65e-2)	unprognostic (1.98e-2)	unprognostic (1.57e-2)	unprognostic (1.37e-1)	unprognostic (3.34e-1)
unprognostic (1.78e-2)	unprognostic (2.11e-1)	unprognostic (8.76e-2)	unprognostic (2.70e-1)	unprognostic (1.62e-1)	unprognostic (4.51e-3)
unprognostic (2.57e-2)	unprognostic (2.18e-2)	unprognostic (1.70e-2)	unprognostic (1.38e-2)		unprognostic (2.43e-1)
unprognostic (1.07e-2)	unprognostic (2.57e-1)	unprognostic (3.42e-2)	unprognostic (1.34e-2)	unprognostic (1.26e-2)	potential prognostic favorable (6.79e-4)
unprognostic (1.75e-1)	unprognostic (4.08e-2)		unprognostic (4.22e-2)		
unprognostic (5.55e-2)	unprognostic (4.96e-1)	unprognostic (4.24e-2)	unprognostic (2.91e-2)	unprognostic (2.93e-1)	unprognostic (2.59e-3)
unprognostic (3.45e-2)	unprognostic (8.75e-2)	unprognostic (4.83e-2)	unprognostic (8.34e-3)	unprognostic (3.74e-1)	unprognostic (5.56e-2)
unprognostic (3.33e-2)	unprognostic (1.61e-1)	unprognostic (1.88e-2)	unprognostic (2.17e-2)	unprognostic (9.72e-2)	unprognostic (4.40e-3)
unprognostic (1.36e-2)	unprognostic (1.37e-1)	unprognostic (6.17e-2)	unprognostic (3.55e-2)	unprognostic (2.86e-2)	unprognostic (4.95e-2)
unprognostic (1.74e-2)	unprognostic (6.28e-2)	unprognostic (5.52e-2)	unprognostic (1.38e-1)	unprognostic (4.72e-2)	unprognostic (7.70e-2)
unprognostic (9.50e-2)	unprognostic (7.96e-2)	unprognostic (2.05e-1)	potential prognostic favorable (5.97e-4)	unprognostic (7.71e-2)	unprognostic (1.98e-1)
unprognostic (2.73e-1)	unprognostic (1.34e-1)	unprognostic (8.76e-2)	unprognostic (2.83e-3)	unprognostic (5.87e-3)	unprognostic (2.71e-2)
unprognostic (4.51e-2)	unprognostic (7.37e-2)	unprognostic (3.81e-3)	unprognostic (1.41e-1)	unprognostic (3.03e-2)	unprognostic (5.14e-2)
unprognostic (9.76e-3)	unprognostic (1.82e-1)	unprognostic (8.51e-2)	unprognostic (4.16e-2)	unprognostic (2.03e-2)	unprognostic (1.28e-1)
unprognostic (3.05e-2)	unprognostic (2.40e-2)	unprognostic (5.75e-2)	unprognostic (1.88e-2)	unprognostic (3.06e-1)	unprognostic (1.48e-1)
unprognostic (1.86e-2)	unprognostic (2.43e-1)	unprognostic (1.82e-1)	unprognostic (1.14e-2)	unprognostic (2.55e-3)	unprognostic (3.09e-2)
potential prognostic unfavorable (1.19e-4)	unprognostic (4.95e-1)	unprognostic (3.74e-1)	unprognostic (4.04e-2)	unprognostic (6.03e-2)	potential prognostic unfavorable (4.77e-4)
unprognostic (1.60e-1)	unprognostic (4.87e-2)	unprognostic (9.48e-3)	unprognostic (1.91e-1)	unprognostic (5.79e-2)	unprognostic (5.27e-2)
unprognostic (6.24e-3)	unprognostic (1.42e-1)	unprognostic (2.44e-1)	unprognostic (1.44e-1)	unprognostic (1.51e-1)	unprognostic (2.88e-1)
unprognostic (1.52e-2)	unprognostic (2.98e-2)	unprognostic (1.28e-2)	unprognostic (2.58e-1)	unprognostic (3.70e-2)	unprognostic (2.12e-2)
		unprognostic (4.65e-2)	unprognostic (2.12e-1)		unprognostic (3.16e-1)
unprognostic (2.47e-3)	unprognostic (2.26e-1)	unprognostic (3.54e-2)	unprognostic (1.23e-1)	unprognostic (6.75e-2)	unprognostic (2.32e-1)
unprognostic (3.98e-2)	unprognostic (1.73e-1)	potential prognostic unfavorable (1.51e-4)	unprognostic (1.84e-1)	unprognostic (4.28e-2)	unprognostic (1.04e-3)
unprognostic (7.66e-3)	unprognostic (2.66e-1)	unprognostic (4.24e-2)	unprognostic (2.08e-1)	unprognostic (1.35e-1)	unprognostic (8.61e-2)
unprognostic (8.75e-2)	unprognostic (5.99e-2)	unprognostic (1.57e-1)	unprognostic (5.02e-2)	unprognostic (2.01e-1)	unprognostic (5.09e-3)
unprognostic (4.77e-2)	unprognostic (8.06e-2)	unprognostic (8.14e-2)	unprognostic (2.13e-1)	unprognostic (6.59e-2)	unprognostic (1.03e-1)
unprognostic (2.96e-1)	unprognostic (3.99e-2)	unprognostic (3.59e-2)	unprognostic (8.98e-2)	unprognostic (4.04e-1)	unprognostic (5.20e-2)
unprognostic (3.47e-2)	unprognostic (1.35e-1)	unprognostic (7.80e-3)	unprognostic (5.64e-2)	unprognostic (3.48e-1)	unprognostic (2.86e-2)
unprognostic (2.36e-1)	unprognostic (7.62e-2)	unprognostic (1.53e-2)	unprognostic (5.11e-2)	unprognostic (2.17e-1)	unprognostic (3.62e-2)
unprognostic (1.05e-1)	unprognostic (3.21e-1)	unprognostic (6.16e-3)	unprognostic (3.04e-1)	unprognostic (3.96e-2)	unprognostic (1.35e-1)
unprognostic (7.50e-2)	unprognostic (1.78e-2)	unprognostic (1.82e-2)	unprognostic (3.34e-1)	unprognostic (4.77e-1)	unprognostic (3.88e-1)
unprognostic (1.01e-2)	unprognostic (2.57e-2)	unprognostic (1.84e-1)	unprognostic (3.22e-1)	unprognostic (1.93e-1)	unprognostic (5.20e-2)
potential prognostic favorable (1.76e-4)	unprognostic (6.06e-2)	unprognostic (8.70e-2)	unprognostic (4.76e-2)	unprognostic (1.17e-3)	unprognostic (7.28e-3)
unprognostic (3.26e-1)	unprognostic (3.51e-1)	unprognostic (4.11e-1)	unprognostic (1.58e-1)	unprognostic (2.05e-1)	unprognostic (1.43e-1)
		unprognostic (3.16e-1)	potential prognostic favorable (6.68e-4)		unprognostic (8.49e-2)
unprognostic (4.51e-2)	unprognostic (1.25e-1)	unprognostic (1.30e-1)	unprognostic (1.18e-1)	unprognostic (8.48e-2)	unprognostic (6.97e-2)
unprognostic (2.02e-1)	unprognostic (8.47e-2)	unprognostic (1.55e-2)	unprognostic (1.09e-2)	unprognostic (2.57e-1)	unprognostic (1.18e-2)
unprognostic (2.45e-1)	unprognostic (3.01e-2)	unprognostic (6.15e-2)	unprognostic (6.56e-2)		unprognostic (1.17e-2)
unprognostic (1.71e-2)	unprognostic (1.06e-1)	unprognostic (2.77e-2)	unprognostic (1.36e-3)	unprognostic (4.85e-2)	unprognostic (7.26e-3)
unprognostic (7.26e-2)	unprognostic (4.79e-3)	unprognostic (4.71e-2)	unprognostic (5.00e-3)	unprognostic (4.74e-1)	unprognostic (6.83e-2)
unprognostic (1.15e-3)		unprognostic (7.22e-3)	unprognostic (5.01e-2)		unprognostic (1.40e-1)
unprognostic (1.10e-2)	unprognostic (1.11e-1)	unprognostic (1.48e-1)	unprognostic (6.37e-2)	unprognostic (1.74e-2)	unprognostic (3.60e-3)
unprognostic (2.08e-1)	unprognostic (9.10e-2)	unprognostic (1.67e-1)	unprognostic (2.87e-1)	unprognostic (1.35e-1)	unprognostic (1.02e-2)
unprognostic (6.15e-2)	unprognostic (1.91e-1)	unprognostic (2.00e-1)	unprognostic (2.75e-3)	unprognostic (2.56e-1)	unprognostic (4.84e-2)
potential prognostic unfavorable (1.56e-4)	unprognostic (1.54e-1)	unprognostic (3.67e-2)	unprognostic (7.24e-3)	unprognostic (1.70e-1)	unprognostic (5.01e-2)
unprognostic (2.56e-3)	unprognostic (9.78e-2)		unprognostic (1.76e-1)	unprognostic (2.44e-2)	unprognostic (5.64e-2)
unprognostic (1.95e-2)	unprognostic (2.95e-1)	unprognostic (3.49e-1)	unprognostic (2.67e-2)	unprognostic (3.10e-1)	unprognostic (1.50e-2)
unprognostic (4.05e-1)	unprognostic (1.46e-1)	unprognostic (1.48e-2)	unprognostic (4.44e-2)	unprognostic (7.58e-3)	unprognostic (1.89e-2)
unprognostic (3.02e-2)	unprognostic (6.66e-2)	unprognostic (9.41e-2)	unprognostic (1.21e-1)		potential prognostic favorable (9.81e-4)
unprognostic (3.84e-1)	unprognostic (8.88e-2)	unprognostic (1.37e-1)	unprognostic (2.94e-2)	unprognostic (3.19e-1)	unprognostic (2.68e-2)
unprognostic (4.37e-3)	unprognostic (3.10e-1)	unprognostic (1.57e-1)	unprognostic (1.14e-3)	unprognostic (2.19e-1)	unprognostic (1.27e-2)
unprognostic (3.08e-1)	unprognostic (2.94e-1)	unprognostic (8.41e-2)	unprognostic (8.50e-2)	unprognostic (3.01e-2)	unprognostic (2.94e-2)
unprognostic (5.14e-2)		unprognostic (3.36e-1)	unprognostic (9.15e-3)		unprognostic (1.04e-1)
unprognostic (1.02e-2)					
unprognostic (1.78e-1)	unprognostic (2.87e-1)	unprognostic (3.11e-2)	unprognostic (2.48e-2)	unprognostic (4.42e-1)	unprognostic (1.78e-1)
potential prognostic unfavorable (7.74e-4)	unprognostic (3.81e-2)	unprognostic (2.04e-1)	unprognostic (1.23e-2)	unprognostic (1.12e-1)	unprognostic (5.41e-2)
unprognostic (2.67e-3)	unprognostic (1.28e-1)	unprognostic (1.14e-1)	unprognostic (1.41e-2)		unprognostic (4.11e-2)
unprognostic (1.41e-2)	unprognostic (1.88e-1)	unprognostic (1.13e-2)	potential prognostic unfavorable (2.37e-5)	unprognostic (4.96e-2)	unprognostic (9.92e-2)
unprognostic (2.07e-1)	unprognostic (1.69e-2)	unprognostic (2.08e-1)	unprognostic (2.81e-1)	unprognostic (1.32e-1)	unprognostic (3.93e-1)
unprognostic (1.16e-1)	unprognostic (1.83e-1)	unprognostic (3.52e-1)	unprognostic (1.98e-1)	unprognostic (2.48e-2)	unprognostic (5.00e-2)
unprognostic (3.60e-1)	unprognostic (1.62e-1)	unprognostic (1.08e-1)	unprognostic (5.58e-2)	unprognostic (1.77e-1)	unprognostic (1.12e-1)
unprognostic (1.19e-2)	unprognostic (1.02e-1)	unprognostic (2.52e-1)	unprognostic (2.04e-1)	unprognostic (2.81e-2)	unprognostic (1.16e-1)
unprognostic (7.30e-3)	unprognostic (1.87e-1)	unprognostic (5.76e-2)	unprognostic (3.82e-2)	unprognostic (7.93e-2)	unprognostic (2.26e-1)
			unprognostic (3.28e-1)		unprognostic (3.44e-3)
potential prognostic unfavorable (9.57e-4)	unprognostic (2.82e-2)	unprognostic (2.49e-1)	unprognostic (7.90e-2)	unprognostic (3.98e-2)	unprognostic (4.83e-2)
potential prognostic unfavorable (4.49e-4)			unprognostic (2.94e-1)		unprognostic (1.71e-1)
	unprognostic (9.00e-2)				
unprognostic (4.07e-3)	unprognostic (5.87e-2)	unprognostic (4.66e-2)	unprognostic (2.05e-1)	unprognostic (1.17e-1)	unprognostic (9.45e-2)
unprognostic (7.47e-2)	unprognostic (1.40e-1)	unprognostic (4.06e-2)	unprognostic (9.70e-3)	unprognostic (7.27e-2)	unprognostic (3.13e-2)
unprognostic (7.96e-2)	unprognostic (1.40e-1)	unprognostic (3.47e-2)	unprognostic (1.88e-3)	unprognostic (7.41e-2)	unprognostic (2.98e-2)
			unprognostic (7.66e-2)		unprognostic (8.59e-3)
potential prognostic unfavorable (5.20e-4)	unprognostic (1.69e-2)	unprognostic (2.10e-3)	unprognostic (4.84e-2)	unprognostic (5.33e-2)	unprognostic (9.69e-2)
unprognostic (5.94e-2)	unprognostic (1.96e-1)	unprognostic (2.79e-2)	unprognostic (1.73e-3)	unprognostic (7.51e-2)	unprognostic (1.29e-1)
unprognostic (5.18e-3)			unprognostic (2.34e-2)		
unprognostic (3.28e-2)	unprognostic (3.17e-1)	unprognostic (6.63e-2)	unprognostic (9.27e-2)	unprognostic (2.03e-1)	unprognostic (2.49e-2)
unprognostic (4.16e-2)	unprognostic (4.79e-2)	unprognostic (3.32e-2)	unprognostic (8.22e-2)	potential prognostic favorable (9.23e-5)	unprognostic (5.80e-2)
validated prognostic unfavorable (9.97e-4)	unprognostic (3.79e-2)	unprognostic (2.32e-1)	unprognostic (6.43e-3)	unprognostic (2.02e-1)	unprognostic (1.83e-1)
unprognostic (4.45e-2)	unprognostic (8.05e-2)	unprognostic (1.70e-1)	unprognostic (1.09e-2)	unprognostic (1.91e-1)	unprognostic (2.24e-1)
unprognostic (4.75e-2)			unprognostic (9.47e-2)		
potential prognostic unfavorable (1.51e-4)	unprognostic (8.69e-2)	unprognostic (8.50e-2)	unprognostic (1.17e-1)	unprognostic (1.10e-1)	unprognostic (1.57e-1)
unprognostic (1.20e-3)	unprognostic (9.14e-2)	unprognostic (1.61e-1)	unprognostic (9.66e-2)	unprognostic (2.09e-1)	unprognostic (2.51e-1)
			unprognostic (1.40e-1)		
unprognostic (1.33e-1)	unprognostic (2.99e-1)	unprognostic (1.32e-2)	unprognostic (2.63e-2)	unprognostic (2.79e-1)	unprognostic (4.08e-2)
unprognostic (3.25e-1)	unprognostic (2.77e-2)	unprognostic (2.89e-1)	unprognostic (2.42e-1)	unprognostic (1.94e-1)	unprognostic (1.46e-1)
unprognostic (2.90e-1)	unprognostic (2.75e-2)	unprognostic (4.36e-2)	unprognostic (6.53e-2)		unprognostic (3.40e-1)
unprognostic (1.03e-1)	unprognostic (2.03e-1)	unprognostic (3.38e-1)	unprognostic (1.08e-1)	unprognostic (2.25e-3)	unprognostic (1.25e-1)
unprognostic (8.22e-2)	unprognostic (2.36e-1)	unprognostic (2.55e-1)	unprognostic (1.80e-1)	unprognostic (1.35e-1)	unprognostic (5.81e-2)
unprognostic (1.23e-1)	unprognostic (3.88e-1)	unprognostic (3.36e-2)	unprognostic (4.83e-3)	unprognostic (2.47e-1)	unprognostic (1.29e-1)
unprognostic (4.18e-3)	unprognostic (7.97e-2)	unprognostic (4.64e-2)	potential prognostic favorable (2.47e-4)	unprognostic (1.19e-1)	unprognostic (1.67e-1)
unprognostic (4.71e-3)	unprognostic (2.65e-1)	unprognostic (6.40e-2)	unprognostic (1.76e-1)	unprognostic (1.70e-2)	unprognostic (2.77e-1)
unprognostic (2.08e-2)	unprognostic (4.98e-2)	unprognostic (2.56e-1)	unprognostic (2.54e-1)	unprognostic (1.06e-3)	unprognostic (5.09e-2)
unprognostic (8.11e-2)	unprognostic (7.08e-2)	unprognostic (3.37e-2)	unprognostic (3.20e-1)	unprognostic (1.42e-2)	unprognostic (1.55e-1)
unprognostic (2.51e-2)	unprognostic (4.42e-1)	unprognostic (1.30e-1)	unprognostic (1.98e-2)	unprognostic (5.78e-2)	unprognostic (1.98e-2)
unprognostic (1.84e-1)	unprognostic (5.62e-2)	unprognostic (9.01e-2)	unprognostic (3.31e-1)	unprognostic (9.92e-2)	unprognostic (8.44e-2)
unprognostic (8.08e-2)	unprognostic (9.84e-2)	unprognostic (2.03e-2)	unprognostic (2.53e-1)	unprognostic (1.10e-1)	unprognostic (2.17e-1)
unprognostic (5.42e-3)	unprognostic (2.10e-2)	unprognostic (1.85e-1)	unprognostic (2.84e-1)	unprognostic (1.17e-1)	unprognostic (1.80e-1)
unprognostic (2.15e-3)	unprognostic (8.67e-2)	unprognostic (1.65e-1)	unprognostic (1.38e-2)	unprognostic (1.34e-2)	unprognostic (4.24e-1)

Cancer prognostics - Testicular Germ Cell Tumor (TCGA)	Cancer prognostics - Thyroid Carcinoma (TCGA)	Cancer prognostics - Uterine Corpus Endometrial Carcinoma (TCGA)
unprognostic (1.11e-1)	unprognostic (8.85e-3)	unprognostic (6.12e-2)
unprognostic (7.45e-2)	unprognostic (2.21e-1)	unprognostic (3.53e-2)
unprognostic (5.47e-2)	unprognostic (2.50e-1)	unprognostic (1.14e-1)
unprognostic (1.29e-1)	unprognostic (1.47e-1)	unprognostic (8.45e-2)
unprognostic (8.59e-2)	unprognostic (1.90e-1)	
unprognostic (2.88e-1)	unprognostic (9.88e-3)	unprognostic (5.17e-2)
unprognostic (7.70e-2)	unprognostic (2.49e-2)	unprognostic (3.06e-1)
unprognostic (1.37e-1)	unprognostic (2.42e-1)	unprognostic (6.76e-2)
unprognostic (1.75e-1)	unprognostic (7.13e-2)	unprognostic (5.03e-2)
unprognostic (2.47e-1)	unprognostic (1.06e-1)	potential prognostic unfavorable (2.79e-5)
unprognostic (9.90e-2)	unprognostic (2.80e-1)	unprognostic (9.01e-2)
unprognostic (1.46e-1)	unprognostic (1.96e-3)	unprognostic (1.57e-1)
unprognostic (1.90e-1)	unprognostic (2.48e-2)	unprognostic (1.41e-3)
unprognostic (2.69e-1)	unprognostic (6.59e-3)	unprognostic (9.67e-3)
unprognostic (2.52e-2)	unprognostic (2.40e-3)	unprognostic (6.36e-3)
unprognostic (7.20e-2)	unprognostic (1.21e-1)	unprognostic (8.78e-3)
unprognostic (1.23e-1)	unprognostic (3.45e-2)	unprognostic (1.13e-2)
	unprognostic (5.23e-2)	
unprognostic (2.74e-1)	unprognostic (3.81e-1)	unprognostic (5.00e-3)
unprognostic (1.49e-1)	unprognostic (1.58e-1)	unprognostic (9.48e-2)
unprognostic (3.29e-1)		unprognostic (7.65e-2)
unprognostic (3.55e-1)	unprognostic (5.54e-2)	unprognostic (3.46e-1)
unprognostic (1.51e-1)	unprognostic (7.14e-2)	unprognostic (2.09e-1)
unprognostic (1.92e-1)	unprognostic (8.13e-3)	unprognostic (1.67e-2)
unprognostic (1.89e-1)	unprognostic (4.53e-2)	unprognostic (1.36e-1)
unprognostic (2.53e-1)	unprognostic (2.72e-1)	unprognostic (8.69e-2)
unprognostic (9.36e-2)	unprognostic (2.44e-2)	unprognostic (2.23e-2)
unprognostic (3.43e-1)		unprognostic (4.79e-2)
	potential prognostic favorable (7.74e-4)	potential prognostic unfavorable (7.17e-4)
unprognostic (5.62e-2)	unprognostic (1.16e-1)	
unprognostic (3.21e-1)	unprognostic (1.27e-2)	unprognostic (1.20e-1)
unprognostic (9.10e-2)	unprognostic (2.14e-1)	
unprognostic (4.96e-2)	unprognostic (5.18e-2)	unprognostic (2.01e-1)
unprognostic (1.39e-1)	unprognostic (1.39e-1)	unprognostic (3.54e-2)
unprognostic (5.22e-1)	unprognostic (3.54e-3)	unprognostic (1.58e-1)
unprognostic (1.46e-1)	unprognostic (2.25e-3)	unprognostic (1.24e-1)
		unprognostic (2.70e-1)
unprognostic (1.63e-1)	unprognostic (2.43e-1)	unprognostic (8.15e-2)
	unprognostic (2.35e-1)	
unprognostic (3.03e-1)	unprognostic (2.61e-2)	
unprognostic (2.77e-1)	unprognostic (1.43e-1)	unprognostic (4.51e-2)
unprognostic (1.79e-1)	unprognostic (1.30e-1)	unprognostic (7.30e-3)
unprognostic (1.75e-1)	unprognostic (1.23e-3)	unprognostic (3.31e-2)
unprognostic (3.01e-1)	unprognostic (2.47e-2)	unprognostic (4.12e-1)
unprognostic (3.10e-1)	unprognostic (5.62e-2)	unprognostic (9.61e-2)
unprognostic (3.43e-1)	unprognostic (1.47e-2)	unprognostic (1.81e-1)
unprognostic (4.98e-1)	unprognostic (1.99e-1)	unprognostic (1.88e-3)
unprognostic (4.32e-2)	unprognostic (1.12e-3)	unprognostic (1.67e-1)
unprognostic (7.55e-2)	unprognostic (1.46e-2)	unprognostic (4.22e-2)
unprognostic (2.38e-1)	unprognostic (1.26e-2)	unprognostic (1.97e-2)
unprognostic (2.20e-1)		unprognostic (1.73e-1)
unprognostic (6.46e-2)	unprognostic (1.91e-1)	potential prognostic unfavorable (7.54e-4)
unprognostic (1.08e-2)	unprognostic (3.55e-2)	unprognostic (6.63e-2)
unprognostic (4.87e-2)	potential prognostic favorable (8.16e-4)	unprognostic (2.84e-2)
unprognostic (2.04e-1)		
unprognostic (2.24e-1)	unprognostic (6.40e-2)	unprognostic (2.71e-2)
unprognostic (5.75e-2)	unprognostic (5.78e-2)	unprognostic (3.22e-2)
unprognostic (4.05e-2)	unprognostic (2.62e-1)	unprognostic (3.21e-1)
unprognostic (7.36e-3)	unprognostic (1.97e-1)	unprognostic (2.40e-1)
unprognostic (3.20e-1)	unprognostic (7.53e-3)	unprognostic (2.04e-1)
unprognostic (6.64e-3)	unprognostic (1.09e-1)	unprognostic (1.01e-1)
unprognostic (1.23e-1)	unprognostic (2.18e-2)	unprognostic (1.28e-2)
unprognostic (2.89e-1)	unprognostic (1.32e-3)	unprognostic (6.53e-2)
unprognostic (3.42e-1)	unprognostic (7.18e-2)	unprognostic (2.61e-1)
unprognostic (2.23e-1)	unprognostic (3.04e-1)	
unprognostic (2.59e-1)	unprognostic (4.26e-2)	unprognostic (7.73e-2)
	unprognostic (9.85e-2)	
unprognostic (1.45e-1)	unprognostic (2.93e-2)	unprognostic (1.75e-1)
unprognostic (1.43e-2)	unprognostic (1.65e-1)	unprognostic (4.48e-3)
unprognostic (3.48e-2)	unprognostic (3.98e-2)	unprognostic (9.04e-2)
unprognostic (1.83e-1)	unprognostic (1.69e-1)	unprognostic (3.10e-2)
unprognostic (3.47e-1)	unprognostic (1.62e-2)	unprognostic (1.18e-1)
unprognostic (3.21e-2)	unprognostic (1.20e-1)	unprognostic (4.16e-2)
unprognostic (8.17e-3)	unprognostic (9.92e-2)	unprognostic (2.59e-1)
unprognostic (1.76e-1)	unprognostic (7.19e-2)	unprognostic (4.24e-2)
unprognostic (1.94e-1)	unprognostic (3.54e-2)	unprognostic (2.33e-1)
unprognostic (1.72e-1)	unprognostic (3.31e-1)	unprognostic (5.76e-2)
unprognostic (2.38e-1)	unprognostic (1.08e-1)	unprognostic (2.29e-2)
unprognostic (2.30e-1)	unprognostic (1.57e-1)	
unprognostic (1.15e-1)	unprognostic (2.02e-1)	unprognostic (4.64e-2)
unprognostic (6.47e-2)	unprognostic (6.22e-2)	unprognostic (1.00e-1)
		unprognostic (2.62e-2)
unprognostic (6.41e-2)	unprognostic (7.70e-2)	unprognostic (7.19e-2)
unprognostic (8.51e-2)	unprognostic (1.83e-2)	unprognostic (4.53e-2)
unprognostic (3.44e-1)	unprognostic (8.00e-2)	unprognostic (4.72e-2)
unprognostic (2.85e-1)	unprognostic (6.50e-2)	unprognostic (6.33e-2)
unprognostic (4.88e-2)		
unprognostic (4.17e-2)	unprognostic (1.61e-1)	unprognostic (1.97e-1)
unprognostic (2.10e-1)		unprognostic (6.29e-2)
unprognostic (1.11e-1)	unprognostic (9.91e-2)	unprognostic (2.05e-1)
unprognostic (8.57e-2)	potential prognostic unfavorable (6.07e-4)	unprognostic (3.10e-3)
unprognostic (1.94e-1)	unprognostic (4.48e-2)	
unprognostic (1.58e-1)	unprognostic (1.05e-2)	unprognostic (6.01e-2)
unprognostic (1.90e-1)	unprognostic (1.16e-1)	unprognostic (1.08e-2)
unprognostic (2.94e-1)	unprognostic (4.82e-2)	unprognostic (1.37e-1)
unprognostic (4.34e-2)	unprognostic (6.28e-2)	unprognostic (4.83e-2)
unprognostic (5.14e-1)	unprognostic (1.47e-1)	unprognostic (2.25e-2)
unprognostic (3.57e-1)	unprognostic (1.36e-1)	unprognostic (8.64e-2)
unprognostic (2.50e-1)	unprognostic (4.05e-2)	unprognostic (1.05e-1)
unprognostic (3.87e-2)	unprognostic (9.23e-3)	unprognostic (3.06e-2)
unprognostic (3.40e-2)	unprognostic (2.76e-2)	unprognostic (1.13e-1)
unprognostic (1.83e-1)	unprognostic (1.32e-1)	unprognostic (1.78e-1)
unprognostic (7.29e-2)	unprognostic (2.01e-1)	unprognostic (3.94e-2)
unprognostic (3.16e-1)	unprognostic (7.67e-2)	unprognostic (3.35e-1)
unprognostic (1.25e-1)	unprognostic (3.55e-2)	unprognostic (3.47e-2)
unprognostic (2.51e-1)	unprognostic (4.02e-2)	unprognostic (1.64e-1)
unprognostic (5.44e-2)	unprognostic (1.54e-2)	unprognostic (3.81e-2)
unprognostic (4.50e-2)	unprognostic (3.65e-2)	unprognostic (1.75e-1)
unprognostic (1.11e-1)	unprognostic (7.03e-3)	unprognostic (2.74e-2)
unprognostic (4.96e-2)	unprognostic (3.15e-2)	unprognostic (4.13e-2)
	unprognostic (8.62e-2)	unprognostic (1.75e-1)
unprognostic (2.51e-1)	unprognostic (1.49e-1)	unprognostic (1.93e-1)
unprognostic (1.41e-1)	unprognostic (1.77e-2)	unprognostic (6.27e-2)
unprognostic (4.51e-3)	unprognostic (4.04e-2)	unprognostic (9.55e-2)
unprognostic (3.04e-1)		unprognostic (2.24e-2)
unprognostic (4.06e-1)	unprognostic (1.22e-3)	unprognostic (4.86e-2)
unprognostic (2.49e-1)	unprognostic (6.52e-2)	unprognostic (1.19e-1)
unprognostic (1.02e-1)	unprognostic (8.34e-2)	potential prognostic favorable (2.66e-4)
unprognostic (1.85e-3)	unprognostic (2.92e-1)	unprognostic (7.99e-2)

unprognostic (1.21e-1)		unprognostic (1.29e-1)
unprognostic (1.10e-1)		
unprognostic (3.04e-1)	unprognostic (9.94e-2)	unprognostic (3.11e-2)
unprognostic (1.13e-1)	unprognostic (6.09e-2)	unprognostic (1.83e-1)
unprognostic (1.33e-1)	unprognostic (8.81e-2)	unprognostic (5.10e-2)
unprognostic (2.64e-1)	unprognostic (2.54e-3)	unprognostic (2.70e-2)
unprognostic (2.95e-1)	unprognostic (1.53e-1)	unprognostic (2.88e-1)
unprognostic (7.65e-2)	unprognostic (4.52e-1)	unprognostic (4.42e-1)
unprognostic (1.09e-1)	unprognostic (2.39e-2)	unprognostic (1.70e-1)
unprognostic (8.69e-2)	unprognostic (2.14e-1)	unprognostic (6.66e-2)
unprognostic (1.78e-1)		
unprognostic (1.34e-1)	unprognostic (2.51e-1)	unprognostic (4.59e-2)
unprognostic (5.70e-2)	unprognostic (1.71e-1)	unprognostic (5.61e-2)
unprognostic (2.56e-1)	unprognostic (2.20e-2)	unprognostic (2.55e-1)
unprognostic (1.54e-1)	unprognostic (3.16e-2)	unprognostic (1.75e-2)
unprognostic (2.38e-1)	unprognostic (2.75e-2)	unprognostic (3.38e-2)
unprognostic (3.24e-1)	unprognostic (4.35e-3)	unprognostic (1.90e-2)
unprognostic (5.48e-1)		unprognostic (3.19e-2)
unprognostic (2.49e-2)	unprognostic (1.26e-1)	unprognostic (2.88e-3)
unprognostic (2.57e-2)	unprognostic (1.22e-1)	
unprognostic (4.17e-3)		unprognostic (1.13e-2)
unprognostic (9.46e-2)	unprognostic (4.12e-1)	unprognostic (2.91e-1)
unprognostic (1.86e-1)	unprognostic (1.31e-1)	unprognostic (6.75e-2)
unprognostic (1.07e-2)	unprognostic (1.40e-1)	unprognostic (2.67e-2)
unprognostic (2.83e-2)		unprognostic (3.22e-1)
unprognostic (5.64e-2)	unprognostic (1.52e-1)	unprognostic (1.15e-1)
unprognostic (4.43e-2)		
unprognostic (2.16e-2)	unprognostic (2.49e-1)	
unprognostic (1.46e-2)		
unprognostic (3.24e-1)	unprognostic (7.58e-2)	unprognostic (2.16e-1)
unprognostic (1.19e-3)	unprognostic (6.67e-2)	unprognostic (1.37e-1)
unprognostic (5.92e-2)	unprognostic (1.06e-1)	unprognostic (2.28e-1)
unprognostic (6.85e-2)	unprognostic (1.29e-1)	unprognostic (3.39e-2)
unprognostic (2.46e-2)	unprognostic (1.07e-2)	unprognostic (8.94e-2)
unprognostic (5.52e-3)	unprognostic (5.86e-2)	unprognostic (3.27e-1)
unprognostic (5.05e-2)	unprognostic (6.57e-2)	unprognostic (4.03e-2)
unprognostic (2.64e-2)		
unprognostic (1.25e-1)	unprognostic (6.97e-2)	unprognostic (8.74e-2)
unprognostic (2.16e-1)	potential prognostic favorable (4.20e-4)	unprognostic (2.13e-2)
unprognostic (7.89e-2)	unprognostic (1.05e-1)	unprognostic (2.47e-1)
unprognostic (1.93e-2)	unprognostic (1.90e-1)	unprognostic (2.24e-1)
unprognostic (1.03e-2)	unprognostic (7.84e-2)	unprognostic (2.94e-1)
unprognostic (4.00e-2)	unprognostic (4.95e-2)	unprognostic (2.65e-1)
unprognostic (2.80e-2)	unprognostic (6.73e-2)	unprognostic (2.73e-1)
unprognostic (2.18e-1)	unprognostic (5.90e-3)	unprognostic (1.05e-1)
unprognostic (3.58e-1)	unprognostic (2.75e-1)	unprognostic (2.46e-1)
unprognostic (1.20e-1)	unprognostic (5.75e-2)	unprognostic (1.19e-1)
unprognostic (1.81e-1)	unprognostic (6.41e-2)	unprognostic (3.37e-2)
unprognostic (1.44e-1)	unprognostic (1.64e-1)	unprognostic (2.14e-1)
unprognostic (3.60e-1)	unprognostic (1.22e-1)	unprognostic (8.51e-2)
unprognostic (9.13e-2)	unprognostic (8.74e-2)	unprognostic (2.69e-2)
unprognostic (1.01e-1)		unprognostic (3.89e-2)
unprognostic (9.80e-2)	unprognostic (1.48e-1)	unprognostic (4.22e-2)
unprognostic (4.73e-1)	unprognostic (3.62e-3)	unprognostic (1.46e-2)
	unprognostic (2.17e-1)	
unprognostic (9.63e-2)	unprognostic (1.07e-1)	unprognostic (1.16e-2)
unprognostic (7.69e-2)	unprognostic (2.04e-1)	unprognostic (7.48e-2)
unprognostic (1.74e-1)	unprognostic (6.09e-2)	unprognostic (4.52e-2)
unprognostic (2.83e-1)	unprognostic (3.74e-2)	unprognostic (2.54e-2)
unprognostic (1.44e-1)		unprognostic (1.31e-1)
		unprognostic (6.80e-2)
unprognostic (1.75e-1)	unprognostic (8.27e-2)	unprognostic (4.10e-3)
unprognostic (1.84e-1)	unprognostic (7.57e-3)	unprognostic (1.03e-1)
unprognostic (1.45e-1)	unprognostic (1.69e-1)	unprognostic (8.77e-2)
unprognostic (1.83e-1)	unprognostic (1.91e-1)	unprognostic (1.72e-1)
unprognostic (3.79e-1)	unprognostic (6.20e-3)	unprognostic (4.13e-2)
unprognostic (2.15e-1)	unprognostic (8.61e-2)	unprognostic (2.56e-1)
unprognostic (2.44e-1)	unprognostic (3.08e-2)	unprognostic (1.16e-3)
		unprognostic (6.80e-2)
unprognostic (3.56e-1)		
unprognostic (3.17e-1)		unprognostic (9.68e-3)
unprognostic (1.67e-1)		
unprognostic (2.19e-1)	unprognostic (2.54e-1)	unprognostic (3.36e-1)
unprognostic (1.59e-1)	unprognostic (6.58e-2)	unprognostic (2.11e-1)
unprognostic (2.46e-1)		
unprognostic (9.88e-2)		unprognostic (2.05e-2)
unprognostic (1.52e-1)	potential prognostic favorable (2.00e-4)	unprognostic (6.34e-2)
unprognostic (2.60e-1)	unprognostic (1.58e-2)	unprognostic (9.35e-3)
unprognostic (1.55e-1)	unprognostic (1.14e-1)	unprognostic (1.03e-1)
unprognostic (2.00e-1)	potential prognostic favorable (1.11e-4)	unprognostic (3.85e-2)
unprognostic (2.62e-1)	unprognostic (6.43e-3)	unprognostic (1.19e-3)
unprognostic (1.55e-1)	unprognostic (9.16e-2)	unprognostic (7.67e-2)
unprognostic (4.31e-2)	unprognostic (9.62e-2)	unprognostic (8.64e-2)
unprognostic (3.98e-1)	unprognostic (3.13e-3)	unprognostic (1.43e-1)
	unprognostic (2.58e-2)	unprognostic (2.32e-2)
unprognostic (1.06e-1)	unprognostic (2.11e-3)	unprognostic (2.42e-1)
unprognostic (1.36e-1)	unprognostic (7.14e-2)	unprognostic (4.77e-2)
unprognostic (1.78e-1)	unprognostic (1.87e-2)	unprognostic (9.43e-2)
unprognostic (1.23e-1)	unprognostic (4.03e-3)	unprognostic (1.56e-1)
unprognostic (3.13e-3)		
unprognostic (9.75e-2)	unprognostic (1.51e-1)	unprognostic (3.92e-2)
unprognostic (1.47e-1)	unprognostic (3.41e-2)	unprognostic (2.28e-1)
unprognostic (1.06e-1)	unprognostic (5.15e-3)	unprognostic (4.34e-2)
unprognostic (1.66e-1)	unprognostic (4.45e-2)	unprognostic (1.80e-1)
unprognostic (1.83e-1)	potential prognostic unfavorable (2.64e-4)	unprognostic (4.54e-1)
unprognostic (2.49e-2)		
unprognostic (2.54e-1)	unprognostic (4.42e-2)	unprognostic (1.87e-2)
unprognostic (1.61e-1)	unprognostic (2.22e-1)	unprognostic (1.15e-2)
unprognostic (3.04e-2)	unprognostic (2.72e-1)	unprognostic (4.02e-2)
potential prognostic unfavorable (5.12e-4)	unprognostic (3.95e-3)	unprognostic (2.40e-1)
unprognostic (2.45e-1)	unprognostic (3.37e-2)	unprognostic (2.27e-1)
unprognostic (1.76e-1)	unprognostic (2.08e-1)	unprognostic (1.13e-1)
unprognostic (2.73e-1)	unprognostic (1.60e-1)	unprognostic (4.10e-3)
unprognostic (1.76e-1)	unprognostic (3.24e-1)	unprognostic (3.34e-1)
unprognostic (3.54e-1)	unprognostic (1.59e-1)	unprognostic (3.18e-1)
unprognostic (1.45e-1)	unprognostic (4.44e-2)	unprognostic (2.95e-2)
unprognostic (1.53e-1)	unprognostic (7.14e-3)	unprognostic (8.43e-2)
unprognostic (2.08e-1)	unprognostic (3.23e-2)	unprognostic (7.93e-3)
unprognostic (7.58e-2)	unprognostic (2.74e-2)	unprognostic (2.15e-1)
unprognostic (3.87e-2)	unprognostic (8.84e-2)	unprognostic (8.60e-2)
unprognostic (2.30e-2)	unprognostic (2.09e-2)	unprognostic (4.03e-2)
unprognostic (1.34e-2)	unprognostic (2.92e-2)	unprognostic (1.07e-1)
unprognostic (1.75e-1)	unprognostic (1.47e-2)	unprognostic (1.66e-1)
unprognostic (2.01e-1)	unprognostic (2.09e-2)	unprognostic (2.16e-1)
unprognostic (1.57e-1)	unprognostic (1.07e-1)	unprognostic (6.15e-2)
unprognostic (2.59e-1)	unprognostic (3.20e-2)	unprognostic (4.63e-2)

unprognostic (6.62e-2)	unprognostic (3.10e-2)	unprognostic (1.49e-1)
unprognostic (1.77e-1)	unprognostic (5.30e-2)	unprognostic (6.00e-2)
unprognostic (1.65e-1)		unprognostic (4.65e-2)
unprognostic (2.03e-1)	unprognostic (6.30e-2)	unprognostic (8.83e-2)
unprognostic (3.81e-1)	unprognostic (4.44e-2)	unprognostic (3.14e-1)
unprognostic (1.21e-1)	unprognostic (9.84e-2)	unprognostic (3.21e-1)
unprognostic (3.24e-1)	unprognostic (1.28e-1)	unprognostic (1.48e-1)
unprognostic (4.72e-1)	unprognostic (1.29e-3)	unprognostic (3.62e-2)
unprognostic (1.69e-1)	unprognostic (2.34e-1)	unprognostic (1.39e-1)
unprognostic (2.26e-1)	unprognostic (8.66e-2)	unprognostic (1.56e-1)
unprognostic (2.72e-2)	unprognostic (1.37e-1)	unprognostic (1.21e-2)
unprognostic (4.55e-3)	unprognostic (1.93e-1)	unprognostic (2.10e-1)
unprognostic (1.06e-1)	unprognostic (2.65e-1)	unprognostic (6.82e-2)
unprognostic (2.91e-1)		unprognostic (1.39e-1)
unprognostic (1.46e-1)	unprognostic (1.44e-1)	unprognostic (7.58e-2)
unprognostic (1.69e-1)	unprognostic (3.87e-1)	
unprognostic (1.93e-1)	unprognostic (1.26e-1)	unprognostic (1.66e-1)
unprognostic (2.10e-1)	unprognostic (4.20e-2)	unprognostic (2.69e-1)
unprognostic (1.32e-1)	unprognostic (2.56e-2)	unprognostic (9.58e-3)
unprognostic (5.27e-2)	unprognostic (6.90e-2)	
unprognostic (2.39e-1)	unprognostic (1.75e-1)	unprognostic (1.34e-1)
unprognostic (1.32e-2)	unprognostic (1.80e-2)	unprognostic (1.97e-2)
unprognostic (8.02e-2)	unprognostic (4.03e-2)	unprognostic (1.84e-1)
unprognostic (8.47e-2)	unprognostic (8.20e-2)	unprognostic (6.54e-2)
unprognostic (1.84e-1)	unprognostic (1.20e-1)	unprognostic (3.74e-3)
unprognostic (7.41e-2)	unprognostic (1.53e-1)	unprognostic (2.60e-2)
unprognostic (1.52e-1)	unprognostic (4.14e-2)	unprognostic (9.81e-2)
unprognostic (1.57e-1)	unprognostic (2.85e-2)	unprognostic (1.04e-2)
unprognostic (4.98e-2)	unprognostic (5.34e-2)	unprognostic (2.08e-1)
unprognostic (7.03e-2)	unprognostic (5.73e-2)	unprognostic (1.30e-1)
unprognostic (1.63e-1)	unprognostic (5.07e-3)	unprognostic (2.26e-2)
unprognostic (2.79e-1)	unprognostic (7.22e-2)	unprognostic (4.20e-2)
unprognostic (1.62e-1)	unprognostic (2.49e-2)	unprognostic (1.58e-1)
unprognostic (8.01e-2)	unprognostic (2.22e-1)	unprognostic (4.65e-2)
unprognostic (2.82e-1)	unprognostic (2.81e-1)	unprognostic (6.99e-2)
unprognostic (3.58e-1)	unprognostic (1.14e-2)	unprognostic (9.17e-2)
unprognostic (1.18e-1)	unprognostic (2.77e-2)	unprognostic (3.94e-1)
unprognostic (8.81e-2)		
unprognostic (4.58e-2)	unprognostic (9.89e-2)	unprognostic (1.16e-1)
unprognostic (2.56e-1)	unprognostic (2.65e-1)	unprognostic (7.94e-2)
unprognostic (3.31e-1)	unprognostic (1.22e-1)	unprognostic (3.79e-1)
unprognostic (2.05e-1)	unprognostic (8.77e-3)	unprognostic (7.64e-2)
unprognostic (5.07e-1)	unprognostic (2.22e-2)	unprognostic (2.00e-1)
unprognostic (7.93e-2)	unprognostic (3.56e-2)	unprognostic (6.07e-2)
unprognostic (1.31e-1)	unprognostic (1.95e-2)	unprognostic (2.45e-1)
unprognostic (2.55e-2)	unprognostic (5.56e-2)	
unprognostic (7.85e-2)	unprognostic (1.77e-3)	unprognostic (5.17e-2)
unprognostic (6.19e-2)	unprognostic (1.31e-2)	unprognostic (7.58e-2)
unprognostic (2.14e-1)	unprognostic (1.20e-1)	unprognostic (9.21e-2)
unprognostic (2.00e-1)	unprognostic (1.04e-1)	unprognostic (1.76e-1)
unprognostic (1.67e-1)	unprognostic (1.77e-2)	unprognostic (6.29e-2)
unprognostic (3.56e-1)	unprognostic (9.50e-2)	unprognostic (2.34e-1)
unprognostic (8.72e-2)	unprognostic (1.18e-3)	unprognostic (2.34e-1)
unprognostic (3.45e-2)	unprognostic (3.35e-2)	unprognostic (6.49e-2)
unprognostic (2.54e-1)	unprognostic (2.79e-1)	unprognostic (2.72e-1)
unprognostic (2.07e-1)	unprognostic (1.22e-1)	unprognostic (1.21e-1)
unprognostic (1.01e-1)	unprognostic (2.92e-1)	unprognostic (4.51e-2)
unprognostic (2.98e-2)	unprognostic (5.49e-2)	unprognostic (3.10e-1)
unprognostic (2.00e-1)	unprognostic (1.73e-2)	unprognostic (5.37e-2)
unprognostic (1.55e-1)	unprognostic (9.54e-3)	unprognostic (1.04e-1)
unprognostic (7.55e-2)	unprognostic (9.69e-3)	unprognostic (6.72e-2)
unprognostic (7.51e-3)	unprognostic (5.60e-2)	unprognostic (1.35e-1)
unprognostic (2.36e-2)	unprognostic (1.91e-1)	unprognostic (4.06e-1)
unprognostic (1.73e-1)	unprognostic (5.27e-2)	unprognostic (2.35e-1)
unprognostic (5.53e-2)	unprognostic (2.90e-2)	unprognostic (1.14e-1)
unprognostic (4.07e-2)	unprognostic (1.19e-2)	unprognostic (1.98e-1)
unprognostic (2.17e-1)	unprognostic (1.41e-2)	unprognostic (1.57e-1)
unprognostic (1.57e-1)	unprognostic (2.79e-3)	unprognostic (4.02e-2)
unprognostic (3.51e-1)	unprognostic (1.03e-1)	unprognostic (2.95e-1)
unprognostic (1.44e-1)	unprognostic (2.07e-1)	unprognostic (3.32e-1)
unprognostic (2.02e-1)	unprognostic (1.90e-1)	unprognostic (1.64e-1)
unprognostic (9.41e-2)	unprognostic (6.36e-3)	unprognostic (8.84e-3)
unprognostic (6.21e-2)	unprognostic (7.24e-2)	unprognostic (6.25e-3)
unprognostic (1.73e-1)	unprognostic (1.09e-1)	unprognostic (4.47e-2)
unprognostic (1.00e-1)	unprognostic (4.40e-1)	unprognostic (2.20e-1)
unprognostic (1.97e-1)	unprognostic (3.00e-2)	unprognostic (1.84e-1)
unprognostic (6.49e-3)	unprognostic (2.81e-1)	unprognostic (8.47e-2)
unprognostic (6.62e-2)	unprognostic (9.55e-2)	unprognostic (8.60e-2)
unprognostic (6.87e-2)	unprognostic (1.48e-2)	unprognostic (1.92e-1)
unprognostic (4.17e-2)	unprognostic (2.44e-1)	unprognostic (2.66e-2)
unprognostic (1.98e-1)	unprognostic (1.94e-1)	unprognostic (3.56e-1)
unprognostic (3.67e-1)	unprognostic (4.62e-2)	unprognostic (2.64e-1)
unprognostic (2.95e-1)	unprognostic (2.41e-1)	unprognostic (3.19e-2)
unprognostic (1.84e-1)	unprognostic (4.55e-2)	unprognostic (1.47e-1)
unprognostic (4.71e-2)		
	unprognostic (2.18e-2)	unprognostic (1.51e-1)
unprognostic (6.91e-2)	unprognostic (2.79e-2)	
unprognostic (1.08e-1)	unprognostic (1.38e-1)	unprognostic (2.95e-3)
unprognostic (1.15e-2)	unprognostic (6.80e-2)	
unprognostic (1.93e-1)	unprognostic (2.97e-2)	unprognostic (2.31e-1)
unprognostic (6.90e-2)	unprognostic (1.13e-1)	unprognostic (1.22e-1)
unprognostic (4.56e-1)		unprognostic (1.95e-2)
unprognostic (1.43e-1)	potential prognostic unfavorable (6.29e-4)	
unprognostic (1.74e-1)	unprognostic (3.50e-2)	unprognostic (7.38e-2)
unprognostic (3.41e-1)	unprognostic (4.44e-2)	unprognostic (1.91e-1)
unprognostic (1.51e-1)	unprognostic (1.57e-2)	unprognostic (2.33e-1)
	unprognostic (2.97e-3)	
unprognostic (2.21e-1)	unprognostic (3.41e-3)	unprognostic (1.66e-2)
unprognostic (1.46e-1)	unprognostic (6.46e-2)	unprognostic (5.91e-2)
unprognostic (8.03e-2)	unprognostic (2.51e-1)	unprognostic (3.13e-2)
	unprognostic (5.63e-3)	unprognostic (2.07e-1)
unprognostic (2.10e-1)	unprognostic (5.86e-2)	unprognostic (1.29e-1)
	unprognostic (2.28e-2)	
unprognostic (5.15e-2)	unprognostic (3.37e-1)	unprognostic (1.80e-1)
unprognostic (2.73e-1)	unprognostic (4.26e-2)	unprognostic (2.83e-1)
unprognostic (4.25e-1)	unprognostic (9.25e-2)	unprognostic (2.64e-1)
unprognostic (1.62e-1)	unprognostic (1.71e-1)	unprognostic (9.88e-2)
unprognostic (1.35e-2)	unprognostic (1.07e-3)	unprognostic (1.99e-3)
unprognostic (1.12e-1)	unprognostic (1.84e-1)	unprognostic (1.68e-2)
unprognostic (2.50e-1)	unprognostic (1.80e-1)	unprognostic (1.10e-1)
unprognostic (1.96e-1)	unprognostic (4.26e-2)	unprognostic (5.23e-3)
unprognostic (7.01e-2)		
unprognostic (2.97e-3)	unprognostic (5.62e-3)	unprognostic (2.58e-1)

		unprognostic (6.30e-2)
unprognostic (7.53e-2)		
	unprognostic (2.64e-1)	
unprognostic (2.03e-1)	unprognostic (3.21e-1)	unprognostic (9.15e-2)
unprognostic (3.83e-2)	unprognostic (2.45e-1)	unprognostic (1.85e-1)
unprognostic (8.79e-2)		unprognostic (4.45e-2)
unprognostic (6.78e-2)	unprognostic (1.67e-1)	unprognostic (1.35e-1)
unprognostic (6.18e-2)	unprognostic (1.67e-1)	unprognostic (6.70e-2)
unprognostic (4.27e-2)	unprognostic (3.55e-3)	unprognostic (4.74e-3)
unprognostic (1.12e-1)	unprognostic (3.87e-2)	unprognostic (1.46e-1)
unprognostic (1.92e-1)	unprognostic (2.51e-3)	unprognostic (1.22e-1)
unprognostic (2.40e-1)		
unprognostic (3.14e-2)	unprognostic (5.13e-2)	unprognostic (8.72e-2)
unprognostic (4.77e-1)		unprognostic (1.54e-1)
unprognostic (1.48e-1)	unprognostic (1.85e-1)	unprognostic (1.53e-1)
unprognostic (1.93e-1)	unprognostic (3.08e-1)	unprognostic (2.00e-1)
unprognostic (1.29e-1)	unprognostic (1.19e-1)	
unprognostic (4.50e-2)	unprognostic (1.61e-1)	unprognostic (4.94e-2)
unprognostic (4.79e-2)	unprognostic (1.69e-1)	unprognostic (3.13e-1)
unprognostic (2.38e-1)	unprognostic (5.95e-2)	unprognostic (9.67e-2)
unprognostic (2.63e-1)	unprognostic (2.15e-1)	unprognostic (3.21e-1)
unprognostic (3.72e-2)	unprognostic (5.58e-2)	unprognostic (2.52e-2)
unprognostic (4.87e-3)	unprognostic (1.57e-2)	unprognostic (1.86e-2)
unprognostic (2.16e-1)	unprognostic (4.79e-2)	unprognostic (8.83e-2)
unprognostic (8.23e-2)	unprognostic (2.19e-2)	unprognostic (3.57e-2)
	unprognostic (1.66e-1)	unprognostic (7.02e-2)
unprognostic (1.64e-1)	unprognostic (7.39e-2)	unprognostic (2.73e-1)
unprognostic (2.14e-1)	unprognostic (1.22e-1)	unprognostic (1.62e-2)
unprognostic (1.50e-1)		
unprognostic (1.07e-1)	unprognostic (3.34e-2)	unprognostic (3.51e-1)
unprognostic (8.43e-2)	unprognostic (4.15e-3)	unprognostic (4.39e-2)
unprognostic (2.51e-1)	unprognostic (1.36e-2)	unprognostic (1.15e-1)
unprognostic (1.36e-1)	unprognostic (2.87e-1)	unprognostic (1.70e-1)
unprognostic (2.37e-2)	unprognostic (1.25e-1)	unprognostic (2.78e-1)
unprognostic (6.49e-2)	unprognostic (1.03e-1)	unprognostic (2.50e-1)
unprognostic (1.21e-1)		
unprognostic (6.69e-2)	unprognostic (1.35e-1)	unprognostic (1.42e-1)
unprognostic (3.04e-1)	unprognostic (6.77e-2)	unprognostic (4.48e-3)
unprognostic (1.73e-1)	unprognostic (3.01e-1)	unprognostic (8.72e-2)
unprognostic (4.26e-3)	unprognostic (2.18e-1)	unprognostic (2.53e-1)
unprognostic (2.85e-2)	unprognostic (1.11e-1)	unprognostic (2.54e-2)
unprognostic (1.10e-1)	unprognostic (3.78e-1)	unprognostic (1.59e-1)
	unprognostic (7.56e-2)	
unprognostic (4.79e-2)	unprognostic (6.17e-2)	unprognostic (2.32e-2)
unprognostic (2.80e-1)	unprognostic (1.69e-1)	unprognostic (5.20e-2)
unprognostic (4.99e-1)	unprognostic (3.20e-1)	unprognostic (1.40e-1)
unprognostic (2.03e-2)	unprognostic (8.41e-2)	unprognostic (5.78e-2)
unprognostic (6.88e-2)	unprognostic (1.76e-1)	unprognostic (1.91e-1)
unprognostic (3.73e-2)	unprognostic (6.24e-2)	unprognostic (6.38e-2)
unprognostic (2.48e-1)	unprognostic (1.45e-1)	unprognostic (2.40e-2)
unprognostic (5.05e-2)	unprognostic (2.49e-1)	unprognostic (2.62e-1)
unprognostic (3.02e-1)	unprognostic (9.42e-2)	unprognostic (1.39e-1)
unprognostic (2.13e-2)	unprognostic (1.74e-1)	unprognostic (1.08e-2)
unprognostic (3.24e-1)	unprognostic (2.20e-1)	unprognostic (3.83e-2)
unprognostic (3.27e-2)	unprognostic (1.10e-1)	unprognostic (1.47e-1)
unprognostic (1.89e-1)	unprognostic (2.28e-3)	unprognostic (1.69e-1)
	unprognostic (2.23e-2)	
unprognostic (2.23e-1)	unprognostic (1.34e-1)	unprognostic (1.53e-2)
unprognostic (4.25e-2)	unprognostic (1.49e-2)	unprognostic (1.93e-1)
unprognostic (1.15e-1)	unprognostic (1.46e-1)	unprognostic (1.50e-1)
unprognostic (3.14e-2)	unprognostic (2.47e-1)	unprognostic (3.64e-2)
unprognostic (1.61e-1)	unprognostic (2.06e-1)	unprognostic (1.53e-1)
unprognostic (9.64e-2)	unprognostic (2.63e-1)	unprognostic (4.42e-2)
unprognostic (1.67e-1)		
unprognostic (1.07e-1)	unprognostic (8.77e-2)	unprognostic (5.98e-2)
unprognostic (1.11e-1)	unprognostic (1.18e-1)	unprognostic (3.22e-1)
	unprognostic (2.14e-1)	unprognostic (1.35e-1)
unprognostic (2.42e-1)	unprognostic (1.29e-1)	unprognostic (4.47e-3)
unprognostic (1.75e-2)	unprognostic (1.29e-1)	unprognostic (4.41e-2)
unprognostic (3.33e-1)		
unprognostic (2.78e-1)	unprognostic (2.52e-1)	unprognostic (4.17e-2)
unprognostic (1.52e-1)		
unprognostic (2.64e-1)	unprognostic (2.08e-1)	unprognostic (2.79e-1)
		unprognostic (7.77e-2)
unprognostic (7.41e-2)	unprognostic (1.10e-2)	unprognostic (3.71e-1)
unprognostic (1.59e-1)	unprognostic (1.38e-2)	unprognostic (2.45e-1)
unprognostic (3.75e-1)	unprognostic (1.38e-2)	unprognostic (8.22e-2)
unprognostic (2.90e-2)	unprognostic (5.00e-3)	unprognostic (1.50e-1)
unprognostic (2.62e-1)		unprognostic (3.16e-3)
unprognostic (1.05e-2)		unprognostic (2.08e-2)
unprognostic (1.94e-1)	unprognostic (1.38e-1)	unprognostic (7.74e-2)
unprognostic (4.69e-2)	unprognostic (1.20e-1)	unprognostic (2.63e-1)
unprognostic (5.16e-2)	unprognostic (1.64e-1)	unprognostic (2.47e-2)
unprognostic (1.37e-3)	unprognostic (1.81e-1)	unprognostic (1.26e-2)
unprognostic (1.02e-1)		
unprognostic (9.42e-2)	unprognostic (7.22e-2)	unprognostic (2.50e-1)
unprognostic (3.69e-1)	unprognostic (3.10e-2)	unprognostic (4.15e-2)
unprognostic (2.88e-1)	unprognostic (1.38e-2)	unprognostic (2.30e-1)
potential prognostic unfavorable (4.73e-4)	unprognostic (1.37e-2)	unprognostic (7.99e-2)
unprognostic (5.36e-2)	unprognostic (1.27e-1)	unprognostic (8.50e-2)
unprognostic (2.23e-2)		unprognostic (3.76e-2)
unprognostic (1.36e-1)		
unprognostic (1.17e-1)	unprognostic (1.65e-2)	unprognostic (2.10e-1)
unprognostic (5.49e-2)	unprognostic (1.01e-3)	unprognostic (1.13e-1)
unprognostic (1.35e-1)	unprognostic (1.58e-2)	unprognostic (1.48e-1)
		unprognostic (9.70e-2)
unprognostic (2.83e-1)	unprognostic (1.01e-1)	unprognostic (3.08e-1)
unprognostic (1.22e-1)	unprognostic (1.97e-1)	unprognostic (2.58e-1)
unprognostic (2.09e-1)		
unprognostic (3.34e-3)	unprognostic (2.78e-1)	unprognostic (2.74e-1)
unprognostic (2.38e-1)	unprognostic (1.88e-1)	unprognostic (1.44e-1)
unprognostic (1.90e-1)	unprognostic (2.76e-1)	unprognostic (3.84e-3)
unprognostic (1.76e-1)	unprognostic (3.12e-2)	unprognostic (2.39e-3)
unprognostic (2.72e-1)	unprognostic (2.21e-1)	unprognostic (3.03e-2)
unprognostic (2.14e-1)	unprognostic (7.83e-2)	unprognostic (1.74e-1)
unprognostic (4.73e-1)	unprognostic (2.87e-1)	unprognostic (1.74e-1)
unprognostic (2.75e-1)	unprognostic (1.35e-2)	unprognostic (1.52e-1)
unprognostic (7.28e-2)		
unprognostic (5.52e-2)	unprognostic (6.03e-2)	unprognostic (9.05e-2)
unprognostic (2.51e-1)	unprognostic (3.25e-1)	unprognostic (1.28e-1)
unprognostic (2.30e-1)	unprognostic (2.74e-2)	unprognostic (3.78e-2)
unprognostic (1.06e-1)	unprognostic (1.47e-1)	unprognostic (1.11e-1)
	unprognostic (1.63e-2)	

unprognostic (2.68e-2)	unprognostic (1.44e-1)	unprognostic (1.24e-1)
unprognostic (1.68e-1)		unprognostic (5.04e-2)
unprognostic (4.09e-2)		unprognostic (2.08e-1)
unprognostic (3.24e-1)	unprognostic (1.40e-1)	unprognostic (3.71e-2)
unprognostic (2.91e-1)	potential prognostic unfavorable (2.39e-5)	unprognostic (1.50e-1)
unprognostic (2.56e-1)	unprognostic (6.46e-2)	unprognostic (3.01e-2)
unprognostic (1.68e-1)	unprognostic (1.08e-2)	unprognostic (4.34e-2)
unprognostic (4.20e-2)	unprognostic (3.85e-3)	unprognostic (1.19e-1)
unprognostic (1.43e-1)	unprognostic (2.21e-1)	unprognostic (1.89e-1)
unprognostic (1.52e-2)	unprognostic (3.36e-1)	unprognostic (4.89e-2)
unprognostic (3.40e-1)		
unprognostic (1.05e-1)	unprognostic (3.37e-3)	unprognostic (3.65e-2)
unprognostic (5.07e-2)		
		unprognostic (9.80e-3)
unprognostic (1.85e-2)	unprognostic (1.41e-1)	unprognostic (1.98e-1)
	unprognostic (6.09e-2)	unprognostic (4.08e-2)
unprognostic (4.77e-1)	unprognostic (2.58e-3)	unprognostic (2.46e-1)
unprognostic (4.59e-2)	unprognostic (1.59e-1)	unprognostic (2.35e-1)
unprognostic (9.94e-2)	unprognostic (1.47e-2)	unprognostic (2.48e-1)
unprognostic (6.81e-2)	unprognostic (6.82e-2)	
unprognostic (1.27e-1)	unprognostic (1.10e-2)	unprognostic (7.09e-2)
unprognostic (7.70e-2)	unprognostic (1.19e-1)	unprognostic (2.03e-1)
unprognostic (2.04e-1)	unprognostic (1.01e-1)	unprognostic (6.11e-2)
unprognostic (1.21e-1)	unprognostic (4.59e-2)	unprognostic (9.12e-2)
unprognostic (1.30e-1)	unprognostic (8.54e-2)	unprognostic (3.52e-3)
unprognostic (2.92e-1)	unprognostic (8.09e-2)	unprognostic (7.18e-2)
unprognostic (1.55e-1)	unprognostic (1.38e-2)	unprognostic (9.05e-2)
unprognostic (2.68e-1)	unprognostic (7.69e-2)	unprognostic (3.17e-1)
unprognostic (1.59e-1)	unprognostic (7.80e-2)	unprognostic (5.18e-2)
unprognostic (2.85e-3)	unprognostic (1.90e-1)	
unprognostic (4.88e-3)	unprognostic (5.23e-2)	unprognostic (4.34e-3)
unprognostic (3.75e-2)	unprognostic (2.00e-2)	unprognostic (1.27e-1)
unprognostic (4.18e-1)	unprognostic (1.76e-1)	
unprognostic (7.60e-2)	unprognostic (2.15e-1)	unprognostic (1.89e-1)
unprognostic (1.40e-1)	unprognostic (3.22e-2)	unprognostic (3.74e-2)
unprognostic (2.75e-2)	unprognostic (8.92e-2)	unprognostic (3.62e-1)
unprognostic (1.26e-1)	unprognostic (9.96e-3)	unprognostic (1.69e-1)
unprognostic (2.90e-1)	unprognostic (2.79e-2)	unprognostic (1.02e-1)
unprognostic (1.49e-1)	unprognostic (3.22e-2)	unprognostic (2.78e-1)
unprognostic (3.45e-1)	unprognostic (2.75e-2)	unprognostic (3.61e-1)
unprognostic (1.90e-1)	unprognostic (5.34e-2)	unprognostic (1.19e-1)
unprognostic (2.89e-1)	unprognostic (5.13e-2)	unprognostic (2.83e-1)
unprognostic (3.41e-2)	unprognostic (5.25e-3)	unprognostic (3.29e-2)
unprognostic (1.25e-1)		unprognostic (7.59e-2)
unprognostic (2.38e-1)	unprognostic (2.06e-1)	unprognostic (8.50e-2)
unprognostic (4.79e-2)	unprognostic (1.66e-1)	unprognostic (3.83e-2)
unprognostic (2.93e-1)	unprognostic (2.92e-2)	unprognostic (2.93e-1)
unprognostic (2.88e-1)	unprognostic (2.60e-2)	unprognostic (7.73e-2)
unprognostic (3.60e-2)	unprognostic (3.95e-2)	unprognostic (1.22e-2)
unprognostic (1.17e-2)	unprognostic (5.59e-2)	unprognostic (8.07e-2)
unprognostic (1.34e-1)		
unprognostic (1.80e-1)	unprognostic (8.08e-3)	unprognostic (1.23e-1)
unprognostic (2.43e-2)	unprognostic (3.23e-1)	unprognostic (8.28e-2)
unprognostic (1.00e-2)		
unprognostic (2.25e-2)	unprognostic (8.57e-2)	unprognostic (3.68e-2)
unprognostic (1.69e-1)	unprognostic (3.08e-1)	unprognostic (1.31e-1)
unprognostic (3.11e-1)	unprognostic (1.18e-1)	unprognostic (2.68e-2)
unprognostic (2.21e-1)	unprognostic (2.80e-2)	unprognostic (1.29e-3)
unprognostic (2.33e-1)		unprognostic (5.08e-2)
unprognostic (1.23e-1)	unprognostic (1.55e-2)	unprognostic (1.16e-2)
unprognostic (5.79e-2)	unprognostic (9.97e-2)	unprognostic (3.15e-1)
		unprognostic (8.42e-2)
unprognostic (1.40e-1)		unprognostic (2.00e-2)
unprognostic (9.23e-2)	unprognostic (2.09e-1)	unprognostic (5.87e-2)
		unprognostic (1.17e-1)
unprognostic (1.85e-1)	unprognostic (7.72e-3)	unprognostic (3.78e-2)
unprognostic (2.25e-2)	unprognostic (1.47e-1)	unprognostic (8.05e-2)
unprognostic (3.30e-2)	unprognostic (1.36e-1)	unprognostic (9.72e-2)
unprognostic (1.81e-1)	unprognostic (2.20e-1)	unprognostic (2.61e-2)
unprognostic (3.11e-1)	unprognostic (3.67e-2)	unprognostic (1.95e-2)
unprognostic (1.98e-1)	unprognostic (3.74e-2)	unprognostic (2.59e-2)
unprognostic (1.82e-1)	unprognostic (9.39e-2)	unprognostic (3.44e-1)
unprognostic (3.12e-1)	unprognostic (1.15e-1)	unprognostic (3.63e-1)
unprognostic (2.70e-1)	unprognostic (2.49e-2)	unprognostic (3.68e-2)
unprognostic (1.06e-1)	unprognostic (1.43e-1)	potential prognostic unfavorable (2.99e-4)
unprognostic (1.98e-1)	unprognostic (7.37e-2)	unprognostic (3.21e-1)
unprognostic (2.18e-1)	unprognostic (1.51e-2)	unprognostic (2.92e-1)
unprognostic (1.09e-1)	unprognostic (1.83e-2)	unprognostic (3.89e-1)
unprognostic (1.74e-1)	unprognostic (2.51e-2)	unprognostic (3.63e-2)
unprognostic (3.57e-2)	unprognostic (5.26e-2)	unprognostic (1.52e-1)
unprognostic (9.75e-3)	unprognostic (8.67e-2)	unprognostic (1.08e-1)
unprognostic (1.17e-1)	unprognostic (3.57e-1)	unprognostic (2.24e-1)
unprognostic (1.15e-1)	unprognostic (1.64e-1)	unprognostic (3.83e-2)
		unprognostic (2.21e-1)
	unprognostic (2.33e-2)	unprognostic (6.13e-2)
unprognostic (5.09e-2)	unprognostic (6.67e-2)	unprognostic (7.26e-3)
unprognostic (2.47e-2)	unprognostic (1.27e-1)	unprognostic (2.38e-1)
unprognostic (2.73e-2)	unprognostic (1.21e-2)	unprognostic (9.13e-2)
unprognostic (1.86e-1)	unprognostic (1.59e-1)	unprognostic (4.06e-2)
	unprognostic (2.81e-2)	unprognostic (2.77e-2)
unprognostic (3.89e-2)	unprognostic (1.59e-2)	unprognostic (3.22e-2)
unprognostic (1.80e-1)	unprognostic (2.88e-2)	unprognostic (3.14e-2)
unprognostic (3.42e-2)		
unprognostic (8.33e-3)	unprognostic (1.06e-2)	unprognostic (1.84e-1)
	unprognostic (3.11e-3)	
unprognostic (2.21e-1)	unprognostic (1.09e-1)	unprognostic (7.07e-2)
unprognostic (1.78e-1)	unprognostic (5.86e-2)	unprognostic (2.22e-1)
unprognostic (1.21e-1)	unprognostic (3.05e-1)	unprognostic (3.40e-1)
unprognostic (1.56e-1)	unprognostic (3.76e-2)	unprognostic (7.01e-2)
unprognostic (2.72e-1)	unprognostic (3.37e-2)	unprognostic (3.27e-1)
unprognostic (2.70e-2)	unprognostic (9.39e-2)	unprognostic (1.26e-1)
unprognostic (4.86e-1)	unprognostic (8.92e-2)	unprognostic (2.73e-1)
unprognostic (1.48e-1)	unprognostic (3.42e-2)	unprognostic (6.76e-2)
unprognostic (9.83e-2)	unprognostic (1.54e-1)	unprognostic (8.21e-2)
unprognostic (4.12e-1)	potential prognostic unfavorable (1.85e-4)	unprognostic (8.75e-2)
unprognostic (6.56e-2)	unprognostic (3.52e-2)	unprognostic (6.37e-2)
unprognostic (3.73e-2)	unprognostic (9.80e-2)	unprognostic (1.65e-1)
unprognostic (4.43e-1)	unprognostic (6.01e-2)	unprognostic (2.42e-1)
unprognostic (1.66e-2)	unprognostic (3.33e-1)	unprognostic (3.69e-1)
unprognostic (5.23e-1)	unprognostic (2.13e-2)	unprognostic (1.66e-1)
	unprognostic (2.32e-2)	unprognostic (1.68e-2)
unprognostic (5.68e-2)		unprognostic (2.28e-3)
unprognostic (1.74e-1)	unprognostic (1.53e-1)	unprognostic (1.12e-1)
unprognostic (2.63e-1)	unprognostic (3.93e-3)	unprognostic (1.54e-3)
unprognostic (2.15e-2)	unprognostic (1.68e-1)	unprognostic (2.86e-2)

unprognostic (4.73e-2)	unprognostic (2.02e-2)	unprognostic (7.02e-2)
unprognostic (3.03e-1)	unprognostic (1.39e-2)	unprognostic (4.00e-3)
unprognostic (2.37e-1)	unprognostic (1.14e-1)	unprognostic (1.60e-1)
unprognostic (3.32e-1)	unprognostic (6.03e-2)	unprognostic (1.49e-1)
unprognostic (4.59e-2)	unprognostic (3.40e-1)	unprognostic (8.35e-2)
unprognostic (2.95e-1)	unprognostic (7.52e-3)	unprognostic (3.02e-1)
unprognostic (1.05e-1)	unprognostic (9.56e-2)	potential prognostic unfavorable (4.06e-4)
unprognostic (1.62e-1)		unprognostic (1.02e-2)
unprognostic (3.18e-1)	unprognostic (4.68e-3)	unprognostic (6.96e-3)
unprognostic (1.63e-1)	unprognostic (1.09e-2)	unprognostic (8.86e-2)
unprognostic (1.67e-1)	potential prognostic unfavorable (3.48e-4)	unprognostic (2.94e-2)
unprognostic (4.28e-1)	unprognostic (4.89e-2)	unprognostic (4.31e-2)
unprognostic (2.33e-2)	unprognostic (2.52e-2)	unprognostic (1.09e-1)
unprognostic (1.73e-1)	unprognostic (4.86e-2)	unprognostic (6.45e-2)
unprognostic (7.98e-2)		unprognostic (3.01e-1)
unprognostic (1.12e-1)	unprognostic (3.58e-3)	unprognostic (1.84e-1)
unprognostic (2.71e-1)	unprognostic (2.11e-1)	unprognostic (3.55e-1)
unprognostic (1.90e-1)	unprognostic (2.67e-1)	unprognostic (4.30e-1)
unprognostic (2.70e-2)	unprognostic (1.45e-1)	unprognostic (3.59e-1)
unprognostic (1.59e-1)	unprognostic (3.98e-2)	unprognostic (2.38e-1)
unprognostic (1.81e-1)	potential prognostic unfavorable (5.38e-5)	unprognostic (9.26e-2)
unprognostic (4.79e-2)	unprognostic (1.31e-2)	unprognostic (2.58e-1)
unprognostic (3.81e-1)	unprognostic (2.25e-1)	unprognostic (1.58e-1)
		unprognostic (9.70e-2)
unprognostic (3.22e-2)	unprognostic (4.98e-2)	unprognostic (3.13e-1)
unprognostic (1.78e-1)	unprognostic (2.58e-1)	unprognostic (8.51e-3)
unprognostic (3.39e-2)	unprognostic (1.79e-1)	unprognostic (2.06e-1)
unprognostic (1.89e-1)	unprognostic (2.15e-2)	unprognostic (2.99e-1)
unprognostic (6.76e-2)	unprognostic (3.53e-3)	unprognostic (1.74e-1)
unprognostic (6.93e-2)	unprognostic (3.06e-2)	unprognostic (1.62e-1)
unprognostic (1.32e-1)	unprognostic (1.75e-1)	unprognostic (2.19e-1)
unprognostic (2.49e-1)	unprognostic (4.67e-2)	unprognostic (1.81e-2)
unprognostic (2.08e-1)	unprognostic (2.55e-1)	unprognostic (8.21e-2)
unprognostic (1.42e-1)	unprognostic (1.79e-1)	unprognostic (1.68e-1)
	unprognostic (3.28e-1)	
		unprognostic (6.23e-2)
unprognostic (2.75e-1)	unprognostic (2.03e-1)	unprognostic (1.24e-1)
unprognostic (3.61e-2)	unprognostic (1.32e-1)	unprognostic (2.22e-1)
unprognostic (2.42e-1)	unprognostic (2.14e-1)	unprognostic (1.43e-1)
unprognostic (5.93e-2)	unprognostic (3.05e-1)	unprognostic (1.90e-1)
unprognostic (2.12e-1)	unprognostic (3.87e-2)	unprognostic (3.30e-1)
unprognostic (1.69e-1)	unprognostic (8.18e-3)	unprognostic (2.35e-2)
unprognostic (2.48e-2)	unprognostic (2.71e-2)	unprognostic (1.26e-1)
unprognostic (3.11e-1)	unprognostic (3.49e-1)	unprognostic (1.11e-1)
unprognostic (6.72e-2)	unprognostic (1.61e-2)	unprognostic (2.12e-1)
unprognostic (2.23e-1)		
unprognostic (4.81e-1)	unprognostic (4.49e-3)	unprognostic (3.49e-2)
unprognostic (1.13e-1)	unprognostic (6.70e-2)	unprognostic (8.19e-2)
unprognostic (3.99e-1)	unprognostic (5.74e-2)	unprognostic (2.21e-1)
unprognostic (6.41e-2)	unprognostic (4.88e-3)	unprognostic (6.66e-3)
unprognostic (2.74e-1)	unprognostic (1.07e-1)	unprognostic (3.89e-2)
unprognostic (1.64e-1)	unprognostic (1.99e-1)	unprognostic (9.52e-2)
unprognostic (2.46e-2)	unprognostic (5.02e-2)	unprognostic (2.85e-1)
unprognostic (3.15e-3)	unprognostic (1.53e-1)	unprognostic (5.48e-3)
unprognostic (8.81e-2)	unprognostic (1.30e-1)	unprognostic (2.19e-1)
unprognostic (1.41e-1)	unprognostic (2.08e-1)	unprognostic (3.23e-1)
unprognostic (2.56e-1)	unprognostic (1.48e-1)	unprognostic (1.55e-2)
unprognostic (4.11e-2)	unprognostic (1.27e-2)	unprognostic (1.47e-2)
unprognostic (1.34e-1)	unprognostic (1.76e-2)	unprognostic (8.33e-2)
unprognostic (7.60e-2)	unprognostic (2.19e-2)	unprognostic (3.02e-1)
unprognostic (1.89e-1)	unprognostic (1.95e-2)	unprognostic (2.36e-1)
unprognostic (2.80e-1)	unprognostic (4.36e-2)	unprognostic (2.37e-1)
unprognostic (3.78e-1)	unprognostic (1.41e-1)	unprognostic (1.45e-1)
unprognostic (4.21e-1)	unprognostic (1.45e-2)	unprognostic (8.65e-2)
unprognostic (7.62e-1)		
unprognostic (1.23e-1)	unprognostic (1.25e-1)	unprognostic (1.34e-1)
unprognostic (4.20e-2)		unprognostic (2.11e-2)
unprognostic (5.00e-1)	unprognostic (4.38e-2)	unprognostic (7.75e-2)
unprognostic (1.21e-1)	unprognostic (1.97e-2)	unprognostic (3.66e-2)
unprognostic (1.18e-1)	unprognostic (2.92e-2)	unprognostic (1.68e-1)
	unprognostic (1.00e-2)	unprognostic (1.38e-2)
unprognostic (2.30e-1)	unprognostic (4.97e-2)	
unprognostic (1.53e-1)		
unprognostic (2.70e-1)		
unprognostic (9.71e-3)	unprognostic (2.04e-1)	unprognostic (3.17e-1)
	unprognostic (1.61e-1)	unprognostic (3.22e-1)
unprognostic (2.15e-1)	unprognostic (1.64e-1)	unprognostic (6.66e-2)
unprognostic (2.06e-1)	potential prognostic unfavorable (7.73e-5)	unprognostic (9.60e-2)
unprognostic (2.09e-1)	unprognostic (1.42e-2)	unprognostic (7.57e-2)
unprognostic (1.47e-1)	unprognostic (9.99e-3)	unprognostic (3.52e-1)
unprognostic (1.05e-1)	unprognostic (4.68e-1)	unprognostic (4.19e-2)
unprognostic (1.50e-1)	unprognostic (1.10e-1)	unprognostic (1.51e-2)
unprognostic (1.13e-2)	unprognostic (3.51e-1)	unprognostic (1.82e-1)
unprognostic (3.95e-2)	unprognostic (9.73e-2)	unprognostic (1.93e-1)
unprognostic (1.18e-1)	unprognostic (5.10e-2)	unprognostic (1.48e-1)
unprognostic (3.06e-1)	unprognostic (2.94e-2)	unprognostic (6.14e-2)
unprognostic (2.31e-1)	unprognostic (1.37e-1)	unprognostic (3.12e-1)
unprognostic (1.43e-1)		
	unprognostic (1.69e-1)	unprognostic (1.77e-1)
unprognostic (3.32e-1)	unprognostic (1.26e-2)	unprognostic (5.01e-2)
unprognostic (1.63e-1)	unprognostic (6.55e-2)	unprognostic (1.26e-1)
unprognostic (2.99e-2)	unprognostic (1.97e-2)	unprognostic (7.34e-2)
unprognostic (7.40e-2)	unprognostic (5.32e-2)	unprognostic (1.04e-1)
unprognostic (1.34e-1)	unprognostic (8.91e-2)	unprognostic (3.88e-1)
unprognostic (1.34e-1)		unprognostic (1.96e-3)
unprognostic (1.30e-1)	unprognostic (6.30e-2)	unprognostic (3.61e-2)
unprognostic (2.37e-2)	unprognostic (4.97e-3)	unprognostic (1.49e-1)
unprognostic (2.38e-1)	unprognostic (1.65e-3)	unprognostic (1.64e-1)
		unprognostic (4.34e-1)
unprognostic (2.55e-1)		unprognostic (2.78e-2)
unprognostic (2.37e-1)	unprognostic (4.75e-3)	unprognostic (8.18e-2)
unprognostic (1.21e-1)		unprognostic (2.18e-2)
unprognostic (1.80e-1)	unprognostic (2.51e-1)	unprognostic (3.37e-2)
unprognostic (2.31e-1)		
unprognostic (1.21e-1)	unprognostic (1.61e-2)	unprognostic (1.15e-2)
unprognostic (2.39e-1)	unprognostic (2.55e-2)	unprognostic (4.38e-2)
unprognostic (1.06e-1)	unprognostic (1.21e-1)	unprognostic (1.19e-2)
unprognostic (5.49e-2)	unprognostic (6.82e-2)	unprognostic (1.10e-1)
unprognostic (5.98e-2)	unprognostic (1.49e-1)	unprognostic (1.74e-2)
unprognostic (4.88e-2)	unprognostic (6.15e-2)	unprognostic (2.22e-1)
unprognostic (8.33e-2)	unprognostic (1.61e-1)	unprognostic (3.90e-1)
unprognostic (2.97e-1)	unprognostic (9.89e-2)	unprognostic (2.03e-1)
unprognostic (2.15e-1)	unprognostic (2.01e-1)	unprognostic (2.12e-1)
	unprognostic (1.32e-3)	unprognostic (6.62e-3)
unprognostic (1.77e-1)	unprognostic (1.18e-1)	unprognostic (3.13e-1)
		unprognostic (2.22e-3)
unprognostic (2.24e-1)	unprognostic (2.07e-1)	unprognostic (4.62e-1)
unprognostic (4.95e-2)	unprognostic (2.59e-1)	unprognostic (5.91e-2)
unprognostic (9.05e-2)	unprognostic (1.27e-3)	unprognostic (7.44e-2)
unprognostic (2.87e-1)	potential prognostic unfavorable (3.43e-4)	unprognostic (2.45e-1)

unprognostic (3.68e-1)		unprognostic (8.56e-3)
unprognostic (2.65e-1)	unprognostic (4.39e-2)	unprognostic (2.52e-1)
		unprognostic (6.76e-2)
unprognostic (4.75e-1)	unprognostic (5.21e-2)	unprognostic (7.49e-2)
unprognostic (2.04e-1)	unprognostic (4.41e-1)	unprognostic (7.42e-2)
unprognostic (3.27e-1)	unprognostic (1.41e-2)	unprognostic (4.95e-2)
unprognostic (2.08e-1)		unprognostic (3.61e-1)
unprognostic (3.38e-2)	unprognostic (2.49e-1)	unprognostic (6.64e-2)
unprognostic (2.17e-1)		unprognostic (1.26e-1)
unprognostic (5.80e-2)	unprognostic (2.58e-1)	unprognostic (3.75e-1)
unprognostic (1.39e-1)		unprognostic (2.88e-2)
unprognostic (2.17e-1)	unprognostic (1.76e-2)	unprognostic (1.79e-1)
unprognostic (1.37e-1)	unprognostic (1.04e-1)	unprognostic (1.46e-1)
unprognostic (3.09e-1)	unprognostic (2.10e-1)	unprognostic (8.50e-2)
unprognostic (1.98e-1)	unprognostic (2.40e-2)	unprognostic (1.52e-1)
unprognostic (1.84e-1)	unprognostic (1.30e-2)	unprognostic (1.70e-1)
unprognostic (7.09e-2)	unprognostic (1.46e-1)	unprognostic (9.26e-2)
unprognostic (5.19e-2)	unprognostic (4.13e-2)	unprognostic (8.49e-2)
unprognostic (2.03e-2)	unprognostic (1.53e-1)	unprognostic (2.39e-2)
unprognostic (4.79e-1)	unprognostic (1.60e-2)	unprognostic (5.32e-2)
unprognostic (2.34e-1)	unprognostic (2.07e-1)	potential prognostic unfavorable (7.62e-4)
unprognostic (8.67e-2)	unprognostic (7.21e-2)	unprognostic (1.55e-2)
unprognostic (2.74e-1)	unprognostic (1.71e-1)	unprognostic (1.24e-1)
unprognostic (7.89e-2)	unprognostic (1.48e-1)	unprognostic (5.15e-3)
unprognostic (2.92e-1)	unprognostic (1.83e-1)	unprognostic (9.13e-2)
unprognostic (4.22e-2)	unprognostic (2.85e-1)	unprognostic (1.17e-1)
unprognostic (3.33e-2)	unprognostic (1.19e-1)	unprognostic (1.37e-2)
unprognostic (1.08e-1)	unprognostic (2.52e-1)	unprognostic (5.16e-2)
unprognostic (1.29e-1)	unprognostic (8.41e-2)	unprognostic (3.17e-2)
		unprognostic (1.49e-1)
unprognostic (3.38e-1)	unprognostic (1.86e-3)	unprognostic (1.18e-2)
unprognostic (7.40e-2)	unprognostic (6.07e-2)	unprognostic (4.13e-1)
unprognostic (3.42e-1)	unprognostic (5.84e-2)	unprognostic (2.48e-1)
unprognostic (1.94e-1)	unprognostic (4.25e-2)	unprognostic (1.61e-1)
unprognostic (1.71e-1)	unprognostic (2.20e-1)	unprognostic (6.72e-2)
unprognostic (6.88e-2)	unprognostic (6.29e-2)	unprognostic (2.41e-1)
		unprognostic (3.18e-2)
unprognostic (3.42e-1)	unprognostic (1.49e-2)	unprognostic (8.16e-2)
	unprognostic (3.84e-3)	
unprognostic (1.32e-2)	unprognostic (7.24e-2)	unprognostic (2.81e-1)
unprognostic (2.66e-1)	unprognostic (2.76e-3)	unprognostic (1.52e-1)
		unprognostic (4.92e-2)
unprognostic (1.39e-1)	unprognostic (4.51e-1)	unprognostic (3.95e-2)
unprognostic (1.64e-1)	unprognostic (2.67e-3)	unprognostic (2.12e-1)
unprognostic (2.33e-1)	unprognostic (1.40e-1)	unprognostic (1.61e-1)
unprognostic (3.71e-2)	unprognostic (5.25e-2)	unprognostic (6.63e-2)
unprognostic (1.66e-1)	unprognostic (8.25e-2)	unprognostic (3.82e-1)
unprognostic (1.94e-2)	unprognostic (3.62e-2)	unprognostic (1.88e-1)
unprognostic (1.59e-1)	potential prognostic favorable (6.81e-4)	unprognostic (1.51e-1)
unprognostic (4.73e-1)	unprognostic (1.45e-3)	unprognostic (5.38e-2)
unprognostic (1.83e-1)	unprognostic (9.71e-2)	unprognostic (5.86e-2)
unprognostic (1.70e-1)	unprognostic (2.07e-2)	unprognostic (1.60e-1)
unprognostic (9.73e-2)	unprognostic (9.59e-2)	unprognostic (9.70e-2)
unprognostic (2.10e-2)	unprognostic (2.61e-2)	unprognostic (1.59e-1)
unprognostic (7.73e-2)	unprognostic (9.89e-2)	unprognostic (4.35e-1)
unprognostic (2.27e-3)	unprognostic (4.00e-3)	unprognostic (5.86e-2)
unprognostic (2.58e-1)		
unprognostic (7.06e-2)	unprognostic (2.68e-2)	unprognostic (4.63e-2)
unprognostic (5.92e-2)	unprognostic (4.74e-2)	unprognostic (1.63e-1)
unprognostic (1.66e-1)	unprognostic (2.22e-1)	unprognostic (4.21e-2)
unprognostic (8.84e-2)		unprognostic (1.47e-1)
unprognostic (1.46e-1)	unprognostic (1.78e-1)	unprognostic (7.11e-3)
unprognostic (2.47e-1)	unprognostic (1.96e-1)	unprognostic (2.04e-2)
unprognostic (6.89e-2)		
unprognostic (3.08e-2)	unprognostic (3.62e-1)	unprognostic (1.56e-2)
unprognostic (3.92e-1)	unprognostic (1.53e-1)	unprognostic (1.61e-1)
unprognostic (1.54e-1)	unprognostic (2.11e-1)	unprognostic (3.14e-1)
unprognostic (3.92e-1)		
unprognostic (3.10e-1)	unprognostic (8.63e-2)	unprognostic (9.35e-2)
unprognostic (2.41e-2)		
unprognostic (2.63e-1)		
unprognostic (2.45e-2)		
unprognostic (3.45e-1)	potential prognostic unfavorable (3.17e-4)	unprognostic (2.77e-2)
unprognostic (7.89e-3)	unprognostic (5.95e-2)	unprognostic (1.60e-1)
unprognostic (4.88e-2)	unprognostic (7.34e-3)	unprognostic (1.01e-1)
unprognostic (1.55e-1)	unprognostic (3.55e-2)	unprognostic (5.70e-2)
unprognostic (1.84e-1)	unprognostic (3.36e-2)	unprognostic (2.64e-2)
		unprognostic (1.09e-1)
unprognostic (1.88e-2)	unprognostic (1.83e-1)	unprognostic (3.12e-2)
unprognostic (1.11e-3)	unprognostic (2.55e-2)	unprognostic (2.21e-1)
unprognostic (1.90e-1)	unprognostic (1.44e-2)	unprognostic (1.57e-3)
unprognostic (2.45e-1)	unprognostic (8.53e-2)	unprognostic (2.14e-2)
unprognostic (5.01e-2)	unprognostic (1.11e-1)	
unprognostic (1.85e-1)	unprognostic (3.32e-1)	unprognostic (5.67e-2)
unprognostic (3.50e-1)	unprognostic (2.28e-1)	unprognostic (2.42e-1)
unprognostic (1.75e-1)		unprognostic (2.02e-2)
unprognostic (3.38e-1)	unprognostic (2.90e-1)	unprognostic (2.40e-1)
unprognostic (1.12e-1)	unprognostic (4.06e-2)	unprognostic (5.53e-2)
unprognostic (1.97e-1)		unprognostic (1.18e-2)
unprognostic (4.55e-1)	unprognostic (2.66e-1)	unprognostic (2.68e-1)
unprognostic (1.72e-1)	unprognostic (1.91e-1)	unprognostic (3.46e-2)
unprognostic (1.78e-1)	unprognostic (3.70e-2)	unprognostic (1.01e-2)
unprognostic (2.40e-1)	unprognostic (2.16e-2)	unprognostic (5.40e-3)
unprognostic (2.11e-1)	unprognostic (2.98e-2)	unprognostic (3.24e-1)
unprognostic (2.77e-2)	unprognostic (7.83e-3)	unprognostic (2.54e-2)
unprognostic (2.20e-1)	unprognostic (3.04e-2)	unprognostic (6.64e-2)
unprognostic (4.73e-1)	unprognostic (3.02e-2)	unprognostic (2.78e-1)
unprognostic (2.71e-1)	unprognostic (2.02e-1)	unprognostic (5.82e-2)
unprognostic (2.63e-1)	unprognostic (8.02e-2)	unprognostic (3.35e-1)
unprognostic (1.03e-1)	unprognostic (1.73e-1)	unprognostic (2.48e-1)
unprognostic (2.23e-1)	unprognostic (1.13e-1)	unprognostic (6.16e-2)
