# Supplementary material for: LINGO1-targeted antibody-drug conjugates improve efficacy and tolerability of antineoplastic therapies in Ewing sarcoma models
Source: J Clin Invest. 2026 Aug 3;136(15):e204641. doi: 10.1172/JCI204641 (PMC13430015; doi:10.1172/JCI204641)
Supplement: Unedited blot and gel images [file jci-136-204641-s188.pdf]

Full unedited blot/gel for Figure 1D

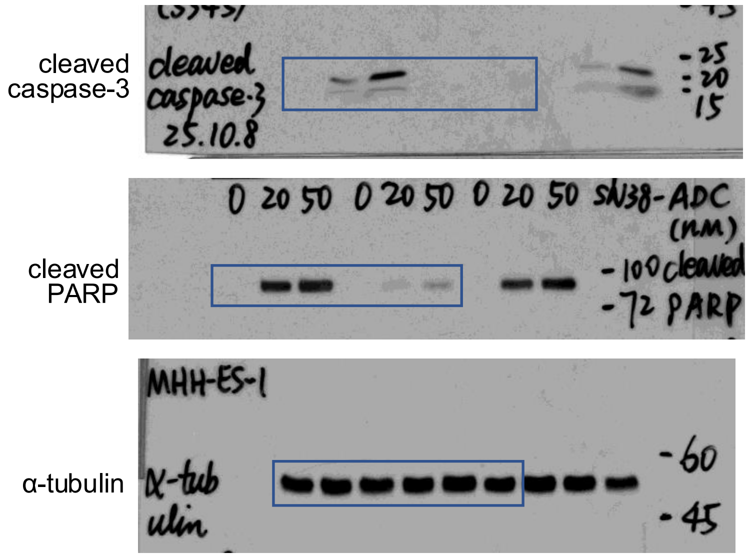

Full unedited blot/gel for Figure S1C

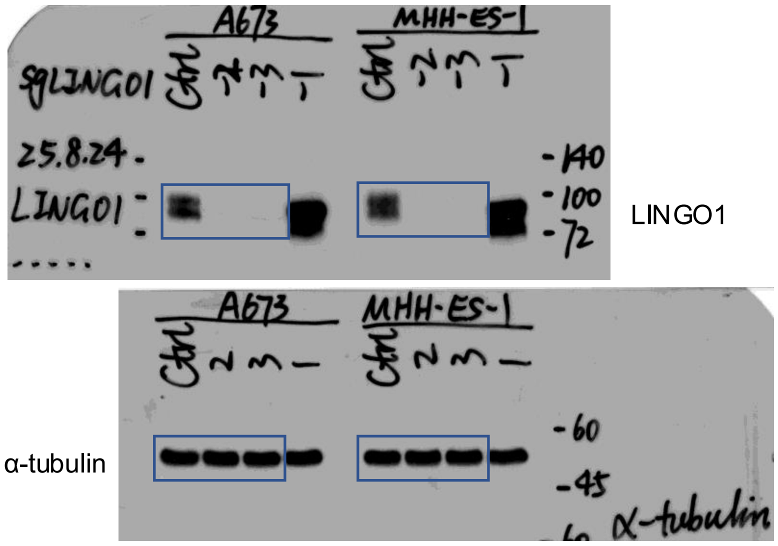

Full unedited blot/gel for Figure S2E

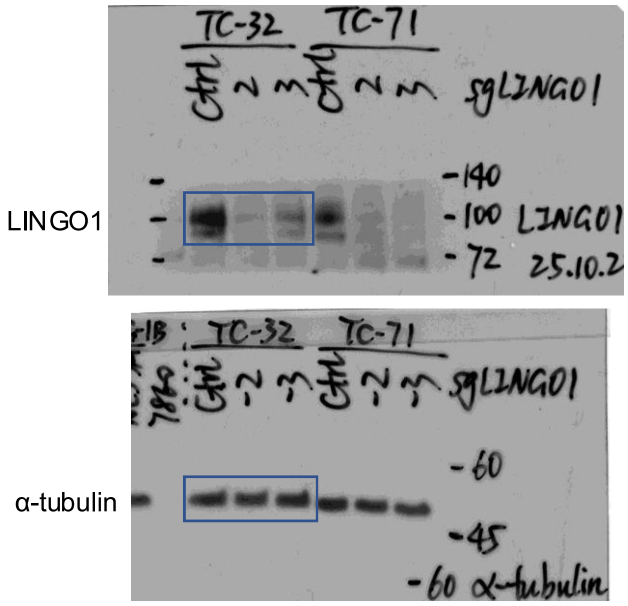

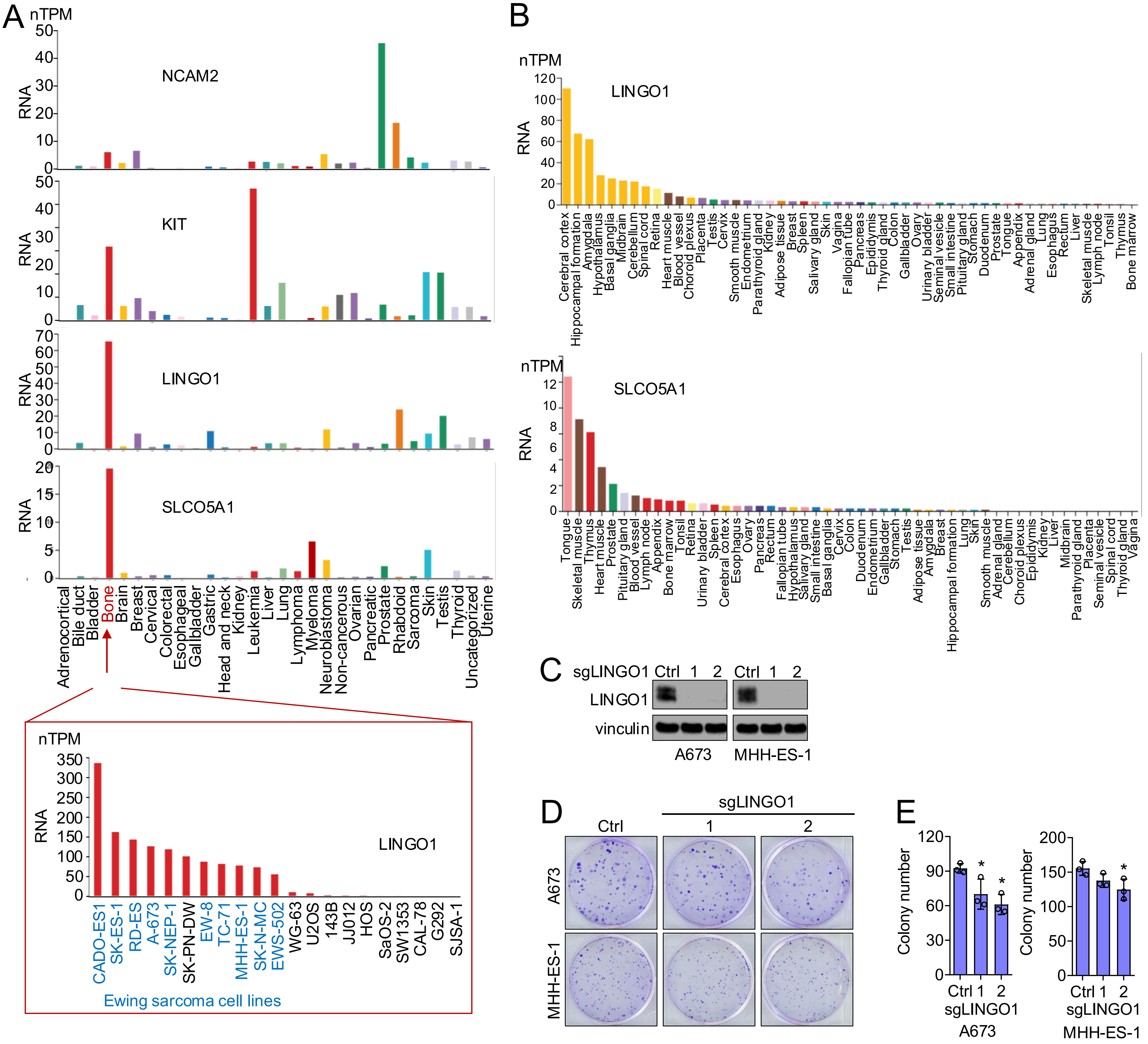

A
nTPM
RNA
NCAM2
KIT
LINGO1
SLCO5A1
Adrenocortical
Bile duct
Bladder
Bone
Brain
Breast
Cervical
Colorectal
Esophageal
Gallbladder
Gastric
Head and neck
Kidney
Leukemia
Liver
Lung
Lymphoma
Myeloma
Neuroblastoma
Non-cancerous
Ovarian
Pancreatic
Prostate
Rhabdoid
Sarcoma
Skin
Testis
Thyroid
Uncategorized
Uterine
CADO-ES1
SK-ES-1
RD-ES
A-673
SK-NEP-1
SK-PN-DW
EW-8
TC-71
MHH-ES-1
SK-N-MC
EWS-502
WG-63
U2OS
143B
JJ012
HOS
SaOS-2
SW1353
CAL-78
G292
SJSA-1
Ewing sarcoma cell lines
B
Cerebral cortex
Hippocampal formation
Amygdala
Hypothalamus
Basal ganglia
Midbrain
Cerebellum
Spinal cord
Retina
Heart muscle
Blood vessel
Choroid plexus
Placenta
Testis
Cervix
Smooth muscle
Endometrium
Parathyroid gland
Kidney
Adipose tissue
Breast
Spleen
Salivary gland
Skin
Vagina
Fallopian tube
Pancreas
Epididymis
Thyroid gland
Colon
Gallbladder
Ovary
Urinary bladder
Seminal vesicle
Small intestine
Pituitary gland
Stomach
Duodenum
Prostate
Tongue
Appendix
Adrenal gland
Lung
Esophagus
Rectum
Liver
Skeletal muscle
Lymph node
Tonsil
Thymus
Bone marrow
C
sgLINGO1 Ctrl 1 2 Ctrl 1 2
LINGO1
vinculin
A673
MHH-ES-1
D
sgLINGO1
Ctrl
1
2
A673
MHH-ES-1
E
Colony number
Ctrl 1 2
sgLINGO1
A673
MHH-ES-1

Figure S1
